# Lithium response in bipolar disorder correlates with improved cell viability of patient derived cell lines

**DOI:** 10.1038/s41598-020-64202-1

**Published:** 2020-05-04

**Authors:** Pradip Paul, Shruti Iyer, Ravi Kumar Nadella, Rashmitha Nayak, Anirudh S. Chellappa, Sheetal Ambardar, Reeteka Sud, Salil K. Sukumaran, Meera Purushottam, Sanjeev Jain, ADBS Consortium (ADBS: The Accelerator program for Discovery in Brain disorders using Stem cells), Naren P. Rao, Janardhanan C. Narayanaswamy, Palanimuthu T. Sivakumar, Arun Kandasamy, Muralidharan Kesavan, Urvakhsh Meherwan Mehta, Ganesan Venkatasubramanian, John P. John, Odity Mukherjee, Ramakrishnan Kannan, Bhupesh Mehta, Thennarasu Kandavel, B. Binukumar, Jitender Saini, Deepak Jayarajan, A. Shyamsundar, Sydney Moirangthem, K. G. Vijay Kumar, Jagadisha Thirthalli, Prabha S. Chandra, Bangalore N. Gangadhar, Pratima Murthy, Mitradas M. Panicker, Upinder S. Bhalla, Sumantra Chattarji, Vivek Benegal, Mathew Varghese, Janardhan Y. C. Reddy, Padinjat Raghu, Mahendra Rao, Biju Viswanath

**Affiliations:** 1 National Institute of Mental Health and Neurosciences (NIMHANS), Bengaluru, India; 2 Institute for Stem Cell Science and Regenerative Medicine (InStem), Bengaluru, India; 3 National Centre for Biological Sciences (NCBS), Bengaluru, India

**Keywords:** Bipolar disorder, Induced pluripotent stem cells

## Abstract

Lithium is an effective, well-established treatment for bipolar disorder (BD). However, the mechanisms of its action, and reasons for variations in clinical response, are unclear. We used neural precursor cells (NPCs) and lymphoblastoid cell lines (LCLs), from BD patients characterized for clinical response to lithium (using the “Alda scale” and “NIMH Retrospective Life chart method”), to interrogate cellular phenotypes related to both disease and clinical lithium response. NPCs from two biologically related BD patients who differed in their clinical response to lithium were compared with healthy controls. RNA-Seq and analysis, mitochondrial membrane potential (MMP), cell viability, and cell proliferation parameters were assessed, with and without *in vitro* lithium. These parameters were also examined in LCLs from 25 BD patients (16 lithium responders and 9 non-responders), and 12 controls. MMP was lower in both NPCs and LCLs from BD; but it was reversed with *in vitro* lithium only in LCLs, and this was unrelated to clinical lithium response. The higher cell proliferation observed in BD was unaffected by *in vitro* lithium. Cell death was greater in BD. However, LCLs from clinical lithium responders could be rescued by addition of *in vitro* lithium. *In vitro* lithium also enhanced *BCL2* and *GSK3B* expression in these cells. Our findings indicate cellular phenotypes related to the disease (MMP, cell proliferation) in both NPCs and LCLs; and those related to clinical lithium response (cell viability, *BCL2/GSK3B* expression) in LCLs.

## Introduction

Bipolar disorder (BD) is a highly heritable psychiatric illness, having a lifetime prevalence of 1–3%^1^. Although considered to have neurodevelopmental origins, the symptoms usually manifest in adulthood^2^. Lithium and valproate have been the mainstay of treatment for BD^3^, but their mechanisms of action and pharmacogenomics are poorly understood. Response to lithium varies considerably among patients, with 40–50% patients showing inadequate clinical response. Family members often share a similar pattern of lithium response, suggesting a genetic basis^4^. In our experience, lithium non-responders tend to be more severely ill^5^. However, it remains unclear whether non-response is the cause or effect of clinical severity. Currently, identification of patients who are likely to show a favorable response to lithium relies upon clinical criteria that lack specificity and sensitivity. Lithium remains a well-established drug for treatment of BD, and reliably predicting lithium response using molecular markers would be useful in initiating effective treatment earlier.

Several studies have used lymphoblastoid cell lines (LCLs) to study lithium response *in vitro*^6^. However, only a few studies have accounted for clinical response to lithium^7–16^. The identification of molecular markers for clinical response to lithium in lymphocytes/LCLs has huge potential for clinical translation. However, cells of neuronal lineage are more appropriate for studying the biological determinants of BD, and mechanisms of lithium action.

Human induced pluripotent stem cell (IPSC) models allow for the derivation of specific brain cell types, thus recapitulating the disease in a physiological context. Recent studies on iPSC-derived NPCs and neurons^2^ have found abnormalities in neural patterning, post-mitotic calcium signaling, and neuronal excitability in BD. Only a limited number of studies have examined IPSC derived neurons from BD patients who have been clinically characterized for lithium response. Increased neuronal excitability in IPSC-derived hippocampal neurons from BD patients has been shown to be selectively reduced by lithium in neurons from those patients who are clinical lithium responders^17,18^. Another recent study has identified an altered set-point regulation of CRMP2 (a downstream target of GSK-3B) phosphorylation to be a hallmark of IPSC derived neurons from lithium responsive BD patients^19^.

In this study, we have used a combination of cellular models (LCLs and IPSC-derived NPCs) to explore cellular parameters that clearly differentiate BD and healthy controls. In addition, we aimed to examine if these parameters could be reversed through addition of *in vitro* lithium, and if so, whether this reversal is associated with clinical lithium response.

We have used iPSC-derived neural precursor cells (NPCs) of BD patients from a family with multiple affected members who differed in their clinical response to lithium, and compared these to healthy population controls. Identified phenotypes were further studied in larger samples of LCLs from BD patients characterized for lithium response. Reversal of these phenotypes was attempted with *in vitro* lithium and valproate; the latter being the drug of choice for clinical lithium non-responders in our sample.

A hypothesis-free approach using RNA-Seq analysis did not reveal genome-wide gene expression differences in NPCs with or without *in vitro* lithium. A hypothesis-based approach based on existing literature (Supplementary Table 1) found cellular phenotypes related to disease [mitochondrial membrane potential (MMP) and cell proliferation] in NPCs and LCLs; and those related to lithium treatment response (cell viability and *BCL2/GSK3B* expression) in LCLs.

## Materials and Methods

### Clinical recruitment

**Figure 1:**
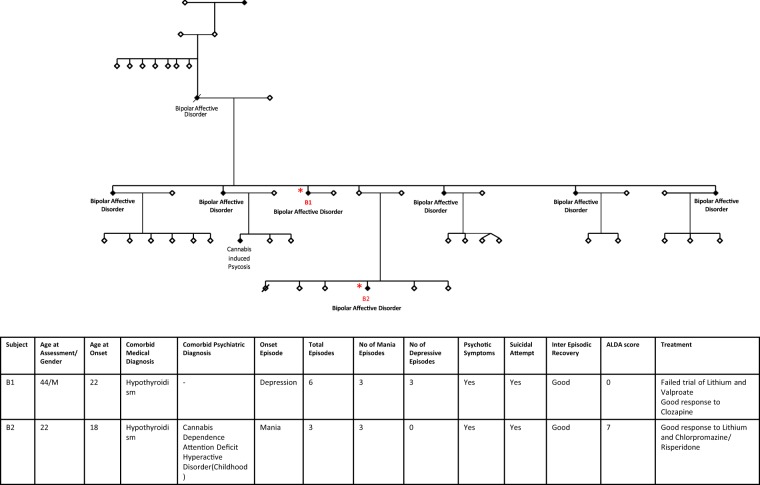
Family A pedigree with clinical details of B1 (lithium non-responder) and B2 (lithium responder).

All BD patients had been recruited as part of a previous study which had systematically characterized 210 patients for clinical lithium response^5^. Family A (Fig. 1) had two BD patients clearly discordant for clinical lithium response (B1 – non-responder and B2 – responder), and had been recruited as part of a family-based cohort study of psychiatric illness in the Indian population, the Accelerator program for Discovery in Brain disorders using Stem cells (ADBS)^20^. All patients were assessed for clinical lithium response using the Alda Scale and NIMH Retrospective Life chart method^4,21^. A subset of 25 BD patients who exhibited extreme phenotypes for clinical lithium response [Lithium responders with Alda score ≥7 (N = 16) and lithium non-responders with Alda score ≤3 (N = 9)] were included in the current study (clinical details in Supplementary Table 2). All DSM-IV psychiatric diagnoses were corroborated by two trained psychiatrists using the Mini International Neuropsychiatric Interview^22^. Healthy controls (N = 12) who had neither Axis-I psychiatric illness nor family history of psychiatric illness in the previous two generations were also recruited. The NIMHANS ethics committee approved the study protocols and written informed consent was obtained from all participants. All research methods were carried out in accordance with the relevant guidelines and regulations.

### LCL generation and characterization

Lymphoblastoid cell lines were generated using Epstein Barr Virus from peripheral blood mononuclear cells as previously described^23^. The cells were grown in RPMI-1640 (Himedia) medium containing 15% heat-inactivated fetal bovine serum (Gibco), 1% Penicillin-Streptomycin (Gibco) and 1% Glutamax (Gibco), as a suspension culture, in 5% CO_2_ incubator at 37 °C. Immunophenotyping of LCLs^24^ by flow cytometry (BD FACSVerse, BD Biosciences, USA) confirmed that the cells were positive for B cell marker CD19, and negative for both the T cell marker CD3 and the Natural Killer cell marker CD56 (Supplementary Figure 1A).

### Differentiation of NPCs from human IPSCs

IPSCs of two patients with BD (lines B1 and B2 from family A), and one unrelated healthy control (C1) were obtained from the ADBS^20^. These IPSCs had been generated from LCLs as previously described^25,26^. Whole exome sequencing from this family has been previously published^27^ and rare damaging variants in B1 and B2 have been identified (Supplementary Table 3). A fibroblast-derived control IPSC (C2) was also used for the experiments. All NPC samples except C1, were from males.

**Figure 2:**
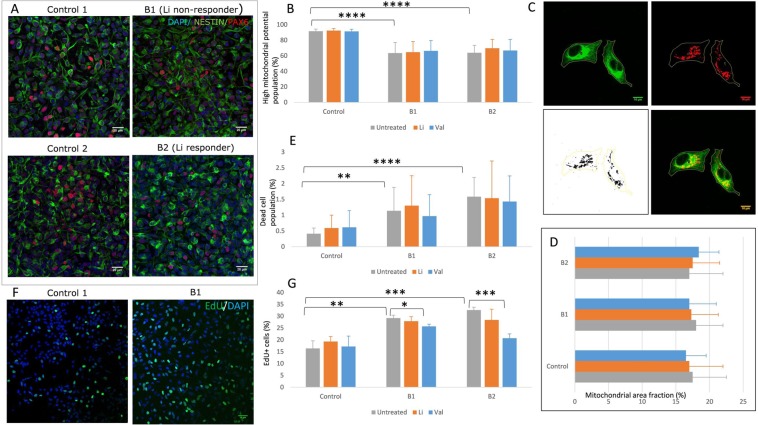
Experiments using NPCs. (**A**) Representative immunocytochemistry of NPCs showing *Nestin*+ cells in all the four cell lines used for experiments. (**B**) Comparison of high MMP population percentage (MTDR) in the three groups at baseline and after *in-vitro* treatment with lithium (1 mM) or valproate (0.7 mM) for 7 days by flow cytometry (N = 5). (**C**) Representative immunocytochemistry of NPCs showing *Nestin*+ cells with mitochondria localized using *TOMM22*, and selection of regions of interest for calculation of mitochondrial area fraction. (**D**) Comparison of mitochondrial area (N = 3). (**E**) Comparison of dead cell population percentage in the three groups at baseline and after *in-vitro* treatment by flow cytometry (N = 5). (**F**) Representative immunocytochemistry of NPCs showing *EdU*+ cells indicating cells in S phase of cell cycle. (**G**) Comparison of *EdU*+ cells in the three groups at baseline and after *in-vitro* treatment (N = 3). All data are shown as mean ± s.e.m.; Experiments were performed in at least 3 independent experiments (N) for each cell line; For group comparisons, initially appropriate ANOVA test was applied and if significant, independent multiple comparison test were performed. Significance level: *p ≤ 0.05, **p ≤ 0.01, ^***^p ≤ 0.001, ****p ≤ 0.0001. Abbreviations: NPCs, neural precursor cells, MTDR, mitotracker deep red; MMP, mitochondrial membrane potential, EdU, 5-ethynyl-2′-deoxyuridine.

NPCs were generated as previously described^28^. A well-characterized high-quality IPSC culture was enzymatically dissociated using StemPro Accutase (Gibco) and cultured in suspension until day 7 in Embryoid body (EB) medium [Knockout DMEM (Gibco), 20% KOSR (Gibco), 0.1 mM Non-Essential Amino Acids (Gibco), 2 mM Glutamax, 1% Penicillin-Streptomycin (Gibco), and 0.1 mM Betamercaptoethanol (Gibco)]. EB medium was replaced with Neural Induction Medium [DMEM/F12 (Gibco), N2 supplement (Gibco), 8 ng/ml bFGF (Gibco), 1x Glutamax (Gibco), 1x Penicillin-Streptomycin (Gibco), 1x Non-essential Amino Acids (Gibco) and 2 µg/ml Heparin (Sigma)] from day 7 to 14. Subsequently, EBs were plated on Matrigel (Corning) coated dishes and allowed to form neural rosettes. The neural rosettes were then passaged by manual selection and tertiary rosettes were mechanically dissociated through pipetting and plated as an NPC monolayer. The medium was then replaced with Neural Expansion Medium [DMEM/F12 (Gibco), N2 supplement (Gibco), B27 supplement without Vitamin A (Gibco), 8 ng/ml bFGF (Gibco), 1x Glutamax (Gibco), 1x Penicillin-Streptomycin (Gibco), 1x Non-essential Amino Acids (Gibco) and 2 µg/ml Heparin (Sigma)]. Quantitative immunolabelling revealed comparable NPC differentiation from IPSC lines (Nestin: C1–99.2 ± 0.4%, C2–99.3 ± 0.4%, B1–99.1 ± 0.6%, B2–99 ± 0.5%; Pax6: C1–95.2 ± 1.4%, C2, 94.2 ± 2% B1–94.8 ± 2.2%, B2–96.3 ± 1.8%; Fig. 2A).

Gross chromosomal integrity as checked through karyotyping was normal. Mycoplasma contamination was checked regularly with an enzyme-based mycoplasma detection kit (Lonza) as per manufacturer’s instructions.

### Drug treatment in NPCs

Cells were seeded at a density of 100 000 cells/cm^2^ on a tissue culture treated surface additionally coated with Matrigel. Approximately 12 hours post-seeding; the drugs were added to the media [Lithium Chloride (Sigma) at a working concentration of 1 mM or Sodium Valproate (Sigma) at a working concentration of 0.7 mM] or kept untreated. Cellular assays and RNA extraction were performed on day 7. Experiments were performed in biological triplicates.

### Drug treatment in LCLs

Four million LCLs were seeded in each of the three T25 flasks having RPMI-1640 complete media as described before 1) 1 mM Lithium Chloride, or 2) 0.7 mM Sodium Valproate, or kept untreated. Post 7 days of treatment, LCLs were processed for cellular assays. Cells were also used for DNA and RNA extraction. Experiments were performed in biological triplicates.

### Immunocytochemical analyses of NPCs

Cells were fixed in 4% paraformaldehyde (Sigma) for 20 min, permeabilized in 0.1% Triton X-100 (Invitrogen) at room temperature for 10 min and blocked in 3% donkey serum for 40 min. They were then incubated in primary antibodies for 60 min (Supplementary Table 5) followed by secondary antibodies for 30 min (Alexa Fluor dyes, Invitrogen; Supplementary Table 5). The nuclei were counterstained with DAPI (4′,6-diamidino-2-phenylindole, Life technologies) for 5 min and coverslips were mounted on slides with Vectashield (Vector labs). Fluorescent imaging was performed on fields of view containing uniform DAPI staining using a Fluoview 3000 (Olympus) microscope. Images were processed with ImageJ64 (v 1.47) software and immunolabelled cells counted manually by a blinded observer. At least 10 representative images were taken for each experiment, which was done in biological triplicates.

### Whole transcriptome sequencing (RNA-Seq) and analysis

RNA-Seq was performed on the Illumina® Hi-Seq platform as per manufacturer’s protocol. There were two biological replicates available per sample. FastQC (v0.11.5; http://www.bioinformatics.babraham.ac.uk/projects/fastqc) was used for the quality of raw reads. It examines per base and per sequence quality scores, per base and per sequence GC content, per base N content and sequence length distribution. Cutadapt (v2.4) was used to remove adapter contamination in raw reads. The filtered reads were aligned to the human reference genome hg19 (GRCh37) using HISAT (v2.1). SAM to BAM conversion and sorting were done with Samtools 1.3 (https://sourceforge.net/projects/samtools/files/samtools/1.3/). The relative abundance of transcripts, measured as FPKM (Fragments Per Kilobase of transcript per Million mapped reads), was estimated using Cuffdiff (v2.02). Differentially expressed genes in response to lithium treatment were determined from the FPKM values obtained for each gene by calculating the fold change. Genes which showed >2-fold difference with FDR adjusted P-value < 0.05 were considered differentially expressed.

### Mitochondrial membrane potential (MMP) and cell death assay

Live staining with Mitotracker Deep Red (MTDR, Invitrogen, at a working concentration of 100 nM) and a vital dye, Propidium Iodide (Invitrogen) at 15 µg/ml in NPCs, Sytox Green (Invitrogen) at 30 nM in LCLs followed by flow cytometry (BD FACSVerse™) was done to assess MMP and cell death respectively. Verapamil (Sigma) was also included at a working concentration of 5 µM to prevent potential dye leak. Experiments were performed in biological triplicates. FlowJo software was used for the analysis. Gates were applied on scatter plot using FSC vs SSC parameters to remove debris. Finally, quadrant gates were applied using MTDR vs Sytox green signal to analyze the cell population of interest. The change in the percentage of the cell population of interest (gated in Q1 and Q3) was examined to study cell viability and MMP (Supplementary Figure 1B).

Pharmacological agents were included as a positive depolarization control. Treatment of cells with Carbonyl cyanide m-chlorophenyl hydrazone (CCCP) (Sigma), a respiratory uncoupler/protonophore, at 50 µM^29,30^, or 2% paraformaldehyde (Sigma)^31,32^ for 30 minutes resulted in significant reduction of MMP (Supplementary Figure 1C). This validates the use of MTDR to investigate MMP, as has been reported in previous studies^33–36^.

### Mitochondrial area in NPCs

Immunocytochemistry and analysis were performed as described earlier. Individual regions of interest were first chosen based on Nestin staining and mitochondrial immunolabelling with anti-TOMM22 was assessed in binary images.

### Mitochondrial DNA content in LCLs

Total genomic DNA was extracted from LCLs and relative mitochondrial DNA (mtDNA) copy number was estimated by SYBR Green assay in a q-PCR system (Thermofisher). Cytochrome B (*Cyt B*) and NADH dehydrogenase 1 (*ND1*) genes were used to represent the mtDNA, and pyruvate kinase (*PK*) gene was used to represent the nuclear DNA. The primers selected for this experiment were based on an earlier study for mtDNA copy number by Gu *et al*.^24^. Relative mtDNA copy numbers of the genes *Cyt B* and *ND1*, normalized to the single-copy nuclear gene *PK* and relative to the calibrator is given by 2^−ΔΔCt^.

### Cell proliferation/cycle assays

NPC proliferation assay was performed using the Click-it EdU Alexa Fluor 488 imaging kit (Invitrogen) in biological triplicates. EdU was added at a concentration of 10 µM in media incubated for an hour at 37 °C. Subsequently, cells were fixed and immunocytochemistry performed using the EdU detection cocktail and DAPI. The ratio of EDU positive to DAPI positive nuclei gave the percentage of cells in the proliferative phase.

Cell cycle assays were performed in LCLs fixed in 70% ethanol and subsequently incubated with Propidium Iodide dye (Invitrogen) at 15 ug/ml and RNase A (Invitrogen) at 40 ug/ml at 37 °C for 30 min. Flow cytometry was performed as described earlier. FlowJo software was used for the analysis. Appropriate gates were applied on scatter plot using parameters required to remove debris and clumps from analysis while including only singlet cells. A histogram plot was generated for the gated singlet cells. The histogram plot was analyzed to estimate the percentage of cells in different phases of cell-cycle (Supplementary Figure 1D, E).

### Gene expression studies in LCLs

To estimate relative gene expression, total RNA was isolated using Trizol (Ambion) and converted to cDNA using SuperScript VILO cDNA Synthesis Kit (Thermofisher). Pooled RNA from untreated control samples was aliquoted and cDNA was synthesized to prepare calibrator. The calibrator sample was used to calculate the relative quantification (RQ). The use of calibrator also normalizes inter plate assay variation for multiple runs.

The TaqMan gene expression assays were used for relative quantification of the target genes. Housekeeping gene *GAPDH* was used for *BCL2* and *GSK3B* expression, whereas *HPRT* was used for *NR1D1* expression. The q-PCR was duplexed by simultaneous amplification and quantification of both, target gene and housekeeping gene in a single q-PCR reaction. The test sample cDNA along with No Template Control (NTC), calibrator, and Reverse Transcriptase (RT) negative sample were run in triplicates using the Real-time q-PCR system (AB7500; Thermofisher) to achieve the threshold cycle (Ct) value. The calculation used to estimate relative gene expression or RQ value is as follows:

$$\begin{array}{c}\Delta {\rm{Ct}}={\rm{Ct}}\,{\rm{Target}}\,{\rm{gene}}-{\rm{Ct}}\,{\rm{Housekeeping}}\,{\rm{gene}};\\ \Delta \Delta {\rm{Ct}}=\Delta {\rm{Ct}}\,{\rm{Test}}\,{\rm{sample}}-\Delta {\rm{Ct}}\,{\rm{Calibrator}}\,{\rm{sample}};\end{array}$$

The relative gene expression or RQ value, normalized to an endogenous gene and relative to a calibrator, is given by 2^−ΔΔCt^.

### Statistical analysis

Deviance from normal distribution was checked using Shapiro-Wilk test. For normal distributions, one-way analysis of variance (ANOVA) and Student t-test were used for comparisons between controls and cases or between therapeutic response groups. For variables that were not normally distributed, Kruskal-Wallis & Mann-Whitney tests were used for comparison between groups. For *in vitro* treatment experiments also, we applied paired ANOVA and paired Student t-test in normally distributed data. For variables that did not follow normal distribution, the respective non-parametric test was applied, i.e. Friedman test (alternative to paired ANOVA) and Wilcoxon test.

While performing multiple tests, p-values were corrected using the appropriate correction method (viz. Dunn’s, Sidak’s, Tukey’s, and Holm-Sidak’s). All tests were two-tailed and the results with p values ≤ 0.05 were considered statistically significant. Results are presented in means with standard error of means (S.E.M). All statistical analyses were performed using GraphPad Prism version 7.00 for Windows, GraphPad Software, La Jolla California, USA (www.graphpad.com).

## Results

### RNA-Seq analysis of NPCs with and without in vitro lithium treatment

B1 (Lithium non-responder), B2 (Lithium responder), and control (C1 and C2) NPCs were treated *in vitro* with lithium (1 mM) for 7 days, followed by RNA-Seq analysis (Supplementary dataset) to investigate molecular markers of drug response. For a false discovery rate of 0.05, no genes were detected to be differentially expressed across the NPCs, before and after *in vitro* lithium. Other comparisons were underpowered due to the genetic variability between the studied NPCs.

### Mitochondrial membrane potential (MMP) and cell viability assays in NPCs

After exploring for deficits in MMP and cell viability in the NPCs, reversal was attempted with 7 days of *in vitro* lithium (1 mM), and valproate (0.7 mM); followed by flow cytometry analyses. The concentrations were based on the physiological dose range and available literature on cellular changes with *in vitro* drug treatment^7,37,38^.

At baseline, the percentage of cells with high MMP was significantly lower in BD NPCs compared to control NPCs (Fig. 2B); however, there were no significant differences between B1 and B2. *In vitro* drug treatment also showed no differences. Mitochondrial area fraction measured by TOMM22 immunolabeling was similar across all groups (Fig. 2C,D).

BD NPCs had a significantly higher percentage of dead cells compared to controls at baseline (Fig. 2E). However, there were no significant differences between B1 and B2, or after *in vitro* drug treatment on this parameter either.

### Cell proliferation assay in NPCs

Our experiments showed more EdU labelled cells (S phase) in BD NPCs compared to control NPCs (Fig. 2F,G). Similar to previous assays, there were no significant differences between B1 and B2, or after *in vitro* drug treatment.

### Experimental validation of NPC results in larger sample size of LCLs

Our experiments on NPCs suggested a clear cellular phenotype in BD NPCs that however, did not reverse with *in vitro* drug treatment, or correlate with clinical lithium response. Hence, we decided to investigate these results in a peripheral model system, to assess its clinical utility. We used LCLs (an accepted cellular model for BD), from healthy controls and patients from the extremes of the clinical lithium response spectrum.

**Figure 3:**
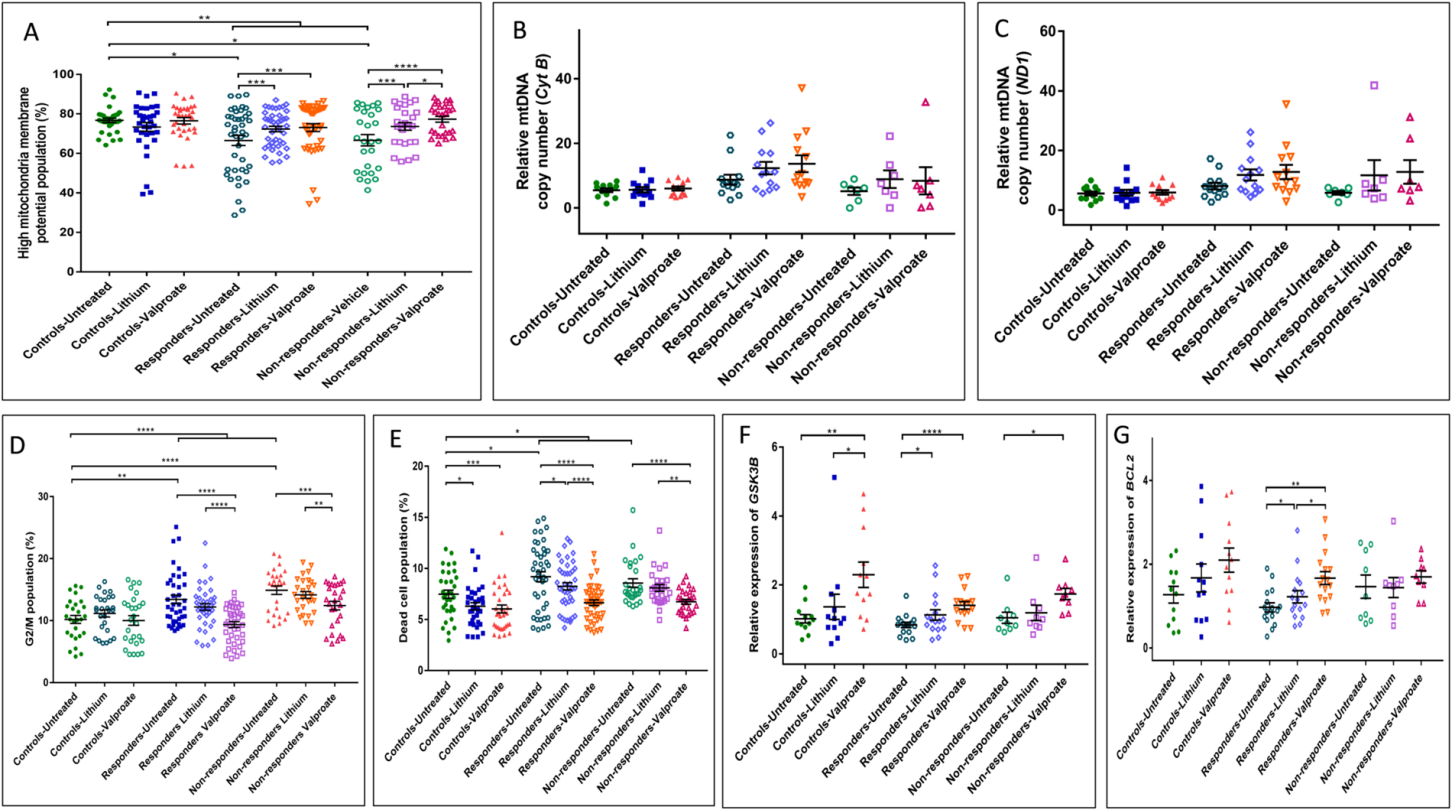
Experiments using LCLs. (**A**) Comparison of high MMP population percentage in the three groups at baseline and after *in-vitro* treatment with lithium (1 mM) or valproate (0.7 mM) for 7 days. (**B**) Comparison of relative quantification values of mitochondrial DNA- *Cyt B*, and (**C**) *ND1*, from qPCR normalized to *PK (*single copy nuclear DNA*)* at baseline and after treatment with lithium. (**D**) Comparison of G2/M population percentage in the three groups at baseline and after *in-vitro* treatment. (**E**) Comparison of dead cell population percentage in the three groups at baseline and after *in-vitro* treatment. (**F**) Relative gene expression of *GSK3B* across groups - comparison for RQ expression values of *GSK3B* gene from qPCR normalized to *GAPDH* (endogenous control). (**G**) Relative gene expression of *BCL2* across groups - comparison for RQ expression values of *BCL2* gene from qPCR normalized to *GAPDH* (endogenous control). All data are shown as mean ± s.e.m.; Experiments were performed in 3 independent experiments for each cell line; N, number of subject LCLs in each group. For group comparisons, initially appropriate ANOVA test was applied and if significant, independent multiple comparison test were performed. Significance level: *p ≤ 0.05, **p ≤ 0.01, ***p ≤ 0.001, ****p ≤ 0.0001. Abbreviations: LCLs, lymphoblastoid cell lines, MTDR, mitotracker deep red; MMP, mitochondrial membrane potential, PI, propidium iodide.

As in the NPCs, the percentage of cells with high MMP was significantly lower in BD LCLs compared to control LCLs (Fig. 3A). But this did not differ significantly between responders and non-responders. Mitochondrial DNA copy numbers in LCLs were also similar across groups (Fig. 3B,C). Interestingly, unlike in NPCs, *in vitro* lithium and valproate reversed the MMP deficit in LCLs derived from both responders and non-responders.

Similar to NPCs, BD LCLs also had greater numbers of proliferative cells (G2/M) in both responders and non-responders (Fig. 3D). The addition of valproate, but not lithium reversed this abnormality.

The dead cell percentage was significantly higher in BD LCLs, and this could be rescued by *in vitro* lithium in clinical responders (Fig. 3E). Valproate could reverse this abnormality in both responders and non-responders.

### Gene expression analysis related to cell viability

We performed LCL gene expression analysis of *BCL2*, *GSK3B* and *NR1D1*. These have been found involved in the mechanism of lithium action/risk for bipolar disorder and cell viability (Supplementary Table 4). The *BCL2* gene encodes for an integral outer mitochondrial membrane protein that regulates cell death by controlling mitochondrial membrane permeability^39^. *GSK3B* is involved in multiple signaling pathways controlling cellular metabolism, differentiation, death, and survival^40^. The *NR1D1* gene, also known as *Rev-ErbAα*, encodes a transcription factor that is a member of the nuclear receptor subfamily 1; deletion of this gene affects the survival of neurons during postnatal development^41^. We found that *in vitro* lithium enhanced the expression of *BCL2* and *GSK3B* specifically in LCLs from clinical lithium responders (Fig. 3F,G), and not in other groups. *NR1D1* expression was similar across groups and remained unchanged after *in vitro* drug treatment.

## Discussion

In BD, genetic factors may underlie risk of occurrence of disease, as well as response to treatment. Therefore, to delineate those mechanisms that could have a bearing on response to lithium, we chose two BD patients from the same family (i.e. shared genetic background) who were discordant in their response to lithium (Fig. 1) and characterized the cellular phenotypes related to disease and lithium treatment. In addition, we also examined cellular phenotypes in LCLs from a set of unrelated patients, and controls.

Mitochondrial dysfunction is one of the most consistently replicated findings across existing literature on BD^18,42^. We also found that the proportion of cells with high MMP is lower in cells derived from BD patients (Figs. 2B, 3A). Independent *in vitro* exposure to either lithium or valproate increased this proportion in patient-derived LCLs (Fig. 3A) but not NPCs (Fig. 2B). Former studies have shown that lithium treatment increased mitochondrial complex activity in leukocytes^43^ while valproate treatment enhanced *NDUFV2* gene expression^44^.

The percentage of proliferating cells were higher in patient-derived cultures [NPCs (Fig. 2G) and LCLs (Fig. 3D)]. Increased cell proliferation in both NPCs and LCLs in BD patients suggests that this is a disease-related marker. Our findings corroborate with previous literature^45–48^ on dysregulation of genes associated with the G1 or G2 checkpoints of the cell cycle in BD. Exposure to *in vitro* lithium did not alter this phenotype, either in LCLs or in NPCs; irrespective of clinical response to lithium. A similar dissonance between clinical response to lithium, and *in vitro* lithium, has been reported by previous researchers^49,50^ in patient-derived T-lymphocytes. Collectively, these findings demonstrate that, although this cell proliferation phenotype strongly correlates with disease, it is not a reliable marker of clinical response to lithium.

Unlike in NPCs, *in vitro* lithium treatment of LCLs from BD helps to maintain cell viability as demonstrated by a decrease in the percentage of dead cells (“Responders-Lithium,” Fig. 3E). We also found an increase in *BCL2* and *GSK3B* expression concomitant with *in vitro* lithium exposure in these LCLs (Fig. 3G). Previous studies have also shown that lymphocytes derived from BD patients have increased expression of the pro-apoptotic BAX protein, and are more prone to apoptosis^50^. GSK3B, a direct target of lithium, has also been shown to modulate cell survival^40,51,52^. *In vitro* lithium exposure (at 1 mM) for eight days has also been previously demonstrated to increase *GSK3B* mRNA^53^. Shorter durations of lithium exposure have not induced changes in *GSK3B* expression in earlier studies^8,53^.

Lithium has been used for the treatment of BD since 1949^54^. Several molecular pathways and cellular processes have been implicated in its actions^55^, including its effect on cell viability^56,57^. Our finding that the addition of *in vitro* lithium is able to reduce the percentage of dead cells, in LCLs from clinical responders alone, suggests that the improvement of impaired cell viability with *in vitro* lithium could be used as a proxy marker for clinical lithium response.

As BD is usually an adolescent/adult onset disorder, it would have been ideal to also examine cortical neurons derived from NPCs. The absence of any effect of *in-vitro* lithium on the NPC based experiments, and lack of significant genome-wide result in the RNA-Seq analysis in our study, along with negative results in PSC derived embryoid bodies from another study^58^, indicate that we may need to use a more differentiated cell type to understand the effects of lithium in BD. Positive results using RNA-Seq have been reported earlier with the same duration and concentration of *in-vitro* lithium exposure in LCLs^15,16^.

The findings of this study, although novel and encouraging, have to be considered in the background of certain limitations. We have used a modest number of cell lines. The patients and controls were not matched for age or sex. The study was conducted in patients who had already been ill and on treatment since many years; they were not medication-naïve. Prospective studies, with greater number of cell lines, from medication-naïve patients and age and sex-matched controls exploring further differentiated cell types would provide more conclusive evidence.

To summarize, our findings indicate that there are cellular phenotypes related to disease (MMP, cell proliferation) in NPCs and LCLs from BD; and that the clinical effectiveness of lithium correlates well with improved cell viability in LCLs following addition of lithium *in vitro*. Confirmation of this finding through prospective studies in treatment-naïve patients who can be followed up after initiation of lithium treatment, would have immense clinical potential. Predicting treatment response to lithium is valuable in initiating appropriate treatment earlier on and improving clinical outcomes.

## Supplementary information

Supplementary Dataset 1.

Supplementary figure and tables.
