# Supplementary material for: Lithium response in bipolar disorder correlates with improved cell viability of patient derived cell lines
Source: Sci Rep. 2020 May 4;10:7428. doi: 10.1038/s41598-020-64202-1 (PMC7198534; doi:10.1038/s41598-020-64202-1)
Supplement: Supplementary file 2 — Supplementary figure and tables. [file 41598_2020_64202_MOESM2_ESM.pdf]

Title

Lithium response in bipolar disorder correlates with improved cell viability of patient derived cell lines

Authors: Pradip Paul[1], Shruti Iyer[2], Ravi Kumar Nadella[1], Rashmitha Nayak[1], Anirudh S. Chellappa[1], Sheetal Ambardar[2,3], Reeteka Sud[1], Salil K. Sukumaran[1], Meera Purushottam[1], Sanjeev Jain[1,3], ADBS Consortium (ADBS: The Accelerator program for Discovery in Brain disorders using Stem cells), Biju Viswanath[1]*

*Corresponding author

1. The National Institute of Mental Health and Neurosciences (NIMHANS), India

2. Institute for Stem Cell Science and Regenerative Medicine (InStem), India

3. The National Centre for Biological Sciences (NCBS), India

<u>Accelerator Program for Discovery in Brain disorders using Stem cells (ADBS) Consortium</u>

Naren P. Rao[1], Janardhanan C. Narayanaswamy[1], Palanimuthu T. Sivakumar[1], Arun Kandasamy[1], Muralidharan Kesavan[1], Urvakhsh Meherwan Mehta[1], Ganesan Venkatasubramanian[1], John P. John[1], Odity Mukherjee[2], Ramakrishnan Kannan[1], Bhupesh Mehta[1], Thennarasu Kandavel[1], B. Binukumar[1], Jitender Saini[1], Deepak Jayarajan[1], A. Shyamsundar[1], Sydney Moirangthem[1], K. G. Vijay Kumar[1], Jagadisha Thirthalli[1], Prabha S. Chandra[1], Bangalore N. Gangadhar[1], Pratima Murthy[1], Mitradas M. Panicker[3], Upinder S. Bhalla[3], Sumantra Chattarji[2], Vivek Benegal[1], Mathew Varghese[1], Janardhan Y. C. Reddy[1], Padinjat Raghu[3], Mahendra Rao[2]

1. National Institute of Mental Health and Neuro Sciences (NIMHANS), India

2. Institute for Stem Cell Biology and Regenerative Medicine (InStem), India

3. National Center for Biological Sciences (NCBS), India

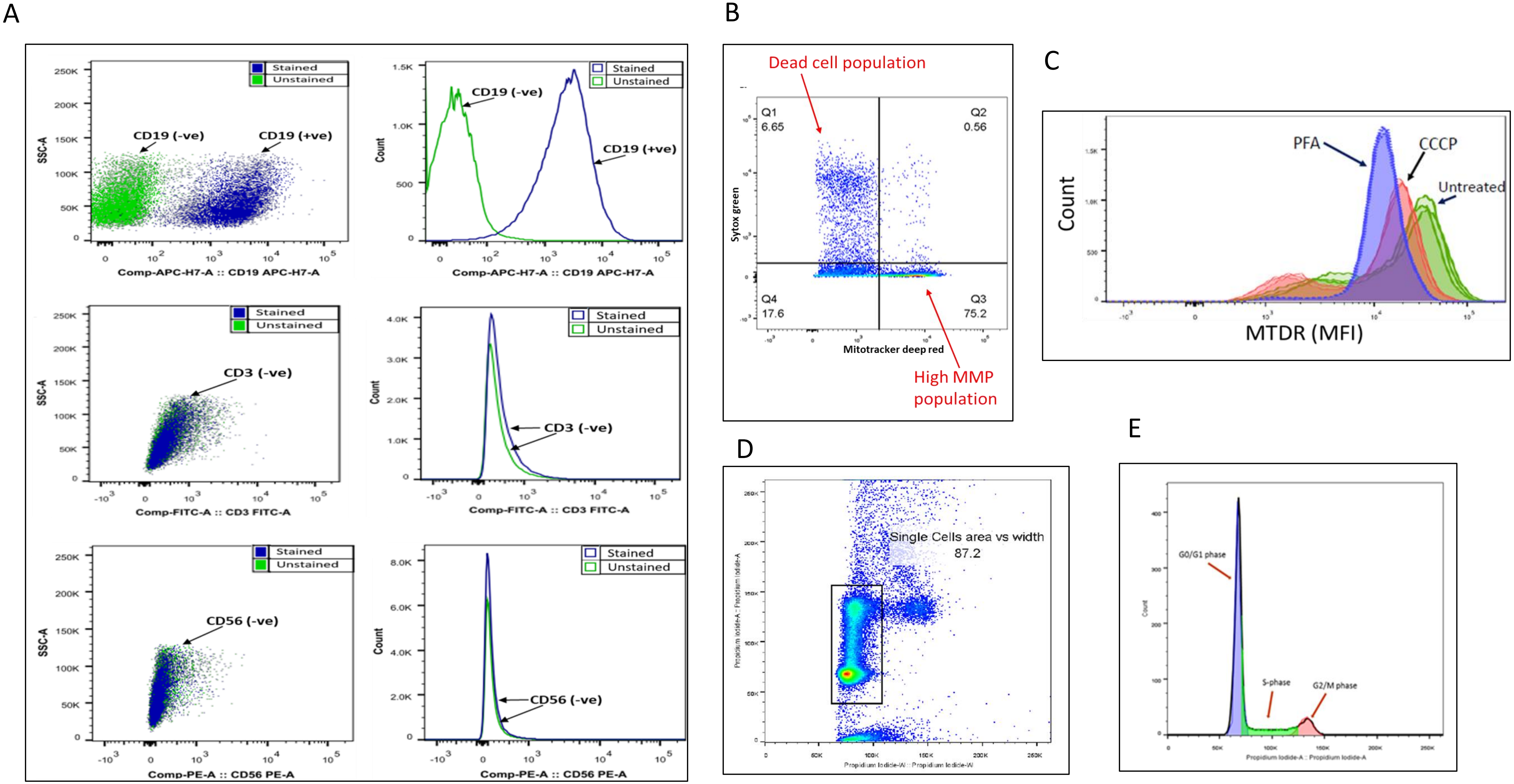

Supplementary figure 1. A) Characterization of LCLs by immunophenotyping- Scatterplot and histogram plot showing LCLs positive for *CD19* (B cell marker) (top panel), negative for *CD3* (T cell marker) (middle panel), negative for *CD56* (NK cells) (bottom panel). B) Representative flow cytometer scatter plot for mitochondrial potential and cell viability assay**.** Dot plot for MTDR signal against Sytox Green, further gated into four quadrants based on live or dead cells (Sytox positive indicate dead cells) and high or low MMP population (based on MTDR signal intensity). The percentage of cells in Q1 (dead cell) and Q3 (live cells with high MMP) were analyzed further to assess cell viability and MMP respectively. C) Mitochondrial membrane depolarization positive control experiment- Histogram plot showing the change in mean MFI of MTDR after incubation with CCCP (50uM) and PFA (2%). D) Representative flow cytometer scatter plot for cell cycle assay. Dot plot for PI**,** to show gating of single cells using width versus area parameters. E) Illustrates PI area parameter histogram plot of the singlet cells to determine percentage of cells in G0/G1, S and G2/M phases of the cell cycle using FlowJo software. Abbreviations: LCLs, lymphoblastoid cell lines, PBMCs, peripheral blood mononuclear cells, EBV, Epstein-barr virus, MFI, mean fluorescence intensity, MTDR, mitotracker deep red, CCCP, carbonyl cyanide m-chlorophenyl hydrazine, PFA, paraformaldehyde.

Supplementary table 1: Important studies related to mitochondrial function, cell death and cell proliferation in bipolar disorder

Author and year	Sample	Methodology	Significant results
**Studies related to mitochondrial function**			
Konradi et al., 2004 [1]	9 BD and 10 control hippocampus from PM brains	Gene array to study mRNA expression in BD & control hippocampus	Expression of nuclear mRNA coding for mitochondrial proteins regulating oxidative phosphorylation in complexes I-V & ATP- dependent process were downregulated in hippocampus of BD.
Iwamoto, Kakiuchi, Bundo, Ikeda, & Kato, 2004 [2]	PM brain tissues:11 BD and 15 controls;<br>LCLs: 14BD & 11 controls	mRNA levels analysis in BD PM brain tissues and LCLS	Altered mRNA levels of proteins involved in aberration of protein translocation system into mitochondria (affecting mitochondrial function) in LCLs and brain tissues of BD.
Andreazza, Shao, Wang, & Young, 2010 [3]	15 each post-mortem DLFC from BD patients & control	Investigated ETC complex I activity & oxidative damage to mitochondrial proteins along with levels of complex I subunit NDUFS7	Levels of NDUFS7 & complex I activity decreased significantly in BD. Protein oxidation & 3-nitrosine increased in BD compared to controls.
Regenold et al., 2012 [4]	PM brain cortex tissue from 15BD and controls	Studied Hexokinase1 (HK1) attachment to outer mitochondrial membrane (OMM) in BD brain tissue	Decreased HK1 attachment in BD compared to controls. HK1 attachment to OMM, a critical feature of brain energy metabolism and survival of neurons through prevention apoptosis.
Gubert et al., 2013 [5]	Plasma and PBMC samples from 12BD and 30HC.	Evaluated oxidative stress marker in plasma and ETC complex activities in PBMCs of BD	No significant difference in oxidative stress markers and ETC complex activities between BD and HC.
de Sousa et al., 2015 [6]	24 HC and 25 BD;<br>Patients treated with lithium for 6 weeks	Evaluated leukocyte ETC complex activities in BD & effect of lithium on ETC	No significant differences in mitochondrial ETC complex activities between BD and HC. Lithium treatment significantly increased mitochondrial complex I activities.
Yoshimi et al., 2016 [7]	CSF: 54 BD & 40 controls;<br>PM brain tissue: 35BD & 34HC.	Evaluated the association of iso-citrate with BD	Iso-citrate levels significantly Increased in CSF from BD. mRNA levels of iso-citrate dehydrogenase in BD PM tissue was low compared to controls

Scaini et al., 2017 [8]	PBMC from 16BD and 16HC	Analysed levels of mRNA, protein and activity of mitochondrial related factors in PBMCs of BD and HC.	Levels of anti-apoptotic proteins and citrate synthase activity were significantly lower, while caspase activity was higher in PBMC of BD.<br>Levels of mRNA, protein related to mitochondria fusion were lower & related to fission were higher in PBMC of BD.<br>Showed mitochondrial dynamics & cell death pathway activation in BD, supporting link between mitochondria & pathophysiology of BD**.**
Bosetti et al., 2002 [9]	Rat-Lithium treatment for 7 days (acute) or 42 days (chronic).	Gene expression analysis of lithium treated rat brain	Chronic treatment at therapeutic concentration altered expression of several genes regulating mitochondrial enzymes in rat brain.
Lai, Zhao, Warsh, & Li, 2006 [10]	Human SH-SY5Y neuroblastoma (1 or 7 days of treatment)	Looked into the effect of lithium (1mM) or valproate (0.6mM) in stress induced human neuroblastoma cells	Pretreatment of SH-SY5Y cells for 7 days with lithium or valproate significantly reduced rotenone or H_2O_2 induced cytochrome C release, caspase activity & cytotoxicity and upregulated BCL2 protein level.<br>No effect was reported on 1 day treatment with the lithium or valproate.
Washizuka, Iwamoto, Kakiuchi, Bundo, & Kato, 2009 [11]	1) LCLs from 25 BDI, 10 BDII & 33 HC.<br>2) 4 HC LCLs for lithium (0.75mM) or VPA (100ug/mL) treatment experiment	Studied gene expression of NDUFV2 in LCLCs of BD & HC; and after treatment of HC LCLS with lithium or VPA for 24hrs or 7 days	1) NDUFV2 gene expression was significantly downregulated in BDI and upregulated in BDII compared to HC.<br>2) VPA treatment significantly increased NDUFV2 compared to vehicle.
Maurer, Schippel, & Volz, 2009 [12]	Human PM brain cortex from 5 Controls treated with lithium (0.1mM-10mM) for 10min	Investigated the effect of lithium on respiratory chain enzyme activities after exposure to lithium in human brain tissue	ETC complexes were significantly increased dose dependently by lithium with max at 1mm.<br>Succinate dehydrogenase was significantly increased at higher concentration of lithium

Bachmann et al., 2009 [13]	1) SH-SY5Y cell line;<br>2) Brains from adult male Wistar Kyoto rats	Examined the effects of mood stabilizers on mitochondrial function and against mitochondrial mediated neurotoxicity	Cell respiration rate was enhanced by long term treatment with lithium or VPA. Mitochondrial function (membrane potential & oxidation) was enhanced by chronic lithium or VPA treatment in SH-SY5Y cells. *In vivo*: long-term treatment with lithium or VPA at therapeutic concentration prevented methamphetamine (meth) induced toxicity at the mitochondrial level (mitochondrial cytochrome c, anti-apoptotic Bcl-2/Bax ratio and COX activity).<br>Oligo array analysis: pre-treatment with lithium or VPA prevented meth induced dysregulation of gene expression of proteins related to apoptotic pathway and mitochondrial functions. *BCL2* expression increased after 6 days treatment.
Cataldo et al., 2010 [14]	1) PM prefrontal cortex: 10 BD & 10 controls;<br>2) Fibroblasts: 8 BD & 8 HC;<br>3) LCLs: 6 BD & 6 HC	Evaluated structure and distribution of mitochondria in BD compared to controls; Effect of therapeutic dosage of lithium in fibroblast after 5 days treatment	Ultra-structure examination revealed smaller mitochondrial areas in BD brain PFC.<br>Altered mitochondria morphology & distribution was reported for BD fibroblasts and LCLs. Significant differences in cytochrome C distribution in BD fibroblasts.<br>No significant differences in mitochondrial differences in either groups on treatment. However, significant difference for distribution of mitochondria between treated BD and treated HC.
Sitarz et al., 2014 [15]	Fibroblasts from 5 POLG-deficient patients & 3 HC	Effect of 10mM VPA for 10 days on mitochondria associated proteins	VPA treatment increased mtDNA copy number. Protein levels of genes involved in mtDNA maintenance (POLG), mitochondria biogenesis & OXPHOS (COX2) increased significantly.
da Costa, Kormann, Galina, & Rehen, 2015 [16]	Neural progenitor cells from human embryonic stem cells	Effect of VPA (0.01,0.1, 1mM) for 24hours on cell size, mitochondrial morphology and function	Cell size and mitochondrial morphology changes after 1mM VPA treatment. Mitochondrial membrane potential (MMP) decreased with 1mM VPA.

Mertens et al., 2015 [17]	6BD and 4HC dentate gyrus neurons derived from fibroblasts	Studied the mitochondria morphology and function in derived neurons from BD and HC.	Increased MMP and mitochondrial gene expression in BD neurons.<br>Size of neuronal mitochondria was smaller in BD compared to HC.<br>Lithium treatment increased mitochondrial size in lithium responsive neurons, whereas MMP remained unaffected.
Kakiuchi et al., 2005 [18]	30 controls and 27 BD frontal cortex from PM brain tissue	Examined mitochondrial DNA (mtDNA) copy number in BD	No significant difference in mtDNA copy number of PM brain tissues between BD and controls.
Vawter et al., 2006 [19]	PM brain tissues from 9BD & 20controls	Analysed mitochondrial related gene expression and mtDNA copy numbers in BD and controls	1) Mitochondrial gene & nDNA encoded mitochondrial genes were differentially expressed in BD. Mitochondrial related gene expression different in BD with lithium prescription Vs. BD without lithium at the time of death. The mtDNA copy number was non-significantly increased in BD compared to controls.
Sabunciyan et al., 2007 [20]	PM frontal cortex from 40BD & 44 controls	Examined mtDNA copy number in BD & controls	No significant difference in mtDNA copy number of PM brain tissues between BD and controls.
Torrell et al., 2013 [21]	PM brain tissues from 15BD & 15 controls	Examined mtDNA copy number and MT-ND1 gene expression in BD & controls	MT-ND1 gene expression was significantly increased in BD compared to controls. No significant difference for mtDNA content in PM brain tissues between BD and controls
C. C. Chang, Jou, Lin, & Liu, 2014) [22]	Leukocyte from 40 BD & 70 HC	Investigated mtDNA & oxidative damage in BD and HC leukocytes.	Leukocyte mtDNA copy number in BD was significantly lower than HC. Mitochondrial oxidative damage was significantly higher in BD compared to controls.
de Sousa et al., 2014) [23]	Leukocyte from 24 HC & 23 BD in depressive episode.	Evaluated mtDNA content in BD & HC. And tested if the content in BD varied after 6weeks of lithium treatment.	No significant difference in mtDNA copy number between BD and HC at baseline. No difference was reported even after 6 weeks of lithium treatment in BD cases.
Gabriel R. Fries et al., 2017 [24]	Peripheral blood samples from 22 BDI & 20 HC	Evaluated mtDNA copy number in BD & HC	The mtDNA copy number distribution in peripheral blood was significantly different in BD compared to HC. (Notably high variability in distribution was reported for BD group).

David Stacey et al., 2018 [25]	Peripheral blood samples from 50 BD (10 lithium responders and 40 lithium non-responders)	Comparison of mRNA expression levels between lithium responders and non-responders	43 mRNA levels downregulated in lithium responders- includes mitochondrial encoded genes- MT-ND1, MT-ATP6, MT-CyB.<br>Genes involved in mitochondrial function pathway (ETC, OXPHOS) overexpressed.
**Studies related to cell death**			
Shao & Vawter, 2008 [26]	PM DLPFC from 29 BD and 27 controls	Gene expression array analysis in brain tissues from BD & HC	Genes involved in nervous system development, cell growth, & cell death were dysregulated.
McCurdy et al., 2006 [27]	Olfactory mucosa from 8 BD & 10 HC	Examined the rate of cell death in BD compared to HC	Cell death was significantly more in BD compared to HC.
F M Benes, Matzilevich, Burke, & Walsh, 2006 [28]	PM brain tissues from 9 BD& 10 controls.	Gene expression array analysis in brain tissues from BD and controls	19 of 44 genes related to apoptosis were upregulated. Antioxidant related genes were downregulated.
Herberth et al., 2011 [29]	PBMCs from 16 BDI, 16BDII & 32HC; Validation in 7 BDI, 7 BDII & 14 HC.	Tested proteome profiling in PBMCs from BD & HCs. And effect of BD serum analytes on PBMCs from HC.	Proteome profiling of PBMC revealed differentially expressed proteins involved in cell death and survival pathways.<br>Addition of BD serum analytes on PBMCs from HC subjects reduced cell survivality.
Kazuno et al., 2013 [30]	1) LCLs from monozygotic twins discordant for BD,<br>2) 8 LCLs each from BD & HC to validate	Evaluated protein markers in whole cell lysate derived from LCLs of twins & validated.	Several proteins involved in cell death & glycolysis was significantly differentially expressed between the patient and the co-twin. Case- control analysis validated only upregulation of PGAM1 (involved in energy metabolism) in BD cases.
Gabriel Rodrigo Fries et al., 2014 [31]	PBMC from 10 BD and 7 HC	Assessed cell death & viability in PBMCs of BD & HC	Cells in early apoptosis was significantly higher in BD.<br>No significant difference in cell viability, late apoptosis & necrosis between BD and HC.
Marianthi, Olga, Aristotelis, Nikolaos, & Fragiskos, 2015 [32]	Skin fibroblasts from 10 BD & 5 HC	Transcriptome profiling in fibroblasts from BD and HC	Genes involved in positive regulation of apoptotic process & cell cycle were differentially expressed in BD compared to HC.

Wollenhaupt-Aguiar et al., 2016 [33]	Neurons differentiated from neuroblastoma cell line (SH-SY5Y) challenged with serum of 12 BD or 6 HC	Investigated whether biochemical changes in the serum of patients induces neurotoxicity in neuronal cell cultures.	Reduced neurite density in neurons treated with serum of BD patients. Neurons challenged with serum of late stage patients showed significant decrease in cell viability
Xiaohua Li, Bijur, & Jope, 2002 [34]	Review article	Reviewed articles related to lithium or VPA treatment in human cell lines	Higher concentration of lithium reduced GSK3B activity & blocked facilitation of apoptosis.<br>VPA provided protection from apoptosis by inhibiting pro-apoptotic (such as reduced caspase 3 activity).
A. J. Kim, Shi, Austin, & Werstuck, 2005 [35]	Human hepatocarcinoma cell line (HEPG2)	Studied the ER stress induced dysfunction after pre-treatment with 0.5mM VPA for 18hours	Pre-treatment with VPA increased the cellular resistance to ER stress induced dysfunction and protects from apoptosis by inhibiting GSK3B.
Lai et al., 2006 [10]	Human SH-SY5Y neuroblastoma (1 or 7 days of treatment)	Looked into the effect of lithium (1mM) or valproate (0.6mM) in stress induced human neuroblastoma cells	Pretreatment of SH-SY5Y cells for 7 days with lithium or valproate significantly reduced rotenone or H_2O_2 induced cytochrome C release, caspase activity& cytotoxicity and upregulated BCL2 protein level. No effect was reported on 1 day treatment with the lithium or valproate.
Wilot et al., 2007 [36]	Hippocampal slices of rats	Evaluated neuroprotective effect of lithium & VPA against ATP induced cell death in rat hippocampus	ATP induced cell death was significantly reduced by lithium or VPA treatment at therapeutic dosage in both *in vitro* (acute) and *in vivo* (chronic) experiments.
Go et al., 2011 [37]	Neuronal progenitor cells (NPC) from embryonic brain of rats	Examined regulation of apoptotic cell death in rat NPCs by VPA	VPA (0.2, 0.5mM) treatment decreased NPC cell death after growth factor withdrawal or H2O2 stimulated peroxide conditions. VPA upregulated BCL-XL protein & mRNA levels in concentration dependent manner and suppressed Bax levels. The result was confirmed by *in vivo* in developing rat brains.

Lowthert et al., 2012 [38]	8 weeks of open label lithium study in 20 BD patients	To assess the change in gene expression in peripheral blood of lithium responders and non-responders	127 genes differentially expressed between responders and non-responders.<br>Pathway analysis showed regulation of apoptosis was significantly affected.<br>Upregulation of anti-apoptotic gene *BCL2* and downregulation of pro-apoptotic genes (*BAD*, *BAK1*) in responders and inverse relation in non-responders after 4 weeks.
Gawlik-Kotelnicka, Mielicki, Rabe-Jabłońska, Lazarek, & Strzelecki, 2016 [39]	Human neuroblastoma cell line (SH-SY5Y)	Assessed the effect of lithium (0.5 & 0.7mmol/L) for 24 hours in neuroblastoma cells.	Cell viability was significantly higher in therapeutic treated lithium samples than vehicle.
Del Grosso et al., 2016 [40]	Human oligodendrocyte cell line	Effect of lithium pre-treatment on cell viability	Psychosine induced autophagy in cells was rescued on lithium pre-treatment, by increasing cell viability.
Z. Li et al., 2017 [41]	Human neuroblastoma cell line (SH-SY5Y)	Effect of VPA in ER stress induced neuroblastoma cell lines on exposure to thapsigargin (TG) and on neuroprotection.	VPA treatment improves cell viability and reduces cell apoptosis in cell exposed to TG. ER stress induced apoptosis response protein were inhibited by VPA treatment. VPA upregulated the ratio of BCL2/Bax proteins in SH-SY5Y cells. VPA promotes cell proliferation through PI3K, AKT, GSK3B pathways.
Breen et al., 2016 [42]	LCLs from 23 Caucasian individual (8 BD lithium responders, 8 BD lithium nonresponders, 7 HC)	Exploring the effect of lithium 1mM (7 days) on transcriptome levels in LCLS from BD lithium response patients	Differential gene expression in apoptosis signalling system, defence response, protein processing pathways and response to ER stress pathways were discovered on treatment with lithium.
**Studies related to cell proliferation**			
McCurdy et al., 2006 [27]	Biopsies of olfactory mucosa from 8 BD & 10 HC	Explored the cell proliferation rates in BD compared to HC	No significant differences in mitosis between the BD and HC, however 11 genes involved in cell proliferation & 4 in neurogenesis were differentially expressed in BD. Cell death was significantly more in BD compared to HC.

F. M. Benes et al., 2007 [43]	Hippocampus tissues from PM brain of 7 BD & 7 controls	Gene expression profiling of brain tissues from BD and controls	*GAD67* (glutamate decarboxylase 67) gene expression was significantly decreased in BD than controls and *CCND2* (Cyclin D2) is known to regulate GAD67. *CCND2* was significantly downregulated in CA2/3 region of brain tissues in BD.
Francine M Benes, Lim, & Subburaju, 2009 [44]	Hippocampus tissues from PM brain of 7 BD & 7 controls	Evaluate the expression profiling of genes involved in G1 & G2 checkpoints of BD	Genes associated with transcriptional complex & G1 or G2 checkpoint of cell cycle regulation in BD was differentially expressed. Gene included *CCND2*, *CDK9* for G1 check point & *P53*, *CHK2*, *CCNE* for G2 checkpoint.
Marianthi et al., 2015 [32]	Skin fibroblasts from 10 BD & 5 HC	Transcriptome profiling in fibroblasts from BD and HC	Genes involved in positive regulation of apoptotic process & mitotic cell cycle were differentially expressed in BD compared to HC.
K. H. Kim et al., 2015 [45]	NPCs and matured neurons from 8 BD & 4 unaffected siblings	Transcriptomic microarray profiling in NPCs and matured neurons (early & late neurons)	Genes related to cell cycle were differentially expressed in BD late neurons compared to unaffected individuals.
Breen et al., 2016 [42]	LCLs from 23 Caucasian individual (8 BD lithium responders, 8 BD lithium nonresponders, 7 HC)	Exploring the effect of lithium 1mM (7 days) on transcriptome levels in LCLS from BD lithium response patients	Gene markers related to cell cycle and nucleotide excision repair were found to be differential in response to lithium between BD lithium responders and non-responders.
Mao, Hoang, & Dicorleto, 2001 [46]	Bovine aortic endothelial cells	Investigated the effect of lithium (5 & 10 mM) on regulation of cell cycle in bovine cells.	Lithium treatment increased G2/M cells without affecting cell viability up to 3 days whereas reduced thereafter.<br>Lithium increased mRNA and protein levels of p21, cyclin dependent kinase inhibitors.<br>Cyclin D mRNA expression was biphasic on lithium treatment--at 4 & 8 hours: It upregulated whereas at 24 & 48 hours: It was downregulated.

Sun et al., 2007 [47]	Human prostate cancer cells	Studied the effect of lithium in cell proliferation of prostate cancer cells.	Lithium (10mM) significantly inhibited cell proliferation at 72 hours treatment. Lithium significantly increased the percentage of cells in S phase & decreased cellular DNA replication.<br>Lithium altered expression of gene regulating DNA replication & Cell cycle. Cyclin A, Cyclin E, *P21* was downregulated whereas Cyclin D was upregulated. Protein level of Cyclin D was also up.
Seelan, Khalyfa, Lakshmanan, Casanova, & Parthasarathy, 2008 [48]	Human neuronal cell line (SK-N-AS)	Microarray expression profiling in human neuronal cell line after lithium (1.5mM) treatment for 33 days	Gene related to neuronal survival, growth, apoptosis, cell cycle regulation were differentially expressed on treatment with lithium.
Zanni et al., 2015 [49]	NPCs from mice	Studied the effect of lithium (1mM or 3mM) in mice NPCs	Lithium attenuated the effect of irradiation exposure induced cell cycle arrest in G1 and G2 phase.
Rattanawarawipa, Pavasant, Osathanon, & Sukarawan, 2016 [50]	Stem cells from human exfoliated deciduous teeth	Evaluated effect of lithium on cell proliferation in the cells after 3 days and 7 days treatment	Lithium significantly reduced colony forming unit ability/ proliferation in dose dependent manner. Lithium increased percentage of cells in subG0 phase, whereas decreased the percentage of cells in G1 phase after 3 days & 7 days of treatment.
Laeng et al., 2004 [51]	Rat neural stem cells	RNA profiling and protein analysis of rat neural stem cell after VPA or lithium treatment	Cell cycle regulating genes – *CCND2* was 5-6 fold increased on treatment by VPA, confirmed by protein analysis.<br>Lithium treatment for 3 days increased *CCND2* levels.
Catalano et al., 2005 [52]	Human papillary thyroid carcinoma cell line	Tested the effect of VPA on cell cycle phases of human carcinoma cell line.	VPA increased subG1 population in time and dose (0.5-3mM) dependent manner. Growth arrest in G1 phase was increased by VPA (1 &3mM) treatment.<br>Gene expression of *P21* and Cyclin A was increased. G2/M cell population was decreased non-significantly by VPA treatment.
X. N. Li et al., 2005 [53]	Human medullo-blastoma and	Investigated the effect of VPA (1 & 2.7mmol/L) on cell cycle phases of cell lines	VPA treatment caused cell cycle arrest for medulloblastoma cell line on day 7, i.e., significantly increased percentage of cells in G0/G1 phase & decreased cells in G2/M phase.

	neuroectodermal tumour cell lines		
Wu & Guo, 2008 [54]	Immortalized human endometrial stromal cells	Examined the effect of VPA on cell cycle phases of stromal cell lines	VPA (3mM) for 16 hours treatment increased percentage of cells in G0/G1 phase and decreased cells in S phase & G2/M phases.
Witt et al., 2013 [55]	Primary murine prostate cancer cells (PCA) and fibroblasts	Studied the effect of VPA on *CCND2* expression in murine cell lines	VPA treatment highly increased *CCND2* gene expression in PCA cell line, however no effect of VPA on *CCND2* expression was seen in murine fibroblast.
Claudia Morich, 2016 [56]	Tumour cell lines	Expression profiling after VPA treatment	*CCND2* gene expression was significantly increased after VPA treatment.
Pietruczuk, Lisowska, Grabowski, Landowski, & Witkowski, 2018 [57]	T cells from 18 BD & 10 HC	Evaluated proliferation capacity & susceptibility to apoptosis in T cells and effect of lithium or valproate on these parameters	Cell cycle longer in BD compared to HC; reduced proliferation in lithium treated BD patients compare to HC and BD treated with VPA.<br>Cell cycle longer in patients treated with VPA compared to lithium treated patients.<br>*In vitro* exposure to VPA reduced cell division and cell proliferation irrespective of the disease state; lithium has no effect on proliferating capacity of T cells from BD patients. Higher doses of lithium shortened cell cycle.<br>Apoptosis higher in BD cells, lithium and VPA prevents apoptosis in T cells from BD.<br>BCl2 level: No significant difference between BD and HC

Bibliography:

1. Konradi C, Eaton M, MacDonald ML, Walsh J, Benes FM, Heckers S. Molecular Evidence for Mitochondrial Dysfunction in Bipolar Disorder. Arch Gen Psychiatry. 2004;61:300.

2. Iwamoto K, Kakiuchi C, Bundo M, Ikeda K, Kato T. Molecular characterization of bipolar disorder by comparing gene expression profiles of postmortem brains of major mental disorders. Mol Psychiatry. 2004;9:406–416.

3. Andreazza AC, Shao L, Wang J-F, Young LT. Mitochondrial Complex I Activity and Oxidative Damage to Mitochondrial Proteins in the

Prefrontal Cortex of Patients With Bipolar Disorder. Arch Gen Psychiatry. 2010;67:360.

4. Regenold WT, Pratt M, Nekkalapu S, Shapiro PS, Kristian T, Fiskum G. Mitochondrial detachment of hexokinase 1 in mood and psychotic disorders: Implications for brain energy metabolism and neurotrophic signaling. J Psychiatr Res. 2012;46:95–104.

5. Gubert C, Stertz L, Pfaffenseller B, Panizzutti BS, Rezin GT, Massuda R, et al. Mitochondrial activity and oxidative stress markers in peripheral blood mononuclear cells of patients with bipolar disorder, schizophrenia, and healthy subjects. J Psychiatr Res. 2013;47:1396–1402.

6. de Sousa RT, Streck EL, Zanetti M V., Ferreira GK, Diniz BS, Brunoni AR, et al. Lithium increases leukocyte mitochondrial complex I activity in bipolar disorder during depressive episodes. Psychopharmacology (Berl). 2015;232:245–250.

7. Yoshimi N, Futamura T, Bergen SE, Iwayama Y, Ishima T, Sellgren C, et al. Cerebrospinal fluid metabolomics identifies a key role of isocitrate dehydrogenase in bipolar disorder: evidence in support of mitochondrial dysfunction hypothesis. Mol Psychiatry. 2016;21:1504–1510.

8. Scaini G, Fries GR, Valvassori SS, Zeni CP, Zunta-Soares G, Berk M, et al. Perturbations in the apoptotic pathway and mitochondrial network dynamics in peripheral blood mononuclear cells from bipolar disorder patients. Transl Psychiatry. 2017;7:e1111.

9. Bosetti F, Seemann R, Bell JM, Zahorchak R, Friedman E, Rapoport SI, et al. Analysis of gene expression with cDNA microarrays in rat brain after 7 and 42 days of oral lithium administration. Brain Res Bull. 2002;57:205–209.

10. Lai JS, Zhao C, Warsh JJ, Li PP. Cytoprotection by lithium and valproate varies between cell types and cellular stresses. Eur J Pharmacol. 2006;539:18–26.

11. Washizuka S, Iwamoto K, Kakiuchi C, Bundo M, Kato T. Expression of mitochondrial complex I subunit gene NDUFV2 in the lymphoblastoid cells derived from patients with bipolar disorder and schizophrenia. Neurosci Res. 2009;63:199–204.

12. Maurer IC, Schippel P, Volz H-P. Lithium-induced enhancement of mitochondrial oxidative phosphorylation in human brain tissue. Bipolar Disord. 2009;11:515–522.

13. Bachmann RF, Wang Y, Yuan P, Zhou R, Li X, Alesci S, et al. Common effects of lithium and valproate on mitochondrial functions: Protection against methamphetamine-induced mitochondrial damage. Int J Neuropsychopharmacol. 2009;12:805–822.

14. Cataldo AM, Mcphie DL, Lange NT, Punzell S, Elmiligy S, Ye NZ, et al. Abnormalities in mitochondrial structure in cells from patients with bipolar disorder. Am J Pathol. 2010;177:575–585.

15. Sitarz KS, Elliott HR, Karaman BS, Relton C, Chinnery PF, Horvath R. Valproic acid triggers increased mitochondrial biogenesis in

POLG-deficient fibroblasts. Mol Genet Metab. 2014;112:57–63.

16. da Costa RFM, Kormann ML, Galina A, Rehen SK. Valproate Disturbs Morphology and Mitochondrial Membrane Potential in Human Neural Cells. Appl Vitr Toxicol. 2015;1:254–261.

17. Mertens J, Wang Q-W, Kim Y, Yu DX, Pham S, Yang B, et al. Differential responses to lithium in hyperexcitable neurons from patients with bipolar disorder. Nature. 2015;527:95–99.

18. Kakiuchi C, Ishiwata M, Kametani M, Nelson C, Iwamoto K, Kato T. Quantitative analysis of mitochondrial DNA deletions in the brains of patients with bipolar disorder and schizophrenia. Int J Neuropsychopharmacol. 2005;8:515.

19. Vawter MP, Tomita H, Meng F, Bolstad B, Li J, Evans S, et al. Mitochondrial-related gene expression changes are sensitive to agonal-pH state: implications for brain disorders. Mol Psychiatry. 2006;11:663–679.

20. Sabunciyan S, Kirches E, Krause G, Bogerts B, Mawrin C, Llenos IC, et al. Quantification of total mitochondrial DNA and mitochondrial common deletion in the frontal cortex of patients with schizophrenia and bipolar disorder. J Neural Transm. 2007;114:665–674.

21. Torrell H, Montaña E, Abasolo N, Roig B, Gaviria AM, Vilella E, et al. Mitochondrial DNA (mtDNA) in brain samples from patients with major psychiatric disorders: Gene expression profiles, MtDNA content and presence of the MtDNA common deletion. Am J Med Genet Part B Neuropsychiatr Genet. 2013;162:213–223.

22. Chang C-C, Jou S-H, Lin T-T, Liu C-S. Mitochondrial DNA variation and increased oxidative damage in euthymic patients with bipolar disorder. Psychiatry Clin Neurosci. 2014;68:551–557.

23. de Sousa RT, Uno M, Zanetti M V., Shinjo SMO, Busatto GF, Gattaz WF, et al. Leukocyte mitochondrial DNA copy number in bipolar disorder. Prog Neuro-Psychopharmacology Biol Psychiatry. 2014;48:32–35.

24. Fries GR, Bauer IE, Scaini G, Wu M-J, Kazimi IF, Valvassori SS, et al. Accelerated epigenetic aging and mitochondrial DNA copy number in bipolar disorder. Transl Psychiatry. 2017;7:1283.

25. Stacey D, Schubert KO, Clark SR, Amare AT, Milanesi E, Maj C, et al. A gene co-expression module implicating the mitochondrial electron transport chain is associated with long-term response to lithium treatment in bipolar affective disorder. Transl Psychiatry. 2018;8:183.

26. Shao L, Vawter MP. Shared Gene Expression Alterations in Schizophrenia and Bipolar Disorder. Biol Psychiatry. 2008;64:89–97.

27. McCurdy RD, Féron F, Perry C, Chant DC, McLean D, Matigian N, et al. Cell cycle alterations in biopsied olfactory neuroepithelium in schizophrenia and bipolar I disorder using cell culture and gene expression analyses. Schizophr Res. 2006;82:163–173.

28. Benes FM, Matzilevich D, Burke RE, Walsh J. The expression of proapoptosis genes is increased in bipolar disorder, but not in schizophrenia. Mol Psychiatry. 2006;11:241–251.

29. Herberth M, Koethe D, Levin Y, Schwarz E, Krzyszton ND, Schoeffmann S, et al. Peripheral profiling analysis for bipolar disorder reveals markers associated with reduced cell survival. Proteomics. 2011;11:94–105.

30. Kazuno A, Ohtawa K, Otsuki K, Usui M, Sugawara H, Okazaki Y, et al. Proteomic Analysis of Lymphoblastoid Cells Derived from Monozygotic Twins Discordant for Bipolar Disorder: A Preliminary Study. PLoS One. 2013;8.

31. Fries GR, Vasconcelos-Moreno MP, Gubert C, Santos BTMQ do, da Rosa ALST, Eisele B, et al. Early apoptosis in peripheral blood mononuclear cells from patients with bipolar disorder. J Affect Disord. 2014;152–154:474–477.

32. Marianthi L, Olga P, Aristotelis C, Nikolaos V, Fragiskos K. Gene Expression Analysis of Fibroblasts from Patients with Bipolar Disorder. J Neuropsychopharmacol Ment Heal. 2015;1:1–9.

33. Wollenhaupt-Aguiar B, Pfaffenseller B, Chagas V de S, Castro MAA, Passos IC, Kauer-Sant'Anna M, et al. Reduced Neurite Density in Neuronal Cell Cultures Exposed to Serum of Patients with Bipolar Disorder. Int J Neuropsychopharmacol. 2016;19.

34. Li X, Bijur GN, Jope RS. Glycogen synthase kinase-3beta, mood stabilizers, and neuroprotection. Bipolar Disord. 2002;4:137–144.

35. Kim AJ, Shi Y, Austin RC, Werstuck GH. Valproate protects cells from ER stress-induced lipid accumulation and apoptosis by inhibiting glycogen synthase kinase-3. J Cell Sci. 2005;118:89–99.

36. Wilot LC, Bernardi A, Frozza RL, Marques AL, Cimarosti H, Salbego C, et al. Lithium and Valproate Protect Hippocampal Slices Against ATP-induced Cell Death. Neurochem Res. 2007;32:1539–1546.

37. Go HS, Seo JE, Kim KC, Han SM, Kim P, Kang YS, et al. Valproic acid inhibits neural progenitor cell death by activation of NF-κB signaling pathway and up-regulation of Bcl-XL. J Biomed Sci. 2011;18:48.

38. Lowthert L, Leffert J, Lin A, Umlauf S, Maloney K, Muralidharan A, et al. Increased ratio of anti-apoptotic to pro-apoptotic Bcl2 gene-family members in lithium-responders one month after treatment initiation. Biol Mood Anxiety Disord. 2012;2:15.

39. Gawlik-Kotelnicka O, Mielicki W, Rabe-Jabłońska J, Lazarek J, Strzelecki D. Impact of lithium alone or in combination with haloperidol on oxidative stress parameters and cell viability in SH-SY5Y cell culture. Acta Neuropsychiatr. 2016;28:38–44.

40. Del Grosso A, Antonini S, Angella L, Tonazzini I, Signore G, Cecchini M. Lithium improves cell viability in psychosine-treated MO3.13 human oligodendrocyte cell line via autophagy activation. J Neurosci Res. 2016;94:1246–1260.

41. Li Z, Wu F, Zhang X, Chai Y, Chen D, Yang Y, et al. Valproate Attenuates Endoplasmic Reticulum Stress-Induced Apoptosis in SH-

SY5Y Cells via the AKT/GSK3β Signaling Pathway. Int J Mol Sci. 2017;18.

42. Breen MS, White CH, Shekhtman T, Lin K, Looney D, Woelk CH, et al. Lithium-responsive genes and gene networks in bipolar disorder patient-derived lymphoblastoid cell lines. Pharmacogenomics J. 2016;16:446–453.

43. Benes FM, Lim B, Matzilevich D, Walsh JP, Subburaju S, Minns M. Regulation of the GABA cell phenotype in hippocampus of schizophrenics and bipolars. Proc Natl Acad Sci. 2007;104:10164–10169.

44. Benes FM, Lim B, Subburaju S. Site-specific regulation of cell cycle and DNA repair in post-mitotic GABA cells in schizophrenic versus bipolars. Proc Natl Acad Sci U S A. 2009;106:11731–11736.

45. Kim KH, Liu J, Sells Galvin RJ, Dage JL, Egeland JA, Smith RC, et al. Transcriptomic Analysis of Induced Pluripotent Stem Cells Derived from Patients with Bipolar Disorder from an Old Order Amish Pedigree. PLoS One. 2015;10:e0142693.

46. Mao CD, Hoang P, Dicorleto PE. Lithium Inhibits Cell Cycle Progression and Induces Stabilization of p53 in Bovine Aortic Endothelial Cells*. J Biol Chem. 2001;276:26180–26188.

47. Sun A, Shanmugam I, Song J, Terranova PF, Thrasher JB, Li B. Lithium suppresses cell proliferation by interrupting E2F–DNA interaction and subsequently reducing S–phase gene expression in prostate cancer. Prostate. 2007;67:976–988.

48. Seelan RS, Khalyfa A, Lakshmanan J, Casanova MF, Parthasarathy RN. Deciphering the lithium transcriptome: Microarray profiling of lithium-modulated gene expression in human neuronal cells. Neuroscience. 2008;151:1184–1197.

49. Zanni G, Di Martino E, Omelyanenko A, Andäng M, Delle U, Elmroth K, et al. Lithium increases proliferation of hippocampal neural stem/progenitor cells and rescues irradiation-induced cell cycle arrest in vitro. Oncotarget. 2015;6:37083–37097.

50. Rattanawarawipa P, Pavasant P, Osathanon T, Sukarawan W. Effect of lithium chloride on cell proliferation and osteogenic differentiation in stem cells from human exfoliated deciduous teeth. Tissue Cell. 2016;48:425–431.

51. Laeng P, Pitts RL, Lemire AL, Drabik CE, Weiner A, Tang H, et al. The mood stabilizer valproic acid stimulates GABA neurogenesis from rat forebrain stem cells. J Neurochem. 2004;91:238–251.

52. Catalano MG, Fortunati N, Pugliese M, Costantino L, Poli R, Bosco O, et al. Valproic Acid Induces Apoptosis and Cell Cycle Arrest in Poorly Differentiated Thyroid Cancer Cells. J Clin Endocrinol Metab. 2005;90:1383–1389.

53. Li X-N, Shu Q, Su JM-F, Perlaky L, Blaney SM, Lau CC. Valproic acid induces growth arrest, apoptosis, and senescence in medulloblastomas by increasing histone hyperacetylation and regulating expression of p21Cip1, CDK4, and CMYC. Mol Cancer Ther. 2005;4:1912–1922.

54. Wu Y, Guo S-W. Histone deacetylase inhibitors trichostatin A and valproic acid induce cell cycle arrest and p21 expression in immortalized human endometrial stromal cells. Eur J Obstet Gynecol Reprod Biol. 2008;137:198–203.

55. Witt D, Burfeind P, von Hardenberg S, Opitz L, Salinas-Riester G, Bremmer F, et al. Valproic acid inhibits the proliferation of cancer cells by re-expressing cyclin D2. Carcinogenesis. 2013;34:1115–1124.

56. Claudia Morich. The influence of valproic acid and the role of cyclin D2 in prostate cancer. Georg-August University, Göttingen, 2016.

57. Pietruczuk K, Lisowska KA, Grabowski K, Landowski J, Witkowski JM. Proliferation and apoptosis of T lymphocytes in patients with bipolar disorder. Sci Rep. 2018;8:3327.

Supplementary table 2. Clinical and demographic characteristics of LCL study

Variables	Total (N=25)	Responders (N=16)	Non-responders (N=9)	Chi square or z value	p value
Age at assessment	40.2(12.8)	37.6(10.5)	44.8(15.6)	-1.0[a]	0.3
Gender-M: F (M %)	15:10(60%)	8:8(50%)	7:2(77.8%)	1.9[b]	0.16
Duration of illness (months)	239.2(146.9)	214.8(93.3)	282.7(212.3)	-0.19[a]	0.8
Duration of lithium treatment (months)	104.8(83.6)	114.9(91.8)	81.8(60.5)	-0.8[a]	0.4
Age of onset	20.4(6.0)	20.4(7.3)	20.4(3.5)	-0.8[a]	0.3
Number of hospitalizations	3.7(4.8)	3.8(5.8)	3.5(2.2)	-0.9[a]	0.3
Psychotic symptoms[#]	20(83.3%)	13(81.3%)	7(87.5%)	0.15[b]	0.69
Total no. of episodes	8.4(6.2)	7.6(5.0)	10.1(8.4)	-0.7[a]	0.4
No. of manic episodes	6.7(5.4)	5.8(4.3)	8.5(7.5)	-0.7[a]	0.5
No. of depression episodes	1.8(2.5)	1.5(2.2)	2.5(3.2)	-0.7[a]	0.4
No of mixed episodes	0.3(0.6)	0.2(0.5)	0.4(0.7)	-0.5[a]	0.5
Family H/o BD	15(62.5%)	8(50%)	7(87.5%)	3.5[b]	0.06
Family H/o psychosis	3(13%)	1(6.7%)	2(25%)	N/A	N/A
Suicide attempt[#]	2(9.1%)	2(13.3%)	0	N/A	N/A
Onset episode	Mania- 19 (76%)	11 (71.4%)	8 (85.7%)	0.56[b]	0.45
	Depression-6 (24%)	5 (28.6%)	1 (14.3%)	0.56[b]	0.45
ALDA total score	5.6(3.0)	7.5(0.6)	2.2(2.4)	-4.1[a]	0.000*
A score	7.88(3.1)	9.8(0.5)	4.4(2.9)	-4.4[a]	0.000*
B score	2.7(1.2)	2.3(0.7)	3.5(1.4)	-2.1[a]	0.03*

[#] Lifetime History, *p<0.05 (statistically significant); Values are mean (±SD), or n (%). [a] Mann-Whitney U test or [b] Pearson chi square were utilized to calculate p values across the variables between the responders and non-responders.

Supplementary table 3: Rare damaging exome variants identified in family A

Gene	Variant	RS id/Novel	No of affected	Presence in BD1/BD2	Signaling pathway	Cellular role
**DENND5A**	c.A2699G	rs779817963	4	BD1/BD2	ERK pathway [1]	Cell migration [2], proliferation, apoptosis
**KIF7**	c.G2690C	rs749711306	3	BD1	Hedgehog signalling [3,4]	Cell proliferation [5], migration
**SCN3A**	c.G83A	rs775711350	3	BD1/BD2	No pathway reported	Neuronal migration [6], Cell cycle [7]
**PARP14**	c.G3467A	Novel	4	BD1/BD2	JNK2 signalling [8]	Apoptosis [9], glycolysis [10]
**PCCB**	c.C595T	rs371155999	3	BD1/BD2	Proprionate metabolism pathway [11]	Mitochondrial oxidative phosphorylation [12]
**TRMT44**	c.C1405T	rs373816157	3	BD1		
**NRG2**	c.C1477T	rs148371256	3	BD1	MAPK and ERBB pathway [13,14]	Cell migration, proliferation [15,16], oxidative stress [17]
**NIPBL**	c.A4496C	Novel	4	BD1/BD2	Notch pathway [18], Cohesion [19], Wnt and PI3K-AKT pathway [18]	Cell migration, proliferation, apoptosis [20,21]
**SCUBE3**	c.C1996T	Novel	3	BD1/BD2	FGF Pathway [22] and hedgehog signaling [23]	Cell proliferation [24]
**ANLN**	c.C128T	rs575071809	3	BD1/BD2	PI3K pathway [25]	Cell migration, Cell cycle [26,27]

Bibliography:

1. Han C, Alkhater R, Froukh T, Minassian AG, Galati M, Liu RH, et al. Epileptic Encephalopathy Caused by Mutations in the Guanine Nucleotide Exchange Factor DENND5A. Am J Hum Genet. 2016;99:1359–1367.

2. Li Y, Xu J, Xiong H, Ma Z, Wang Z, Kipreos ET, et al. Cancer driver candidate genes AVL9, DENND5A and NUPL1 contribute to MDCK cystogenesis. Oncoscience. 2014;1:854–865.

3. He M, Subramanian R, Bangs F, Omelchenko T, Liem Jr KF, Kapoor TM, et al. The kinesin-4 protein Kif7 regulates mammalian Hedgehog signalling by organizing the cilium tip compartment. Nat Cell Biol. 2014;16:663–672.

4. Dafinger C, Liebau MC, Elsayed SM, Hellenbroich Y, Boltshauser E, Korenke GC, et al. Mutations in KIF7 link Joubert syndrome with Sonic Hedgehog signaling and microtubule dynamics. J Clin Invest. 2011;121:2662–2667.

5. Ho J, Du Y, Wong OG-W, Siu MKY, Chan KKL, Cheung ANY. Downregulation of the Gli Transcription Factors Regulator Kif7 Facilitates Cell Survival and Migration of Choriocarcinoma Cells. PLoS One. 2014;9:e108248.

6. Brackenbury WJ, Djamgoz MBA, Isom LL. An Emerging Role for Voltage-Gated Na+ Channels in Cellular Migration: Regulation of Central Nervous System Development and Potentiation of Invasive Cancers. Neurosci. 2008;14:571–583.

7. Besson P, Driffort V, Bon É, Gradek F, Chevalier S, Roger S. How do voltage-gated sodium channels enhance migration and invasiveness in cancer cells? Biochim Biophys Acta - Biomembr. 2015;1848:2493–2501.

8. Iansante V, Choy PM, Fung SW, Liu Y, Chai J-G, Dyson J, et al. PARP14 promotes the Warburg effect in hepatocellular carcinoma by inhibiting JNK1-dependent PKM2 phosphorylation and activation. Nat Commun. 2015;6:7882.

9. Barbarulo A, Iansante V, Chaidos A, Naresh K, Rahemtulla A, Franzoso G, et al. Poly(ADP-ribose) polymerase family member 14 (PARP14) is a novel effector of the JNK2-dependent pro-survival signal in multiple myeloma. Oncogene. 2013;32:4231–4242.

10. Cho SH, Ahn AK, Bhargava P, Lee C-H, Eischen CM, McGuinness O, et al. Glycolytic rate and lymphomagenesis depend on PARP14, an ADP ribosyltransferase of the B aggressive lymphoma (BAL) family. Proc Natl Acad Sci U S A. 2011;108:15972–15977.

11. Porntaveetus T, Srichomthong C, Suphapeetiporn K, Shotelersuk V. A novel PCCB mutation in a Thai patient with propionic acidemia identified by exome sequencing. Hum Genome Var. 2015;2:15033.

12. Chapman KA, Ostrovsky J, Rao M, Dingley SD, Polyak E, Yudkoff M, et al. Propionyl-CoA carboxylase pcca-1 and pccb-1

gene deletions in Caenorhabditis elegans globally impair mitochondrial energy metabolism. J Inherit Metab Dis. 2018;41:157–168.

13. Benzel I, Bansal A, Browning BL, Galwey NW, Maycox PR, McGinnis R, et al. Interactions among genes in the ErbB-Neuregulin signalling network are associated with increased susceptibility to schizophrenia. Behav Brain Funct. 2007;3:31.

14. Falls DL. Neuregulins: functions, forms, and signaling strategies. Exp Cell Res. 2003;284:14–30.

15. Ghashghaei HT, Weber J, Pevny L, Schmid R, Schwab MH, Lloyd KCK, et al. The role of neuregulin-ErbB4 interactions on the proliferation and organization of cells in the subventricular zone. Proc Natl Acad Sci U S A. 2006;103:1930–1935.

16. Wu J, Li M, Zhang Y. Long noncoding RNA *HOXA-AS2* regulates the expression of *SCN3A* by sponging *miR-106a* in breast cancer. J Cell Biochem. 2019;120:14465–14475.

17. Vyas VK, Berkey CD, Miyao T, Carlson M. Repressors Nrg1 and Nrg2 regulate a set of stress-responsive genes in Saccharomyces cerevisiae. Eukaryot Cell. 2005;4:1882–1891.

18. Pistocchi A, Fazio G, Cereda A, Ferrari L, Bettini LR, Messina G, et al. Cornelia de Lange Syndrome: NIPBL haploinsufficiency downregulates canonical Wnt pathway in zebrafish embryos and patients fibroblasts. Cell Death Dis. 2013;4:e866–e866.

19. Liu J, Zhang Z, Bando M, Itoh T, Deardorff MA, Clark D, et al. Transcriptional Dysregulation in NIPBL and Cohesin Mutant Human Cells. PLoS Biol. 2009;7:e1000119.

20. Xu W, Ying Y, Shan L, Feng J, Zhang S, Gao Y, et al. Enhanced expression of cohesin loading factor NIPBL confers poor prognosis and chemotherapy resistance in non-small cell lung cancer. J Transl Med. 2015;13:153.

21. Yuen KC, Xu B, Krantz ID, Gerton JL. NIPBL Controls RNA Biogenesis to Prevent Activation of the Stress Kinase PKR. Cell Rep. 2016;14:93–102.

22. Tu C-F, Tsao K-C, Lee S-J, Yang R-B. SCUBE3 (Signal Peptide-CUB-EGF Domain-containing Protein 3) Modulates Fibroblast Growth Factor Signaling during Fast Muscle Development. J Biol Chem. 2014;289:18928–18942.

23. Xavier GM, Panousopoulos L, Cobourne MT. Scube3 Is Expressed in Multiple Tissues during Development but Is Dispensable for Embryonic Survival in the Mouse. PLoS One. 2013;8:e55274.

24. Liang W, Yang C, Peng J, Qian Y, Wang Z. The Expression of HSPD1, SCUBE3, CXCL14 and Its Relations with the Prognosis in Osteosarcoma. Cell Biochem Biophys. 2015;73:763–768.

25. Zeng S, Yu X, Ma C, Song R, Zhang Z, Zi X, et al. Transcriptome sequencing identifies ANLN as a promising prognostic biomarker in bladder urothelial carcinoma. Sci Rep. 2017;7:3151.

26. Magnusson K, Gremel G, Rydén L, Pontén V, Uhlén M, Dimberg A, et al. ANLN is a prognostic biomarker independent of Ki-67 and essential for cell cycle progression in primary breast cancer. BMC Cancer. 2016;16:904.

27. Zhou W, Wang Z, Shen N, Pi W, Jiang W, Huang J, et al. Knockdown of ANLN by lentivirus inhibits cell growth and migration in human breast cancer. Mol Cell Biochem. 2015;398:11–19.

Supplementary table 4: Studies of *BCL2. GSK3B and NR1D1* genes in bipolar disorder

Author and Year	Type of study	Sample details	Objective of study	Significant results
BCL2				
H.-W. Kim, Rapoport, & Rao, 2010 [1]	Case- control study (protein/ mRNA level)	10BD &10 control frontal cortex from PM brains	To investigate levels of BCL2 protein & mRNA in BD compared to HC	Decreased BCL2 protein and mRNA levels in frontal cortex from BD brain tissues compared to controls.<br>BAX/BCL2 ratio was increased in BD brain tissues.
Moutsatsou et al., 2014 [2]	Case- control study (mRNA level & apoptotic activity)	Lymphocyte from 35 BD and 10 HC	To investigate the level of *BAX/BCL2* mRNA ratio level, caspae3 activity and cytochrome C release in BD and controls.	Higher *BAX/BCL2* mRNA levels in BD patients in manic and depressed state compared to HCs.<br>Cytochrome c release, caspase-3 activity was increased in manic and depressed BD patients compared to HC, indicating higher apoptotic activity in BD.
W. T. Chen, Huang, & Tsai, 2015 [3]	Case- control study (protein level)	20 BD patients in manic phase and 40 HC from Taiwan	To examine the serum BCL2 levels in BD and HCs	Serum BCL2 levels higher in manic state of BD patients than HC, though statistically not significant.
Uemura et al., 2011 [4]	Functional study and case- control association study	LCLs from 245 patients (150 BD-I, 65 BD-II &30 MDD) and 70 HC subjects	To study the role of *BCL2* rs956572 SNP on basal intra cellular calcium, mRNA and protein levels in BD and control subjects. .	1) No significant association of the *BCL2* SNP with any of the disorders.<br>2) Basal calcium levels of LCLs: Significantly higher in BD compared to controls, subjects with GG genotype being the highest compared to other genotype (AA<AG<GG). BD with GG reported to have higher levels compared to other groups carrying same GG.<br>3) *BCL2* mRNA and protein levels were lower in BD than HC. GG genotype subjects lower levels compared to AA or AG, effect was more prominent in BD.

Soeiro-de-Souza et al., 2013 [5]	Case-control association study	40 BD euthymic patients and 40 HC from Brazil. MRS is done to obtain glutamate levels of anterior cingulate cortex (ACC)	Tested the association of *BCL2* (rs956572) SNP with ACC glutamate levels	AA genotype at the *BCL2* SNP was associated with elevated ACC glutamate metabolites in the BD patients, not in controls.
Uemura, Green, & Warsh, 2015 [6]	Functional study and case- control association study	LCLs derived from 215 BD cases, including 150 BD-I and 65 BD-II; and 70 healthy controls	To investigate whether the *BCL2* rs956572 variant associates with intracellular calcium dyshomeostasis in BD and HC	1) Lower $Ca2^{+}$ in subjects with BCL2 rs956572 AA variant compared to AG or GG as a whole.<br>2) BD patients carrying GG genotype- highest $Ca2^{+}$ compared to AA or AG.<br>3) BD patients carrying GG genotype- higher Ca^{2+} compared to HC with GG genotype.
Corson, Woo, Li, & Warsh, 2004 [7]	Pharmacological study	Human hNT neurons from NT2 teratoma cells and SVG p12 SV40 glia cells (treated with lithium or valproate for 7days)	To test the effect of lithium and valproate on *BCL2* mRNA levels in hNT neurons	Treatment of hNT cells with valproate (0.35, 0.75, 1 mM) for 7 days upregulated BCL2 mRNA (max increase with 0.75mM), whereas lithium (0.75-2mM) could not alter. The SVG glia cells *BCL2* mRNA was not changed by both the treatments.
Lai et al., 2006 [8]	Pharmacological study	Human SH-SY5Y neuroblastoma, SVGp12 glial cells and U87 glioma cells (treated with lithium [1mM] or valproate [0.6mM] for 1 or 7 days)	Looked into the effect of lithium or valproate in stress induced human cells	Pretreatment of SH-SY5Y cells for 7 days with lithium or valproate significantly reduced rotenone or H_2O_2 induced cytochrome C release, caspase activity & cytotoxicity and upregulated BCL2 protein level. No effect was reported on 1 day treatment with the lithium or valproate. Other cell types were not affected by any such treatments.
Creson, Yuan, Manji, & Chen, 2009 [9]	Pharmacological study	Human SH-SY5Y neuroblastoma cells	Looked in the effect of valproate on BCL2 protein and mRNA levels in human neuroblastoma cells.	Increased BCL2 protein in concentration (0.125-2mM) & time (1-3 days on 0.8mM) dependent manner. 0.5-2mM for concentration and 2 or 3 days treatment induced the increase in BCL2 level whereas 1 day did not. Similarly, valproate increased the BCL2 mRNA in time dependent manner, 3rd day being the highest increase. The enhancement of BCL2 levels was selective as the HK gene GAPDH was not altered.

Odeya D, Galila A, & Lilah T, 2018 [10]	Pharmacological study	10-12 weeks old wild type mice from *IMPA1* colony	Effect of lithium on *BCL2* gene expression in hippocampi of mice	On treatment with lithium *BCL2* gene expression levels were increase when normalized to *ACTB*, whereas decreased on normalizing with *MAPK6* or *ANKRD11*.
Machado-Vieira et al., 2011 [11]	Functional study- 1) SNP- with intracellular calcium, mRNA & protein, 2)SNP-lithium effect	LCLs from 18 BD subjects with equal numbers of individuals carrying the rs956572 variants for AA, AG & GG	To study the role of *BCL2* rs956572 SNP on basal intra cellular calcium and after 1mmol/L lithium treatment in BD LCLs for 7 days.	1) Basal and stimulated intracellular $Ca2^{+}$ were higher in BD LCLs with AA variant compared to GG variant though, BCL2 mRNA and protein level was least in AA variant of the *BCL2* polymorphism.<br>2) Li treatment increased the *BCL2* expression in the AA variants of BD LCLs.
Lowthert et al., 2012 [12]	Pharmacogenetic study	8 weeks of open label lithium study in 20 BD patients	To assess the change in gene expression in peripheral blood of lithium responders and non-responders	127 genes differentially expressed between responders and non-responders.<br>Pathway analysis showed regulation of apoptosis was significantly affected.<br>Upregulation of anti-apoptotic gene *BCL2* & downregulation of pro-apoptotic genes (*BAD*, *BAK1*) in responders and inverse relation in NR after 4 weeks.
GSK3B				
Xiaohong Li, Liu, Cai, Wang, & Li, 2010 [13]	Case- control study and anti-manic treatment response (protein level)	1) 30 medication free BD manic subjects were compared with 30 healthy controls from Beijing Anding Hospital, China.<br>2) 47 BD (4 weeks) and 28 BD (8 weeks) subjects were analyzed for pre and post treatment with lithium, valproate and atypical anti-psychotics	To test the regulation of GSK3B in BD patients with manic episode and in response to treatment - examined the protein level and the inhibitory serine phosphorylation of GSK3B in PBMCs of patients compared with healthy controls	1) The total protein levels of GSK3B was significantly higher in BD manic subjects than in healthy controls.<br>2) Phospho-Ser9-GSK3B was reported to be trend toward lower in bipolar manic subjects than in healthy controls.<br>3) Significant increase in phospho-Ser9-GSK3B was reported in 28 BD subjects post 4 weeks and 8 weeks treatment.<br>4) Total GSK3B among the 47 subjects was not significantly different at treatment.

Pandey, Ren, Rizavi, & Dwivedi, 2010 [14]	Case- control study (protein level)	1) 21 BD patients and 21 HC from Chicago were investigated for GSK3B level in platelet,<br>2) The patients compared for GSK3B level before and after treatment (lithium or valproate or antipsychotics) for 8 weeks.	To explore the role of GSK3B in BD and in response to treatment	1) GSK3B protein level in cytosol and membrane fraction of platelets from BD were decreased in comparison to controls.<br>2) The protein level after 8 weeks of treatment was increased.
Lesort, Greendorfer, Johnson, 1999 [15]	Case- control study in PM brain tissues	DLPFC of 5 BD and 5 controls from Ohio.	To compare the levels of GSK3B protein in brain tissues of BD and HCs	No significant difference was reported.
Munkholm, Peijs, Vinberg, & Kessing, 2015 [16]	Case-control candidate gene expression study	37 rapid cycling BD patients and 40 HC of Danish population	Tested *GSK3B* gene expression in BD & HC	*GSK3B* mRNA level was significantly downregulated in BD, however after Bonferroni correction the result was not significant.
Benedetti, Bernasconi, et al., 2004 [17]	Candidate gene association study	185 Italian BD patients	To test the effect of the *GSK3B* rs334558 SNP on age at onset of BD	Homozygote TT was reported to be associated with earlier age at onset of BD.
Benedetti, Serretti, et al., 2004 [18]	Candidate gene association study	60 depressed BD patients	To test the effect of *GSK3B* -50T/C polymorphism on age at onset of BD and acute response to total sleep deprivation	Homozygotes for the mutant allele was associated with later age at onset of BD, less severe symptomatology when depressed (HDRS score), & better acute effects of total sleep deprivation treatment on perceived mood (VAS score).
Nishiguchi, Breen, Russ, St Clair, & Collier, 2006 [19]	Case-control candidate gene association study	280 Caucasian BD patients and 407 HC.	To test the association of the *GSK3B* -50T/C SNP and BD.	No significant association

Szczepankiewicz, Skibinska, et al., 2006 [20]	Case-control candidate gene association study	416 Polish patients and 408 HC	To test the association of the *GSK3B* -50T/C SNP and BD.	1) Trend association of heterozygous T/C genotype with BD was reported.<br>2) Significant association of SNP with the female BDII patients (n=57).<br>3) No significant association with AAO of BD
Serretti et al., 2008 [21]	Candidate gene association study	365 Italian mood disorder patients, included 122 MDD and 243 BD patients.	To evaluate the association of the polymorphism with symptomatic and personality feature in mood disorder	The GSK3B polymorphism was found associated with delusional symptomatology and with the personality features linked to self-transcendence.
Subhashree et al., 2009 [22]	Case-control association study (NIMHANS)	186 subjects with BD and 186 healthy controls from NIMHANS, Bangalore, INDIA	To investigate the association of -50T/C SNP in *GSK3B* gene with BD.	No significant association
E. Jiménez et al., 2013 [23]	Candidate gene association study	192 Caucasian BD subjects included 66 suicide attempters and 126 suicide non attempters	To investigate association between the *GSK3B* -50T/C SNP & suicide behavior in BD	C allele of the *GSK3B* SNP showed trend association (p=0.052) with suicide attempters.
Esther Jiménez et al., 2014 [24]	Candidate gene association study	199 Caucasian BD subjects	To evaluate the effect of the SNP on impulsivity in BD	C allele carrier was associated with higher level of impulsivity in BD
Tang et al., 2013 [25]	Meta-analysis study	48 relevant studies screened and 5 BD studies (3 Asian and 2 Caucasian) finally included for analysis. Total 971 cases and 1397 controls	To test the association of the *GSK3B* -50T/C SNP and BD.	No significant association of the *GSK3B* -50T/C SNP and BD.
G. Chen et al., 2014 [26]	Meta-analysis study	95 relevant studies screened and 10 BD studies included<br>1) For association with BD	To investigate the association between the *GSK3B* -50T/C SNP and the susceptibility or age at	No significant association of the GSK3B -50T/C SNP with risk of BD or age at onset of BD was reported in any of the genetic model that was analyzed.

		risk, 6 (3 Asian and 3 Caucasian) publications. Total of 1251 cases & 1804 controls.<br>2) For association with age at onset, 6 (2 Asian and 4 Caucasian) publications were analyzed. Total of 659 cases.	onset of BD.	
De Sarno, Li, & Jope, 2002 [27]	Pharmacological study	1) *In vitro*: Human neuroblastoma SH-SY5Y cells- with valproate or lithium for 24 hours at varying concentration<br>2) *In vivo*: Adult male C57BL/6 mice treated with 0.4% lithium (0.7mM at serum)	Evaluated the effect of lithium or valproate on phosphorylation of GSK3B.	1) Sodium valproate treatment effected gradual increase in the inhibition-associated phospho-Ser9-GSK3B.<br>2) Lithium treatment increased the phospho-Ser9-GSK3B both in cells and in mouse brain after chronic administration.
Zhang, Phiel, Spece, Gurvich, & Klein, 2003 [28]	Pharmacological study	293T cells, Neuro2A, and NIH3T3 cells from American Type Culture Collection- treated with lithium or VPA (varying concentration and duration)	Evaluated the effect of lithium or valproate on GSK3B.	1) Lithium treatment inhibited GSK3B activity which was mediated through increased phospho-Ser9-GSK3Bin cells.<br>2) VPA was not reported to inhibit GSK3B & did not induce increase in phosphorylation of GSK3B.
Jonathan Ryves, Dalton, Harwood, & Williams, 2005 [29]	Pharmacological study- using rat primary cells	Rat neocortical neurons – treated with 3mM lithium or 1.8mM valproic acid (VPA).	To examine the effect of lithium and VPA on GSK3B protein levels.	1) No effect of lithium and VPA on GSK3B protein level.
Abdul A, De Silva B, & Gary R, 2018 [30]	Pharmacological study	NIH-3T3 mouse embryo fibroblast, A172 human glioblastoma	Evaluated the effect of lithium or beryllium on phosphorylation of GSK3B and its substrate.	Lithium (20mM) and beryllium (30 & 100 uM) decreases phosphorylation of glycogen synthase (GS) and increased phosphorylation of Ser9-GSK3B in NIH3T3. No change in levels of total GS or GSK3B.

				In A172 cells, lithium increased phosphor-Ser33/Ser37-B catenin.
Xiaohua Li et al., 2007 [31]	Case-control study (protein level) using PBMCs of subjects and *in vitro* study by lithium treatment of PBMCs	23 HC, 9 lithium treated BD and 13 lithium free BD subjects of Caucasian population.	Evaluated change in serine phosphorylation of GSK3B in PBMCs of BD and HC subjects at baseline and *in vitro* treatment with lithium (20mMol/L) for 1 hour	1) Basal level of phospho-Ser9-GSK3B was reported to be lowest in HC followed by 3-fold increase in lithium free BD and highest in lithium treated BD subjects.<br>2) *In vitro* lithium treatment was also associated with elevation of phospho-Ser9-GSK3B level.<br>3) No change in total GSK3B protein level.
Mendes et al., 2009 [32]	Animal model study (gene expression)– lithium treatment using Wistar rat	1) *In vitro*: cortical and hippocampal neurons- 5days LiCl treatment (0.02 to 2mM)<br>2) *In vivo*: 12 rats- (0.12mmol lithium; 0.24mmol lithium)	To test the role of role of *GSK3B* in response to lithium treatment.	1) *In vitro*: *GSK3B* mRNA level was reduced in hippocampal neurons on treatment but no changes in cortical neurons.<br>2) *In vivo*: *GSK3B* mRNA reduced in hippocampus but not in cortex or in leukocyte of treated rats.
McCarthy et al., 2011 [33]	Pharmacogenetic study- gene expression in LCLs	LCLs from BD lithium responders (N=13) and lithium non-responders (N=18)	To test the effect of lithium 1mM for 72 hours on *GSK3B* expression in LCLS from BD lithium response patients	No effect on *GSK3B* gene expression in both the BD lithium response groups
Geoffroy et al., 2017 [34]	Pharmacogenetic study- gene expression in LCLs	38 French Caucasian BD patients which included 16 ER and 20 NR of lithium.	To test the effect of lithium 1mM (2 -8 days) on *GSK3B* expression in LCLS from BD lithium response patients	Only on day 8 lithium significantly increased *GSK3B* gene expression in BD lithium non-responders
Benedetti et al., 2005 [35]	Pharmacogenetic association study	88 BD patients- 2 years on lithium.	To test the association of the *GSK3B* -50T/C SNP with therapeutic response to lithium.	Mutant allele C carriers improved recurrent rate of mood episode after 2 years on lithium treatment.
Szczepankiewicz,	Pharmacogenetic association study	89 polish BD patients-5 years on lithium	To test the association of G*SK3B* -50T/C SNP with	No significant differences in genotypic and allelic frequencies between the SNP

Rybakowski, et al., 2006 [36]			therapeutic response to lithium.	and the degree of lithium response .
Numajiri et al., 2012 [37]	Pharmacogenetic association study	29 Japanese BD patients	To test the association of the SNP with therapeutic response to lithium.	T allele significantly associated with lithium responders.
Y. F. Lin, Huang, & Liu, 2013 [38]	Case-control and Pharmacogenetic association study	138 Taiwanese BD patients and 131 controls. 83 patients out of 138 cases were evaluated for lithium treatment (24 months) efficacy.	To test the association of the SNP with BD risk and therapeutic response to lithium treatment.	1) No significant association of the *GSK3B* -50T/C SNP and BD.<br>2) TT genotype was associated with poor lithium treatment response.
Iwahashi et al., 2014 [39]	Pharmacogenetic association study	42 Japanese patients: 27 were lithium responders and 15 were non-responders.	To test the association of SNP with lithium treatment response.	No significant difference was reported in genotype and allele frequency of the SNP between lithium responders and non-responders.<br>However, haplotype blocks T-A and C-A (with another SNP [-1727A/T]) was reported to be associated with higher lithium response and lower lithium response respectively.
Mitjans et al., 2015 [40]	Pharmacogenetic association study	Total 131 BD patients from Barcelona which included 26 excellent responders (ER); 62 partial responders (PR) and 43 non-responders (NR) based on lithium response.	To test the association of SNP with lithium treatment response.	No significant difference was reported in genotype and allele distribution between the lithium response groups.<br>However haplotype rs1732170-rs11921360-rs34558 was associated with lithium response. The C-C-A haploblock was significantly less frequent in group of lithium partial and non-responders than excellent responders.
NR1D1				
Yang, Van Dongen, Wang,	Case-control association study	2 set of fibroblast samples from Corriel Cell	To study the expression of core clock genes in BD and	Set-I: No difference in circadian period.<br>Amplitude of rhythmic expression of *NR1D1*

Berrettini, & Bućan, 2009 [41]		Repositories. Set-I: 12BD &12 HC; Set-II: 18BD & 35HC.	HC.	reduced in BD. Overall expression of *NR1D1* reduced in BD though statistically not significant.<br>Set-II: *GSK3B* mRNA & protein level no significant difference between BD and HC, whereas serine-9-phospho GSK3B was significantly reduced in BD.
Nováková, Praško, Látalová, Sládek, & Sumová, 2015 [42]	Case-control association study	Buccal cells from 19 HC, 22 BD depressive & 19 BD in manic subjects from Czech Republic.	To investigate the *NR1D1* expression profiling for 24 hours in buccal cells of BD and HC	*NR1D1* expression profiling for 24 hours in buccal cells. *NR1D1* expression profiles of BD in mania was advanced compared to depression and trend advanced compared to control. Amplitude *NR1D1* expression higher in mania.
Warburton et al., 2015 [43]	Pharmacological study	Human SH-SY5Y neuroblastoma cells	To examine the expression profiling in neuroblastoma cells on treatment with lithium (1mM) or valproate (5mM) for 1 hr.	Lithium treatment of neuroblastoma cells showed trend change in *NR1D1* expression, whereas valproate did not alter the expression.
McCarthy et al., 2011 [33]	Pharmacogenetic study	Genetic association: 282 BD Caucasian origin (148 lithium responders and 134 non-responders.<br>LCL experiment: 13 responders and 18 non-responders (1mM lithium for 72hours treatment)	To test the role of NR1D1 lithium treatment response.	1) Allele A at rs2071427 SNP associated with lithium good response.<br>2) Homozygous allele A at the SNP decreased *NR1D1* mRNA (full length transcript) after lithium treatment compared to homozygous G allele.<br>3) AA genotype was associated with trend increase of *NR1D1* (both full and truncated transcript) mRNA compared to GG after treatment.
Geoffroy et al., 2017 [34]	Pharmacogenetic study	LCLs from 36 BD subjects (20 lithium responders and 16 non-responders of Caucasian origin	To analyse the gene expression of BD LCLs at day2,4,8 on 1mM lithium treatment	*NR1D1* gene expression was downregulated at day 2 in responders. *NR1D1* was upregulated at day 4 for both responders and non-responders. No significant changes at day 8.

Campos-de-Sousa et al., 2010 [44]; Kishi et al., 2008 [45]; Kripke, Nievergelt, Joo, Shekhtman, & Kelsoe, 2009 [46]; Severino et al., 2009 [47].	Case-control candidate gene association studies	BD and HC subjects in various studies	Tested the association of different *NR1D1* polymorphisms with risk of BD	The studies have reported positive association of the *NR1D1* polymorphisms with risk for BD.

Bibliography:

1. Kim H-W, Rapoport SI, Rao JS. Altered expression of apoptotic factors and synaptic markers in postmortem brain from bipolar disorder patients. Neurobiol Dis. 2010;37:596–603.

2. Moutsatsou P, Tsoporis JN, Salpeas V, Bei E, Alevizos B, Anagnostara C, et al. Peripheral blood lymphocytes from patients with bipolar disorder demonstrate apoptosis and differential regulation of advanced glycation end products and S100B. Clin Chem Lab Med. 2014;52:999–1007.

3. Chen W-T, Huang T-L, Tsai M-C. Bcl-2 associated with severity of manic symptoms in bipolar patients in a manic phase. Psychiatry Res. 2015;225:305–308.

4. Uemura T, Green M, Corson TW, Perova T, Li PP, Warsh JJ. Bcl-2 SNP rs956572 associates with disrupted intracellular calcium homeostasis in bipolar I disorder. Bipolar Disord. 2011;13:41–51.

5. Soeiro-de-Souza MG, Salvadore G, Moreno RA, Otaduy MCG, Chaim KT, Gattaz WF, et al. Bcl-2 rs956572 polymorphism is associated with increased anterior cingulate cortical glutamate in euthymic bipolar I disorder. Neuropsychopharmacology. 2013;38:468–475.

6. Uemura T, Green M, Warsh JJ. CACNA1C SNP rs1006737 associates with bipolar I disorder independent of the Bcl-2 SNP rs956572 variant and its associated effect on intracellular calcium homeostasis. World J Biol Psychiatry. 2015;17:1–10.

7. Corson TW, Woo KK, Li PP, Warsh JJ. Cell-type specific regulation of calreticulin and Bcl-2 expression by mood stabilizer drugs. Eur Neuropsychopharmacol. 2004;14:143–150.

8. Lai JS, Zhao C, Warsh JJ, Li PP. Cytoprotection by lithium and valproate varies between cell types and cellular stresses. Eur J Pharmacol. 2006;539:18–26.

9. Creson TK, Yuan P, Manji HK, Chen G. Evidence for involvement of ERK, PI3K, and RSK in induction of Bcl-2 by valproate. J Mol Neurosci. 2009;37:123–134.

10. Odeya D, Galila A, Lilah T. The observed alteration in BCL2 expression following lithium treatment is influenced by the choice of normalization method. Sci Rep. 2018;8:6399.

11. Machado-Vieira R, Pivovarova NB, Stanika RI, Yuan P, Wang Y, Zhou R, et al. The Bcl-2 gene polymorphism rs956572AA increases inositol 1,4,5-trisphosphate receptor-mediated endoplasmic reticulum calcium release in subjects with bipolar disorder. Biol Psychiatry. 2011;69:344–352.

12. Lowthert L, Leffert J, Lin A, Umlauf S, Maloney K, Muralidharan A, et al. Increased ratio of anti-apoptotic to pro-apoptotic Bcl2 gene-family members in lithium-responders one month after treatment initiation. Biol Mood Anxiety Disord. 2012;2:15.

13. Li X, Liu M, Cai Z, Wang G, Li X. Regulation of glycogen synthase kinase-3 during bipolar mania treatment. Bipolar Disord. 2010;12:741–752.

14. Pandey GN, Ren X, Rizavi HS, Dwivedi Y. Glycogen synthase kinase-3beta in the platelets of patients with mood disorders: effect of treatment. J Psychiatr Res. 2010;44:143–148.

15. Lesort M, Greendorfer A, Stockmeier C, Johnson GVW, Jope RS. Glycogen synthase kinase-3β, β-catenin, and tau in postmortem bipolar brain. J Neural Transm. 1999;106:1217–1222.

16. Munkholm K, Peijs L, Vinberg M, Kessing L. A composite peripheral blood gene expression measure as a potential diagnostic biomarker in bipolar disorder. Transl Psychiatry. 2015;5.

17. Benedetti F, Bernasconi A, Lorenzi C, Pontiggia A, Serretti A, Colombo C, et al. A single nucleotide polymorphism in glycogen synthase kinase 3B promoter gene influences onset of illness in patients affected by bipolar disorder. Neurosci Lett. 2004;355:37–40.

18. Benedetti F, Serretti A, Colombo C, Lorenzi C, Tubazio V, Smeraldi E. A glycogen synthase kinase 3-β promoter gene single nucleotide polymorphism is associated with age at onset and response to total sleep deprivation in bipolar depression. Neurosci Lett. 2004;368:123–126.

19. Nishiguchi N, Breen G, Russ C, St Clair D, Collier D. Association analysis of the glycogen synthase kinase-3β gene in bipolar disorder. Neurosci Lett. 2006;394:243–245.

20. Szczepankiewicz A, Skibinska M, Hauser J, Slopien A, Leszczynska-Rodziewicz A, Kapelski P, et al. Association analysis of the GSK-3beta T-50C gene polymorphism with schizophrenia and bipolar disorder. Neuropsychobiology. 2006;53:51–56.

21. Serretti A, Benedetti F, Mandelli L, Calati R, Caneva B, Lorenzi C, et al. Association between GSK-3β -50T/C polymorphism and personality

and psychotic symptoms in mood disorders. Psychiatry Res. 2008;158:132–140.

22. Subhashree D, Kiran Kumar H, Purushottam M, Shubha G, Vallikiran M, Krishna N, et al. Identification of interaction between serotonin transporter and glycogen synthase kinase-3β gene polymorphisms: role in susceptibility to bipolar disorder. Future Neurol. 2009;4:363–370.

23. Jiménez E, Arias B, Mitjans M, Goikolea JM, Roda E, Sáiz PA, et al. Genetic variability at IMPA2, INPP1 and GSK3β increases the risk of suicidal behavior in bipolar patients. Eur Neuropsychopharmacol. 2013;23:1452–1462.

24. Jiménez E, Arias B, Mitjans M, Goikolea JM, Roda E, Ruíz V, et al. Association between GSK3β gene and increased impulsivity in bipolar disorder. Eur Neuropsychopharmacol. 2014;24:510–518.

25. Tang H, Shen N, Jin H, Liu D, Miao X, Zhu L-Q. GSK-3β Polymorphism Discriminates Bipolar Disorder and Schizophrenia: A Systematic Meta-Analysis. Mol Neurobiol. 2013;48:404–411.

26. Chen G, Tang J, Yu G, Chen Y, Wang L, Zhang Y. Meta-analysis demonstrates lack of association of the GSK3B −50C/T polymorphism with risk of bipolar disorder. Mol Biol Rep. 2014;41:5711–5718.

27. De Sarno P, Li X, Jope RS. Regulation of Akt and glycogen synthase kinase-3 beta phosphorylation by sodium valproate and lithium. Neuropharmacology. 2002;43:1158–1164.

28. Zhang F, Phiel CJ, Spece L, Gurvich N, Klein PS. Inhibitory phosphorylation of glycogen synthase kinase-3 (GSK-3) in response to lithium. Evidence for autoregulation of GSK-3. J Biol Chem. 2003;278:33067–33077.

29. Jonathan Ryves W, Dalton EC, Harwood AJ, Williams RS. GSK-3 activity in neocortical cells is inhibited by lithium but not carbamazepine or valproic acid. Bipolar Disord. 2005;7:260–265.

30. Abdul AURM, De Silva B, Gary RK. The GSK3 kinase inhibitor lithium produces unexpected hyperphosphorylation of β-catenin, a GSK3 substrate, in human glioblastoma cells. Biol Open. 2018;7.

31. Li X, Friedman AB, Zhu W, Wang L, Boswell S, May RS, et al. Lithium Regulates Glycogen Synthase Kinase-3β in Human Peripheral Blood Mononuclear Cells: Implication in the Treatment of Bipolar Disorder. Biol Psychiatry. 2007;61:216–222.

32. Mendes CT, Mury FB, de Sá Moreira E, Alberto FL, Forlenza OV, Dias-Neto E, et al. Lithium reduces Gsk3b mRNA levels: implications for Alzheimer Disease. Eur Arch Psychiatry Clin Neurosci. 2009;259:16–22.

33. McCarthy MJ, Nievergelt CM, Shekhtman T, Kripke DF, Welsh DK, Kelsoe JR. Functional genetic variation in the Rev-Erbα pathway and lithium response in the treatment of bipolar disorder. Genes Brain Behav. 2011;10:852–861.

34. Geoffroy PA, Curis E, Courtin C, Moreira J, Morvillers T, Etain B, et al. Lithium response in bipolar disorders and core clock genes

expression. World J Biol Psychiatry. 2017:1–14.

35. Benedetti F, Serretti A, Pontiggia A, Bernasconi A, Lorenzi C, Colombo C, et al. Long-term response to lithium salts in bipolar illness is influenced by the glycogen synthase kinase 3-β −50 T/C SNP. Neurosci Lett. 2005;376:51–55.

36. Szczepankiewicz A, Rybakowski JK, Suwalska A, Skibinska M, Leszczynska-Rodziewicz A, Dmitrzak-Weglarz M, et al. Association study of the glycogen synthase kinase-3β gene polymorphism with prophylactic lithium response in bipolar patients. World J Biol Psychiatry. 2006;7:158–161.

37. Numajiri M, Aoki J, Iwahashi K, Fukamauchi F, Enomoto M, Yoshihara E, et al. [Association between lithium sensitivity and GSK3beta gene polymorphisms in bipolar disorder]. Nihon Shinkei Seishin Yakurigaku Zasshi. 2012;32:161–163.

38. Lin Y-F, Huang M-C, Liu H-C. Glycogen synthase kinase 3β gene polymorphisms may be associated with bipolar I disorder and the therapeutic response to lithium. J Affect Disord. 2013;147:401–406.

39. Iwahashi K, Nishizawa D, Narita S, Numajiri M, Murayama O, Yoshihara E, et al. Haplotype analysis of GSK-3β gene polymorphisms in bipolar disorder lithium responders and nonresponders. Clin Neuropharmacol. 2014;37:108–110.

40. Mitjans M, Arias B, Jiménez E, Goikolea JM, Sáiz PA, García-Portilla MP, et al. Exploring Genetic Variability at PI, GSK3, HPA, and Glutamatergic Pathways in Lithium Response. J Clin Psychopharmacol. 2015;35:600–604.

41. Yang S, Van Dongen HPA, Wang K, Berrettini W, Bućan M. Assessment of circadian function in fibroblasts of patients with bipolar disorder. Mol Psychiatry. 2009;14:143–155.

42. Nováková M, Praško J, Látalová K, Sládek M, Sumová A. The circadian system of patients with bipolar disorder differs in episodes of mania and depression. Bipolar Disord. 2015;17:303–314.

43. Warburton A, Savage AL, Myers P, Peeney D, Bubb VJ, Quinn JP. Molecular signatures of mood stabilisers highlight the role of the transcription factor REST/NRSF. J Affect Disord. 2015;172:63–73.

44. Campos-de-Sousa S, Guindalini C, Tondo L, Munro J, Osborne S, Floris G, et al. Nuclear Receptor Rev-Erb-α Circadian Gene Variants and Lithium Carbonate Prophylaxis in Bipolar Affective Disorder. J Biol Rhythms. 2010;25:132–137.

45. Kishi T, Kitajima T, Ikeda M, Yamanouchi Y, Kinoshita Y, Kawashima K, et al. Association analysis of nuclear receptor Rev-erb alpha gene (NR1D1) with mood disorders in the Japanese population. Neurosci Res. 2008;62:211–215.

46. Kripke DF, Nievergelt CM, Joo E, Shekhtman T, Kelsoe JR. Circadian polymorphisms associated with affective disorders. J Circadian Rhythms. 2009;7:2.

47. Severino G, Manchia M, Contu P, Squassina A, Lampus S, Ardau R, et al. Association study in a Sardinian sample between bipolar disorder

and the nuclear receptor REV-ERBalpha gene, a critical component of the circadian clock system. Bipolar Disord. 2009;11:215–220.

Supplementary table 5: Reagents and antibodies

1. Anti-Nestin (Life technologies, Cat # A24354)
2. Anti-Pax6 (Sigma-Aldrich, Cat # AB2237)
3. Anti-TOMM22, (Sigma-Aldrich, Cat # HPA003037)
4. B27 supplement without Vitamin A (Thermofisher-Gibco, Cat #12587-010)
5. Beta-mercaptoethanol (Thermofisher-Gibco, Cat # 21985-023)
6. BFGF (Thermofisher-Gibco, Cat # PHG6015)
7. Carbonyl cyanide m-chorophenyl hydrazone (CCCP) (Sigma-Aldrich, Cat # C2759)
8. Click-it Edu Alexa Fluor 488 imaging kit (Thermofisher-Invitrogen, Cat # C10337)
9. DAPI (40, 6-diamidino-2-phenylindole) (Thermofisher-Life Technologies, Cat # R37606).
10. DMEM/F12 (Thermofisher-Gibco, Cat #10565-018)
11. DNA isolation kit (Macherey-Nagel, Cat # 740951.50)
12. Fetal bovine serum (Thermofisher-Gibco, Cat # 10270106)
13. Glutamax (Thermofisher-Gibco, Cat # 35050-061)
14. Heparin (Sigma-Aldrich, Cat #H3149)
15. Knockout DMEM (Thermofisher-Gibco, Cat # 10829-018)
16. KOSR (Thermofisher-Gibco, Cat # 10828-028)
17. Lithium Chloride (Sigma-Aldrich, Cat # L7026)
18. Lonza Mycoplasma detection kit (Lonza, Cat # LT07-318)
19. Matrigel (Corning, Cat. #354277)
20. MitoTracker™ Deep Red FM (Thermofisher-Invitrogen, Cat # M22426)
21. N2 supplement (Thermofisher-Gibco, Cat #17502-048)
22. Non-Essential Amino Acids (Thermofisher-Gibco, Cat # 11140-050)
23. Paraformaldehyde (Sigma-Aldrich, Cat # P6148)

24. Penicillin-Streptomycin (Thermofisher-Invitrogen, Cat # 15140-122)
25. Propidium Iodide dye (Thermofisher-Invitrogen, Cat # P3566)
26. Real Time PCR (q-PCR) system (Thermofisher, Cat # AB7500)
27. RNase A (Thermofisher-Invitrogen, Cat # 12091021)
28. RPMI-1640 (Himedia, Cat # AL060A)
29. Secondary antibody Alexa flour 488 donkey anti-mouse (Life Technologies, Cat # A24350)
30. Secondary antibody Alexa flour 594 donkey anti-rabbit (Life Technologies, Cat # A24343)
31. StemPro Accutase (Thermofisher-Gibco, Cat # A1110501)
32. SuperScript™ VILO™ cDNA Synthesis Kit (Thermofisher-Invitrogen, Cat # 11754050)
33. Sytox Green (Thermofisher-Invitrogen, Cat # S7020)
34. Taqman gene expression -housekeeping gene assays (Thermofisher-Applied Biosystems, Cat # 4448485)
35. Taqman gene expression master mix (Thermofisher-Applied Biosystems, Cat # 4369016)
36. Taqman gene expression -target gene Assays (Thermofisher-Applied Biosystems, Cat # 4331182)
37. Triton X-100 (Invitrogen, Cat # A24352)
38. Trizol (Thermofisher-Ambion, # 15596-026)
39. Valproic acid sodium salt (Sigma-Aldrich, Cat # P4543)
40. Vectashield (Vector labs, Cat # H-1000)
41. Verapamil (Sigma-Aldrich, Cat # V4629)
