# Supplementary material for: Lithium response in bipolar disorder correlates with improved cell viability of patient derived cell lines
Source: Sci Rep. 2020 May 4;10:7428. doi: 10.1038/s41598-020-64202-1 (PMC7198534; doi:10.1038/s41598-020-64202-1)
Supplement: Supplementary file 1 — Supplementary Dataset 1. [file 41598_2020_64202_MOESM1_ESM.docx]

**Title**

Lithium response in bipolar disorder correlates with improved cell viability of patient derived cell lines

**Authors:** Pradip Paul^1^, Shruti Iyer^2^, Ravi Kumar Nadella^1^, Rashmitha Nayak^1^, Anirudh S. Chellappa^1^, Sheetal Ambardar^2,3^, Reeteka Sud^1^, Salil K. Sukumaran^1^, Meera Purushottam^1^, Sanjeev Jain^1,3^, ADBS Consortium (ADBS: The Accelerator program for Discovery in Brain disorders using Stem cells), Biju Viswanath^1^*

*Corresponding author

^1. The National Institute of Mental Health and Neurosciences (NIMHANS), India^

^2. Institute for Stem Cell Science and Regenerative Medicine (InStem), India^

^3. The National Centre for Biological Sciences (NCBS), India^

Accelerator Program for Discovery in Brain disorders using Stem cells (ADBS) Consortium

Naren P. Rao^1^, Janardhanan C. Narayanaswamy^1^, Palanimuthu T. Sivakumar^1^, Arun Kandasamy^1^, Muralidharan Kesavan^1^, Urvakhsh Meherwan Mehta^1^, Ganesan Venkatasubramanian^1^, John P. John^1^, Odity Mukherjee^2^, Ramakrishnan Kannan^1^, Bhupesh Mehta^1^, Thennarasu Kandavel^1^, B. Binukumar^1^, Jitender Saini^1^, Deepak Jayarajan^1^, A. Shyamsundar^1^, Sydney Moirangthem^1^, K. G. Vijay Kumar^1^, Jagadisha Thirthalli^1^, Prabha S. Chandra^1^, Bangalore N. Gangadhar^1^, Pratima Murthy^1^, Mitradas M. Panicker^3^, Upinder S. Bhalla^3^, Sumantra Chattarji^2^, Vivek Benegal^1^, Mathew Varghese^1^, Janardhan Y. C. Reddy^1^, Padinjat Raghu^3^, Mahendra Rao^2^

^1. National Institute of Mental Health and Neuro Sciences (NIMHANS), India^

^2. Institute for Stem Cell Biology and Regenerative Medicine (InStem), India^

^3. National Center for Biological Sciences (NCBS), Ind^^ia^

B2 vehicle versus B2 lithium

test_id	gene_id	gene	locus	sample_1	sample_2	status	value_1	value_2	log2(fold_change)	test_stat	p_value	q_value	significant

ENSG00000000003	ENSG00000000003	TSPAN6	X:99883666-99894988	q1	q2	OK	60.4392	63.3091	0.0669286	0.162402	0.81625	0.99995	no

ENSG00000000005	ENSG00000000005	TNMD	X:99839798-99854882	q1	q2	OK	9.77221	9.19522	-0.0878006	-0.133157	0.8454	0.99995	no

ENSG00000000419	ENSG00000000419	DPM1	20:49505584-49575092	q1	q2	OK	37.1283	40.9797	0.142389	0.0936919	0.8842	0.99995	no

ENSG00000000457	ENSG00000000457	SCYL3	1:169631244-169863408	q1	q2	OK	4.26558	4.41514	0.0497171	0.0540728	0.9323	0.99995	no

ENSG00000000460	ENSG00000000460	C1orf112	1:169631244-169863408	q1	q2	OK	11.7418	11.6547	-0.0107487	-0.0132415	0.9807	0.99995	no

ENSG00000000938	ENSG00000000938	FGR	1:27938574-27961788	q1	q2	NOTEST	0	0.0252873	inf	0	1	1	no

ENSG00000000971	ENSG00000000971	CFH	1:196621007-196716634	q1	q2	OK	0.888621	0.769193	-0.208222	-0.172521	0.7688	0.99995	no

ENSG00000001036	ENSG00000001036	FUCA2	6:143771943-143832827	q1	q2	OK	17.3248	17.549	0.0185501	0.0341089	0.96025	0.99995	no

ENSG00000001084	ENSG00000001084	GCLC	6:53362138-53481969	q1	q2	OK	10.4494	10.4712	0.00301545	0.00399618	0.9939	0.99995	no

ENSG00000001167	ENSG00000001167	NFYA	6:40994771-41067715	q1	q2	OK	31.6035	37.2784	0.238256	0.481228	0.5033	0.99995	no

ENSG00000001460	ENSG00000001460	STPG1	1:24634045-24799466	q1	q2	OK	1.37268	1.33104	-0.0444406	-0.0254626	0.9494	0.99995	no

ENSG00000001461	ENSG00000001461	NIPAL3	1:24634045-24799466	q1	q2	OK	1.68622	1.42667	-0.24114	-0.222729	0.63895	0.99995	no

ENSG00000001497	ENSG00000001497	LAS1L	X:64732461-64754655	q1	q2	OK	24.6692	23.6078	-0.0634458	-0.110007	0.8475	0.99995	no

ENSG00000001561	ENSG00000001561	ENPP4	6:46097729-46114436	q1	q2	OK	3.11187	3.25389	0.0643861	0.112438	0.8698	0.99995	no

ENSG00000001617	ENSG00000001617	SEMA3F	3:49977439-50226508	q1	q2	OK	5.86932	9.94186	0.760322	0.293674	0.5922	0.99995	no

ENSG00000001626	ENSG00000001626	CFTR	7:117105837-117514193	q1	q2	OK	0.567485	0.124449	-2.18903	-0.24654	0.07115	0.99995	no

ENSG00000001629	ENSG00000001629	ANKIB1	7:91875547-92030698	q1	q2	OK	17.7327	20.9151	0.23813	0.379345	0.4786	0.99995	no

ENSG00000001630	ENSG00000001630	CYP51A1	7:91741464-91810039	q1	q2	OK	66.7288	82.9479	0.313896	0.72394	0.28045	0.99995	no

ENSG00000001631	ENSG00000001631	KRIT1	7:91828282-91875480	q1	q2	OK	21.9151	22.4759	0.0364555	0.0602097	0.90805	0.99995	no

ENSG00000002016	ENSG00000002016	RAD52	12:1021242-1605099	q1	q2	OK	16.323	14.6462	-0.156378	-0.109551	0.79865	0.99995	no

ENSG00000002079	ENSG00000002079	MYH16	7:98836416-98908753	q1	q2	NOTEST	0.0112968	0.0741851	2.71521	0	1	1	no

ENSG00000002330	ENSG00000002330	BAD	11:64037301-64056972	q1	q2	OK	38.5745	33.3064	-0.211848	-0.319018	0.63085	0.99995	no

ENSG00000002549	ENSG00000002549	LAP3	4:17578814-17616194	q1	q2	OK	25.2714	27.2377	0.108098	0.232714	0.72975	0.99995	no

ENSG00000002586	ENSG00000002586	CD99	X:2609219-2659350	q1	q2	OK	219.378	190.42	-0.204234	-0.474437	0.4933	0.99995	no

ENSG00000002587	ENSG00000002587	HS3ST1	4:11394773-11431389	q1	q2	NOTEST	0.468616	0.430358	-0.122869	0	1	1	no

ENSG00000002726	ENSG00000002726	AOC1	7:150521714-150558592	q1	q2	NOTEST	0	0	0	0	1	1	no

ENSG00000002745	ENSG00000002745	WNT16	7:120965420-120981158	q1	q2	NOTEST	0.0551696	0.0456842	-0.272178	0	1	1	no

ENSG00000002746	ENSG00000002746	HECW1	7:43152197-43605600	q1	q2	NOTEST	0.264628	0.292126	0.142622	0	1	1	no

ENSG00000002822	ENSG00000002822	MAD1L1	7:1855428-2272878	q1	q2	OK	18.8812	15.6589	-0.269969	-0.459364	0.40605	0.99995	no

ENSG00000002834	ENSG00000002834	LASP1	17:37026111-37078023	q1	q2	OK	67.207	70.8863	0.0768958	0.185952	0.78915	0.99995	no

ENSG00000002919	ENSG00000002919	SNX11	17:46180718-46200436	q1	q2	OK	12.2937	13.6447	0.150426	0.251482	0.6824	0.99995	no

ENSG00000002933	ENSG00000002933	TMEM176A	7:150488372-150502208	q1	q2	NOTEST	0.0933431	0.146817	0.653407	0	1	1	no

ENSG00000003056	ENSG00000003056	M6PR	12:9066491-9102551	q1	q2	OK	45.9639	49.5877	0.109481	0.120947	0.7887	0.99995	no

ENSG00000003096	ENSG00000003096	KLHL13	X:117031775-117251303	q1	q2	OK	9.39087	9.80702	0.0625561	0.126611	0.8523	0.99995	no

ENSG00000003137	ENSG00000003137	CYP26B1	2:72356366-72375167	q1	q2	OK	36.7048	34.7951	-0.0770844	-0.143691	0.7975	0.99995	no

ENSG00000003147	ENSG00000003147	ICA1	7:8152813-8384146	q1	q2	OK	3.77822	3.0148	-0.325648	-0.329584	0.5324	0.99995	no

ENSG00000003249	ENSG00000003249	DBNDD1	16:90071272-90114181	q1	q2	OK	1.5283	1.24671	-0.293803	-0.264334	0.66795	0.99995	no

ENSG00000003393	ENSG00000003393	ALS2	2:202565276-202645912	q1	q2	OK	15.4807	17.1282	0.145907	0.243789	0.63895	0.99995	no

ENSG00000003400	ENSG00000003400	CASP10	2:202047603-202094129	q1	q2	NOTEST	0.0459552	0.14758	1.6832	0	1	1	no

ENSG00000003402	ENSG00000003402	CFLAR	2:201980826-202041410	q1	q2	OK	7.09975	7.89326	0.152853	0.162384	0.69945	0.99995	no

ENSG00000003436	ENSG00000003436	TFPI	2:187866602-188430487	q1	q2	OK	18.0067	19.9995	0.151434	0.215808	0.7545	0.99995	no

ENSG00000003509	ENSG00000003509	NDUFAF7	2:37394962-37551951	q1	q2	OK	15.8219	15.0668	-0.0705502	-0.0450625	0.94555	0.99995	no

ENSG00000003756	ENSG00000003756	RBM5	3:49977439-50226508	q1	q2	OK	137.718	136.789	-0.00976472	-0.0117411	0.9796	0.99995	no

ENSG00000003987	ENSG00000003987	MTMR7	8:17086736-17271037	q1	q2	OK	1.42249	1.66624	0.228175	0.0803101	0.8852	0.99995	no

ENSG00000003989	ENSG00000003989	SLC7A2	8:17354596-17428082	q1	q2	OK	1.54442	1.99591	0.369986	0.590034	0.38035	0.99995	no

ENSG00000004059	ENSG00000004059	ARF5	7:127220671-127242198	q1	q2	OK	123.965	114.307	-0.11702	-0.219118	0.75665	0.99995	no

ENSG00000004139	ENSG00000004139	SARM1	17:26684603-26734215	q1	q2	NOTEST	0.118528	0.0545016	-1.12086	0	1	1	no

ENSG00000004142	ENSG00000004142	POLDIP2	17:26662627-26684545	q1	q2	NOTEST	0	0	0	0	1	1	no

ENSG00000004399	ENSG00000004399	PLXND1	3:129274017-129325661	q1	q2	OK	21.2138	20.821	-0.026966	-0.0357985	0.9509	0.99995	no

ENSG00000004455	ENSG00000004455	AK2	1:33452552-33546597	q1	q2	OK	60.3642	61.6639	0.0307314	0.0574459	0.93275	0.99995	no

ENSG00000004468	ENSG00000004468	CD38	4:15779897-15854853	q1	q2	NOTEST	0.0897058	0.0886181	-0.0175993	0	1	1	no

ENSG00000004478	ENSG00000004478	FKBP4	12:2904118-3050306	q1	q2	OK	50.1941	50.5488	0.0101586	0.0104511	0.987	0.99995	no

ENSG00000004487	ENSG00000004487	KDM1A	1:23345940-23504301	q1	q2	OK	122.974	121.709	-0.0149222	-0.0292292	0.9626	0.99995	no

ENSG00000004534	ENSG00000004534	RBM6	3:49977439-50226508	q1	q2	OK	98.9571	94.8398	-0.0613093	-0.0673104	0.89885	0.99995	no

ENSG00000004660	ENSG00000004660	CAMKK1	17:3763608-3798185	q1	q2	OK	2.02003	2.12046	0.0699976	0.103043	0.88665	0.99995	no

ENSG00000004700	ENSG00000004700	RECQL	12:21590548-21654603	q1	q2	OK	17.7715	20.5011	0.206136	0.35118	0.53175	0.99995	no

ENSG00000004766	ENSG00000004766	CCDC132	7:92861652-92988338	q1	q2	OK	9.78387	10.3015	0.0743747	0.117465	0.8216	0.99995	no

ENSG00000004776	ENSG00000004776	HSPB6	19:36245466-36248980	q1	q2	NOTEST	0.308296	0.229001	-0.42896	0	1	1	no

ENSG00000004777	ENSG00000004777	ARHGAP33	19:36265433-36279724	q1	q2	OK	24.5718	22.3593	-0.136129	-0.105996	0.69425	0.99995	no

ENSG00000004779	ENSG00000004779	NDUFAB1	16:23592322-23607677	q1	q2	OK	87.7629	90.9663	0.0517203	0.116222	0.8651	0.99995	no

ENSG00000004799	ENSG00000004799	PDK4	7:95212810-95225803	q1	q2	NOTEST	0.471201	0.401781	-0.229934	0	1	1	no

ENSG00000004809	ENSG00000004809	SLC22A16	6:110745889-110797844	q1	q2	NOTEST	0	0.0174174	inf	0	1	1	no

ENSG00000004838	ENSG00000004838	ZMYND10	3:50378536-50384283	q1	q2	OK	1.49242	1.19945	-0.315282	-0.260099	0.6669	0.99995	no

ENSG00000004846	ENSG00000004846	ABCB5	7:20654829-20816658	q1	q2	NOTEST	0.0532663	0.031514	-0.75723	0	1	1	no

ENSG00000004848	ENSG00000004848	ARX	X:25021810-25034065	q1	q2	NOTEST	0.0186966	0.00922116	-1.01976	0	1	1	no

ENSG00000004864	ENSG00000004864	SLC25A13	7:95749531-95951459	q1	q2	OK	27.2327	29.9752	0.138432	0.282954	0.6329	0.99995	no

ENSG00000004866	ENSG00000004866	ST7	7:116592499-116870157	q1	q2	OK	14.7298	15.9857	0.118045	0.202354	0.7425	0.99995	no

ENSG00000004897	ENSG00000004897	CDC27	17:45195068-45266788	q1	q2	OK	39.9778	42.8508	0.100123	0.136134	0.7786	0.99995	no

ENSG00000004939	ENSG00000004939	SLC4A1	17:42325752-42355825	q1	q2	NOTEST	0.0137803	0.0048176	-1.51623	0	1	1	no

ENSG00000004948	ENSG00000004948	CALCR	7:93053798-93204042	q1	q2	NOTEST	0.0478712	0.0392651	-0.285912	0	1	1	no

ENSG00000004961	ENSG00000004961	HCCS	X:11129420-11683821	q1	q2	OK	13.7386	15.6695	0.18972	0.270565	0.6961	0.99995	no

ENSG00000004975	ENSG00000004975	DVL2	17:7128659-7137864	q1	q2	OK	31.4196	29.7098	-0.0807264	-0.151349	0.7953	0.99995	no

ENSG00000005001	ENSG00000005001	PRSS22	16:2902727-2909727	q1	q2	NOTEST	0	0	0	0	1	1	no

ENSG00000005007	ENSG00000005007	UPF1	19:18942746-18979045	q1	q2	OK	24.025	21.5641	-0.155905	-0.34415	0.57375	0.99995	no

ENSG00000005020	ENSG00000005020	SKAP2	7:26706680-27034858	q1	q2	OK	2.14097	2.19603	0.036634	0.0360803	0.9351	0.99995	no

ENSG00000005022	ENSG00000005022	SLC25A5	X:118599996-118605282	q1	q2	OK	192.412	199.486	0.0520885	0.122826	0.85395	0.99995	no

ENSG00000005059	ENSG00000005059	CCDC109B	4:110481360-110624739	q1	q2	OK	8.13987	8.95814	0.138193	0.186645	0.77885	0.99995	no

ENSG00000005073	ENSG00000005073	HOXA11	7:27221128-27233067	q1	q2	NOTEST	0.0607594	0.0439341	-0.467765	0	1	1	no

ENSG00000005075	ENSG00000005075	POLR2J	7:102105375-102119354	q1	q2	OK	32.1149	29.0333	-0.145531	-0.188401	0.7911	0.99995	no

ENSG00000005100	ENSG00000005100	DHX33	17:5185557-5372380	q1	q2	OK	10.6507	12.5327	0.234748	0.176769	0.76915	0.99995	no

ENSG00000005102	ENSG00000005102	MEOX1	17:41717755-41739322	q1	q2	OK	0.547433	1.18181	1.11025	1.03272	0.1274	0.99995	no

ENSG00000005108	ENSG00000005108	THSD7A	7:11221358-11871824	q1	q2	OK	8.90994	10.1235	0.184222	0.314326	0.62015	0.99995	no

ENSG00000005156	ENSG00000005156	LIG3	17:33254877-33332083	q1	q2	OK	22.3931	22.9876	0.0378013	0.048357	0.9141	0.99995	no

ENSG00000005175	ENSG00000005175	RPAP3	12:48057069-48099844	q1	q2	OK	22.9338	23.5352	0.0373444	0.0619489	0.90945	0.99995	no

ENSG00000005187	ENSG00000005187	ACSM3	16:20621564-20936328	q1	q2	OK	1.20321	1.35585	0.172308	0.0268534	0.8231	0.99995	no

ENSG00000005189	ENSG00000005189	AC004381.6	16:20621564-20936328	q1	q2	OK	5.19073	4.5059	-0.204123	-0.0789783	0.81015	0.99995	no

ENSG00000005194	ENSG00000005194	CIAPIN1	16:57462080-57521239	q1	q2	OK	47.7228	47.8235	0.00304183	0.00402908	0.9943	0.99995	no

ENSG00000005206	ENSG00000005206	SPPL2B	19:2321515-2355099	q1	q2	OK	41.2212	37.2956	-0.144381	-0.193587	0.7366	0.99995	no

ENSG00000005238	ENSG00000005238	FAM214B	9:35104108-35116338	q1	q2	OK	10.1557	9.56442	-0.0865435	-0.159809	0.8082	0.99995	no

ENSG00000005243	ENSG00000005243	COPZ2	17:46103532-46115392	q1	q2	OK	3.51376	3.11856	-0.172135	-0.148983	0.8106	0.99995	no

ENSG00000005249	ENSG00000005249	PRKAR2B	7:106685093-106802256	q1	q2	OK	12.2625	11.8173	-0.0533562	-0.116974	0.86335	0.99995	no

ENSG00000005302	ENSG00000005302	MSL3	X:11776277-11793870	q1	q2	OK	24.8201	26.7478	0.10791	0.171521	0.76045	0.99995	no

ENSG00000005339	ENSG00000005339	CREBBP	16:3775054-3930727	q1	q2	OK	24.4527	25.8199	0.0784904	0.0820186	0.8061	0.99995	no

ENSG00000005379	ENSG00000005379	BZRAP1	17:56378591-56494956	q1	q2	OK	7.56769	6.68871	-0.178123	-0.103931	0.8197	0.99995	no

ENSG00000005381	ENSG00000005381	MPO	17:56347216-56358296	q1	q2	NOTEST	0	0	0	0	1	1	no

ENSG00000005421	ENSG00000005421	PON1	7:94926987-95025680	q1	q2	NOTEST	0.0105653	0	#NAME?	0	1	1	no

ENSG00000005436	ENSG00000005436	GCFC2	2:75873908-75938115	q1	q2	OK	10.9656	11.5086	0.0697359	0.055955	0.9065	0.99995	no

ENSG00000005448	ENSG00000005448	WDR54	2:74648804-74652882	q1	q2	OK	27.7019	24.3665	-0.185088	-0.339946	0.63325	0.99995	no

ENSG00000005469	ENSG00000005469	CROT	7:86974996-87029111	q1	q2	OK	8.5102	9.12863	0.101204	0.126265	0.8197	0.99995	no

ENSG00000005471	ENSG00000005471	ABCB4	7:87031012-87109751	q1	q2	OK	1.15876	1.06827	-0.117307	-0.0850552	0.84915	0.99995	no

ENSG00000005483	ENSG00000005483	KMT2E	7:104653871-105039755	q1	q2	OK	32.0813	33.5579	0.0649211	0.07862	0.8657	0.99995	no

ENSG00000005486	ENSG00000005486	RHBDD2	7:75471919-75518244	q1	q2	OK	1.5618	1.53988	-0.0203918	-0.0153306	0.979	0.99995	no

ENSG00000005513	ENSG00000005513	SOX8	16:1031807-1036979	q1	q2	OK	13.2375	14.8239	0.163302	0.357814	0.6109	0.99995	no

ENSG00000005700	ENSG00000005700	IBTK	6:82879699-82957471	q1	q2	OK	16.9345	18.5374	0.130474	0.298521	0.6733	0.99995	no

ENSG00000005801	ENSG00000005801	ZNF195	11:3357950-3400448	q1	q2	OK	39.3034	43.6713	0.152029	0.294579	0.5979	0.99995	no

ENSG00000005810	ENSG00000005810	MYCBP2	13:77618791-77901185	q1	q2	OK	27.8693	28.801	0.0474427	0.0519453	0.9232	0.99995	no

ENSG00000005812	ENSG00000005812	FBXL3	13:77564794-77601330	q1	q2	OK	17.3361	17.2098	-0.010544	-0.0153962	0.97675	0.99995	no

ENSG00000005844	ENSG00000005844	ITGAL	16:30483978-30534506	q1	q2	NOTEST	0.010895	0.0303425	1.47768	0	1	1	no

ENSG00000005882	ENSG00000005882	PDK2	17:48172100-48207246	q1	q2	OK	13.531	12.9793	-0.0600548	-0.0617441	0.90775	0.99995	no

ENSG00000005884	ENSG00000005884	ITGA3	17:48133331-48167845	q1	q2	OK	4.12237	3.84463	-0.100628	-0.0873614	0.82435	0.99995	no

ENSG00000005889	ENSG00000005889	ZFX	X:24164341-24234372	q1	q2	OK	10.4858	11.0278	0.0726966	0.12197	0.82425	0.99995	no

ENSG00000005893	ENSG00000005893	LAMP2	X:119561681-119603220	q1	q2	OK	30.5581	31.6192	0.0492465	0.10896	0.879	0.99995	no

ENSG00000005955	ENSG00000005955	GGNBP2	17:34900736-34946278	q1	q2	OK	0.516135	0.604132	0.227116	0.174081	0.7043	0.99995	no

ENSG00000005961	ENSG00000005961	ITGA2B	17:42449547-42466873	q1	q2	OK	1.7137	1.21881	-0.491642	-0.253909	0.5107	0.99995	no

ENSG00000005981	ENSG00000005981	ASB4	7:95107755-95169544	q1	q2	NOTEST	0.0314165	0.0628222	0.999754	0	1	1	no

ENSG00000006007	ENSG00000006007	GDE1	16:19513010-19533467	q1	q2	OK	50.0644	47.8448	-0.0654222	-0.122133	0.86155	0.99995	no

ENSG00000006015	ENSG00000006015	C19orf60	19:18682539-18731849	q1	q2	OK	27.2743	22.4162	-0.282999	-0.196192	0.82005	0.99995	no

ENSG00000006016	ENSG00000006016	CRLF1	19:18682539-18731849	q1	q2	OK	2.12838	2.56945	0.271704	0.0548837	0.87445	0.99995	no

ENSG00000006025	ENSG00000006025	OSBPL7	17:45884737-45899200	q1	q2	OK	4.95423	4.09372	-0.27525	-0.30629	0.59355	0.99995	no

ENSG00000006042	ENSG00000006042	TMEM98	17:31254927-31272124	q1	q2	OK	86.8336	86.2392	-0.00990866	-0.0207866	0.97245	0.99995	no

ENSG00000006047	ENSG00000006047	YBX2	17:7184985-7197934	q1	q2	OK	1.0912	0.629906	-0.792706	-0.463964	0.42175	0.99995	no

ENSG00000006059	ENSG00000006059	KRT33A	17:39502343-39507064	q1	q2	NOTEST	0	0	0	0	1	1	no

ENSG00000006062	ENSG00000006062	MAP3K14	17:43268297-43394414	q1	q2	OK	3.64518	3.39344	-0.10324	-0.117266	0.82545	0.99995	no

ENSG00000006071	ENSG00000006071	ABCC8	11:17414431-17498449	q1	q2	OK	0.893643	0.675263	-0.404249	-0.245589	0.56805	0.99995	no

ENSG00000006074	ENSG00000006074	CCL18	17:34391639-34399392	q1	q2	NOTEST	0	0	0	0	1	1	no

ENSG00000006075	ENSG00000006075	CCL3	17:34400225-34417515	q1	q2	NOTEST	0.0821231	0	#NAME?	0	1	1	no

ENSG00000006114	ENSG00000006114	SYNRG	17:35874899-35969544	q1	q2	OK	19.3783	20.0709	0.0506598	0.079213	0.86815	0.99995	no

ENSG00000006116	ENSG00000006116	CACNG3	16:24266873-24374122	q1	q2	NOTEST	0.00866159	0.00795832	-0.122167	0	1	1	no

ENSG00000006118	ENSG00000006118	TMEM132A	11:60680637-60720002	q1	q2	OK	78.0422	74.8403	-0.0604388	-0.0868494	0.86085	0.99995	no

ENSG00000006125	ENSG00000006125	AP2B1	17:33901813-34053436	q1	q2	OK	51.6756	61.2367	0.244912	0.44709	0.43215	0.99995	no

ENSG00000006128	ENSG00000006128	TAC1	7:97361219-97369784	q1	q2	OK	1.76654	1.7878	0.0172541	0.01798	0.97765	0.99995	no

ENSG00000006194	ENSG00000006194	ZNF263	16:3313742-3368852	q1	q2	OK	18.9859	18.5407	-0.0342329	-0.0471982	0.94205	0.99995	no

ENSG00000006210	ENSG00000006210	CX3CL1	16:57406369-57418960	q1	q2	OK	9.2491	8.40765	-0.137612	-0.251502	0.68495	0.99995	no

ENSG00000006282	ENSG00000006282	SPATA20	17:48609903-48633213	q1	q2	OK	16.1869	13.5915	-0.252121	-0.424279	0.43555	0.99995	no

ENSG00000006283	ENSG00000006283	CACNA1G	17:48633567-48709301	q1	q2	OK	3.73829	4.23954	0.181529	0.26295	0.6654	0.99995	no

ENSG00000006327	ENSG00000006327	TNFRSF12A	16:3064712-3072384	q1	q2	OK	4.91602	5.36217	0.125325	0.157739	0.8115	0.99995	no

ENSG00000006377	ENSG00000006377	DLX6	7:96584452-96643377	q1	q2	NOTEST	0.0509318	0	#NAME?	0	1	1	no

ENSG00000006432	ENSG00000006432	MAP3K9	14:71189242-71276251	q1	q2	OK	2.93026	2.69684	-0.119759	-0.198563	0.72595	0.99995	no

ENSG00000006451	ENSG00000006451	RALA	7:39663081-39747723	q1	q2	OK	31.1827	34.5689	0.148727	0.355493	0.60655	0.99995	no

ENSG00000006453	ENSG00000006453	BAIAP2L1	7:97881690-98030380	q1	q2	OK	0.837824	1.02438	0.290031	0.0801353	0.8445	0.99995	no

ENSG00000006459	ENSG00000006459	KDM7A	7:139784545-139876835	q1	q2	OK	4.91072	4.94694	0.0106044	0.0231594	0.9744	0.99995	no

ENSG00000006468	ENSG00000006468	ETV1	7:13930852-14031050	q1	q2	OK	51.3916	55.9524	0.122667	0.215122	0.65835	0.99995	no

ENSG00000006530	ENSG00000006530	AGK	7:141229002-141401953	q1	q2	OK	13.5025	14.0976	0.0622253	0.084089	0.90345	0.99995	no

ENSG00000006534	ENSG00000006534	ALDH3B1	11:67776047-67797928	q1	q2	OK	0.533056	0.430342	-0.308802	-0.178054	0.6698	0.99995	no

ENSG00000006555	ENSG00000006555	TTC22	1:55245384-55266940	q1	q2	NOTEST	0	0.0480175	inf	0	1	1	no

ENSG00000006576	ENSG00000006576	PHTF2	7:77428121-77586818	q1	q2	OK	13.7506	14.7679	0.102967	0.18726	0.72805	0.99995	no

ENSG00000006606	ENSG00000006606	CCL26	7:75398850-75419214	q1	q2	NOTEST	0	0	0	0	1	1	no

ENSG00000006607	ENSG00000006607	FARP2	2:242295657-242449145	q1	q2	OK	12.3354	14.4365	0.226915	0.15175	0.65435	0.99995	no

ENSG00000006611	ENSG00000006611	USH1C	11:17515441-17565963	q1	q2	NOTEST	0.0282522	0	#NAME?	0	1	1	no

ENSG00000006625	ENSG00000006625	GGCT	7:30536236-30673649	q1	q2	OK	13.2013	16.9395	0.359712	0.196145	0.81525	0.99995	no

ENSG00000006634	ENSG00000006634	DBF4	7:87462882-87538856	q1	q2	OK	34.4942	40.8338	0.243412	0.346364	0.5758	0.99995	no

ENSG00000006638	ENSG00000006638	TBXA2R	19:3594503-3606838	q1	q2	NOTEST	0.19345	0.141661	-0.449526	0	1	1	no

ENSG00000006652	ENSG00000006652	IFRD1	7:112063022-112131117	q1	q2	OK	51.0875	56.124	0.135646	0.297351	0.6618	0.99995	no

ENSG00000006659	ENSG00000006659	LGALS14	19:40194945-40200084	q1	q2	NOTEST	0	0	0	0	1	1	no

ENSG00000006695	ENSG00000006695	COX10	17:13972812-14111994	q1	q2	OK	6.36019	6.77787	0.0917623	0.138598	0.83615	0.99995	no

ENSG00000006704	ENSG00000006704	GTF2IRD1	7:73868119-74016931	q1	q2	OK	0.350653	1.07384	1.61466	1.16154	0.0776	0.99995	no

ENSG00000006712	ENSG00000006712	PAF1	19:39876491-39881835	q1	q2	OK	43.1434	40.3634	-0.0960945	-0.177788	0.72925	0.99995	no

ENSG00000006715	ENSG00000006715	VPS41	7:38762562-38971994	q1	q2	OK	25.7125	26.0992	0.0215329	0.0304063	0.9544	0.99995	no

ENSG00000006740	ENSG00000006740	ARHGAP44	17:12569206-12894960	q1	q2	OK	2.35777	2.42654	0.0414768	0.0488968	0.92255	0.99995	no

ENSG00000006744	ENSG00000006744	ELAC2	17:12895707-12921504	q1	q2	OK	22.7251	22.9886	0.0166323	0.0310721	0.95415	0.99995	no

ENSG00000006747	ENSG00000006747	SCIN	7:12609750-12693228	q1	q2	NOTEST	0.151097	0.203892	0.432333	0	1	1	no

ENSG00000006756	ENSG00000006756	ARSD	X:2822010-2847392	q1	q2	OK	2.79816	2.83477	0.0187511	0.0227467	0.96375	0.99995	no

ENSG00000006757	ENSG00000006757	PNPLA4	X:7866287-7895780	q1	q2	OK	0.396155	0.689162	0.798779	0.379088	0.4724	0.99995	no

ENSG00000006788	ENSG00000006788	MYH13	17:10195136-10276447	q1	q2	NOTEST	0	0	0	0	1	1	no

ENSG00000006831	ENSG00000006831	ADIPOR2	12:1797739-1897844	q1	q2	OK	32.2403	34.8104	0.110652	0.247094	0.6981	0.99995	no

ENSG00000006837	ENSG00000006837	CDKL3	5:133450401-133706738	q1	q2	OK	6.79774	7.02003	0.046423	0.00887973	0.96595	0.99995	no

ENSG00000007001	ENSG00000007001	UPP2	2:158733213-158992666	q1	q2	NOTEST	0	0.0441541	inf	0	1	1	no

ENSG00000007038	ENSG00000007038	PRSS21	16:2867163-2876305	q1	q2	NOTEST	0	0	0	0	1	1	no

ENSG00000007047	ENSG00000007047	MARK4	19:45574757-45808541	q1	q2	OK	16.5918	17.4834	0.0755157	0.0926452	0.8633	0.99995	no

ENSG00000007062	ENSG00000007062	PROM1	4:15961865-16086001	q1	q2	OK	57.4792	58.9666	0.0368595	0.0673952	0.8988	0.99995	no

ENSG00000007080	ENSG00000007080	CCDC124	19:18043824-18054800	q1	q2	OK	74.0595	69.3392	-0.095013	-0.216544	0.75125	0.99995	no

ENSG00000007129	ENSG00000007129	CEACAM21	19:42051553-42093197	q1	q2	NOTEST	0	0.0209972	inf	0	1	1	no

ENSG00000007168	ENSG00000007168	PAFAH1B1	17:2496503-2588909	q1	q2	OK	80.1937	84.418	0.0740626	0.114182	0.7867	0.99995	no

ENSG00000007171	ENSG00000007171	NOS2	17:26083791-26221778	q1	q2	NOTEST	0.102745	0.0963102	-0.0933102	0	1	1	no

ENSG00000007174	ENSG00000007174	DNAH9	17:11501747-11900827	q1	q2	OK	1.55316	1.29595	-0.261206	-0.129768	0.74405	0.99995	no

ENSG00000007202	ENSG00000007202	KIAA0100	17:26782769-26972472	q1	q2	OK	40.1772	43.868	0.126793	0.0989271	0.7595	0.99995	no

ENSG00000007216	ENSG00000007216	SLC13A2	17:26782769-26972472	q1	q2	NOTEST	0	0	0	0	1	1	no

ENSG00000007237	ENSG00000007237	GAS7	17:9813925-10101868	q1	q2	OK	2.94026	2.19259	-0.423311	-0.501048	0.23815	0.99995	no

ENSG00000007255	ENSG00000007255	TRAPPC6A	19:45574757-45808541	q1	q2	OK	11.5476	7.08193	-0.705378	-0.333152	0.51085	0.99995	no

ENSG00000007264	ENSG00000007264	MATK	19:3777970-3802127	q1	q2	NOTEST	0	0	0	0	1	1	no

ENSG00000007306	ENSG00000007306	CEACAM7	19:42177234-42210895	q1	q2	NOTEST	0	0	0	0	1	1	no

ENSG00000007312	ENSG00000007312	CD79B	17:62006099-62009714	q1	q2	NOTEST	0.0508627	0.0256697	-0.986539	0	1	1	no

ENSG00000007314	ENSG00000007314	SCN4A	17:62015913-62050278	q1	q2	NOTEST	0.00624023	0.00301643	-1.04876	0	1	1	no

ENSG00000007341	ENSG00000007341	ST7L	1:113009162-113214241	q1	q2	OK	8.07414	8.02671	-0.00849829	-0.00372084	0.9901	0.99995	no

ENSG00000007350	ENSG00000007350	TKTL1	X:153524023-153558700	q1	q2	NOTEST	0.0450584	0	#NAME?	0	1	1	no

ENSG00000007372	ENSG00000007372	PAX6	11:31806339-32127301	q1	q2	OK	61.6598	48.1401	-0.357092	-0.246524	0.5762	0.99995	no

ENSG00000007376	ENSG00000007376	RPUSD1	16:834973-850737	q1	q2	OK	11.5685	10.1982	-0.181881	-0.18752	0.7726	0.99995	no

ENSG00000007384	ENSG00000007384	RHBDF1	16:108057-126354	q1	q2	OK	13.5122	12.8542	-0.0720208	-0.104269	0.88585	0.99995	no

ENSG00000007392	ENSG00000007392	LUC7L	16:238967-279462	q1	q2	OK	102.2	99.971	-0.0318151	-0.0680583	0.91985	0.99995	no

ENSG00000007402	ENSG00000007402	CACNA2D2	3:50384760-50541675	q1	q2	OK	3.78474	3.24545	-0.221776	-0.212782	0.77015	0.99995	no

ENSG00000007516	ENSG00000007516	BAIAP3	16:1383601-1401912	q1	q2	OK	10.6417	10.064	-0.0805217	-0.0697561	0.8635	0.99995	no

ENSG00000007520	ENSG00000007520	TSR3	16:1383601-1401912	q1	q2	OK	30.4992	26.4685	-0.204493	-0.300198	0.66675	0.99995	no

ENSG00000007541	ENSG00000007541	PIGQ	16:616994-634136	q1	q2	OK	29.561	26.2513	-0.171303	-0.317385	0.5846	0.99995	no

ENSG00000007545	ENSG00000007545	CRAMP1L	16:1662325-1752281	q1	q2	OK	15.3939	14.8992	-0.0471201	-0.0298418	0.9319	0.99995	no

ENSG00000007866	ENSG00000007866	TEAD3	6:35441373-35464853	q1	q2	OK	20.1642	19.4445	-0.0524342	-0.109664	0.8646	0.99995	no

ENSG00000007908	ENSG00000007908	SELE	1:169631244-169863408	q1	q2	NOTEST	0.163059	0.0154788	-3.39703	0	1	1	no

ENSG00000007923	ENSG00000007923	DNAJC11	1:6684925-6761984	q1	q2	OK	28.1565	29.3921	0.0619598	0.112635	0.85855	0.99995	no

ENSG00000007933	ENSG00000007933	FMO3	1:171060017-171086959	q1	q2	NOTEST	0.0265935	0	#NAME?	0	1	1	no

ENSG00000007944	ENSG00000007944	MYLIP	6:16129355-16148479	q1	q2	OK	9.30878	8.68595	-0.0999092	-0.172891	0.80145	0.99995	no

ENSG00000007952	ENSG00000007952	NOX1	X:100098312-100129334	q1	q2	NOTEST	0.0622393	0.0526731	-0.24076	0	1	1	no

ENSG00000007968	ENSG00000007968	E2F2	1:23832921-23857712	q1	q2	OK	10.7477	9.65981	-0.153958	-0.231187	0.64635	0.99995	no

ENSG00000008018	ENSG00000008018	PSMB1	6:170844204-170862429	q1	q2	OK	170.501	158.195	-0.108078	-0.262962	0.7003	0.99995	no

ENSG00000008056	ENSG00000008056	SYN1	X:47420515-47479252	q1	q2	OK	2.56111	2.09945	-0.286755	-0.160034	0.8643	0.99995	no

ENSG00000008083	ENSG00000008083	JARID2	6:15246526-15522252	q1	q2	OK	28.5054	32.9762	0.210189	0.51629	0.451	0.99995	no

ENSG00000008086	ENSG00000008086	CDKL5	X:18443702-18690229	q1	q2	OK	1.27284	1.12613	-0.176679	-0.178536	0.7883	0.99995	no

ENSG00000008118	ENSG00000008118	CAMK1G	1:209757061-209787283	q1	q2	OK	1.60483	2.24583	0.484827	0.530101	0.3994	0.99995	no

ENSG00000008128	ENSG00000008128	CDK11A	1:1634168-1677431	q1	q2	OK	19.8573	18.4551	-0.105648	-0.120624	0.84215	0.99995	no

ENSG00000008130	ENSG00000008130	NADK	1:1682670-1711896	q1	q2	OK	33.635	28.1499	-0.256832	-0.334742	0.51875	0.99995	no

ENSG00000008196	ENSG00000008196	TFAP2B	6:50786435-50815326	q1	q2	NOTEST	0.100684	0.0514337	-0.969044	0	1	1	no

ENSG00000008197	ENSG00000008197	TFAP2D	6:50681540-50740701	q1	q2	NOTEST	0	0	0	0	1	1	no

ENSG00000008226	ENSG00000008226	DLEC1	3:38080695-38178733	q1	q2	OK	0.924844	0.763627	-0.276343	-0.100505	0.80975	0.99995	no

ENSG00000008256	ENSG00000008256	CYTH3	7:6201406-6312275	q1	q2	OK	15.5857	14.6857	-0.0858137	-0.161044	0.76755	0.99995	no

ENSG00000008277	ENSG00000008277	ADAM22	7:87563457-87832204	q1	q2	OK	5.76218	6.02885	0.0652688	0.082115	0.8382	0.99995	no

ENSG00000008282	ENSG00000008282	SYPL1	7:105730948-105753022	q1	q2	OK	22.6227	23.3695	0.0468528	0.0988223	0.8797	0.99995	no

ENSG00000008283	ENSG00000008283	CYB561	17:61509664-61523739	q1	q2	OK	3.06015	2.71283	-0.173803	-0.205159	0.66645	0.99995	no

ENSG00000008294	ENSG00000008294	SPAG9	17:49039534-49198226	q1	q2	OK	83.9538	81.7716	-0.0379968	-0.0510584	0.9215	0.99995	no

ENSG00000008300	ENSG00000008300	CELSR3	3:48673901-48700348	q1	q2	OK	16.9776	14.7184	-0.206005	-0.267021	0.6086	0.99995	no

ENSG00000008311	ENSG00000008311	AASS	7:121715700-121784334	q1	q2	OK	40.4215	45.2717	0.163486	0.361525	0.598	0.99995	no

ENSG00000008323	ENSG00000008323	PLEKHG6	12:6419601-6437672	q1	q2	NOTEST	0.148631	0.211262	0.507293	0	1	1	no

ENSG00000008324	ENSG00000008324	SS18L2	3:42589460-42709072	q1	q2	OK	24.9404	27.8823	0.160863	0.071699	0.9479	0.99995	no

ENSG00000008382	ENSG00000008382	MPND	19:4343523-4360083	q1	q2	OK	17.545	14.741	-0.25123	-0.378012	0.52375	0.99995	no

ENSG00000008394	ENSG00000008394	MGST1	12:16500075-16763528	q1	q2	OK	15.7569	15.9841	0.020658	0.0170158	0.98005	0.99995	no

ENSG00000008405	ENSG00000008405	CRY1	12:107385141-107517092	q1	q2	OK	16.3729	16.8931	0.0451295	0.0757368	0.88985	0.99995	no

ENSG00000008438	ENSG00000008438	PGLYRP1	19:46498338-46526323	q1	q2	NOTEST	0.0965192	0.0944022	-0.031995	0	1	1	no

ENSG00000008441	ENSG00000008441	NFIX	19:13106421-13209610	q1	q2	OK	7.47593	7.07377	-0.0797742	-0.109048	0.86015	0.99995	no

ENSG00000008513	ENSG00000008513	ST3GAL1	8:134467090-134584183	q1	q2	OK	2.79955	2.96998	0.0852549	0.0871835	0.82145	0.99995	no

ENSG00000008516	ENSG00000008516	MMP25	16:3096681-3110727	q1	q2	OK	2.04789	1.88641	-0.118495	-0.0755325	0.9142	0.99995	no

ENSG00000008517	ENSG00000008517	IL32	16:3115297-3137101	q1	q2	NOTEST	0.431937	0.337546	-0.355738	0	1	1	no

ENSG00000008710	ENSG00000008710	PKD1	16:2089815-2185899	q1	q2	OK	28.4996	26.8845	-0.0841676	-0.0596308	0.8741	0.99995	no

ENSG00000008735	ENSG00000008735	MAPK8IP2	22:51007289-51052409	q1	q2	OK	12.2657	10.8146	-0.181645	-0.216472	0.7655	0.99995	no

ENSG00000008838	ENSG00000008838	MED24	17:38175349-38256978	q1	q2	OK	38.7961	41.4033	0.0938346	0.124227	0.79885	0.99995	no

ENSG00000008853	ENSG00000008853	RHOBTB2	8:22545171-22941132	q1	q2	OK	15.5702	14.1724	-0.135704	-0.127578	0.79565	0.99995	no

ENSG00000008869	ENSG00000008869	HEATR5B	2:37195525-37311485	q1	q2	OK	7.20325	7.80183	0.115165	0.187991	0.7117	0.99995	no

ENSG00000008952	ENSG00000008952	SEC62	3:169657140-169716161	q1	q2	OK	49.6314	51.4922	0.0530994	0.0933519	0.88275	0.99995	no

ENSG00000008988	ENSG00000008988	RPS20	8:56979853-56987069	q1	q2	OK	1208.07	1159.17	-0.0596161	-0.136023	0.8424	0.99995	no

ENSG00000009307	ENSG00000009307	CSDE1	1:115259533-115301297	q1	q2	OK	283.062	295.341	0.0612642	0.0902101	0.85645	0.99995	no

ENSG00000009335	ENSG00000009335	UBE3C	7:156931606-157062066	q1	q2	OK	21.9216	24.8203	0.179169	0.256201	0.53755	0.99995	no

ENSG00000009413	ENSG00000009413	REV3L	6:111620233-111927481	q1	q2	OK	16.6826	16.9505	0.0229782	0.0284255	0.95795	0.99995	no

ENSG00000009694	ENSG00000009694	TENM1	X:123093082-124097666	q1	q2	OK	9.74931	9.4277	-0.0483934	-0.067778	0.9231	0.99995	no

ENSG00000009709	ENSG00000009709	PAX7	1:18957499-19075360	q1	q2	OK	0.964727	0.838863	-0.201686	-0.249041	0.69235	0.99995	no

ENSG00000009724	ENSG00000009724	MASP2	1:11086579-11107290	q1	q2	OK	2.65349	2.6367	-0.00916067	-0.00972754	0.98765	0.99995	no

ENSG00000009765	ENSG00000009765	IYD	6:150690027-150727105	q1	q2	NOTEST	0	0	0	0	1	1	no

ENSG00000009780	ENSG00000009780	FAM76A	1:28052489-28089633	q1	q2	OK	3.93957	4.09721	0.0566005	0.0695799	0.89635	0.99995	no

ENSG00000009790	ENSG00000009790	TRAF3IP3	1:209929376-209957904	q1	q2	OK	1.31601	1.18965	-0.145624	-0.0773291	0.8499	0.99995	no

ENSG00000009830	ENSG00000009830	POMT2	14:77741298-77797940	q1	q2	OK	17.9312	17.0612	-0.0717524	-0.0802284	0.85485	0.99995	no

ENSG00000009844	ENSG00000009844	VTA1	6:142468366-142545826	q1	q2	OK	35.5339	38.7233	0.124006	0.213892	0.75985	0.99995	no

ENSG00000009950	ENSG00000009950	MLXIPL	7:73007523-73038873	q1	q2	NOTEST	0.00893428	0	#NAME?	0	1	1	no

ENSG00000009954	ENSG00000009954	BAZ1B	7:72854727-72936608	q1	q2	OK	1.70383	1.2078	-0.496398	-0.704406	0.30835	0.99995	no

ENSG00000010017	ENSG00000010017	RANBP9	6:13615558-13711796	q1	q2	OK	30.3301	33.2767	0.133765	0.224979	0.7223	0.99995	no

ENSG00000010030	ENSG00000010030	ETV7	6:36322418-36458920	q1	q2	NOTEST	0	0	0	0	1	1	no

ENSG00000010072	ENSG00000010072	SPRTN	1:231468479-231490769	q1	q2	OK	7.56798	8.16401	0.109369	0.15921	0.80045	0.99995	no

ENSG00000010165	ENSG00000010165	METTL13	1:171750787-171783163	q1	q2	OK	14.3634	14.7863	0.0418617	0.0750052	0.9016	0.99995	no

ENSG00000010219	ENSG00000010219	DYRK4	12:4671369-4960277	q1	q2	OK	20.7919	20.6284	-0.0113864	-0.00914314	0.99025	0.99995	no

ENSG00000010244	ENSG00000010244	ZNF207	17:30593194-30708905	q1	q2	OK	136.064	137.36	0.0136715	0.0206191	0.9697	0.99995	no

ENSG00000010256	ENSG00000010256	UQCRC1	3:48636434-48648409	q1	q2	OK	157.345	158.379	0.00944755	0.0163622	0.9791	0.99995	no

ENSG00000010270	ENSG00000010270	STARD3NL	7:38217823-38270272	q1	q2	OK	55.9259	59.0477	0.0783657	0.184246	0.79225	0.99995	no

ENSG00000010278	ENSG00000010278	CD9	12:6308880-6347425	q1	q2	OK	1.81103	1.88573	0.0583142	0.0601077	0.9225	0.99995	no

ENSG00000010282	ENSG00000010282	HHATL	3:42734154-42846023	q1	q2	NOTEST	0.0941835	0.16931	0.846124	0	1	1	no

ENSG00000010292	ENSG00000010292	NCAPD2	12:6601149-6641121	q1	q2	OK	52.7775	52.9013	0.00337847	0.00435217	0.992	0.99995	no

ENSG00000010295	ENSG00000010295	IFFO1	12:6647540-6665239	q1	q2	OK	10.7775	10.213	-0.0776263	-0.147997	0.8338	0.99995	no

ENSG00000010310	ENSG00000010310	GIPR	19:46171501-46186982	q1	q2	OK	1.3017	1.52351	0.227005	0.170229	0.72655	0.99995	no

ENSG00000010318	ENSG00000010318	PHF7	3:52444672-52457657	q1	q2	OK	3.73037	2.2568	-0.72504	-0.725009	0.2731	0.99995	no

ENSG00000010319	ENSG00000010319	SEMA3G	3:52467068-52479101	q1	q2	NOTEST	0.135427	0.105913	-0.354639	0	1	1	no

ENSG00000010322	ENSG00000010322	NISCH	3:52489133-52527087	q1	q2	OK	57.8699	55.0702	-0.0715414	-0.107469	0.8322	0.99995	no

ENSG00000010327	ENSG00000010327	STAB1	3:52529353-52742182	q1	q2	NOTEST	0.0358308	0.0207953	-0.784939	0	1	1	no

ENSG00000010361	ENSG00000010361	FUZ	19:50270224-50320633	q1	q2	OK	19.5474	15.0306	-0.379071	-0.315314	0.54015	0.99995	no

ENSG00000010379	ENSG00000010379	SLC6A13	12:329788-372039	q1	q2	NOTEST	0.21336	0.157368	-0.43915	0	1	1	no

ENSG00000010404	ENSG00000010404	IDS	X:148558520-148632055	q1	q2	OK	1.08721	2.15772	0.988876	0.99454	0.0709	0.99995	no

ENSG00000010438	ENSG00000010438	PRSS3	9:33750514-33920402	q1	q2	NOTEST	0.0974472	0.289654	1.57164	0	1	1	no

ENSG00000010539	ENSG00000010539	ZNF200	16:3238211-3286221	q1	q2	OK	10.2646	10.6142	0.0483289	0.0881303	0.89115	0.99995	no

ENSG00000010610	ENSG00000010610	CD4	12:6896023-6929974	q1	q2	NOTEST	0.0629437	0.310948	2.30454	0	1	1	no

ENSG00000010626	ENSG00000010626	LRRC23	12:6976282-7032861	q1	q2	NOTEST	0.0235429	0	#NAME?	0	1	1	no

ENSG00000010671	ENSG00000010671	BTK	X:100604434-100641183	q1	q2	NOTEST	0	0	0	0	1	1	no

ENSG00000010704	ENSG00000010704	HFE	6:26087508-26098571	q1	q2	OK	0.667717	0.56247	-0.24746	-0.130589	0.74275	0.99995	no

ENSG00000010803	ENSG00000010803	SCMH1	1:41492871-41707826	q1	q2	OK	16.2867	16.4285	0.0125088	0.0201347	0.96485	0.99995	no

ENSG00000010810	ENSG00000010810	FYN	6:111981534-112194655	q1	q2	OK	198.689	204.26	0.0398942	0.0718096	0.90155	0.99995	no

ENSG00000010818	ENSG00000010818	HIVEP2	6:143069579-143266338	q1	q2	OK	1.07083	1.05448	-0.0222005	-0.0284238	0.9618	0.99995	no

ENSG00000010932	ENSG00000010932	FMO1	1:171216718-171255117	q1	q2	NOTEST	0.0172958	0.0743893	2.10467	0	1	1	no

ENSG00000011007	ENSG00000011007	TCEB3	1:24069644-24104777	q1	q2	OK	24.0583	24.3626	0.0181366	0.0378981	0.95455	0.99995	no

ENSG00000011009	ENSG00000011009	LYPLA2	1:24117459-24122029	q1	q2	OK	37.0025	32.4704	-0.188499	-0.399802	0.5458	0.99995	no

ENSG00000011021	ENSG00000011021	CLCN6	1:11821843-11908402	q1	q2	OK	19.7234	21.5722	0.129267	0.19887	0.715	0.99995	no

ENSG00000011028	ENSG00000011028	MRC2	17:60704761-60770958	q1	q2	OK	43.6954	37.444	-0.222745	-0.503419	0.48015	0.99995	no

ENSG00000011052	ENSG00000011052	NME2	17:49230896-49249108	q1	q2	OK	26.5908	29.256	0.137802	0.0851408	0.9024	0.99995	no

ENSG00000011083	ENSG00000011083	SLC6A7	5:149569519-149669854	q1	q2	NOTEST	0.0212723	0.0103113	-1.04475	0	1	1	no

ENSG00000011105	ENSG00000011105	TSPAN9	12:3186520-3395730	q1	q2	OK	18.5871	15.1454	-0.295425	-0.590824	0.35445	0.99995	no

ENSG00000011114	ENSG00000011114	BTBD7	14:93703895-93799438	q1	q2	OK	11.0809	12.3892	0.161011	0.33828	0.63215	0.99995	no

ENSG00000011132	ENSG00000011132	APBA3	19:3750816-3761697	q1	q2	OK	9.17872	8.77289	-0.0652408	-0.0759397	0.9103	0.99995	no

ENSG00000011143	ENSG00000011143	MKS1	17:56282802-56345879	q1	q2	OK	15.9732	14.2628	-0.1634	-0.24359	0.7051	0.99995	no

ENSG00000011198	ENSG00000011198	ABHD5	3:43328003-43775863	q1	q2	OK	6.48997	6.09831	-0.0898025	-0.0725905	0.90285	0.99995	no

ENSG00000011201	ENSG00000011201	KAL1	X:8496914-8700227	q1	q2	OK	2.90978	2.5085	-0.214082	-0.276696	0.6028	0.99995	no

ENSG00000011243	ENSG00000011243	AKAP8L	19:15464195-15529952	q1	q2	OK	66.2309	61.6055	-0.104445	-0.172272	0.78375	0.99995	no

ENSG00000011258	ENSG00000011258	MBTD1	17:49254785-49337524	q1	q2	OK	22.6366	23.6435	0.0627865	0.134827	0.82895	0.99995	no

ENSG00000011260	ENSG00000011260	UTP18	17:49337888-49375297	q1	q2	OK	26.8935	31.5185	0.228945	0.495456	0.45	0.99995	no

ENSG00000011275	ENSG00000011275	RNF216	7:5659677-5821370	q1	q2	OK	16.1461	15.763	-0.0346398	-0.0768525	0.9052	0.99995	no

ENSG00000011295	ENSG00000011295	TTC19	17:15879873-16256970	q1	q2	OK	29.7236	29.8601	0.00660831	0.00541659	0.99465	0.99995	no

ENSG00000011304	ENSG00000011304	PTBP1	19:797074-812327	q1	q2	OK	140.151	135.951	-0.0438982	-0.0723352	0.8728	0.99995	no

ENSG00000011332	ENSG00000011332	DPF1	19:38701645-38720354	q1	q2	OK	27.972	26.4871	-0.0786953	-0.156298	0.80575	0.99995	no

ENSG00000011347	ENSG00000011347	SYT7	11:61282784-61348620	q1	q2	OK	2.35209	2.269	-0.0518889	-0.0704475	0.90235	0.99995	no

ENSG00000011376	ENSG00000011376	LARS2	3:45429997-45590913	q1	q2	OK	9.4502	10.3304	0.128476	0.240582	0.68705	0.99995	no

ENSG00000011405	ENSG00000011405	PIK3C2A	11:17095935-17229530	q1	q2	OK	23.017	28.3786	0.302109	0.209969	0.5742	0.99995	no

ENSG00000011422	ENSG00000011422	PLAUR	19:44150246-44174699	q1	q2	OK	6.21266	6.03578	-0.0416711	-0.0478751	0.94385	0.99995	no

ENSG00000011426	ENSG00000011426	ANLN	7:36363829-36493400	q1	q2	OK	31.1272	32.6344	0.0682204	0.129719	0.8276	0.99995	no

ENSG00000011451	ENSG00000011451	WIZ	19:15532318-15560762	q1	q2	OK	40.7593	36.5029	-0.159117	-0.355537	0.57665	0.99995	no

ENSG00000011454	ENSG00000011454	RABGAP1	9:125703111-125867145	q1	q2	OK	35.208	37.3806	0.0863861	0.149459	0.7734	0.99995	no

ENSG00000011465	ENSG00000011465	DCN	12:91539024-91576900	q1	q2	OK	215.227	238.742	0.149591	0.214258	0.7205	0.99995	no

ENSG00000011478	ENSG00000011478	QPCTL	19:46190711-46207247	q1	q2	OK	5.15533	5.58131	0.114538	0.0613913	0.9147	0.99995	no

ENSG00000011485	ENSG00000011485	PPP5C	19:46850250-46896238	q1	q2	OK	57.4952	56.3221	-0.029741	-0.0583421	0.92045	0.99995	no

ENSG00000011523	ENSG00000011523	CEP68	2:65283499-65357240	q1	q2	OK	12.1406	11.9068	-0.028048	-0.0292391	0.96435	0.99995	no

ENSG00000011566	ENSG00000011566	MAP4K3	2:39476406-39945103	q1	q2	OK	18.5136	21.0063	0.182238	0.247507	0.59145	0.99995	no

ENSG00000011590	ENSG00000011590	ZBTB32	19:36195428-36207940	q1	q2	NOTEST	0.353067	0.242201	-0.54374	0	1	1	no

ENSG00000011600	ENSG00000011600	TYROBP	19:36395302-36399197	q1	q2	NOTEST	0	0	0	0	1	1	no

ENSG00000011638	ENSG00000011638	TMEM159	16:20944432-21191937	q1	q2	OK	16.6084	17.5248	0.0774847	0.124159	0.85585	0.99995	no

ENSG00000011677	ENSG00000011677	GABRA3	X:151307354-151619830	q1	q2	NOTEST	0.285783	0.34382	0.266734	0	1	1	no

ENSG00000012048	ENSG00000012048	BRCA1	17:41196311-41277500	q1	q2	OK	20.4573	19.0595	-0.102102	-0.142036	0.7619	0.99995	no

ENSG00000012061	ENSG00000012061	ERCC1	19:45882891-45982086	q1	q2	OK	43.7336	42.4839	-0.0418265	-0.0714091	0.91425	0.99995	no

ENSG00000012124	ENSG00000012124	CD22	19:35810163-35838258	q1	q2	NOTEST	0	0	0	0	1	1	no

ENSG00000012171	ENSG00000012171	SEMA3B	3:50304989-50314977	q1	q2	NOTEST	0.216748	0.198846	-0.124366	0	1	1	no

ENSG00000012174	ENSG00000012174	MBTPS2	X:21857753-21903542	q1	q2	OK	9.29698	9.43282	0.0209269	0.0447852	0.9495	0.99995	no

ENSG00000012211	ENSG00000012211	PRICKLE3	X:49028272-49042845	q1	q2	NOTEST	0	0	0	0	1	1	no

ENSG00000012223	ENSG00000012223	LTF	3:46477135-46526724	q1	q2	NOTEST	0	0	0	0	1	1	no

ENSG00000012232	ENSG00000012232	EXTL3	8:28457985-28613116	q1	q2	OK	29.8734	29.832	-0.00200246	-0.0039001	0.99295	0.99995	no

ENSG00000012504	ENSG00000012504	NR1H4	12:100867485-100958191	q1	q2	NOTEST	0.0781623	0.0736856	-0.0850906	0	1	1	no

ENSG00000012660	ENSG00000012660	ELOVL5	6:53132195-53213947	q1	q2	OK	78.297	88.0325	0.169079	0.375459	0.54595	0.99995	no

ENSG00000012779	ENSG00000012779	ALOX5	10:45869660-45948569	q1	q2	NOTEST	0.010859	0	#NAME?	0	1	1	no

ENSG00000012817	ENSG00000012817	KDM5D	Y:21865750-21906825	q1	q2	OK	19.2978	17.2602	-0.160985	-0.259486	0.63565	0.99995	no

ENSG00000012822	ENSG00000012822	CALCOCO1	12:54104902-54121529	q1	q2	OK	25.0187	19.4623	-0.362321	-0.614617	0.2821	0.99995	no

ENSG00000012963	ENSG00000012963	UBR7	14:93651295-93695561	q1	q2	OK	25.6176	26.4441	0.045811	0.087988	0.88825	0.99995	no

ENSG00000012983	ENSG00000012983	MAP4K5	14:50885218-51135049	q1	q2	OK	22.6306	25.2058	0.155485	0.165503	0.70575	0.99995	no

ENSG00000013016	ENSG00000013016	EHD3	2:31456879-31492313	q1	q2	OK	4.13045	3.87942	-0.0904611	-0.154823	0.8231	0.99995	no

ENSG00000013275	ENSG00000013275	PSMC4	19:40476911-40487348	q1	q2	OK	69.1119	63.4919	-0.122361	-0.293681	0.66795	0.99995	no

ENSG00000013288	ENSG00000013288	MAN2B2	4:6576901-6625089	q1	q2	OK	3.72297	3.42712	-0.119459	-0.187727	0.7525	0.99995	no

ENSG00000013293	ENSG00000013293	SLC7A14	3:170136652-170578169	q1	q2	NOTEST	0.0973867	0.0778487	-0.323053	0	1	1	no

ENSG00000013297	ENSG00000013297	CLDN11	3:170136652-170578169	q1	q2	OK	2.43841	2.55885	0.0695517	0.077731	0.9001	0.99995	no

ENSG00000013306	ENSG00000013306	SLC25A39	17:42396992-42402238	q1	q2	OK	36.6984	31.4887	-0.220883	-0.459429	0.48835	0.99995	no

ENSG00000013364	ENSG00000013364	MVP	16:29817426-29859355	q1	q2	OK	1.74061	1.2124	-0.521726	-0.0923871	0.70865	0.99995	no

ENSG00000013374	ENSG00000013374	NUB1	7:151038784-151110440	q1	q2	OK	30.3588	30.6512	0.0138321	0.0219622	0.97005	0.99995	no

ENSG00000013375	ENSG00000013375	PGM3	6:83777384-83908651	q1	q2	OK	17.9976	23.1086	0.360622	0.294322	0.30985	0.99995	no

ENSG00000013392	ENSG00000013392	RWDD2A	6:83777384-83908651	q1	q2	OK	6.41363	5.88807	-0.123348	-0.0919312	0.90445	0.99995	no

ENSG00000013441	ENSG00000013441	CLK1	2:201717731-201729422	q1	q2	OK	77.316	79.6767	0.043392	0.0786644	0.88355	0.99995	no

ENSG00000013503	ENSG00000013503	POLR3B	12:106751435-107283090	q1	q2	OK	5.57236	6.16667	0.146202	0.122796	0.86545	0.99995	no

ENSG00000013523	ENSG00000013523	ANGEL1	14:77253587-77292589	q1	q2	OK	10.557	9.84445	-0.100819	-0.165401	0.78165	0.99995	no

ENSG00000013561	ENSG00000013561	RNF14	5:141323149-141369856	q1	q2	OK	20.3211	22.1533	0.124543	0.199844	0.72105	0.99995	no

ENSG00000013563	ENSG00000013563	DNASE1L1	X:153618314-153650065	q1	q2	OK	0.538944	0.757414	0.490946	0.15135	0.61985	0.99995	no

ENSG00000013573	ENSG00000013573	DDX11	12:31173696-31257725	q1	q2	OK	38.8492	36.296	-0.0980777	-0.145703	0.76425	0.99995	no

ENSG00000013583	ENSG00000013583	HEBP1	12:13080659-13193613	q1	q2	OK	18.4039	15.4977	-0.247961	-0.437075	0.5256	0.99995	no

ENSG00000013588	ENSG00000013588	GPRC5A	12:13043715-13070871	q1	q2	NOTEST	0.0838427	0.1031	0.298285	0	1	1	no

ENSG00000013619	ENSG00000013619	MAMLD1	X:149529688-149682448	q1	q2	OK	1.13077	1.14155	0.0136901	0.0161834	0.97725	0.99995	no

ENSG00000013725	ENSG00000013725	CD6	11:60739114-60787849	q1	q2	NOTEST	0.0842867	0.0328197	-1.36074	0	1	1	no

ENSG00000013810	ENSG00000013810	TACC3	4:1723226-1746898	q1	q2	OK	69.3664	67.6053	-0.0370999	-0.0694897	0.90205	0.99995	no

ENSG00000014123	ENSG00000014123	UFL1	6:96806249-97003152	q1	q2	OK	10.8138	11.4477	0.0821839	0.179522	0.79325	0.99995	no

ENSG00000014138	ENSG00000014138	POLA2	11:65029232-65073060	q1	q2	OK	27.2165	24.7192	-0.138851	-0.235887	0.7137	0.99995	no

ENSG00000014164	ENSG00000014164	ZC3H3	8:144519824-144623623	q1	q2	OK	7.54603	6.56806	-0.20025	-0.377392	0.5882	0.99995	no

ENSG00000014216	ENSG00000014216	CAPN1	11:64945076-64979477	q1	q2	OK	26.3642	23.1869	-0.185269	-0.348243	0.54165	0.99995	no

ENSG00000014257	ENSG00000014257	ACPP	3:132036210-132087142	q1	q2	NOTEST	0.0239237	0.0970063	2.01964	0	1	1	no

ENSG00000014641	ENSG00000014641	MDH1	2:63348517-64054977	q1	q2	OK	124.786	124.699	-0.00101101	-0.00234122	0.9978	0.99995	no

ENSG00000014824	ENSG00000014824	SLC30A9	4:41992488-42092474	q1	q2	OK	42.5819	40.574	-0.0696839	-0.116975	0.8726	0.99995	no

ENSG00000014914	ENSG00000014914	MTMR11	1:149900542-149908791	q1	q2	NOTEST	0.277488	0.279941	0.012698	0	1	1	no

ENSG00000014919	ENSG00000014919	COX15	10:101419262-101515891	q1	q2	OK	25.6627	24.6574	-0.0576514	-0.0628568	0.8901	0.99995	no

ENSG00000015133	ENSG00000015133	CCDC88C	14:91737666-91884188	q1	q2	OK	11.2795	12.0435	0.0945569	0.0926115	0.80015	0.99995	no

ENSG00000015153	ENSG00000015153	YAF2	12:42550905-42632151	q1	q2	OK	18.5125	19.59	0.0816188	0.106789	0.8157	0.99995	no

ENSG00000015171	ENSG00000015171	ZMYND11	10:180404-300577	q1	q2	OK	36.263	39.9631	0.14017	0.295273	0.6228	0.99995	no

ENSG00000015285	ENSG00000015285	WAS	X:48534984-48549818	q1	q2	NOTEST	0.195596	0.0329088	-2.57133	0	1	1	no

ENSG00000015413	ENSG00000015413	DPEP1	16:89679715-89704839	q1	q2	NOTEST	0	0.231766	inf	0	1	1	no

ENSG00000015475	ENSG00000015475	BID	22:18216905-18257536	q1	q2	OK	8.42641	8.30545	-0.02086	-0.0359923	0.9575	0.99995	no

ENSG00000015479	ENSG00000015479	MATR3	5:138282408-138667360	q1	q2	OK	376.466	414.122	0.137536	0.239326	0.70605	0.99995	no

ENSG00000015520	ENSG00000015520	NPC1L1	7:44552133-44580914	q1	q2	NOTEST	0.0264569	0.0349519	0.401726	0	1	1	no

ENSG00000015532	ENSG00000015532	XYLT2	17:48423452-48440499	q1	q2	OK	9.38855	9.91405	0.0785737	0.138544	0.8304	0.99995	no

ENSG00000015568	ENSG00000015568	RGPD5	2:110550334-110615272	q1	q2	OK	0.626964	1.01634	0.696927	0.517253	0.2335	0.99995	no

ENSG00000015592	ENSG00000015592	STMN4	8:27092839-27115937	q1	q2	OK	11.7059	10.7741	-0.119659	-0.199558	0.78155	0.99995	no

ENSG00000015676	ENSG00000015676	NUDCD3	7:44418719-44530479	q1	q2	OK	38.4746	37.6395	-0.0316611	-0.0440461	0.9467	0.99995	no

ENSG00000016082	ENSG00000016082	ISL1	5:50668569-50690564	q1	q2	NOTEST	0.0293741	0.0179983	-0.706684	0	1	1	no

ENSG00000016391	ENSG00000016391	CHDH	3:53528682-53880417	q1	q2	OK	1.18924	1.20755	0.0220474	0.0253068	0.96895	0.99995	no

ENSG00000016402	ENSG00000016402	IL20RA	6:137321107-137366298	q1	q2	NOTEST	0.0390547	0.0759959	0.960426	0	1	1	no

ENSG00000016490	ENSG00000016490	CLCA1	1:86934050-86965972	q1	q2	NOTEST	0	0	0	0	1	1	no

ENSG00000016602	ENSG00000016602	CLCA4	1:87012760-87158886	q1	q2	NOTEST	0	0	0	0	1	1	no

ENSG00000016864	ENSG00000016864	GLT8D1	3:52529353-52742182	q1	q2	OK	67.0826	57.8291	-0.214143	-0.142511	0.80245	0.99995	no

ENSG00000017260	ENSG00000017260	ATP2C1	3:130569438-131100319	q1	q2	OK	38.1419	41.5382	0.12306	0.207916	0.7063	0.99995	no

ENSG00000017373	ENSG00000017373	SRCIN1	17:36686250-36762183	q1	q2	OK	1.58646	1.57395	-0.0114154	-0.0113679	0.986	0.99995	no

ENSG00000017427	ENSG00000017427	IGF1	12:102789644-102874423	q1	q2	NOTEST	0.0134259	0.00320212	-2.06792	0	1	1	no

ENSG00000017483	ENSG00000017483	SLC38A5	X:48316919-48328644	q1	q2	NOTEST	0.0418033	0.477329	3.51329	0	1	1	no

ENSG00000017621	ENSG00000017621	MAGIX	X:49019060-49024822	q1	q2	NOTEST	0	0	0	0	1	1	no

ENSG00000017797	ENSG00000017797	RALBP1	18:9475006-9538114	q1	q2	OK	18.5231	17.975	-0.0433374	-0.103009	0.8851	0.99995	no

ENSG00000018189	ENSG00000018189	RUFY3	4:71569463-71673032	q1	q2	OK	77.1274	73.2313	-0.0747843	-0.1198	0.8215	0.99995	no

ENSG00000018236	ENSG00000018236	CNTN1	12:41086243-41466220	q1	q2	OK	1.32126	1.30183	-0.0213749	-0.0250056	0.96195	0.99995	no

ENSG00000018280	ENSG00000018280	SLC11A1	2:219246751-219261617	q1	q2	NOTEST	0.0263411	0.0256273	-0.0396345	0	1	1	no

ENSG00000018408	ENSG00000018408	WWTR1	3:149235021-149454501	q1	q2	OK	28.9158	28.0234	-0.0452294	-0.0923942	0.8861	0.99995	no

ENSG00000018510	ENSG00000018510	AGPS	2:178092322-178408564	q1	q2	OK	10.8353	12.6019	0.217908	0.275961	0.60385	0.99995	no

ENSG00000018607	ENSG00000018607	ZNF806	2:133066881-133076309	q1	q2	NOTEST	0.204641	0.38522	0.912588	0	1	1	no

ENSG00000018610	ENSG00000018610	CXorf56	X:118672111-118699397	q1	q2	OK	12.2642	12.5277	0.0306779	0.0478972	0.9421	0.99995	no

ENSG00000018625	ENSG00000018625	ATP1A2	1:160085548-160113381	q1	q2	OK	30.7718	33.6742	0.130035	0.17585	0.7053	0.99995	no

ENSG00000018699	ENSG00000018699	TTC27	2:32853098-33046118	q1	q2	OK	7.76519	7.85686	0.0169309	0.0304298	0.96345	0.99995	no

ENSG00000018869	ENSG00000018869	ZNF582	19:56732680-56904914	q1	q2	OK	2.14527	2.40492	0.164828	0.0825998	0.8636	0.99995	no

ENSG00000019102	ENSG00000019102	VSIG2	11:124617367-124635832	q1	q2	NOTEST	0.401759	0.319804	-0.329146	0	1	1	no

ENSG00000019144	ENSG00000019144	PHLDB1	11:118477154-118550399	q1	q2	OK	2.08858	2.74466	0.394106	0.26225	0.6736	0.99995	no

ENSG00000019169	ENSG00000019169	MARCO	2:119699741-119752236	q1	q2	NOTEST	0.258866	0.137696	-0.910719	0	1	1	no

ENSG00000019186	ENSG00000019186	CYP24A1	20:52769987-52790512	q1	q2	OK	1.02101	1.35408	0.407314	0.265224	0.59655	0.99995	no

ENSG00000019485	ENSG00000019485	PRDM11	11:45115563-45256843	q1	q2	OK	1.99111	2.62946	0.401192	0.37618	0.61045	0.99995	no

ENSG00000019505	ENSG00000019505	SYT13	11:45261851-45307870	q1	q2	OK	2.06501	1.74498	-0.242941	-0.366833	0.5818	0.99995	no

ENSG00000019549	ENSG00000019549	SNAI2	8:49830248-49834299	q1	q2	OK	15.5573	15.3613	-0.0182929	-0.0344319	0.96305	0.99995	no

ENSG00000019582	ENSG00000019582	CD74	5:149781199-149792492	q1	q2	OK	0.638686	0.34928	-0.870725	-0.512278	0.1708	0.99995	no

ENSG00000019991	ENSG00000019991	HGF	7:81328321-81399754	q1	q2	NOTEST	0.34925	0.245188	-0.510371	0	1	1	no

ENSG00000019995	ENSG00000019995	ZRANB1	10:126605711-126849739	q1	q2	OK	19.6711	20.1601	0.0354264	0.0414949	0.9457	0.99995	no

ENSG00000020129	ENSG00000020129	NCDN	1:35899090-36032875	q1	q2	OK	12.7899	12.0776	-0.082673	-0.0988653	0.867	0.99995	no

ENSG00000020181	ENSG00000020181	GPR124	8:37641708-37707422	q1	q2	OK	12.7806	12.0428	-0.0857829	-0.132441	0.87185	0.99995	no

ENSG00000020219	ENSG00000020219	CCT8L1P	7:152142561-152144235	q1	q2	NOTEST	0.0177373	0.0512698	1.53133	0	1	1	no

ENSG00000020256	ENSG00000020256	ZFP64	20:50668201-50820847	q1	q2	OK	14.6732	13.9295	-0.0750448	-0.151884	0.81505	0.99995	no

ENSG00000020426	ENSG00000020426	MNAT1	14:61201459-61550451	q1	q2	OK	28.7818	32.854	0.190913	0.225454	0.74415	0.99995	no

ENSG00000020577	ENSG00000020577	SAMD4A	14:55033814-55260033	q1	q2	OK	7.01281	7.10158	0.018147	0.0278654	0.9566	0.99995	no

ENSG00000020633	ENSG00000020633	RUNX3	1:25226001-25291612	q1	q2	NOTEST	0.0133123	0.0192399	0.53134	0	1	1	no

ENSG00000020922	ENSG00000020922	MRE11A	11:94152894-94232749	q1	q2	OK	11.4479	12.9694	0.180032	0.280383	0.65295	0.99995	no

ENSG00000021300	ENSG00000021300	PLEKHB1	11:73357222-73373864	q1	q2	OK	12.7189	11.0407	-0.204138	-0.359087	0.55335	0.99995	no

ENSG00000021355	ENSG00000021355	SERPINB1	6:2832565-2842240	q1	q2	OK	1.17282	1.42214	0.278087	0.259098	0.6458	0.99995	no

ENSG00000021461	ENSG00000021461	CYP3A43	7:99425635-99463718	q1	q2	NOTEST	0	0.0479167	inf	0	1	1	no

ENSG00000021488	ENSG00000021488	SLC7A9	19:33321414-33360672	q1	q2	OK	0.411562	0.486992	0.242787	0.197853	0.774	0.99995	no

ENSG00000021574	ENSG00000021574	SPAST	2:32288679-32382706	q1	q2	OK	13.8693	14.8513	0.0986984	0.23293	0.73365	0.99995	no

ENSG00000021645	ENSG00000021645	NRXN3	14:78708733-80330762	q1	q2	OK	1.52236	2.37368	0.640816	0.705266	0.15395	0.99995	no

ENSG00000021762	ENSG00000021762	OSBPL5	11:3108345-3187969	q1	q2	OK	7.65042	6.04586	-0.339591	-0.464176	0.40025	0.99995	no

ENSG00000021776	ENSG00000021776	AQR	15:35147731-35262040	q1	q2	OK	17.2782	18.5508	0.102525	0.173813	0.76395	0.99995	no

ENSG00000021826	ENSG00000021826	CPS1	2:211342405-211543831	q1	q2	OK	1.18726	1.32595	0.159396	0.148502	0.75085	0.99995	no

ENSG00000021852	ENSG00000021852	C8B	1:57394882-59012474	q1	q2	NOTEST	0	0	0	0	1	1	no

ENSG00000022267	ENSG00000022267	FHL1	X:135229558-135293518	q1	q2	OK	23.8109	23.0071	-0.0495428	-0.110402	0.86915	0.99995	no

ENSG00000022277	ENSG00000022277	RTFDC1	20:55043646-55101198	q1	q2	OK	63.0957	62.1235	-0.0224029	-0.0504398	0.9367	0.99995	no

ENSG00000022355	ENSG00000022355	GABRA1	5:161274196-161326975	q1	q2	NOTEST	0.0388365	0.053987	0.475198	0	1	1	no

ENSG00000022556	ENSG00000022556	NLRP2	19:55434876-55512510	q1	q2	NOTEST	0	0	0	0	1	1	no

ENSG00000022567	ENSG00000022567	SLC45A4	8:142217264-142318404	q1	q2	OK	7.40906	8.43069	0.186361	0.203583	0.7749	0.99995	no

ENSG00000022840	ENSG00000022840	RNF10	12:120941076-121015397	q1	q2	OK	66.3595	67.2197	0.0185816	0.0248425	0.95575	0.99995	no

ENSG00000022976	ENSG00000022976	ZNF839	14:102783713-102829253	q1	q2	OK	9.96327	9.54379	-0.0620582	-0.0788024	0.89595	0.99995	no

ENSG00000023041	ENSG00000023041	ZDHHC6	10:114190057-114206672	q1	q2	OK	28.6644	31.8072	0.150092	0.258729	0.68685	0.99995	no

ENSG00000023171	ENSG00000023171	GRAMD1B	11:123396343-123498482	q1	q2	OK	1.82639	1.97037	0.109468	0.138519	0.8032	0.99995	no

ENSG00000023191	ENSG00000023191	RNH1	11:494511-507300	q1	q2	OK	33.7145	31.0053	-0.120854	-0.257812	0.70755	0.99995	no

ENSG00000023228	ENSG00000023228	NDUFS1	2:206979540-207027652	q1	q2	OK	37.519	43.3346	0.207897	0.236282	0.70915	0.99995	no

ENSG00000023287	ENSG00000023287	RB1CC1	8:53535015-53658403	q1	q2	OK	19.8405	19.0447	-0.0590588	-0.103417	0.8357	0.99995	no

ENSG00000023318	ENSG00000023318	ERP44	9:102741460-102861322	q1	q2	OK	11.0529	11.1341	0.0105662	0.0240921	0.97395	0.99995	no

ENSG00000023330	ENSG00000023330	ALAS1	3:52232101-52248343	q1	q2	OK	23.3908	23.1287	-0.0162579	-0.0339695	0.9578	0.99995	no

ENSG00000023445	ENSG00000023445	BIRC3	11:102188214-102210134	q1	q2	NOTEST	0.119489	0.176954	0.566494	0	1	1	no

ENSG00000023516	ENSG00000023516	AKAP11	13:42846288-42897396	q1	q2	OK	15.2265	17.3389	0.18743	0.461222	0.5111	0.99995	no

ENSG00000023572	ENSG00000023572	GLRX2	1:193065597-193075244	q1	q2	OK	10.1435	10.8152	0.092511	0.125399	0.8557	0.99995	no

ENSG00000023608	ENSG00000023608	SNAPC1	14:62147758-62263146	q1	q2	OK	5.09806	5.28745	0.0526249	0.0222382	0.64565	0.99995	no

ENSG00000023697	ENSG00000023697	DERA	12:16064105-16190220	q1	q2	OK	29.8037	29.0453	-0.0371863	-0.0722989	0.91665	0.99995	no

ENSG00000023734	ENSG00000023734	STRAP	12:16035324-16056412	q1	q2	OK	161.356	170.712	0.0813103	0.185882	0.7792	0.99995	no

ENSG00000023839	ENSG00000023839	ABCC2	10:101542488-101611949	q1	q2	NOTEST	0.261798	0.282051	0.107498	0	1	1	no

ENSG00000023892	ENSG00000023892	DEF6	6:35265594-35289548	q1	q2	OK	0.647918	0.423611	-0.61307	-0.394771	0.42025	0.99995	no

ENSG00000023902	ENSG00000023902	PLEKHO1	1:150121372-150136916	q1	q2	OK	0.954272	0.813547	-0.230175	-0.185411	0.73905	0.99995	no

ENSG00000023909	ENSG00000023909	GCLM	1:94350760-94374966	q1	q2	OK	7.07525	6.20131	-0.190208	-0.250599	0.64605	0.99995	no

ENSG00000024048	ENSG00000024048	UBR2	6:42531799-42661242	q1	q2	OK	8.3321	8.66339	0.0562513	0.131394	0.8525	0.99995	no

ENSG00000024422	ENSG00000024422	EHD2	19:48110780-48246391	q1	q2	OK	8.35891	6.94015	-0.268348	-0.245587	0.7623	0.99995	no

ENSG00000024526	ENSG00000024526	DEPDC1	1:68939834-69004310	q1	q2	OK	13.4515	14.8124	0.139041	0.246035	0.65365	0.99995	no

ENSG00000024862	ENSG00000024862	CCDC28A	6:139094656-139114456	q1	q2	OK	4.18419	4.26171	0.0264834	0.0365957	0.95795	0.99995	no

ENSG00000025039	ENSG00000025039	RRAGD	6:90074354-90121989	q1	q2	OK	8.0217	7.43831	-0.108934	-0.136128	0.8338	0.99995	no

ENSG00000025156	ENSG00000025156	HSF2	6:122720690-122754264	q1	q2	OK	22.1082	24.5821	0.153026	0.315603	0.62155	0.99995	no

ENSG00000025293	ENSG00000025293	PHF20	20:34359895-34538303	q1	q2	OK	14.9641	15.4967	0.0504583	0.0785292	0.8587	0.99995	no

ENSG00000025423	ENSG00000025423	HSD17B6	12:57106211-57181574	q1	q2	OK	0.71395	1.44617	1.01834	0.0834163	0.3647	0.99995	no

ENSG00000025434	ENSG00000025434	NR1H3	11:47260852-47290396	q1	q2	OK	5.88311	4.55842	-0.368046	-0.232706	0.5855	0.99995	no

ENSG00000025708	ENSG00000025708	TYMP	22:50961996-50971009	q1	q2	OK	0.896782	0.726785	-0.303228	-0.150665	0.81295	0.99995	no

ENSG00000025770	ENSG00000025770	NCAPH2	22:50946644-50961901	q1	q2	OK	34.7969	33.066	-0.0736129	-0.161339	0.8086	0.99995	no

ENSG00000025772	ENSG00000025772	TOMM34	20:43538702-43589127	q1	q2	OK	17.4511	18.301	0.0685989	0.109706	0.8699	0.99995	no

ENSG00000025796	ENSG00000025796	SEC63	6:108188959-108281190	q1	q2	OK	54.7438	57.8797	0.0803628	0.122747	0.82155	0.99995	no

ENSG00000025800	ENSG00000025800	KPNA6	1:32573638-32642169	q1	q2	OK	16.24	16.9199	0.059164	0.145715	0.8314	0.99995	no

ENSG00000026025	ENSG00000026025	VIM	10:17256237-17279592	q1	q2	OK	1715.76	1629.77	-0.0741779	-0.113092	0.87005	0.99995	no

ENSG00000026036	ENSG00000026036	RTEL1-TNFRSF6B	20:62289162-62374858	q1	q2	OK	4.11769	3.50906	-0.23075	-0.206046	0.7814	0.99995	no

ENSG00000026103	ENSG00000026103	FAS	10:90639490-90775542	q1	q2	OK	3.53112	3.40161	-0.0539088	-0.0256678	0.962	0.99995	no

ENSG00000026297	ENSG00000026297	RNASET2	6:166822851-167370679	q1	q2	OK	6.08389	5.13831	-0.2437	-0.116844	0.8085	0.99995	no

ENSG00000026508	ENSG00000026508	CD44	11:35160416-35253949	q1	q2	OK	7.23853	7.57662	0.0658576	0.0714672	0.893	0.99995	no

ENSG00000026559	ENSG00000026559	KCNG1	20:49615906-49639666	q1	q2	OK	3.80029	2.51291	-0.596749	-0.700021	0.27055	0.99995	no

ENSG00000026652	ENSG00000026652	AGPAT4	6:161551010-161695093	q1	q2	OK	20.613	19.2673	-0.0974047	-0.149061	0.7752	0.99995	no

ENSG00000026751	ENSG00000026751	SLAMF7	1:160709036-160724611	q1	q2	NOTEST	0.0109592	0.0220187	1.00658	0	1	1	no

ENSG00000026950	ENSG00000026950	BTN3A1	6:26402464-26415444	q1	q2	OK	6.44193	6.14776	-0.0674324	-0.0889158	0.87985	0.99995	no

ENSG00000027001	ENSG00000027001	MIPEP	13:24304327-24476794	q1	q2	OK	5.50797	5.83951	0.0843253	0.124352	0.8348	0.99995	no

ENSG00000027075	ENSG00000027075	PRKCH	14:61654276-62125414	q1	q2	OK	0.652194	0.517701	-0.33318	-0.131092	0.5019	0.99995	no

ENSG00000027644	ENSG00000027644	INSRR	1:156776034-156851642	q1	q2	NOTEST	0.0198883	0.0237725	0.257375	0	1	1	no

ENSG00000027697	ENSG00000027697	IFNGR1	6:137518620-137540586	q1	q2	OK	22.7857	23.2495	0.0290668	0.062091	0.92675	0.99995	no

ENSG00000027847	ENSG00000027847	B4GALT7	5:177027100-177037348	q1	q2	OK	12.5661	10.8836	-0.20739	-0.322385	0.60085	0.99995	no

ENSG00000027869	ENSG00000027869	SH2D2A	1:156776034-156851642	q1	q2	NOTEST	0.0502959	0.0681367	0.437991	0	1	1	no

ENSG00000028116	ENSG00000028116	VRK2	2:58134785-58468821	q1	q2	OK	2.10518	2.2622	0.10378	0.0534008	0.95035	0.99995	no

ENSG00000028137	ENSG00000028137	TNFRSF1B	1:12227059-12269285	q1	q2	OK	0.665373	0.787574	0.243253	0.218336	0.6883	0.99995	no

ENSG00000028203	ENSG00000028203	VEZT	12:95611521-95696566	q1	q2	OK	42.3883	41.3941	-0.0342422	-0.0647117	0.903	0.99995	no

ENSG00000028277	ENSG00000028277	POU2F2	19:42590262-42700737	q1	q2	OK	5.89991	4.93056	-0.258942	-0.265554	0.5117	0.99995	no

ENSG00000028310	ENSG00000028310	BRD9	5:710470-919472	q1	q2	OK	36.1787	37.9381	0.0685065	0.115142	0.8574	0.99995	no

ENSG00000028528	ENSG00000028528	SNX1	15:64386321-64438289	q1	q2	OK	59.8832	55.1651	-0.118397	-0.201221	0.71445	0.99995	no

ENSG00000028839	ENSG00000028839	TBPL1	6:134273307-134373774	q1	q2	OK	43.8931	51.7546	0.237693	0.385355	0.57685	0.99995	no

ENSG00000029153	ENSG00000029153	ARNTL2	12:27485786-27599558	q1	q2	OK	3.57046	3.668	0.038883	0.0583408	0.9344	0.99995	no

ENSG00000029363	ENSG00000029363	BCLAF1	6:136578000-136610989	q1	q2	OK	110.703	118.587	0.0992451	0.127016	0.7936	0.99995	no

ENSG00000029364	ENSG00000029364	SLC39A9	14:69846847-69929105	q1	q2	OK	24.8958	25.1278	0.0133786	0.0157364	0.97795	0.99995	no

ENSG00000029534	ENSG00000029534	ANK1	8:41510738-41754280	q1	q2	OK	0.78975	0.777186	-0.0231359	-0.0175478	0.9681	0.99995	no

ENSG00000029559	ENSG00000029559	IBSP	4:88720732-88733074	q1	q2	NOTEST	0	0.0278021	inf	0	1	1	no

ENSG00000029639	ENSG00000029639	TFB1M	6:155054458-155635627	q1	q2	OK	8.7099	7.22209	-0.270239	-0.100922	0.82465	0.99995	no

ENSG00000029725	ENSG00000029725	RABEP1	17:5185557-5372380	q1	q2	OK	36.9652	39.2335	0.0859203	0.0739033	0.86155	0.99995	no

ENSG00000029993	ENSG00000029993	HMGB3	X:150148981-150159248	q1	q2	NOTEST	0	0.0714315	inf	0	1	1	no

ENSG00000030066	ENSG00000030066	NUP160	11:47799638-47870107	q1	q2	OK	34.084	36.9664	0.117118	0.178336	0.6703	0.99995	no

ENSG00000030110	ENSG00000030110	BAK1	6:33540328-33548019	q1	q2	OK	11.6227	10.9715	-0.0831778	-0.158438	0.81925	0.99995	no

ENSG00000030304	ENSG00000030304	MUSK	9:113431050-113563859	q1	q2	NOTEST	0.0855697	0.0945692	0.14427	0	1	1	no

ENSG00000030419	ENSG00000030419	IKZF2	2:213446080-214017151	q1	q2	OK	2.47136	2.33432	-0.0823057	-0.105083	0.81775	0.99995	no

ENSG00000030582	ENSG00000030582	GRN	17:42422613-42430470	q1	q2	OK	42.8963	37.0438	-0.211619	-0.42317	0.4519	0.99995	no

ENSG00000031003	ENSG00000031003	FAM13B	5:137150021-137387650	q1	q2	OK	20.0316	21.757	0.1192	0.180507	0.6839	0.99995	no

ENSG00000031081	ENSG00000031081	ARHGAP31	3:119013219-119139561	q1	q2	OK	3.06106	2.89134	-0.0822942	-0.136853	0.81915	0.99995	no

ENSG00000031544	ENSG00000031544	NR2E3	15:72084976-72110600	q1	q2	NOTEST	0.080499	0.0793602	-0.0205555	0	1	1	no

ENSG00000031691	ENSG00000031691	CENPQ	6:49431090-49460820	q1	q2	OK	11.874	12.1629	0.0346763	0.0638364	0.9321	0.99995	no

ENSG00000031698	ENSG00000031698	SARS	1:109756539-109780791	q1	q2	OK	129.528	126.127	-0.0383863	-0.0915069	0.89265	0.99995	no

ENSG00000031823	ENSG00000031823	RANBP3	19:5916149-5978153	q1	q2	OK	44.5117	40.9642	-0.119821	-0.189509	0.7501	0.99995	no

ENSG00000032219	ENSG00000032219	ARID4A	14:58765102-58862359	q1	q2	OK	20.1256	20.2868	0.0115075	0.0166314	0.97145	0.99995	no

ENSG00000032389	ENSG00000032389	TSSC1	2:3192695-3381653	q1	q2	OK	18.5167	17.0603	-0.118184	-0.228081	0.72125	0.99995	no

ENSG00000032444	ENSG00000032444	PNPLA6	19:7587511-7626650	q1	q2	OK	19.2885	14.2762	-0.434135	-0.491699	0.38905	0.99995	no

ENSG00000032742	ENSG00000032742	IFT88	13:21140584-21265503	q1	q2	OK	11.4304	13.3888	0.228151	0.31281	0.59155	0.99995	no

ENSG00000033011	ENSG00000033011	ALG1	16:4932507-5147809	q1	q2	OK	8.84133	8.50918	-0.055242	-0.0743328	0.91605	0.99995	no

ENSG00000033030	ENSG00000033030	ZCCHC8	12:122957416-122985518	q1	q2	OK	26.8633	27.5028	0.033944	0.0709241	0.9184	0.99995	no

ENSG00000033050	ENSG00000033050	ABCF2	7:150904922-150924316	q1	q2	OK	28.7773	28.0668	-0.03607	-0.0358017	0.92635	0.99995	no

ENSG00000033100	ENSG00000033100	CHPF2	7:150929574-150974982	q1	q2	OK	14.1589	12.0454	-0.233227	-0.338175	0.5784	0.99995	no

ENSG00000033122	ENSG00000033122	LRRC7	1:70034080-70671303	q1	q2	OK	4.9067	4.39497	-0.1589	-0.144457	0.82195	0.99995	no

ENSG00000033170	ENSG00000033170	FUT8	14:65877309-66210839	q1	q2	OK	41.0054	42.7803	0.0611352	0.134208	0.8328	0.99995	no

ENSG00000033178	ENSG00000033178	UBA6	4:68478369-68566897	q1	q2	OK	18.634	20.8806	0.16423	0.188584	0.6815	0.99995	no

ENSG00000033327	ENSG00000033327	GAB2	11:77926342-78285919	q1	q2	OK	7.62251	7.34547	-0.0534115	-0.0763715	0.8875	0.99995	no

ENSG00000033627	ENSG00000033627	ATP6V0A1	17:40610861-40674629	q1	q2	OK	27.4405	26.9294	-0.0271243	-0.046618	0.92695	0.99995	no

ENSG00000033800	ENSG00000033800	PIAS1	15:68346516-68549549	q1	q2	OK	20.3308	20.2352	-0.00679967	-0.00915726	0.98985	0.99995	no

ENSG00000033867	ENSG00000033867	SLC4A7	3:27414213-27525911	q1	q2	OK	19.7323	22.4391	0.185454	0.315981	0.5287	0.99995	no

ENSG00000034053	ENSG00000034053	APBA2	15:29129628-29410518	q1	q2	OK	30.453	28.9945	-0.0708051	-0.163609	0.81715	0.99995	no

ENSG00000034063	ENSG00000034063	UHRF1	19:4903091-4962165	q1	q2	OK	44.0236	46.7888	0.0878874	0.205197	0.7745	0.99995	no

ENSG00000034152	ENSG00000034152	MAP2K3	17:21187983-21218552	q1	q2	OK	7.08608	6.21334	-0.189618	-0.283921	0.6313	0.99995	no

ENSG00000034239	ENSG00000034239	EFCAB1	8:49623347-49647870	q1	q2	OK	0.558904	0.720992	0.367383	0.26946	0.6091	0.99995	no

ENSG00000034510	ENSG00000034510	TMSB10	2:85048773-85134132	q1	q2	OK	2046.19	1958.67	-0.0630663	-0.140609	0.84025	0.99995	no

ENSG00000034533	ENSG00000034533	ASTE1	3:130569438-131100319	q1	q2	OK	0.942895	0.937919	-0.00763439	-0.00226808	0.9953	0.99995	no

ENSG00000034677	ENSG00000034677	RNF19A	8:101170133-101348446	q1	q2	OK	12.7398	12.0683	-0.0781145	-0.104816	0.80825	0.99995	no

ENSG00000034693	ENSG00000034693	PEX3	6:143771943-143832827	q1	q2	OK	8.36569	8.06941	-0.0520216	-0.045376	0.9357	0.99995	no

ENSG00000034713	ENSG00000034713	GABARAPL2	16:75562429-75657198	q1	q2	OK	90.0423	90.644	0.00960865	0.0172967	0.97995	0.99995	no

ENSG00000034971	ENSG00000034971	MYOC	1:171604556-171621823	q1	q2	NOTEST	0	0	0	0	1	1	no

ENSG00000035115	ENSG00000035115	SH3YL1	2:217729-278283	q1	q2	OK	23.4717	25.6549	0.128315	0.115686	0.83555	0.99995	no

ENSG00000035141	ENSG00000035141	FAM136A	2:70523106-70529222	q1	q2	OK	41.8586	45.6868	0.126256	0.293345	0.66015	0.99995	no

ENSG00000035403	ENSG00000035403	VCL	10:75757871-75879918	q1	q2	OK	40.4014	47.8548	0.244258	0.501919	0.3913	0.99995	no

ENSG00000035499	ENSG00000035499	DEPDC1B	5:59892738-59996017	q1	q2	OK	30.004	33.4496	0.156834	0.350908	0.60755	0.99995	no

ENSG00000035664	ENSG00000035664	DAPK2	15:64199234-64364232	q1	q2	OK	1.74371	1.32678	-0.39423	-0.300606	0.4865	0.99995	no

ENSG00000035681	ENSG00000035681	NSMAF	8:59496062-59572403	q1	q2	OK	14.9186	16.8116	0.172346	0.241292	0.6757	0.99995	no

ENSG00000035687	ENSG00000035687	ADSS	1:244515936-244615436	q1	q2	OK	23.3475	23.3747	0.00167971	0.00359982	0.9961	0.99995	no

ENSG00000035720	ENSG00000035720	STAP1	4:68424445-68473055	q1	q2	NOTEST	0	0	0	0	1	1	no

ENSG00000035862	ENSG00000035862	TIMP2	17:76849058-76921469	q1	q2	OK	89.2403	84.5191	-0.0784182	-0.185998	0.78665	0.99995	no

ENSG00000035928	ENSG00000035928	RFC1	4:39289075-39367995	q1	q2	OK	30.6015	33.8308	0.144735	0.243262	0.6346	0.99995	no

ENSG00000036054	ENSG00000036054	TBC1D23	3:99979843-100044095	q1	q2	OK	13.9761	14.0882	0.0115265	0.0243233	0.97095	0.99995	no

ENSG00000036257	ENSG00000036257	CUL3	2:225334866-225450110	q1	q2	OK	62.2978	64.2733	0.0450385	0.0755013	0.9026	0.99995	no

ENSG00000036448	ENSG00000036448	MYOM2	8:1993154-2113475	q1	q2	NOTEST	0	0	0	0	1	1	no

ENSG00000036473	ENSG00000036473	OTC	X:37208527-38548169	q1	q2	NOTEST	0	0	0	0	1	1	no

ENSG00000036530	ENSG00000036530	CYP46A1	14:100150640-100193638	q1	q2	OK	4.08014	3.78465	-0.108456	-0.116485	0.8364	0.99995	no

ENSG00000036549	ENSG00000036549	ZZZ3	1:78028100-78149104	q1	q2	OK	22.0688	24.8903	0.17358	0.265562	0.57655	0.99995	no

ENSG00000036565	ENSG00000036565	SLC18A1	8:20002365-20040717	q1	q2	NOTEST	0.357324	0.359488	0.00870971	0	1	1	no

ENSG00000036672	ENSG00000036672	USP2	11:119225924-119252436	q1	q2	OK	0.5065	0.820983	0.69679	0.50922	0.2852	0.99995	no

ENSG00000036828	ENSG00000036828	CASR	3:121902529-122005342	q1	q2	NOTEST	0	0.0078073	inf	0	1	1	no

ENSG00000037042	ENSG00000037042	TUBG2	17:40811322-40819024	q1	q2	OK	12.6047	13.3418	0.0819877	0.129979	0.8485	0.99995	no

ENSG00000037241	ENSG00000037241	RPL26L1	5:172381788-172396774	q1	q2	OK	47.6267	45.9841	-0.0506356	-0.0964521	0.8943	0.99995	no

ENSG00000037280	ENSG00000037280	FLT4	5:180028505-180076624	q1	q2	OK	6.89088	6.50073	-0.0840846	-0.0879607	0.8843	0.99995	no

ENSG00000037474	ENSG00000037474	NSUN2	5:6599351-6633404	q1	q2	OK	44.6503	46.0762	0.0453527	0.082649	0.89085	0.99995	no

ENSG00000037637	ENSG00000037637	FBXO42	1:16573333-16678949	q1	q2	OK	13.2582	14.0459	0.0832609	0.127839	0.84835	0.99995	no

ENSG00000037749	ENSG00000037749	MFAP3	5:153369687-153825410	q1	q2	OK	19.7375	21.2907	0.109285	0.0914408	0.81565	0.99995	no

ENSG00000037757	ENSG00000037757	MRI1	19:13875345-13889276	q1	q2	OK	8.45955	6.89443	-0.29515	-0.280722	0.639	0.99995	no

ENSG00000037897	ENSG00000037897	METTL1	12:58156116-58212487	q1	q2	OK	9.15773	11.699	0.353319	0.315184	0.6493	0.99995	no

ENSG00000037965	ENSG00000037965	HOXC8	12:54375292-54449814	q1	q2	NOTEST	0	0	0	0	1	1	no

ENSG00000038002	ENSG00000038002	AGA	4:178351923-178363657	q1	q2	OK	6.19084	6.49261	0.0686643	0.101402	0.8654	0.99995	no

ENSG00000038210	ENSG00000038210	PI4K2B	4:25162262-25280714	q1	q2	OK	8.97852	9.52352	0.0850167	0.0878503	0.8531	0.99995	no

ENSG00000038219	ENSG00000038219	BOD1L1	4:13570361-13629347	q1	q2	OK	19.8371	22.7693	0.198887	0.286979	0.5574	0.99995	no

ENSG00000038274	ENSG00000038274	MAT2B	5:162930119-162946342	q1	q2	OK	59.3061	62.1769	0.0681983	0.160937	0.8132	0.99995	no

ENSG00000038295	ENSG00000038295	TLL1	4:166794409-167025047	q1	q2	OK	0.483838	0.483704	-0.000402308	-0.000364567	0.99955	0.99995	no

ENSG00000038358	ENSG00000038358	EDC4	16:67906925-67922758	q1	q2	OK	29.978	28.2269	-0.0868334	-0.132338	0.81285	0.99995	no

ENSG00000038382	ENSG00000038382	TRIO	5:14143810-14532235	q1	q2	OK	67.5333	62.6998	-0.107137	-0.133869	0.7408	0.99995	no

ENSG00000038427	ENSG00000038427	VCAN	5:82767283-82878122	q1	q2	OK	97.9507	114.833	0.229415	0.256918	0.59105	0.99995	no

ENSG00000038532	ENSG00000038532	CLEC16A	16:11038344-11276046	q1	q2	OK	10.3787	9.14928	-0.181897	-0.36401	0.5752	0.99995	no

ENSG00000038945	ENSG00000038945	MSR1	8:15965386-16424999	q1	q2	NOTEST	0.0164539	0.00939651	-0.808231	0	1	1	no

ENSG00000039068	ENSG00000039068	CDH1	16:68771127-68869451	q1	q2	NOTEST	0.0306397	0.0457595	0.578667	0	1	1	no

ENSG00000039123	ENSG00000039123	SKIV2L2	5:54603587-54830878	q1	q2	OK	44.8376	50.3457	0.16716	0.266176	0.6171	0.99995	no

ENSG00000039139	ENSG00000039139	DNAH5	5:13690439-13944652	q1	q2	NOTEST	0.0817093	0.0803985	-0.0233318	0	1	1	no

ENSG00000039319	ENSG00000039319	ZFYVE16	5:79703831-79775169	q1	q2	OK	24.9445	29.179	0.226207	0.29871	0.475	0.99995	no

ENSG00000039523	ENSG00000039523	FAM65A	16:67552320-67580691	q1	q2	OK	21.5446	19.2332	-0.16373	-0.247836	0.57985	0.99995	no

ENSG00000039537	ENSG00000039537	C6	5:41142335-41261540	q1	q2	NOTEST	0	0	0	0	1	1	no

ENSG00000039560	ENSG00000039560	RAI14	5:34656341-34832732	q1	q2	OK	69.2405	93.0168	0.425875	0.262041	0.3306	0.99995	no

ENSG00000039600	ENSG00000039600	SOX30	5:157052686-157098488	q1	q2	NOTEST	0.0648123	0.068596	0.0818567	0	1	1	no

ENSG00000039650	ENSG00000039650	PNKP	19:50364460-50371166	q1	q2	OK	23.7511	22.1224	-0.102492	-0.150765	0.78735	0.99995	no

ENSG00000039987	ENSG00000039987	BEST2	19:12862515-12869272	q1	q2	NOTEST	0.099116	0.191866	0.952905	0	1	1	no

ENSG00000040199	ENSG00000040199	PHLPP2	16:71657610-71758604	q1	q2	OK	5.53613	5.76523	0.0585006	0.110415	0.8534	0.99995	no

ENSG00000040275	ENSG00000040275	SPDL1	5:169010637-169031782	q1	q2	OK	30.3575	34.1422	0.169505	0.332366	0.54845	0.99995	no

ENSG00000040341	ENSG00000040341	STAU2	8:74332238-74791145	q1	q2	OK	39.1167	42.7618	0.128536	0.209244	0.70445	0.99995	no

ENSG00000040487	ENSG00000040487	PQLC2	1:19638819-19655794	q1	q2	OK	11.8541	11.9615	0.0130128	0.0195202	0.97635	0.99995	no

ENSG00000040531	ENSG00000040531	CTNS	17:3539761-3564836	q1	q2	OK	7.26106	6.67525	-0.121357	-0.153798	0.75495	0.99995	no

ENSG00000040608	ENSG00000040608	RTN4R	22:20228937-20270769	q1	q2	OK	13.3016	11.4103	-0.221263	-0.419798	0.54585	0.99995	no

ENSG00000040633	ENSG00000040633	PHF23	17:7138346-7143041	q1	q2	OK	38.8762	37.244	-0.0618787	-0.131352	0.83725	0.99995	no

ENSG00000040731	ENSG00000040731	CDH10	5:24487208-24645087	q1	q2	OK	8.81271	8.1281	-0.116668	-0.204439	0.7682	0.99995	no

ENSG00000040933	ENSG00000040933	INPP4A	2:99061316-99210853	q1	q2	OK	10.0447	10.4748	0.0604895	0.113508	0.84765	0.99995	no

ENSG00000041353	ENSG00000041353	RAB27B	18:52385090-52565314	q1	q2	NOTEST	0.112925	0.109642	-0.0425626	0	1	1	no

ENSG00000041357	ENSG00000041357	PSMA4	15:78832746-78841604	q1	q2	OK	177.173	187.975	0.0853842	0.205483	0.7615	0.99995	no

ENSG00000041515	ENSG00000041515	MYO16	13:109248499-109860355	q1	q2	OK	2.43465	2.90994	0.257272	0.394463	0.50395	0.99995	no

ENSG00000041802	ENSG00000041802	LSG1	3:194304739-194393206	q1	q2	OK	25.39	26.4726	0.0602377	0.0681151	0.89605	0.99995	no

ENSG00000041880	ENSG00000041880	PARP3	3:51976360-51982883	q1	q2	OK	2.06731	2.0408	-0.0186234	-0.0191315	0.9728	0.99995	no

ENSG00000041982	ENSG00000041982	TNC	9:117782805-117880536	q1	q2	OK	136.652	144.913	0.0846872	0.103978	0.85665	0.99995	no

ENSG00000041988	ENSG00000041988	THAP3	1:6684925-6761984	q1	q2	OK	12.3973	12.7033	0.0351685	0.0273614	0.9596	0.99995	no

ENSG00000042062	ENSG00000042062	FAM65C	20:49202644-49308065	q1	q2	OK	0.684228	0.547949	-0.320436	-0.236925	0.6886	0.99995	no

ENSG00000042088	ENSG00000042088	TDP1	14:90421282-90511106	q1	q2	OK	21.8679	21.9147	0.00308823	0.00540091	0.99205	0.99995	no

ENSG00000042286	ENSG00000042286	AIFM2	10:71812551-71892690	q1	q2	OK	1.9659	1.8365	-0.098234	-0.0357557	0.95095	0.99995	no

ENSG00000042304	ENSG00000042304	C2orf83	2:228474805-228513322	q1	q2	NOTEST	0	0	0	0	1	1	no

ENSG00000042317	ENSG00000042317	SPATA7	14:88851267-89021077	q1	q2	OK	8.17367	6.46864	-0.337521	-0.249724	0.6211	0.99995	no

ENSG00000042429	ENSG00000042429	MED17	11:93394804-93547861	q1	q2	OK	42.0209	43.8544	0.0616147	0.0496177	0.92875	0.99995	no

ENSG00000042445	ENSG00000042445	RETSAT	2:85569210-85618875	q1	q2	OK	9.95806	9.04298	-0.139067	-0.184033	0.7455	0.99995	no

ENSG00000042493	ENSG00000042493	CAPG	2:85621870-85645555	q1	q2	OK	1.40442	1.39976	-0.0048012	-0.0033878	0.9942	0.99995	no

ENSG00000042753	ENSG00000042753	AP2S1	19:47341392-47354249	q1	q2	OK	133.06	122.501	-0.119279	-0.255939	0.70865	0.99995	no

ENSG00000042781	ENSG00000042781	USH2A	1:215796235-216596738	q1	q2	NOTEST	0.0925881	0.0660616	-0.487015	0	1	1	no

ENSG00000042813	ENSG00000042813	ZPBP	7:49813256-50199426	q1	q2	NOTEST	0	0	0	0	1	1	no

ENSG00000042832	ENSG00000042832	TG	8:133879202-134147147	q1	q2	NOTEST	0.164728	0.110747	-0.572824	0	1	1	no

ENSG00000042980	ENSG00000042980	ADAM28	8:24151552-24769586	q1	q2	NOTEST	0.0740095	0.00334834	-4.46619	0	1	1	no

ENSG00000043039	ENSG00000043039	BARX2	11:129245834-129322171	q1	q2	NOTEST	0.150619	0.329227	1.12818	0	1	1	no

ENSG00000043093	ENSG00000043093	DCUN1D1	3:182655861-182703741	q1	q2	OK	15.3965	17.7261	0.203276	0.242283	0.703	0.99995	no

ENSG00000043143	ENSG00000043143	JADE2	5:133860002-133918918	q1	q2	OK	0.682073	0.483659	-0.495935	-0.356366	0.45475	0.99995	no

ENSG00000043355	ENSG00000043355	ZIC2	13:100634025-100639018	q1	q2	OK	15.42	14.995	-0.0403215	-0.0533152	0.9222	0.99995	no

ENSG00000043462	ENSG00000043462	LCP2	5:169659450-169725231	q1	q2	NOTEST	0.110666	0.172367	0.639267	0	1	1	no

ENSG00000043514	ENSG00000043514	TRIT1	1:40306722-40349183	q1	q2	OK	23.2548	26.8248	0.206037	0.379427	0.5175	0.99995	no

ENSG00000043591	ENSG00000043591	ADRB1	10:115803805-115806667	q1	q2	NOTEST	0.0366231	0.0185392	-0.982177	0	1	1	no

ENSG00000044012	ENSG00000044012	GUCA2B	1:42619091-42621495	q1	q2	NOTEST	0	0	0	0	1	1	no

ENSG00000044090	ENSG00000044090	CUL7	6:43005354-43042837	q1	q2	OK	13.9474	13.1065	-0.0897128	-0.152711	0.809	0.99995	no

ENSG00000044115	ENSG00000044115	CTNNA1	5:137946655-138270723	q1	q2	OK	162.361	160.373	-0.0177714	-0.0289599	0.95245	0.99995	no

ENSG00000044446	ENSG00000044446	PHKA2	X:18908413-19002716	q1	q2	OK	7.02655	6.62258	-0.0854226	-0.0903761	0.86305	0.99995	no

ENSG00000044459	ENSG00000044459	CNTLN	9:17134979-17503921	q1	q2	OK	5.66272	6.12793	0.113906	0.214376	0.73335	0.99995	no

ENSG00000044524	ENSG00000044524	EPHA3	3:89156673-89531284	q1	q2	OK	18.5125	19.7332	0.0921291	0.22234	0.7494	0.99995	no

ENSG00000044574	ENSG00000044574	HSPA5	9:127997131-128003609	q1	q2	OK	234.183	210.796	-0.151792	-0.301243	0.6627	0.99995	no

ENSG00000046604	ENSG00000046604	DSG2	18:29078005-29136874	q1	q2	OK	3.39635	3.82496	0.171461	0.307367	0.6485	0.99995	no

ENSG00000046647	ENSG00000046647	GEMIN8	X:14026397-14048012	q1	q2	OK	9.49079	8.713	-0.123358	-0.182454	0.8005	0.99995	no

ENSG00000046651	ENSG00000046651	OFD1	X:13752831-13787480	q1	q2	OK	10.3238	10.6797	0.0489036	0.0700634	0.90475	0.99995	no

ENSG00000046653	ENSG00000046653	GPM6B	X:13789149-13956757	q1	q2	OK	97.1149	100.644	0.0514977	0.119328	0.8605	0.99995	no

ENSG00000046774	ENSG00000046774	MAGEC2	X:141290130-141293076	q1	q2	NOTEST	0	0	0	0	1	1	no

ENSG00000046889	ENSG00000046889	PREX2	8:68864352-69149265	q1	q2	OK	8.22596	9.51327	0.209758	0.271348	0.49535	0.99995	no

ENSG00000047056	ENSG00000047056	WDR37	10:1095477-1178237	q1	q2	OK	10.9568	10.3808	-0.0779207	-0.149257	0.80425	0.99995	no

ENSG00000047188	ENSG00000047188	YTHDC2	5:112849379-112930982	q1	q2	OK	18.5075	19.5038	0.0756413	0.114434	0.81845	0.99995	no

ENSG00000047230	ENSG00000047230	CTPS2	X:16606125-16731059	q1	q2	OK	17.8756	18.7695	0.0703966	0.103006	0.85165	0.99995	no

ENSG00000047249	ENSG00000047249	ATP6V1H	8:54628116-54756118	q1	q2	OK	22.8248	23.0918	0.0167745	0.0320787	0.95715	0.99995	no

ENSG00000047315	ENSG00000047315	POLR2B	4:57829535-58071676	q1	q2	OK	78.6311	88.8359	0.176043	0.23901	0.6037	0.99995	no

ENSG00000047346	ENSG00000047346	FAM214A	15:52870038-53002014	q1	q2	OK	19.4735	17.9433	-0.118073	-0.193886	0.74115	0.99995	no

ENSG00000047365	ENSG00000047365	ARAP2	4:35949842-36275842	q1	q2	OK	0.420461	0.706515	0.748747	0.340832	0.3022	0.99995	no

ENSG00000047410	ENSG00000047410	TPR	1:186265404-186344825	q1	q2	OK	45.5796	42.4519	-0.102559	-0.108061	0.7448	0.99995	no

ENSG00000047457	ENSG00000047457	CP	3:148847370-148939842	q1	q2	OK	4.3615	4.05017	-0.106841	-0.0636262	0.92105	0.99995	no

ENSG00000047578	ENSG00000047578	KIAA0556	16:27561453-27791690	q1	q2	OK	7.18471	6.94398	-0.0491685	-0.050925	0.9075	0.99995	no

ENSG00000047579	ENSG00000047579	DTNBP1	6:15523031-15663289	q1	q2	OK	13.1725	11.5103	-0.194605	-0.334561	0.6424	0.99995	no

ENSG00000047597	ENSG00000047597	XK	X:37208527-38548169	q1	q2	NOTEST	0	0	0	0	1	1	no

ENSG00000047617	ENSG00000047617	ANO2	12:5641034-6055398	q1	q2	OK	0.666852	0.750912	0.171277	0.115947	0.76835	0.99995	no

ENSG00000047621	ENSG00000047621	C12orf4	12:4596893-4647674	q1	q2	OK	8.60715	9.24676	0.103413	0.159846	0.7505	0.99995	no

ENSG00000047634	ENSG00000047634	SCML1	X:17755587-17773105	q1	q2	OK	17.6302	18.192	0.0452542	0.0938955	0.88755	0.99995	no

ENSG00000047644	ENSG00000047644	WWC3	X:9983601-10112518	q1	q2	OK	9.28	10.5419	0.183944	0.240549	0.57905	0.99995	no

ENSG00000047648	ENSG00000047648	ARHGAP6	X:11129420-11683821	q1	q2	OK	4.30984	3.99298	-0.110167	-0.120493	0.8421	0.99995	no

ENSG00000047662	ENSG00000047662	FAM184B	4:17616253-17783135	q1	q2	NOTEST	0.0490696	0.0327775	-0.582126	0	1	1	no

ENSG00000047849	ENSG00000047849	MAP4	3:47892181-48130769	q1	q2	OK	63.5521	63.2179	-0.00760735	-0.0104912	0.9774	0.99995	no

ENSG00000047932	ENSG00000047932	GOPC	6:117609462-117923691	q1	q2	OK	21.0479	23.527	0.160639	0.368697	0.6017	0.99995	no

ENSG00000047936	ENSG00000047936	ROS1	6:117609462-117923691	q1	q2	NOTEST	0.00328486	0	#NAME?	0	1	1	no

ENSG00000048028	ENSG00000048028	USP28	11:113668595-113746292	q1	q2	OK	20.2577	21.0064	0.0523635	0.0818044	0.86835	0.99995	no

ENSG00000048052	ENSG00000048052	HDAC9	7:18126571-19042039	q1	q2	OK	8.91015	7.27576	-0.292351	-0.354586	0.48925	0.99995	no

ENSG00000048140	ENSG00000048140	TSPAN17	5:176074387-176086058	q1	q2	OK	23.2109	21.8793	-0.0852407	-0.169608	0.7909	0.99995	no

ENSG00000048162	ENSG00000048162	NOP16	5:175792470-175843570	q1	q2	OK	16.3914	17.1404	0.0644675	0.0452894	0.93595	0.99995	no

ENSG00000048342	ENSG00000048342	CC2D2A	4:15471488-15603180	q1	q2	OK	13.2433	12.2561	-0.111753	-0.135888	0.79075	0.99995	no

ENSG00000048392	ENSG00000048392	RRM2B	8:103216729-103251346	q1	q2	OK	11.9293	12.7746	0.0987666	0.167162	0.7377	0.99995	no

ENSG00000048405	ENSG00000048405	ZNF800	7:126986843-127071978	q1	q2	OK	8.75603	9.4917	0.116389	0.195369	0.7626	0.99995	no

ENSG00000048462	ENSG00000048462	TNFRSF17	16:12021229-12070500	q1	q2	NOTEST	0.0807902	0.102304	0.340609	0	1	1	no

ENSG00000048471	ENSG00000048471	SNX29	16:12070593-12704901	q1	q2	OK	4.31966	4.17314	-0.0497847	-0.0713673	0.88115	0.99995	no

ENSG00000048540	ENSG00000048540	LMO3	12:16500075-16763528	q1	q2	OK	7.65149	7.28034	-0.0717343	-0.091832	0.8679	0.99995	no

ENSG00000048544	ENSG00000048544	MRPS10	6:42174538-42185603	q1	q2	OK	21.6728	22.9152	0.0804166	0.178689	0.803	0.99995	no

ENSG00000048545	ENSG00000048545	GUCA1A	6:42123143-42147794	q1	q2	NOTEST	0	0	0	0	1	1	no

ENSG00000048649	ENSG00000048649	RSF1	11:77371040-77532063	q1	q2	OK	43.5551	39.0013	-0.159319	-0.156813	0.66585	0.99995	no

ENSG00000048707	ENSG00000048707	VPS13D	1:12290123-12572099	q1	q2	OK	9.58193	8.95161	-0.0981687	-0.120436	0.8387	0.99995	no

ENSG00000048740	ENSG00000048740	CELF2	10:11047258-11378674	q1	q2	OK	10.7499	8.78331	-0.291491	-0.575718	0.39155	0.99995	no

ENSG00000048828	ENSG00000048828	FAM120A	9:96208775-96328397	q1	q2	OK	20.3732	20.6985	0.0228485	0.0378411	0.9483	0.99995	no

ENSG00000048991	ENSG00000048991	R3HDM1	2:136289024-136482840	q1	q2	OK	22.3468	24.2978	0.120761	0.214923	0.67635	0.99995	no

ENSG00000049089	ENSG00000049089	COL9A2	1:40766158-40783488	q1	q2	OK	1.39598	1.20317	-0.214436	-0.185295	0.7016	0.99995	no

ENSG00000049130	ENSG00000049130	KITLG	12:88886569-88974628	q1	q2	OK	11.7771	12.2933	0.061889	0.112724	0.8608	0.99995	no

ENSG00000049167	ENSG00000049167	ERCC8	5:60169657-60240900	q1	q2	OK	11.1875	11.9402	0.0939355	0.152831	0.81075	0.99995	no

ENSG00000049192	ENSG00000049192	ADAMTS6	5:64444562-64777747	q1	q2	OK	1.96154	2.39953	0.290766	0.264942	0.53415	0.99995	no

ENSG00000049239	ENSG00000049239	H6PD	1:9294833-9331396	q1	q2	OK	8.28471	7.66775	-0.111647	-0.264922	0.70235	0.99995	no

ENSG00000049245	ENSG00000049245	VAMP3	1:7831328-7841492	q1	q2	OK	24.6007	26.0432	0.0822094	0.127676	0.81165	0.99995	no

ENSG00000049246	ENSG00000049246	PER3	1:7844379-7913572	q1	q2	OK	2.30708	2.99832	0.378087	0.493994	0.33245	0.99995	no

ENSG00000049247	ENSG00000049247	UTS2	1:7844379-7913572	q1	q2	NOTEST	0	0	0	0	1	1	no

ENSG00000049249	ENSG00000049249	TNFRSF9	1:7979906-8000926	q1	q2	NOTEST	0.0137724	0	#NAME?	0	1	1	no

ENSG00000049283	ENSG00000049283	EPN3	17:48609903-48633213	q1	q2	NOTEST	0.17735	0.119141	-0.57393	0	1	1	no

ENSG00000049319	ENSG00000049319	SRD5A2	2:31747549-31806136	q1	q2	NOTEST	0.044338	0.0105826	-2.06684	0	1	1	no

ENSG00000049323	ENSG00000049323	LTBP1	2:33172038-33624576	q1	q2	OK	11.4193	9.59295	-0.251425	-0.442136	0.47435	0.99995	no

ENSG00000049449	ENSG00000049449	RCN1	11:31806339-32127301	q1	q2	OK	156.36	158.69	0.0213381	0.0405904	0.9539	0.99995	no

ENSG00000049540	ENSG00000049540	ELN	7:73442118-73484237	q1	q2	OK	0.899666	0.620058	-0.536988	-0.238921	0.37355	0.99995	no

ENSG00000049541	ENSG00000049541	RFC2	7:73645828-73668774	q1	q2	OK	7.95678	12.2167	0.618596	0.708897	0.3034	0.99995	no

ENSG00000049618	ENSG00000049618	ARID1B	6:157099062-157531913	q1	q2	OK	18.5424	17.79	-0.0597658	-0.129556	0.83455	0.99995	no

ENSG00000049656	ENSG00000049656	CLPTM1L	5:1317858-1345214	q1	q2	OK	48.9183	46.7353	-0.0658629	-0.105447	0.87505	0.99995	no

ENSG00000049759	ENSG00000049759	NEDD4L	18:55711598-56068772	q1	q2	OK	18.4491	13.2212	-0.480697	-0.351819	0.1616	0.99995	no

ENSG00000049768	ENSG00000049768	FOXP3	X:49091926-49121288	q1	q2	NOTEST	0	0	0	0	1	1	no

ENSG00000049769	ENSG00000049769	PPP1R3F	X:49126305-49157929	q1	q2	NOTEST	0	0	0	0	1	1	no

ENSG00000049860	ENSG00000049860	HEXB	5:73923233-74072737	q1	q2	OK	25.9068	26.9339	0.0560967	0.0348172	0.9531	0.99995	no

ENSG00000049883	ENSG00000049883	PTCD2	5:71515235-71656052	q1	q2	OK	5.91758	6.203	0.0679594	0.0497882	0.9332	0.99995	no

ENSG00000050030	ENSG00000050030	KIAA2022	X:73952683-74145282	q1	q2	OK	1.2522	1.1526	-0.119572	-0.190775	0.76915	0.99995	no

ENSG00000050130	ENSG00000050130	JKAMP	14:59895739-60043549	q1	q2	OK	47.9198	48.0008	0.00243642	0.00451883	0.99405	0.99995	no

ENSG00000050165	ENSG00000050165	DKK3	11:11984652-12031316	q1	q2	OK	28.4364	28.0739	-0.0185124	-0.0352887	0.95505	0.99995	no

ENSG00000050327	ENSG00000050327	ARHGEF5	7:143883175-144077725	q1	q2	NOTEST	0.143101	0.0911671	-0.650452	0	1	1	no

ENSG00000050344	ENSG00000050344	NFE2L3	7:26191859-26226745	q1	q2	OK	7.0893	7.25091	0.0325178	0.0558094	0.9349	0.99995	no

ENSG00000050393	ENSG00000050393	MCUR1	6:13786788-13814800	q1	q2	OK	8.9562	8.46294	-0.0817277	-0.129451	0.80855	0.99995	no

ENSG00000050405	ENSG00000050405	LIMA1	12:50569570-50677329	q1	q2	OK	37.1551	44.1014	0.247263	0.543578	0.3964	0.99995	no

ENSG00000050426	ENSG00000050426	LETMD1	12:51441744-51454207	q1	q2	OK	22.1009	23.0253	0.0591157	0.0976358	0.87905	0.99995	no

ENSG00000050438	ENSG00000050438	SLC4A8	12:51745030-51902980	q1	q2	OK	18.0326	18.6974	0.0522274	0.0966058	0.8705	0.99995	no

ENSG00000050555	ENSG00000050555	LAMC3	9:133884468-133969860	q1	q2	OK	14.884	12.5923	-0.241218	-0.253794	0.69105	0.99995	no

ENSG00000050628	ENSG00000050628	PTGER3	1:71318035-71513491	q1	q2	NOTEST	0.0906673	0.0746224	-0.280973	0	1	1	no

ENSG00000050730	ENSG00000050730	TNIP3	4:122052562-122148621	q1	q2	NOTEST	0.0810238	0.0376426	-1.10598	0	1	1	no

ENSG00000050748	ENSG00000050748	MAPK9	5:179660142-179719099	q1	q2	OK	22.3433	23.1572	0.051615	0.104364	0.87505	0.99995	no

ENSG00000050767	ENSG00000050767	COL23A1	5:177664618-178017556	q1	q2	NOTEST	0.304876	0.167777	-0.861679	0	1	1	no

ENSG00000050820	ENSG00000050820	BCAR1	16:75262927-75301951	q1	q2	OK	25.8518	26.7615	0.0498917	0.0953347	0.86925	0.99995	no

ENSG00000051009	ENSG00000051009	FAM160A2	11:6232564-6255941	q1	q2	OK	10.8516	9.79554	-0.147713	-0.244268	0.7255	0.99995	no

ENSG00000051108	ENSG00000051108	HERPUD1	16:56965959-56977798	q1	q2	OK	36.8924	30.7859	-0.261057	-0.495951	0.41245	0.99995	no

ENSG00000051128	ENSG00000051128	HOMER3	19:19010322-19057640	q1	q2	OK	77.3184	74.028	-0.0627396	-0.0966669	0.8753	0.99995	no

ENSG00000051180	ENSG00000051180	RAD51	15:40978921-41024354	q1	q2	OK	15.7015	14.5292	-0.111943	-0.166902	0.78685	0.99995	no

ENSG00000051341	ENSG00000051341	POLQ	3:121150277-121264853	q1	q2	OK	6.55165	7.06505	0.108843	0.12341	0.77885	0.99995	no

ENSG00000051382	ENSG00000051382	PIK3CB	3:138372859-138553780	q1	q2	OK	5.44199	5.7482	0.078976	0.0915969	0.8604	0.99995	no

ENSG00000051523	ENSG00000051523	CYBA	16:88709690-88717560	q1	q2	OK	2.2691	2.96182	0.384362	0.373927	0.55015	0.99995	no

ENSG00000051596	ENSG00000051596	THOC3	5:175333941-175461683	q1	q2	OK	29.3676	27.4963	-0.0949856	-0.159832	0.8002	0.99995	no

ENSG00000051620	ENSG00000051620	HEBP2	6:138723721-139018425	q1	q2	OK	16.046	15.7017	-0.0312874	-0.0173289	0.9839	0.99995	no

ENSG00000051825	ENSG00000051825	MPHOSPH9	12:123636866-123756881	q1	q2	OK	25.1145	29.553	0.234785	0.135158	0.6399	0.99995	no

ENSG00000052126	ENSG00000052126	PLEKHA5	12:19282647-19529334	q1	q2	OK	14.0187	13.7221	-0.030843	-0.0560568	0.9281	0.99995	no

ENSG00000052344	ENSG00000052344	PRSS8	16:31142753-31147083	q1	q2	NOTEST	0.143927	0.129293	-0.1547	0	1	1	no

ENSG00000052723	ENSG00000052723	SIKE1	1:115312099-115323306	q1	q2	OK	24.4785	25.4265	0.0548226	0.0579714	0.88535	0.99995	no

ENSG00000052749	ENSG00000052749	RRP12	10:99116114-99185831	q1	q2	OK	8.65154	7.35015	-0.235184	-0.337464	0.54895	0.99995	no

ENSG00000052795	ENSG00000052795	FNIP2	4:159690289-159959912	q1	q2	OK	12.8258	14.1838	0.145198	0.196462	0.68885	0.99995	no

ENSG00000052802	ENSG00000052802	MSMO1	4:166248774-166264312	q1	q2	OK	104.056	130.337	0.324882	0.737933	0.26235	0.99995	no

ENSG00000052841	ENSG00000052841	TTC17	11:43333512-43516483	q1	q2	OK	43.695	44.7904	0.0357242	0.0309201	0.9467	0.99995	no

ENSG00000052850	ENSG00000052850	ALX4	11:44281993-44331716	q1	q2	NOTEST	0.154836	0.189533	0.291707	0	1	1	no

ENSG00000053108	ENSG00000053108	FSTL4	5:132496129-132948255	q1	q2	NOTEST	0.019166	0.0723345	1.91614	0	1	1	no

ENSG00000053254	ENSG00000053254	FOXN3	14:89591214-90421121	q1	q2	OK	15.1015	15.121	0.0018696	0.00185202	0.99545	0.99995	no

ENSG00000053328	ENSG00000053328	METTL24	6:110501343-110679475	q1	q2	NOTEST	0.133428	0.0519598	-1.36059	0	1	1	no

ENSG00000053371	ENSG00000053371	AKR7A2	1:19630458-19638640	q1	q2	OK	26.2426	21.7982	-0.267703	-0.529336	0.4354	0.99995	no

ENSG00000053372	ENSG00000053372	MRTO4	1:19536994-19586622	q1	q2	OK	25.3864	25.0726	-0.0179462	-0.0208645	0.96755	0.99995	no

ENSG00000053438	ENSG00000053438	NNAT	20:36120873-36156333	q1	q2	OK	40.1089	35.5657	-0.173434	-0.20819	0.7755	0.99995	no

ENSG00000053501	ENSG00000053501	USE1	19:17326154-17330638	q1	q2	OK	11.7852	12.0907	0.0369267	0.0534437	0.9391	0.99995	no

ENSG00000053524	ENSG00000053524	MCF2L2	3:182895830-183146566	q1	q2	OK	0.714234	0.604143	-0.241507	-0.0444981	0.78395	0.99995	no

ENSG00000053702	ENSG00000053702	NRIP2	12:2904118-3050306	q1	q2	OK	1.037	1.11255	0.101457	0.0197155	0.9582	0.99995	no

ENSG00000053747	ENSG00000053747	LAMA3	18:21269406-21535030	q1	q2	NOTEST	0.205551	0.231238	0.16988	0	1	1	no

ENSG00000053770	ENSG00000053770	AP5M1	14:57670517-57756797	q1	q2	OK	20.6958	20.3096	-0.0271776	-0.0243886	0.96535	0.99995	no

ENSG00000053900	ENSG00000053900	ANAPC4	4:25378834-25420120	q1	q2	OK	16.922	19.2085	0.182847	0.304319	0.59465	0.99995	no

ENSG00000053918	ENSG00000053918	KCNQ1	11:2465913-2882798	q1	q2	NOTEST	0.237605	0.204312	-0.217789	0	1	1	no

ENSG00000054116	ENSG00000054116	TRAPPC3	1:36602172-36615098	q1	q2	OK	36.7075	33.5972	-0.127732	-0.261802	0.7023	0.99995	no

ENSG00000054118	ENSG00000054118	THRAP3	1:36690016-36770958	q1	q2	OK	60.5	61.9711	0.034661	0.0665214	0.90205	0.99995	no

ENSG00000054148	ENSG00000054148	PHPT1	9:139743175-139755249	q1	q2	OK	113.43	96.5045	-0.233129	-0.467924	0.51405	0.99995	no

ENSG00000054179	ENSG00000054179	ENTPD2	9:139942549-139948497	q1	q2	NOTEST	0.161924	0.035513	-2.1889	0	1	1	no

ENSG00000054219	ENSG00000054219	LY75	2:160568999-160761260	q1	q2	NOTEST	0.0179413	0.0105638	-0.76415	0	1	1	no

ENSG00000054267	ENSG00000054267	ARID4B	1:235294497-235507847	q1	q2	OK	28.0163	28.3646	0.0178263	0.0173078	0.9642	0.99995	no

ENSG00000054277	ENSG00000054277	OPN3	1:241695433-241965435	q1	q2	OK	17.5719	18.9953	0.112367	0.127446	0.84685	0.99995	no

ENSG00000054282	ENSG00000054282	SDCCAG8	1:243419319-244014381	q1	q2	OK	15.2292	14.6569	-0.0552616	-0.0380766	0.93645	0.99995	no

ENSG00000054356	ENSG00000054356	PTPRN	2:220154344-220174370	q1	q2	OK	1.08303	0.652957	-0.730012	-0.466213	0.2337	0.99995	no

ENSG00000054392	ENSG00000054392	HHAT	1:210501595-210849638	q1	q2	OK	3.08738	2.44417	-0.337039	-0.502288	0.46365	0.99995	no

ENSG00000054523	ENSG00000054523	KIF1B	1:10270862-10441661	q1	q2	OK	78.0476	78.8092	0.0140105	0.0223058	0.9648	0.99995	no

ENSG00000054598	ENSG00000054598	FOXC1	6:1610680-1614127	q1	q2	OK	6.39595	8.72021	0.447205	0.860093	0.22865	0.99995	no

ENSG00000054611	ENSG00000054611	TBC1D22A	22:47158517-47571336	q1	q2	OK	7.48686	7.80178	0.0594428	0.100417	0.8883	0.99995	no

ENSG00000054654	ENSG00000054654	SYNE2	14:64319682-64805317	q1	q2	OK	30.5403	29.2988	-0.0598723	-0.0670081	0.89835	0.99995	no

ENSG00000054690	ENSG00000054690	PLEKHH1	14:67913800-68067004	q1	q2	OK	13.9729	14.6939	0.0725869	0.0877671	0.84445	0.99995	no

ENSG00000054793	ENSG00000054793	ATP9A	20:50213052-50385173	q1	q2	OK	27.8299	26.7135	-0.0590694	-0.143475	0.84215	0.99995	no

ENSG00000054796	ENSG00000054796	SPO11	20:55904814-55919050	q1	q2	NOTEST	0	0	0	0	1	1	no

ENSG00000054803	ENSG00000054803	CBLN4	20:54572495-54580528	q1	q2	NOTEST	0	0	0	0	1	1	no

ENSG00000054938	ENSG00000054938	CHRDL2	11:74407473-74442430	q1	q2	NOTEST	0.050606	0.0863332	0.770606	0	1	1	no

ENSG00000054965	ENSG00000054965	FAM168A	11:73111531-73309234	q1	q2	OK	32.7185	32.0083	-0.0316597	-0.0667583	0.9118	0.99995	no

ENSG00000054967	ENSG00000054967	RELT	11:73087308-73108519	q1	q2	OK	6.25841	5.84261	-0.0991842	-0.166123	0.80555	0.99995	no

ENSG00000054983	ENSG00000054983	GALC	14:88176762-88460009	q1	q2	OK	6.41057	7.41016	0.209051	0.371704	0.52645	0.99995	no

ENSG00000055044	ENSG00000055044	NOP58	2:203130438-203168389	q1	q2	OK	58.6377	70.4909	0.265608	0.524364	0.39005	0.99995	no

ENSG00000055070	ENSG00000055070	SZRD1	1:16679069-16763919	q1	q2	OK	72.8714	66.7318	-0.126978	-0.264089	0.69285	0.99995	no

ENSG00000055118	ENSG00000055118	KCNH2	7:150642048-150675403	q1	q2	OK	5.4313	4.9397	-0.136873	-0.236967	0.74395	0.99995	no

ENSG00000055130	ENSG00000055130	CUL1	7:148393558-148498128	q1	q2	OK	34.5084	34.6711	0.00678501	0.0163173	0.98095	0.99995	no

ENSG00000055147	ENSG00000055147	FAM114A2	5:153369687-153825410	q1	q2	OK	11.0497	10.8637	-0.0244848	-0.0163971	0.97365	0.99995	no

ENSG00000055163	ENSG00000055163	CYFIP2	5:156687131-157002783	q1	q2	OK	32.705	27.2308	-0.264275	-0.29972	0.5928	0.99995	no

ENSG00000055208	ENSG00000055208	TAB2	6:149539061-149732749	q1	q2	OK	33.5175	35.1576	0.0689211	0.153215	0.812	0.99995	no

ENSG00000055211	ENSG00000055211	GINM1	6:149887429-149912884	q1	q2	OK	16.3186	16.9369	0.0536502	0.104021	0.8745	0.99995	no

ENSG00000055332	ENSG00000055332	EIF2AK2	2:37311593-37384208	q1	q2	OK	8.34425	9.81257	0.23385	0.332874	0.46245	0.99995	no

ENSG00000055483	ENSG00000055483	USP36	17:76783462-76837523	q1	q2	OK	10.3049	10.1411	-0.0231167	-0.0364854	0.9459	0.99995	no

ENSG00000055609	ENSG00000055609	KMT2C	7:151832009-152133090	q1	q2	OK	15.4723	15.2418	-0.0216479	-0.02632	0.95005	0.99995	no

ENSG00000055732	ENSG00000055732	MCOLN3	1:85464829-85598821	q1	q2	NOTEST	0.147275	0.131676	-0.161523	0	1	1	no

ENSG00000055813	ENSG00000055813	CCDC85A	2:56179292-56613308	q1	q2	NOTEST	0.160115	0.0795018	-1.01005	0	1	1	no

ENSG00000055917	ENSG00000055917	PUM2	2:20448451-20551995	q1	q2	OK	43.5519	47.4047	0.122294	0.204379	0.6764	0.99995	no

ENSG00000055950	ENSG00000055950	MRPL43	10:102729214-102754158	q1	q2	OK	46.7579	41.4448	-0.174017	-0.257041	0.7143	0.99995	no

ENSG00000055955	ENSG00000055955	ITIH4	3:52846990-52931612	q1	q2	OK	0.887007	1.1181	0.334032	0.0663766	0.69965	0.99995	no

ENSG00000055957	ENSG00000055957	ITIH1	3:52811602-52826078	q1	q2	NOTEST	0.0375524	0	#NAME?	0	1	1	no

ENSG00000056050	ENSG00000056050	C4orf27	4:170650615-170679104	q1	q2	OK	39.7009	43.7055	0.138642	0.276867	0.6856	0.99995	no

ENSG00000056097	ENSG00000056097	ZFR	5:32354455-32444867	q1	q2	OK	52.0609	56.2386	0.111362	0.244976	0.7033	0.99995	no

ENSG00000056277	ENSG00000056277	ZNF280C	X:129336684-129402873	q1	q2	OK	5.01393	5.56352	0.150056	0.29042	0.67395	0.99995	no

ENSG00000056291	ENSG00000056291	NPFFR2	4:72897520-73013784	q1	q2	NOTEST	0	0.0507075	inf	0	1	1	no

ENSG00000056487	ENSG00000056487	PHF21B	22:45277041-45405880	q1	q2	OK	21.9496	22.2143	0.0172931	0.0351035	0.9512	0.99995	no

ENSG00000056558	ENSG00000056558	TRAF1	9:123664670-123691451	q1	q2	NOTEST	0.170862	0.181246	0.0851171	0	1	1	no

ENSG00000056586	ENSG00000056586	RC3H2	9:125606834-125667620	q1	q2	OK	16.8172	19.2432	0.194404	0.306747	0.49835	0.99995	no

ENSG00000056661	ENSG00000056661	PCGF2	17:36886487-36906969	q1	q2	OK	34.9366	32.9005	-0.0866281	-0.182407	0.80205	0.99995	no

ENSG00000056736	ENSG00000056736	IL17RB	3:53880606-53899827	q1	q2	OK	3.14011	3.1942	0.0246366	0.025454	0.97195	0.99995	no

ENSG00000056972	ENSG00000056972	TRAF3IP2	6:111620233-111927481	q1	q2	OK	4.97353	5.45851	0.134235	0.056418	0.89395	0.99995	no

ENSG00000056998	ENSG00000056998	GYG2	X:2746828-2800859	q1	q2	OK	13.3617	14.2827	0.0961722	0.167124	0.7838	0.99995	no

ENSG00000057019	ENSG00000057019	DCBLD2	3:98433173-98620533	q1	q2	OK	43.4356	42.6429	-0.0265735	-0.039834	0.9292	0.99995	no

ENSG00000057149	ENSG00000057149	SERPINB3	18:61314812-61391127	q1	q2	NOTEST	0	0	0	0	1	1	no

ENSG00000057252	ENSG00000057252	SOAT1	1:179262924-179327815	q1	q2	OK	14.2114	14.4362	0.0226423	0.0363286	0.95525	0.99995	no

ENSG00000057294	ENSG00000057294	PKP2	12:32943678-33049774	q1	q2	OK	1.31964	1.18331	-0.157315	-0.14741	0.7631	0.99995	no

ENSG00000057468	ENSG00000057468	MSH4	1:76262566-76378923	q1	q2	NOTEST	0.00773943	0.0155445	1.0061	0	1	1	no

ENSG00000057593	ENSG00000057593	F7	13:113760104-113774995	q1	q2	NOTEST	0.0113261	0.0254534	1.1682	0	1	1	no

ENSG00000057608	ENSG00000057608	GDI2	10:5807185-5884095	q1	q2	OK	206.548	221.199	0.0988696	0.229547	0.73615	0.99995	no

ENSG00000057657	ENSG00000057657	PRDM1	6:106534194-106557814	q1	q2	OK	0.869009	1.1109	0.354285	0.378427	0.49815	0.99995	no

ENSG00000057663	ENSG00000057663	ATG5	6:106632350-106773666	q1	q2	OK	11.9095	13.4965	0.180475	0.364562	0.5795	0.99995	no

ENSG00000057704	ENSG00000057704	TMCC3	12:94960899-95044338	q1	q2	OK	1.27531	1.50419	0.238139	0.321616	0.6175	0.99995	no

ENSG00000057757	ENSG00000057757	PITHD1	1:24104894-24114722	q1	q2	OK	37.5501	36.1867	-0.0533565	-0.118617	0.864	0.99995	no

ENSG00000057935	ENSG00000057935	MTA3	2:42721708-42984087	q1	q2	OK	30.671	30.1681	-0.0238502	-0.0451992	0.94005	0.99995	no

ENSG00000058056	ENSG00000058056	USP13	3:179370542-179507189	q1	q2	OK	6.26495	6.84968	0.128733	0.253099	0.68325	0.99995	no

ENSG00000058063	ENSG00000058063	ATP11B	3:182511287-182639726	q1	q2	OK	13.8787	14.07	0.0197538	0.0299574	0.95895	0.99995	no

ENSG00000058085	ENSG00000058085	LAMC2	1:183155372-183214035	q1	q2	NOTEST	0.144216	0.0918977	-0.650135	0	1	1	no

ENSG00000058091	ENSG00000058091	CDK14	7:89964536-90839905	q1	q2	OK	23.3392	25.1014	0.105017	0.178179	0.77265	0.99995	no

ENSG00000058262	ENSG00000058262	SEC61A1	3:127770483-128127485	q1	q2	OK	100.981	92.0943	-0.132894	-0.255729	0.6944	0.99995	no

ENSG00000058272	ENSG00000058272	PPP1R12A	12:79968758-80334448	q1	q2	OK	30.1026	32.5223	0.111542	0.148436	0.7149	0.99995	no

ENSG00000058335	ENSG00000058335	RASGRF1	15:79252288-79383115	q1	q2	NOTEST	0.0572136	0.00408544	-3.8078	0	1	1	no

ENSG00000058404	ENSG00000058404	CAMK2B	7:44256748-44374176	q1	q2	NOTEST	0.324733	0.28056	-0.21094	0	1	1	no

ENSG00000058453	ENSG00000058453	CROCC	1:17066767-17299474	q1	q2	OK	15.5092	13.0912	-0.244531	-0.25825	0.6342	0.99995	no

ENSG00000058600	ENSG00000058600	POLR3E	16:22308729-22346424	q1	q2	OK	20.5403	21.019	0.0332378	0.0539242	0.9208	0.99995	no

ENSG00000058668	ENSG00000058668	ATP2B4	1:203595688-203713209	q1	q2	OK	26.7793	24.6519	-0.119417	-0.178808	0.7105	0.99995	no

ENSG00000058673	ENSG00000058673	ZC3H11A	1:203764781-203823252	q1	q2	OK	76.9676	82.0493	0.0922409	0.150829	0.782	0.99995	no

ENSG00000058729	ENSG00000058729	RIOK2	5:96424778-96772921	q1	q2	OK	13.0337	12.4455	-0.066624	-0.0791511	0.86775	0.99995	no

ENSG00000058799	ENSG00000058799	YIPF1	1:54317391-54356407	q1	q2	OK	18.7519	18.5025	-0.0193131	-0.03653	0.96025	0.99995	no

ENSG00000058804	ENSG00000058804	NDC1	1:54231132-54304533	q1	q2	OK	21.6147	24.2943	0.168607	0.405532	0.55985	0.99995	no

ENSG00000058866	ENSG00000058866	DGKG	3:185764096-186080026	q1	q2	NOTEST	0.2803	0.0704444	-1.99241	0	1	1	no

ENSG00000059122	ENSG00000059122	FLYWCH1	16:2961937-3004277	q1	q2	OK	12.0188	9.87728	-0.283103	-0.427542	0.47275	0.99995	no

ENSG00000059145	ENSG00000059145	UNKL	16:1401923-1464752	q1	q2	OK	11.2653	8.07074	-0.481111	-0.340208	0.2406	0.99995	no

ENSG00000059377	ENSG00000059377	TBXAS1	7:139246315-139720125	q1	q2	OK	2.82409	2.71278	-0.0580164	-0.00930196	0.95735	0.99995	no

ENSG00000059378	ENSG00000059378	PARP12	7:139723543-139763521	q1	q2	OK	1.97751	1.71116	-0.208708	-0.168565	0.7265	0.99995	no

ENSG00000059573	ENSG00000059573	ALDH18A1	10:97365695-97416463	q1	q2	OK	33.7396	37.7721	0.162877	0.331915	0.58095	0.99995	no

ENSG00000059588	ENSG00000059588	TARBP1	1:234527058-234614849	q1	q2	OK	18.6949	17.7948	-0.0711869	-0.113514	0.83615	0.99995	no

ENSG00000059691	ENSG00000059691	PET112	4:152591655-152682175	q1	q2	OK	10.7217	12.5169	0.223347	0.352532	0.5775	0.99995	no

ENSG00000059728	ENSG00000059728	MXD1	2:70120691-70170077	q1	q2	OK	2.61524	2.50536	-0.0619269	-0.0689667	0.9088	0.99995	no

ENSG00000059758	ENSG00000059758	CDK17	12:96672038-96794338	q1	q2	OK	20.2887	21.907	0.110715	0.213349	0.7149	0.99995	no

ENSG00000059769	ENSG00000059769	DNAJC25	9:114393631-114432526	q1	q2	OK	8.14552	12.149	0.57676	0.717063	0.3178	0.99995	no

ENSG00000059804	ENSG00000059804	SLC2A3	12:8071825-8088871	q1	q2	OK	66.1213	95.006	0.522903	1.09637	0.07825	0.99995	no

ENSG00000059915	ENSG00000059915	PSD	10:104162375-104182893	q1	q2	OK	17.9697	16.0544	-0.162594	-0.210735	0.7541	0.99995	no

ENSG00000060069	ENSG00000060069	CTDP1	18:77439800-77514510	q1	q2	OK	9.29958	8.91271	-0.0613013	-0.121549	0.85965	0.99995	no

ENSG00000060138	ENSG00000060138	YBX3	12:10851682-10875911	q1	q2	OK	45.1379	43.5889	-0.0503759	-0.0887815	0.89605	0.99995	no

ENSG00000060140	ENSG00000060140	STYK1	12:10771537-10826917	q1	q2	NOTEST	0.0259297	0	#NAME?	0	1	1	no

ENSG00000060237	ENSG00000060237	WNK1	12:861758-1020618	q1	q2	OK	48.366	49.5631	0.0352738	0.0323074	0.9019	0.99995	no

ENSG00000060303	ENSG00000060303	RPS17P5	6:50824967-50825375	q1	q2	NOTEST	0	0	0	0	1	1	no

ENSG00000060339	ENSG00000060339	CCAR1	10:70480768-70552134	q1	q2	OK	83.6813	84.3242	0.0110408	0.0170553	0.97365	0.99995	no

ENSG00000060491	ENSG00000060491	OGFR	20:61431978-61445352	q1	q2	OK	13.9419	12.809	-0.122268	-0.233554	0.713	0.99995	no

ENSG00000060558	ENSG00000060558	GNA15	19:3136190-3163767	q1	q2	NOTEST	0.0122761	0	#NAME?	0	1	1	no

ENSG00000060566	ENSG00000060566	CREB3L3	19:4153597-4173051	q1	q2	NOTEST	0.0200741	0	#NAME?	0	1	1	no

ENSG00000060642	ENSG00000060642	PIGV	1:27113962-27124889	q1	q2	OK	4.40438	3.48826	-0.336433	-0.456685	0.4548	0.99995	no

ENSG00000060656	ENSG00000060656	PTPRU	1:29563027-29653325	q1	q2	OK	3.46694	3.66149	0.0787652	0.0715027	0.89645	0.99995	no

ENSG00000060688	ENSG00000060688	SNRNP40	1:31732416-31769662	q1	q2	OK	55.2402	59.654	0.110899	0.246652	0.7202	0.99995	no

ENSG00000060709	ENSG00000060709	RIMBP2	12:130880681-131200826	q1	q2	OK	2.6528	2.06261	-0.363047	-0.334236	0.48725	0.99995	no

ENSG00000060718	ENSG00000060718	COL11A1	1:103342022-103574052	q1	q2	OK	15.0669	15.4029	0.0318212	0.0346256	0.96285	0.99995	no

ENSG00000060749	ENSG00000060749	QSER1	11:32914723-33014862	q1	q2	OK	17.2946	19.625	0.182368	0.360702	0.5077	0.99995	no

ENSG00000060762	ENSG00000060762	MPC1	6:166778406-166796486	q1	q2	OK	26.4101	28.3802	0.103796	0.186167	0.7966	0.99995	no

ENSG00000060971	ENSG00000060971	ACAA1	3:38080695-38178733	q1	q2	OK	25.3177	23.7427	-0.0926601	-0.169456	0.80435	0.99995	no

ENSG00000060982	ENSG00000060982	BCAT1	12:24964294-25113092	q1	q2	OK	71.8175	85.3684	0.249367	0.447999	0.4014	0.99995	no

ENSG00000061273	ENSG00000061273	HDAC7	12:48099867-48231681	q1	q2	OK	38.6685	39.1707	0.0186138	0.0277984	0.94995	0.99995	no

ENSG00000061337	ENSG00000061337	LZTS1	8:20103675-20161474	q1	q2	OK	7.33438	8.26363	0.172102	0.370085	0.59115	0.99995	no

ENSG00000061455	ENSG00000061455	PRDM6	5:122422942-122529960	q1	q2	OK	0.349401	0.505081	0.531632	0.231438	0.5231	0.99995	no

ENSG00000061492	ENSG00000061492	WNT8A	5:137419580-137428054	q1	q2	NOTEST	0.0365595	0	#NAME?	0	1	1	no

ENSG00000061656	ENSG00000061656	SPAG4	20:34203813-34208971	q1	q2	NOTEST	0.418527	0.479522	0.196276	0	1	1	no

ENSG00000061676	ENSG00000061676	NCKAP1	2:183773842-183903586	q1	q2	OK	62.0862	70.6385	0.186182	0.321247	0.4947	0.99995	no

ENSG00000061794	ENSG00000061794	MRPS35	12:27863705-27909228	q1	q2	OK	29.2944	32.0004	0.127468	0.284983	0.6727	0.99995	no

ENSG00000061918	ENSG00000061918	GUCY1B3	4:156680143-156728743	q1	q2	OK	7.66402	8.28969	0.113216	0.219835	0.7598	0.99995	no

ENSG00000061936	ENSG00000061936	SFSWAP	12:132195625-132284282	q1	q2	OK	29.068	26.9691	-0.108125	-0.22834	0.72175	0.99995	no

ENSG00000061938	ENSG00000061938	TNK2	3:195590234-195638816	q1	q2	OK	24.4523	20.9074	-0.225961	-0.393355	0.4722	0.99995	no

ENSG00000061987	ENSG00000061987	MON2	12:62860596-62991363	q1	q2	OK	22.464	23.2046	0.0467926	0.0579494	0.8972	0.99995	no

ENSG00000062038	ENSG00000062038	CDH3	16:68670091-68756519	q1	q2	OK	2.49329	1.86866	-0.416049	-0.401324	0.3851	0.99995	no

ENSG00000062096	ENSG00000062096	ARSF	X:2959511-3030767	q1	q2	NOTEST	0.0420633	0.0572413	0.444493	0	1	1	no

ENSG00000062194	ENSG00000062194	GPBP1	5:56469774-56560506	q1	q2	OK	66.9234	71.7941	0.101354	0.209869	0.7248	0.99995	no

ENSG00000062282	ENSG00000062282	DGAT2	11:75469499-75514460	q1	q2	OK	0.582014	0.525717	-0.146768	-0.0913024	0.8579	0.99995	no

ENSG00000062370	ENSG00000062370	ZNF112	19:44830707-44952766	q1	q2	OK	5.58868	5.77832	0.0481427	0.0745594	0.90955	0.99995	no

ENSG00000062485	ENSG00000062485	CS	12:56665482-56727837	q1	q2	OK	95.5973	97.0386	0.0215891	0.0371126	0.94805	0.99995	no

ENSG00000062524	ENSG00000062524	LTK	15:41795835-41806085	q1	q2	NOTEST	0.0880248	0.123183	0.484827	0	1	1	no

ENSG00000062582	ENSG00000062582	MRPS24	7:43906156-43966010	q1	q2	OK	65.391	61.0126	-0.0999854	-0.164914	0.81145	0.99995	no

ENSG00000062598	ENSG00000062598	ELMO2	20:44994687-45087915	q1	q2	OK	30.1898	30.9126	0.0341351	0.0641285	0.9055	0.99995	no

ENSG00000062650	ENSG00000062650	WAPAL	10:88195012-88281572	q1	q2	OK	21.0949	23.8883	0.179413	0.355393	0.58075	0.99995	no

ENSG00000062716	ENSG00000062716	VMP1	17:57697218-57919616	q1	q2	OK	32.7547	37.2347	0.184945	0.109635	0.8182	0.99995	no

ENSG00000062725	ENSG00000062725	APPBP2	17:58520519-58603580	q1	q2	OK	19.9338	21.0615	0.0793869	0.155825	0.77625	0.99995	no

ENSG00000062822	ENSG00000062822	POLD1	19:50887460-50934570	q1	q2	OK	31.6164	27.9185	-0.179455	-0.282309	0.61345	0.99995	no

ENSG00000063015	ENSG00000063015	SEZ6	17:27232267-27384234	q1	q2	OK	4.60355	6.15681	0.419436	0.179425	0.69295	0.99995	no

ENSG00000063046	ENSG00000063046	EIF4B	12:53399941-53458156	q1	q2	OK	125.66	126.58	0.0105273	0.0200724	0.97195	0.99995	no

ENSG00000063127	ENSG00000063127	SLC6A16	19:49792894-49828482	q1	q2	OK	11.557	11.1821	-0.0475881	-0.050786	0.91595	0.99995	no

ENSG00000063169	ENSG00000063169	GLTSCR1	19:48110780-48246391	q1	q2	OK	9.90071	9.48486	-0.0619048	-0.0491667	0.9271	0.99995	no

ENSG00000063176	ENSG00000063176	SPHK2	19:49118584-49140695	q1	q2	OK	19.7545	16.6155	-0.249648	-0.159096	0.7529	0.99995	no

ENSG00000063177	ENSG00000063177	RPL18	19:49118584-49140695	q1	q2	OK	641.725	622.865	-0.043035	-0.0900304	0.8928	0.99995	no

ENSG00000063180	ENSG00000063180	CA11	19:49141198-49184461	q1	q2	OK	10.6377	9.86973	-0.108103	-0.160971	0.77015	0.99995	no

ENSG00000063241	ENSG00000063241	ISOC2	19:55964351-55973710	q1	q2	OK	19.365	15.8876	-0.285554	-0.488976	0.4801	0.99995	no

ENSG00000063244	ENSG00000063244	U2AF2	19:56165511-56186081	q1	q2	OK	165.334	154.386	-0.0988356	-0.196889	0.75085	0.99995	no

ENSG00000063245	ENSG00000063245	EPN1	19:56186591-56249768	q1	q2	OK	43.7845	39.8043	-0.137494	-0.324955	0.6323	0.99995	no

ENSG00000063322	ENSG00000063322	MED29	19:39881942-39891277	q1	q2	OK	13.5072	12.7556	-0.0826022	-0.18102	0.7841	0.99995	no

ENSG00000063438	ENSG00000063438	AHRR	5:271735-438406	q1	q2	OK	1.11879	0.805144	-0.474625	-0.15488	0.5977	0.99995	no

ENSG00000063515	ENSG00000063515	GSC2	22:19136088-19137796	q1	q2	NOTEST	0	0	0	0	1	1	no

ENSG00000063587	ENSG00000063587	ZNF275	X:152599612-152625568	q1	q2	NOTEST	0	0	0	0	1	1	no

ENSG00000063601	ENSG00000063601	MTMR1	X:149861434-149933576	q1	q2	OK	0.755916	3.33278	2.14043	1.64804	0.00925	0.99995	no

ENSG00000063660	ENSG00000063660	GPC1	2:241375087-241407493	q1	q2	OK	36.0597	31.5169	-0.19426	-0.44211	0.49725	0.99995	no

ENSG00000063761	ENSG00000063761	ADCK1	14:78266425-78401355	q1	q2	OK	4.05361	3.90061	-0.0555077	-0.0734148	0.9018	0.99995	no

ENSG00000063854	ENSG00000063854	HAGH	16:1845620-1934295	q1	q2	OK	27.5314	23.7022	-0.216056	-0.273496	0.69005	0.99995	no

ENSG00000063978	ENSG00000063978	RNF4	4:2420700-2734292	q1	q2	OK	38.4145	40.5686	0.0787131	0.11505	0.81915	0.99995	no

ENSG00000064012	ENSG00000064012	CASP8	2:202098165-202152434	q1	q2	OK	2.42314	2.27099	-0.0935546	-0.0849492	0.85275	0.99995	no

ENSG00000064042	ENSG00000064042	LIMCH1	4:41361623-41702061	q1	q2	OK	28.2073	30.8865	0.13091	0.253333	0.6409	0.99995	no

ENSG00000064102	ENSG00000064102	ASUN	12:27058113-27091259	q1	q2	OK	15.5378	16.0459	0.0464228	0.0833197	0.89755	0.99995	no

ENSG00000064115	ENSG00000064115	TM7SF3	12:27124518-27167367	q1	q2	OK	28.6322	29.5212	0.044113	0.0546575	0.89195	0.99995	no

ENSG00000064195	ENSG00000064195	DLX3	17:48067368-48072588	q1	q2	NOTEST	0.438644	0.272617	-0.686173	0	1	1	no

ENSG00000064199	ENSG00000064199	SPA17	11:124492731-124567414	q1	q2	OK	20.3087	20.8644	0.038947	0.0365755	0.9545	0.99995	no

ENSG00000064201	ENSG00000064201	TSPAN32	11:2316874-2339430	q1	q2	NOTEST	0.0915307	0.0411414	-1.15366	0	1	1	no

ENSG00000064205	ENSG00000064205	WISP2	20:43285091-43379675	q1	q2	NOTEST	0.0221449	0.0423752	0.936245	0	1	1	no

ENSG00000064218	ENSG00000064218	DMRT3	9:976963-991731	q1	q2	NOTEST	0	0	0	0	1	1	no

ENSG00000064225	ENSG00000064225	ST3GAL6	3:98433173-98620533	q1	q2	OK	2.93057	2.82616	-0.0523355	-0.0155192	0.9471	0.99995	no

ENSG00000064270	ENSG00000064270	ATP2C2	16:84402132-84500967	q1	q2	OK	0.682783	0.706529	0.0493233	0.0158754	0.95915	0.99995	no

ENSG00000064300	ENSG00000064300	NGFR	17:47572654-47661189	q1	q2	OK	81.3997	85.0916	0.063992	0.134182	0.84445	0.99995	no

ENSG00000064309	ENSG00000064309	CDON	11:125820010-125933230	q1	q2	OK	9.22362	10.3223	0.162361	0.229439	0.69145	0.99995	no

ENSG00000064313	ENSG00000064313	TAF2	8:120743014-120845103	q1	q2	OK	16.4282	18.6319	0.181607	0.308066	0.5601	0.99995	no

ENSG00000064393	ENSG00000064393	HIPK2	7:139246315-139720125	q1	q2	OK	20.7385	22.864	0.140768	0.322139	0.63285	0.99995	no

ENSG00000064419	ENSG00000064419	TNPO3	7:128594947-128695198	q1	q2	OK	25.1875	25.8698	0.0385579	0.0908478	0.89605	0.99995	no

ENSG00000064489	ENSG00000064489	MEF2BNB-MEF2B	19:19256375-19314233	q1	q2	OK	2.75368	1.49618	-0.880072	-0.426264	0.50285	0.99995	no

ENSG00000064490	ENSG00000064490	RFXANK	19:19256375-19314233	q1	q2	OK	59.1139	51.4285	-0.200931	-0.254864	0.69765	0.99995	no

ENSG00000064545	ENSG00000064545	TMEM161A	19:19229977-19249322	q1	q2	OK	13.2623	9.78393	-0.438848	-0.627221	0.252	0.99995	no

ENSG00000064547	ENSG00000064547	LPAR2	19:19734476-19739739	q1	q2	OK	59.6002	53.7001	-0.150394	-0.252539	0.7021	0.99995	no

ENSG00000064601	ENSG00000064601	CTSA	20:44517263-44540794	q1	q2	OK	26.6748	23.8213	-0.163222	-0.17199	0.74	0.99995	no

ENSG00000064607	ENSG00000064607	SUGP2	19:19101696-19170563	q1	q2	OK	58.4772	58.2317	-0.00607131	-0.00900654	0.9865	0.99995	no

ENSG00000064651	ENSG00000064651	SLC12A2	5:127419457-127525380	q1	q2	OK	12.2625	15.1598	0.306003	0.358571	0.4058	0.99995	no

ENSG00000064652	ENSG00000064652	SNX24	5:122179133-122390362	q1	q2	OK	15.5783	16.4905	0.0820935	0.0898796	0.8955	0.99995	no

ENSG00000064655	ENSG00000064655	EYA2	20:45523262-45817492	q1	q2	OK	0.608853	0.590726	-0.0436043	-0.0215415	0.9506	0.99995	no

ENSG00000064666	ENSG00000064666	CNN2	19:1026297-1039068	q1	q2	OK	86.5843	90.9813	0.0714647	0.146154	0.80125	0.99995	no

ENSG00000064687	ENSG00000064687	ABCA7	19:1040101-1065571	q1	q2	OK	7.44085	5.92355	-0.329006	-0.328527	0.50615	0.99995	no

ENSG00000064692	ENSG00000064692	SNCAIP	5:121646991-121814782	q1	q2	OK	13.7826	11.0727	-0.315847	-0.512589	0.382	0.99995	no

ENSG00000064703	ENSG00000064703	DDX20	1:112025969-112310638	q1	q2	OK	13.8108	15.1268	0.131314	0.15503	0.79965	0.99995	no

ENSG00000064726	ENSG00000064726	BTBD1	15:83509837-83772195	q1	q2	OK	31.5222	35.1477	0.157063	0.302642	0.6291	0.99995	no

ENSG00000064763	ENSG00000064763	FAR2	12:29302035-29534122	q1	q2	OK	4.60687	4.95096	0.103921	0.0682808	0.87985	0.99995	no

ENSG00000064787	ENSG00000064787	BCAS1	20:52553315-52687304	q1	q2	NOTEST	0.142411	0.0944865	-0.591879	0	1	1	no

ENSG00000064835	ENSG00000064835	POU1F1	3:87308553-87325737	q1	q2	NOTEST	0	0	0	0	1	1	no

ENSG00000064886	ENSG00000064886	CHI3L2	1:111729795-111786062	q1	q2	NOTEST	0.0703914	0.0602014	-0.225601	0	1	1	no

ENSG00000064932	ENSG00000064932	SBNO2	19:1107635-1174282	q1	q2	OK	5.84721	5.95436	0.026198	0.0309285	0.9482	0.99995	no

ENSG00000064933	ENSG00000064933	PMS1	2:190649106-190742355	q1	q2	OK	20.3947	23.676	0.215226	0.370592	0.5159	0.99995	no

ENSG00000064961	ENSG00000064961	HMG20B	19:3506270-3579086	q1	q2	OK	47.9303	38.9378	-0.299765	-0.417106	0.4933	0.99995	no

ENSG00000064989	ENSG00000064989	CALCRL	2:187866602-188430487	q1	q2	OK	1.65253	1.86094	0.171355	0.0720213	0.8427	0.99995	no

ENSG00000064995	ENSG00000064995	TAF11	6:34759856-34855866	q1	q2	OK	35.3969	35.1768	-0.00899987	-0.0128816	0.98485	0.99995	no

ENSG00000064999	ENSG00000064999	ANKS1A	6:34857041-35059179	q1	q2	OK	12.0489	11.4246	-0.076769	-0.152019	0.8017	0.99995	no

ENSG00000065000	ENSG00000065000	AP3D1	19:2100987-2248678	q1	q2	OK	42.3456	39.9459	-0.0841677	-0.0911123	0.85305	0.99995	no

ENSG00000065029	ENSG00000065029	ZNF76	6:35226685-35263762	q1	q2	OK	24.4488	20.5579	-0.250069	-0.431788	0.51375	0.99995	no

ENSG00000065054	ENSG00000065054	SLC9A3R2	16:2075356-2089027	q1	q2	OK	8.33382	6.8075	-0.291853	-0.406127	0.5607	0.99995	no

ENSG00000065057	ENSG00000065057	NTHL1	16:2089815-2185899	q1	q2	OK	13.5591	13.6125	0.00566321	0.00294243	0.99625	0.99995	no

ENSG00000065060	ENSG00000065060	UHRF1BP1	6:34759856-34855866	q1	q2	OK	8.28529	8.92268	0.106925	0.204396	0.7774	0.99995	no

ENSG00000065135	ENSG00000065135	GNAI3	1:110091232-110136975	q1	q2	OK	59.6342	65.4129	0.133435	0.332837	0.62805	0.99995	no

ENSG00000065150	ENSG00000065150	IPO5	13:98605911-98676551	q1	q2	OK	96.5939	102.871	0.0908379	0.165248	0.76225	0.99995	no

ENSG00000065154	ENSG00000065154	OAT	10:126085871-126107545	q1	q2	OK	32.3355	31.5123	-0.0372016	-0.0760474	0.90095	0.99995	no

ENSG00000065183	ENSG00000065183	WDR3	1:118472342-118727846	q1	q2	OK	10.8455	13.3945	0.304542	0.323946	0.64915	0.99995	no

ENSG00000065243	ENSG00000065243	PKN2	1:89003195-89301938	q1	q2	OK	25.9946	25.9443	-0.00279292	-0.00348816	0.99425	0.99995	no

ENSG00000065268	ENSG00000065268	WDR18	19:983173-994569	q1	q2	OK	36.8372	34.1123	-0.110872	-0.239308	0.71955	0.99995	no

ENSG00000065308	ENSG00000065308	TRAM2	6:52285105-52441713	q1	q2	OK	10.0038	9.70466	-0.0437995	-0.0734726	0.9151	0.99995	no

ENSG00000065320	ENSG00000065320	NTN1	17:8924858-9147317	q1	q2	OK	34.0635	27.9699	-0.284354	-0.694603	0.3118	0.99995	no

ENSG00000065325	ENSG00000065325	GLP2R	17:9725522-9795419	q1	q2	NOTEST	0.00542744	0.0105031	0.952464	0	1	1	no

ENSG00000065328	ENSG00000065328	MCM10	10:13203553-13253104	q1	q2	OK	23.049	23.5053	0.0282836	0.0487608	0.92245	0.99995	no

ENSG00000065357	ENSG00000065357	DGKA	12:56295196-56347811	q1	q2	OK	6.30153	5.67227	-0.151776	-0.107237	0.7781	0.99995	no

ENSG00000065361	ENSG00000065361	ERBB3	12:56473640-56507691	q1	q2	OK	0.62577	0.701996	0.16583	0.0238571	0.8435	0.99995	no

ENSG00000065371	ENSG00000065371	ROPN1	3:123687367-123711025	q1	q2	NOTEST	0	0	0	0	1	1	no

ENSG00000065413	ENSG00000065413	ANKRD44	2:197831740-198175897	q1	q2	OK	4.34507	6.58957	0.600804	0.606898	0.30065	0.99995	no

ENSG00000065427	ENSG00000065427	KARS	16:75661621-75795770	q1	q2	OK	70.2788	74.3206	0.0806718	0.141177	0.82995	0.99995	no

ENSG00000065457	ENSG00000065457	ADAT1	16:75562429-75657198	q1	q2	OK	33.6179	33.2842	-0.0143907	-0.0118521	0.98365	0.99995	no

ENSG00000065485	ENSG00000065485	PDIA5	3:122785908-122992977	q1	q2	OK	17.5577	19.7903	0.172692	0.246079	0.70985	0.99995	no

ENSG00000065491	ENSG00000065491	TBC1D22B	6:37179955-37300746	q1	q2	OK	6.03357	5.69469	-0.0833951	-0.154496	0.82955	0.99995	no

ENSG00000065518	ENSG00000065518	NDUFB4	3:120315155-120321347	q1	q2	OK	137.411	136.112	-0.0137042	-0.0314834	0.9632	0.99995	no

ENSG00000065526	ENSG00000065526	SPEN	1:16160559-16266955	q1	q2	OK	21.304	19.8986	-0.0984586	-0.0956883	0.8152	0.99995	no

ENSG00000065534	ENSG00000065534	MYLK	3:123304388-123603178	q1	q2	OK	14.2918	20.3082	0.506878	0.444314	0.2019	0.99995	no

ENSG00000065548	ENSG00000065548	ZC3H15	2:187350882-187374090	q1	q2	OK	57.2027	62.8626	0.136118	0.313175	0.62925	0.99995	no

ENSG00000065559	ENSG00000065559	MAP2K4	17:11901669-12047147	q1	q2	OK	22.6283	23.4723	0.0528365	0.113124	0.8513	0.99995	no

ENSG00000065600	ENSG00000065600	TMEM206	1:212537272-212588243	q1	q2	OK	14.4437	15.0285	0.0572573	0.112712	0.8684	0.99995	no

ENSG00000065609	ENSG00000065609	SNAP91	6:84262598-84419410	q1	q2	OK	1.89814	1.73095	-0.133021	-0.133489	0.74675	0.99995	no

ENSG00000065613	ENSG00000065613	SLK	10:105726958-105788991	q1	q2	OK	14.2596	14.3943	0.0135633	0.0274028	0.9619	0.99995	no

ENSG00000065615	ENSG00000065615	CYB5R4	6:84569361-84677143	q1	q2	OK	8.29273	9.47896	0.192881	0.186459	0.7913	0.99995	no

ENSG00000065618	ENSG00000065618	COL17A1	10:105791043-105845760	q1	q2	NOTEST	0.0179482	0.0309462	0.785927	0	1	1	no

ENSG00000065621	ENSG00000065621	GSTO2	10:106028630-106064703	q1	q2	NOTEST	0.203923	0.187314	-0.122565	0	1	1	no

ENSG00000065665	ENSG00000065665	SEC61A2	10:12171635-12292588	q1	q2	OK	24.0943	26.8141	0.154299	0.130259	0.8095	0.99995	no

ENSG00000065675	ENSG00000065675	PRKCQ	10:6469104-6622263	q1	q2	OK	0.965166	1.14464	0.246049	0.303583	0.6533	0.99995	no

ENSG00000065717	ENSG00000065717	TLE2	19:2997635-3047633	q1	q2	OK	2.19529	2.24359	0.0314021	0.0277652	0.94815	0.99995	no

ENSG00000065802	ENSG00000065802	ASB1	2:239335382-239360891	q1	q2	OK	14.1078	15.2803	0.115183	0.141883	0.7851	0.99995	no

ENSG00000065809	ENSG00000065809	FAM107B	10:14560555-14816896	q1	q2	OK	35.8592	37.6357	0.0697557	0.139865	0.80805	0.99995	no

ENSG00000065833	ENSG00000065833	ME1	6:83920107-84140797	q1	q2	OK	0.781872	0.570945	-0.453582	-0.428057	0.55225	0.99995	no

ENSG00000065882	ENSG00000065882	TBC1D1	4:37892707-38140796	q1	q2	OK	45.1746	39.68	-0.1871	-0.29328	0.6568	0.99995	no

ENSG00000065883	ENSG00000065883	CDK13	7:39989635-40136733	q1	q2	OK	22.209	22.8955	0.0439141	0.0802037	0.89335	0.99995	no

ENSG00000065911	ENSG00000065911	MTHFD2	2:74425688-74621009	q1	q2	OK	66.0461	68.1432	0.0450946	0.0494101	0.9419	0.99995	no

ENSG00000065923	ENSG00000065923	SLC9A7	X:46464752-46618490	q1	q2	OK	0.994268	0.976965	-0.0253279	-0.0184251	0.9693	0.99995	no

ENSG00000065970	ENSG00000065970	FOXJ2	12:8185298-8208099	q1	q2	OK	7.17012	7.19913	0.00582454	0.0122597	0.98525	0.99995	no

ENSG00000065978	ENSG00000065978	YBX1	1:43148097-43168020	q1	q2	OK	842.209	864.441	0.0375895	0.0762629	0.91355	0.99995	no

ENSG00000065989	ENSG00000065989	PDE4A	19:10501809-10580305	q1	q2	OK	1.04442	1.08796	0.05893	0.0256995	0.9677	0.99995	no

ENSG00000066027	ENSG00000066027	PPP2R5A	1:212458878-212535200	q1	q2	OK	4.48053	4.64149	0.0509166	0.0844829	0.89825	0.99995	no

ENSG00000066032	ENSG00000066032	CTNNA2	2:79384131-80875905	q1	q2	OK	31.158	28.2843	-0.139603	-0.304999	0.63425	0.99995	no

ENSG00000066044	ENSG00000066044	ELAVL1	19:8023462-8070543	q1	q2	OK	64.126	68.3946	0.0929722	0.173087	0.80565	0.99995	no

ENSG00000066056	ENSG00000066056	TIE1	1:43766663-43788779	q1	q2	OK	1.81692	2.01725	0.150897	0.115385	0.76915	0.99995	no

ENSG00000066084	ENSG00000066084	DIP2B	12:50898767-51142450	q1	q2	OK	14.5734	16.5135	0.180303	0.393924	0.52455	0.99995	no

ENSG00000066117	ENSG00000066117	SMARCD1	12:50478754-50494495	q1	q2	OK	98.5595	102.052	0.0502442	0.0906467	0.86005	0.99995	no

ENSG00000066135	ENSG00000066135	KDM4A	1:44115828-44396831	q1	q2	OK	32.7071	33.7843	0.0467501	0.0900272	0.8821	0.99995	no

ENSG00000066136	ENSG00000066136	NFYC	1:41156051-41237275	q1	q2	OK	33.8801	34.5101	0.0265813	0.050917	0.9386	0.99995	no

ENSG00000066185	ENSG00000066185	ZMYND12	1:42895999-43120335	q1	q2	NOTEST	0.116561	0.102447	-0.186205	0	1	1	no

ENSG00000066230	ENSG00000066230	SLC9A3	5:473350-524447	q1	q2	NOTEST	0.403772	0.354584	-0.187411	0	1	1	no

ENSG00000066248	ENSG00000066248	NGEF	2:233562008-233880595	q1	q2	OK	1.72118	1.72663	0.00456547	0.00134122	0.9967	0.99995	no

ENSG00000066279	ENSG00000066279	ASPM	1:197053257-197115824	q1	q2	OK	21.3251	23.3381	0.130134	0.251888	0.71305	0.99995	no

ENSG00000066294	ENSG00000066294	CD84	1:160506862-160549306	q1	q2	NOTEST	0	0.00895419	inf	0	1	1	no

ENSG00000066322	ENSG00000066322	ELOVL1	1:43829067-43833696	q1	q2	OK	16.1987	17.6048	0.120097	0.210597	0.7482	0.99995	no

ENSG00000066336	ENSG00000066336	SPI1	11:47376410-47400127	q1	q2	NOTEST	0.0504943	0	#NAME?	0	1	1	no

ENSG00000066379	ENSG00000066379	ZNRD1	6:29968787-30032686	q1	q2	NOTEST	0.0755227	0	#NAME?	0	1	1	no

ENSG00000066382	ENSG00000066382	MPPED2	11:30406039-30652055	q1	q2	OK	7.3188	7.54992	0.0448556	0.0707692	0.9117	0.99995	no

ENSG00000066405	ENSG00000066405	CLDN18	3:137717576-137752494	q1	q2	NOTEST	0.251981	0.415013	0.719846	0	1	1	no

ENSG00000066422	ENSG00000066422	ZBTB11	3:101367732-101398061	q1	q2	OK	8.95306	9.06183	0.017422	0.0360378	0.95495	0.99995	no

ENSG00000066427	ENSG00000066427	ATXN3	14:92524895-92572965	q1	q2	OK	13.4104	11.6589	-0.201924	-0.238201	0.6392	0.99995	no

ENSG00000066455	ENSG00000066455	GOLGA5	14:93260575-93306308	q1	q2	OK	15.5479	15.9433	0.0362329	0.0779	0.9091	0.99995	no

ENSG00000066468	ENSG00000066468	FGFR2	10:123237847-123357972	q1	q2	OK	10.4636	9.26163	-0.176045	-0.303753	0.5947	0.99995	no

ENSG00000066557	ENSG00000066557	LRRC40	1:70034080-70671303	q1	q2	OK	23.3397	25.487	0.126975	0.272152	0.6979	0.99995	no

ENSG00000066583	ENSG00000066583	ISOC1	5:128430443-128449721	q1	q2	OK	17.5065	19.1518	0.12959	0.271932	0.6976	0.99995	no

ENSG00000066629	ENSG00000066629	EML1	14:100204029-100408397	q1	q2	OK	16.7936	16.4445	-0.0303105	-0.0561996	0.9154	0.99995	no

ENSG00000066651	ENSG00000066651	TRMT11	6:126307575-126360422	q1	q2	OK	14.3044	16.4629	0.202759	0.368565	0.5515	0.99995	no

ENSG00000066654	ENSG00000066654	THUMPD1	16:20621564-20936328	q1	q2	OK	25.216	26.3593	0.0639708	0.0917944	0.8783	0.99995	no

ENSG00000066697	ENSG00000066697	MSANTD3	9:103189437-103339918	q1	q2	OK	29.197	29.3344	0.00677262	0.00644606	0.99185	0.99995	no

ENSG00000066735	ENSG00000066735	KIF26A	14:104605059-104647231	q1	q2	OK	11.2215	11.4619	0.0305766	0.0729233	0.91555	0.99995	no

ENSG00000066739	ENSG00000066739	ATG2B	14:96747594-96853625	q1	q2	OK	8.34924	8.73779	0.0656226	0.0391247	0.87565	0.99995	no

ENSG00000066777	ENSG00000066777	ARFGEF1	8:67876333-68255912	q1	q2	OK	19.9793	17.8194	-0.165052	-0.173858	0.73275	0.99995	no

ENSG00000066813	ENSG00000066813	ACSM2B	16:20547547-20587749	q1	q2	NOTEST	0.0230233	0	#NAME?	0	1	1	no

ENSG00000066827	ENSG00000066827	ZFAT	8:135490030-135725292	q1	q2	OK	2.4086	2.50378	0.0559109	0.0692206	0.90275	0.99995	no

ENSG00000066855	ENSG00000066855	MTFR1	8:66556968-66754557	q1	q2	OK	29.8121	31.6678	0.0871224	0.0726696	0.8593	0.99995	no

ENSG00000066923	ENSG00000066923	STAG3	7:99775185-99869855	q1	q2	OK	8.09474	7.70587	-0.0710257	-0.051687	0.9201	0.99995	no

ENSG00000066926	ENSG00000066926	FECH	18:55215514-55254004	q1	q2	OK	8.30317	7.41723	-0.162782	-0.241464	0.66665	0.99995	no

ENSG00000066933	ENSG00000066933	MYO9A	15:72110736-72447800	q1	q2	OK	6.03372	7.33549	0.281845	0.349688	0.3811	0.99995	no

ENSG00000067048	ENSG00000067048	DDX3Y	Y:15016018-15032390	q1	q2	OK	35.1965	37.6369	0.0967145	0.129754	0.7788	0.99995	no

ENSG00000067057	ENSG00000067057	PFKP	10:3108524-3179904	q1	q2	OK	6.00966	5.65312	-0.0882367	-0.133292	0.8281	0.99995	no

ENSG00000067064	ENSG00000067064	IDI1	10:1034337-1095110	q1	q2	OK	129.144	158.504	0.295539	0.551212	0.39765	0.99995	no

ENSG00000067066	ENSG00000067066	SP100	2:231280656-231444721	q1	q2	OK	3.03166	1.43577	-1.07829	-0.76459	0.1324	0.99995	no

ENSG00000067082	ENSG00000067082	KLF6	10:3818187-3827473	q1	q2	OK	26.4208	27.8214	0.0745211	0.125403	0.80695	0.99995	no

ENSG00000067113	ENSG00000067113	PPAP2A	5:54603587-54830878	q1	q2	OK	4.81782	5.61114	0.219914	0.0985454	0.8833	0.99995	no

ENSG00000067141	ENSG00000067141	NEO1	15:73344050-73597547	q1	q2	OK	78.1728	72.6183	-0.106335	-0.168803	0.743	0.99995	no

ENSG00000067167	ENSG00000067167	TRAM1	8:71485676-71520622	q1	q2	OK	48.848	50.8686	0.058475	0.139074	0.83405	0.99995	no

ENSG00000067177	ENSG00000067177	PHKA1	X:71798663-71934167	q1	q2	NOTEST	0	0.0107392	inf	0	1	1	no

ENSG00000067182	ENSG00000067182	TNFRSF1A	12:6437922-6451280	q1	q2	OK	29.4927	27.3093	-0.110965	-0.229982	0.7182	0.99995	no

ENSG00000067191	ENSG00000067191	CACNB1	17:37329542-37353956	q1	q2	OK	5.63191	5.84083	0.0525506	0.053033	0.8984	0.99995	no

ENSG00000067208	ENSG00000067208	EVI5	1:92974252-93257961	q1	q2	OK	6.57828	7.62776	0.213549	0.232223	0.647	0.99995	no

ENSG00000067221	ENSG00000067221	STOML1	15:74275546-74286963	q1	q2	OK	4.70653	4.52737	-0.055992	-0.0705581	0.91085	0.99995	no

ENSG00000067225	ENSG00000067225	PKM	15:72491369-72524164	q1	q2	OK	488.792	481.951	-0.0203332	-0.0375194	0.95225	0.99995	no

ENSG00000067248	ENSG00000067248	DHX29	5:54529761-54603550	q1	q2	OK	14.1457	15.43	0.125368	0.275141	0.6659	0.99995	no

ENSG00000067334	ENSG00000067334	DNTTIP2	1:94333372-94345474	q1	q2	OK	33.212	35.7288	0.105386	0.21304	0.7266	0.99995	no

ENSG00000067365	ENSG00000067365	METTL22	16:8715539-8740081	q1	q2	OK	21.3906	20.958	-0.0294779	-0.0408412	0.94245	0.99995	no

ENSG00000067369	ENSG00000067369	TP53BP1	15:43699406-43802926	q1	q2	OK	49.7914	47.8941	-0.0560493	-0.0735637	0.85705	0.99995	no

ENSG00000067445	ENSG00000067445	TRO	X:54946894-54957864	q1	q2	OK	72.793	67.1874	-0.115608	-0.217946	0.6785	0.99995	no

ENSG00000067533	ENSG00000067533	RRP15	1:218458628-218511325	q1	q2	OK	6.84823	10.797	0.656827	0.57668	0.22275	0.99995	no

ENSG00000067560	ENSG00000067560	RHOA	3:49396577-49453908	q1	q2	OK	253.434	271.202	0.0977565	0.221779	0.75215	0.99995	no

ENSG00000067596	ENSG00000067596	DHX8	17:41561232-41687706	q1	q2	OK	22.3065	21.3513	-0.0631383	-0.0455108	0.911	0.99995	no

ENSG00000067601	ENSG00000067601	PMS2P4	7:66752527-66786513	q1	q2	OK	3.91868	2.7703	-0.500325	-0.239256	0.824	0.99995	no

ENSG00000067606	ENSG00000067606	PRKCZ	1:1981908-2152177	q1	q2	OK	9.57478	8.09423	-0.242345	-0.240946	0.6509	0.99995	no

ENSG00000067646	ENSG00000067646	ZFY	Y:2803111-2870667	q1	q2	OK	7.7025	7.75621	0.0100264	0.0163374	0.97545	0.99995	no

ENSG00000067704	ENSG00000067704	IARS2	1:220267443-220321380	q1	q2	OK	33.4067	34.2186	0.0346442	0.0808124	0.9001	0.99995	no

ENSG00000067715	ENSG00000067715	SYT1	12:79257772-79897176	q1	q2	OK	42.8403	43.8934	0.0350347	0.0769144	0.8996	0.99995	no

ENSG00000067798	ENSG00000067798	NAV3	12:78224684-78606790	q1	q2	OK	3.13602	3.67542	0.228976	0.251096	0.5703	0.99995	no

ENSG00000067829	ENSG00000067829	IDH3G	X:153051220-153063960	q1	q2	OK	1.11024	5.75522	2.374	0.826464	0.1921	0.99995	no

ENSG00000067836	ENSG00000067836	ROGDI	16:4846968-4852951	q1	q2	OK	10.408	8.91229	-0.223828	-0.352077	0.57165	0.99995	no

ENSG00000067840	ENSG00000067840	PDZD4	X:153067620-153096020	q1	q2	OK	4.27179	0.765716	-2.47996	-1.94611	0.00575	0.99995	no

ENSG00000067842	ENSG00000067842	ATP2B3	X:152783133-152848397	q1	q2	NOTEST	0	0	0	0	1	1	no

ENSG00000067900	ENSG00000067900	ROCK1	18:18526866-18691812	q1	q2	OK	15.1132	15.4121	0.0282499	0.0307393	0.9276	0.99995	no

ENSG00000067955	ENSG00000067955	CBFB	16:67063018-67134961	q1	q2	OK	27.0442	31.829	0.235022	0.463066	0.4269	0.99995	no

ENSG00000067992	ENSG00000067992	PDK3	X:24483337-24557954	q1	q2	OK	3.50977	2.91303	-0.268858	-0.360588	0.6047	0.99995	no

ENSG00000068001	ENSG00000068001	HYAL2	3:50355220-50365682	q1	q2	OK	0.65281	0.128525	-2.34461	-0.671963	0.03745	0.99995	no

ENSG00000068024	ENSG00000068024	HDAC4	2:239969863-240324058	q1	q2	OK	9.1615	6.18774	-0.566172	-0.347664	0.3032	0.99995	no

ENSG00000068028	ENSG00000068028	RASSF1	3:50367218-50378411	q1	q2	OK	5.12461	4.92291	-0.0579298	-0.0860276	0.9019	0.99995	no

ENSG00000068078	ENSG00000068078	FGFR3	4:1795033-1810599	q1	q2	OK	7.30488	5.50216	-0.408863	-0.537129	0.2829	0.99995	no

ENSG00000068079	ENSG00000068079	IFI35	17:41158741-41166473	q1	q2	OK	0.535544	0.33599	-0.672587	-0.271331	0.4372	0.99995	no

ENSG00000068097	ENSG00000068097	HEATR6	17:58120554-58156292	q1	q2	OK	11.7353	11.2478	-0.06121	-0.0919689	0.8551	0.99995	no

ENSG00000068120	ENSG00000068120	COASY	17:40688189-40718295	q1	q2	OK	20.9879	18.7078	-0.165918	-0.216297	0.68175	0.99995	no

ENSG00000068137	ENSG00000068137	PLEKHH3	17:40819931-40829048	q1	q2	OK	7.27871	5.05742	-0.525283	-0.698978	0.25835	0.99995	no

ENSG00000068305	ENSG00000068305	MEF2A	15:100017369-100273766	q1	q2	OK	14.1576	14.6842	0.0526883	0.0741645	0.87835	0.99995	no

ENSG00000068308	ENSG00000068308	OTUD5	X:48779304-48815648	q1	q2	NOTEST	0.00955243	0.0254037	1.4111	0	1	1	no

ENSG00000068323	ENSG00000068323	TFE3	X:48886241-48901012	q1	q2	OK	4.10316	0.740239	-2.47067	-2.32616	0.0027	0.99995	no

ENSG00000068354	ENSG00000068354	TBC1D25	X:48397844-48420997	q1	q2	NOTEST	0	0	0	0	1	1	no

ENSG00000068366	ENSG00000068366	ACSL4	X:108866928-108976632	q1	q2	OK	10.3186	12.6166	0.290069	0.550329	0.32615	0.99995	no

ENSG00000068383	ENSG00000068383	INPP5A	10:134351323-134598269	q1	q2	OK	13.7469	13.5448	-0.0213651	-0.0419566	0.94575	0.99995	no

ENSG00000068394	ENSG00000068394	GPKOW	X:48970333-48980151	q1	q2	OK	0.143369	2.93459	4.35535	3.89441	0.0021	0.99995	no

ENSG00000068400	ENSG00000068400	GRIPAP1	X:48830133-48858675	q1	q2	OK	5.06144	5.1129	0.0145951	0.0203493	0.97505	0.99995	no

ENSG00000068438	ENSG00000068438	FTSJ1	X:48334540-48344752	q1	q2	NOTEST	0	0	0	0	1	1	no

ENSG00000068489	ENSG00000068489	PRR11	17:57232859-57292608	q1	q2	OK	36.8733	37.0924	0.00854639	0.0158128	0.9811	0.99995	no

ENSG00000068615	ENSG00000068615	REEP1	2:86441115-86565206	q1	q2	OK	8.12622	7.63787	-0.0894144	-0.16678	0.78525	0.99995	no

ENSG00000068650	ENSG00000068650	ATP11A	13:113344642-113541482	q1	q2	OK	10.8146	12.3579	0.19246	0.206766	0.54485	0.99995	no

ENSG00000068654	ENSG00000068654	POLR1A	2:86247338-86333278	q1	q2	OK	9.2166	9.64523	0.0655804	0.0768851	0.8247	0.99995	no

ENSG00000068697	ENSG00000068697	LAPTM4A	2:20232410-20251789	q1	q2	OK	97.3547	97.1247	-0.00341236	-0.00766349	0.99075	0.99995	no

ENSG00000068724	ENSG00000068724	TTC7A	2:47126413-47403740	q1	q2	OK	3.17202	3.05358	-0.0549002	-0.0132725	0.9507	0.99995	no

ENSG00000068745	ENSG00000068745	IP6K2	3:48725435-48777786	q1	q2	OK	113.52	103.692	-0.130649	-0.283002	0.6677	0.99995	no

ENSG00000068781	ENSG00000068781	STON1-GTF2A1L	2:48756521-49003654	q1	q2	NOTEST	0.415967	0.0113924	-5.19033	0	1	1	no

ENSG00000068784	ENSG00000068784	SRBD1	2:45615818-45839304	q1	q2	OK	10.7986	11.7519	0.122047	0.232662	0.71385	0.99995	no

ENSG00000068793	ENSG00000068793	CYFIP1	15:22892004-23034427	q1	q2	OK	42.665	41.3764	-0.044245	-0.0651865	0.8871	0.99995	no

ENSG00000068796	ENSG00000068796	KIF2A	5:61601988-61924409	q1	q2	OK	46.1094	50.2532	0.124154	0.18628	0.77535	0.99995	no

ENSG00000068831	ENSG00000068831	RASGRP2	11:64494382-64512928	q1	q2	OK	4.03683	4.17798	0.0495818	0.050242	0.91845	0.99995	no

ENSG00000068878	ENSG00000068878	PSME4	2:54091203-54532437	q1	q2	OK	37.286	39.7338	0.0917329	0.148419	0.8003	0.99995	no

ENSG00000068885	ENSG00000068885	IFT80	3:159943422-160203561	q1	q2	OK	28.0168	28.5814	0.0287857	0.0162752	0.9629	0.99995	no

ENSG00000068903	ENSG00000068903	SIRT2	19:39369196-39399533	q1	q2	OK	15.9843	14.0806	-0.182946	-0.265252	0.63555	0.99995	no

ENSG00000068912	ENSG00000068912	ERLEC1	2:53759809-54087297	q1	q2	OK	24.3268	23.5188	-0.0487321	-0.0670639	0.90985	0.99995	no

ENSG00000068971	ENSG00000068971	PPP2R5B	11:64685024-64703360	q1	q2	OK	7.21192	7.11576	-0.0193653	-0.0236771	0.9629	0.99995	no

ENSG00000068976	ENSG00000068976	PYGM	11:64513860-64527769	q1	q2	OK	0.532995	0.521663	-0.031005	-0.0168753	0.97485	0.99995	no

ENSG00000068985	ENSG00000068985	PAGE1	X:49452052-49460596	q1	q2	NOTEST	0	0	0	0	1	1	no

ENSG00000069011	ENSG00000069011	PITX1	5:134363424-134736986	q1	q2	NOTEST	0.0226635	0.0447014	0.979954	0	1	1	no

ENSG00000069018	ENSG00000069018	TRPC7	5:135712035-135732730	q1	q2	NOTEST	0.0384655	0	#NAME?	0	1	1	no

ENSG00000069020	ENSG00000069020	MAST4	5:65892175-66465423	q1	q2	OK	3.30632	3.29611	-0.00446231	-0.0043901	0.98905	0.99995	no

ENSG00000069122	ENSG00000069122	GPR116	6:46820248-46922680	q1	q2	OK	0.464019	0.680927	0.553316	0.523882	0.37695	0.99995	no

ENSG00000069188	ENSG00000069188	SDK2	17:71330522-71640228	q1	q2	OK	10.9056	9.02691	-0.27276	-0.637055	0.3426	0.99995	no

ENSG00000069206	ENSG00000069206	ADAM7	8:24151552-24769586	q1	q2	NOTEST	0	0.021797	inf	0	1	1	no

ENSG00000069248	ENSG00000069248	NUP133	1:229577044-229644103	q1	q2	OK	24.9732	28.4608	0.188598	0.371048	0.5242	0.99995	no

ENSG00000069275	ENSG00000069275	NUCKS1	1:205681946-205719404	q1	q2	OK	143.588	156.727	0.126311	0.0838923	0.72715	0.99995	no

ENSG00000069329	ENSG00000069329	VPS35	16:46690053-46723430	q1	q2	OK	55.1695	60.8694	0.141845	0.22343	0.63855	0.99995	no

ENSG00000069345	ENSG00000069345	DNAJA2	16:46989298-47007699	q1	q2	OK	36.9128	40.7374	0.142232	0.325475	0.64265	0.99995	no

ENSG00000069399	ENSG00000069399	BCL3	19:45250961-45263301	q1	q2	OK	2.19953	1.98176	-0.150412	-0.121642	0.8262	0.99995	no

ENSG00000069424	ENSG00000069424	KCNAB2	1:5922870-6161253	q1	q2	OK	2.77994	2.35599	-0.238717	-0.138751	0.6875	0.99995	no

ENSG00000069431	ENSG00000069431	ABCC9	12:21950334-22094336	q1	q2	OK	4.29492	4.10121	-0.0665839	-0.115841	0.86005	0.99995	no

ENSG00000069482	ENSG00000069482	GAL	11:68451246-68458643	q1	q2	NOTEST	0.236955	0.229975	-0.0431369	0	1	1	no

ENSG00000069493	ENSG00000069493	CLEC2D	12:9817564-9848823	q1	q2	OK	11.6624	13.6738	0.229551	0.297078	0.61955	0.99995	no

ENSG00000069509	ENSG00000069509	FUNDC1	X:44382884-44402247	q1	q2	OK	17.767	18.9517	0.0931253	0.170636	0.80905	0.99995	no

ENSG00000069535	ENSG00000069535	MAOB	X:43625857-43741693	q1	q2	NOTEST	0.157062	0.136506	-0.20237	0	1	1	no

ENSG00000069667	ENSG00000069667	RORA	15:60771376-61521518	q1	q2	OK	2.34927	2.72546	0.214289	0.116193	0.73955	0.99995	no

ENSG00000069696	ENSG00000069696	DRD4	11:637292-640706	q1	q2	NOTEST	0.457695	0.467728	0.0312821	0	1	1	no

ENSG00000069702	ENSG00000069702	TGFBR3	1:92145901-92371892	q1	q2	OK	3.35714	2.59364	-0.372253	-0.574944	0.2999	0.99995	no

ENSG00000069712	ENSG00000069712	KIAA1107	1:92632541-92650280	q1	q2	OK	1.25737	1.19954	-0.0679234	-0.0934725	0.89615	0.99995	no

ENSG00000069764	ENSG00000069764	PLA2G10	16:14766404-14788526	q1	q2	NOTEST	0.0297767	0.030867	0.0518816	0	1	1	no

ENSG00000069812	ENSG00000069812	HES2	1:6472477-6484730	q1	q2	NOTEST	0.316479	0.0846603	-1.90235	0	1	1	no

ENSG00000069849	ENSG00000069849	ATP1B3	3:141594965-141645356	q1	q2	OK	90.4514	85.8578	-0.0751937	-0.147628	0.822	0.99995	no

ENSG00000069869	ENSG00000069869	NEDD4	15:56119119-56285944	q1	q2	OK	6.01228	7.43244	0.305923	0.62906	0.3236	0.99995	no

ENSG00000069943	ENSG00000069943	PIGB	15:55495163-55800432	q1	q2	OK	5.56473	4.66479	-0.2545	-0.140915	0.7186	0.99995	no

ENSG00000069956	ENSG00000069956	MAPK6	15:52121824-52392720	q1	q2	OK	45.6075	52.7898	0.210987	0.354057	0.57535	0.99995	no

ENSG00000069966	ENSG00000069966	GNB5	15:52408770-52587995	q1	q2	OK	8.53073	7.53695	-0.178688	-0.254606	0.68425	0.99995	no

ENSG00000069974	ENSG00000069974	RAB27A	15:55495163-55800432	q1	q2	OK	5.49697	4.42768	-0.312085	-0.233236	0.6584	0.99995	no

ENSG00000069998	ENSG00000069998	CECR5	22:17618400-17646335	q1	q2	OK	20.3049	20.4616	0.0110852	0.0227501	0.9738	0.99995	no

ENSG00000070010	ENSG00000070010	UFD1L	22:19318220-19466738	q1	q2	OK	49.524	49.8275	0.00881447	0.00881802	0.9873	0.99995	no

ENSG00000070018	ENSG00000070018	LRP6	12:12202777-12419946	q1	q2	OK	19.5568	20.8516	0.0924846	0.115203	0.76845	0.99995	no

ENSG00000070019	ENSG00000070019	GUCY2C	12:14656594-14910897	q1	q2	NOTEST	0	0	0	0	1	1	no

ENSG00000070031	ENSG00000070031	SCT	11:626430-627143	q1	q2	NOTEST	0	0	0	0	1	1	no

ENSG00000070047	ENSG00000070047	PHRF1	11:576485-612222	q1	q2	OK	12.5128	12.6343	0.0139485	0.0316543	0.9597	0.99995	no

ENSG00000070061	ENSG00000070061	IKBKAP	9:111629796-111696396	q1	q2	OK	20.1618	22.4305	0.153834	0.312643	0.6124	0.99995	no

ENSG00000070081	ENSG00000070081	NUCB2	11:17229699-17371521	q1	q2	OK	38.1871	34.4101	-0.150254	-0.250808	0.71195	0.99995	no

ENSG00000070087	ENSG00000070087	PFN2	3:149478891-149942977	q1	q2	OK	198.92	200.389	0.0106172	0.021741	0.9748	0.99995	no

ENSG00000070159	ENSG00000070159	PTPN3	9:112137745-112260590	q1	q2	OK	0.751546	1.02746	0.451143	0.408745	0.48935	0.99995	no

ENSG00000070182	ENSG00000070182	SPTB	14:65170819-65346601	q1	q2	OK	1.29551	1.16801	-0.149476	-0.0752217	0.8831	0.99995	no

ENSG00000070190	ENSG00000070190	DAPP1	4:100737989-100791311	q1	q2	NOTEST	0.020192	0.0187054	-0.11033	0	1	1	no

ENSG00000070193	ENSG00000070193	FGF10	5:44303645-44414091	q1	q2	OK	3.83773	3.20495	-0.259952	-0.338419	0.6131	0.99995	no

ENSG00000070214	ENSG00000070214	SLC44A1	9:108006902-108201452	q1	q2	OK	8.04527	6.86475	-0.228934	-0.385567	0.51535	0.99995	no

ENSG00000070269	ENSG00000070269	TMEM260	14:56955071-57197253	q1	q2	OK	8.32385	8.27974	-0.00766545	-0.0113964	0.9825	0.99995	no

ENSG00000070366	ENSG00000070366	SMG6	17:1963132-2284352	q1	q2	OK	23.7603	21.5563	-0.140445	-0.121147	0.82395	0.99995	no

ENSG00000070367	ENSG00000070367	EXOC5	14:57670517-57756797	q1	q2	OK	45.6379	50.9002	0.157441	0.260526	0.65675	0.99995	no

ENSG00000070371	ENSG00000070371	CLTCL1	22:19166985-19279239	q1	q2	OK	0.366133	0.789456	1.10849	0.67612	0.27375	0.99995	no

ENSG00000070388	ENSG00000070388	FGF22	19:639878-643703	q1	q2	NOTEST	0.226605	0.350628	0.629761	0	1	1	no

ENSG00000070404	ENSG00000070404	FSTL3	19:676391-683385	q1	q2	OK	5.2639	3.74146	-0.492532	-0.592104	0.2888	0.99995	no

ENSG00000070413	ENSG00000070413	DGCR2	22:19023794-19115962	q1	q2	OK	34.8705	31.5962	-0.142254	-0.309992	0.63055	0.99995	no

ENSG00000070423	ENSG00000070423	RNF126	19:647525-663277	q1	q2	OK	23.3967	20.3434	-0.201743	-0.355897	0.58285	0.99995	no

ENSG00000070444	ENSG00000070444	MNT	17:2287353-2304412	q1	q2	OK	10.5691	8.29755	-0.349098	-0.487482	0.33465	0.99995	no

ENSG00000070476	ENSG00000070476	ZXDC	3:126156443-126194762	q1	q2	OK	14.6412	13.9152	-0.0733768	-0.153241	0.8319	0.99995	no

ENSG00000070495	ENSG00000070495	JMJD6	17:74708918-74722866	q1	q2	OK	15.4958	15.4745	-0.00198056	-0.00300608	0.9965	0.99995	no

ENSG00000070501	ENSG00000070501	POLB	8:42195971-42229326	q1	q2	OK	28.2898	33.2965	0.235087	0.410393	0.55095	0.99995	no

ENSG00000070526	ENSG00000070526	ST6GALNAC1	17:74620842-74639920	q1	q2	NOTEST	0	0.107594	inf	0	1	1	no

ENSG00000070540	ENSG00000070540	WIPI1	17:66255322-66760023	q1	q2	OK	8.84236	9.15269	0.0497653	0.0189986	0.966	0.99995	no

ENSG00000070601	ENSG00000070601	FRMPD1	9:37510888-38069210	q1	q2	NOTEST	0.0814566	0.0816855	0.0040484	0	1	1	no

ENSG00000070610	ENSG00000070610	GBA2	9:35696944-35758572	q1	q2	OK	48.9752	47.1264	-0.0555161	-0.0449581	0.93295	0.99995	no

ENSG00000070614	ENSG00000070614	NDST1	5:149855093-149937773	q1	q2	OK	33.3158	35.645	0.0974913	0.158008	0.72915	0.99995	no

ENSG00000070669	ENSG00000070669	ASNS	7:97481429-97501854	q1	q2	OK	105.712	107.875	0.0292234	0.0661189	0.91455	0.99995	no

ENSG00000070718	ENSG00000070718	AP3M2	8:42010463-42029191	q1	q2	OK	15.0637	13.3497	-0.174277	-0.272814	0.58385	0.99995	no

ENSG00000070729	ENSG00000070729	CNGB1	16:57917502-58005020	q1	q2	OK	0.676721	0.435229	-0.636785	-0.363917	0.5083	0.99995	no

ENSG00000070731	ENSG00000070731	ST6GALNAC2	17:74553847-74583038	q1	q2	NOTEST	0.139579	0.123279	-0.179154	0	1	1	no

ENSG00000070748	ENSG00000070748	CHAT	10:50817140-50918307	q1	q2	OK	0.31335	0.642155	1.03514	0.344772	0.42475	0.99995	no

ENSG00000070756	ENSG00000070756	PABPC1	8:101698043-101735037	q1	q2	OK	431.575	414.3	-0.0589372	-0.0984056	0.88155	0.99995	no

ENSG00000070759	ENSG00000070759	TESK2	1:45794834-45956872	q1	q2	OK	0.862736	0.998949	0.211493	0.083326	0.86885	0.99995	no

ENSG00000070761	ENSG00000070761	C16orf80	16:58147495-58163354	q1	q2	OK	48.2385	49.778	0.0453217	0.0899312	0.8806	0.99995	no

ENSG00000070770	ENSG00000070770	CSNK2A2	16:58163432-58251709	q1	q2	OK	32.6717	34.1147	0.0623525	0.121439	0.8525	0.99995	no

ENSG00000070778	ENSG00000070778	PTPN21	14:88851267-89021077	q1	q2	OK	10.2812	11.284	0.134272	0.236571	0.67155	0.99995	no

ENSG00000070785	ENSG00000070785	EIF2B3	1:45316449-45452282	q1	q2	OK	17.5214	18.3404	0.065909	0.128059	0.8469	0.99995	no

ENSG00000070808	ENSG00000070808	CAMK2A	5:149569519-149669854	q1	q2	OK	1.32935	1.38044	0.0544106	0.0627989	0.90955	0.99995	no

ENSG00000070814	ENSG00000070814	TCOF1	5:149737201-149779871	q1	q2	OK	21.4862	21.9378	0.0300093	0.0605003	0.9166	0.99995	no

ENSG00000070831	ENSG00000070831	CDC42	1:22379119-22419437	q1	q2	OK	145.394	156.313	0.104463	0.249626	0.7256	0.99995	no

ENSG00000070882	ENSG00000070882	OSBPL3	7:24836157-25021253	q1	q2	OK	13.8592	15.522	0.163468	0.200113	0.60775	0.99995	no

ENSG00000070886	ENSG00000070886	EPHA8	1:22890056-22930087	q1	q2	NOTEST	0.0354367	0.0344959	-0.0388203	0	1	1	no

ENSG00000070915	ENSG00000070915	SLC12A3	16:56899118-56949762	q1	q2	NOTEST	0	0	0	0	1	1	no

ENSG00000070950	ENSG00000070950	RAD18	3:8661085-9005457	q1	q2	OK	6.70692	7.71072	0.201216	0.285659	0.5668	0.99995	no

ENSG00000070961	ENSG00000070961	ATP2B1	12:89954905-90106367	q1	q2	OK	26.9451	30.8338	0.19449	0.307066	0.53505	0.99995	no

ENSG00000070985	ENSG00000070985	TRPM5	11:2425744-2444275	q1	q2	NOTEST	0	0.00687835	inf	0	1	1	no

ENSG00000071051	ENSG00000071051	NCK2	2:106361353-106510730	q1	q2	OK	48.4653	48.3614	-0.00309632	-0.00672157	0.99215	0.99995	no

ENSG00000071054	ENSG00000071054	MAP4K4	2:102313311-102511150	q1	q2	OK	118.131	119.999	0.0226279	0.0339178	0.9436	0.99995	no

ENSG00000071073	ENSG00000071073	MGAT4A	2:99235568-99347589	q1	q2	OK	5.8893	7.07125	0.26387	0.273275	0.54885	0.99995	no

ENSG00000071082	ENSG00000071082	RPL31	2:101436613-101886778	q1	q2	OK	975.858	982.618	0.00995947	0.0173196	0.97905	0.99995	no

ENSG00000071127	ENSG00000071127	WDR1	4:10075962-10118573	q1	q2	OK	68.4051	72.8173	0.0901769	0.197568	0.7662	0.99995	no

ENSG00000071189	ENSG00000071189	SNX13	7:17830384-17980124	q1	q2	OK	11.2985	10.4097	-0.118196	-0.22462	0.70715	0.99995	no

ENSG00000071203	ENSG00000071203	MS4A12	11:60260250-60274903	q1	q2	NOTEST	0	0	0	0	1	1	no

ENSG00000071205	ENSG00000071205	ARHGAP10	4:148653213-148993931	q1	q2	OK	3.4033	2.91807	-0.221919	-0.298359	0.6642	0.99995	no

ENSG00000071242	ENSG00000071242	RPS6KA2	6:166822851-167370679	q1	q2	OK	27.0019	25.2415	-0.0972637	-0.203576	0.7327	0.99995	no

ENSG00000071243	ENSG00000071243	ING3	7:120590802-120617270	q1	q2	OK	21.5688	23.2142	0.106068	0.208083	0.77025	0.99995	no

ENSG00000071246	ENSG00000071246	VASH1	14:77228531-77253067	q1	q2	OK	25.2316	25.9434	0.0401362	0.0728268	0.8917	0.99995	no

ENSG00000071282	ENSG00000071282	LMCD1	3:7994491-8653610	q1	q2	OK	2.90766	2.8141	-0.0471873	-0.0572407	0.9319	0.99995	no

ENSG00000071462	ENSG00000071462	WBSCR22	7:73095298-73134002	q1	q2	OK	2.4215	25.1498	3.37657	3.56553	5.00E-05	0.0795045	no

ENSG00000071537	ENSG00000071537	SEL1L	14:81937892-82000205	q1	q2	OK	19.2371	20.0604	0.0604643	0.115081	0.819	0.99995	no

ENSG00000071539	ENSG00000071539	TRIP13	5:710470-919472	q1	q2	OK	16.7668	20.1718	0.266732	0.261537	0.58285	0.99995	no

ENSG00000071553	ENSG00000071553	ATP6AP1	X:153656977-153664862	q1	q2	OK	2.74161	8.66236	1.65973	1.41099	0.0305	0.99995	no

ENSG00000071564	ENSG00000071564	TCF3	19:1609290-1652604	q1	q2	OK	110.676	110.781	0.00137011	0.0024368	0.99735	0.99995	no

ENSG00000071575	ENSG00000071575	TRIB2	2:12857014-12882860	q1	q2	OK	208.104	218.747	0.0719546	0.135977	0.82465	0.99995	no

ENSG00000071626	ENSG00000071626	DAZAP1	19:1407567-1435719	q1	q2	OK	60.7937	51.6018	-0.236499	-0.40097	0.4836	0.99995	no

ENSG00000071655	ENSG00000071655	MBD3	19:1576638-1605480	q1	q2	OK	66.5342	58.9437	-0.174758	-0.325807	0.597	0.99995	no

ENSG00000071677	ENSG00000071677	PRLH	2:238475216-238475818	q1	q2	NOTEST	0	0	0	0	1	1	no

ENSG00000071794	ENSG00000071794	HLTF	3:148747913-148820610	q1	q2	OK	24.7615	29.4874	0.252002	0.425966	0.388	0.99995	no

ENSG00000071859	ENSG00000071859	FAM50A	X:153672472-153679002	q1	q2	OK	6.33938	1.13929	-2.47621	-2.45285	0.00045	0.414261	no

ENSG00000071889	ENSG00000071889	FAM3A	X:153733349-153744566	q1	q2	OK	2.9612	2.98617	0.0121147	0.0145484	0.9819	0.99995	no

ENSG00000071894	ENSG00000071894	CPSF1	8:145596789-145634753	q1	q2	OK	1.18377	1.24065	0.0677107	0.0431379	0.9346	0.99995	no

ENSG00000071909	ENSG00000071909	MYO3B	2:171034654-171551884	q1	q2	NOTEST	0.0532257	0.0979207	0.879489	0	1	1	no

ENSG00000071967	ENSG00000071967	CYBRD1	2:172378756-172414643	q1	q2	OK	6.23468	5.68714	-0.132611	-0.224261	0.7082	0.99995	no

ENSG00000071991	ENSG00000071991	CDH19	18:64168319-64271375	q1	q2	NOTEST	0.0243367	0.0252128	0.0510205	0	1	1	no

ENSG00000071994	ENSG00000071994	PDCD2	6:170884382-170893780	q1	q2	OK	44.6068	48.1312	0.109707	0.21245	0.76285	0.99995	no

ENSG00000072041	ENSG00000072041	SLC6A15	12:85253491-85307394	q1	q2	OK	2.90877	2.46352	-0.239691	-0.317481	0.5649	0.99995	no

ENSG00000072042	ENSG00000072042	RDH11	14:68086514-68162531	q1	q2	OK	53.0814	56.5157	0.0904435	0.145182	0.83015	0.99995	no

ENSG00000072062	ENSG00000072062	PRKACA	19:14202499-14228896	q1	q2	OK	41.0777	41.5228	0.0155495	0.0341402	0.9577	0.99995	no

ENSG00000072071	ENSG00000072071	LPHN1	19:14247990-14316999	q1	q2	OK	14.767	10.9494	-0.431528	-0.494802	0.28035	0.99995	no

ENSG00000072080	ENSG00000072080	SPP2	2:234959322-234985778	q1	q2	NOTEST	0	0.129646	inf	0	1	1	no

ENSG00000072110	ENSG00000072110	ACTN1	14:69340859-69446157	q1	q2	OK	38.5634	42.8837	0.153198	0.324743	0.5867	0.99995	no

ENSG00000072121	ENSG00000072121	ZFYVE26	14:68168602-68283307	q1	q2	OK	6.75656	6.67054	-0.0184839	-0.0235143	0.9595	0.99995	no

ENSG00000072133	ENSG00000072133	RPS6KA6	X:83318983-83442933	q1	q2	OK	3.53587	4.00568	0.17998	0.25453	0.68675	0.99995	no

ENSG00000072134	ENSG00000072134	EPN2	17:19118927-19240028	q1	q2	OK	18.7646	21.7801	0.214997	0.361084	0.45655	0.99995	no

ENSG00000072135	ENSG00000072135	PTPN18	2:131113579-131132982	q1	q2	OK	7.27222	6.93332	-0.06885	-0.076605	0.87525	0.99995	no

ENSG00000072163	ENSG00000072163	LIMS2	2:128395955-128439360	q1	q2	OK	2.52903	1.73534	-0.543362	-0.497336	0.47655	0.99995	no

ENSG00000072182	ENSG00000072182	ASIC4	2:220299567-220403494	q1	q2	OK	0.868014	1.04534	0.268177	0.126107	0.8372	0.99995	no

ENSG00000072195	ENSG00000072195	SPEG	2:220299567-220403494	q1	q2	OK	18.6881	15.3456	-0.284294	-0.31801	0.5697	0.99995	no

ENSG00000072201	ENSG00000072201	LNX1	4:54243809-55164414	q1	q2	OK	0.742282	0.779966	0.0714442	0.0250639	0.9721	0.99995	no

ENSG00000072210	ENSG00000072210	ALDH3A2	17:19551448-19580911	q1	q2	OK	14.1842	16.1623	0.188343	0.333671	0.5968	0.99995	no

ENSG00000072274	ENSG00000072274	TFRC	3:195754053-195809060	q1	q2	OK	25.1694	23.7105	-0.0861474	-0.140086	0.75265	0.99995	no

ENSG00000072310	ENSG00000072310	SREBF1	17:17584786-17740325	q1	q2	OK	25.9128	26.8056	0.0488722	0.0399458	0.9061	0.99995	no

ENSG00000072315	ENSG00000072315	TRPC5	X:111017542-111326004	q1	q2	NOTEST	0	0.0378642	inf	0	1	1	no

ENSG00000072364	ENSG00000072364	AFF4	5:132211070-132299326	q1	q2	OK	26.5168	29.8753	0.172043	0.269472	0.53735	0.99995	no

ENSG00000072401	ENSG00000072401	UBE2D1	10:60094734-60130513	q1	q2	OK	22.0898	25.1886	0.189391	0.35623	0.60945	0.99995	no

ENSG00000072415	ENSG00000072415	MPP5	14:67707825-67853233	q1	q2	OK	27.9722	27.2843	-0.0359227	-0.0395105	0.93465	0.99995	no

ENSG00000072422	ENSG00000072422	RHOBTB1	10:62629195-62761198	q1	q2	OK	8.41766	8.21119	-0.0358268	-0.0472985	0.9161	0.99995	no

ENSG00000072444	ENSG00000072444	ASAH2C	10:48001602-48055018	q1	q2	NOTEST	0	0	0	0	1	1	no

ENSG00000072501	ENSG00000072501	SMC1A	X:53401069-53458059	q1	q2	OK	0.43097	1.09609	1.34671	0.788763	0.2292	0.99995	no

ENSG00000072506	ENSG00000072506	HSD17B10	X:53458205-53461320	q1	q2	NOTEST	0	0	0	0	1	1	no

ENSG00000072518	ENSG00000072518	MARK2	11:63606399-63678491	q1	q2	OK	11.6965	10.7764	-0.118194	-0.188402	0.7434	0.99995	no

ENSG00000072571	ENSG00000072571	HMMR	5:162887208-162921064	q1	q2	OK	33.4485	41.0495	0.295422	0.518055	0.36145	0.99995	no

ENSG00000072609	ENSG00000072609	CHFR	12:133345494-133532892	q1	q2	OK	16.3853	16.1309	-0.0225721	-0.0194543	0.96885	0.99995	no

ENSG00000072657	ENSG00000072657	TRHDE	12:72332625-73059422	q1	q2	NOTEST	0.215241	0.221975	0.0444431	0	1	1	no

ENSG00000072682	ENSG00000072682	P4HA2	5:131520568-131731306	q1	q2	OK	5.70315	4.69645	-0.28019	-0.295399	0.5664	0.99995	no

ENSG00000072694	ENSG00000072694	FCGR2B	1:161475219-161648444	q1	q2	NOTEST	0	0.0241136	inf	0	1	1	no

ENSG00000072736	ENSG00000072736	NFATC3	16:68021296-68272005	q1	q2	OK	17.7567	18.4426	0.0546804	0.0833848	0.8822	0.99995	no

ENSG00000072756	ENSG00000072756	TRNT1	3:3168599-3221394	q1	q2	OK	16.93	18.0257	0.0904767	0.11027	0.84495	0.99995	no

ENSG00000072778	ENSG00000072778	ACADVL	17:7093208-7128592	q1	q2	OK	105.7	88.095	-0.26284	-0.373187	0.55075	0.99995	no

ENSG00000072786	ENSG00000072786	STK10	5:171469076-171615390	q1	q2	OK	7.58498	8.93001	0.235517	0.317736	0.51245	0.99995	no

ENSG00000072803	ENSG00000072803	FBXW11	5:171288552-171433877	q1	q2	OK	33.0048	35.6448	0.111016	0.213549	0.7053	0.99995	no

ENSG00000072818	ENSG00000072818	ACAP1	17:7239847-7254797	q1	q2	OK	1.6173	0.558253	-1.5346	-0.663195	0.0633	0.99995	no

ENSG00000072832	ENSG00000072832	CRMP1	4:5712923-5894785	q1	q2	OK	152.287	151.384	-0.00857871	-0.0195312	0.97735	0.99995	no

ENSG00000072840	ENSG00000072840	EVC	4:5712923-5894785	q1	q2	OK	4.189	3.94126	-0.0879461	-0.0370471	0.9349	0.99995	no

ENSG00000072849	ENSG00000072849	DERL2	17:5374570-5394134	q1	q2	OK	37.2702	38.4613	0.0453854	0.0477551	0.93835	0.99995	no

ENSG00000072858	ENSG00000072858	SIDT1	3:113251142-113348425	q1	q2	OK	1.01124	0.913277	-0.147	-0.09108	0.8115	0.99995	no

ENSG00000072864	ENSG00000072864	NDE1	16:15737123-15950890	q1	q2	OK	27.3271	31.0873	0.185994	0.332055	0.6129	0.99995	no

ENSG00000072952	ENSG00000072952	MRVI1	11:10562818-10715535	q1	q2	OK	1.72094	1.91125	0.151322	0.167431	0.7007	0.99995	no

ENSG00000072954	ENSG00000072954	TMEM38A	19:16771937-16800840	q1	q2	OK	2.54645	2.23407	-0.188813	-0.182439	0.78705	0.99995	no

ENSG00000072958	ENSG00000072958	AP1M1	19:16308388-16346160	q1	q2	OK	39.7267	38.3523	-0.0507959	-0.0990569	0.8587	0.99995	no

ENSG00000073008	ENSG00000073008	PVR	19:45041044-45222031	q1	q2	OK	6.53505	6.74624	0.045884	0.0729918	0.9098	0.99995	no

ENSG00000073009	ENSG00000073009	IKBKG	X:153759605-153796782	q1	q2	NOTEST	0.111188	0.0614779	-0.854859	0	1	1	no

ENSG00000073050	ENSG00000073050	XRCC1	19:44047191-44100287	q1	q2	OK	25.574	21.3698	-0.259104	-0.324141	0.63455	0.99995	no

ENSG00000073060	ENSG00000073060	SCARB1	12:125261401-125367214	q1	q2	OK	12.1419	11.9413	-0.0240314	-0.034961	0.9587	0.99995	no

ENSG00000073067	ENSG00000073067	CYP2W1	7:1022834-1029276	q1	q2	NOTEST	0.0822421	0.0234438	-1.81067	0	1	1	no

ENSG00000073111	ENSG00000073111	MCM2	3:127291911-127341276	q1	q2	OK	64.709	64.4285	-0.00626676	-0.0135108	0.9822	0.99995	no

ENSG00000073146	ENSG00000073146	MOV10L1	22:50528307-50600119	q1	q2	OK	0.477368	0.514945	0.109317	0.0876782	0.8807	0.99995	no

ENSG00000073150	ENSG00000073150	PANX2	22:50609159-50618723	q1	q2	OK	2.32001	1.47707	-0.651397	-0.886529	0.2008	0.99995	no

ENSG00000073169	ENSG00000073169	SELO	22:50639407-50656045	q1	q2	OK	12.9617	10.7443	-0.270688	-0.478385	0.4973	0.99995	no

ENSG00000073282	ENSG00000073282	TP63	3:189349204-189615068	q1	q2	NOTEST	0.108039	0.0158305	-2.77077	0	1	1	no

ENSG00000073331	ENSG00000073331	ALPK1	4:113195697-113363776	q1	q2	OK	0.521795	0.684753	0.392102	0.166176	0.59075	0.99995	no

ENSG00000073350	ENSG00000073350	LLGL2	17:73521160-73571289	q1	q2	OK	0.692082	0.638127	-0.1171	-0.0601744	0.8513	0.99995	no

ENSG00000073417	ENSG00000073417	PDE8A	15:85523670-85682376	q1	q2	OK	14.0359	12.8989	-0.121867	-0.186401	0.7383	0.99995	no

ENSG00000073464	ENSG00000073464	CLCN4	X:10125023-10205700	q1	q2	OK	1.80661	1.78951	-0.0137173	-0.0218049	0.9737	0.99995	no

ENSG00000073536	ENSG00000073536	NLE1	17:33448597-33469334	q1	q2	OK	7.90666	7.06073	-0.163251	-0.242281	0.6848	0.99995	no

ENSG00000073578	ENSG00000073578	SDHA	5:218355-256815	q1	q2	OK	40.242	36.7013	-0.132871	-0.28929	0.64955	0.99995	no

ENSG00000073584	ENSG00000073584	SMARCE1	17:38781213-38821433	q1	q2	OK	124.717	137.217	0.137801	0.286003	0.64535	0.99995	no

ENSG00000073598	ENSG00000073598	FNDC8	17:33448597-33469334	q1	q2	NOTEST	0.0734486	0.0232514	-1.65942	0	1	1	no

ENSG00000073605	ENSG00000073605	GSDMB	17:38060847-38076107	q1	q2	OK	9.17001	9.48971	0.0494409	0.0673652	0.9149	0.99995	no

ENSG00000073614	ENSG00000073614	KDM5A	12:389294-551811	q1	q2	OK	12.9087	11.9055	-0.116712	-0.0938005	0.7615	0.99995	no

ENSG00000073670	ENSG00000073670	ADAM11	17:42836398-42859214	q1	q2	OK	0.83144	0.53506	-0.635912	-0.556233	0.2998	0.99995	no

ENSG00000073711	ENSG00000073711	PPP2R3A	3:135684514-135866733	q1	q2	OK	8.06351	7.35136	-0.133396	-0.253612	0.66505	0.99995	no

ENSG00000073712	ENSG00000073712	FERMT2	14:53323985-53419153	q1	q2	OK	46.4553	50.8852	0.131402	0.302924	0.6528	0.99995	no

ENSG00000073734	ENSG00000073734	ABCB11	2:169779447-169887832	q1	q2	NOTEST	0.0185909	0.0570922	1.6187	0	1	1	no

ENSG00000073737	ENSG00000073737	DHRS9	2:169921298-169952677	q1	q2	NOTEST	0	0	0	0	1	1	no

ENSG00000073754	ENSG00000073754	CD5L	1:157800703-157868046	q1	q2	NOTEST	0	0.0123882	inf	0	1	1	no

ENSG00000073756	ENSG00000073756	PTGS2	1:186640922-186649559	q1	q2	NOTEST	0.167387	0.0957496	-0.805851	0	1	1	no

ENSG00000073792	ENSG00000073792	IGF2BP2	3:185361526-185542844	q1	q2	OK	60.1694	59.719	-0.0108391	-0.0159583	0.97035	0.99995	no

ENSG00000073803	ENSG00000073803	MAP3K13	3:185000728-185216845	q1	q2	OK	14.5475	14.3535	-0.0193673	-0.022772	0.97325	0.99995	no

ENSG00000073849	ENSG00000073849	ST6GAL1	3:186648273-186796341	q1	q2	OK	12.0716	11.8963	-0.0211012	-0.03766	0.9398	0.99995	no

ENSG00000073861	ENSG00000073861	TBX21	17:45810609-45823485	q1	q2	NOTEST	0.0430734	0.0394894	-0.125332	0	1	1	no

ENSG00000073905	ENSG00000073905	VDAC1P1	X:80184998-80185846	q1	q2	OK	2.742	3.4254	0.321043	0.365859	0.5952	0.99995	no

ENSG00000073910	ENSG00000073910	FRY	13:32599450-32870794	q1	q2	OK	2.39813	2.15554	-0.15386	-0.105128	0.8322	0.99995	no

ENSG00000073921	ENSG00000073921	PICALM	11:85668726-85780924	q1	q2	OK	67.1395	72.3454	0.10774	0.193229	0.70965	0.99995	no

ENSG00000073969	ENSG00000073969	NSF	17:44668034-44834830	q1	q2	OK	15.4567	14.8978	-0.0531335	-0.0967105	0.87075	0.99995	no

ENSG00000074047	ENSG00000074047	GLI2	2:121493198-121750229	q1	q2	OK	22.8121	20.7059	-0.139758	-0.128988	0.70485	0.99995	no

ENSG00000074054	ENSG00000074054	CLASP1	2:122095351-122407163	q1	q2	OK	29.8673	29.7868	-0.00389179	-0.00783032	0.98995	0.99995	no

ENSG00000074071	ENSG00000074071	MRPS34	16:1821890-1823156	q1	q2	OK	48.3462	50.7084	0.0688228	0.145076	0.8392	0.99995	no

ENSG00000074181	ENSG00000074181	NOTCH3	19:15270443-15311792	q1	q2	OK	50.9979	49.8354	-0.0332664	-0.0540969	0.90865	0.99995	no

ENSG00000074201	ENSG00000074201	CLNS1A	11:77225980-77348850	q1	q2	OK	116.482	120.47	0.0485644	0.109062	0.8722	0.99995	no

ENSG00000074211	ENSG00000074211	PPP2R2C	4:6322304-6565327	q1	q2	NOTEST	0.279888	0.157282	-0.831499	0	1	1	no

ENSG00000074219	ENSG00000074219	TEAD2	19:49834873-49891338	q1	q2	OK	70.3877	70.1733	-0.00440078	-0.00741798	0.98865	0.99995	no

ENSG00000074266	ENSG00000074266	EED	11:85955585-85989855	q1	q2	OK	27.4418	26.6302	-0.0433137	-0.0863178	0.894	0.99995	no

ENSG00000074276	ENSG00000074276	CDHR2	5:175969511-176037134	q1	q2	OK	2.80922	2.1633	-0.376933	-0.101745	0.81795	0.99995	no

ENSG00000074317	ENSG00000074317	SNCB	5:176047084-176057530	q1	q2	NOTEST	0.0701229	0.193112	1.46148	0	1	1	no

ENSG00000074319	ENSG00000074319	TSG101	11:18477370-18548779	q1	q2	OK	48.8235	48.0247	-0.0237987	-0.0458052	0.9443	0.99995	no

ENSG00000074356	ENSG00000074356	C17orf85	17:3714459-3749545	q1	q2	OK	45.5859	48.4453	0.087769	0.0942408	0.81895	0.99995	no

ENSG00000074370	ENSG00000074370	ATP2A3	17:3827168-3867736	q1	q2	NOTEST	0.277849	0.268077	-0.0516567	0	1	1	no

ENSG00000074410	ENSG00000074410	CA12	15:63613576-63674360	q1	q2	OK	2.82903	2.12183	-0.414999	-0.517428	0.4127	0.99995	no

ENSG00000074416	ENSG00000074416	MGLL	3:127407908-127542051	q1	q2	OK	0.737516	0.298996	-1.30255	-0.465875	0.08815	0.99995	no

ENSG00000074527	ENSG00000074527	NTN4	12:96043030-96184930	q1	q2	OK	5.29533	4.71722	-0.166782	-0.26658	0.6689	0.99995	no

ENSG00000074582	ENSG00000074582	BCS1L	2:219502638-219528166	q1	q2	OK	25.4947	23.1456	-0.139455	-0.159749	0.8099	0.99995	no

ENSG00000074590	ENSG00000074590	NUAK1	12:106457117-106533811	q1	q2	OK	12.6187	11.7591	-0.101793	-0.188212	0.7233	0.99995	no

ENSG00000074603	ENSG00000074603	DPP8	15:65734800-65810042	q1	q2	OK	10.3331	10.1833	-0.0210652	-0.0255647	0.9536	0.99995	no

ENSG00000074621	ENSG00000074621	SLC24A1	15:65903703-66184329	q1	q2	OK	4.60004	3.85534	-0.254789	-0.0791086	0.80715	0.99995	no

ENSG00000074657	ENSG00000074657	ZNF532	18:56529831-56653712	q1	q2	OK	80.4759	90.804	0.1742	0.216947	0.5431	0.99995	no

ENSG00000074660	ENSG00000074660	SCARF1	17:1537151-1549041	q1	q2	OK	0.652089	0.693218	0.0882388	0.0834312	0.8873	0.99995	no

ENSG00000074695	ENSG00000074695	LMAN1	18:56995054-57027194	q1	q2	OK	31.1783	33.4657	0.102139	0.191735	0.70675	0.99995	no

ENSG00000074696	ENSG00000074696	PTPLAD1	15:65822755-65870687	q1	q2	OK	119.189	124.536	0.063316	0.145375	0.82755	0.99995	no

ENSG00000074706	ENSG00000074706	IPCEF1	6:154331630-154677926	q1	q2	NOTEST	0.0765431	0.0597869	-0.356444	0	1	1	no

ENSG00000074755	ENSG00000074755	ZZEF1	17:3907738-4046314	q1	q2	OK	8.0335	8.55358	0.0904988	0.120277	0.79115	0.99995	no

ENSG00000074771	ENSG00000074771	NOX3	6:155716503-155777037	q1	q2	NOTEST	0	0	0	0	1	1	no

ENSG00000074800	ENSG00000074800	ENO1	1:8921060-8939953	q1	q2	OK	452.757	447.38	-0.0172366	-0.037794	0.95585	0.99995	no

ENSG00000074803	ENSG00000074803	SLC12A1	15:48481233-48596275	q1	q2	NOTEST	0.0615587	0.0278566	-1.14394	0	1	1	no

ENSG00000074842	ENSG00000074842	C19orf10	19:4639529-4670382	q1	q2	OK	51.9628	45.2422	-0.199811	-0.365908	0.6039	0.99995	no

ENSG00000074855	ENSG00000074855	ANO8	19:17402939-17445638	q1	q2	OK	14.3492	12.8875	-0.15499	-0.205415	0.7451	0.99995	no

ENSG00000074935	ENSG00000074935	TUBE1	6:112375274-112408732	q1	q2	NOTEST	0	0	0	0	1	1	no

ENSG00000074964	ENSG00000074964	ARHGEF10L	1:17866329-18024369	q1	q2	OK	13.3684	12.2305	-0.128344	-0.201792	0.7014	0.99995	no

ENSG00000074966	ENSG00000074966	TXK	4:48068409-48136273	q1	q2	NOTEST	0.0350292	0.0721709	1.04286	0	1	1	no

ENSG00000075035	ENSG00000075035	WSCD2	12:108523247-108644314	q1	q2	OK	2.93647	2.77869	-0.0796796	-0.0851498	0.8528	0.99995	no

ENSG00000075043	ENSG00000075043	KCNQ2	20:62037541-62103993	q1	q2	OK	68.0671	63.268	-0.105481	-0.241134	0.73	0.99995	no

ENSG00000075073	ENSG00000075073	TACR2	10:71163658-71176623	q1	q2	NOTEST	0.143747	0.184793	0.362381	0	1	1	no

ENSG00000075089	ENSG00000075089	ACTR6	12:100550134-100660857	q1	q2	OK	21.0995	21.179	0.00542323	0.00885551	0.9885	0.99995	no

ENSG00000075131	ENSG00000075131	TIPIN	15:66628543-66679084	q1	q2	OK	11.7709	14.8689	0.337069	0.57269	0.39815	0.99995	no

ENSG00000075142	ENSG00000075142	SRI	7:87834432-87936206	q1	q2	OK	88.7796	96.534	0.120809	0.238202	0.7331	0.99995	no

ENSG00000075151	ENSG00000075151	EIF4G3	1:21132962-21503377	q1	q2	OK	52.5653	54.5111	0.0524386	0.105187	0.85555	0.99995	no

ENSG00000075188	ENSG00000075188	NUP37	12:102457132-102513902	q1	q2	OK	20.4191	21.8263	0.0961466	0.186238	0.7902	0.99995	no

ENSG00000075213	ENSG00000075213	SEMA3A	7:83585092-84122040	q1	q2	OK	22.3041	25.2457	0.17873	0.237028	0.64375	0.99995	no

ENSG00000075218	ENSG00000075218	GTSE1	22:46692637-46726707	q1	q2	OK	32.1523	30.8253	-0.0608048	-0.118361	0.85125	0.99995	no

ENSG00000075223	ENSG00000075223	SEMA3C	7:80371853-80551675	q1	q2	OK	3.69864	2.72916	-0.438536	-0.716233	0.2399	0.99995	no

ENSG00000075234	ENSG00000075234	TTC38	22:46663857-46689905	q1	q2	OK	10.8726	10.0103	-0.119219	-0.169492	0.7604	0.99995	no

ENSG00000075239	ENSG00000075239	ACAT1	11:107879458-108018503	q1	q2	OK	28.7986	30.6362	0.0892391	0.101211	0.86395	0.99995	no

ENSG00000075240	ENSG00000075240	GRAMD4	22:46971908-47075688	q1	q2	OK	9.28499	9.99647	0.106518	0.0935464	0.8013	0.99995	no

ENSG00000075275	ENSG00000075275	CELSR1	22:46756730-46933067	q1	q2	OK	7.20475	6.24925	-0.205267	-0.32684	0.61265	0.99995	no

ENSG00000075290	ENSG00000075290	WNT8B	10:102222797-102243501	q1	q2	NOTEST	0.429187	0.460071	0.100251	0	1	1	no

ENSG00000075292	ENSG00000075292	ZNF638	2:71503690-71662199	q1	q2	OK	89.961	103.592	0.203542	0.307921	0.54425	0.99995	no

ENSG00000075303	ENSG00000075303	SLC25A40	7:87462882-87538856	q1	q2	OK	17.9584	18.3873	0.0340477	0.0269993	0.9472	0.99995	no

ENSG00000075336	ENSG00000075336	TIMM21	18:71815745-71826197	q1	q2	OK	23.8712	24.4651	0.0354506	0.065451	0.9262	0.99995	no

ENSG00000075340	ENSG00000075340	ADD2	2:70834749-70995357	q1	q2	NOTEST	0	0	0	0	1	1	no

ENSG00000075388	ENSG00000075388	FGF4	11:69587796-69590171	q1	q2	NOTEST	0	0	0	0	1	1	no

ENSG00000075391	ENSG00000075391	RASAL2	1:178060642-178448644	q1	q2	OK	14.113	12.3691	-0.190284	-0.272741	0.5526	0.99995	no

ENSG00000075399	ENSG00000075399	VPS9D1	16:89773541-89883065	q1	q2	OK	9.5208	7.70583	-0.305132	-0.25221	0.6312	0.99995	no

ENSG00000075407	ENSG00000075407	ZNF37A	10:38383263-38414472	q1	q2	OK	8.74199	9.83343	0.169732	0.228396	0.63425	0.99995	no

ENSG00000075413	ENSG00000075413	MARK3	14:103851728-103970168	q1	q2	OK	62.965	65.5915	0.0589566	0.1163	0.84835	0.99995	no

ENSG00000075415	ENSG00000075415	SLC25A3	12:98987368-98995946	q1	q2	OK	418.331	414.637	-0.012797	-0.0293137	0.96625	0.99995	no

ENSG00000075420	ENSG00000075420	FNDC3B	3:171757417-172119455	q1	q2	OK	23.2266	22.4168	-0.0511981	-0.078637	0.87595	0.99995	no

ENSG00000075426	ENSG00000075426	FOSL2	2:28607275-28640179	q1	q2	OK	2.45436	1.97554	-0.313105	-0.28437	0.54795	0.99995	no

ENSG00000075429	ENSG00000075429	CACNG5	17:64831234-64881603	q1	q2	NOTEST	0.359489	0.307621	-0.224793	0	1	1	no

ENSG00000075461	ENSG00000075461	CACNG4	17:64961025-65029514	q1	q2	OK	34.2424	32.3079	-0.0838964	-0.203498	0.77455	0.99995	no

ENSG00000075539	ENSG00000075539	FRYL	4:48499377-48782339	q1	q2	OK	20.2909	20.0544	-0.0169194	-0.0256488	0.9589	0.99995	no

ENSG00000075568	ENSG00000075568	TMEM131	2:98372798-98612388	q1	q2	OK	16.0956	15.6741	-0.0382831	-0.0531739	0.89855	0.99995	no

ENSG00000075618	ENSG00000075618	FSCN1	7:5632438-5646286	q1	q2	OK	177.553	161.266	-0.138804	-0.323845	0.63515	0.99995	no

ENSG00000075624	ENSG00000075624	ACTB	7:5566781-5603415	q1	q2	OK	1793.3	1811.59	0.0146335	0.0218565	0.97275	0.99995	no

ENSG00000075643	ENSG00000075643	MOCOS	18:33767481-33852120	q1	q2	NOTEST	0.0446514	0.0559538	0.32553	0	1	1	no

ENSG00000075651	ENSG00000075651	PLD1	3:171318194-171528740	q1	q2	OK	1.91981	1.85695	-0.0480306	-0.0389984	0.9194	0.99995	no

ENSG00000075673	ENSG00000075673	ATP12A	13:25254548-25285921	q1	q2	NOTEST	0.014189	0.00739304	-0.94053	0	1	1	no

ENSG00000075702	ENSG00000075702	WDR62	19:36545782-36596008	q1	q2	OK	12.6338	11.3001	-0.160955	-0.317076	0.62105	0.99995	no

ENSG00000075711	ENSG00000075711	DLG1	3:196769430-197030618	q1	q2	OK	32.741	35.7633	0.127383	0.183326	0.6945	0.99995	no

ENSG00000075785	ENSG00000075785	RAB7A	3:128444964-128533639	q1	q2	OK	75.1895	78.2247	0.057092	0.134179	0.8403	0.99995	no

ENSG00000075790	ENSG00000075790	BCAP29	7:107220001-107269943	q1	q2	OK	19.4083	19.7294	0.0236763	0.0337546	0.95585	0.99995	no

ENSG00000075826	ENSG00000075826	SEC31B	10:102246398-102319755	q1	q2	OK	9.74701	8.87901	-0.134561	-0.0941298	0.8798	0.99995	no

ENSG00000075856	ENSG00000075856	SART3	12:108908961-108955176	q1	q2	OK	36.6544	39.3508	0.102406	0.161804	0.7231	0.99995	no

ENSG00000075884	ENSG00000075884	ARHGAP15	2:143848930-144533642	q1	q2	NOTEST	0.229855	0.255003	0.149789	0	1	1	no

ENSG00000075886	ENSG00000075886	TUBA3D	2:132222472-132250316	q1	q2	NOTEST	0.253468	0.40024	0.659061	0	1	1	no

ENSG00000075891	ENSG00000075891	PAX2	10:102495359-102589698	q1	q2	OK	0.591522	0.504962	-0.228257	-0.161669	0.77825	0.99995	no

ENSG00000075914	ENSG00000075914	EXOSC7	3:44956748-45077563	q1	q2	OK	32.6674	32.5583	-0.00482805	-0.00632451	0.9936	0.99995	no

ENSG00000075945	ENSG00000075945	KIFAP3	1:169890466-170054349	q1	q2	OK	15.7392	17.0209	0.11294	0.218894	0.73495	0.99995	no

ENSG00000075975	ENSG00000075975	MKRN2	3:12525930-12705725	q1	q2	OK	19.4588	18.7837	-0.0509401	-0.05352	0.94095	0.99995	no

ENSG00000076003	ENSG00000076003	MCM6	2:136597195-136633996	q1	q2	OK	63.1511	65.0073	0.0417951	0.0949371	0.88345	0.99995	no

ENSG00000076043	ENSG00000076043	REXO2	11:114262164-114321001	q1	q2	OK	29.4696	27.941	-0.0768422	-0.098482	0.8773	0.99995	no

ENSG00000076053	ENSG00000076053	RBM7	11:114262164-114321001	q1	q2	OK	19.1824	19.8175	0.0469915	0.070538	0.91655	0.99995	no

ENSG00000076067	ENSG00000076067	RBMS2	12:56915712-56984745	q1	q2	OK	8.31238	7.88309	-0.0765002	-0.135491	0.82265	0.99995	no

ENSG00000076108	ENSG00000076108	BAZ2A	12:56989379-57030600	q1	q2	OK	33.8489	34.4359	0.0248036	0.0284691	0.9278	0.99995	no

ENSG00000076201	ENSG00000076201	PTPN23	3:47422500-47454931	q1	q2	OK	12.4423	12.1737	-0.031494	-0.0558401	0.9144	0.99995	no

ENSG00000076242	ENSG00000076242	MLH1	3:37034822-37225180	q1	q2	OK	41.3344	39.31	-0.0724446	-0.108583	0.85035	0.99995	no

ENSG00000076248	ENSG00000076248	UNG	12:109535378-109548797	q1	q2	OK	40.3456	39.7288	-0.0222268	-0.0522019	0.9409	0.99995	no

ENSG00000076258	ENSG00000076258	FMO4	1:171283346-171311223	q1	q2	NOTEST	0.445659	0.36488	-0.28852	0	1	1	no

ENSG00000076321	ENSG00000076321	KLHL20	1:173684079-173755840	q1	q2	OK	9.21457	8.96776	-0.0391698	-0.0556708	0.9173	0.99995	no

ENSG00000076344	ENSG00000076344	RGS11	16:284544-333003	q1	q2	OK	0.588749	0.154711	-1.92808	-0.35841	0.0958	0.99995	no

ENSG00000076351	ENSG00000076351	SLC46A1	17:26684603-26734215	q1	q2	NOTEST	0.019791	0.0999146	2.33585	0	1	1	no

ENSG00000076356	ENSG00000076356	PLXNA2	1:208195586-208417665	q1	q2	OK	10.1287	8.93012	-0.181692	-0.205822	0.5566	0.99995	no

ENSG00000076382	ENSG00000076382	SPAG5	17:26782769-26972472	q1	q2	OK	59.1654	61.1139	0.0467475	0.0370438	0.9205	0.99995	no

ENSG00000076513	ENSG00000076513	ANKRD13A	12:110436990-110511491	q1	q2	OK	18.8067	19.5092	0.0529041	0.0889936	0.88175	0.99995	no

ENSG00000076554	ENSG00000076554	TPD52	8:80830951-81143467	q1	q2	OK	4.80126	4.34194	-0.145074	-0.105443	0.77975	0.99995	no

ENSG00000076555	ENSG00000076555	ACACB	12:109549022-109706031	q1	q2	OK	3.22323	2.61251	-0.303072	-0.236722	0.51875	0.99995	no

ENSG00000076604	ENSG00000076604	TRAF4	17:27071001-27077974	q1	q2	OK	78.1377	91.7568	0.231796	0.395322	0.46305	0.99995	no

ENSG00000076641	ENSG00000076641	PAG1	8:81880044-82024303	q1	q2	OK	3.89135	3.46811	-0.166121	-0.228858	0.65125	0.99995	no

ENSG00000076650	ENSG00000076650	GPATCH1	19:33571785-33621448	q1	q2	OK	5.73689	5.88505	0.0367871	0.0610686	0.92545	0.99995	no

ENSG00000076662	ENSG00000076662	ICAM3	19:10444451-10450499	q1	q2	OK	3.32949	2.37629	-0.48659	-0.494489	0.4015	0.99995	no

ENSG00000076685	ENSG00000076685	NT5C2	10:104678049-104953056	q1	q2	OK	38.1913	38.7698	0.0216888	0.0306278	0.9429	0.99995	no

ENSG00000076706	ENSG00000076706	MCAM	11:119179240-119192231	q1	q2	OK	9.24871	8.48888	-0.123677	-0.176286	0.71605	0.99995	no

ENSG00000076716	ENSG00000076716	GPC4	X:132434130-132549518	q1	q2	OK	137.633	132.219	-0.0578956	-0.116597	0.86675	0.99995	no

ENSG00000076770	ENSG00000076770	MBNL3	X:131337052-131623996	q1	q2	OK	3.26073	3.86881	0.246695	0.290529	0.62285	0.99995	no

ENSG00000076826	ENSG00000076826	CAMSAP3	19:7660787-7683190	q1	q2	OK	5.04376	3.29627	-0.613665	-0.89336	0.1781	0.99995	no

ENSG00000076864	ENSG00000076864	RAP1GAP	1:21922707-21995801	q1	q2	OK	1.00983	0.93885	-0.105138	-0.0941586	0.83885	0.99995	no

ENSG00000076924	ENSG00000076924	XAB2	19:7684410-7694451	q1	q2	OK	17.8256	15.6046	-0.191979	-0.4093	0.53835	0.99995	no

ENSG00000076928	ENSG00000076928	ARHGEF1	19:42387227-42434302	q1	q2	OK	0.979091	1.26532	0.369987	0.140422	0.6164	0.99995	no

ENSG00000076944	ENSG00000076944	STXBP2	19:7694622-7712759	q1	q2	OK	0.704528	0.915643	0.378128	0.0952073	0.56585	0.99995	no

ENSG00000076984	ENSG00000076984	MAP2K7	19:7968727-7979363	q1	q2	OK	17.7192	16.4586	-0.106477	-0.192573	0.74835	0.99995	no

ENSG00000077009	ENSG00000077009	NMRK2	19:3933100-3942414	q1	q2	NOTEST	0	0.0499675	inf	0	1	1	no

ENSG00000077044	ENSG00000077044	DGKD	2:234263152-234380750	q1	q2	OK	11.8752	13.1195	0.143761	0.111469	0.72565	0.99995	no

ENSG00000077063	ENSG00000077063	CTTNBP2	7:117105837-117514193	q1	q2	OK	2.69799	2.58262	-0.0630519	-0.0719141	0.8747	0.99995	no

ENSG00000077080	ENSG00000077080	ACTL6B	7:100240719-100254084	q1	q2	OK	1.78525	1.36968	-0.382288	-0.292878	0.598	0.99995	no

ENSG00000077092	ENSG00000077092	RARB	3:25215822-25639423	q1	q2	OK	0.941446	0.638351	-0.560529	-0.549675	0.33885	0.99995	no

ENSG00000077097	ENSG00000077097	TOP2B	3:25639474-25706430	q1	q2	OK	124.914	128.826	0.0444934	0.0785862	0.8979	0.99995	no

ENSG00000077147	ENSG00000077147	TM9SF3	10:98277865-98347209	q1	q2	OK	46.5148	53.2803	0.195913	0.311219	0.5058	0.99995	no

ENSG00000077150	ENSG00000077150	NFKB2	10:104153866-104162281	q1	q2	OK	9.34645	10.59	0.180216	0.23929	0.6392	0.99995	no

ENSG00000077152	ENSG00000077152	UBE2T	1:202300784-202311108	q1	q2	OK	53.4819	56.5855	0.0813814	0.177683	0.80035	0.99995	no

ENSG00000077157	ENSG00000077157	PPP1R12B	1:202317826-202679545	q1	q2	OK	5.5184	4.38019	-0.333258	-0.224697	0.4609	0.99995	no

ENSG00000077232	ENSG00000077232	DNAJC10	2:183580998-183659191	q1	q2	OK	41.4455	45.8615	0.146068	0.258228	0.6517	0.99995	no

ENSG00000077235	ENSG00000077235	GTF3C1	16:27470875-27561234	q1	q2	OK	36.8827	30.9202	-0.254394	-0.340433	0.44025	0.99995	no

ENSG00000077238	ENSG00000077238	IL4R	16:27324988-27376099	q1	q2	OK	6.44255	5.35088	-0.267855	-0.341296	0.467	0.99995	no

ENSG00000077254	ENSG00000077254	USP33	1:78161671-78225537	q1	q2	OK	42.6343	42.7793	0.00489927	0.0078194	0.9857	0.99995	no

ENSG00000077264	ENSG00000077264	PAK3	X:110187512-110470589	q1	q2	OK	4.92091	4.78306	-0.0409902	-0.0797968	0.8949	0.99995	no

ENSG00000077274	ENSG00000077274	CAPN6	X:110488330-110513751	q1	q2	NOTEST	0.240688	0.302667	0.330566	0	1	1	no

ENSG00000077279	ENSG00000077279	DCX	X:110537006-110655603	q1	q2	OK	37.6612	36.0364	-0.0636243	-0.148547	0.82755	0.99995	no

ENSG00000077312	ENSG00000077312	SNRPA	19:41197433-41271294	q1	q2	OK	71.758	71.8741	0.00233218	0.0041434	0.99545	0.99995	no

ENSG00000077327	ENSG00000077327	SPAG6	10:22634398-22743153	q1	q2	OK	0.500674	0.371496	-0.430526	-0.27801	0.5423	0.99995	no

ENSG00000077348	ENSG00000077348	EXOSC5	19:41768400-41930910	q1	q2	OK	8.14758	7.35104	-0.148424	-0.0410354	0.9344	0.99995	no

ENSG00000077380	ENSG00000077380	DYNC1I2	2:172543918-172604930	q1	q2	OK	90.9816	94.9706	0.0619069	0.141588	0.82565	0.99995	no

ENSG00000077420	ENSG00000077420	APBB1IP	10:26727131-26856732	q1	q2	NOTEST	0	0	0	0	1	1	no

ENSG00000077454	ENSG00000077454	LRCH4	7:100169854-100205798	q1	q2	OK	21.9445	20.3143	-0.111364	-0.119259	0.86615	0.99995	no

ENSG00000077458	ENSG00000077458	FAM76B	11:95502105-95565857	q1	q2	OK	21.9039	24.966	0.188771	0.148772	0.71565	0.99995	no

ENSG00000077463	ENSG00000077463	SIRT6	19:4174105-4182601	q1	q2	OK	18.8177	15.9433	-0.239146	-0.333949	0.5905	0.99995	no

ENSG00000077498	ENSG00000077498	TYR	11:88910619-89028927	q1	q2	NOTEST	0	0	0	0	1	1	no

ENSG00000077514	ENSG00000077514	POLD3	11:74202756-74380162	q1	q2	OK	26.5203	28.6963	0.113767	0.187651	0.76265	0.99995	no

ENSG00000077522	ENSG00000077522	ACTN2	1:236849753-236927931	q1	q2	OK	0.813402	0.559435	-0.539999	-0.32671	0.54135	0.99995	no

ENSG00000077549	ENSG00000077549	CAPZB	1:19665266-19812066	q1	q2	OK	131.923	141.172	0.0977558	0.235958	0.7339	0.99995	no

ENSG00000077585	ENSG00000077585	GPR137B	1:236305831-236445319	q1	q2	OK	5.98575	5.75236	-0.0573781	-0.0716597	0.91535	0.99995	no

ENSG00000077616	ENSG00000077616	NAALAD2	11:89864682-89926062	q1	q2	OK	3.71915	3.35755	-0.147564	-0.221508	0.7361	0.99995	no

ENSG00000077684	ENSG00000077684	JADE1	4:129730778-130037795	q1	q2	OK	13.6283	12.8973	-0.0795398	-0.106766	0.8405	0.99995	no

ENSG00000077713	ENSG00000077713	SLC25A43	X:118533022-118588441	q1	q2	OK	1.48856	1.23684	-0.267256	-0.270125	0.66435	0.99995	no

ENSG00000077721	ENSG00000077721	UBE2A	X:118708500-118718381	q1	q2	OK	50.0162	50.98	0.0275363	0.057513	0.92315	0.99995	no

ENSG00000077782	ENSG00000077782	FGFR1	8:38268655-38326352	q1	q2	OK	166.45	169.193	0.0235767	0.0289393	0.9405	0.99995	no

ENSG00000077800	ENSG00000077800	FKBP6	7:72742166-72772634	q1	q2	NOTEST	0	0	0	0	1	1	no

ENSG00000077809	ENSG00000077809	GTF2I	7:74071993-74306731	q1	q2	OK	12.9365	9.30448	-0.475453	-0.395369	0.59005	0.99995	no

ENSG00000077935	ENSG00000077935	SMC1B	22:45739943-45809500	q1	q2	NOTEST	0.0299738	0.0467775	0.642113	0	1	1	no

ENSG00000077942	ENSG00000077942	FBLN1	22:45898117-45997015	q1	q2	OK	97.2469	91.9911	-0.0801583	-0.176961	0.766	0.99995	no

ENSG00000077943	ENSG00000077943	ITGA8	10:15555947-15762124	q1	q2	OK	0.936365	0.786312	-0.251969	-0.195559	0.67975	0.99995	no

ENSG00000077984	ENSG00000077984	CST7	20:24929865-24940564	q1	q2	NOTEST	0	0	0	0	1	1	no

ENSG00000078018	ENSG00000078018	MAP2	2:210288781-210598842	q1	q2	OK	105.501	104.027	-0.0202889	-0.033008	0.9466	0.99995	no

ENSG00000078043	ENSG00000078043	PIAS2	18:44388352-44627658	q1	q2	OK	8.59266	8.46888	-0.0209348	-0.0320513	0.95775	0.99995	no

ENSG00000078053	ENSG00000078053	AMPH	7:38423304-38671167	q1	q2	OK	23.5103	21.3263	-0.140663	-0.284256	0.64895	0.99995	no

ENSG00000078061	ENSG00000078061	ARAF	X:47420515-47479252	q1	q2	OK	16.1907	15.332	-0.0786173	-0.0674634	0.9102	0.99995	no

ENSG00000078070	ENSG00000078070	MCCC1	3:182733005-182833863	q1	q2	OK	10.3239	10.3703	0.0064616	0.010475	0.9859	0.99995	no

ENSG00000078081	ENSG00000078081	LAMP3	3:182840000-182881627	q1	q2	NOTEST	0.107872	0.137098	0.345879	0	1	1	no

ENSG00000078098	ENSG00000078098	FAP	2:163016271-163101661	q1	q2	OK	2.39614	3.37163	0.492736	0.39192	0.4792	0.99995	no

ENSG00000078114	ENSG00000078114	NEBL	10:21068901-21463977	q1	q2	OK	3.46114	3.21171	-0.107902	-0.102485	0.80985	0.99995	no

ENSG00000078124	ENSG00000078124	ACER3	11:76571910-76737841	q1	q2	OK	4.03086	6.27679	0.63894	0.666053	0.31755	0.99995	no

ENSG00000078140	ENSG00000078140	UBE2K	4:39699663-39784412	q1	q2	OK	43.7726	52.2389	0.255095	0.465837	0.4189	0.99995	no

ENSG00000078142	ENSG00000078142	PIK3C3	18:39535170-39667794	q1	q2	OK	27.0461	28.2617	0.0634313	0.073967	0.9119	0.99995	no

ENSG00000078177	ENSG00000078177	N4BP2	4:40044536-40159872	q1	q2	OK	10.6738	10.869	0.0261431	0.0458264	0.933	0.99995	no

ENSG00000078237	ENSG00000078237	C12orf5	12:4430370-4462338	q1	q2	OK	19.5517	23.2734	0.251388	0.496072	0.4692	0.99995	no

ENSG00000078246	ENSG00000078246	TULP3	12:2904118-3050306	q1	q2	OK	21.8461	21.2617	-0.0391148	-0.02803	0.9659	0.99995	no

ENSG00000078269	ENSG00000078269	SYNJ2	6:158402887-158520208	q1	q2	OK	4.67002	6.07419	0.379262	0.659636	0.23935	0.99995	no

ENSG00000078295	ENSG00000078295	ADCY2	5:7396320-7830194	q1	q2	OK	0.815892	0.934687	0.196105	0.0914451	0.85075	0.99995	no

ENSG00000078304	ENSG00000078304	PPP2R5C	14:102228134-102394326	q1	q2	OK	63.2084	63.1887	-0.000448116	-0.000870163	0.99905	0.99995	no

ENSG00000078319	ENSG00000078319	PMS2P1	7:99905324-99997719	q1	q2	OK	6.04171	5.84844	-0.0469058	-0.0125438	0.97915	0.99995	no

ENSG00000078328	ENSG00000078328	RBFOX1	16:5289802-7763340	q1	q2	OK	1.25717	1.37986	0.134345	0.0993097	0.7869	0.99995	no

ENSG00000078369	ENSG00000078369	GNB1	1:1716728-1822495	q1	q2	OK	191.669	204.261	0.0918028	0.200896	0.7557	0.99995	no

ENSG00000078399	ENSG00000078399	HOXA9	7:27202053-27219880	q1	q2	NOTEST	0.349813	0.2789	-0.326835	0	1	1	no

ENSG00000078401	ENSG00000078401	EDN1	6:12290595-12297427	q1	q2	OK	0.558563	0.494286	-0.176376	-0.172645	0.81435	0.99995	no

ENSG00000078403	ENSG00000078403	MLLT10	10:21823093-22032559	q1	q2	OK	26.9303	24.3959	-0.142591	-0.171885	0.6441	0.99995	no

ENSG00000078487	ENSG00000078487	ZCWPW1	7:99998448-100031741	q1	q2	OK	1.88843	1.62825	-0.213864	-0.0807184	0.84845	0.99995	no

ENSG00000078549	ENSG00000078549	ADCYAP1R1	7:31092075-31151089	q1	q2	OK	11.2748	13.3059	0.23897	0.41177	0.4421	0.99995	no

ENSG00000078579	ENSG00000078579	FGF20	8:16849677-16859690	q1	q2	NOTEST	0.104228	0.117301	0.170479	0	1	1	no

ENSG00000078589	ENSG00000078589	P2RY10	X:78200828-78217451	q1	q2	NOTEST	0.0169845	0	#NAME?	0	1	1	no

ENSG00000078596	ENSG00000078596	ITM2A	X:78615880-78623164	q1	q2	OK	14.8181	21.0801	0.508518	0.74604	0.23385	0.99995	no

ENSG00000078618	ENSG00000078618	NRD1	1:52027537-52344477	q1	q2	OK	73.3094	79.9847	0.125725	0.16314	0.73965	0.99995	no

ENSG00000078668	ENSG00000078668	VDAC3	8:42249141-42263415	q1	q2	OK	113.796	113.685	-0.00141512	-0.00335677	0.9961	0.99995	no

ENSG00000078674	ENSG00000078674	PCM1	8:17780348-17885478	q1	q2	OK	94.3285	100.634	0.0933577	0.127216	0.7681	0.99995	no

ENSG00000078687	ENSG00000078687	TNRC6C	17:76000248-76139049	q1	q2	OK	8.09542	6.89874	-0.230772	-0.262402	0.5587	0.99995	no

ENSG00000078699	ENSG00000078699	CBFA2T2	20:32077880-32237842	q1	q2	OK	13.9929	14.4664	0.0480072	0.0832972	0.8711	0.99995	no

ENSG00000078725	ENSG00000078725	BRINP1	9:121915735-122131745	q1	q2	NOTEST	0.275171	0.331704	0.269568	0	1	1	no

ENSG00000078747	ENSG00000078747	ITCH	20:32951040-33099198	q1	q2	OK	17.7539	18.7042	0.0752258	0.0669012	0.79615	0.99995	no

ENSG00000078795	ENSG00000078795	PKD2L2	5:137150021-137387650	q1	q2	NOTEST	0.0137223	0.0275096	1.00341	0	1	1	no

ENSG00000078804	ENSG00000078804	TP53INP2	20:33284721-33413452	q1	q2	OK	8.9576	8.93607	-0.00347244	-0.00325741	0.9958	0.99995	no

ENSG00000078808	ENSG00000078808	SDF4	1:1152287-1167411	q1	q2	OK	46.1399	42.0913	-0.132492	-0.267859	0.70215	0.99995	no

ENSG00000078814	ENSG00000078814	MYH7B	20:33563205-33680674	q1	q2	OK	0.393448	0.488764	0.312964	0.0467428	0.7581	0.99995	no

ENSG00000078898	ENSG00000078898	BPIFB2	20:31595405-31611515	q1	q2	NOTEST	0	0	0	0	1	1	no

ENSG00000078900	ENSG00000078900	TP73	1:3547330-3663900	q1	q2	OK	1.55491	1.63412	0.0716798	0.0378057	0.94745	0.99995	no

ENSG00000078902	ENSG00000078902	TOLLIP	11:1295600-1330884	q1	q2	OK	14.313	12.8038	-0.160756	-0.2959	0.62915	0.99995	no

ENSG00000078967	ENSG00000078967	UBE2D4	7:43966036-44058774	q1	q2	OK	5.93904	5.28464	-0.168424	-0.152404	0.7553	0.99995	no

ENSG00000079101	ENSG00000079101	CLUL1	18:596987-712676	q1	q2	NOTEST	0.282969	0.385389	0.445669	0	1	1	no

ENSG00000079102	ENSG00000079102	RUNX1T1	8:92967202-93115514	q1	q2	OK	3.03879	2.88519	-0.0748324	-0.0692801	0.8118	0.99995	no

ENSG00000079112	ENSG00000079112	CDH17	8:95139398-95229531	q1	q2	NOTEST	0	0	0	0	1	1	no

ENSG00000079134	ENSG00000079134	THOC1	18:158382-268050	q1	q2	OK	49.113	53.5741	0.125433	0.134328	0.78315	0.99995	no

ENSG00000079150	ENSG00000079150	FKBP7	2:179328390-179343327	q1	q2	OK	11.5615	13.2248	0.193917	0.24616	0.7231	0.99995	no

ENSG00000079156	ENSG00000079156	OSBPL6	2:179059207-179264160	q1	q2	OK	6.0023	6.2455	0.0573026	0.10658	0.8709	0.99995	no

ENSG00000079215	ENSG00000079215	SLC1A3	5:36606456-36725297	q1	q2	OK	28.4889	25.9285	-0.135862	-0.254889	0.62845	0.99995	no

ENSG00000079246	ENSG00000079246	XRCC5	2:216972186-217071026	q1	q2	OK	205.928	226.553	0.137713	0.266072	0.6515	0.99995	no

ENSG00000079257	ENSG00000079257	LXN	3:158362066-158410364	q1	q2	NOTEST	0.341128	0.336429	-0.0200138	0	1	1	no

ENSG00000079263	ENSG00000079263	SP140	2:230990025-231268447	q1	q2	NOTEST	0.217421	0.168018	-0.371881	0	1	1	no

ENSG00000079277	ENSG00000079277	MKNK1	1:47004367-47082563	q1	q2	OK	12.6103	13.4972	0.0980521	0.115758	0.7899	0.99995	no

ENSG00000079308	ENSG00000079308	TNS1	2:218664511-218867718	q1	q2	OK	6.71096	7.27189	0.115811	0.140004	0.78015	0.99995	no

ENSG00000079313	ENSG00000079313	REXO1	19:1815247-1848452	q1	q2	OK	11.683	10.9284	-0.0963198	-0.116247	0.7907	0.99995	no

ENSG00000079332	ENSG00000079332	SAR1A	10:71907044-71930279	q1	q2	OK	48.6985	50.931	0.0646661	0.14422	0.81855	0.99995	no

ENSG00000079335	ENSG00000079335	CDC14A	1:100810032-100985833	q1	q2	OK	6.24831	6.8858	0.140157	0.259531	0.69125	0.99995	no

ENSG00000079337	ENSG00000079337	RAPGEF3	12:48099867-48231681	q1	q2	OK	0.554709	0.664535	0.260613	0.0450805	0.74205	0.99995	no

ENSG00000079385	ENSG00000079385	CEACAM1	19:42901279-43156507	q1	q2	NOTEST	0.291018	0.297151	0.0300891	0	1	1	no

ENSG00000079387	ENSG00000079387	SENP1	12:48436680-48540187	q1	q2	OK	14.3884	16.2107	0.172038	0.213826	0.72065	0.99995	no

ENSG00000079393	ENSG00000079393	DUSP13	10:76854191-76941881	q1	q2	NOTEST	0	0.0482194	inf	0	1	1	no

ENSG00000079432	ENSG00000079432	CIC	19:42772688-42799949	q1	q2	OK	26.5011	24.7247	-0.100097	-0.142575	0.8017	0.99995	no

ENSG00000079435	ENSG00000079435	LIPE	19:42901279-43156507	q1	q2	OK	6.7217	6.2357	-0.108274	-0.114412	0.85385	0.99995	no

ENSG00000079459	ENSG00000079459	FDFT1	8:11653081-11696818	q1	q2	OK	294.776	342.906	0.218198	0.463165	0.47445	0.99995	no

ENSG00000079462	ENSG00000079462	PAFAH1B3	19:42801184-42814973	q1	q2	OK	86.348	77.532	-0.15537	-0.346708	0.61855	0.99995	no

ENSG00000079482	ENSG00000079482	OPHN1	X:67262185-67653755	q1	q2	OK	2.74038	2.64569	-0.0507306	-0.0425769	0.9045	0.99995	no

ENSG00000079557	ENSG00000079557	AFM	4:74347399-74369691	q1	q2	NOTEST	0	0	0	0	1	1	no

ENSG00000079616	ENSG00000079616	KIF22	16:29802039-29816706	q1	q2	OK	82.5875	76.6294	-0.108026	-0.256509	0.7014	0.99995	no

ENSG00000079689	ENSG00000079689	SCGN	6:25652463-25702011	q1	q2	NOTEST	0	0	0	0	1	1	no

ENSG00000079691	ENSG00000079691	LRRC16A	6:25279305-25620758	q1	q2	OK	2.37724	2.26542	-0.0695096	-0.0863654	0.88475	0.99995	no

ENSG00000079739	ENSG00000079739	PGM1	1:63906440-64125916	q1	q2	OK	14.4997	14.9055	0.0398223	0.0562832	0.93785	0.99995	no

ENSG00000079785	ENSG00000079785	DDX1	2:15731301-15771235	q1	q2	OK	86.3898	95.2544	0.140925	0.302385	0.6117	0.99995	no

ENSG00000079805	ENSG00000079805	DNM2	19:10828754-10946994	q1	q2	OK	37.9215	35.0683	-0.112848	-0.166137	0.7155	0.99995	no

ENSG00000079819	ENSG00000079819	EPB41L2	6:131160486-131384462	q1	q2	OK	37.6051	44.8264	0.253422	0.366905	0.4086	0.99995	no

ENSG00000079841	ENSG00000079841	RIMS1	6:72596405-73112845	q1	q2	OK	0.58476	0.665644	0.186906	0.181983	0.73625	0.99995	no

ENSG00000079931	ENSG00000079931	MOXD1	6:132617193-132722684	q1	q2	OK	9.76631	10.423	0.0938863	0.178413	0.79315	0.99995	no

ENSG00000079950	ENSG00000079950	STX7	6:132767005-132834337	q1	q2	OK	16.6187	16.4784	-0.0122356	-0.0141533	0.98305	0.99995	no

ENSG00000079974	ENSG00000079974	RABL2B	22:51195375-51239737	q1	q2	NOTEST	0.0292272	0.0138084	-1.08176	0	1	1	no

ENSG00000079999	ENSG00000079999	KEAP1	19:10596795-10614417	q1	q2	OK	35.273	31.2493	-0.174744	-0.360544	0.55535	0.99995	no

ENSG00000080007	ENSG00000080007	DDX43	6:74078277-74161999	q1	q2	NOTEST	0	0	0	0	1	1	no

ENSG00000080031	ENSG00000080031	PTPRH	19:55692615-55720874	q1	q2	NOTEST	0.117425	0.10695	-0.134799	0	1	1	no

ENSG00000080166	ENSG00000080166	DCT	13:95089557-95131936	q1	q2	NOTEST	0.0938556	0.0234396	-2.00149	0	1	1	no

ENSG00000080189	ENSG00000080189	SLC35C2	20:44978166-44993043	q1	q2	OK	21.0511	18.0557	-0.22144	-0.389258	0.5632	0.99995	no

ENSG00000080200	ENSG00000080200	CRYBG3	3:97595818-97691301	q1	q2	OK	6.63047	6.36958	-0.057913	-0.0814199	0.9058	0.99995	no

ENSG00000080224	ENSG00000080224	EPHA6	3:96533424-97471304	q1	q2	OK	0.41761	0.484802	0.215242	0.156883	0.74165	0.99995	no

ENSG00000080293	ENSG00000080293	SCTR	2:120197418-120282070	q1	q2	NOTEST	0.0114359	0	#NAME?	0	1	1	no

ENSG00000080298	ENSG00000080298	RFX3	9:3218296-3526004	q1	q2	OK	18.6103	19.2326	0.0474503	0.0906824	0.8659	0.99995	no

ENSG00000080345	ENSG00000080345	RIF1	2:152266396-152591001	q1	q2	OK	18.1566	21.4186	0.238371	0.346112	0.4331	0.99995	no

ENSG00000080371	ENSG00000080371	RAB21	12:72148653-72184699	q1	q2	OK	26.482	28.7531	0.118704	0.14976	0.81865	0.99995	no

ENSG00000080493	ENSG00000080493	SLC4A4	4:72053002-72437804	q1	q2	OK	4.13926	4.54276	0.134196	0.249391	0.7089	0.99995	no

ENSG00000080503	ENSG00000080503	SMARCA2	9:2015341-2193624	q1	q2	OK	22.4388	22.1625	-0.017876	-0.026374	0.9645	0.99995	no

ENSG00000080511	ENSG00000080511	RDH8	19:10123924-10132955	q1	q2	NOTEST	0.0472794	0	#NAME?	0	1	1	no

ENSG00000080546	ENSG00000080546	SESN1	6:109307639-109416022	q1	q2	OK	16.8006	14.2595	-0.236585	-0.45917	0.4599	0.99995	no

ENSG00000080561	ENSG00000080561	MID2	X:107068984-107179210	q1	q2	OK	1.20382	0.93956	-0.357567	-0.326781	0.58465	0.99995	no

ENSG00000080572	ENSG00000080572	PIH1D3	X:106449861-106487473	q1	q2	NOTEST	0.0137485	0	#NAME?	0	1	1	no

ENSG00000080573	ENSG00000080573	COL5A3	19:10070236-10121147	q1	q2	OK	5.50954	4.14729	-0.409762	-0.741504	0.26135	0.99995	no

ENSG00000080603	ENSG00000080603	SRCAP	16:30709029-30787628	q1	q2	OK	28.0316	27.6358	-0.0205161	-0.0206349	0.964	0.99995	no

ENSG00000080608	ENSG00000080608	KIAA0020	9:2717501-2844241	q1	q2	OK	16.7361	18.3576	0.133416	0.222176	0.7008	0.99995	no

ENSG00000080618	ENSG00000080618	CPB2	13:46626940-46687467	q1	q2	NOTEST	0.0356832	0.103136	1.53122	0	1	1	no

ENSG00000080644	ENSG00000080644	CHRNA3	15:78857861-78913637	q1	q2	OK	1.71128	1.48484	-0.204764	-0.122032	0.7877	0.99995	no

ENSG00000080709	ENSG00000080709	KCNN2	5:113696641-114004107	q1	q2	NOTEST	0.452282	0.24335	-0.894191	0	1	1	no

ENSG00000080802	ENSG00000080802	CNOT4	7:135046546-135194875	q1	q2	OK	10.1948	9.96673	-0.0326441	-0.0665086	0.92045	0.99995	no

ENSG00000080815	ENSG00000080815	PSEN1	14:73603125-73690399	q1	q2	OK	32.9185	33.0817	0.00713599	0.0134333	0.97965	0.99995	no

ENSG00000080819	ENSG00000080819	CPOX	3:98216447-98312567	q1	q2	OK	11.2922	10.8958	-0.0515526	-0.0499643	0.9262	0.99995	no

ENSG00000080822	ENSG00000080822	CLDND1	3:98216447-98312567	q1	q2	OK	55.6143	60.4494	0.12027	0.204554	0.72115	0.99995	no

ENSG00000080823	ENSG00000080823	MOK	14:102547074-102771537	q1	q2	OK	9.18728	7.45481	-0.301468	-0.032196	0.5914	0.99995	no

ENSG00000080824	ENSG00000080824	HSP90AA1	14:102547074-102771537	q1	q2	OK	521.065	554.657	0.0901328	0.152102	0.8172	0.99995	no

ENSG00000080839	ENSG00000080839	RBL1	20:35624751-35725581	q1	q2	OK	8.09036	8.56969	0.0830399	0.173427	0.79015	0.99995	no

ENSG00000080845	ENSG00000080845	DLGAP4	20:34894257-35274619	q1	q2	OK	52.1116	48.2837	-0.110069	-0.167118	0.80405	0.99995	no

ENSG00000080854	ENSG00000080854	IGSF9B	11:133778458-133826880	q1	q2	OK	3.37606	2.63089	-0.359791	-0.286193	0.62845	0.99995	no

ENSG00000080910	ENSG00000080910	CFHR2	1:196788886-196928356	q1	q2	NOTEST	0	0	0	0	1	1	no

ENSG00000080947	ENSG00000080947	CROCCP3	1:16787442-16825752	q1	q2	OK	7.05182	3.46725	-1.0242	-0.605915	0.098	0.99995	no

ENSG00000080986	ENSG00000080986	NDC80	18:2571509-2616634	q1	q2	OK	41.6666	44.8312	0.105612	0.19767	0.71315	0.99995	no

ENSG00000081014	ENSG00000081014	AP4E1	15:51200868-51298097	q1	q2	OK	4.74364	5.61945	0.244433	0.431489	0.48165	0.99995	no

ENSG00000081019	ENSG00000081019	RSBN1	1:114304453-114456708	q1	q2	OK	9.49407	9.3049	-0.0290358	-0.0341528	0.9482	0.99995	no

ENSG00000081026	ENSG00000081026	MAGI3	1:113933370-114228545	q1	q2	OK	7.4901	8.16648	0.124729	0.195905	0.7257	0.99995	no

ENSG00000081041	ENSG00000081041	CXCL2	4:74962751-74965010	q1	q2	NOTEST	0.212018	0.139199	-0.607041	0	1	1	no

ENSG00000081051	ENSG00000081051	AFP	4:74296854-74321891	q1	q2	NOTEST	0.143802	0.0882288	-0.704765	0	1	1	no

ENSG00000081052	ENSG00000081052	COL4A4	2:227867426-228028829	q1	q2	NOTEST	0.0251319	0.0218362	-0.202798	0	1	1	no

ENSG00000081059	ENSG00000081059	TCF7	5:133450401-133706738	q1	q2	OK	3.76115	3.69579	-0.0252891	-0.00667403	0.9741	0.99995	no

ENSG00000081087	ENSG00000081087	OSTM1	6:108362612-108487058	q1	q2	OK	12.1333	14.5698	0.264012	0.405521	0.5051	0.99995	no

ENSG00000081138	ENSG00000081138	CDH7	18:63417487-63548638	q1	q2	OK	13.9049	12.3938	-0.165976	-0.251642	0.68365	0.99995	no

ENSG00000081148	ENSG00000081148	IMPG2	3:100941389-101039404	q1	q2	NOTEST	0.328197	0.361878	0.140939	0	1	1	no

ENSG00000081154	ENSG00000081154	PCNP	3:101292938-101313281	q1	q2	OK	101.241	113.98	0.170992	0.276042	0.6706	0.99995	no

ENSG00000081177	ENSG00000081177	EXD2	14:69649736-69821183	q1	q2	OK	13.0755	12.2615	-0.0927347	-0.110944	0.86035	0.99995	no

ENSG00000081181	ENSG00000081181	ARG2	14:68086514-68162531	q1	q2	OK	7.5487	8.37024	0.149042	0.0765608	0.9053	0.99995	no

ENSG00000081189	ENSG00000081189	MEF2C	5:87803362-88762215	q1	q2	OK	2.38985	2.0683	-0.208473	-0.0973854	0.7927	0.99995	no

ENSG00000081237	ENSG00000081237	PTPRC	1:198607800-198726545	q1	q2	NOTEST	0	0	0	0	1	1	no

ENSG00000081248	ENSG00000081248	CACNA1S	1:201004223-201081694	q1	q2	NOTEST	0.0209151	0.00792008	-1.40095	0	1	1	no

ENSG00000081277	ENSG00000081277	PKP1	1:201252579-201302121	q1	q2	NOTEST	0	0	0	0	1	1	no

ENSG00000081307	ENSG00000081307	UBA5	3:132276981-132593067	q1	q2	OK	29.2923	31.2298	0.0924034	0.127934	0.8193	0.99995	no

ENSG00000081320	ENSG00000081320	STK17B	2:196998289-197041227	q1	q2	OK	2.78958	2.81696	0.0140891	0.0189962	0.9723	0.99995	no

ENSG00000081377	ENSG00000081377	CDC14B	9:99212482-99382112	q1	q2	OK	6.56114	7.0663	0.107007	0.138494	0.78645	0.99995	no

ENSG00000081386	ENSG00000081386	ZNF510	9:99518146-99540411	q1	q2	OK	7.89836	7.75236	-0.0269184	-0.0479195	0.9298	0.99995	no

ENSG00000081479	ENSG00000081479	LRP2	2:169983618-170219195	q1	q2	OK	19.2925	15.9913	-0.270754	-0.263054	0.5329	0.99995	no

ENSG00000081665	ENSG00000081665	ZNF506	19:19868174-19945736	q1	q2	OK	4.41405	3.5838	-0.300612	-0.333968	0.53555	0.99995	no

ENSG00000081692	ENSG00000081692	JMJD4	1:227916239-227968927	q1	q2	OK	10.2733	9.16507	-0.164679	-0.205505	0.7543	0.99995	no

ENSG00000081721	ENSG00000081721	DUSP12	1:161719547-161727028	q1	q2	OK	39.0701	39.7827	0.0260763	0.0483648	0.94285	0.99995	no

ENSG00000081760	ENSG00000081760	AACS	12:125549924-125627873	q1	q2	OK	10.7831	10.7739	-0.00123233	-0.00228838	0.9967	0.99995	no

ENSG00000081791	ENSG00000081791	KIAA0141	5:141303372-141321612	q1	q2	OK	40.8302	37.9522	-0.105456	-0.158982	0.8155	0.99995	no

ENSG00000081800	ENSG00000081800	SLC13A1	7:122753584-122840040	q1	q2	NOTEST	0	0	0	0	1	1	no

ENSG00000081803	ENSG00000081803	CADPS2	7:121958480-122526813	q1	q2	OK	2.46902	2.14566	-0.202521	-0.27374	0.69655	0.99995	no

ENSG00000081818	ENSG00000081818	PCDHB4	5:140501580-140505201	q1	q2	NOTEST	0.0142476	0.0201455	0.499746	0	1	1	no

ENSG00000081842	ENSG00000081842	PCDHA6	5:140165875-140391936	q1	q2	NOTEST	0.0116809	0	#NAME?	0	1	1	no

ENSG00000081853	ENSG00000081853	PCDHGA2	5:140710251-140892546	q1	q2	NOTEST	0.142981	0.154375	0.110616	0	1	1	no

ENSG00000081870	ENSG00000081870	HSPB11	1:54382246-54411602	q1	q2	OK	26.7705	28.6714	0.0989717	0.184022	0.80545	0.99995	no

ENSG00000081913	ENSG00000081913	PHLPP1	18:60382671-60647666	q1	q2	OK	31.3273	31.3602	0.0015123	0.00348417	0.9958	0.99995	no

ENSG00000081923	ENSG00000081923	ATP8B1	18:55297533-55470333	q1	q2	OK	0.236745	0.565015	1.25496	0.398711	0.27465	0.99995	no

ENSG00000081985	ENSG00000081985	IL12RB2	1:67773046-67862583	q1	q2	NOTEST	0.192351	0.0773613	-1.31405	0	1	1	no

ENSG00000082014	ENSG00000082014	SMARCD3	7:150929574-150974982	q1	q2	OK	37.1813	34.8146	-0.0948834	-0.136507	0.82985	0.99995	no

ENSG00000082068	ENSG00000082068	WDR70	5:37379313-37753537	q1	q2	OK	33.7201	32.7706	-0.0412037	-0.0618505	0.9269	0.99995	no

ENSG00000082074	ENSG00000082074	FYB	5:39105337-39274630	q1	q2	NOTEST	0.052738	0	#NAME?	0	1	1	no

ENSG00000082126	ENSG00000082126	MPP4	2:202509592-202563417	q1	q2	NOTEST	0.128553	0.169268	0.396947	0	1	1	no

ENSG00000082146	ENSG00000082146	STRADB	2:202241929-202345569	q1	q2	OK	29.4214	29.2512	-0.00837215	-0.0100719	0.98555	0.99995	no

ENSG00000082153	ENSG00000082153	BZW1	2:201542955-201688569	q1	q2	OK	157.071	178.266	0.182618	0.339872	0.58635	0.99995	no

ENSG00000082175	ENSG00000082175	PGR	11:100900354-101001255	q1	q2	NOTEST	0.0053482	0.00169237	-1.66001	0	1	1	no

ENSG00000082196	ENSG00000082196	C1QTNF3	5:33986282-34124633	q1	q2	OK	7.40162	7.35722	-0.00868014	-0.00971057	0.9897	0.99995	no

ENSG00000082212	ENSG00000082212	ME2	18:48405418-48474691	q1	q2	OK	31.3026	39.6927	0.342589	0.557737	0.3001	0.99995	no

ENSG00000082213	ENSG00000082213	C5orf22	5:31532372-31555165	q1	q2	OK	16.853	16.4711	-0.0330734	-0.0568536	0.9186	0.99995	no

ENSG00000082258	ENSG00000082258	CCNT2	2:135493033-135716912	q1	q2	OK	30.0848	31.1019	0.0479672	0.0892422	0.8708	0.99995	no

ENSG00000082269	ENSG00000082269	FAM135A	6:71122530-71270877	q1	q2	OK	12.4744	12.6182	0.0165378	0.0234094	0.9659	0.99995	no

ENSG00000082293	ENSG00000082293	COL19A1	6:70576462-70919679	q1	q2	NOTEST	0.18711	0.119961	-0.641325	0	1	1	no

ENSG00000082397	ENSG00000082397	EPB41L3	18:5392382-5630699	q1	q2	OK	23.0163	22.3171	-0.0445068	-0.0788081	0.87665	0.99995	no

ENSG00000082438	ENSG00000082438	COBLL1	2:165510133-165705844	q1	q2	OK	5.66692	6.37802	0.170545	0.179636	0.6453	0.99995	no

ENSG00000082458	ENSG00000082458	DLG3	X:69664710-69725337	q1	q2	OK	23.2735	28.0422	0.268911	0.44523	0.41005	0.99995	no

ENSG00000082482	ENSG00000082482	KCNK2	1:215179117-215410436	q1	q2	OK	6.61851	6.21022	-0.0918636	-0.150294	0.80885	0.99995	no

ENSG00000082497	ENSG00000082497	SERTAD4	1:210404800-210419976	q1	q2	OK	8.69193	9.38843	0.111208	0.139199	0.77405	0.99995	no

ENSG00000082512	ENSG00000082512	TRAF5	1:211499956-211548288	q1	q2	OK	3.97292	4.86178	0.291286	0.460776	0.48095	0.99995	no

ENSG00000082515	ENSG00000082515	MRPL22	5:154320629-154348971	q1	q2	OK	41.5326	44.0315	0.084289	0.146191	0.8324	0.99995	no

ENSG00000082516	ENSG00000082516	GEMIN5	5:154266975-154317769	q1	q2	OK	8.94756	9.53822	0.0922254	0.171647	0.7617	0.99995	no

ENSG00000082556	ENSG00000082556	OPRK1	8:54090130-54164257	q1	q2	OK	1.9405	1.75147	-0.147869	-0.217034	0.74435	0.99995	no

ENSG00000082641	ENSG00000082641	NFE2L1	17:46125690-46138849	q1	q2	OK	80.4359	77.4264	-0.0550138	-0.0909849	0.8578	0.99995	no

ENSG00000082684	ENSG00000082684	SEMA5B	3:122628040-122747452	q1	q2	OK	135.799	154.465	0.185804	0.353931	0.5892	0.99995	no

ENSG00000082701	ENSG00000082701	GSK3B	3:119540169-119813264	q1	q2	OK	27.6171	28.4112	0.0408953	0.0753085	0.88565	0.99995	no

ENSG00000082781	ENSG00000082781	ITGB5	3:124480794-124620265	q1	q2	OK	81.7163	76.2766	-0.0993832	-0.157551	0.77915	0.99995	no

ENSG00000082805	ENSG00000082805	ERC1	12:1021242-1605099	q1	q2	OK	8.30549	9.9833	0.265452	0.274099	0.49855	0.99995	no

ENSG00000082898	ENSG00000082898	XPO1	2:61698322-61765761	q1	q2	OK	123.198	129.996	0.077482	0.105788	0.7915	0.99995	no

ENSG00000082929	ENSG00000082929	C4orf6	4:5526295-5529528	q1	q2	NOTEST	0.0352642	0.0508933	0.529272	0	1	1	no

ENSG00000082996	ENSG00000082996	RNF13	3:149478891-149942977	q1	q2	OK	22.5253	20.5713	-0.130918	-0.0846079	0.8738	0.99995	no

ENSG00000083067	ENSG00000083067	TRPM3	9:73143978-74061820	q1	q2	OK	1.25263	1.15019	-0.123092	-0.115216	0.80035	0.99995	no

ENSG00000083093	ENSG00000083093	PALB2	16:23614487-23652631	q1	q2	OK	9.37662	10.1242	0.110665	0.159209	0.7644	0.99995	no

ENSG00000083097	ENSG00000083097	DOPEY1	6:83777384-83908651	q1	q2	OK	10.0006	11.1973	0.163064	0.112478	0.77325	0.99995	no

ENSG00000083099	ENSG00000083099	LYRM2	6:90142888-90348472	q1	q2	OK	28.8428	31.7734	0.139609	0.174362	0.75685	0.99995	no

ENSG00000083123	ENSG00000083123	BCKDHB	6:80816363-81055987	q1	q2	OK	10.6145	11.8056	0.153428	0.283449	0.67255	0.99995	no

ENSG00000083168	ENSG00000083168	KAT6A	8:41786996-41909508	q1	q2	OK	21.0892	21.6898	0.0405122	0.0747423	0.8877	0.99995	no

ENSG00000083223	ENSG00000083223	ZCCHC6	9:88902647-88969369	q1	q2	OK	6.45747	6.71276	0.0559386	0.0889074	0.8672	0.99995	no

ENSG00000083290	ENSG00000083290	ULK2	17:19674141-19771249	q1	q2	OK	8.94548	7.39848	-0.27393	-0.399773	0.3983	0.99995	no

ENSG00000083307	ENSG00000083307	GRHL2	8:102473404-102681954	q1	q2	NOTEST	0.0433959	0.051486	0.24662	0	1	1	no

ENSG00000083312	ENSG00000083312	TNPO1	5:72090231-72212560	q1	q2	OK	105.038	125.385	0.255445	0.262951	0.513	0.99995	no

ENSG00000083444	ENSG00000083444	PLOD1	1:11994261-12035595	q1	q2	OK	35.6797	32.5406	-0.132862	-0.260233	0.66425	0.99995	no

ENSG00000083454	ENSG00000083454	P2RX5	17:3566195-3599698	q1	q2	OK	0.948998	0.752473	-0.334766	-0.0726677	0.77145	0.99995	no

ENSG00000083457	ENSG00000083457	ITGAE	17:3617921-3704537	q1	q2	OK	42.7402	43.0014	0.00879251	0.0138053	0.9846	0.99995	no

ENSG00000083520	ENSG00000083520	DIS3	13:73302060-73590591	q1	q2	OK	18.9765	20.9917	0.145602	0.261828	0.7097	0.99995	no

ENSG00000083535	ENSG00000083535	PIBF1	13:73302060-73590591	q1	q2	OK	11.0432	10.4868	-0.0745817	-0.0596756	0.91265	0.99995	no

ENSG00000083544	ENSG00000083544	TDRD3	13:60970590-61148012	q1	q2	OK	11.227	10.837	-0.0510035	-0.0801351	0.88745	0.99995	no

ENSG00000083622	ENSG00000083622	AC000111.6	7:117105837-117514193	q1	q2	NOTEST	0	0	0	0	1	1	no

ENSG00000083635	ENSG00000083635	NUFIP1	13:45513383-45657260	q1	q2	OK	6.24795	7.18602	0.20181	0.273809	0.6954	0.99995	no

ENSG00000083642	ENSG00000083642	PDS5B	13:33160563-33352157	q1	q2	OK	37.1706	39.7865	0.0981177	0.140963	0.82615	0.99995	no

ENSG00000083720	ENSG00000083720	OXCT1	5:41730166-41871059	q1	q2	OK	17.2801	18.3423	0.0860593	0.145703	0.78555	0.99995	no

ENSG00000083750	ENSG00000083750	RRAGB	X:55744171-55785207	q1	q2	OK	11.7696	9.78862	-0.265884	-0.327133	0.53835	0.99995	no

ENSG00000083782	ENSG00000083782	EPYC	12:91357455-91398803	q1	q2	NOTEST	0.0177225	0.0394269	1.1536	0	1	1	no

ENSG00000083799	ENSG00000083799	CYLD	16:50774820-50837249	q1	q2	OK	6.56213	5.74033	-0.193032	-0.250446	0.61975	0.99995	no

ENSG00000083807	ENSG00000083807	SLC27A5	19:58985383-59023780	q1	q2	OK	5.07654	3.91567	-0.374587	-0.24866	0.7258	0.99995	no

ENSG00000083812	ENSG00000083812	ZNF324	19:58978422-58984945	q1	q2	OK	5.2635	4.56255	-0.206181	-0.358592	0.6052	0.99995	no

ENSG00000083814	ENSG00000083814	ZNF671	19:58193336-58269527	q1	q2	OK	4.37186	3.77012	-0.213639	-0.134365	0.8417	0.99995	no

ENSG00000083817	ENSG00000083817	ZNF416	19:58082933-58105145	q1	q2	OK	4.31032	4.44818	0.0454207	0.0474816	0.94445	0.99995	no

ENSG00000083828	ENSG00000083828	ZNF586	19:58281022-58427978	q1	q2	OK	6.11665	6.17736	0.0142493	0.00745083	0.98765	0.99995	no

ENSG00000083838	ENSG00000083838	ZNF446	19:58985383-59023780	q1	q2	OK	7.71355	6.32218	-0.286973	-0.403762	0.5155	0.99995	no

ENSG00000083842	ENSG00000083842	ZNF8	19:58739959-58827023	q1	q2	OK	1.20218	1.19252	-0.0116388	-0.00439755	0.8467	0.99995	no

ENSG00000083844	ENSG00000083844	ZNF264	19:57702867-57724724	q1	q2	OK	4.26492	5.22449	0.292772	0.409313	0.49915	0.99995	no

ENSG00000083845	ENSG00000083845	RPS5	19:58897766-58906173	q1	q2	OK	567.158	531.19	-0.0945243	-0.227728	0.7473	0.99995	no

ENSG00000083857	ENSG00000083857	FAT1	4:187508936-187647876	q1	q2	OK	75.6516	72.9293	-0.0528703	-0.056593	0.92145	0.99995	no

ENSG00000083896	ENSG00000083896	YTHDC1	4:69176104-69215807	q1	q2	NOTEST	0	0.0247777	inf	0	1	1	no

ENSG00000083937	ENSG00000083937	CHMP2B	3:87276420-87304698	q1	q2	OK	14.8295	15.5208	0.0657303	0.125961	0.8422	0.99995	no

ENSG00000084070	ENSG00000084070	SMAP2	1:40810521-40888998	q1	q2	OK	19.1358	18.3572	-0.0599304	-0.112483	0.84805	0.99995	no

ENSG00000084072	ENSG00000084072	PPIE	1:40157853-40254533	q1	q2	OK	34.6139	34.2072	-0.0170506	-0.0347815	0.96125	0.99995	no

ENSG00000084073	ENSG00000084073	ZMPSTE24	1:40723778-40759856	q1	q2	OK	25.8322	25.8349	0.000152539	0.000336585	0.99955	0.99995	no

ENSG00000084090	ENSG00000084090	STARD7	2:96850596-96908359	q1	q2	OK	60.7228	61.9455	0.0287608	0.0436901	0.91885	0.99995	no

ENSG00000084092	ENSG00000084092	NOA1	4:57829535-58071676	q1	q2	OK	6.07016	5.95225	-0.0282995	-0.0147694	0.98435	0.99995	no

ENSG00000084093	ENSG00000084093	REST	4:57774074-57802010	q1	q2	OK	12.4475	13.0776	0.0712417	0.136601	0.8029	0.99995	no

ENSG00000084110	ENSG00000084110	HAL	12:96366439-96390143	q1	q2	NOTEST	0.0757246	0.146432	0.951399	0	1	1	no

ENSG00000084112	ENSG00000084112	SSH1	12:109176465-109251366	q1	q2	OK	7.33467	7.92392	0.111482	0.209865	0.69145	0.99995	no

ENSG00000084207	ENSG00000084207	GSTP1	11:67351065-67354131	q1	q2	OK	398.076	334.192	-0.252367	-0.599591	0.38955	0.99995	no

ENSG00000084234	ENSG00000084234	APLP2	11:129936835-130014699	q1	q2	OK	137.614	137.617	2.77E-05	6.09E-05	0.99995	0.99995	no

ENSG00000084444	ENSG00000084444	KIAA1467	12:13197217-13295455	q1	q2	OK	8.16018	9.06151	0.15115	0.319009	0.63845	0.99995	no

ENSG00000084453	ENSG00000084453	SLCO1A2	12:21417533-21572528	q1	q2	NOTEST	0.0358741	0.0337868	-0.086481	0	1	1	no

ENSG00000084463	ENSG00000084463	WBP11	12:14939409-14956474	q1	q2	OK	52.3883	54.0105	0.0439934	0.10746	0.87125	0.99995	no

ENSG00000084623	ENSG00000084623	EIF3I	1:32674694-32697205	q1	q2	OK	95.1554	97.1452	0.0298562	0.0691259	0.91685	0.99995	no

ENSG00000084628	ENSG00000084628	NKAIN1	1:31652591-31712401	q1	q2	OK	27.6288	27.2968	-0.0174416	-0.0395369	0.95365	0.99995	no

ENSG00000084636	ENSG00000084636	COL16A1	1:32083286-32169920	q1	q2	OK	5.98594	4.72116	-0.342439	-0.197347	0.7565	0.99995	no

ENSG00000084652	ENSG00000084652	TXLNA	1:32645286-32663886	q1	q2	OK	22.5959	22.8323	0.0150142	0.0370267	0.95705	0.99995	no

ENSG00000084674	ENSG00000084674	APOB	2:21224300-21266945	q1	q2	NOTEST	0	0.00329399	inf	0	1	1	no

ENSG00000084676	ENSG00000084676	NCOA1	2:24714782-24993571	q1	q2	OK	11.5806	11.8842	0.0373363	0.0613914	0.8959	0.99995	no

ENSG00000084693	ENSG00000084693	AGBL5	2:27265231-27294641	q1	q2	OK	33.5029	30.1111	-0.15399	-0.193391	0.7233	0.99995	no

ENSG00000084710	ENSG00000084710	EFR3B	2:25264998-25378243	q1	q2	OK	7.7878	7.76901	-0.00348563	-0.00696519	0.9921	0.99995	no

ENSG00000084731	ENSG00000084731	KIF3C	2:26149470-26205618	q1	q2	OK	19.016	17.9601	-0.0824202	-0.0879885	0.78645	0.99995	no

ENSG00000084733	ENSG00000084733	RAB10	2:26256975-26360323	q1	q2	OK	64.8343	73.5887	0.182726	0.324477	0.5737	0.99995	no

ENSG00000084734	ENSG00000084734	GCKR	2:27719708-27746554	q1	q2	NOTEST	0.237935	0.149307	-0.672286	0	1	1	no

ENSG00000084754	ENSG00000084754	HADHA	2:26413503-26513336	q1	q2	OK	58.0867	57.82	-0.00663842	-0.0122194	0.98345	0.99995	no

ENSG00000084764	ENSG00000084764	MAPRE3	2:27070614-27250064	q1	q2	OK	21.6889	17.5524	-0.305283	-0.13251	0.81155	0.99995	no

ENSG00000084774	ENSG00000084774	CAD	2:27440257-27466811	q1	q2	OK	15.4934	15.263	-0.0216177	-0.0273926	0.94645	0.99995	no

ENSG00000085063	ENSG00000085063	CD59	11:33719806-33757991	q1	q2	OK	61.7376	54.6849	-0.175007	-0.3836	0.5703	0.99995	no

ENSG00000085117	ENSG00000085117	CD82	11:44585976-44641913	q1	q2	OK	4.61926	3.90598	-0.241977	-0.288013	0.62765	0.99995	no

ENSG00000085185	ENSG00000085185	BCORL1	X:129115082-129192058	q1	q2	OK	5.46493	4.82151	-0.180718	-0.287451	0.63345	0.99995	no

ENSG00000085224	ENSG00000085224	ATRX	X:76760355-77041702	q1	q2	OK	0.780543	3.03233	1.95788	1.52201	0.02375	0.99995	no

ENSG00000085231	ENSG00000085231	TAF9	5:68646810-68710628	q1	q2	NOTEST	0.156109	0.309157	0.985783	0	1	1	no

ENSG00000085265	ENSG00000085265	FCN1	9:137801430-137809809	q1	q2	NOTEST	0	0	0	0	1	1	no

ENSG00000085274	ENSG00000085274	MYNN	3:169490618-169507504	q1	q2	OK	10.3022	9.98675	-0.0448654	-0.0848119	0.9094	0.99995	no

ENSG00000085276	ENSG00000085276	MECOM	3:168801286-169381406	q1	q2	OK	0.743397	0.943415	0.343759	0.157517	0.62995	0.99995	no

ENSG00000085365	ENSG00000085365	SCAMP1	5:77656338-77776562	q1	q2	OK	12.6964	14.6209	0.203619	0.429526	0.50555	0.99995	no

ENSG00000085377	ENSG00000085377	PREP	6:105725439-105850959	q1	q2	OK	22.331	24.2312	0.117818	0.256157	0.69935	0.99995	no

ENSG00000085382	ENSG00000085382	HACE1	6:105175967-105307794	q1	q2	OK	14.1725	14.7268	0.0553468	0.102724	0.87905	0.99995	no

ENSG00000085415	ENSG00000085415	SEH1L	18:12947131-12991172	q1	q2	OK	20.5446	24.2837	0.24123	0.448394	0.4441	0.99995	no

ENSG00000085433	ENSG00000085433	WDR47	1:109512835-109584850	q1	q2	OK	9.84403	9.85801	0.00204714	0.00306204	0.9967	0.99995	no

ENSG00000085449	ENSG00000085449	WDFY1	2:224720432-224810104	q1	q2	OK	27.4415	31.4884	0.198462	0.31801	0.5015	0.99995	no

ENSG00000085465	ENSG00000085465	OVGP1	1:111956935-111970399	q1	q2	OK	1.31694	1.49867	0.186488	0.166873	0.77925	0.99995	no

ENSG00000085491	ENSG00000085491	SLC25A24	1:108676657-108744113	q1	q2	OK	26.4527	32.8087	0.310661	0.743478	0.28255	0.99995	no

ENSG00000085511	ENSG00000085511	MAP3K4	6:161411349-161538417	q1	q2	OK	19.1983	18.6528	-0.0415824	-0.0442769	0.90525	0.99995	no

ENSG00000085514	ENSG00000085514	PILRA	7:99905324-99997719	q1	q2	OK	1.40369	1.06169	-0.402864	-0.0973194	0.70815	0.99995	no

ENSG00000085552	ENSG00000085552	IGSF9	1:159896828-159915386	q1	q2	OK	10.4089	9.50944	-0.13038	-0.279223	0.6957	0.99995	no

ENSG00000085563	ENSG00000085563	ABCB1	7:87133174-87461611	q1	q2	OK	1.92757	2.32083	0.267856	0.193364	0.674	0.99995	no

ENSG00000085644	ENSG00000085644	ZNF213	16:3160460-3192806	q1	q2	OK	7.73358	6.48779	-0.25341	-0.265862	0.59695	0.99995	no

ENSG00000085662	ENSG00000085662	AKR1B1	7:134127101-134144036	q1	q2	OK	97.3759	89.4754	-0.122075	-0.281559	0.6581	0.99995	no

ENSG00000085719	ENSG00000085719	CPNE3	8:87345502-87777251	q1	q2	OK	19.2856	21.6753	0.168528	0.243825	0.65385	0.99995	no

ENSG00000085721	ENSG00000085721	RRN3	16:15068447-15248421	q1	q2	OK	20.0242	19.7296	-0.0213815	-0.0259595	0.9636	0.99995	no

ENSG00000085733	ENSG00000085733	CTTN	11:70116805-70282690	q1	q2	OK	70.3762	69.7242	-0.013428	-0.0190479	0.97155	0.99995	no

ENSG00000085741	ENSG00000085741	WNT11	11:75897368-75921780	q1	q2	OK	2.91774	2.22359	-0.39196	-0.475361	0.46315	0.99995	no

ENSG00000085760	ENSG00000085760	MTIF2	2:55463730-55496483	q1	q2	OK	14.8403	15.0549	0.0207106	0.039729	0.94935	0.99995	no

ENSG00000085788	ENSG00000085788	DDHD2	8:38082735-38239790	q1	q2	OK	39.4943	43.2031	0.129491	0.0898384	0.7725	0.99995	no

ENSG00000085831	ENSG00000085831	TTC39A	1:51752929-51810788	q1	q2	OK	0.51267	0.214888	-1.25445	-0.526285	0.04215	0.99995	no

ENSG00000085832	ENSG00000085832	EPS15	1:51819934-52027301	q1	q2	OK	18.5286	20.7566	0.163817	0.278771	0.6617	0.99995	no

ENSG00000085840	ENSG00000085840	ORC1	1:52838500-52870131	q1	q2	OK	12.4548	12.0706	-0.0452111	-0.0954498	0.89015	0.99995	no

ENSG00000085871	ENSG00000085871	MGST2	4:140586921-141075338	q1	q2	OK	9.33972	10.0705	0.108687	0.0720144	0.9109	0.99995	no

ENSG00000085872	ENSG00000085872	CHERP	19:16589867-16771253	q1	q2	OK	31.6572	28.932	-0.129869	-0.124051	0.78365	0.99995	no

ENSG00000085978	ENSG00000085978	ATG16L1	2:234118696-234204320	q1	q2	OK	9.48546	9.82625	0.0509228	0.0818462	0.8785	0.99995	no

ENSG00000085982	ENSG00000085982	USP40	2:234384165-234475428	q1	q2	OK	15.7191	15.1687	-0.0514199	-0.0800314	0.87295	0.99995	no

ENSG00000085998	ENSG00000085998	POMGNT1	1:46654353-46686933	q1	q2	OK	34.0611	31.3607	-0.119164	-0.210104	0.7159	0.99995	no

ENSG00000085999	ENSG00000085999	RAD54L	1:46713359-46769280	q1	q2	OK	19.8688	18.9336	-0.0695535	-0.0806046	0.896	0.99995	no

ENSG00000086015	ENSG00000086015	MAST2	1:46252658-46501796	q1	q2	OK	19.5117	17.387	-0.166326	-0.182757	0.63415	0.99995	no

ENSG00000086061	ENSG00000086061	DNAJA1	9:33025208-33039905	q1	q2	OK	263.034	281.559	0.0981836	0.215401	0.75375	0.99995	no

ENSG00000086062	ENSG00000086062	B4GALT1	9:33104079-33179981	q1	q2	OK	4.44583	5.14825	0.211631	0.387696	0.5737	0.99995	no

ENSG00000086065	ENSG00000086065	CHMP5	9:33264939-33281977	q1	q2	OK	45.2304	47.5193	0.0712195	0.162332	0.8187	0.99995	no

ENSG00000086102	ENSG00000086102	NFX1	9:33290508-33371155	q1	q2	OK	17.2115	16.5205	-0.0591153	-0.115024	0.85075	0.99995	no

ENSG00000086159	ENSG00000086159	AQP6	12:50360976-50426919	q1	q2	NOTEST	0.0561092	0.00500923	-3.48558	0	1	1	no

ENSG00000086189	ENSG00000086189	DIMT1	5:61601988-61924409	q1	q2	OK	19.6185	22.0522	0.168709	0.171222	0.76295	0.99995	no

ENSG00000086200	ENSG00000086200	IPO11	5:61601988-61924409	q1	q2	OK	13.2235	15.3686	0.216882	0.216233	0.73425	0.99995	no

ENSG00000086205	ENSG00000086205	FOLH1	11:49168186-49230222	q1	q2	OK	58.5918	60.5497	0.0474217	0.0805028	0.87875	0.99995	no

ENSG00000086232	ENSG00000086232	EIF2AK1	7:6048875-6098861	q1	q2	OK	50.9751	51.709	0.0206237	0.0350544	0.9522	0.99995	no

ENSG00000086288	ENSG00000086288	NME8	7:37723398-38065297	q1	q2	NOTEST	0.0977038	0.0116796	-3.06442	0	1	1	no

ENSG00000086289	ENSG00000086289	EPDR1	7:37723398-38065297	q1	q2	OK	3.75719	3.1323	-0.262434	-0.360828	0.5757	0.99995	no

ENSG00000086300	ENSG00000086300	SNX10	7:26331540-26416321	q1	q2	OK	2.89336	3.03908	0.0708856	0.0762795	0.89945	0.99995	no

ENSG00000086475	ENSG00000086475	SEPHS1	10:13359423-13390297	q1	q2	OK	94.1542	91.7096	-0.0379521	-0.0685917	0.90895	0.99995	no

ENSG00000086504	ENSG00000086504	MRPL28	16:417383-420527	q1	q2	OK	56.719	49.6248	-0.192769	-0.427086	0.5396	0.99995	no

ENSG00000086506	ENSG00000086506	HBQ1	16:230451-231180	q1	q2	OK	0.594075	1.06854	0.846927	0.613185	0.38135	0.99995	no

ENSG00000086544	ENSG00000086544	ITPKC	19:41197433-41271294	q1	q2	OK	4.7725	4.22925	-0.174341	-0.111622	0.86845	0.99995	no

ENSG00000086548	ENSG00000086548	CEACAM6	19:42212503-42290801	q1	q2	NOTEST	0	0	0	0	1	1	no

ENSG00000086570	ENSG00000086570	FAT2	5:150883653-150948505	q1	q2	NOTEST	0.106783	0.110079	0.0438593	0	1	1	no

ENSG00000086589	ENSG00000086589	RBM22	5:150070355-150080669	q1	q2	OK	40.1607	40.7462	0.0208796	0.0474965	0.94115	0.99995	no

ENSG00000086598	ENSG00000086598	TMED2	12:124066766-124083116	q1	q2	OK	84.8712	89.852	0.0822753	0.186219	0.7778	0.99995	no

ENSG00000086619	ENSG00000086619	ERO1LB	1:236305831-236445319	q1	q2	OK	2.16143	1.54408	-0.485234	-0.49087	0.439	0.99995	no

ENSG00000086666	ENSG00000086666	ZFAND6	15:80351909-80430735	q1	q2	OK	83.3974	84.3788	0.0168778	0.0372015	0.95445	0.99995	no

ENSG00000086696	ENSG00000086696	HSD17B2	16:82068608-82173236	q1	q2	NOTEST	0.0623272	0.0849219	0.446275	0	1	1	no

ENSG00000086712	ENSG00000086712	TXLNG	X:16804549-16888537	q1	q2	OK	7.37382	7.51609	0.0275694	0.0257904	0.96955	0.99995	no

ENSG00000086717	ENSG00000086717	PPEF1	X:18694028-18846039	q1	q2	OK	0.804281	0.795743	-0.0153977	-0.00473337	0.98645	0.99995	no

ENSG00000086730	ENSG00000086730	LAT2	7:73613981-73644161	q1	q2	NOTEST	0.207376	0.0276033	-2.90934	0	1	1	no

ENSG00000086758	ENSG00000086758	HUWE1	X:53559056-53713673	q1	q2	OK	17.4036	13.8083	-0.333852	-0.38564	0.557	0.99995	no

ENSG00000086827	ENSG00000086827	ZW10	11:113603908-113644533	q1	q2	OK	15.3149	16.1285	0.074676	0.164656	0.81705	0.99995	no

ENSG00000086848	ENSG00000086848	ALG9	11:111652918-111756699	q1	q2	NOTEST	0	0.0384346	inf	0	1	1	no

ENSG00000086967	ENSG00000086967	MYBPC2	19:50936159-50969578	q1	q2	NOTEST	0.0294866	0.104484	1.82515	0	1	1	no

ENSG00000086991	ENSG00000086991	NOX4	11:89057523-89322779	q1	q2	OK	1.30135	1.49637	0.20145	0.157982	0.81675	0.99995	no

ENSG00000087008	ENSG00000087008	ACOX3	4:8368008-8514337	q1	q2	OK	6.08478	5.34646	-0.186623	-0.272778	0.69685	0.99995	no

ENSG00000087053	ENSG00000087053	MTMR2	11:95566045-95658479	q1	q2	OK	25.3574	29.9129	0.238362	0.507776	0.4019	0.99995	no

ENSG00000087074	ENSG00000087074	PPP1R15A	19:49375648-49379314	q1	q2	OK	16.48	15.7318	-0.0670372	-0.142544	0.8394	0.99995	no

ENSG00000087076	ENSG00000087076	HSD17B14	19:49316273-49339935	q1	q2	OK	2.72515	2.37045	-0.201176	-0.225927	0.73995	0.99995	no

ENSG00000087077	ENSG00000087077	TRIP6	7:100464759-100471076	q1	q2	OK	17.815	15.6004	-0.191506	-0.325875	0.6059	0.99995	no

ENSG00000087085	ENSG00000087085	ACHE	7:100487614-100494594	q1	q2	OK	1.39666	0.899062	-0.635493	-0.538114	0.3319	0.99995	no

ENSG00000087086	ENSG00000087086	FTL	19:49468557-49470135	q1	q2	OK	560.772	457.109	-0.294878	-0.698117	0.3191	0.99995	no

ENSG00000087087	ENSG00000087087	SRRT	7:100472732-100486285	q1	q2	OK	94.1176	99.9466	0.0866927	0.128457	0.78745	0.99995	no

ENSG00000087088	ENSG00000087088	BAX	19:49458071-49465055	q1	q2	OK	117.35	97.2371	-0.271236	-0.587965	0.38585	0.99995	no

ENSG00000087095	ENSG00000087095	NLK	17:26368762-26523407	q1	q2	OK	13.054	13.9658	0.0974076	0.200799	0.7558	0.99995	no

ENSG00000087111	ENSG00000087111	PIGS	17:26782769-26972472	q1	q2	OK	24.5172	22.1868	-0.144095	-0.0829766	0.90775	0.99995	no

ENSG00000087116	ENSG00000087116	ADAMTS2	5:178537851-178772431	q1	q2	OK	1.82226	1.38425	-0.396628	-0.229285	0.5835	0.99995	no

ENSG00000087128	ENSG00000087128	TMPRSS11E	4:69313166-69363322	q1	q2	NOTEST	0	0	0	0	1	1	no

ENSG00000087152	ENSG00000087152	ATXN7L3	17:42269172-42301078	q1	q2	OK	39.9973	39.1644	-0.0303589	-0.0309112	0.93885	0.99995	no

ENSG00000087157	ENSG00000087157	PGS1	17:76374720-76573476	q1	q2	OK	19.2679	20.0752	0.0592135	0.111578	0.86295	0.99995	no

ENSG00000087191	ENSG00000087191	PSMC5	17:61896792-61909379	q1	q2	OK	128.529	129.098	0.00636934	0.0112185	0.9857	0.99995	no

ENSG00000087206	ENSG00000087206	UIMC1	5:176332005-176449634	q1	q2	OK	19.0683	19.6538	0.0436343	0.0733422	0.895	0.99995	no

ENSG00000087237	ENSG00000087237	CETP	16:56995761-57017757	q1	q2	NOTEST	0.0177481	0	#NAME?	0	1	1	no

ENSG00000087245	ENSG00000087245	MMP2	16:55423611-55540603	q1	q2	OK	72.3875	71.921	-0.00932855	-0.0199709	0.9754	0.99995	no

ENSG00000087250	ENSG00000087250	MT3	16:56622985-56625000	q1	q2	NOTEST	0.320073	0.262115	-0.288203	0	1	1	no

ENSG00000087253	ENSG00000087253	LPCAT2	16:55542909-55620582	q1	q2	OK	1.74653	1.96416	0.169419	0.159045	0.70835	0.99995	no

ENSG00000087258	ENSG00000087258	GNAO1	16:56225301-56391356	q1	q2	OK	33.4381	29.8663	-0.162973	-0.179114	0.77405	0.99995	no

ENSG00000087263	ENSG00000087263	OGFOD1	16:56463044-56554195	q1	q2	OK	26.1163	26.57	0.0248474	0.0162019	0.979	0.99995	no

ENSG00000087266	ENSG00000087266	SH3BP2	4:2794749-2842825	q1	q2	OK	10.1329	10.4921	0.0502584	0.0625848	0.89415	0.99995	no

ENSG00000087269	ENSG00000087269	NOP14	4:2936625-2965112	q1	q2	OK	12.1035	13.4027	0.1471	0.292574	0.67445	0.99995	no

ENSG00000087274	ENSG00000087274	ADD1	4:2845583-2931803	q1	q2	OK	113.76	105.261	-0.112031	-0.182595	0.7005	0.99995	no

ENSG00000087299	ENSG00000087299	L2HGDH	14:50704280-50883179	q1	q2	OK	3.57551	3.22467	-0.149	-0.139612	0.8218	0.99995	no

ENSG00000087301	ENSG00000087301	TXNDC16	14:52897307-53019240	q1	q2	OK	7.08927	7.75146	0.12883	0.205264	0.70055	0.99995	no

ENSG00000087302	ENSG00000087302	C14orf166	14:52456192-52471420	q1	q2	OK	144.431	149.364	0.0484492	0.118194	0.8587	0.99995	no

ENSG00000087303	ENSG00000087303	NID2	14:52471520-52535712	q1	q2	OK	34.7983	37.6884	0.115106	0.172084	0.79605	0.99995	no

ENSG00000087338	ENSG00000087338	GMCL1	2:70056773-70108528	q1	q2	OK	10.13	10.939	0.110848	0.222182	0.72205	0.99995	no

ENSG00000087365	ENSG00000087365	SF3B2	11:65818199-65837090	q1	q2	OK	125.419	118.863	-0.077457	-0.15881	0.79845	0.99995	no

ENSG00000087448	ENSG00000087448	KLHL42	12:27932753-27955973	q1	q2	OK	14.1692	16.6521	0.232942	0.382967	0.4659	0.99995	no

ENSG00000087460	ENSG00000087460	GNAS	20:57393973-57486247	q1	q2	OK	921.9	864.105	-0.0934047	-0.168749	0.80605	0.99995	no

ENSG00000087470	ENSG00000087470	DNM1L	12:32832133-32908836	q1	q2	OK	67.2424	72.813	0.114824	0.158015	0.7354	0.99995	no

ENSG00000087494	ENSG00000087494	PTHLH	12:28111016-28125638	q1	q2	OK	0.520022	0.506705	-0.0374266	-0.0197535	0.9618	0.99995	no

ENSG00000087495	ENSG00000087495	PHACTR3	20:58152563-58422766	q1	q2	OK	5.07598	5.17053	0.0266247	0.0423121	0.95335	0.99995	no

ENSG00000087502	ENSG00000087502	ERGIC2	12:29302035-29534122	q1	q2	OK	38.5782	41.9987	0.12256	0.163217	0.82105	0.99995	no

ENSG00000087510	ENSG00000087510	TFAP2C	20:55204357-55214339	q1	q2	NOTEST	0.278627	0.239786	-0.216591	0	1	1	no

ENSG00000087586	ENSG00000087586	AURKA	20:54944444-54967393	q1	q2	OK	29.8755	34.2254	0.196101	0.434479	0.53075	0.99995	no

ENSG00000087589	ENSG00000087589	CASS4	20:54987167-55034396	q1	q2	NOTEST	0.25772	0.339856	0.39912	0	1	1	no

ENSG00000087842	ENSG00000087842	PIR	X:15402920-15574652	q1	q2	OK	9.8493	9.25012	-0.0905487	-0.138673	0.8332	0.99995	no

ENSG00000087884	ENSG00000087884	AAMDC	11:77532154-77705724	q1	q2	OK	17.0704	15.82	-0.109746	-0.0879336	0.908	0.99995	no

ENSG00000087903	ENSG00000087903	RFX2	19:5978413-6199583	q1	q2	OK	9.06322	8.65289	-0.0668417	-0.0618032	0.85165	0.99995	no

ENSG00000087916	ENSG00000087916	SLC6A14	X:115567789-115592625	q1	q2	NOTEST	0	0	0	0	1	1	no

ENSG00000087995	ENSG00000087995	METTL2A	17:60501227-60527454	q1	q2	OK	11.5246	12.7367	0.144276	0.246023	0.6998	0.99995	no

ENSG00000088002	ENSG00000088002	SULT2B1	19:49055331-49102682	q1	q2	NOTEST	0.0216704	0.135537	2.64489	0	1	1	no

ENSG00000088035	ENSG00000088035	ALG6	1:63833260-63904233	q1	q2	OK	7.58695	8.17923	0.108445	0.16729	0.8139	0.99995	no

ENSG00000088038	ENSG00000088038	CNOT3	19:54641443-54663620	q1	q2	NOTEST	0	0	0	0	1	1	no

ENSG00000088053	ENSG00000088053	GP6	19:55517560-55582659	q1	q2	NOTEST	0	0	0	0	1	1	no

ENSG00000088179	ENSG00000088179	PTPN4	2:120517206-120741394	q1	q2	OK	7.33221	8.31596	0.181636	0.168568	0.67295	0.99995	no

ENSG00000088205	ENSG00000088205	DDX18	2:118572225-118589955	q1	q2	OK	36.7266	41.7298	0.184252	0.384126	0.5161	0.99995	no

ENSG00000088247	ENSG00000088247	KHSRP	19:6413358-6424805	q1	q2	OK	204.064	200.377	-0.0263082	-0.0495407	0.9381	0.99995	no

ENSG00000088256	ENSG00000088256	GNA11	19:3094407-3124002	q1	q2	OK	17.3551	18.3895	0.0835244	0.139562	0.7922	0.99995	no

ENSG00000088280	ENSG00000088280	ASAP3	1:23755055-23811061	q1	q2	OK	23.3714	23.3031	-0.00421835	-0.00809693	0.9901	0.99995	no

ENSG00000088298	ENSG00000088298	EDEM2	20:33703166-33872788	q1	q2	OK	5.02602	4.87694	-0.0434418	-0.0302945	0.96475	0.99995	no

ENSG00000088305	ENSG00000088305	DNMT3B	20:31350190-31397162	q1	q2	OK	35.8412	39.4039	0.13672	0.328206	0.6349	0.99995	no

ENSG00000088320	ENSG00000088320	REM1	20:30053308-30072708	q1	q2	NOTEST	0.0503712	0.0482965	-0.0606794	0	1	1	no

ENSG00000088325	ENSG00000088325	TPX2	20:30327073-30389608	q1	q2	OK	81.2508	86.2046	0.0853819	0.206566	0.76165	0.99995	no

ENSG00000088340	ENSG00000088340	FER1L4	20:34146506-34195484	q1	q2	NOTEST	0.368801	0.472431	0.357262	0	1	1	no

ENSG00000088356	ENSG00000088356	PDRG1	20:30435439-30539895	q1	q2	OK	22.8303	20.8817	-0.128705	-0.249605	0.7233	0.99995	no

ENSG00000088367	ENSG00000088367	EPB41L1	20:34679425-34820721	q1	q2	OK	18.017	16.0805	-0.164046	-0.243741	0.6448	0.99995	no

ENSG00000088386	ENSG00000088386	SLC15A1	13:99336054-99404908	q1	q2	NOTEST	0.00816557	0	#NAME?	0	1	1	no

ENSG00000088387	ENSG00000088387	DOCK9	13:99445740-99738879	q1	q2	OK	7.68486	8.22159	0.0973981	0.0896696	0.8261	0.99995	no

ENSG00000088448	ENSG00000088448	ANKRD10	13:111530886-111567416	q1	q2	OK	134.233	131.661	-0.027907	-0.0452557	0.9422	0.99995	no

ENSG00000088451	ENSG00000088451	TGDS	13:95226307-95248511	q1	q2	OK	5.69875	7.5278	0.401583	0.609566	0.3616	0.99995	no

ENSG00000088538	ENSG00000088538	DOCK3	3:50712671-51421629	q1	q2	OK	0.493063	0.405222	-0.283061	-0.348263	0.61045	0.99995	no

ENSG00000088543	ENSG00000088543	C3orf18	3:50595461-50622366	q1	q2	OK	4.70227	3.9851	-0.238742	-0.183659	0.70095	0.99995	no

ENSG00000088682	ENSG00000088682	COQ9	16:57462080-57521239	q1	q2	OK	31.9531	31.5393	-0.0188016	-0.017335	0.97725	0.99995	no

ENSG00000088726	ENSG00000088726	TMEM40	3:12775023-12810956	q1	q2	NOTEST	0	0	0	0	1	1	no

ENSG00000088727	ENSG00000088727	KIF9	3:47205986-47388306	q1	q2	OK	5.98056	5.79517	-0.0454292	-0.0255766	0.9708	0.99995	no

ENSG00000088756	ENSG00000088756	ARHGAP28	18:6728819-6915715	q1	q2	OK	6.04044	6.59567	0.126867	0.179997	0.71675	0.99995	no

ENSG00000088766	ENSG00000088766	CRLS1	20:5986735-6020699	q1	q2	OK	26.6419	28.7815	0.111444	0.180884	0.7955	0.99995	no

ENSG00000088782	ENSG00000088782	DEFB127	20:138110-139804	q1	q2	NOTEST	0	0	0	0	1	1	no

ENSG00000088808	ENSG00000088808	PPP1R13B	14:104200088-104313927	q1	q2	OK	3.72454	3.15386	-0.239941	-0.212096	0.5893	0.99995	no

ENSG00000088812	ENSG00000088812	ATRN	20:3451686-3631769	q1	q2	OK	13.8332	13.5733	-0.0273635	-0.0654973	0.92725	0.99995	no

ENSG00000088826	ENSG00000088826	SMOX	20:4101626-4168394	q1	q2	OK	10.8016	9.63965	-0.164198	-0.284983	0.66045	0.99995	no

ENSG00000088827	ENSG00000088827	SIGLEC1	20:3667616-3687775	q1	q2	NOTEST	0.0115021	0.0111306	-0.0473711	0	1	1	no

ENSG00000088832	ENSG00000088832	FKBP1A	20:1290618-1373806	q1	q2	OK	105.549	111.025	0.0729762	0.152173	0.8283	0.99995	no

ENSG00000088833	ENSG00000088833	NSFL1C	20:1422806-1472233	q1	q2	OK	60.129	56.6664	-0.0855676	-0.166197	0.79745	0.99995	no

ENSG00000088836	ENSG00000088836	SLC4A11	20:3208062-3219836	q1	q2	OK	0.825014	0.555582	-0.570418	-0.445185	0.3721	0.99995	no

ENSG00000088854	ENSG00000088854	C20orf194	20:3229950-3388272	q1	q2	OK	9.15137	8.94708	-0.0325702	-0.0738454	0.9131	0.99995	no

ENSG00000088876	ENSG00000088876	ZNF343	20:2442279-2505348	q1	q2	OK	5.77969	6.24686	0.112137	0.0569796	0.9121	0.99995	no

ENSG00000088881	ENSG00000088881	EBF4	20:2673523-2740753	q1	q2	OK	12.8201	9.57742	-0.420696	-0.736307	0.2633	0.99995	no

ENSG00000088882	ENSG00000088882	CPXM1	20:2774714-2781283	q1	q2	OK	63.2103	57.2288	-0.143419	-0.354171	0.6141	0.99995	no

ENSG00000088888	ENSG00000088888	MAVS	20:3827486-3849280	q1	q2	OK	8.25684	7.56225	-0.126774	-0.248814	0.71025	0.99995	no

ENSG00000088899	ENSG00000088899	LZTS3	20:3143262-3154192	q1	q2	OK	6.55634	5.62072	-0.222137	-0.437882	0.5358	0.99995	no

ENSG00000088926	ENSG00000088926	F11	4:187187098-187476721	q1	q2	NOTEST	0.109727	0.0646322	-0.763588	0	1	1	no

ENSG00000088930	ENSG00000088930	XRN2	20:21283941-21370463	q1	q2	OK	53.206	57.2793	0.106426	0.263217	0.70305	0.99995	no

ENSG00000088970	ENSG00000088970	PLK1S1	20:21106623-21227260	q1	q2	OK	5.27315	4.16961	-0.338753	-0.476328	0.4418	0.99995	no

ENSG00000088986	ENSG00000088986	DYNLL1	12:120907652-120936296	q1	q2	OK	360.022	381.17	0.0823469	0.197547	0.7786	0.99995	no

ENSG00000088992	ENSG00000088992	TESC	12:117476727-117537284	q1	q2	NOTEST	0.230119	0.234122	0.0248838	0	1	1	no

ENSG00000089006	ENSG00000089006	SNX5	20:17922240-18039832	q1	q2	OK	67.1536	69.1175	0.0415852	0.0690586	0.9193	0.99995	no

ENSG00000089009	ENSG00000089009	RPL6	12:112842993-112947717	q1	q2	OK	908.525	898.02	-0.0167792	-0.030314	0.96865	0.99995	no

ENSG00000089012	ENSG00000089012	SIRPG	20:1609797-1638425	q1	q2	NOTEST	0.0907216	0.0368287	-1.30062	0	1	1	no

ENSG00000089022	ENSG00000089022	MAPKAPK5	12:112277570-112334343	q1	q2	OK	26.3137	27.8564	0.0821967	0.138133	0.8419	0.99995	no

ENSG00000089041	ENSG00000089041	P2RX7	12:121570621-121623876	q1	q2	NOTEST	0	0.0178822	inf	0	1	1	no

ENSG00000089048	ENSG00000089048	ESF1	20:13694968-13765532	q1	q2	OK	15.394	18.122	0.235375	0.536589	0.4498	0.99995	no

ENSG00000089050	ENSG00000089050	RBBP9	20:18467183-18477887	q1	q2	OK	13.1604	13.1679	0.00082087	0.00161	0.998	0.99995	no

ENSG00000089053	ENSG00000089053	ANAPC5	12:121746047-121837699	q1	q2	OK	34.8156	31.6046	-0.139602	-0.288567	0.67635	0.99995	no

ENSG00000089057	ENSG00000089057	SLC23A2	20:4833001-4990939	q1	q2	OK	11.8942	11.9292	0.00423756	0.00969539	0.98845	0.99995	no

ENSG00000089060	ENSG00000089060	SLC8B1	12:113736563-113827203	q1	q2	OK	3.97482	3.9059	-0.025233	-0.0229848	0.9666	0.99995	no

ENSG00000089063	ENSG00000089063	TMEM230	20:5080485-5093749	q1	q2	OK	77.4054	78.8567	0.0267988	0.0641487	0.92855	0.99995	no

ENSG00000089091	ENSG00000089091	DZANK1	20:18364010-18465287	q1	q2	OK	3.93024	3.67096	-0.0984625	-0.0972686	0.8932	0.99995	no

ENSG00000089094	ENSG00000089094	KDM2B	12:121837843-122031409	q1	q2	OK	4.87781	4.4728	-0.125057	-0.0773111	0.9051	0.99995	no

ENSG00000089101	ENSG00000089101	C20orf26	20:20015011-20341346	q1	q2	NOTEST	0.411258	0.362364	-0.182605	0	1	1	no

ENSG00000089116	ENSG00000089116	LHX5	12:113899838-113918429	q1	q2	OK	3.02641	3.0411	0.00698777	0.009645	0.9878	0.99995	no

ENSG00000089123	ENSG00000089123	TASP1	20:13202417-13619587	q1	q2	OK	2.64186	2.70435	0.0337288	0.0394025	0.94685	0.99995	no

ENSG00000089127	ENSG00000089127	OAS1	12:113344581-113455556	q1	q2	OK	4.45536	4.80548	0.109139	0.113807	0.85265	0.99995	no

ENSG00000089154	ENSG00000089154	GCN1L1	12:120565006-120632513	q1	q2	OK	26.9651	25.2693	-0.0937101	-0.0574836	0.79015	0.99995	no

ENSG00000089157	ENSG00000089157	RPLP0	12:120634488-120639038	q1	q2	OK	1068.52	992.145	-0.106986	-0.216929	0.75545	0.99995	no

ENSG00000089159	ENSG00000089159	PXN	12:120639093-120703574	q1	q2	OK	20.3022	19.6429	-0.0476274	-0.0898644	0.8772	0.99995	no

ENSG00000089163	ENSG00000089163	SIRT4	12:120740118-120751144	q1	q2	OK	2.13489	1.62564	-0.39315	-0.409315	0.5336	0.99995	no

ENSG00000089169	ENSG00000089169	RPH3A	12:113008183-113336686	q1	q2	OK	3.87565	5.14952	0.41	0.548043	0.2889	0.99995	no

ENSG00000089177	ENSG00000089177	KIF16B	20:16252748-16554078	q1	q2	OK	2.58132	2.63765	0.0311459	0.0386193	0.94395	0.99995	no

ENSG00000089195	ENSG00000089195	TRMT6	20:5917880-5931182	q1	q2	OK	12.1148	15.1446	0.322035	0.662352	0.34935	0.99995	no

ENSG00000089199	ENSG00000089199	CHGB	20:5892075-5906007	q1	q2	OK	3.08377	2.80688	-0.135729	-0.199925	0.77155	0.99995	no

ENSG00000089220	ENSG00000089220	PEBP1	12:118501397-118583389	q1	q2	OK	166.097	161	-0.0449604	-0.0960601	0.884	0.99995	no

ENSG00000089225	ENSG00000089225	TBX5	12:114791735-114850636	q1	q2	NOTEST	0.00738808	0	#NAME?	0	1	1	no

ENSG00000089234	ENSG00000089234	BRAP	12:112079949-112123790	q1	q2	OK	10.0594	10.8085	0.103615	0.205799	0.7664	0.99995	no

ENSG00000089248	ENSG00000089248	ERP29	12:112451119-112461255	q1	q2	OK	74.8884	71.1472	-0.0739342	-0.173311	0.79245	0.99995	no

ENSG00000089250	ENSG00000089250	NOS1	12:117645946-117889975	q1	q2	NOTEST	0.0118319	0.0179913	0.604615	0	1	1	no

ENSG00000089280	ENSG00000089280	FUS	16:31191430-31203127	q1	q2	OK	519.026	530.344	0.0311239	0.0502984	0.93805	0.99995	no

ENSG00000089289	ENSG00000089289	IGBP1	X:69353298-69386174	q1	q2	OK	25.8586	23.3235	-0.148863	-0.321461	0.64935	0.99995	no

ENSG00000089327	ENSG00000089327	FXYD5	19:35645632-35660786	q1	q2	OK	2.05872	1.23105	-0.741859	-0.401123	0.3703	0.99995	no

ENSG00000089335	ENSG00000089335	ZNF302	19:35168543-35177302	q1	q2	OK	35.0571	37.5279	0.098257	0.188983	0.7423	0.99995	no

ENSG00000089351	ENSG00000089351	GRAMD1A	19:35485687-35517375	q1	q2	OK	34.4672	34.8328	0.0152196	0.0233728	0.96815	0.99995	no

ENSG00000089356	ENSG00000089356	FXYD3	19:35606731-35615228	q1	q2	NOTEST	0.0682712	0	#NAME?	0	1	1	no

ENSG00000089472	ENSG00000089472	HEPH	X:65382390-65488709	q1	q2	OK	12.7244	11.9764	-0.0874051	-0.143073	0.8355	0.99995	no

ENSG00000089486	ENSG00000089486	CDIP1	16:4560675-4588829	q1	q2	OK	25.3525	20.0174	-0.340873	-0.735008	0.27665	0.99995	no

ENSG00000089505	ENSG00000089505	CMTM1	16:66586465-66613040	q1	q2	OK	2.54193	1.9232	-0.402418	-0.137079	0.669	0.99995	no

ENSG00000089558	ENSG00000089558	KCNH4	17:40308908-40333296	q1	q2	NOTEST	0.0560654	0.0536181	-0.0643918	0	1	1	no

ENSG00000089597	ENSG00000089597	GANAB	11:62392297-62414104	q1	q2	OK	144.907	141.088	-0.0385315	-0.0679014	0.89275	0.99995	no

ENSG00000089639	ENSG00000089639	GMIP	19:19740284-19754476	q1	q2	OK	10.9209	12.1206	0.150368	0.253708	0.67295	0.99995	no

ENSG00000089682	ENSG00000089682	RBM41	X:106307649-106362057	q1	q2	OK	8.16519	8.42648	0.0454446	0.07128	0.9054	0.99995	no

ENSG00000089685	ENSG00000089685	BIRC5	17:76210266-76221717	q1	q2	OK	67.8362	67.9413	0.00223282	0.00465256	0.9934	0.99995	no

ENSG00000089692	ENSG00000089692	LAG3	12:6881677-6887621	q1	q2	OK	1.67286	2.20238	0.396747	0.349193	0.64855	0.99995	no

ENSG00000089693	ENSG00000089693	MLF2	12:6857169-6880116	q1	q2	OK	102.591	99.4932	-0.0442333	-0.0536481	0.93285	0.99995	no

ENSG00000089723	ENSG00000089723	OTUB2	14:94492674-94515276	q1	q2	OK	2.52958	2.50771	-0.0125284	-0.0197486	0.9771	0.99995	no

ENSG00000089737	ENSG00000089737	DDX24	14:94517265-94547591	q1	q2	OK	62.99	65.982	0.0669502	0.139961	0.81795	0.99995	no

ENSG00000089775	ENSG00000089775	ZBTB25	14:64854748-65009955	q1	q2	OK	3.67512	3.13016	-0.231555	-0.104541	0.76265	0.99995	no

ENSG00000089818	ENSG00000089818	NECAP1	12:8234806-8250367	q1	q2	OK	14.9689	16.2947	0.122437	0.207085	0.72035	0.99995	no

ENSG00000089820	ENSG00000089820	ARHGAP4	X:153126968-153200676	q1	q2	NOTEST	0.0824284	0.124445	0.5943	0	1	1	no

ENSG00000089847	ENSG00000089847	ANKRD24	19:4183350-4224811	q1	q2	NOTEST	0.293002	0.255951	-0.195045	0	1	1	no

ENSG00000089876	ENSG00000089876	DHX32	10:127512114-127585005	q1	q2	OK	15.1706	14.3363	-0.0815978	-0.0921857	0.88895	0.99995	no

ENSG00000089902	ENSG00000089902	RCOR1	14:103058997-103196913	q1	q2	OK	16.0662	16.8928	0.0723804	0.117406	0.80135	0.99995	no

ENSG00000089916	ENSG00000089916	GPATCH2L	14:76618258-76729817	q1	q2	OK	17.7777	17.5662	-0.0172715	-0.0162732	0.97265	0.99995	no

ENSG00000090006	ENSG00000090006	LTBP4	19:41098788-41135725	q1	q2	OK	60.791	52.566	-0.209727	-0.30553	0.5209	0.99995	no

ENSG00000090013	ENSG00000090013	BLVRB	19:40953695-40971747	q1	q2	OK	5.45285	3.66828	-0.571907	-0.648829	0.3484	0.99995	no

ENSG00000090020	ENSG00000090020	SLC9A1	1:27425305-27493472	q1	q2	OK	7.16904	7.13853	-0.00615303	-0.0102724	0.9864	0.99995	no

ENSG00000090054	ENSG00000090054	SPTLC1	9:94794280-94877666	q1	q2	OK	27.3328	28.7403	0.0724425	0.145488	0.81745	0.99995	no

ENSG00000090060	ENSG00000090060	PAPOLA	14:96967146-97033448	q1	q2	OK	102.108	111.245	0.123647	0.202752	0.67505	0.99995	no

ENSG00000090061	ENSG00000090061	CCNK	14:99947505-100070363	q1	q2	OK	35.3147	37.1058	0.0713761	0.0823221	0.88525	0.99995	no

ENSG00000090097	ENSG00000090097	PCBP4	3:51989329-52023213	q1	q2	OK	74.142	70.5475	-0.071695	-0.148115	0.82475	0.99995	no

ENSG00000090104	ENSG00000090104	RGS1	1:192544856-192549161	q1	q2	NOTEST	0.102811	0.154104	0.583904	0	1	1	no

ENSG00000090238	ENSG00000090238	YPEL3	16:30103634-30115437	q1	q2	OK	9.20142	8.59954	-0.0975974	-0.128127	0.85115	0.99995	no

ENSG00000090263	ENSG00000090263	MRPS33	7:140705853-140715028	q1	q2	OK	41.1906	43.9522	0.0936192	0.172158	0.807	0.99995	no

ENSG00000090266	ENSG00000090266	NDUFB2	7:140218219-140624564	q1	q2	OK	124.691	118.103	-0.0783118	-0.0859423	0.90265	0.99995	no

ENSG00000090273	ENSG00000090273	NUDC	1:27216978-27273353	q1	q2	OK	64.5499	61.627	-0.0668521	-0.147891	0.8235	0.99995	no

ENSG00000090316	ENSG00000090316	MAEA	4:1283638-1333935	q1	q2	OK	44.8908	39.7097	-0.176927	-0.400149	0.54945	0.99995	no

ENSG00000090339	ENSG00000090339	ICAM1	19:10362576-10399695	q1	q2	OK	0.441293	0.597933	0.438247	0.181968	0.75255	0.99995	no

ENSG00000090372	ENSG00000090372	STRN4	19:47222763-47291851	q1	q2	OK	51.5133	48.5662	-0.0849908	-0.116606	0.8186	0.99995	no

ENSG00000090376	ENSG00000090376	IRAK3	12:66582658-66651214	q1	q2	NOTEST	0.143146	0.114095	-0.327247	0	1	1	no

ENSG00000090382	ENSG00000090382	LYZ	12:69742120-69748014	q1	q2	NOTEST	0.07651	0.0992725	0.375747	0	1	1	no

ENSG00000090402	ENSG00000090402	SI	3:164696685-164796283	q1	q2	NOTEST	0	0	0	0	1	1	no

ENSG00000090432	ENSG00000090432	MUL1	1:20825942-20834654	q1	q2	OK	14.7804	13.9124	-0.0873101	-0.183803	0.79305	0.99995	no

ENSG00000090447	ENSG00000090447	TFAP4	16:4307186-4323076	q1	q2	OK	6.70295	7.40547	0.143795	0.198245	0.7373	0.99995	no

ENSG00000090470	ENSG00000090470	PDCD7	15:65409716-65426174	q1	q2	OK	16.9955	17.9425	0.0782261	0.128457	0.79985	0.99995	no

ENSG00000090487	ENSG00000090487	SPG21	15:65255361-65282648	q1	q2	OK	51.0795	54.3062	0.0883747	0.199726	0.75675	0.99995	no

ENSG00000090512	ENSG00000090512	FETUB	3:186353757-186396029	q1	q2	NOTEST	0	0	0	0	1	1	no

ENSG00000090520	ENSG00000090520	DNAJB11	3:186256229-186315061	q1	q2	OK	147.763	140.085	-0.0769835	-0.0901679	0.88615	0.99995	no

ENSG00000090530	ENSG00000090530	LEPREL1	3:189674516-189862635	q1	q2	OK	4.89847	6.46216	0.399684	0.43231	0.3592	0.99995	no

ENSG00000090534	ENSG00000090534	THPO	3:183851806-184402546	q1	q2	NOTEST	0.30472	0.107411	-1.50435	0	1	1	no

ENSG00000090539	ENSG00000090539	CHRD	3:183851806-184402546	q1	q2	OK	2.94269	3.24135	0.139462	0.0121919	0.81505	0.99995	no

ENSG00000090554	ENSG00000090554	FLT3LG	19:49949554-49995565	q1	q2	NOTEST	0.343104	0.172186	-0.994676	0	1	1	no

ENSG00000090565	ENSG00000090565	RAB11FIP3	16:475618-573011	q1	q2	OK	39.6299	36.7322	-0.109545	-0.172329	0.72845	0.99995	no

ENSG00000090581	ENSG00000090581	GNPTG	16:1401923-1464752	q1	q2	OK	23.5858	20.3185	-0.215123	-0.300268	0.6588	0.99995	no

ENSG00000090612	ENSG00000090612	ZNF268	12:133707160-133783698	q1	q2	OK	12.1844	12.8134	0.0726226	0.110546	0.847	0.99995	no

ENSG00000090615	ENSG00000090615	GOLGA3	12:133345494-133532892	q1	q2	OK	15.5164	16.0911	0.0524639	0.0351085	0.8947	0.99995	no

ENSG00000090621	ENSG00000090621	PABPC4	1:40026487-40042462	q1	q2	OK	81.1986	83.5834	0.0417607	0.0684579	0.8892	0.99995	no

ENSG00000090659	ENSG00000090659	CD209	19:7804878-7812464	q1	q2	NOTEST	0.00637812	0	#NAME?	0	1	1	no

ENSG00000090661	ENSG00000090661	CERS4	19:8271619-8327305	q1	q2	OK	9.72835	8.31398	-0.226655	-0.278785	0.6708	0.99995	no

ENSG00000090674	ENSG00000090674	MCOLN1	19:7587511-7626650	q1	q2	OK	6.97525	7.3334	0.0722376	0.0644261	0.90935	0.99995	no

ENSG00000090686	ENSG00000090686	USP48	1:22004790-22110099	q1	q2	OK	31.3238	32.4359	0.0503284	0.0962851	0.8625	0.99995	no

ENSG00000090776	ENSG00000090776	EFNB1	X:68048839-68061990	q1	q2	OK	32.5804	30.4676	-0.0967326	-0.235087	0.7401	0.99995	no

ENSG00000090857	ENSG00000090857	PDPR	16:70147528-70221264	q1	q2	OK	13.4938	12.8767	-0.0675325	-0.143962	0.82885	0.99995	no

ENSG00000090861	ENSG00000090861	AARS	16:70286197-70323446	q1	q2	OK	46.3075	43.3484	-0.095266	-0.193732	0.73665	0.99995	no

ENSG00000090863	ENSG00000090863	GLG1	16:74485855-74641012	q1	q2	OK	50.775	48.8308	-0.0563259	-0.118119	0.84215	0.99995	no

ENSG00000090889	ENSG00000090889	KIF4A	X:69506444-69640682	q1	q2	OK	31.6894	32.5345	0.0379686	0.074543	0.90655	0.99995	no

ENSG00000090905	ENSG00000090905	TNRC6A	16:24741015-24838953	q1	q2	OK	37.6996	38.802	0.0415821	0.0497944	0.89805	0.99995	no

ENSG00000090920	ENSG00000090920	FCGBP	19:40353962-40440533	q1	q2	NOTEST	0.0512449	0.0655875	0.356012	0	1	1	no

ENSG00000090924	ENSG00000090924	PLEKHG2	19:39903224-39919055	q1	q2	OK	48.1478	47.0024	-0.0347333	-0.0512783	0.91605	0.99995	no

ENSG00000090932	ENSG00000090932	DLL3	19:39989534-39999121	q1	q2	OK	20.2451	18.0588	-0.164866	-0.348049	0.61505	0.99995	no

ENSG00000090971	ENSG00000090971	NAT14	19:55996370-55998935	q1	q2	OK	38.6961	33.1595	-0.222766	-0.418701	0.5374	0.99995	no

ENSG00000090975	ENSG00000090975	PITPNM2	12:123468026-123634562	q1	q2	OK	1.63948	1.25047	-0.390773	-0.524663	0.3654	0.99995	no

ENSG00000090989	ENSG00000090989	EXOC1	4:56719781-56771200	q1	q2	OK	20.5329	23.4015	0.18867	0.36867	0.5297	0.99995	no

ENSG00000091009	ENSG00000091009	RBM27	5:145583112-145756526	q1	q2	OK	11.4483	12.688	0.148332	0.274855	0.62905	0.99995	no

ENSG00000091010	ENSG00000091010	POU4F3	5:145583112-145756526	q1	q2	NOTEST	0.0241664	0.0250724	0.0530935	0	1	1	no

ENSG00000091039	ENSG00000091039	OSBPL8	12:76745576-76953589	q1	q2	OK	27.9151	31.473	0.17307	0.287513	0.54315	0.99995	no

ENSG00000091073	ENSG00000091073	DTX2	7:76090992-76135312	q1	q2	OK	11.2439	7.98896	-0.493063	-0.653069	0.30925	0.99995	no

ENSG00000091106	ENSG00000091106	NLRC4	2:32449521-32490923	q1	q2	NOTEST	0.220833	0.373697	0.758914	0	1	1	no

ENSG00000091127	ENSG00000091127	PUS7	7:105080107-105162714	q1	q2	OK	9.71262	11.672	0.265118	0.314469	0.45835	0.99995	no

ENSG00000091128	ENSG00000091128	LAMB4	7:107663992-107770801	q1	q2	NOTEST	0.342441	0.256823	-0.415082	0	1	1	no

ENSG00000091129	ENSG00000091129	NRCAM	7:107788067-108097161	q1	q2	OK	35.4321	29.891	-0.245342	-0.423136	0.45175	0.99995	no

ENSG00000091136	ENSG00000091136	LAMB1	7:107531414-107643700	q1	q2	OK	104.014	94.5204	-0.138077	-0.125485	0.7526	0.99995	no

ENSG00000091137	ENSG00000091137	SLC26A4	7:107294420-107358254	q1	q2	NOTEST	0.264635	0.183654	-0.52701	0	1	1	no

ENSG00000091138	ENSG00000091138	SLC26A3	7:107405911-107443670	q1	q2	NOTEST	0.0630953	0.025899	-1.28464	0	1	1	no

ENSG00000091140	ENSG00000091140	DLD	7:107531414-107643700	q1	q2	OK	52.9025	55.85	0.0782218	0.0405522	0.9532	0.99995	no

ENSG00000091157	ENSG00000091157	WDR7	18:54264438-54706076	q1	q2	OK	4.90331	5.47236	0.158407	0.155268	0.75455	0.99995	no

ENSG00000091164	ENSG00000091164	TXNL1	18:54264438-54706076	q1	q2	OK	61.4146	65.2652	0.0877323	0.162091	0.8186	0.99995	no

ENSG00000091181	ENSG00000091181	IL5RA	3:3111232-3168297	q1	q2	NOTEST	0.0115632	0	#NAME?	0	1	1	no

ENSG00000091262	ENSG00000091262	ABCC6	16:16242784-16318118	q1	q2	OK	0.718943	0.755601	0.0717473	0.0497434	0.9384	0.99995	no

ENSG00000091317	ENSG00000091317	CMTM6	3:32433162-32544900	q1	q2	OK	30.4212	33.7765	0.150941	0.298628	0.6328	0.99995	no

ENSG00000091409	ENSG00000091409	ITGA6	2:173292081-173489823	q1	q2	OK	11.4455	10.7656	-0.0883534	-0.0775954	0.8393	0.99995	no

ENSG00000091428	ENSG00000091428	RAPGEF4	2:173541868-173917621	q1	q2	OK	0.498674	0.556184	0.157466	0.0809256	0.8264	0.99995	no

ENSG00000091436	ENSG00000091436	MLTK	2:173940162-174146764	q1	q2	OK	8.95216	10.0848	0.171878	0.349903	0.6085	0.99995	no

ENSG00000091482	ENSG00000091482	SMPX	X:21724089-21776281	q1	q2	NOTEST	0.0339649	0	#NAME?	0	1	1	no

ENSG00000091483	ENSG00000091483	FH	1:241660902-241683061	q1	q2	OK	55.2393	55.8373	0.0155322	0.032342	0.95385	0.99995	no

ENSG00000091490	ENSG00000091490	SEL1L3	4:25749054-25931435	q1	q2	OK	4.77513	4.58649	-0.0581476	-0.0646605	0.89075	0.99995	no

ENSG00000091513	ENSG00000091513	TF	3:133380841-133497850	q1	q2	OK	1.53986	1.12503	-0.452839	-0.366225	0.41605	0.99995	no

ENSG00000091527	ENSG00000091527	CDV3	3:133292573-133309105	q1	q2	OK	69.5706	75.2926	0.11403	0.230384	0.71285	0.99995	no

ENSG00000091536	ENSG00000091536	MYO15A	17:18012019-18113268	q1	q2	NOTEST	0.383771	0.422633	0.139159	0	1	1	no

ENSG00000091542	ENSG00000091542	ALKBH5	17:18012019-18113268	q1	q2	OK	26.6242	25.9143	-0.038988	-0.0898394	0.89605	0.99995	no

ENSG00000091583	ENSG00000091583	APOH	17:64208150-64252643	q1	q2	NOTEST	0	0	0	0	1	1	no

ENSG00000091592	ENSG00000091592	NLRP1	17:5402746-5522744	q1	q2	OK	50.1058	47.8959	-0.0650767	-0.15333	0.8225	0.99995	no

ENSG00000091622	ENSG00000091622	PITPNM3	17:6347734-6459814	q1	q2	OK	1.09945	0.98851	-0.153456	-0.0821343	0.88465	0.99995	no

ENSG00000091640	ENSG00000091640	SPAG7	17:4862520-4871167	q1	q2	OK	55.56	52.1667	-0.0909192	-0.196308	0.7669	0.99995	no

ENSG00000091651	ENSG00000091651	ORC6	16:46723554-46732306	q1	q2	OK	55.0276	55.857	0.0215839	0.0496702	0.94465	0.99995	no

ENSG00000091656	ENSG00000091656	ZFHX4	8:77316232-77779521	q1	q2	OK	7.14303	6.49472	-0.137268	-0.150627	0.6468	0.99995	no

ENSG00000091664	ENSG00000091664	SLC17A6	11:22305275-22401049	q1	q2	OK	1.06298	1.13349	0.0926544	0.104248	0.86485	0.99995	no

ENSG00000091704	ENSG00000091704	CPA1	7:130020179-130027955	q1	q2	NOTEST	0	0	0	0	1	1	no

ENSG00000091732	ENSG00000091732	ZC3HC1	7:129593073-129691291	q1	q2	OK	18.2454	19.3053	0.0814633	0.157843	0.8084	0.99995	no

ENSG00000091831	ENSG00000091831	ESR1	6:151977825-152958936	q1	q2	NOTEST	0.215118	0.311087	0.532188	0	1	1	no

ENSG00000091844	ENSG00000091844	RGS17	6:153325593-153452384	q1	q2	OK	3.02348	3.14007	0.0545875	0.0802783	0.9039	0.99995	no

ENSG00000091879	ENSG00000091879	ANGPT2	8:6261071-6565730	q1	q2	NOTEST	0.219179	0.20092	-0.125486	0	1	1	no

ENSG00000091947	ENSG00000091947	TMEM101	17:42088555-42101314	q1	q2	OK	14.2682	13.1094	-0.122199	-0.188308	0.75745	0.99995	no

ENSG00000091972	ENSG00000091972	CD200	3:112021324-112081659	q1	q2	OK	34.4491	39.9653	0.214284	0.468759	0.4663	0.99995	no

ENSG00000091986	ENSG00000091986	CCDC80	3:112323406-112368377	q1	q2	OK	46.4583	50.3331	0.115572	0.193755	0.7465	0.99995	no

ENSG00000092009	ENSG00000092009	CMA1	14:24970799-24977894	q1	q2	NOTEST	0.0579946	0	#NAME?	0	1	1	no

ENSG00000092010	ENSG00000092010	PSME1	14:24605366-24610797	q1	q2	OK	51.4519	48.0345	-0.0991538	-0.200468	0.7711	0.99995	no

ENSG00000092020	ENSG00000092020	PPP2R3C	14:35514112-35786699	q1	q2	OK	31.6392	32.2355	0.026938	0.0239461	0.9574	0.99995	no

ENSG00000092036	ENSG00000092036	HAUS4	14:23398817-23451851	q1	q2	OK	22.6754	19.4775	-0.219321	-0.176196	0.75865	0.99995	no

ENSG00000092051	ENSG00000092051	JPH4	14:23980968-24048009	q1	q2	OK	3.71854	2.79583	-0.411456	-0.352107	0.5922	0.99995	no

ENSG00000092054	ENSG00000092054	MYH7	14:23881946-23904927	q1	q2	NOTEST	0.250952	0.17113	-0.55232	0	1	1	no

ENSG00000092067	ENSG00000092067	CEBPE	14:23586512-23588825	q1	q2	NOTEST	0	0.0170339	inf	0	1	1	no

ENSG00000092068	ENSG00000092068	SLC7A8	14:23594503-23652883	q1	q2	OK	4.00477	3.02139	-0.406508	-0.403883	0.4544	0.99995	no

ENSG00000092094	ENSG00000092094	OSGEP	14:20914569-20923264	q1	q2	OK	30.8231	32.1312	0.0599596	0.100463	0.87125	0.99995	no

ENSG00000092096	ENSG00000092096	SLC22A17	14:23815514-23822121	q1	q2	OK	32.7699	27.6924	-0.24288	-0.505959	0.43855	0.99995	no

ENSG00000092098	ENSG00000092098	RNF31	14:24612573-24636611	q1	q2	OK	20.2467	15.7316	-0.364021	-0.290586	0.4446	0.99995	no

ENSG00000092108	ENSG00000092108	SCFD1	14:31091317-31291908	q1	q2	OK	34.2802	34.9377	0.0274088	0.0511743	0.93095	0.99995	no

ENSG00000092140	ENSG00000092140	G2E3	14:30907197-31089269	q1	q2	OK	19.4138	22.485	0.211877	0.354827	0.49935	0.99995	no

ENSG00000092148	ENSG00000092148	HECTD1	14:31569317-31677010	q1	q2	OK	55.4755	58.1164	0.0670952	0.0917985	0.8438	0.99995	no

ENSG00000092199	ENSG00000092199	HNRNPC	14:21677294-21737653	q1	q2	OK	540.944	547.928	0.0185064	0.0382615	0.95305	0.99995	no

ENSG00000092200	ENSG00000092200	RPGRIP1	14:21756097-21819460	q1	q2	NOTEST	0.0131361	0.053755	2.03286	0	1	1	no

ENSG00000092201	ENSG00000092201	SUPT16H	14:21819630-21852425	q1	q2	OK	87.542	93.1427	0.0894682	0.188074	0.7774	0.99995	no

ENSG00000092203	ENSG00000092203	TOX4	14:21927178-21979517	q1	q2	OK	26.0067	23.9656	-0.117919	-0.14121	0.82	0.99995	no

ENSG00000092208	ENSG00000092208	GEMIN2	14:39583426-39606177	q1	q2	OK	21.5439	22.7057	0.0757734	0.13371	0.84345	0.99995	no

ENSG00000092295	ENSG00000092295	TGM1	14:24718319-24733638	q1	q2	OK	1.40201	1.63873	0.225082	0.179865	0.74235	0.99995	no

ENSG00000092330	ENSG00000092330	TINF2	14:24708848-24711880	q1	q2	OK	28.2116	30.2975	0.102908	0.174325	0.78915	0.99995	no

ENSG00000092345	ENSG00000092345	DAZL	3:16628298-16711813	q1	q2	NOTEST	0.00832129	0	#NAME?	0	1	1	no

ENSG00000092377	ENSG00000092377	TBL1Y	Y:6778726-6959724	q1	q2	OK	1.06866	1.01279	-0.0774689	-0.0885177	0.8965	0.99995	no

ENSG00000092421	ENSG00000092421	SEMA6A	5:115779311-115927094	q1	q2	OK	59.1228	53.1857	-0.152677	-0.295089	0.6018	0.99995	no

ENSG00000092439	ENSG00000092439	TRPM7	15:50844669-50979012	q1	q2	OK	11.8792	14.696	0.306986	0.491765	0.3749	0.99995	no

ENSG00000092445	ENSG00000092445	TYRO3	15:41849872-41871536	q1	q2	OK	24.3082	22.4493	-0.114772	-0.23921	0.7007	0.99995	no

ENSG00000092470	ENSG00000092470	WDR76	15:44119160-44160617	q1	q2	OK	17.0977	18.7804	0.135422	0.255357	0.70635	0.99995	no

ENSG00000092529	ENSG00000092529	CAPN3	15:42500610-42704516	q1	q2	OK	0.490471	0.656205	0.419977	0.0845996	0.5921	0.99995	no

ENSG00000092531	ENSG00000092531	SNAP23	15:42783430-42841000	q1	q2	OK	32.2695	36.1188	0.162576	0.25669	0.64185	0.99995	no

ENSG00000092607	ENSG00000092607	TBX15	1:119425668-119532179	q1	q2	OK	9.38745	8.51749	-0.140304	-0.249305	0.7206	0.99995	no

ENSG00000092621	ENSG00000092621	PHGDH	1:120202420-120286838	q1	q2	OK	135.663	128.964	-0.0730524	-0.174794	0.79545	0.99995	no

ENSG00000092758	ENSG00000092758	COL9A3	20:61447595-61493115	q1	q2	OK	2.35097	2.09776	-0.164411	-0.0972018	0.8477	0.99995	no

ENSG00000092820	ENSG00000092820	EZR	6:159186772-159241625	q1	q2	OK	44.0993	47.3124	0.10146	0.25053	0.7228	0.99995	no

ENSG00000092841	ENSG00000092841	MYL6	12:56544579-56584068	q1	q2	OK	1196.65	1185.13	-0.0139573	-0.0246673	0.9709	0.99995	no

ENSG00000092847	ENSG00000092847	AGO1	1:36335408-36395211	q1	q2	OK	23.1199	24.3238	0.073234	0.179293	0.7965	0.99995	no

ENSG00000092850	ENSG00000092850	TEKT2	1:36549675-36553876	q1	q2	OK	6.6883	3.86106	-0.792642	-1.08808	0.12375	0.99995	no

ENSG00000092853	ENSG00000092853	CLSPN	1:36185818-36235568	q1	q2	OK	25.852	28.3047	0.130765	0.211113	0.718	0.99995	no

ENSG00000092871	ENSG00000092871	RFFL	17:33332106-33448541	q1	q2	OK	6.83909	5.75884	-0.248025	-0.240453	0.5872	0.99995	no

ENSG00000092929	ENSG00000092929	UNC13D	17:73823305-73840798	q1	q2	OK	0.801174	0.368581	-1.12014	-0.465032	0.1436	0.99995	no

ENSG00000092931	ENSG00000092931	MFSD11	17:74722911-74777531	q1	q2	OK	15.4771	12.8234	-0.271354	-0.110148	0.7077	0.99995	no

ENSG00000092964	ENSG00000092964	DPYSL2	8:26371790-26515694	q1	q2	OK	121.289	131.652	0.118277	0.259465	0.7084	0.99995	no

ENSG00000092969	ENSG00000092969	TGFB2	1:218519576-218617961	q1	q2	OK	3.01688	2.91671	-0.048715	-0.0480696	0.93185	0.99995	no

ENSG00000092978	ENSG00000092978	GPATCH2	1:217600333-217804424	q1	q2	OK	8.62483	10.3298	0.260239	0.310757	0.46555	0.99995	no

ENSG00000093000	ENSG00000093000	NUP50	22:45559721-45583896	q1	q2	OK	43.0375	46.7412	0.119099	0.206812	0.6577	0.99995	no

ENSG00000093009	ENSG00000093009	CDC45	22:19466981-19508135	q1	q2	OK	24.9462	23.2697	-0.10037	-0.184436	0.75995	0.99995	no

ENSG00000093010	ENSG00000093010	COMT	22:19863039-20004331	q1	q2	OK	30.2936	24.886	-0.283681	-0.269313	0.66935	0.99995	no

ENSG00000093072	ENSG00000093072	CECR1	22:17660193-17702879	q1	q2	OK	5.48834	4.95097	-0.148659	-0.199109	0.71185	0.99995	no

ENSG00000093100	ENSG00000093100	XXbac-B461K10.4	22:18270414-18507325	q1	q2	OK	3.58554	2.99177	-0.261195	-0.210544	0.7522	0.99995	no

ENSG00000093134	ENSG00000093134	VNN3	6:133043925-133055904	q1	q2	NOTEST	0.0305208	0	#NAME?	0	1	1	no

ENSG00000093144	ENSG00000093144	ECHDC1	6:127609854-127664754	q1	q2	OK	53.5555	56.4494	0.0759245	0.151317	0.8183	0.99995	no

ENSG00000093167	ENSG00000093167	LRRFIP2	3:37034822-37225180	q1	q2	OK	25.7249	30.6391	0.25221	0.272266	0.56745	0.99995	no

ENSG00000093183	ENSG00000093183	SEC22C	3:42589460-42709072	q1	q2	OK	22.0679	23.5234	0.0921432	0.0599111	0.91495	0.99995	no

ENSG00000093217	ENSG00000093217	XYLB	3:38388250-38462839	q1	q2	OK	1.24882	1.74952	0.486391	0.334395	0.4453	0.99995	no

ENSG00000094631	ENSG00000094631	HDAC6	X:48659783-48683392	q1	q2	OK	0.669548	4.32764	2.69232	1.6125	0.0001	0.134546	no

ENSG00000094661	ENSG00000094661	OR1I1	19:15197790-15199032	q1	q2	NOTEST	0	0	0	0	1	1	no

ENSG00000094755	ENSG00000094755	GABRP	5:170174050-170241051	q1	q2	NOTEST	0.00762918	0	#NAME?	0	1	1	no

ENSG00000094796	ENSG00000094796	KRT31	17:39549975-39553844	q1	q2	NOTEST	0	0	0	0	1	1	no

ENSG00000094804	ENSG00000094804	CDC6	17:38443884-38459171	q1	q2	OK	29.1087	32.9511	0.178874	0.353367	0.536	0.99995	no

ENSG00000094841	ENSG00000094841	UPRT	X:74493919-74524435	q1	q2	OK	8.75126	8.57943	-0.0286082	-0.0470986	0.9463	0.99995	no

ENSG00000094880	ENSG00000094880	CDC23	5:137475454-137549032	q1	q2	OK	28.1969	30.333	0.10535	0.0940471	0.8463	0.99995	no

ENSG00000094914	ENSG00000094914	AAAS	12:53701239-53718648	q1	q2	OK	42.0638	40.5965	-0.0512248	-0.101101	0.8733	0.99995	no

ENSG00000094916	ENSG00000094916	CBX5	12:54624723-54745633	q1	q2	OK	80.3918	84.7766	0.0766174	0.0367696	0.93445	0.99995	no

ENSG00000094963	ENSG00000094963	FMO2	1:171154346-171196927	q1	q2	NOTEST	0.0703551	0	#NAME?	0	1	1	no

ENSG00000094975	ENSG00000094975	SUCO	1:172501488-172580971	q1	q2	OK	27.5336	30.4538	0.145431	0.260926	0.6026	0.99995	no

ENSG00000095002	ENSG00000095002	MSH2	2:47630107-47798078	q1	q2	OK	53.0454	59.0748	0.155316	0.36427	0.59485	0.99995	no

ENSG00000095015	ENSG00000095015	MAP3K1	5:56111400-56191979	q1	q2	OK	6.92065	8.45357	0.288653	0.603729	0.3939	0.99995	no

ENSG00000095059	ENSG00000095059	DHPS	19:12754088-12792716	q1	q2	OK	62.3818	58.5073	-0.0925085	-0.0997381	0.86695	0.99995	no

ENSG00000095066	ENSG00000095066	HOOK2	19:12873816-12985765	q1	q2	OK	22.6754	20.9683	-0.112923	-0.0584549	0.85335	0.99995	no

ENSG00000095110	ENSG00000095110	NXPE1	11:114392436-114430617	q1	q2	NOTEST	0	0	0	0	1	1	no

ENSG00000095139	ENSG00000095139	ARCN1	11:118307204-118473748	q1	q2	OK	1.54791	1.82727	0.239368	0.202575	0.76275	0.99995	no

ENSG00000095203	ENSG00000095203	EPB41L4B	9:111934254-112083244	q1	q2	OK	4.55125	4.9962	0.134569	0.249685	0.7191	0.99995	no

ENSG00000095209	ENSG00000095209	TMEM38B	9:108456824-108538893	q1	q2	OK	11.2991	11.2598	-0.00503052	-0.0078466	0.98995	0.99995	no

ENSG00000095261	ENSG00000095261	PSMD5	9:123577773-123616651	q1	q2	OK	20.0695	20.8285	0.0535589	0.0636761	0.88285	0.99995	no

ENSG00000095303	ENSG00000095303	PTGS1	9:125132823-125157982	q1	q2	NOTEST	0.154972	0.142353	-0.122528	0	1	1	no

ENSG00000095319	ENSG00000095319	NUP188	9:131683173-131790582	q1	q2	OK	31.4817	29.8083	-0.0787998	-0.0856088	0.81085	0.99995	no

ENSG00000095321	ENSG00000095321	CRAT	9:131857088-131911225	q1	q2	OK	14.6513	12.9537	-0.177662	-0.152028	0.7985	0.99995	no

ENSG00000095370	ENSG00000095370	SH2D3C	9:130500595-130541020	q1	q2	OK	6.63359	5.8646	-0.177757	-0.248267	0.67925	0.99995	no

ENSG00000095380	ENSG00000095380	NANS	9:100819020-100881494	q1	q2	OK	24.7704	23.2538	-0.0911505	-0.173367	0.7985	0.99995	no

ENSG00000095383	ENSG00000095383	TBC1D2	9:100961310-101017915	q1	q2	OK	0.690038	0.871794	0.337311	0.316195	0.61615	0.99995	no

ENSG00000095397	ENSG00000095397	DFNB31	9:117164359-117267730	q1	q2	OK	4.47788	3.5193	-0.347529	-0.529413	0.44715	0.99995	no

ENSG00000095464	ENSG00000095464	PDE6C	10:95372344-95425767	q1	q2	NOTEST	0.107776	0.273368	1.3428	0	1	1	no

ENSG00000095485	ENSG00000095485	CWF19L1	10:101989423-102027437	q1	q2	OK	14.4289	14.2075	-0.0223114	-0.0356129	0.95045	0.99995	no

ENSG00000095539	ENSG00000095539	SEMA4G	10:102729214-102754158	q1	q2	OK	4.82673	4.58703	-0.0734859	-0.0357686	0.9075	0.99995	no

ENSG00000095564	ENSG00000095564	BTAF1	10:93683525-93790082	q1	q2	OK	23.3463	23.8741	0.0322474	0.063652	0.92255	0.99995	no

ENSG00000095574	ENSG00000095574	IKZF5	10:124690418-124768333	q1	q2	OK	7.96982	8.44016	0.0827222	0.0908958	0.8375	0.99995	no

ENSG00000095585	ENSG00000095585	BLNK	10:97889471-98031344	q1	q2	NOTEST	0	0	0	0	1	1	no

ENSG00000095587	ENSG00000095587	TLL2	10:98124362-98273675	q1	q2	OK	1.21046	0.960125	-0.334263	-0.331045	0.58565	0.99995	no

ENSG00000095596	ENSG00000095596	CYP26A1	10:94833231-94837647	q1	q2	OK	3.79779	2.71617	-0.483588	-0.693842	0.3302	0.99995	no

ENSG00000095627	ENSG00000095627	TDRD1	10:115939028-115992063	q1	q2	NOTEST	0.00647598	0	#NAME?	0	1	1	no

ENSG00000095637	ENSG00000095637	SORBS1	10:97071527-97321171	q1	q2	OK	5.62455	5.26878	-0.0942683	-0.167504	0.77595	0.99995	no

ENSG00000095713	ENSG00000095713	CRTAC1	10:99609995-99790585	q1	q2	OK	1.56179	1.17179	-0.414488	-0.226725	0.68075	0.99995	no

ENSG00000095739	ENSG00000095739	BAMBI	10:28966270-28971868	q1	q2	OK	4.45897	4.13233	-0.109755	-0.162523	0.8166	0.99995	no

ENSG00000095752	ENSG00000095752	IL11	19:55875756-55881831	q1	q2	OK	1.10972	0.821278	-0.434258	-0.398304	0.524	0.99995	no

ENSG00000095777	ENSG00000095777	MYO3A	10:26223195-26501456	q1	q2	OK	0.969968	0.877899	-0.143882	-0.118687	0.8058	0.99995	no

ENSG00000095787	ENSG00000095787	WAC	10:28811580-28912041	q1	q2	OK	65.8019	69.0252	0.0689922	0.128725	0.8199	0.99995	no

ENSG00000095794	ENSG00000095794	CREM	10:35386933-35501886	q1	q2	OK	24.3906	23.7867	-0.036168	-0.06197	0.9214	0.99995	no

ENSG00000095906	ENSG00000095906	NUBP2	16:1823207-1844972	q1	q2	OK	39.5309	33.0851	-0.256798	-0.357883	0.601	0.99995	no

ENSG00000095917	ENSG00000095917	TPSD1	16:1306059-1308532	q1	q2	NOTEST	0	0	0	0	1	1	no

ENSG00000095932	ENSG00000095932	C19orf77	19:3474404-3480540	q1	q2	NOTEST	0	0	0	0	1	1	no

ENSG00000095951	ENSG00000095951	HIVEP1	6:12007902-12165232	q1	q2	OK	5.89925	5.19552	-0.183262	-0.225358	0.6818	0.99995	no

ENSG00000095970	ENSG00000095970	TREM2	6:41126243-41130924	q1	q2	NOTEST	0.0359574	0	#NAME?	0	1	1	no

ENSG00000095981	ENSG00000095981	KCNK16	6:39282473-39290744	q1	q2	NOTEST	0	0	0	0	1	1	no

ENSG00000096006	ENSG00000096006	CRISP3	6:49695096-49712150	q1	q2	NOTEST	0	0	0	0	1	1	no

ENSG00000096060	ENSG00000096060	FKBP5	6:35541361-35696360	q1	q2	OK	24.2393	30.5712	0.334826	0.807538	0.2575	0.99995	no

ENSG00000096063	ENSG00000096063	SRPK1	6:35773069-35889119	q1	q2	OK	32.6006	34.9148	0.0989429	0.191887	0.72565	0.99995	no

ENSG00000096070	ENSG00000096070	BRPF3	6:36114474-36200567	q1	q2	OK	22.3591	22.5034	0.00927972	0.0165489	0.97585	0.99995	no

ENSG00000096080	ENSG00000096080	MRPS18A	6:43612782-43655528	q1	q2	OK	25.2192	24.5379	-0.0395089	-0.072364	0.9199	0.99995	no

ENSG00000096088	ENSG00000096088	PGC	6:41704448-41721847	q1	q2	NOTEST	0.0202654	0	#NAME?	0	1	1	no

ENSG00000096092	ENSG00000096092	TMEM14A	6:52535906-52551386	q1	q2	OK	18.7499	17.9469	-0.0631448	-0.110734	0.8721	0.99995	no

ENSG00000096093	ENSG00000096093	EFHC1	6:52285105-52441713	q1	q2	OK	15.6813	17.1136	0.126105	0.161948	0.7744	0.99995	no

ENSG00000096264	ENSG00000096264	NCR2	6:41303392-41318625	q1	q2	NOTEST	0	0	0	0	1	1	no

ENSG00000096384	ENSG00000096384	HSP90AB1	6:44214823-44221620	q1	q2	OK	606.34	648.478	0.0969318	0.184487	0.79165	0.99995	no

ENSG00000096395	ENSG00000096395	MLN	6:33762449-33771788	q1	q2	OK	2.56019	1.47465	-0.795881	-0.723429	0.27565	0.99995	no

ENSG00000096401	ENSG00000096401	CDC5L	6:44355261-44418163	q1	q2	OK	19.5987	22.0158	0.167781	0.414579	0.5415	0.99995	no

ENSG00000096433	ENSG00000096433	ITPR3	6:33588141-33679504	q1	q2	OK	1.18397	1.19206	0.00982569	0.00858539	0.99095	0.99995	no

ENSG00000096654	ENSG00000096654	ZNF184	6:27418521-27440897	q1	q2	OK	12.2466	12.2182	-0.0033504	-0.0071512	0.9918	0.99995	no

ENSG00000096696	ENSG00000096696	DSP	6:7541807-7586950	q1	q2	OK	0.743651	0.859399	0.208702	0.294029	0.6509	0.99995	no

ENSG00000096717	ENSG00000096717	SIRT1	10:69644426-69678147	q1	q2	OK	19.6719	20.0532	0.0276944	0.0643197	0.9288	0.99995	no

ENSG00000096746	ENSG00000096746	HNRNPH3	10:70042416-70167051	q1	q2	OK	336.503	380.689	0.177994	0.304109	0.64185	0.99995	no

ENSG00000096872	ENSG00000096872	IFT74	9:26904080-27062928	q1	q2	OK	8.14781	8.96073	0.137205	0.143089	0.823	0.99995	no

ENSG00000096968	ENSG00000096968	JAK2	9:4985032-5128183	q1	q2	OK	3.66886	3.6094	-0.0235705	-0.0363655	0.95125	0.99995	no

ENSG00000096996	ENSG00000096996	IL12RB1	19:18169804-18262502	q1	q2	NOTEST	0	0	0	0	1	1	no

ENSG00000097007	ENSG00000097007	ABL1	9:133589332-133763062	q1	q2	OK	42.2833	41.3197	-0.0332558	-0.0554987	0.9148	0.99995	no

ENSG00000097021	ENSG00000097021	ACOT7	1:6324328-6454451	q1	q2	OK	49.4052	49.1326	-0.00798099	-0.0183748	0.9782	0.99995	no

ENSG00000097033	ENSG00000097033	SH3GLB1	1:87170258-87213867	q1	q2	OK	29.6569	30.6856	0.0491895	0.0791412	0.89865	0.99995	no

ENSG00000097046	ENSG00000097046	CDC7	1:91966407-91991321	q1	q2	OK	33.6428	34.8046	0.0489785	0.101363	0.8708	0.99995	no

ENSG00000097096	ENSG00000097096	SYDE2	1:85622555-85666729	q1	q2	OK	1.7549	1.72315	-0.0263364	-0.0352213	0.9614	0.99995	no

ENSG00000099139	ENSG00000099139	PCSK5	9:78505559-78977255	q1	q2	OK	1.29213	1.42955	0.145807	0.201549	0.75285	0.99995	no

ENSG00000099194	ENSG00000099194	SCD	10:102106880-102124591	q1	q2	OK	279.368	325.55	0.220712	0.361268	0.59795	0.99995	no

ENSG00000099203	ENSG00000099203	TMED1	19:10828754-10946994	q1	q2	OK	19.757	20.7933	0.073761	0.0733384	0.91385	0.99995	no

ENSG00000099204	ENSG00000099204	ABLIM1	10:116190871-116444762	q1	q2	OK	46.6782	51.9515	0.154417	0.225131	0.6143	0.99995	no

ENSG00000099219	ENSG00000099219	ERMP1	9:5629024-5833117	q1	q2	OK	3.58726	3.61106	0.00954186	0.00966655	0.9886	0.99995	no

ENSG00000099246	ENSG00000099246	RAB18	10:27793196-27831143	q1	q2	OK	60.274	68.2553	0.179404	0.312952	0.649	0.99995	no

ENSG00000099250	ENSG00000099250	NRP1	10:33466419-33625190	q1	q2	OK	11.0215	10.9132	-0.014239	-0.0147517	0.97355	0.99995	no

ENSG00000099251	ENSG00000099251	HSD17B7P2	10:38645304-38667433	q1	q2	OK	1.84821	1.51246	-0.289225	-0.325647	0.61175	0.99995	no

ENSG00000099256	ENSG00000099256	PRTFDC1	10:25137535-25241533	q1	q2	OK	14.1264	13.9186	-0.0213802	-0.038112	0.95535	0.99995	no

ENSG00000099260	ENSG00000099260	PALMD	1:100111498-100160097	q1	q2	OK	3.25701	2.63839	-0.303889	-0.434993	0.5437	0.99995	no

ENSG00000099282	ENSG00000099282	TSPAN15	10:71211228-71267425	q1	q2	OK	4.70843	3.81906	-0.302029	-0.393352	0.52425	0.99995	no

ENSG00000099284	ENSG00000099284	H2AFY2	10:71812551-71892690	q1	q2	OK	128.207	124.598	-0.0411987	-0.10025	0.88235	0.99995	no

ENSG00000099290	ENSG00000099290	FAM21A	10:51827647-51893269	q1	q2	OK	1.68756	1.39488	-0.274796	-0.332183	0.5687	0.99995	no

ENSG00000099308	ENSG00000099308	MAST3	19:18169804-18262502	q1	q2	OK	2.74416	2.28269	-0.265631	-0.295126	0.50985	0.99995	no

ENSG00000099326	ENSG00000099326	MZF1	19:59070495-59111168	q1	q2	OK	21.1379	19.3037	-0.130954	-0.212287	0.7483	0.99995	no

ENSG00000099330	ENSG00000099330	OCEL1	19:17337012-17340028	q1	q2	OK	6.9636	5.62935	-0.306865	-0.353662	0.58425	0.99995	no

ENSG00000099331	ENSG00000099331	MYO9B	19:17186590-17325346	q1	q2	OK	52.8642	51.8959	-0.0266712	-0.0363454	0.94245	0.99995	no

ENSG00000099337	ENSG00000099337	KCNK6	19:38810483-38819660	q1	q2	NOTEST	0.0993607	0.112582	0.180223	0	1	1	no

ENSG00000099338	ENSG00000099338	CATSPERG	19:38826414-38861589	q1	q2	OK	1.87952	1.58295	-0.247752	-0.172873	0.7577	0.99995	no

ENSG00000099341	ENSG00000099341	PSMD8	19:38865175-38874464	q1	q2	OK	110.85	103.91	-0.0932765	-0.216032	0.7644	0.99995	no

ENSG00000099364	ENSG00000099364	FBXL19	16:30930639-30967832	q1	q2	OK	28.5259	26.4542	-0.108779	-0.229471	0.73585	0.99995	no

ENSG00000099365	ENSG00000099365	STX1B	16:31000576-31021949	q1	q2	OK	5.40548	6.02194	0.155806	0.168271	0.75265	0.99995	no

ENSG00000099377	ENSG00000099377	HSD3B7	16:30996518-31000473	q1	q2	OK	1.2239	1.16683	-0.0688977	-0.0709009	0.9129	0.99995	no

ENSG00000099381	ENSG00000099381	SETD1A	16:30968614-30996437	q1	q2	OK	9.87849	9.55646	-0.0478141	-0.0859723	0.88055	0.99995	no

ENSG00000099385	ENSG00000099385	BCL7C	16:30844946-30906541	q1	q2	OK	18.1335	17.0342	-0.0902209	-0.168189	0.807	0.99995	no

ENSG00000099399	ENSG00000099399	MAGEB2	X:30233676-30238206	q1	q2	NOTEST	0	0	0	0	1	1	no

ENSG00000099617	ENSG00000099617	EFNA2	19:1285889-1378430	q1	q2	OK	13.2317	11.8056	-0.164533	-0.17383	0.801	0.99995	no

ENSG00000099622	ENSG00000099622	CIRBP	19:1259383-1274879	q1	q2	OK	259.066	234.388	-0.144419	-0.292057	0.65985	0.99995	no

ENSG00000099624	ENSG00000099624	ATP5D	19:1241748-1244824	q1	q2	OK	48.4	42.2342	-0.196597	-0.371668	0.607	0.99995	no

ENSG00000099625	ENSG00000099625	C19orf26	19:1189405-1238026	q1	q2	OK	9.9691	7.78938	-0.355955	-0.362975	0.55345	0.99995	no

ENSG00000099715	ENSG00000099715	PCDH11Y	Y:4868266-5610265	q1	q2	NOTEST	0.149528	0.111102	-0.428529	0	1	1	no

ENSG00000099721	ENSG00000099721	AMELY	Y:6733958-6742068	q1	q2	NOTEST	0	0	0	0	1	1	no

ENSG00000099725	ENSG00000099725	PRKY	Y:7142012-7249589	q1	q2	OK	4.45541	5.04586	0.17954	0.282884	0.5903	0.99995	no

ENSG00000099769	ENSG00000099769	IGFALS	16:1823207-1844972	q1	q2	NOTEST	0.142688	0.093111	-0.615845	0	1	1	no

ENSG00000099783	ENSG00000099783	HNRNPM	19:8509650-8553998	q1	q2	OK	287.39	310.727	0.112639	0.226084	0.732	0.99995	no

ENSG00000099785	ENSG00000099785	Mar-02	19:8478153-8503901	q1	q2	OK	8.36764	7.49191	-0.159487	-0.210435	0.7577	0.99995	no

ENSG00000099795	ENSG00000099795	NDUFB7	19:14676889-14682874	q1	q2	OK	105.491	94.2737	-0.162195	-0.339982	0.61915	0.99995	no

ENSG00000099797	ENSG00000099797	TECR	19:14625581-14676792	q1	q2	OK	74.1806	71.9721	-0.0436038	-0.0646548	0.91665	0.99995	no

ENSG00000099800	ENSG00000099800	TIMM13	19:2389768-2456994	q1	q2	OK	40.751	36.8628	-0.144671	-0.0975124	0.89735	0.99995	no

ENSG00000099804	ENSG00000099804	CDC34	19:531713-542097	q1	q2	OK	78.1923	68.2427	-0.196352	-0.401482	0.51635	0.99995	no

ENSG00000099810	ENSG00000099810	MTAP	9:21802541-22121096	q1	q2	OK	26.3387	28.5388	0.115741	0.149563	0.7477	0.99995	no

ENSG00000099812	ENSG00000099812	MISP	19:751125-764319	q1	q2	NOTEST	0.0626466	0.0535162	-0.227259	0	1	1	no

ENSG00000099814	ENSG00000099814	CEP170B	14:105331616-105363107	q1	q2	OK	7.13993	6.39424	-0.159136	-0.262477	0.63605	0.99995	no

ENSG00000099817	ENSG00000099817	POLR2E	19:1065921-1095598	q1	q2	OK	74.6315	65.8078	-0.181527	-0.37667	0.55935	0.99995	no

ENSG00000099821	ENSG00000099821	POLRMT	19:617222-633597	q1	q2	OK	15.1065	15.518	0.0387752	0.0504111	0.9149	0.99995	no

ENSG00000099822	ENSG00000099822	HCN2	19:589892-617159	q1	q2	OK	0.883517	0.847034	-0.0608386	-0.0704639	0.9212	0.99995	no

ENSG00000099834	ENSG00000099834	CDHR5	11:616564-626078	q1	q2	NOTEST	0.142793	0.077079	-0.889519	0	1	1	no

ENSG00000099840	ENSG00000099840	IZUMO4	19:2071036-2099592	q1	q2	OK	3.60291	3.34175	-0.108558	-0.0575949	0.9019	0.99995	no

ENSG00000099849	ENSG00000099849	RASSF7	11:537526-564021	q1	q2	OK	4.36385	3.54429	-0.300107	-0.275806	0.63955	0.99995	no

ENSG00000099860	ENSG00000099860	GADD45B	19:2476119-2478257	q1	q2	OK	3.01836	2.79881	-0.108952	-0.117305	0.8505	0.99995	no

ENSG00000099864	ENSG00000099864	PALM	19:708952-748329	q1	q2	OK	43.5898	35.5231	-0.295233	-0.644262	0.31765	0.99995	no

ENSG00000099866	ENSG00000099866	MADCAM1	19:489175-519654	q1	q2	OK	0.501999	0.190021	-1.40152	-0.375623	0.41835	0.99995	no

ENSG00000099869	ENSG00000099869	IGF2-AS	11:2150341-2182571	q1	q2	NOTEST	0.334849	0.28387	-0.238277	0	1	1	no

ENSG00000099875	ENSG00000099875	MKNK2	19:2037469-2051243	q1	q2	OK	25.159	22.4687	-0.163158	-0.218783	0.64645	0.99995	no

ENSG00000099889	ENSG00000099889	ARVCF	22:19863039-20004331	q1	q2	OK	41.4813	37.8508	-0.132137	-0.202329	0.7131	0.99995	no

ENSG00000099899	ENSG00000099899	TRMT2A	22:20067754-20114878	q1	q2	OK	26.1366	20.6026	-0.343249	-0.183972	0.59845	0.99995	no

ENSG00000099901	ENSG00000099901	RANBP1	22:20067754-20114878	q1	q2	OK	274.652	267.093	-0.0402633	-0.0698058	0.9197	0.99995	no

ENSG00000099904	ENSG00000099904	ZDHHC8	22:20116978-20135530	q1	q2	OK	28.6737	26.5451	-0.11128	-0.151538	0.81525	0.99995	no

ENSG00000099910	ENSG00000099910	KLHL22	22:20778873-20850170	q1	q2	OK	22.3805	21.5098	-0.0572466	-0.0792934	0.89205	0.99995	no

ENSG00000099917	ENSG00000099917	MED15	22:20850199-20946505	q1	q2	OK	24.7316	23.0996	-0.0984871	-0.175986	0.7283	0.99995	no

ENSG00000099937	ENSG00000099937	SERPIND1	22:21061978-21245506	q1	q2	NOTEST	0.0217923	0.0411401	0.916728	0	1	1	no

ENSG00000099940	ENSG00000099940	SNAP29	22:21061978-21245506	q1	q2	OK	14.7133	14.339	-0.0371791	-0.0370186	0.94505	0.99995	no

ENSG00000099942	ENSG00000099942	CRKL	22:21271713-21308037	q1	q2	OK	36.6653	36.8136	0.00582707	0.0144773	0.9834	0.99995	no

ENSG00000099949	ENSG00000099949	LZTR1	22:21319395-21353327	q1	q2	OK	31.5927	27.7507	-0.187068	-0.255015	0.66855	0.99995	no

ENSG00000099953	ENSG00000099953	MMP11	22:24105207-24126503	q1	q2	OK	5.44037	4.83318	-0.170732	-0.211923	0.69635	0.99995	no

ENSG00000099954	ENSG00000099954	CECR2	22:17840836-18037850	q1	q2	OK	10.536	10.0666	-0.0657467	-0.115273	0.84035	0.99995	no

ENSG00000099956	ENSG00000099956	SMARCB1	22:24129149-24181315	q1	q2	OK	121.352	118.626	-0.0327845	-0.0776189	0.90995	0.99995	no

ENSG00000099957	ENSG00000099957	P2RX6	22:21353392-21399306	q1	q2	OK	1.16806	0.784316	-0.574607	-0.232178	0.50455	0.99995	no

ENSG00000099958	ENSG00000099958	DERL3	22:24129149-24181315	q1	q2	OK	2.02399	2.1164	0.0644109	0.0170652	0.94175	0.99995	no

ENSG00000099960	ENSG00000099960	SLC7A4	22:21353392-21399306	q1	q2	NOTEST	0.0118703	0.0109145	-0.121113	0	1	1	no

ENSG00000099968	ENSG00000099968	BCL2L13	22:18111620-18213388	q1	q2	OK	21.0864	20.8947	-0.0131734	-0.027377	0.96925	0.99995	no

ENSG00000099974	ENSG00000099974	DDTL	22:24309088-24326106	q1	q2	OK	2.06715	1.71102	-0.272781	-0.176253	0.81095	0.99995	no

ENSG00000099977	ENSG00000099977	DDT	22:24309088-24326106	q1	q2	OK	22.9079	21.0325	-0.123223	-0.169158	0.80695	0.99995	no

ENSG00000099984	ENSG00000099984	GSTT2	22:24309088-24326106	q1	q2	NOTEST	0	0	0	0	1	1	no

ENSG00000099985	ENSG00000099985	OSM	22:30658817-30662829	q1	q2	NOTEST	0.0290031	0	#NAME?	0	1	1	no

ENSG00000099991	ENSG00000099991	CABIN1	22:24407641-24574596	q1	q2	OK	18.6718	18.1533	-0.0406348	-0.0491779	0.91095	0.99995	no

ENSG00000099992	ENSG00000099992	TBC1D10A	22:30681105-30723035	q1	q2	OK	4.90913	4.79357	-0.0343679	-0.038924	0.94845	0.99995	no

ENSG00000099994	ENSG00000099994	SUSD2	22:24577226-24585078	q1	q2	NOTEST	0.186634	0.134563	-0.47193	0	1	1	no

ENSG00000099995	ENSG00000099995	SF3A1	22:30727976-30825045	q1	q2	OK	40.3527	35.7186	-0.175992	-0.236646	0.61215	0.99995	no

ENSG00000099998	ENSG00000099998	GGT5	22:24615621-24641110	q1	q2	OK	1.42724	1.57964	0.146368	0.0747631	0.8553	0.99995	no

ENSG00000099999	ENSG00000099999	RNF215	22:30727976-30825045	q1	q2	OK	14.1159	11.4761	-0.298684	-0.135017	0.75865	0.99995	no

ENSG00000100003	ENSG00000100003	SEC14L2	22:30727976-30825045	q1	q2	OK	2.85502	2.32487	-0.296353	-0.0883602	0.7153	0.99995	no

ENSG00000100012	ENSG00000100012	SEC14L3	22:30843945-30868036	q1	q2	NOTEST	0	0.0108726	inf	0	1	1	no

ENSG00000100014	ENSG00000100014	SPECC1L	22:24666785-24924358	q1	q2	OK	31.2201	32.3962	0.0533518	0.0930903	0.86565	0.99995	no

ENSG00000100023	ENSG00000100023	PPIL2	22:22006558-22090123	q1	q2	OK	22.846	20.6311	-0.147123	-0.210554	0.6948	0.99995	no

ENSG00000100024	ENSG00000100024	UPB1	22:24666785-24924358	q1	q2	NOTEST	0.272869	0.135208	-1.01302	0	1	1	no

ENSG00000100027	ENSG00000100027	YPEL1	22:22006558-22090123	q1	q2	OK	10.7321	10.0798	-0.090464	-0.123358	0.85305	0.99995	no

ENSG00000100028	ENSG00000100028	SNRPD3	22:24936405-25024972	q1	q2	OK	120.944	121.595	0.00774414	0.0112212	0.98805	0.99995	no

ENSG00000100029	ENSG00000100029	PES1	22:30972611-31023265	q1	q2	OK	38.5066	36.8253	-0.0644111	-0.116132	0.86245	0.99995	no

ENSG00000100030	ENSG00000100030	MAPK1	22:22108788-22221970	q1	q2	OK	32.4584	32.7528	0.0130273	0.0127201	0.9798	0.99995	no

ENSG00000100031	ENSG00000100031	GGT1	22:24936405-25024972	q1	q2	OK	1.48081	0.950244	-0.640019	-0.122677	0.3731	0.99995	no

ENSG00000100033	ENSG00000100033	PRODH	22:18893540-18924066	q1	q2	NOTEST	0.377227	0.255658	-0.561215	0	1	1	no

ENSG00000100034	ENSG00000100034	PPM1F	22:22273792-22307209	q1	q2	OK	12.1834	11.8852	-0.0357523	-0.0604265	0.9112	0.99995	no

ENSG00000100036	ENSG00000100036	SLC35E4	22:31031638-31065003	q1	q2	OK	1.61713	1.28763	-0.328727	-0.294466	0.67865	0.99995	no

ENSG00000100038	ENSG00000100038	TOP3B	22:22311396-22337213	q1	q2	OK	32.3179	30.3922	-0.0886326	-0.116884	0.8303	0.99995	no

ENSG00000100053	ENSG00000100053	CRYBB3	22:25595816-25603330	q1	q2	NOTEST	0	0.0696321	inf	0	1	1	no

ENSG00000100055	ENSG00000100055	CYTH4	22:37678067-37711382	q1	q2	NOTEST	0.00790393	0.0142389	0.849193	0	1	1	no

ENSG00000100056	ENSG00000100056	DGCR14	22:19117791-19132197	q1	q2	OK	10.3713	9.06466	-0.194274	-0.321313	0.6348	0.99995	no

ENSG00000100058	ENSG00000100058	CRYBB2P1	22:25844071-25916821	q1	q2	OK	29.9663	27.8492	-0.105706	-0.15186	0.8303	0.99995	no

ENSG00000100060	ENSG00000100060	MFNG	22:37865100-37882439	q1	q2	OK	1.36353	1.27851	-0.0928823	-0.0763066	0.8749	0.99995	no

ENSG00000100065	ENSG00000100065	CARD10	22:37886399-37915549	q1	q2	OK	3.91311	3.92077	0.00282279	0.00366471	0.9948	0.99995	no

ENSG00000100068	ENSG00000100068	LRP5L	22:25747384-25801344	q1	q2	OK	2.28388	2.41394	0.0799007	0.0883915	0.89375	0.99995	no

ENSG00000100075	ENSG00000100075	SLC25A1	22:19163094-19166343	q1	q2	OK	2.23357	7.75892	1.7965	2.15286	0.0017	0.901052	no

ENSG00000100077	ENSG00000100077	ADRBK2	22:25960815-26125261	q1	q2	OK	6.78179	6.64429	-0.0295511	-0.0484361	0.92465	0.99995	no

ENSG00000100078	ENSG00000100078	PLA2G3	22:31530794-31536593	q1	q2	OK	4.19196	5.20008	0.310907	0.512685	0.45705	0.99995	no

ENSG00000100079	ENSG00000100079	LGALS2	22:37966254-37978623	q1	q2	NOTEST	0	0	0	0	1	1	no

ENSG00000100083	ENSG00000100083	GGA1	22:38004480-38029571	q1	q2	OK	27.9245	28.7968	0.044378	0.0780387	0.88305	0.99995	no

ENSG00000100084	ENSG00000100084	HIRA	22:19318220-19466738	q1	q2	OK	15.724	14.3836	-0.128543	-0.15128	0.8309	0.99995	no

ENSG00000100092	ENSG00000100092	SH3BP1	22:38030660-38062941	q1	q2	OK	6.81347	6.7187	-0.0202061	-0.0194312	0.969	0.99995	no

ENSG00000100095	ENSG00000100095	SEZ6L	22:26565439-26779562	q1	q2	OK	6.52778	6.08953	-0.100262	-0.170427	0.7595	0.99995	no

ENSG00000100097	ENSG00000100097	LGALS1	22:38071614-38075813	q1	q2	OK	284.126	277.529	-0.03389	-0.0668714	0.9225	0.99995	no

ENSG00000100099	ENSG00000100099	HPS4	22:26825238-26879803	q1	q2	OK	17.0267	17.1224	0.00809194	0.0122834	0.98015	0.99995	no

ENSG00000100100	ENSG00000100100	PIK3IP1	22:31677578-31742218	q1	q2	OK	2.33493	1.78049	-0.391111	-0.147825	0.84545	0.99995	no

ENSG00000100101	ENSG00000100101	NOL12	22:38077679-38172563	q1	q2	OK	16.3222	16.2969	-0.00223366	-0.00182905	0.9979	0.99995	no

ENSG00000100104	ENSG00000100104	SRRD	22:26879842-26908471	q1	q2	OK	16.9904	14.7273	-0.206225	-0.193948	0.7401	0.99995	no

ENSG00000100105	ENSG00000100105	PATZ1	22:31677578-31742218	q1	q2	OK	56.2259	57.0715	0.0215361	0.0473531	0.94365	0.99995	no

ENSG00000100106	ENSG00000100106	TRIOBP	22:38077679-38172563	q1	q2	OK	26.2123	21.5299	-0.283903	-0.470157	0.4697	0.99995	no

ENSG00000100109	ENSG00000100109	TFIP11	22:26879842-26908471	q1	q2	OK	18.1239	19.172	0.0811037	0.107753	0.829	0.99995	no

ENSG00000100116	ENSG00000100116	GCAT	22:38203911-38213183	q1	q2	OK	13.4017	12.9625	-0.0480762	-0.0792691	0.90135	0.99995	no

ENSG00000100121	ENSG00000100121	GGTLC2	22:22988779-22990368	q1	q2	NOTEST	0	0	0	0	1	1	no

ENSG00000100122	ENSG00000100122	CRYBB1	22:26995241-27014052	q1	q2	NOTEST	0.05262	0.0785962	0.578849	0	1	1	no

ENSG00000100124	ENSG00000100124	ANKRD54	22:38226861-38285414	q1	q2	OK	16.3893	14.9925	-0.128511	-0.0691334	0.88035	0.99995	no

ENSG00000100129	ENSG00000100129	EIF3L	22:38226861-38285414	q1	q2	OK	338.681	323.452	-0.0663734	-0.122799	0.83115	0.99995	no

ENSG00000100138	ENSG00000100138	NHP2L1	22:42069933-42094140	q1	q2	OK	78.7056	75.1025	-0.0676052	-0.113536	0.85905	0.99995	no

ENSG00000100139	ENSG00000100139	MICALL1	22:38301663-38338829	q1	q2	OK	15.1385	15.3999	0.0246985	0.0422247	0.9464	0.99995	no

ENSG00000100142	ENSG00000100142	POLR2F	22:38339527-38437922	q1	q2	OK	76.5875	69.5803	-0.138431	-0.265891	0.70545	0.99995	no

ENSG00000100146	ENSG00000100146	SOX10	22:38339527-38437922	q1	q2	NOTEST	0.128053	0.114488	-0.161552	0	1	1	no

ENSG00000100147	ENSG00000100147	CCDC134	22:42196682-42222303	q1	q2	OK	3.3911	3.90241	0.202611	0.251743	0.7184	0.99995	no

ENSG00000100150	ENSG00000100150	DEPDC5	22:32149943-32303012	q1	q2	OK	7.23696	8.27774	0.193855	0.0972143	0.74145	0.99995	no

ENSG00000100151	ENSG00000100151	PICK1	22:38452317-38471708	q1	q2	OK	5.41836	4.35843	-0.314048	-0.370824	0.45665	0.99995	no

ENSG00000100154	ENSG00000100154	TTC28	22:28247656-29075853	q1	q2	OK	14.7093	14.3449	-0.0361856	-0.0362115	0.91355	0.99995	no

ENSG00000100156	ENSG00000100156	SLC16A8	22:38474140-38480100	q1	q2	NOTEST	0.0778079	0.102199	0.393398	0	1	1	no

ENSG00000100162	ENSG00000100162	CENPM	22:42334724-42343168	q1	q2	OK	18.9382	12.9042	-0.553457	-0.890589	0.19035	0.99995	no

ENSG00000100167	ENSG00000100167	Sep-03	22:42372275-42394225	q1	q2	OK	48.1078	45.2819	-0.087336	-0.186215	0.7684	0.99995	no

ENSG00000100170	ENSG00000100170	SLC5A1	22:32439018-32509016	q1	q2	NOTEST	0.0181357	0.0637386	1.81334	0	1	1	no

ENSG00000100181	ENSG00000100181	TPTEP1	22:17082776-17185367	q1	q2	NOTEST	0.110882	0.17077	0.623031	0	1	1	no

ENSG00000100191	ENSG00000100191	SLC5A4	22:32601101-32669097	q1	q2	NOTEST	0.159318	0.247372	0.634775	0	1	1	no

ENSG00000100196	ENSG00000100196	KDELR3	22:38864066-38903665	q1	q2	OK	8.81194	9.22924	0.0667526	0.0127509	0.97315	0.99995	no

ENSG00000100197	ENSG00000100197	CYP2D6	22:42481528-42535938	q1	q2	OK	0.628529	0.453333	-0.471406	-0.305067	0.62495	0.99995	no

ENSG00000100201	ENSG00000100201	DDX17	22:38864066-38903665	q1	q2	OK	304.577	312.071	0.0350693	0.0544426	0.92325	0.99995	no

ENSG00000100206	ENSG00000100206	DMC1	22:38914953-38966291	q1	q2	OK	0.836614	1.5252	0.866364	0.669561	0.28395	0.99995	no

ENSG00000100207	ENSG00000100207	TCF20	22:42556018-42739622	q1	q2	NOTEST	0.274919	0.134294	-1.03361	0	1	1	no

ENSG00000100209	ENSG00000100209	HSCB	22:29083730-29153503	q1	q2	OK	10.4881	11.0123	0.0703596	0.0723384	0.9183	0.99995	no

ENSG00000100211	ENSG00000100211	CBY1	22:39052640-39077825	q1	q2	OK	32.6611	29.3508	-0.154172	-0.279637	0.67065	0.99995	no

ENSG00000100216	ENSG00000100216	TOMM22	22:39077952-39080818	q1	q2	OK	40.6171	42.1407	0.0531274	0.121269	0.86305	0.99995	no

ENSG00000100218	ENSG00000100218	RTDR1	22:23401592-23487208	q1	q2	OK	1.29067	0.90588	-0.510734	-0.161356	0.6959	0.99995	no

ENSG00000100219	ENSG00000100219	XBP1	22:29190542-29196585	q1	q2	OK	64.2855	59.8631	-0.102826	-0.230639	0.7437	0.99995	no

ENSG00000100220	ENSG00000100220	RTCB	22:32783568-32808242	q1	q2	OK	67.9578	67.399	-0.0119129	-0.0271611	0.96555	0.99995	no

ENSG00000100221	ENSG00000100221	JOSD1	22:39081547-39097561	q1	q2	OK	17.5629	18.1761	0.0495119	0.0949474	0.86105	0.99995	no

ENSG00000100225	ENSG00000100225	FBXO7	22:32870662-32894818	q1	q2	OK	44.5409	41.9495	-0.0864776	-0.187945	0.78255	0.99995	no

ENSG00000100226	ENSG00000100226	GTPBP1	22:39101727-39190203	q1	q2	OK	21.4133	19.7991	-0.113067	-0.106637	0.79645	0.99995	no

ENSG00000100227	ENSG00000100227	POLDIP3	22:42979726-43010968	q1	q2	OK	45.9051	44.7036	-0.0382621	-0.0923559	0.89235	0.99995	no

ENSG00000100228	ENSG00000100228	RAB36	22:23487512-23506537	q1	q2	OK	5.40127	4.50392	-0.262118	-0.335268	0.53405	0.99995	no

ENSG00000100234	ENSG00000100234	TIMP3	22:32908538-33454358	q1	q2	OK	33.0245	32.8614	-0.00714137	-0.0156641	0.98225	0.99995	no

ENSG00000100239	ENSG00000100239	PPP6R2	22:50781732-50913454	q1	q2	OK	37.2778	31.539	-0.241179	-0.261923	0.6797	0.99995	no

ENSG00000100241	ENSG00000100241	SBF1	22:50781732-50913454	q1	q2	OK	30.3872	26.689	-0.187217	-0.217786	0.628	0.99995	no

ENSG00000100242	ENSG00000100242	SUN2	22:39101727-39190203	q1	q2	OK	29.1048	24.9305	-0.223343	-0.282778	0.5846	0.99995	no

ENSG00000100243	ENSG00000100243	CYB5R3	22:43013845-43045574	q1	q2	OK	52.1973	45.5475	-0.196602	-0.462335	0.5067	0.99995	no

ENSG00000100246	ENSG00000100246	DNAL4	22:39101727-39190203	q1	q2	OK	13.6038	14.1477	0.0565624	0.0408042	0.9473	0.99995	no

ENSG00000100249	ENSG00000100249	C22orf31	22:29454659-29457832	q1	q2	NOTEST	0.0315234	0.0600903	0.930709	0	1	1	no

ENSG00000100253	ENSG00000100253	MIOX	22:50925212-50929077	q1	q2	NOTEST	0.0327546	0.0988664	1.59378	0	1	1	no

ENSG00000100258	ENSG00000100258	LMF2	22:50941375-50946135	q1	q2	OK	31.0662	26.7935	-0.213462	-0.373333	0.5956	0.99995	no

ENSG00000100263	ENSG00000100263	RHBDD3	22:29655840-29696515	q1	q2	OK	27.2468	23.3219	-0.2244	-0.0972889	0.8285	0.99995	no

ENSG00000100266	ENSG00000100266	PACSIN2	22:43192507-43411151	q1	q2	OK	39.9505	41.4622	0.0535827	0.0988328	0.87945	0.99995	no

ENSG00000100271	ENSG00000100271	TTLL1	22:43434590-43485434	q1	q2	OK	9.65912	9.2979	-0.0549858	-0.093452	0.89155	0.99995	no

ENSG00000100276	ENSG00000100276	RASL10A	22:29708921-29715668	q1	q2	NOTEST	0.40916	0.362186	-0.175934	0	1	1	no

ENSG00000100280	ENSG00000100280	AP1B1	22:29723668-29819182	q1	q2	OK	23.5359	21.5252	-0.128838	-0.281018	0.66315	0.99995	no

ENSG00000100281	ENSG00000100281	HMGXB4	22:35653444-35691800	q1	q2	OK	22.7346	24.9698	0.135296	0.2537	0.6643	0.99995	no

ENSG00000100284	ENSG00000100284	TOM1	22:35694830-35743985	q1	q2	OK	8.0559	6.4536	-0.31994	-0.492803	0.3916	0.99995	no

ENSG00000100285	ENSG00000100285	NEFH	22:29876218-29887379	q1	q2	OK	1.43245	1.51113	0.0771399	0.106311	0.8797	0.99995	no

ENSG00000100288	ENSG00000100288	CHKB	22:51007289-51052409	q1	q2	OK	36.5406	33.8917	-0.108569	-0.117265	0.84545	0.99995	no

ENSG00000100290	ENSG00000100290	BIK	22:43506753-43525718	q1	q2	NOTEST	0.318709	0.400283	0.32878	0	1	1	no

ENSG00000100292	ENSG00000100292	HMOX1	22:35776353-35790207	q1	q2	OK	6.79272	3.85497	-0.817269	-1.09285	0.0896	0.99995	no

ENSG00000100294	ENSG00000100294	MCAT	22:43528211-43539400	q1	q2	OK	12.101	9.43256	-0.359404	-0.524433	0.40075	0.99995	no

ENSG00000100296	ENSG00000100296	THOC5	22:29901867-29977326	q1	q2	OK	17.0491	16.2915	-0.0655754	-0.0568807	0.91955	0.99995	no

ENSG00000100297	ENSG00000100297	MCM5	22:35796055-35821423	q1	q2	OK	57.9262	54.4566	-0.0891103	-0.157761	0.78915	0.99995	no

ENSG00000100298	ENSG00000100298	APOBEC3H	22:39493228-39500072	q1	q2	NOTEST	0.0288237	0.0558382	0.953997	0	1	1	no

ENSG00000100299	ENSG00000100299	ARSA	22:51061181-51066607	q1	q2	OK	13.5254	9.84168	-0.45869	-0.658213	0.32625	0.99995	no

ENSG00000100300	ENSG00000100300	TSPO	22:43547519-43559248	q1	q2	OK	3.00137	3.06889	0.0320985	0.0277161	0.96415	0.99995	no

ENSG00000100302	ENSG00000100302	RASD2	22:35936914-35950048	q1	q2	OK	0.499974	0.518247	0.0517865	0.0575834	0.92945	0.99995	no

ENSG00000100304	ENSG00000100304	TTLL12	22:43562627-43583139	q1	q2	OK	11.5432	10.6375	-0.117887	-0.23224	0.7124	0.99995	no

ENSG00000100307	ENSG00000100307	CBX7	22:39516171-39548679	q1	q2	OK	1.72474	1.02406	-0.752085	-0.706262	0.1455	0.99995	no

ENSG00000100311	ENSG00000100311	PDGFB	22:39619363-39640756	q1	q2	OK	3.61929	3.87795	0.0995861	0.13209	0.8384	0.99995	no

ENSG00000100312	ENSG00000100312	ACR	22:51176623-51183762	q1	q2	NOTEST	0.13579	0.0199827	-2.76455	0	1	1	no

ENSG00000100314	ENSG00000100314	CABP7	22:30116072-30163000	q1	q2	NOTEST	0.385193	0.210533	-0.871538	0	1	1	no

ENSG00000100316	ENSG00000100316	RPL3	22:39708886-39716394	q1	q2	OK	907.266	848.763	-0.0961643	-0.19734	0.7733	0.99995	no

ENSG00000100319	ENSG00000100319	ZMAT5	22:30116072-30163000	q1	q2	OK	15.5878	14.9038	-0.0647367	-0.102264	0.8822	0.99995	no

ENSG00000100320	ENSG00000100320	RBFOX2	22:36134782-36424473	q1	q2	OK	63.4947	70.4399	0.149758	0.263443	0.6288	0.99995	no

ENSG00000100321	ENSG00000100321	SYNGR1	22:39745929-39781593	q1	q2	OK	11.9101	10.6222	-0.165102	-0.278197	0.6679	0.99995	no

ENSG00000100324	ENSG00000100324	TAB1	22:39795745-39833065	q1	q2	OK	18.6788	16.148	-0.210044	-0.326256	0.48875	0.99995	no

ENSG00000100325	ENSG00000100325	ASCC2	22:30184596-30234271	q1	q2	OK	17.2209	15.464	-0.155252	-0.231087	0.6132	0.99995	no

ENSG00000100330	ENSG00000100330	MTMR3	22:30279143-30573064	q1	q2	OK	15.9153	16.4536	0.0479851	0.0597797	0.87175	0.99995	no

ENSG00000100335	ENSG00000100335	MIEF1	22:39895436-39914137	q1	q2	OK	17.753	17.4583	-0.0241567	-0.0481149	0.92905	0.99995	no

ENSG00000100336	ENSG00000100336	APOL4	22:36585171-36600886	q1	q2	OK	0.645383	0.452877	-0.511037	-0.278816	0.4403	0.99995	no

ENSG00000100341	ENSG00000100341	PNPLA5	22:44275557-44287893	q1	q2	NOTEST	0	0	0	0	1	1	no

ENSG00000100342	ENSG00000100342	APOL1	22:36649055-36663576	q1	q2	NOTEST	0.0559743	0.0521303	-0.102642	0	1	1	no

ENSG00000100344	ENSG00000100344	PNPLA3	22:44319618-44568829	q1	q2	OK	8.94901	10.5097	0.231919	0.274438	0.67795	0.99995	no

ENSG00000100345	ENSG00000100345	MYH9	22:36677326-36784063	q1	q2	OK	35.2266	37.9029	0.105642	0.185497	0.706	0.99995	no

ENSG00000100346	ENSG00000100346	CACNA1I	22:39966757-40085742	q1	q2	NOTEST	0.0872617	0.065228	-0.419857	0	1	1	no

ENSG00000100347	ENSG00000100347	SAMM50	22:44319618-44568829	q1	q2	OK	30.8116	30.0962	-0.0338955	-0.063179	0.9291	0.99995	no

ENSG00000100348	ENSG00000100348	TXN2	22:36863082-36878077	q1	q2	OK	43.8121	39.4887	-0.149891	-0.333354	0.63135	0.99995	no

ENSG00000100350	ENSG00000100350	FOXRED2	22:36883236-36903148	q1	q2	OK	6.47826	5.93651	-0.125991	-0.138003	0.8099	0.99995	no

ENSG00000100351	ENSG00000100351	GRAP2	22:40297085-40369725	q1	q2	NOTEST	0.220389	0.093122	-1.24286	0	1	1	no

ENSG00000100353	ENSG00000100353	EIF3D	22:36906896-36925483	q1	q2	OK	93.0951	88.6987	-0.0697926	-0.142475	0.824	0.99995	no

ENSG00000100354	ENSG00000100354	TNRC6B	22:40440820-40731812	q1	q2	OK	9.45078	8.24226	-0.197395	-0.197491	0.4995	0.99995	no

ENSG00000100359	ENSG00000100359	SGSM3	22:40742506-41032706	q1	q2	OK	34.1368	30.1113	-0.18102	-0.192351	0.69875	0.99995	no

ENSG00000100360	ENSG00000100360	IFT27	22:37154245-37172300	q1	q2	OK	35.0347	30.7015	-0.190472	-0.250946	0.66755	0.99995	no

ENSG00000100362	ENSG00000100362	PVALB	22:37196727-37215780	q1	q2	OK	0.626061	0.55495	-0.173946	-0.0990373	0.83245	0.99995	no

ENSG00000100364	ENSG00000100364	KIAA0930	22:45586218-45636650	q1	q2	OK	16.3959	14.7009	-0.157436	-0.294947	0.64055	0.99995	no

ENSG00000100365	ENSG00000100365	NCF4	22:37243414-37274057	q1	q2	NOTEST	0	0	0	0	1	1	no

ENSG00000100368	ENSG00000100368	CSF2RB	22:37309669-37336491	q1	q2	NOTEST	0	0.00492159	inf	0	1	1	no

ENSG00000100372	ENSG00000100372	SLC25A17	22:41165633-41215403	q1	q2	OK	15.8492	16.4736	0.0557523	0.108374	0.86825	0.99995	no

ENSG00000100373	ENSG00000100373	UPK3A	22:45680862-45691755	q1	q2	NOTEST	0	0	0	0	1	1	no

ENSG00000100376	ENSG00000100376	FAM118A	22:45704848-45737836	q1	q2	OK	8.86442	9.26356	0.0635407	0.0829585	0.89595	0.99995	no

ENSG00000100379	ENSG00000100379	KCTD17	22:37447778-37459430	q1	q2	OK	36.2331	32.9384	-0.137537	-0.273072	0.6905	0.99995	no

ENSG00000100380	ENSG00000100380	ST13	22:41220538-41253026	q1	q2	OK	143.258	145.792	0.0253001	0.0386969	0.9563	0.99995	no

ENSG00000100385	ENSG00000100385	IL2RB	22:37521877-37595425	q1	q2	NOTEST	0.347555	0.31031	-0.163534	0	1	1	no

ENSG00000100387	ENSG00000100387	RBX1	22:41253080-41369313	q1	q2	OK	61.6778	61.826	0.00346334	0.00651058	0.99235	0.99995	no

ENSG00000100393	ENSG00000100393	EP300	22:41487789-41593460	q1	q2	OK	20.5879	20.7655	0.0123946	0.0300859	0.9656	0.99995	no

ENSG00000100395	ENSG00000100395	L3MBTL2	22:41601208-41636938	q1	q2	OK	13.5929	11.7069	-0.215489	-0.3917	0.5184	0.99995	no

ENSG00000100399	ENSG00000100399	CHADL	22:41601208-41636938	q1	q2	NOTEST	0.0448161	0.078508	0.808824	0	1	1	no

ENSG00000100401	ENSG00000100401	RANGAP1	22:41641614-41682255	q1	q2	OK	43.7227	39.3298	-0.152758	-0.355922	0.59245	0.99995	no

ENSG00000100403	ENSG00000100403	ZC3H7B	22:41697525-41756151	q1	q2	OK	32.4979	31.7703	-0.0326695	-0.0815718	0.9098	0.99995	no

ENSG00000100410	ENSG00000100410	PHF5A	22:41855720-41864729	q1	q2	OK	51.9829	55.8204	0.102755	0.231054	0.74115	0.99995	no

ENSG00000100412	ENSG00000100412	ACO2	22:41865128-41940610	q1	q2	OK	33.5293	31.3433	-0.0972635	-0.155624	0.775	0.99995	no

ENSG00000100413	ENSG00000100413	POLR3H	22:41865128-41940610	q1	q2	OK	15.0645	12.3521	-0.2864	-0.337401	0.56345	0.99995	no

ENSG00000100416	ENSG00000100416	TRMU	22:46726771-46753237	q1	q2	OK	34.9457	34.7319	-0.00885473	-0.0176735	0.9796	0.99995	no

ENSG00000100417	ENSG00000100417	PMM1	22:41956766-41985894	q1	q2	OK	13.5388	11.435	-0.243649	-0.384102	0.58085	0.99995	no

ENSG00000100418	ENSG00000100418	DESI1	22:41994031-42017100	q1	q2	OK	15.723	15.0959	-0.0587124	-0.0915575	0.8628	0.99995	no

ENSG00000100422	ENSG00000100422	CERK	22:47080307-47134158	q1	q2	OK	41.4136	42.3886	0.0335714	0.0798907	0.90215	0.99995	no

ENSG00000100425	ENSG00000100425	BRD1	22:50166930-50221160	q1	q2	OK	24.4593	22.9503	-0.0918698	-0.177543	0.755	0.99995	no

ENSG00000100426	ENSG00000100426	ZBED4	22:50247489-50283726	q1	q2	OK	17.7335	18.2045	0.0378141	0.092559	0.8959	0.99995	no

ENSG00000100427	ENSG00000100427	MLC1	22:50497819-50524331	q1	q2	OK	31.7331	32.1763	0.0200111	0.0342096	0.94545	0.99995	no

ENSG00000100429	ENSG00000100429	HDAC10	22:50683611-50700254	q1	q2	OK	15.9931	13.8834	-0.204083	-0.217754	0.7423	0.99995	no

ENSG00000100433	ENSG00000100433	KCNK10	14:88649112-88793251	q1	q2	OK	2.30385	2.1144	-0.123798	-0.142041	0.7963	0.99995	no

ENSG00000100439	ENSG00000100439	ABHD4	14:23064714-23081265	q1	q2	OK	11.3861	8.84154	-0.364908	-0.653165	0.28335	0.99995	no

ENSG00000100441	ENSG00000100441	KHNYN	14:24895737-24912111	q1	q2	OK	21.705	20.0011	-0.117951	-0.184785	0.76235	0.99995	no

ENSG00000100442	ENSG00000100442	FKBP3	14:45553301-45604522	q1	q2	OK	133.782	141.402	0.0799193	0.156098	0.82115	0.99995	no

ENSG00000100445	ENSG00000100445	SDR39U1	14:24895737-24912111	q1	q2	OK	45.7371	38.864	-0.234932	-0.251363	0.7009	0.99995	no

ENSG00000100448	ENSG00000100448	CTSG	14:25042727-25045466	q1	q2	NOTEST	0.0215127	0	#NAME?	0	1	1	no

ENSG00000100450	ENSG00000100450	GZMH	14:25064928-25126980	q1	q2	NOTEST	0	0	0	0	1	1	no

ENSG00000100453	ENSG00000100453	GZMB	14:25064928-25126980	q1	q2	NOTEST	0	0	0	0	1	1	no

ENSG00000100461	ENSG00000100461	RBM23	14:23369853-23388393	q1	q2	OK	54.263	48.8121	-0.15273	-0.279673	0.58645	0.99995	no

ENSG00000100462	ENSG00000100462	PRMT5	14:23388664-23398794	q1	q2	OK	55.7861	54.5704	-0.0317861	-0.0543956	0.9094	0.99995	no

ENSG00000100473	ENSG00000100473	COCH	14:31343719-31562818	q1	q2	OK	12.9544	12.5956	-0.0405222	-0.0435271	0.9407	0.99995	no

ENSG00000100478	ENSG00000100478	AP4S1	14:31343719-31562818	q1	q2	OK	5.32736	6.6643	0.323032	0.171138	0.8191	0.99995	no

ENSG00000100479	ENSG00000100479	POLE2	14:50110272-50155140	q1	q2	OK	15.9173	15.9889	0.0064804	0.00892765	0.98765	0.99995	no

ENSG00000100483	ENSG00000100483	VCPKMT	14:50575349-50583318	q1	q2	OK	7.67137	8.7642	0.192138	0.308175	0.65675	0.99995	no

ENSG00000100485	ENSG00000100485	SOS2	14:50583846-50698276	q1	q2	OK	14.128	15.6177	0.144622	0.302421	0.6409	0.99995	no

ENSG00000100490	ENSG00000100490	CDKL1	14:50704280-50883179	q1	q2	OK	3.22648	4.25554	0.399382	0.268533	0.6499	0.99995	no

ENSG00000100503	ENSG00000100503	NIN	14:51186480-51297839	q1	q2	OK	36.5258	41.0627	0.168912	0.247607	0.58005	0.99995	no

ENSG00000100504	ENSG00000100504	PYGL	14:51314839-51411454	q1	q2	OK	5.30903	5.62689	0.083888	0.104077	0.8642	0.99995	no

ENSG00000100505	ENSG00000100505	TRIM9	14:51441979-51562779	q1	q2	OK	6.02762	6.69238	0.15093	0.178963	0.69865	0.99995	no

ENSG00000100519	ENSG00000100519	PSMC6	14:53173889-53195305	q1	q2	OK	85.4708	90.8326	0.0877785	0.200065	0.7579	0.99995	no

ENSG00000100522	ENSG00000100522	GNPNAT1	14:53241911-53258386	q1	q2	OK	13.8662	16.1544	0.220359	0.448289	0.46265	0.99995	no

ENSG00000100523	ENSG00000100523	DDHD1	14:53510685-53620000	q1	q2	OK	8.12103	8.4682	0.0603926	0.073185	0.87265	0.99995	no

ENSG00000100526	ENSG00000100526	CDKN3	14:54863566-54886936	q1	q2	OK	33.1078	35.1474	0.0862462	0.155476	0.8202	0.99995	no

ENSG00000100528	ENSG00000100528	CNIH1	14:54893653-54908149	q1	q2	OK	57.6564	60.1092	0.0601065	0.141418	0.8404	0.99995	no

ENSG00000100532	ENSG00000100532	CGRRF1	14:54976529-55005567	q1	q2	OK	14.5511	13.3247	-0.127023	-0.198982	0.76325	0.99995	no

ENSG00000100554	ENSG00000100554	ATP6V1D	14:67707825-67853233	q1	q2	OK	44.3843	46.6703	0.0724545	0.0679967	0.91795	0.99995	no

ENSG00000100557	ENSG00000100557	C14orf105	14:57936018-57960585	q1	q2	NOTEST	0.0374691	0.0680061	0.859963	0	1	1	no

ENSG00000100558	ENSG00000100558	PLEK2	14:67853699-67878917	q1	q2	NOTEST	0.018558	0.0170975	-0.118261	0	1	1	no

ENSG00000100564	ENSG00000100564	PIGH	14:67913800-68067004	q1	q2	OK	12.4291	12.0159	-0.0487868	-0.0435104	0.94185	0.99995	no

ENSG00000100565	ENSG00000100565	C14orf166B	14:77292714-77336645	q1	q2	NOTEST	0	0	0	0	1	1	no

ENSG00000100567	ENSG00000100567	PSMA3	14:58466452-58764857	q1	q2	OK	129.454	142.835	0.141906	0.1879	0.7908	0.99995	no

ENSG00000100568	ENSG00000100568	VTI1B	14:68086514-68162531	q1	q2	OK	53.0027	53.1672	0.00446988	0.00490731	0.99415	0.99995	no

ENSG00000100575	ENSG00000100575	TIMM9	14:58862633-59015216	q1	q2	OK	22.9495	24.0869	0.0697864	0.0668395	0.91235	0.99995	no

ENSG00000100577	ENSG00000100577	GSTZ1	14:77741298-77797940	q1	q2	OK	16.9208	14.4436	-0.228369	-0.235932	0.74015	0.99995	no

ENSG00000100578	ENSG00000100578	KIAA0586	14:58862633-59015216	q1	q2	OK	17.4963	17.9846	0.0397112	0.0486483	0.92305	0.99995	no

ENSG00000100580	ENSG00000100580	TMED8	14:77801363-77857840	q1	q2	OK	9.09524	9.88605	0.120283	0.277159	0.68915	0.99995	no

ENSG00000100583	ENSG00000100583	SAMD15	14:77801363-77857840	q1	q2	OK	2.44049	2.83328	0.2153	0.103825	0.8513	0.99995	no

ENSG00000100591	ENSG00000100591	AHSA1	14:77893017-77935817	q1	q2	OK	79.0743	78.2965	-0.0142612	-0.0270319	0.968	0.99995	no

ENSG00000100592	ENSG00000100592	DAAM1	14:59655363-59838123	q1	q2	OK	40.994	41.966	0.033808	0.0434793	0.94045	0.99995	no

ENSG00000100593	ENSG00000100593	ISM2	14:77940739-77965210	q1	q2	NOTEST	0.00982891	0	#NAME?	0	1	1	no

ENSG00000100596	ENSG00000100596	SPTLC2	14:77972339-78083116	q1	q2	OK	9.1039	10.2591	0.172353	0.360504	0.5533	0.99995	no

ENSG00000100599	ENSG00000100599	RIN3	14:92980117-93155339	q1	q2	OK	4.05548	3.73884	-0.117281	-0.175751	0.774	0.99995	no

ENSG00000100600	ENSG00000100600	LGMN	14:93170151-93215047	q1	q2	OK	19.1086	16.9657	-0.171597	-0.299062	0.6125	0.99995	no

ENSG00000100601	ENSG00000100601	ALKBH1	14:78138746-78174363	q1	q2	OK	8.23157	8.83787	0.10253	0.160334	0.79425	0.99995	no

ENSG00000100603	ENSG00000100603	SNW1	14:78174413-78236085	q1	q2	OK	52.5308	55.6047	0.0820426	0.176242	0.8041	0.99995	no

ENSG00000100604	ENSG00000100604	CHGA	14:93389424-93401638	q1	q2	OK	1.72756	1.65123	-0.0651865	-0.074322	0.90715	0.99995	no

ENSG00000100605	ENSG00000100605	ITPK1	14:93403258-93582665	q1	q2	OK	13.1804	11.5486	-0.190675	-0.338606	0.57075	0.99995	no

ENSG00000100612	ENSG00000100612	DHRS7	14:60558628-60636574	q1	q2	OK	20.8199	20.5522	-0.0186717	-0.0153302	0.98375	0.99995	no

ENSG00000100614	ENSG00000100614	PPM1A	14:60706838-60765805	q1	q2	OK	20.7521	20.4986	-0.0177302	-0.0304835	0.9641	0.99995	no

ENSG00000100625	ENSG00000100625	SIX4	14:61176245-61191066	q1	q2	OK	1.64837	1.6618	0.0117049	0.015917	0.98145	0.99995	no

ENSG00000100626	ENSG00000100626	GALNT16	14:69649736-69821183	q1	q2	OK	21.3535	17.6088	-0.278179	-0.391556	0.48985	0.99995	no

ENSG00000100628	ENSG00000100628	ASB2	14:94400498-94443137	q1	q2	NOTEST	0.186152	0.155677	-0.257924	0	1	1	no

ENSG00000100629	ENSG00000100629	CEP128	14:80943329-81636758	q1	q2	OK	4.78093	5.83056	0.286341	0.339962	0.44315	0.99995	no

ENSG00000100632	ENSG00000100632	ERH	14:69846847-69929105	q1	q2	OK	223.623	243.829	0.1248	0.236805	0.7345	0.99995	no

ENSG00000100644	ENSG00000100644	HIF1A	14:62147758-62263146	q1	q2	OK	113.283	127.293	0.168216	0.292272	0.58045	0.99995	no

ENSG00000100647	ENSG00000100647	KIAA0247	14:70078312-70181859	q1	q2	OK	6.10132	5.18853	-0.233797	-0.316018	0.5208	0.99995	no

ENSG00000100650	ENSG00000100650	SRSF5	14:70193616-70238722	q1	q2	OK	247.415	241.807	-0.0330759	-0.0619232	0.9253	0.99995	no

ENSG00000100652	ENSG00000100652	SLC10A1	14:70242133-70264006	q1	q2	NOTEST	0.0270682	0.0506932	0.905193	0	1	1	no

ENSG00000100664	ENSG00000100664	EIF5	14:103799880-103811362	q1	q2	OK	193.672	196.452	0.0205644	0.0272455	0.965	0.99995	no

ENSG00000100665	ENSG00000100665	SERPINA4	14:95027427-95090983	q1	q2	NOTEST	0	0	0	0	1	1	no

ENSG00000100678	ENSG00000100678	SLC8A3	14:70510933-70696904	q1	q2	OK	3.09287	3.48293	0.171356	0.252365	0.70825	0.99995	no

ENSG00000100697	ENSG00000100697	DICER1	14:95552564-95646263	q1	q2	OK	30.4002	30.4553	0.00261262	0.00301453	0.99355	0.99995	no

ENSG00000100711	ENSG00000100711	ZFYVE21	14:104182066-104200005	q1	q2	OK	30.0063	31.7782	0.0827698	0.148503	0.80825	0.99995	no

ENSG00000100714	ENSG00000100714	MTHFD1	14:64854748-65009955	q1	q2	OK	32.4867	35.1693	0.114464	0.119349	0.78565	0.99995	no

ENSG00000100721	ENSG00000100721	TCL1A	14:96176303-96223993	q1	q2	NOTEST	0	0	0	0	1	1	no

ENSG00000100722	ENSG00000100722	ZC3H14	14:89029252-89259096	q1	q2	OK	41.9584	44.0104	0.0688837	0.129315	0.83195	0.99995	no

ENSG00000100726	ENSG00000100726	TELO2	16:1543344-1662111	q1	q2	OK	24.6093	21.3528	-0.204782	-0.232838	0.65635	0.99995	no

ENSG00000100731	ENSG00000100731	PCNX	14:71374121-71582099	q1	q2	OK	14.0336	12.7093	-0.143	-0.193515	0.65815	0.99995	no

ENSG00000100739	ENSG00000100739	BDKRB1	14:96671015-96735304	q1	q2	NOTEST	0.0188796	0	#NAME?	0	1	1	no

ENSG00000100744	ENSG00000100744	GSKIP	14:96747594-96853625	q1	q2	OK	22.6646	25.2607	0.156452	0.232598	0.72865	0.99995	no

ENSG00000100749	ENSG00000100749	VRK1	14:97263640-97411731	q1	q2	OK	34.5629	36.3181	0.0714646	0.14847	0.81105	0.99995	no

ENSG00000100764	ENSG00000100764	PSMC1	14:90722838-90738968	q1	q2	OK	100.961	102.984	0.0286228	0.06824	0.9178	0.99995	no

ENSG00000100767	ENSG00000100767	PAPLN	14:73704204-73741348	q1	q2	OK	2.3997	1.60656	-0.578874	-0.504077	0.39275	0.99995	no

ENSG00000100784	ENSG00000100784	RPS6KA5	14:91336798-91691976	q1	q2	OK	1.01892	0.805111	-0.339785	-0.238141	0.66025	0.99995	no

ENSG00000100796	ENSG00000100796	SMEK1	14:91923954-91976898	q1	q2	OK	64.083	67.1004	0.0663784	0.118216	0.86435	0.99995	no

ENSG00000100802	ENSG00000100802	C14orf93	14:23451906-23479375	q1	q2	OK	4.38225	4.64691	0.0845981	0.10234	0.8476	0.99995	no

ENSG00000100804	ENSG00000100804	PSMB5	14:23485751-23504439	q1	q2	OK	117.777	113.42	-0.0543781	-0.125531	0.8537	0.99995	no

ENSG00000100811	ENSG00000100811	YY1	14:100673743-100772884	q1	q2	OK	56.72	60.6165	0.0958533	0.0988387	0.8759	0.99995	no

ENSG00000100813	ENSG00000100813	ACIN1	14:23527772-23569665	q1	q2	OK	141.879	134.801	-0.0738282	-0.107994	0.837	0.99995	no

ENSG00000100814	ENSG00000100814	CCNB1IP1	14:20779526-20801471	q1	q2	OK	63.8454	72.1217	0.17585	0.41108	0.53905	0.99995	no

ENSG00000100815	ENSG00000100815	TRIP11	14:92432334-92507240	q1	q2	OK	7.64433	8.11339	0.0859158	0.166251	0.78675	0.99995	no

ENSG00000100823	ENSG00000100823	APEX1	14:20923349-20929771	q1	q2	OK	217.93	215.98	-0.012966	-0.0282071	0.96725	0.99995	no

ENSG00000100836	ENSG00000100836	PABPN1	14:23654524-23795394	q1	q2	OK	176.53	172.866	-0.0302589	-0.0502706	0.9416	0.99995	no

ENSG00000100842	ENSG00000100842	EFS	14:23825610-23834961	q1	q2	OK	37.4627	35.6041	-0.0734118	-0.175605	0.79955	0.99995	no

ENSG00000100852	ENSG00000100852	ARHGAP5	14:32544624-32628934	q1	q2	OK	35.4985	36.6353	0.0454778	0.101377	0.88325	0.99995	no

ENSG00000100865	ENSG00000100865	CINP	14:102783713-102829253	q1	q2	OK	20.5292	21.4369	0.0624211	0.0862506	0.89785	0.99995	no

ENSG00000100867	ENSG00000100867	DHRS2	14:24099323-24114848	q1	q2	NOTEST	0.210903	0.23196	0.137293	0	1	1	no

ENSG00000100883	ENSG00000100883	SRP54	14:35390063-35498773	q1	q2	OK	37.7061	40.8023	0.113854	0.22087	0.6997	0.99995	no

ENSG00000100884	ENSG00000100884	CPNE6	14:24540045-24547309	q1	q2	NOTEST	0.0151941	0	#NAME?	0	1	1	no

ENSG00000100888	ENSG00000100888	CHD8	14:21853352-21924285	q1	q2	OK	37.7	37.6319	-0.00260865	-0.00286495	0.99355	0.99995	no

ENSG00000100889	ENSG00000100889	PCK2	14:24549315-24602058	q1	q2	OK	11.3424	10.5285	-0.107416	-0.122398	0.84695	0.99995	no

ENSG00000100890	ENSG00000100890	KIAA0391	14:35514112-35786699	q1	q2	OK	17.9716	16.0562	-0.162582	-0.113471	0.83815	0.99995	no

ENSG00000100897	ENSG00000100897	DCAF11	14:24549315-24602058	q1	q2	OK	21.1695	20.9991	-0.0116606	-0.0170299	0.9719	0.99995	no

ENSG00000100902	ENSG00000100902	PSMA6	14:35514112-35786699	q1	q2	OK	137.732	147.914	0.102899	0.179038	0.79575	0.99995	no

ENSG00000100906	ENSG00000100906	NFKBIA	14:35870716-35873955	q1	q2	OK	11.9176	11.1394	-0.0974248	-0.148825	0.8032	0.99995	no

ENSG00000100908	ENSG00000100908	EMC9	14:24605366-24610797	q1	q2	OK	15.7008	15.1493	-0.051589	-0.0477217	0.94745	0.99995	no

ENSG00000100911	ENSG00000100911	PSME2	14:24612573-24636611	q1	q2	OK	45.1486	52.2739	0.21141	0.294197	0.6462	0.99995	no

ENSG00000100916	ENSG00000100916	BRMS1L	14:36288429-36536396	q1	q2	OK	12.4072	14.2687	0.201678	0.303416	0.56555	0.99995	no

ENSG00000100918	ENSG00000100918	REC8	14:24641061-24708448	q1	q2	OK	5.39202	3.73625	-0.529238	-0.132828	0.4403	0.99995	no

ENSG00000100926	ENSG00000100926	TM9SF1	14:24641061-24708448	q1	q2	OK	29.1055	30.4487	0.0650855	0.0606897	0.92745	0.99995	no

ENSG00000100934	ENSG00000100934	SEC23A	14:39501122-39578850	q1	q2	OK	29.9696	32.8472	0.13227	0.260242	0.6483	0.99995	no

ENSG00000100938	ENSG00000100938	GMPR2	14:24641061-24708448	q1	q2	OK	33.8416	33.3332	-0.0218348	-0.0186176	0.97175	0.99995	no

ENSG00000100941	ENSG00000100941	PNN	14:39644088-39652422	q1	q2	OK	116.393	124.081	0.0922824	0.205129	0.7484	0.99995	no

ENSG00000100949	ENSG00000100949	RABGGTA	14:24734743-24740945	q1	q2	OK	9.02819	7.32993	-0.300638	-0.401425	0.5087	0.99995	no

ENSG00000100968	ENSG00000100968	NFATC4	14:24834878-24848810	q1	q2	OK	28.2756	22.1676	-0.351107	-0.494179	0.4245	0.99995	no

ENSG00000100979	ENSG00000100979	PLTP	20:44517263-44540794	q1	q2	OK	50.0391	41.6652	-0.264212	-0.504049	0.47045	0.99995	no

ENSG00000100982	ENSG00000100982	PCIF1	20:44563266-44576662	q1	q2	OK	17.0458	15.2993	-0.15595	-0.343149	0.61935	0.99995	no

ENSG00000100983	ENSG00000100983	GSS	20:33516235-33543620	q1	q2	OK	24.1998	21.5671	-0.166163	-0.361646	0.6129	0.99995	no

ENSG00000100985	ENSG00000100985	MMP9	20:44637546-44688784	q1	q2	OK	5.13325	4.66811	-0.137035	-0.112724	0.9146	0.99995	no

ENSG00000100987	ENSG00000100987	VSX1	20:25051520-25062996	q1	q2	OK	1.88724	1.70568	-0.145933	-0.142936	0.8175	0.99995	no

ENSG00000100991	ENSG00000100991	TRPC4AP	20:33563205-33680674	q1	q2	OK	34.2969	33.0758	-0.0523048	-0.125166	0.86385	0.99995	no

ENSG00000100994	ENSG00000100994	PYGB	20:25228704-25371619	q1	q2	OK	41.5926	37.4023	-0.153202	-0.282147	0.6295	0.99995	no

ENSG00000100997	ENSG00000100997	ABHD12	20:25228704-25371619	q1	q2	OK	25.0999	20.7965	-0.271336	-0.286346	0.63885	0.99995	no

ENSG00000101000	ENSG00000101000	PROCR	20:33703166-33872788	q1	q2	OK	4.37414	3.95453	-0.145493	-0.0751687	0.937	0.99995	no

ENSG00000101003	ENSG00000101003	GINS1	20:25388362-25433264	q1	q2	OK	27.0338	26.7332	-0.0161286	-0.0320145	0.9548	0.99995	no

ENSG00000101004	ENSG00000101004	NINL	20:25433340-25566153	q1	q2	OK	36.0305	33.3288	-0.112446	-0.173465	0.7458	0.99995	no

ENSG00000101017	ENSG00000101017	CD40	20:44746910-44758502	q1	q2	NOTEST	0.473223	0.35667	-0.407928	0	1	1	no

ENSG00000101019	ENSG00000101019	UQCC1	20:33890368-33999944	q1	q2	OK	26.7195	27.0055	0.0153608	0.0263554	0.9608	0.99995	no

ENSG00000101040	ENSG00000101040	ZMYND8	20:45837858-45985567	q1	q2	OK	42.3813	44.0133	0.0545138	0.122308	0.8476	0.99995	no

ENSG00000101049	ENSG00000101049	SGK2	20:42187607-42216877	q1	q2	NOTEST	0.437204	0.349309	-0.323803	0	1	1	no

ENSG00000101052	ENSG00000101052	IFT52	20:42219570-42275936	q1	q2	OK	37.596	38.8825	0.0485417	0.100011	0.86885	0.99995	no

ENSG00000101057	ENSG00000101057	MYBL2	20:42295753-42345136	q1	q2	OK	53.3223	48.3782	-0.140382	-0.344618	0.61815	0.99995	no

ENSG00000101074	ENSG00000101074	R3HDML	20:42965625-42983825	q1	q2	NOTEST	0	0	0	0	1	1	no

ENSG00000101076	ENSG00000101076	HNF4A	20:42984339-43061485	q1	q2	NOTEST	0	0	0	0	1	1	no

ENSG00000101079	ENSG00000101079	NDRG3	20:35280168-35374533	q1	q2	OK	16.3774	15.6514	-0.0654099	-0.144285	0.83525	0.99995	no

ENSG00000101082	ENSG00000101082	SLA2	20:34894257-35274619	q1	q2	NOTEST	0.12351	0.0930741	-0.408172	0	1	1	no

ENSG00000101084	ENSG00000101084	C20orf24	20:34894257-35274619	q1	q2	OK	60.073	57.4804	-0.0636471	-0.0652103	0.92145	0.99995	no

ENSG00000101096	ENSG00000101096	NFATC2	20:50003493-50179370	q1	q2	NOTEST	0.271132	0.336783	0.312828	0	1	1	no

ENSG00000101098	ENSG00000101098	RIMS4	20:43380448-43438979	q1	q2	OK	2.7205	2.6646	-0.0299512	-0.0514432	0.9412	0.99995	no

ENSG00000101104	ENSG00000101104	PABPC1L	20:43538702-43589127	q1	q2	OK	28.2435	27.2063	-0.0539816	-0.0766656	0.8908	0.99995	no

ENSG00000101109	ENSG00000101109	STK4	20:43595114-43708600	q1	q2	OK	13.1269	14.7491	0.168094	0.344608	0.5608	0.99995	no

ENSG00000101115	ENSG00000101115	SALL4	20:50400580-50419059	q1	q2	OK	23.1361	21.5506	-0.102423	-0.139579	0.8239	0.99995	no

ENSG00000101126	ENSG00000101126	ADNP	20:49505584-49575092	q1	q2	OK	61.54	65.8544	0.0977556	0.202752	0.7536	0.99995	no

ENSG00000101132	ENSG00000101132	PFDN4	20:52824385-52844591	q1	q2	OK	64.4079	68.0501	0.0793609	0.152399	0.82635	0.99995	no

ENSG00000101134	ENSG00000101134	DOK5	20:53092135-53267710	q1	q2	OK	28.7401	26.9581	-0.0923477	-0.207354	0.76335	0.99995	no

ENSG00000101138	ENSG00000101138	CSTF1	20:54967426-54979518	q1	q2	OK	21.6543	22.1561	0.0330499	0.0637998	0.92575	0.99995	no

ENSG00000101144	ENSG00000101144	BMP7	20:55743803-55841685	q1	q2	OK	3.6723	3.23179	-0.184347	-0.273663	0.6486	0.99995	no

ENSG00000101146	ENSG00000101146	RAE1	20:55926065-55954267	q1	q2	OK	33.1267	33.3478	0.00959689	0.0205001	0.97705	0.99995	no

ENSG00000101150	ENSG00000101150	TPD52L2	20:62496595-62522898	q1	q2	OK	43.0853	41.8785	-0.0409866	-0.095414	0.88825	0.99995	no

ENSG00000101152	ENSG00000101152	DNAJC5	20:62526517-62567384	q1	q2	OK	24.3975	22.5995	-0.110446	-0.24224	0.70275	0.99995	no

ENSG00000101158	ENSG00000101158	NELFCD	20:57556262-57570188	q1	q2	OK	81.858	80.9677	-0.015777	-0.0334961	0.9578	0.99995	no

ENSG00000101160	ENSG00000101160	CTSZ	20:57570239-57582302	q1	q2	OK	0.603255	0.480849	-0.327184	-0.240547	0.682	0.99995	no

ENSG00000101161	ENSG00000101161	PRPF6	20:62588054-62680994	q1	q2	OK	39.1688	36.4814	-0.102544	-0.183353	0.79215	0.99995	no

ENSG00000101162	ENSG00000101162	TUBB1	20:57594308-57607437	q1	q2	NOTEST	0.261965	0.364455	0.476364	0	1	1	no

ENSG00000101166	ENSG00000101166	SLMO2	20:57608199-57617964	q1	q2	OK	10.9398	13.6213	0.316269	0.563293	0.359	0.99995	no

ENSG00000101180	ENSG00000101180	HRH3	20:60790025-60795323	q1	q2	OK	1.70853	1.28948	-0.405966	-0.504059	0.4634	0.99995	no

ENSG00000101181	ENSG00000101181	MTG2	20:60758084-60778624	q1	q2	OK	22.4119	18.4859	-0.277839	-0.394054	0.56585	0.99995	no

ENSG00000101182	ENSG00000101182	PSMA7	20:60711790-60718496	q1	q2	OK	188.255	182.995	-0.0408857	-0.100728	0.88705	0.99995	no

ENSG00000101187	ENSG00000101187	SLCO4A1	20:61272070-61317137	q1	q2	OK	1.21233	0.856076	-0.501972	-0.338643	0.5414	0.99995	no

ENSG00000101188	ENSG00000101188	NTSR1	20:61340188-61394123	q1	q2	NOTEST	0.133221	0.0931773	-0.515776	0	1	1	no

ENSG00000101189	ENSG00000101189	MRGBP	20:61427804-61431945	q1	q2	OK	21.8361	20.1992	-0.112416	-0.233443	0.7433	0.99995	no

ENSG00000101190	ENSG00000101190	TCFL5	20:61447595-61493115	q1	q2	OK	5.95247	4.90669	-0.278737	-0.420167	0.5462	0.99995	no

ENSG00000101191	ENSG00000101191	DIDO1	20:61509089-61569304	q1	q2	OK	24.1718	23.3352	-0.0508126	-0.115119	0.87185	0.99995	no

ENSG00000101193	ENSG00000101193	GID8	20:61569470-61576996	q1	q2	OK	43.9731	47.0391	0.0972373	0.185068	0.7491	0.99995	no

ENSG00000101194	ENSG00000101194	SLC17A9	20:61584051-61599949	q1	q2	NOTEST	0.137166	0.027174	-2.33562	0	1	1	no

ENSG00000101197	ENSG00000101197	BIRC7	20:61867234-61871859	q1	q2	NOTEST	0.0442518	0.20863	2.23714	0	1	1	no

ENSG00000101198	ENSG00000101198	NKAIN4	20:61872135-61904046	q1	q2	OK	26.4847	21.5928	-0.29461	-0.589131	0.3987	0.99995	no

ENSG00000101199	ENSG00000101199	ARFGAP1	20:61904136-61921142	q1	q2	OK	54.4873	48.4849	-0.168384	-0.327338	0.59325	0.99995	no

ENSG00000101200	ENSG00000101200	AVP	20:3063201-3065370	q1	q2	NOTEST	0	0	0	0	1	1	no

ENSG00000101203	ENSG00000101203	COL20A1	20:61924537-61966203	q1	q2	NOTEST	0.235078	0.0717985	-1.71112	0	1	1	no

ENSG00000101204	ENSG00000101204	CHRNA4	20:61975419-62009753	q1	q2	OK	1.16936	0.986314	-0.2456	-0.313184	0.6482	0.99995	no

ENSG00000101210	ENSG00000101210	EEF1A2	20:62119365-62130505	q1	q2	OK	11.352	8.60956	-0.398938	-0.70252	0.31935	0.99995	no

ENSG00000101213	ENSG00000101213	PTK6	20:62159777-62168723	q1	q2	NOTEST	0.171616	0.103134	-0.734657	0	1	1	no

ENSG00000101216	ENSG00000101216	GMEB2	20:62218954-62258394	q1	q2	OK	7.158	7.32898	0.0340554	0.0706631	0.9193	0.99995	no

ENSG00000101220	ENSG00000101220	C20orf27	20:3734154-3749034	q1	q2	OK	20.7665	19.6333	-0.0809515	-0.151874	0.82555	0.99995	no

ENSG00000101222	ENSG00000101222	SPEF1	20:3758150-3762095	q1	q2	OK	0.5862	0.308835	-0.924554	-0.856403	0.21025	0.99995	no

ENSG00000101224	ENSG00000101224	CDC25B	20:3767577-3786762	q1	q2	OK	40.9088	35.8027	-0.192345	-0.387064	0.49555	0.99995	no

ENSG00000101230	ENSG00000101230	ISM1	20:13202417-13619587	q1	q2	NOTEST	0.253292	0.236251	-0.10048	0	1	1	no

ENSG00000101236	ENSG00000101236	RNF24	20:3907955-3996229	q1	q2	OK	20.0982	20.2876	0.0135305	0.0273557	0.9665	0.99995	no

ENSG00000101246	ENSG00000101246	ARFRP1	20:62289162-62374858	q1	q2	OK	14.6691	12.9593	-0.178784	-0.149384	0.8055	0.99995	no

ENSG00000101247	ENSG00000101247	NDUFAF5	20:13765595-13799067	q1	q2	OK	16.9093	19.4004	0.198272	0.285313	0.68345	0.99995	no

ENSG00000101251	ENSG00000101251	SEL1L2	20:13829892-16033842	q1	q2	NOTEST	0.0263165	0	#NAME?	0	1	1	no

ENSG00000101255	ENSG00000101255	TRIB3	20:361260-378203	q1	q2	OK	8.18699	7.45076	-0.135946	-0.236334	0.72475	0.99995	no

ENSG00000101265	ENSG00000101265	RASSF2	20:4760668-4804291	q1	q2	OK	46.7667	51.2185	0.131183	0.322154	0.6502	0.99995	no

ENSG00000101266	ENSG00000101266	CSNK2A1	20:459115-524465	q1	q2	OK	53.19	59.8479	0.170145	0.364976	0.54515	0.99995	no

ENSG00000101276	ENSG00000101276	SLC52A3	20:740723-749131	q1	q2	NOTEST	0	0	0	0	1	1	no

ENSG00000101278	ENSG00000101278	RPS10L	20:814357-838106	q1	q2	OK	1.4715	3.36502	1.19333	0.439061	0.66695	0.99995	no

ENSG00000101280	ENSG00000101280	ANGPT4	20:853295-896977	q1	q2	NOTEST	0.0333291	0.0481677	0.531284	0	1	1	no

ENSG00000101282	ENSG00000101282	RSPO4	20:939094-982907	q1	q2	NOTEST	0.0194442	0.019014	-0.0322808	0	1	1	no

ENSG00000101290	ENSG00000101290	CDS2	20:5107431-5178533	q1	q2	OK	29.6177	27.1047	-0.12792	-0.176403	0.77845	0.99995	no

ENSG00000101292	ENSG00000101292	PROKR2	20:5282316-5297378	q1	q2	NOTEST	0	0	0	0	1	1	no

ENSG00000101294	ENSG00000101294	HM13	20:30102230-30161066	q1	q2	OK	70.5049	69.5966	-0.0187067	-0.0401213	0.9536	0.99995	no

ENSG00000101298	ENSG00000101298	SNPH	20:1206699-1289972	q1	q2	OK	2.4317	2.02187	-0.266279	-0.428895	0.541	0.99995	no

ENSG00000101306	ENSG00000101306	MYLK2	20:30407110-30422492	q1	q2	OK	0.736281	0.826747	0.16719	0.170453	0.8137	0.99995	no

ENSG00000101307	ENSG00000101307	SIRPB1	20:1497404-1600707	q1	q2	OK	0.143744	0.657736	2.19401	0.78109	0.00305	0.99995	no

ENSG00000101310	ENSG00000101310	SEC23B	20:18488136-18542059	q1	q2	OK	20.1749	20.1796	0.000334962	0.000724935	0.999	0.99995	no

ENSG00000101311	ENSG00000101311	FERMT1	20:6055491-6104191	q1	q2	OK	0.599096	0.723339	0.271885	0.242002	0.69595	0.99995	no

ENSG00000101323	ENSG00000101323	HAO1	20:7863627-7921121	q1	q2	NOTEST	0	0	0	0	1	1	no

ENSG00000101327	ENSG00000101327	PDYN	20:1927891-1988163	q1	q2	OK	0.590259	0.988892	0.744464	0.859571	0.22015	0.99995	no

ENSG00000101331	ENSG00000101331	CCM2L	20:30598244-30619984	q1	q2	NOTEST	0.157224	0.159356	0.0194272	0	1	1	no

ENSG00000101333	ENSG00000101333	PLCB4	20:9049409-9461889	q1	q2	OK	5.28381	5.64149	0.0944969	0.0998219	0.8244	0.99995	no

ENSG00000101335	ENSG00000101335	MYL9	20:34894257-35274619	q1	q2	OK	11.4952	8.8551	-0.37645	-0.157929	0.86205	0.99995	no

ENSG00000101336	ENSG00000101336	HCK	20:30639990-30689659	q1	q2	NOTEST	0.0256001	0.0159834	-0.679572	0	1	1	no

ENSG00000101337	ENSG00000101337	TM9SF4	20:30697308-30755061	q1	q2	OK	23.389	22.0281	-0.0864878	-0.186568	0.76915	0.99995	no

ENSG00000101342	ENSG00000101342	TLDC2	20:35504523-35580246	q1	q2	NOTEST	0.407116	0.37707	-0.110609	0	1	1	no

ENSG00000101343	ENSG00000101343	CRNKL1	20:20015011-20341346	q1	q2	OK	18.3512	17.6783	-0.0538894	-0.0848822	0.90155	0.99995	no

ENSG00000101346	ENSG00000101346	POFUT1	20:30795682-30826470	q1	q2	OK	16.9382	16.945	0.000578399	0.00130271	0.9981	0.99995	no

ENSG00000101347	ENSG00000101347	SAMHD1	20:35504523-35580246	q1	q2	OK	5.13021	5.19116	0.0170388	0.0280666	0.96065	0.99995	no

ENSG00000101349	ENSG00000101349	PAK7	20:9518035-9819689	q1	q2	OK	1.66274	1.68936	0.0229114	0.033386	0.961	0.99995	no

ENSG00000101350	ENSG00000101350	KIF3B	20:30865466-30922814	q1	q2	OK	17.0631	17.3334	0.0226822	0.0550817	0.93725	0.99995	no

ENSG00000101353	ENSG00000101353	MROH8	20:35729628-35870022	q1	q2	OK	3.22181	2.65453	-0.279415	-0.0531395	0.74085	0.99995	no

ENSG00000101361	ENSG00000101361	NOP56	20:2632790-2639039	q1	q2	OK	181.696	196.029	0.109544	0.241596	0.7325	0.99995	no

ENSG00000101363	ENSG00000101363	MANBAL	20:35918040-35945663	q1	q2	OK	35.828	33.3167	-0.104841	-0.224852	0.7474	0.99995	no

ENSG00000101365	ENSG00000101365	IDH3B	20:2639040-2644865	q1	q2	OK	51.1151	49.3994	-0.049256	-0.100186	0.87505	0.99995	no

ENSG00000101367	ENSG00000101367	MAPRE1	20:31407698-31442063	q1	q2	OK	104.972	117.394	0.161346	0.395481	0.57105	0.99995	no

ENSG00000101384	ENSG00000101384	JAG1	20:10618331-10975572	q1	q2	OK	24.9608	23.624	-0.0794142	-0.19654	0.77575	0.99995	no

ENSG00000101391	ENSG00000101391	CDK5RAP1	20:31946644-31989367	q1	q2	OK	21.5719	24.157	0.163286	0.27765	0.68785	0.99995	no

ENSG00000101400	ENSG00000101400	SNTA1	20:31995760-32031698	q1	q2	OK	2.74709	2.48336	-0.145611	-0.199581	0.77525	0.99995	no

ENSG00000101405	ENSG00000101405	OXT	20:3052265-3053163	q1	q2	NOTEST	0.0802744	0.0751435	-0.0952919	0	1	1	no

ENSG00000101407	ENSG00000101407	TTI1	20:36611408-36661870	q1	q2	OK	17.1579	16.9215	-0.0200103	-0.0416477	0.94885	0.99995	no

ENSG00000101412	ENSG00000101412	E2F1	20:32263488-32274210	q1	q2	OK	29.3636	27.9663	-0.0703366	-0.165243	0.8065	0.99995	no

ENSG00000101413	ENSG00000101413	RPRD1B	20:36661947-36720768	q1	q2	OK	16.6431	18.0783	0.119339	0.263948	0.6788	0.99995	no

ENSG00000101417	ENSG00000101417	PXMP4	20:32294511-32308125	q1	q2	NOTEST	0.122984	0.0574039	-1.09926	0	1	1	no

ENSG00000101421	ENSG00000101421	CHMP4B	20:32399109-32442172	q1	q2	OK	74.7901	74.9376	0.00284211	0.00689243	0.99055	0.99995	no

ENSG00000101425	ENSG00000101425	BPI	20:36838889-36965907	q1	q2	OK	0.464882	0.956536	1.04096	0.223144	0.55785	0.99995	no

ENSG00000101435	ENSG00000101435	CST9L	20:23545368-23549386	q1	q2	NOTEST	0	0	0	0	1	1	no

ENSG00000101438	ENSG00000101438	SLC32A1	20:37353104-37358015	q1	q2	NOTEST	0.247495	0.273551	0.144411	0	1	1	no

ENSG00000101439	ENSG00000101439	CST3	20:23608533-23619110	q1	q2	OK	80.4061	68.8449	-0.223954	-0.505293	0.4707	0.99995	no

ENSG00000101440	ENSG00000101440	ASIP	20:32782374-32899608	q1	q2	NOTEST	0.126429	0.308998	1.28927	0	1	1	no

ENSG00000101441	ENSG00000101441	CST4	20:23666276-23669677	q1	q2	NOTEST	0	0	0	0	1	1	no

ENSG00000101442	ENSG00000101442	ACTR5	20:37377084-37400834	q1	q2	OK	7.03511	7.18133	0.0296793	0.0517067	0.9438	0.99995	no

ENSG00000101443	ENSG00000101443	WFDC2	20:44095909-44110172	q1	q2	OK	1.1261	1.25972	0.161772	0.129205	0.8285	0.99995	no

ENSG00000101444	ENSG00000101444	AHCY	20:32782374-32899608	q1	q2	OK	75.4569	72.7087	-0.0535242	-0.128587	0.85215	0.99995	no

ENSG00000101445	ENSG00000101445	PPP1R16B	20:37434347-37551667	q1	q2	OK	2.48856	3.01257	0.275685	0.395016	0.49885	0.99995	no

ENSG00000101446	ENSG00000101446	SPINT3	20:44141100-44144264	q1	q2	NOTEST	0	0	0	0	1	1	no

ENSG00000101447	ENSG00000101447	FAM83D	20:37554954-37581703	q1	q2	OK	20.4808	22.0264	0.104963	0.238019	0.7303	0.99995	no

ENSG00000101448	ENSG00000101448	EPPIN	20:44162834-44176391	q1	q2	NOTEST	0.0131047	0.0129344	-0.0188714	0	1	1	no

ENSG00000101452	ENSG00000101452	DHX35	20:37590941-37668366	q1	q2	OK	10.1749	10.9578	0.106941	0.183818	0.7627	0.99995	no

ENSG00000101457	ENSG00000101457	DNTTIP1	20:44420575-44440066	q1	q2	OK	24.9032	24.6882	-0.0125121	-0.024659	0.97245	0.99995	no

ENSG00000101460	ENSG00000101460	MAP1LC3A	20:33134657-33148149	q1	q2	OK	3.03251	2.64629	-0.196537	-0.191074	0.8139	0.99995	no

ENSG00000101463	ENSG00000101463	SYNDIG1	20:24449834-24647252	q1	q2	OK	1.76384	1.27577	-0.467353	-0.547461	0.41775	0.99995	no

ENSG00000101464	ENSG00000101464	PIGU	20:33148345-33264910	q1	q2	OK	20.9501	20.9354	-0.00101604	-0.00207978	0.99745	0.99995	no

ENSG00000101470	ENSG00000101470	TNNC2	20:44451852-44462384	q1	q2	NOTEST	0.14495	0.137156	-0.0797423	0	1	1	no

ENSG00000101473	ENSG00000101473	ACOT8	20:44462448-44486045	q1	q2	OK	8.13347	7.1806	-0.179767	-0.199116	0.765	0.99995	no

ENSG00000101474	ENSG00000101474	APMAP	20:24943560-24973615	q1	q2	OK	65.0366	63.6217	-0.0317328	-0.0770045	0.91255	0.99995	no

ENSG00000101489	ENSG00000101489	CELF4	18:34823009-35146000	q1	q2	OK	3.58476	3.07129	-0.223031	-0.245788	0.60125	0.99995	no

ENSG00000101493	ENSG00000101493	ZNF516	18:74069643-74207146	q1	q2	OK	16.8151	17.5622	0.0627177	0.0987427	0.832	0.99995	no

ENSG00000101542	ENSG00000101542	CDH20	18:59000814-59223006	q1	q2	OK	2.73307	2.41421	-0.178967	-0.229193	0.68755	0.99995	no

ENSG00000101544	ENSG00000101544	ADNP2	18:77866914-77905406	q1	q2	OK	15.5088	18.303	0.238989	0.51585	0.40855	0.99995	no

ENSG00000101546	ENSG00000101546	RBFA	18:77794345-77854968	q1	q2	OK	18.0937	17.2819	-0.0662275	-0.112796	0.87215	0.99995	no

ENSG00000101557	ENSG00000101557	USP14	18:158382-268050	q1	q2	OK	44.3613	47.2205	0.0901131	0.15765	0.81145	0.99995	no

ENSG00000101558	ENSG00000101558	VAPA	18:9913998-9960018	q1	q2	OK	124.367	119.554	-0.0569395	-0.0994163	0.8843	0.99995	no

ENSG00000101574	ENSG00000101574	METTL4	18:2537523-2571508	q1	q2	OK	9.15731	8.93552	-0.0353714	-0.0512711	0.93155	0.99995	no

ENSG00000101577	ENSG00000101577	LPIN2	18:2916991-3013313	q1	q2	OK	11.2256	12.3091	0.132928	0.22497	0.6877	0.99995	no

ENSG00000101596	ENSG00000101596	SMCHD1	18:2655736-2833063	q1	q2	OK	30.1198	32.4356	0.106864	0.14983	0.7336	0.99995	no

ENSG00000101605	ENSG00000101605	MYOM1	18:3066804-3247275	q1	q2	NOTEST	0.222822	0.428904	0.944764	0	1	1	no

ENSG00000101608	ENSG00000101608	MYL12A	18:3247478-3261848	q1	q2	OK	123.921	153.462	0.308469	0.734858	0.2841	0.99995	no

ENSG00000101624	ENSG00000101624	CEP76	18:12438888-12725739	q1	q2	OK	8.23556	8.27351	0.00663329	0.00453081	0.99355	0.99995	no

ENSG00000101638	ENSG00000101638	ST8SIA5	18:44259080-44340307	q1	q2	NOTEST	0.341502	0.212256	-0.68609	0	1	1	no

ENSG00000101639	ENSG00000101639	CEP192	18:12991360-13125051	q1	q2	OK	15.3897	16.2185	0.075676	0.112134	0.84555	0.99995	no

ENSG00000101654	ENSG00000101654	RNMT	18:13663345-13764557	q1	q2	OK	18.698	17.8215	-0.0692689	-0.135901	0.8237	0.99995	no

ENSG00000101665	ENSG00000101665	SMAD7	18:46446222-46477081	q1	q2	OK	2.50942	2.11741	-0.245055	-0.290536	0.617	0.99995	no

ENSG00000101670	ENSG00000101670	LIPG	18:47087068-47119272	q1	q2	OK	15.5279	15.2263	-0.0282939	-0.056767	0.9262	0.99995	no

ENSG00000101680	ENSG00000101680	LAMA1	18:6941742-7117813	q1	q2	OK	41.647	38.8252	-0.101219	-0.13338	0.79995	0.99995	no

ENSG00000101695	ENSG00000101695	RNF125	18:29598334-30050447	q1	q2	NOTEST	0.382825	0.271833	-0.493965	0	1	1	no

ENSG00000101745	ENSG00000101745	ANKRD12	18:9102627-9285983	q1	q2	OK	17.3784	16.5115	-0.0738302	-0.0678672	0.86995	0.99995	no

ENSG00000101746	ENSG00000101746	NOL4	18:31431063-31804916	q1	q2	OK	1.23714	0.767914	-0.687997	-0.475459	0.26635	0.99995	no

ENSG00000101751	ENSG00000101751	POLI	18:51795773-51847636	q1	q2	OK	24.1113	27.9235	0.21177	0.361571	0.5183	0.99995	no

ENSG00000101752	ENSG00000101752	MIB1	18:19284917-19450918	q1	q2	OK	32.5463	36.406	0.161682	0.380448	0.57765	0.99995	no

ENSG00000101773	ENSG00000101773	RBBP8	18:20279443-20606451	q1	q2	OK	23.4923	25.0467	0.0924313	0.175244	0.76845	0.99995	no

ENSG00000101782	ENSG00000101782	RIOK3	18:21032786-21066567	q1	q2	OK	25.9503	25.972	0.00120139	0.00227885	0.9963	0.99995	no

ENSG00000101811	ENSG00000101811	CSTF2	X:100075383-100095921	q1	q2	OK	11.5569	12.6648	0.132072	0.251146	0.7232	0.99995	no

ENSG00000101812	ENSG00000101812	H2BFM	X:103268268-103297021	q1	q2	NOTEST	0	0	0	0	1	1	no

ENSG00000101825	ENSG00000101825	MXRA5	X:3226605-3264682	q1	q2	OK	4.16951	3.72534	-0.162506	-0.327604	0.6396	0.99995	no

ENSG00000101842	ENSG00000101842	VSIG1	X:107288199-107322414	q1	q2	NOTEST	0	0.0933926	inf	0	1	1	no

ENSG00000101843	ENSG00000101843	PSMD10	X:107327436-107334848	q1	q2	OK	41.7116	45.1269	0.113541	0.24896	0.7186	0.99995	no

ENSG00000101844	ENSG00000101844	ATG4A	X:107334897-107682727	q1	q2	OK	3.31752	2.97515	-0.157146	-0.111339	0.8762	0.99995	no

ENSG00000101846	ENSG00000101846	STS	X:7137496-7272851	q1	q2	OK	2.01448	1.85624	-0.118021	-0.199393	0.77395	0.99995	no

ENSG00000101849	ENSG00000101849	TBL1X	X:9431334-9687780	q1	q2	OK	12.6535	13.3508	0.0773857	0.139622	0.7928	0.99995	no

ENSG00000101850	ENSG00000101850	GPR143	X:9693385-9754337	q1	q2	OK	1.24585	1.33269	0.0972048	0.0854688	0.887	0.99995	no

ENSG00000101856	ENSG00000101856	PGRMC1	X:118370215-118378429	q1	q2	OK	173.866	181.257	0.0600601	0.145392	0.8314	0.99995	no

ENSG00000101868	ENSG00000101868	POLA1	X:24712035-25015103	q1	q2	OK	15.2164	15.3568	0.0132422	0.0267399	0.9684	0.99995	no

ENSG00000101871	ENSG00000101871	MID1	X:10413349-10851773	q1	q2	OK	35.4626	30.5276	-0.216185	-0.33625	0.54185	0.99995	no

ENSG00000101882	ENSG00000101882	NKAP	X:119059013-119077735	q1	q2	OK	11.9756	11.3667	-0.0752755	-0.130786	0.84865	0.99995	no

ENSG00000101883	ENSG00000101883	RHOXF1	X:119170200-119379122	q1	q2	NOTEST	0	0	0	0	1	1	no

ENSG00000101888	ENSG00000101888	NXT2	X:108779009-108787919	q1	q2	OK	7.24604	6.74717	-0.102911	-0.163689	0.81715	0.99995	no

ENSG00000101890	ENSG00000101890	GUCY2F	X:108616134-108725301	q1	q2	NOTEST	0.00711768	0.00653701	-0.122776	0	1	1	no

ENSG00000101892	ENSG00000101892	ATP1B4	X:119495966-119516226	q1	q2	NOTEST	0.0129597	0	#NAME?	0	1	1	no

ENSG00000101898	ENSG00000101898	RP3-324O17.4	20:30102230-30161066	q1	q2	OK	5.20023	6.15432	0.243024	0.0891229	0.8346	0.99995	no

ENSG00000101901	ENSG00000101901	ALG13	X:110909042-111003877	q1	q2	OK	17.8669	18.8064	0.0739288	0.0952261	0.85	0.99995	no

ENSG00000101911	ENSG00000101911	PRPS2	X:12809473-12842341	q1	q2	OK	9.02133	9.50281	0.0750152	0.137958	0.83585	0.99995	no

ENSG00000101916	ENSG00000101916	TLR8	X:12920935-12941288	q1	q2	NOTEST	0.00749241	0.00725504	-0.0464467	0	1	1	no

ENSG00000101928	ENSG00000101928	MOSPD1	X:134021655-134049297	q1	q2	OK	9.29441	10.2467	0.140728	0.215072	0.7127	0.99995	no

ENSG00000101935	ENSG00000101935	AMMECR1	X:109437413-109699562	q1	q2	OK	4.54347	5.07255	0.158915	0.237391	0.65295	0.99995	no

ENSG00000101938	ENSG00000101938	CHRDL1	X:109917083-110039286	q1	q2	OK	2.39122	2.44273	0.0307495	0.0380078	0.95635	0.99995	no

ENSG00000101940	ENSG00000101940	WDR13	X:48448429-48463581	q1	q2	NOTEST	0.0679922	0	#NAME?	0	1	1	no

ENSG00000101945	ENSG00000101945	SUV39H1	X:48553944-48595570	q1	q2	OK	1.14006	0.928315	-0.296427	-0.23564	0.71125	0.99995	no

ENSG00000101951	ENSG00000101951	PAGE4	X:49593862-49598576	q1	q2	NOTEST	0	0	0	0	1	1	no

ENSG00000101955	ENSG00000101955	SRPX	X:37208527-38548169	q1	q2	OK	3.04436	2.7504	-0.146499	-0.0862172	0.91975	0.99995	no

ENSG00000101958	ENSG00000101958	GLRA2	X:14547419-14749934	q1	q2	OK	0.728547	0.534501	-0.44683	-0.428152	0.50945	0.99995	no

ENSG00000101966	ENSG00000101966	XIAP	X:122993573-123047829	q1	q2	OK	11.3964	10.8245	-0.0742798	-0.0997206	0.80535	0.99995	no

ENSG00000101972	ENSG00000101972	STAG2	X:123093082-124097666	q1	q2	OK	42.7979	44.9716	0.0714732	0.0840007	0.84295	0.99995	no

ENSG00000101974	ENSG00000101974	ATP11C	X:138808504-139027435	q1	q2	OK	11.7783	14.1934	0.269098	0.472449	0.44795	0.99995	no

ENSG00000101977	ENSG00000101977	MCF2	X:138663928-138790386	q1	q2	NOTEST	0.281258	0.177291	-0.665776	0	1	1	no

ENSG00000101981	ENSG00000101981	F9	X:138612916-138645617	q1	q2	NOTEST	0	0	0	0	1	1	no

ENSG00000101986	ENSG00000101986	ABCD1	X:152990322-153051187	q1	q2	NOTEST	0.388423	0.124393	-1.64272	0	1	1	no

ENSG00000101997	ENSG00000101997	CCDC22	X:49091926-49121288	q1	q2	OK	0.697125	0.82585	0.244462	0.246773	0.68565	0.99995	no

ENSG00000102001	ENSG00000102001	CACNA1F	X:49061522-49089833	q1	q2	NOTEST	0	0	0	0	1	1	no

ENSG00000102003	ENSG00000102003	SYP	X:49044268-49058913	q1	q2	NOTEST	0.271332	0.213615	-0.345048	0	1	1	no

ENSG00000102007	ENSG00000102007	PLP2	X:49028272-49042845	q1	q2	NOTEST	0.267077	0.111421	-1.26123	0	1	1	no

ENSG00000102010	ENSG00000102010	BMX	X:15402920-15574652	q1	q2	NOTEST	0.0516004	0.083655	0.697068	0	1	1	no

ENSG00000102021	ENSG00000102021	LUZP4	X:114524292-114542121	q1	q2	NOTEST	0	0	0	0	1	1	no

ENSG00000102024	ENSG00000102024	PLS3	X:114752489-114885181	q1	q2	OK	4.22641	5.46855	0.371726	0.418784	0.52935	0.99995	no

ENSG00000102030	ENSG00000102030	NAA10	X:153126968-153200676	q1	q2	OK	1.64352	1.38631	-0.245539	-0.149895	0.79425	0.99995	no

ENSG00000102032	ENSG00000102032	RENBP	X:153200715-153210232	q1	q2	OK	0.062633	0.675889	3.43179	0.545211	0.0007	0.45347	no

ENSG00000102034	ENSG00000102034	ELF4	X:129198848-129244691	q1	q2	OK	0.483767	0.420264	-0.203017	-0.179012	0.76655	0.99995	no

ENSG00000102038	ENSG00000102038	SMARCA1	X:128580479-128657477	q1	q2	OK	44.8507	49.1039	0.130709	0.325857	0.6459	0.99995	no

ENSG00000102043	ENSG00000102043	MTMR8	X:63444075-63615333	q1	q2	OK	0.990281	1.57343	0.668001	0.589866	0.3084	0.99995	no

ENSG00000102048	ENSG00000102048	ASB9	X:15253409-15288589	q1	q2	NOTEST	0.212365	0.293361	0.466132	0	1	1	no

ENSG00000102053	ENSG00000102053	ZC3H12B	X:64708614-64727767	q1	q2	OK	1.87463	1.89184	0.0131812	0.0221775	0.9754	0.99995	no

ENSG00000102054	ENSG00000102054	RBBP7	X:16804549-16888537	q1	q2	OK	105.354	106.145	0.0107846	0.0206627	0.9759	0.99995	no

ENSG00000102055	ENSG00000102055	PPP1R2P9	X:42636721-42637330	q1	q2	NOTEST	0	0	0	0	1	1	no

ENSG00000102057	ENSG00000102057	KCND1	X:48818638-48827976	q1	q2	NOTEST	0.0230782	0.0380861	0.722732	0	1	1	no

ENSG00000102069	ENSG00000102069	UBE2NL	X:142967172-142968355	q1	q2	NOTEST	0.0241417	0.1677	2.79628	0	1	1	no

ENSG00000102076	ENSG00000102076	OPN1LW	X:153409697-153424507	q1	q2	NOTEST	0	0	0	0	1	1	no

ENSG00000102078	ENSG00000102078	SLC25A14	X:129473873-129507335	q1	q2	OK	16.9797	17.3805	0.0336614	0.0543745	0.93985	0.99995	no

ENSG00000102080	ENSG00000102080	TEX28P2	X:153424681-153445634	q1	q2	NOTEST	0	0	0	0	1	1	no

ENSG00000102081	ENSG00000102081	FMR1	X:146993468-147032645	q1	q2	NOTEST	0.0412037	0.037801	-0.124348	0	1	1	no

ENSG00000102096	ENSG00000102096	PIM2	X:48770458-48776301	q1	q2	NOTEST	0	0	0	0	1	1	no

ENSG00000102098	ENSG00000102098	SCML2	X:18257433-18372847	q1	q2	OK	13.7553	14.2896	0.054986	0.106883	0.8753	0.99995	no

ENSG00000102100	ENSG00000102100	SLC35A2	X:48760458-48769235	q1	q2	OK	0.111151	2.24297	4.33482	1.88575	0.00025	0.291517	no

ENSG00000102103	ENSG00000102103	PQBP1	X:48750729-48760420	q1	q2	OK	10.5864	2.96438	-1.83641	-1.64828	0.01465	0.99995	no

ENSG00000102104	ENSG00000102104	RS1	X:18443702-18690229	q1	q2	NOTEST	0.0181834	0.0182629	0.00629779	0	1	1	no

ENSG00000102109	ENSG00000102109	PCSK1N	X:48689503-48694035	q1	q2	NOTEST	0.0853183	0	#NAME?	0	1	1	no

ENSG00000102119	ENSG00000102119	EMD	X:153607556-153609883	q1	q2	NOTEST	0.0400888	0	#NAME?	0	1	1	no

ENSG00000102125	ENSG00000102125	TAZ	X:153618314-153650065	q1	q2	OK	1.29896	0.857719	-0.598776	-0.220318	0.46565	0.99995	no

ENSG00000102128	ENSG00000102128	RAB40AL	X:102192199-102195489	q1	q2	NOTEST	0.181365	0.0576182	-1.6543	0	1	1	no

ENSG00000102144	ENSG00000102144	PGK1	X:77320684-77384793	q1	q2	NOTEST	0	0	0	0	1	1	no

ENSG00000102145	ENSG00000102145	GATA1	X:48644961-48652716	q1	q2	NOTEST	0	0.0177876	inf	0	1	1	no

ENSG00000102158	ENSG00000102158	MAGT1	X:77081860-77151090	q1	q2	OK	0.378816	3.04178	3.00535	2.48621	5.00E-05	0.0795045	no

ENSG00000102172	ENSG00000102172	SMS	X:21958690-22025798	q1	q2	OK	118.993	132.434	0.154397	0.367023	0.5994	0.99995	no

ENSG00000102174	ENSG00000102174	PHEX	X:22050558-22269427	q1	q2	NOTEST	0.305054	0.273568	-0.157164	0	1	1	no

ENSG00000102178	ENSG00000102178	UBL4A	X:153712055-153715009	q1	q2	OK	5.77029	5.75536	-0.00373809	-0.00579737	0.99295	0.99995	no

ENSG00000102181	ENSG00000102181	CD99L2	X:149934809-150067289	q1	q2	OK	4.91755	6.22041	0.339069	0.400947	0.5759	0.99995	no

ENSG00000102189	ENSG00000102189	EEA1	12:93115280-93323107	q1	q2	OK	5.42523	5.72692	0.0780748	0.109922	0.8068	0.99995	no

ENSG00000102195	ENSG00000102195	GPR50	X:150343663-150349937	q1	q2	OK	15.0864	11.7884	-0.35588	-0.688991	0.32925	0.99995	no

ENSG00000102218	ENSG00000102218	RP2	X:46696374-46741793	q1	q2	NOTEST	0	0	0	0	1	1	no

ENSG00000102221	ENSG00000102221	JADE3	X:46771710-46920641	q1	q2	NOTEST	0.00528976	0.0259787	2.29606	0	1	1	no

ENSG00000102225	ENSG00000102225	CDK16	X:47077258-47089396	q1	q2	OK	60.7215	49.8123	-0.285704	-0.540687	0.3571	0.99995	no

ENSG00000102226	ENSG00000102226	USP11	X:47092088-47107727	q1	q2	OK	67.8997	66.4574	-0.0309746	-0.055939	0.9134	0.99995	no

ENSG00000102230	ENSG00000102230	PCYT1B	X:24576203-24690794	q1	q2	OK	5.49419	5.17137	-0.0873594	-0.138416	0.8415	0.99995	no

ENSG00000102239	ENSG00000102239	BRS3	X:135570045-135575939	q1	q2	NOTEST	0.0185133	0	#NAME?	0	1	1	no

ENSG00000102241	ENSG00000102241	HTATSF1	X:135579237-135594505	q1	q2	OK	74.3901	75.3808	0.019086	0.0473406	0.9467	0.99995	no

ENSG00000102243	ENSG00000102243	VGLL1	X:135614310-135638966	q1	q2	NOTEST	0	0	0	0	1	1	no

ENSG00000102245	ENSG00000102245	CD40LG	X:135730351-135742549	q1	q2	NOTEST	0	0	0	0	1	1	no

ENSG00000102265	ENSG00000102265	TIMP1	X:47420515-47479252	q1	q2	OK	71.7756	64.1067	-0.163017	-0.227139	0.77675	0.99995	no

ENSG00000102271	ENSG00000102271	KLHL4	X:86772751-86925050	q1	q2	OK	2.87409	3.64248	0.341817	0.514528	0.45685	0.99995	no

ENSG00000102287	ENSG00000102287	GABRE	X:151121595-151143152	q1	q2	OK	1.1409	1.0408	-0.132481	-0.0868959	0.81315	0.99995	no

ENSG00000102290	ENSG00000102290	PCDH11X	X:91034259-91878229	q1	q2	OK	0.550446	0.639094	0.215425	0.149036	0.82725	0.99995	no

ENSG00000102302	ENSG00000102302	FGD1	X:54466833-54522599	q1	q2	OK	10.1169	9.42148	-0.102738	-0.178366	0.7979	0.99995	no

ENSG00000102309	ENSG00000102309	PIN4	X:71401202-71527037	q1	q2	OK	18.9151	18.7749	-0.0107322	-0.00443493	0.99555	0.99995	no

ENSG00000102312	ENSG00000102312	PORCN	X:48367349-48379202	q1	q2	NOTEST	0	0	0	0	1	1	no

ENSG00000102313	ENSG00000102313	ITIH6	X:54775331-54824673	q1	q2	NOTEST	0.00497666	0.0148073	1.57306	0	1	1	no

ENSG00000102316	ENSG00000102316	MAGED2	X:54834031-54842445	q1	q2	OK	241.957	232.049	-0.0603229	-0.119702	0.8321	0.99995	no

ENSG00000102317	ENSG00000102317	RBM3	X:48431481-48437454	q1	q2	NOTEST	0	0	0	0	1	1	no

ENSG00000102349	ENSG00000102349	KLF8	X:56258853-56314322	q1	q2	NOTEST	0.12866	0.16155	0.328414	0	1	1	no

ENSG00000102359	ENSG00000102359	SRPX2	X:99899214-99926296	q1	q2	OK	4.97104	4.52082	-0.136964	-0.183264	0.7874	0.99995	no

ENSG00000102362	ENSG00000102362	SYTL4	X:99929487-99987110	q1	q2	OK	4.46946	4.88469	0.128168	0.151856	0.7973	0.99995	no

ENSG00000102383	ENSG00000102383	ZDHHC15	X:74588261-74743337	q1	q2	OK	3.40936	3.04691	-0.162153	-0.184041	0.7314	0.99995	no

ENSG00000102384	ENSG00000102384	CENPI	X:100353177-100418670	q1	q2	OK	11.9087	14.5663	0.290613	0.495064	0.3834	0.99995	no

ENSG00000102385	ENSG00000102385	DRP2	X:100474757-100519486	q1	q2	OK	2.13567	2.06172	-0.0508425	-0.0861949	0.9054	0.99995	no

ENSG00000102387	ENSG00000102387	TAF7L	X:100523240-100548059	q1	q2	NOTEST	0.251123	0.284136	0.178186	0	1	1	no

ENSG00000102390	ENSG00000102390	PBDC1	X:75392770-75398039	q1	q2	OK	2.14289	2.91731	0.445082	0.429001	0.5351	0.99995	no

ENSG00000102393	ENSG00000102393	GLA	X:100645811-100669121	q1	q2	OK	0.489313	2.19892	2.16796	0.204999	0.22845	0.99995	no

ENSG00000102401	ENSG00000102401	ARMCX3	X:100877786-100882833	q1	q2	OK	4.66496	0.645188	-2.85407	-2.39107	5.00E-05	0.0795045	no

ENSG00000102409	ENSG00000102409	BEX4	X:102470019-102472174	q1	q2	OK	64.8148	63.3876	-0.0321225	-0.0753642	0.91395	0.99995	no

ENSG00000102445	ENSG00000102445	KIAA0226L	13:46916138-47012325	q1	q2	OK	3.21251	4.04169	0.331258	0.412427	0.55385	0.99995	no

ENSG00000102452	ENSG00000102452	NALCN	13:101360578-102068843	q1	q2	OK	0.793279	0.728687	-0.12253	-0.107015	0.8242	0.99995	no

ENSG00000102466	ENSG00000102466	FGF14	13:102104965-103054807	q1	q2	OK	1.08687	1.13446	0.0618242	0.0565161	0.9276	0.99995	no

ENSG00000102468	ENSG00000102468	HTR2A	13:47405684-47471169	q1	q2	NOTEST	0.0378144	0.0437648	0.210833	0	1	1	no

ENSG00000102471	ENSG00000102471	NDFIP2	13:80051498-80130210	q1	q2	OK	14.7847	15.7326	0.0896561	0.16097	0.8146	0.99995	no

ENSG00000102524	ENSG00000102524	TNFSF13B	13:108903587-108960832	q1	q2	OK	0.490016	0.612921	0.322874	0.208653	0.63395	0.99995	no

ENSG00000102531	ENSG00000102531	FNDC3A	13:49550047-49783915	q1	q2	OK	35.1003	38.4836	0.132758	0.321491	0.6361	0.99995	no

ENSG00000102539	ENSG00000102539	MLNR	13:49794473-49796513	q1	q2	NOTEST	0	0.0207027	inf	0	1	1	no

ENSG00000102543	ENSG00000102543	CDADC1	13:49822046-49867618	q1	q2	OK	7.1074	6.38888	-0.153759	-0.162097	0.82015	0.99995	no

ENSG00000102547	ENSG00000102547	CAB39L	13:49882785-50069138	q1	q2	OK	9.2685	9.62781	0.0548719	0.0897391	0.8922	0.99995	no

ENSG00000102554	ENSG00000102554	KLF5	13:73629113-73651676	q1	q2	OK	0.851176	0.711951	-0.25768	-0.187605	0.70045	0.99995	no

ENSG00000102572	ENSG00000102572	STK24	13:99102454-99231084	q1	q2	OK	47.6446	49.956	0.0683453	0.159267	0.81325	0.99995	no

ENSG00000102575	ENSG00000102575	ACP5	19:11670188-11729950	q1	q2	NOTEST	0.100651	0.198915	0.982794	0	1	1	no

ENSG00000102580	ENSG00000102580	DNAJC3	13:96329392-96447243	q1	q2	OK	13.4659	12.4	-0.118971	-0.271451	0.69545	0.99995	no

ENSG00000102595	ENSG00000102595	UGGT2	13:96453833-96705736	q1	q2	OK	22.8887	24.1643	0.0782411	0.0863907	0.88285	0.99995	no

ENSG00000102606	ENSG00000102606	ARHGEF7	13:111766158-111958084	q1	q2	OK	40.9491	40.5698	-0.0134258	-0.0210998	0.9638	0.99995	no

ENSG00000102678	ENSG00000102678	FGF9	13:22245521-22278637	q1	q2	OK	6.67121	5.62614	-0.245804	-0.222021	0.72105	0.99995	no

ENSG00000102683	ENSG00000102683	SGCG	13:23755090-23899304	q1	q2	OK	0.563651	1.09043	0.952029	0.968772	0.15205	0.99995	no

ENSG00000102699	ENSG00000102699	PARP4	13:24982294-25171814	q1	q2	OK	13.711	14.0853	0.0388558	0.0794271	0.89155	0.99995	no

ENSG00000102710	ENSG00000102710	SUPT20H	13:37523911-37633850	q1	q2	OK	50.3408	48.7315	-0.0468738	-0.0618842	0.8989	0.99995	no

ENSG00000102738	ENSG00000102738	MRPS31	13:41303431-41345309	q1	q2	OK	23.8297	26.2915	0.14183	0.257788	0.7024	0.99995	no

ENSG00000102743	ENSG00000102743	SLC25A15	13:41363547-41384247	q1	q2	OK	6.5115	7.15094	0.135142	0.210211	0.7553	0.99995	no

ENSG00000102753	ENSG00000102753	KPNA3	13:50273446-50367057	q1	q2	OK	33.836	37.5187	0.149049	0.363583	0.5984	0.99995	no

ENSG00000102755	ENSG00000102755	FLT1	13:28874488-29069265	q1	q2	NOTEST	0.176131	0.197894	0.168077	0	1	1	no

ENSG00000102760	ENSG00000102760	RGCC	13:42031694-42045018	q1	q2	OK	11.9921	10.5859	-0.179944	-0.248048	0.7112	0.99995	no

ENSG00000102763	ENSG00000102763	VWA8	13:42140972-42535256	q1	q2	OK	3.71373	4.14541	0.158648	0.257455	0.6477	0.99995	no

ENSG00000102780	ENSG00000102780	DGKH	13:42614175-42830714	q1	q2	OK	2.65731	3.25323	0.291907	0.600963	0.3352	0.99995	no

ENSG00000102781	ENSG00000102781	KATNAL1	13:30776766-30881621	q1	q2	OK	9.86818	10.8102	0.131534	0.173397	0.71315	0.99995	no

ENSG00000102786	ENSG00000102786	INTS6	13:51909908-52126500	q1	q2	OK	17.8127	18.5403	0.0577606	0.100466	0.8574	0.99995	no

ENSG00000102794	ENSG00000102794	IRG1	13:77522631-77532777	q1	q2	NOTEST	0	0	0	0	1	1	no

ENSG00000102796	ENSG00000102796	DHRS12	13:52342128-52378293	q1	q2	OK	2.65263	2.52	-0.0739998	-0.0721051	0.90425	0.99995	no

ENSG00000102802	ENSG00000102802	MEDAG	13:31456069-31551760	q1	q2	NOTEST	0	0.0105828	inf	0	1	1	no

ENSG00000102804	ENSG00000102804	TSC22D1	13:45007654-45151283	q1	q2	OK	114.184	122.208	0.0979769	0.203125	0.75175	0.99995	no

ENSG00000102805	ENSG00000102805	CLN5	13:77564794-77601330	q1	q2	OK	8.46441	8.84208	0.0629768	0.0593536	0.91745	0.99995	no

ENSG00000102837	ENSG00000102837	OLFM4	13:53602893-53626192	q1	q2	NOTEST	0.0537444	0.0609178	0.180748	0	1	1	no

ENSG00000102854	ENSG00000102854	MSLN	16:810761-818865	q1	q2	NOTEST	0.0288641	0.0533371	0.885862	0	1	1	no

ENSG00000102858	ENSG00000102858	MGRN1	16:4666493-4740975	q1	q2	OK	24.3384	22.8122	-0.0934296	-0.176863	0.76375	0.99995	no

ENSG00000102870	ENSG00000102870	ZNF629	16:30789769-30798523	q1	q2	OK	14.1639	14.3577	0.0196077	0.0395819	0.9554	0.99995	no

ENSG00000102871	ENSG00000102871	TRADD	16:67188082-67203848	q1	q2	OK	0.618153	0.535239	-0.20778	-0.120533	0.85155	0.99995	no

ENSG00000102878	ENSG00000102878	HSF4	16:67188082-67203848	q1	q2	OK	2.53829	1.26896	-1.00021	-0.552092	0.1553	0.99995	no

ENSG00000102879	ENSG00000102879	CORO1A	16:30194147-30200397	q1	q2	OK	10.5397	8.20583	-0.361117	-0.570916	0.3577	0.99995	no

ENSG00000102882	ENSG00000102882	MAPK3	16:30125425-30134827	q1	q2	OK	17.7865	17.0888	-0.057737	-0.100585	0.87795	0.99995	no

ENSG00000102886	ENSG00000102886	GDPD3	16:30116130-30125177	q1	q2	OK	1.31679	1.26661	-0.0560534	-0.0371049	0.93675	0.99995	no

ENSG00000102890	ENSG00000102890	ELMO3	16:67233013-67237932	q1	q2	OK	1.41778	1.18676	-0.256601	-0.294095	0.6513	0.99995	no

ENSG00000102891	ENSG00000102891	MT4	16:56598960-56602869	q1	q2	NOTEST	0	0	0	0	1	1	no

ENSG00000102893	ENSG00000102893	PHKB	16:47178233-47735434	q1	q2	OK	35.2625	34.1219	-0.0474363	-0.0644564	0.9033	0.99995	no

ENSG00000102897	ENSG00000102897	LYRM1	16:20621564-20936328	q1	q2	OK	23.4967	23.7807	0.0173321	0.013717	0.9789	0.99995	no

ENSG00000102898	ENSG00000102898	NUTF2	16:67840667-67906470	q1	q2	OK	106.855	104.947	-0.0259836	-0.0403424	0.95385	0.99995	no

ENSG00000102900	ENSG00000102900	NUP93	16:56764016-56878797	q1	q2	OK	43.1498	42.9699	-0.00603068	-0.0114157	0.9821	0.99995	no

ENSG00000102901	ENSG00000102901	CENPT	16:67840667-67906470	q1	q2	OK	28.3224	27.5796	-0.0383436	-0.0404083	0.94085	0.99995	no

ENSG00000102904	ENSG00000102904	TSNAXIP1	16:67840667-67906470	q1	q2	OK	4.54853	3.28428	-0.469825	-0.163825	0.62095	0.99995	no

ENSG00000102908	ENSG00000102908	NFAT5	16:69598996-69738569	q1	q2	OK	8.75809	9.37317	0.0979204	0.138421	0.75155	0.99995	no

ENSG00000102910	ENSG00000102910	LONP2	16:48198862-48482313	q1	q2	OK	14.7863	15.1516	0.0352089	0.0402325	0.94325	0.99995	no

ENSG00000102921	ENSG00000102921	N4BP1	16:48572636-48654059	q1	q2	OK	17.2167	18.2317	0.0826339	0.0955636	0.8186	0.99995	no

ENSG00000102924	ENSG00000102924	CBLN1	16:49311827-49315742	q1	q2	OK	2.65599	2.3173	-0.196804	-0.259139	0.6906	0.99995	no

ENSG00000102931	ENSG00000102931	ARL2BP	16:57279009-57287516	q1	q2	OK	60.696	67.9179	0.162189	0.391931	0.57285	0.99995	no

ENSG00000102934	ENSG00000102934	PLLP	16:57290003-57318599	q1	q2	OK	2.7054	3.04625	0.171192	0.201542	0.7591	0.99995	no

ENSG00000102935	ENSG00000102935	ZNF423	16:49521434-49891830	q1	q2	OK	19.0482	18.1632	-0.0686337	-0.123244	0.82095	0.99995	no

ENSG00000102962	ENSG00000102962	CCL22	16:57392683-57400102	q1	q2	NOTEST	0.0357236	0.0265081	-0.430445	0	1	1	no

ENSG00000102967	ENSG00000102967	DHODH	16:72042486-72058954	q1	q2	OK	5.71834	5.59523	-0.0313973	-0.0449359	0.9398	0.99995	no

ENSG00000102970	ENSG00000102970	CCL17	16:57438678-57449974	q1	q2	NOTEST	0	0	0	0	1	1	no

ENSG00000102974	ENSG00000102974	CTCF	16:67583116-67673086	q1	q2	OK	40.6881	41.397	0.0249214	0.061694	0.9281	0.99995	no

ENSG00000102977	ENSG00000102977	ACD	16:67678821-67694713	q1	q2	OK	18.5958	16.6211	-0.161966	-0.258268	0.69825	0.99995	no

ENSG00000102978	ENSG00000102978	POLR2C	16:57462080-57521239	q1	q2	OK	44.587	44.0641	-0.0170187	-0.0218711	0.9692	0.99995	no

ENSG00000102981	ENSG00000102981	PARD6A	16:67694848-67696681	q1	q2	OK	5.57518	4.49546	-0.31055	-0.433735	0.547	0.99995	no

ENSG00000102984	ENSG00000102984	ZNF821	16:71879893-71962913	q1	q2	OK	21.9777	21.6456	-0.0219677	-0.0152418	0.98125	0.99995	no

ENSG00000102996	ENSG00000102996	MMP15	16:58059469-58080805	q1	q2	OK	25.6029	20.0356	-0.353743	-0.646652	0.22675	0.99995	no

ENSG00000103005	ENSG00000103005	USB1	16:58028571-58055522	q1	q2	OK	35.6259	34.5728	-0.0432896	-0.0816527	0.8922	0.99995	no

ENSG00000103018	ENSG00000103018	CYB5B	16:69458427-69500169	q1	q2	OK	115.402	128.736	0.157749	0.279441	0.6741	0.99995	no

ENSG00000103021	ENSG00000103021	CCDC113	16:58265060-58328951	q1	q2	OK	1.75078	1.5584	-0.167933	-0.245029	0.7038	0.99995	no

ENSG00000103023	ENSG00000103023	PRSS54	16:58265060-58328951	q1	q2	NOTEST	0	0	0	0	1	1	no

ENSG00000103024	ENSG00000103024	NME3	16:1756183-1821731	q1	q2	OK	6.32155	5.23322	-0.272579	-0.105175	0.7643	0.99995	no

ENSG00000103034	ENSG00000103034	NDRG4	16:58496749-58547532	q1	q2	OK	36.3592	33.2791	-0.127706	-0.218622	0.63535	0.99995	no

ENSG00000103035	ENSG00000103035	PSMD7	16:74330672-74403724	q1	q2	OK	92.5297	91.1057	-0.0223759	-0.0517489	0.9368	0.99995	no

ENSG00000103037	ENSG00000103037	SETD6	16:58549382-58663790	q1	q2	OK	3.19185	3.22091	0.0130774	0.00281043	0.98795	0.99995	no

ENSG00000103042	ENSG00000103042	SLC38A7	16:58699012-58719008	q1	q2	OK	11.6308	10.5588	-0.139504	-0.190589	0.7154	0.99995	no

ENSG00000103043	ENSG00000103043	VAC14	16:70721341-70835064	q1	q2	OK	16.8165	16.7493	-0.00577641	-0.0110918	0.98525	0.99995	no

ENSG00000103044	ENSG00000103044	HAS3	16:69139466-69442474	q1	q2	OK	2.39463	2.27425	-0.0744129	-0.0170366	0.96895	0.99995	no

ENSG00000103047	ENSG00000103047	TANGO6	16:68877506-69119083	q1	q2	OK	5.55234	5.40276	-0.0393993	-0.045547	0.92015	0.99995	no

ENSG00000103051	ENSG00000103051	COG4	16:70514470-70557468	q1	q2	OK	35.8292	32.437	-0.143495	-0.262383	0.669	0.99995	no

ENSG00000103056	ENSG00000103056	SMPD3	16:68344876-68482591	q1	q2	OK	2.93064	2.0772	-0.496576	-0.3212	0.3543	0.99995	no

ENSG00000103061	ENSG00000103061	SLC7A6OS	16:68298432-68344849	q1	q2	OK	12.976	13.1739	0.0218352	0.0209336	0.9625	0.99995	no

ENSG00000103064	ENSG00000103064	SLC7A6	16:68298432-68344849	q1	q2	OK	14.2451	15.6139	0.132361	0.127823	0.7223	0.99995	no

ENSG00000103066	ENSG00000103066	PLA2G15	16:68279206-68294961	q1	q2	OK	8.17131	7.14584	-0.193464	-0.312542	0.6067	0.99995	no

ENSG00000103067	ENSG00000103067	ESRP2	16:68021296-68272005	q1	q2	NOTEST	0.317201	0.475461	0.583927	0	1	1	no

ENSG00000103089	ENSG00000103089	FA2H	16:74746852-74808729	q1	q2	NOTEST	0.0260009	0.0618981	1.25134	0	1	1	no

ENSG00000103091	ENSG00000103091	WDR59	16:74907467-75144892	q1	q2	OK	20.8775	21.1221	0.0168003	0.0161887	0.9659	0.99995	no

ENSG00000103111	ENSG00000103111	MON1B	16:77224731-77478233	q1	q2	OK	17.0017	16.6972	-0.0260713	-0.041295	0.9459	0.99995	no

ENSG00000103121	ENSG00000103121	CMC2	16:81009697-81069364	q1	q2	OK	62.0722	60.4719	-0.0376812	-0.0484111	0.94385	0.99995	no

ENSG00000103126	ENSG00000103126	AXIN1	16:337439-402673	q1	q2	OK	10.8095	10.3756	-0.0591144	-0.122442	0.8583	0.99995	no

ENSG00000103145	ENSG00000103145	HCFC1R1	16:3072620-3089134	q1	q2	OK	17.0167	15.1483	-0.1678	-0.184274	0.7943	0.99995	no

ENSG00000103148	ENSG00000103148	NPRL3	16:127005-188859	q1	q2	OK	22.1415	18.5551	-0.254937	-0.407606	0.47915	0.99995	no

ENSG00000103150	ENSG00000103150	MLYCD	16:83932730-83999937	q1	q2	OK	4.781	3.64594	-0.391024	-0.588778	0.40255	0.99995	no

ENSG00000103152	ENSG00000103152	MPG	16:127005-188859	q1	q2	OK	14.6797	14.5243	-0.0153497	-0.0166693	0.98195	0.99995	no

ENSG00000103154	ENSG00000103154	NECAB2	16:84002236-84036381	q1	q2	OK	6.06483	4.2867	-0.500598	-0.541953	0.40885	0.99995	no

ENSG00000103160	ENSG00000103160	HSDL1	16:84155885-84178797	q1	q2	OK	40.1968	39.7682	-0.0154672	-0.0286754	0.9591	0.99995	no

ENSG00000103168	ENSG00000103168	TAF1C	16:84178864-84220669	q1	q2	OK	26.2216	23.3345	-0.168292	-0.22071	0.6118	0.99995	no

ENSG00000103174	ENSG00000103174	NAGPA	16:4932507-5147809	q1	q2	OK	4.98252	3.94967	-0.335141	-0.226475	0.5505	0.99995	no

ENSG00000103175	ENSG00000103175	WFDC1	16:84328251-84363450	q1	q2	OK	0.646263	0.421633	-0.616134	-0.574533	0.42145	0.99995	no

ENSG00000103184	ENSG00000103184	SEC14L5	16:4932507-5147809	q1	q2	NOTEST	0.0633121	0.0459481	-0.462476	0	1	1	no

ENSG00000103187	ENSG00000103187	COTL1	16:84599199-84651683	q1	q2	OK	426.413	432.481	0.0203865	0.0433811	0.94925	0.99995	no

ENSG00000103194	ENSG00000103194	USP10	16:84733583-84813528	q1	q2	OK	53.3746	58.2821	0.126898	0.254644	0.63995	0.99995	no

ENSG00000103196	ENSG00000103196	CRISPLD2	16:84853589-84954374	q1	q2	NOTEST	0.282465	0.408951	0.533859	0	1	1	no

ENSG00000103197	ENSG00000103197	TSC2	16:2089815-2185899	q1	q2	OK	38.4678	30.5201	-0.33389	-0.357404	0.40065	0.99995	no

ENSG00000103199	ENSG00000103199	ZNF500	16:4746512-4817625	q1	q2	OK	5.9759	5.04797	-0.243454	-0.251782	0.64145	0.99995	no

ENSG00000103200	ENSG00000103200	RP11-723C11.2	7:140135115-140135587	q1	q2	NOTEST	0	0	0	0	1	1	no

ENSG00000103202	ENSG00000103202	NME4	16:446724-462487	q1	q2	OK	117.798	106.8	-0.141397	-0.320582	0.6545	0.99995	no

ENSG00000103222	ENSG00000103222	ABCC1	16:16043433-16236931	q1	q2	OK	7.96379	7.42965	-0.100162	-0.185129	0.75415	0.99995	no

ENSG00000103226	ENSG00000103226	NOMO3	16:16326351-16388668	q1	q2	OK	7.03914	5.88198	-0.259097	-0.21745	0.7123	0.99995	no

ENSG00000103227	ENSG00000103227	LMF1	16:903633-1031663	q1	q2	OK	5.88067	7.55847	0.362113	0.32029	0.5785	0.99995	no

ENSG00000103241	ENSG00000103241	FOXF1	16:86544132-86548076	q1	q2	NOTEST	0.030607	0.0104188	-1.55467	0	1	1	no

ENSG00000103245	ENSG00000103245	NARFL	16:770580-791329	q1	q2	OK	17.2116	14.6511	-0.232375	-0.296572	0.596	0.99995	no

ENSG00000103248	ENSG00000103248	MTHFSD	16:86563781-86588841	q1	q2	OK	12.4535	11.9367	-0.0611476	-0.0784415	0.9088	0.99995	no

ENSG00000103249	ENSG00000103249	CLCN7	16:1494934-1525581	q1	q2	OK	25.2122	20.8306	-0.275419	-0.390285	0.5144	0.99995	no

ENSG00000103253	ENSG00000103253	HAGHL	16:770580-791329	q1	q2	OK	4.27927	4.65824	0.122421	0.072165	0.8529	0.99995	no

ENSG00000103254	ENSG00000103254	FAM173A	16:770580-791329	q1	q2	OK	13.4186	11.8407	-0.180475	-0.154131	0.81305	0.99995	no

ENSG00000103257	ENSG00000103257	SLC7A5	16:87863628-87903094	q1	q2	OK	21.3659	19.5918	-0.125055	-0.293782	0.6698	0.99995	no

ENSG00000103260	ENSG00000103260	METRN	16:765114-769655	q1	q2	OK	53.8315	47.1404	-0.191485	-0.334495	0.64395	0.99995	no

ENSG00000103264	ENSG00000103264	FBXO31	16:87117167-87438385	q1	q2	OK	6.13436	6.60431	0.106494	0.0722745	0.89025	0.99995	no

ENSG00000103266	ENSG00000103266	STUB1	16:730223-734529	q1	q2	OK	41.8488	37.5541	-0.156217	-0.248099	0.7094	0.99995	no

ENSG00000103269	ENSG00000103269	RHBDL1	16:725665-728268	q1	q2	OK	1.57231	1.29595	-0.278878	-0.302816	0.66735	0.99995	no

ENSG00000103274	ENSG00000103274	NUBP1	16:10837642-10912651	q1	q2	OK	19.9335	19.8706	-0.00456173	-0.00827135	0.99075	0.99995	no

ENSG00000103275	ENSG00000103275	UBE2I	16:1355547-1377019	q1	q2	OK	142.654	155.973	0.128776	0.272533	0.6829	0.99995	no

ENSG00000103310	ENSG00000103310	ZP2	16:21206167-21225831	q1	q2	NOTEST	0	0	0	0	1	1	no

ENSG00000103313	ENSG00000103313	MEFV	16:3292027-3306627	q1	q2	NOTEST	0.0152999	0.0148579	-0.0422864	0	1	1	no

ENSG00000103316	ENSG00000103316	CRYM	16:21244985-21329912	q1	q2	OK	18.8189	19.9942	0.0874011	0.148498	0.82175	0.99995	no

ENSG00000103319	ENSG00000103319	EEF2K	16:22217602-22300059	q1	q2	OK	5.40916	5.63853	0.0599151	0.0554384	0.9074	0.99995	no

ENSG00000103326	ENSG00000103326	CAPN15	16:577716-604636	q1	q2	OK	14.4748	12.3546	-0.228495	-0.430665	0.50535	0.99995	no

ENSG00000103335	ENSG00000103335	PIEZO1	16:88772870-88851619	q1	q2	OK	8.37345	8.08146	-0.0512059	-0.0449419	0.9311	0.99995	no

ENSG00000103342	ENSG00000103342	GSPT1	16:11945505-12009939	q1	q2	OK	60.8853	68.3937	0.16777	0.330035	0.61705	0.99995	no

ENSG00000103343	ENSG00000103343	ZNF174	16:3415098-3627401	q1	q2	OK	10.477	8.52358	-0.297696	-0.221458	0.76605	0.99995	no

ENSG00000103351	ENSG00000103351	CLUAP1	16:3415098-3627401	q1	q2	OK	18.0061	17.9027	-0.00831032	-0.00825922	0.98965	0.99995	no

ENSG00000103353	ENSG00000103353	UBFD1	16:23533334-23585710	q1	q2	OK	33.3541	33.7652	0.0176704	0.0338482	0.9557	0.99995	no

ENSG00000103355	ENSG00000103355	PRSS33	16:2833953-2837949	q1	q2	NOTEST	0.0651632	0.0156003	-2.06249	0	1	1	no

ENSG00000103356	ENSG00000103356	EARS2	16:23533334-23585710	q1	q2	OK	11.0672	10.18	-0.120561	-0.0802779	0.84825	0.99995	no

ENSG00000103363	ENSG00000103363	TCEB2	16:2787076-2827298	q1	q2	OK	160.217	155.824	-0.0401148	-0.0178588	0.973	0.99995	no

ENSG00000103365	ENSG00000103365	GGA2	16:23474862-23533316	q1	q2	OK	30.4813	32.9442	0.112101	0.152248	0.7825	0.99995	no

ENSG00000103375	ENSG00000103375	AQP8	16:25227051-25240261	q1	q2	NOTEST	0.0220519	0	#NAME?	0	1	1	no

ENSG00000103381	ENSG00000103381	CPPED1	16:12756918-12897874	q1	q2	OK	3.1218	3.47997	0.156696	0.217709	0.7404	0.99995	no

ENSG00000103404	ENSG00000103404	USP31	16:23072726-23160591	q1	q2	OK	3.45824	4.21315	0.284861	0.3627	0.44225	0.99995	no

ENSG00000103415	ENSG00000103415	HMOX2	16:4511680-4560348	q1	q2	OK	15.8506	13.9915	-0.179987	-0.251179	0.71445	0.99995	no

ENSG00000103423	ENSG00000103423	DNAJA3	16:4475805-4506776	q1	q2	OK	20.358	20.6871	0.0231356	0.0369235	0.9438	0.99995	no

ENSG00000103426	ENSG00000103426	CORO7-PAM16	16:4364761-4475706	q1	q2	NOTEST	0.138912	0	#NAME?	0	1	1	no

ENSG00000103429	ENSG00000103429	BFAR	16:14726671-14763093	q1	q2	OK	31.5932	32.9157	0.0591627	0.111896	0.8486	0.99995	no

ENSG00000103449	ENSG00000103449	SALL1	16:51169885-51185278	q1	q2	OK	61.5975	53.9079	-0.192374	-0.280142	0.53525	0.99995	no

ENSG00000103460	ENSG00000103460	TOX3	16:52471916-52581714	q1	q2	OK	29.7979	28.9998	-0.0391698	-0.0931153	0.8966	0.99995	no

ENSG00000103472	ENSG00000103472	RRN3P2	16:29065052-29231352	q1	q2	OK	0.541772	0.414831	-0.385161	-0.207073	0.7351	0.99995	no

ENSG00000103479	ENSG00000103479	RBL2	16:53467888-53538323	q1	q2	OK	32.7063	30.8136	-0.0860003	-0.114169	0.84755	0.99995	no

ENSG00000103485	ENSG00000103485	QPRT	16:29674299-29710020	q1	q2	OK	38.4529	34.8296	-0.14278	-0.315209	0.6336	0.99995	no

ENSG00000103489	ENSG00000103489	XYLT1	16:17195625-17564738	q1	q2	OK	4.37039	4.49433	0.040345	0.0820893	0.9021	0.99995	no

ENSG00000103490	ENSG00000103490	PYCARD	16:31212805-31214773	q1	q2	OK	1.36112	0.743604	-0.872193	-0.615638	0.32865	0.99995	no

ENSG00000103494	ENSG00000103494	RPGRIP1L	16:53631594-53737850	q1	q2	OK	8.0767	8.20099	0.0220335	0.0306745	0.95625	0.99995	no

ENSG00000103495	ENSG00000103495	MAZ	16:29817426-29859355	q1	q2	OK	190.67	174.201	-0.130329	-0.19359	0.7726	0.99995	no

ENSG00000103496	ENSG00000103496	STX4	16:31044209-31054296	q1	q2	OK	15.4504	14.3971	-0.10186	-0.170606	0.8035	0.99995	no

ENSG00000103502	ENSG00000103502	CDIPT	16:29869677-29879371	q1	q2	OK	21.3075	16.9713	-0.328263	-0.640331	0.37535	0.99995	no

ENSG00000103507	ENSG00000103507	BCKDK	16:31117427-31124110	q1	q2	OK	20.0774	18.0264	-0.15546	-0.294842	0.63295	0.99995	no

ENSG00000103510	ENSG00000103510	KAT8	16:31127074-31142714	q1	q2	OK	43.5839	41.7424	-0.0622795	-0.133349	0.84515	0.99995	no

ENSG00000103512	ENSG00000103512	NOMO1	16:14927537-14990017	q1	q2	OK	20.6174	19.1483	-0.106648	-0.165051	0.7862	0.99995	no

ENSG00000103522	ENSG00000103522	IL21R	16:27413482-27464714	q1	q2	NOTEST	0.347354	0.279928	-0.31135	0	1	1	no

ENSG00000103528	ENSG00000103528	SYT17	16:19125253-19279652	q1	q2	OK	3.24443	2.28232	-0.507463	-0.343326	0.4938	0.99995	no

ENSG00000103534	ENSG00000103534	TMC5	16:19421817-19510435	q1	q2	NOTEST	0.091046	0.102268	0.167685	0	1	1	no

ENSG00000103540	ENSG00000103540	CCP110	16:19535132-19564730	q1	q2	OK	22.0436	23.4244	0.0876516	0.162884	0.7742	0.99995	no

ENSG00000103544	ENSG00000103544	C16orf62	16:19566561-19897489	q1	q2	OK	20.3733	20.732	0.0251761	0.0218586	0.96105	0.99995	no

ENSG00000103546	ENSG00000103546	SLC6A2	16:55689515-55740104	q1	q2	NOTEST	0.00941166	0.00775799	-0.278765	0	1	1	no

ENSG00000103549	ENSG00000103549	RNF40	16:30709029-30787628	q1	q2	OK	59.1509	52.0612	-0.184192	-0.175002	0.73305	0.99995	no

ENSG00000103550	ENSG00000103550	KNOP1	16:19566561-19897489	q1	q2	OK	42.0855	47.2601	0.167298	0.16852	0.7912	0.99995	no

ENSG00000103569	ENSG00000103569	AQP9	15:58245621-58861151	q1	q2	NOTEST	0	0.00947942	inf	0	1	1	no

ENSG00000103591	ENSG00000103591	AAGAB	15:67493370-67819628	q1	q2	OK	42.5761	44.3081	0.0575252	0.125301	0.85155	0.99995	no

ENSG00000103599	ENSG00000103599	IQCH	15:67493370-67819628	q1	q2	OK	1.36972	1.51424	0.144718	0.0477884	0.86095	0.99995	no

ENSG00000103642	ENSG00000103642	LACTB	15:63413998-63450220	q1	q2	OK	5.94767	6.30368	0.0838717	0.0553486	0.93465	0.99995	no

ENSG00000103647	ENSG00000103647	CORO2B	15:68871307-69020145	q1	q2	OK	36.7474	35.6768	-0.0426567	-0.101839	0.8826	0.99995	no

ENSG00000103653	ENSG00000103653	CSK	15:75074397-75095539	q1	q2	OK	38.2709	35.4444	-0.110693	-0.216434	0.70125	0.99995	no

ENSG00000103657	ENSG00000103657	HERC1	15:63900816-64126141	q1	q2	OK	27.3244	25.0945	-0.12282	-0.0922804	0.8403	0.99995	no

ENSG00000103671	ENSG00000103671	TRIP4	15:64679946-64747502	q1	q2	OK	19.7413	21.8195	0.144405	0.275185	0.64135	0.99995	no

ENSG00000103707	ENSG00000103707	MTFMT	15:65294844-65321977	q1	q2	OK	6.86365	7.6763	0.161435	0.257714	0.7017	0.99995	no

ENSG00000103710	ENSG00000103710	RASL12	15:65337707-65369028	q1	q2	OK	1.04971	1.29601	0.304087	0.320413	0.634	0.99995	no

ENSG00000103723	ENSG00000103723	AP3B2	15:83205503-83378666	q1	q2	OK	14.3311	10.9026	-0.394484	-0.292084	0.5568	0.99995	no

ENSG00000103740	ENSG00000103740	ACSBG1	15:78370149-78538030	q1	q2	OK	3.33526	2.46617	-0.435525	-0.238424	0.5095	0.99995	no

ENSG00000103742	ENSG00000103742	IGDCC4	15:65673801-65715410	q1	q2	OK	16.617	16.9923	0.0322245	0.0617527	0.9155	0.99995	no

ENSG00000103769	ENSG00000103769	RAB11A	15:65903703-66184329	q1	q2	OK	176.32	182.665	0.0510112	0.0765756	0.9112	0.99995	no

ENSG00000103811	ENSG00000103811	CTSH	15:79213399-79241916	q1	q2	OK	1.31127	0.988369	-0.407846	-0.298497	0.47735	0.99995	no

ENSG00000103832	ENSG00000103832	GOLGA8UP	15:31083775-31094062	q1	q2	NOTEST	0.0643853	0.0306735	-1.06974	0	1	1	no

ENSG00000103852	ENSG00000103852	TTC23	15:99676527-99791428	q1	q2	OK	8.95904	10.1206	0.175885	0.218649	0.7167	0.99995	no

ENSG00000103855	ENSG00000103855	CD276	15:73976306-74006859	q1	q2	OK	115.051	113.682	-0.0172732	-0.037313	0.9559	0.99995	no

ENSG00000103876	ENSG00000103876	FAH	15:80444831-80479288	q1	q2	OK	5.2404	4.16479	-0.331431	-0.319228	0.5383	0.99995	no

ENSG00000103888	ENSG00000103888	KIAA1199	15:81071683-81282219	q1	q2	OK	1.27966	1.94415	0.603375	0.158033	0.5842	0.99995	no

ENSG00000103932	ENSG00000103932	RPAP1	15:41809373-41836467	q1	q2	OK	15.012	15.0736	0.00590422	0.0085847	0.98635	0.99995	no

ENSG00000103942	ENSG00000103942	HOMER2	15:83509837-83772195	q1	q2	OK	5.86585	6.22796	0.0864186	0.0382309	0.92385	0.99995	no

ENSG00000103966	ENSG00000103966	EHD4	15:42190949-42264776	q1	q2	OK	8.26452	10.2003	0.303609	0.628358	0.36445	0.99995	no

ENSG00000103978	ENSG00000103978	TMEM87A	15:42500610-42704516	q1	q2	OK	18.0563	17.7252	-0.0267022	-0.0430163	0.94175	0.99995	no

ENSG00000103994	ENSG00000103994	ZNF106	15:42705020-42783321	q1	q2	OK	16.4686	18.6067	0.176104	0.296842	0.55205	0.99995	no

ENSG00000103995	ENSG00000103995	CEP152	15:49005124-49103785	q1	q2	OK	6.02006	7.00678	0.218974	0.335287	0.51835	0.99995	no

ENSG00000104043	ENSG00000104043	ATP8B4	15:50150434-50528592	q1	q2	NOTEST	0.0471699	0.0263272	-0.841314	0	1	1	no

ENSG00000104044	ENSG00000104044	OCA2	15:28000020-28344504	q1	q2	NOTEST	0.0426524	0.0338236	-0.334597	0	1	1	no

ENSG00000104047	ENSG00000104047	DTWD1	15:49913176-49937333	q1	q2	OK	21.1767	20.8217	-0.0243917	-0.0347086	0.95875	0.99995	no

ENSG00000104055	ENSG00000104055	TGM5	15:43524792-43559055	q1	q2	NOTEST	0.0163311	0.0158655	-0.0417258	0	1	1	no

ENSG00000104059	ENSG00000104059	FAM189A1	15:29412456-29862927	q1	q2	OK	2.02537	2.01898	-0.00455906	-0.00454255	0.9942	0.99995	no

ENSG00000104064	ENSG00000104064	GABPB1	15:50569388-50659636	q1	q2	OK	18.5749	22.1272	0.252465	0.365779	0.49805	0.99995	no

ENSG00000104067	ENSG00000104067	TJP1	15:29991570-30261068	q1	q2	OK	39.5342	42.7532	0.112932	0.170945	0.78255	0.99995	no

ENSG00000104081	ENSG00000104081	BMF	15:40380090-40401093	q1	q2	OK	7.07992	6.32928	-0.161692	-0.212653	0.6408	0.99995	no

ENSG00000104093	ENSG00000104093	DMXL2	15:51706293-51915030	q1	q2	OK	28.4074	27.1842	-0.0634992	-0.074041	0.89075	0.99995	no

ENSG00000104112	ENSG00000104112	SCG3	15:51973549-52013223	q1	q2	OK	10.8645	12.4165	0.192639	0.381558	0.5833	0.99995	no

ENSG00000104129	ENSG00000104129	DNAJC17	15:41060066-41106767	q1	q2	OK	12.1405	13.0411	0.103243	0.117448	0.85695	0.99995	no

ENSG00000104131	ENSG00000104131	EIF3J	15:44829254-44955876	q1	q2	OK	66.2932	70.2931	0.084523	0.127381	0.82925	0.99995	no

ENSG00000104133	ENSG00000104133	SPG11	15:44829254-44955876	q1	q2	OK	25.5325	23.9073	-0.0948844	-0.0792701	0.82095	0.99995	no

ENSG00000104140	ENSG00000104140	RHOV	15:41164411-41166487	q1	q2	NOTEST	0.436095	0.391387	-0.156048	0	1	1	no

ENSG00000104142	ENSG00000104142	VPS18	15:41186627-41196173	q1	q2	OK	11.313	10.0049	-0.177269	-0.375569	0.5704	0.99995	no

ENSG00000104147	ENSG00000104147	OIP5	15:41576187-41624819	q1	q2	OK	18.0677	18.132	0.00512788	0.0026947	0.99755	0.99995	no

ENSG00000104154	ENSG00000104154	SLC30A4	15:45722726-45878488	q1	q2	OK	0.89757	0.762844	-0.234637	-0.246262	0.67375	0.99995	no

ENSG00000104164	ENSG00000104164	BLOC1S6	15:45879320-45983492	q1	q2	OK	30.156	31.7039	0.0722154	0.129735	0.80595	0.99995	no

ENSG00000104177	ENSG00000104177	MYEF2	15:48413168-48470714	q1	q2	OK	74.7235	79.9415	0.0973827	0.172901	0.7645	0.99995	no

ENSG00000104205	ENSG00000104205	SGK3	8:67579830-67774257	q1	q2	OK	0.757917	0.774539	0.0312982	0.0161458	0.96205	0.99995	no

ENSG00000104213	ENSG00000104213	PDGFRL	8:17433941-17658426	q1	q2	OK	4.10557	4.71853	0.200753	0.0874441	0.9186	0.99995	no

ENSG00000104218	ENSG00000104218	CSPP1	8:67876333-68255912	q1	q2	OK	10.5238	11.175	0.0866251	0.0693358	0.87835	0.99995	no

ENSG00000104219	ENSG00000104219	ZDHHC2	8:17013537-17082308	q1	q2	OK	9.0046	10.2126	0.181617	0.313168	0.54535	0.99995	no

ENSG00000104221	ENSG00000104221	BRF2	8:37641708-37707422	q1	q2	OK	9.57655	8.6713	-0.143257	-0.0815006	0.8733	0.99995	no

ENSG00000104228	ENSG00000104228	TRIM35	8:27142403-27168836	q1	q2	OK	6.19571	5.72989	-0.112762	-0.158224	0.76595	0.99995	no

ENSG00000104231	ENSG00000104231	ZFAND1	8:82613568-82671750	q1	q2	OK	13.3389	15.7232	0.237254	0.381786	0.50755	0.99995	no

ENSG00000104237	ENSG00000104237	RP1	8:55528626-55543394	q1	q2	NOTEST	0	0	0	0	1	1	no

ENSG00000104267	ENSG00000104267	CA2	8:86239836-86393722	q1	q2	OK	4.84331	4.17369	-0.21467	-0.291861	0.66465	0.99995	no

ENSG00000104290	ENSG00000104290	FZD3	8:28351728-28431775	q1	q2	OK	33.2693	32.9269	-0.0149229	-0.0183655	0.96945	0.99995	no

ENSG00000104299	ENSG00000104299	INTS9	8:28625177-28747759	q1	q2	OK	12.4472	11.7919	-0.0780289	-0.114845	0.8086	0.99995	no

ENSG00000104312	ENSG00000104312	RIPK2	8:90769974-90803291	q1	q2	OK	7.3312	7.27965	-0.0101799	-0.0171942	0.97885	0.99995	no

ENSG00000104313	ENSG00000104313	EYA1	8:72067691-72274467	q1	q2	OK	2.36261	3.03626	0.361914	0.465576	0.4488	0.99995	no

ENSG00000104320	ENSG00000104320	NBN	8:90945563-91064320	q1	q2	OK	17.7294	19.4079	0.130494	0.155037	0.7292	0.99995	no

ENSG00000104321	ENSG00000104321	TRPA1	8:72740401-73030628	q1	q2	OK	2.38035	4.4468	0.901592	0.639072	0.3083	0.99995	no

ENSG00000104324	ENSG00000104324	CPQ	8:97657454-98161882	q1	q2	OK	3.66159	3.56687	-0.0378125	-0.0496145	0.93745	0.99995	no

ENSG00000104325	ENSG00000104325	DECR1	8:90945563-91064320	q1	q2	OK	47.4749	48.8897	0.0423685	0.0584209	0.92545	0.99995	no

ENSG00000104327	ENSG00000104327	CALB1	8:91070835-91107703	q1	q2	OK	17.3502	15.0324	-0.206877	-0.358355	0.5731	0.99995	no

ENSG00000104331	ENSG00000104331	IMPAD1	8:57870491-57906403	q1	q2	OK	26.8366	27.1343	0.0159168	0.0339148	0.9523	0.99995	no

ENSG00000104332	ENSG00000104332	SFRP1	8:41119480-41167016	q1	q2	OK	48.5506	51.4619	0.0840172	0.20314	0.7696	0.99995	no

ENSG00000104341	ENSG00000104341	LAPTM4B	8:98787284-98865241	q1	q2	OK	81.0807	82.5912	0.0266285	0.0643288	0.92595	0.99995	no

ENSG00000104343	ENSG00000104343	UBE2W	8:74332238-74791145	q1	q2	OK	8.70142	9.2991	0.0958408	0.100986	0.8804	0.99995	no

ENSG00000104356	ENSG00000104356	POP1	8:99129524-99172062	q1	q2	OK	8.67783	8.86394	0.0306133	0.051121	0.923	0.99995	no

ENSG00000104361	ENSG00000104361	NIPAL2	8:99202060-99306621	q1	q2	OK	0.870418	0.557614	-0.642441	-0.361971	0.3385	0.99995	no

ENSG00000104365	ENSG00000104365	IKBKB	8:42128819-42189973	q1	q2	OK	11.398	10.8662	-0.0689357	-0.0941311	0.83785	0.99995	no

ENSG00000104368	ENSG00000104368	PLAT	8:42032235-42065242	q1	q2	OK	6.9397	7.06236	0.0252756	0.0384397	0.94725	0.99995	no

ENSG00000104369	ENSG00000104369	JPH1	8:75146934-75401107	q1	q2	NOTEST	0.256984	0.228928	-0.166783	0	1	1	no

ENSG00000104371	ENSG00000104371	DKK4	8:42231585-42234750	q1	q2	NOTEST	0	0	0	0	1	1	no

ENSG00000104375	ENSG00000104375	STK3	8:99413630-99955055	q1	q2	OK	8.80865	10.0537	0.190739	0.304057	0.5788	0.99995	no

ENSG00000104381	ENSG00000104381	GDAP1	8:75146934-75401107	q1	q2	OK	21.9893	24.5585	0.159416	0.328569	0.60445	0.99995	no

ENSG00000104388	ENSG00000104388	RAB2A	8:61429415-61536186	q1	q2	OK	48.8375	49.5804	0.0217814	0.0295914	0.96255	0.99995	no

ENSG00000104408	ENSG00000104408	EIF3E	8:109213444-109447562	q1	q2	OK	175.961	178.322	0.0192249	0.0455776	0.94405	0.99995	no

ENSG00000104412	ENSG00000104412	EMC2	8:109455829-109499145	q1	q2	OK	17.3914	20.419	0.231535	0.431147	0.5298	0.99995	no

ENSG00000104413	ENSG00000104413	ESRP1	8:95653301-95719694	q1	q2	NOTEST	0	0.0127196	inf	0	1	1	no

ENSG00000104415	ENSG00000104415	WISP1	8:134203281-134242587	q1	q2	OK	0.582529	0.283727	-1.03782	-0.348297	0.27885	0.99995	no

ENSG00000104419	ENSG00000104419	NDRG1	8:134249413-134314265	q1	q2	OK	5.27852	4.32284	-0.288154	-0.2752	0.57045	0.99995	no

ENSG00000104427	ENSG00000104427	ZC2HC1A	8:79578281-79717758	q1	q2	OK	13.9073	14.8867	0.0981746	0.180318	0.75595	0.99995	no

ENSG00000104432	ENSG00000104432	IL7	8:79578281-79717758	q1	q2	OK	0.810453	0.803726	-0.0120256	-0.00462746	0.9925	0.99995	no

ENSG00000104435	ENSG00000104435	STMN2	8:80523048-80578410	q1	q2	OK	59.4526	61.0005	0.0370818	0.0831297	0.90005	0.99995	no

ENSG00000104442	ENSG00000104442	ARMC1	8:66514693-66546442	q1	q2	OK	22.2105	23.8412	0.102213	0.22578	0.72375	0.99995	no

ENSG00000104447	ENSG00000104447	TRPS1	8:116420723-116821899	q1	q2	OK	3.71542	4.14122	0.156531	0.200401	0.68155	0.99995	no

ENSG00000104450	ENSG00000104450	SPAG1	8:101170133-101348446	q1	q2	OK	0.432283	0.567764	0.393316	0.132478	0.7824	0.99995	no

ENSG00000104472	ENSG00000104472	CHRAC1	8:141521396-141527236	q1	q2	OK	22.144	24.0239	0.117554	0.207532	0.77195	0.99995	no

ENSG00000104490	ENSG00000104490	NCALD	8:102698770-103137135	q1	q2	OK	96.7203	98.9969	0.0335654	0.0585921	0.89625	0.99995	no

ENSG00000104497	ENSG00000104497	SNX16	8:82704057-82755101	q1	q2	OK	4.23226	5.17339	0.289683	0.383862	0.5064	0.99995	no

ENSG00000104499	ENSG00000104499	GML	8:143915553-143999259	q1	q2	NOTEST	0	0	0	0	1	1	no

ENSG00000104517	ENSG00000104517	UBR5	8:103251621-103425069	q1	q2	OK	57.6088	59.2723	0.0410686	0.0437146	0.9385	0.99995	no

ENSG00000104518	ENSG00000104518	GSDMD	8:144635376-144645232	q1	q2	OK	0.735725	0.361006	-1.02715	-0.46231	0.16305	0.99995	no

ENSG00000104522	ENSG00000104522	TSTA3	8:144694787-144700218	q1	q2	OK	23.8973	22.6572	-0.0768791	-0.136344	0.8379	0.99995	no

ENSG00000104524	ENSG00000104524	PYCRL	8:144686082-144691943	q1	q2	OK	4.85651	4.16094	-0.223009	-0.314277	0.6123	0.99995	no

ENSG00000104529	ENSG00000104529	EEF1D	8:144661805-144682485	q1	q2	OK	278.736	256.555	-0.119628	-0.227159	0.74855	0.99995	no

ENSG00000104537	ENSG00000104537	ANXA13	8:124693033-124749647	q1	q2	NOTEST	0	0.0388325	inf	0	1	1	no

ENSG00000104549	ENSG00000104549	SQLE	8:126010738-126034525	q1	q2	OK	123.741	154.201	0.317484	0.60364	0.30575	0.99995	no

ENSG00000104611	ENSG00000104611	SH2D4A	8:19171127-19253729	q1	q2	NOTEST	0.187971	0.251266	0.418706	0	1	1	no

ENSG00000104613	ENSG00000104613	INTS10	8:19674650-19709594	q1	q2	OK	28.384	31.318	0.141916	0.231112	0.6578	0.99995	no

ENSG00000104626	ENSG00000104626	ERI1	8:8859656-8974256	q1	q2	OK	11.4829	12.6556	0.140282	0.262524	0.6605	0.99995	no

ENSG00000104635	ENSG00000104635	SLC39A14	8:22224761-22291642	q1	q2	OK	12.5874	13.7253	0.124857	0.253556	0.6748	0.99995	no

ENSG00000104643	ENSG00000104643	MTMR9	8:11141924-11185646	q1	q2	OK	8.40693	9.4704	0.171847	0.347901	0.5782	0.99995	no

ENSG00000104660	ENSG00000104660	LEPROTL1	8:29952913-30041156	q1	q2	OK	76.8975	89.8034	0.223834	0.44851	0.46095	0.99995	no

ENSG00000104671	ENSG00000104671	DCTN6	8:29952913-30041156	q1	q2	OK	40.989	44.8765	0.130725	0.116047	0.86215	0.99995	no

ENSG00000104679	ENSG00000104679	R3HCC1	8:23127632-23153792	q1	q2	OK	23.4767	20.7306	-0.179467	-0.341761	0.60135	0.99995	no

ENSG00000104687	ENSG00000104687	GSR	8:30535582-30585443	q1	q2	OK	19.2256	19.0944	-0.00987632	-0.0224045	0.97435	0.99995	no

ENSG00000104689	ENSG00000104689	TNFRSF10A	8:23046791-23088439	q1	q2	NOTEST	0.389061	0.224818	-0.791236	0	1	1	no

ENSG00000104691	ENSG00000104691	UBXN8	8:30589763-30624522	q1	q2	OK	4.98922	5.6618	0.182446	0.237189	0.68665	0.99995	no

ENSG00000104695	ENSG00000104695	PPP2CB	8:30631972-30671830	q1	q2	OK	79.5201	85.3702	0.102412	0.245711	0.70135	0.99995	no

ENSG00000104714	ENSG00000104714	ERICH1	8:564745-1212580	q1	q2	OK	13.011	13.9915	0.104813	0.160733	0.7839	0.99995	no

ENSG00000104722	ENSG00000104722	NEFM	8:24769677-24776607	q1	q2	OK	165.02	209.81	0.346442	0.696187	0.2673	0.99995	no

ENSG00000104723	ENSG00000104723	TUSC3	8:15274723-15624158	q1	q2	OK	100.052	103.998	0.0558056	0.128768	0.8509	0.99995	no

ENSG00000104725	ENSG00000104725	NEFL	8:24808467-24814624	q1	q2	OK	42.9125	46.1886	0.106138	0.260667	0.70505	0.99995	no

ENSG00000104728	ENSG00000104728	ARHGEF10	8:1772141-1906807	q1	q2	OK	14.9123	15.0983	0.0178853	0.0307349	0.95275	0.99995	no

ENSG00000104731	ENSG00000104731	KLHDC4	16:87634131-87799598	q1	q2	OK	14.7719	13.7284	-0.105689	-0.122748	0.8078	0.99995	no

ENSG00000104738	ENSG00000104738	MCM4	8:48872744-48890720	q1	q2	OK	93.5881	89.1671	-0.0698127	-0.088812	0.81415	0.99995	no

ENSG00000104755	ENSG00000104755	ADAM2	8:39601253-39695808	q1	q2	NOTEST	0	0	0	0	1	1	no

ENSG00000104756	ENSG00000104756	KCTD9	8:25285365-25315992	q1	q2	OK	12.9961	15.2458	0.230329	0.34859	0.4623	0.99995	no

ENSG00000104760	ENSG00000104760	FGL1	8:17721888-17767874	q1	q2	NOTEST	0.041419	0	#NAME?	0	1	1	no

ENSG00000104763	ENSG00000104763	ASAH1	8:17913933-17953903	q1	q2	OK	38.7769	41.9552	0.113653	0.208265	0.7317	0.99995	no

ENSG00000104765	ENSG00000104765	BNIP3L	8:26236774-26371608	q1	q2	OK	53.3679	49.643	-0.104383	-0.148034	0.80505	0.99995	no

ENSG00000104774	ENSG00000104774	MAN2B1	19:12754088-12792716	q1	q2	OK	19.2872	15.8092	-0.286878	-0.199143	0.5838	0.99995	no

ENSG00000104783	ENSG00000104783	KCNN4	19:44270684-44285409	q1	q2	NOTEST	0.199605	0.108599	-0.878141	0	1	1	no

ENSG00000104804	ENSG00000104804	TULP2	19:49384221-49401990	q1	q2	NOTEST	0.031377	0.0296582	-0.0812784	0	1	1	no

ENSG00000104805	ENSG00000104805	NUCB1	19:49403306-49426629	q1	q2	OK	63.9804	57.3849	-0.15696	-0.317975	0.63315	0.99995	no

ENSG00000104808	ENSG00000104808	DHDH	19:49436938-49448226	q1	q2	NOTEST	0.364247	0.143082	-1.34808	0	1	1	no

ENSG00000104812	ENSG00000104812	GYS1	19:49471381-49496567	q1	q2	OK	18.2843	16.9453	-0.109716	-0.156137	0.73995	0.99995	no

ENSG00000104814	ENSG00000104814	MAP4K1	19:39078280-39109522	q1	q2	OK	0.416529	0.865757	1.05554	0.553549	0.1908	0.99995	no

ENSG00000104818	ENSG00000104818	CGB2	19:49526125-49552400	q1	q2	NOTEST	0	0	0	0	1	1	no

ENSG00000104823	ENSG00000104823	ECH1	19:39306061-39342987	q1	q2	OK	33.7785	32.7775	-0.0433993	-0.0240982	0.9642	0.99995	no

ENSG00000104824	ENSG00000104824	HNRNPL	19:39306061-39342987	q1	q2	OK	227.327	233.727	0.0400512	0.0809613	0.9003	0.99995	no

ENSG00000104825	ENSG00000104825	NFKBIB	19:39369196-39399533	q1	q2	OK	8.65121	7.90743	-0.129694	-0.152514	0.8258	0.99995	no

ENSG00000104826	ENSG00000104826	LHB	19:49496704-49520338	q1	q2	NOTEST	0.0800592	0.233259	1.54279	0	1	1	no

ENSG00000104827	ENSG00000104827	CGB	19:49526125-49552400	q1	q2	NOTEST	0	0	0	0	1	1	no

ENSG00000104833	ENSG00000104833	TUBB4A	19:6494329-6502859	q1	q2	OK	16.0967	13.7339	-0.229021	-0.465526	0.4643	0.99995	no

ENSG00000104835	ENSG00000104835	SARS2	19:39405905-39466550	q1	q2	OK	21.3339	19.4632	-0.1324	-0.130731	0.8441	0.99995	no

ENSG00000104848	ENSG00000104848	KCNA7	19:49570674-49576198	q1	q2	NOTEST	0.029052	0.0228424	-0.346926	0	1	1	no

ENSG00000104852	ENSG00000104852	SNRNP70	19:49588675-49611862	q1	q2	OK	152.435	145.083	-0.0713169	-0.151929	0.81815	0.99995	no

ENSG00000104853	ENSG00000104853	CLPTM1	19:45457841-45496599	q1	q2	OK	43.9943	36.9943	-0.250013	-0.52786	0.39225	0.99995	no

ENSG00000104856	ENSG00000104856	RELB	19:45504687-45541452	q1	q2	OK	2.37796	2.6035	0.130728	0.13302	0.83645	0.99995	no

ENSG00000104859	ENSG00000104859	CLASRP	19:45542297-45574214	q1	q2	OK	43.6812	37.9208	-0.204023	-0.258622	0.63755	0.99995	no

ENSG00000104863	ENSG00000104863	LIN7B	19:49617580-49622397	q1	q2	OK	5.2022	3.77273	-0.463511	-0.4243	0.57935	0.99995	no

ENSG00000104866	ENSG00000104866	PPP1R37	19:45574757-45808541	q1	q2	OK	14.3063	12.1278	-0.238333	-0.242191	0.64275	0.99995	no

ENSG00000104870	ENSG00000104870	FCGRT	19:50010072-50029590	q1	q2	OK	19.7864	15.7739	-0.326974	-0.553777	0.39525	0.99995	no

ENSG00000104872	ENSG00000104872	PIH1D1	19:49949554-49995565	q1	q2	OK	52.2766	49.5459	-0.0774015	-0.0354533	0.9333	0.99995	no

ENSG00000104879	ENSG00000104879	CKM	19:45809671-45826235	q1	q2	NOTEST	0.0171805	0.0802073	2.22296	0	1	1	no

ENSG00000104880	ENSG00000104880	ARHGEF18	19:7413847-7537363	q1	q2	OK	12.6835	12.3844	-0.0344284	-0.0564784	0.91165	0.99995	no

ENSG00000104881	ENSG00000104881	PPP1R13L	19:45882891-45982086	q1	q2	OK	2.64951	1.71879	-0.624336	-0.221512	0.55185	0.99995	no

ENSG00000104883	ENSG00000104883	PEX11G	19:7541760-7585912	q1	q2	OK	0.92075	0.355542	-1.37279	-0.254073	0.3951	0.99995	no

ENSG00000104884	ENSG00000104884	ERCC2	19:45836691-45874176	q1	q2	OK	22.0896	19.3906	-0.18801	-0.333205	0.56565	0.99995	no

ENSG00000104885	ENSG00000104885	DOT1L	19:2100987-2248678	q1	q2	OK	35.1992	31.0337	-0.181707	-0.0902637	0.7668	0.99995	no

ENSG00000104886	ENSG00000104886	PLEKHJ1	19:2100987-2248678	q1	q2	OK	66.6676	59.9271	-0.153776	-0.124395	0.84715	0.99995	no

ENSG00000104888	ENSG00000104888	SLC17A7	19:49932657-49945617	q1	q2	NOTEST	0.297206	0.343584	0.209202	0	1	1	no

ENSG00000104889	ENSG00000104889	RNASEH2A	19:12873816-12985765	q1	q2	OK	58.4251	51.0821	-0.193771	-0.192747	0.75985	0.99995	no

ENSG00000104892	ENSG00000104892	KLC3	19:45836691-45874176	q1	q2	NOTEST	0.0391222	0.0296928	-0.397878	0	1	1	no

ENSG00000104894	ENSG00000104894	CD37	19:49834873-49891338	q1	q2	OK	10.679	10.4572	-0.030281	-0.0166747	0.97365	0.99995	no

ENSG00000104897	ENSG00000104897	SF3A2	19:2100987-2248678	q1	q2	OK	52.1779	46.9609	-0.151978	-0.117814	0.82895	0.99995	no

ENSG00000104899	ENSG00000104899	AMH	19:2249307-2252072	q1	q2	OK	20.3914	15.9879	-0.350975	-0.558781	0.42505	0.99995	no

ENSG00000104901	ENSG00000104901	DKKL1	19:49834873-49891338	q1	q2	OK	4.73723	3.62025	-0.387955	-0.0925246	0.8157	0.99995	no

ENSG00000104903	ENSG00000104903	LYL1	19:13209846-13213975	q1	q2	NOTEST	0.111387	0.0715393	-0.638771	0	1	1	no

ENSG00000104904	ENSG00000104904	OAZ1	19:2252250-2273487	q1	q2	OK	290.93	270.253	-0.106359	-0.249897	0.7224	0.99995	no

ENSG00000104907	ENSG00000104907	TRMT1	19:13215715-13228381	q1	q2	OK	19.2774	22.1549	0.200715	0.294949	0.5984	0.99995	no

ENSG00000104915	ENSG00000104915	STX10	19:13254871-13261197	q1	q2	OK	25.2601	20.5078	-0.300693	-0.538715	0.44035	0.99995	no

ENSG00000104918	ENSG00000104918	RETN	19:7733929-7735334	q1	q2	NOTEST	0	0	0	0	1	1	no

ENSG00000104921	ENSG00000104921	FCER2	19:7753643-7767032	q1	q2	NOTEST	0.0803243	0	#NAME?	0	1	1	no

ENSG00000104936	ENSG00000104936	DMPK	19:46268042-46296060	q1	q2	OK	48.7227	43.2551	-0.171725	-0.174643	0.65855	0.99995	no

ENSG00000104938	ENSG00000104938	CLEC4M	19:7828034-7834491	q1	q2	NOTEST	0	0.0600413	inf	0	1	1	no

ENSG00000104941	ENSG00000104941	RSPH6A	19:46298967-46318577	q1	q2	NOTEST	0.0152996	0	#NAME?	0	1	1	no

ENSG00000104946	ENSG00000104946	TBC1D17	19:50372294-50392005	q1	q2	OK	13.1069	10.4661	-0.324603	-0.368391	0.54825	0.99995	no

ENSG00000104951	ENSG00000104951	IL4I1	19:50392910-50464429	q1	q2	NOTEST	0.179147	0.102814	-0.801111	0	1	1	no

ENSG00000104953	ENSG00000104953	TLE6	19:2977443-2995177	q1	q2	OK	0.951469	1.11233	0.225357	0.211116	0.70045	0.99995	no

ENSG00000104957	ENSG00000104957	CCDC130	19:13842573-13874110	q1	q2	OK	25.5084	23.2122	-0.136091	-0.244454	0.7159	0.99995	no

ENSG00000104960	ENSG00000104960	PTOV1	19:50321538-50364001	q1	q2	OK	180.01	153.617	-0.228738	-0.438146	0.48735	0.99995	no

ENSG00000104964	ENSG00000104964	AES	19:3052907-3063105	q1	q2	OK	202.978	199.828	-0.0225622	-0.0519177	0.938	0.99995	no

ENSG00000104967	ENSG00000104967	NOVA2	19:46436991-46476804	q1	q2	OK	21.398	19.9114	-0.103884	-0.226849	0.7089	0.99995	no

ENSG00000104969	ENSG00000104969	SGTA	19:2754711-2783369	q1	q2	OK	46.2795	41.6819	-0.150951	-0.345121	0.5911	0.99995	no

ENSG00000104970	ENSG00000104970	KIR3DX1	19:55043908-55057053	q1	q2	NOTEST	0	0	0	0	1	1	no

ENSG00000104972	ENSG00000104972	LILRB1	19:55084386-55150343	q1	q2	NOTEST	0	0	0	0	1	1	no

ENSG00000104973	ENSG00000104973	MED25	19:50321538-50364001	q1	q2	OK	25.1001	20.0442	-0.32451	-0.25927	0.64845	0.99995	no

ENSG00000104974	ENSG00000104974	LILRA1	19:55084386-55150343	q1	q2	NOTEST	0	0	0	0	1	1	no

ENSG00000104976	ENSG00000104976	SNAPC2	19:7985200-7988135	q1	q2	OK	9.60266	8.75914	-0.132646	-0.191903	0.7564	0.99995	no

ENSG00000104979	ENSG00000104979	C19orf53	19:13875345-13889276	q1	q2	OK	78.9884	75.1214	-0.0724165	-0.127516	0.85745	0.99995	no

ENSG00000104980	ENSG00000104980	TIMM44	19:7989371-8008805	q1	q2	OK	30.6479	29.3785	-0.0610275	-0.0762658	0.89835	0.99995	no

ENSG00000104983	ENSG00000104983	CCDC61	19:46498338-46526323	q1	q2	OK	3.68866	2.89786	-0.348107	-0.357532	0.59425	0.99995	no

ENSG00000104998	ENSG00000104998	IL27RA	19:14142559-14163743	q1	q2	OK	3.70774	3.70741	-0.000127946	-0.000197713	0.9971	0.99995	no

ENSG00000105011	ENSG00000105011	ASF1B	19:14229917-14247768	q1	q2	OK	23.3793	21.1289	-0.146013	-0.289455	0.66085	0.99995	no

ENSG00000105048	ENSG00000105048	TNNT1	19:55644161-55660722	q1	q2	NOTEST	0.300582	0.163749	-0.876278	0	1	1	no

ENSG00000105053	ENSG00000105053	VRK3	19:50479723-50570050	q1	q2	OK	15.955	18.4823	0.21213	0.277212	0.63855	0.99995	no

ENSG00000105058	ENSG00000105058	FAM32A	19:16296190-16302857	q1	q2	OK	80.1661	71.0448	-0.174264	-0.417223	0.55235	0.99995	no

ENSG00000105063	ENSG00000105063	PPP6R1	19:55738006-55770363	q1	q2	OK	19.1548	17.82	-0.104208	-0.183758	0.75165	0.99995	no

ENSG00000105072	ENSG00000105072	C19orf44	19:16589867-16771253	q1	q2	OK	5.32454	3.65611	-0.542347	-0.2317	0.65	0.99995	no

ENSG00000105085	ENSG00000105085	MED26	19:16589867-16771253	q1	q2	OK	4.09896	3.91024	-0.0679992	-0.0323114	0.9683	0.99995	no

ENSG00000105088	ENSG00000105088	OLFM2	19:9964393-10047228	q1	q2	OK	25.2931	18.5862	-0.444516	-0.878857	0.2102	0.99995	no

ENSG00000105122	ENSG00000105122	RASAL3	19:15562434-15575382	q1	q2	NOTEST	0	0.0181765	inf	0	1	1	no

ENSG00000105127	ENSG00000105127	AKAP8	19:15464195-15529952	q1	q2	OK	15.4383	16.2304	0.0721816	0.0906014	0.8818	0.99995	no

ENSG00000105131	ENSG00000105131	EPHX3	19:15337729-15344246	q1	q2	NOTEST	0.185383	0.148019	-0.324724	0	1	1	no

ENSG00000105135	ENSG00000105135	ILVBL	19:15218213-15236985	q1	q2	OK	23.9429	19.0776	-0.327718	-0.435818	0.45305	0.99995	no

ENSG00000105136	ENSG00000105136	ZNF419	19:57999078-58029772	q1	q2	OK	14.8975	13.0795	-0.187756	-0.275221	0.6365	0.99995	no

ENSG00000105137	ENSG00000105137	SYDE1	19:15218213-15236985	q1	q2	OK	11.8432	11.0777	-0.096402	-0.152377	0.8263	0.99995	no

ENSG00000105141	ENSG00000105141	CASP14	19:15160194-15169104	q1	q2	NOTEST	0	0	0	0	1	1	no

ENSG00000105143	ENSG00000105143	SLC1A6	19:15060845-15134081	q1	q2	OK	3.54729	4.30687	0.279923	0.360226	0.55725	0.99995	no

ENSG00000105146	ENSG00000105146	AURKC	19:57742376-57746916	q1	q2	OK	0.469722	0.543943	0.211648	0.142586	0.77405	0.99995	no

ENSG00000105171	ENSG00000105171	POP4	19:30094923-30108144	q1	q2	OK	39.9992	40.1692	0.00611719	0.0102624	0.98805	0.99995	no

ENSG00000105173	ENSG00000105173	CCNE1	19:30302804-30315215	q1	q2	OK	13.2559	12.8466	-0.0452458	-0.0764248	0.90845	0.99995	no

ENSG00000105176	ENSG00000105176	URI1	19:30414550-30506611	q1	q2	OK	41.0718	44.501	0.115688	0.261881	0.70525	0.99995	no

ENSG00000105185	ENSG00000105185	PDCD5	19:33071973-33078358	q1	q2	OK	79.5849	87.4725	0.136335	0.281345	0.6813	0.99995	no

ENSG00000105186	ENSG00000105186	ANKRD27	19:33087912-33169206	q1	q2	OK	6.69803	7.34636	0.133294	0.167076	0.7062	0.99995	no

ENSG00000105193	ENSG00000105193	RPS16	19:39923846-39926588	q1	q2	OK	819.536	796.339	-0.0414234	-0.0986733	0.88545	0.99995	no

ENSG00000105197	ENSG00000105197	TIMM50	19:39971051-39984422	q1	q2	OK	42.381	42.8485	0.0158256	0.032954	0.9558	0.99995	no

ENSG00000105198	ENSG00000105198	LGALS13	19:40093163-40098116	q1	q2	NOTEST	0	0.0702595	inf	0	1	1	no

ENSG00000105202	ENSG00000105202	FBL	19:40325097-40337054	q1	q2	OK	203.685	202.394	-0.00917146	-0.0217969	0.97595	0.99995	no

ENSG00000105204	ENSG00000105204	DYRK1B	19:40315989-40324841	q1	q2	OK	17.1958	14.8625	-0.210383	-0.33954	0.55725	0.99995	no

ENSG00000105205	ENSG00000105205	CLC	19:40221889-40228668	q1	q2	OK	0.895135	0.660614	-0.438297	-0.364474	0.60375	0.99995	no

ENSG00000105219	ENSG00000105219	CNTD2	19:40728114-40732597	q1	q2	NOTEST	0.304321	0.0592339	-2.3611	0	1	1	no

ENSG00000105220	ENSG00000105220	GPI	19:34850384-34919178	q1	q2	OK	54.3109	52.1841	-0.0576301	-0.12562	0.84855	0.99995	no

ENSG00000105221	ENSG00000105221	AKT2	19:40736223-40791443	q1	q2	OK	56.7565	49.1494	-0.20761	-0.33985	0.50215	0.99995	no

ENSG00000105223	ENSG00000105223	PLD3	19:40825442-40896094	q1	q2	OK	156.989	139.215	-0.173348	-0.321214	0.55505	0.99995	no

ENSG00000105227	ENSG00000105227	PRX	19:40899674-40919273	q1	q2	OK	1.67147	1.40329	-0.252307	-0.362405	0.5895	0.99995	no

ENSG00000105229	ENSG00000105229	PIAS4	19:4007643-4039384	q1	q2	OK	15.8175	14.6811	-0.107558	-0.131713	0.81515	0.99995	no

ENSG00000105245	ENSG00000105245	NUMBL	19:41172595-41196877	q1	q2	OK	10.6549	9.43393	-0.175589	-0.259676	0.65285	0.99995	no

ENSG00000105246	ENSG00000105246	EBI3	19:4229494-4237528	q1	q2	NOTEST	0	0.049942	inf	0	1	1	no

ENSG00000105248	ENSG00000105248	CCDC94	19:4247075-4269087	q1	q2	OK	13.4544	14.4369	0.101678	0.185921	0.78675	0.99995	no

ENSG00000105251	ENSG00000105251	SHD	19:4278597-4290721	q1	q2	OK	12.3089	8.90328	-0.467291	-0.62067	0.2585	0.99995	no

ENSG00000105254	ENSG00000105254	TBCB	19:36602103-36616849	q1	q2	OK	127.669	121.973	-0.0658459	-0.134769	0.84435	0.99995	no

ENSG00000105255	ENSG00000105255	FSD1	19:4304596-4323840	q1	q2	OK	29.1104	27.3025	-0.0925019	-0.184639	0.783	0.99995	no

ENSG00000105258	ENSG00000105258	POLR2I	19:36602103-36616849	q1	q2	OK	31.6672	31.8887	0.0100555	0.00941915	0.98945	0.99995	no

ENSG00000105261	ENSG00000105261	OVOL3	19:36602103-36616849	q1	q2	NOTEST	0	0	0	0	1	1	no

ENSG00000105270	ENSG00000105270	CLIP3	19:36505409-36545664	q1	q2	OK	32.4874	28.1656	-0.205948	-0.469389	0.4865	0.99995	no

ENSG00000105278	ENSG00000105278	ZFR2	19:3804021-3869030	q1	q2	OK	2.84978	1.71946	-0.728897	-0.592573	0.2881	0.99995	no

ENSG00000105281	ENSG00000105281	SLC1A5	19:47222763-47291851	q1	q2	OK	36.8324	34.8226	-0.0809536	-0.100441	0.89465	0.99995	no

ENSG00000105287	ENSG00000105287	PRKD2	19:47150868-47220384	q1	q2	OK	14.9354	15.0131	0.00748677	0.00895719	0.98535	0.99995	no

ENSG00000105289	ENSG00000105289	TJP3	19:3708106-3750811	q1	q2	NOTEST	0.282397	0.378997	0.424464	0	1	1	no

ENSG00000105290	ENSG00000105290	APLP1	19:36316865-36370693	q1	q2	OK	119.506	116.484	-0.036951	-0.0518036	0.93055	0.99995	no

ENSG00000105298	ENSG00000105298	CACTIN	19:3607244-3626813	q1	q2	OK	13.0307	11.7059	-0.154678	-0.298864	0.64695	0.99995	no

ENSG00000105321	ENSG00000105321	CCDC9	19:47759236-47775210	q1	q2	OK	13.0003	12.6693	-0.0372027	-0.0456214	0.93435	0.99995	no

ENSG00000105323	ENSG00000105323	HNRNPUL1	19:41768400-41930910	q1	q2	OK	127.235	123.257	-0.045825	-0.0725412	0.89345	0.99995	no

ENSG00000105325	ENSG00000105325	FZR1	19:3506270-3579086	q1	q2	OK	23.1304	20.6099	-0.166458	-0.164	0.76255	0.99995	no

ENSG00000105327	ENSG00000105327	BBC3	19:47724080-47736023	q1	q2	OK	13.6764	8.62165	-0.665653	-1.10942	0.12525	0.99995	no

ENSG00000105329	ENSG00000105329	TGFB1	19:41768400-41930910	q1	q2	OK	8.05127	5.90325	-0.447707	-0.13303	0.77635	0.99995	no

ENSG00000105339	ENSG00000105339	DENND3	8:142127376-142205907	q1	q2	OK	3.506	3.85432	0.13665	0.136964	0.78665	0.99995	no

ENSG00000105341	ENSG00000105341	ATP5SL	19:41937222-41946622	q1	q2	OK	3.90565	1.94566	-1.0053	-0.878555	0.1742	0.99995	no

ENSG00000105352	ENSG00000105352	CEACAM4	19:42125343-42133442	q1	q2	NOTEST	0	0	0	0	1	1	no

ENSG00000105355	ENSG00000105355	PLIN3	19:4838343-4867780	q1	q2	OK	24.2612	21.8415	-0.151582	-0.339247	0.62795	0.99995	no

ENSG00000105357	ENSG00000105357	MYH14	19:50691442-50813802	q1	q2	NOTEST	0.47937	0.35312	-0.44098	0	1	1	no

ENSG00000105364	ENSG00000105364	MRPL4	19:10362576-10399695	q1	q2	OK	26.2794	25.0623	-0.0684151	-0.13196	0.85105	0.99995	no

ENSG00000105366	ENSG00000105366	SIGLEC8	19:51954100-51961710	q1	q2	NOTEST	0	0	0	0	1	1	no

ENSG00000105369	ENSG00000105369	CD79A	19:42381189-42385439	q1	q2	NOTEST	0	0	0	0	1	1	no

ENSG00000105370	ENSG00000105370	LIM2	19:51883162-51891214	q1	q2	NOTEST	0.0379318	0.0371826	-0.0287805	0	1	1	no

ENSG00000105371	ENSG00000105371	ICAM4	19:10362576-10399695	q1	q2	NOTEST	0.0241908	0	#NAME?	0	1	1	no

ENSG00000105372	ENSG00000105372	RPS19	19:42363987-42376994	q1	q2	OK	2.34998	1.9982	-0.233951	-0.191161	0.75945	0.99995	no

ENSG00000105373	ENSG00000105373	GLTSCR2	19:48248778-48272097	q1	q2	OK	100.842	90.2091	-0.160756	-0.366624	0.5949	0.99995	no

ENSG00000105374	ENSG00000105374	NKG7	19:51874859-51875969	q1	q2	NOTEST	0	0.0760181	inf	0	1	1	no

ENSG00000105376	ENSG00000105376	ICAM5	19:10400656-10407454	q1	q2	NOTEST	0.0946699	0.00958598	-3.30391	0	1	1	no

ENSG00000105379	ENSG00000105379	ETFB	19:51848422-51872257	q1	q2	OK	36.379	28.9003	-0.332022	-0.595899	0.39145	0.99995	no

ENSG00000105383	ENSG00000105383	CD33	19:51728319-51747115	q1	q2	NOTEST	0.217042	0.0727389	-1.57718	0	1	1	no

ENSG00000105388	ENSG00000105388	CEACAM5	19:42212503-42290801	q1	q2	NOTEST	0	0	0	0	1	1	no

ENSG00000105392	ENSG00000105392	CRX	19:48322702-48364769	q1	q2	NOTEST	0	0.00578302	inf	0	1	1	no

ENSG00000105393	ENSG00000105393	BABAM1	19:17342691-17398455	q1	q2	OK	49.1793	43.0773	-0.191121	-0.291427	0.65	0.99995	no

ENSG00000105397	ENSG00000105397	TYK2	19:10461208-10491352	q1	q2	OK	37.9909	36.508	-0.057441	-0.0753908	0.90875	0.99995	no

ENSG00000105398	ENSG00000105398	SULT2A1	19:48373722-48389654	q1	q2	NOTEST	0	0	0	0	1	1	no

ENSG00000105401	ENSG00000105401	CDC37	19:10501809-10580305	q1	q2	OK	93.7345	88.9335	-0.075852	-0.177938	0.7933	0.99995	no

ENSG00000105402	ENSG00000105402	NAPA	19:47987538-48018497	q1	q2	OK	38.0871	32.5297	-0.227546	-0.475923	0.45285	0.99995	no

ENSG00000105404	ENSG00000105404	RABAC1	19:42460832-42463542	q1	q2	OK	0.96215	1.24605	0.373023	0.310048	0.58205	0.99995	no

ENSG00000105409	ENSG00000105409	ATP1A3	19:42470733-42501649	q1	q2	OK	0.283163	0.614256	1.11721	0.667966	0.10235	0.99995	no

ENSG00000105419	ENSG00000105419	MEIS3	19:47906380-47922780	q1	q2	OK	65.6388	60.0649	-0.128027	-0.274686	0.67835	0.99995	no

ENSG00000105426	ENSG00000105426	PTPRS	19:5158505-5340814	q1	q2	OK	79.6708	72.6014	-0.134054	-0.192894	0.68135	0.99995	no

ENSG00000105427	ENSG00000105427	CNFN	19:42891170-42894439	q1	q2	OK	1.22722	1.48687	0.276894	0.231812	0.72215	0.99995	no

ENSG00000105428	ENSG00000105428	ZNRF4	19:5455425-5456867	q1	q2	NOTEST	0	0	0	0	1	1	no

ENSG00000105429	ENSG00000105429	MEGF8	19:42829760-42882921	q1	q2	OK	21.5789	19.3061	-0.16056	-0.271669	0.6561	0.99995	no

ENSG00000105438	ENSG00000105438	KDELR1	19:48885826-48894810	q1	q2	OK	107.426	104.448	-0.0405618	-0.0995503	0.88815	0.99995	no

ENSG00000105443	ENSG00000105443	CYTH2	19:48949029-48985571	q1	q2	OK	86.1031	78.2009	-0.138881	-0.258528	0.70595	0.99995	no

ENSG00000105447	ENSG00000105447	GRWD1	19:48949029-48985571	q1	q2	OK	17.6848	17.0474	-0.0529579	-0.026602	0.9642	0.99995	no

ENSG00000105464	ENSG00000105464	GRIN2D	19:48898131-48948188	q1	q2	OK	2.88122	2.1267	-0.438067	-0.732083	0.28735	0.99995	no

ENSG00000105467	ENSG00000105467	SYNGR4	19:48867651-48879633	q1	q2	NOTEST	0.123644	0.20568	0.734207	0	1	1	no

ENSG00000105472	ENSG00000105472	CLEC11A	19:51226585-51228974	q1	q2	OK	3.66891	3.42694	-0.0984325	-0.126839	0.8455	0.99995	no

ENSG00000105479	ENSG00000105479	CCDC114	19:48799713-48833810	q1	q2	NOTEST	0.225665	0.167733	-0.42802	0	1	1	no

ENSG00000105483	ENSG00000105483	CARD8	19:48673948-48790865	q1	q2	OK	27.1409	28.6589	0.0785148	0.101855	0.82345	0.99995	no

ENSG00000105486	ENSG00000105486	LIG1	19:48618701-48673860	q1	q2	OK	42.4532	38.4329	-0.143533	-0.228021	0.68845	0.99995	no

ENSG00000105492	ENSG00000105492	SIGLEC6	19:52022778-52035110	q1	q2	NOTEST	0	0	0	0	1	1	no

ENSG00000105497	ENSG00000105497	ZNF175	19:52074550-52092991	q1	q2	OK	5.43031	5.80704	0.0967678	0.130174	0.79345	0.99995	no

ENSG00000105499	ENSG00000105499	PLA2G4C	19:48551099-48614074	q1	q2	OK	1.97386	1.11927	-0.818461	-0.577203	0.18415	0.99995	no

ENSG00000105501	ENSG00000105501	SIGLEC5	19:52114780-52150151	q1	q2	NOTEST	0	0	0	0	1	1	no

ENSG00000105507	ENSG00000105507	CABP5	19:48533209-48547310	q1	q2	NOTEST	0	0	0	0	1	1	no

ENSG00000105509	ENSG00000105509	HAS1	19:52216364-52227247	q1	q2	NOTEST	0.156946	0.0740696	-1.08332	0	1	1	no

ENSG00000105514	ENSG00000105514	RAB3D	19:11406823-11465620	q1	q2	OK	4.25293	4.27251	0.00662683	0.00963625	0.9893	0.99995	no

ENSG00000105516	ENSG00000105516	DBP	19:49118584-49140695	q1	q2	OK	2.77777	2.92885	0.0764059	0.015991	0.9438	0.99995	no

ENSG00000105518	ENSG00000105518	TMEM205	19:11406823-11465620	q1	q2	OK	22.2029	17.5717	-0.337495	-0.427547	0.5544	0.99995	no

ENSG00000105519	ENSG00000105519	CAPS	19:5904851-5915888	q1	q2	OK	3.00987	1.97766	-0.605902	-0.553157	0.35475	0.99995	no

ENSG00000105520	ENSG00000105520	DKFZP761J1410	19:11466061-11476374	q1	q2	OK	20.8724	17.6898	-0.238674	-0.431391	0.44745	0.99995	no

ENSG00000105523	ENSG00000105523	FAM83E	19:49104066-49118111	q1	q2	NOTEST	0.449566	0.275123	-0.708453	0	1	1	no

ENSG00000105538	ENSG00000105538	RASIP1	19:49223843-49243978	q1	q2	OK	0.768501	0.827427	0.106585	0.0800252	0.88455	0.99995	no

ENSG00000105549	ENSG00000105549	THEG	19:361749-376013	q1	q2	NOTEST	0.0494919	0.0497868	0.00856973	0	1	1	no

ENSG00000105550	ENSG00000105550	FGF21	19:49258815-49261587	q1	q2	NOTEST	0	0	0	0	1	1	no

ENSG00000105552	ENSG00000105552	BCAT2	19:49298318-49314286	q1	q2	OK	14.569	13.413	-0.119264	-0.191581	0.7762	0.99995	no

ENSG00000105556	ENSG00000105556	MIER2	19:305572-344798	q1	q2	OK	13.3081	12.8511	-0.0504146	-0.0893656	0.87605	0.99995	no

ENSG00000105559	ENSG00000105559	PLEKHA4	19:49340353-49371889	q1	q2	OK	9.194	9.14156	-0.00825284	-0.0111864	0.9838	0.99995	no

ENSG00000105568	ENSG00000105568	PPP2R1A	19:52693291-52734547	q1	q2	OK	152.429	144.218	-0.0798823	-0.173169	0.7817	0.99995	no

ENSG00000105576	ENSG00000105576	TNPO2	19:12810007-12834825	q1	q2	OK	33.4505	34.3265	0.0372916	0.0501887	0.8999	0.99995	no

ENSG00000105583	ENSG00000105583	WDR83OS	19:12754088-12792716	q1	q2	OK	90.2967	74.5297	-0.276857	-0.28561	0.6767	0.99995	no

ENSG00000105605	ENSG00000105605	CACNG7	19:54412588-54447195	q1	q2	OK	47.6132	43.2023	-0.140255	-0.337559	0.6259	0.99995	no

ENSG00000105607	ENSG00000105607	GCDH	19:13001839-13030090	q1	q2	OK	13.9228	11.6548	-0.256517	-0.392937	0.52805	0.99995	no

ENSG00000105609	ENSG00000105609	LILRB5	19:54754262-54761164	q1	q2	NOTEST	0	0	0	0	1	1	no

ENSG00000105610	ENSG00000105610	KLF1	19:12995236-12997995	q1	q2	NOTEST	0.0539517	0.0519776	-0.0537795	0	1	1	no

ENSG00000105612	ENSG00000105612	DNASE2	19:12986024-12994902	q1	q2	OK	5.59246	4.39526	-0.347533	-0.531492	0.4285	0.99995	no

ENSG00000105613	ENSG00000105613	MAST1	19:12873816-12985765	q1	q2	OK	11.4596	11.4654	0.000724437	0.00035044	0.9993	0.99995	no

ENSG00000105617	ENSG00000105617	LENG1	19:54641443-54663620	q1	q2	NOTEST	0	0.146911	inf	0	1	1	no

ENSG00000105618	ENSG00000105618	PRPF31	19:54606035-54635140	q1	q2	NOTEST	0	0	0	0	1	1	no

ENSG00000105619	ENSG00000105619	TFPT	19:54606035-54635140	q1	q2	NOTEST	0	0	0	0	1	1	no

ENSG00000105639	ENSG00000105639	JAK3	19:17935588-17958880	q1	q2	NOTEST	0.179317	0.111912	-0.680145	0	1	1	no

ENSG00000105640	ENSG00000105640	RPL18A	19:17970684-17974962	q1	q2	OK	672.878	615.945	-0.127543	-0.308261	0.6571	0.99995	no

ENSG00000105641	ENSG00000105641	SLC5A5	19:17982781-18005983	q1	q2	NOTEST	0.00702873	0	#NAME?	0	1	1	no

ENSG00000105642	ENSG00000105642	KCNN1	19:18062101-18110889	q1	q2	OK	2.64276	1.7293	-0.611858	-0.557041	0.2355	0.99995	no

ENSG00000105643	ENSG00000105643	ARRDC2	19:18111940-18124911	q1	q2	NOTEST	0.467483	0.432085	-0.113599	0	1	1	no

ENSG00000105647	ENSG00000105647	PIK3R2	19:18263927-18288927	q1	q2	OK	21.9112	19.009	-0.204987	-0.339547	0.48915	0.99995	no

ENSG00000105649	ENSG00000105649	RAB3A	19:18303991-18314884	q1	q2	OK	5.06857	3.90313	-0.376945	-0.348665	0.59405	0.99995	no

ENSG00000105650	ENSG00000105650	PDE4C	19:18315539-18366229	q1	q2	NOTEST	0.316686	0.0726055	-2.1249	0	1	1	no

ENSG00000105655	ENSG00000105655	ISYNA1	19:18529673-18549111	q1	q2	OK	120.431	101.383	-0.24839	-0.429079	0.50565	0.99995	no

ENSG00000105656	ENSG00000105656	ELL	19:18552228-18632937	q1	q2	OK	3.74294	3.06782	-0.286957	-0.45917	0.4624	0.99995	no

ENSG00000105662	ENSG00000105662	CRTC1	19:18794486-18893004	q1	q2	OK	12.0248	9.43241	-0.350312	-0.633076	0.36665	0.99995	no

ENSG00000105663	ENSG00000105663	KMT2B	19:36208920-36229779	q1	q2	OK	6.21027	7.67192	0.304933	0.167255	0.83675	0.99995	no

ENSG00000105664	ENSG00000105664	COMP	19:18893582-18902123	q1	q2	NOTEST	0.0233008	0	#NAME?	0	1	1	no

ENSG00000105668	ENSG00000105668	UPK1A	19:36157714-36169367	q1	q2	NOTEST	0.072165	0.0722127	0.000952689	0	1	1	no

ENSG00000105669	ENSG00000105669	COPE	19:19010322-19057640	q1	q2	OK	80.8025	72.2064	-0.162273	-0.218125	0.73715	0.99995	no

ENSG00000105671	ENSG00000105671	DDX49	19:19010322-19057640	q1	q2	OK	27.0879	22.2088	-0.286514	-0.223311	0.6733	0.99995	no

ENSG00000105672	ENSG00000105672	ETV2	19:36132646-36135773	q1	q2	OK	1.01458	0.569767	-0.832438	-0.593661	0.3759	0.99995	no

ENSG00000105675	ENSG00000105675	ATP4A	19:36040944-36054560	q1	q2	NOTEST	0.0146183	0.00703431	-1.0553	0	1	1	no

ENSG00000105676	ENSG00000105676	ARMC6	19:19101696-19170563	q1	q2	OK	17.7482	15.9526	-0.153876	-0.104649	0.8643	0.99995	no

ENSG00000105677	ENSG00000105677	TMEM147	19:36024313-36038428	q1	q2	OK	50.6002	44.8824	-0.172992	-0.31795	0.6454	0.99995	no

ENSG00000105679	ENSG00000105679	GAPDHS	19:36024313-36038428	q1	q2	NOTEST	0	0	0	0	1	1	no

ENSG00000105694	ENSG00000105694	TCEB1P28	19:2511216-2702707	q1	q2	NOTEST	0	0	0	0	1	1	no

ENSG00000105695	ENSG00000105695	MAG	19:35783027-35804707	q1	q2	NOTEST	0	0	0	0	1	1	no

ENSG00000105696	ENSG00000105696	TMEM59L	19:18682539-18731849	q1	q2	OK	6.53574	5.37358	-0.282467	-0.125295	0.8566	0.99995	no

ENSG00000105697	ENSG00000105697	HAMP	19:35771618-35776046	q1	q2	NOTEST	0.00978935	0.0655482	2.74327	0	1	1	no

ENSG00000105698	ENSG00000105698	USF2	19:35759880-35770724	q1	q2	OK	53.2619	48.0285	-0.149213	-0.322487	0.61365	0.99995	no

ENSG00000105699	ENSG00000105699	LSR	19:35739232-35758867	q1	q2	OK	0.881648	0.770819	-0.193811	-0.100756	0.80625	0.99995	no

ENSG00000105700	ENSG00000105700	KXD1	19:18668571-18680197	q1	q2	OK	46.7904	45.3805	-0.0441396	-0.0947475	0.8896	0.99995	no

ENSG00000105701	ENSG00000105701	FKBP8	19:18642422-18654887	q1	q2	OK	87.7412	79.6971	-0.138727	-0.321675	0.6234	0.99995	no

ENSG00000105705	ENSG00000105705	SUGP1	19:19386826-19469563	q1	q2	OK	34.2286	28.0837	-0.285468	-0.312967	0.5819	0.99995	no

ENSG00000105707	ENSG00000105707	HPN	19:35531409-35598141	q1	q2	NOTEST	0	0.0913232	inf	0	1	1	no

ENSG00000105708	ENSG00000105708	ZNF14	19:19821279-19843906	q1	q2	OK	4.76237	4.7744	0.00363849	0.00624616	0.99295	0.99995	no

ENSG00000105711	ENSG00000105711	SCN1B	19:35521587-35531352	q1	q2	OK	0.478529	0.678328	0.503378	0.383881	0.51955	0.99995	no

ENSG00000105717	ENSG00000105717	PBX4	19:19672521-19729725	q1	q2	OK	5.88947	4.81432	-0.290806	-0.333177	0.65795	0.99995	no

ENSG00000105722	ENSG00000105722	ERF	19:42734337-42759309	q1	q2	OK	28.0485	25.4934	-0.137796	-0.165015	0.74795	0.99995	no

ENSG00000105723	ENSG00000105723	GSK3A	19:42734337-42759309	q1	q2	OK	36.2773	32.4932	-0.158932	-0.262725	0.6929	0.99995	no

ENSG00000105726	ENSG00000105726	ATP13A1	19:19756006-19774502	q1	q2	OK	20.6853	16.2811	-0.345405	-0.540575	0.3247	0.99995	no

ENSG00000105732	ENSG00000105732	ZNF574	19:42502472-42585701	q1	q2	OK	8.30886	7.74217	-0.101914	-0.18597	0.78685	0.99995	no

ENSG00000105737	ENSG00000105737	GRIK5	19:42502472-42585701	q1	q2	OK	1.37218	1.05958	-0.372984	-0.261091	0.73125	0.99995	no

ENSG00000105738	ENSG00000105738	SIPA1L3	19:38397867-38699012	q1	q2	OK	7.03816	7.8541	0.158248	0.133176	0.6859	0.99995	no

ENSG00000105750	ENSG00000105750	ZNF85	19:21106027-21133503	q1	q2	NOTEST	0.0887388	0.0832094	-0.0928185	0	1	1	no

ENSG00000105755	ENSG00000105755	ETHE1	19:44010870-44040282	q1	q2	OK	9.09462	8.53726	-0.0912399	-0.125135	0.861	0.99995	no

ENSG00000105767	ENSG00000105767	CADM4	19:44126519-44143991	q1	q2	OK	77.936	69.4088	-0.167171	-0.36981	0.5426	0.99995	no

ENSG00000105771	ENSG00000105771	SMG9	19:44235300-44259142	q1	q2	OK	35.8496	32.868	-0.125274	-0.195843	0.7654	0.99995	no

ENSG00000105778	ENSG00000105778	AVL9	7:32535037-33102409	q1	q2	OK	11.7282	14.1829	0.27416	0.179171	0.64495	0.99995	no

ENSG00000105784	ENSG00000105784	RUNDC3B	7:87133174-87461611	q1	q2	OK	0.876161	0.920522	0.0712564	0.0428922	0.91635	0.99995	no

ENSG00000105792	ENSG00000105792	C7orf63	7:89874487-89940377	q1	q2	OK	1.87838	1.40502	-0.418894	-0.274979	0.53665	0.99995	no

ENSG00000105793	ENSG00000105793	GTPBP10	7:89964536-90839905	q1	q2	OK	8.28235	9.86232	0.251888	0.136083	0.7494	0.99995	no

ENSG00000105808	ENSG00000105808	RASA4	7:102122891-102312088	q1	q2	OK	4.78452	6.47048	0.435497	0.231031	0.6479	0.99995	no

ENSG00000105810	ENSG00000105810	CDK6	7:92234234-92546501	q1	q2	OK	24.0718	27.1056	0.171247	0.326957	0.5534	0.99995	no

ENSG00000105819	ENSG00000105819	PMPCB	7:102937868-103629963	q1	q2	OK	55.9584	55.7523	-0.00532278	-0.00735788	0.9923	0.99995	no

ENSG00000105821	ENSG00000105821	DNAJC2	7:102937868-103629963	q1	q2	OK	18.5259	19.8819	0.101916	0.0838758	0.88515	0.99995	no

ENSG00000105825	ENSG00000105825	TFPI2	7:93220884-93540577	q1	q2	OK	6.45099	11.2885	0.807257	1.00806	0.13495	0.99995	no

ENSG00000105829	ENSG00000105829	BET1	7:93592073-93640425	q1	q2	OK	20.9248	20.9066	-0.00125089	-0.00230457	0.99735	0.99995	no

ENSG00000105835	ENSG00000105835	NAMPT	7:105888730-105926772	q1	q2	OK	11.5796	13.2899	0.198744	0.320133	0.6089	0.99995	no

ENSG00000105849	ENSG00000105849	TWISTNB	7:19735084-19748710	q1	q2	OK	8.67397	10.2251	0.237355	0.466125	0.4635	0.99995	no

ENSG00000105851	ENSG00000105851	PIK3CG	7:106505722-106547590	q1	q2	NOTEST	0.00500314	0.00680365	0.443477	0	1	1	no

ENSG00000105852	ENSG00000105852	PON3	7:94926987-95025680	q1	q2	NOTEST	0.0448211	0	#NAME?	0	1	1	no

ENSG00000105854	ENSG00000105854	PON2	7:95034174-95064510	q1	q2	OK	110.948	128.17	0.208175	0.427153	0.50745	0.99995	no

ENSG00000105855	ENSG00000105855	ITGB8	7:20367921-20455377	q1	q2	OK	16.7429	16.0303	-0.0627401	-0.140968	0.82955	0.99995	no

ENSG00000105856	ENSG00000105856	HBP1	7:106809405-107218906	q1	q2	OK	25.6311	21.347	-0.263867	-0.363462	0.5066	0.99995	no

ENSG00000105865	ENSG00000105865	DUS4L	7:106809405-107218906	q1	q2	OK	7.80047	8.30287	0.0900507	0.0624063	0.8992	0.99995	no

ENSG00000105866	ENSG00000105866	SP4	7:21467651-21554440	q1	q2	OK	6.83946	7.36986	0.107754	0.218336	0.74855	0.99995	no

ENSG00000105875	ENSG00000105875	WDR91	7:134868589-134896316	q1	q2	OK	5.61069	6.5163	0.215873	0.213066	0.63185	0.99995	no

ENSG00000105877	ENSG00000105877	DNAH11	7:21582832-21985702	q1	q2	OK	0.781753	1.1518	0.5591	0.140281	0.6138	0.99995	no

ENSG00000105879	ENSG00000105879	CBLL1	7:107383261-107402112	q1	q2	OK	15.6658	17.2654	0.140267	0.218484	0.68395	0.99995	no

ENSG00000105880	ENSG00000105880	DLX5	7:96649703-96654409	q1	q2	NOTEST	0	0.0865906	inf	0	1	1	no

ENSG00000105887	ENSG00000105887	MTPN	7:135611202-135662101	q1	q2	OK	64.5312	73.6278	0.190255	0.362762	0.57455	0.99995	no

ENSG00000105889	ENSG00000105889	STEAP1B	7:22459062-22705152	q1	q2	OK	1.41619	1.43013	0.0141271	0.00462666	0.9936	0.99995	no

ENSG00000105894	ENSG00000105894	PTN	7:136912087-137028611	q1	q2	OK	227.327	216.762	-0.0686614	-0.146888	0.83445	0.99995	no

ENSG00000105926	ENSG00000105926	MPP6	7:24612886-24733812	q1	q2	OK	9.46113	10.0691	0.0898484	0.122075	0.8278	0.99995	no

ENSG00000105928	ENSG00000105928	DFNA5	7:24737971-24809244	q1	q2	OK	15.864	13.8178	-0.199229	-0.323499	0.5638	0.99995	no

ENSG00000105929	ENSG00000105929	ATP6V0A4	7:138391039-138666064	q1	q2	NOTEST	0.0423067	0.0238584	-0.82639	0	1	1	no

ENSG00000105939	ENSG00000105939	ZC3HAV1	7:138728265-138794465	q1	q2	OK	3.17613	3.11669	-0.0272545	-0.047387	0.94305	0.99995	no

ENSG00000105948	ENSG00000105948	TTC26	7:138818489-138876732	q1	q2	OK	5.56855	6.19876	0.154678	0.244338	0.68995	0.99995	no

ENSG00000105953	ENSG00000105953	OGDH	7:44646170-44748665	q1	q2	OK	20.2567	20.4559	0.0141169	0.030092	0.9624	0.99995	no

ENSG00000105954	ENSG00000105954	NPVF	7:25264188-25268105	q1	q2	NOTEST	0	0	0	0	1	1	no

ENSG00000105963	ENSG00000105963	ADAP1	7:937539-1015235	q1	q2	OK	1.86327	1.55487	-0.261043	-0.131461	0.7326	0.99995	no

ENSG00000105967	ENSG00000105967	TFEC	7:115575201-115799950	q1	q2	NOTEST	0.00413525	0.0261974	2.66338	0	1	1	no

ENSG00000105968	ENSG00000105968	H2AFV	7:44866389-44887682	q1	q2	OK	233.194	230.363	-0.0176241	-0.0307703	0.96815	0.99995	no

ENSG00000105971	ENSG00000105971	CAV2	7:115850546-116148595	q1	q2	OK	1.10961	1.07365	-0.0475403	-0.012518	0.9612	0.99995	no

ENSG00000105974	ENSG00000105974	CAV1	7:116164838-116201233	q1	q2	OK	3.19894	2.81525	-0.184327	-0.197969	0.754	0.99995	no

ENSG00000105976	ENSG00000105976	MET	7:116312443-116438440	q1	q2	NOTEST	0.196431	0.0956051	-1.03887	0	1	1	no

ENSG00000105982	ENSG00000105982	RNF32	7:156264889-156685924	q1	q2	OK	1.18904	1.4046	0.240359	0.0557996	0.7615	0.99995	no

ENSG00000105983	ENSG00000105983	LMBR1	7:156264889-156685924	q1	q2	OK	23.3675	24.8319	0.0876895	0.146169	0.77485	0.99995	no

ENSG00000105988	ENSG00000105988	NHP2P1	10:93806448-94050844	q1	q2	OK	0.180718	0.809531	2.16335	0.55712	0.48535	0.99995	no

ENSG00000105989	ENSG00000105989	WNT2	7:116916684-116963343	q1	q2	OK	0.914587	0.604391	-0.59764	-0.519036	0.4118	0.99995	no

ENSG00000105991	ENSG00000105991	HOXA1	7:27132611-27142430	q1	q2	OK	1.20474	1.34633	0.160305	0.0832985	0.93405	0.99995	no

ENSG00000105993	ENSG00000105993	DNAJB6	7:157128074-157210133	q1	q2	OK	81.4019	76.9337	-0.0814468	-0.171985	0.78	0.99995	no

ENSG00000105996	ENSG00000105996	HOXA2	7:27132611-27142430	q1	q2	OK	17.6747	16.4524	-0.103388	-0.187057	0.7889	0.99995	no

ENSG00000105997	ENSG00000105997	HOXA3	7:27145802-27197555	q1	q2	OK	0.681201	0.64101	-0.0877323	-0.0692071	0.87875	0.99995	no

ENSG00000106003	ENSG00000106003	LFNG	7:2552162-2568811	q1	q2	OK	25.1546	22.1129	-0.185935	-0.386759	0.5827	0.99995	no

ENSG00000106004	ENSG00000106004	HOXA5	7:27145802-27197555	q1	q2	NOTEST	0.0325741	0.159897	2.29535	0	1	1	no

ENSG00000106006	ENSG00000106006	HOXA6	7:27145802-27197555	q1	q2	NOTEST	0.0635275	0	#NAME?	0	1	1	no

ENSG00000106009	ENSG00000106009	BRAT1	7:2577510-2595361	q1	q2	OK	16.7254	13.4073	-0.319022	-0.596547	0.34485	0.99995	no

ENSG00000106012	ENSG00000106012	IQCE	7:2598631-2654368	q1	q2	OK	6.21042	5.65497	-0.135171	-0.205931	0.67695	0.99995	no

ENSG00000106013	ENSG00000106013	ANKRD7	7:117854726-117882785	q1	q2	NOTEST	0.043628	0.0272566	-0.67865	0	1	1	no

ENSG00000106018	ENSG00000106018	VIPR2	7:158799212-158937649	q1	q2	OK	0.740078	0.745021	0.00960459	0.0100913	0.9879	0.99995	no

ENSG00000106025	ENSG00000106025	TSPAN12	7:120427375-120498456	q1	q2	OK	15.0139	16.6974	0.153324	0.29958	0.6329	0.99995	no

ENSG00000106028	ENSG00000106028	SSBP1	7:141404137-141488440	q1	q2	OK	37.3364	42.605	0.19044	0.341152	0.6199	0.99995	no

ENSG00000106031	ENSG00000106031	HOXA13	7:27233121-27246878	q1	q2	NOTEST	0.0457641	0.073507	0.683664	0	1	1	no

ENSG00000106034	ENSG00000106034	CPED1	7:120628730-120937498	q1	q2	OK	1.16448	1.65619	0.508183	0.421666	0.38655	0.99995	no

ENSG00000106038	ENSG00000106038	EVX1	7:27281047-27290112	q1	q2	NOTEST	0	0	0	0	1	1	no

ENSG00000106049	ENSG00000106049	HIBADH	7:27565060-27702614	q1	q2	OK	15.866	15.8709	0.000445809	0.000876355	0.999	0.99995	no

ENSG00000106052	ENSG00000106052	TAX1BP1	7:27773013-28283536	q1	q2	OK	62.5602	65.2267	0.0602171	0.129719	0.84315	0.99995	no

ENSG00000106066	ENSG00000106066	CPVL	7:29019582-29603286	q1	q2	OK	2.77575	3.23166	0.2194	0.151697	0.791	0.99995	no

ENSG00000106069	ENSG00000106069	CHN2	7:29019582-29603286	q1	q2	OK	11.5232	11.2897	-0.0295343	-0.0437128	0.92945	0.99995	no

ENSG00000106070	ENSG00000106070	GRB10	7:50657759-50861159	q1	q2	OK	29.2292	33.9889	0.217653	0.368803	0.4361	0.99995	no

ENSG00000106077	ENSG00000106077	ABHD11	7:73150423-73153197	q1	q2	NOTEST	0.265614	0.436692	0.717285	0	1	1	no

ENSG00000106078	ENSG00000106078	COBL	7:50934443-51384515	q1	q2	NOTEST	0.131184	0.0806468	-0.701903	0	1	1	no

ENSG00000106080	ENSG00000106080	FKBP14	7:29959718-30066300	q1	q2	OK	9.17666	10.59	0.206658	0.135244	0.79625	0.99995	no

ENSG00000106086	ENSG00000106086	PLEKHA8	7:30067019-30170096	q1	q2	OK	20.9537	23.0009	0.134485	0.276593	0.66145	0.99995	no

ENSG00000106089	ENSG00000106089	STX1A	7:73095298-73134002	q1	q2	OK	1.16196	1.49062	0.359359	0.159974	0.76275	0.99995	no

ENSG00000106100	ENSG00000106100	NOD1	7:30464142-30518400	q1	q2	OK	2.86502	2.47412	-0.211629	-0.204846	0.73845	0.99995	no

ENSG00000106105	ENSG00000106105	GARS	7:30536236-30673649	q1	q2	OK	100.019	95.9291	-0.0602291	-0.0901993	0.8636	0.99995	no

ENSG00000106113	ENSG00000106113	CRHR2	7:30692199-30965131	q1	q2	NOTEST	0.0855291	0.0296144	-1.53011	0	1	1	no

ENSG00000106123	ENSG00000106123	EPHB6	7:142552791-142568847	q1	q2	OK	0.794831	0.645076	-0.301178	-0.238942	0.6576	0.99995	no

ENSG00000106125	ENSG00000106125	FAM188B	7:30692199-30965131	q1	q2	OK	1.92664	2.16118	0.165728	0.207574	0.77105	0.99995	no

ENSG00000106128	ENSG00000106128	GHRHR	7:30978283-31032869	q1	q2	NOTEST	0.0293404	0.589448	4.32841	0	1	1	no

ENSG00000106133	ENSG00000106133	NSUN5P2	7:72349935-72425329	q1	q2	NOTEST	0.0678626	0.0862586	0.346052	0	1	1	no

ENSG00000106144	ENSG00000106144	CASP2	7:142985307-143004789	q1	q2	OK	22.1691	23.3997	0.0779406	0.175982	0.79195	0.99995	no

ENSG00000106153	ENSG00000106153	CHCHD2	7:56169261-56174269	q1	q2	OK	17.5895	16.4203	-0.0992325	-0.15969	0.8167	0.99995	no

ENSG00000106178	ENSG00000106178	CCL24	7:75440982-75452674	q1	q2	NOTEST	0	0	0	0	1	1	no

ENSG00000106211	ENSG00000106211	HSPB1	7:75931860-75933612	q1	q2	OK	55.9389	46.3815	-0.270304	-0.537073	0.44515	0.99995	no

ENSG00000106236	ENSG00000106236	NPTX2	7:98246608-98259180	q1	q2	OK	33.374	34.3744	0.0426084	0.0977647	0.88205	0.99995	no

ENSG00000106244	ENSG00000106244	PDAP1	7:98923520-99063954	q1	q2	OK	111.159	111.15	-0.000116544	-0.000115582	0.9998	0.99995	no

ENSG00000106245	ENSG00000106245	BUD31	7:98923520-99063954	q1	q2	OK	79.9485	80.0252	0.00138291	0.00112708	0.99825	0.99995	no

ENSG00000106246	ENSG00000106246	PTCD1	7:98923520-99063954	q1	q2	OK	4.11472	2.89671	-0.50638	-0.165702	0.7628	0.99995	no

ENSG00000106258	ENSG00000106258	CYP3A5	7:99245816-99277621	q1	q2	OK	0.405875	0.798403	0.97608	0.721927	0.1291	0.99995	no

ENSG00000106261	ENSG00000106261	ZKSCAN1	7:99613203-99639312	q1	q2	OK	29.9615	29.5614	-0.0193928	-0.0420186	0.9462	0.99995	no

ENSG00000106263	ENSG00000106263	EIF3B	7:2291404-2420380	q1	q2	OK	72.3061	73.1905	0.017539	0.0310454	0.95715	0.99995	no

ENSG00000106266	ENSG00000106266	SNX8	7:2291404-2420380	q1	q2	OK	7.0771	6.50554	-0.121491	-0.0624057	0.8569	0.99995	no

ENSG00000106268	ENSG00000106268	NUDT1	7:2281856-2290781	q1	q2	OK	47.4211	44.0806	-0.105385	-0.201906	0.7776	0.99995	no

ENSG00000106278	ENSG00000106278	PTPRZ1	7:121513142-121702090	q1	q2	OK	183.054	203.416	0.152165	0.157103	0.69605	0.99995	no

ENSG00000106290	ENSG00000106290	TAF6	7:99690350-99726118	q1	q2	OK	36.1365	32.7279	-0.142933	-0.084113	0.8345	0.99995	no

ENSG00000106299	ENSG00000106299	WASL	7:123321988-123389121	q1	q2	OK	18.7768	20.3906	0.118953	0.287032	0.6782	0.99995	no

ENSG00000106302	ENSG00000106302	HYAL4	7:123454192-123517532	q1	q2	NOTEST	0	0.0326754	inf	0	1	1	no

ENSG00000106304	ENSG00000106304	SPAM1	7:123565285-123611468	q1	q2	NOTEST	0	0.0438503	inf	0	1	1	no

ENSG00000106305	ENSG00000106305	AIMP2	7:6048875-6098861	q1	q2	OK	23.8629	23.6905	-0.0104615	-0.00920057	0.98975	0.99995	no

ENSG00000106327	ENSG00000106327	TFR2	7:100218038-100240402	q1	q2	OK	0.624802	0.676489	0.114668	0.0852812	0.8691	0.99995	no

ENSG00000106328	ENSG00000106328	FSCN3	7:127220671-127242198	q1	q2	OK	0.726795	0.602259	-0.271165	-0.0347494	0.87015	0.99995	no

ENSG00000106330	ENSG00000106330	MOSPD3	7:100209724-100213007	q1	q2	OK	19.6614	17.738	-0.148525	-0.259597	0.7124	0.99995	no

ENSG00000106331	ENSG00000106331	PAX4	7:127250345-127255982	q1	q2	NOTEST	0	0	0	0	1	1	no

ENSG00000106333	ENSG00000106333	PCOLCE	7:100169854-100205798	q1	q2	OK	32.3456	26.0029	-0.314897	-0.306408	0.6716	0.99995	no

ENSG00000106336	ENSG00000106336	FBXO24	7:100169854-100205798	q1	q2	NOTEST	0.433394	0.371798	-0.221161	0	1	1	no

ENSG00000106341	ENSG00000106341	PPP1R17	7:31726328-31748069	q1	q2	OK	1.86399	1.83231	-0.0247305	-0.0253401	0.96615	0.99995	no

ENSG00000106344	ENSG00000106344	RBM28	7:127937737-127983962	q1	q2	OK	29.3731	32.5057	0.146196	0.194152	0.76905	0.99995	no

ENSG00000106346	ENSG00000106346	USP42	7:6144514-6201195	q1	q2	OK	10.109	10.4507	0.0479562	0.107357	0.8741	0.99995	no

ENSG00000106348	ENSG00000106348	IMPDH1	7:128032330-128050306	q1	q2	OK	12.6858	10.5269	-0.269132	-0.43742	0.465	0.99995	no

ENSG00000106351	ENSG00000106351	AGFG2	7:100136833-100165842	q1	q2	OK	4.42687	3.65258	-0.27737	-0.371089	0.5847	0.99995	no

ENSG00000106355	ENSG00000106355	LSM5	7:32524950-32534895	q1	q2	OK	88.5536	92.899	0.0691124	0.128206	0.85905	0.99995	no

ENSG00000106366	ENSG00000106366	SERPINE1	7:100770369-100782547	q1	q2	OK	1.60485	1.31774	-0.28437	-0.363413	0.5948	0.99995	no

ENSG00000106367	ENSG00000106367	AP1S1	7:100797677-100804877	q1	q2	OK	69.2405	62.2757	-0.152947	-0.33804	0.62685	0.99995	no

ENSG00000106384	ENSG00000106384	MOGAT3	7:100838287-100844302	q1	q2	NOTEST	0	0	0	0	1	1	no

ENSG00000106392	ENSG00000106392	C1GALT1	7:7196564-7288282	q1	q2	OK	9.00052	9.27712	0.0436675	0.0535544	0.93375	0.99995	no

ENSG00000106397	ENSG00000106397	PLOD3	7:100849257-100867471	q1	q2	OK	25.2831	25.482	0.011306	0.012153	0.9806	0.99995	no

ENSG00000106399	ENSG00000106399	RPA3	7:7676148-8133902	q1	q2	OK	39.6345	46.1445	0.219401	0.213269	0.75375	0.99995	no

ENSG00000106400	ENSG00000106400	ZNHIT1	7:100849257-100867471	q1	q2	OK	34.6801	32.2099	-0.106602	-0.16193	0.80615	0.99995	no

ENSG00000106404	ENSG00000106404	CLDN15	7:100875372-100882101	q1	q2	OK	6.4145	5.27766	-0.281439	-0.402253	0.5526	0.99995	no

ENSG00000106410	ENSG00000106410	NOBOX	7:144094332-144107320	q1	q2	NOTEST	0	0	0	0	1	1	no

ENSG00000106415	ENSG00000106415	GLCCI1	7:7676148-8133902	q1	q2	OK	22.7326	23.916	0.0732162	0.07925	0.86025	0.99995	no

ENSG00000106436	ENSG00000106436	MYL10	7:101256604-101272576	q1	q2	NOTEST	0	0	0	0	1	1	no

ENSG00000106443	ENSG00000106443	PHF14	7:11013498-11209250	q1	q2	OK	39.6934	42.9918	0.115162	0.239061	0.6894	0.99995	no

ENSG00000106459	ENSG00000106459	NRF1	7:129251554-129396922	q1	q2	OK	14.6448	14.3688	-0.0274502	-0.0426815	0.93895	0.99995	no

ENSG00000106460	ENSG00000106460	TMEM106B	7:12250866-12282993	q1	q2	OK	16.6103	17.5434	0.0788537	0.104201	0.8478	0.99995	no

ENSG00000106462	ENSG00000106462	EZH2	7:148504474-148581413	q1	q2	OK	70.543	72.6552	0.0425631	0.0871846	0.88115	0.99995	no

ENSG00000106477	ENSG00000106477	CEP41	7:130033611-130082274	q1	q2	OK	8.32727	0.35689	-4.54429	-2.36981	5.00E-05	0.0795045	no

ENSG00000106479	ENSG00000106479	ZNF862	7:149535455-149564568	q1	q2	OK	7.39784	5.881	-0.331043	-0.340334	0.46445	0.99995	no

ENSG00000106483	ENSG00000106483	SFRP4	7:37723398-38065297	q1	q2	NOTEST	0.436349	0.500143	0.196858	0	1	1	no

ENSG00000106484	ENSG00000106484	MEST	7:130125882-130148500	q1	q2	OK	6.52384	4.08312	-0.676051	-0.694241	0.2408	0.99995	no

ENSG00000106511	ENSG00000106511	MEOX2	7:15650836-15726437	q1	q2	OK	17.6478	17.9859	0.0273737	0.0509249	0.9407	0.99995	no

ENSG00000106524	ENSG00000106524	ANKMY2	7:16639400-16685442	q1	q2	OK	13.8962	14.1741	0.0285734	0.0604302	0.93095	0.99995	no

ENSG00000106526	ENSG00000106526	ACTR3C	7:149941004-150035239	q1	q2	NOTEST	0.0525165	0.0922976	0.813522	0	1	1	no

ENSG00000106536	ENSG00000106536	POU6F2	7:39017597-39532694	q1	q2	NOTEST	0.313841	0.258908	-0.277595	0	1	1	no

ENSG00000106537	ENSG00000106537	TSPAN13	7:16793159-16824161	q1	q2	OK	50.0389	51.7235	0.0477696	0.116536	0.86495	0.99995	no

ENSG00000106538	ENSG00000106538	RARRES2	7:150035407-150038763	q1	q2	OK	5.21667	4.82094	-0.113815	-0.127708	0.8574	0.99995	no

ENSG00000106540	ENSG00000106540	AC004837.3	7:39605974-39649919	q1	q2	NOTEST	0	0.081828	inf	0	1	1	no

ENSG00000106541	ENSG00000106541	AGR2	7:16831434-16873057	q1	q2	NOTEST	0	0	0	0	1	1	no

ENSG00000106546	ENSG00000106546	AHR	7:17319457-17385776	q1	q2	OK	2.51456	3.20003	0.347781	0.31725	0.53755	0.99995	no

ENSG00000106554	ENSG00000106554	CHCHD3	7:132469628-132766848	q1	q2	OK	93.86	96.0879	0.0338443	0.0647338	0.9295	0.99995	no

ENSG00000106560	ENSG00000106560	GIMAP2	7:150382784-150390729	q1	q2	NOTEST	0.0569991	0.0751558	0.398945	0	1	1	no

ENSG00000106565	ENSG00000106565	TMEM176B	7:150488372-150502208	q1	q2	NOTEST	0.0539304	0.118085	1.13065	0	1	1	no

ENSG00000106571	ENSG00000106571	GLI3	7:42000547-42277469	q1	q2	OK	24.5796	25.5868	0.0579381	0.0828698	0.83835	0.99995	no

ENSG00000106588	ENSG00000106588	PSMA2	7:42948324-42988557	q1	q2	OK	118.511	128.307	0.114575	0.194305	0.7827	0.99995	no

ENSG00000106591	ENSG00000106591	MRPL32	7:42948324-42988557	q1	q2	OK	37.8655	39.6911	0.0679326	0.0733629	0.91745	0.99995	no

ENSG00000106603	ENSG00000106603	COA1	7:43622356-43769316	q1	q2	OK	69.2793	74.0537	0.0961468	0.158621	0.8115	0.99995	no

ENSG00000106605	ENSG00000106605	BLVRA	7:43798278-43846939	q1	q2	OK	19.5267	18.3453	-0.090034	-0.158967	0.81825	0.99995	no

ENSG00000106608	ENSG00000106608	URGCP	7:43906156-43966010	q1	q2	OK	10.3242	9.66306	-0.0954711	-0.0943924	0.8213	0.99995	no

ENSG00000106609	ENSG00000106609	TMEM248	7:66386211-66423538	q1	q2	OK	26.1018	27.1809	0.0584399	0.120138	0.8335	0.99995	no

ENSG00000106610	ENSG00000106610	STAG3L4	7:66752527-66786513	q1	q2	OK	6.71161	5.75043	-0.22299	-0.277568	0.66285	0.99995	no

ENSG00000106615	ENSG00000106615	RHEB	7:151163097-151217206	q1	q2	OK	57.7317	57.5511	-0.00451991	-0.00920401	0.989	0.99995	no

ENSG00000106617	ENSG00000106617	PRKAG2	7:151253196-151576299	q1	q2	OK	5.59929	5.73586	0.0347652	0.0393417	0.937	0.99995	no

ENSG00000106624	ENSG00000106624	AEBP1	7:44143959-44154161	q1	q2	OK	30.7383	28.1518	-0.126811	-0.224578	0.67595	0.99995	no

ENSG00000106628	ENSG00000106628	POLD2	7:44154285-44163957	q1	q2	OK	70.4423	64.0017	-0.138331	-0.254884	0.70235	0.99995	no

ENSG00000106631	ENSG00000106631	MYL7	7:44178462-44180931	q1	q2	NOTEST	0	0.12727	inf	0	1	1	no

ENSG00000106633	ENSG00000106633	GCK	7:44183871-44237769	q1	q2	OK	0.669988	0.550817	-0.282563	-0.22877	0.68675	0.99995	no

ENSG00000106635	ENSG00000106635	BCL7B	7:72950685-72972332	q1	q2	OK	1.1743	5.21362	2.15048	1.87606	0.0016	0.87455	no

ENSG00000106636	ENSG00000106636	YKT6	7:44240566-44253893	q1	q2	OK	73.5026	70.5002	-0.0601668	-0.114375	0.8387	0.99995	no

ENSG00000106638	ENSG00000106638	TBL2	7:72983261-72993121	q1	q2	OK	2.2795	0.435972	-2.38641	-1.52984	0.00245	0.99995	no

ENSG00000106648	ENSG00000106648	GALNTL5	7:151653463-151717019	q1	q2	NOTEST	0.0422309	0.0201825	-1.06519	0	1	1	no

ENSG00000106665	ENSG00000106665	CLIP2	7:73703804-73820273	q1	q2	OK	1.25222	1.90374	0.604342	0.509265	0.28395	0.99995	no

ENSG00000106682	ENSG00000106682	EIF4H	7:73588574-73611431	q1	q2	OK	13.7344	2.58969	-2.40694	-2.67809	0.0001	0.134546	no

ENSG00000106683	ENSG00000106683	LIMK1	7:73497262-73536855	q1	q2	OK	4.68535	1.29619	-1.85388	-2.14861	0.00365	0.99995	no

ENSG00000106686	ENSG00000106686	SPATA6L	9:4490443-4666674	q1	q2	OK	0.243436	0.492378	1.01622	0.225588	0.2303	0.99995	no

ENSG00000106688	ENSG00000106688	SLC1A1	9:4490443-4666674	q1	q2	NOTEST	0.235934	0.24697	0.0659553	0	1	1	no

ENSG00000106689	ENSG00000106689	LHX2	9:126763948-126795580	q1	q2	OK	0.553589	0.614145	0.149764	0.0854766	0.87925	0.99995	no

ENSG00000106692	ENSG00000106692	FKTN	9:108320410-108403399	q1	q2	OK	7.44409	7.31111	-0.0260068	-0.0230237	0.94055	0.99995	no

ENSG00000106701	ENSG00000106701	FSD1L	9:108210076-108314714	q1	q2	OK	7.66326	8.98994	0.230354	0.287431	0.51385	0.99995	no

ENSG00000106714	ENSG00000106714	CNTNAP3	9:39072763-39288312	q1	q2	OK	2.19344	2.41371	0.138057	0.142688	0.7761	0.99995	no

ENSG00000106723	ENSG00000106723	SPIN1	9:91003333-91093609	q1	q2	OK	49.183	53.2987	0.11594	0.229612	0.682	0.99995	no

ENSG00000106733	ENSG00000106733	NMRK1	9:77675488-77703133	q1	q2	OK	0.815349	0.630676	-0.370518	-0.300313	0.6355	0.99995	no

ENSG00000106771	ENSG00000106771	TMEM245	9:111777431-111882225	q1	q2	OK	19.5718	20.2806	0.0513254	0.106557	0.87535	0.99995	no

ENSG00000106772	ENSG00000106772	PRUNE2	9:79226291-79521003	q1	q2	OK	1.40349	1.75126	0.319375	0.242786	0.58385	0.99995	no

ENSG00000106780	ENSG00000106780	MEGF9	9:123363090-123476748	q1	q2	OK	11.5339	11.9727	0.0538761	0.127859	0.85125	0.99995	no

ENSG00000106785	ENSG00000106785	TRIM14	9:100819020-100881494	q1	q2	NOTEST	0.439793	0.446342	0.0213243	0	1	1	no

ENSG00000106789	ENSG00000106789	CORO2A	9:100883256-100954922	q1	q2	OK	0.59982	0.571733	-0.0691875	-0.0824872	0.9068	0.99995	no

ENSG00000106799	ENSG00000106799	TGFBR1	9:101866319-101916474	q1	q2	OK	65.5879	71.4333	0.123166	0.20151	0.6764	0.99995	no

ENSG00000106803	ENSG00000106803	SEC61B	9:101984345-101992897	q1	q2	OK	155.934	159.938	0.0365794	0.0835788	0.90185	0.99995	no

ENSG00000106804	ENSG00000106804	C5	9:123714615-123812554	q1	q2	OK	2.36244	2.18444	-0.113017	-0.112734	0.79785	0.99995	no

ENSG00000106809	ENSG00000106809	OGN	9:95059639-95432547	q1	q2	OK	5.41743	6.61717	0.288605	0.163323	0.8429	0.99995	no

ENSG00000106819	ENSG00000106819	ASPN	9:95059639-95432547	q1	q2	OK	0.40996	0.512082	0.320891	0.0664705	0.86615	0.99995	no

ENSG00000106823	ENSG00000106823	ECM2	9:95059639-95432547	q1	q2	OK	0.43285	0.588905	0.444167	0.107089	0.75345	0.99995	no

ENSG00000106829	ENSG00000106829	TLE4	9:82186687-82341658	q1	q2	OK	81.4756	77.0237	-0.0810654	-0.123017	0.8329	0.99995	no

ENSG00000106852	ENSG00000106852	LHX6	9:124964855-124991905	q1	q2	NOTEST	0.0177759	0.0689145	1.95489	0	1	1	no

ENSG00000106853	ENSG00000106853	PTGR1	9:114287438-114393569	q1	q2	OK	9.91785	10.0425	0.0180243	0.0236835	0.9747	0.99995	no

ENSG00000106868	ENSG00000106868	SUSD1	9:114803064-114937688	q1	q2	OK	6.07094	6.84244	0.172591	0.263033	0.63905	0.99995	no

ENSG00000106927	ENSG00000106927	AMBP	9:116822406-116840752	q1	q2	NOTEST	0.0433237	0	#NAME?	0	1	1	no

ENSG00000106948	ENSG00000106948	AKNA	9:117096435-117156685	q1	q2	OK	6.8824	6.82445	-0.0121998	-0.0211842	0.96965	0.99995	no

ENSG00000106952	ENSG00000106952	TNFSF8	9:117656002-117692697	q1	q2	NOTEST	0	0	0	0	1	1	no

ENSG00000106976	ENSG00000106976	DNM1	9:130928342-131017527	q1	q2	OK	33.2728	27.7363	-0.262568	-0.232917	0.69535	0.99995	no

ENSG00000106991	ENSG00000106991	ENG	9:130577290-130617035	q1	q2	OK	12.4789	9.87796	-0.337206	-0.533717	0.40625	0.99995	no

ENSG00000106992	ENSG00000106992	AK1	9:130628758-130667687	q1	q2	OK	27.4343	21.483	-0.352785	-0.346375	0.62465	0.99995	no

ENSG00000106993	ENSG00000106993	CDC37L1	9:4679558-4708398	q1	q2	OK	6.55311	6.40451	-0.0330901	-0.0527504	0.94065	0.99995	no

ENSG00000107014	ENSG00000107014	RLN2	9:5299867-5304969	q1	q2	NOTEST	0.0470766	0.140724	1.57979	0	1	1	no

ENSG00000107018	ENSG00000107018	RLN1	9:5334968-5339873	q1	q2	NOTEST	0.175523	0.169628	-0.0492886	0	1	1	no

ENSG00000107020	ENSG00000107020	PLGRKT	9:5357972-5437878	q1	q2	OK	14.6047	14.9492	0.0336334	0.0524347	0.937	0.99995	no

ENSG00000107021	ENSG00000107021	TBC1D13	9:131549482-131572711	q1	q2	OK	16.6102	15.2633	-0.122005	-0.238339	0.6752	0.99995	no

ENSG00000107036	ENSG00000107036	KIAA1432	9:5629024-5833117	q1	q2	OK	13.3437	13.4437	0.010774	0.0160397	0.97805	0.99995	no

ENSG00000107077	ENSG00000107077	KDM4C	9:6716547-7175648	q1	q2	OK	4.44079	4.55335	0.0361101	0.0523426	0.9282	0.99995	no

ENSG00000107099	ENSG00000107099	DOCK8	9:213107-746105	q1	q2	NOTEST	0.233334	0.117863	-0.985285	0	1	1	no

ENSG00000107104	ENSG00000107104	KANK1	9:213107-746105	q1	q2	OK	2.37134	2.31427	-0.0351398	-0.0448981	0.94885	0.99995	no

ENSG00000107105	ENSG00000107105	ELAVL2	9:23690101-23826335	q1	q2	OK	16.7089	16.986	0.0237345	0.0453885	0.9388	0.99995	no

ENSG00000107130	ENSG00000107130	NCS1	9:132934856-132999583	q1	q2	NOTEST	0	0	0	0	1	1	no

ENSG00000107140	ENSG00000107140	TESK1	9:35605366-35647096	q1	q2	OK	19.009	18.1183	-0.0692404	-0.103335	0.87475	0.99995	no

ENSG00000107147	ENSG00000107147	KCNT1	9:138594030-138684992	q1	q2	NOTEST	0.03213	0.0561151	0.80447	0	1	1	no

ENSG00000107159	ENSG00000107159	CA9	9:35658300-35681156	q1	q2	NOTEST	0	0	0	0	1	1	no

ENSG00000107164	ENSG00000107164	FUBP3	9:133454351-133513739	q1	q2	OK	38.5133	41.3946	0.104086	0.207884	0.7113	0.99995	no

ENSG00000107165	ENSG00000107165	TYRP1	9:12685438-12822130	q1	q2	NOTEST	0.0555668	0.00999593	-2.47481	0	1	1	no

ENSG00000107175	ENSG00000107175	CREB3	9:35696944-35758572	q1	q2	OK	26.0441	25.3715	-0.0377452	-0.0266737	0.97	0.99995	no

ENSG00000107185	ENSG00000107185	RGP1	9:35696944-35758572	q1	q2	OK	11.2149	11.3784	0.0208851	0.0193928	0.9763	0.99995	no

ENSG00000107186	ENSG00000107186	MPDZ	9:13105702-13279589	q1	q2	OK	37.7546	39.5879	0.0684093	0.119203	0.8506	0.99995	no

ENSG00000107187	ENSG00000107187	LHX3	9:139088095-139096955	q1	q2	NOTEST	0.033464	0.0113888	-1.555	0	1	1	no

ENSG00000107201	ENSG00000107201	DDX58	9:32455299-32526322	q1	q2	OK	2.11119	2.26193	0.0994972	0.154677	0.82225	0.99995	no

ENSG00000107223	ENSG00000107223	EDF1	9:139756570-139760738	q1	q2	OK	207.545	194.551	-0.0932744	-0.221473	0.75085	0.99995	no

ENSG00000107242	ENSG00000107242	PIP5K1B	9:71320574-71624092	q1	q2	OK	18.9486	27.1047	0.516448	1.01679	0.14615	0.99995	no

ENSG00000107249	ENSG00000107249	GLIS3	9:3824126-4348392	q1	q2	OK	2.53896	1.70567	-0.573902	-0.577483	0.1826	0.99995	no

ENSG00000107262	ENSG00000107262	BAG1	9:33218362-33264761	q1	q2	OK	29.4253	27.9061	-0.0764777	-0.151997	0.8197	0.99995	no

ENSG00000107263	ENSG00000107263	RAPGEF1	9:134452156-134615461	q1	q2	OK	18.094	18.3792	0.0225611	0.0288044	0.95495	0.99995	no

ENSG00000107281	ENSG00000107281	NPDC1	9:139933921-139940655	q1	q2	OK	32.3028	24.7244	-0.385726	-0.552157	0.3151	0.99995	no

ENSG00000107282	ENSG00000107282	APBA1	9:72042445-72287222	q1	q2	NOTEST	0.321415	0.198346	-0.696417	0	1	1	no

ENSG00000107290	ENSG00000107290	SETX	9:135136742-135230372	q1	q2	OK	23.6512	24.5743	0.0552405	0.0790405	0.885	0.99995	no

ENSG00000107295	ENSG00000107295	SH3GL2	9:17579079-17797127	q1	q2	OK	1.34579	1.2312	-0.128392	-0.133546	0.8293	0.99995	no

ENSG00000107317	ENSG00000107317	PTGDS	9:139871955-139880862	q1	q2	NOTEST	0.314855	0.182285	-0.788489	0	1	1	no

ENSG00000107331	ENSG00000107331	ABCA2	9:139901685-139931234	q1	q2	OK	13.6512	10.1523	-0.427222	-0.417406	0.33095	0.99995	no

ENSG00000107338	ENSG00000107338	SHB	9:37510888-38069210	q1	q2	OK	1.70584	1.43052	-0.253943	-0.0826507	0.9146	0.99995	no

ENSG00000107341	ENSG00000107341	UBE2R2	9:33750514-33920402	q1	q2	OK	50.059	53.1432	0.0862565	0.214243	0.7595	0.99995	no

ENSG00000107362	ENSG00000107362	ABHD17B	9:74477367-74525847	q1	q2	OK	22.6093	22.0075	-0.0389196	-0.0814453	0.9065	0.99995	no

ENSG00000107371	ENSG00000107371	EXOSC3	9:37510888-38069210	q1	q2	OK	36.5673	38.6019	0.0781184	0.0568708	0.93185	0.99995	no

ENSG00000107372	ENSG00000107372	ZFAND5	9:74966340-74980163	q1	q2	OK	115.194	116.388	0.0148779	0.0276251	0.96335	0.99995	no

ENSG00000107404	ENSG00000107404	DVL1	1:1266693-1284730	q1	q2	OK	15.1488	13.3872	-0.178349	-0.356413	0.5735	0.99995	no

ENSG00000107438	ENSG00000107438	PDLIM1	10:96997328-97050781	q1	q2	OK	13.925	12.2724	-0.182269	-0.307544	0.6351	0.99995	no

ENSG00000107443	ENSG00000107443	CCNJ	10:97471535-97849995	q1	q2	OK	35.6357	41.0955	0.205657	0.365926	0.604	0.99995	no

ENSG00000107447	ENSG00000107447	DNTT	10:98052441-98098321	q1	q2	NOTEST	0	0	0	0	1	1	no

ENSG00000107485	ENSG00000107485	GATA3	10:8095566-8117161	q1	q2	NOTEST	0.292188	0.437492	0.58236	0	1	1	no

ENSG00000107518	ENSG00000107518	ATRNL1	10:116853123-117708503	q1	q2	OK	1.40608	1.67326	0.250981	0.20847	0.75455	0.99995	no

ENSG00000107521	ENSG00000107521	HPS1	10:100175954-100206684	q1	q2	OK	1.53718	1.87131	0.283756	0.243016	0.64245	0.99995	no

ENSG00000107537	ENSG00000107537	PHYH	10:13319795-13344412	q1	q2	OK	13.9132	13.7624	-0.0157213	-0.0287451	0.96685	0.99995	no

ENSG00000107551	ENSG00000107551	RASSF4	10:45306471-45491339	q1	q2	OK	9.33227	8.6433	-0.110647	-0.150182	0.77505	0.99995	no

ENSG00000107554	ENSG00000107554	DNMBP	10:101635333-101769676	q1	q2	OK	9.14574	8.85961	-0.0458576	-0.0625182	0.9009	0.99995	no

ENSG00000107560	ENSG00000107560	RAB11FIP2	10:119764426-119969663	q1	q2	OK	4.69393	5.97435	0.347987	0.54312	0.34175	0.99995	no

ENSG00000107562	ENSG00000107562	CXCL12	10:44793037-44881941	q1	q2	OK	7.25221	6.51912	-0.153745	-0.240225	0.6993	0.99995	no

ENSG00000107566	ENSG00000107566	ERLIN1	10:101909850-101989376	q1	q2	OK	17.1408	17.6581	0.0428967	0.054041	0.93415	0.99995	no

ENSG00000107581	ENSG00000107581	EIF3A	10:120794355-120840316	q1	q2	OK	76.598	81.3717	0.08722	0.20886	0.75815	0.99995	no

ENSG00000107593	ENSG00000107593	PKD2L1	10:102047902-102090243	q1	q2	NOTEST	0.112721	0.137228	0.283814	0	1	1	no

ENSG00000107611	ENSG00000107611	CUBN	10:16865962-17171830	q1	q2	OK	0.735852	0.90922	0.305214	0.349832	0.4932	0.99995	no

ENSG00000107614	ENSG00000107614	TRDMT1	10:17184252-17244053	q1	q2	OK	8.58852	9.27343	0.110693	0.156295	0.7669	0.99995	no

ENSG00000107618	ENSG00000107618	RBP3	10:48381486-48390991	q1	q2	NOTEST	0	0	0	0	1	1	no

ENSG00000107623	ENSG00000107623	GDF10	10:48425814-48438976	q1	q2	NOTEST	0.0105091	0.0327767	1.64104	0	1	1	no

ENSG00000107625	ENSG00000107625	DDX50	10:70661033-70706603	q1	q2	OK	31.6459	32.6497	0.045051	0.097432	0.88255	0.99995	no

ENSG00000107643	ENSG00000107643	MAPK8	10:49514697-49647403	q1	q2	OK	35.6656	35.055	-0.0249116	-0.0343201	0.9506	0.99995	no

ENSG00000107651	ENSG00000107651	SEC23IP	10:121652222-121702014	q1	q2	OK	19.7653	21.3106	0.108597	0.1614	0.7047	0.99995	no

ENSG00000107669	ENSG00000107669	ATE1	10:123499938-123711480	q1	q2	OK	13.6094	14.0859	0.0496486	0.0829485	0.8821	0.99995	no

ENSG00000107672	ENSG00000107672	NSMCE4A	10:123716602-123734732	q1	q2	OK	49.3033	51.0848	0.0512109	0.1075	0.8787	0.99995	no

ENSG00000107679	ENSG00000107679	PLEKHA1	10:124134211-124191867	q1	q2	OK	11.0042	10.1421	-0.117699	-0.238506	0.72795	0.99995	no

ENSG00000107719	ENSG00000107719	PALD1	10:72238576-72328205	q1	q2	OK	4.67194	5.04226	0.110049	0.206901	0.7703	0.99995	no

ENSG00000107731	ENSG00000107731	UNC5B	10:72972326-73062621	q1	q2	OK	3.39695	3.17201	-0.0988397	-0.179038	0.8009	0.99995	no

ENSG00000107736	ENSG00000107736	CDH23	10:73156690-73575702	q1	q2	OK	0.918115	0.493111	-0.896763	-0.787676	0.20725	0.99995	no

ENSG00000107738	ENSG00000107738	C10orf54	10:73156690-73575702	q1	q2	NOTEST	0.10754	0.222914	1.05161	0	1	1	no

ENSG00000107742	ENSG00000107742	SPOCK2	10:73818792-73848790	q1	q2	OK	0.668203	0.663599	-0.00997551	-0.00788628	0.98475	0.99995	no

ENSG00000107745	ENSG00000107745	MICU1	10:74127097-74385899	q1	q2	OK	26.7222	26.3684	-0.019224	-0.0402129	0.94805	0.99995	no

ENSG00000107758	ENSG00000107758	PPP3CB	10:75196185-75385711	q1	q2	OK	40.5836	42.9736	0.0825538	0.138398	0.84395	0.99995	no

ENSG00000107771	ENSG00000107771	CCSER2	10:86088341-86278273	q1	q2	OK	14.1502	14.2727	0.0124386	0.0189782	0.97075	0.99995	no

ENSG00000107779	ENSG00000107779	BMPR1A	10:88516406-88692595	q1	q2	OK	22.4104	39.9227	0.833043	0.604572	0.10975	0.99995	no

ENSG00000107789	ENSG00000107789	MINPP1	10:89264631-89313217	q1	q2	OK	21.1387	21.8003	0.0444612	0.101109	0.88475	0.99995	no

ENSG00000107796	ENSG00000107796	ACTA2	10:90639490-90775542	q1	q2	OK	69.1391	73.2193	0.0827213	0.165091	0.81345	0.99995	no

ENSG00000107798	ENSG00000107798	LIPA	10:90973325-91174314	q1	q2	OK	16.4084	18.3096	0.15817	0.314785	0.62935	0.99995	no

ENSG00000107807	ENSG00000107807	TLX1	10:102849077-102899468	q1	q2	NOTEST	0.244534	0.111992	-1.12664	0	1	1	no

ENSG00000107815	ENSG00000107815	C10orf2	10:102729214-102754158	q1	q2	OK	7.02265	7.14362	0.0246389	0.0242634	0.9676	0.99995	no

ENSG00000107816	ENSG00000107816	LZTS2	10:102756374-102790890	q1	q2	OK	34.3837	31.3501	-0.13325	-0.256367	0.6972	0.99995	no

ENSG00000107819	ENSG00000107819	SFXN3	10:102790990-102800998	q1	q2	OK	5.47688	5.14282	-0.0907946	-0.112064	0.84035	0.99995	no

ENSG00000107821	ENSG00000107821	KAZALD1	10:102821597-102827888	q1	q2	OK	4.28385	4.35796	0.0247444	0.0276845	0.9681	0.99995	no

ENSG00000107829	ENSG00000107829	FBXW4	10:103370422-103455052	q1	q2	OK	6.72395	6.88054	0.0332125	0.0452621	0.9432	0.99995	no

ENSG00000107831	ENSG00000107831	FGF8	10:103529898-103535854	q1	q2	NOTEST	0.0841739	0.0246965	-1.76907	0	1	1	no

ENSG00000107833	ENSG00000107833	NPM3	10:103541081-103543170	q1	q2	OK	23.3495	25.3955	0.12118	0.213563	0.76335	0.99995	no

ENSG00000107854	ENSG00000107854	TNKS2	10:93558068-93625033	q1	q2	OK	27.3704	30.5909	0.160486	0.396153	0.57205	0.99995	no

ENSG00000107859	ENSG00000107859	PITX3	10:103989942-104001231	q1	q2	NOTEST	0.0797041	0.114384	0.521162	0	1	1	no

ENSG00000107862	ENSG00000107862	GBF1	10:104005288-104142656	q1	q2	OK	21.095	19.1835	-0.137039	-0.266647	0.6231	0.99995	no

ENSG00000107863	ENSG00000107863	ARHGAP21	10:24872537-25012597	q1	q2	OK	99.6885	104.11	0.0626036	0.0880244	0.83405	0.99995	no

ENSG00000107864	ENSG00000107864	CPEB3	10:93806448-94050844	q1	q2	OK	0.498869	0.401852	-0.311997	-0.375503	0.6029	0.99995	no

ENSG00000107872	ENSG00000107872	FBXL15	10:104162375-104182893	q1	q2	OK	6.63789	6.58568	-0.0113922	-0.00989511	0.98935	0.99995	no

ENSG00000107874	ENSG00000107874	CUEDC2	10:104183001-104192418	q1	q2	OK	58.5659	53.1531	-0.139908	-0.315938	0.64965	0.99995	no

ENSG00000107882	ENSG00000107882	SUFU	10:104263743-104393292	q1	q2	OK	10.285	10.01	-0.0391033	-0.0863515	0.898	0.99995	no

ENSG00000107890	ENSG00000107890	ANKRD26	10:27280842-27389421	q1	q2	OK	9.25519	9.09938	-0.0244945	-0.0493007	0.93895	0.99995	no

ENSG00000107897	ENSG00000107897	ACBD5	10:27484145-27531059	q1	q2	OK	9.91766	10.189	0.038935	0.0749399	0.91235	0.99995	no

ENSG00000107902	ENSG00000107902	LHPP	10:126150402-126480439	q1	q2	OK	6.26214	4.19891	-0.576641	-0.294651	0.6583	0.99995	no

ENSG00000107929	ENSG00000107929	LARP4B	10:855483-977564	q1	q2	OK	15.8133	17.0181	0.105932	0.174638	0.7176	0.99995	no

ENSG00000107937	ENSG00000107937	GTPBP4	10:1034337-1095110	q1	q2	OK	39.0845	42.0736	0.106319	0.0933067	0.8534	0.99995	no

ENSG00000107938	ENSG00000107938	EDRF1	10:127371797-127452712	q1	q2	OK	23.8879	23.6797	-0.0126282	-0.0222059	0.97255	0.99995	no

ENSG00000107949	ENSG00000107949	BCCIP	10:127512114-127585005	q1	q2	OK	69.6328	76.4429	0.134616	0.258823	0.70165	0.99995	no

ENSG00000107951	ENSG00000107951	MTPAP	10:30598729-30663377	q1	q2	OK	7.67367	8.91727	0.216686	0.28201	0.6143	0.99995	no

ENSG00000107954	ENSG00000107954	NEURL1	10:105239359-105615301	q1	q2	OK	0.997419	0.886362	-0.170304	-0.101491	0.90435	0.99995	no

ENSG00000107957	ENSG00000107957	SH3PXD2A	10:105239359-105615301	q1	q2	OK	3.82169	4.09216	0.0986538	0.186636	0.7566	0.99995	no

ENSG00000107959	ENSG00000107959	PITRM1	10:3179919-3215003	q1	q2	OK	20.1686	19.7005	-0.0338841	-0.053772	0.9182	0.99995	no

ENSG00000107960	ENSG00000107960	OBFC1	10:105642299-105677963	q1	q2	OK	6.53844	6.64969	0.0243408	0.0365818	0.95905	0.99995	no

ENSG00000107968	ENSG00000107968	MAP3K8	10:30722865-30750762	q1	q2	OK	1.3406	1.47643	0.139239	0.121207	0.8672	0.99995	no

ENSG00000107984	ENSG00000107984	DKK1	10:54074055-54077802	q1	q2	OK	22.6512	31.7982	0.489357	0.624397	0.31925	0.99995	no

ENSG00000108001	ENSG00000108001	EBF3	10:131633546-131762105	q1	q2	OK	13.3776	14.6947	0.135477	0.261862	0.68165	0.99995	no

ENSG00000108010	ENSG00000108010	GLRX3	10:131934662-131982785	q1	q2	OK	74.8683	81.3236	0.11932	0.249461	0.678	0.99995	no

ENSG00000108018	ENSG00000108018	SORCS1	10:108333420-108924292	q1	q2	OK	10.1862	9.16217	-0.152857	-0.242164	0.68515	0.99995	no

ENSG00000108021	ENSG00000108021	FAM208B	10:5726800-5805703	q1	q2	OK	17.0747	19.2167	0.170497	0.177289	0.5708	0.99995	no

ENSG00000108039	ENSG00000108039	XPNPEP1	10:111624523-111683311	q1	q2	OK	35.4582	39.6878	0.162577	0.284641	0.6522	0.99995	no

ENSG00000108055	ENSG00000108055	SMC3	10:112327448-112364394	q1	q2	OK	64.1941	68.6002	0.095771	0.233236	0.731	0.99995	no

ENSG00000108061	ENSG00000108061	SHOC2	10:112679300-112773425	q1	q2	OK	18.0531	18.7927	0.0579277	0.0911907	0.84035	0.99995	no

ENSG00000108064	ENSG00000108064	TFAM	10:60144781-60158981	q1	q2	OK	31.0859	30.7629	-0.0150697	-0.0241036	0.9729	0.99995	no

ENSG00000108091	ENSG00000108091	CCDC6	10:61548520-61666414	q1	q2	OK	12.3858	13.0934	0.0801512	0.186074	0.77955	0.99995	no

ENSG00000108094	ENSG00000108094	CUL2	10:35297478-35379570	q1	q2	OK	21.7305	21.422	-0.0206286	-0.0404913	0.9456	0.99995	no

ENSG00000108100	ENSG00000108100	CCNY	10:35535952-35860852	q1	q2	OK	24.0076	21.7067	-0.14535	-0.204813	0.65615	0.99995	no

ENSG00000108106	ENSG00000108106	UBE2S	19:55896712-55919145	q1	q2	OK	112.061	112.359	0.00383267	0.00423532	0.9943	0.99995	no

ENSG00000108107	ENSG00000108107	RPL28	19:55896712-55919145	q1	q2	OK	537.305	479.625	-0.163834	-0.277812	0.6956	0.99995	no

ENSG00000108175	ENSG00000108175	ZMIZ1	10:80828791-81076276	q1	q2	OK	38.6097	37.8017	-0.0305098	-0.0664062	0.91085	0.99995	no

ENSG00000108176	ENSG00000108176	DNAJC12	10:69556426-69597924	q1	q2	OK	5.79472	5.59105	-0.0516209	-0.0670864	0.9253	0.99995	no

ENSG00000108179	ENSG00000108179	PPIF	10:81107224-81115093	q1	q2	OK	29.855	27.915	-0.0969321	-0.205918	0.7526	0.99995	no

ENSG00000108187	ENSG00000108187	PBLD	10:70042416-70167051	q1	q2	OK	3.44992	3.21455	-0.101947	-0.0229202	0.90895	0.99995	no

ENSG00000108219	ENSG00000108219	TSPAN14	10:82213921-82295698	q1	q2	OK	68.862	64.2879	-0.0991602	-0.164917	0.7974	0.99995	no

ENSG00000108231	ENSG00000108231	LGI1	10:95517565-95557916	q1	q2	OK	10.5245	16.0887	0.612297	0.68505	0.1613	0.99995	no

ENSG00000108239	ENSG00000108239	TBC1D12	10:96162260-96295687	q1	q2	OK	5.85435	5.73708	-0.0291932	-0.0574716	0.93115	0.99995	no

ENSG00000108242	ENSG00000108242	CYP2C18	10:96443250-96613017	q1	q2	NOTEST	0	0	0	0	1	1	no

ENSG00000108244	ENSG00000108244	KRT23	17:39077681-39132178	q1	q2	NOTEST	0	0	0	0	1	1	no

ENSG00000108255	ENSG00000108255	CRYBA1	17:27573880-27581512	q1	q2	OK	2.97093	3.1506	0.0847085	0.0917744	0.89115	0.99995	no

ENSG00000108256	ENSG00000108256	NUFIP2	17:27582853-27621136	q1	q2	OK	18.998	21.6574	0.189016	0.436555	0.50595	0.99995	no

ENSG00000108262	ENSG00000108262	GIT1	17:27887564-28513493	q1	q2	OK	22.5625	23.1966	0.0399835	0.0335872	0.9413	0.99995	no

ENSG00000108264	ENSG00000108264	TADA2A	17:35766964-35839835	q1	q2	OK	14.608	14.3153	-0.0292007	-0.0436878	0.9403	0.99995	no

ENSG00000108270	ENSG00000108270	AATF	17:35306174-35414171	q1	q2	OK	25.5399	27.0755	0.0842395	0.185152	0.7783	0.99995	no

ENSG00000108272	ENSG00000108272	DHRS11	17:34948227-34957235	q1	q2	OK	1.21671	0.121003	-3.32988	-1.35902	0.00215	0.99995	no

ENSG00000108278	ENSG00000108278	ZNHIT3	17:34842472-34899284	q1	q2	OK	3.61668	3.51285	-0.0420223	-0.0447782	0.94905	0.99995	no

ENSG00000108292	ENSG00000108292	MLLT6	17:36858704-36886056	q1	q2	OK	23.1269	22.6451	-0.0303745	-0.0417561	0.9227	0.99995	no

ENSG00000108294	ENSG00000108294	PSMB3	17:36908988-36920484	q1	q2	OK	118.992	112.578	-0.0799401	-0.187131	0.7906	0.99995	no

ENSG00000108296	ENSG00000108296	CWC25	17:36956686-36981734	q1	q2	OK	11.9259	10.9411	-0.124336	-0.204443	0.74235	0.99995	no

ENSG00000108298	ENSG00000108298	RPL19	17:37356535-37360980	q1	q2	OK	747.419	733.746	-0.0266374	-0.0620264	0.9265	0.99995	no

ENSG00000108306	ENSG00000108306	FBXL20	17:37415383-37607539	q1	q2	OK	6.93843	6.24723	-0.151394	-0.109064	0.85695	0.99995	no

ENSG00000108309	ENSG00000108309	RUNDC3A	17:42376941-42396039	q1	q2	OK	7.87477	5.71561	-0.46233	-0.644864	0.3648	0.99995	no

ENSG00000108312	ENSG00000108312	UBTF	17:42269172-42301078	q1	q2	OK	43.3685	41.4864	-0.0640097	-0.0916564	0.8688	0.99995	no

ENSG00000108342	ENSG00000108342	CSF3	17:38171613-38174066	q1	q2	NOTEST	0	0.0212317	inf	0	1	1	no

ENSG00000108344	ENSG00000108344	PSMD3	17:38137049-38154213	q1	q2	OK	73.9191	68.4747	-0.110376	-0.258825	0.6935	0.99995	no

ENSG00000108349	ENSG00000108349	CASC3	17:38296570-38328436	q1	q2	OK	43.9155	44.7207	0.0262101	0.0474942	0.92505	0.99995	no

ENSG00000108352	ENSG00000108352	RAPGEFL1	17:38333262-38351908	q1	q2	OK	12.0223	10.8959	-0.141931	-0.202568	0.70725	0.99995	no

ENSG00000108370	ENSG00000108370	RGS9	17:63133548-63223821	q1	q2	OK	0.41964	0.633966	0.595255	0.456825	0.44895	0.99995	no

ENSG00000108375	ENSG00000108375	RNF43	17:56378591-56494956	q1	q2	NOTEST	0.097764	0.11358	0.21633	0	1	1	no

ENSG00000108379	ENSG00000108379	WNT3	17:44839871-44910520	q1	q2	OK	6.09532	5.12971	-0.248825	-0.456219	0.4991	0.99995	no

ENSG00000108381	ENSG00000108381	ASPA	17:3343312-3461289	q1	q2	NOTEST	0.0533326	0.0259298	-1.04041	0	1	1	no

ENSG00000108384	ENSG00000108384	RAD51C	17:56769933-56811703	q1	q2	OK	17.9452	18.7288	0.0616586	0.0995741	0.888	0.99995	no

ENSG00000108387	ENSG00000108387	Sep-04	17:56597197-56769416	q1	q2	OK	2.30189	1.83749	-0.325081	-0.275826	0.5848	0.99995	no

ENSG00000108389	ENSG00000108389	MTMR4	17:56566897-56595266	q1	q2	OK	30.3564	34.7996	0.19707	0.305143	0.5001	0.99995	no

ENSG00000108395	ENSG00000108395	TRIM37	17:57059998-57232630	q1	q2	OK	22.5621	22.5643	0.000140916	8.64E-05	0.99975	0.99995	no

ENSG00000108405	ENSG00000108405	P2RX1	17:3799885-3819794	q1	q2	NOTEST	0	0	0	0	1	1	no

ENSG00000108406	ENSG00000108406	DHX40	17:57642885-57685706	q1	q2	OK	47.2552	45.2034	-0.0640424	-0.139367	0.81495	0.99995	no

ENSG00000108417	ENSG00000108417	KRT37	17:39558667-39581301	q1	q2	NOTEST	0	0	0	0	1	1	no

ENSG00000108423	ENSG00000108423	TUBD1	17:57936850-57970304	q1	q2	OK	8.66971	8.14542	-0.0899944	-0.126332	0.8294	0.99995	no

ENSG00000108424	ENSG00000108424	KPNB1	17:45726841-45762871	q1	q2	OK	218.419	238.074	0.124312	0.163005	0.76455	0.99995	no

ENSG00000108433	ENSG00000108433	GOSR2	17:45000482-45177689	q1	q2	OK	37.2221	37.1469	-0.00291723	-0.00464285	0.9944	0.99995	no

ENSG00000108439	ENSG00000108439	PNPO	17:46018871-46073562	q1	q2	NOTEST	0.100987	0.230891	1.19304	0	1	1	no

ENSG00000108442	ENSG00000108442	TVP23BP2	2:74785009-74875465	q1	q2	NOTEST	0	0	0	0	1	1	no

ENSG00000108443	ENSG00000108443	RPS6KB1	17:57970446-58096413	q1	q2	OK	19.2367	19.9513	0.0526211	0.0641497	0.8839	0.99995	no

ENSG00000108448	ENSG00000108448	TRIM16L	17:18601310-18639431	q1	q2	OK	3.90707	3.47679	-0.168333	-0.179326	0.75055	0.99995	no

ENSG00000108452	ENSG00000108452	ZNF29P	17:15468796-15587625	q1	q2	NOTEST	0	0	0	0	1	1	no

ENSG00000108465	ENSG00000108465	CDK5RAP3	17:46018871-46073562	q1	q2	OK	102.919	92.7066	-0.15077	-0.277023	0.5948	0.99995	no

ENSG00000108468	ENSG00000108468	CBX1	17:46147413-46178883	q1	q2	OK	205.197	212.378	0.0496259	0.116012	0.8648	0.99995	no

ENSG00000108469	ENSG00000108469	RECQL5	17:73584138-73704142	q1	q2	OK	12.337	11.191	-0.140656	-0.0916144	0.8216	0.99995	no

ENSG00000108474	ENSG00000108474	PIGL	17:15879873-16256970	q1	q2	OK	12.8507	13.7217	0.0946119	0.0297687	0.88425	0.99995	no

ENSG00000108479	ENSG00000108479	GALK1	17:73717407-73761792	q1	q2	OK	4.94284	4.1491	-0.25254	-0.286448	0.62345	0.99995	no

ENSG00000108506	ENSG00000108506	INTS2	17:59942730-60005377	q1	q2	OK	8.93197	9.86712	0.143651	0.220218	0.6519	0.99995	no

ENSG00000108509	ENSG00000108509	CAMTA2	17:4871286-4890972	q1	q2	OK	7.55509	6.01981	-0.32773	-0.358639	0.46115	0.99995	no

ENSG00000108510	ENSG00000108510	MED13	17:60019965-60142643	q1	q2	OK	16.4471	17.4259	0.0833974	0.139955	0.7588	0.99995	no

ENSG00000108511	ENSG00000108511	HOXB6	17:46618255-46683776	q1	q2	NOTEST	0.0325072	0.100829	1.63308	0	1	1	no

ENSG00000108515	ENSG00000108515	ENO3	17:4848946-4860426	q1	q2	OK	7.12384	4.56415	-0.642308	-0.10539	0.27985	0.99995	no

ENSG00000108516	ENSG00000108516	AC003958.6	17:39490883-39491985	q1	q2	NOTEST	0	0	0	0	1	1	no

ENSG00000108518	ENSG00000108518	PFN1	17:4848946-4860426	q1	q2	OK	382.135	396.134	0.051907	0.122228	0.859	0.99995	no

ENSG00000108523	ENSG00000108523	RNF167	17:4840424-4848517	q1	q2	OK	47.1978	43.3714	-0.121978	-0.181915	0.74975	0.99995	no

ENSG00000108528	ENSG00000108528	SLC25A11	17:4840424-4848517	q1	q2	OK	30.3641	25.7421	-0.238239	-0.333852	0.609	0.99995	no

ENSG00000108551	ENSG00000108551	RASD1	17:17397750-17399709	q1	q2	OK	6.53183	5.31701	-0.296872	-0.464955	0.5118	0.99995	no

ENSG00000108556	ENSG00000108556	CHRNE	17:4736682-4806369	q1	q2	NOTEST	0.207825	0.137868	-0.592082	0	1	1	no

ENSG00000108557	ENSG00000108557	RAI1	17:17584786-17740325	q1	q2	OK	20.3774	23.0352	0.17687	0.175275	0.66695	0.99995	no

ENSG00000108559	ENSG00000108559	NUP88	17:5185557-5372380	q1	q2	OK	34.0541	33.2331	-0.0352094	-0.0187068	0.96155	0.99995	no

ENSG00000108561	ENSG00000108561	C1QBP	17:5185557-5372380	q1	q2	OK	90.7338	104.278	0.200716	0.162015	0.80815	0.99995	no

ENSG00000108576	ENSG00000108576	SLC6A4	17:28521336-28563020	q1	q2	NOTEST	0.138496	0.221239	0.675761	0	1	1	no

ENSG00000108578	ENSG00000108578	BLMH	17:28565758-28619074	q1	q2	OK	47.2216	47.0163	-0.00628536	-0.012808	0.98295	0.99995	no

ENSG00000108582	ENSG00000108582	CPD	17:28705922-28797007	q1	q2	OK	23.9648	25.4305	0.0856445	0.134231	0.7689	0.99995	no

ENSG00000108587	ENSG00000108587	GOSR1	17:28804379-28854610	q1	q2	OK	19.112	21.1035	0.143003	0.204927	0.73325	0.99995	no

ENSG00000108588	ENSG00000108588	CCDC47	17:61780191-61896677	q1	q2	OK	46.55	50.9142	0.129286	0.164675	0.8094	0.99995	no

ENSG00000108590	ENSG00000108590	MED31	17:6481467-6554954	q1	q2	OK	21.3398	17.1204	-0.317832	-0.217321	0.8019	0.99995	no

ENSG00000108591	ENSG00000108591	DRG2	17:17991199-18011285	q1	q2	OK	18.0781	16.6885	-0.115392	-0.181204	0.76865	0.99995	no

ENSG00000108592	ENSG00000108592	FTSJ3	17:61896792-61909379	q1	q2	OK	34.0661	32.6521	-0.061161	-0.0621379	0.88395	0.99995	no

ENSG00000108599	ENSG00000108599	AKAP10	17:19807614-19881656	q1	q2	OK	10.3858	10.2971	-0.012368	-0.0192536	0.96955	0.99995	no

ENSG00000108602	ENSG00000108602	ALDH3A1	17:19640994-19652256	q1	q2	NOTEST	0.0431654	0	#NAME?	0	1	1	no

ENSG00000108604	ENSG00000108604	SMARCD2	17:61909443-61920425	q1	q2	OK	23.3776	22.8453	-0.0332311	-0.0548514	0.92145	0.99995	no

ENSG00000108622	ENSG00000108622	ICAM2	17:62073430-62097994	q1	q2	NOTEST	0.022829	0.0487259	1.09382	0	1	1	no

ENSG00000108639	ENSG00000108639	SYNGR2	17:76164638-76169608	q1	q2	OK	4.79419	3.79261	-0.338094	-0.469023	0.4933	0.99995	no

ENSG00000108641	ENSG00000108641	B9D1	17:19240866-19290553	q1	q2	OK	11.0201	9.6257	-0.195167	-0.143491	0.8155	0.99995	no

ENSG00000108651	ENSG00000108651	UTP6	17:30187922-30228784	q1	q2	OK	38.5079	41.2513	0.0992847	0.144585	0.8279	0.99995	no

ENSG00000108654	ENSG00000108654	DDX5	17:62495733-62658186	q1	q2	NOTEST	0.128523	0.127048	-0.0166497	0	1	1	no

ENSG00000108666	ENSG00000108666	C17orf75	17:30593194-30708905	q1	q2	OK	22.5477	26.0704	0.209433	0.0947645	0.78865	0.99995	no

ENSG00000108669	ENSG00000108669	CYTH1	17:76670129-76778379	q1	q2	OK	15.6841	16.0643	0.034554	0.0709628	0.90975	0.99995	no

ENSG00000108671	ENSG00000108671	PSMD11	17:30771278-30810336	q1	q2	OK	73.1542	72.064	-0.0216619	-0.0309366	0.96195	0.99995	no

ENSG00000108679	ENSG00000108679	LGALS3BP	17:76967319-76976191	q1	q2	OK	15.6208	11.4506	-0.44805	-0.784306	0.20525	0.99995	no

ENSG00000108684	ENSG00000108684	ASIC2	17:31340104-32522956	q1	q2	NOTEST	0.144365	0.202505	0.488237	0	1	1	no

ENSG00000108688	ENSG00000108688	CCL7	17:32597239-32599261	q1	q2	NOTEST	0	0	0	0	1	1	no

ENSG00000108691	ENSG00000108691	CCL2	17:32581899-32584222	q1	q2	OK	33.9919	26.627	-0.352302	-0.559983	0.42135	0.99995	no

ENSG00000108700	ENSG00000108700	CCL8	17:32646054-32648421	q1	q2	NOTEST	0	0	0	0	1	1	no

ENSG00000108702	ENSG00000108702	CCL1	17:32687346-32690250	q1	q2	NOTEST	0	0	0	0	1	1	no

ENSG00000108733	ENSG00000108733	PEX12	17:33901813-34053436	q1	q2	OK	3.60536	3.58217	-0.00930852	-0.0051226	0.99465	0.99995	no

ENSG00000108753	ENSG00000108753	HNF1B	17:36046434-36108563	q1	q2	NOTEST	0.0427399	0.0509029	0.252165	0	1	1	no

ENSG00000108759	ENSG00000108759	KRT32	17:39616062-39623681	q1	q2	NOTEST	0	0	0	0	1	1	no

ENSG00000108771	ENSG00000108771	DHX58	17:40253421-40264751	q1	q2	NOTEST	0	0	0	0	1	1	no

ENSG00000108773	ENSG00000108773	KAT2A	17:40265125-40307990	q1	q2	OK	0.475684	1.65741	1.80086	0.681122	0.31115	0.99995	no

ENSG00000108774	ENSG00000108774	RAB5C	17:40265125-40307990	q1	q2	OK	46.3483	42.0349	-0.140929	-0.256847	0.699	0.99995	no

ENSG00000108784	ENSG00000108784	NAGLU	17:40688189-40718295	q1	q2	OK	8.86637	7.26616	-0.28715	-0.254455	0.6604	0.99995	no

ENSG00000108785	ENSG00000108785	HSD17B1P1	17:40688189-40718295	q1	q2	NOTEST	0.192143	0.191722	-0.00316533	0	1	1	no

ENSG00000108786	ENSG00000108786	HSD17B1	17:40688189-40718295	q1	q2	OK	0.907672	0.957785	0.0775312	0.041055	0.9462	0.99995	no

ENSG00000108788	ENSG00000108788	MLX	17:40719085-40729849	q1	q2	OK	20.5059	21.2989	0.0547462	0.0744622	0.89405	0.99995	no

ENSG00000108797	ENSG00000108797	CNTNAP1	17:40830906-40851832	q1	q2	OK	8.46099	7.62645	-0.149814	-0.194486	0.7747	0.99995	no

ENSG00000108798	ENSG00000108798	ABI3	17:47280152-47300587	q1	q2	NOTEST	0.293013	0.151363	-0.952956	0	1	1	no

ENSG00000108799	ENSG00000108799	EZH1	17:40852292-40897071	q1	q2	OK	16.1197	14.4763	-0.155129	-0.199778	0.687	0.99995	no

ENSG00000108813	ENSG00000108813	DLX4	17:48046333-48052321	q1	q2	NOTEST	0	0.0385046	inf	0	1	1	no

ENSG00000108819	ENSG00000108819	PPP1R9B	17:48211103-48227991	q1	q2	OK	25.9434	23.7297	-0.128676	-0.250412	0.68035	0.99995	no

ENSG00000108821	ENSG00000108821	COL1A1	17:48260649-48278993	q1	q2	OK	99.3147	74.1532	-0.421498	-0.500093	0.2859	0.99995	no

ENSG00000108823	ENSG00000108823	SGCA	17:48240883-48258539	q1	q2	NOTEST	0.0568849	0.0494619	-0.201726	0	1	1	no

ENSG00000108825	ENSG00000108825	PTGES3L-AARSD1	17:41102542-41132545	q1	q2	OK	0.647529	0.19077	-1.76311	-0.43131	0.23005	0.99995	no

ENSG00000108826	ENSG00000108826	MRPL27	17:48445217-48450575	q1	q2	OK	50.288	49.8056	-0.0139071	-0.0268503	0.97025	0.99995	no

ENSG00000108828	ENSG00000108828	VAT1	17:41166621-41177140	q1	q2	OK	63.7317	59.4679	-0.099899	-0.226868	0.7203	0.99995	no

ENSG00000108829	ENSG00000108829	LRRC59	17:48450580-48475249	q1	q2	OK	49.1315	49.4806	0.010213	0.0221478	0.97315	0.99995	no

ENSG00000108830	ENSG00000108830	RND2	17:41177257-41184057	q1	q2	OK	57.377	52.411	-0.130604	-0.273335	0.69155	0.99995	no

ENSG00000108839	ENSG00000108839	ALOX12	17:6757179-6922978	q1	q2	NOTEST	0.267107	0.432396	0.694934	0	1	1	no

ENSG00000108840	ENSG00000108840	HDAC5	17:42154113-42201070	q1	q2	OK	12.3722	9.76504	-0.341408	-0.555954	0.31915	0.99995	no

ENSG00000108846	ENSG00000108846	ABCC3	17:48712137-48769613	q1	q2	NOTEST	0.181032	0.135654	-0.41631	0	1	1	no

ENSG00000108848	ENSG00000108848	LUC7L3	17:48796904-48833574	q1	q2	OK	191.228	194.748	0.026313	0.0426255	0.93705	0.99995	no

ENSG00000108849	ENSG00000108849	PPY	17:42018171-42019836	q1	q2	NOTEST	0	0	0	0	1	1	no

ENSG00000108852	ENSG00000108852	MPP2	17:41952724-41987068	q1	q2	OK	5.87642	5.52888	-0.0879491	-0.161478	0.79075	0.99995	no

ENSG00000108854	ENSG00000108854	SMURF2	17:62495733-62658186	q1	q2	NOTEST	0.279363	0.256105	-0.12541	0	1	1	no

ENSG00000108861	ENSG00000108861	DUSP3	17:41843488-41856356	q1	q2	OK	12.672	11.5326	-0.135926	-0.289576	0.65095	0.99995	no

ENSG00000108878	ENSG00000108878	CACNG1	17:65040705-65052909	q1	q2	NOTEST	0.228405	0.0927592	-1.30003	0	1	1	no

ENSG00000108883	ENSG00000108883	EFTUD2	17:42923720-42994305	q1	q2	OK	57.6795	59.0718	0.0344099	0.0551885	0.92975	0.99995	no

ENSG00000108924	ENSG00000108924	HLF	17:53342372-53402426	q1	q2	NOTEST	0.0920115	0.167631	0.865406	0	1	1	no

ENSG00000108932	ENSG00000108932	SLC16A6	17:66255322-66760023	q1	q2	NOTEST	0	0	0	0	1	1	no

ENSG00000108946	ENSG00000108946	PRKAR1A	17:66255322-66760023	q1	q2	OK	118.982	130.865	0.137329	0.24058	0.6756	0.99995	no

ENSG00000108947	ENSG00000108947	EFNB3	17:7608519-7614696	q1	q2	OK	20.8823	17.7473	-0.23468	-0.546461	0.4382	0.99995	no

ENSG00000108950	ENSG00000108950	FAM20A	17:66255322-66760023	q1	q2	OK	0.675548	0.590473	-0.194188	-0.0242231	0.8421	0.99995	no

ENSG00000108953	ENSG00000108953	YWHAE	17:1247565-1303672	q1	q2	OK	806.183	840.897	0.0608223	0.0937345	0.874	0.99995	no

ENSG00000108958	ENSG00000108958	AC016292.3	17:1682778-1803376	q1	q2	OK	2.5403	3.18911	0.328155	0.0434757	0.8696	0.99995	no

ENSG00000108960	ENSG00000108960	MMD	17:53469973-53499353	q1	q2	OK	36.6682	40.1851	0.132132	0.282796	0.643	0.99995	no

ENSG00000108961	ENSG00000108961	RANGRF	17:8191080-8198661	q1	q2	OK	18.9429	18.9349	-0.000604849	-0.0010091	0.9988	0.99995	no

ENSG00000108963	ENSG00000108963	DPH1	17:1933403-1946724	q1	q2	OK	15.475	12.9839	-0.253216	-0.346244	0.56015	0.99995	no

ENSG00000108984	ENSG00000108984	MAP2K6	17:67410838-67539472	q1	q2	OK	12.482	13.5531	0.118779	0.166691	0.7775	0.99995	no

ENSG00000109016	ENSG00000109016	DHRS7B	17:21026676-21096578	q1	q2	OK	10.7283	10.6268	-0.0137117	-0.0182587	0.97695	0.99995	no

ENSG00000109046	ENSG00000109046	WSB1	17:25621101-25640657	q1	q2	OK	174.906	172.125	-0.0231224	-0.0456482	0.94195	0.99995	no

ENSG00000109047	ENSG00000109047	RCVRN	17:9799636-9808938	q1	q2	NOTEST	0.0971223	0.233861	1.26778	0	1	1	no

ENSG00000109061	ENSG00000109061	MYH1	17:10286448-10527203	q1	q2	NOTEST	0	0	0	0	1	1	no

ENSG00000109062	ENSG00000109062	SLC9A3R1	17:72666716-72765492	q1	q2	OK	24.0706	22.0783	-0.124647	-0.262539	0.70625	0.99995	no

ENSG00000109063	ENSG00000109063	MYH3	17:10531842-10560626	q1	q2	OK	1.2664	1.95234	0.624474	0.405224	0.3179	0.99995	no

ENSG00000109065	ENSG00000109065	NAT9	17:72766685-72772506	q1	q2	OK	26.8084	26.8712	0.00337565	0.00558165	0.9926	0.99995	no

ENSG00000109066	ENSG00000109066	TMEM104	17:72772621-72835918	q1	q2	OK	7.30804	7.37545	0.0132475	0.0193581	0.96855	0.99995	no

ENSG00000109072	ENSG00000109072	VTN	17:26684603-26734215	q1	q2	NOTEST	0	0	0	0	1	1	no

ENSG00000109079	ENSG00000109079	TNFAIP1	17:26662627-26684545	q1	q2	NOTEST	0	0.0144049	inf	0	1	1	no

ENSG00000109083	ENSG00000109083	IFT20	17:26646120-26662515	q1	q2	NOTEST	0.0787552	0.0946859	0.265775	0	1	1	no

ENSG00000109084	ENSG00000109084	TMEM97	17:26646120-26662515	q1	q2	NOTEST	0.0935825	0.0628013	-0.575444	0	1	1	no

ENSG00000109089	ENSG00000109089	CDR2L	17:72983726-73001895	q1	q2	OK	10.8298	9.55026	-0.181391	-0.382383	0.58585	0.99995	no

ENSG00000109099	ENSG00000109099	PMP22	17:15133094-15169024	q1	q2	OK	33.5794	34.644	0.0450303	0.0908766	0.8957	0.99995	no

ENSG00000109101	ENSG00000109101	FOXN1	17:26782769-26972472	q1	q2	NOTEST	0	0	0	0	1	1	no

ENSG00000109103	ENSG00000109103	UNC119	17:26782769-26972472	q1	q2	OK	27.6197	26.1209	-0.0804891	-0.045181	0.9509	0.99995	no

ENSG00000109107	ENSG00000109107	ALDOC	17:26782769-26972472	q1	q2	OK	6.57143	6.90759	0.0719753	0.0214472	0.9469	0.99995	no

ENSG00000109111	ENSG00000109111	SUPT6H	17:26975373-27029697	q1	q2	OK	32.1342	32.411	0.012377	0.0157357	0.96985	0.99995	no

ENSG00000109113	ENSG00000109113	RAB34	17:27041298-27045447	q1	q2	OK	43.949	46.6279	0.0853653	0.181535	0.8016	0.99995	no

ENSG00000109118	ENSG00000109118	PHF12	17:27232267-27384234	q1	q2	OK	28.7307	27.9782	-0.0382894	-0.0503857	0.9218	0.99995	no

ENSG00000109132	ENSG00000109132	PHOX2B	4:41746098-41826136	q1	q2	NOTEST	0.0726909	0.02616	-1.47441	0	1	1	no

ENSG00000109133	ENSG00000109133	TMEM33	4:41937136-41962589	q1	q2	OK	19.0574	21.1287	0.148852	0.216197	0.72285	0.99995	no

ENSG00000109158	ENSG00000109158	GABRA4	4:46920916-47428461	q1	q2	NOTEST	0.238659	0.176909	-0.431942	0	1	1	no

ENSG00000109163	ENSG00000109163	GNRHR	4:68566997-68995598	q1	q2	OK	0.48438	0.549182	0.181144	0.0931415	0.8813	0.99995	no

ENSG00000109171	ENSG00000109171	SLAIN2	4:48343338-48428229	q1	q2	OK	20.1971	21.5744	0.0951737	0.0823878	0.8508	0.99995	no

ENSG00000109180	ENSG00000109180	OCIAD1	4:48807228-48863834	q1	q2	OK	110.938	115.303	0.0556721	0.127925	0.85155	0.99995	no

ENSG00000109181	ENSG00000109181	UGT2B10	4:69681710-69696914	q1	q2	NOTEST	0	0	0	0	1	1	no

ENSG00000109182	ENSG00000109182	CWH43	4:48988263-49064098	q1	q2	NOTEST	0	0	0	0	1	1	no

ENSG00000109184	ENSG00000109184	DCUN1D4	4:52709165-52783003	q1	q2	OK	34.9488	35.7718	0.0335791	0.0548604	0.8989	0.99995	no

ENSG00000109189	ENSG00000109189	USP46	4:53457137-53525502	q1	q2	OK	12.7105	13.9666	0.135967	0.241766	0.6614	0.99995	no

ENSG00000109193	ENSG00000109193	SULT1E1	4:70657589-70725870	q1	q2	NOTEST	0.35622	0.137079	-1.37776	0	1	1	no

ENSG00000109205	ENSG00000109205	ODAM	4:71062212-71070293	q1	q2	NOTEST	0	0	0	0	1	1	no

ENSG00000109208	ENSG00000109208	SMR3A	4:71226477-71232875	q1	q2	NOTEST	0	0	0	0	1	1	no

ENSG00000109220	ENSG00000109220	CHIC2	4:54243809-55164414	q1	q2	OK	6.34385	5.77297	-0.136046	-0.0599302	0.92115	0.99995	no

ENSG00000109255	ENSG00000109255	NMU	4:56461395-56502865	q1	q2	OK	20.2028	21.5692	0.0944198	0.154262	0.82325	0.99995	no

ENSG00000109265	ENSG00000109265	KIAA1211	4:57036360-57194791	q1	q2	OK	12.1339	12.8876	0.0869398	0.194916	0.7754	0.99995	no

ENSG00000109270	ENSG00000109270	LAMTOR3	4:100799492-100815647	q1	q2	OK	38.452	40.1122	0.0609836	0.116385	0.86655	0.99995	no

ENSG00000109272	ENSG00000109272	PF4V1	4:74718905-74719872	q1	q2	NOTEST	0	0	0	0	1	1	no

ENSG00000109320	ENSG00000109320	NFKB1	4:103422485-103538459	q1	q2	OK	5.62297	5.76216	0.0352782	0.0643707	0.91915	0.99995	no

ENSG00000109321	ENSG00000109321	AREG	4:75310850-75320726	q1	q2	NOTEST	0	0	0	0	1	1	no

ENSG00000109323	ENSG00000109323	MANBA	4:103552659-103682151	q1	q2	OK	6.30984	6.4087	0.0224285	0.0320383	0.9571	0.99995	no

ENSG00000109332	ENSG00000109332	UBE2D3	4:103715539-103790053	q1	q2	OK	161.811	171.118	0.0806851	0.169626	0.78245	0.99995	no

ENSG00000109339	ENSG00000109339	MAPK10	4:86936275-87515284	q1	q2	OK	61.6003	55.9232	-0.139492	-0.25049	0.62195	0.99995	no

ENSG00000109381	ENSG00000109381	ELF2	4:139936942-140098372	q1	q2	OK	20.001	19.6252	-0.0273641	-0.0478485	0.9371	0.99995	no

ENSG00000109390	ENSG00000109390	NDUFC1	4:140187316-140397763	q1	q2	OK	68.1402	71.5187	0.0698153	0.0513429	0.9452	0.99995	no

ENSG00000109424	ENSG00000109424	UCP1	4:141480587-141489959	q1	q2	NOTEST	0.0386434	0.0195247	-0.984918	0	1	1	no

ENSG00000109436	ENSG00000109436	TBC1D9	4:141541918-141677274	q1	q2	OK	8.12329	7.88748	-0.0424989	-0.0926941	0.8899	0.99995	no

ENSG00000109445	ENSG00000109445	ZNF330	4:142142040-142155851	q1	q2	OK	23.2316	23.1905	-0.00255653	-0.00483908	0.9939	0.99995	no

ENSG00000109452	ENSG00000109452	INPP4B	4:142944312-143768585	q1	q2	OK	9.05386	10.2273	0.175819	0.224997	0.57455	0.99995	no

ENSG00000109458	ENSG00000109458	GAB1	4:144256417-144395721	q1	q2	OK	14.8418	14.1742	-0.0663946	-0.0964208	0.8603	0.99995	no

ENSG00000109466	ENSG00000109466	KLHL2	4:165995573-166244308	q1	q2	OK	9.54947	10.2911	0.107906	0.168922	0.79425	0.99995	no

ENSG00000109471	ENSG00000109471	IL2	4:123372624-123377880	q1	q2	NOTEST	0	0	0	0	1	1	no

ENSG00000109472	ENSG00000109472	CPE	4:166282345-166419472	q1	q2	OK	73.8003	67.8981	-0.120255	-0.212895	0.74755	0.99995	no

ENSG00000109475	ENSG00000109475	RPL34	4:109541721-109551568	q1	q2	OK	818.439	819.326	0.00156223	0.00374087	0.99585	0.99995	no

ENSG00000109501	ENSG00000109501	WFS1	4:6271575-6304992	q1	q2	OK	14.0541	11.2257	-0.324187	-0.582676	0.3102	0.99995	no

ENSG00000109511	ENSG00000109511	ANXA10	4:168867824-169108841	q1	q2	NOTEST	0	0	0	0	1	1	no

ENSG00000109519	ENSG00000109519	GRPEL1	4:7060632-7069924	q1	q2	OK	15.6849	15.4878	-0.018245	-0.0291824	0.96555	0.99995	no

ENSG00000109534	ENSG00000109534	GAR1	4:110736665-110745893	q1	q2	OK	27.3682	31.6246	0.208547	0.417176	0.5537	0.99995	no

ENSG00000109536	ENSG00000109536	FRG1	4:190861942-190884359	q1	q2	OK	5.96011	2.27395	-1.39014	-1.51447	0.02685	0.99995	no

ENSG00000109572	ENSG00000109572	CLCN3	4:170533783-170644824	q1	q2	OK	19.2239	19.8367	0.0452687	0.0908058	0.87865	0.99995	no

ENSG00000109576	ENSG00000109576	AADAT	4:170981372-171012850	q1	q2	OK	20.5179	21.6785	0.0793806	0.152194	0.8202	0.99995	no

ENSG00000109586	ENSG00000109586	GALNT7	4:174053078-174250845	q1	q2	OK	22.8591	26.8023	0.229585	0.430593	0.4878	0.99995	no

ENSG00000109606	ENSG00000109606	DHX15	4:24519063-24586173	q1	q2	OK	127.181	142.25	0.161545	0.330469	0.5805	0.99995	no

ENSG00000109610	ENSG00000109610	SOD3	4:24791533-24802464	q1	q2	NOTEST	0.261577	0.153653	-0.767559	0	1	1	no

ENSG00000109618	ENSG00000109618	SEPSECS	4:25121626-25162204	q1	q2	OK	4.59195	5.01864	0.12819	0.172057	0.7222	0.99995	no

ENSG00000109625	ENSG00000109625	CPZ	4:8560451-8621488	q1	q2	NOTEST	0	0.0492549	inf	0	1	1	no

ENSG00000109654	ENSG00000109654	TRIM2	4:154073493-154260472	q1	q2	OK	48.2651	47.5351	-0.0219879	-0.0314581	0.93985	0.99995	no

ENSG00000109667	ENSG00000109667	SLC2A9	4:9772776-10056560	q1	q2	NOTEST	0.146373	0.0959932	-0.608643	0	1	1	no

ENSG00000109670	ENSG00000109670	FBXW7	4:153242409-153457253	q1	q2	OK	24.8719	24.0768	-0.0468699	-0.0794759	0.8838	0.99995	no

ENSG00000109674	ENSG00000109674	NEIL3	4:178230989-178284097	q1	q2	OK	7.92871	8.24871	0.0570824	0.104006	0.8779	0.99995	no

ENSG00000109680	ENSG00000109680	TBC1D19	4:26578058-26756973	q1	q2	OK	6.8306	6.02923	-0.180038	-0.261356	0.68895	0.99995	no

ENSG00000109684	ENSG00000109684	CLNK	4:10488018-10686489	q1	q2	NOTEST	0	0	0	0	1	1	no

ENSG00000109685	ENSG00000109685	WHSC1	4:1873150-1983934	q1	q2	OK	85.1564	89.6327	0.0739094	0.117746	0.8149	0.99995	no

ENSG00000109686	ENSG00000109686	SH3D19	4:152020724-152246784	q1	q2	OK	10.2051	12.3392	0.273952	0.0782076	0.78135	0.99995	no

ENSG00000109689	ENSG00000109689	STIM2	4:26859299-27027003	q1	q2	OK	19.8118	22.7941	0.202301	0.246718	0.6219	0.99995	no

ENSG00000109705	ENSG00000109705	NKX3-2	4:13542453-13546674	q1	q2	OK	0.86033	1.07131	0.316417	0.350757	0.61015	0.99995	no

ENSG00000109736	ENSG00000109736	MFSD10	4:2932287-2936586	q1	q2	OK	34.2828	30.3507	-0.175756	-0.348761	0.60925	0.99995	no

ENSG00000109738	ENSG00000109738	GLRB	4:157997208-158093242	q1	q2	OK	4.88123	4.19023	-0.220215	-0.316814	0.62495	0.99995	no

ENSG00000109743	ENSG00000109743	BST1	4:15606161-15739936	q1	q2	OK	0.410439	0.572172	0.479279	0.0787223	0.72295	0.99995	no

ENSG00000109756	ENSG00000109756	RAPGEF2	4:160025329-160281321	q1	q2	OK	45.1868	43.1614	-0.0661573	-0.0873808	0.8831	0.99995	no

ENSG00000109758	ENSG00000109758	HGFAC	4:3443613-3451211	q1	q2	OK	2.1164	1.15049	-0.879357	-0.829344	0.27185	0.99995	no

ENSG00000109762	ENSG00000109762	SNX25	4:186080818-186317053	q1	q2	OK	4.68254	4.65256	-0.00926626	-0.00904935	0.98945	0.99995	no

ENSG00000109771	ENSG00000109771	LRP2BP	4:186080818-186317053	q1	q2	OK	0.784281	0.799623	0.027949	0.0134808	0.983	0.99995	no

ENSG00000109775	ENSG00000109775	UFSP2	4:186317174-186347139	q1	q2	OK	20.4505	21.996	0.105106	0.204172	0.75665	0.99995	no

ENSG00000109787	ENSG00000109787	KLF3	4:38614321-38702663	q1	q2	OK	24.2046	27.3645	0.177021	0.413839	0.54175	0.99995	no

ENSG00000109790	ENSG00000109790	KLHL5	4:39046658-39128477	q1	q2	OK	23.5232	23.9485	0.0258506	0.0566191	0.93565	0.99995	no

ENSG00000109794	ENSG00000109794	FAM149A	4:187025572-187093821	q1	q2	OK	1.51333	1.82526	0.270373	0.206286	0.68625	0.99995	no

ENSG00000109805	ENSG00000109805	NCAPG	4:17812524-18023499	q1	q2	OK	46.3854	51.4772	0.150264	0.237095	0.65945	0.99995	no

ENSG00000109814	ENSG00000109814	UGDH	4:39500374-39640710	q1	q2	OK	25.6258	29.1213	0.184475	0.286945	0.6293	0.99995	no

ENSG00000109819	ENSG00000109819	PPARGC1A	4:23562545-23905712	q1	q2	OK	0.665274	0.575087	-0.210169	-0.112182	0.6936	0.99995	no

ENSG00000109832	ENSG00000109832	DDX25	11:125773141-125808811	q1	q2	OK	3.05198	2.7249	-0.163541	-0.184727	0.77345	0.99995	no

ENSG00000109846	ENSG00000109846	CRYAB	11:111779288-111797596	q1	q2	NOTEST	0	0	0	0	1	1	no

ENSG00000109851	ENSG00000109851	DBX1	11:20177700-20182159	q1	q2	OK	0.707071	0.604028	-0.227239	-0.218935	0.7554	0.99995	no

ENSG00000109854	ENSG00000109854	HTATIP2	11:20385230-20405329	q1	q2	NOTEST	0.342269	0.196631	-0.799645	0	1	1	no

ENSG00000109861	ENSG00000109861	CTSC	11:88026759-88070955	q1	q2	OK	12.5345	13.63	0.120871	0.188675	0.74435	0.99995	no

ENSG00000109881	ENSG00000109881	CCDC34	11:27352373-27385415	q1	q2	OK	27.296	31.432	0.203544	0.350795	0.6165	0.99995	no

ENSG00000109906	ENSG00000109906	ZBTB16	11:113930314-114227293	q1	q2	OK	6.76106	5.6009	-0.271591	-0.396455	0.5369	0.99995	no

ENSG00000109911	ENSG00000109911	ELP4	11:31531296-31805546	q1	q2	OK	10.1391	11.4781	0.178951	0.324175	0.64645	0.99995	no

ENSG00000109917	ENSG00000109917	ZNF259	11:116648435-116658766	q1	q2	OK	13.1999	13.4046	0.022199	0.0405172	0.9479	0.99995	no

ENSG00000109919	ENSG00000109919	MTCH2	11:47638866-47664175	q1	q2	OK	60.6696	60.5436	-0.00300025	-0.00561643	0.993	0.99995	no

ENSG00000109920	ENSG00000109920	FNBP4	11:47738071-47788995	q1	q2	OK	67.2404	64.0337	-0.0704964	-0.0967002	0.8152	0.99995	no

ENSG00000109927	ENSG00000109927	TECTA	11:120971881-121062202	q1	q2	OK	1.34533	1.14416	-0.233669	-0.280444	0.61555	0.99995	no

ENSG00000109929	ENSG00000109929	SC5D	11:121163161-121179403	q1	q2	OK	68.2859	82.6203	0.274907	0.617834	0.36895	0.99995	no

ENSG00000109943	ENSG00000109943	CRTAM	11:122709207-122743347	q1	q2	NOTEST	0	0.0597667	inf	0	1	1	no

ENSG00000109944	ENSG00000109944	C11orf63	11:122753390-122830506	q1	q2	OK	0.936729	0.762502	-0.29689	-0.305501	0.67025	0.99995	no

ENSG00000109956	ENSG00000109956	B3GAT1	11:134248397-134281812	q1	q2	OK	21.2001	21.09	-0.00751262	-0.017133	0.9796	0.99995	no

ENSG00000109971	ENSG00000109971	HSPA8	11:122928196-122933938	q1	q2	OK	854.296	905.748	0.0843732	0.116493	0.83695	0.99995	no

ENSG00000109991	ENSG00000109991	P2RX3	11:57105847-57138073	q1	q2	OK	5.1053	6.00504	0.234178	0.228518	0.7178	0.99995	no

ENSG00000110002	ENSG00000110002	VWA5A	11:123986068-124018428	q1	q2	OK	6.96033	5.82866	-0.255992	-0.390764	0.56225	0.99995	no

ENSG00000110011	ENSG00000110011	DNAJC4	11:63974149-64001824	q1	q2	OK	13.4278	10.2361	-0.391554	-0.402468	0.5624	0.99995	no

ENSG00000110013	ENSG00000110013	SIAE	11:124492731-124567414	q1	q2	OK	3.67634	3.82264	0.0563015	0.0461832	0.9476	0.99995	no

ENSG00000110025	ENSG00000110025	SNX15	11:64781584-64808044	q1	q2	OK	16.7063	14.3961	-0.214718	-0.318817	0.62925	0.99995	no

ENSG00000110031	ENSG00000110031	LPXN	11:58294343-58345693	q1	q2	OK	2.57933	2.54597	-0.0187835	-0.0241216	0.97345	0.99995	no

ENSG00000110042	ENSG00000110042	DTX4	11:58938902-58980424	q1	q2	OK	99.7228	86.1956	-0.210309	-0.352831	0.4982	0.99995	no

ENSG00000110046	ENSG00000110046	ATG2A	11:64662006-64684722	q1	q2	OK	4.21271	3.66712	-0.200101	-0.233173	0.59235	0.99995	no

ENSG00000110047	ENSG00000110047	EHD1	11:64619113-64655768	q1	q2	OK	23.9826	20.9863	-0.192546	-0.352015	0.5574	0.99995	no

ENSG00000110048	ENSG00000110048	OSBP	11:59341870-59383617	q1	q2	OK	17.4779	17.3672	-0.00916652	-0.0198522	0.97445	0.99995	no

ENSG00000110057	ENSG00000110057	UNC93B1	11:67758574-67772452	q1	q2	OK	3.52228	2.48847	-0.501247	-0.595162	0.29285	0.99995	no

ENSG00000110060	ENSG00000110060	PUS3	11:125753508-125773116	q1	q2	OK	10.0674	10.226	0.022542	0.0368118	0.95705	0.99995	no

ENSG00000110063	ENSG00000110063	DCPS	11:126152959-126215644	q1	q2	OK	12.7472	12.2521	-0.057147	-0.0887912	0.8974	0.99995	no

ENSG00000110066	ENSG00000110066	SUV420H1	11:67922329-67981295	q1	q2	OK	29.1442	30.7382	0.0768255	0.132154	0.81225	0.99995	no

ENSG00000110074	ENSG00000110074	FOXRED1	11:126071992-126148026	q1	q2	OK	30.3313	26.4546	-0.197289	-0.166526	0.71395	0.99995	no

ENSG00000110075	ENSG00000110075	PPP6R3	11:68228185-68384179	q1	q2	OK	47.1691	51.2199	0.118861	0.178469	0.6868	0.99995	no

ENSG00000110076	ENSG00000110076	NRXN2	11:64373645-64490660	q1	q2	OK	15.6845	15.404	-0.0260353	-0.0339885	0.94475	0.99995	no

ENSG00000110077	ENSG00000110077	MS4A6A	11:59939080-59952139	q1	q2	NOTEST	0	0.0103813	inf	0	1	1	no

ENSG00000110079	ENSG00000110079	MS4A4A	11:60048013-60076445	q1	q2	NOTEST	0	0	0	0	1	1	no

ENSG00000110080	ENSG00000110080	ST3GAL4	11:126225534-126875953	q1	q2	OK	8.81356	9.1607	0.0557334	0.0821133	0.90475	0.99995	no

ENSG00000110090	ENSG00000110090	CPT1A	11:68522087-68611878	q1	q2	OK	2.89009	2.82408	-0.0333366	-0.0527176	0.92905	0.99995	no

ENSG00000110092	ENSG00000110092	CCND1	11:69455854-69490184	q1	q2	OK	70.1931	74.9146	0.0939181	0.184091	0.75325	0.99995	no

ENSG00000110104	ENSG00000110104	CCDC86	11:60603468-60623444	q1	q2	OK	11.3224	11.512	0.0239519	0.0444975	0.9461	0.99995	no

ENSG00000110107	ENSG00000110107	PRPF19	11:60658201-60674060	q1	q2	OK	79.9961	78.9871	-0.0183115	-0.0441922	0.94625	0.99995	no

ENSG00000110108	ENSG00000110108	TMEM109	11:60680637-60720002	q1	q2	OK	32.1668	29.0135	-0.148844	-0.120696	0.867	0.99995	no

ENSG00000110148	ENSG00000110148	CCKBR	11:6280965-6293357	q1	q2	NOTEST	0.0163164	0.01607	-0.0219576	0	1	1	no

ENSG00000110169	ENSG00000110169	HPX	11:6452278-6463847	q1	q2	OK	0.949145	0.774539	-0.29329	-0.172404	0.66695	0.99995	no

ENSG00000110171	ENSG00000110171	TRIM3	11:6469842-6495689	q1	q2	OK	15.1567	11.8556	-0.354386	-0.434712	0.48095	0.99995	no

ENSG00000110172	ENSG00000110172	CHORDC1	11:89934327-89956532	q1	q2	OK	34.1104	38.9609	0.191817	0.353572	0.57585	0.99995	no

ENSG00000110195	ENSG00000110195	FOLR1	11:71874365-71920285	q1	q2	NOTEST	0.031768	0	#NAME?	0	1	1	no

ENSG00000110200	ENSG00000110200	ANAPC15	11:71709586-71823826	q1	q2	OK	45.6298	48.0211	0.0736903	0.0391016	0.9297	0.99995	no

ENSG00000110203	ENSG00000110203	FOLR3	11:71825914-71850936	q1	q2	NOTEST	0.0433854	0	#NAME?	0	1	1	no

ENSG00000110218	ENSG00000110218	PANX1	11:93862093-93915138	q1	q2	OK	21.6745	23.9027	0.141173	0.328433	0.6423	0.99995	no

ENSG00000110237	ENSG00000110237	ARHGEF17	11:73018279-73080136	q1	q2	OK	33.6923	31.2624	-0.107989	-0.119884	0.8199	0.99995	no

ENSG00000110243	ENSG00000110243	APOA5	11:116660082-116663136	q1	q2	NOTEST	0	0	0	0	1	1	no

ENSG00000110244	ENSG00000110244	APOA4	11:116691418-116694022	q1	q2	NOTEST	0	0	0	0	1	1	no

ENSG00000110245	ENSG00000110245	APOC3	11:116700421-116703788	q1	q2	NOTEST	0	0	0	0	1	1	no

ENSG00000110274	ENSG00000110274	CEP164	11:117103340-117283984	q1	q2	OK	15.8557	14.0354	-0.175932	-0.144069	0.7561	0.99995	no

ENSG00000110315	ENSG00000110315	RNF141	11:10533224-10562777	q1	q2	OK	14.4891	15.4896	0.09634	0.21393	0.75175	0.99995	no

ENSG00000110318	ENSG00000110318	KIAA1377	11:101761404-101871789	q1	q2	OK	1.27092	1.59969	0.331926	0.335113	0.54605	0.99995	no

ENSG00000110321	ENSG00000110321	EIF4G2	11:10804859-10830657	q1	q2	OK	527.811	602.862	0.191809	0.27642	0.62395	0.99995	no

ENSG00000110324	ENSG00000110324	IL10RA	11:117857062-117872196	q1	q2	NOTEST	0.378793	0.223882	-0.758673	0	1	1	no

ENSG00000110328	ENSG00000110328	GALNT18	11:11292422-11643552	q1	q2	OK	5.57514	5.16166	-0.111174	-0.135153	0.8449	0.99995	no

ENSG00000110330	ENSG00000110330	BIRC2	11:102217941-102249401	q1	q2	OK	27.6032	30.3185	0.135365	0.295921	0.65085	0.99995	no

ENSG00000110344	ENSG00000110344	UBE4A	11:118230299-118302211	q1	q2	OK	2.56476	2.80532	0.129343	0.215908	0.7635	0.99995	no

ENSG00000110347	ENSG00000110347	MMP12	11:102733466-102745764	q1	q2	NOTEST	0	0	0	0	1	1	no

ENSG00000110367	ENSG00000110367	DDX6	11:118575315-118661858	q1	q2	OK	17.8957	19.3025	0.109176	0.165343	0.80915	0.99995	no

ENSG00000110375	ENSG00000110375	UPK2	11:118754474-118829269	q1	q2	NOTEST	0.132567	0	#NAME?	0	1	1	no

ENSG00000110395	ENSG00000110395	CBL	11:119076751-119178859	q1	q2	OK	16.5606	18.4734	0.157694	0.385713	0.5858	0.99995	no

ENSG00000110400	ENSG00000110400	PVRL1	11:119479134-119599794	q1	q2	OK	19.0412	16.9637	-0.166671	-0.276175	0.57635	0.99995	no

ENSG00000110422	ENSG00000110422	HIPK3	11:33278217-33378569	q1	q2	OK	6.58598	6.72197	0.0294873	0.0626687	0.92165	0.99995	no

ENSG00000110427	ENSG00000110427	KIAA1549L	11:33563617-33718247	q1	q2	OK	4.16581	4.01671	-0.0525839	-0.108706	0.8717	0.99995	no

ENSG00000110429	ENSG00000110429	FBXO3	11:33762484-33796089	q1	q2	OK	16.9038	17.7181	0.0678795	0.109551	0.8416	0.99995	no

ENSG00000110435	ENSG00000110435	PDHX	11:34874640-35042138	q1	q2	OK	16.9476	16.9226	-0.00212585	-0.00309324	0.9956	0.99995	no

ENSG00000110436	ENSG00000110436	SLC1A2	11:35272752-35442549	q1	q2	OK	2.44883	2.47348	0.0144472	0.0232679	0.96735	0.99995	no

ENSG00000110442	ENSG00000110442	COMMD9	11:36295050-36310999	q1	q2	OK	23.8063	21.2544	-0.163582	-0.283517	0.662	0.99995	no

ENSG00000110446	ENSG00000110446	SLC15A3	11:60680637-60720002	q1	q2	OK	8.73866	6.48531	-0.430236	-0.151762	0.6553	0.99995	no

ENSG00000110448	ENSG00000110448	CD5	11:60869866-60895324	q1	q2	NOTEST	0.0766508	0.0081693	-3.23002	0	1	1	no

ENSG00000110455	ENSG00000110455	ACCS	11:44087474-44105772	q1	q2	NOTEST	0.0937404	0.115707	0.303739	0	1	1	no

ENSG00000110484	ENSG00000110484	SCGB2A2	11:62037626-62040628	q1	q2	NOTEST	0	0.0209033	inf	0	1	1	no

ENSG00000110492	ENSG00000110492	MDK	11:46402305-46405375	q1	q2	OK	503.165	459.627	-0.130568	-0.311763	0.6574	0.99995	no

ENSG00000110497	ENSG00000110497	AMBRA1	11:46417963-46615675	q1	q2	OK	11.2238	10.1697	-0.142287	-0.196891	0.69205	0.99995	no

ENSG00000110514	ENSG00000110514	MADD	11:47290711-47351582	q1	q2	OK	28.2542	26.2652	-0.105314	-0.149328	0.8244	0.99995	no

ENSG00000110536	ENSG00000110536	PTPMT1	11:47487495-47606114	q1	q2	OK	11.9612	9.50624	-0.331413	-0.124133	0.7694	0.99995	no

ENSG00000110583	ENSG00000110583	NAA40	11:63706430-63724800	q1	q2	OK	30.848	29.3902	-0.069841	-0.125912	0.8142	0.99995	no

ENSG00000110619	ENSG00000110619	CARS	11:3022151-3078843	q1	q2	OK	30.6167	32.1514	0.070561	0.143011	0.83415	0.99995	no

ENSG00000110628	ENSG00000110628	SLC22A18	11:2909009-2946476	q1	q2	OK	1.73714	1.48307	-0.228127	-0.180694	0.73955	0.99995	no

ENSG00000110651	ENSG00000110651	CD81	11:2349978-2418649	q1	q2	OK	145.485	131.706	-0.143545	-0.334245	0.6281	0.99995	no

ENSG00000110660	ENSG00000110660	SLC35F2	11:107661716-107799019	q1	q2	OK	1.46535	1.21993	-0.264448	-0.312151	0.5919	0.99995	no

ENSG00000110665	ENSG00000110665	C11orf21	11:2316874-2339430	q1	q2	NOTEST	0.0214756	0	#NAME?	0	1	1	no

ENSG00000110675	ENSG00000110675	ELMOD1	11:107461816-107537505	q1	q2	OK	1.04762	1.06407	0.0224799	0.0212073	0.97215	0.99995	no

ENSG00000110680	ENSG00000110680	CALCA	11:14926542-15103888	q1	q2	NOTEST	0	0	0	0	1	1	no

ENSG00000110693	ENSG00000110693	SOX6	11:15987994-16778428	q1	q2	OK	0.730202	0.744385	0.0277539	0.00478896	0.98105	0.99995	no

ENSG00000110696	ENSG00000110696	C11orf58	11:15987994-16778428	q1	q2	OK	207.194	226.631	0.129366	0.275916	0.6603	0.99995	no

ENSG00000110697	ENSG00000110697	PITPNM1	11:67259238-67273734	q1	q2	OK	17.8958	16.832	-0.0884129	-0.117008	0.77475	0.99995	no

ENSG00000110700	ENSG00000110700	RPS13	11:17095935-17229530	q1	q2	OK	604.577	617.765	0.0311301	0.0528345	0.9421	0.99995	no

ENSG00000110711	ENSG00000110711	AIP	11:67250511-67258574	q1	q2	OK	32.6357	31.1564	-0.0669199	-0.136843	0.844	0.99995	no

ENSG00000110713	ENSG00000110713	NUP98	11:3686816-3847601	q1	q2	OK	31.02	30.5462	-0.0222069	-0.0239676	0.94575	0.99995	no

ENSG00000110717	ENSG00000110717	NDUFS8	11:67798083-67804111	q1	q2	OK	65.1488	58.6217	-0.152304	-0.304839	0.64075	0.99995	no

ENSG00000110719	ENSG00000110719	TCIRG1	11:67806482-67888911	q1	q2	OK	1.58339	1.3441	-0.236376	-0.0806592	0.74185	0.99995	no

ENSG00000110721	ENSG00000110721	CHKA	11:67806482-67888911	q1	q2	OK	29.7386	26.7016	-0.155411	-0.268965	0.68535	0.99995	no

ENSG00000110723	ENSG00000110723	EXPH5	11:108376157-108464465	q1	q2	NOTEST	0.174482	0.158663	-0.13711	0	1	1	no

ENSG00000110756	ENSG00000110756	HPS5	11:18300222-18343745	q1	q2	OK	8.87295	9.84183	0.149513	0.24221	0.6434	0.99995	no

ENSG00000110768	ENSG00000110768	GTF2H1	11:18343841-18388591	q1	q2	OK	20.7855	20.7957	0.00070332	0.00124404	0.99845	0.99995	no

ENSG00000110777	ENSG00000110777	POU2AF1	11:111222976-111328876	q1	q2	NOTEST	0.00766691	0.0079742	0.0566941	0	1	1	no

ENSG00000110786	ENSG00000110786	PTPN5	11:18714668-18814268	q1	q2	OK	2.15204	1.63725	-0.394433	-0.42354	0.5549	0.99995	no

ENSG00000110799	ENSG00000110799	VWF	12:6058039-6233936	q1	q2	NOTEST	0.0282076	0.0134805	-1.06521	0	1	1	no

ENSG00000110801	ENSG00000110801	PSMD9	12:122326636-122441833	q1	q2	OK	37.4008	33.8351	-0.144551	-0.247752	0.7271	0.99995	no

ENSG00000110811	ENSG00000110811	LEPREL2	12:6930710-6949018	q1	q2	NOTEST	0	0	0	0	1	1	no

ENSG00000110841	ENSG00000110841	PPFIBP1	12:27676363-27848497	q1	q2	OK	10.7891	11.0248	0.0311893	0.0385581	0.9414	0.99995	no

ENSG00000110844	ENSG00000110844	PRPF40B	12:49962000-50158717	q1	q2	OK	9.48145	7.87773	-0.267329	-0.0631735	0.7095	0.99995	no

ENSG00000110848	ENSG00000110848	CD69	12:9905081-9913497	q1	q2	NOTEST	0.0489661	0.0578125	0.239597	0	1	1	no

ENSG00000110851	ENSG00000110851	PRDM4	12:108126642-108155049	q1	q2	OK	16.4627	16.2751	-0.0165308	-0.027037	0.9582	0.99995	no

ENSG00000110852	ENSG00000110852	CLEC2B	12:10005582-10022735	q1	q2	NOTEST	0.247566	0.247725	0.000928009	0	1	1	no

ENSG00000110871	ENSG00000110871	COQ5	12:120941076-121015397	q1	q2	OK	33.9714	32.4439	-0.0663742	-0.0749131	0.9099	0.99995	no

ENSG00000110876	ENSG00000110876	SELPLG	12:109015685-109035094	q1	q2	NOTEST	0.447033	0.283526	-0.656899	0	1	1	no

ENSG00000110880	ENSG00000110880	CORO1C	12:109038884-109125372	q1	q2	OK	77.1683	84.946	0.138539	0.285128	0.61265	0.99995	no

ENSG00000110881	ENSG00000110881	ASIC1	12:50451330-50477394	q1	q2	OK	11.6334	9.98526	-0.220397	-0.302546	0.54265	0.99995	no

ENSG00000110887	ENSG00000110887	DAO	12:109252707-109294819	q1	q2	NOTEST	0.0410939	0.0777529	0.919972	0	1	1	no

ENSG00000110888	ENSG00000110888	CAPRIN2	12:30862485-30907885	q1	q2	OK	9.47324	9.89299	0.0625484	0.0879297	0.8588	0.99995	no

ENSG00000110900	ENSG00000110900	TSPAN11	12:31079361-31149534	q1	q2	OK	19.0278	15.1022	-0.333349	-0.714069	0.25735	0.99995	no

ENSG00000110906	ENSG00000110906	KCTD10	12:109785707-109974507	q1	q2	OK	31.6208	35.1609	0.153096	0.268591	0.65445	0.99995	no

ENSG00000110911	ENSG00000110911	SLC11A2	12:51373183-51422349	q1	q2	OK	18.2365	19.3291	0.0839437	0.132661	0.77335	0.99995	no

ENSG00000110917	ENSG00000110917	MLEC	12:121124671-121139667	q1	q2	OK	63.3588	63.1189	-0.00547382	-0.00676121	0.9874	0.99995	no

ENSG00000110921	ENSG00000110921	MVK	12:109991541-110035067	q1	q2	OK	25.5014	27.9351	0.131499	0.181327	0.76795	0.99995	no

ENSG00000110925	ENSG00000110925	CSRNP2	12:51454989-51477447	q1	q2	OK	23.5461	23.2009	-0.0213025	-0.0353618	0.94655	0.99995	no

ENSG00000110931	ENSG00000110931	CAMKK2	12:121675496-121736111	q1	q2	OK	13.6804	14.3829	0.0722408	0.156567	0.8167	0.99995	no

ENSG00000110934	ENSG00000110934	BIN2	12:51674821-51718452	q1	q2	NOTEST	0	0	0	0	1	1	no

ENSG00000110944	ENSG00000110944	IL23A	12:56732662-56734193	q1	q2	NOTEST	0.144246	0.0585175	-1.30159	0	1	1	no

ENSG00000110955	ENSG00000110955	ATP5B	12:57031958-57039798	q1	q2	OK	346.218	345.824	-0.00164283	-0.0037004	0.99615	0.99995	no

ENSG00000110958	ENSG00000110958	PTGES3	12:57057126-57082159	q1	q2	OK	207.788	223.735	0.106678	0.254006	0.71155	0.99995	no

ENSG00000110975	ENSG00000110975	SYT10	12:33527172-33592754	q1	q2	NOTEST	0.165295	0.330413	0.999233	0	1	1	no

ENSG00000110987	ENSG00000110987	BCL7A	12:122445339-122499948	q1	q2	OK	20.4726	20.5856	0.00794122	0.0157546	0.9818	0.99995	no

ENSG00000111011	ENSG00000111011	RSRC2	12:122989189-123011547	q1	q2	OK	95.6965	101.812	0.0893729	0.186797	0.74515	0.99995	no

ENSG00000111012	ENSG00000111012	CYP27B1	12:58156116-58212487	q1	q2	OK	0.728509	1.24024	0.767595	0.218448	0.51125	0.99995	no

ENSG00000111046	ENSG00000111046	MYF6	12:81101276-81103253	q1	q2	NOTEST	0.0385517	0.0559601	0.537606	0	1	1	no

ENSG00000111049	ENSG00000111049	MYF5	12:81110690-81113452	q1	q2	NOTEST	0	0	0	0	1	1	no

ENSG00000111052	ENSG00000111052	LIN7A	12:81186298-81650533	q1	q2	OK	6.24274	6.17653	-0.0153828	-0.0269618	0.96415	0.99995	no

ENSG00000111057	ENSG00000111057	KRT18	12:53290976-53346686	q1	q2	OK	1.61636	1.25453	-0.365595	-0.338781	0.57665	0.99995	no

ENSG00000111058	ENSG00000111058	ACSS3	12:81186298-81650533	q1	q2	OK	1.20207	1.24446	0.0500023	0.02136	0.9685	0.99995	no

ENSG00000111077	ENSG00000111077	TENC1	12:53399941-53458156	q1	q2	OK	8.60845	7.02926	-0.29238	-0.0858809	0.80225	0.99995	no

ENSG00000111087	ENSG00000111087	GLI1	12:57853917-57914300	q1	q2	OK	1.72563	1.53484	-0.169034	-0.0272926	0.881	0.99995	no

ENSG00000111110	ENSG00000111110	PPM1H	12:63037761-63328817	q1	q2	OK	1.29972	1.28239	-0.0193663	-0.0213521	0.96495	0.99995	no

ENSG00000111142	ENSG00000111142	METAP2	12:95867295-95909615	q1	q2	OK	82.8242	90.5377	0.128465	0.214246	0.64355	0.99995	no

ENSG00000111144	ENSG00000111144	LTA4H	12:96390298-96437298	q1	q2	OK	34.128	33.8356	-0.0124118	-0.0266651	0.9661	0.99995	no

ENSG00000111145	ENSG00000111145	ELK3	12:96588159-96663613	q1	q2	OK	14.2859	16.1793	0.179561	0.345108	0.57775	0.99995	no

ENSG00000111181	ENSG00000111181	SLC6A12	12:299242-323736	q1	q2	NOTEST	0.13111	0.202806	0.629327	0	1	1	no

ENSG00000111186	ENSG00000111186	WNT5B	12:1639056-1756409	q1	q2	OK	7.83491	6.38286	-0.295715	-0.278794	0.58465	0.99995	no

ENSG00000111196	ENSG00000111196	MAGOHB	12:10758611-10766222	q1	q2	OK	32.2499	35.8368	0.152145	0.279877	0.6927	0.99995	no

ENSG00000111199	ENSG00000111199	TRPV4	12:110220889-110271241	q1	q2	NOTEST	0.211194	0.175853	-0.264202	0	1	1	no

ENSG00000111203	ENSG00000111203	ITFG2	12:2904118-3050306	q1	q2	OK	19.0612	18.6466	-0.0317255	-0.0145583	0.9657	0.99995	no

ENSG00000111206	ENSG00000111206	FOXM1	12:2904118-3050306	q1	q2	OK	58.7512	58.0759	-0.0166793	-0.0224391	0.9699	0.99995	no

ENSG00000111215	ENSG00000111215	PRR4	12:10977558-11324212	q1	q2	OK	3.60634	2.13493	-0.756344	-0.664316	0.19745	0.99995	no

ENSG00000111218	ENSG00000111218	PRMT8	12:3490514-3703139	q1	q2	OK	8.15526	7.18665	-0.182412	-0.322607	0.64825	0.99995	no

ENSG00000111224	ENSG00000111224	PARP11	12:3900212-4019504	q1	q2	OK	9.97055	9.755	-0.0315311	-0.0421932	0.9297	0.99995	no

ENSG00000111229	ENSG00000111229	ARPC3	12:110810704-110888227	q1	q2	OK	134.624	142.269	0.0796897	0.12874	0.83655	0.99995	no

ENSG00000111231	ENSG00000111231	GPN3	12:110890288-110928190	q1	q2	OK	15.8016	15.8766	0.00682657	0.0101949	0.98775	0.99995	no

ENSG00000111237	ENSG00000111237	VPS29	12:110928901-111021125	q1	q2	OK	84.5783	95.8736	0.180847	0.323464	0.6526	0.99995	no

ENSG00000111241	ENSG00000111241	FGF6	12:4537320-4554780	q1	q2	NOTEST	0	0	0	0	1	1	no

ENSG00000111245	ENSG00000111245	MYL2	12:111348622-111358526	q1	q2	NOTEST	0	0	0	0	1	1	no

ENSG00000111247	ENSG00000111247	RAD51AP1	12:4647949-4669214	q1	q2	OK	33.4531	33.4334	-0.000851537	-0.00165744	0.99775	0.99995	no

ENSG00000111249	ENSG00000111249	CUX2	12:111471827-111788358	q1	q2	OK	5.86484	5.89517	0.00743972	0.0145402	0.98195	0.99995	no

ENSG00000111252	ENSG00000111252	SH2B3	12:111843751-111889427	q1	q2	OK	4.93436	5.44443	0.14192	0.282189	0.69145	0.99995	no

ENSG00000111254	ENSG00000111254	AKAP3	12:4671369-4960277	q1	q2	OK	1.95904	1.55592	-0.332381	-0.0876557	0.82665	0.99995	no

ENSG00000111261	ENSG00000111261	MANSC1	12:12482197-12503475	q1	q2	OK	2.741	3.09225	0.173953	0.235702	0.7333	0.99995	no

ENSG00000111262	ENSG00000111262	KCNA1	12:5019070-5040527	q1	q2	OK	2.05161	3.09224	0.591895	0.417674	0.3119	0.99995	no

ENSG00000111266	ENSG00000111266	DUSP16	12:12628828-12715317	q1	q2	OK	7.8964	7.09991	-0.153395	-0.290421	0.65805	0.99995	no

ENSG00000111269	ENSG00000111269	CREBL2	12:12764760-12798042	q1	q2	OK	8.27392	8.46445	0.032846	0.0610144	0.92295	0.99995	no

ENSG00000111271	ENSG00000111271	ACAD10	12:112123856-112247782	q1	q2	OK	15.9421	14.446	-0.142168	-0.140426	0.75495	0.99995	no

ENSG00000111275	ENSG00000111275	ALDH2	12:112123856-112247782	q1	q2	OK	30.5074	27.3105	-0.159706	-0.303075	0.669	0.99995	no

ENSG00000111276	ENSG00000111276	CDKN1B	12:12867991-12875305	q1	q2	OK	43.4342	45.7026	0.073442	0.176596	0.80325	0.99995	no

ENSG00000111291	ENSG00000111291	GPRC5D	12:13080659-13193613	q1	q2	NOTEST	0	0	0	0	1	1	no

ENSG00000111300	ENSG00000111300	NAA25	12:112464499-112546826	q1	q2	OK	12.1504	13.3194	0.132519	0.255767	0.6493	0.99995	no

ENSG00000111305	ENSG00000111305	GSG1	12:13197217-13295455	q1	q2	OK	0.457247	0.553111	0.274593	0.0772538	0.78315	0.99995	no

ENSG00000111319	ENSG00000111319	SCNN1A	12:6456008-6500733	q1	q2	NOTEST	0.0887145	0.117083	0.400293	0	1	1	no

ENSG00000111321	ENSG00000111321	LTBR	12:6456008-6500733	q1	q2	NOTEST	0.242099	0.166399	-0.540953	0	1	1	no

ENSG00000111325	ENSG00000111325	OGFOD2	12:123405497-123467456	q1	q2	OK	6.61557	7.46182	0.173662	0.113862	0.8107	0.99995	no

ENSG00000111328	ENSG00000111328	CDK2AP1	12:123636866-123756881	q1	q2	OK	194.496	221.74	0.189131	0.365119	0.60755	0.99995	no

ENSG00000111331	ENSG00000111331	OAS3	12:113344581-113455556	q1	q2	NOTEST	0.118017	0.159305	0.43279	0	1	1	no

ENSG00000111335	ENSG00000111335	OAS2	12:113344581-113455556	q1	q2	NOTEST	0.223544	0.285775	0.354322	0	1	1	no

ENSG00000111339	ENSG00000111339	ART4	12:14956505-15059520	q1	q2	NOTEST	0	0.0246945	inf	0	1	1	no

ENSG00000111341	ENSG00000111341	MGP	12:14956505-15059520	q1	q2	OK	15.4162	14.1072	-0.128007	-0.0982876	0.93205	0.99995	no

ENSG00000111344	ENSG00000111344	RASAL1	12:113536623-113574044	q1	q2	NOTEST	0.00656039	0.010716	0.707909	0	1	1	no

ENSG00000111348	ENSG00000111348	ARHGDIB	12:15094950-15114662	q1	q2	OK	5.73407	6.35734	0.148865	0.15086	0.80335	0.99995	no

ENSG00000111358	ENSG00000111358	GTF2H3	12:124118374-124147153	q1	q2	OK	16.5552	17.9274	0.114882	0.181993	0.7399	0.99995	no

ENSG00000111361	ENSG00000111361	EIF2B1	12:124086623-124118313	q1	q2	OK	27.3151	27.7584	0.0232231	0.0400158	0.9552	0.99995	no

ENSG00000111364	ENSG00000111364	DDX55	12:124086623-124118313	q1	q2	OK	21.7074	23.0566	0.0869877	0.098896	0.8405	0.99995	no

ENSG00000111371	ENSG00000111371	SLC38A1	12:46576845-46663800	q1	q2	OK	55.9125	65.8717	0.236488	0.450961	0.42345	0.99995	no

ENSG00000111404	ENSG00000111404	RERGL	12:18233802-18801348	q1	q2	NOTEST	0	0	0	0	1	1	no

ENSG00000111405	ENSG00000111405	ENDOU	12:48099867-48231681	q1	q2	NOTEST	0	0.0749697	inf	0	1	1	no

ENSG00000111412	ENSG00000111412	C12orf49	12:117153592-117175875	q1	q2	OK	28.4556	28.869	0.0208094	0.0456138	0.945	0.99995	no

ENSG00000111424	ENSG00000111424	VDR	12:48235319-48336831	q1	q2	NOTEST	0.111094	0.111816	0.00934902	0	1	1	no

ENSG00000111432	ENSG00000111432	FZD10	12:130647003-130650285	q1	q2	OK	6.71661	6.78238	0.0140578	0.0265754	0.9715	0.99995	no

ENSG00000111445	ENSG00000111445	RFC5	12:118451392-118500235	q1	q2	OK	30.3975	28.2541	-0.105492	-0.134815	0.83485	0.99995	no

ENSG00000111450	ENSG00000111450	STX2	12:131274144-131323811	q1	q2	OK	18.0376	18.7022	0.0521959	0.087986	0.8757	0.99995	no

ENSG00000111452	ENSG00000111452	GPR133	12:131438451-131626014	q1	q2	NOTEST	0.266956	0.152412	-0.808626	0	1	1	no

ENSG00000111481	ENSG00000111481	COPZ1	12:54624723-54745633	q1	q2	OK	100.903	103.462	0.0361388	0.00928797	0.9798	0.99995	no

ENSG00000111490	ENSG00000111490	TBC1D30	12:65174588-65274812	q1	q2	OK	0.884605	0.693577	-0.350979	-0.246696	0.57685	0.99995	no

ENSG00000111530	ENSG00000111530	CAND1	12:67663060-67713731	q1	q2	OK	93.6608	96.2411	0.0392087	0.0543387	0.9079	0.99995	no

ENSG00000111536	ENSG00000111536	IL26	12:68383224-68628466	q1	q2	NOTEST	0	0	0	0	1	1	no

ENSG00000111537	ENSG00000111537	IFNG	12:68383224-68628466	q1	q2	NOTEST	0	0	0	0	1	1	no

ENSG00000111540	ENSG00000111540	RAB5B	12:56367696-56388490	q1	q2	OK	41.3396	40.0533	-0.0456025	-0.0708287	0.9015	0.99995	no

ENSG00000111554	ENSG00000111554	MDM1	12:68666222-68726161	q1	q2	OK	13.0988	13.5028	0.0438174	0.0637627	0.90905	0.99995	no

ENSG00000111581	ENSG00000111581	NUP107	12:69068150-69136785	q1	q2	OK	50.3165	48.7381	-0.0459809	-0.0831513	0.8894	0.99995	no

ENSG00000111596	ENSG00000111596	CNOT2	12:70132460-70748773	q1	q2	OK	48.4984	50.6083	0.0614351	0.0925638	0.8422	0.99995	no

ENSG00000111602	ENSG00000111602	TIMELESS	12:56810902-56843187	q1	q2	OK	50.3899	48.8042	-0.0461302	-0.10175	0.8724	0.99995	no

ENSG00000111605	ENSG00000111605	CPSF6	12:69633316-69668138	q1	q2	OK	63.1944	63.497	0.00689177	0.0130784	0.98355	0.99995	no

ENSG00000111615	ENSG00000111615	KRR1	12:75874459-75905416	q1	q2	OK	19.7574	23.3558	0.241383	0.364253	0.5209	0.99995	no

ENSG00000111639	ENSG00000111639	MRPL51	12:6601149-6641121	q1	q2	OK	164.163	167.431	0.0284396	0.025264	0.9757	0.99995	no

ENSG00000111640	ENSG00000111640	GAPDH	12:6643092-6647537	q1	q2	OK	1447.23	1389.55	-0.058672	-0.105794	0.8774	0.99995	no

ENSG00000111641	ENSG00000111641	NOP2	12:6666028-6677857	q1	q2	OK	25.0535	27.282	0.122939	0.196212	0.7355	0.99995	no

ENSG00000111642	ENSG00000111642	CHD4	12:6679248-6716642	q1	q2	OK	130.926	132.635	0.0187061	0.0176485	0.9583	0.99995	no

ENSG00000111644	ENSG00000111644	ACRBP	12:6747240-6756626	q1	q2	OK	0.955254	1.02642	0.103665	0.0868926	0.88645	0.99995	no

ENSG00000111647	ENSG00000111647	UHRF1BP1L	12:100422232-100536626	q1	q2	OK	8.2565	9.39331	0.186104	0.294999	0.5631	0.99995	no

ENSG00000111652	ENSG00000111652	COPS7A	12:6832906-6841041	q1	q2	OK	20.1123	20.6509	0.0381264	0.0709052	0.91045	0.99995	no

ENSG00000111653	ENSG00000111653	ING4	12:6759445-6772314	q1	q2	OK	43.6684	39.6735	-0.138415	-0.264271	0.6988	0.99995	no

ENSG00000111664	ENSG00000111664	GNB3	12:6949117-6961230	q1	q2	NOTEST	0	0	0	0	1	1	no

ENSG00000111665	ENSG00000111665	CDCA3	12:6949117-6961230	q1	q2	NOTEST	0	0	0	0	1	1	no

ENSG00000111666	ENSG00000111666	CHPT1	12:102090724-102137918	q1	q2	OK	7.21609	7.81835	0.115647	0.170772	0.7922	0.99995	no

ENSG00000111667	ENSG00000111667	USP5	12:6961291-6975796	q1	q2	NOTEST	0.00867424	0	#NAME?	0	1	1	no

ENSG00000111669	ENSG00000111669	TPI1	12:6976282-7032861	q1	q2	NOTEST	0	0.415316	inf	0	1	1	no

ENSG00000111670	ENSG00000111670	GNPTAB	12:102139274-102224716	q1	q2	OK	5.93074	5.95832	0.00669443	0.00793086	0.9853	0.99995	no

ENSG00000111671	ENSG00000111671	SPSB2	12:6976282-7032861	q1	q2	NOTEST	0	0	0	0	1	1	no

ENSG00000111674	ENSG00000111674	ENO2	12:6976282-7032861	q1	q2	NOTEST	0	0	0	0	1	1	no

ENSG00000111676	ENSG00000111676	ATN1	12:7033625-7051484	q1	q2	NOTEST	0	0	0	0	1	1	no

ENSG00000111678	ENSG00000111678	C12orf57	12:7052140-7055166	q1	q2	NOTEST	0	0	0	0	1	1	no

ENSG00000111679	ENSG00000111679	PTPN6	12:7055630-7070479	q1	q2	NOTEST	0	0	0	0	1	1	no

ENSG00000111684	ENSG00000111684	LPCAT3	12:7072407-7178336	q1	q2	OK	0.826143	4.82893	2.54724	1.61964	0.00615	0.99995	no

ENSG00000111696	ENSG00000111696	NT5DC3	12:104164230-104234975	q1	q2	OK	1.24714	1.28355	0.041518	0.0532733	0.9273	0.99995	no

ENSG00000111700	ENSG00000111700	SLCO1B3	12:20963635-21392180	q1	q2	NOTEST	0	0	0	0	1	1	no

ENSG00000111701	ENSG00000111701	APOBEC1	12:7801995-7818499	q1	q2	NOTEST	0	0	0	0	1	1	no

ENSG00000111704	ENSG00000111704	NANOG	12:7940389-7948655	q1	q2	NOTEST	0.026327	0.210458	2.99892	0	1	1	no

ENSG00000111707	ENSG00000111707	SUDS3	12:118814184-118855840	q1	q2	OK	17.3832	18.6555	0.101901	0.193877	0.73265	0.99995	no

ENSG00000111711	ENSG00000111711	GOLT1B	12:21654714-21671342	q1	q2	OK	28.5079	30.9962	0.120728	0.231202	0.68725	0.99995	no

ENSG00000111713	ENSG00000111713	GYS2	12:21679240-21757781	q1	q2	NOTEST	0.134313	0.130625	-0.0401701	0	1	1	no

ENSG00000111716	ENSG00000111716	LDHB	12:21788275-21928515	q1	q2	OK	861.353	909.441	0.0783756	0.160533	0.8155	0.99995	no

ENSG00000111725	ENSG00000111725	PRKAB1	12:119772516-120119435	q1	q2	OK	18.1012	16.5156	-0.132254	-0.253879	0.68765	0.99995	no

ENSG00000111726	ENSG00000111726	CMAS	12:22199107-22589975	q1	q2	OK	34.9118	38.2407	0.131391	0.185089	0.7814	0.99995	no

ENSG00000111727	ENSG00000111727	HCFC2	12:104458234-104498639	q1	q2	OK	5.73488	5.43334	-0.0779227	-0.105571	0.872	0.99995	no

ENSG00000111728	ENSG00000111728	ST8SIA1	12:22199107-22589975	q1	q2	OK	21.7294	19.5533	-0.152235	-0.16536	0.7651	0.99995	no

ENSG00000111729	ENSG00000111729	CLEC4A	12:8276227-8371507	q1	q2	OK	1.35404	0.75323	-0.846104	-0.483397	0.453	0.99995	no

ENSG00000111731	ENSG00000111731	C2CD5	12:22601516-22697480	q1	q2	OK	30.0042	31.3271	0.0622495	0.0901669	0.85335	0.99995	no

ENSG00000111732	ENSG00000111732	AICDA	12:8754761-8765467	q1	q2	NOTEST	0	0	0	0	1	1	no

ENSG00000111737	ENSG00000111737	RAB35	12:120532898-120555306	q1	q2	OK	21.2558	22.42	0.0769325	0.155825	0.7926	0.99995	no

ENSG00000111752	ENSG00000111752	PHC1	12:9066491-9102551	q1	q2	OK	30.1631	32.9453	0.127288	0.1524	0.77905	0.99995	no

ENSG00000111775	ENSG00000111775	COX6A1	12:120875892-120899389	q1	q2	OK	257.224	251.299	-0.0336234	-0.0633228	0.9298	0.99995	no

ENSG00000111780	ENSG00000111780	AL021546.6	12:120875892-120899389	q1	q2	NOTEST	0.441197	0.111023	-1.99056	0	1	1	no

ENSG00000111783	ENSG00000111783	RFX4	12:106751435-107283090	q1	q2	OK	38.3931	35.765	-0.102299	-0.180533	0.77495	0.99995	no

ENSG00000111785	ENSG00000111785	RIC8B	12:106751435-107283090	q1	q2	OK	14.8558	14.198	-0.0653434	-0.0526081	0.91395	0.99995	no

ENSG00000111786	ENSG00000111786	SRSF9	12:120899470-120907596	q1	q2	OK	262.013	269.158	0.0388161	0.091093	0.89285	0.99995	no

ENSG00000111788	ENSG00000111788	RP11-22B23.1	12:9429830-9466684	q1	q2	OK	5.30845	4.72901	-0.166751	-0.301613	0.65935	0.99995	no

ENSG00000111790	ENSG00000111790	FGFR1OP2	12:27091315-27119583	q1	q2	OK	33.5111	34.3576	0.0359884	0.0679792	0.9229	0.99995	no

ENSG00000111796	ENSG00000111796	KLRB1	12:9747146-9760482	q1	q2	NOTEST	0	0.018491	inf	0	1	1	no

ENSG00000111799	ENSG00000111799	COL12A1	6:75794041-75915767	q1	q2	OK	39.5127	41.1084	0.0571151	0.090947	0.898	0.99995	no

ENSG00000111801	ENSG00000111801	BTN3A3	6:26440699-26453643	q1	q2	OK	2.63514	2.82877	0.102295	0.087968	0.8382	0.99995	no

ENSG00000111802	ENSG00000111802	TDP2	6:24650204-24667261	q1	q2	OK	31.8881	35.2761	0.145673	0.317497	0.6332	0.99995	no

ENSG00000111816	ENSG00000111816	FRK	6:116252311-116381921	q1	q2	NOTEST	0.0808698	0.0496996	-0.702366	0	1	1	no

ENSG00000111817	ENSG00000111817	DSE	6:116575335-116762424	q1	q2	OK	15.6528	18.0724	0.207362	0.26573	0.7152	0.99995	no

ENSG00000111832	ENSG00000111832	RWDD1	6:116892529-116918838	q1	q2	OK	31.1656	34.081	0.129014	0.25879	0.7033	0.99995	no

ENSG00000111834	ENSG00000111834	RSPH4A	6:116937641-116954148	q1	q2	OK	2.25294	1.69684	-0.408952	-0.542613	0.4357	0.99995	no

ENSG00000111837	ENSG00000111837	MAK	6:10747991-10979553	q1	q2	OK	0.442256	0.512171	0.211746	0.0803596	0.90585	0.99995	no

ENSG00000111843	ENSG00000111843	TMEM14C	6:10723147-10731362	q1	q2	OK	74.8439	75.6392	0.0152493	0.0354247	0.95955	0.99995	no

ENSG00000111845	ENSG00000111845	PAK1IP1	6:10671650-10710015	q1	q2	OK	13.9784	16.325	0.223885	0.435736	0.5243	0.99995	no

ENSG00000111846	ENSG00000111846	GCNT2	6:10492455-10629601	q1	q2	OK	5.77831	6.85292	0.246073	0.436734	0.4531	0.99995	no

ENSG00000111850	ENSG00000111850	SMIM8	6:87992695-88109467	q1	q2	OK	5.67159	6.22974	0.135421	0.156671	0.82175	0.99995	no

ENSG00000111859	ENSG00000111859	NEDD9	6:11173684-11382581	q1	q2	OK	7.71188	9.01875	0.225845	0.442247	0.4666	0.99995	no

ENSG00000111860	ENSG00000111860	CEP85L	6:118781934-119031238	q1	q2	OK	4.32838	5.23897	0.275456	0.518305	0.39615	0.99995	no

ENSG00000111863	ENSG00000111863	ADTRP	6:11712286-11807279	q1	q2	NOTEST	0	0	0	0	1	1	no

ENSG00000111875	ENSG00000111875	ASF1A	6:119134604-119470552	q1	q2	OK	33.6033	34.7519	0.0484915	0.0589426	0.92535	0.99995	no

ENSG00000111877	ENSG00000111877	MCM9	6:119134604-119470552	q1	q2	OK	8.0715	8.23568	0.0290511	0.0143938	0.96905	0.99995	no

ENSG00000111879	ENSG00000111879	FAM184A	6:119134604-119470552	q1	q2	OK	29.8803	44.8724	0.586633	0.689661	0.24205	0.99995	no

ENSG00000111880	ENSG00000111880	RNGTT	6:89319984-89673348	q1	q2	OK	11.8379	14.2232	0.264839	0.613432	0.3784	0.99995	no

ENSG00000111885	ENSG00000111885	MAN1A1	6:119498373-119670926	q1	q2	OK	6.65773	7.37402	0.14742	0.28981	0.6816	0.99995	no

ENSG00000111886	ENSG00000111886	GABRR2	6:89966926-90025018	q1	q2	NOTEST	0.227182	0.254865	0.165885	0	1	1	no

ENSG00000111897	ENSG00000111897	SERINC1	6:122764498-122792967	q1	q2	OK	86.2622	81.3412	-0.0847415	-0.209231	0.77045	0.99995	no

ENSG00000111906	ENSG00000111906	HDDC2	6:125440194-125623282	q1	q2	OK	44.1563	45.5635	0.0452589	0.080231	0.9026	0.99995	no

ENSG00000111907	ENSG00000111907	TPD52L1	6:125440194-125623282	q1	q2	OK	5.60115	4.93011	-0.184103	-0.115693	0.87275	0.99995	no

ENSG00000111911	ENSG00000111911	HINT3	6:126277926-126301387	q1	q2	OK	13.124	14.3215	0.125977	0.2787	0.69	0.99995	no

ENSG00000111912	ENSG00000111912	NCOA7	6:126102306-126252266	q1	q2	OK	3.37218	3.41495	0.018184	0.0226757	0.97045	0.99995	no

ENSG00000111913	ENSG00000111913	FAM65B	6:24797548-25057301	q1	q2	OK	20.9892	26.0315	0.310615	0.559859	0.3256	0.99995	no

ENSG00000111961	ENSG00000111961	SASH1	6:148558720-148873186	q1	q2	OK	10.0134	9.7052	-0.0451081	-0.096508	0.8778	0.99995	no

ENSG00000111962	ENSG00000111962	UST	6:149068463-149398126	q1	q2	OK	7.57038	10.4876	0.470246	0.683136	0.19385	0.99995	no

ENSG00000111981	ENSG00000111981	ULBP1	6:150285142-150294846	q1	q2	OK	2.24409	2.41824	0.107825	0.15811	0.8158	0.99995	no

ENSG00000112029	ENSG00000112029	FBXO5	6:153291663-153304714	q1	q2	OK	40.7039	44.9362	0.142709	0.34438	0.62615	0.99995	no

ENSG00000112031	ENSG00000112031	MTRF1L	6:153304884-153323820	q1	q2	OK	6.91722	8.70354	0.33141	0.440997	0.3894	0.99995	no

ENSG00000112033	ENSG00000112033	PPARD	6:35310334-35395968	q1	q2	OK	8.44566	7.19328	-0.231559	-0.467994	0.5052	0.99995	no

ENSG00000112038	ENSG00000112038	OPRM1	6:154331630-154677926	q1	q2	NOTEST	0	0.0109976	inf	0	1	1	no

ENSG00000112039	ENSG00000112039	FANCE	6:35420137-35434880	q1	q2	OK	6.56869	7.32789	0.157794	0.285426	0.6827	0.99995	no

ENSG00000112041	ENSG00000112041	TULP1	6:35465650-35480715	q1	q2	NOTEST	0.0567573	0.0132273	-2.10129	0	1	1	no

ENSG00000112053	ENSG00000112053	SLC26A8	6:35911290-35992645	q1	q2	NOTEST	0	0.0120901	inf	0	1	1	no

ENSG00000112062	ENSG00000112062	MAPK14	6:35995487-36079013	q1	q2	OK	16.6338	17.4826	0.0717969	0.134839	0.81025	0.99995	no

ENSG00000112077	ENSG00000112077	RHAG	6:49572870-49604552	q1	q2	NOTEST	0	0.0240238	inf	0	1	1	no

ENSG00000112078	ENSG00000112078	KCTD20	6:36322418-36458920	q1	q2	OK	29.0183	29.5232	0.0248878	0.0508333	0.9317	0.99995	no

ENSG00000112079	ENSG00000112079	STK38	6:36461668-36515247	q1	q2	OK	22.9917	23.3262	0.0208356	0.0496712	0.9407	0.99995	no

ENSG00000112081	ENSG00000112081	SRSF3	6:36562144-36573377	q1	q2	OK	200.357	209.944	0.0674341	0.115517	0.86575	0.99995	no

ENSG00000112096	ENSG00000112096	SOD2	6:160090088-160210781	q1	q2	OK	28.8955	33.226	0.201469	0.0863486	0.8073	0.99995	no

ENSG00000112110	ENSG00000112110	MRPL18	6:160210843-160219468	q1	q2	OK	41.9093	38.7534	-0.112947	-0.233381	0.743	0.99995	no

ENSG00000112115	ENSG00000112115	IL17A	6:52051184-52055436	q1	q2	NOTEST	0	0	0	0	1	1	no

ENSG00000112116	ENSG00000112116	IL17F	6:52101478-52109335	q1	q2	NOTEST	0	0	0	0	1	1	no

ENSG00000112118	ENSG00000112118	MCM3	6:52128806-52149679	q1	q2	OK	70.2222	77.0802	0.134433	0.311219	0.63995	0.99995	no

ENSG00000112130	ENSG00000112130	RNF8	6:37321747-37362514	q1	q2	OK	17.4578	16.7159	-0.0626488	-0.116753	0.8596	0.99995	no

ENSG00000112137	ENSG00000112137	PHACTR1	6:12717892-13328815	q1	q2	OK	7.60866	7.63399	0.00479544	0.00445979	0.99365	0.99995	no

ENSG00000112139	ENSG00000112139	MDGA1	6:37600283-37667082	q1	q2	OK	1.83336	1.51271	-0.277351	-0.255887	0.56845	0.99995	no

ENSG00000112144	ENSG00000112144	ICK	6:52866076-52965671	q1	q2	OK	8.16482	8.34887	0.0321592	0.0565658	0.9356	0.99995	no

ENSG00000112146	ENSG00000112146	FBXO9	6:52866076-52965671	q1	q2	OK	23.416	21.064	-0.152721	-0.163365	0.7479	0.99995	no

ENSG00000112149	ENSG00000112149	CD83	6:14117871-14137149	q1	q2	OK	6.56524	7.37937	0.168648	0.301398	0.66355	0.99995	no

ENSG00000112159	ENSG00000112159	MDN1	6:90352217-90529442	q1	q2	OK	10.9384	12.8612	0.233632	0.167701	0.7967	0.99995	no

ENSG00000112164	ENSG00000112164	GLP1R	6:39016573-39055519	q1	q2	OK	1.58807	2.22243	0.484859	0.655498	0.34515	0.99995	no

ENSG00000112167	ENSG00000112167	SAYSD1	6:39071839-39082965	q1	q2	OK	8.09507	8.47033	0.0653743	0.0940052	0.89475	0.99995	no

ENSG00000112175	ENSG00000112175	BMP5	6:55618442-55740362	q1	q2	OK	0.79849	1.03324	0.371829	0.416995	0.539	0.99995	no

ENSG00000112182	ENSG00000112182	BACH2	6:90636247-91006627	q1	q2	OK	6.42717	6.02938	-0.0921749	-0.107669	0.76185	0.99995	no

ENSG00000112183	ENSG00000112183	RBM24	6:17281576-17294106	q1	q2	OK	4.46498	4.94406	0.147042	0.215582	0.74585	0.99995	no

ENSG00000112186	ENSG00000112186	CAP2	6:17393446-17558023	q1	q2	OK	19.4588	21.4388	0.139801	0.310711	0.6643	0.99995	no

ENSG00000112195	ENSG00000112195	TREML2	6:41158014-41168932	q1	q2	NOTEST	0	0	0	0	1	1	no

ENSG00000112200	ENSG00000112200	ZNF451	6:56951641-57087078	q1	q2	OK	20.813	22.8613	0.135421	0.145114	0.7441	0.99995	no

ENSG00000112208	ENSG00000112208	BAG2	6:56951641-57087078	q1	q2	OK	8.62034	8.07819	-0.0937124	-0.0576113	0.9486	0.99995	no

ENSG00000112210	ENSG00000112210	RAB23	6:56951641-57087078	q1	q2	OK	14.0045	14.9564	0.0948741	0.105617	0.88725	0.99995	no

ENSG00000112212	ENSG00000112212	TSPO2	6:40994771-41067715	q1	q2	NOTEST	0.0797557	0.142346	0.835739	0	1	1	no

ENSG00000112214	ENSG00000112214	FHL5	6:97010423-97064512	q1	q2	NOTEST	0	0	0	0	1	1	no

ENSG00000112218	ENSG00000112218	GPR63	6:97233012-97285353	q1	q2	OK	1.73077	2.09379	0.274702	0.438571	0.5208	0.99995	no

ENSG00000112232	ENSG00000112232	KHDRBS2	6:62340137-62996132	q1	q2	OK	10.8069	11.3687	0.0731273	0.141777	0.8425	0.99995	no

ENSG00000112234	ENSG00000112234	FBXL4	6:99316419-99395849	q1	q2	OK	3.39337	3.59855	0.0847003	0.136581	0.84355	0.99995	no

ENSG00000112237	ENSG00000112237	CCNC	6:99990255-100016972	q1	q2	OK	29.9966	31.5472	0.0727119	0.0922289	0.82485	0.99995	no

ENSG00000112238	ENSG00000112238	PRDM13	6:100054605-100063454	q1	q2	NOTEST	0.00824994	0.00757925	-0.122329	0	1	1	no

ENSG00000112242	ENSG00000112242	E2F3	6:20402397-20493941	q1	q2	OK	22.7444	24.251	0.0925297	0.222725	0.7466	0.99995	no

ENSG00000112245	ENSG00000112245	PTP4A1	6:64231665-64293492	q1	q2	OK	48.5246	55.3973	0.191099	0.45672	0.5141	0.99995	no

ENSG00000112246	ENSG00000112246	SIM1	6:100832890-100912805	q1	q2	OK	0.44064	0.610324	0.469975	0.366695	0.4977	0.99995	no

ENSG00000112249	ENSG00000112249	ASCC3	6:100956069-101329248	q1	q2	OK	17.9645	19.0366	0.0836265	0.111984	0.8372	0.99995	no

ENSG00000112273	ENSG00000112273	HDGFL1	6:22569677-22571892	q1	q2	NOTEST	0.0408288	0.0125541	-1.70143	0	1	1	no

ENSG00000112276	ENSG00000112276	BVES	6:105544696-105627870	q1	q2	OK	3.47638	3.81168	0.132843	0.244942	0.72625	0.99995	no

ENSG00000112280	ENSG00000112280	COL9A1	6:70924763-71012786	q1	q2	OK	14.2988	10.4976	-0.445839	-0.594332	0.1797	0.99995	no

ENSG00000112282	ENSG00000112282	MED23	6:131894283-131949369	q1	q2	OK	16.7266	17.999	0.105774	0.175298	0.76985	0.99995	no

ENSG00000112290	ENSG00000112290	WASF1	6:110421021-110501207	q1	q2	OK	39.5951	39.0309	-0.0207058	-0.0457428	0.94255	0.99995	no

ENSG00000112293	ENSG00000112293	GPLD1	6:24403152-24537435	q1	q2	OK	0.847611	1.1444	0.433112	0.122753	0.7435	0.99995	no

ENSG00000112294	ENSG00000112294	ALDH5A1	6:24403152-24537435	q1	q2	OK	7.62968	7.39046	-0.0459584	-0.0607723	0.9212	0.99995	no

ENSG00000112297	ENSG00000112297	AIM1	6:106959729-107018326	q1	q2	NOTEST	0.249804	0.325687	0.382689	0	1	1	no

ENSG00000112299	ENSG00000112299	VNN1	6:133002728-133035188	q1	q2	NOTEST	0	0	0	0	1	1	no

ENSG00000112303	ENSG00000112303	VNN2	6:133065008-133084598	q1	q2	NOTEST	0.0514208	0.124757	1.27869	0	1	1	no

ENSG00000112304	ENSG00000112304	ACOT13	6:24667262-24721064	q1	q2	OK	18.5106	20.9198	0.17652	0.119068	0.8545	0.99995	no

ENSG00000112305	ENSG00000112305	SMAP1	6:71377478-71666741	q1	q2	OK	24.0445	23.5283	-0.0313057	-0.0679097	0.924	0.99995	no

ENSG00000112306	ENSG00000112306	RPS12	6:133135579-133138703	q1	q2	OK	812	780.789	-0.0565467	-0.13667	0.8425	0.99995	no

ENSG00000112308	ENSG00000112308	C6orf62	6:24667262-24721064	q1	q2	OK	53.4923	50.2643	-0.0897969	-0.167304	0.7813	0.99995	no

ENSG00000112309	ENSG00000112309	B3GAT2	6:71377478-71666741	q1	q2	OK	1.02767	1.03546	0.010893	0.0072991	0.9927	0.99995	no

ENSG00000112312	ENSG00000112312	GMNN	6:24775158-24786327	q1	q2	OK	64.0598	70.2262	0.13259	0.301662	0.66295	0.99995	no

ENSG00000112319	ENSG00000112319	EYA4	6:133561735-134216691	q1	q2	OK	1.02161	0.963808	-0.0840252	-0.0780605	0.86605	0.99995	no

ENSG00000112320	ENSG00000112320	SOBP	6:107811161-107981357	q1	q2	OK	7.35829	7.14906	-0.0416172	-0.0856039	0.89525	0.99995	no

ENSG00000112333	ENSG00000112333	NR2E1	6:108487261-108510013	q1	q2	NOTEST	0	0	0	0	1	1	no

ENSG00000112335	ENSG00000112335	SNX3	6:108532425-108582464	q1	q2	OK	82.5506	85.9054	0.0574703	0.13163	0.85345	0.99995	no

ENSG00000112337	ENSG00000112337	SLC17A2	6:25912981-25930946	q1	q2	NOTEST	0	0	0	0	1	1	no

ENSG00000112339	ENSG00000112339	HBS1L	6:135281515-135424194	q1	q2	OK	48.3664	54.5033	0.172338	0.278196	0.67535	0.99995	no

ENSG00000112343	ENSG00000112343	TRIM38	6:25963029-26002003	q1	q2	OK	0.644977	0.66749	0.0494981	0.0483896	0.95135	0.99995	no

ENSG00000112357	ENSG00000112357	PEX7	6:137143716-137235075	q1	q2	OK	2.2665	2.65931	0.230588	0.269062	0.6955	0.99995	no

ENSG00000112365	ENSG00000112365	ZBTB24	6:109765264-109804440	q1	q2	OK	2.60062	2.88356	0.148996	0.125037	0.86145	0.99995	no

ENSG00000112367	ENSG00000112367	FIG4	6:110012498-110146631	q1	q2	OK	7.9717	8.85731	0.151982	0.264174	0.6677	0.99995	no

ENSG00000112378	ENSG00000112378	PERP	6:138409641-138428648	q1	q2	OK	2.57791	2.87282	0.156263	0.25292	0.71165	0.99995	no

ENSG00000112379	ENSG00000112379	KIAA1244	6:138483057-138665800	q1	q2	NOTEST	0.165744	0.217525	0.392229	0	1	1	no

ENSG00000112394	ENSG00000112394	SLC16A10	6:111408780-111580237	q1	q2	OK	1.439	1.02666	-0.487111	-0.398875	0.4723	0.99995	no

ENSG00000112406	ENSG00000112406	HECA	6:139456248-139613276	q1	q2	OK	6.72331	6.18966	-0.119311	-0.244224	0.723	0.99995	no

ENSG00000112414	ENSG00000112414	GPR126	6:142622990-142767403	q1	q2	OK	9.76417	12.1846	0.319489	0.385782	0.3023	0.99995	no

ENSG00000112419	ENSG00000112419	PHACTR2	6:143857981-144184949	q1	q2	OK	4.61276	4.71448	0.0314683	0.0243545	0.9461	0.99995	no

ENSG00000112425	ENSG00000112425	EPM2A	6:145822718-146058354	q1	q2	OK	4.47397	4.29897	-0.0575648	-0.0812966	0.88795	0.99995	no

ENSG00000112462	ENSG00000112462	OR12D3	6:29323006-29424848	q1	q2	NOTEST	0	0	0	0	1	1	no

ENSG00000112473	ENSG00000112473	SLC39A7	6:33161364-33172216	q1	q2	NOTEST	0	0	0	0	1	1	no

ENSG00000112486	ENSG00000112486	CCR6	6:167412669-167553184	q1	q2	NOTEST	0	0	0	0	1	1	no

ENSG00000112494	ENSG00000112494	UNC93A	6:167684656-167729507	q1	q2	NOTEST	0	0	0	0	1	1	no

ENSG00000112499	ENSG00000112499	SLC22A2	6:160592092-160698670	q1	q2	NOTEST	0.0357565	0	#NAME?	0	1	1	no

ENSG00000112511	ENSG00000112511	PHF1	6:33378175-33386094	q1	q2	OK	1.02235	0.31083	-1.71769	-0.430199	0.2393	0.99995	no

ENSG00000112514	ENSG00000112514	CUTA	6:33378175-33386094	q1	q2	OK	28.6052	2.83345	-3.33565	-3.71542	5.00E-05	0.0795045	no

ENSG00000112530	ENSG00000112530	PACRG	6:161768451-163736524	q1	q2	OK	0.596112	1.16942	0.972141	0.456008	0.30175	0.99995	no

ENSG00000112531	ENSG00000112531	QKI	6:163835031-163999628	q1	q2	OK	182.194	191.627	0.0728274	0.0945486	0.84915	0.99995	no

ENSG00000112539	ENSG00000112539	C6orf118	6:165693152-165723096	q1	q2	OK	5.96053	6.7731	0.184375	0.185383	0.7928	0.99995	no

ENSG00000112541	ENSG00000112541	PDE10A	6:165740775-166401536	q1	q2	OK	9.72957	11.3519	0.222491	0.525221	0.4463	0.99995	no

ENSG00000112559	ENSG00000112559	MDFI	6:41604619-41621984	q1	q2	OK	65.6862	60.784	-0.111897	-0.233838	0.72055	0.99995	no

ENSG00000112561	ENSG00000112561	TFEB	6:41651715-41703997	q1	q2	OK	1.29889	1.06475	-0.286755	-0.223628	0.61995	0.99995	no

ENSG00000112562	ENSG00000112562	SMOC2	6:168841830-169073984	q1	q2	OK	7.96555	7.4638	-0.093865	-0.15157	0.8113	0.99995	no

ENSG00000112576	ENSG00000112576	CCND3	6:41902670-42018095	q1	q2	OK	17.5089	15.3194	-0.192727	-0.343418	0.56715	0.99995	no

ENSG00000112578	ENSG00000112578	BYSL	6:41888925-41900784	q1	q2	OK	17.1692	17.3055	0.0114087	0.0221877	0.9733	0.99995	no

ENSG00000112584	ENSG00000112584	FAM120B	6:170599790-170716153	q1	q2	OK	9.21265	8.90032	-0.049759	-0.0899876	0.8821	0.99995	no

ENSG00000112592	ENSG00000112592	TBP	6:170863389-170881958	q1	q2	OK	12.0447	11.7729	-0.0329304	-0.0618171	0.9292	0.99995	no

ENSG00000112599	ENSG00000112599	GUCA1B	6:42152138-42162654	q1	q2	OK	2.23406	1.86994	-0.256676	-0.276977	0.6945	0.99995	no

ENSG00000112619	ENSG00000112619	PRPH2	6:42664339-42690312	q1	q2	NOTEST	0.0864688	0.0929788	0.104722	0	1	1	no

ENSG00000112624	ENSG00000112624	GLTSCR1L	6:42749764-42836298	q1	q2	OK	3.96946	4.01154	0.0152135	0.0307281	0.9668	0.99995	no

ENSG00000112640	ENSG00000112640	PPP2R5D	6:42952236-42981706	q1	q2	OK	32.0958	35.3243	0.138278	0.22701	0.70575	0.99995	no

ENSG00000112651	ENSG00000112651	MRPL2	6:43005354-43042837	q1	q2	OK	22.5673	20.0218	-0.172664	-0.134375	0.83455	0.99995	no

ENSG00000112655	ENSG00000112655	PTK7	6:43044005-43129457	q1	q2	OK	56.4505	47.8193	-0.239394	-0.477534	0.46635	0.99995	no

ENSG00000112658	ENSG00000112658	SRF	6:43139036-43149243	q1	q2	OK	15.2726	15.9581	0.0633488	0.146938	0.8293	0.99995	no

ENSG00000112659	ENSG00000112659	CUL9	6:43149912-43192325	q1	q2	OK	16.2785	13.5088	-0.269071	-0.28762	0.63015	0.99995	no

ENSG00000112667	ENSG00000112667	DNPH1	6:43193366-43197222	q1	q2	OK	23.8181	19.7957	-0.266875	-0.42488	0.54295	0.99995	no

ENSG00000112679	ENSG00000112679	DUSP22	6:291629-351355	q1	q2	OK	23.0135	21.8988	-0.0716314	-0.129046	0.8305	0.99995	no

ENSG00000112685	ENSG00000112685	EXOC2	6:485132-693117	q1	q2	OK	12.5442	13.7317	0.130495	0.253888	0.66105	0.99995	no

ENSG00000112695	ENSG00000112695	COX7A2	6:75947390-75960039	q1	q2	OK	147.598	157.682	0.0953416	0.211567	0.74985	0.99995	no

ENSG00000112697	ENSG00000112697	TMEM30A	6:75962639-75994684	q1	q2	OK	41.0917	43.5395	0.083478	0.201013	0.7663	0.99995	no

ENSG00000112699	ENSG00000112699	GMDS	6:1624040-2245926	q1	q2	OK	9.45892	11.4782	0.279145	0.362979	0.5773	0.99995	no

ENSG00000112701	ENSG00000112701	SENP6	6:76311224-76427997	q1	q2	OK	60.1772	65.2111	0.1159	0.201349	0.72395	0.99995	no

ENSG00000112706	ENSG00000112706	IMPG1	6:76630831-76782395	q1	q2	NOTEST	0.308598	0.40997	0.409792	0	1	1	no

ENSG00000112715	ENSG00000112715	VEGFA	6:43737920-43754224	q1	q2	OK	18.3334	20.4532	0.157851	0.217536	0.6714	0.99995	no

ENSG00000112739	ENSG00000112739	PRPF4B	6:4021500-4157619	q1	q2	OK	51.5133	54.212	0.0736649	0.114984	0.82525	0.99995	no

ENSG00000112742	ENSG00000112742	TTK	6:80713603-80752244	q1	q2	OK	26.4736	29.2096	0.141888	0.300487	0.63795	0.99995	no

ENSG00000112759	ENSG00000112759	SLC29A1	6:44187241-44201888	q1	q2	OK	21.4256	22.5563	0.0741986	0.152371	0.832	0.99995	no

ENSG00000112761	ENSG00000112761	WISP3	6:112375274-112408732	q1	q2	NOTEST	0	0	0	0	1	1	no

ENSG00000112763	ENSG00000112763	BTN2A1	6:26458149-26476849	q1	q2	OK	17.7359	20.4094	0.202559	0.345055	0.54775	0.99995	no

ENSG00000112769	ENSG00000112769	LAMA4	6:112429962-112672498	q1	q2	NOTEST	0.00410241	0	#NAME?	0	1	1	no

ENSG00000112773	ENSG00000112773	FAM46A	6:82201155-82462491	q1	q2	OK	1.95998	1.91041	-0.0369619	-0.0480863	0.9318	0.99995	no

ENSG00000112782	ENSG00000112782	CLIC5	6:45868044-46048132	q1	q2	NOTEST	0.0439297	0.0203228	-1.1121	0	1	1	no

ENSG00000112787	ENSG00000112787	FBRSL1	12:133066136-133161774	q1	q2	OK	26.5276	19.2726	-0.460942	-0.419151	0.3354	0.99995	no

ENSG00000112796	ENSG00000112796	ENPP5	6:46126923-46138708	q1	q2	NOTEST	0.130879	0.258058	0.979462	0	1	1	no

ENSG00000112799	ENSG00000112799	LY86	6:6346697-6655216	q1	q2	NOTEST	0	0	0	0	1	1	no

ENSG00000112812	ENSG00000112812	PRSS16	6:27215479-27224403	q1	q2	OK	1.2956	1.14423	-0.179248	-0.157097	0.7328	0.99995	no

ENSG00000112818	ENSG00000112818	MEP1A	6:46761126-46807515	q1	q2	NOTEST	0	0	0	0	1	1	no

ENSG00000112837	ENSG00000112837	TBX18	6:85397068-85474237	q1	q2	OK	0.966657	1.23456	0.35292	0.279479	0.52475	0.99995	no

ENSG00000112851	ENSG00000112851	ERBB2IP	5:65222302-65378377	q1	q2	OK	31.8301	35.8098	0.169963	0.211155	0.60535	0.99995	no

ENSG00000112852	ENSG00000112852	PCDHB2	5:140474226-140476962	q1	q2	NOTEST	0.0197296	0.00906681	-1.12169	0	1	1	no

ENSG00000112855	ENSG00000112855	HARS2	5:140044260-140086248	q1	q2	OK	22.6879	21.7143	-0.0632745	-0.0581108	0.93235	0.99995	no

ENSG00000112874	ENSG00000112874	NUDT12	5:102884555-102898494	q1	q2	OK	9.16804	9.94951	0.118012	0.201532	0.75355	0.99995	no

ENSG00000112877	ENSG00000112877	CEP72	5:612386-693510	q1	q2	OK	6.34892	8.01216	0.33568	0.399686	0.5052	0.99995	no

ENSG00000112893	ENSG00000112893	MAN2A1	5:109025066-109205326	q1	q2	OK	14.1825	14.9938	0.0802538	0.131359	0.7991	0.99995	no

ENSG00000112902	ENSG00000112902	SEMA5A	5:9001885-9546187	q1	q2	OK	15.1043	13.8979	-0.1201	-0.289376	0.66525	0.99995	no

ENSG00000112936	ENSG00000112936	C7	5:40909353-40983041	q1	q2	OK	2.24624	2.78622	0.310803	0.120519	0.74855	0.99995	no

ENSG00000112941	ENSG00000112941	PAPD7	5:6714717-6757161	q1	q2	OK	23.9613	25.0038	0.0614442	0.102023	0.84085	0.99995	no

ENSG00000112964	ENSG00000112964	GHR	5:42423878-42721979	q1	q2	OK	0.896483	0.838151	-0.0970674	-0.0946591	0.8773	0.99995	no

ENSG00000112972	ENSG00000112972	HMGCS1	5:43287702-43313614	q1	q2	OK	344.305	446.473	0.374885	0.57039	0.29275	0.99995	no

ENSG00000112977	ENSG00000112977	DAP	5:10679341-10770406	q1	q2	OK	56.473	53.0132	-0.0912083	-0.21337	0.74135	0.99995	no

ENSG00000112981	ENSG00000112981	NME5	5:137450865-137475132	q1	q2	OK	4.6624	5.13938	0.140523	0.168662	0.77855	0.99995	no

ENSG00000112983	ENSG00000112983	BRD8	5:137475454-137549032	q1	q2	OK	70.6234	68.1905	-0.0505769	-0.0556298	0.90775	0.99995	no

ENSG00000112984	ENSG00000112984	KIF20A	5:137475454-137549032	q1	q2	OK	41.7019	43.0209	0.0449271	0.0534333	0.9256	0.99995	no

ENSG00000112992	ENSG00000112992	NNT	5:43571695-43707507	q1	q2	OK	27.5216	30.4426	0.145527	0.18806	0.7169	0.99995	no

ENSG00000112996	ENSG00000112996	MRPS30	5:44809026-44820530	q1	q2	OK	23.6978	25.6181	0.11241	0.216864	0.7541	0.99995	no

ENSG00000113013	ENSG00000113013	HSPA9	5:137890570-137911133	q1	q2	OK	109.448	117.064	0.0970472	0.207594	0.7355	0.99995	no

ENSG00000113048	ENSG00000113048	MRPS27	5:71515235-71656052	q1	q2	OK	31.9205	33.6377	0.0755947	0.163257	0.79875	0.99995	no

ENSG00000113068	ENSG00000113068	PFDN1	5:139554226-139682706	q1	q2	OK	69.3287	69.8708	0.0112351	0.0245281	0.97275	0.99995	no

ENSG00000113070	ENSG00000113070	HBEGF	5:139712427-139726216	q1	q2	OK	1.77699	1.90266	0.0985813	0.121736	0.8509	0.99995	no

ENSG00000113073	ENSG00000113073	SLC4A9	5:139739786-139780952	q1	q2	NOTEST	0	0.109068	inf	0	1	1	no

ENSG00000113083	ENSG00000113083	LOX	5:121297655-121413980	q1	q2	OK	2.95679	2.75186	-0.103624	-0.128606	0.84475	0.99995	no

ENSG00000113088	ENSG00000113088	GZMK	5:54273691-54338672	q1	q2	NOTEST	0	0	0	0	1	1	no

ENSG00000113100	ENSG00000113100	CDH9	5:26880708-27121257	q1	q2	NOTEST	0.103179	0.222303	1.10738	0	1	1	no

ENSG00000113108	ENSG00000113108	APBB3	5:139781398-139973337	q1	q2	OK	19.532	13.8696	-0.493913	-0.217439	0.50515	0.99995	no

ENSG00000113119	ENSG00000113119	TMCO6	5:140018324-140042064	q1	q2	OK	9.58676	9.60857	0.00327851	0.0015919	0.99645	0.99995	no

ENSG00000113140	ENSG00000113140	SPARC	5:151040656-151104343	q1	q2	OK	825.125	772.283	-0.0954829	-0.146762	0.803	0.99995	no

ENSG00000113141	ENSG00000113141	IK	5:140018324-140042064	q1	q2	OK	74.4548	73.9444	-0.00992309	-0.0184686	0.97765	0.99995	no

ENSG00000113161	ENSG00000113161	HMGCR	5:74615979-74657929	q1	q2	OK	139.305	177.216	0.347266	0.499619	0.2408	0.99995	no

ENSG00000113163	ENSG00000113163	COL4A3BP	5:74664310-74896969	q1	q2	OK	12.3276	13.8335	0.166267	0.209397	0.71295	0.99995	no

ENSG00000113194	ENSG00000113194	FAF2	5:175874628-175937075	q1	q2	OK	26.2853	27.6369	0.0723367	0.147357	0.79855	0.99995	no

ENSG00000113196	ENSG00000113196	HAND1	5:153854531-153857824	q1	q2	NOTEST	0	0	0	0	1	1	no

ENSG00000113205	ENSG00000113205	PCDHB3	5:140479828-140483406	q1	q2	NOTEST	0.0660135	0.0652665	-0.0164186	0	1	1	no

ENSG00000113209	ENSG00000113209	PCDHB5	5:140514799-140517703	q1	q2	NOTEST	0	0	0	0	1	1	no

ENSG00000113211	ENSG00000113211	PCDHB6	5:140529682-140532868	q1	q2	NOTEST	0.020848	0.01744	-0.257509	0	1	1	no

ENSG00000113212	ENSG00000113212	PCDHB7	5:140552242-140555957	q1	q2	NOTEST	0	0	0	0	1	1	no

ENSG00000113231	ENSG00000113231	PDE8B	5:76506273-76916436	q1	q2	OK	1.29086	1.54533	0.259582	0.150557	0.79465	0.99995	no

ENSG00000113240	ENSG00000113240	CLK4	5:178029664-178057616	q1	q2	OK	20.3939	17.9255	-0.186122	-0.352501	0.601	0.99995	no

ENSG00000113248	ENSG00000113248	PCDHB15	5:140625146-140627799	q1	q2	NOTEST	0	0	0	0	1	1	no

ENSG00000113249	ENSG00000113249	HAVCR1	5:156456423-156486130	q1	q2	NOTEST	0	0.0184704	inf	0	1	1	no

ENSG00000113262	ENSG00000113262	GRM6	5:178396390-178423207	q1	q2	NOTEST	0.0039927	0.00386125	-0.0482968	0	1	1	no

ENSG00000113263	ENSG00000113263	ITK	5:156512842-156682201	q1	q2	NOTEST	0.00788006	0.00723693	-0.122829	0	1	1	no

ENSG00000113269	ENSG00000113269	RNF130	5:179338650-179499118	q1	q2	OK	94.7453	92.9721	-0.0272563	-0.0514307	0.93005	0.99995	no

ENSG00000113272	ENSG00000113272	THG1L	5:157158204-157168456	q1	q2	OK	7.6013	7.63129	0.0056799	0.00720585	0.9914	0.99995	no

ENSG00000113273	ENSG00000113273	ARSB	5:78073031-78281910	q1	q2	OK	7.80535	8.38641	0.103591	0.185461	0.7409	0.99995	no

ENSG00000113282	ENSG00000113282	CLINT1	5:157212750-157286183	q1	q2	OK	36.3309	37.7552	0.0554773	0.116774	0.84185	0.99995	no

ENSG00000113296	ENSG00000113296	THBS4	5:79287133-79379477	q1	q2	OK	3.9126	3.55109	-0.139865	-0.136062	0.79965	0.99995	no

ENSG00000113300	ENSG00000113300	CNOT6	5:179921411-180005405	q1	q2	OK	29.7572	32.4215	0.123713	0.210138	0.66565	0.99995	no

ENSG00000113302	ENSG00000113302	IL12B	5:158737940-158789842	q1	q2	NOTEST	0.0277969	0.008978	-1.63046	0	1	1	no

ENSG00000113303	ENSG00000113303	BTNL8	5:180326076-180377906	q1	q2	NOTEST	0	0	0	0	1	1	no

ENSG00000113312	ENSG00000113312	TTC1	5:159436119-159546430	q1	q2	OK	40.7261	41.1784	0.015936	0.0274053	0.96825	0.99995	no

ENSG00000113318	ENSG00000113318	MSH3	5:79922046-80172279	q1	q2	OK	6.12666	7.06862	0.206328	0.193137	0.7515	0.99995	no

ENSG00000113319	ENSG00000113319	RASGRF2	5:80243515-80597379	q1	q2	OK	0.630529	0.58926	-0.0976583	-0.0846477	0.88175	0.99995	no

ENSG00000113327	ENSG00000113327	GABRG2	5:161494545-161582542	q1	q2	NOTEST	0.22922	0.143472	-0.675958	0	1	1	no

ENSG00000113328	ENSG00000113328	CCNG1	5:162864574-162887146	q1	q2	OK	124.365	124.064	-0.00349095	-0.0068463	0.99205	0.99995	no

ENSG00000113356	ENSG00000113356	POLR3G	5:89754019-89810370	q1	q2	OK	1.93619	2.33721	0.271564	0.214126	0.65695	0.99995	no

ENSG00000113360	ENSG00000113360	DROSHA	5:31400603-31532303	q1	q2	OK	44.2452	46.0729	0.0583978	0.0769929	0.9026	0.99995	no

ENSG00000113361	ENSG00000113361	CDH6	5:31094083-31329253	q1	q2	OK	41.5489	41.1307	-0.0145939	-0.023875	0.96415	0.99995	no

ENSG00000113368	ENSG00000113368	LMNB1	5:126112314-126172712	q1	q2	OK	203.16	226.831	0.159	0.33304	0.6133	0.99995	no

ENSG00000113369	ENSG00000113369	ARRDC3	5:90664540-90679176	q1	q2	OK	19.0736	17.6895	-0.108686	-0.201466	0.7039	0.99995	no

ENSG00000113384	ENSG00000113384	GOLPH3	5:32124809-32174456	q1	q2	OK	41.7021	44.2042	0.0840623	0.20364	0.76845	0.99995	no

ENSG00000113387	ENSG00000113387	SUB1	5:32531738-32604185	q1	q2	OK	273.682	304.208	0.15256	0.273247	0.6877	0.99995	no

ENSG00000113389	ENSG00000113389	NPR3	5:32689175-32791819	q1	q2	OK	0.544756	0.733286	0.428767	0.369437	0.47705	0.99995	no

ENSG00000113391	ENSG00000113391	FAM172A	5:92953774-93447404	q1	q2	OK	8.49147	8.62006	0.0216839	0.0241996	0.96545	0.99995	no

ENSG00000113396	ENSG00000113396	SLC27A6	5:127593600-128369335	q1	q2	NOTEST	0.0512667	0.0828451	0.692395	0	1	1	no

ENSG00000113407	ENSG00000113407	TARS	5:33440801-33469644	q1	q2	OK	90.8815	102.407	0.172263	0.370463	0.5594	0.99995	no

ENSG00000113430	ENSG00000113430	IRX4	5:1877540-1887350	q1	q2	NOTEST	0.221175	0.0650585	-1.76538	0	1	1	no

ENSG00000113441	ENSG00000113441	LNPEP	5:96149730-96373219	q1	q2	OK	4.33913	4.30055	-0.012885	-0.0189965	0.97245	0.99995	no

ENSG00000113448	ENSG00000113448	PDE4D	5:58264864-59843484	q1	q2	OK	4.5869	5.95934	0.377634	0.623048	0.232	0.99995	no

ENSG00000113456	ENSG00000113456	RAD1	5:34905368-34926101	q1	q2	OK	20.9373	21.5041	0.0385376	0.046359	0.94395	0.99995	no

ENSG00000113460	ENSG00000113460	BRIX1	5:34905368-34926101	q1	q2	OK	25.7485	32.2207	0.323501	0.466711	0.51185	0.99995	no

ENSG00000113492	ENSG00000113492	AGXT2	5:34998205-35048198	q1	q2	NOTEST	0	0.0423757	inf	0	1	1	no

ENSG00000113494	ENSG00000113494	PRLR	5:35048860-35230794	q1	q2	OK	1.58784	1.70515	0.102831	0.102458	0.81585	0.99995	no

ENSG00000113504	ENSG00000113504	SLC12A7	5:1050498-1112150	q1	q2	OK	16.1742	16.2987	0.0110625	0.0148433	0.9725	0.99995	no

ENSG00000113520	ENSG00000113520	IL4	5:132009677-132018368	q1	q2	NOTEST	0	0	0	0	1	1	no

ENSG00000113522	ENSG00000113522	RAD50	5:131877135-131999964	q1	q2	OK	11.5538	11.3506	-0.0256028	-0.0389985	0.9356	0.99995	no

ENSG00000113525	ENSG00000113525	IL5	5:131877135-131999964	q1	q2	NOTEST	0	0	0	0	1	1	no

ENSG00000113532	ENSG00000113532	ST8SIA4	5:100142638-100238970	q1	q2	OK	10.1537	12.0588	0.248081	0.513656	0.41945	0.99995	no

ENSG00000113552	ENSG00000113552	GNPDA1	5:141371313-141392606	q1	q2	OK	23.0661	23.4526	0.0239738	0.0481133	0.93875	0.99995	no

ENSG00000113555	ENSG00000113555	PCDH12	5:141323149-141369856	q1	q2	OK	0.680264	0.879442	0.370493	0.240756	0.69755	0.99995	no

ENSG00000113558	ENSG00000113558	SKP1	5:133450401-133706738	q1	q2	OK	422.937	425.386	0.00832745	0.0112893	0.98615	0.99995	no

ENSG00000113569	ENSG00000113569	NUP155	5:37288238-37371283	q1	q2	OK	14.0881	15.5885	0.146008	0.24097	0.66425	0.99995	no

ENSG00000113575	ENSG00000113575	PPP2CA	5:133450401-133706738	q1	q2	OK	96.8396	106.169	0.132688	0.113949	0.80565	0.99995	no

ENSG00000113578	ENSG00000113578	FGF1	5:141689991-142077617	q1	q2	NOTEST	0.229947	0.357364	0.636092	0	1	1	no

ENSG00000113580	ENSG00000113580	NR3C1	5:142657495-142815077	q1	q2	OK	3.51338	3.42283	-0.0376689	-0.0585101	0.91645	0.99995	no

ENSG00000113583	ENSG00000113583	C5orf15	5:133291200-133304478	q1	q2	OK	33.4353	35.5616	0.0889466	0.208164	0.76435	0.99995	no

ENSG00000113593	ENSG00000113593	PPWD1	5:64859062-64883376	q1	q2	OK	33.7221	34.6212	0.0379608	0.0710326	0.91075	0.99995	no

ENSG00000113594	ENSG00000113594	LIFR	5:38475064-38671318	q1	q2	OK	44.9946	44.833	-0.00518898	-0.00860259	0.9858	0.99995	no

ENSG00000113595	ENSG00000113595	TRIM23	5:64885506-65167553	q1	q2	OK	6.81614	7.42485	0.123407	0.112409	0.84545	0.99995	no

ENSG00000113597	ENSG00000113597	TRAPPC13	5:64885506-65167553	q1	q2	OK	17.9594	19.0331	0.0837739	0.0766093	0.89805	0.99995	no

ENSG00000113600	ENSG00000113600	C9	5:39284363-39462402	q1	q2	NOTEST	0.00944265	0.0274503	1.53956	0	1	1	no

ENSG00000113615	ENSG00000113615	SEC24A	5:133936833-134063513	q1	q2	OK	11.4089	12.1147	0.0866041	0.139093	0.81345	0.99995	no

ENSG00000113621	ENSG00000113621	TXNDC15	5:134209492-134237215	q1	q2	OK	37.6904	32.7157	-0.204216	-0.305169	0.6244	0.99995	no

ENSG00000113638	ENSG00000113638	TTC33	5:40714576-40756077	q1	q2	OK	10.3046	10.7335	0.0588313	0.111509	0.8734	0.99995	no

ENSG00000113643	ENSG00000113643	RARS	5:167913449-167946304	q1	q2	OK	58.2201	59.8869	0.0407254	0.0865286	0.8858	0.99995	no

ENSG00000113645	ENSG00000113645	WWC1	5:167718655-167899308	q1	q2	OK	15.2525	13.1855	-0.21009	-0.293285	0.5228	0.99995	no

ENSG00000113648	ENSG00000113648	H2AFY	5:134363424-134736986	q1	q2	OK	205.289	205.531	0.00169783	0.00356424	0.9959	0.99995	no

ENSG00000113649	ENSG00000113649	TCERG1	5:145826873-145891524	q1	q2	OK	83.5981	81.8717	-0.0301056	-0.0476145	0.92745	0.99995	no

ENSG00000113657	ENSG00000113657	DPYSL3	5:146770373-146889619	q1	q2	OK	42.1685	41.6064	-0.01936	-0.0409524	0.94715	0.99995	no

ENSG00000113658	ENSG00000113658	SMAD5	5:135465195-135524435	q1	q2	FAIL	31.9208	36.6328	0	0	1	1	no

ENSG00000113712	ENSG00000113712	CSNK1A1	5:148871759-148931007	q1	q2	OK	104.965	110.415	0.0730213	0.120667	0.85365	0.99995	no

ENSG00000113716	ENSG00000113716	HMGXB3	5:149340299-149432386	q1	q2	OK	20.2674	18.8676	-0.103251	-0.112044	0.838	0.99995	no

ENSG00000113719	ENSG00000113719	ERGIC1	5:172261277-172379688	q1	q2	OK	53.2323	53.7604	0.0142398	0.033608	0.96025	0.99995	no

ENSG00000113721	ENSG00000113721	PDGFRB	5:149493399-149535435	q1	q2	OK	19.0807	17.7233	-0.106471	-0.140623	0.7444	0.99995	no

ENSG00000113722	ENSG00000113722	CDX1	5:149546357-149564120	q1	q2	NOTEST	0.0152409	0.0296007	0.957682	0	1	1	no

ENSG00000113732	ENSG00000113732	ATP6V0E1	5:172410759-172462448	q1	q2	OK	48.4711	50.1188	0.0482277	0.094145	0.8952	0.99995	no

ENSG00000113734	ENSG00000113734	BNIP1	5:172571164-172591390	q1	q2	OK	9.07281	9.21352	0.0222038	0.0352112	0.9631	0.99995	no

ENSG00000113739	ENSG00000113739	STC2	5:172741715-172756506	q1	q2	OK	6.306	4.70819	-0.421554	-0.454846	0.3221	0.99995	no

ENSG00000113742	ENSG00000113742	CPEB4	5:173315282-173388979	q1	q2	OK	4.93128	5.39863	0.130631	0.197753	0.70135	0.99995	no

ENSG00000113749	ENSG00000113749	HRH2	5:175085032-175113245	q1	q2	OK	0.559953	0.530948	-0.076735	-0.0811563	0.9104	0.99995	no

ENSG00000113758	ENSG00000113758	DBN1	5:176883608-176901402	q1	q2	OK	170.002	158.26	-0.103259	-0.197588	0.72265	0.99995	no

ENSG00000113761	ENSG00000113761	ZNF346	5:176449696-176508190	q1	q2	OK	15.0015	16.6297	0.148654	0.231408	0.67355	0.99995	no

ENSG00000113763	ENSG00000113763	UNC5A	5:176237477-176326333	q1	q2	NOTEST	0.241445	0.291556	0.272079	0	1	1	no

ENSG00000113790	ENSG00000113790	EHHADH	3:184880658-184999778	q1	q2	OK	1.37923	1.74516	0.339496	0.281908	0.52475	0.99995	no

ENSG00000113805	ENSG00000113805	CNTN3	3:74311718-74570291	q1	q2	OK	1.71931	1.91332	0.154251	0.105336	0.8466	0.99995	no

ENSG00000113810	ENSG00000113810	SMC4	3:159943422-160203561	q1	q2	OK	117.883	121.225	0.0403298	0.0458498	0.89855	0.99995	no

ENSG00000113811	ENSG00000113811	SELK	3:53918436-53926015	q1	q2	OK	57.8835	56.1982	-0.0426273	-0.0890979	0.8956	0.99995	no

ENSG00000113812	ENSG00000113812	ACTR8	3:53901092-53916296	q1	q2	OK	22.2442	20.173	-0.141005	-0.203489	0.74555	0.99995	no

ENSG00000113838	ENSG00000113838	TBCCD1	3:186256229-186315061	q1	q2	OK	8.00881	8.56885	0.097514	0.0723872	0.9104	0.99995	no

ENSG00000113845	ENSG00000113845	TIMMDC1	3:119217378-119278449	q1	q2	OK	58.2714	52.3818	-0.153723	-0.287395	0.67955	0.99995	no

ENSG00000113851	ENSG00000113851	CRBN	3:3168599-3221394	q1	q2	OK	25.7911	24.8596	-0.0530665	-0.0812947	0.88675	0.99995	no

ENSG00000113889	ENSG00000113889	KNG1	3:186435037-186478330	q1	q2	NOTEST	0	0.00762947	inf	0	1	1	no

ENSG00000113905	ENSG00000113905	HRG	3:186353757-186396029	q1	q2	NOTEST	0	0.0253724	inf	0	1	1	no

ENSG00000113916	ENSG00000113916	BCL6	3:187416046-187463515	q1	q2	OK	5.08965	3.80378	-0.420133	-0.459519	0.38815	0.99995	no

ENSG00000113924	ENSG00000113924	HGD	3:120347019-120401418	q1	q2	OK	0.712968	1.01258	0.506131	0.310962	0.4946	0.99995	no

ENSG00000113946	ENSG00000113946	CLDN16	3:190040329-190129932	q1	q2	NOTEST	0	0	0	0	1	1	no

ENSG00000113966	ENSG00000113966	ARL6	3:97483364-97519953	q1	q2	OK	4.74299	4.72704	-0.00486141	-0.00592485	0.9932	0.99995	no

ENSG00000113971	ENSG00000113971	NPHP3	3:132276981-132593067	q1	q2	OK	9.36584	11.0713	0.241347	0.206737	0.67705	0.99995	no

ENSG00000114013	ENSG00000114013	CD86	3:121774212-121839983	q1	q2	NOTEST	0.0330884	0.0191544	-0.788652	0	1	1	no

ENSG00000114019	ENSG00000114019	AMOTL2	3:134070693-134094321	q1	q2	OK	13.5197	14.4809	0.0990916	0.178247	0.7505	0.99995	no

ENSG00000114021	ENSG00000114021	NIT2	3:100053544-100075710	q1	q2	OK	14.0483	13.8097	-0.0247152	-0.0395524	0.95555	0.99995	no

ENSG00000114023	ENSG00000114023	FAM162A	3:122103022-122134882	q1	q2	OK	13.6315	14.4459	0.0837161	0.0863669	0.9097	0.99995	no

ENSG00000114026	ENSG00000114026	OGG1	3:9791627-9896822	q1	q2	OK	18.3319	16.8194	-0.124227	-0.089165	0.87985	0.99995	no

ENSG00000114030	ENSG00000114030	KPNA1	3:122135053-122233792	q1	q2	OK	32.6848	34.3131	0.0701384	0.104398	0.8442	0.99995	no

ENSG00000114054	ENSG00000114054	PCCB	3:135969147-136471220	q1	q2	OK	18.0481	17.8067	-0.0194311	-0.0218013	0.9755	0.99995	no

ENSG00000114062	ENSG00000114062	UBE3A	15:25497371-25684128	q1	q2	OK	32.8282	38.2339	0.219917	0.506383	0.43885	0.99995	no

ENSG00000114098	ENSG00000114098	ARMC8	3:137906108-138048728	q1	q2	OK	19.1209	20.3365	0.0889204	0.0960355	0.8092	0.99995	no

ENSG00000114107	ENSG00000114107	CEP70	3:138213185-138313380	q1	q2	OK	13.8219	13.3233	-0.0530042	-0.0812847	0.8963	0.99995	no

ENSG00000114113	ENSG00000114113	RBP2	3:139108656-139396859	q1	q2	NOTEST	0	0.149004	inf	0	1	1	no

ENSG00000114115	ENSG00000114115	RBP1	3:139108656-139396859	q1	q2	OK	67.9981	68.9471	0.0199944	0.0435918	0.95245	0.99995	no

ENSG00000114120	ENSG00000114120	SLC25A36	3:140660671-140698775	q1	q2	OK	52.4213	52.3896	-0.000874627	-0.00133692	0.99785	0.99995	no

ENSG00000114124	ENSG00000114124	GRK7	3:141496989-141537332	q1	q2	NOTEST	0	0.0534914	inf	0	1	1	no

ENSG00000114125	ENSG00000114125	RNF7	3:141457045-141466402	q1	q2	OK	71.2946	70.4569	-0.0170512	-0.0352552	0.96295	0.99995	no

ENSG00000114126	ENSG00000114126	TFDP2	3:141663269-141868386	q1	q2	OK	44.6932	36.8747	-0.277426	-0.321818	0.54595	0.99995	no

ENSG00000114127	ENSG00000114127	XRN1	3:142025448-142166904	q1	q2	OK	7.20289	7.67383	0.0913706	0.112796	0.77945	0.99995	no

ENSG00000114166	ENSG00000114166	KAT2B	3:20081514-20195896	q1	q2	OK	7.42284	7.86233	0.0829868	0.103585	0.8505	0.99995	no

ENSG00000114200	ENSG00000114200	BCHE	3:165490691-165555260	q1	q2	OK	48.7612	49.543	0.0229484	0.0421357	0.949	0.99995	no

ENSG00000114204	ENSG00000114204	SERPINI2	3:167159576-167371771	q1	q2	OK	1.16536	1.13103	-0.0431369	-0.0249669	0.96915	0.99995	no

ENSG00000114209	ENSG00000114209	PDCD10	3:167401085-167452727	q1	q2	OK	27.4755	32.2571	0.231469	0.474491	0.4852	0.99995	no

ENSG00000114248	ENSG00000114248	LRRC31	3:169556966-169587718	q1	q2	NOTEST	0	0	0	0	1	1	no

ENSG00000114251	ENSG00000114251	WNT5A	3:55499742-55523973	q1	q2	OK	15.3394	13.7146	-0.161526	-0.318618	0.57435	0.99995	no

ENSG00000114268	ENSG00000114268	PFKFB4	3:48555116-48601206	q1	q2	OK	5.95017	6.20739	0.0610554	0.0771693	0.86815	0.99995	no

ENSG00000114270	ENSG00000114270	COL7A1	3:48601505-48632700	q1	q2	OK	7.06601	5.42023	-0.382542	-0.375082	0.50575	0.99995	no

ENSG00000114279	ENSG00000114279	FGF12	3:191857183-192485553	q1	q2	OK	4.19291	2.9675	-0.498702	-0.570891	0.277	0.99995	no

ENSG00000114302	ENSG00000114302	PRKAR2A	3:48782029-48889414	q1	q2	OK	11.6061	12.1587	0.0671053	0.129017	0.8138	0.99995	no

ENSG00000114315	ENSG00000114315	HES1	3:193853933-193856521	q1	q2	OK	34.7653	35.4575	0.028444	0.0612986	0.9302	0.99995	no

ENSG00000114316	ENSG00000114316	USP4	3:49306034-49378145	q1	q2	OK	19.9196	19.5801	-0.0247941	-0.0377401	0.9445	0.99995	no

ENSG00000114331	ENSG00000114331	ACAP2	3:194995464-195163807	q1	q2	OK	14.9477	17.6545	0.240111	0.261499	0.4061	0.99995	no

ENSG00000114346	ENSG00000114346	ECT2	3:172468471-172539264	q1	q2	OK	40.6025	45.1921	0.154504	0.303182	0.59555	0.99995	no

ENSG00000114349	ENSG00000114349	GNAT1	3:50229044-50233949	q1	q2	NOTEST	0	0.0111273	inf	0	1	1	no

ENSG00000114353	ENSG00000114353	GNAI2	3:50263723-50296787	q1	q2	OK	3.59638	2.79616	-0.363097	-0.504417	0.4481	0.99995	no

ENSG00000114354	ENSG00000114354	TFG	3:100428204-100467810	q1	q2	OK	87.9467	90.9207	0.047979	0.11423	0.8681	0.99995	no

ENSG00000114374	ENSG00000114374	USP9Y	Y:14813159-14972764	q1	q2	OK	19.0313	17.5368	-0.117992	-0.147427	0.81305	0.99995	no

ENSG00000114378	ENSG00000114378	HYAL1	3:50337319-50349812	q1	q2	NOTEST	0	0	0	0	1	1	no

ENSG00000114383	ENSG00000114383	TUSC2	3:50355220-50365682	q1	q2	OK	0.254768	0.569983	1.16173	0.516865	0.2488	0.99995	no

ENSG00000114388	ENSG00000114388	NPRL2	3:50384760-50541675	q1	q2	OK	13.8484	12.8832	-0.104229	-0.117502	0.8595	0.99995	no

ENSG00000114391	ENSG00000114391	RPL24	3:101399934-101405626	q1	q2	OK	754.991	780.664	0.0482426	0.115924	0.86815	0.99995	no

ENSG00000114395	ENSG00000114395	CYB561D2	3:50384760-50541675	q1	q2	OK	7.64653	6.87868	-0.152673	-0.111887	0.89365	0.99995	no

ENSG00000114405	ENSG00000114405	C3orf14	3:61547242-62355005	q1	q2	OK	20.7781	25.0202	0.268034	0.167612	0.87515	0.99995	no

ENSG00000114416	ENSG00000114416	FXR1	3:180319917-180700541	q1	q2	OK	102.376	105.698	0.0460682	0.0756643	0.8866	0.99995	no

ENSG00000114423	ENSG00000114423	CBLB	3:105374304-105588396	q1	q2	OK	14.9612	15.2453	0.0271425	0.0481675	0.9338	0.99995	no

ENSG00000114439	ENSG00000114439	BBX	3:107241782-107530171	q1	q2	OK	12.4825	11.8027	-0.0807843	-0.0972404	0.77765	0.99995	no

ENSG00000114446	ENSG00000114446	IFT57	3:107879658-107941417	q1	q2	OK	20.4954	22.2081	0.115786	0.170693	0.80095	0.99995	no

ENSG00000114450	ENSG00000114450	GNB4	3:179116989-179169378	q1	q2	OK	15.325	19.6783	0.360719	0.753379	0.2208	0.99995	no

ENSG00000114455	ENSG00000114455	HHLA2	3:108015375-108097132	q1	q2	NOTEST	0.0108798	0.0198704	0.868969	0	1	1	no

ENSG00000114473	ENSG00000114473	IQCG	3:197615945-197687013	q1	q2	OK	7.83378	8.30229	0.0838009	0.0384506	0.93845	0.99995	no

ENSG00000114480	ENSG00000114480	GBE1	3:81538849-81811312	q1	q2	OK	11.0459	14.7527	0.417469	0.798203	0.2167	0.99995	no

ENSG00000114487	ENSG00000114487	MORC1	3:108677085-108836989	q1	q2	NOTEST	0	0	0	0	1	1	no

ENSG00000114491	ENSG00000114491	UMPS	3:124449212-124464040	q1	q2	OK	24.8817	27.127	0.124643	0.215979	0.7446	0.99995	no

ENSG00000114503	ENSG00000114503	NCBP2	3:196662272-196669468	q1	q2	OK	87.299	91.0318	0.0604055	0.140872	0.8331	0.99995	no

ENSG00000114520	ENSG00000114520	SNX4	3:125165494-125239041	q1	q2	OK	27.6305	30.1363	0.125239	0.283606	0.67745	0.99995	no

ENSG00000114529	ENSG00000114529	C3orf52	3:111805181-111852152	q1	q2	OK	1.9179	1.91874	0.000635116	0.000693137	0.99885	0.99995	no

ENSG00000114541	ENSG00000114541	FRMD4B	3:69219140-69591734	q1	q2	OK	5.033	6.27084	0.31724	0.34378	0.4146	0.99995	no

ENSG00000114544	ENSG00000114544	SLC41A3	3:125725197-125820404	q1	q2	OK	17.4935	16.8434	-0.054634	-0.101367	0.86275	0.99995	no

ENSG00000114547	ENSG00000114547	ROPN1B	3:125687986-125702297	q1	q2	NOTEST	0.0135268	0.0205648	0.604359	0	1	1	no

ENSG00000114554	ENSG00000114554	PLXNA1	3:126707436-126756235	q1	q2	OK	37.0978	41.7062	0.16893	0.26475	0.56345	0.99995	no

ENSG00000114573	ENSG00000114573	ATP6V1A	3:113465718-113530903	q1	q2	OK	21.0503	20.8707	-0.012359	-0.0281464	0.96525	0.99995	no

ENSG00000114626	ENSG00000114626	ABTB1	3:127391777-127399768	q1	q2	OK	2.81329	1.78778	-0.654088	-0.645919	0.21055	0.99995	no

ENSG00000114631	ENSG00000114631	PODXL2	3:127348023-127391652	q1	q2	OK	40.0841	34.7718	-0.205113	-0.48599	0.4821	0.99995	no

ENSG00000114638	ENSG00000114638	UPK1B	3:118864996-118924000	q1	q2	NOTEST	0.0136603	0	#NAME?	0	1	1	no

ENSG00000114646	ENSG00000114646	CSPG5	3:47603728-47622282	q1	q2	OK	13.3954	12.0375	-0.154193	-0.303565	0.66265	0.99995	no

ENSG00000114648	ENSG00000114648	KLHL18	3:47205986-47388306	q1	q2	OK	11.4248	9.87938	-0.209677	-0.168115	0.689	0.99995	no

ENSG00000114650	ENSG00000114650	SCAP	3:47455202-47518616	q1	q2	OK	28.0644	23.5716	-0.251689	-0.388045	0.40155	0.99995	no

ENSG00000114654	ENSG00000114654	EFCC1	3:128598438-128759585	q1	q2	OK	8.19191	7.56682	-0.114512	-0.120776	0.8742	0.99995	no

ENSG00000114656	ENSG00000114656	KIAA1257	3:128598438-128759585	q1	q2	OK	3.33073	3.74146	0.167763	0.0870119	0.88645	0.99995	no

ENSG00000114670	ENSG00000114670	NEK11	3:130569438-131100319	q1	q2	OK	0.921449	0.810884	-0.184409	-0.0399144	0.82995	0.99995	no

ENSG00000114686	ENSG00000114686	MRPL3	3:131181055-131221827	q1	q2	OK	69.9832	80.846	0.208168	0.497688	0.4628	0.99995	no

ENSG00000114698	ENSG00000114698	PLSCR4	3:145910125-145968966	q1	q2	OK	0.725836	0.914031	0.332599	0.278501	0.5717	0.99995	no

ENSG00000114735	ENSG00000114735	HEMK1	3:50595461-50622366	q1	q2	OK	8.30949	7.46885	-0.153873	-0.210079	0.7558	0.99995	no

ENSG00000114737	ENSG00000114737	CISH	3:50643920-50686720	q1	q2	OK	2.18452	2.21691	0.0212354	0.0150258	0.9846	0.99995	no

ENSG00000114738	ENSG00000114738	MAPKAPK3	3:50643920-50686720	q1	q2	OK	10.8625	10.2432	-0.0846802	-0.135037	0.84285	0.99995	no

ENSG00000114739	ENSG00000114739	ACVR2B	3:38492517-38534633	q1	q2	OK	20.3408	21.6965	0.0930885	0.218268	0.754	0.99995	no

ENSG00000114742	ENSG00000114742	WDR48	3:39093488-39180394	q1	q2	OK	28.7473	27.3103	-0.0739857	-0.120287	0.8277	0.99995	no

ENSG00000114744	ENSG00000114744	COMMD2	3:149456256-149470286	q1	q2	OK	28.3441	31.0831	0.133086	0.209353	0.73085	0.99995	no

ENSG00000114745	ENSG00000114745	GORASP1	3:39093488-39180394	q1	q2	OK	23.1425	24.5968	0.0879266	0.0767679	0.8418	0.99995	no

ENSG00000114757	ENSG00000114757	PEX5L	3:179512745-179754841	q1	q2	OK	2.63815	2.67504	0.0200317	0.0171048	0.9599	0.99995	no

ENSG00000114767	ENSG00000114767	RRP9	3:51967445-51975957	q1	q2	OK	9.89552	10.8581	0.133923	0.23403	0.732	0.99995	no

ENSG00000114770	ENSG00000114770	ABCC5	3:183637721-183735803	q1	q2	OK	18.2847	18.2498	-0.00275386	-0.00578574	0.9932	0.99995	no

ENSG00000114771	ENSG00000114771	AADAC	3:151451703-151645963	q1	q2	NOTEST	0	0	0	0	1	1	no

ENSG00000114779	ENSG00000114779	ABHD14B	3:51989329-52023213	q1	q2	OK	4.45727	4.17773	-0.0934407	-0.0439881	0.9427	0.99995	no

ENSG00000114784	ENSG00000114784	EIF1B	3:40351174-40353915	q1	q2	OK	120.918	115.569	-0.0652648	-0.156642	0.8206	0.99995	no

ENSG00000114786	ENSG00000114786	ABHD14A-ACY1	3:51989329-52023213	q1	q2	NOTEST	0.338997	0.136327	-1.3142	0	1	1	no

ENSG00000114790	ENSG00000114790	ARHGEF26	3:153742189-153975616	q1	q2	OK	6.9445	6.7933	-0.0317569	-0.0602941	0.9312	0.99995	no

ENSG00000114796	ENSG00000114796	KLHL24	3:183353355-183402265	q1	q2	OK	11.8528	9.63978	-0.298155	-0.357196	0.33835	0.99995	no

ENSG00000114805	ENSG00000114805	PLCH1	3:155093368-155462856	q1	q2	OK	20.2064	20.1185	-0.00628676	-0.0151214	0.9823	0.99995	no

ENSG00000114812	ENSG00000114812	VIPR1	3:42530790-42579059	q1	q2	OK	0.450899	0.548388	0.282392	0.16474	0.61865	0.99995	no

ENSG00000114841	ENSG00000114841	DNAH1	3:52350334-52434507	q1	q2	OK	1.11028	1.04448	-0.0881389	-0.0625817	0.89165	0.99995	no

ENSG00000114850	ENSG00000114850	SSR3	3:156257928-156272973	q1	q2	OK	55.5112	59.0384	0.0888744	0.148148	0.79515	0.99995	no

ENSG00000114853	ENSG00000114853	ZBTB47	3:42589460-42709072	q1	q2	OK	2.24338	1.95135	-0.201202	-0.0699728	0.9352	0.99995	no

ENSG00000114854	ENSG00000114854	TNNC1	3:52485117-52488086	q1	q2	NOTEST	0.283862	0.370602	0.384679	0	1	1	no

ENSG00000114857	ENSG00000114857	NKTR	3:42589460-42709072	q1	q2	OK	64.1898	71.108	0.147669	0.18305	0.64565	0.99995	no

ENSG00000114859	ENSG00000114859	CLCN2	3:183851806-184402546	q1	q2	OK	3.5347	3.9342	0.154484	0.0163962	0.8701	0.99995	no

ENSG00000114861	ENSG00000114861	FOXP1	3:71003843-71633140	q1	q2	OK	10.0352	10.1349	0.0142625	0.0198648	0.9675	0.99995	no

ENSG00000114867	ENSG00000114867	EIF4G1	3:183851806-184402546	q1	q2	OK	116.669	108.435	-0.105597	-0.0795594	0.87925	0.99995	no

ENSG00000114902	ENSG00000114902	SPCS1	3:52529353-52742182	q1	q2	OK	94.2652	96.0183	0.0265839	0.0178089	0.97915	0.99995	no

ENSG00000114904	ENSG00000114904	NEK4	3:52744799-52804965	q1	q2	OK	16.4306	16.943	0.0442993	0.0570729	0.89485	0.99995	no

ENSG00000114923	ENSG00000114923	SLC4A3	2:220492048-220506702	q1	q2	OK	4.00818	3.45598	-0.213852	-0.290606	0.61895	0.99995	no

ENSG00000114933	ENSG00000114933	INO80D	2:206854308-206951288	q1	q2	OK	3.03937	3.49488	0.201468	0.403111	0.52125	0.99995	no

ENSG00000114942	ENSG00000114942	EEF1B2	2:206979540-207027652	q1	q2	OK	303.649	298.113	-0.0265464	-0.0454809	0.9449	0.99995	no

ENSG00000114948	ENSG00000114948	ADAM23	2:207308262-207485851	q1	q2	OK	10.6651	9.73564	-0.131551	-0.124972	0.79685	0.99995	no

ENSG00000114956	ENSG00000114956	DGUOK	2:74153952-74208568	q1	q2	OK	72.0595	77.0909	0.0973707	0.209019	0.7636	0.99995	no

ENSG00000114978	ENSG00000114978	MOB1A	2:74379654-74406025	q1	q2	OK	35.427	38.8536	0.133198	0.261936	0.6248	0.99995	no

ENSG00000114982	ENSG00000114982	KANSL3	2:97258906-97370624	q1	q2	OK	25.345	22.9834	-0.141105	-0.270852	0.61995	0.99995	no

ENSG00000114988	ENSG00000114988	LMAN2L	2:97371665-97405801	q1	q2	OK	16.427	15.772	-0.0587047	-0.111855	0.8536	0.99995	no

ENSG00000114993	ENSG00000114993	RTKN	2:74652962-74669549	q1	q2	OK	16.5101	16.1178	-0.0346981	-0.0541485	0.9373	0.99995	no

ENSG00000114999	ENSG00000114999	TTL	2:113239730-113299316	q1	q2	OK	34.7572	37.3909	0.105373	0.173166	0.7546	0.99995	no

ENSG00000115008	ENSG00000115008	IL1A	2:113531491-113542167	q1	q2	NOTEST	0.013006	0.0128188	-0.020911	0	1	1	no

ENSG00000115009	ENSG00000115009	CCL20	2:228678557-228682272	q1	q2	NOTEST	0	0.0486268	inf	0	1	1	no

ENSG00000115020	ENSG00000115020	PIKFYVE	2:209130990-209223475	q1	q2	OK	11.3288	12.5668	0.149618	0.322558	0.5988	0.99995	no

ENSG00000115041	ENSG00000115041	KCNIP3	2:95963051-96051825	q1	q2	OK	2.09885	2.0139	-0.0596047	-0.038951	0.9523	0.99995	no

ENSG00000115042	ENSG00000115042	FAHD2A	2:96068468-96082364	q1	q2	OK	19.027	18.6907	-0.0257295	-0.0379903	0.95765	0.99995	no

ENSG00000115053	ENSG00000115053	NCL	2:232318241-232348352	q1	q2	OK	419.007	466.517	0.154955	0.208625	0.73585	0.99995	no

ENSG00000115073	ENSG00000115073	ACTR1B	2:98272430-98280570	q1	q2	OK	28.8773	25.598	-0.173902	-0.353907	0.59735	0.99995	no

ENSG00000115084	ENSG00000115084	SLC35F5	2:114462587-114514400	q1	q2	OK	14.0263	16.3319	0.219566	0.305796	0.6373	0.99995	no

ENSG00000115085	ENSG00000115085	ZAP70	2:98330022-98356325	q1	q2	OK	0.443608	0.584739	0.398509	0.259506	0.6212	0.99995	no

ENSG00000115091	ENSG00000115091	ACTR3	2:114647536-114720173	q1	q2	OK	124.039	136.868	0.141988	0.253397	0.6935	0.99995	no

ENSG00000115107	ENSG00000115107	STEAP3	2:119981383-120023228	q1	q2	NOTEST	0.443727	0.374639	-0.244171	0	1	1	no

ENSG00000115109	ENSG00000115109	EPB41L5	2:120770580-120974855	q1	q2	OK	18.9821	19.893	0.0676222	0.155108	0.80795	0.99995	no

ENSG00000115112	ENSG00000115112	TFCP2L1	2:121974162-122042783	q1	q2	NOTEST	0.0108597	0.0052391	-1.05159	0	1	1	no

ENSG00000115128	ENSG00000115128	SF3B14	2:24290453-24299313	q1	q2	OK	92.1839	99.6562	0.112444	0.260271	0.71585	0.99995	no

ENSG00000115129	ENSG00000115129	TP53I3	2:24299395-24424567	q1	q2	OK	36.4878	33.303	-0.131761	-0.271385	0.69165	0.99995	no

ENSG00000115137	ENSG00000115137	DNAJC27	2:25166504-25262563	q1	q2	OK	3.3889	3.38258	-0.00269503	-0.00290596	0.99435	0.99995	no

ENSG00000115138	ENSG00000115138	POMC	2:25383721-25391772	q1	q2	NOTEST	0.400362	0.25055	-0.676207	0	1	1	no

ENSG00000115145	ENSG00000115145	STAM2	2:152973314-153032506	q1	q2	OK	10.4265	12.2913	0.237386	0.416598	0.51155	0.99995	no

ENSG00000115155	ENSG00000115155	OTOF	2:26680070-26781566	q1	q2	NOTEST	0.0208318	0.012369	-0.752054	0	1	1	no

ENSG00000115159	ENSG00000115159	GPD2	2:157291801-157470247	q1	q2	OK	4.76822	5.06304	0.0865531	0.103613	0.8199	0.99995	no

ENSG00000115163	ENSG00000115163	CENPA	2:26987151-27023935	q1	q2	OK	18.2496	18.0362	-0.0169714	-0.0239047	0.9703	0.99995	no

ENSG00000115165	ENSG00000115165	CYTIP	2:158271130-158345473	q1	q2	NOTEST	0	0.0365104	inf	0	1	1	no

ENSG00000115170	ENSG00000115170	ACVR1	2:158592957-158732374	q1	q2	OK	7.27289	7.9492	0.128281	0.210475	0.73245	0.99995	no

ENSG00000115183	ENSG00000115183	TANC1	2:159825145-160089170	q1	q2	OK	9.14373	8.3828	-0.12535	-0.23498	0.7469	0.99995	no

ENSG00000115194	ENSG00000115194	SLC30A3	2:27476551-27504367	q1	q2	OK	0.52848	0.463992	-0.187749	-0.11911	0.77035	0.99995	no

ENSG00000115204	ENSG00000115204	MPV17	2:27532359-27548547	q1	q2	OK	42.9055	38.5169	-0.155672	-0.284902	0.68915	0.99995	no

ENSG00000115207	ENSG00000115207	GTF3C2	2:27548715-27593353	q1	q2	OK	36.5479	35.1863	-0.0547727	-0.0665177	0.882	0.99995	no

ENSG00000115211	ENSG00000115211	EIF2B4	2:27548715-27593353	q1	q2	OK	30.5863	27.9712	-0.128943	-0.134138	0.8245	0.99995	no

ENSG00000115216	ENSG00000115216	NRBP1	2:27650656-27665126	q1	q2	OK	55.3558	57.6719	0.0591322	0.13144	0.8386	0.99995	no

ENSG00000115221	ENSG00000115221	ITGB6	2:160956176-161128399	q1	q2	NOTEST	0.0866224	0.0840621	-0.0432847	0	1	1	no

ENSG00000115226	ENSG00000115226	FNDC4	2:27714749-27718112	q1	q2	OK	7.88942	6.80455	-0.21342	-0.342165	0.62085	0.99995	no

ENSG00000115232	ENSG00000115232	ITGA4	2:182321928-182400914	q1	q2	OK	3.45947	4.29672	0.312685	0.434419	0.47525	0.99995	no

ENSG00000115233	ENSG00000115233	PSMD14	2:162164548-162268228	q1	q2	OK	71.4506	75.1262	0.0723715	0.169472	0.79955	0.99995	no

ENSG00000115234	ENSG00000115234	SNX17	2:27593388-27599995	q1	q2	OK	56.4561	53.2117	-0.0853869	-0.187728	0.75485	0.99995	no

ENSG00000115239	ENSG00000115239	GPR75-ASB3	2:53759809-54087297	q1	q2	OK	14.5675	15.3284	0.0734482	0.0863275	0.89015	0.99995	no

ENSG00000115241	ENSG00000115241	PPM1G	2:27604060-27632554	q1	q2	OK	86.8979	85.6996	-0.0200334	-0.049751	0.94235	0.99995	no

ENSG00000115252	ENSG00000115252	PDE1A	2:183004762-183387919	q1	q2	OK	13.1772	11.8051	-0.158635	-0.200093	0.7105	0.99995	no

ENSG00000115255	ENSG00000115255	REEP6	19:1491164-1497926	q1	q2	OK	5.81022	5.07687	-0.194655	-0.276197	0.67255	0.99995	no

ENSG00000115257	ENSG00000115257	PCSK4	19:1481426-1490751	q1	q2	OK	4.84306	3.79473	-0.351923	-0.318222	0.6289	0.99995	no

ENSG00000115263	ENSG00000115263	GCG	2:162999391-163008914	q1	q2	NOTEST	0	0.0613606	inf	0	1	1	no

ENSG00000115266	ENSG00000115266	APC2	19:1446299-1479555	q1	q2	OK	38.8309	30.4323	-0.351602	-0.426769	0.32935	0.99995	no

ENSG00000115267	ENSG00000115267	IFIH1	2:163123588-163175213	q1	q2	NOTEST	0.179556	0.244038	0.44267	0	1	1	no

ENSG00000115268	ENSG00000115268	RPS15	19:1438357-1440583	q1	q2	OK	642.339	560.798	-0.195854	-0.476757	0.49775	0.99995	no

ENSG00000115271	ENSG00000115271	GCA	2:163175349-163695240	q1	q2	OK	4.37685	4.60503	0.0733172	0.0733952	0.8956	0.99995	no

ENSG00000115274	ENSG00000115274	INO80B	2:74682149-74688018	q1	q2	OK	17.5581	14.1207	-0.31432	-0.324579	0.58845	0.99995	no

ENSG00000115275	ENSG00000115275	MOGS	2:74688183-74692537	q1	q2	OK	23.0655	21.3021	-0.114746	-0.245589	0.72025	0.99995	no

ENSG00000115282	ENSG00000115282	TTC31	2:74699084-74722013	q1	q2	OK	15.5998	14.8801	-0.0681464	-0.0826213	0.8674	0.99995	no

ENSG00000115286	ENSG00000115286	NDUFS7	19:1383525-1396466	q1	q2	OK	31.063	28.4002	-0.129298	-0.201857	0.7805	0.99995	no

ENSG00000115289	ENSG00000115289	PCGF1	2:74732169-74735707	q1	q2	OK	9.82314	8.93521	-0.136683	-0.123065	0.80285	0.99995	no

ENSG00000115290	ENSG00000115290	GRB14	2:165349321-165478358	q1	q2	OK	1.01233	0.468467	-1.11166	-0.548934	0.2921	0.99995	no

ENSG00000115295	ENSG00000115295	CLIP4	2:29320570-29412509	q1	q2	OK	3.67396	3.16895	-0.213331	-0.184715	0.6478	0.99995	no

ENSG00000115297	ENSG00000115297	TLX2	2:74740589-74744274	q1	q2	NOTEST	0.0374394	0	#NAME?	0	1	1	no

ENSG00000115306	ENSG00000115306	SPTBN1	2:54683421-54896812	q1	q2	OK	64.4769	68.032	0.0774298	0.160025	0.8217	0.99995	no

ENSG00000115307	ENSG00000115307	AUP1	2:74753771-74784681	q1	q2	OK	45.1505	43.7452	-0.0456187	-0.0663162	0.9164	0.99995	no

ENSG00000115310	ENSG00000115310	RTN4	2:55199324-55339757	q1	q2	OK	166.04	174.818	0.0743201	0.152843	0.81825	0.99995	no

ENSG00000115317	ENSG00000115317	HTRA2	2:74753771-74784681	q1	q2	OK	21.2009	19.3084	-0.134891	-0.144607	0.85155	0.99995	no

ENSG00000115318	ENSG00000115318	LOXL3	2:74753771-74784681	q1	q2	OK	10.055	8.74096	-0.202049	-0.204284	0.7772	0.99995	no

ENSG00000115325	ENSG00000115325	DOK1	2:74753771-74784681	q1	q2	OK	10.0836	8.50162	-0.2462	-0.182644	0.79965	0.99995	no

ENSG00000115339	ENSG00000115339	GALNT3	2:166604100-166651192	q1	q2	OK	1.29966	1.34861	0.0533412	0.0457573	0.9274	0.99995	no

ENSG00000115350	ENSG00000115350	POLE4	2:75185618-75197255	q1	q2	OK	23.6837	29.0669	0.295483	0.431453	0.54515	0.99995	no

ENSG00000115353	ENSG00000115353	TACR1	2:75273589-75426826	q1	q2	OK	4.58011	2.9673	-0.626232	-0.528879	0.14745	0.99995	no

ENSG00000115355	ENSG00000115355	CCDC88A	2:55514977-55647057	q1	q2	OK	43.0054	47.441	0.141618	0.246705	0.64125	0.99995	no

ENSG00000115361	ENSG00000115361	ACADL	2:211036241-211095107	q1	q2	NOTEST	0.103229	0.0610389	-0.758054	0	1	1	no

ENSG00000115363	ENSG00000115363	EVA1A	2:75696427-75796848	q1	q2	OK	6.36753	5.38123	-0.242798	-0.274434	0.6083	0.99995	no

ENSG00000115364	ENSG00000115364	MRPL19	2:75873908-75938115	q1	q2	OK	27.7148	27.8573	0.00739889	0.009675	0.98735	0.99995	no

ENSG00000115365	ENSG00000115365	LANCL1	2:211189482-211342376	q1	q2	OK	20.3588	21.8496	0.101952	0.244164	0.72495	0.99995	no

ENSG00000115368	ENSG00000115368	WDR75	2:190306158-190340291	q1	q2	OK	25.302	27.5584	0.12324	0.243205	0.70085	0.99995	no

ENSG00000115380	ENSG00000115380	EFEMP1	2:56093101-56151274	q1	q2	OK	3.90055	4.24917	0.123506	0.164403	0.77865	0.99995	no

ENSG00000115386	ENSG00000115386	REG1A	2:79347487-79350545	q1	q2	NOTEST	0	0	0	0	1	1	no

ENSG00000115392	ENSG00000115392	FANCL	2:58134785-58468821	q1	q2	OK	33.2777	34.3911	0.0474767	0.0841316	0.8979	0.99995	no

ENSG00000115414	ENSG00000115414	FN1	2:216225162-216300895	q1	q2	OK	214.306	186.059	-0.20391	-0.196147	0.64485	0.99995	no

ENSG00000115415	ENSG00000115415	STAT1	2:191745522-191885686	q1	q2	OK	16.6587	17.6879	0.0864869	0.126818	0.84055	0.99995	no

ENSG00000115419	ENSG00000115419	GLS	2:191745522-191885686	q1	q2	OK	27.6893	32.7289	0.241237	0.264076	0.5098	0.99995	no

ENSG00000115421	ENSG00000115421	PAPOLG	2:60983364-61029220	q1	q2	OK	11.5812	11.3955	-0.023313	-0.0392799	0.9456	0.99995	no

ENSG00000115423	ENSG00000115423	DNAH6	2:84743578-85046713	q1	q2	NOTEST	0.205715	0.263017	0.354509	0	1	1	no

ENSG00000115425	ENSG00000115425	PECR	2:216809212-216967506	q1	q2	OK	6.26083	5.19425	-0.26944	-0.155715	0.7704	0.99995	no

ENSG00000115446	ENSG00000115446	UNC50	2:99225041-99234978	q1	q2	OK	34.9766	36.2263	0.0506492	0.103561	0.875	0.99995	no

ENSG00000115457	ENSG00000115457	IGFBP2	2:217497550-217529159	q1	q2	OK	548.4	556.938	0.0222878	0.0475042	0.9484	0.99995	no

ENSG00000115459	ENSG00000115459	ELMOD3	2:85569210-85618875	q1	q2	OK	13.5116	9.95393	-0.440864	-0.482721	0.3887	0.99995	no

ENSG00000115461	ENSG00000115461	IGFBP5	2:217536827-217858802	q1	q2	OK	112.115	109.584	-0.0329466	-0.0702992	0.91525	0.99995	no

ENSG00000115464	ENSG00000115464	USP34	2:61404552-61697904	q1	q2	OK	56.1308	61.6883	0.136205	0.118469	0.7547	0.99995	no

ENSG00000115468	ENSG00000115468	EFHD1	2:233412778-233547491	q1	q2	OK	7.86911	6.93911	-0.181449	-0.107117	0.88185	0.99995	no

ENSG00000115474	ENSG00000115474	KCNJ13	2:233562008-233880595	q1	q2	NOTEST	0.135106	0.178827	0.40447	0	1	1	no

ENSG00000115484	ENSG00000115484	CCT4	2:62095223-62374382	q1	q2	OK	122.239	131.468	0.105005	0.229186	0.72515	0.99995	no

ENSG00000115486	ENSG00000115486	GGCX	2:85766287-85788670	q1	q2	OK	16.8355	15.7366	-0.0973811	-0.0400211	0.8922	0.99995	no

ENSG00000115488	ENSG00000115488	NEU2	2:233897381-233899767	q1	q2	NOTEST	0	0.0506338	inf	0	1	1	no

ENSG00000115504	ENSG00000115504	EHBP1	2:62900985-63275775	q1	q2	OK	15.2629	16.0352	0.0712111	0.123642	0.81845	0.99995	no

ENSG00000115507	ENSG00000115507	OTX1	2:63277191-63284971	q1	q2	OK	2.00834	1.38659	-0.534463	-0.546825	0.3174	0.99995	no

ENSG00000115514	ENSG00000115514	TXNDC9	2:99613723-100106497	q1	q2	OK	29.3216	33.1265	0.176023	0.119727	0.83835	0.99995	no

ENSG00000115520	ENSG00000115520	COQ10B	2:198318146-198340032	q1	q2	OK	8.17136	7.60082	-0.104421	-0.174921	0.79965	0.99995	no

ENSG00000115523	ENSG00000115523	GNLY	2:85912297-85925977	q1	q2	NOTEST	0.0565632	0.0132947	-2.08901	0	1	1	no

ENSG00000115524	ENSG00000115524	SF3B1	2:198254507-198299815	q1	q2	OK	219.357	219.688	0.00217697	0.00267842	0.99665	0.99995	no

ENSG00000115525	ENSG00000115525	ST3GAL5	2:86066266-86116137	q1	q2	OK	7.31307	9.72172	0.410735	0.610313	0.2828	0.99995	no

ENSG00000115526	ENSG00000115526	CHST10	2:101008326-101034118	q1	q2	OK	16.1412	14.8357	-0.121677	-0.220573	0.6944	0.99995	no

ENSG00000115539	ENSG00000115539	PDCL3	2:101179151-101193197	q1	q2	OK	28.1442	30.654	0.123239	0.25855	0.7042	0.99995	no

ENSG00000115540	ENSG00000115540	MOB4	2:198351304-198418423	q1	q2	OK	33.5106	40.7626	0.282629	0.156626	0.83145	0.99995	no

ENSG00000115541	ENSG00000115541	HSPE1	2:198351304-198418423	q1	q2	OK	181.379	205.311	0.178805	0.153688	0.8262	0.99995	no

ENSG00000115548	ENSG00000115548	KDM3A	2:86667769-86719839	q1	q2	OK	26.9894	27.1133	0.00660682	0.0131473	0.97995	0.99995	no

ENSG00000115556	ENSG00000115556	PLCD4	2:219472487-219501907	q1	q2	OK	3.15058	2.90164	-0.118747	-0.142452	0.7985	0.99995	no

ENSG00000115561	ENSG00000115561	CHMP3	2:86730553-87005164	q1	q2	OK	43.1054	43.6672	0.0186823	0.0241984	0.9671	0.99995	no

ENSG00000115568	ENSG00000115568	ZNF142	2:219502638-219528166	q1	q2	OK	10.7732	10.9777	0.0271363	0.0312955	0.9569	0.99995	no

ENSG00000115590	ENSG00000115590	IL1R2	2:102608305-102645006	q1	q2	NOTEST	0.156489	0	#NAME?	0	1	1	no

ENSG00000115592	ENSG00000115592	PRKAG3	2:219687105-219696809	q1	q2	NOTEST	0	0	0	0	1	1	no

ENSG00000115593	ENSG00000115593	SMYD1	2:88367298-88412906	q1	q2	NOTEST	0.0210936	0.00583216	-1.8547	0	1	1	no

ENSG00000115594	ENSG00000115594	IL1R1	2:102681003-102798568	q1	q2	NOTEST	0.433219	0.375004	-0.208193	0	1	1	no

ENSG00000115596	ENSG00000115596	WNT6	2:219724543-219738955	q1	q2	NOTEST	0	0.0153353	inf	0	1	1	no

ENSG00000115598	ENSG00000115598	IL1RL2	2:102803432-102856462	q1	q2	NOTEST	0	0	0	0	1	1	no

ENSG00000115602	ENSG00000115602	IL1RL1	2:102927961-103015218	q1	q2	NOTEST	0	0	0	0	1	1	no

ENSG00000115604	ENSG00000115604	IL18R1	2:102927961-103015218	q1	q2	NOTEST	0.313578	0.185899	-0.754307	0	1	1	no

ENSG00000115607	ENSG00000115607	IL18RAP	2:103035148-103069025	q1	q2	NOTEST	0.0140412	0.0136293	-0.042955	0	1	1	no

ENSG00000115616	ENSG00000115616	SLC9A2	2:103236165-103327777	q1	q2	NOTEST	0.115848	0.123843	0.0962749	0	1	1	no

ENSG00000115641	ENSG00000115641	FHL2	2:105974168-106054970	q1	q2	OK	8.36936	9.79465	0.226876	0.352933	0.60825	0.99995	no

ENSG00000115648	ENSG00000115648	MLPH	2:238394070-238463961	q1	q2	NOTEST	0.217529	0.0915702	-1.24826	0	1	1	no

ENSG00000115649	ENSG00000115649	CNPPD1	2:220036618-220050201	q1	q2	OK	23.1432	20.9256	-0.145321	-0.21335	0.75875	0.99995	no

ENSG00000115652	ENSG00000115652	UXS1	2:106709758-106810795	q1	q2	OK	23.4862	26.1223	0.153466	0.301608	0.629	0.99995	no

ENSG00000115657	ENSG00000115657	ABCB6	2:220074489-220094439	q1	q2	OK	14.3515	11.5156	-0.317619	-0.305549	0.5547	0.99995	no

ENSG00000115661	ENSG00000115661	STK16	2:220094478-220142892	q1	q2	OK	16.0882	15.0275	-0.0984021	-0.103623	0.8763	0.99995	no

ENSG00000115665	ENSG00000115665	SLC5A7	2:108602978-108630450	q1	q2	NOTEST	0.00479174	0.00463535	-0.0478728	0	1	1	no

ENSG00000115677	ENSG00000115677	HDLBP	2:242166678-242293442	q1	q2	OK	117.302	117.19	-0.00137994	-0.00103891	0.9986	0.99995	no

ENSG00000115685	ENSG00000115685	PPP1R7	2:242045513-242123067	q1	q2	OK	38.9733	40.8249	0.0669655	0.088425	0.90165	0.99995	no

ENSG00000115687	ENSG00000115687	PASK	2:242045513-242123067	q1	q2	OK	14.5918	15.3611	0.0741233	0.087731	0.87425	0.99995	no

ENSG00000115694	ENSG00000115694	STK25	2:242295657-242449145	q1	q2	OK	114.062	112.009	-0.0262064	-0.0422253	0.94735	0.99995	no

ENSG00000115705	ENSG00000115705	TPO	2:1377994-1547483	q1	q2	NOTEST	0.0373076	0.0564024	0.596286	0	1	1	no

ENSG00000115718	ENSG00000115718	PROC	2:128176002-128186822	q1	q2	NOTEST	0.421889	0.343124	-0.298131	0	1	1	no

ENSG00000115738	ENSG00000115738	ID2	2:8806765-8824583	q1	q2	OK	38.8865	35.4426	-0.133786	-0.283999	0.6613	0.99995	no

ENSG00000115750	ENSG00000115750	TAF1B	2:9983482-10074545	q1	q2	OK	12.0204	12.9037	0.102301	0.188395	0.76985	0.99995	no

ENSG00000115756	ENSG00000115756	HPCAL1	2:10443014-10567743	q1	q2	OK	7.16021	6.71653	-0.0922865	-0.148369	0.82385	0.99995	no

ENSG00000115758	ENSG00000115758	ODC1	2:10580093-10588630	q1	q2	OK	196.216	197.789	0.0115178	0.0266536	0.972	0.99995	no

ENSG00000115760	ENSG00000115760	BIRC6	2:32582095-32843966	q1	q2	OK	25.1699	26.4294	0.0704426	0.0777011	0.86285	0.99995	no

ENSG00000115761	ENSG00000115761	NOL10	2:10710891-10830101	q1	q2	OK	13.5743	15.1645	0.159824	0.334108	0.61865	0.99995	no

ENSG00000115762	ENSG00000115762	PLEKHB2	2:131862419-132111282	q1	q2	OK	33.5489	36.51	0.122024	0.286972	0.6547	0.99995	no

ENSG00000115806	ENSG00000115806	GORASP2	2:171784973-171823639	q1	q2	OK	48.3423	47.7481	-0.0178444	-0.0371784	0.95205	0.99995	no

ENSG00000115808	ENSG00000115808	STRN	2:37070782-37193615	q1	q2	OK	8.82637	10.2608	0.217252	0.514534	0.46015	0.99995	no

ENSG00000115816	ENSG00000115816	CEBPZ	2:37394962-37551951	q1	q2	OK	26.6401	31.2677	0.231072	0.220364	0.6681	0.99995	no

ENSG00000115825	ENSG00000115825	PRKD3	2:37394962-37551951	q1	q2	OK	26.9934	30.9717	0.198344	0.207619	0.6095	0.99995	no

ENSG00000115827	ENSG00000115827	DCAF17	2:172173918-172341562	q1	q2	OK	10.4931	11.4669	0.128038	0.129366	0.7991	0.99995	no

ENSG00000115828	ENSG00000115828	QPCT	2:37571716-37600465	q1	q2	OK	0.608453	0.735781	0.274129	0.226622	0.6731	0.99995	no

ENSG00000115839	ENSG00000115839	RAB3GAP1	2:135809834-136288806	q1	q2	OK	22.8621	23.1299	0.0168031	0.0335739	0.9584	0.99995	no

ENSG00000115840	ENSG00000115840	SLC25A12	2:172640879-172947158	q1	q2	OK	8.30612	8.63448	0.0559341	0.0505055	0.93345	0.99995	no

ENSG00000115841	ENSG00000115841	RMDN2	2:38150329-38408997	q1	q2	OK	2.24297	2.3002	0.0363516	0.0117807	0.97755	0.99995	no

ENSG00000115844	ENSG00000115844	DLX2	2:172964166-172967628	q1	q2	OK	0.35215	0.514001	0.545582	0.525368	0.4499	0.99995	no

ENSG00000115850	ENSG00000115850	LCT	2:136545409-136594750	q1	q2	NOTEST	0.0228764	0.00783686	-1.54551	0	1	1	no

ENSG00000115866	ENSG00000115866	DARS	2:136664246-136779888	q1	q2	OK	31.6723	36.3728	0.19964	0.434154	0.50385	0.99995	no

ENSG00000115875	ENSG00000115875	SRSF7	2:38970740-38978636	q1	q2	OK	188.427	211.217	0.164715	0.362625	0.595	0.99995	no

ENSG00000115884	ENSG00000115884	SDC1	2:20400557-20425194	q1	q2	OK	25.2303	22.9362	-0.137531	-0.321645	0.6344	0.99995	no

ENSG00000115896	ENSG00000115896	PLCL1	2:199164086-199637080	q1	q2	OK	1.52833	1.10799	-0.464005	-0.639831	0.3662	0.99995	no

ENSG00000115902	ENSG00000115902	SLC1A4	2:65215610-65250999	q1	q2	OK	15.351	12.7543	-0.267349	-0.459909	0.3806	0.99995	no

ENSG00000115904	ENSG00000115904	SOS1	2:39208536-39351486	q1	q2	OK	17.6913	18.5569	0.0689091	0.0757969	0.84845	0.99995	no

ENSG00000115919	ENSG00000115919	KYNU	2:143635066-143799890	q1	q2	NOTEST	0.0181923	0.0502791	1.46663	0	1	1	no

ENSG00000115934	ENSG00000115934	RP11-153K16.2	12:22852792-23404433	q1	q2	NOTEST	0	0	0	0	1	1	no

ENSG00000115935	ENSG00000115935	WIPF1	2:175411868-175639555	q1	q2	OK	10.8484	12.597	0.215595	0.156946	0.75635	0.99995	no

ENSG00000115942	ENSG00000115942	ORC2	2:201773695-201950473	q1	q2	OK	12.8651	14.9549	0.217157	0.251962	0.64295	0.99995	no

ENSG00000115944	ENSG00000115944	COX7A2L	2:42560685-42652228	q1	q2	OK	81.5428	80.4353	-0.0197288	-0.0465141	0.9468	0.99995	no

ENSG00000115946	ENSG00000115946	PNO1	2:68350067-68488362	q1	q2	OK	9.92014	11.4628	0.208527	0.177557	0.77525	0.99995	no

ENSG00000115947	ENSG00000115947	ORC4	2:148602085-149275805	q1	q2	OK	32.5736	31.2927	-0.0578789	-0.0580978	0.91045	0.99995	no

ENSG00000115956	ENSG00000115956	PLEK	2:68588345-68624585	q1	q2	NOTEST	0.0641634	0.0708034	0.142068	0	1	1	no

ENSG00000115963	ENSG00000115963	RND3	2:151324708-151395525	q1	q2	OK	67.1109	68.5961	0.0315797	0.0665678	0.9086	0.99995	no

ENSG00000115966	ENSG00000115966	ATF2	2:175936977-176033250	q1	q2	OK	25.9905	27.4372	0.0781453	0.162567	0.78215	0.99995	no

ENSG00000115970	ENSG00000115970	THADA	2:43393799-43823185	q1	q2	OK	9.75032	9.73324	-0.00253034	-0.00280428	0.99555	0.99995	no

ENSG00000115977	ENSG00000115977	AAK1	2:69685128-70053596	q1	q2	OK	4.82996	5.9043	0.289755	0.209164	0.65195	0.99995	no

ENSG00000115993	ENSG00000115993	TRAK2	2:202241929-202345569	q1	q2	OK	9.43223	9.92247	0.0730999	0.126032	0.84915	0.99995	no

ENSG00000115998	ENSG00000115998	C2orf42	2:70377011-70475792	q1	q2	OK	7.55745	7.15893	-0.0781541	-0.025017	0.93855	0.99995	no

ENSG00000116001	ENSG00000116001	TIA1	2:70377011-70475792	q1	q2	OK	161.156	164.74	0.031734	0.0414415	0.9263	0.99995	no

ENSG00000116005	ENSG00000116005	PCYOX1	2:70484517-70508323	q1	q2	OK	25.7003	27.6119	0.103505	0.230592	0.72375	0.99995	no

ENSG00000116014	ENSG00000116014	KISS1R	19:917286-921015	q1	q2	OK	4.36438	4.0963	-0.0914552	-0.0821868	0.90715	0.99995	no

ENSG00000116016	ENSG00000116016	EPAS1	2:46520805-46613836	q1	q2	OK	1.08948	1.12979	0.05241	0.0338775	0.9414	0.99995	no

ENSG00000116017	ENSG00000116017	ARID3A	19:925780-975939	q1	q2	OK	11.8046	10.0574	-0.231098	-0.223929	0.5195	0.99995	no

ENSG00000116030	ENSG00000116030	SUMO1	2:203070902-203103331	q1	q2	OK	158.007	165.211	0.0643214	0.154398	0.8194	0.99995	no

ENSG00000116031	ENSG00000116031	CD207	2:71057346-71062952	q1	q2	NOTEST	0	0	0	0	1	1	no

ENSG00000116032	ENSG00000116032	GRIN3B	19:999600-1021117	q1	q2	NOTEST	0.383639	0.298067	-0.364114	0	1	1	no

ENSG00000116035	ENSG00000116035	VAX2	2:71127719-71160576	q1	q2	NOTEST	0	0	0	0	1	1	no

ENSG00000116039	ENSG00000116039	ATP6V1B1	2:71163011-71306935	q1	q2	NOTEST	0	0	0	0	1	1	no

ENSG00000116044	ENSG00000116044	NFE2L2	2:178092322-178408564	q1	q2	OK	24.4377	23.9959	-0.0263205	-0.0296704	0.9656	0.99995	no

ENSG00000116062	ENSG00000116062	MSH6	2:47922668-48134949	q1	q2	OK	85.1947	94.6848	0.152368	0.167371	0.7451	0.99995	no

ENSG00000116095	ENSG00000116095	PLEKHA3	2:179345194-179381189	q1	q2	OK	6.34585	6.03879	-0.0715558	-0.0536295	0.9334	0.99995	no

ENSG00000116096	ENSG00000116096	SPR	2:73114488-73119287	q1	q2	OK	4.39896	3.92455	-0.164635	-0.227131	0.74275	0.99995	no

ENSG00000116106	ENSG00000116106	EPHA4	2:222282746-222439174	q1	q2	OK	44.2274	51.8574	0.229611	0.350284	0.46385	0.99995	no

ENSG00000116117	ENSG00000116117	PARD3B	2:205410515-206484886	q1	q2	OK	7.38742	7.52424	0.0264746	0.0470282	0.93405	0.99995	no

ENSG00000116120	ENSG00000116120	FARSB	2:223435254-223521056	q1	q2	OK	28.5889	31.7905	0.153143	0.349849	0.62605	0.99995	no

ENSG00000116127	ENSG00000116127	ALMS1	2:73612885-73837920	q1	q2	OK	12.3435	12.7906	0.0513289	0.0406686	0.91665	0.99995	no

ENSG00000116128	ENSG00000116128	BCL9	1:147013181-147098017	q1	q2	OK	0.403187	0.701186	0.798348	0.313864	0.5434	0.99995	no

ENSG00000116132	ENSG00000116132	PRRX1	1:170631868-170708560	q1	q2	OK	22.4548	23.0016	0.0347099	0.0555509	0.93225	0.99995	no

ENSG00000116133	ENSG00000116133	DHCR24	1:55315305-55354523	q1	q2	OK	125.195	140.162	0.162919	0.3548	0.5992	0.99995	no

ENSG00000116138	ENSG00000116138	DNAJC16	1:15853307-15930121	q1	q2	OK	8.25034	7.11721	-0.213142	-0.229018	0.58875	0.99995	no

ENSG00000116141	ENSG00000116141	MARK1	1:220701567-220837803	q1	q2	OK	11.3981	11.3033	-0.0120465	-0.0251614	0.97095	0.99995	no

ENSG00000116147	ENSG00000116147	TNR	1:175276353-175712906	q1	q2	NOTEST	0.0226338	0.0347275	0.617603	0	1	1	no

ENSG00000116151	ENSG00000116151	MORN1	1:2252691-2323146	q1	q2	OK	4.30436	4.94905	0.201353	0.165617	0.7837	0.99995	no

ENSG00000116157	ENSG00000116157	GPX7	1:53068043-53074723	q1	q2	OK	3.81645	3.87929	0.0235602	0.0294264	0.9649	0.99995	no

ENSG00000116161	ENSG00000116161	CACYBP	1:174968299-174992561	q1	q2	OK	84.7485	88.987	0.0704068	0.122166	0.85825	0.99995	no

ENSG00000116171	ENSG00000116171	SCP2	1:53392900-53517375	q1	q2	OK	68.9594	74.3282	0.108162	0.229914	0.74615	0.99995	no

ENSG00000116176	ENSG00000116176	TPSG1	16:1203240-1275257	q1	q2	NOTEST	0.294783	0	#NAME?	0	1	1	no

ENSG00000116183	ENSG00000116183	PAPPA2	1:176432306-176814735	q1	q2	NOTEST	0.309066	0.326986	0.0813112	0	1	1	no

ENSG00000116191	ENSG00000116191	RALGPS2	1:178693440-178889238	q1	q2	OK	21.4746	18.9944	-0.177055	-0.162667	0.7496	0.99995	no

ENSG00000116194	ENSG00000116194	ANGPTL1	1:178693440-178889238	q1	q2	OK	12.2698	13.3036	0.116711	0.0670454	0.9286	0.99995	no

ENSG00000116198	ENSG00000116198	CEP104	1:3728644-3773778	q1	q2	OK	11.7934	11.5692	-0.0276896	-0.0278169	0.95455	0.99995	no

ENSG00000116199	ENSG00000116199	FAM20B	1:178994938-179045697	q1	q2	OK	15.2156	15.7625	0.0509486	0.123543	0.8547	0.99995	no

ENSG00000116205	ENSG00000116205	TCEANC2	1:54519259-54578192	q1	q2	OK	4.085	3.72289	-0.133914	-0.206802	0.7417	0.99995	no

ENSG00000116209	ENSG00000116209	TMEM59	1:54497346-54519177	q1	q2	OK	50.4674	51.4352	0.0274039	0.0622075	0.9284	0.99995	no

ENSG00000116212	ENSG00000116212	LRRC42	1:54411749-54433841	q1	q2	OK	30.1875	33.7771	0.162096	0.356345	0.60575	0.99995	no

ENSG00000116213	ENSG00000116213	WRAP73	1:3547330-3663900	q1	q2	OK	16.5441	16.1152	-0.037891	-0.0362651	0.94435	0.99995	no

ENSG00000116218	ENSG00000116218	NPHS2	1:179334854-179545087	q1	q2	NOTEST	0.0170272	0.0156816	-0.118767	0	1	1	no

ENSG00000116221	ENSG00000116221	MRPL37	1:54598746-54879152	q1	q2	OK	42.6436	42.2607	-0.0130157	-0.0141574	0.98245	0.99995	no

ENSG00000116237	ENSG00000116237	ICMT	1:6241328-6296032	q1	q2	OK	34.1347	35.1681	0.043029	0.0483196	0.94495	0.99995	no

ENSG00000116251	ENSG00000116251	RPL22	1:6241328-6296032	q1	q2	OK	334.47	341.157	0.0285584	0.0402935	0.95045	0.99995	no

ENSG00000116254	ENSG00000116254	CHD5	1:6161852-6240183	q1	q2	NOTEST	0.157413	0.0438597	-1.84359	0	1	1	no

ENSG00000116260	ENSG00000116260	QSOX1	1:180123968-180173165	q1	q2	OK	15.2005	15.9629	0.0706009	0.108789	0.87505	0.99995	no

ENSG00000116266	ENSG00000116266	STXBP3	1:109289295-109352148	q1	q2	OK	22.826	25.2625	0.146317	0.252672	0.6529	0.99995	no

ENSG00000116273	ENSG00000116273	PHF13	1:6650783-6684093	q1	q2	OK	16.9208	16.8789	-0.00357429	-0.00702824	0.99225	0.99995	no

ENSG00000116285	ENSG00000116285	ERRFI1	1:8064463-8086368	q1	q2	OK	36.2496	37.4257	0.0460649	0.103573	0.87405	0.99995	no

ENSG00000116288	ENSG00000116288	PARK7	1:8014350-8045565	q1	q2	OK	224.921	234.1	0.0577052	0.138133	0.843	0.99995	no

ENSG00000116299	ENSG00000116299	KIAA1324	1:109648572-109749401	q1	q2	OK	0.682419	0.454263	-0.587131	-0.282645	0.38705	0.99995	no

ENSG00000116329	ENSG00000116329	OPRD1	1:29138653-29190208	q1	q2	NOTEST	0.0159018	0.136873	3.10558	0	1	1	no

ENSG00000116337	ENSG00000116337	AMPD2	1:110158725-110174673	q1	q2	OK	31.7549	31.2927	-0.021151	-0.0313653	0.95915	0.99995	no

ENSG00000116350	ENSG00000116350	SRSF4	1:29474254-29508499	q1	q2	OK	89.2231	89.9529	0.0117523	0.0108648	0.96855	0.99995	no

ENSG00000116353	ENSG00000116353	MECR	1:29519384-29557454	q1	q2	OK	12.5369	12.0811	-0.0534304	-0.0801281	0.88875	0.99995	no

ENSG00000116396	ENSG00000116396	KCNC4	1:110753964-110825722	q1	q2	OK	1.28408	1.08715	-0.240191	-0.274956	0.6706	0.99995	no

ENSG00000116406	ENSG00000116406	EDEM3	1:184659364-184724047	q1	q2	OK	10.1489	10.7721	0.0859675	0.183714	0.79095	0.99995	no

ENSG00000116455	ENSG00000116455	WDR77	1:111981259-112005395	q1	q2	OK	27.2418	28.1025	0.044875	0.0517165	0.9304	0.99995	no

ENSG00000116459	ENSG00000116459	ATP5F1	1:111981259-112005395	q1	q2	OK	201.46	199.747	-0.0123224	-0.02351	0.97275	0.99995	no

ENSG00000116473	ENSG00000116473	RAP1A	1:112025969-112310638	q1	q2	OK	30.3794	34.1955	0.170711	0.226495	0.73635	0.99995	no

ENSG00000116478	ENSG00000116478	HDAC1	1:32757686-32799236	q1	q2	OK	78.5394	79.2525	0.0130401	0.0283955	0.9653	0.99995	no

ENSG00000116489	ENSG00000116489	CAPZA1	1:113009162-113214241	q1	q2	OK	72.3816	79.494	0.135224	0.255201	0.65085	0.99995	no

ENSG00000116497	ENSG00000116497	S100PBP	1:33240839-33324476	q1	q2	OK	28.4656	26.494	-0.103556	-0.0805274	0.80325	0.99995	no

ENSG00000116514	ENSG00000116514	RNF19B	1:33402045-33430286	q1	q2	OK	7.83658	7.0916	-0.144112	-0.259768	0.70765	0.99995	no

ENSG00000116521	ENSG00000116521	SCAMP3	1:155225769-155232221	q1	q2	NOTEST	0	0	0	0	1	1	no

ENSG00000116525	ENSG00000116525	TRIM62	1:33611002-33647660	q1	q2	OK	10.1956	9.27616	-0.136349	-0.224139	0.6898	0.99995	no

ENSG00000116539	ENSG00000116539	ASH1L	1:155305058-155533775	q1	q2	OK	9.38158	10.6112	0.177685	0.20346	0.55945	0.99995	no

ENSG00000116544	ENSG00000116544	DLGAP3	1:35331036-35395186	q1	q2	OK	3.89203	3.6948	-0.0750249	-0.123537	0.8503	0.99995	no

ENSG00000116560	ENSG00000116560	SFPQ	1:35641978-35658749	q1	q2	OK	389.223	436.12	0.164126	0.246985	0.6808	0.99995	no

ENSG00000116574	ENSG00000116574	RHOU	1:228870823-228882416	q1	q2	OK	13.4143	10.0501	-0.416558	-0.411375	0.3232	0.99995	no

ENSG00000116580	ENSG00000116580	GON4L	1:155579566-155829191	q1	q2	OK	15.038	14.6478	-0.0379298	-0.0289527	0.9398	0.99995	no

ENSG00000116584	ENSG00000116584	ARHGEF2	1:155916629-155976861	q1	q2	OK	38.8446	44.0617	0.18181	0.321603	0.5445	0.99995	no

ENSG00000116586	ENSG00000116586	LAMTOR2	1:156024542-156028301	q1	q2	OK	45.1013	40.3005	-0.16237	-0.30387	0.6628	0.99995	no

ENSG00000116604	ENSG00000116604	MEF2D	1:156433518-156470620	q1	q2	OK	4.50852	3.80465	-0.244891	-0.375778	0.53005	0.99995	no

ENSG00000116641	ENSG00000116641	DOCK7	1:62920398-63153969	q1	q2	OK	24.6977	28.5547	0.209353	0.419383	0.51235	0.99995	no

ENSG00000116649	ENSG00000116649	SRM	1:11114640-11120081	q1	q2	OK	43.1828	40.2892	-0.100063	-0.20949	0.75205	0.99995	no

ENSG00000116652	ENSG00000116652	DLEU2L	1:63906440-64125916	q1	q2	OK	0.876475	0.387869	-1.17614	-0.164987	0.5698	0.99995	no

ENSG00000116661	ENSG00000116661	FBXO2	1:11708423-11723384	q1	q2	OK	1.26009	0.437509	-1.52614	-0.567504	0.2564	0.99995	no

ENSG00000116663	ENSG00000116663	FBXO6	1:11724180-11734411	q1	q2	OK	0.852426	0.959512	0.170726	0.175015	0.79795	0.99995	no

ENSG00000116667	ENSG00000116667	C1orf21	1:184356191-184598154	q1	q2	OK	25.153	26.6129	0.0813961	0.0861601	0.8983	0.99995	no

ENSG00000116668	ENSG00000116668	SWT1	1:185126211-185260897	q1	q2	OK	3.48723	3.32392	-0.0691947	-0.106979	0.86925	0.99995	no

ENSG00000116670	ENSG00000116670	MAD2L2	1:11734536-11751707	q1	q2	OK	45.0766	36.8475	-0.290809	-0.609532	0.39435	0.99995	no

ENSG00000116675	ENSG00000116675	DNAJC6	1:65713901-65881689	q1	q2	OK	2.69337	2.52807	-0.0913728	-0.0972784	0.82845	0.99995	no

ENSG00000116678	ENSG00000116678	LEPR	1:65886247-66107242	q1	q2	OK	1.35724	1.26304	-0.103777	-0.0618516	0.91305	0.99995	no

ENSG00000116679	ENSG00000116679	IVNS1ABP	1:185265519-185286461	q1	q2	OK	89.084	96.0705	0.108928	0.212206	0.71545	0.99995	no

ENSG00000116685	ENSG00000116685	KIAA2013	1:11979647-11986485	q1	q2	OK	13.1579	12.4036	-0.085172	-0.177883	0.7985	0.99995	no

ENSG00000116688	ENSG00000116688	MFN2	1:12040237-12073571	q1	q2	OK	31.6118	30.0465	-0.0732657	-0.142807	0.79665	0.99995	no

ENSG00000116690	ENSG00000116690	PRG4	1:186265404-186344825	q1	q2	NOTEST	0.151531	0.158541	0.0652425	0	1	1	no

ENSG00000116691	ENSG00000116691	MIIP	1:12079522-12092102	q1	q2	OK	21.7828	19.5897	-0.153096	-0.288502	0.67185	0.99995	no

ENSG00000116698	ENSG00000116698	SMG7	1:183441350-183567381	q1	q2	OK	30.6351	29.6114	-0.0490294	-0.0858811	0.8573	0.99995	no

ENSG00000116701	ENSG00000116701	NCF2	1:183441350-183567381	q1	q2	NOTEST	0.0873136	0.0117197	-2.89728	0	1	1	no

ENSG00000116703	ENSG00000116703	PDC	1:186412697-186430254	q1	q2	NOTEST	0.17132	0.307767	0.845147	0	1	1	no

ENSG00000116704	ENSG00000116704	SLC35D1	1:67465014-67519782	q1	q2	OK	6.41483	6.41558	0.000168042	0.000350465	0.99915	0.99995	no

ENSG00000116711	ENSG00000116711	PLA2G4A	1:186798084-186958113	q1	q2	OK	2.33579	1.47501	-0.663184	-0.873014	0.2015	0.99995	no

ENSG00000116717	ENSG00000116717	GADD45A	1:68150743-68154021	q1	q2	OK	20.0581	18.0549	-0.151797	-0.285942	0.67675	0.99995	no

ENSG00000116721	ENSG00000116721	PRAMEF1	1:12851545-12856777	q1	q2	NOTEST	0	0	0	0	1	1	no

ENSG00000116726	ENSG00000116726	PRAMEF12	1:12834983-12838049	q1	q2	NOTEST	0	0	0	0	1	1	no

ENSG00000116729	ENSG00000116729	WLS	1:68167148-68698803	q1	q2	OK	85.5528	76.6215	-0.159065	-0.260587	0.6604	0.99995	no

ENSG00000116731	ENSG00000116731	PRDM2	1:14026692-14151574	q1	q2	OK	18.8438	19.3842	0.0407942	0.0736945	0.8882	0.99995	no

ENSG00000116741	ENSG00000116741	RGS2	1:192778168-192781403	q1	q2	OK	8.06575	8.23332	0.0296653	0.0458065	0.94425	0.99995	no

ENSG00000116745	ENSG00000116745	RPE65	1:68894504-68915642	q1	q2	NOTEST	0.160213	0.183394	0.194952	0	1	1	no

ENSG00000116747	ENSG00000116747	TROVE2	1:192981379-193060907	q1	q2	OK	25.1495	28.5958	0.185275	0.178695	0.64365	0.99995	no

ENSG00000116748	ENSG00000116748	AMPD1	1:115215718-115238239	q1	q2	NOTEST	0.0108899	0	#NAME?	0	1	1	no

ENSG00000116750	ENSG00000116750	UCHL5	1:192981379-193060907	q1	q2	OK	19.1115	22.6469	0.24487	0.225115	0.69825	0.99995	no

ENSG00000116752	ENSG00000116752	BCAS2	1:115110177-115124260	q1	q2	OK	43.0044	45.7237	0.0884568	0.201427	0.7765	0.99995	no

ENSG00000116754	ENSG00000116754	SRSF11	1:70671364-70718735	q1	q2	OK	176.448	180.311	0.0312439	0.0522708	0.9289	0.99995	no

ENSG00000116761	ENSG00000116761	CTH	1:70876900-70905534	q1	q2	OK	6.14387	5.74179	-0.0976457	-0.139049	0.8259	0.99995	no

ENSG00000116771	ENSG00000116771	AGMAT	1:15853307-15930121	q1	q2	NOTEST	0.367829	0.255104	-0.527951	0	1	1	no

ENSG00000116774	ENSG00000116774	OLFML3	1:114522062-114524876	q1	q2	OK	18.571	18.7999	0.0176711	0.0345092	0.96135	0.99995	no

ENSG00000116783	ENSG00000116783	TNNI3K	1:74663895-75010112	q1	q2	NOTEST	0.102033	0.104313	0.0318863	0	1	1	no

ENSG00000116785	ENSG00000116785	CFHR3	1:196743924-196763203	q1	q2	NOTEST	0	0	0	0	1	1	no

ENSG00000116786	ENSG00000116786	PLEKHM2	1:16010826-16076391	q1	q2	OK	19.5145	17.2185	-0.180585	-0.413842	0.5504	0.99995	no

ENSG00000116791	ENSG00000116791	CRYZ	1:75171169-75232362	q1	q2	OK	22.0581	23.9907	0.121168	0.211116	0.7644	0.99995	no

ENSG00000116793	ENSG00000116793	PHTF1	1:114239452-114302111	q1	q2	OK	37.7667	36.3127	-0.0566385	-0.103121	0.8759	0.99995	no

ENSG00000116809	ENSG00000116809	ZBTB17	1:16268363-16302627	q1	q2	OK	24.715	23.8992	-0.0484248	-0.0737176	0.89645	0.99995	no

ENSG00000116815	ENSG00000116815	CD58	1:117057156-117113661	q1	q2	OK	7.76183	8.39874	0.113776	0.156614	0.8198	0.99995	no

ENSG00000116819	ENSG00000116819	TFAP2E	1:36035413-36060929	q1	q2	NOTEST	0.390225	0.404208	0.0507927	0	1	1	no

ENSG00000116824	ENSG00000116824	CD2	1:117297006-117311850	q1	q2	NOTEST	0	0	0	0	1	1	no

ENSG00000116830	ENSG00000116830	TTF2	1:117602924-117650075	q1	q2	OK	10.0855	11.8621	0.234082	0.253291	0.64165	0.99995	no

ENSG00000116833	ENSG00000116833	NR5A2	1:199996729-200146552	q1	q2	OK	11.6493	12.6247	0.116006	0.250303	0.71325	0.99995	no

ENSG00000116852	ENSG00000116852	KIF21B	1:200938519-200992828	q1	q2	OK	10.053	9.62302	-0.0630657	-0.142578	0.8376	0.99995	no

ENSG00000116857	ENSG00000116857	TMEM9	1:201103899-201140702	q1	q2	OK	53.3468	49.3144	-0.113393	-0.253578	0.7137	0.99995	no

ENSG00000116863	ENSG00000116863	ADPRHL2	1:36554475-36559533	q1	q2	OK	22.017	20.0575	-0.134479	-0.280778	0.68535	0.99995	no

ENSG00000116871	ENSG00000116871	MAP7D1	1:36621179-36646450	q1	q2	OK	77.776	77.254	-0.00971481	-0.0210489	0.97485	0.99995	no

ENSG00000116874	ENSG00000116874	WARS2	1:119573838-119869926	q1	q2	OK	6.25897	6.83069	0.126108	0.196775	0.75545	0.99995	no

ENSG00000116882	ENSG00000116882	HAO2	1:119911401-119936753	q1	q2	NOTEST	0.0205735	0.0189646	-0.117474	0	1	1	no

ENSG00000116883	ENSG00000116883	RP11-268J15.5	1:36771987-36794822	q1	q2	OK	2.42634	1.67811	-0.531943	-0.374614	0.58775	0.99995	no

ENSG00000116885	ENSG00000116885	OSCP1	1:36881427-36916086	q1	q2	OK	7.17365	8.03714	0.163976	0.173619	0.74525	0.99995	no

ENSG00000116898	ENSG00000116898	MRPS15	1:36921318-36930038	q1	q2	OK	43.4334	45.2469	0.0590111	0.121946	0.86325	0.99995	no

ENSG00000116903	ENSG00000116903	EXOC8	1:231468479-231490769	q1	q2	OK	3.86117	4.30427	0.156731	0.233602	0.7466	0.99995	no

ENSG00000116906	ENSG00000116906	GNPAT	1:231376952-231413719	q1	q2	OK	42.1972	44.2525	0.0686113	0.144658	0.80985	0.99995	no

ENSG00000116918	ENSG00000116918	TSNAX	1:231664398-232177018	q1	q2	OK	22.6762	24.5233	0.11297	0.212544	0.74185	0.99995	no

ENSG00000116922	ENSG00000116922	C1orf109	1:38147241-38157914	q1	q2	OK	19.6016	19.775	0.012712	0.0207998	0.9733	0.99995	no

ENSG00000116954	ENSG00000116954	RRAGC	1:39303869-39325495	q1	q2	OK	17.4252	17.1971	-0.0190072	-0.0405311	0.95205	0.99995	no

ENSG00000116957	ENSG00000116957	TBCE	1:235530674-235612283	q1	q2	OK	22.157	23.1258	0.0617393	0.0978778	0.86645	0.99995	no

ENSG00000116962	ENSG00000116962	NID1	1:236139129-236228462	q1	q2	OK	36.3264	39.7985	0.131693	0.320543	0.64675	0.99995	no

ENSG00000116977	ENSG00000116977	LGALS8	1:236681299-236767804	q1	q2	OK	7.86845	9.89656	0.330849	0.175551	0.5703	0.99995	no

ENSG00000116981	ENSG00000116981	NT5C1A	1:40124792-40137710	q1	q2	NOTEST	0	0.106925	inf	0	1	1	no

ENSG00000116983	ENSG00000116983	HPCAL4	1:40144319-40157361	q1	q2	OK	0.876221	0.984164	0.167603	0.213282	0.76195	0.99995	no

ENSG00000116984	ENSG00000116984	MTR	1:236958609-237067281	q1	q2	OK	9.29748	9.91182	0.0923103	0.213583	0.76095	0.99995	no

ENSG00000116985	ENSG00000116985	BMP8B	1:40157853-40254533	q1	q2	NOTEST	0.325194	0.397622	0.290097	0	1	1	no

ENSG00000116990	ENSG00000116990	MYCL	1:40361097-40367928	q1	q2	OK	28.5527	21.9475	-0.379572	-0.80954	0.2316	0.99995	no

ENSG00000116991	ENSG00000116991	SIPA1L2	1:232533710-232697304	q1	q2	OK	43.2134	46.4538	0.104318	0.165512	0.7304	0.99995	no

ENSG00000116996	ENSG00000116996	ZP4	1:238025474-238091621	q1	q2	NOTEST	0	0	0	0	1	1	no

ENSG00000117000	ENSG00000117000	RLF	1:40627044-40706593	q1	q2	OK	11.3147	12.4508	0.13804	0.327808	0.6371	0.99995	no

ENSG00000117009	ENSG00000117009	KMO	1:241695433-241965435	q1	q2	NOTEST	0.0933276	0.103805	0.153497	0	1	1	no

ENSG00000117010	ENSG00000117010	ZNF684	1:40997232-41013841	q1	q2	OK	3.38649	4.41438	0.382421	0.472676	0.44525	0.99995	no

ENSG00000117013	ENSG00000117013	KCNQ4	1:41249683-41306124	q1	q2	OK	0.520078	0.425891	-0.288245	-0.276246	0.68675	0.99995	no

ENSG00000117016	ENSG00000117016	RIMS3	1:41086350-41131329	q1	q2	OK	9.1081	9.5771	0.0724398	0.16628	0.81295	0.99995	no

ENSG00000117020	ENSG00000117020	AKT3	1:243419319-244014381	q1	q2	OK	18.002	18.6493	0.0509675	0.101458	0.8717	0.99995	no

ENSG00000117036	ENSG00000117036	ETV3	1:157090982-157108266	q1	q2	OK	9.69461	10.4465	0.107764	0.226775	0.7313	0.99995	no

ENSG00000117054	ENSG00000117054	ACADM	1:76190035-76260764	q1	q2	OK	57.4139	59.585	0.0535496	0.0945653	0.88505	0.99995	no

ENSG00000117069	ENSG00000117069	ST6GALNAC5	1:77333125-77531396	q1	q2	OK	36.5047	58.6073	0.682999	1.3293	0.03825	0.99995	no

ENSG00000117090	ENSG00000117090	SLAMF1	1:160577889-160617085	q1	q2	NOTEST	0.0503907	0	#NAME?	0	1	1	no

ENSG00000117091	ENSG00000117091	CD48	1:160639937-160681641	q1	q2	NOTEST	0.07831	0.0499659	-0.648251	0	1	1	no

ENSG00000117114	ENSG00000117114	LPHN2	1:81771844-82458120	q1	q2	OK	105.717	110.07	0.0582097	0.0941117	0.84785	0.99995	no

ENSG00000117115	ENSG00000117115	PADI2	1:17393255-17445948	q1	q2	NOTEST	0.0351439	0.0223632	-0.652148	0	1	1	no

ENSG00000117118	ENSG00000117118	SDHB	1:17345216-17380665	q1	q2	OK	32.437	33.2664	0.0364257	0.0737069	0.90865	0.99995	no

ENSG00000117122	ENSG00000117122	MFAP2	1:17300996-17338423	q1	q2	OK	29.4665	26.4129	-0.15783	-0.21575	0.74745	0.99995	no

ENSG00000117133	ENSG00000117133	RPF1	1:84944941-84963473	q1	q2	OK	29.352	32.7577	0.158377	0.339884	0.6272	0.99995	no

ENSG00000117139	ENSG00000117139	KDM5B	1:202696525-202778598	q1	q2	OK	56.1877	54.796	-0.036183	-0.0677541	0.9001	0.99995	no

ENSG00000117143	ENSG00000117143	UAP1	1:162531322-162569627	q1	q2	OK	20.3332	21.4234	0.0753513	0.153588	0.815	0.99995	no

ENSG00000117148	ENSG00000117148	ACTL8	1:18081807-18153558	q1	q2	NOTEST	0.0571462	0.0445329	-0.359786	0	1	1	no

ENSG00000117151	ENSG00000117151	CTBS	1:84964007-85040163	q1	q2	OK	1.98801	2.06994	0.0582627	0.0291631	0.9698	0.99995	no

ENSG00000117152	ENSG00000117152	RGS4	1:163038564-163046592	q1	q2	OK	2.30555	2.43321	0.0777497	0.0692327	0.90855	0.99995	no

ENSG00000117153	ENSG00000117153	KLHL12	1:202860227-202897764	q1	q2	OK	18.9871	20.0353	0.077529	0.176648	0.80595	0.99995	no

ENSG00000117154	ENSG00000117154	IGSF21	1:18434239-18704977	q1	q2	NOTEST	0	0	0	0	1	1	no

ENSG00000117155	ENSG00000117155	SSX2IP	1:85109389-85156486	q1	q2	OK	19.3839	17.6807	-0.132687	-0.231536	0.70045	0.99995	no

ENSG00000117174	ENSG00000117174	ZNHIT6	1:86115105-86174116	q1	q2	OK	11.7285	13.6304	0.216811	0.453443	0.5143	0.99995	no

ENSG00000117215	ENSG00000117215	PLA2G2D	1:20438431-20446050	q1	q2	NOTEST	0	0.00930239	inf	0	1	1	no

ENSG00000117222	ENSG00000117222	RBBP5	1:205055269-205091143	q1	q2	OK	9.86097	10.3024	0.0631781	0.138556	0.84075	0.99995	no

ENSG00000117226	ENSG00000117226	GBP3	1:89472348-89488577	q1	q2	OK	0.552523	0.265372	-1.05802	-0.486807	0.1437	0.99995	no

ENSG00000117228	ENSG00000117228	GBP1	1:89518001-89531043	q1	q2	OK	0.934405	0.965461	0.0471707	0.0334769	0.93975	0.99995	no

ENSG00000117242	ENSG00000117242	PINK1-AS	1:20959947-20988000	q1	q2	OK	2.51805	2.08981	-0.26894	-0.140732	0.8366	0.99995	no

ENSG00000117245	ENSG00000117245	KIF17	1:20990506-21044510	q1	q2	OK	1.12134	0.708009	-0.66338	-0.468275	0.32955	0.99995	no

ENSG00000117262	ENSG00000117262	GPR89A	1:145764410-145827103	q1	q2	OK	0.891189	1.22394	0.457725	0.353824	0.5041	0.99995	no

ENSG00000117266	ENSG00000117266	CDK18	1:205473722-205501921	q1	q2	OK	0.817109	1.10685	0.437862	0.297769	0.4289	0.99995	no

ENSG00000117280	ENSG00000117280	RAB7L1	1:205737113-205744588	q1	q2	OK	3.70273	3.25963	-0.183883	-0.257526	0.6707	0.99995	no

ENSG00000117281	ENSG00000117281	CD160	1:145611035-145715614	q1	q2	NOTEST	0	0.0486887	inf	0	1	1	no

ENSG00000117289	ENSG00000117289	TXNIP	1:145438468-145442635	q1	q2	OK	0.723359	0.404149	-0.839826	-0.702836	0.27055	0.99995	no

ENSG00000117298	ENSG00000117298	ECE1	1:21543739-21671997	q1	q2	OK	17.9989	17.4776	-0.0424039	-0.0742966	0.88625	0.99995	no

ENSG00000117305	ENSG00000117305	HMGCL	1:24128374-24165110	q1	q2	OK	11.5817	10.5618	-0.132989	-0.209408	0.7495	0.99995	no

ENSG00000117308	ENSG00000117308	GALE	1:24122088-24127271	q1	q2	OK	20.9788	19.2872	-0.121291	-0.193513	0.78585	0.99995	no

ENSG00000117318	ENSG00000117318	ID3	1:23884408-23886285	q1	q2	OK	83.8023	81.2429	-0.0447486	-0.0907451	0.8985	0.99995	no

ENSG00000117322	ENSG00000117322	CR2	1:207627574-207663240	q1	q2	NOTEST	0.0133536	0.00677107	-0.979771	0	1	1	no

ENSG00000117335	ENSG00000117335	CD46	1:207925401-207968858	q1	q2	OK	67.8682	73.5539	0.116064	0.195081	0.71805	0.99995	no

ENSG00000117360	ENSG00000117360	PRPF3	1:150293924-150325671	q1	q2	NOTEST	0.466874	0.387721	-0.268013	0	1	1	no

ENSG00000117362	ENSG00000117362	APH1A	1:150237803-150253327	q1	q2	OK	5.13994	1.4034	-1.87282	-1.51729	0.01965	0.99995	no

ENSG00000117385	ENSG00000117385	LEPRE1	1:43212005-43232755	q1	q2	OK	44.3462	39.6229	-0.162476	-0.302787	0.59435	0.99995	no

ENSG00000117394	ENSG00000117394	SLC2A1	1:43391051-43424530	q1	q2	OK	133.082	146.556	0.139132	0.244887	0.70895	0.99995	no

ENSG00000117395	ENSG00000117395	EBNA1BP2	1:43629845-43739673	q1	q2	OK	34.8901	38.0371	0.12459	0.263363	0.71565	0.99995	no

ENSG00000117399	ENSG00000117399	CDC20	1:43824625-43828874	q1	q2	OK	80.5833	80.6947	0.00199311	0.00473383	0.9942	0.99995	no

ENSG00000117400	ENSG00000117400	MPL	1:43803477-43818443	q1	q2	NOTEST	0	0	0	0	1	1	no

ENSG00000117407	ENSG00000117407	ARTN	1:44398991-44402913	q1	q2	OK	1.2834	0.611126	-1.07043	-0.742148	0.10015	0.99995	no

ENSG00000117408	ENSG00000117408	IPO13	1:44412610-44433694	q1	q2	OK	15.0036	14.9578	-0.00441598	-0.00554208	0.9905	0.99995	no

ENSG00000117410	ENSG00000117410	ATP6V0B	1:44440158-44443967	q1	q2	OK	57.1387	51.1043	-0.161023	-0.336177	0.62445	0.99995	no

ENSG00000117411	ENSG00000117411	B4GALT2	1:44444614-44456840	q1	q2	OK	37.2748	33.4554	-0.155963	-0.341848	0.6076	0.99995	no

ENSG00000117419	ENSG00000117419	ERI3	1:44686741-44820932	q1	q2	OK	48.4038	43.0768	-0.168208	-0.353943	0.592	0.99995	no

ENSG00000117425	ENSG00000117425	PTCH2	1:45285515-45308735	q1	q2	OK	1.33408	0.987753	-0.433627	-0.475448	0.43785	0.99995	no

ENSG00000117448	ENSG00000117448	AKR1A1	1:46016214-46035721	q1	q2	OK	71.199	67.6549	-0.0736613	-0.152783	0.82195	0.99995	no

ENSG00000117450	ENSG00000117450	PRDX1	1:45959537-45988719	q1	q2	OK	235.184	244.639	0.0568653	0.135379	0.8429	0.99995	no

ENSG00000117461	ENSG00000117461	PIK3R3	1:46505811-46651630	q1	q2	OK	44.137	48.5161	0.136475	0.265425	0.6528	0.99995	no

ENSG00000117472	ENSG00000117472	TSPAN1	1:46505811-46651630	q1	q2	NOTEST	0.136193	0.0999304	-0.446653	0	1	1	no

ENSG00000117475	ENSG00000117475	BLZF1	1:169337207-169429907	q1	q2	OK	9.07959	9.69439	0.0945242	0.150108	0.8311	0.99995	no

ENSG00000117477	ENSG00000117477	CCDC181	1:169337207-169429907	q1	q2	OK	6.90538	7.49273	0.11777	0.137818	0.84815	0.99995	no

ENSG00000117479	ENSG00000117479	SLC19A2	1:169433146-169455241	q1	q2	OK	7.54813	8.70859	0.20632	0.411064	0.56015	0.99995	no

ENSG00000117480	ENSG00000117480	FAAH	1:46859936-46879520	q1	q2	OK	2.24621	1.68648	-0.413476	-0.355169	0.59245	0.99995	no

ENSG00000117481	ENSG00000117481	NSUN4	1:46805848-46830824	q1	q2	OK	6.58935	6.95171	0.077231	0.125613	0.8475	0.99995	no

ENSG00000117500	ENSG00000117500	TMED5	1:93615298-93835055	q1	q2	OK	31.398	34.1397	0.120779	0.157331	0.7766	0.99995	no

ENSG00000117501	ENSG00000117501	MROH9	1:170904611-171033906	q1	q2	NOTEST	0	0	0	0	1	1	no

ENSG00000117505	ENSG00000117505	DR1	1:93615298-93835055	q1	q2	OK	27.2877	28.7149	0.0735521	0.0801906	0.9054	0.99995	no

ENSG00000117507	ENSG00000117507	FMO6P	1:171106878-171130707	q1	q2	NOTEST	0	0	0	0	1	1	no

ENSG00000117519	ENSG00000117519	CNN3	1:95362506-95392834	q1	q2	OK	259.547	288.327	0.15171	0.330262	0.61625	0.99995	no

ENSG00000117523	ENSG00000117523	PRRC2C	1:171454650-171562650	q1	q2	OK	53.2687	56.5033	0.0850472	0.111133	0.7929	0.99995	no

ENSG00000117525	ENSG00000117525	F3	1:94994780-95007356	q1	q2	OK	2.79555	3.62499	0.374844	0.401544	0.50345	0.99995	no

ENSG00000117528	ENSG00000117528	ABCD3	1:94883932-94984222	q1	q2	OK	28.3211	30.2289	0.0940499	0.197352	0.749	0.99995	no

ENSG00000117533	ENSG00000117533	VAMP4	1:171669299-171711387	q1	q2	OK	5.11919	5.76574	0.171588	0.285794	0.6589	0.99995	no

ENSG00000117543	ENSG00000117543	DPH5	1:101455178-101552821	q1	q2	OK	25.863	26.9926	0.0616758	0.106311	0.878	0.99995	no

ENSG00000117560	ENSG00000117560	FASLG	1:172628153-172636014	q1	q2	NOTEST	0	0	0	0	1	1	no

ENSG00000117569	ENSG00000117569	PTBP2	1:97187220-97289294	q1	q2	OK	70.3061	75.136	0.0958556	0.145097	0.7593	0.99995	no

ENSG00000117586	ENSG00000117586	TNFSF4	1:173152872-173176452	q1	q2	NOTEST	0.104461	0.0663165	-0.655518	0	1	1	no

ENSG00000117592	ENSG00000117592	PRDX6	1:173446404-173457946	q1	q2	OK	109.055	109.88	0.0108829	0.024671	0.97245	0.99995	no

ENSG00000117593	ENSG00000117593	DARS2	1:173768687-173827684	q1	q2	OK	12.9255	13.483	0.0609125	0.115691	0.8606	0.99995	no

ENSG00000117594	ENSG00000117594	HSD11B1	1:209834708-209908295	q1	q2	NOTEST	0	0.0206368	inf	0	1	1	no

ENSG00000117595	ENSG00000117595	IRF6	1:209959035-209979465	q1	q2	NOTEST	0.275963	0.281273	0.0274976	0	1	1	no

ENSG00000117597	ENSG00000117597	DIEXF	1:210001351-210030910	q1	q2	OK	2.6044	2.77824	0.0932215	0.167222	0.80585	0.99995	no

ENSG00000117598	ENSG00000117598	LPPR5	1:99355805-99614408	q1	q2	OK	3.57758	4.45477	0.316368	0.402611	0.4898	0.99995	no

ENSG00000117600	ENSG00000117600	LPPR4	1:99729508-99775146	q1	q2	NOTEST	0.308079	0.2197	-0.487766	0	1	1	no

ENSG00000117601	ENSG00000117601	SERPINC1	1:173872946-173886516	q1	q2	NOTEST	0.140336	0.016542	-3.08467	0	1	1	no

ENSG00000117602	ENSG00000117602	RCAN3	1:24829386-24882515	q1	q2	NOTEST	0.280982	0.35631	0.342656	0	1	1	no

ENSG00000117614	ENSG00000117614	SYF2	1:25549169-25558993	q1	q2	OK	35.8074	37.1756	0.0540984	0.116772	0.8652	0.99995	no

ENSG00000117616	ENSG00000117616	C1orf63	1:25568727-25756683	q1	q2	OK	45.677	45.7563	0.00250337	0.00303745	0.99565	0.99995	no

ENSG00000117620	ENSG00000117620	SLC35A3	1:100433938-100492535	q1	q2	OK	5.242	6.17742	0.236889	0.378822	0.581	0.99995	no

ENSG00000117625	ENSG00000117625	RCOR3	1:211431718-211489727	q1	q2	OK	27.1499	27.0632	-0.00461249	-0.00682437	0.98845	0.99995	no

ENSG00000117632	ENSG00000117632	STMN1	1:26210671-26233482	q1	q2	OK	1117.62	1138.47	0.0266593	0.0513392	0.9398	0.99995	no

ENSG00000117640	ENSG00000117640	MTFR1L	1:26126666-26185903	q1	q2	OK	43.7926	40.907	-0.0983391	-0.112547	0.86735	0.99995	no

ENSG00000117643	ENSG00000117643	MAN1C1	1:25943958-26112698	q1	q2	OK	3.43221	3.07319	-0.159403	-0.226097	0.72595	0.99995	no

ENSG00000117650	ENSG00000117650	NEK2	1:211836113-211848960	q1	q2	OK	23.2147	23.6867	0.0290416	0.0588631	0.9324	0.99995	no

ENSG00000117676	ENSG00000117676	RPS6KA1	1:26856251-26901521	q1	q2	OK	7.41017	6.97814	-0.0866638	-0.138685	0.79645	0.99995	no

ENSG00000117682	ENSG00000117682	DHDDS	1:26758772-26797785	q1	q2	OK	14.0703	15.9487	0.180787	0.256489	0.606	0.99995	no

ENSG00000117691	ENSG00000117691	NENF	1:212606228-212619714	q1	q2	OK	16.4925	15.8048	-0.0614444	-0.102122	0.88605	0.99995	no

ENSG00000117697	ENSG00000117697	NSL1	1:212899494-212965124	q1	q2	OK	27.7623	29.9532	0.109581	0.220357	0.7521	0.99995	no

ENSG00000117707	ENSG00000117707	PROX1	1:213991093-214214595	q1	q2	OK	4.65703	4.55858	-0.0308245	-0.0631651	0.92615	0.99995	no

ENSG00000117713	ENSG00000117713	ARID1A	1:27022523-27108595	q1	q2	OK	55.5837	53.8331	-0.0461694	-0.0820408	0.8896	0.99995	no

ENSG00000117724	ENSG00000117724	CENPF	1:214776537-214837931	q1	q2	OK	102.89	110.171	0.0986385	0.0963076	0.84065	0.99995	no

ENSG00000117748	ENSG00000117748	RPA2	1:28218034-28241257	q1	q2	OK	43.6359	42.6733	-0.0321835	-0.0748008	0.9111	0.99995	no

ENSG00000117751	ENSG00000117751	PPP1R8	1:28157288-28178187	q1	q2	OK	43.3632	43.8465	0.0159902	0.0381727	0.95485	0.99995	no

ENSG00000117758	ENSG00000117758	STX12	1:28099693-28150963	q1	q2	OK	22.6248	22.7133	0.00563681	0.0083511	0.98655	0.99995	no

ENSG00000117791	ENSG00000117791	Mar-02	1:220921566-220958150	q1	q2	OK	2.3499	3.23453	0.460958	0.440131	0.5327	0.99995	no

ENSG00000117834	ENSG00000117834	SLC5A9	1:48688356-48714316	q1	q2	NOTEST	0.187638	0.146079	-0.361206	0	1	1	no

ENSG00000117859	ENSG00000117859	OSBPL9	1:52027537-52344477	q1	q2	OK	46.1399	46.8467	0.021933	0.0274992	0.96955	0.99995	no

ENSG00000117862	ENSG00000117862	TXNDC12	1:52485802-52556388	q1	q2	OK	33.4281	36.134	0.112294	0.16386	0.8119	0.99995	no

ENSG00000117868	ENSG00000117868	ESYT2	7:158523685-158622944	q1	q2	OK	12.9199	12.1232	-0.0918142	-0.206807	0.76075	0.99995	no

ENSG00000117877	ENSG00000117877	CD3EAP	19:45882891-45982086	q1	q2	OK	2.027	2.53102	0.320372	0.174346	0.7465	0.99995	no

ENSG00000117899	ENSG00000117899	MESDC2	15:81071683-81282219	q1	q2	OK	24.7392	24.9181	0.0103932	0.0226759	0.97135	0.99995	no

ENSG00000117906	ENSG00000117906	RCN2	15:77223959-77242601	q1	q2	OK	171.22	184.592	0.10849	0.246355	0.7227	0.99995	no

ENSG00000117971	ENSG00000117971	CHRNB4	15:78916460-79027837	q1	q2	NOTEST	0.404463	0.336634	-0.264826	0	1	1	no

ENSG00000117983	ENSG00000117983	MUC5B	11:1244295-1283406	q1	q2	NOTEST	0.0183523	0.00969345	-0.92088	0	1	1	no

ENSG00000117984	ENSG00000117984	CTSD	11:1753639-1785255	q1	q2	OK	51.6078	40.7022	-0.34248	-0.801787	0.24965	0.99995	no

ENSG00000118004	ENSG00000118004	COLEC11	2:3642425-3692048	q1	q2	NOTEST	0.0535583	0.154544	1.52883	0	1	1	no

ENSG00000118007	ENSG00000118007	STAG1	3:135969147-136471220	q1	q2	OK	18.7873	20.9689	0.158494	0.300748	0.6108	0.99995	no

ENSG00000118017	ENSG00000118017	A4GNT	3:137842559-137851229	q1	q2	NOTEST	0	0.0303606	inf	0	1	1	no

ENSG00000118046	ENSG00000118046	STK11	19:1189405-1238026	q1	q2	OK	25.9861	21.0355	-0.304914	-0.46831	0.47425	0.99995	no

ENSG00000118058	ENSG00000118058	KMT2A	11:118307204-118473748	q1	q2	OK	0.794554	2.17491	1.45274	0.794669	0.1171	0.99995	no

ENSG00000118094	ENSG00000118094	TREH	11:118477154-118550399	q1	q2	NOTEST	0	0	0	0	1	1	no

ENSG00000118096	ENSG00000118096	IFT46	11:118307204-118473748	q1	q2	OK	2.18917	1.12916	-0.95513	-0.427051	0.45835	0.99995	no

ENSG00000118113	ENSG00000118113	MMP8	11:102582525-102597781	q1	q2	NOTEST	0.0112546	0	#NAME?	0	1	1	no

ENSG00000118137	ENSG00000118137	APOA1	11:116706466-116969153	q1	q2	NOTEST	0.40874	0.250869	-0.704245	0	1	1	no

ENSG00000118156	ENSG00000118156	ZNF541	19:48023941-48059113	q1	q2	NOTEST	0	0	0	0	1	1	no

ENSG00000118160	ENSG00000118160	SLC8A2	19:47931273-47975425	q1	q2	OK	0.620859	0.765084	0.301352	0.190923	0.6416	0.99995	no

ENSG00000118162	ENSG00000118162	KPTN	19:47978400-47987525	q1	q2	OK	12.2797	9.08124	-0.43532	-0.584835	0.35635	0.99995	no

ENSG00000118181	ENSG00000118181	RPS25	11:118868851-118901616	q1	q2	NOTEST	0.0789837	0.0365936	-1.10996	0	1	1	no

ENSG00000118193	ENSG00000118193	KIF14	1:200520627-200589862	q1	q2	OK	10.1639	11.3461	0.158737	0.373105	0.58835	0.99995	no

ENSG00000118194	ENSG00000118194	TNNT2	1:201328135-201368736	q1	q2	NOTEST	0	0.0582045	inf	0	1	1	no

ENSG00000118197	ENSG00000118197	DDX59	1:200593023-200663378	q1	q2	OK	9.05444	9.16825	0.0180209	0.0256277	0.96635	0.99995	no

ENSG00000118200	ENSG00000118200	CAMSAP2	1:200708685-200829832	q1	q2	OK	20.3815	22.5055	0.143021	0.253956	0.62445	0.99995	no

ENSG00000118217	ENSG00000118217	ATF6	1:161736083-161933860	q1	q2	OK	9.85525	10.2473	0.0562848	0.133876	0.85165	0.99995	no

ENSG00000118231	ENSG00000118231	CRYGD	2:208986330-208989225	q1	q2	NOTEST	0.0599397	0	#NAME?	0	1	1	no

ENSG00000118242	ENSG00000118242	MREG	2:216809212-216967506	q1	q2	OK	4.54802	4.06276	-0.162778	-0.0799119	0.9039	0.99995	no

ENSG00000118245	ENSG00000118245	TNP1	2:217536827-217858802	q1	q2	NOTEST	0	0	0	0	1	1	no

ENSG00000118246	ENSG00000118246	FASTKD2	2:207602486-207657233	q1	q2	OK	16.2724	17.1911	0.0792373	0.162313	0.80695	0.99995	no

ENSG00000118257	ENSG00000118257	NRP2	2:206546713-206662857	q1	q2	OK	50.1385	62.072	0.308025	0.426552	0.42695	0.99995	no

ENSG00000118260	ENSG00000118260	CREB1	2:208104373-208490652	q1	q2	OK	20.8777	23.3739	0.162934	0.215777	0.59645	0.99995	no

ENSG00000118263	ENSG00000118263	KLF7	2:207938860-208031991	q1	q2	OK	11.2206	11.7922	0.07168	0.119062	0.8044	0.99995	no

ENSG00000118271	ENSG00000118271	TTR	18:29171688-29178974	q1	q2	NOTEST	0.0313919	0	#NAME?	0	1	1	no

ENSG00000118276	ENSG00000118276	B4GALT6	18:29202209-29266786	q1	q2	OK	9.47817	10.4714	0.143769	0.261602	0.69845	0.99995	no

ENSG00000118292	ENSG00000118292	C1orf54	1:150237803-150253327	q1	q2	OK	0.526312	3.90413	2.89101	0.673956	0.286	0.99995	no

ENSG00000118298	ENSG00000118298	CA14	1:150227488-150237478	q1	q2	NOTEST	0.300355	0.0223974	-3.74526	0	1	1	no

ENSG00000118307	ENSG00000118307	CASC1	12:25261353-25348096	q1	q2	OK	0.689998	0.227142	-1.603	-0.7079	0.03545	0.99995	no

ENSG00000118308	ENSG00000118308	LRMP	12:25173935-25261268	q1	q2	NOTEST	0.149708	0.194637	0.378636	0	1	1	no

ENSG00000118322	ENSG00000118322	ATP10B	5:159990126-160279221	q1	q2	OK	0.910304	0.750772	-0.277974	-0.171251	0.59435	0.99995	no

ENSG00000118363	ENSG00000118363	SPCS2	11:74660291-74690076	q1	q2	OK	120.594	133.414	0.145751	0.254579	0.69825	0.99995	no

ENSG00000118369	ENSG00000118369	USP35	11:77850816-77925757	q1	q2	OK	5.23169	4.36439	-0.261497	-0.298649	0.63335	0.99995	no

ENSG00000118402	ENSG00000118402	ELOVL4	6:80624528-80657297	q1	q2	OK	8.25831	8.97899	0.120707	0.239763	0.733	0.99995	no

ENSG00000118407	ENSG00000118407	FILIP1	6:75994729-76203454	q1	q2	OK	1.27629	1.215	-0.0709976	-0.0875103	0.90025	0.99995	no

ENSG00000118412	ENSG00000118412	CASP8AP2	6:90539612-90584155	q1	q2	OK	15.3585	16.8155	0.130751	0.264722	0.66625	0.99995	no

ENSG00000118418	ENSG00000118418	HMGN3	6:79910961-79946514	q1	q2	OK	130.447	140.462	0.10671	0.254299	0.7192	0.99995	no

ENSG00000118420	ENSG00000118420	UBE3D	6:83602116-83775560	q1	q2	OK	5.11178	4.64323	-0.138697	-0.183804	0.7725	0.99995	no

ENSG00000118432	ENSG00000118432	CNR1	6:88849582-88876078	q1	q2	OK	6.47086	5.08814	-0.34682	-0.489267	0.32875	0.99995	no

ENSG00000118434	ENSG00000118434	SPACA1	6:88757506-88776550	q1	q2	NOTEST	0	0.1353	inf	0	1	1	no

ENSG00000118454	ENSG00000118454	ANKRD13C	1:70726270-70820417	q1	q2	OK	11.0458	13.6338	0.303697	0.536547	0.35005	0.99995	no

ENSG00000118473	ENSG00000118473	SGIP1	1:66999065-67213982	q1	q2	OK	0.715673	0.573284	-0.320051	-0.158572	0.6655	0.99995	no

ENSG00000118482	ENSG00000118482	PHF3	6:64345724-66417118	q1	q2	OK	19.4569	19.4796	0.0016854	0.00385583	0.99675	0.99995	no

ENSG00000118491	ENSG00000118491	ZC2HC1B	6:144185572-144259483	q1	q2	NOTEST	0	0.0639149	inf	0	1	1	no

ENSG00000118492	ENSG00000118492	ADGB	6:146920100-147136598	q1	q2	NOTEST	0.011758	0.0117562	-0.000221387	0	1	1	no

ENSG00000118495	ENSG00000118495	PLAGL1	6:144261436-144385735	q1	q2	OK	60.2417	59.4635	-0.0187569	-0.0346463	0.9511	0.99995	no

ENSG00000118496	ENSG00000118496	FBXO30	6:146114637-146135889	q1	q2	OK	7.39493	7.40041	0.00107029	0.00252794	0.9973	0.99995	no

ENSG00000118503	ENSG00000118503	TNFAIP3	6:138144809-138204449	q1	q2	OK	4.09579	4.75725	0.215986	0.177803	0.66305	0.99995	no

ENSG00000118507	ENSG00000118507	AKAP7	6:131456805-131604675	q1	q2	OK	4.62795	4.49829	-0.0409992	-0.0583557	0.92635	0.99995	no

ENSG00000118508	ENSG00000118508	RAB32	6:146864828-146876101	q1	q2	OK	6.67996	7.17286	0.102708	0.145949	0.83575	0.99995	no

ENSG00000118513	ENSG00000118513	MYB	6:135502452-135540311	q1	q2	OK	3.49897	3.91364	0.161584	0.236739	0.68775	0.99995	no

ENSG00000118514	ENSG00000118514	ALDH8A1	6:135238527-135271260	q1	q2	OK	1.5096	1.34313	-0.168566	-0.1661	0.7783	0.99995	no

ENSG00000118515	ENSG00000118515	SGK1	6:134490383-134639250	q1	q2	OK	2.10513	2.50534	0.251101	0.239526	0.5602	0.99995	no

ENSG00000118518	ENSG00000118518	RNF146	6:127587754-127609712	q1	q2	OK	59.1835	59.7041	0.0126337	0.0257257	0.966	0.99995	no

ENSG00000118520	ENSG00000118520	ARG1	6:131894283-131949369	q1	q2	NOTEST	0.087202	0.103403	0.245846	0	1	1	no

ENSG00000118523	ENSG00000118523	CTGF	6:132269315-132398533	q1	q2	OK	82.6345	84.0465	0.0244436	0.051503	0.9446	0.99995	no

ENSG00000118526	ENSG00000118526	TCF21	6:133561735-134216691	q1	q2	NOTEST	0.0109073	0	#NAME?	0	1	1	no

ENSG00000118557	ENSG00000118557	PMFBP1	16:72078187-72210777	q1	q2	OK	1.42027	0.826907	-0.780368	-0.172417	0.4989	0.99995	no

ENSG00000118564	ENSG00000118564	FBXL5	4:15606161-15739936	q1	q2	OK	38.9646	37.4426	-0.0574857	-0.0763242	0.89825	0.99995	no

ENSG00000118579	ENSG00000118579	MED28	4:17616253-17783135	q1	q2	OK	49.0822	53.8871	0.134739	0.0987515	0.85845	0.99995	no

ENSG00000118596	ENSG00000118596	SLC16A7	12:59989847-60176395	q1	q2	OK	0.734165	0.679558	-0.111506	-0.0645248	0.86315	0.99995	no

ENSG00000118600	ENSG00000118600	TMEM5	12:63952692-64217675	q1	q2	OK	10.0106	10.6657	0.0914516	0.119128	0.8447	0.99995	no

ENSG00000118620	ENSG00000118620	ZNF430	19:21203425-21242856	q1	q2	OK	4.76494	5.09663	0.0970831	0.17043	0.7919	0.99995	no

ENSG00000118640	ENSG00000118640	VAMP8	2:85788684-85809154	q1	q2	OK	0.560576	0.616618	0.137468	0.106374	0.8626	0.99995	no

ENSG00000118655	ENSG00000118655	DCLRE1B	1:114304453-114456708	q1	q2	OK	8.52774	8.53153	0.000640205	0.000790547	0.9993	0.99995	no

ENSG00000118680	ENSG00000118680	MYL12B	18:3261906-3278282	q1	q2	OK	122.977	125.626	0.0307496	0.074765	0.91805	0.99995	no

ENSG00000118689	ENSG00000118689	FOXO3	6:108881037-109005977	q1	q2	OK	4.13776	4.02164	-0.0410667	-0.082393	0.90845	0.99995	no

ENSG00000118690	ENSG00000118690	ARMC2	6:109169618-109295186	q1	q2	OK	3.56501	3.97169	0.155844	0.148535	0.84085	0.99995	no

ENSG00000118702	ENSG00000118702	GHRH	20:35879488-35890238	q1	q2	NOTEST	0	0	0	0	1	1	no

ENSG00000118705	ENSG00000118705	RPN2	20:35729628-35870022	q1	q2	OK	123.419	116.418	-0.0842503	-0.19037	0.7697	0.99995	no

ENSG00000118707	ENSG00000118707	TGIF2	20:34894257-35274619	q1	q2	OK	27.2154	26.964	-0.0133847	-0.0147741	0.98065	0.99995	no

ENSG00000118729	ENSG00000118729	CASQ2	1:116242627-116311402	q1	q2	NOTEST	0.0580697	0.0664203	0.193839	0	1	1	no

ENSG00000118733	ENSG00000118733	OLFM3	1:102268129-102462586	q1	q2	OK	0.758228	0.651926	-0.217923	-0.211677	0.7303	0.99995	no

ENSG00000118762	ENSG00000118762	PKD2	4:88928819-88998929	q1	q2	OK	28.918	26.8788	-0.105499	-0.197063	0.74805	0.99995	no

ENSG00000118777	ENSG00000118777	ABCG2	4:89011415-89152474	q1	q2	NOTEST	0.144003	0.110423	-0.38306	0	1	1	no

ENSG00000118785	ENSG00000118785	SPP1	4:88896818-88904562	q1	q2	OK	11.5386	10.6182	-0.119929	-0.187143	0.77565	0.99995	no

ENSG00000118804	ENSG00000118804	FAM47E-STBD1	4:77135192-77232752	q1	q2	OK	0.674268	0.549738	-0.294577	-0.253341	0.7254	0.99995	no

ENSG00000118816	ENSG00000118816	CCNI	4:77968310-77997158	q1	q2	OK	200.204	206.835	0.0470084	0.103027	0.87025	0.99995	no

ENSG00000118849	ENSG00000118849	RARRES1	3:158414680-158547508	q1	q2	NOTEST	0.176209	0.132503	-0.411262	0	1	1	no

ENSG00000118855	ENSG00000118855	MFSD1	3:158414680-158547508	q1	q2	OK	8.07118	9.31729	0.207129	0.354835	0.57345	0.99995	no

ENSG00000118873	ENSG00000118873	RAB3GAP2	1:220321634-220445796	q1	q2	OK	13.9765	14.0191	0.00438379	0.00429489	0.99155	0.99995	no

ENSG00000118894	ENSG00000118894	FAM86A	16:4932507-5147809	q1	q2	OK	6.5693	7.01709	0.0951349	0.0944151	0.8657	0.99995	no

ENSG00000118898	ENSG00000118898	PPL	16:4932507-5147809	q1	q2	NOTEST	0.240058	0.202274	-0.24707	0	1	1	no

ENSG00000118900	ENSG00000118900	UBN1	16:4853203-4932361	q1	q2	OK	18.0901	18.3349	0.0193941	0.0191862	0.9647	0.99995	no

ENSG00000118903	ENSG00000118903	BTF3P11	13:77502199-77502970	q1	q2	NOTEST	0	0	0	0	1	1	no

ENSG00000118922	ENSG00000118922	KLF12	13:74260225-74708394	q1	q2	OK	11.9405	12.3865	0.0529121	0.107265	0.8573	0.99995	no

ENSG00000118939	ENSG00000118939	UCHL3	13:76123618-76434004	q1	q2	OK	18.8522	19.7839	0.0695894	0.0989355	0.88635	0.99995	no

ENSG00000118946	ENSG00000118946	PCDH17	13:58205943-58303445	q1	q2	OK	5.30776	5.05969	-0.0690545	-0.142919	0.8381	0.99995	no

ENSG00000118960	ENSG00000118960	HS1BP3	2:20760207-20850849	q1	q2	OK	7.60958	7.0212	-0.116099	-0.189697	0.76915	0.99995	no

ENSG00000118961	ENSG00000118961	C2orf43	2:20883787-21022882	q1	q2	OK	29.2186	26.9484	-0.116689	-0.197376	0.7394	0.99995	no

ENSG00000118965	ENSG00000118965	WDR35	2:20110020-20189892	q1	q2	OK	9.93702	11.0016	0.146829	0.215539	0.6588	0.99995	no

ENSG00000118971	ENSG00000118971	CCND2	12:4357930-4414516	q1	q2	OK	23.8704	27.946	0.227417	0.492606	0.4113	0.99995	no

ENSG00000118972	ENSG00000118972	FGF23	12:4477392-4488894	q1	q2	NOTEST	0.0088769	0.00815663	-0.122082	0	1	1	no

ENSG00000118976	ENSG00000118976	HIN1L	12:3900212-4019504	q1	q2	NOTEST	0.043177	0.0664975	0.623039	0	1	1	no

ENSG00000118985	ENSG00000118985	ELL2	5:95220801-96143803	q1	q2	OK	3.86355	2.84601	-0.440987	-0.212664	0.6288	0.99995	no

ENSG00000118990	ENSG00000118990	GLRXP3	5:161178453-161178774	q1	q2	NOTEST	0	0	0	0	1	1	no

ENSG00000118997	ENSG00000118997	DNAH7	2:196602426-196933536	q1	q2	NOTEST	0.427905	0.208232	-1.03909	0	1	1	no

ENSG00000119004	ENSG00000119004	CYP20A1	2:204103662-204163009	q1	q2	OK	7.49441	8.39766	0.164171	0.247981	0.7041	0.99995	no

ENSG00000119013	ENSG00000119013	NDUFB3	2:201773695-201950473	q1	q2	OK	73.7171	75.8252	0.0406762	0.0530187	0.94105	0.99995	no

ENSG00000119041	ENSG00000119041	GTF3C3	2:197504277-197664449	q1	q2	OK	24.7168	29.7542	0.267601	0.329036	0.49785	0.99995	no

ENSG00000119042	ENSG00000119042	SATB2	2:200134222-200341658	q1	q2	OK	3.18143	3.42857	0.107931	0.175173	0.77465	0.99995	no

ENSG00000119048	ENSG00000119048	UBE2B	5:133706869-133727683	q1	q2	OK	58.6297	59.6839	0.02571	0.0458846	0.94535	0.99995	no

ENSG00000119121	ENSG00000119121	TRPM6	9:77337410-77503010	q1	q2	NOTEST	0.0408434	0.0332885	-0.295082	0	1	1	no

ENSG00000119125	ENSG00000119125	GDA	9:74729510-74872109	q1	q2	NOTEST	0.00461647	0.0337518	2.8701	0	1	1	no

ENSG00000119138	ENSG00000119138	KLF9	9:72999502-73029540	q1	q2	NOTEST	0.188462	0.108345	-0.798648	0	1	1	no

ENSG00000119139	ENSG00000119139	TJP2	9:71736208-71870124	q1	q2	OK	12.2715	13.6118	0.149543	0.251208	0.64855	0.99995	no

ENSG00000119147	ENSG00000119147	C2orf40	2:106679701-106694615	q1	q2	NOTEST	0.352597	0.339884	-0.0529771	0	1	1	no

ENSG00000119185	ENSG00000119185	ITGB1BP1	2:9346893-9563676	q1	q2	OK	51.9727	54.782	0.0759485	0.0846958	0.91125	0.99995	no

ENSG00000119203	ENSG00000119203	CPSF3	2:9563696-9613230	q1	q2	OK	51.5239	50.7052	-0.0231071	-0.0484386	0.93565	0.99995	no

ENSG00000119227	ENSG00000119227	PIGZ	3:196673213-196695931	q1	q2	NOTEST	0.46287	0.428974	-0.109716	0	1	1	no

ENSG00000119231	ENSG00000119231	SENP5	3:196594726-196661585	q1	q2	OK	18.1181	18.5143	0.031211	0.0444696	0.92815	0.99995	no

ENSG00000119242	ENSG00000119242	CCDC92	12:124247041-124800570	q1	q2	OK	24.6157	21.635	-0.186217	-0.100368	0.89675	0.99995	no

ENSG00000119280	ENSG00000119280	C1orf198	1:230972864-231005335	q1	q2	OK	38.6028	36.8439	-0.0672807	-0.164366	0.81275	0.99995	no

ENSG00000119283	ENSG00000119283	TRIM67	1:231297857-231357302	q1	q2	OK	1.1186	0.842357	-0.409186	-0.604524	0.35835	0.99995	no

ENSG00000119285	ENSG00000119285	HEATR1	1:236681299-236767804	q1	q2	OK	12.0469	13.8161	0.197689	0.311408	0.527	0.99995	no

ENSG00000119314	ENSG00000119314	PTBP3	9:114980714-115095947	q1	q2	OK	11.0259	12.1124	0.13558	0.311383	0.63925	0.99995	no

ENSG00000119318	ENSG00000119318	RAD23B	9:110045417-110094475	q1	q2	OK	114.989	118.206	0.0398118	0.083391	0.9046	0.99995	no

ENSG00000119321	ENSG00000119321	FKBP15	9:115913221-115983641	q1	q2	OK	8.19938	9.09337	0.1493	0.281123	0.66155	0.99995	no

ENSG00000119326	ENSG00000119326	CTNNAL1	9:111696460-111775809	q1	q2	OK	30.1174	33.7484	0.164219	0.329101	0.64435	0.99995	no

ENSG00000119328	ENSG00000119328	FAM206A	9:111696460-111775809	q1	q2	OK	12.8485	13.0223	0.0193813	0.0201165	0.9728	0.99995	no

ENSG00000119333	ENSG00000119333	WDR34	9:131314865-131419066	q1	q2	OK	50.997	44.9041	-0.183564	-0.140036	0.80265	0.99995	no

ENSG00000119335	ENSG00000119335	SET	9:131445702-131458679	q1	q2	OK	422.799	449.202	0.0873925	0.147718	0.81945	0.99995	no

ENSG00000119383	ENSG00000119383	PPP2R4	9:131857088-131911225	q1	q2	OK	80.7011	75.3823	-0.0983637	-0.189164	0.76145	0.99995	no

ENSG00000119392	ENSG00000119392	GLE1	9:131266978-131314689	q1	q2	OK	12.0207	12.8798	0.0995848	0.215347	0.75855	0.99995	no

ENSG00000119396	ENSG00000119396	RAB14	9:123940414-124095121	q1	q2	OK	39.8397	42.6799	0.0993506	0.166603	0.8081	0.99995	no

ENSG00000119397	ENSG00000119397	CNTRL	9:123837140-123939888	q1	q2	OK	10.057	11.1496	0.148788	0.275268	0.65725	0.99995	no

ENSG00000119401	ENSG00000119401	TRIM32	9:119187503-120177348	q1	q2	OK	9.04766	9.07079	0.00368286	0.00708	0.99255	0.99995	no

ENSG00000119402	ENSG00000119402	FBXW2	9:123514255-123555690	q1	q2	OK	19.8963	20.8124	0.0649398	0.0932167	0.88285	0.99995	no

ENSG00000119403	ENSG00000119403	PHF19	9:123617976-123639606	q1	q2	OK	24.0395	24.7582	0.0425011	0.0569034	0.923	0.99995	no

ENSG00000119408	ENSG00000119408	NEK6	9:127019884-127115586	q1	q2	OK	21.4324	18.1866	-0.236914	-0.498096	0.4571	0.99995	no

ENSG00000119411	ENSG00000119411	BSPRY	9:116111820-116133513	q1	q2	NOTEST	0.0124304	0	#NAME?	0	1	1	no

ENSG00000119414	ENSG00000119414	PPP6C	9:127908851-127952218	q1	q2	OK	32.3853	32.6864	0.0133495	0.0236164	0.97165	0.99995	no

ENSG00000119421	ENSG00000119421	NDUFA8	9:124894744-124922098	q1	q2	OK	53.9038	54.1534	0.00666489	0.0140466	0.9828	0.99995	no

ENSG00000119431	ENSG00000119431	HDHD3	9:116135698-116139279	q1	q2	OK	5.84914	5.13927	-0.186662	-0.272948	0.69815	0.99995	no

ENSG00000119440	ENSG00000119440	LCN1P1	9:136100291-136103993	q1	q2	NOTEST	0	0	0	0	1	1	no

ENSG00000119446	ENSG00000119446	RBM18	9:124999902-125085743	q1	q2	OK	8.8544	10.1338	0.194711	0.262607	0.622	0.99995	no

ENSG00000119457	ENSG00000119457	SLC46A2	9:115513117-115653193	q1	q2	NOTEST	0	0.0149469	inf	0	1	1	no

ENSG00000119471	ENSG00000119471	HSDL2	9:115142216-115431677	q1	q2	OK	18.1947	17.6982	-0.0399148	-0.0458411	0.9436	0.99995	no

ENSG00000119487	ENSG00000119487	MAPKAP1	9:128199671-128469513	q1	q2	OK	48.0082	45.0411	-0.0920376	-0.151776	0.8028	0.99995	no

ENSG00000119508	ENSG00000119508	NR4A3	9:102584136-102629173	q1	q2	OK	1.46031	1.53777	0.0745602	0.108263	0.8806	0.99995	no

ENSG00000119509	ENSG00000119509	INVS	9:102861537-103063282	q1	q2	OK	6.41067	6.6306	0.048664	0.0782097	0.89905	0.99995	no

ENSG00000119514	ENSG00000119514	GALNT12	9:101569980-101634697	q1	q2	OK	2.25554	2.15098	-0.0684782	-0.0730688	0.9089	0.99995	no

ENSG00000119522	ENSG00000119522	DENND1A	9:126118448-126692431	q1	q2	OK	11.1593	9.89912	-0.172876	-0.102221	0.81485	0.99995	no

ENSG00000119523	ENSG00000119523	ALG2	9:101978707-101984238	q1	q2	OK	19.1268	19.0522	-0.00563662	-0.0113241	0.98715	0.99995	no

ENSG00000119535	ENSG00000119535	CSF3R	1:36931643-36948879	q1	q2	NOTEST	0.00829661	0.0318614	1.94121	0	1	1	no

ENSG00000119537	ENSG00000119537	KDSR	18:60994958-61048862	q1	q2	OK	11.7322	11.6264	-0.013061	-0.0155244	0.97785	0.99995	no

ENSG00000119541	ENSG00000119541	VPS4B	18:61056422-61089716	q1	q2	OK	16.3894	17.2502	0.0738487	0.154839	0.8035	0.99995	no

ENSG00000119547	ENSG00000119547	ONECUT2	18:55102916-55158529	q1	q2	OK	0.958494	1.07651	0.167522	0.233295	0.7013	0.99995	no

ENSG00000119559	ENSG00000119559	C19orf25	19:1446299-1479555	q1	q2	OK	16.7614	18.5379	0.145338	0.0771474	0.84985	0.99995	no

ENSG00000119574	ENSG00000119574	ZBTB45	19:59024896-59050278	q1	q2	OK	9.11433	7.61409	-0.259466	-0.411272	0.50225	0.99995	no

ENSG00000119596	ENSG00000119596	YLPM1	14:75230068-75330537	q1	q2	OK	46.2568	48.3156	0.0628255	0.0869792	0.84385	0.99995	no

ENSG00000119599	ENSG00000119599	DCAF4	14:73393039-73426411	q1	q2	OK	3.6484	2.42742	-0.587838	-0.567933	0.2775	0.99995	no

ENSG00000119608	ENSG00000119608	PROX2	14:75230068-75330537	q1	q2	OK	0.612257	0.560868	-0.126477	-0.0277504	0.94695	0.99995	no

ENSG00000119614	ENSG00000119614	VSX2	14:74706174-74729441	q1	q2	OK	2.51226	2.70249	0.105302	0.154323	0.8302	0.99995	no

ENSG00000119616	ENSG00000119616	FCF1	14:75179846-75203394	q1	q2	OK	25.0183	28.4566	0.185778	0.318351	0.62725	0.99995	no

ENSG00000119630	ENSG00000119630	PGF	14:75408536-75422487	q1	q2	OK	4.98841	3.57476	-0.480734	-0.601191	0.391	0.99995	no

ENSG00000119632	ENSG00000119632	IFI27L2	14:94594115-94596590	q1	q2	OK	23.2954	16.3054	-0.514696	-0.724159	0.299	0.99995	no

ENSG00000119636	ENSG00000119636	CCDC176	14:74318546-74551196	q1	q2	OK	3.03967	3.26503	0.103179	0.0337966	0.92775	0.99995	no

ENSG00000119638	ENSG00000119638	NEK9	14:75548821-75643334	q1	q2	OK	28.3332	28.4356	0.00520509	0.00538344	0.9933	0.99995	no

ENSG00000119640	ENSG00000119640	ACYP1	14:75519923-75545126	q1	q2	OK	21.3198	23.3301	0.13	0.200339	0.77975	0.99995	no

ENSG00000119650	ENSG00000119650	IFT43	14:76041230-76550928	q1	q2	OK	28.2722	25.571	-0.144875	-0.116936	0.86145	0.99995	no

ENSG00000119655	ENSG00000119655	NPC2	14:74942894-74963809	q1	q2	OK	57.2115	57.0598	-0.00383064	-0.00758062	0.99125	0.99995	no

ENSG00000119660	ENSG00000119660	RP11-293M10.4	14:75758808-75761016	q1	q2	NOTEST	0	0	0	0	1	1	no

ENSG00000119661	ENSG00000119661	DNAL1	14:74111577-74170435	q1	q2	OK	6.44076	7.31699	0.184018	0.166051	0.67695	0.99995	no

ENSG00000119669	ENSG00000119669	IRF2BPL	14:77490887-77495034	q1	q2	OK	45.9011	43.9739	-0.0618807	-0.153404	0.82835	0.99995	no

ENSG00000119673	ENSG00000119673	ACOT2	14:74034323-74042357	q1	q2	OK	1.81283	1.67895	-0.110685	-0.107454	0.8723	0.99995	no

ENSG00000119681	ENSG00000119681	LTBP2	14:74964872-75079306	q1	q2	OK	0.833969	0.781957	-0.0929047	-0.0738589	0.8616	0.99995	no

ENSG00000119682	ENSG00000119682	AREL1	14:75120139-75179818	q1	q2	OK	19.8213	19.3887	-0.031838	-0.0546975	0.9063	0.99995	no

ENSG00000119684	ENSG00000119684	MLH3	14:75480466-75518235	q1	q2	OK	9.31226	10.036	0.107987	0.137946	0.7927	0.99995	no

ENSG00000119685	ENSG00000119685	TTLL5	14:76041230-76550928	q1	q2	OK	13.8397	15.7424	0.185846	0.135442	0.6925	0.99995	no

ENSG00000119686	ENSG00000119686	FLVCR2	14:76041230-76550928	q1	q2	OK	2.0702	1.62599	-0.348454	-0.14286	0.7363	0.99995	no

ENSG00000119688	ENSG00000119688	ABCD4	14:74752125-74769759	q1	q2	OK	56.0492	46.4749	-0.270241	-0.504882	0.41365	0.99995	no

ENSG00000119689	ENSG00000119689	DLST	14:75348593-75370448	q1	q2	OK	49.2845	48.8881	-0.0116494	-0.0241402	0.9653	0.99995	no

ENSG00000119698	ENSG00000119698	PPP4R4	14:94612464-94746072	q1	q2	OK	0.810555	0.607161	-0.41683	-0.331622	0.4937	0.99995	no

ENSG00000119699	ENSG00000119699	TGFB3	14:76041230-76550928	q1	q2	OK	5.63363	4.72224	-0.254592	-0.113207	0.8481	0.99995	no

ENSG00000119703	ENSG00000119703	ZC2HC1C	14:75519923-75545126	q1	q2	OK	0.508823	0.549289	0.110404	0.0351904	0.9087	0.99995	no

ENSG00000119705	ENSG00000119705	SLIRP	14:78174413-78236085	q1	q2	OK	126.487	136.866	0.113772	0.130346	0.8477	0.99995	no

ENSG00000119707	ENSG00000119707	RBM25	14:73525138-73588122	q1	q2	OK	79.1865	84.3355	0.0908853	0.187671	0.75725	0.99995	no

ENSG00000119711	ENSG00000119711	ALDH6A1	14:74318546-74551196	q1	q2	OK	9.34749	8.73057	-0.0985031	-0.0911935	0.8672	0.99995	no

ENSG00000119714	ENSG00000119714	GPR68	14:91698875-91720269	q1	q2	NOTEST	0.296501	0.164167	-0.85287	0	1	1	no

ENSG00000119715	ENSG00000119715	ESRRB	14:76776956-77227731	q1	q2	OK	0.491385	0.561997	0.193708	0.129827	0.81445	0.99995	no

ENSG00000119718	ENSG00000119718	EIF2B2	14:75469613-75476292	q1	q2	OK	27.1362	29.0221	0.0969336	0.207365	0.763	0.99995	no

ENSG00000119720	ENSG00000119720	NRDE2	14:90742579-90798481	q1	q2	OK	8.01974	7.51308	-0.0941507	-0.129967	0.82115	0.99995	no

ENSG00000119723	ENSG00000119723	COQ6	14:74318546-74551196	q1	q2	OK	13.8039	12.533	-0.139351	-0.104594	0.84245	0.99995	no

ENSG00000119725	ENSG00000119725	ZNF410	14:74318546-74551196	q1	q2	OK	31.7564	30.341	-0.06578	-0.0917874	0.8832	0.99995	no

ENSG00000119729	ENSG00000119729	RHOQ	2:46717888-46852881	q1	q2	OK	26.8835	27.3894	0.0268941	0.04196	0.94345	0.99995	no

ENSG00000119737	ENSG00000119737	GPR75	2:53759809-54087297	q1	q2	OK	3.25443	3.30564	0.0225259	0.0142168	0.98235	0.99995	no

ENSG00000119760	ENSG00000119760	SUPT7L	2:27805896-27917840	q1	q2	OK	26.8462	25.8735	-0.0532462	-0.0629342	0.92865	0.99995	no

ENSG00000119771	ENSG00000119771	KLHL29	2:23608087-23931481	q1	q2	OK	2.38954	2.40005	0.00633469	0.0102492	0.98885	0.99995	no

ENSG00000119772	ENSG00000119772	DNMT3A	2:25455844-25565459	q1	q2	OK	26.982	26.13	-0.0462888	-0.0839304	0.88235	0.99995	no

ENSG00000119777	ENSG00000119777	TMEM214	2:27255777-27264563	q1	q2	OK	23.8959	21.5345	-0.150114	-0.267161	0.64575	0.99995	no

ENSG00000119778	ENSG00000119778	ATAD2B	2:23971533-24149984	q1	q2	OK	12.0059	13.9188	0.213287	0.301735	0.50605	0.99995	no

ENSG00000119782	ENSG00000119782	FKBP1B	2:24232950-24286551	q1	q2	OK	14.8585	13.7991	-0.10672	-0.104373	0.88515	0.99995	no

ENSG00000119787	ENSG00000119787	ATL2	2:38522021-38604427	q1	q2	OK	29.5143	32.2956	0.129922	0.219576	0.71595	0.99995	no

ENSG00000119801	ENSG00000119801	YPEL5	2:30369806-30383399	q1	q2	OK	32.5788	30.5304	-0.0936865	-0.211159	0.76055	0.99995	no

ENSG00000119812	ENSG00000119812	FAM98A	2:33808724-33824449	q1	q2	OK	25.4497	25.4275	-0.00126079	-0.00116551	0.9983	0.99995	no

ENSG00000119820	ENSG00000119820	YIPF4	2:32502978-32541663	q1	q2	OK	20.1311	21.4992	0.0948632	0.080518	0.8751	0.99995	no

ENSG00000119844	ENSG00000119844	AFTPH	2:64751464-64820139	q1	q2	OK	9.84015	10.634	0.111938	0.208125	0.73445	0.99995	no

ENSG00000119862	ENSG00000119862	LGALSL	2:64681102-64688515	q1	q2	OK	14.6714	20.4074	0.476087	0.961658	0.1191	0.99995	no

ENSG00000119865	ENSG00000119865	CNRIP1	2:68511302-68547183	q1	q2	OK	12.1705	11.427	-0.0909413	-0.157728	0.82005	0.99995	no

ENSG00000119866	ENSG00000119866	BCL11A	2:60678301-60780702	q1	q2	OK	4.39506	4.123	-0.0921875	-0.074805	0.9053	0.99995	no

ENSG00000119878	ENSG00000119878	CRIPT	2:46717888-46852881	q1	q2	OK	9.20381	8.6819	-0.084221	-0.0741891	0.9137	0.99995	no

ENSG00000119888	ENSG00000119888	EPCAM	2:47572296-47614740	q1	q2	NOTEST	0.418171	0.281736	-0.56975	0	1	1	no

ENSG00000119899	ENSG00000119899	SLC17A5	6:74303101-74363878	q1	q2	OK	4.03125	4.31317	0.097522	0.164095	0.80925	0.99995	no

ENSG00000119900	ENSG00000119900	OGFRL1	6:71931159-72130472	q1	q2	OK	3.77921	5.06022	0.421116	0.461501	0.3401	0.99995	no

ENSG00000119906	ENSG00000119906	FAM178A	10:102670859-102724893	q1	q2	OK	22.9389	26.3568	0.200382	0.244356	0.56775	0.99995	no

ENSG00000119912	ENSG00000119912	IDE	10:94211440-94333833	q1	q2	OK	13.5717	13.7397	0.017754	0.0272147	0.95135	0.99995	no

ENSG00000119913	ENSG00000119913	TECTB	10:114043492-114064793	q1	q2	NOTEST	0.0387956	0.0379371	-0.0322827	0	1	1	no

ENSG00000119915	ENSG00000119915	ELOVL3	10:103986084-103989346	q1	q2	NOTEST	0.433403	0.243201	-0.833558	0	1	1	no

ENSG00000119917	ENSG00000119917	IFIT3	10:90973325-91174314	q1	q2	OK	1.70004	1.52182	-0.159768	-0.0906951	0.89755	0.99995	no

ENSG00000119919	ENSG00000119919	NKX2-3	10:101292689-101296278	q1	q2	NOTEST	0	0	0	0	1	1	no

ENSG00000119922	ENSG00000119922	IFIT2	10:90973325-91174314	q1	q2	OK	0.659466	0.528337	-0.31984	-0.142849	0.8641	0.99995	no

ENSG00000119927	ENSG00000119927	GPAM	10:113909623-113975135	q1	q2	OK	7.2128	7.50938	0.0581349	0.126665	0.85195	0.99995	no

ENSG00000119929	ENSG00000119929	CUTC	10:101419262-101515891	q1	q2	OK	13.348	13.1202	-0.0248361	-0.023234	0.97205	0.99995	no

ENSG00000119938	ENSG00000119938	PPP1R3C	10:93388198-93392811	q1	q2	NOTEST	0.165589	0.122358	-0.436496	0	1	1	no

ENSG00000119943	ENSG00000119943	PYROXD2	10:100143321-100174941	q1	q2	OK	1.02119	0.97651	-0.0645477	-0.0479072	0.93815	0.99995	no

ENSG00000119946	ENSG00000119946	CNNM1	10:101088855-101154087	q1	q2	NOTEST	0.337583	0.276985	-0.285436	0	1	1	no

ENSG00000119950	ENSG00000119950	MXI1	10:111967362-112047123	q1	q2	OK	17.6065	16.5451	-0.0897072	-0.184714	0.7821	0.99995	no

ENSG00000119953	ENSG00000119953	SMNDC1	10:112050487-112064709	q1	q2	OK	21.2117	22.7493	0.100958	0.167234	0.79355	0.99995	no

ENSG00000119965	ENSG00000119965	C10orf88	10:124690418-124768333	q1	q2	OK	6.68696	8.46306	0.339827	0.333285	0.5847	0.99995	no

ENSG00000119969	ENSG00000119969	HELLS	10:96305546-96373662	q1	q2	OK	53.6898	56.0142	0.0611421	0.112719	0.84805	0.99995	no

ENSG00000119973	ENSG00000119973	PRLHR	10:120352915-120355160	q1	q2	NOTEST	0	0	0	0	1	1	no

ENSG00000119977	ENSG00000119977	TCTN3	10:97423157-97453900	q1	q2	OK	22.4057	22.3517	-0.00347994	-0.00772029	0.9906	0.99995	no

ENSG00000119979	ENSG00000119979	FAM45A	10:120863597-120897496	q1	q2	OK	30.6561	28.4428	-0.108114	-0.199212	0.76815	0.99995	no

ENSG00000119986	ENSG00000119986	AVPI1	10:99437180-99447080	q1	q2	OK	0.796903	0.493479	-0.691417	-0.671171	0.3572	0.99995	no

ENSG00000120008	ENSG00000120008	WDR11	10:122521323-122669036	q1	q2	OK	29.7726	29.5153	-0.0125225	-0.020475	0.964	0.99995	no

ENSG00000120029	ENSG00000120029	C10orf76	10:103605355-103815950	q1	q2	OK	9.13125	8.73852	-0.0634231	-0.113803	0.8494	0.99995	no

ENSG00000120049	ENSG00000120049	KCNIP2	10:103578834-103603677	q1	q2	OK	3.61968	2.76545	-0.38835	-0.44615	0.4202	0.99995	no

ENSG00000120051	ENSG00000120051	CCDC147	10:106113521-106214848	q1	q2	OK	0.636407	0.926373	0.541645	0.531597	0.3707	0.99995	no

ENSG00000120053	ENSG00000120053	GOT1	10:101156626-101190381	q1	q2	OK	28.7647	28.9638	0.00995224	0.0203322	0.9756	0.99995	no

ENSG00000120054	ENSG00000120054	CPN1	10:101801949-101841634	q1	q2	NOTEST	0	0	0	0	1	1	no

ENSG00000120055	ENSG00000120055	C10orf95	10:104209573-104220863	q1	q2	NOTEST	0.11356	0.0282574	-2.00676	0	1	1	no

ENSG00000120057	ENSG00000120057	SFRP5	10:99526507-99531709	q1	q2	NOTEST	0.0145049	0.0291525	1.00708	0	1	1	no

ENSG00000120063	ENSG00000120063	GNA13	17:63006832-63052957	q1	q2	OK	24.6156	27.9709	0.184358	0.454879	0.5012	0.99995	no

ENSG00000120068	ENSG00000120068	HOXB8	17:46684593-46712294	q1	q2	NOTEST	0.0616843	0.306487	2.31285	0	1	1	no

ENSG00000120071	ENSG00000120071	KANSL1	17:44107281-44302733	q1	q2	OK	18.1195	14.1059	-0.361247	-0.501741	0.2968	0.99995	no

ENSG00000120075	ENSG00000120075	HOXB5	17:46618255-46683776	q1	q2	NOTEST	0.298584	0.357289	0.258954	0	1	1	no

ENSG00000120088	ENSG00000120088	CRHR1	17:43697693-43913194	q1	q2	NOTEST	0.114194	0.0244443	-2.22392	0	1	1	no

ENSG00000120093	ENSG00000120093	HOXB3	17:46618255-46683776	q1	q2	OK	17.5291	17.2444	-0.023626	-0.0275391	0.9624	0.99995	no

ENSG00000120094	ENSG00000120094	HOXB1	17:46605887-46608359	q1	q2	OK	0.392001	0.622535	0.667298	0.641368	0.35745	0.99995	no

ENSG00000120129	ENSG00000120129	DUSP1	5:172185228-172204777	q1	q2	OK	7.91284	8.45766	0.0960629	0.168881	0.80805	0.99995	no

ENSG00000120137	ENSG00000120137	PANK3	5:167975499-168006605	q1	q2	OK	39.1032	48.4679	0.309744	0.682516	0.29775	0.99995	no

ENSG00000120149	ENSG00000120149	MSX2	5:174151535-174157896	q1	q2	OK	0.574762	0.394654	-0.542379	-0.385711	0.5222	0.99995	no

ENSG00000120156	ENSG00000120156	TEK	9:27109138-27230173	q1	q2	OK	10.732	12.9289	0.268681	0.516858	0.45725	0.99995	no

ENSG00000120158	ENSG00000120158	RCL1	9:4792868-4885917	q1	q2	OK	5.49279	5.903	0.103908	0.158516	0.80235	0.99995	no

ENSG00000120159	ENSG00000120159	CAAP1	9:26840682-26892802	q1	q2	OK	16.8671	16.7675	-0.00854457	-0.0156601	0.97905	0.99995	no

ENSG00000120160	ENSG00000120160	EQTN	9:27284655-27297137	q1	q2	NOTEST	0.252529	0.209663	-0.268376	0	1	1	no

ENSG00000120162	ENSG00000120162	MOB3B	9:27325206-27529779	q1	q2	OK	14.021	15.4596	0.140913	0.335369	0.62305	0.99995	no

ENSG00000120210	ENSG00000120210	INSL6	9:5131978-5185668	q1	q2	NOTEST	0.139843	0	#NAME?	0	1	1	no

ENSG00000120211	ENSG00000120211	INSL4	9:5231418-5235304	q1	q2	NOTEST	0	0	0	0	1	1	no

ENSG00000120215	ENSG00000120215	MLANA	9:5881595-6007904	q1	q2	NOTEST	0.379876	0.193711	-0.971626	0	1	1	no

ENSG00000120217	ENSG00000120217	CD274	9:5450502-5470566	q1	q2	NOTEST	0.129571	0.154668	0.255433	0	1	1	no

ENSG00000120235	ENSG00000120235	IFNA6	9:21349833-21351377	q1	q2	NOTEST	0	0	0	0	1	1	no

ENSG00000120242	ENSG00000120242	IFNA8	9:21409145-21410184	q1	q2	NOTEST	0	0	0	0	1	1	no

ENSG00000120251	ENSG00000120251	GRIA2	4:158125333-158287227	q1	q2	OK	7.28986	7.06846	-0.0444945	-0.0446568	0.92925	0.99995	no

ENSG00000120253	ENSG00000120253	NUP43	6:150045450-150132556	q1	q2	OK	21.5564	22.8443	0.08372	0.130939	0.8496	0.99995	no

ENSG00000120254	ENSG00000120254	MTHFD1L	6:151186684-151549582	q1	q2	OK	23.159	27.5301	0.249436	0.339608	0.52715	0.99995	no

ENSG00000120256	ENSG00000120256	LRP11	6:150139933-150244257	q1	q2	OK	12.8133	12.9912	0.0198856	0.0320348	0.94875	0.99995	no

ENSG00000120262	ENSG00000120262	CCDC170	6:151815164-151942328	q1	q2	NOTEST	0.152198	0.219702	0.529599	0	1	1	no

ENSG00000120265	ENSG00000120265	PCMT1	6:150045450-150132556	q1	q2	OK	47.7697	49.7833	0.059568	0.101837	0.8861	0.99995	no

ENSG00000120278	ENSG00000120278	PLEKHG1	6:150920998-151164799	q1	q2	OK	8.51706	9.3805	0.139309	0.291599	0.6452	0.99995	no

ENSG00000120279	ENSG00000120279	MYCT1	6:153019029-153045702	q1	q2	NOTEST	0.01084	0	#NAME?	0	1	1	no

ENSG00000120280	ENSG00000120280	CXorf21	X:30576940-30595961	q1	q2	NOTEST	0	0	0	0	1	1	no

ENSG00000120289	ENSG00000120289	MAGEB4	X:30260056-30270155	q1	q2	NOTEST	0	0	0	0	1	1	no

ENSG00000120306	ENSG00000120306	CYSTM1	5:139554226-139682706	q1	q2	OK	10.8631	11.0034	0.0185235	0.0136998	0.9816	0.99995	no

ENSG00000120314	ENSG00000120314	WDR55	5:140044260-140086248	q1	q2	OK	11.6844	11.3017	-0.0480408	-0.0432814	0.95225	0.99995	no

ENSG00000120318	ENSG00000120318	ARAP3	5:141032967-141061788	q1	q2	OK	27.9595	33.1696	0.246523	0.548325	0.4271	0.99995	no

ENSG00000120322	ENSG00000120322	PCDHB8	5:140557370-140560081	q1	q2	NOTEST	0	0	0	0	1	1	no

ENSG00000120324	ENSG00000120324	PCDHB10	5:140571941-140575215	q1	q2	NOTEST	0.00771929	0	#NAME?	0	1	1	no

ENSG00000120327	ENSG00000120327	PCDHB14	5:140602930-140605858	q1	q2	NOTEST	0.0619289	0.0424677	-0.544248	0	1	1	no

ENSG00000120328	ENSG00000120328	PCDHB12	5:140588268-140591696	q1	q2	NOTEST	0	0	0	0	1	1	no

ENSG00000120329	ENSG00000120329	SLC25A2	5:140682195-140683612	q1	q2	NOTEST	0	0	0	0	1	1	no

ENSG00000120332	ENSG00000120332	TNN	1:175036993-175117202	q1	q2	OK	1.26239	1.1078	-0.18847	-0.264785	0.70435	0.99995	no

ENSG00000120333	ENSG00000120333	MRPS14	1:174968299-174992561	q1	q2	OK	21.9694	21.0286	-0.0631399	-0.068873	0.92135	0.99995	no

ENSG00000120334	ENSG00000120334	CENPL	1:173768687-173827684	q1	q2	OK	8.04374	8.91442	0.148273	0.163641	0.7981	0.99995	no

ENSG00000120337	ENSG00000120337	TNFSF18	1:172745044-173033155	q1	q2	NOTEST	0.0269454	0.0270712	0.00671892	0	1	1	no

ENSG00000120341	ENSG00000120341	SEC16B	1:177893090-178007142	q1	q2	OK	0.240698	0.615988	1.35568	0.487751	0.19875	0.99995	no

ENSG00000120370	ENSG00000120370	GORAB	1:170429593-170522587	q1	q2	OK	10.5589	10.5394	-0.00266363	-0.00434521	0.99385	0.99995	no

ENSG00000120436	ENSG00000120436	GPR31	6:167559901-167610401	q1	q2	NOTEST	0	0	0	0	1	1	no

ENSG00000120437	ENSG00000120437	ACAT2	6:160090088-160210781	q1	q2	OK	145.136	183.601	0.339176	0.366165	0.60355	0.99995	no

ENSG00000120438	ENSG00000120438	TCP1	6:160090088-160210781	q1	q2	OK	158.226	169.553	0.0997551	0.111696	0.8473	0.99995	no

ENSG00000120440	ENSG00000120440	TTLL2	6:167738573-167797998	q1	q2	NOTEST	0.196741	0.218546	0.151639	0	1	1	no

ENSG00000120451	ENSG00000120451	SNX19	11:130745330-130786404	q1	q2	OK	20.6491	21.1137	0.0321037	0.0479109	0.92365	0.99995	no

ENSG00000120457	ENSG00000120457	KCNJ5	11:128761250-128790930	q1	q2	NOTEST	0.179409	0.252442	0.492699	0	1	1	no

ENSG00000120458	ENSG00000120458	MSANTD2	11:124636393-124704383	q1	q2	OK	22.2181	22.737	0.033308	0.0677057	0.92215	0.99995	no

ENSG00000120471	ENSG00000120471	TP53AIP1	11:128804625-128813040	q1	q2	NOTEST	0.202936	0.239422	0.238526	0	1	1	no

ENSG00000120498	ENSG00000120498	TEX11	X:69748789-70128581	q1	q2	NOTEST	0	0.024373	inf	0	1	1	no

ENSG00000120500	ENSG00000120500	ARR3	X:69488154-69501690	q1	q2	NOTEST	0.0719942	0.204167	1.5038	0	1	1	no

ENSG00000120509	ENSG00000120509	PDZD11	X:69506444-69640682	q1	q2	OK	30.9672	33.5233	0.114422	0.0988276	0.88065	0.99995	no

ENSG00000120519	ENSG00000120519	SLC10A7	4:147175126-147443123	q1	q2	OK	3.24704	3.30506	0.0255482	0.0365005	0.95515	0.99995	no

ENSG00000120526	ENSG00000120526	NUDCD1	8:110253147-110358182	q1	q2	OK	15.2874	18.1666	0.248947	0.266732	0.70095	0.99995	no

ENSG00000120533	ENSG00000120533	ENY2	8:110253147-110358182	q1	q2	OK	153.731	174.535	0.183109	0.293143	0.6715	0.99995	no

ENSG00000120539	ENSG00000120539	MASTL	10:27399382-27475853	q1	q2	OK	23.1771	26.2282	0.178422	0.18946	0.79705	0.99995	no

ENSG00000120549	ENSG00000120549	KIAA1217	10:23983674-24836772	q1	q2	OK	1.69306	1.73222	0.0329924	0.0373084	0.9454	0.99995	no

ENSG00000120555	ENSG00000120555	SEPT7P9	10:38671996-38691844	q1	q2	NOTEST	0.149361	0.164061	0.135436	0	1	1	no

ENSG00000120563	ENSG00000120563	LYZL1	10:29577989-29607257	q1	q2	NOTEST	0	0	0	0	1	1	no

ENSG00000120586	ENSG00000120586	MRC1	10:18098351-18200091	q1	q2	NOTEST	0	0.0046176	inf	0	1	1	no

ENSG00000120594	ENSG00000120594	PLXDC2	10:20105167-20578785	q1	q2	OK	5.31565	3.91356	-0.441765	-0.505021	0.44835	0.99995	no

ENSG00000120616	ENSG00000120616	EPC1	10:32555216-32667726	q1	q2	OK	22.1372	22.4463	0.0200028	0.0309411	0.94955	0.99995	no

ENSG00000120645	ENSG00000120645	IQSEC3	12:175930-291565	q1	q2	NOTEST	0.157206	0.0919073	-0.774408	0	1	1	no

ENSG00000120647	ENSG00000120647	CCDC77	12:389294-551811	q1	q2	OK	22.5717	19.7717	-0.191072	-0.173894	0.71945	0.99995	no

ENSG00000120656	ENSG00000120656	TAF12	1:28915834-28969597	q1	q2	OK	14.6643	14.9789	0.0306152	0.0536088	0.93675	0.99995	no

ENSG00000120658	ENSG00000120658	ENOX1	13:43787653-44361044	q1	q2	OK	3.91795	3.45189	-0.182712	-0.219283	0.70155	0.99995	no

ENSG00000120659	ENSG00000120659	TNFSF11	13:43136871-43182149	q1	q2	NOTEST	0.0122601	0	#NAME?	0	1	1	no

ENSG00000120662	ENSG00000120662	MTRF1	13:41790504-41837742	q1	q2	OK	9.66012	9.58726	-0.0109227	-0.0148083	0.9823	0.99995	no

ENSG00000120664	ENSG00000120664	SPG20OS	13:36875774-36944317	q1	q2	OK	1.56341	0.803415	-0.960482	-0.137529	0.4438	0.99995	no

ENSG00000120669	ENSG00000120669	SOHLH2	13:36742344-36871979	q1	q2	NOTEST	0.299248	0.468312	0.64613	0	1	1	no

ENSG00000120675	ENSG00000120675	DNAJC15	13:43597338-43683045	q1	q2	OK	1.48722	1.92947	0.375588	0.472359	0.49385	0.99995	no

ENSG00000120685	ENSG00000120685	PROSER1	13:39584002-39612252	q1	q2	OK	31.7173	36.4664	0.201299	0.289462	0.4849	0.99995	no

ENSG00000120686	ENSG00000120686	UFM1	13:38923985-38937140	q1	q2	OK	35.3474	39.9124	0.175233	0.362964	0.56055	0.99995	no

ENSG00000120688	ENSG00000120688	WBP4	13:41506055-41658137	q1	q2	OK	17.1304	17.9473	0.0672073	0.114216	0.86745	0.99995	no

ENSG00000120690	ENSG00000120690	ELF1	13:41506055-41658137	q1	q2	OK	8.2986	8.56	0.0447425	0.0501753	0.9386	0.99995	no

ENSG00000120693	ENSG00000120693	SMAD9	13:37418967-37494902	q1	q2	OK	2.54488	2.69807	0.0843308	0.144707	0.83195	0.99995	no

ENSG00000120694	ENSG00000120694	HSPH1	13:31710761-31736525	q1	q2	OK	65.6598	68.9354	0.0702337	0.129275	0.84035	0.99995	no

ENSG00000120696	ENSG00000120696	KBTBD7	13:41763968-41768702	q1	q2	OK	6.54772	6.88491	0.0724459	0.150552	0.83105	0.99995	no

ENSG00000120697	ENSG00000120697	ALG5	13:37523911-37633850	q1	q2	OK	22.5003	22.5755	0.00481371	0.00355158	0.9954	0.99995	no

ENSG00000120699	ENSG00000120699	EXOSC8	13:37523911-37633850	q1	q2	OK	71.2472	78.9409	0.147939	0.177022	0.74695	0.99995	no

ENSG00000120705	ENSG00000120705	ETF1	5:137841783-137878989	q1	q2	OK	45.2523	52.6273	0.217822	0.448807	0.424	0.99995	no

ENSG00000120708	ENSG00000120708	TGFBI	5:135364583-135399507	q1	q2	OK	52.1516	49.2943	-0.0812907	-0.119713	0.85015	0.99995	no

ENSG00000120709	ENSG00000120709	FAM53C	5:137620953-137685416	q1	q2	OK	31.9213	31.2156	-0.0322554	-0.0664309	0.92005	0.99995	no

ENSG00000120725	ENSG00000120725	SIL1	5:138282408-138667360	q1	q2	OK	11.621	11.8364	0.0264917	0.0041845	0.984	0.99995	no

ENSG00000120727	ENSG00000120727	PAIP2	5:138677275-138720242	q1	q2	OK	134.339	135.916	0.0168331	0.040714	0.9528	0.99995	no

ENSG00000120729	ENSG00000120729	MYOT	5:137150021-137387650	q1	q2	NOTEST	0.0662888	0	#NAME?	0	1	1	no

ENSG00000120733	ENSG00000120733	KDM3B	5:137688284-137772717	q1	q2	OK	55.596	57.286	0.0432028	0.0674848	0.88635	0.99995	no

ENSG00000120738	ENSG00000120738	EGR1	5:137801178-137805004	q1	q2	OK	9.4513	13.0144	0.461521	0.900578	0.2026	0.99995	no

ENSG00000120742	ENSG00000120742	SERP1	3:150259780-150348222	q1	q2	OK	53.5084	54.1279	0.0166069	0.0262994	0.96775	0.99995	no

ENSG00000120756	ENSG00000120756	PLS1	3:142315228-142432506	q1	q2	OK	2.31321	1.8971	-0.286105	-0.304963	0.52015	0.99995	no

ENSG00000120784	ENSG00000120784	ZFP30	19:37988062-38210691	q1	q2	OK	12.6777	14.7964	0.222955	0.222067	0.603	0.99995	no

ENSG00000120798	ENSG00000120798	NR2C1	12:95414004-95467479	q1	q2	OK	32.9439	31.4442	-0.0672155	-0.0989351	0.87875	0.99995	no

ENSG00000120800	ENSG00000120800	UTP20	12:101673886-101780394	q1	q2	OK	6.58173	7.86349	0.256702	0.568716	0.38915	0.99995	no

ENSG00000120802	ENSG00000120802	TMPO	12:98906750-98944157	q1	q2	OK	176.141	198.42	0.17183	0.305897	0.5908	0.99995	no

ENSG00000120805	ENSG00000120805	ARL1	12:101786897-101801598	q1	q2	OK	27.55	30.5108	0.147267	0.281826	0.64805	0.99995	no

ENSG00000120820	ENSG00000120820	GLT8D2	12:104382761-104457961	q1	q2	OK	5.73516	5.81073	0.0188856	0.0169134	0.9833	0.99995	no

ENSG00000120832	ENSG00000120832	MTERFD3	12:107349496-107380944	q1	q2	OK	13.5698	12.7441	-0.0905666	-0.0747655	0.89185	0.99995	no

ENSG00000120833	ENSG00000120833	SOCS2	12:93936238-93977263	q1	q2	OK	36.7065	35.9618	-0.0295735	-0.0572307	0.9245	0.99995	no

ENSG00000120837	ENSG00000120837	NFYB	12:104510854-104532067	q1	q2	OK	30.8678	31.341	0.021947	0.0384213	0.9476	0.99995	no

ENSG00000120860	ENSG00000120860	CCDC53	12:102406704-102455927	q1	q2	OK	13.6539	12.8183	-0.0911057	-0.135734	0.84885	0.99995	no

ENSG00000120868	ENSG00000120868	APAF1	12:99038918-100378432	q1	q2	OK	17.3838	19.5682	0.170769	0.264982	0.6715	0.99995	no

ENSG00000120875	ENSG00000120875	DUSP4	8:29190580-29208185	q1	q2	OK	38.3162	47.4859	0.309546	0.761019	0.26895	0.99995	no

ENSG00000120885	ENSG00000120885	CLU	8:27454433-27472548	q1	q2	OK	33.9905	28.1808	-0.27042	-0.371886	0.54525	0.99995	no

ENSG00000120889	ENSG00000120889	TNFRSF10B	8:22545171-22941132	q1	q2	OK	64.7931	60.868	-0.0901569	-0.150239	0.80175	0.99995	no

ENSG00000120896	ENSG00000120896	SORBS3	8:22298331-22433301	q1	q2	OK	37.8418	34.9287	-0.115571	-0.185457	0.77445	0.99995	no

ENSG00000120899	ENSG00000120899	PTK2B	8:27168998-27316903	q1	q2	OK	3.58521	2.7082	-0.404722	-0.348197	0.3748	0.99995	no

ENSG00000120903	ENSG00000120903	CHRNA2	8:27317278-27337400	q1	q2	NOTEST	0.0334494	0.0369604	0.144002	0	1	1	no

ENSG00000120907	ENSG00000120907	ADRA1A	8:26605666-26724790	q1	q2	NOTEST	0.131611	0.0392942	-1.74389	0	1	1	no

ENSG00000120910	ENSG00000120910	PPP3CC	8:22298331-22433301	q1	q2	OK	5.37136	4.88657	-0.136464	-0.111651	0.85775	0.99995	no

ENSG00000120913	ENSG00000120913	PDLIM2	8:22435791-22461663	q1	q2	OK	32.9977	32.7024	-0.0129723	-0.0169696	0.9806	0.99995	no

ENSG00000120915	ENSG00000120915	EPHX2	8:27348295-27403081	q1	q2	OK	4.10925	3.86521	-0.0883308	-0.0947831	0.86985	0.99995	no

ENSG00000120925	ENSG00000120925	RNF170	8:42704779-42940931	q1	q2	OK	9.71435	10.347	0.091025	0.0582587	0.9147	0.99995	no

ENSG00000120937	ENSG00000120937	NPPB	1:11917520-11918988	q1	q2	NOTEST	0	0	0	0	1	1	no

ENSG00000120942	ENSG00000120942	UBIAD1	1:11333262-11356106	q1	q2	OK	8.36233	8.4431	0.0138682	0.024406	0.96805	0.99995	no

ENSG00000120948	ENSG00000120948	TARDBP	1:11072413-11085796	q1	q2	OK	161.064	170.915	0.0856436	0.162053	0.7798	0.99995	no

ENSG00000120949	ENSG00000120949	TNFRSF8	1:12123433-12204264	q1	q2	NOTEST	0.0699352	0.0128445	-2.44487	0	1	1	no

ENSG00000120952	ENSG00000120952	PRAMEF2	1:12916940-12921764	q1	q2	NOTEST	0	0	0	0	1	1	no

ENSG00000120963	ENSG00000120963	ZNF706	8:102190105-102218421	q1	q2	OK	64.7678	65.6886	0.0203655	0.0355596	0.9585	0.99995	no

ENSG00000120992	ENSG00000120992	LYPLA1	8:54958937-55014577	q1	q2	OK	45.3316	47.5733	0.0696348	0.131177	0.7999	0.99995	no

ENSG00000121005	ENSG00000121005	CRISPLD1	8:75896749-75946793	q1	q2	OK	49.7641	56.6107	0.185971	0.404807	0.5453	0.99995	no

ENSG00000121022	ENSG00000121022	COPS5	8:67876333-68255912	q1	q2	OK	46.8142	46.7341	-0.00247134	-0.00325801	0.99615	0.99995	no

ENSG00000121039	ENSG00000121039	RDH10	8:74202505-74268696	q1	q2	OK	14.8539	16.0715	0.113659	0.032797	0.9236	0.99995	no

ENSG00000121053	ENSG00000121053	EPX	17:56270097-56282535	q1	q2	NOTEST	0.0672573	0.0478508	-0.491148	0	1	1	no

ENSG00000121057	ENSG00000121057	AKAP1	17:55162452-55198710	q1	q2	OK	12.147	10.782	-0.171984	-0.342003	0.5656	0.99995	no

ENSG00000121058	ENSG00000121058	COIL	17:55015547-55038415	q1	q2	OK	17.4567	17.9371	0.039169	0.0871975	0.89825	0.99995	no

ENSG00000121060	ENSG00000121060	TRIM25	17:54965269-54991399	q1	q2	OK	5.18208	5.25954	0.0214032	0.0227264	0.9542	0.99995	no

ENSG00000121064	ENSG00000121064	SCPEP1	17:55055465-55084129	q1	q2	OK	26.7072	23.5217	-0.183234	-0.33353	0.59755	0.99995	no

ENSG00000121067	ENSG00000121067	SPOP	17:47676245-47755596	q1	q2	OK	34.8269	34.6359	-0.00793176	-0.0147437	0.97825	0.99995	no

ENSG00000121068	ENSG00000121068	TBX2	17:58754813-59488916	q1	q2	OK	10.5781	8.76495	-0.271266	-0.446902	0.5202	0.99995	no

ENSG00000121073	ENSG00000121073	SLC35B1	17:47778304-47906458	q1	q2	OK	48.7659	46.0554	-0.082503	-0.0785564	0.8858	0.99995	no

ENSG00000121075	ENSG00000121075	TBX4	17:59529764-59562471	q1	q2	NOTEST	0	0.0738837	inf	0	1	1	no

ENSG00000121089	ENSG00000121089	NACA3P	4:165864441-165865089	q1	q2	OK	2.46379	5.13951	1.06075	1.1368	0.11215	0.99995	no

ENSG00000121101	ENSG00000121101	TEX14	17:56597197-56769416	q1	q2	NOTEST	0.252454	0.203365	-0.311947	0	1	1	no

ENSG00000121104	ENSG00000121104	FAM117A	17:47778304-47906458	q1	q2	OK	6.4561	5.61984	-0.200135	-0.109678	0.85795	0.99995	no

ENSG00000121152	ENSG00000121152	NCAPH	2:97001524-97039583	q1	q2	OK	34.5282	33.5348	-0.0421151	-0.0788268	0.9072	0.99995	no

ENSG00000121207	ENSG00000121207	LRAT	4:155548096-155674270	q1	q2	NOTEST	0.2362	0.271146	0.199062	0	1	1	no

ENSG00000121210	ENSG00000121210	KIAA0922	4:154387497-154557863	q1	q2	OK	13.9809	14.3574	0.0383394	0.0713007	0.9007	0.99995	no

ENSG00000121211	ENSG00000121211	MND1	4:154265800-154336270	q1	q2	OK	18.5532	19.4427	0.0675603	0.114615	0.868	0.99995	no

ENSG00000121236	ENSG00000121236	TRIM6	11:5274419-5667019	q1	q2	OK	0.820383	0.926722	0.175839	0.13879	0.79735	0.99995	no

ENSG00000121270	ENSG00000121270	ABCC11	16:48198862-48482313	q1	q2	NOTEST	0.0785501	0.118031	0.587485	0	1	1	no

ENSG00000121274	ENSG00000121274	PAPD5	16:50186828-50269221	q1	q2	OK	5.19778	5.94122	0.192865	0.365976	0.59945	0.99995	no

ENSG00000121281	ENSG00000121281	ADCY7	16:50280047-50405391	q1	q2	OK	1.56937	1.11613	-0.49168	-0.122217	0.6254	0.99995	no

ENSG00000121289	ENSG00000121289	CEP89	19:33369901-33462897	q1	q2	OK	16.7043	18.0854	0.114605	0.250409	0.71465	0.99995	no

ENSG00000121297	ENSG00000121297	TSHZ3	19:31765850-31908700	q1	q2	OK	1.92492	1.89427	-0.023163	-0.0336089	0.9607	0.99995	no

ENSG00000121310	ENSG00000121310	ECHDC2	1:53361655-53392884	q1	q2	OK	1.56905	1.87825	0.259499	0.214782	0.6626	0.99995	no

ENSG00000121314	ENSG00000121314	TAS2R8	12:10958649-10959892	q1	q2	NOTEST	0	0	0	0	1	1	no

ENSG00000121316	ENSG00000121316	PLBD1	12:14656594-14910897	q1	q2	NOTEST	0.361103	0.363499	0.00954354	0	1	1	no

ENSG00000121318	ENSG00000121318	TAS2R10	12:10977558-11324212	q1	q2	NOTEST	0	0	0	0	1	1	no

ENSG00000121335	ENSG00000121335	PRB2	12:11504756-11653975	q1	q2	NOTEST	0	0.114293	inf	0	1	1	no

ENSG00000121350	ENSG00000121350	PYROXD1	12:21590548-21654603	q1	q2	OK	3.99218	3.87132	-0.0443498	-0.0239987	0.95275	0.99995	no

ENSG00000121351	ENSG00000121351	IAPP	12:21417533-21572528	q1	q2	NOTEST	0	0	0	0	1	1	no

ENSG00000121361	ENSG00000121361	KCNJ8	12:21788275-21928515	q1	q2	OK	4.56261	4.28923	-0.0891407	-0.0188001	0.9654	0.99995	no

ENSG00000121377	ENSG00000121377	TAS2R7	12:10954130-10955226	q1	q2	NOTEST	0	0	0	0	1	1	no

ENSG00000121380	ENSG00000121380	BCL2L14	12:12202777-12419946	q1	q2	OK	1.08521	0.777564	-0.480948	-0.100546	0.69135	0.99995	no

ENSG00000121381	ENSG00000121381	TAS2R9	12:10961692-10962767	q1	q2	NOTEST	0	0	0	0	1	1	no

ENSG00000121388	ENSG00000121388	RP11-408E5.4	13:19756172-19761496	q1	q2	NOTEST	0	0	0	0	1	1	no

ENSG00000121390	ENSG00000121390	PSPC1	13:20248763-20357142	q1	q2	OK	56.6817	59.3797	0.0670888	0.14169	0.82345	0.99995	no

ENSG00000121406	ENSG00000121406	ZNF549	19:58038692-58071231	q1	q2	OK	7.21558	7.8482	0.121247	0.207789	0.76025	0.99995	no

ENSG00000121410	ENSG00000121410	A1BG	19:58856543-58877958	q1	q2	OK	11.8929	9.80781	-0.278097	-0.185718	0.80965	0.99995	no

ENSG00000121413	ENSG00000121413	ZSCAN18	19:58570606-58629794	q1	q2	OK	27.7008	25.3088	-0.130284	-0.240847	0.7111	0.99995	no

ENSG00000121417	ENSG00000121417	ZNF211	19:58141760-58154147	q1	q2	OK	16.8904	16.1513	-0.0645524	-0.108006	0.8595	0.99995	no

ENSG00000121440	ENSG00000121440	PDZRN3	3:73431583-73675149	q1	q2	OK	30.0755	30.0287	-0.00224612	-0.00472927	0.9937	0.99995	no

ENSG00000121446	ENSG00000121446	RGSL1	1:182419255-182529734	q1	q2	NOTEST	0	0	0	0	1	1	no

ENSG00000121454	ENSG00000121454	LHX4	1:180199420-180472089	q1	q2	OK	3.34504	3.81895	0.191152	0.196633	0.748	0.99995	no

ENSG00000121481	ENSG00000121481	RNF2	1:185014495-185071740	q1	q2	OK	24.8191	31.0347	0.322435	0.57923	0.4043	0.99995	no

ENSG00000121486	ENSG00000121486	TRMT1L	1:185087219-185126204	q1	q2	OK	9.00736	9.73321	0.111812	0.202911	0.7236	0.99995	no

ENSG00000121542	ENSG00000121542	SEC22A	3:122785908-122992977	q1	q2	OK	14.2737	14.6788	0.0403686	0.0384928	0.9578	0.99995	no

ENSG00000121552	ENSG00000121552	CSTA	3:122044090-122060819	q1	q2	NOTEST	0.131683	0.129089	-0.0287076	0	1	1	no

ENSG00000121570	ENSG00000121570	DPPA4	3:109044987-109056419	q1	q2	OK	68.4495	78.9717	0.206296	0.410927	0.4859	0.99995	no

ENSG00000121577	ENSG00000121577	POPDC2	3:119355303-119396301	q1	q2	OK	1.91603	0.999153	-0.939342	-0.439906	0.39665	0.99995	no

ENSG00000121578	ENSG00000121578	B4GALT4	3:118930578-119009513	q1	q2	OK	7.22517	7.99744	0.146506	0.236287	0.68925	0.99995	no

ENSG00000121579	ENSG00000121579	NAA50	3:113435306-113465147	q1	q2	OK	54.2309	60.6452	0.161279	0.267256	0.7021	0.99995	no

ENSG00000121594	ENSG00000121594	CD80	3:119217378-119278449	q1	q2	NOTEST	0	0.0596542	inf	0	1	1	no

ENSG00000121621	ENSG00000121621	KIF18A	11:28042166-28355054	q1	q2	OK	13.3425	13.2125	-0.0141309	-0.0237423	0.9695	0.99995	no

ENSG00000121634	ENSG00000121634	GJA8	1:147374945-147381393	q1	q2	NOTEST	0	0	0	0	1	1	no

ENSG00000121644	ENSG00000121644	DESI2	1:244816236-244872335	q1	q2	OK	27.3666	28.3506	0.0509652	0.115528	0.8601	0.99995	no

ENSG00000121653	ENSG00000121653	MAPK8IP1	11:45907201-45928016	q1	q2	OK	11.0853	9.2394	-0.262773	-0.519194	0.44725	0.99995	no

ENSG00000121671	ENSG00000121671	CRY2	11:45868668-45904798	q1	q2	OK	7.43178	6.90476	-0.106116	-0.14413	0.76985	0.99995	no

ENSG00000121680	ENSG00000121680	PEX16	11:45931219-45940363	q1	q2	OK	7.71693	6.32838	-0.286192	-0.366403	0.53865	0.99995	no

ENSG00000121690	ENSG00000121690	DEPDC7	11:33037409-33055128	q1	q2	OK	2.93271	4.42295	0.592774	0.768783	0.2923	0.99995	no

ENSG00000121691	ENSG00000121691	CAT	11:34460471-34493609	q1	q2	OK	9.71383	11.9177	0.294993	0.397582	0.4651	0.99995	no

ENSG00000121716	ENSG00000121716	PILRB	7:99905324-99997719	q1	q2	OK	23.359	20.0401	-0.221089	-0.335672	0.63945	0.99995	no

ENSG00000121741	ENSG00000121741	ZMYM2	13:20532809-20665968	q1	q2	OK	52.4061	57.4178	0.131764	0.217647	0.6786	0.99995	no

ENSG00000121742	ENSG00000121742	GJB6	13:20796109-20806534	q1	q2	NOTEST	0	0	0	0	1	1	no

ENSG00000121743	ENSG00000121743	GJA3	13:20712393-20735188	q1	q2	NOTEST	0.0289222	0.0235966	-0.293598	0	1	1	no

ENSG00000121749	ENSG00000121749	TBC1D15	12:72233486-72320629	q1	q2	OK	19.2659	20.6902	0.102903	0.200321	0.7494	0.99995	no

ENSG00000121753	ENSG00000121753	BAI2	1:32192717-32230494	q1	q2	OK	25.6553	21.2042	-0.274905	-0.454607	0.48915	0.99995	no

ENSG00000121764	ENSG00000121764	HCRTR1	1:32083286-32169920	q1	q2	NOTEST	0.14758	0.0785283	-0.910218	0	1	1	no

ENSG00000121766	ENSG00000121766	ZCCHC17	1:31769841-31837783	q1	q2	OK	36.6283	35.067	-0.0628474	-0.127016	0.84785	0.99995	no

ENSG00000121769	ENSG00000121769	FABP3	1:31838471-31849697	q1	q2	OK	13.5896	17.5738	0.370923	0.534989	0.44645	0.99995	no

ENSG00000121774	ENSG00000121774	KHDRBS1	1:32479429-32568467	q1	q2	OK	175.903	183.595	0.0617466	0.140448	0.84255	0.99995	no

ENSG00000121775	ENSG00000121775	TMEM39B	1:32479429-32568467	q1	q2	OK	19.6032	17.3563	-0.175626	-0.097249	0.88215	0.99995	no

ENSG00000121797	ENSG00000121797	CCRL2	3:46448653-46454488	q1	q2	NOTEST	0	0.0165507	inf	0	1	1	no

ENSG00000121807	ENSG00000121807	CCR2	3:46395224-46402419	q1	q2	NOTEST	0	0	0	0	1	1	no

ENSG00000121848	ENSG00000121848	RNF115	1:145611035-145715614	q1	q2	NOTEST	0.368679	0.478613	0.376495	0	1	1	no

ENSG00000121851	ENSG00000121851	POLR3GL	1:145456235-145470388	q1	q2	OK	0.636662	0.608048	-0.0663442	-0.0569199	0.92635	0.99995	no

ENSG00000121853	ENSG00000121853	GHSR	3:172162922-172166246	q1	q2	NOTEST	0.105626	0	#NAME?	0	1	1	no

ENSG00000121858	ENSG00000121858	TNFSF10	3:172223297-172241297	q1	q2	NOTEST	0.188544	0.149601	-0.333789	0	1	1	no

ENSG00000121864	ENSG00000121864	ZNF639	3:179040778-179053323	q1	q2	OK	26.1227	28.7152	0.136512	0.273874	0.66155	0.99995	no

ENSG00000121871	ENSG00000121871	SLITRK3	3:164904507-164914897	q1	q2	OK	0.690706	0.365112	-0.919734	-0.874785	0.1614	0.99995	no

ENSG00000121879	ENSG00000121879	PIK3CA	3:178865901-178984790	q1	q2	OK	11.7515	11.5793	-0.021298	-0.020341	0.96235	0.99995	no

ENSG00000121892	ENSG00000121892	PDS5A	4:39824482-39979576	q1	q2	OK	35.1588	39.1278	0.15431	0.282403	0.577	0.99995	no

ENSG00000121895	ENSG00000121895	TMEM156	4:38968364-39034542	q1	q2	NOTEST	0	0	0	0	1	1	no

ENSG00000121897	ENSG00000121897	LIAS	4:39460619-39479273	q1	q2	OK	5.47909	6.80112	0.311836	0.46895	0.51595	0.99995	no

ENSG00000121898	ENSG00000121898	CPXM2	10:125465722-125699783	q1	q2	NOTEST	0.0385188	0.0563262	0.548245	0	1	1	no

ENSG00000121900	ENSG00000121900	TMEM54	1:33351594-33367039	q1	q2	OK	3.37905	2.95611	-0.192918	-0.178891	0.7854	0.99995	no

ENSG00000121903	ENSG00000121903	ZSCAN20	1:33938245-33962107	q1	q2	OK	2.44295	2.62062	0.101283	0.158214	0.82415	0.99995	no

ENSG00000121904	ENSG00000121904	CSMD2	1:33979608-34631443	q1	q2	OK	0.890586	0.734814	-0.277375	-0.223103	0.63845	0.99995	no

ENSG00000121905	ENSG00000121905	HPCA	1:33351594-33367039	q1	q2	OK	1.66971	1.65361	-0.0139723	-0.00912087	0.9859	0.99995	no

ENSG00000121931	ENSG00000121931	LRIF1	1:111486088-111507271	q1	q2	OK	14.4261	17.1951	0.253317	0.507038	0.46755	0.99995	no

ENSG00000121933	ENSG00000121933	ADORA3	1:112025969-112310638	q1	q2	NOTEST	0	0.0607195	inf	0	1	1	no

ENSG00000121940	ENSG00000121940	CLCC1	1:109358519-109506111	q1	q2	OK	14.0432	13.6299	-0.0431016	-0.058275	0.93695	0.99995	no

ENSG00000121957	ENSG00000121957	GPSM2	1:109358519-109506111	q1	q2	OK	21.1279	19.2406	-0.134997	-0.167622	0.7736	0.99995	no

ENSG00000121964	ENSG00000121964	GTDC1	2:144694639-145090135	q1	q2	OK	11.5974	13.3325	0.201153	0.31027	0.60045	0.99995	no

ENSG00000121966	ENSG00000121966	CXCR4	2:136871918-136875735	q1	q2	OK	75.001	75.6143	0.0117496	0.0287305	0.96765	0.99995	no

ENSG00000121988	ENSG00000121988	ZRANB3	2:135809834-136288806	q1	q2	OK	4.91961	4.68897	-0.0692736	-0.0356648	0.9265	0.99995	no

ENSG00000121989	ENSG00000121989	ACVR2A	2:148602085-149275805	q1	q2	OK	24.3509	21.4123	-0.185535	-0.216911	0.71595	0.99995	no

ENSG00000122008	ENSG00000122008	POLK	5:74664310-74896969	q1	q2	OK	8.68292	9.72565	0.163615	0.236135	0.73005	0.99995	no

ENSG00000122012	ENSG00000122012	SV2C	5:75377770-75649764	q1	q2	NOTEST	0.234535	0.241511	0.0422867	0	1	1	no

ENSG00000122025	ENSG00000122025	FLT3	13:28577410-28674729	q1	q2	NOTEST	0.0360381	0.0242904	-0.569135	0	1	1	no

ENSG00000122026	ENSG00000122026	RPL21	13:27825445-27830828	q1	q2	OK	521.766	514.819	-0.0193388	-0.0397995	0.9522	0.99995	no

ENSG00000122033	ENSG00000122033	MTIF3	13:27998680-28024739	q1	q2	OK	31.1357	30.4857	-0.0304329	-0.0352804	0.9532	0.99995	no

ENSG00000122034	ENSG00000122034	GTF3A	13:27998680-28024739	q1	q2	OK	60.9021	60.6922	-0.00498108	-0.00966725	0.98845	0.99995	no

ENSG00000122035	ENSG00000122035	RASL11A	13:27844463-27847827	q1	q2	OK	2.2135	1.23756	-0.838824	-0.762252	0.1945	0.99995	no

ENSG00000122042	ENSG00000122042	UBL3	13:30338507-30424821	q1	q2	OK	25.2476	25.1771	-0.00403568	-0.00951482	0.98855	0.99995	no

ENSG00000122043	ENSG00000122043	LINC00544	13:30510041-30524625	q1	q2	NOTEST	0	0	0	0	1	1	no

ENSG00000122068	ENSG00000122068	FYTTD1	3:197398263-197514467	q1	q2	OK	41.4748	42.8239	0.0461835	0.0686722	0.9097	0.99995	no

ENSG00000122085	ENSG00000122085	MTERFD2	2:241938254-242041747	q1	q2	OK	30.597	30.1281	-0.022283	-0.0381307	0.95095	0.99995	no

ENSG00000122121	ENSG00000122121	XPNPEP2	X:128872949-128903514	q1	q2	OK	0.731396	0.724617	-0.0134335	-0.00842793	0.98765	0.99995	no

ENSG00000122122	ENSG00000122122	SASH3	X:128913954-128930939	q1	q2	NOTEST	0.00978127	0.173331	4.14736	0	1	1	no

ENSG00000122126	ENSG00000122126	OCRL	X:128673825-128726538	q1	q2	OK	12.5175	13.526	0.111788	0.228374	0.711	0.99995	no

ENSG00000122133	ENSG00000122133	PAEP	9:138453601-138458801	q1	q2	NOTEST	0	0	0	0	1	1	no

ENSG00000122136	ENSG00000122136	OBP2A	9:138437984-138441815	q1	q2	NOTEST	0	0	0	0	1	1	no

ENSG00000122140	ENSG00000122140	MRPS2	9:138387026-138398293	q1	q2	OK	28.6889	24.6752	-0.217434	-0.423054	0.5292	0.99995	no

ENSG00000122145	ENSG00000122145	TBX22	X:79270254-79287268	q1	q2	NOTEST	0	0	0	0	1	1	no

ENSG00000122176	ENSG00000122176	FMOD	1:203309755-203320617	q1	q2	NOTEST	0.0228898	0.022713	-0.0111855	0	1	1	no

ENSG00000122180	ENSG00000122180	MYOG	1:203052259-203055164	q1	q2	NOTEST	0.0188519	0.0185569	-0.022756	0	1	1	no

ENSG00000122188	ENSG00000122188	LAX1	1:203734303-203745361	q1	q2	NOTEST	0	0.00912754	inf	0	1	1	no

ENSG00000122194	ENSG00000122194	PLG	6:161123269-161174347	q1	q2	NOTEST	0.0104766	0	#NAME?	0	1	1	no

ENSG00000122203	ENSG00000122203	KIAA1191	5:175773063-175788971	q1	q2	OK	69.0207	69.2674	0.0051477	0.0109891	0.98625	0.99995	no

ENSG00000122218	ENSG00000122218	COPA	1:160259062-160328742	q1	q2	OK	57.6967	54.5252	-0.0815651	-0.117936	0.83005	0.99995	no

ENSG00000122223	ENSG00000122223	CD244	1:160799949-160832692	q1	q2	NOTEST	0	0	0	0	1	1	no

ENSG00000122224	ENSG00000122224	LY9	1:160765863-160798051	q1	q2	NOTEST	0	0	0	0	1	1	no

ENSG00000122254	ENSG00000122254	HS3ST2	16:22825497-22927659	q1	q2	NOTEST	0.148578	0.0461447	-1.68698	0	1	1	no

ENSG00000122257	ENSG00000122257	RBBP6	16:24549013-24584184	q1	q2	OK	42.0762	41.4385	-0.0220319	-0.0270005	0.9559	0.99995	no

ENSG00000122299	ENSG00000122299	ZC3H7A	16:11844441-11891123	q1	q2	OK	44.3369	47.5284	0.100282	0.172184	0.73785	0.99995	no

ENSG00000122304	ENSG00000122304	PRM2	16:11343475-11445619	q1	q2	NOTEST	0	0	0	0	1	1	no

ENSG00000122335	ENSG00000122335	SERAC1	6:158530535-158589312	q1	q2	OK	11.3463	13.5961	0.260972	0.337683	0.5223	0.99995	no

ENSG00000122359	ENSG00000122359	ANXA11	10:81910644-81965328	q1	q2	OK	6.71432	5.86849	-0.194251	-0.159574	0.732	0.99995	no

ENSG00000122367	ENSG00000122367	LDB3	10:88428205-88495825	q1	q2	OK	0.694896	1.05973	0.608834	0.371536	0.4454	0.99995	no

ENSG00000122375	ENSG00000122375	OPN4	10:88414313-88426605	q1	q2	NOTEST	0	0	0	0	1	1	no

ENSG00000122376	ENSG00000122376	FAM35A	10:88810242-88951225	q1	q2	OK	7.62694	8.4849	0.153793	0.148803	0.8256	0.99995	no

ENSG00000122378	ENSG00000122378	FAM213A	10:82167584-82192753	q1	q2	OK	73.5256	80.9814	0.139343	0.339257	0.62445	0.99995	no

ENSG00000122386	ENSG00000122386	ZNF205	16:3160460-3192806	q1	q2	OK	11.6374	9.62862	-0.273367	-0.320055	0.61715	0.99995	no

ENSG00000122390	ENSG00000122390	NAA60	16:3415098-3627401	q1	q2	OK	33.7763	31.9162	-0.0817206	-0.0805693	0.84775	0.99995	no

ENSG00000122406	ENSG00000122406	RPL5	1:93297581-93307481	q1	q2	OK	924.38	934.468	0.0156583	0.0342192	0.95985	0.99995	no

ENSG00000122417	ENSG00000122417	ODF2L	1:86814343-86862023	q1	q2	OK	23.6896	26.3058	0.15113	0.21342	0.69955	0.99995	no

ENSG00000122420	ENSG00000122420	PTGFR	1:78695282-79005434	q1	q2	NOTEST	0.112373	0.146645	0.384035	0	1	1	no

ENSG00000122432	ENSG00000122432	SPATA1	1:84964007-85040163	q1	q2	OK	1.01619	1.52609	0.586667	0.0973765	0.56925	0.99995	no

ENSG00000122435	ENSG00000122435	TRMT13	1:100598705-100643771	q1	q2	OK	12.8856	13.5533	0.0728928	0.11933	0.8355	0.99995	no

ENSG00000122477	ENSG00000122477	LRRC39	1:100598705-100643771	q1	q2	OK	1.20635	1.18769	-0.0224881	-0.00981119	0.9613	0.99995	no

ENSG00000122481	ENSG00000122481	RWDD3	1:95582893-95729610	q1	q2	OK	26.299	25.628	-0.0372891	-0.061516	0.92675	0.99995	no

ENSG00000122482	ENSG00000122482	ZNF644	1:91380858-91487829	q1	q2	OK	31.5596	31.2946	-0.0121656	-0.019144	0.97255	0.99995	no

ENSG00000122483	ENSG00000122483	CCDC18	1:93615298-93835055	q1	q2	OK	10.7513	13.4989	0.328323	0.142763	0.7186	0.99995	no

ENSG00000122484	ENSG00000122484	RPAP2	1:92711958-92867613	q1	q2	OK	10.3313	9.11133	-0.181281	-0.15015	0.7868	0.99995	no

ENSG00000122490	ENSG00000122490	PQLC1	18:77662419-77711664	q1	q2	OK	17.3335	14.5563	-0.25192	-0.480197	0.4615	0.99995	no

ENSG00000122497	ENSG00000122497	NBPF14	1:148003641-148025863	q1	q2	OK	3.97135	4.0382	0.0240822	0.0409362	0.95165	0.99995	no

ENSG00000122507	ENSG00000122507	BBS9	7:33168855-33645680	q1	q2	OK	6.21302	6.1986	-0.00335291	-0.00512535	0.994	0.99995	no

ENSG00000122512	ENSG00000122512	PMS2	7:6012869-6048756	q1	q2	OK	6.04865	6.32256	0.0638941	0.100229	0.8758	0.99995	no

ENSG00000122515	ENSG00000122515	ZMIZ2	7:44788179-44809477	q1	q2	OK	32.9726	29.8422	-0.143916	-0.244943	0.6499	0.99995	no

ENSG00000122543	ENSG00000122543	OCM	7:5919457-5925993	q1	q2	NOTEST	0.106811	0.0553037	-0.94961	0	1	1	no

ENSG00000122545	ENSG00000122545	Sep-07	7:35840541-35944917	q1	q2	OK	146.883	164.57	0.164031	0.366114	0.58135	0.99995	no

ENSG00000122547	ENSG00000122547	EEPD1	7:36192757-36341152	q1	q2	OK	1.84945	2.30342	0.316683	0.246708	0.63585	0.99995	no

ENSG00000122548	ENSG00000122548	KIAA0087	7:26572739-26578407	q1	q2	NOTEST	0.175124	0.140888	-0.313828	0	1	1	no

ENSG00000122550	ENSG00000122550	KLHL7	7:23145352-23240630	q1	q2	OK	71.9535	73.5669	0.0319915	0.0545304	0.9282	0.99995	no

ENSG00000122557	ENSG00000122557	HERPUD2	7:35672268-35739023	q1	q2	OK	10.9599	11.1526	0.0251437	0.036532	0.95495	0.99995	no

ENSG00000122565	ENSG00000122565	CBX3	7:26229546-26252976	q1	q2	OK	223.015	243.651	0.12768	0.0768039	0.8955	0.99995	no

ENSG00000122566	ENSG00000122566	HNRNPA2B1	7:26229546-26252976	q1	q2	OK	1094.7	1165.64	0.0905897	0.130344	0.84395	0.99995	no

ENSG00000122574	ENSG00000122574	WIPF3	7:29846101-29956682	q1	q2	OK	0.73712	0.823757	0.160319	0.192952	0.7945	0.99995	no

ENSG00000122584	ENSG00000122584	NXPH1	7:8473584-8792593	q1	q2	OK	3.88204	3.12547	-0.312741	-0.358005	0.57975	0.99995	no

ENSG00000122585	ENSG00000122585	NPY	7:24323781-24331484	q1	q2	OK	2.6519	2.85144	0.104665	0.1033	0.88	0.99995	no

ENSG00000122591	ENSG00000122591	FAM126A	7:22980877-23053749	q1	q2	OK	24.0917	28.8659	0.260834	0.413836	0.40085	0.99995	no

ENSG00000122592	ENSG00000122592	HOXA7	7:27145802-27197555	q1	q2	NOTEST	0.145615	0.01355	-3.4258	0	1	1	no

ENSG00000122641	ENSG00000122641	INHBA	7:41706765-41818986	q1	q2	OK	0.657962	0.748733	0.186448	0.174153	0.7802	0.99995	no

ENSG00000122642	ENSG00000122642	FKBP9	7:32535037-33102409	q1	q2	OK	28.944	28.4533	-0.0246691	-0.0219355	0.96605	0.99995	no

ENSG00000122643	ENSG00000122643	NT5C3A	7:32535037-33102409	q1	q2	OK	22.1418	24.0088	0.116788	0.0726289	0.8914	0.99995	no

ENSG00000122644	ENSG00000122644	ARL4A	7:12726480-12730559	q1	q2	OK	48.0516	42.8955	-0.163758	-0.302852	0.65985	0.99995	no

ENSG00000122674	ENSG00000122674	CCZ1	7:5938355-6010314	q1	q2	OK	28.8385	29.6905	0.0420012	0.0563875	0.9321	0.99995	no

ENSG00000122678	ENSG00000122678	POLM	7:44111845-44122139	q1	q2	OK	6.74081	5.50565	-0.292009	-0.409159	0.50125	0.99995	no

ENSG00000122679	ENSG00000122679	RAMP3	7:45197389-45225901	q1	q2	NOTEST	0	0	0	0	1	1	no

ENSG00000122687	ENSG00000122687	FTSJ2	7:2273865-2281840	q1	q2	OK	27.4637	26.9479	-0.027355	-0.0590855	0.93035	0.99995	no

ENSG00000122691	ENSG00000122691	TWIST1	7:19060613-19157295	q1	q2	OK	5.34338	5.9155	0.146747	0.174051	0.79105	0.99995	no

ENSG00000122692	ENSG00000122692	SMU1	9:33041761-33076665	q1	q2	OK	37.7438	39.3059	0.0585059	0.105119	0.8847	0.99995	no

ENSG00000122694	ENSG00000122694	GLIPR2	9:36136731-36163910	q1	q2	OK	27.9374	28.7741	0.0425743	0.0901374	0.8964	0.99995	no

ENSG00000122696	ENSG00000122696	SLC25A51	9:37510888-38069210	q1	q2	OK	10.3702	11.1363	0.102818	0.0605886	0.93845	0.99995	no

ENSG00000122705	ENSG00000122705	CLTA	9:36190852-36304921	q1	q2	OK	144.465	139.754	-0.0478274	-0.0994434	0.8914	0.99995	no

ENSG00000122707	ENSG00000122707	RECK	9:36036429-36124448	q1	q2	OK	9.0216	9.54877	0.0819318	0.13701	0.8351	0.99995	no

ENSG00000122711	ENSG00000122711	SPINK4	9:33218362-33264761	q1	q2	NOTEST	0	0	0	0	1	1	no

ENSG00000122718	ENSG00000122718	OR2S2	9:35957104-35958151	q1	q2	NOTEST	0	0.0273377	inf	0	1	1	no

ENSG00000122728	ENSG00000122728	TAF1L	9:32629451-32648683	q1	q2	NOTEST	0.0735435	0.0630614	-0.22184	0	1	1	no

ENSG00000122729	ENSG00000122729	ACO1	9:32384617-32454767	q1	q2	OK	19.4527	20.3199	0.062923	0.147938	0.8329	0.99995	no

ENSG00000122733	ENSG00000122733	KIAA1045	9:34957483-34982541	q1	q2	NOTEST	0.0744903	0.0673558	-0.145251	0	1	1	no

ENSG00000122735	ENSG00000122735	DNAI1	9:34398181-34520982	q1	q2	OK	2.9352	1.93734	-0.599381	-0.131284	0.59555	0.99995	no

ENSG00000122741	ENSG00000122741	DCAF10	9:37510888-38069210	q1	q2	OK	12.1296	13.2427	0.126666	0.0807129	0.8423	0.99995	no

ENSG00000122756	ENSG00000122756	CNTFR	9:34551429-34590121	q1	q2	OK	72.805	66.1562	-0.138162	-0.333015	0.6415	0.99995	no

ENSG00000122778	ENSG00000122778	KIAA1549	7:138391039-138666064	q1	q2	OK	10.3419	10.4099	0.00945109	0.0216026	0.97715	0.99995	no

ENSG00000122779	ENSG00000122779	TRIM24	7:138145078-138274738	q1	q2	OK	49.1777	47.4794	-0.0507014	-0.114069	0.8662	0.99995	no

ENSG00000122783	ENSG00000122783	C7orf49	7:134671258-134855547	q1	q2	OK	24.7183	23.535	-0.0707701	-0.115998	0.8587	0.99995	no

ENSG00000122786	ENSG00000122786	CALD1	7:134429002-134655479	q1	q2	OK	73.6945	82.6867	0.166099	0.274085	0.5724	0.99995	no

ENSG00000122787	ENSG00000122787	AKR1D1	7:137687069-137802732	q1	q2	NOTEST	0	0	0	0	1	1	no

ENSG00000122824	ENSG00000122824	NUDT10	X:51075082-51080377	q1	q2	NOTEST	0.185704	0.110151	-0.753525	0	1	1	no

ENSG00000122852	ENSG00000122852	SFTPA1	10:81370694-81375196	q1	q2	NOTEST	0.203304	0	#NAME?	0	1	1	no

ENSG00000122859	ENSG00000122859	NEUROG3	10:71331453-71332994	q1	q2	OK	1.31806	1.18592	-0.152409	-0.160199	0.8277	0.99995	no

ENSG00000122861	ENSG00000122861	PLAU	10:75668934-75682535	q1	q2	OK	10.4846	7.91206	-0.406148	-0.677373	0.31285	0.99995	no

ENSG00000122862	ENSG00000122862	SRGN	10:70847861-70864567	q1	q2	NOTEST	0.0813086	0.0229326	-1.82601	0	1	1	no

ENSG00000122863	ENSG00000122863	CHST3	10:73724122-73773322	q1	q2	OK	6.12311	5.94825	-0.0418011	-0.0920221	0.89925	0.99995	no

ENSG00000122870	ENSG00000122870	BICC1	10:60272899-60591195	q1	q2	OK	7.98388	7.98624	0.000426194	0.000846153	0.99895	0.99995	no

ENSG00000122872	ENSG00000122872	ARL4P	10:61786055-62493248	q1	q2	NOTEST	0	0	0	0	1	1	no

ENSG00000122873	ENSG00000122873	CISD1	10:60028817-60049346	q1	q2	OK	24.0542	22.1849	-0.11671	-0.164746	0.8095	0.99995	no

ENSG00000122877	ENSG00000122877	EGR2	10:64571755-64679660	q1	q2	OK	9.6281	11.2301	0.222051	0.43996	0.52825	0.99995	no

ENSG00000122882	ENSG00000122882	ECD	10:74870216-75118617	q1	q2	OK	16.6314	17.5788	0.0799273	0.0661811	0.92935	0.99995	no

ENSG00000122884	ENSG00000122884	P4HA1	10:74766974-74856732	q1	q2	OK	18.2823	20.7209	0.180638	0.372916	0.5589	0.99995	no

ENSG00000122912	ENSG00000122912	SLC25A16	10:70237755-70287231	q1	q2	OK	4.62548	5.23796	0.179403	0.178869	0.7096	0.99995	no

ENSG00000122952	ENSG00000122952	ZWINT	10:58116988-58121036	q1	q2	OK	63.2095	65.727	0.0563436	0.131397	0.84805	0.99995	no

ENSG00000122958	ENSG00000122958	VPS26A	10:70883267-70932617	q1	q2	OK	38.0411	44.0078	0.210201	0.463932	0.4589	0.99995	no

ENSG00000122965	ENSG00000122965	RBM19	12:114254542-114404176	q1	q2	OK	12.3469	12.8573	0.0584275	0.0960405	0.88305	0.99995	no

ENSG00000122966	ENSG00000122966	CIT	12:120123594-120315095	q1	q2	OK	15.8258	16.9006	0.094797	0.0896692	0.7938	0.99995	no

ENSG00000122970	ENSG00000122970	IFT81	12:110562139-110656602	q1	q2	OK	35.7208	35.5905	-0.0052729	-0.0120064	0.9851	0.99995	no

ENSG00000122971	ENSG00000122971	ACADS	12:121163537-121177811	q1	q2	OK	1.7783	1.75197	-0.0215276	-0.0246024	0.9729	0.99995	no

ENSG00000122986	ENSG00000122986	HVCN1	12:111051831-111142755	q1	q2	OK	1.74455	1.93153	0.146891	0.0706138	0.915	0.99995	no

ENSG00000123009	ENSG00000123009	NME2P1	12:120719939-120720632	q1	q2	OK	2.47105	4.93582	0.998165	0.916802	0.19615	0.99995	no

ENSG00000123064	ENSG00000123064	DDX54	12:113587662-113623284	q1	q2	OK	23.4916	19.0179	-0.304786	-0.376467	0.4987	0.99995	no

ENSG00000123066	ENSG00000123066	MED13L	12:116395710-116715143	q1	q2	OK	16.989	16.8489	-0.011945	-0.0142703	0.96625	0.99995	no

ENSG00000123080	ENSG00000123080	CDKN2C	1:51426416-51440305	q1	q2	OK	2.85057	2.62247	-0.120324	-0.160301	0.8183	0.99995	no

ENSG00000123091	ENSG00000123091	RNF11	1:51701942-51739127	q1	q2	OK	40.0068	36.1265	-0.147189	-0.207828	0.74275	0.99995	no

ENSG00000123094	ENSG00000123094	RASSF8	12:26092261-26232825	q1	q2	OK	20.5684	22.2452	0.113061	0.175555	0.7105	0.99995	no

ENSG00000123095	ENSG00000123095	BHLHE41	12:26272958-26986131	q1	q2	NOTEST	0.328688	0.36658	0.15741	0	1	1	no

ENSG00000123096	ENSG00000123096	SSPN	12:26272958-26986131	q1	q2	OK	1.85172	2.25136	0.281928	0.139121	0.7463	0.99995	no

ENSG00000123104	ENSG00000123104	ITPR2	12:26272958-26986131	q1	q2	OK	2.8616	3.33055	0.218936	0.265019	0.51115	0.99995	no

ENSG00000123106	ENSG00000123106	CCDC91	12:28286181-28732883	q1	q2	OK	10.7932	11.2257	0.0566867	0.070234	0.88555	0.99995	no

ENSG00000123119	ENSG00000123119	NECAB1	8:91634222-91997485	q1	q2	OK	0.424431	0.517578	0.286246	0.153994	0.76615	0.99995	no

ENSG00000123124	ENSG00000123124	WWP1	8:87345502-87777251	q1	q2	OK	13.9701	14.7136	0.074811	0.0623673	0.9014	0.99995	no

ENSG00000123130	ENSG00000123130	ACOT9	X:23720369-23784592	q1	q2	OK	12.154	11.9444	-0.0250956	-0.0384306	0.95165	0.99995	no

ENSG00000123131	ENSG00000123131	PRDX4	X:23682378-23704516	q1	q2	OK	48.0837	47.4546	-0.0189994	-0.0411591	0.95325	0.99995	no

ENSG00000123136	ENSG00000123136	DDX39A	19:14519630-14530192	q1	q2	OK	63.0041	59.5461	-0.0814373	-0.180299	0.77565	0.99995	no

ENSG00000123143	ENSG00000123143	PKN1	19:14543864-14582679	q1	q2	OK	36.0735	32.023	-0.171835	-0.3677	0.547	0.99995	no

ENSG00000123144	ENSG00000123144	C19orf43	19:12841453-12845589	q1	q2	OK	174.454	157.412	-0.148295	-0.366293	0.6061	0.99995	no

ENSG00000123146	ENSG00000123146	CD97	19:14491312-14519537	q1	q2	OK	3.29662	2.49522	-0.401819	-0.272658	0.48365	0.99995	no

ENSG00000123154	ENSG00000123154	WDR83	19:12754088-12792716	q1	q2	OK	10.5431	9.91032	-0.0892993	-0.0475165	0.9371	0.99995	no

ENSG00000123159	ENSG00000123159	GIPC1	19:14588571-14606944	q1	q2	OK	47.5078	39.365	-0.271249	-0.537592	0.4119	0.99995	no

ENSG00000123165	ENSG00000123165	ACTRT1	X:127184942-127186382	q1	q2	NOTEST	0.0393296	0	#NAME?	0	1	1	no

ENSG00000123171	ENSG00000123171	CCDC70	13:52436116-52440366	q1	q2	NOTEST	0	0	0	0	1	1	no

ENSG00000123178	ENSG00000123178	SPRYD7	13:50486841-50510626	q1	q2	OK	3.11182	3.05576	-0.026225	-0.0400286	0.9524	0.99995	no

ENSG00000123179	ENSG00000123179	EBPL	13:50234858-50265611	q1	q2	OK	31.1787	34.5247	0.147065	0.279262	0.68695	0.99995	no

ENSG00000123191	ENSG00000123191	ATP7B	13:52506808-52585630	q1	q2	OK	3.44707	3.71187	0.106777	0.119985	0.79275	0.99995	no

ENSG00000123200	ENSG00000123200	ZC3H13	13:46528599-46626894	q1	q2	OK	38.6075	39.6827	0.0396282	0.0785151	0.8867	0.99995	no

ENSG00000123201	ENSG00000123201	GUCY1B2	13:51568646-51654998	q1	q2	NOTEST	0	0	0	0	1	1	no

ENSG00000123213	ENSG00000123213	NLN	5:64885506-65167553	q1	q2	OK	13.4266	15.0779	0.167345	0.179542	0.75725	0.99995	no

ENSG00000123219	ENSG00000123219	CENPK	5:64813592-64858998	q1	q2	OK	36.1295	39.8247	0.140485	0.286991	0.6621	0.99995	no

ENSG00000123240	ENSG00000123240	OPTN	10:12938626-13180291	q1	q2	OK	6.99342	7.15378	0.0327074	0.0367439	0.94685	0.99995	no

ENSG00000123243	ENSG00000123243	ITIH5	10:7601231-7708961	q1	q2	OK	0.5851	0.941642	0.686494	0.575391	0.3008	0.99995	no

ENSG00000123268	ENSG00000123268	ATF1	12:51157492-51214905	q1	q2	OK	11.8356	12.9764	0.132753	0.207688	0.72945	0.99995	no

ENSG00000123297	ENSG00000123297	TSFM	12:58156116-58212487	q1	q2	OK	19.1475	16.3842	-0.224852	-0.309366	0.6692	0.99995	no

ENSG00000123307	ENSG00000123307	NEUROD4	12:55413728-55423798	q1	q2	OK	3.02831	3.76613	0.314571	0.532725	0.4511	0.99995	no

ENSG00000123329	ENSG00000123329	ARHGAP9	12:57853917-57914300	q1	q2	OK	0.578752	0.541017	-0.0972715	-0.010081	0.90705	0.99995	no

ENSG00000123338	ENSG00000123338	NCKAP1L	12:54891494-54937889	q1	q2	NOTEST	0.0908966	0.149366	0.716555	0	1	1	no

ENSG00000123342	ENSG00000123342	MMP19	12:56137063-56236750	q1	q2	OK	0.716668	0.464016	-0.627131	-0.0891968	0.7123	0.99995	no

ENSG00000123349	ENSG00000123349	PFDN5	12:53689074-53700961	q1	q2	OK	282.707	280.281	-0.0124361	-0.0269423	0.9687	0.99995	no

ENSG00000123352	ENSG00000123352	SPATS2	12:49760366-49921205	q1	q2	OK	27.9156	28.4085	0.0252545	0.0483366	0.93225	0.99995	no

ENSG00000123353	ENSG00000123353	ORMDL2	12:56137063-56236750	q1	q2	OK	17.7234	17.2806	-0.0364993	-0.0177763	0.97695	0.99995	no

ENSG00000123358	ENSG00000123358	NR4A1	12:52416615-52453291	q1	q2	OK	8.40491	7.8584	-0.0969957	-0.130809	0.8115	0.99995	no

ENSG00000123360	ENSG00000123360	PDE1B	12:54943133-54982443	q1	q2	OK	0.742606	0.372983	-0.993485	-0.601185	0.3763	0.99995	no

ENSG00000123364	ENSG00000123364	HOXC13	12:54329111-54340328	q1	q2	NOTEST	0	0.020567	inf	0	1	1	no

ENSG00000123374	ENSG00000123374	CDK2	12:56347888-56367101	q1	q2	OK	63.1466	60.1726	-0.0695999	-0.125231	0.83015	0.99995	no

ENSG00000123384	ENSG00000123384	LRP1	12:57482676-57607134	q1	q2	OK	44.9618	40.7623	-0.141464	-0.180739	0.73005	0.99995	no

ENSG00000123388	ENSG00000123388	HOXC11	12:54356091-54371427	q1	q2	NOTEST	0	0	0	0	1	1	no

ENSG00000123395	ENSG00000123395	C12orf44	12:52463029-52502045	q1	q2	OK	25.0073	23.5898	-0.0841832	-0.162722	0.8165	0.99995	no

ENSG00000123405	ENSG00000123405	NFE2	12:54624723-54745633	q1	q2	NOTEST	0.0944824	0.0806773	-0.227882	0	1	1	no

ENSG00000123407	ENSG00000123407	HOXC12	12:54348617-54352745	q1	q2	NOTEST	0	0	0	0	1	1	no

ENSG00000123411	ENSG00000123411	IKZF4	12:56401442-56435590	q1	q2	OK	13.9071	13.6426	-0.0277076	-0.044414	0.92605	0.99995	no

ENSG00000123415	ENSG00000123415	SMUG1	12:54555848-54582778	q1	q2	OK	19.6967	16.333	-0.270167	-0.401106	0.518	0.99995	no

ENSG00000123416	ENSG00000123416	TUBA1B	12:49521564-49541652	q1	q2	OK	1286.55	1284.61	-0.00217139	-0.00370518	0.99565	0.99995	no

ENSG00000123427	ENSG00000123427	METTL21B	12:58156116-58212487	q1	q2	OK	2.35209	2.32684	-0.0155741	-0.0113322	0.9866	0.99995	no

ENSG00000123444	ENSG00000123444	KBTBD4	11:47487495-47606114	q1	q2	OK	20.64	20.0554	-0.0414511	-0.0301047	0.96595	0.99995	no

ENSG00000123447	ENSG00000123447	TYRL	11:49426526-49437299	q1	q2	NOTEST	0	0	0	0	1	1	no

ENSG00000123453	ENSG00000123453	SARDH	9:136528681-136605077	q1	q2	OK	1.1271	1.20606	0.0976892	0.107433	0.8638	0.99995	no

ENSG00000123454	ENSG00000123454	DBH	9:136501481-136524466	q1	q2	OK	0.948735	1.08306	0.191036	0.183864	0.79185	0.99995	no

ENSG00000123472	ENSG00000123472	ATPAF1	1:47098408-47184824	q1	q2	OK	39.8307	39.9154	0.00306439	0.00406124	0.99495	0.99995	no

ENSG00000123473	ENSG00000123473	STIL	1:47715810-47779819	q1	q2	OK	12.2485	13.849	0.17718	0.339722	0.55835	0.99995	no

ENSG00000123485	ENSG00000123485	HJURP	2:234684369-234763212	q1	q2	OK	29.8346	30.7026	0.0413729	0.0756123	0.8926	0.99995	no

ENSG00000123496	ENSG00000123496	IL13RA2	X:114238537-114254540	q1	q2	NOTEST	0	0	0	0	1	1	no

ENSG00000123500	ENSG00000123500	COL10A1	6:116422011-116570660	q1	q2	NOTEST	0.0598905	0.0393698	-0.605238	0	1	1	no

ENSG00000123505	ENSG00000123505	AMD1	6:111195972-111216916	q1	q2	OK	38.859	43.8843	0.175457	0.411148	0.53525	0.99995	no

ENSG00000123545	ENSG00000123545	NDUFAF4	6:97337188-97345757	q1	q2	OK	26.5287	32.5084	0.293257	0.423893	0.55405	0.99995	no

ENSG00000123552	ENSG00000123552	USP45	6:99880189-99979794	q1	q2	OK	6.754	7.00273	0.0521747	0.067547	0.90515	0.99995	no

ENSG00000123560	ENSG00000123560	PLP1	X:103028646-103047548	q1	q2	OK	45.6945	37.8502	-0.271719	-0.52098	0.3396	0.99995	no

ENSG00000123561	ENSG00000123561	SERPINA7	X:105277196-105282729	q1	q2	NOTEST	0	0	0	0	1	1	no

ENSG00000123562	ENSG00000123562	MORF4L2	X:102930423-102946700	q1	q2	OK	281.018	291.204	0.0513661	0.0821072	0.89215	0.99995	no

ENSG00000123569	ENSG00000123569	H2BFWT	X:103265718-103268259	q1	q2	NOTEST	0	0	0	0	1	1	no

ENSG00000123570	ENSG00000123570	RAB9B	X:103077251-103087158	q1	q2	OK	2.96692	3.03305	0.0318051	0.052186	0.94115	0.99995	no

ENSG00000123572	ENSG00000123572	NRK	X:105066535-105202602	q1	q2	OK	1.73242	1.94375	0.166051	0.254702	0.68145	0.99995	no

ENSG00000123575	ENSG00000123575	FAM199X	X:103411300-103440583	q1	q2	NOTEST	0	0	0	0	1	1	no

ENSG00000123576	ENSG00000123576	ESX1	X:103494718-103499614	q1	q2	NOTEST	0	0	0	0	1	1	no

ENSG00000123584	ENSG00000123584	MAGEA9B	X:148663308-148669116	q1	q2	NOTEST	0	0	0	0	1	1	no

ENSG00000123594	ENSG00000123594	ATXN3L	X:13328770-13338518	q1	q2	NOTEST	0	0	0	0	1	1	no

ENSG00000123595	ENSG00000123595	RAB9A	X:13707243-13728625	q1	q2	OK	15.4628	15.0616	-0.0379215	-0.0566166	0.9349	0.99995	no

ENSG00000123600	ENSG00000123600	METTL8	2:172173918-172341562	q1	q2	OK	6.39139	8.40047	0.394341	0.277357	0.5122	0.99995	no

ENSG00000123607	ENSG00000123607	TTC21B	2:166713984-166810353	q1	q2	OK	10.9548	11.4905	0.0688751	0.101547	0.8386	0.99995	no

ENSG00000123609	ENSG00000123609	NMI	2:152126978-152146571	q1	q2	OK	1.07034	0.671024	-0.673632	-0.584642	0.32195	0.99995	no

ENSG00000123610	ENSG00000123610	TNFAIP6	2:152214105-152236560	q1	q2	OK	2.901	3.86218	0.412864	0.36573	0.54245	0.99995	no

ENSG00000123612	ENSG00000123612	ACVR1C	2:158383278-158485517	q1	q2	NOTEST	0.0368928	0.060021	0.702128	0	1	1	no

ENSG00000123636	ENSG00000123636	BAZ2B	2:160175489-160473593	q1	q2	OK	36.4295	36.7852	0.0140168	0.0149721	0.9747	0.99995	no

ENSG00000123643	ENSG00000123643	SLC36A1	5:150816606-150871942	q1	q2	OK	4.9293	5.01372	0.024497	0.0400587	0.9412	0.99995	no

ENSG00000123684	ENSG00000123684	LPGAT1	1:211916798-212027045	q1	q2	OK	9.50912	9.45854	-0.00769444	-0.00686761	0.98205	0.99995	no

ENSG00000123685	ENSG00000123685	BATF3	1:212859759-212873327	q1	q2	OK	1.5409	1.04293	-0.563141	-0.401038	0.54435	0.99995	no

ENSG00000123689	ENSG00000123689	G0S2	1:209834708-209908295	q1	q2	OK	1.68893	1.94422	0.203082	0.19403	0.77775	0.99995	no

ENSG00000123700	ENSG00000123700	KCNJ2	17:68163101-68176189	q1	q2	OK	1.54068	1.85105	0.264777	0.378251	0.586	0.99995	no

ENSG00000123728	ENSG00000123728	RAP2C	X:131337052-131623996	q1	q2	OK	15.9133	16.6318	0.0637086	0.113786	0.86475	0.99995	no

ENSG00000123737	ENSG00000123737	EXOSC9	4:122722471-122745087	q1	q2	OK	45.9633	50.7558	0.143091	0.174761	0.78285	0.99995	no

ENSG00000123739	ENSG00000123739	PLA2G12A	4:110631144-110651233	q1	q2	OK	8.48301	8.33796	-0.0248814	-0.0136236	0.97055	0.99995	no

ENSG00000123810	ENSG00000123810	B9D2	19:41768400-41930910	q1	q2	OK	8.50789	6.31783	-0.429373	-0.105791	0.8325	0.99995	no

ENSG00000123815	ENSG00000123815	ADCK4	19:41197433-41271294	q1	q2	OK	18.0146	15.8814	-0.181827	-0.126158	0.7624	0.99995	no

ENSG00000123836	ENSG00000123836	PFKFB2	1:207217193-207254369	q1	q2	OK	2.85657	3.11364	0.12432	0.107419	0.82005	0.99995	no

ENSG00000123838	ENSG00000123838	C4BPA	1:207277606-207318317	q1	q2	NOTEST	0	0	0	0	1	1	no

ENSG00000123843	ENSG00000123843	C4BPB	1:207262186-207273338	q1	q2	NOTEST	0	0	0	0	1	1	no

ENSG00000123870	ENSG00000123870	ZNF137P	19:53091757-53193749	q1	q2	OK	3.92204	3.09199	-0.343066	-0.120304	0.71975	0.99995	no

ENSG00000123892	ENSG00000123892	RAB38	11:87831758-87908635	q1	q2	NOTEST	0.3316	0.279998	-0.244029	0	1	1	no

ENSG00000123901	ENSG00000123901	GPR83	11:94110476-94134585	q1	q2	NOTEST	0.182836	0.18199	-0.00669188	0	1	1	no

ENSG00000123908	ENSG00000123908	AGO2	8:141541263-141645718	q1	q2	OK	8.25827	8.75793	0.0847504	0.0858929	0.81605	0.99995	no

ENSG00000123933	ENSG00000123933	MXD4	4:2249158-2264021	q1	q2	OK	31.3873	24.8871	-0.334782	-0.573552	0.2588	0.99995	no

ENSG00000123965	ENSG00000123965	PMS2P5	7:74306893-74366314	q1	q2	OK	0.482636	0.10235	-2.23742	-0.934006	0.04665	0.99995	no

ENSG00000123975	ENSG00000123975	CKS2	9:91926112-91931618	q1	q2	OK	154.797	166.905	0.108643	0.255524	0.71155	0.99995	no

ENSG00000123977	ENSG00000123977	DAW1	2:228735769-228789060	q1	q2	NOTEST	0	0.0317151	inf	0	1	1	no

ENSG00000123983	ENSG00000123983	ACSL3	2:223725651-223809357	q1	q2	OK	55.398	60.5225	0.127638	0.182203	0.7661	0.99995	no

ENSG00000123989	ENSG00000123989	CHPF	2:220403668-220415317	q1	q2	OK	20.2367	16.2648	-0.315219	-0.571531	0.409	0.99995	no

ENSG00000123992	ENSG00000123992	DNPEP	2:220238267-220268355	q1	q2	OK	13.1841	12.0022	-0.1355	-0.152308	0.80445	0.99995	no

ENSG00000123999	ENSG00000123999	INHA	2:220415450-220440435	q1	q2	OK	0.622725	0.31096	-1.00186	-0.132383	0.47755	0.99995	no

ENSG00000124003	ENSG00000124003	MOGAT1	2:223536504-223574649	q1	q2	NOTEST	0.0590969	0	#NAME?	0	1	1	no

ENSG00000124006	ENSG00000124006	OBSL1	2:220415450-220440435	q1	q2	OK	47.2976	43.4655	-0.121895	-0.206954	0.73385	0.99995	no

ENSG00000124019	ENSG00000124019	FAM124B	2:225243414-225266802	q1	q2	NOTEST	0.0108325	0.0206405	0.930111	0	1	1	no

ENSG00000124067	ENSG00000124067	SLC12A4	16:67973652-68003504	q1	q2	OK	12.6503	13.1513	0.0560345	0.0583471	0.88935	0.99995	no

ENSG00000124074	ENSG00000124074	ENKD1	16:67696847-67702661	q1	q2	OK	14.665	13.1424	-0.158147	-0.239747	0.69235	0.99995	no

ENSG00000124089	ENSG00000124089	MC3R	20:54823787-54824871	q1	q2	NOTEST	0	0	0	0	1	1	no

ENSG00000124091	ENSG00000124091	GCNT7	20:55043646-55101198	q1	q2	NOTEST	0.0671262	0.0926162	0.464389	0	1	1	no

ENSG00000124092	ENSG00000124092	CTCFL	20:56071034-56100708	q1	q2	NOTEST	0.0485485	0.0581989	0.261567	0	1	1	no

ENSG00000124097	ENSG00000124097	HMGB1P1	20:56063447-56064083	q1	q2	OK	0.217741	1.17984	2.43791	1.65523	0.1129	0.99995	no

ENSG00000124098	ENSG00000124098	FAM210B	20:54933970-54943719	q1	q2	OK	26.4477	25.0268	-0.0796684	-0.181484	0.7871	0.99995	no

ENSG00000124102	ENSG00000124102	PI3	20:43803516-43805185	q1	q2	NOTEST	0.0629437	0	#NAME?	0	1	1	no

ENSG00000124103	ENSG00000124103	FAM209A	20:55043646-55101198	q1	q2	NOTEST	0.0705189	0.109904	0.640166	0	1	1	no

ENSG00000124104	ENSG00000124104	SNX21	20:44462448-44486045	q1	q2	OK	8.01915	6.40412	-0.324448	-0.446579	0.5125	0.99995	no

ENSG00000124107	ENSG00000124107	SLPI	20:43880879-43883205	q1	q2	NOTEST	0.0599285	0	#NAME?	0	1	1	no

ENSG00000124116	ENSG00000124116	WFDC3	20:44376582-44420571	q1	q2	NOTEST	0.427706	0.128433	-1.7356	0	1	1	no

ENSG00000124120	ENSG00000124120	TTPAL	20:43104525-43123244	q1	q2	OK	7.26612	7.39087	0.0245578	0.0534325	0.9372	0.99995	no

ENSG00000124126	ENSG00000124126	PREX1	20:47240789-47444420	q1	q2	OK	68.7829	67.4661	-0.0278877	-0.0527314	0.9279	0.99995	no

ENSG00000124134	ENSG00000124134	KCNS1	20:43720950-43729753	q1	q2	NOTEST	0.01641	0.0249921	0.606902	0	1	1	no

ENSG00000124140	ENSG00000124140	SLC12A5	20:44637546-44688784	q1	q2	OK	4.38954	3.03202	-0.533791	-0.224073	0.63325	0.99995	no

ENSG00000124143	ENSG00000124143	ARHGAP40	20:37230576-37279678	q1	q2	NOTEST	0.0179699	0.0174112	-0.0455654	0	1	1	no

ENSG00000124145	ENSG00000124145	SDC4	20:43953927-43977064	q1	q2	OK	3.77231	3.40783	-0.146596	-0.203913	0.7796	0.99995	no

ENSG00000124151	ENSG00000124151	NCOA3	20:46130600-46415360	q1	q2	OK	8.19258	9.14583	0.158797	0.191209	0.7594	0.99995	no

ENSG00000124155	ENSG00000124155	PIGT	20:44044716-44054884	q1	q2	OK	29.0313	27.131	-0.0976669	-0.226733	0.7468	0.99995	no

ENSG00000124157	ENSG00000124157	SEMG2	20:43849940-43853099	q1	q2	NOTEST	0	0	0	0	1	1	no

ENSG00000124159	ENSG00000124159	MATN4	20:43922084-43945803	q1	q2	NOTEST	0.0599963	0.0136442	-2.13659	0	1	1	no

ENSG00000124160	ENSG00000124160	NCOA5	20:44689623-44718591	q1	q2	OK	35.2474	34.6871	-0.0231178	-0.051562	0.9345	0.99995	no

ENSG00000124164	ENSG00000124164	VAPB	20:56964177-57026157	q1	q2	OK	25.6027	28.2583	0.142378	0.245879	0.71435	0.99995	no

ENSG00000124171	ENSG00000124171	PARD6B	20:49348080-49373332	q1	q2	OK	2.03176	1.8637	-0.124561	-0.187908	0.7766	0.99995	no

ENSG00000124172	ENSG00000124172	ATP5E	20:57594308-57607437	q1	q2	OK	37.9125	38.5745	0.0249757	0.0586746	0.92835	0.99995	no

ENSG00000124177	ENSG00000124177	CHD6	20:40030740-40247133	q1	q2	OK	15.1567	16.0129	0.0792756	0.134918	0.8057	0.99995	no

ENSG00000124181	ENSG00000124181	PLCG1	20:39657457-39946312	q1	q2	OK	120.426	117.713	-0.0328711	-0.0291691	0.942	0.99995	no

ENSG00000124191	ENSG00000124191	TOX2	20:42543503-42698256	q1	q2	OK	8.74902	7.98694	-0.131479	-0.206207	0.76345	0.99995	no

ENSG00000124193	ENSG00000124193	SRSF6	20:42086567-42092245	q1	q2	OK	145.369	150.923	0.0540884	0.11428	0.86675	0.99995	no

ENSG00000124194	ENSG00000124194	GDAP1L1	20:42875886-42909013	q1	q2	OK	13.2162	10.8348	-0.286642	-0.434788	0.5437	0.99995	no

ENSG00000124196	ENSG00000124196	GTSF1L	20:42354803-42355638	q1	q2	NOTEST	0	0.0386992	inf	0	1	1	no

ENSG00000124198	ENSG00000124198	ARFGEF2	20:47538426-47653230	q1	q2	OK	8.41486	8.65315	0.040287	0.0684486	0.893	0.99995	no

ENSG00000124201	ENSG00000124201	ZNFX1	20:47835883-47905797	q1	q2	OK	4.75142	4.86666	0.0345751	0.0304961	0.95105	0.99995	no

ENSG00000124203	ENSG00000124203	ZNF831	20:57766074-57834168	q1	q2	NOTEST	0	0	0	0	1	1	no

ENSG00000124205	ENSG00000124205	EDN3	20:57875481-57901047	q1	q2	OK	0.517064	0.629147	0.283055	0.241442	0.7057	0.99995	no

ENSG00000124207	ENSG00000124207	CSE1L	20:47662848-47713489	q1	q2	OK	95.2285	103.756	0.123727	0.294077	0.66815	0.99995	no

ENSG00000124208	ENSG00000124208	TMEM189-UBE2V1	20:48697660-48770335	q1	q2	NOTEST	0.168664	0	#NAME?	0	1	1	no

ENSG00000124209	ENSG00000124209	RAB22A	20:56884751-56942563	q1	q2	OK	8.0041	9.03172	0.17426	0.379136	0.5394	0.99995	no

ENSG00000124212	ENSG00000124212	PTGIS	20:48120410-48184683	q1	q2	OK	3.01714	2.89325	-0.0604906	-0.103213	0.87755	0.99995	no

ENSG00000124214	ENSG00000124214	STAU1	20:47729877-47804904	q1	q2	OK	56.2695	58.0083	0.043905	0.107241	0.87565	0.99995	no

ENSG00000124215	ENSG00000124215	CDH26	20:58533470-58609066	q1	q2	NOTEST	0.265592	0.194075	-0.452602	0	1	1	no

ENSG00000124216	ENSG00000124216	SNAI1	20:48599535-48605423	q1	q2	OK	1.38298	1.60437	0.214225	0.224484	0.7446	0.99995	no

ENSG00000124217	ENSG00000124217	MOCS3	20:49575362-49577820	q1	q2	OK	6.316	5.51612	-0.195358	-0.33648	0.63	0.99995	no

ENSG00000124222	ENSG00000124222	STX16	20:57226327-57294294	q1	q2	OK	25.5821	26.4233	0.0466766	0.0674663	0.8945	0.99995	no

ENSG00000124224	ENSG00000124224	PPP4R1L	20:56806187-56884495	q1	q2	OK	5.35532	6.36167	0.248431	0.296746	0.5924	0.99995	no

ENSG00000124225	ENSG00000124225	PMEPA1	20:56223447-56286592	q1	q2	OK	8.80467	8.28769	-0.0872983	-0.127063	0.8028	0.99995	no

ENSG00000124226	ENSG00000124226	RNF114	20:48552947-48570429	q1	q2	OK	29.6035	29.8527	0.0120929	0.0286092	0.9682	0.99995	no

ENSG00000124227	ENSG00000124227	ANKRD60	20:56793550-56803709	q1	q2	NOTEST	0	0	0	0	1	1	no

ENSG00000124228	ENSG00000124228	DDX27	20:47835883-47905797	q1	q2	OK	18.9867	18.9977	0.000837208	0.0011497	0.99885	0.99995	no

ENSG00000124232	ENSG00000124232	RBPJL	20:43922084-43945803	q1	q2	NOTEST	0	0	0	0	1	1	no

ENSG00000124233	ENSG00000124233	SEMG1	20:43835637-43838413	q1	q2	NOTEST	0.137113	0.190753	0.476343	0	1	1	no

ENSG00000124237	ENSG00000124237	C20orf85	20:56725959-56736206	q1	q2	NOTEST	0	0	0	0	1	1	no

ENSG00000124243	ENSG00000124243	BCAS4	20:49411430-49493714	q1	q2	OK	9.27701	7.1079	-0.384237	-0.372784	0.52235	0.99995	no

ENSG00000124249	ENSG00000124249	KCNK15	20:43285091-43379675	q1	q2	NOTEST	0	0	0	0	1	1	no

ENSG00000124251	ENSG00000124251	TP53TG5	20:43990576-44039250	q1	q2	NOTEST	0.295809	0.285082	-0.0532904	0	1	1	no

ENSG00000124253	ENSG00000124253	PCK1	20:56136135-56141513	q1	q2	NOTEST	0	0	0	0	1	1	no

ENSG00000124256	ENSG00000124256	ZBP1	20:56178901-56195632	q1	q2	NOTEST	0.0605858	0.0717644	0.244287	0	1	1	no

ENSG00000124257	ENSG00000124257	NEURL2	20:44517263-44540794	q1	q2	OK	2.17948	1.51873	-0.521118	-0.14687	0.80325	0.99995	no

ENSG00000124260	ENSG00000124260	MAGEA10	X:151282525-151307033	q1	q2	NOTEST	0	0	0	0	1	1	no

ENSG00000124275	ENSG00000124275	MTRR	5:7830490-7906138	q1	q2	OK	16.7048	16.9864	0.024113	0.0288513	0.9459	0.99995	no

ENSG00000124279	ENSG00000124279	FASTKD3	5:7830490-7906138	q1	q2	OK	7.64226	9.15346	0.260318	0.219218	0.68945	0.99995	no

ENSG00000124299	ENSG00000124299	PEPD	19:33877855-34012700	q1	q2	OK	29.3022	27.3826	-0.0977525	-0.186184	0.77145	0.99995	no

ENSG00000124302	ENSG00000124302	CHST8	19:34112860-34264414	q1	q2	OK	0.35042	0.493555	0.494125	0.417574	0.46965	0.99995	no

ENSG00000124313	ENSG00000124313	IQSEC2	X:53262057-53350522	q1	q2	NOTEST	0	0	0	0	1	1	no

ENSG00000124333	ENSG00000124333	VAMP7	X:155110955-155173433	q1	q2	OK	15.4435	17.157	0.1518	0.291236	0.6358	0.99995	no

ENSG00000124334	ENSG00000124334	IL9R	X:155227245-155257848	q1	q2	NOTEST	0.0143503	0	#NAME?	0	1	1	no

ENSG00000124343	ENSG00000124343	XG	X:2670090-2734539	q1	q2	NOTEST	0.0112192	0.0315771	1.49291	0	1	1	no

ENSG00000124356	ENSG00000124356	STAMBP	2:74056085-74100786	q1	q2	OK	20.8429	20.0689	-0.0545956	-0.0959479	0.87555	0.99995	no

ENSG00000124357	ENSG00000124357	NAGK	2:71163011-71306935	q1	q2	OK	41.7062	40.4505	-0.0441024	-0.0784719	0.90355	0.99995	no

ENSG00000124370	ENSG00000124370	MCEE	2:71336813-71357369	q1	q2	OK	7.86352	8.58627	0.126857	0.159577	0.81965	0.99995	no

ENSG00000124374	ENSG00000124374	PAIP2B	2:71409868-71454213	q1	q2	OK	1.6725	1.44637	-0.209565	-0.329056	0.63135	0.99995	no

ENSG00000124380	ENSG00000124380	SNRNP27	2:70120691-70170077	q1	q2	OK	24.8208	24.3086	-0.0300832	-0.0450668	0.94775	0.99995	no

ENSG00000124383	ENSG00000124383	MPHOSPH10	2:71357443-71377231	q1	q2	OK	26.2152	27.6908	0.0790024	0.171914	0.79085	0.99995	no

ENSG00000124391	ENSG00000124391	IL17C	16:88704998-88706881	q1	q2	NOTEST	0.0875563	0	#NAME?	0	1	1	no

ENSG00000124399	ENSG00000124399	RP11-663P9.2	4:43900473-43901368	q1	q2	OK	2.9415	4.74531	0.689947	0.677109	0.3503	0.99995	no

ENSG00000124406	ENSG00000124406	ATP8A1	4:42410389-42659122	q1	q2	OK	3.62495	3.55415	-0.0284572	-0.0273465	0.9471	0.99995	no

ENSG00000124422	ENSG00000124422	USP22	17:20717932-20947073	q1	q2	OK	112.148	112.254	0.00136378	0.00217121	0.99615	0.99995	no

ENSG00000124429	ENSG00000124429	POF1B	X:84532401-84634748	q1	q2	NOTEST	0.026057	0.0452712	0.796925	0	1	1	no

ENSG00000124440	ENSG00000124440	HIF3A	19:46800302-46846690	q1	q2	OK	4.989	4.67233	-0.0946097	-0.059765	0.85925	0.99995	no

ENSG00000124444	ENSG00000124444	ZNF576	19:44100543-44124026	q1	q2	OK	6.86944	6.52629	-0.0739293	-0.0499469	0.9258	0.99995	no

ENSG00000124449	ENSG00000124449	IRGC	19:44220161-44224173	q1	q2	NOTEST	0.0171216	0	#NAME?	0	1	1	no

ENSG00000124459	ENSG00000124459	ZNF45	19:44406152-44439430	q1	q2	OK	10.9644	10.4898	-0.063835	-0.104817	0.85215	0.99995	no

ENSG00000124466	ENSG00000124466	LYPD3	19:43964938-43969812	q1	q2	NOTEST	0.350721	0.213986	-0.712807	0	1	1	no

ENSG00000124467	ENSG00000124467	PSG8	19:43256837-43359870	q1	q2	NOTEST	0	0	0	0	1	1	no

ENSG00000124469	ENSG00000124469	CEACAM8	19:42901279-43156507	q1	q2	NOTEST	0.0344341	0.0682684	0.987378	0	1	1	no

ENSG00000124479	ENSG00000124479	NDP	X:43808021-43832750	q1	q2	OK	1.1178	0.899446	-0.313556	-0.305459	0.62315	0.99995	no

ENSG00000124486	ENSG00000124486	USP9X	X:40944887-41095832	q1	q2	OK	34.0247	35.9005	0.0774215	0.101921	0.79805	0.99995	no

ENSG00000124490	ENSG00000124490	CRISP2	6:49660072-49681274	q1	q2	NOTEST	0	0	0	0	1	1	no

ENSG00000124491	ENSG00000124491	F13A1	6:6144317-6321246	q1	q2	NOTEST	0.0565521	0.144886	1.35726	0	1	1	no

ENSG00000124493	ENSG00000124493	GRM4	6:33989627-34123399	q1	q2	NOTEST	0.114998	0.157261	0.451555	0	1	1	no

ENSG00000124496	ENSG00000124496	TRERF1	6:42192668-42419789	q1	q2	OK	6.87364	6.73238	-0.0299566	-0.0674689	0.9216	0.99995	no

ENSG00000124507	ENSG00000124507	PACSIN1	6:34433915-34503006	q1	q2	NOTEST	0.107073	0.0455908	-1.23178	0	1	1	no

ENSG00000124508	ENSG00000124508	BTN2A2	6:26383323-26395102	q1	q2	OK	11.0643	11.7657	0.0886706	0.139494	0.8059	0.99995	no

ENSG00000124523	ENSG00000124523	SIRT5	6:13574815-13615387	q1	q2	OK	6.80119	6.16674	-0.141279	-0.207919	0.7649	0.99995	no

ENSG00000124529	ENSG00000124529	HIST1H4B	6:26027123-26027480	q1	q2	NOTEST	0.157245	0	#NAME?	0	1	1	no

ENSG00000124532	ENSG00000124532	MRS2	6:24403152-24537435	q1	q2	OK	22.8118	23.5224	0.0442558	0.0772626	0.91695	0.99995	no

ENSG00000124535	ENSG00000124535	WRNIP1	6:2765647-2787186	q1	q2	OK	23.3743	23.8246	0.0275276	0.057535	0.93575	0.99995	no

ENSG00000124541	ENSG00000124541	RRP36	6:42989382-43002143	q1	q2	OK	45.4625	45.1911	-0.00863751	-0.0187365	0.97885	0.99995	no

ENSG00000124549	ENSG00000124549	BTN2A3P	6:26421618-26432611	q1	q2	OK	3.54759	3.70703	0.0634266	0.0822278	0.8979	0.99995	no

ENSG00000124557	ENSG00000124557	BTN1A1	6:26501448-26510650	q1	q2	NOTEST	0.00880491	0	#NAME?	0	1	1	no

ENSG00000124562	ENSG00000124562	SNRPC	6:34725182-34741571	q1	q2	OK	121.046	122.706	0.019642	0.0466279	0.94585	0.99995	no

ENSG00000124564	ENSG00000124564	SLC17A3	6:25833293-25882623	q1	q2	NOTEST	0.0457216	0.151586	1.72919	0	1	1	no

ENSG00000124568	ENSG00000124568	SLC17A1	6:25783124-25832287	q1	q2	NOTEST	0.015778	0	#NAME?	0	1	1	no

ENSG00000124570	ENSG00000124570	SERPINB6	6:2948392-2972090	q1	q2	OK	17.5662	15.9125	-0.142645	-0.255468	0.71145	0.99995	no

ENSG00000124571	ENSG00000124571	XPO5	6:43474706-43543812	q1	q2	OK	29.4099	30.4218	0.0488036	0.0568181	0.9075	0.99995	no

ENSG00000124574	ENSG00000124574	ABCC10	6:43395103-43424370	q1	q2	OK	10.5081	8.68083	-0.275598	-0.254226	0.5219	0.99995	no

ENSG00000124575	ENSG00000124575	HIST1H1D	6:26234439-26235216	q1	q2	NOTEST	0.166874	0.120988	-0.463896	0	1	1	no

ENSG00000124578	ENSG00000124578	HIST1H4G	6:26246885-26247259	q1	q2	NOTEST	0	0	0	0	1	1	no

ENSG00000124587	ENSG00000124587	PEX6	6:42928495-42946958	q1	q2	OK	3.23171	2.65309	-0.284626	-0.434981	0.53325	0.99995	no

ENSG00000124588	ENSG00000124588	NQO2	6:2988200-3019996	q1	q2	OK	14.6468	15.5662	0.0878268	0.130328	0.8504	0.99995	no

ENSG00000124593	ENSG00000124593	PRICKLE4	6:41737913-41863099	q1	q2	OK	10.5606	7.32702	-0.527387	-0.257356	0.58895	0.99995	no

ENSG00000124596	ENSG00000124596	OARD1	6:40994771-41067715	q1	q2	OK	35.8356	35.3066	-0.0214567	-0.022051	0.9706	0.99995	no

ENSG00000124602	ENSG00000124602	UNC5CL	6:40994771-41067715	q1	q2	OK	0.943208	0.915517	-0.0429904	-0.011117	0.9812	0.99995	no

ENSG00000124608	ENSG00000124608	AARS2	6:44238202-44346694	q1	q2	OK	9.1346	8.43768	-0.114495	-0.128533	0.8346	0.99995	no

ENSG00000124610	ENSG00000124610	HIST1H1A	6:26017259-26018040	q1	q2	NOTEST	0	0	0	0	1	1	no

ENSG00000124613	ENSG00000124613	ZNF391	6:27342393-27371683	q1	q2	OK	4.03738	4.16164	0.0437325	0.0675934	0.9126	0.99995	no

ENSG00000124614	ENSG00000124614	RPS10	6:34247455-34393902	q1	q2	OK	708.128	665.14	-0.090353	-0.154833	0.8216	0.99995	no

ENSG00000124615	ENSG00000124615	MOCS1	6:39760141-39902290	q1	q2	OK	5.24526	5.30382	0.0160183	0.0201681	0.97665	0.99995	no

ENSG00000124635	ENSG00000124635	HIST1H2BJ	6:27093675-27100541	q1	q2	OK	0.97974	1.00299	0.0338329	0.0226161	0.97325	0.99995	no

ENSG00000124641	ENSG00000124641	MED20	6:41873091-41888877	q1	q2	OK	17.3994	15.8133	-0.137897	-0.266388	0.69815	0.99995	no

ENSG00000124657	ENSG00000124657	OR2B6	6:27925018-27925960	q1	q2	NOTEST	0.161719	0.161939	0.00196738	0	1	1	no

ENSG00000124659	ENSG00000124659	TBCC	6:42712218-42714558	q1	q2	OK	18.9276	17.9382	-0.0774554	-0.156105	0.8243	0.99995	no

ENSG00000124664	ENSG00000124664	SPDEF	6:34505578-34524110	q1	q2	NOTEST	0.0838545	0.0995305	0.24725	0	1	1	no

ENSG00000124678	ENSG00000124678	TCP11	6:35085847-35116387	q1	q2	NOTEST	0.123775	0	#NAME?	0	1	1	no

ENSG00000124688	ENSG00000124688	MAD2L1BP	6:43597276-43608689	q1	q2	OK	23.3989	24.4718	0.064676	0.122697	0.85645	0.99995	no

ENSG00000124693	ENSG00000124693	HIST1H3B	6:26031816-26032288	q1	q2	NOTEST	0.358797	0.173233	-1.05045	0	1	1	no

ENSG00000124701	ENSG00000124701	APOBEC2	6:40994771-41067715	q1	q2	NOTEST	0.207956	0.176507	-0.236555	0	1	1	no

ENSG00000124702	ENSG00000124702	KLHDC3	6:42981950-42989036	q1	q2	OK	64.4194	57.4654	-0.164801	-0.402397	0.5631	0.99995	no

ENSG00000124713	ENSG00000124713	GNMT	6:42928495-42946958	q1	q2	OK	1.17492	1.34099	0.190734	0.0947086	0.89665	0.99995	no

ENSG00000124721	ENSG00000124721	DNAH8	6:38683116-38998301	q1	q2	NOTEST	0.0209065	0.128005	2.61417	0	1	1	no

ENSG00000124731	ENSG00000124731	TREM1	6:41235663-41254457	q1	q2	NOTEST	0	0	0	0	1	1	no

ENSG00000124733	ENSG00000124733	MEA1	6:42952236-42981706	q1	q2	OK	48.9653	47.1606	-0.054178	-0.0764558	0.91165	0.99995	no

ENSG00000124743	ENSG00000124743	KLHL31	6:53512698-53530506	q1	q2	NOTEST	0.149236	0.173322	0.215861	0	1	1	no

ENSG00000124749	ENSG00000124749	COL21A1	6:55921387-56258892	q1	q2	OK	3.81946	3.7408	-0.0300239	-0.0362906	0.9492	0.99995	no

ENSG00000124762	ENSG00000124762	CDKN1A	6:36644304-36655116	q1	q2	OK	59.1621	37.0579	-0.674894	-1.47712	0.024	0.99995	no

ENSG00000124766	ENSG00000124766	SOX4	6:21593971-21598847	q1	q2	OK	82.3951	76.1184	-0.114312	-0.275029	0.6906	0.99995	no

ENSG00000124767	ENSG00000124767	GLO1	6:38643700-38670917	q1	q2	OK	145.739	156.464	0.102449	0.252073	0.7187	0.99995	no

ENSG00000124772	ENSG00000124772	CPNE5	6:36708551-36807778	q1	q2	OK	5.53629	5.62108	0.0219276	0.0365301	0.95765	0.99995	no

ENSG00000124780	ENSG00000124780	KCNK17	6:39266776-39282329	q1	q2	NOTEST	0	0	0	0	1	1	no

ENSG00000124782	ENSG00000124782	RREB1	6:7107829-7252213	q1	q2	OK	4.88218	4.98545	0.0301999	0.0376706	0.92905	0.99995	no

ENSG00000124783	ENSG00000124783	SSR1	6:7268538-7418270	q1	q2	OK	59.6688	59.6834	0.00035377	0.000661476	0.9993	0.99995	no

ENSG00000124784	ENSG00000124784	RIOK1	6:7268538-7418270	q1	q2	OK	10.5967	13.3458	0.332765	0.184099	0.67165	0.99995	no

ENSG00000124785	ENSG00000124785	NRN1	6:5998231-6007200	q1	q2	OK	1.59025	1.85254	0.220257	0.239159	0.72155	0.99995	no

ENSG00000124786	ENSG00000124786	SLC35B3	6:8413300-8435794	q1	q2	OK	10.5937	11.1453	0.0732399	0.132856	0.8558	0.99995	no

ENSG00000124787	ENSG00000124787	RPP40	6:4994965-5004297	q1	q2	OK	7.80023	9.4327	0.274154	0.343864	0.62285	0.99995	no

ENSG00000124788	ENSG00000124788	ATXN1	6:16299342-16762883	q1	q2	OK	2.36545	2.57144	0.120465	0.123349	0.76075	0.99995	no

ENSG00000124789	ENSG00000124789	NUP153	6:17615268-17707575	q1	q2	OK	27.3376	29.7285	0.120964	0.293488	0.67965	0.99995	no

ENSG00000124795	ENSG00000124795	DEK	6:18224098-18265054	q1	q2	OK	142.369	155.515	0.127411	0.262215	0.66815	0.99995	no

ENSG00000124802	ENSG00000124802	EEF1E1	6:7727029-8102811	q1	q2	OK	31.481	37.4801	0.251643	0.165332	0.7988	0.99995	no

ENSG00000124812	ENSG00000124812	CRISP1	6:49801969-49844809	q1	q2	NOTEST	0	0	0	0	1	1	no

ENSG00000124813	ENSG00000124813	RUNX2	6:44777053-45632086	q1	q2	OK	1.70408	2.38921	0.48754	0.287201	0.62855	0.99995	no

ENSG00000124818	ENSG00000124818	OPN5	6:47749717-47800516	q1	q2	NOTEST	0.1129	0.201486	0.835637	0	1	1	no

ENSG00000124827	ENSG00000124827	GCM2	6:10747991-10979553	q1	q2	NOTEST	0.0105888	0.0110083	0.0560572	0	1	1	no

ENSG00000124831	ENSG00000124831	LRRFIP1	2:238536218-238751451	q1	q2	OK	29.0717	30.3082	0.0600923	0.103866	0.85895	0.99995	no

ENSG00000124835	ENSG00000124835	AC105760.3	2:237957241-237964810	q1	q2	NOTEST	0.0123201	0.0234757	0.930157	0	1	1	no

ENSG00000124839	ENSG00000124839	RAB17	2:238482964-238510257	q1	q2	NOTEST	0.328471	0.269984	-0.282895	0	1	1	no

ENSG00000124875	ENSG00000124875	CXCL6	4:74702213-74714781	q1	q2	OK	3.35628	2.60183	-0.36734	-0.438122	0.5088	0.99995	no

ENSG00000124882	ENSG00000124882	EREG	4:75230859-75254468	q1	q2	NOTEST	0.0214789	0.0103911	-1.04757	0	1	1	no

ENSG00000124900	ENSG00000124900	TRIM51	11:55650772-55659286	q1	q2	NOTEST	0	0	0	0	1	1	no

ENSG00000124915	ENSG00000124915	RP11-467L20.10	11:61447904-61634826	q1	q2	NOTEST	0	0.0229882	inf	0	1	1	no

ENSG00000124920	ENSG00000124920	MYRF	11:61447904-61634826	q1	q2	OK	6.2935	5.88732	-0.0962515	-0.024028	0.91835	0.99995	no

ENSG00000124935	ENSG00000124935	SCGB1D2	11:61976139-62028021	q1	q2	NOTEST	0	0	0	0	1	1	no

ENSG00000124939	ENSG00000124939	SCGB2A1	11:61976139-62028021	q1	q2	NOTEST	0	0	0	0	1	1	no

ENSG00000124942	ENSG00000124942	AHNAK	11:62201015-62323707	q1	q2	OK	10.9041	12.2574	0.168794	0.175055	0.6046	0.99995	no

ENSG00000125037	ENSG00000125037	EMC3	3:10000400-10052893	q1	q2	OK	29.2008	27.3066	-0.0967563	-0.10838	0.8814	0.99995	no

ENSG00000125046	ENSG00000125046	SSUH2	3:8661085-9005457	q1	q2	OK	7.31976	6.02365	-0.281158	-0.198235	0.7414	0.99995	no

ENSG00000125084	ENSG00000125084	WNT1	12:49372397-49375459	q1	q2	NOTEST	0	0	0	0	1	1	no

ENSG00000125089	ENSG00000125089	SH3TC1	4:8183798-8243530	q1	q2	OK	0.674955	0.564691	-0.257329	-0.123316	0.6274	0.99995	no

ENSG00000125107	ENSG00000125107	CNOT1	16:58549382-58663790	q1	q2	OK	65.6498	72.7646	0.148447	0.172845	0.6817	0.99995	no

ENSG00000125122	ENSG00000125122	LRRC29	16:67241041-67260951	q1	q2	OK	1.58972	1.20324	-0.401854	-0.40609	0.5614	0.99995	no

ENSG00000125124	ENSG00000125124	BBS2	16:56463044-56554195	q1	q2	OK	32.969	30.7231	-0.101786	-0.0597386	0.8765	0.99995	no

ENSG00000125144	ENSG00000125144	MT1G	16:56700642-56701977	q1	q2	OK	2.15714	1.32285	-0.705465	-0.535033	0.42735	0.99995	no

ENSG00000125148	ENSG00000125148	MT2A	16:56642110-56643409	q1	q2	OK	9.77091	7.26046	-0.428432	-0.568574	0.40525	0.99995	no

ENSG00000125149	ENSG00000125149	C16orf70	16:67143860-67185117	q1	q2	OK	11.8823	12.2393	0.042705	0.0674279	0.90225	0.99995	no

ENSG00000125166	ENSG00000125166	GOT2	16:58741034-59142878	q1	q2	OK	56.3418	55.9154	-0.0109594	-0.0262523	0.96955	0.99995	no

ENSG00000125170	ENSG00000125170	DOK4	16:57462080-57521239	q1	q2	OK	9.70307	9.39187	-0.0470296	-0.0349832	0.95645	0.99995	no

ENSG00000125207	ENSG00000125207	PIWIL1	12:130822431-130857182	q1	q2	NOTEST	0	0	0	0	1	1	no

ENSG00000125245	ENSG00000125245	GPR18	13:99853027-100038688	q1	q2	NOTEST	0.133057	0.0959146	-0.472228	0	1	1	no

ENSG00000125246	ENSG00000125246	CLYBL	13:100258918-100549387	q1	q2	OK	3.47932	3.33972	-0.0590784	-0.0619094	0.9243	0.99995	no

ENSG00000125247	ENSG00000125247	TMTC4	13:101256180-101327347	q1	q2	OK	31.6389	31.3348	-0.0139345	-0.0260432	0.9645	0.99995	no

ENSG00000125249	ENSG00000125249	RAP2A	13:98086475-98121382	q1	q2	OK	14.6297	16.1108	0.139127	0.325184	0.6316	0.99995	no

ENSG00000125255	ENSG00000125255	SLC10A2	13:103696349-103719196	q1	q2	NOTEST	0.013626	0	#NAME?	0	1	1	no

ENSG00000125257	ENSG00000125257	ABCC4	13:95672082-95953687	q1	q2	OK	3.40978	3.22927	-0.078471	-0.124109	0.8291	0.99995	no

ENSG00000125266	ENSG00000125266	EFNB2	13:107142078-107187462	q1	q2	OK	64.0204	75.0292	0.228922	0.556021	0.4241	0.99995	no

ENSG00000125285	ENSG00000125285	SOX21	13:95361885-95364389	q1	q2	OK	42.7861	36.2219	-0.240281	-0.574413	0.40895	0.99995	no

ENSG00000125304	ENSG00000125304	TM9SF2	13:100153670-100216260	q1	q2	OK	50.0481	51.9173	0.0529007	0.123103	0.8477	0.99995	no

ENSG00000125319	ENSG00000125319	C17orf53	17:42219273-42239844	q1	q2	OK	5.4399	5.24854	-0.0516653	-0.0733836	0.9124	0.99995	no

ENSG00000125337	ENSG00000125337	KIF25	6:168393501-168445769	q1	q2	NOTEST	0.104489	0.183192	0.810005	0	1	1	no

ENSG00000125347	ENSG00000125347	IRF1	5:131817300-131826490	q1	q2	OK	2.46854	1.93307	-0.352764	-0.352804	0.5291	0.99995	no

ENSG00000125351	ENSG00000125351	UPF3B	X:118967984-118986961	q1	q2	OK	28.1897	26.0595	-0.113358	-0.263869	0.70175	0.99995	no

ENSG00000125352	ENSG00000125352	RNF113A	X:119004496-119010625	q1	q2	OK	8.9679	8.44672	-0.0863786	-0.0861491	0.9003	0.99995	no

ENSG00000125354	ENSG00000125354	Sep-06	X:118749686-118827333	q1	q2	OK	39.1863	39.7109	0.0191845	0.0423057	0.9487	0.99995	no

ENSG00000125355	ENSG00000125355	TMEM255A	X:119392504-119445411	q1	q2	NOTEST	0	0.0153062	inf	0	1	1	no

ENSG00000125356	ENSG00000125356	NDUFA1	X:119004496-119010625	q1	q2	OK	65.4477	63.4912	-0.0437859	-0.0870705	0.8983	0.99995	no

ENSG00000125363	ENSG00000125363	AMELX	X:11129420-11683821	q1	q2	NOTEST	0	0	0	0	1	1	no

ENSG00000125375	ENSG00000125375	ATP5S	14:50704280-50883179	q1	q2	OK	7.92544	8.29544	0.0658281	0.0748002	0.89735	0.99995	no

ENSG00000125378	ENSG00000125378	BMP4	14:54416453-54425479	q1	q2	OK	4.09952	4.1019	0.000839891	0.000982012	0.99885	0.99995	no

ENSG00000125384	ENSG00000125384	PTGER2	14:52781022-52795324	q1	q2	NOTEST	0.0879597	0.0678054	-0.375443	0	1	1	no

ENSG00000125385	ENSG00000125385	RP11-297B17.2	9:37046831-37047234	q1	q2	NOTEST	0	0	0	0	1	1	no

ENSG00000125386	ENSG00000125386	FAM193A	4:2420700-2734292	q1	q2	OK	20.5839	18.2301	-0.175197	-0.163652	0.7187	0.99995	no

ENSG00000125388	ENSG00000125388	GRK4	4:2965334-3042474	q1	q2	OK	4.6934	3.87859	-0.275103	-0.291601	0.5932	0.99995	no

ENSG00000125398	ENSG00000125398	SOX9	17:70017991-70216859	q1	q2	OK	23.793	23.6857	-0.00652102	-0.01541	0.9815	0.99995	no

ENSG00000125409	ENSG00000125409	TEKT3	17:15207127-15244958	q1	q2	OK	0.52167	1.17775	1.17482	0.726825	0.15275	0.99995	no

ENSG00000125414	ENSG00000125414	MYH2	17:10286448-10527203	q1	q2	NOTEST	0.23814	0.0865084	-1.4609	0	1	1	no

ENSG00000125430	ENSG00000125430	HS3ST3B1	17:14204399-14252721	q1	q2	OK	15.2809	16.182	0.0826591	0.185134	0.797	0.99995	no

ENSG00000125434	ENSG00000125434	SLC25A35	17:8191080-8198661	q1	q2	OK	1.29023	1.17492	-0.135073	-0.0912857	0.8919	0.99995	no

ENSG00000125445	ENSG00000125445	MRPS7	17:73232693-73267308	q1	q2	OK	55.1292	51.4103	-0.100759	-0.147781	0.82425	0.99995	no

ENSG00000125447	ENSG00000125447	GGA3	17:73232693-73267308	q1	q2	OK	21.036	20.1784	-0.0600535	-0.0581081	0.88825	0.99995	no

ENSG00000125449	ENSG00000125449	ARMC7	17:73106046-73127890	q1	q2	OK	9.38113	8.70336	-0.108189	-0.144107	0.83045	0.99995	no

ENSG00000125450	ENSG00000125450	NUP85	17:73201753-73231853	q1	q2	OK	35.3647	38.4104	0.119184	0.210446	0.6808	0.99995	no

ENSG00000125454	ENSG00000125454	SLC25A19	17:73267449-73285591	q1	q2	OK	4.29483	3.45945	-0.312059	-0.334156	0.57275	0.99995	no

ENSG00000125457	ENSG00000125457	MIF4GD	17:73232693-73267308	q1	q2	OK	10.9851	9.47829	-0.21285	-0.120072	0.799	0.99995	no

ENSG00000125458	ENSG00000125458	NT5C	17:73106046-73127890	q1	q2	OK	38.0571	32.6866	-0.219469	-0.342045	0.61775	0.99995	no

ENSG00000125459	ENSG00000125459	MSTO1	1:155579566-155829191	q1	q2	OK	17.2996	15.4434	-0.163753	-0.0805661	0.81285	0.99995	no

ENSG00000125462	ENSG00000125462	C1orf61	1:156374041-156400493	q1	q2	OK	8.64668	5.1482	-0.748079	-0.642185	0.3406	0.99995	no

ENSG00000125482	ENSG00000125482	TTF1	9:135251007-135282209	q1	q2	OK	14.9254	17.3671	0.21859	0.419641	0.4983	0.99995	no

ENSG00000125484	ENSG00000125484	GTF3C4	9:135468383-135570342	q1	q2	OK	17.1775	18.262	0.0883203	0.204026	0.7762	0.99995	no

ENSG00000125485	ENSG00000125485	DDX31	9:135468383-135570342	q1	q2	OK	7.79371	7.83973	0.00849282	0.00639852	0.99395	0.99995	no

ENSG00000125492	ENSG00000125492	BARHL1	9:135457571-135465653	q1	q2	NOTEST	0.146778	0.0578975	-1.34207	0	1	1	no

ENSG00000125498	ENSG00000125498	KIR2DL1	19:55235968-55378662	q1	q2	NOTEST	0	0	0	0	1	1	no

ENSG00000125503	ENSG00000125503	PPP1R12C	19:55602280-55628927	q1	q2	OK	45.4019	39.4493	-0.202751	-0.363855	0.57	0.99995	no

ENSG00000125505	ENSG00000125505	MBOAT7	19:54677106-54693733	q1	q2	NOTEST	0	0	0	0	1	1	no

ENSG00000125508	ENSG00000125508	SRMS	20:62172162-62178857	q1	q2	NOTEST	0.0180323	0	#NAME?	0	1	1	no

ENSG00000125510	ENSG00000125510	OPRL1	20:62711525-62731996	q1	q2	OK	1.24532	0.922597	-0.432743	-0.514665	0.4558	0.99995	no

ENSG00000125514	ENSG00000125514	LINC00029	20:61640734-61716423	q1	q2	NOTEST	0	0.0344501	inf	0	1	1	no

ENSG00000125520	ENSG00000125520	SLC2A4RG	20:62289162-62374858	q1	q2	OK	31.3524	30.7089	-0.02992	-0.0275186	0.95785	0.99995	no

ENSG00000125522	ENSG00000125522	NPBWR2	20:62737172-62738524	q1	q2	NOTEST	0	0	0	0	1	1	no

ENSG00000125531	ENSG00000125531	C20orf195	20:62184372-62188061	q1	q2	OK	1.28117	1.28025	-0.0010431	-0.00103788	0.9842	0.99995	no

ENSG00000125533	ENSG00000125533	BHLHE23	20:61637330-61638387	q1	q2	NOTEST	0.113858	0.0540308	-1.07538	0	1	1	no

ENSG00000125534	ENSG00000125534	PPDPF	20:62152076-62153559	q1	q2	OK	142.468	122.336	-0.219785	-0.514174	0.4429	0.99995	no

ENSG00000125538	ENSG00000125538	IL1B	2:113587327-113594480	q1	q2	NOTEST	0.0894779	0.0172244	-2.37707	0	1	1	no

ENSG00000125551	ENSG00000125551	PLGLB2	2:88047605-88285309	q1	q2	OK	0.727977	0.563303	-0.369982	-0.218011	0.75085	0.99995	no

ENSG00000125571	ENSG00000125571	IL37	2:113670547-113676459	q1	q2	NOTEST	0.0610004	0	#NAME?	0	1	1	no

ENSG00000125611	ENSG00000125611	CHCHD5	2:113341816-113372008	q1	q2	OK	21.128	18.1376	-0.220171	-0.346402	0.62325	0.99995	no

ENSG00000125618	ENSG00000125618	PAX8	2:113969098-114036527	q1	q2	OK	2.73279	4.12202	0.592975	0.479382	0.3478	0.99995	no

ENSG00000125629	ENSG00000125629	INSIG2	2:118846027-118868573	q1	q2	OK	12.074	12.7916	0.0832937	0.129327	0.83255	0.99995	no

ENSG00000125630	ENSG00000125630	POLR1B	2:113299491-113334635	q1	q2	OK	12.2306	13.7807	0.172151	0.224107	0.65225	0.99995	no

ENSG00000125631	ENSG00000125631	HTR5BP	2:118617002-118771709	q1	q2	NOTEST	0.0504498	0.101202	1.00432	0	1	1	no

ENSG00000125633	ENSG00000125633	CCDC93	2:118617002-118771709	q1	q2	OK	27.7195	30.7355	0.149004	0.141966	0.7627	0.99995	no

ENSG00000125637	ENSG00000125637	PSD4	2:113914901-113966973	q1	q2	OK	0.621627	0.563181	-0.142449	-0.100415	0.8194	0.99995	no

ENSG00000125648	ENSG00000125648	SLC25A23	19:6436089-6482568	q1	q2	OK	27.765	26.2119	-0.083048	-0.152734	0.7957	0.99995	no

ENSG00000125650	ENSG00000125650	PSPN	19:6375158-6379069	q1	q2	OK	1.07784	1.03734	-0.0552576	-0.0657328	0.92025	0.99995	no

ENSG00000125651	ENSG00000125651	GTF2F1	19:6379579-6412415	q1	q2	OK	37.2201	38.4187	0.0457234	0.0865993	0.89055	0.99995	no

ENSG00000125652	ENSG00000125652	ALKBH7	19:6372443-6375042	q1	q2	OK	29.0905	24.4602	-0.250114	-0.452594	0.5231	0.99995	no

ENSG00000125656	ENSG00000125656	CLPP	19:6343771-6368919	q1	q2	OK	32.2041	30.8497	-0.0619881	-0.124457	0.86275	0.99995	no

ENSG00000125657	ENSG00000125657	TNFSF9	19:6531009-6535931	q1	q2	OK	2.31771	1.68009	-0.46416	-0.547076	0.4408	0.99995	no

ENSG00000125675	ENSG00000125675	GRIA3	X:122318005-122624766	q1	q2	OK	1.90338	1.60442	-0.246518	-0.195049	0.68855	0.99995	no

ENSG00000125676	ENSG00000125676	THOC2	X:122734411-122866906	q1	q2	OK	45.2284	47.0371	0.056572	0.0832608	0.8726	0.99995	no

ENSG00000125686	ENSG00000125686	MED1	17:37415383-37607539	q1	q2	OK	24.104	26.6392	0.144277	0.273877	0.6724	0.99995	no

ENSG00000125691	ENSG00000125691	RPL23	17:37004117-37010096	q1	q2	OK	939.62	921.62	-0.0279056	-0.064424	0.9281	0.99995	no

ENSG00000125695	ENSG00000125695	RP11-51F16.8	17:61780191-61896677	q1	q2	NOTEST	0	0.1036	inf	0	1	1	no

ENSG00000125703	ENSG00000125703	ATG4C	1:63249805-63331184	q1	q2	OK	5.99755	7.32519	0.288492	0.471808	0.4513	0.99995	no

ENSG00000125726	ENSG00000125726	CD70	19:6583193-6604114	q1	q2	OK	0.543392	0.365057	-0.573871	-0.386336	0.5199	0.99995	no

ENSG00000125730	ENSG00000125730	C3	19:6677714-6737614	q1	q2	NOTEST	0.00468771	0	#NAME?	0	1	1	no

ENSG00000125731	ENSG00000125731	SH2D3A	19:6752170-6767599	q1	q2	NOTEST	0.0743663	0.26036	1.80778	0	1	1	no

ENSG00000125733	ENSG00000125733	TRIP10	19:6737935-6751537	q1	q2	OK	12.8573	11.6219	-0.145737	-0.203774	0.7157	0.99995	no

ENSG00000125734	ENSG00000125734	GPR108	19:6677714-6737614	q1	q2	OK	21.0285	18.2244	-0.206472	-0.300514	0.6043	0.99995	no

ENSG00000125735	ENSG00000125735	TNFSF14	19:6663147-6670599	q1	q2	NOTEST	0	0	0	0	1	1	no

ENSG00000125740	ENSG00000125740	FOSB	19:45882891-45982086	q1	q2	NOTEST	0.286067	0.203547	-0.490992	0	1	1	no

ENSG00000125741	ENSG00000125741	OPA3	19:46030684-46105470	q1	q2	OK	5.71722	5.56438	-0.0390937	-0.0630646	0.92795	0.99995	no

ENSG00000125743	ENSG00000125743	SNRPD2	19:46190711-46207247	q1	q2	OK	309.02	282.651	-0.128677	-0.262028	0.701	0.99995	no

ENSG00000125744	ENSG00000125744	RTN2	19:45988546-46005768	q1	q2	OK	14.2717	11.7682	-0.278268	-0.396033	0.4772	0.99995	no

ENSG00000125746	ENSG00000125746	EML2	19:46110251-46148887	q1	q2	OK	4.3804	3.30027	-0.408479	-0.337621	0.52865	0.99995	no

ENSG00000125753	ENSG00000125753	VASP	19:46009836-46030241	q1	q2	OK	19.0567	19.2431	0.0140436	0.0267573	0.9636	0.99995	no

ENSG00000125755	ENSG00000125755	SYMPK	19:46318667-46366548	q1	q2	OK	30.3624	25.3175	-0.262151	-0.455912	0.4415	0.99995	no

ENSG00000125772	ENSG00000125772	GPCPD1	20:5525084-5591672	q1	q2	OK	17.0621	17.2465	0.0155083	0.0298938	0.958	0.99995	no

ENSG00000125775	ENSG00000125775	SDCBP2	20:1290618-1373806	q1	q2	OK	0.938066	0.805239	-0.220273	-0.0436377	0.8629	0.99995	no

ENSG00000125779	ENSG00000125779	PANK2	20:3868885-3907605	q1	q2	OK	27.0683	28.5847	0.0786355	0.159303	0.80065	0.99995	no

ENSG00000125780	ENSG00000125780	TGM3	20:2276646-2321724	q1	q2	NOTEST	0.246095	0.155581	-0.661556	0	1	1	no

ENSG00000125787	ENSG00000125787	GNRH2	20:3024267-3026393	q1	q2	NOTEST	0.482436	0.409716	-0.235713	0	1	1	no

ENSG00000125788	ENSG00000125788	DEFB126	20:123009-126392	q1	q2	NOTEST	0	0	0	0	1	1	no

ENSG00000125798	ENSG00000125798	FOXA2	20:22561642-22566093	q1	q2	NOTEST	0.0113553	0	#NAME?	0	1	1	no

ENSG00000125804	ENSG00000125804	FAM182A	20:26035290-26073683	q1	q2	OK	0.962663	0.596317	-0.69095	-0.305665	0.4092	0.99995	no

ENSG00000125810	ENSG00000125810	CD93	20:23059985-23066977	q1	q2	OK	0.713643	1.13232	0.666006	0.936929	0.1717	0.99995	no

ENSG00000125812	ENSG00000125812	GZF1	20:23342786-23353700	q1	q2	OK	9.54983	9.08095	-0.0726322	-0.0871175	0.8432	0.99995	no

ENSG00000125813	ENSG00000125813	PAX1	20:21686296-21696620	q1	q2	NOTEST	0.053971	0.0435753	-0.308675	0	1	1	no

ENSG00000125814	ENSG00000125814	NAPB	20:23355158-23402125	q1	q2	OK	10.7414	10.8334	0.0123114	0.0224558	0.9695	0.99995	no

ENSG00000125815	ENSG00000125815	CST8	20:23471737-23476655	q1	q2	NOTEST	0	0	0	0	1	1	no

ENSG00000125816	ENSG00000125816	NKX2-4	20:21376004-21381029	q1	q2	NOTEST	0	0	0	0	1	1	no

ENSG00000125817	ENSG00000125817	CENPB	20:3764497-3767337	q1	q2	OK	34.6593	31.9783	-0.116149	-0.280893	0.6858	0.99995	no

ENSG00000125818	ENSG00000125818	PSMF1	20:1093905-1160596	q1	q2	OK	40.053	38.7733	-0.0468458	-0.0766607	0.9108	0.99995	no

ENSG00000125820	ENSG00000125820	NKX2-2	20:21491647-21494664	q1	q2	NOTEST	0	0	0	0	1	1	no

ENSG00000125821	ENSG00000125821	DTD1	20:18568536-18744561	q1	q2	OK	57.6914	59.5266	0.0451785	0.107034	0.8778	0.99995	no

ENSG00000125823	ENSG00000125823	CSTL1	20:23420321-23425567	q1	q2	NOTEST	0	0	0	0	1	1	no

ENSG00000125826	ENSG00000125826	RBCK1	20:388141-411610	q1	q2	OK	26.035	21.2359	-0.293943	-0.566834	0.41295	0.99995	no

ENSG00000125827	ENSG00000125827	TMX4	20:7957994-8024159	q1	q2	OK	10.8996	10.7581	-0.0188592	-0.0436774	0.94885	0.99995	no

ENSG00000125831	ENSG00000125831	CST11	20:23431039-23433494	q1	q2	NOTEST	0	0	0	0	1	1	no

ENSG00000125834	ENSG00000125834	STK35	20:2082256-2157684	q1	q2	OK	8.65755	8.78577	0.0212094	0.0488367	0.9429	0.99995	no

ENSG00000125835	ENSG00000125835	SNRPB	20:2442279-2505348	q1	q2	OK	206.311	202.625	-0.0260123	-0.0613022	0.93245	0.99995	no

ENSG00000125841	ENSG00000125841	NRSN2	20:300956-340304	q1	q2	OK	28.5932	24.9392	-0.197259	-0.217961	0.73175	0.99995	no

ENSG00000125843	ENSG00000125843	AP5S1	20:3801177-3805949	q1	q2	OK	5.41522	4.97393	-0.122635	-0.183039	0.78765	0.99995	no

ENSG00000125844	ENSG00000125844	RRBP1	20:17594322-17662940	q1	q2	OK	21.6397	21.1829	-0.0307771	-0.0580051	0.9335	0.99995	no

ENSG00000125845	ENSG00000125845	BMP2	20:6748310-6760927	q1	q2	OK	1.06542	1.19098	0.160721	0.202986	0.7693	0.99995	no

ENSG00000125846	ENSG00000125846	ZNF133	20:18269120-18297640	q1	q2	OK	12.7381	10.4069	-0.291608	-0.457967	0.46675	0.99995	no

ENSG00000125848	ENSG00000125848	FLRT3	20:13829892-16033842	q1	q2	OK	38.9566	40.4006	0.0525088	0.0940734	0.8671	0.99995	no

ENSG00000125850	ENSG00000125850	OVOL2	20:17922240-18039832	q1	q2	NOTEST	0	0	0	0	1	1	no

ENSG00000125851	ENSG00000125851	PCSK2	20:17206751-17465223	q1	q2	OK	4.51465	3.9587	-0.189587	-0.294928	0.66245	0.99995	no

ENSG00000125861	ENSG00000125861	GFRA4	20:3639938-3644046	q1	q2	NOTEST	0	0	0	0	1	1	no

ENSG00000125863	ENSG00000125863	MKKS	20:10381656-10414870	q1	q2	OK	16.9063	16.9697	0.00540178	0.0112957	0.9868	0.99995	no

ENSG00000125864	ENSG00000125864	BFSP1	20:17474549-17549865	q1	q2	OK	1.15841	1.00674	-0.202447	-0.212679	0.7277	0.99995	no

ENSG00000125868	ENSG00000125868	DSTN	20:17550507-17590564	q1	q2	OK	144.709	150.491	0.0565262	0.116229	0.8662	0.99995	no

ENSG00000125869	ENSG00000125869	LAMP5	20:9485826-9511171	q1	q2	OK	4.01009	3.70116	-0.115657	-0.169199	0.80735	0.99995	no

ENSG00000125870	ENSG00000125870	SNRPB2	20:16695488-16722421	q1	q2	OK	60.3069	66.1823	0.134121	0.312853	0.64225	0.99995	no

ENSG00000125871	ENSG00000125871	MGME1	20:17922240-18039832	q1	q2	OK	30.385	31.6278	0.0578324	0.0742455	0.90925	0.99995	no

ENSG00000125872	ENSG00000125872	LRRN4	20:6021423-6034695	q1	q2	NOTEST	0.0396395	0.0990108	1.32065	0	1	1	no

ENSG00000125875	ENSG00000125875	TBC1D20	20:416123-443197	q1	q2	OK	16.2766	15.8968	-0.0340673	-0.0791446	0.90525	0.99995	no

ENSG00000125877	ENSG00000125877	ITPA	20:3189513-3204516	q1	q2	OK	36.6076	32.6736	-0.164019	-0.305683	0.6614	0.99995	no

ENSG00000125878	ENSG00000125878	TCF15	20:584440-591042	q1	q2	NOTEST	0.321975	0.0704187	-2.19292	0	1	1	no

ENSG00000125879	ENSG00000125879	OTOR	20:16729002-16750707	q1	q2	NOTEST	0	0.0361373	inf	0	1	1	no

ENSG00000125885	ENSG00000125885	MCM8	20:5931297-5975852	q1	q2	OK	13.6395	15.8813	0.21954	0.49539	0.483	0.99995	no

ENSG00000125888	ENSG00000125888	BANF2	20:17674316-17716516	q1	q2	NOTEST	0	0	0	0	1	1	no

ENSG00000125895	ENSG00000125895	TMEM74B	20:1161204-1166059	q1	q2	OK	1.92537	1.50176	-0.35848	-0.365953	0.5815	0.99995	no

ENSG00000125898	ENSG00000125898	FAM110A	20:814357-838106	q1	q2	OK	7.06819	6.86543	-0.0419903	-0.0655071	0.9276	0.99995	no

ENSG00000125899	ENSG00000125899	C20orf187	20:11008407-11010014	q1	q2	NOTEST	0	0	0	0	1	1	no

ENSG00000125900	ENSG00000125900	SIRPD	20:1497404-1600707	q1	q2	NOTEST	0	0	0	0	1	1	no

ENSG00000125901	ENSG00000125901	MRPS26	20:3026590-3028900	q1	q2	OK	33.9857	31.8867	-0.0919704	-0.19155	0.7904	0.99995	no

ENSG00000125903	ENSG00000125903	DEFB129	20:207898-210527	q1	q2	NOTEST	0	0	0	0	1	1	no

ENSG00000125910	ENSG00000125910	S1PR4	19:3172343-3180329	q1	q2	NOTEST	0	0.016542	inf	0	1	1	no

ENSG00000125912	ENSG00000125912	NCLN	19:3185560-3209573	q1	q2	OK	20.1686	17.8763	-0.174067	-0.374875	0.5699	0.99995	no

ENSG00000125931	ENSG00000125931	CITED1	X:71401202-71527037	q1	q2	OK	9.21244	8.86585	-0.0553231	-0.0129626	0.96155	0.99995	no

ENSG00000125944	ENSG00000125944	HNRNPR	1:23630263-23670829	q1	q2	OK	184.904	196.014	0.0841872	0.183496	0.79135	0.99995	no

ENSG00000125945	ENSG00000125945	ZNF436	1:23685940-23698332	q1	q2	OK	17.8323	17.7179	-0.00928561	-0.0197377	0.97755	0.99995	no

ENSG00000125952	ENSG00000125952	MAX	14:65381078-65569413	q1	q2	OK	26.3994	23.6691	-0.1575	-0.118428	0.77715	0.99995	no

ENSG00000125954	ENSG00000125954	CHURC1-FNTB	14:65381078-65569413	q1	q2	OK	0.556486	1.1964	1.10429	0.0953667	0.59705	0.99995	no

ENSG00000125962	ENSG00000125962	ARMCX5	X:101854095-101973607	q1	q2	OK	8.49571	9.73282	0.196123	0.237351	0.73285	0.99995	no

ENSG00000125965	ENSG00000125965	GDF5	20:34020826-34042568	q1	q2	NOTEST	0.230045	0.0752941	-1.61131	0	1	1	no

ENSG00000125966	ENSG00000125966	MMP24	20:33703166-33872788	q1	q2	OK	0.884488	0.851296	-0.0551823	-0.0254111	0.96465	0.99995	no

ENSG00000125967	ENSG00000125967	NECAB3	20:32244892-32262269	q1	q2	OK	9.714	6.53166	-0.572617	-0.73508	0.1731	0.99995	no

ENSG00000125968	ENSG00000125968	ID1	20:30193085-30194318	q1	q2	OK	17.8233	13.4038	-0.411118	-0.691826	0.3304	0.99995	no

ENSG00000125970	ENSG00000125970	RALY	20:32570857-32700138	q1	q2	OK	147.612	140.421	-0.0720581	-0.118696	0.8569	0.99995	no

ENSG00000125971	ENSG00000125971	DYNLRB1	20:33104213-33128762	q1	q2	OK	125.623	125.131	-0.0056588	-0.0131454	0.98485	0.99995	no

ENSG00000125975	ENSG00000125975	C20orf173	20:34108568-34117481	q1	q2	NOTEST	0.0284555	0	#NAME?	0	1	1	no

ENSG00000125977	ENSG00000125977	EIF2S2	20:32570857-32700138	q1	q2	OK	62.0514	62.2676	0.00501666	0.0079144	0.99015	0.99995	no

ENSG00000125991	ENSG00000125991	ERGIC3	20:34129769-34145405	q1	q2	OK	144.383	124.091	-0.21851	-0.486704	0.4424	0.99995	no

ENSG00000125995	ENSG00000125995	ROMO1	20:34213952-34288906	q1	q2	OK	115.084	97.2665	-0.242669	-0.139841	0.8792	0.99995	no

ENSG00000125997	ENSG00000125997	BPIFB9P	20:31935525-31942250	q1	q2	NOTEST	0.171238	0.0251745	-2.76596	0	1	1	no

ENSG00000125998	ENSG00000125998	FAM83C	20:33873053-33880204	q1	q2	NOTEST	0.00849383	0	#NAME?	0	1	1	no

ENSG00000125999	ENSG00000125999	BPIFB1	20:31861285-31897684	q1	q2	NOTEST	0	0	0	0	1	1	no

ENSG00000126001	ENSG00000126001	CEP250	20:34042984-34099804	q1	q2	OK	16.5009	15.4602	-0.0939889	-0.0897795	0.7963	0.99995	no

ENSG00000126003	ENSG00000126003	PLAGL2	20:30780305-30795594	q1	q2	OK	16.7819	18.1749	0.115045	0.276856	0.68685	0.99995	no

ENSG00000126005	ENSG00000126005	MMP24-AS1	20:33703166-33872788	q1	q2	OK	16.6555	14.2567	-0.224366	-0.206517	0.7527	0.99995	no

ENSG00000126010	ENSG00000126010	GRPR	X:16141678-16189587	q1	q2	NOTEST	0.0838476	0.113152	0.432421	0	1	1	no

ENSG00000126012	ENSG00000126012	KDM5C	X:53220502-53254604	q1	q2	NOTEST	0	0	0	0	1	1	no

ENSG00000126016	ENSG00000126016	AMOT	X:112017730-112084043	q1	q2	OK	26.7878	29.385	0.133502	0.163408	0.71255	0.99995	no

ENSG00000126062	ENSG00000126062	TMEM115	3:50384760-50541675	q1	q2	OK	17.0496	14.5366	-0.230051	-0.350762	0.61875	0.99995	no

ENSG00000126067	ENSG00000126067	PSMB2	1:36067184-36107445	q1	q2	OK	59.5811	60.0801	0.0120323	0.0300152	0.96745	0.99995	no

ENSG00000126070	ENSG00000126070	AGO3	1:36396318-36538101	q1	q2	OK	10.0168	10.0617	0.00645231	0.00619945	0.98825	0.99995	no

ENSG00000126088	ENSG00000126088	UROD	1:45477818-45481247	q1	q2	OK	39.5333	35.0847	-0.172225	-0.345407	0.60825	0.99995	no

ENSG00000126091	ENSG00000126091	ST3GAL3	1:44115828-44396831	q1	q2	OK	14.7699	14.8366	0.00650233	0.00514141	0.99175	0.99995	no

ENSG00000126106	ENSG00000126106	TMEM53	1:44870865-45140227	q1	q2	OK	5.71353	5.81159	0.0245507	0.0118932	0.97805	0.99995	no

ENSG00000126107	ENSG00000126107	HECTD3	1:45468211-45477001	q1	q2	OK	13.7735	11.6707	-0.239	-0.390223	0.51445	0.99995	no

ENSG00000126214	ENSG00000126214	KLC1	14:104019757-104181841	q1	q2	OK	150.14	147.499	-0.0256005	-0.0350864	0.94995	0.99995	no

ENSG00000126215	ENSG00000126215	XRCC3	14:104019757-104181841	q1	q2	OK	21.9723	17.8169	-0.302442	-0.174888	0.7413	0.99995	no

ENSG00000126216	ENSG00000126216	TUBGCP3	13:113139324-113242481	q1	q2	OK	18.0665	19.7333	0.127315	0.207626	0.72315	0.99995	no

ENSG00000126217	ENSG00000126217	MCF2L	13:113548691-113754053	q1	q2	OK	14.8196	15.9949	0.110107	0.164785	0.72365	0.99995	no

ENSG00000126218	ENSG00000126218	F10	13:113777127-113803843	q1	q2	NOTEST	0.0745337	0.129589	0.797984	0	1	1	no

ENSG00000126226	ENSG00000126226	PCID2	13:113831890-113919399	q1	q2	OK	23.3887	23.2509	-0.00852408	-0.00935734	0.98735	0.99995	no

ENSG00000126231	ENSG00000126231	PROZ	13:113812967-113826694	q1	q2	NOTEST	0.036931	0.017951	-1.04077	0	1	1	no

ENSG00000126233	ENSG00000126233	SLURP1	8:143822361-143823829	q1	q2	NOTEST	0	0	0	0	1	1	no

ENSG00000126243	ENSG00000126243	LRFN3	19:36426259-36436669	q1	q2	OK	15.3934	13.8638	-0.150992	-0.192001	0.7529	0.99995	no

ENSG00000126246	ENSG00000126246	IGFLR1	19:36230057-36245420	q1	q2	OK	3.39405	2.89811	-0.227895	-0.0984958	0.7959	0.99995	no

ENSG00000126247	ENSG00000126247	CAPNS1	19:36630476-36641255	q1	q2	OK	129.518	121.218	-0.0955522	-0.195671	0.7749	0.99995	no

ENSG00000126249	ENSG00000126249	PDCD2L	19:34850384-34919178	q1	q2	OK	7.40338	5.64314	-0.391686	-0.202738	0.7251	0.99995	no

ENSG00000126251	ENSG00000126251	GPR42	19:35861830-35863855	q1	q2	NOTEST	0	0	0	0	1	1	no

ENSG00000126254	ENSG00000126254	RBM42	19:36119931-36128588	q1	q2	OK	39.348	33.8697	-0.216297	-0.488295	0.48335	0.99995	no

ENSG00000126259	ENSG00000126259	KIRREL2	19:36316865-36370693	q1	q2	OK	3.34452	2.87576	-0.217857	-0.117785	0.8295	0.99995	no

ENSG00000126261	ENSG00000126261	UBA2	19:34919256-34960853	q1	q2	OK	97.2667	104.692	0.106128	0.220018	0.7333	0.99995	no

ENSG00000126262	ENSG00000126262	FFAR2	19:35934808-35942669	q1	q2	NOTEST	0	0	0	0	1	1	no

ENSG00000126264	ENSG00000126264	HCST	19:36393381-36395279	q1	q2	NOTEST	0.217324	0.476614	1.13297	0	1	1	no

ENSG00000126266	ENSG00000126266	FFAR1	19:35842444-35843367	q1	q2	NOTEST	0	0	0	0	1	1	no

ENSG00000126267	ENSG00000126267	COX6B1	19:36139124-36149763	q1	q2	OK	156.755	148.752	-0.0756035	-0.175785	0.799	0.99995	no

ENSG00000126337	ENSG00000126337	KRT36	17:39642387-39648798	q1	q2	NOTEST	0	0	0	0	1	1	no

ENSG00000126351	ENSG00000126351	THRA	17:38175349-38256978	q1	q2	OK	9.64267	8.62311	-0.161225	-0.159182	0.8119	0.99995	no

ENSG00000126353	ENSG00000126353	CCR7	17:38710020-38721724	q1	q2	NOTEST	0.0962176	0.123455	0.359612	0	1	1	no

ENSG00000126368	ENSG00000126368	NR1D1	17:38175349-38256978	q1	q2	OK	1.31118	1.09942	-0.254118	-0.103422	0.82675	0.99995	no

ENSG00000126391	ENSG00000126391	FRMD8	11:65154069-65180996	q1	q2	OK	8.08704	6.67319	-0.277236	-0.325018	0.46325	0.99995	no

ENSG00000126432	ENSG00000126432	PRDX5	11:64085559-64089283	q1	q2	OK	165.874	157.875	-0.0713018	-0.160164	0.82265	0.99995	no

ENSG00000126453	ENSG00000126453	BCL2L12	19:50162825-50177173	q1	q2	OK	23.4505	26.3285	0.167003	0.194701	0.7527	0.99995	no

ENSG00000126456	ENSG00000126456	IRF3	19:50162825-50177173	q1	q2	OK	32.606	33.5418	0.0408208	0.0541069	0.9149	0.99995	no

ENSG00000126457	ENSG00000126457	PRMT1	19:50179042-50193832	q1	q2	OK	222.568	223.44	0.00564389	0.0131817	0.9837	0.99995	no

ENSG00000126458	ENSG00000126458	RRAS	19:50138548-50143458	q1	q2	OK	4.0967	2.48991	-0.718368	-0.783806	0.25075	0.99995	no

ENSG00000126460	ENSG00000126460	PRRG2	19:50058967-50094272	q1	q2	NOTEST	0.102903	0.395002	1.94057	0	1	1	no

ENSG00000126461	ENSG00000126461	SCAF1	19:50145381-50161899	q1	q2	OK	21.4869	17.262	-0.315861	-0.560357	0.2834	0.99995	no

ENSG00000126464	ENSG00000126464	PRR12	19:50094899-50129696	q1	q2	OK	12.4018	11.5261	-0.105638	-0.199731	0.7199	0.99995	no

ENSG00000126467	ENSG00000126467	TSKS	19:50243009-50266587	q1	q2	NOTEST	0	0	0	0	1	1	no

ENSG00000126500	ENSG00000126500	FLRT1	11:63742078-63933578	q1	q2	OK	1.98463	2.06933	0.0602939	0.0291167	0.95155	0.99995	no

ENSG00000126522	ENSG00000126522	ASL	7:65540784-65629998	q1	q2	OK	10.4002	9.3257	-0.157322	-0.168441	0.8106	0.99995	no

ENSG00000126524	ENSG00000126524	SBDS	7:66452663-66704501	q1	q2	OK	32.3865	31.459	-0.041921	-0.0794194	0.90655	0.99995	no

ENSG00000126542	ENSG00000126542	PTPN20CP	10:49272333-49327498	q1	q2	NOTEST	0	0	0	0	1	1	no

ENSG00000126545	ENSG00000126545	CSN1S1	4:70796798-70812289	q1	q2	NOTEST	0	0	0	0	1	1	no

ENSG00000126549	ENSG00000126549	STATH	4:70861646-70868287	q1	q2	NOTEST	0	0	0	0	1	1	no

ENSG00000126550	ENSG00000126550	HTN1	4:70916118-70924562	q1	q2	NOTEST	0	0	0	0	1	1	no

ENSG00000126561	ENSG00000126561	STAT5A	17:40439564-40463961	q1	q2	OK	0.787514	0.913023	0.213345	0.159512	0.69965	0.99995	no

ENSG00000126562	ENSG00000126562	WNK4	17:40932695-40950722	q1	q2	NOTEST	0.302083	0.410286	0.441683	0	1	1	no

ENSG00000126581	ENSG00000126581	BECN1	17:40950809-40995774	q1	q2	OK	31.6463	30.2777	-0.063784	-0.0574767	0.90165	0.99995	no

ENSG00000126583	ENSG00000126583	PRKCG	19:54382443-54410906	q1	q2	OK	1.32766	0.931654	-0.511018	-0.461587	0.37065	0.99995	no

ENSG00000126602	ENSG00000126602	TRAP1	16:3661728-3767598	q1	q2	OK	37.0269	36.6163	-0.0160851	-0.0232852	0.9627	0.99995	no

ENSG00000126603	ENSG00000126603	GLIS2	16:4364761-4475706	q1	q2	OK	6.83614	6.54707	-0.0623332	-0.0921616	0.8968	0.99995	no

ENSG00000126653	ENSG00000126653	NSRP1	17:27887564-28513493	q1	q2	OK	29.4888	31.9575	0.115988	0.114543	0.8328	0.99995	no

ENSG00000126698	ENSG00000126698	DNAJC8	1:28525966-28559536	q1	q2	OK	103.231	108.509	0.0719447	0.173655	0.7967	0.99995	no

ENSG00000126705	ENSG00000126705	AHDC1	1:27860545-27930942	q1	q2	OK	6.82497	6.0866	-0.165187	-0.27705	0.59955	0.99995	no

ENSG00000126709	ENSG00000126709	IFI6	1:27992571-27998729	q1	q2	OK	9.4817	9.73762	0.0384233	0.053577	0.93895	0.99995	no

ENSG00000126733	ENSG00000126733	DACH2	X:85403461-86087607	q1	q2	OK	0.783019	0.814796	0.0573911	0.0552571	0.9319	0.99995	no

ENSG00000126746	ENSG00000126746	ZNF384	12:6772425-6798738	q1	q2	OK	42.4507	39.9487	-0.0876402	-0.159328	0.7955	0.99995	no

ENSG00000126749	ENSG00000126749	EMG1	12:7072407-7178336	q1	q2	OK	0.630263	0.536141	-0.233344	-0.0778363	0.81595	0.99995	no

ENSG00000126752	ENSG00000126752	SSX1	X:48114751-48126879	q1	q2	NOTEST	0	0	0	0	1	1	no

ENSG00000126756	ENSG00000126756	UXT	X:47511196-47519776	q1	q2	OK	50.4641	48.2706	-0.0641107	-0.118483	0.86755	0.99995	no

ENSG00000126759	ENSG00000126759	CFP	X:47483611-47489704	q1	q2	OK	0.85923	0.53356	-0.687395	-0.429011	0.45265	0.99995	no

ENSG00000126767	ENSG00000126767	ELK1	X:47494919-47510003	q1	q2	OK	12.8156	12.7136	-0.0115312	-0.0151845	0.97895	0.99995	no

ENSG00000126768	ENSG00000126768	TIMM17B	X:48750729-48760420	q1	q2	OK	0.877609	0.815407	-0.106058	-0.0394985	0.9316	0.99995	no

ENSG00000126773	ENSG00000126773	PCNXL4	14:60558628-60636574	q1	q2	OK	43.2166	44.0229	0.0266684	0.0427684	0.93515	0.99995	no

ENSG00000126775	ENSG00000126775	ATG14	14:55833109-55878576	q1	q2	OK	8.46389	7.74457	-0.128136	-0.273207	0.6981	0.99995	no

ENSG00000126777	ENSG00000126777	KTN1	14:55965995-56168244	q1	q2	OK	59.9349	64.4423	0.104613	0.212494	0.7133	0.99995	no

ENSG00000126778	ENSG00000126778	SIX1	14:61110132-61124977	q1	q2	OK	2.44286	1.96853	-0.311453	-0.193175	0.7024	0.99995	no

ENSG00000126785	ENSG00000126785	RHOJ	14:63670831-63759937	q1	q2	OK	16.7659	16.9941	0.0195001	0.0313627	0.95645	0.99995	no

ENSG00000126787	ENSG00000126787	DLGAP5	14:55614829-55658396	q1	q2	OK	50.0443	55.2499	0.142766	0.321863	0.6139	0.99995	no

ENSG00000126790	ENSG00000126790	L3HYPDH	14:59895739-60043549	q1	q2	OK	9.67504	9.39891	-0.0417742	-0.0271858	0.9613	0.99995	no

ENSG00000126803	ENSG00000126803	HSPA2	14:64854748-65009955	q1	q2	OK	0.637981	0.402582	-0.664232	-0.127199	0.69905	0.99995	no

ENSG00000126804	ENSG00000126804	ZBTB1	14:64854748-65009955	q1	q2	OK	29.4907	30.2801	0.0381089	0.0501989	0.92855	0.99995	no

ENSG00000126814	ENSG00000126814	TRMT5	14:61201459-61550451	q1	q2	OK	5.8429	7.47707	0.355787	0.356627	0.43185	0.99995	no

ENSG00000126821	ENSG00000126821	SGPP1	14:64150931-64194757	q1	q2	OK	4.18439	3.88771	-0.106096	-0.178552	0.79735	0.99995	no

ENSG00000126822	ENSG00000126822	PLEKHG3	14:65170819-65346601	q1	q2	OK	16.8687	15.6614	-0.107135	-0.206525	0.74245	0.99995	no

ENSG00000126838	ENSG00000126838	PZP	12:9301435-9360966	q1	q2	NOTEST	0.166026	0.227048	0.451589	0	1	1	no

ENSG00000126856	ENSG00000126856	PRDM7	16:90122973-90158480	q1	q2	NOTEST	0	0	0	0	1	1	no

ENSG00000126858	ENSG00000126858	RHOT1	17:30468244-30580393	q1	q2	OK	21.4865	21.8472	0.0240153	0.0441692	0.9371	0.99995	no

ENSG00000126860	ENSG00000126860	EVI2A	17:29335514-29709134	q1	q2	NOTEST	0.13458	0.149791	0.154482	0	1	1	no

ENSG00000126861	ENSG00000126861	OMG	17:29335514-29709134	q1	q2	OK	0.373098	0.513161	0.459854	0.0472226	0.69445	0.99995	no

ENSG00000126870	ENSG00000126870	WDR60	7:158649268-158749438	q1	q2	OK	14.0961	12.7364	-0.14634	-0.225043	0.6626	0.99995	no

ENSG00000126878	ENSG00000126878	AIF1L	9:133971862-133998539	q1	q2	OK	32.2064	30.6794	-0.0700768	-0.163012	0.80825	0.99995	no

ENSG00000126882	ENSG00000126882	FAM78A	9:134133462-134151934	q1	q2	OK	2.39923	2.10671	-0.187578	-0.28661	0.68205	0.99995	no

ENSG00000126883	ENSG00000126883	NUP214	9:134000947-134110057	q1	q2	OK	14.9493	14.9378	-0.00111272	-0.00166347	0.997	0.99995	no

ENSG00000126890	ENSG00000126890	CTAG2	X:153880245-153881853	q1	q2	NOTEST	0	0	0	0	1	1	no

ENSG00000126895	ENSG00000126895	AVPR2	X:153126968-153200676	q1	q2	NOTEST	0	0	0	0	1	1	no

ENSG00000126903	ENSG00000126903	SLC10A3	X:153715644-153719016	q1	q2	NOTEST	0.0259044	0.0493735	0.930541	0	1	1	no

ENSG00000126934	ENSG00000126934	MAP2K2	19:4090318-4124126	q1	q2	OK	68.9056	58.2628	-0.242045	-0.552256	0.41625	0.99995	no

ENSG00000126945	ENSG00000126945	HNRNPH2	X:100645811-100669121	q1	q2	OK	1.61079	1.79198	0.153792	0.0293495	0.9296	0.99995	no

ENSG00000126947	ENSG00000126947	ARMCX1	X:100805513-100809683	q1	q2	NOTEST	0	0	0	0	1	1	no

ENSG00000126950	ENSG00000126950	TMEM35	X:100264334-100351353	q1	q2	OK	13.1055	11.9532	-0.132775	-0.235006	0.73695	0.99995	no

ENSG00000126952	ENSG00000126952	NXF5	X:101087084-101112549	q1	q2	NOTEST	0.0141603	0.0149593	0.0791971	0	1	1	no

ENSG00000126953	ENSG00000126953	TIMM8A	X:100600648-100604184	q1	q2	NOTEST	0.142528	0.205784	0.529881	0	1	1	no

ENSG00000126970	ENSG00000126970	ZC4H2	X:64136249-64254593	q1	q2	OK	32.4166	34.8668	0.105123	0.229963	0.72545	0.99995	no

ENSG00000127022	ENSG00000127022	CANX	5:179105583-179157926	q1	q2	OK	273.629	253.206	-0.111911	-0.117341	0.7675	0.99995	no

ENSG00000127054	ENSG00000127054	CPSF3L	1:1227755-1260071	q1	q2	OK	55.1524	48.3136	-0.190994	-0.220867	0.6246	0.99995	no

ENSG00000127074	ENSG00000127074	RGS13	1:192605274-192629390	q1	q2	NOTEST	0	0.0100388	inf	0	1	1	no

ENSG00000127080	ENSG00000127080	IPPK	9:95059639-95432547	q1	q2	OK	6.4001	6.18362	-0.049642	-0.0380192	0.9476	0.99995	no

ENSG00000127081	ENSG00000127081	ZNF484	9:95571669-95661890	q1	q2	OK	5.08857	5.06896	-0.00557	-0.00936184	0.9895	0.99995	no

ENSG00000127083	ENSG00000127083	OMD	9:95059639-95432547	q1	q2	NOTEST	0.0105511	0.0760483	2.84952	0	1	1	no

ENSG00000127084	ENSG00000127084	FGD3	9:95709732-95798518	q1	q2	OK	5.6089	4.32776	-0.374097	-0.614898	0.3366	0.99995	no

ENSG00000127124	ENSG00000127124	HIVEP3	1:41972035-42501596	q1	q2	OK	0.846244	0.829112	-0.0295069	-0.0251813	0.96785	0.99995	no

ENSG00000127125	ENSG00000127125	PPCS	1:42895999-43120335	q1	q2	OK	20.8745	21.2539	0.0259823	0.0414571	0.9532	0.99995	no

ENSG00000127129	ENSG00000127129	EDN2	1:41944445-41950344	q1	q2	NOTEST	0.0240595	0.0409432	0.767016	0	1	1	no

ENSG00000127152	ENSG00000127152	BCL11B	14:99635623-99737861	q1	q2	OK	1.01876	0.98109	-0.0543613	-0.079055	0.9079	0.99995	no

ENSG00000127184	ENSG00000127184	COX7C	5:85913720-85916779	q1	q2	OK	394.949	398.106	0.0114842	0.0261862	0.96755	0.99995	no

ENSG00000127191	ENSG00000127191	TRAF2	9:139776363-139821059	q1	q2	OK	12.3595	9.89297	-0.321146	-0.574082	0.35815	0.99995	no

ENSG00000127220	ENSG00000127220	ABHD8	19:17402939-17445638	q1	q2	OK	5.26978	4.48106	-0.233902	-0.148814	0.83495	0.99995	no

ENSG00000127241	ENSG00000127241	MASP1	3:186935941-187009810	q1	q2	OK	3.59686	2.42469	-0.568938	-0.592059	0.21575	0.99995	no

ENSG00000127249	ENSG00000127249	ATP13A4	3:193119865-193310900	q1	q2	NOTEST	0.159393	0.256774	0.687911	0	1	1	no

ENSG00000127252	ENSG00000127252	HRASLS	3:192958913-193096632	q1	q2	OK	0.825435	1.02998	0.319391	0.259367	0.71205	0.99995	no

ENSG00000127311	ENSG00000127311	HELB	12:66696324-66737423	q1	q2	OK	0.843657	0.934694	0.147837	0.144188	0.79975	0.99995	no

ENSG00000127314	ENSG00000127314	RAP1B	12:69004618-69054372	q1	q2	OK	90.1712	94.8334	0.0727285	0.146046	0.821	0.99995	no

ENSG00000127318	ENSG00000127318	IL22	12:68642021-68647387	q1	q2	NOTEST	0	0	0	0	1	1	no

ENSG00000127324	ENSG00000127324	TSPAN8	12:71518864-71980090	q1	q2	NOTEST	0.0540964	0.0416487	-0.37726	0	1	1	no

ENSG00000127325	ENSG00000127325	BEST3	12:70037139-70093256	q1	q2	NOTEST	0.00933655	0.0165217	0.823403	0	1	1	no

ENSG00000127328	ENSG00000127328	RAB3IP	12:70132460-70748773	q1	q2	OK	8.62748	8.19747	-0.0737604	-0.0505552	0.9058	0.99995	no

ENSG00000127329	ENSG00000127329	PTPRB	12:70861859-71031220	q1	q2	NOTEST	0.205081	0.30369	0.566405	0	1	1	no

ENSG00000127334	ENSG00000127334	DYRK2	12:68042117-68064699	q1	q2	OK	8.00436	9.25242	0.209045	0.387992	0.554	0.99995	no

ENSG00000127337	ENSG00000127337	YEATS4	12:69753482-69784576	q1	q2	OK	30.7603	30.2718	-0.0230955	-0.0471726	0.94465	0.99995	no

ENSG00000127362	ENSG00000127362	TAS2R3	7:141404137-141488440	q1	q2	NOTEST	0.244389	0.18959	-0.366298	0	1	1	no

ENSG00000127364	ENSG00000127364	TAS2R4	7:141404137-141488440	q1	q2	OK	0.895246	1.23431	0.463347	0.176779	0.7173	0.99995	no

ENSG00000127366	ENSG00000127366	TAS2R5	7:141490016-141491166	q1	q2	OK	1.93325	1.89964	-0.0253019	-0.0272412	0.96505	0.99995	no

ENSG00000127377	ENSG00000127377	CRYGN	7:151124768-151137899	q1	q2	NOTEST	0.101525	0.0212341	-2.25738	0	1	1	no

ENSG00000127399	ENSG00000127399	LRRC61	7:149941004-150035239	q1	q2	NOTEST	0.0319803	0.10765	1.75109	0	1	1	no

ENSG00000127412	ENSG00000127412	TRPV5	7:142605266-142630905	q1	q2	NOTEST	0	0	0	0	1	1	no

ENSG00000127415	ENSG00000127415	IDUA	4:952674-998316	q1	q2	OK	6.98657	4.74633	-0.557771	-0.423627	0.4271	0.99995	no

ENSG00000127418	ENSG00000127418	FGFRL1	4:1003723-1020685	q1	q2	OK	25.1368	22.3639	-0.168629	-0.396541	0.568	0.99995	no

ENSG00000127419	ENSG00000127419	TMEM175	4:926174-952444	q1	q2	OK	9.70243	7.63407	-0.345894	-0.40283	0.44625	0.99995	no

ENSG00000127423	ENSG00000127423	AUNIP	1:26126666-26185903	q1	q2	OK	4.73391	4.02938	-0.232473	-0.0964639	0.93725	0.99995	no

ENSG00000127445	ENSG00000127445	PIN1	19:9945932-9960358	q1	q2	OK	69.3529	64.9549	-0.0945173	-0.201075	0.7682	0.99995	no

ENSG00000127452	ENSG00000127452	FBXL12	19:9920942-9938492	q1	q2	OK	18.7716	19.4739	0.0529883	0.0965781	0.8757	0.99995	no

ENSG00000127463	ENSG00000127463	EMC1	1:19536994-19586622	q1	q2	OK	24.2872	21.8446	-0.152917	-0.211397	0.64335	0.99995	no

ENSG00000127472	ENSG00000127472	PLA2G5	1:20354671-20417683	q1	q2	NOTEST	0.0142011	0.0256176	0.851137	0	1	1	no

ENSG00000127481	ENSG00000127481	UBR4	1:19398603-19536770	q1	q2	OK	60.7089	52.5891	-0.207144	-0.213241	0.6533	0.99995	no

ENSG00000127483	ENSG00000127483	HP1BP3	1:21069153-21113816	q1	q2	OK	182.686	181.975	-0.00563224	-0.00840836	0.9865	0.99995	no

ENSG00000127507	ENSG00000127507	EMR2	19:14729928-14889353	q1	q2	NOTEST	0.0970268	0.0439767	-1.14165	0	1	1	no

ENSG00000127511	ENSG00000127511	SIN3B	19:16940210-16991164	q1	q2	OK	28.5641	26.8617	-0.0886536	-0.135265	0.78935	0.99995	no

ENSG00000127515	ENSG00000127515	OR7A10	19:14951759-14952689	q1	q2	NOTEST	0	0	0	0	1	1	no

ENSG00000127526	ENSG00000127526	SLC35E1	19:16589867-16771253	q1	q2	OK	13.7996	13.0591	-0.0795688	-0.0569893	0.9026	0.99995	no

ENSG00000127527	ENSG00000127527	EPS15L1	19:16466049-16582896	q1	q2	OK	20.2211	19.1918	-0.0753679	-0.139037	0.8181	0.99995	no

ENSG00000127528	ENSG00000127528	KLF2	19:16435627-16438685	q1	q2	OK	1.26864	1.01897	-0.316169	-0.27609	0.65245	0.99995	no

ENSG00000127529	ENSG00000127529	OR7C2	19:15052300-15053260	q1	q2	NOTEST	0	0	0	0	1	1	no

ENSG00000127530	ENSG00000127530	OR7C1	19:14903120-14946188	q1	q2	NOTEST	0	0	0	0	1	1	no

ENSG00000127533	ENSG00000127533	F2RL3	19:16999670-17003417	q1	q2	NOTEST	0.0225341	0.015082	-0.579287	0	1	1	no

ENSG00000127540	ENSG00000127540	UQCR11	19:1576638-1605480	q1	q2	OK	69.5486	65.7501	-0.0810297	-0.0779122	0.896	0.99995	no

ENSG00000127554	ENSG00000127554	GFER	16:2034207-2044276	q1	q2	OK	21.3668	18.7465	-0.188753	-0.251885	0.68055	0.99995	no

ENSG00000127561	ENSG00000127561	SYNGR3	16:2034207-2044276	q1	q2	OK	1.30269	1.44344	0.148017	0.0674783	0.9099	0.99995	no

ENSG00000127564	ENSG00000127564	PKMYT1	16:3013944-3030540	q1	q2	OK	27.1285	23.2044	-0.225409	-0.258774	0.6826	0.99995	no

ENSG00000127578	ENSG00000127578	WFIKKN1	16:639356-684116	q1	q2	OK	4.79038	4.01581	-0.254449	-0.194655	0.8069	0.99995	no

ENSG00000127580	ENSG00000127580	WDR24	16:734621-755829	q1	q2	OK	7.33105	6.28441	-0.222244	-0.2548	0.6649	0.99995	no

ENSG00000127585	ENSG00000127585	FBXL16	16:734621-755829	q1	q2	OK	8.71153	8.08027	-0.108522	-0.178718	0.79145	0.99995	no

ENSG00000127586	ENSG00000127586	CHTF18	16:834973-850737	q1	q2	OK	23.5192	21.671	-0.118079	-0.149601	0.80535	0.99995	no

ENSG00000127588	ENSG00000127588	GNG13	16:834973-850737	q1	q2	NOTEST	0.186716	0.276939	0.568722	0	1	1	no

ENSG00000127589	ENSG00000127589	TUBBP1	8:30209388-30211034	q1	q2	OK	147.752	142.349	-0.0537434	-0.132164	0.85545	0.99995	no

ENSG00000127603	ENSG00000127603	MACF1	1:39546987-39952849	q1	q2	OK	111.908	121.296	0.116222	0.105304	0.8046	0.99995	no

ENSG00000127616	ENSG00000127616	SMARCA4	19:11071597-11176071	q1	q2	OK	131.833	134.599	0.0299526	0.0395975	0.9418	0.99995	no

ENSG00000127663	ENSG00000127663	KDM4B	19:4969124-5153606	q1	q2	OK	16.0015	14.6295	-0.129327	-0.268789	0.6524	0.99995	no

ENSG00000127666	ENSG00000127666	TICAM1	19:4815943-4831716	q1	q2	NOTEST	0.232091	0.218352	-0.0880362	0	1	1	no

ENSG00000127720	ENSG00000127720	METTL25	12:82617459-82873015	q1	q2	OK	2.89677	3.32829	0.200335	0.12653	0.8287	0.99995	no

ENSG00000127743	ENSG00000127743	IL17B	5:148750886-148783765	q1	q2	NOTEST	0.253043	0.166165	-0.606767	0	1	1	no

ENSG00000127774	ENSG00000127774	EMC6	17:3566195-3599698	q1	q2	OK	38.4809	38.3617	-0.00447532	-0.00519811	0.99415	0.99995	no

ENSG00000127780	ENSG00000127780	OR1E2	17:3336163-3337135	q1	q2	NOTEST	0.0307412	0.0300244	-0.0340379	0	1	1	no

ENSG00000127804	ENSG00000127804	METTL16	17:2308855-2415185	q1	q2	OK	13.4954	14.0566	0.0587757	0.108758	0.85105	0.99995	no

ENSG00000127824	ENSG00000127824	TUBA4A	2:220094478-220142892	q1	q2	OK	1.18048	1.03362	-0.191671	-0.0566952	0.8588	0.99995	no

ENSG00000127831	ENSG00000127831	VIL1	2:219283814-219433084	q1	q2	NOTEST	0.0833422	0.0210451	-1.98557	0	1	1	no

ENSG00000127837	ENSG00000127837	AAMP	2:219128849-219134980	q1	q2	OK	43.1915	37.1134	-0.218807	-0.484892	0.4771	0.99995	no

ENSG00000127838	ENSG00000127838	PNKD	2:219135114-219232822	q1	q2	OK	29.605	24.8907	-0.250235	-0.385554	0.5717	0.99995	no

ENSG00000127863	ENSG00000127863	TNFRSF19	13:24144508-24250232	q1	q2	OK	22.6718	26.8952	0.246449	0.521048	0.4466	0.99995	no

ENSG00000127870	ENSG00000127870	RNF6	13:26688546-26796791	q1	q2	OK	7.76351	8.52001	0.134146	0.171572	0.7144	0.99995	no

ENSG00000127884	ENSG00000127884	ECHS1	10:135175983-135187193	q1	q2	OK	59.1386	59.2898	0.0036829	0.00889493	0.98995	0.99995	no

ENSG00000127903	ENSG00000127903	ZNF835	19:57174019-57183151	q1	q2	NOTEST	0.0475025	0.0933632	0.974851	0	1	1	no

ENSG00000127914	ENSG00000127914	AKAP9	7:91570180-91739987	q1	q2	OK	22.8554	24.1245	0.0779647	0.122193	0.80825	0.99995	no

ENSG00000127920	ENSG00000127920	GNG11	7:93551010-93557922	q1	q2	OK	12.8232	16.0901	0.32742	0.718713	0.3043	0.99995	no

ENSG00000127922	ENSG00000127922	SHFM1	7:96110937-96339203	q1	q2	OK	187.088	220.497	0.237036	0.448857	0.515	0.99995	no

ENSG00000127928	ENSG00000127928	GNGT1	7:93220884-93540577	q1	q2	NOTEST	0	0.0695012	inf	0	1	1	no

ENSG00000127946	ENSG00000127946	HIP1	7:75162620-75368280	q1	q2	OK	3.25264	1.32343	-1.29733	-0.983664	0.15405	0.99995	no

ENSG00000127947	ENSG00000127947	PTPN12	7:77166591-77269388	q1	q2	OK	51.0705	57.1051	0.161129	0.336619	0.5501	0.99995	no

ENSG00000127948	ENSG00000127948	POR	7:75528517-75623977	q1	q2	OK	5.73565	1.98294	-1.53232	-1.00773	0.03815	0.99995	no

ENSG00000127951	ENSG00000127951	FGL2	7:76139744-77045717	q1	q2	NOTEST	0.0664821	0.0925954	0.477975	0	1	1	no

ENSG00000127952	ENSG00000127952	STYXL1	7:75625655-75677322	q1	q2	OK	1.22471	1.54276	0.333073	0.298595	0.64065	0.99995	no

ENSG00000127954	ENSG00000127954	STEAP4	7:87834432-87936206	q1	q2	NOTEST	0.0597646	0.039757	-0.588082	0	1	1	no

ENSG00000127955	ENSG00000127955	GNAI1	7:79763270-79848718	q1	q2	OK	20.5451	21.0515	0.0351287	0.0664197	0.9171	0.99995	no

ENSG00000127957	ENSG00000127957	PMS2P3	7:75123400-75157478	q1	q2	OK	0.953605	0.720013	-0.40537	-0.265642	0.5873	0.99995	no

ENSG00000127980	ENSG00000127980	PEX1	7:92076766-92157845	q1	q2	OK	18.4479	18.6052	0.0122462	0.0119822	0.9795	0.99995	no

ENSG00000127989	ENSG00000127989	MTERF	7:91321322-91510034	q1	q2	OK	10.0433	8.31646	-0.272187	-0.400424	0.51965	0.99995	no

ENSG00000127990	ENSG00000127990	SGCE	7:94214541-94285521	q1	q2	OK	27.4877	26.4161	-0.0573711	-0.120185	0.8597	0.99995	no

ENSG00000127993	ENSG00000127993	RBM48	7:92158086-92167319	q1	q2	OK	10.0775	8.56688	-0.234297	-0.369581	0.58155	0.99995	no

ENSG00000127995	ENSG00000127995	CASD1	7:94138530-94186331	q1	q2	OK	36.1053	39.4364	0.127315	0.245605	0.6717	0.99995	no

ENSG00000128000	ENSG00000128000	ZNF780B	19:40534166-40600313	q1	q2	OK	8.83372	9.64714	0.12708	0.100467	0.7832	0.99995	no

ENSG00000128011	ENSG00000128011	LRFN1	19:39797207-39811498	q1	q2	OK	1.71893	1.38248	-0.314256	-0.4188	0.54945	0.99995	no

ENSG00000128016	ENSG00000128016	ZFP36	19:39897452-39900052	q1	q2	OK	3.03002	2.55428	-0.246413	-0.304856	0.64005	0.99995	no

ENSG00000128039	ENSG00000128039	SRD5A3	4:56212275-56262014	q1	q2	OK	3.71258	3.53129	-0.0722281	-0.062096	0.93015	0.99995	no

ENSG00000128040	ENSG00000128040	SPINK2	4:57676025-57687908	q1	q2	NOTEST	0	0.0436181	inf	0	1	1	no

ENSG00000128045	ENSG00000128045	RASL11B	4:53728456-53733000	q1	q2	OK	3.14521	3.91542	0.316011	0.42809	0.5402	0.99995	no

ENSG00000128050	ENSG00000128050	PAICS	4:57301906-57327534	q1	q2	OK	130.942	148.617	0.182666	0.381074	0.56875	0.99995	no

ENSG00000128052	ENSG00000128052	KDR	4:55919226-55991756	q1	q2	OK	4.23727	6.27366	0.566171	1.01213	0.10505	0.99995	no

ENSG00000128059	ENSG00000128059	PPAT	4:57259527-57301781	q1	q2	OK	16.8093	19.7177	0.230229	0.471495	0.44235	0.99995	no

ENSG00000128159	ENSG00000128159	TUBGCP6	22:50656117-50683421	q1	q2	OK	17.2406	14.6635	-0.233581	-0.467776	0.474	0.99995	no

ENSG00000128165	ENSG00000128165	ADM2	22:50919984-50924869	q1	q2	OK	1.68837	1.50021	-0.17047	-0.238522	0.7241	0.99995	no

ENSG00000128185	ENSG00000128185	DGCR6L	22:20301798-20307603	q1	q2	OK	27.8369	22.1264	-0.33123	-0.636266	0.3725	0.99995	no

ENSG00000128191	ENSG00000128191	DGCR8	22:20067754-20114878	q1	q2	OK	37.7067	31.9773	-0.237774	-0.21069	0.62505	0.99995	no

ENSG00000128203	ENSG00000128203	ASPHD2	22:26825238-26879803	q1	q2	OK	5.82394	6.14834	0.0782002	0.083112	0.9092	0.99995	no

ENSG00000128218	ENSG00000128218	VPREB3	22:24094929-24096655	q1	q2	NOTEST	0	0	0	0	1	1	no

ENSG00000128228	ENSG00000128228	SDF2L1	22:21996549-21998587	q1	q2	OK	26.5917	18.3835	-0.532561	-0.905481	0.1873	0.99995	no

ENSG00000128242	ENSG00000128242	GAL3ST1	22:30950621-30970574	q1	q2	NOTEST	0.217034	0.0914611	-1.24669	0	1	1	no

ENSG00000128245	ENSG00000128245	YWHAH	22:32329506-32353590	q1	q2	OK	119.607	126.095	0.0761995	0.165202	0.8075	0.99995	no

ENSG00000128250	ENSG00000128250	RFPL1	22:29832817-29874164	q1	q2	NOTEST	0.0808725	0	#NAME?	0	1	1	no

ENSG00000128253	ENSG00000128253	RFPL2	22:32586421-32600718	q1	q2	NOTEST	0.0250065	0.0731148	1.54786	0	1	1	no

ENSG00000128254	ENSG00000128254	C22orf24	22:32329506-32353590	q1	q2	OK	1.43815	1.26863	-0.180947	-0.033568	0.8827	0.99995	no

ENSG00000128262	ENSG00000128262	POM121L9P	22:24647795-24661493	q1	q2	NOTEST	0.0795965	0.0976413	0.294785	0	1	1	no

ENSG00000128266	ENSG00000128266	GNAZ	22:23401592-23487208	q1	q2	OK	10.0997	9.3149	-0.116708	-0.216173	0.73055	0.99995	no

ENSG00000128268	ENSG00000128268	MGAT3	22:39853348-39888199	q1	q2	OK	13.8205	13.1654	-0.0700572	-0.154801	0.8099	0.99995	no

ENSG00000128271	ENSG00000128271	ADORA2A	22:24666785-24924358	q1	q2	OK	2.88966	1.9928	-0.5361	-0.170889	0.74725	0.99995	no

ENSG00000128272	ENSG00000128272	ATF4	22:39915699-39918691	q1	q2	OK	296.241	295.526	-0.00348889	-0.00835132	0.9916	0.99995	no

ENSG00000128274	ENSG00000128274	A4GALT	22:43088126-43117304	q1	q2	OK	1.51855	1.07186	-0.502574	-0.430153	0.48235	0.99995	no

ENSG00000128276	ENSG00000128276	RFPL3	22:32750871-32781618	q1	q2	NOTEST	0.0768878	0.0453223	-0.762535	0	1	1	no

ENSG00000128283	ENSG00000128283	CDC42EP1	22:37956453-37965412	q1	q2	OK	10.9426	10.7529	-0.025229	-0.0475906	0.9417	0.99995	no

ENSG00000128284	ENSG00000128284	APOL3	22:36536371-36562225	q1	q2	NOTEST	0.175113	0.0757275	-1.20939	0	1	1	no

ENSG00000128285	ENSG00000128285	MCHR1	22:41074753-41078818	q1	q2	OK	0.505778	0.529797	0.0669373	0.0507779	0.92685	0.99995	no

ENSG00000128294	ENSG00000128294	TPST2	22:26921457-26992681	q1	q2	OK	7.83248	7.7593	-0.0135434	-0.0215621	0.97435	0.99995	no

ENSG00000128298	ENSG00000128298	BAIAP2L2	22:38480895-38506677	q1	q2	NOTEST	0.332172	0.0594752	-2.48157	0	1	1	no

ENSG00000128309	ENSG00000128309	MPST	22:37406899-37425863	q1	q2	OK	38.6821	34.377	-0.170224	-0.320826	0.6514	0.99995	no

ENSG00000128310	ENSG00000128310	GALR3	22:38219388-38221502	q1	q2	NOTEST	0.0771493	0	#NAME?	0	1	1	no

ENSG00000128311	ENSG00000128311	TST	22:37406899-37425863	q1	q2	OK	9.3329	8.23723	-0.180165	-0.160648	0.81745	0.99995	no

ENSG00000128313	ENSG00000128313	APOL5	22:36113918-36125530	q1	q2	NOTEST	0	0	0	0	1	1	no

ENSG00000128322	ENSG00000128322	IGLL1	22:23909250-23922495	q1	q2	NOTEST	0.101575	0	#NAME?	0	1	1	no

ENSG00000128335	ENSG00000128335	APOL2	22:36622255-36636000	q1	q2	OK	7.23386	5.88891	-0.296766	-0.422867	0.4779	0.99995	no

ENSG00000128340	ENSG00000128340	RAC2	22:37621300-37640488	q1	q2	NOTEST	0.12876	0.0635612	-1.01847	0	1	1	no

ENSG00000128342	ENSG00000128342	LIF	22:30635182-30642987	q1	q2	OK	1.69735	1.70938	0.0101912	0.0110292	0.98395	0.99995	no

ENSG00000128346	ENSG00000128346	C22orf23	22:38339527-38437922	q1	q2	OK	3.59036	3.43832	-0.0624264	-0.0455551	0.94705	0.99995	no

ENSG00000128383	ENSG00000128383	APOBEC3A	22:39348745-39359188	q1	q2	NOTEST	0	0	0	0	1	1	no

ENSG00000128394	ENSG00000128394	APOBEC3F	22:39436608-39483748	q1	q2	OK	1.00103	1.10731	0.145567	0.0986157	0.86185	0.99995	no

ENSG00000128408	ENSG00000128408	RIBC2	22:45809571-45828376	q1	q2	NOTEST	0.076682	0.0735195	-0.0607604	0	1	1	no

ENSG00000128422	ENSG00000128422	KRT17	17:39775688-39943183	q1	q2	NOTEST	0.0358466	0.0348652	-0.0400478	0	1	1	no

ENSG00000128438	ENSG00000128438	TBC1D27	17:16826228-16875432	q1	q2	NOTEST	0.00511616	0	#NAME?	0	1	1	no

ENSG00000128463	ENSG00000128463	EMC4	15:34517199-34522357	q1	q2	OK	84.9317	81.922	-0.0520527	-0.120004	0.8623	0.99995	no

ENSG00000128482	ENSG00000128482	RNF112	17:19314437-19320589	q1	q2	OK	0.923675	0.432029	-1.09626	-0.659944	0.234	0.99995	no

ENSG00000128487	ENSG00000128487	SPECC1	17:19912656-20306870	q1	q2	OK	23.8174	24.9295	0.0658423	0.113478	0.85995	0.99995	no

ENSG00000128510	ENSG00000128510	CPA4	7:129932973-129964020	q1	q2	OK	1.13872	1.43618	0.334826	0.265073	0.56635	0.99995	no

ENSG00000128512	ENSG00000128512	DOCK4	7:111366165-111846466	q1	q2	OK	10.3027	11.5609	0.166227	0.231699	0.62315	0.99995	no

ENSG00000128513	ENSG00000128513	POT1	7:124462439-125019375	q1	q2	OK	12.4444	11.9922	-0.0533954	-0.0676356	0.89055	0.99995	no

ENSG00000128519	ENSG00000128519	TAS2R16	7:122634758-122635754	q1	q2	NOTEST	0	0	0	0	1	1	no

ENSG00000128524	ENSG00000128524	ATP6V1F	7:128470430-128550773	q1	q2	OK	99.5918	93.8263	-0.0860344	-0.108826	0.86465	0.99995	no

ENSG00000128534	ENSG00000128534	NAA38	7:117824085-117832878	q1	q2	OK	33.6081	38.0546	0.17926	0.320768	0.5845	0.99995	no

ENSG00000128536	ENSG00000128536	CDHR3	7:105517241-105676877	q1	q2	OK	3.14949	2.18202	-0.529453	-0.42426	0.4267	0.99995	no

ENSG00000128563	ENSG00000128563	PRKRIP1	7:102004318-102067123	q1	q2	OK	0.492359	5.47211	3.47432	2.04504	0.0006	0.437275	no

ENSG00000128564	ENSG00000128564	VGF	7:100805789-100808874	q1	q2	OK	9.31857	12.7709	0.454678	0.879587	0.2132	0.99995	no

ENSG00000128567	ENSG00000128567	PODXL	7:131185020-131242976	q1	q2	OK	35.3473	32.6381	-0.11504	-0.209869	0.7181	0.99995	no

ENSG00000128573	ENSG00000128573	FOXP2	7:113726381-114333827	q1	q2	OK	10.1558	9.42392	-0.107906	-0.0983797	0.7651	0.99995	no

ENSG00000128578	ENSG00000128578	STRIP2	7:129074273-129128240	q1	q2	OK	3.87612	4.95629	0.354648	0.525237	0.36725	0.99995	no

ENSG00000128581	ENSG00000128581	RABL5	7:100956974-100965104	q1	q2	OK	32.1967	34.8637	0.114813	0.218893	0.75635	0.99995	no

ENSG00000128585	ENSG00000128585	MKLN1	7:130626518-131182453	q1	q2	OK	15.1589	17.0858	0.172627	0.237721	0.6027	0.99995	no

ENSG00000128590	ENSG00000128590	DNAJB9	7:108110865-108215294	q1	q2	OK	16.9935	14.0284	-0.276631	-0.305746	0.6153	0.99995	no

ENSG00000128591	ENSG00000128591	FLNC	7:128470430-128550773	q1	q2	OK	5.50156	5.49987	-0.000442481	-0.000563615	0.9992	0.99995	no

ENSG00000128594	ENSG00000128594	LRRC4	7:127292233-127732661	q1	q2	OK	11.801	11.6595	-0.0174098	-0.0156486	0.98215	0.99995	no

ENSG00000128595	ENSG00000128595	CALU	7:128379345-128411861	q1	q2	OK	132.609	142.908	0.107899	0.240754	0.7217	0.99995	no

ENSG00000128596	ENSG00000128596	CCDC136	7:128430810-128462186	q1	q2	OK	22.6343	23.5344	0.0562586	0.0739533	0.9034	0.99995	no

ENSG00000128602	ENSG00000128602	SMO	7:128828712-128853386	q1	q2	OK	45.6701	41.277	-0.145912	-0.296221	0.6101	0.99995	no

ENSG00000128604	ENSG00000128604	IRF5	7:128577665-128590089	q1	q2	NOTEST	0.100528	0.0417205	-1.26877	0	1	1	no

ENSG00000128606	ENSG00000128606	LRRC17	7:102453307-102715286	q1	q2	OK	46.3661	50.8371	0.132809	0.290412	0.6504	0.99995	no

ENSG00000128607	ENSG00000128607	KLHDC10	7:129710349-129775560	q1	q2	OK	12.8545	13.1466	0.0324124	0.0462713	0.9177	0.99995	no

ENSG00000128609	ENSG00000128609	NDUFA5	7:123177050-123198309	q1	q2	OK	52.981	52.4751	-0.0138442	-0.0300874	0.96355	0.99995	no

ENSG00000128610	ENSG00000128610	FEZF1	7:121941447-121950745	q1	q2	NOTEST	0.0214386	0	#NAME?	0	1	1	no

ENSG00000128617	ENSG00000128617	OPN1SW	7:128412544-128415844	q1	q2	NOTEST	0.326689	0.479677	0.554146	0	1	1	no

ENSG00000128626	ENSG00000128626	MRPS12	19:39405905-39466550	q1	q2	OK	16.3025	14.8207	-0.137479	-0.15956	0.8216	0.99995	no

ENSG00000128641	ENSG00000128641	MYO1B	2:192093890-192290115	q1	q2	OK	67.7962	76.2322	0.169196	0.349595	0.57015	0.99995	no

ENSG00000128645	ENSG00000128645	HOXD1	2:177037922-177055688	q1	q2	OK	0.943863	1.33417	0.499294	0.369168	0.5677	0.99995	no

ENSG00000128652	ENSG00000128652	HOXD3	2:176986338-177037830	q1	q2	OK	0.768977	0.76097	-0.0151007	-0.00843523	0.989	0.99995	no

ENSG00000128654	ENSG00000128654	MTX2	2:177134122-177202753	q1	q2	OK	31.5542	32.9491	0.0624096	0.133883	0.8484	0.99995	no

ENSG00000128655	ENSG00000128655	PDE11A	2:178487979-178973066	q1	q2	NOTEST	0.370004	0.338354	-0.129008	0	1	1	no

ENSG00000128656	ENSG00000128656	CHN1	2:175664090-175870097	q1	q2	OK	65.661	71.1882	0.116602	0.228597	0.7203	0.99995	no

ENSG00000128683	ENSG00000128683	GAD1	2:171669722-171717661	q1	q2	OK	9.07609	8.63513	-0.0718519	-0.09429	0.86485	0.99995	no

ENSG00000128692	ENSG00000128692	EIF2S2P4	2:171579718-171627276	q1	q2	OK	11.5302	13.9181	0.271542	0.437883	0.5279	0.99995	no

ENSG00000128694	ENSG00000128694	OSGEPL1	2:190539015-190630282	q1	q2	OK	11.6246	10.8683	-0.0970515	-0.159546	0.81935	0.99995	no

ENSG00000128699	ENSG00000128699	ORMDL1	2:190635048-190649097	q1	q2	OK	61.5182	64.4023	0.0660998	0.141949	0.84	0.99995	no

ENSG00000128708	ENSG00000128708	HAT1	2:172640879-172947158	q1	q2	OK	56.4921	60.0818	0.088879	0.179467	0.7942	0.99995	no

ENSG00000128709	ENSG00000128709	HOXD9	2:176986338-177037830	q1	q2	NOTEST	0.26805	0.198569	-0.432859	0	1	1	no

ENSG00000128710	ENSG00000128710	HOXD10	2:176968943-176984670	q1	q2	NOTEST	0	0.0286003	inf	0	1	1	no

ENSG00000128713	ENSG00000128713	HOXD11	2:176968943-176984670	q1	q2	NOTEST	0	0	0	0	1	1	no

ENSG00000128714	ENSG00000128714	HOXD13	2:176957618-176960666	q1	q2	NOTEST	0.0581608	0.023372	-1.31527	0	1	1	no

ENSG00000128731	ENSG00000128731	HERC2	15:28356185-28567298	q1	q2	OK	22.1753	20.1349	-0.139258	-0.134408	0.7934	0.99995	no

ENSG00000128739	ENSG00000128739	SNRPN	15:25068793-25492435	q1	q2	OK	30.6799	27.6679	-0.14908	-0.096009	0.86855	0.99995	no

ENSG00000128789	ENSG00000128789	PSMG2	18:12438888-12725739	q1	q2	OK	46.8174	46.6197	-0.00610422	-0.00669963	0.99235	0.99995	no

ENSG00000128791	ENSG00000128791	TWSG1	18:9334764-9402418	q1	q2	OK	28.9798	31.2593	0.109235	0.260632	0.69915	0.99995	no

ENSG00000128802	ENSG00000128802	GDF2	10:48413091-48416853	q1	q2	NOTEST	0	0	0	0	1	1	no

ENSG00000128805	ENSG00000128805	ARHGAP22	10:49654076-49864310	q1	q2	OK	0.963019	0.826859	-0.219924	-0.188494	0.68565	0.99995	no

ENSG00000128815	ENSG00000128815	WDFY4	10:49892920-50323554	q1	q2	NOTEST	0.00593818	0.00623197	0.069668	0	1	1	no

ENSG00000128829	ENSG00000128829	EIF2AK4	15:40226346-40327797	q1	q2	OK	13.9593	14.8309	0.0873718	0.127571	0.8079	0.99995	no

ENSG00000128833	ENSG00000128833	MYO5C	15:52408770-52587995	q1	q2	OK	1.77252	1.6942	-0.0651983	-0.0385351	0.9145	0.99995	no

ENSG00000128849	ENSG00000128849	CGNL1	15:57668164-57842925	q1	q2	OK	2.69919	3.349	0.311207	0.565089	0.3827	0.99995	no

ENSG00000128872	ENSG00000128872	TMOD2	15:52015207-52108565	q1	q2	OK	4.26271	4.84693	0.185303	0.281196	0.5521	0.99995	no

ENSG00000128881	ENSG00000128881	TTBK2	15:43030931-43213007	q1	q2	OK	7.48589	7.16958	-0.0622843	-0.0765994	0.84575	0.99995	no

ENSG00000128886	ENSG00000128886	ELL3	15:44019115-44095241	q1	q2	OK	0.572786	0.544846	-0.0721464	-0.00307061	0.9511	0.99995	no

ENSG00000128891	ENSG00000128891	C15orf57	15:40780239-40857256	q1	q2	OK	12.4902	12.7337	0.0278635	0.0270881	0.9528	0.99995	no

ENSG00000128908	ENSG00000128908	INO80	15:41271077-41408552	q1	q2	OK	8.14319	9.18967	0.174419	0.257702	0.593	0.99995	no

ENSG00000128915	ENSG00000128915	NARG2	15:60711807-60771359	q1	q2	OK	19.8875	23.1289	0.217829	0.244285	0.68585	0.99995	no

ENSG00000128917	ENSG00000128917	DLL4	15:41221537-41231237	q1	q2	OK	2.25954	2.28465	0.0159434	0.0217187	0.97505	0.99995	no

ENSG00000128918	ENSG00000128918	ALDH1A2	15:58245621-58861151	q1	q2	OK	19.8994	19.2011	-0.0515412	-0.0894627	0.88105	0.99995	no

ENSG00000128923	ENSG00000128923	FAM63B	15:59063390-59389618	q1	q2	OK	6.12105	6.7527	0.141685	0.1418	0.8367	0.99995	no

ENSG00000128928	ENSG00000128928	IVD	15:40697685-40728146	q1	q2	OK	23.1717	23.8439	0.0412605	0.0574056	0.9131	0.99995	no

ENSG00000128944	ENSG00000128944	KNSTRN	15:40674921-40686447	q1	q2	OK	40.6357	44.692	0.137267	0.295892	0.64295	0.99995	no

ENSG00000128951	ENSG00000128951	DUT	15:48604549-48635570	q1	q2	OK	75.9445	69.3029	-0.13203	-0.232284	0.7121	0.99995	no

ENSG00000128965	ENSG00000128965	CHAC1	15:41245159-41248710	q1	q2	OK	13.9613	10.3083	-0.437631	-0.715768	0.30435	0.99995	no

ENSG00000128973	ENSG00000128973	CLN6	15:68346516-68549549	q1	q2	OK	16.4304	16.2364	-0.0171377	-0.0211143	0.97485	0.99995	no

ENSG00000128989	ENSG00000128989	ARPP19	15:52839241-52862080	q1	q2	OK	83.8204	88.8189	0.0835659	0.127833	0.8121	0.99995	no

ENSG00000129003	ENSG00000129003	VPS13C	15:62144587-62352672	q1	q2	OK	12.8929	14.6249	0.181849	0.232688	0.67515	0.99995	no

ENSG00000129007	ENSG00000129007	CALML4	15:68346516-68549549	q1	q2	OK	11.6344	12.1405	0.0614289	0.0474852	0.92775	0.99995	no

ENSG00000129009	ENSG00000129009	ISLR	15:74445140-74471567	q1	q2	OK	17.5984	15.7692	-0.158336	-0.288007	0.67715	0.99995	no

ENSG00000129028	ENSG00000129028	THAP10	15:71123862-71342414	q1	q2	OK	6.05083	5.9813	-0.0166754	-0.012235	0.982	0.99995	no

ENSG00000129038	ENSG00000129038	LOXL1	15:74200423-74244478	q1	q2	OK	8.29794	6.20521	-0.419274	-0.488818	0.45785	0.99995	no

ENSG00000129048	ENSG00000129048	ACKR4	3:132276981-132593067	q1	q2	NOTEST	0.0999417	0.153512	0.61919	0	1	1	no

ENSG00000129055	ENSG00000129055	ANAPC13	3:134196547-134293859	q1	q2	OK	59.27	64.5951	0.124123	0.232627	0.7404	0.99995	no

ENSG00000129071	ENSG00000129071	MBD4	3:129149786-129158878	q1	q2	OK	22.3402	20.5123	-0.123154	-0.19924	0.711	0.99995	no

ENSG00000129083	ENSG00000129083	COPB1	11:14464985-14665181	q1	q2	OK	59.4702	66.938	0.17066	0.289005	0.6213	0.99995	no

ENSG00000129084	ENSG00000129084	PSMA1	11:14464985-14665181	q1	q2	OK	123.928	127.526	0.0412874	0.0580033	0.92805	0.99995	no

ENSG00000129103	ENSG00000129103	SUMF2	7:56131694-56148363	q1	q2	OK	42.4627	35.2552	-0.26836	-0.518194	0.40955	0.99995	no

ENSG00000129116	ENSG00000129116	PALLD	4:169277885-169931426	q1	q2	OK	42.8376	42.8924	0.0018468	0.00324575	0.99465	0.99995	no

ENSG00000129128	ENSG00000129128	SPCS3	4:177229418-177253396	q1	q2	OK	39.5015	42.65	0.110637	0.24537	0.6993	0.99995	no

ENSG00000129151	ENSG00000129151	BBOX1	11:27062271-27241660	q1	q2	OK	0.688903	0.538594	-0.355104	-0.23602	0.73785	0.99995	no

ENSG00000129152	ENSG00000129152	MYOD1	11:17741114-17743678	q1	q2	NOTEST	0	0	0	0	1	1	no

ENSG00000129158	ENSG00000129158	SERGEF	11:17809594-18063973	q1	q2	OK	13.2628	9.22293	-0.524091	-0.579114	0.3368	0.99995	no

ENSG00000129159	ENSG00000129159	KCNC1	11:17756358-17804602	q1	q2	OK	5.77891	5.25094	-0.138223	-0.220317	0.6961	0.99995	no

ENSG00000129167	ENSG00000129167	TPH1	11:17809594-18063973	q1	q2	OK	1.30692	1.5487	0.244886	0.191518	0.688	0.99995	no

ENSG00000129170	ENSG00000129170	CSRP3	11:19203577-19302973	q1	q2	NOTEST	0	0	0	0	1	1	no

ENSG00000129173	ENSG00000129173	E2F8	11:19203577-19302973	q1	q2	OK	3.48994	3.09323	-0.174089	-0.157537	0.72445	0.99995	no

ENSG00000129187	ENSG00000129187	DCTD	4:183811212-183839089	q1	q2	OK	44.0287	43.1973	-0.0275017	-0.0567904	0.9248	0.99995	no

ENSG00000129194	ENSG00000129194	SOX15	17:7465191-7536700	q1	q2	OK	2.06995	1.76247	-0.232001	-0.0216533	0.8423	0.99995	no

ENSG00000129195	ENSG00000129195	FAM64A	17:6347734-6459814	q1	q2	OK	52.052	52.7759	0.0199237	0.0421997	0.95225	0.99995	no

ENSG00000129197	ENSG00000129197	RPAIN	17:5185557-5372380	q1	q2	OK	63.8911	64.1288	0.00535553	0.00427222	0.9928	0.99995	no

ENSG00000129204	ENSG00000129204	USP6	17:5008835-5078329	q1	q2	NOTEST	0.232898	0.311562	0.419822	0	1	1	no

ENSG00000129214	ENSG00000129214	SHBG	17:7465191-7536700	q1	q2	OK	1.15749	1.30102	0.168644	0.00989454	0.8694	0.99995	no

ENSG00000129219	ENSG00000129219	PLD2	17:4710390-4726729	q1	q2	OK	14.5615	14.8892	0.0321131	0.0339895	0.9444	0.99995	no

ENSG00000129221	ENSG00000129221	AIPL1	17:6297012-6338519	q1	q2	NOTEST	0.0236836	0.0088999	-1.41203	0	1	1	no

ENSG00000129226	ENSG00000129226	CD68	17:7465191-7536700	q1	q2	OK	0.422359	0.63121	0.579652	0.0235267	0.767	0.99995	no

ENSG00000129235	ENSG00000129235	TXNDC17	17:6481467-6554954	q1	q2	OK	121.034	120.145	-0.0106324	-0.0121498	0.9863	0.99995	no

ENSG00000129244	ENSG00000129244	ATP1B2	17:7549944-7561086	q1	q2	OK	8.09647	6.14985	-0.396742	-0.746479	0.27295	0.99995	no

ENSG00000129245	ENSG00000129245	FXR2	17:7465191-7536700	q1	q2	OK	29.0885	27.4597	-0.0831344	-0.0296017	0.9264	0.99995	no

ENSG00000129250	ENSG00000129250	KIF1C	17:4901242-4931696	q1	q2	OK	20.3318	21.3539	0.0707604	0.0846478	0.89495	0.99995	no

ENSG00000129255	ENSG00000129255	MPDU1	17:7465191-7536700	q1	q2	OK	63.5488	63.6434	0.00214491	0.00108215	0.9975	0.99995	no

ENSG00000129270	ENSG00000129270	MMP28	17:34083267-34122711	q1	q2	NOTEST	0.0137401	0.0266715	0.956905	0	1	1	no

ENSG00000129277	ENSG00000129277	CCL4	17:34430982-34433014	q1	q2	NOTEST	0	0	0	0	1	1	no

ENSG00000129282	ENSG00000129282	MRM1	17:34958000-34965407	q1	q2	NOTEST	0.0668868	0	#NAME?	0	1	1	no

ENSG00000129292	ENSG00000129292	PHF20L1	8:133787617-133861052	q1	q2	OK	33.7617	36.6596	0.118802	0.22111	0.69365	0.99995	no

ENSG00000129295	ENSG00000129295	LRRC6	8:133584319-133687838	q1	q2	OK	2.26016	3.03249	0.424075	0.422882	0.4384	0.99995	no

ENSG00000129315	ENSG00000129315	CCNT1	12:49082246-49110681	q1	q2	OK	14.9628	14.9937	0.00297063	0.00498069	0.99205	0.99995	no

ENSG00000129317	ENSG00000129317	PUS7L	12:44122409-44152620	q1	q2	NOTEST	0.246024	0.133002	-0.887353	0	1	1	no

ENSG00000129347	ENSG00000129347	KRI1	19:10654570-10676713	q1	q2	OK	14.0821	13.4676	-0.0643658	-0.102502	0.8658	0.99995	no

ENSG00000129351	ENSG00000129351	ILF3	19:10764936-10803093	q1	q2	OK	234.15	224.414	-0.0612709	-0.097236	0.8445	0.99995	no

ENSG00000129353	ENSG00000129353	SLC44A2	19:10713132-10755235	q1	q2	OK	45.9931	43.4293	-0.0827485	-0.185038	0.7812	0.99995	no

ENSG00000129354	ENSG00000129354	AP1M2	19:10683346-10697991	q1	q2	NOTEST	0.0810499	0.0787388	-0.0417374	0	1	1	no

ENSG00000129355	ENSG00000129355	CDKN2D	19:10677137-10679735	q1	q2	OK	12.0339	11.4214	-0.0753629	-0.122529	0.8597	0.99995	no

ENSG00000129422	ENSG00000129422	MTUS1	8:17433941-17658426	q1	q2	OK	27.1059	30.3805	0.164541	0.300901	0.57735	0.99995	no

ENSG00000129437	ENSG00000129437	KLK14	19:51580751-51587502	q1	q2	NOTEST	0.310896	0.359839	0.210922	0	1	1	no

ENSG00000129450	ENSG00000129450	SIGLEC9	19:51628164-51639908	q1	q2	NOTEST	0	0	0	0	1	1	no

ENSG00000129451	ENSG00000129451	KLK10	19:51515994-51523431	q1	q2	NOTEST	0.0203868	0	#NAME?	0	1	1	no

ENSG00000129455	ENSG00000129455	KLK8	19:51446558-51515385	q1	q2	NOTEST	0	0	0	0	1	1	no

ENSG00000129460	ENSG00000129460	NGDN	14:23938896-23979071	q1	q2	OK	35.5629	36.7818	0.0486189	0.0955147	0.88505	0.99995	no

ENSG00000129465	ENSG00000129465	RIPK3	14:24787554-24809251	q1	q2	NOTEST	0.140344	0	#NAME?	0	1	1	no

ENSG00000129467	ENSG00000129467	ADCY4	14:24787554-24809251	q1	q2	OK	0.569614	0.322696	-0.819808	-0.358938	0.21875	0.99995	no

ENSG00000129472	ENSG00000129472	RAB2B	14:21927178-21979517	q1	q2	OK	19.0107	19.4759	0.0348784	0.0321018	0.95185	0.99995	no

ENSG00000129473	ENSG00000129473	BCL2L2	14:23654524-23795394	q1	q2	OK	14.8463	15.6063	0.0720238	0.0480149	0.9263	0.99995	no

ENSG00000129474	ENSG00000129474	AJUBA	14:23398817-23451851	q1	q2	OK	32.024	33.8038	0.0780323	0.121981	0.8212	0.99995	no

ENSG00000129480	ENSG00000129480	DTD2	14:31717833-31926716	q1	q2	OK	10.4742	11.6037	0.147741	0.142977	0.8222	0.99995	no

ENSG00000129484	ENSG00000129484	PARP2	14:20811206-20826064	q1	q2	OK	43.1053	47.365	0.135958	0.268587	0.6701	0.99995	no

ENSG00000129493	ENSG00000129493	HEATR5A	14:31717833-31926716	q1	q2	OK	17.4344	15.3095	-0.187508	-0.157322	0.7181	0.99995	no

ENSG00000129514	ENSG00000129514	FOXA1	14:38059188-38672128	q1	q2	NOTEST	0.0336825	0.00863622	-1.96353	0	1	1	no

ENSG00000129515	ENSG00000129515	SNX6	14:35025979-35099389	q1	q2	OK	42.3785	45.9333	0.116206	0.256491	0.68845	0.99995	no

ENSG00000129518	ENSG00000129518	EAPP	14:34985134-35008916	q1	q2	OK	23.1906	21.0741	-0.138074	-0.268093	0.6876	0.99995	no

ENSG00000129521	ENSG00000129521	EGLN3	14:34393436-34931980	q1	q2	OK	1.25331	1.246	-0.00843358	-0.00346126	0.99455	0.99995	no

ENSG00000129534	ENSG00000129534	MIS18BP1	14:45672392-45722743	q1	q2	OK	26.0441	27.064	0.0554181	0.0817889	0.89075	0.99995	no

ENSG00000129535	ENSG00000129535	NRL	14:24549315-24602058	q1	q2	OK	0.483245	0.327331	-0.562005	-0.111436	0.56945	0.99995	no

ENSG00000129538	ENSG00000129538	RNASE1	14:21269386-21271437	q1	q2	OK	0.276944	0.671171	1.27709	0.796208	0.14985	0.99995	no

ENSG00000129559	ENSG00000129559	NEDD8	14:24641061-24708448	q1	q2	OK	156.32	157.995	0.0153786	0.020152	0.9769	0.99995	no

ENSG00000129562	ENSG00000129562	DAD1	14:23033804-23058175	q1	q2	OK	150.072	149.444	-0.00605175	-0.0142689	0.98355	0.99995	no

ENSG00000129566	ENSG00000129566	TEP1	14:20833825-20881588	q1	q2	OK	7.65807	8.0405	0.0703046	0.0778815	0.8849	0.99995	no

ENSG00000129595	ENSG00000129595	EPB41L4A	5:111478137-111755013	q1	q2	OK	5.55022	5.88399	0.0842487	0.0821745	0.86085	0.99995	no

ENSG00000129596	ENSG00000129596	CDO1	5:115140429-115152651	q1	q2	OK	33.7441	30.2092	-0.15965	-0.32377	0.59665	0.99995	no

ENSG00000129625	ENSG00000129625	REEP5	5:112043194-112258236	q1	q2	OK	19.3764	17.9604	-0.109478	-0.10273	0.86775	0.99995	no

ENSG00000129636	ENSG00000129636	ITFG1	16:47178233-47735434	q1	q2	OK	34.9802	34.2871	-0.028871	-0.0341076	0.9572	0.99995	no

ENSG00000129646	ENSG00000129646	QRICH2	17:74270129-74303761	q1	q2	OK	2.84157	2.02431	-0.489259	-0.661098	0.28285	0.99995	no

ENSG00000129654	ENSG00000129654	FOXJ1	17:74132413-74236454	q1	q2	OK	4.01493	2.95195	-0.443707	-0.30503	0.651	0.99995	no

ENSG00000129657	ENSG00000129657	SEC14L1	17:75082797-75213179	q1	q2	OK	97.1453	120.954	0.316241	0.490316	0.27615	0.99995	no

ENSG00000129667	ENSG00000129667	RHBDF2	17:74466972-74497872	q1	q2	OK	0.6953	0.587641	-0.242702	-0.131246	0.66715	0.99995	no

ENSG00000129673	ENSG00000129673	AANAT	17:74449432-74466199	q1	q2	NOTEST	0.0420842	0.0847303	1.0096	0	1	1	no

ENSG00000129675	ENSG00000129675	ARHGEF6	X:135747705-135864247	q1	q2	OK	15.7772	12.0674	-0.386722	-0.880747	0.20415	0.99995	no

ENSG00000129680	ENSG00000129680	MAP7D3	X:135295380-135338641	q1	q2	OK	5.1682	5.00867	-0.0452357	-0.0745177	0.90675	0.99995	no

ENSG00000129682	ENSG00000129682	FGF13	X:137713734-138304939	q1	q2	OK	26.4844	26.482	-0.000130177	-0.000250932	0.99985	0.99995	no

ENSG00000129691	ENSG00000129691	ASH2L	8:37962759-38070819	q1	q2	OK	29.5253	32.2333	0.126599	0.180007	0.76775	0.99995	no

ENSG00000129696	ENSG00000129696	TTI2	8:33228341-33371119	q1	q2	OK	10.0231	9.05752	-0.14614	-0.176287	0.8115	0.99995	no

ENSG00000129744	ENSG00000129744	ART1	11:3666357-3685646	q1	q2	NOTEST	0	0	0	0	1	1	no

ENSG00000129749	ENSG00000129749	CHRNA10	11:3686816-3847601	q1	q2	OK	1.84648	1.27237	-0.537259	-0.136913	0.67315	0.99995	no

ENSG00000129757	ENSG00000129757	CDKN1C	11:2904442-2907111	q1	q2	OK	33.9302	28.2998	-0.261781	-0.556206	0.43665	0.99995	no

ENSG00000129810	ENSG00000129810	SGOL1	3:20202084-20227919	q1	q2	OK	15.1003	16.7042	0.145631	0.285186	0.6799	0.99995	no

ENSG00000129816	ENSG00000129816	TTTY1B	Y:6258471-6292186	q1	q2	NOTEST	0	0	0	0	1	1	no

ENSG00000129824	ENSG00000129824	RPS4Y1	Y:2709526-2800041	q1	q2	OK	230.261	234.348	0.0253812	0.061302	0.9291	0.99995	no

ENSG00000129845	ENSG00000129845	TTTY1	Y:9578192-9611898	q1	q2	NOTEST	0	0	0	0	1	1	no

ENSG00000129862	ENSG00000129862	VCY1B	Y:16168096-16168838	q1	q2	NOTEST	0	0	0	0	1	1	no

ENSG00000129864	ENSG00000129864	VCY	Y:16097651-16098393	q1	q2	NOTEST	0	0	0	0	1	1	no

ENSG00000129873	ENSG00000129873	CDY2B	Y:19989289-19992100	q1	q2	NOTEST	0	0	0	0	1	1	no

ENSG00000129910	ENSG00000129910	CDH15	16:89238174-89261900	q1	q2	NOTEST	0.108127	0.0712849	-0.601059	0	1	1	no

ENSG00000129911	ENSG00000129911	KLF16	19:1852397-1863567	q1	q2	OK	5.23208	4.71112	-0.151312	-0.245897	0.72385	0.99995	no

ENSG00000129925	ENSG00000129925	TMEM8A	16:420772-442960	q1	q2	OK	25.5392	22.6246	-0.174818	-0.311512	0.57735	0.99995	no

ENSG00000129932	ENSG00000129932	DOHH	19:3490818-3500938	q1	q2	OK	7.88593	7.15798	-0.139727	-0.213433	0.7567	0.99995	no

ENSG00000129933	ENSG00000129933	MAU2	19:19386826-19469563	q1	q2	OK	26.3973	23.3084	-0.179543	-0.235402	0.58715	0.99995	no

ENSG00000129946	ENSG00000129946	SHC2	19:416582-460996	q1	q2	OK	24.8245	20.008	-0.311187	-0.657982	0.3468	0.99995	no

ENSG00000129951	ENSG00000129951	LPPR3	19:812517-821967	q1	q2	OK	40.2156	30.4032	-0.403529	-0.790129	0.2185	0.99995	no

ENSG00000129965	ENSG00000129965	INS-IGF2	11:2150341-2182571	q1	q2	NOTEST	0	0	0	0	1	1	no

ENSG00000129968	ENSG00000129968	ABHD17A	19:1876808-1885546	q1	q2	OK	25.0343	20.5396	-0.285495	-0.551507	0.4156	0.99995	no

ENSG00000129988	ENSG00000129988	LBP	20:36974758-37005665	q1	q2	NOTEST	0	0.0130772	inf	0	1	1	no

ENSG00000129990	ENSG00000129990	SYT5	19:55663136-55691809	q1	q2	OK	2.95195	2.22154	-0.410107	-0.326947	0.5393	0.99995	no

ENSG00000129991	ENSG00000129991	TNNI3	19:55663136-55691809	q1	q2	OK	0.914051	0.771874	-0.24391	-0.0541586	0.8174	0.99995	no

ENSG00000129993	ENSG00000129993	CBFA2T3	16:88941265-89043612	q1	q2	OK	3.60263	3.02595	-0.251664	-0.316221	0.58745	0.99995	no

ENSG00000130005	ENSG00000130005	GAMT	19:1397090-1401569	q1	q2	OK	21.685	17.9711	-0.27102	-0.485489	0.48605	0.99995	no

ENSG00000130021	ENSG00000130021	HDHD1	X:6966960-7066231	q1	q2	OK	13.8358	14.6934	0.0867598	0.175307	0.8014	0.99995	no

ENSG00000130023	ENSG00000130023	ERMARD	6:170151717-170181680	q1	q2	OK	14.4449	12.6731	-0.188788	-0.272011	0.63515	0.99995	no

ENSG00000130024	ENSG00000130024	PHF10	6:170102232-170124151	q1	q2	OK	57.9541	61.1847	0.0782616	0.136545	0.8333	0.99995	no

ENSG00000130032	ENSG00000130032	PRRG3	X:150863595-150874396	q1	q2	OK	0.681884	0.51397	-0.407843	-0.289986	0.52145	0.99995	no

ENSG00000130035	ENSG00000130035	GALNT8	12:4671369-4960277	q1	q2	OK	4.75088	1.92602	-1.30257	-0.39082	0.43	0.99995	no

ENSG00000130037	ENSG00000130037	KCNA5	12:5153084-5155949	q1	q2	NOTEST	0.0647427	0.0536569	-0.270952	0	1	1	no

ENSG00000130038	ENSG00000130038	EFCAB4B	12:3715798-3873985	q1	q2	OK	0.643812	0.483206	-0.414001	-0.324163	0.6046	0.99995	no

ENSG00000130045	ENSG00000130045	NXNL2	9:91150015-91199189	q1	q2	NOTEST	0.0484494	0.0784088	0.694536	0	1	1	no

ENSG00000130052	ENSG00000130052	STARD8	X:67867507-67945684	q1	q2	OK	0.513157	0.482783	-0.0880268	-0.0786467	0.8915	0.99995	no

ENSG00000130054	ENSG00000130054	FAM155B	X:68725083-68752351	q1	q2	OK	2.62491	2.35456	-0.156813	-0.24476	0.73485	0.99995	no

ENSG00000130055	ENSG00000130055	GDPD2	X:69642880-69653240	q1	q2	OK	8.27814	6.44321	-0.361528	-0.5892	0.40575	0.99995	no

ENSG00000130066	ENSG00000130066	SAT1	X:23801289-23804343	q1	q2	OK	73.0291	78.4949	0.104127	0.228524	0.7425	0.99995	no

ENSG00000130119	ENSG00000130119	GNL3L	X:54556643-54587504	q1	q2	OK	7.34651	8.0023	0.123356	0.219774	0.754	0.99995	no

ENSG00000130147	ENSG00000130147	SH3BP4	2:235860616-235964358	q1	q2	OK	31.3517	32.0847	0.0333432	0.0762358	0.9039	0.99995	no

ENSG00000130150	ENSG00000130150	MOSPD2	X:14891562-14940449	q1	q2	OK	6.06281	6.51394	0.103543	0.159599	0.78275	0.99995	no

ENSG00000130158	ENSG00000130158	DOCK6	19:11309970-11373157	q1	q2	OK	17.6101	18.756	0.0909469	0.108684	0.8024	0.99995	no

ENSG00000130159	ENSG00000130159	ECSIT	19:11562140-11639989	q1	q2	OK	18.7455	17.3449	-0.112032	-0.102533	0.8635	0.99995	no

ENSG00000130164	ENSG00000130164	LDLR	19:11200037-11266484	q1	q2	OK	42.5274	41.7802	-0.0255758	-0.0317418	0.9452	0.99995	no

ENSG00000130165	ENSG00000130165	ELOF1	19:11661961-11670051	q1	q2	OK	42.1799	37.9163	-0.153738	-0.321082	0.63875	0.99995	no

ENSG00000130167	ENSG00000130167	TSPAN16	19:11406823-11465620	q1	q2	NOTEST	0.0298306	0.0638115	1.09702	0	1	1	no

ENSG00000130173	ENSG00000130173	C19orf80	19:11309970-11373157	q1	q2	NOTEST	0.210712	0	#NAME?	0	1	1	no

ENSG00000130175	ENSG00000130175	PRKCSH	19:11531271-11561783	q1	q2	OK	119.254	101.92	-0.226599	-0.481582	0.40815	0.99995	no

ENSG00000130176	ENSG00000130176	CNN1	19:11649531-11661138	q1	q2	OK	1.92774	1.53113	-0.332316	-0.333774	0.53315	0.99995	no

ENSG00000130177	ENSG00000130177	CDC16	13:115000361-115038198	q1	q2	OK	46.3481	49.1311	0.0841259	0.181168	0.80005	0.99995	no

ENSG00000130182	ENSG00000130182	ZSCAN10	16:3138890-3149318	q1	q2	NOTEST	0.180355	0.0337254	-2.41894	0	1	1	no

ENSG00000130193	ENSG00000130193	THEM6	8:143808620-143818345	q1	q2	OK	4.26138	3.85719	-0.143771	-0.22032	0.75555	0.99995	no

ENSG00000130201	ENSG00000130201	EXOC3L2	19:45574757-45808541	q1	q2	NOTEST	0.208262	0.31563	0.599833	0	1	1	no

ENSG00000130202	ENSG00000130202	PVRL2	19:45349431-45406946	q1	q2	OK	78.2325	76.5409	-0.0315384	-0.0518633	0.9318	0.99995	no

ENSG00000130203	ENSG00000130203	APOE	19:45409010-45412650	q1	q2	OK	16.5873	14.3046	-0.213599	-0.344611	0.61535	0.99995	no

ENSG00000130204	ENSG00000130204	TOMM40	19:45349431-45406946	q1	q2	OK	42.7446	43.4913	0.0249841	0.0343396	0.96	0.99995	no

ENSG00000130208	ENSG00000130208	APOC1	19:45417503-45422606	q1	q2	OK	3.13925	4.08933	0.381444	0.359416	0.59385	0.99995	no

ENSG00000130222	ENSG00000130222	GADD45G	9:92219927-92221470	q1	q2	OK	20.0692	13.9125	-0.528606	-0.917622	0.2023	0.99995	no

ENSG00000130224	ENSG00000130224	LRCH2	X:114345184-114468635	q1	q2	OK	0.874411	0.715603	-0.289152	-0.352486	0.61005	0.99995	no

ENSG00000130226	ENSG00000130226	DPP6	7:153584181-154685995	q1	q2	OK	1.67878	1.24033	-0.436688	-0.370477	0.37105	0.99995	no

ENSG00000130227	ENSG00000130227	XPO7	8:21777179-21864096	q1	q2	OK	30.7895	30.6242	-0.00776485	-0.0153075	0.97865	0.99995	no

ENSG00000130234	ENSG00000130234	ACE2	X:15579155-15620271	q1	q2	NOTEST	0.146389	0.125692	-0.219922	0	1	1	no

ENSG00000130244	ENSG00000130244	FAM98C	19:38893774-38916945	q1	q2	OK	6.19699	4.91729	-0.333706	-0.326681	0.57515	0.99995	no

ENSG00000130254	ENSG00000130254	SAFB2	19:5587009-5668489	q1	q2	OK	35.9571	37.7209	0.0690876	0.0610691	0.884	0.99995	no

ENSG00000130255	ENSG00000130255	RPL36	19:5674957-5720583	q1	q2	OK	288.715	263.437	-0.132186	-0.236094	0.7404	0.99995	no

ENSG00000130270	ENSG00000130270	ATP8B3	19:1782073-1812275	q1	q2	OK	0.576383	0.772557	0.422613	0.357073	0.59275	0.99995	no

ENSG00000130283	ENSG00000130283	GDF1	19:18979360-19007536	q1	q2	OK	7.29345	5.79583	-0.331586	-0.47395	0.49765	0.99995	no

ENSG00000130287	ENSG00000130287	NCAN	19:19322781-19363042	q1	q2	OK	36.4099	31.7	-0.199845	-0.374856	0.47795	0.99995	no

ENSG00000130294	ENSG00000130294	KIF1A	2:241653180-241759725	q1	q2	OK	26.752	24.4708	-0.128584	-0.189381	0.64945	0.99995	no

ENSG00000130299	ENSG00000130299	GTPBP3	19:17445728-17453544	q1	q2	OK	22.3182	20.2883	-0.137573	-0.200385	0.7287	0.99995	no

ENSG00000130300	ENSG00000130300	PLVAP	19:17462256-17488159	q1	q2	NOTEST	0.0789107	0.138403	0.81058	0	1	1	no

ENSG00000130303	ENSG00000130303	BST2	19:17513747-17516457	q1	q2	OK	0.49909	0.22626	-1.14132	-0.578196	0.4036	0.99995	no

ENSG00000130304	ENSG00000130304	SLC27A1	19:17579577-17632097	q1	q2	OK	5.17948	4.84862	-0.0952344	-0.104753	0.8597	0.99995	no

ENSG00000130305	ENSG00000130305	NSUN5	7:72716513-72722864	q1	q2	OK	2.33916	3.60929	0.625724	0.794199	0.2366	0.99995	no

ENSG00000130307	ENSG00000130307	USHBP1	19:17342691-17398455	q1	q2	NOTEST	0.102748	0.388898	1.92028	0	1	1	no

ENSG00000130309	ENSG00000130309	COLGALT1	19:17666402-17693971	q1	q2	OK	27.524	26.9632	-0.0296981	-0.0671861	0.9143	0.99995	no

ENSG00000130311	ENSG00000130311	DDA1	19:17402939-17445638	q1	q2	OK	33.1494	40.0746	0.273707	0.203	0.76375	0.99995	no

ENSG00000130312	ENSG00000130312	MRPL34	19:17402939-17445638	q1	q2	OK	39.0115	31.6162	-0.303238	-0.288552	0.6686	0.99995	no

ENSG00000130313	ENSG00000130313	PGLS	19:17579577-17632097	q1	q2	OK	44.0786	35.7278	-0.303034	-0.556061	0.4399	0.99995	no

ENSG00000130332	ENSG00000130332	LSM7	19:2321515-2355099	q1	q2	OK	139.969	136.873	-0.0322678	-0.0462909	0.94595	0.99995	no

ENSG00000130338	ENSG00000130338	TULP4	6:158733691-158932860	q1	q2	OK	7.08172	7.67586	0.116227	0.27524	0.6917	0.99995	no

ENSG00000130340	ENSG00000130340	SNX9	6:158244295-158366109	q1	q2	OK	9.41736	10.2143	0.117203	0.255538	0.7087	0.99995	no

ENSG00000130347	ENSG00000130347	RTN4IP1	6:107018902-107077373	q1	q2	OK	6.46071	6.73043	0.0590059	0.0918413	0.88915	0.99995	no

ENSG00000130348	ENSG00000130348	QRSL1	6:107077452-107116292	q1	q2	OK	9.91678	11.2549	0.182606	0.303834	0.5758	0.99995	no

ENSG00000130349	ENSG00000130349	C6orf203	6:107349406-107372546	q1	q2	NOTEST	0	0	0	0	1	1	no

ENSG00000130363	ENSG00000130363	RSPH3	6:159393902-159486305	q1	q2	OK	1.99468	2.00489	0.00736856	0.0116959	0.987	0.99995	no

ENSG00000130368	ENSG00000130368	MAS1	6:160327973-160329108	q1	q2	NOTEST	0	0	0	0	1	1	no

ENSG00000130377	ENSG00000130377	ACSBG2	19:5978413-6199583	q1	q2	OK	0.521921	0.543481	0.0583994	0.0125915	0.95815	0.99995	no

ENSG00000130382	ENSG00000130382	MLLT1	19:6212965-6279959	q1	q2	OK	25.278	23.7526	-0.0897939	-0.157921	0.82115	0.99995	no

ENSG00000130383	ENSG00000130383	FUT5	19:5865836-5904017	q1	q2	NOTEST	0.0132717	0	#NAME?	0	1	1	no

ENSG00000130385	ENSG00000130385	BMP15	X:50653783-50659607	q1	q2	NOTEST	0	0	0	0	1	1	no

ENSG00000130396	ENSG00000130396	MLLT4	6:168227601-168372703	q1	q2	OK	146.38	137.694	-0.0882522	-0.117951	0.7967	0.99995	no

ENSG00000130402	ENSG00000130402	ACTN4	19:39138288-39260544	q1	q2	OK	125.517	146.798	0.225955	0.342346	0.6162	0.99995	no

ENSG00000130413	ENSG00000130413	STK33	11:8413417-8615836	q1	q2	OK	18.6373	17.2644	-0.110393	-0.178867	0.7753	0.99995	no

ENSG00000130414	ENSG00000130414	NDUFA10	2:240831866-240964819	q1	q2	OK	64.398	57.255	-0.169614	-0.261364	0.7087	0.99995	no

ENSG00000130427	ENSG00000130427	EPO	7:100318422-100321323	q1	q2	NOTEST	0.0210008	0.0831101	1.98458	0	1	1	no

ENSG00000130429	ENSG00000130429	ARPC1B	7:98923520-99063954	q1	q2	OK	13.7728	14.0855	0.0323844	0.0118746	0.9789	0.99995	no

ENSG00000130433	ENSG00000130433	CACNG6	19:54495541-54515923	q1	q2	OK	0.560428	0.504202	-0.152528	-0.139233	0.82215	0.99995	no

ENSG00000130449	ENSG00000130449	ZSWIM6	5:60628099-60841997	q1	q2	OK	24.1591	23.7154	-0.0267376	-0.0641749	0.92745	0.99995	no

ENSG00000130475	ENSG00000130475	FCHO1	19:17858526-17899377	q1	q2	OK	3.36946	3.01249	-0.161561	-0.155853	0.6658	0.99995	no

ENSG00000130477	ENSG00000130477	UNC13A	19:17712136-17799401	q1	q2	OK	6.32313	6.24064	-0.0189448	-0.0250351	0.9523	0.99995	no

ENSG00000130479	ENSG00000130479	MAP1S	19:17830050-17845325	q1	q2	OK	15.6187	12.9235	-0.273279	-0.479538	0.35485	0.99995	no

ENSG00000130487	ENSG00000130487	KLHDC7B	22:50986461-50989451	q1	q2	NOTEST	0.0170102	0.0170836	0.00621366	0	1	1	no

ENSG00000130489	ENSG00000130489	SCO2	22:50961996-50971009	q1	q2	OK	13.4473	12.6271	-0.0907991	-0.12776	0.85515	0.99995	no

ENSG00000130508	ENSG00000130508	PXDN	2:1635658-1748624	q1	q2	OK	94.4842	100.126	0.0836678	0.120066	0.7922	0.99995	no

ENSG00000130511	ENSG00000130511	SSBP4	19:18529673-18549111	q1	q2	OK	43.7609	39.8382	-0.135491	-0.114548	0.85285	0.99995	no

ENSG00000130513	ENSG00000130513	GDF15	19:18485540-18499986	q1	q2	OK	2.43313	1.75285	-0.47311	-0.478531	0.4263	0.99995	no

ENSG00000130517	ENSG00000130517	PGPEP1	19:18451396-18480760	q1	q2	OK	8.61104	8.32965	-0.0479301	-0.0522238	0.9055	0.99995	no

ENSG00000130518	ENSG00000130518	KIAA1683	19:18367907-18385319	q1	q2	OK	1.61413	1.30323	-0.308661	-0.408611	0.50055	0.99995	no

ENSG00000130520	ENSG00000130520	LSM4	19:18417039-18434084	q1	q2	OK	155.142	135.87	-0.191365	-0.453163	0.51055	0.99995	no

ENSG00000130522	ENSG00000130522	JUND	19:18390562-18392432	q1	q2	OK	62.4633	52.7757	-0.243136	-0.396879	0.565	0.99995	no

ENSG00000130528	ENSG00000130528	HRC	19:49654454-49658681	q1	q2	NOTEST	0.235213	0.247565	0.0738404	0	1	1	no

ENSG00000130529	ENSG00000130529	TRPM4	19:49660997-49715093	q1	q2	OK	4.61413	4.58365	-0.00956044	-0.0102366	0.9835	0.99995	no

ENSG00000130538	ENSG00000130538	OR11H1	22:16448823-16449805	q1	q2	NOTEST	0	0	0	0	1	1	no

ENSG00000130540	ENSG00000130540	SULT4A1	22:44220388-44258398	q1	q2	OK	1.78843	1.67438	-0.0950639	-0.0864004	0.88625	0.99995	no

ENSG00000130544	ENSG00000130544	ZNF557	19:7069454-7087979	q1	q2	OK	3.81703	4.38478	0.200051	0.332609	0.6348	0.99995	no

ENSG00000130545	ENSG00000130545	CRB3	19:6436089-6482568	q1	q2	NOTEST	0.0381828	0.0897718	1.23334	0	1	1	no

ENSG00000130558	ENSG00000130558	OLFM1	9:137967267-138013025	q1	q2	OK	26.1234	23.3723	-0.160545	-0.307583	0.64775	0.99995	no

ENSG00000130559	ENSG00000130559	CAMSAP1	9:138700332-138799074	q1	q2	OK	27.4396	27.5548	0.00604363	0.0113591	0.98375	0.99995	no

ENSG00000130560	ENSG00000130560	UBAC1	9:138824814-138853226	q1	q2	OK	24.1436	20.1079	-0.263877	-0.526127	0.43815	0.99995	no

ENSG00000130561	ENSG00000130561	SAG	2:234216461-234255701	q1	q2	NOTEST	0	0.00355235	inf	0	1	1	no

ENSG00000130584	ENSG00000130584	ZBTB46	20:62375018-62462597	q1	q2	OK	9.21756	9.52298	0.0470275	0.103943	0.87855	0.99995	no

ENSG00000130589	ENSG00000130589	HELZ2	20:62189438-62205592	q1	q2	OK	3.43346	3.03542	-0.177765	-0.24436	0.66195	0.99995	no

ENSG00000130590	ENSG00000130590	SAMD10	20:62588054-62680994	q1	q2	OK	5.04977	4.61022	-0.131381	-0.059283	0.91505	0.99995	no

ENSG00000130592	ENSG00000130592	LSP1	11:1874199-1913497	q1	q2	OK	1.06768	0.612606	-0.801446	-0.377778	0.2986	0.99995	no

ENSG00000130595	ENSG00000130595	TNNT3	11:1940791-1959936	q1	q2	NOTEST	0.292482	0.138467	-1.07881	0	1	1	no

ENSG00000130598	ENSG00000130598	TNNI2	11:1860218-1862910	q1	q2	NOTEST	0.0496456	0.0488323	-0.0238286	0	1	1	no

ENSG00000130600	ENSG00000130600	H19	11:2016405-2022700	q1	q2	OK	277.853	230.143	-0.271792	-0.431256	0.49575	0.99995	no

ENSG00000130612	ENSG00000130612	CYP2G1P	19:41277552-41406413	q1	q2	NOTEST	0.127833	0.175709	0.458925	0	1	1	no

ENSG00000130635	ENSG00000130635	COL5A1	9:137533619-137736686	q1	q2	OK	28.465	23.1378	-0.298935	-0.552989	0.32305	0.99995	no

ENSG00000130638	ENSG00000130638	ATXN10	22:46067677-46241187	q1	q2	OK	138.795	150.07	0.112678	0.180363	0.7977	0.99995	no

ENSG00000130640	ENSG00000130640	TUBGCP2	10:135093134-135166187	q1	q2	OK	29.9589	28.8997	-0.0519301	-0.0928494	0.8794	0.99995	no

ENSG00000130643	ENSG00000130643	CALY	10:135093134-135166187	q1	q2	OK	0.580288	0.102728	-2.49794	-0.203188	0.07695	0.99995	no

ENSG00000130649	ENSG00000130649	CYP2E1	10:135192694-135382916	q1	q2	OK	0.770833	0.321893	-1.25984	-0.214983	0.22165	0.99995	no

ENSG00000130653	ENSG00000130653	PNPLA7	9:140354403-140444986	q1	q2	OK	0.772842	0.780899	0.0149622	0.0141301	0.98275	0.99995	no

ENSG00000130656	ENSG00000130656	HBZ	16:202685-216767	q1	q2	NOTEST	0	0	0	0	1	1	no

ENSG00000130669	ENSG00000130669	PAK4	19:39616409-39673456	q1	q2	OK	21.3164	19.676	-0.115525	-0.222825	0.7291	0.99995	no

ENSG00000130675	ENSG00000130675	MNX1	7:156786744-156803345	q1	q2	NOTEST	0.101508	0.0991072	-0.0345366	0	1	1	no

ENSG00000130684	ENSG00000130684	ZNF337	20:25593570-25677477	q1	q2	OK	17.8285	16.1988	-0.138298	-0.278412	0.6834	0.99995	no

ENSG00000130695	ENSG00000130695	CEP85	1:26560690-26605299	q1	q2	OK	16.7907	18.0307	0.102796	0.163069	0.7522	0.99995	no

ENSG00000130699	ENSG00000130699	TAF4	20:60528524-60640866	q1	q2	OK	11.9023	11.803	-0.012088	-0.0205902	0.97125	0.99995	no

ENSG00000130700	ENSG00000130700	GATA5	20:61038552-61051026	q1	q2	NOTEST	0	0	0	0	1	1	no

ENSG00000130701	ENSG00000130701	RBBP8NL	20:60985292-61002589	q1	q2	NOTEST	0	0	0	0	1	1	no

ENSG00000130702	ENSG00000130702	LAMA5	20:60877148-60942368	q1	q2	OK	17.3398	13.3909	-0.372835	-0.254407	0.49105	0.99995	no

ENSG00000130703	ENSG00000130703	OSBPL2	20:60813579-60871268	q1	q2	OK	12.5793	11.5867	-0.118573	-0.250524	0.70565	0.99995	no

ENSG00000130706	ENSG00000130706	ADRM1	20:60877148-60942368	q1	q2	OK	64.726	57.068	-0.181664	-0.272435	0.68275	0.99995	no

ENSG00000130707	ENSG00000130707	ASS1	9:133320315-133376661	q1	q2	OK	8.52539	7.35936	-0.212186	-0.291037	0.6609	0.99995	no

ENSG00000130711	ENSG00000130711	PRDM12	9:133539980-133558368	q1	q2	OK	1.54283	0.962779	-0.680301	-0.797701	0.2574	0.99995	no

ENSG00000130713	ENSG00000130713	EXOSC2	9:133569107-133580248	q1	q2	OK	30.1309	31.01	0.0414903	0.0805136	0.90625	0.99995	no

ENSG00000130714	ENSG00000130714	POMT1	9:134378288-134406655	q1	q2	OK	25.5665	25.7734	0.0116285	0.0151104	0.98005	0.99995	no

ENSG00000130717	ENSG00000130717	UCK1	9:134378288-134406655	q1	q2	OK	23.6126	20.9289	-0.17406	-0.252284	0.6801	0.99995	no

ENSG00000130720	ENSG00000130720	FIBCD1	9:133777824-133820907	q1	q2	NOTEST	0.0617893	0.0520914	-0.246313	0	1	1	no

ENSG00000130723	ENSG00000130723	PRRC2B	9:134269479-134375584	q1	q2	OK	96.0381	95.3229	-0.0107839	-0.0161255	0.97685	0.99995	no

ENSG00000130724	ENSG00000130724	CHMP2A	19:59062932-59066491	q1	q2	OK	60.3599	56.5379	-0.0943714	-0.200325	0.7778	0.99995	no

ENSG00000130725	ENSG00000130725	UBE2M	19:59067078-59070327	q1	q2	OK	39.1009	37.5288	-0.0592033	-0.10649	0.84555	0.99995	no

ENSG00000130726	ENSG00000130726	TRIM28	19:59055457-59062089	q1	q2	OK	321.554	296.925	-0.114961	-0.202555	0.74465	0.99995	no

ENSG00000130731	ENSG00000130731	C16orf13	16:684428-686358	q1	q2	OK	27.3938	26.7047	-0.0367534	-0.057307	0.9392	0.99995	no

ENSG00000130733	ENSG00000130733	YIPF2	19:10982188-11039357	q1	q2	OK	13.7244	11.3467	-0.274468	-0.202798	0.74325	0.99995	no

ENSG00000130734	ENSG00000130734	ATG4D	19:10654570-10676713	q1	q2	OK	20.6105	19.0104	-0.116594	-0.142685	0.8213	0.99995	no

ENSG00000130741	ENSG00000130741	EIF2S3	X:24072832-24096088	q1	q2	OK	71.6392	72.825	0.0236857	0.0348214	0.95105	0.99995	no

ENSG00000130748	ENSG00000130748	TMEM160	19:47549164-47551888	q1	q2	OK	20.9906	15.6735	-0.421415	-0.643388	0.35725	0.99995	no

ENSG00000130749	ENSG00000130749	ZC3H4	19:47567443-47713886	q1	q2	OK	18.5693	17.8932	-0.0535106	-0.0364727	0.9341	0.99995	no

ENSG00000130751	ENSG00000130751	NPAS1	19:47523076-47549033	q1	q2	OK	1.46059	1.24473	-0.23072	-0.197373	0.7346	0.99995	no

ENSG00000130755	ENSG00000130755	GMFG	19:39818992-39833012	q1	q2	NOTEST	0.396779	0.237176	-0.742376	0	1	1	no

ENSG00000130758	ENSG00000130758	MAP3K10	19:40697650-40721482	q1	q2	OK	8.94909	6.66306	-0.425558	-0.527895	0.3867	0.99995	no

ENSG00000130762	ENSG00000130762	ARHGEF16	1:3370989-3397677	q1	q2	OK	3.92744	3.86995	-0.0212761	-0.0265702	0.96415	0.99995	no

ENSG00000130764	ENSG00000130764	LRRC47	1:3696783-3713068	q1	q2	OK	26.7886	25.8013	-0.0541722	-0.0925146	0.87985	0.99995	no

ENSG00000130766	ENSG00000130766	SESN2	1:28586037-28609002	q1	q2	OK	19.5571	16.0306	-0.28686	-0.664177	0.3381	0.99995	no

ENSG00000130768	ENSG00000130768	SMPDL3B	1:28261503-28294607	q1	q2	NOTEST	0.0185676	0.0319984	0.785214	0	1	1	no

ENSG00000130770	ENSG00000130770	ATPIF1	1:28562619-28573417	q1	q2	OK	124.377	114.513	-0.119204	-0.211979	0.76415	0.99995	no

ENSG00000130772	ENSG00000130772	MED18	1:28655512-28662476	q1	q2	OK	11.6524	11.4084	-0.0305251	-0.053315	0.9396	0.99995	no

ENSG00000130775	ENSG00000130775	THEMIS2	1:28199054-28213196	q1	q2	OK	1.58533	1.46095	-0.117882	-0.0892821	0.87515	0.99995	no

ENSG00000130779	ENSG00000130779	CLIP1	12:122755978-122907179	q1	q2	OK	45.7926	50.406	0.13848	0.248443	0.6708	0.99995	no

ENSG00000130783	ENSG00000130783	CCDC62	12:123258873-123312075	q1	q2	NOTEST	0.358172	0.444669	0.31208	0	1	1	no

ENSG00000130787	ENSG00000130787	HIP1R	12:123318999-123347507	q1	q2	OK	19.3736	17.1286	-0.177687	-0.307267	0.61065	0.99995	no

ENSG00000130803	ENSG00000130803	ZNF317	19:9251055-9274100	q1	q2	OK	13.9814	14.5602	0.0585276	0.0922455	0.8641	0.99995	no

ENSG00000130810	ENSG00000130810	PPAN	19:10216898-10230596	q1	q2	OK	21.7419	21.4943	-0.016529	-0.0163877	0.98055	0.99995	no

ENSG00000130811	ENSG00000130811	EIF3G	19:10216898-10230596	q1	q2	OK	98.3777	89.6964	-0.133282	-0.264759	0.68525	0.99995	no

ENSG00000130812	ENSG00000130812	ANGPTL6	19:10196797-10213472	q1	q2	NOTEST	0.149978	0.0909431	-0.72171	0	1	1	no

ENSG00000130813	ENSG00000130813	C19orf66	19:10196797-10213472	q1	q2	OK	2.73317	2.25701	-0.276162	-0.291988	0.64725	0.99995	no

ENSG00000130816	ENSG00000130816	DNMT1	19:10244020-10341962	q1	q2	OK	77.7971	76.3025	-0.0279855	-0.0348596	0.9345	0.99995	no

ENSG00000130818	ENSG00000130818	ZNF426	19:9638666-9649303	q1	q2	OK	7.19297	7.43366	0.0474846	0.0835263	0.9047	0.99995	no

ENSG00000130821	ENSG00000130821	SLC6A8	X:152935184-152962048	q1	q2	OK	0.9858	2.53775	1.36418	0.913513	0.05305	0.99995	no

ENSG00000130822	ENSG00000130822	PNCK	X:152935184-152962048	q1	q2	NOTEST	0.348938	0.1252	-1.47874	0	1	1	no

ENSG00000130826	ENSG00000130826	DKC1	X:153991030-154005964	q1	q2	NOTEST	0	0	0	0	1	1	no

ENSG00000130827	ENSG00000130827	PLXNA3	X:153686620-153701989	q1	q2	OK	3.66536	3.42606	-0.0974051	-0.0992994	0.8281	0.99995	no

ENSG00000130829	ENSG00000130829	DUSP9	X:152907945-152916781	q1	q2	NOTEST	0.0119761	0.0110135	-0.120878	0	1	1	no

ENSG00000130830	ENSG00000130830	MPP1	X:154006958-154049282	q1	q2	NOTEST	0	0	0	0	1	1	no

ENSG00000130844	ENSG00000130844	ZNF331	19:54024234-54083523	q1	q2	OK	13.2687	14.1847	0.096304	0.174003	0.7567	0.99995	no

ENSG00000130856	ENSG00000130856	ZNF236	18:74534562-74682683	q1	q2	OK	4.20967	4.27516	0.0222719	0.034308	0.9456	0.99995	no

ENSG00000130876	ENSG00000130876	SLC7A10	19:33699569-33716756	q1	q2	NOTEST	0.268932	0.208154	-0.369592	0	1	1	no

ENSG00000130881	ENSG00000130881	LRP3	19:33668508-33698694	q1	q2	OK	27.9556	24.5507	-0.187373	-0.421096	0.53805	0.99995	no

ENSG00000130921	ENSG00000130921	C12orf65	12:123636866-123756881	q1	q2	OK	17.4898	18.2213	0.0591115	0.0394345	0.94795	0.99995	no

ENSG00000130935	ENSG00000130935	NOL11	17:65713948-65740647	q1	q2	OK	39.8309	41.5613	0.0613549	0.131368	0.83395	0.99995	no

ENSG00000130939	ENSG00000130939	UBE4B	1:10092889-10241297	q1	q2	OK	29.255	28.4942	-0.038014	-0.0584295	0.92935	0.99995	no

ENSG00000130940	ENSG00000130940	CASZ1	1:10696660-10856707	q1	q2	OK	0.944746	1.21001	0.35702	0.226102	0.5445	0.99995	no

ENSG00000130943	ENSG00000130943	PKDREJ	22:46651559-46659219	q1	q2	NOTEST	0.0489082	0.05416	0.147154	0	1	1	no

ENSG00000130948	ENSG00000130948	HSD17B3	9:98997587-99064434	q1	q2	NOTEST	0.157302	0.129361	-0.282133	0	1	1	no

ENSG00000130950	ENSG00000130950	NUTM2F	9:97077604-97090926	q1	q2	NOTEST	0.0267473	0	#NAME?	0	1	1	no

ENSG00000130956	ENSG00000130956	HABP4	9:99212482-99382112	q1	q2	OK	12.0987	13.8038	0.190221	0.303629	0.67555	0.99995	no

ENSG00000130957	ENSG00000130957	FBP2	9:97317350-97356075	q1	q2	NOTEST	0.0226634	0	#NAME?	0	1	1	no

ENSG00000130958	ENSG00000130958	SLC35D2	9:99082987-99145992	q1	q2	OK	7.43338	8.97229	0.271458	0.360031	0.6146	0.99995	no

ENSG00000130962	ENSG00000130962	PRRG1	X:37208527-38548169	q1	q2	OK	0.797182	0.388464	-1.03713	-0.177845	0.4702	0.99995	no

ENSG00000130985	ENSG00000130985	UBA1	X:47050259-47074527	q1	q2	NOTEST	0	0.134961	inf	0	1	1	no

ENSG00000130988	ENSG00000130988	RGN	X:46937774-46952712	q1	q2	NOTEST	0	0	0	0	1	1	no

ENSG00000130997	ENSG00000130997	POLN	4:2073644-2243891	q1	q2	OK	1.24062	1.01564	-0.288672	-0.0918731	0.727	0.99995	no

ENSG00000131002	ENSG00000131002	TXLNG2P	Y:21729198-21768160	q1	q2	OK	29.4724	32.1943	0.127443	0.186933	0.7332	0.99995	no

ENSG00000131007	ENSG00000131007	TTTY9B	Y:20652800-20790963	q1	q2	NOTEST	0	0	0	0	1	1	no

ENSG00000131013	ENSG00000131013	PPIL4	6:149825868-149867174	q1	q2	OK	25.7592	26.498	0.0407954	0.0932737	0.8899	0.99995	no

ENSG00000131015	ENSG00000131015	ULBP2	6:150263135-150270371	q1	q2	OK	0.687267	0.576131	-0.254475	-0.233251	0.7381	0.99995	no

ENSG00000131016	ENSG00000131016	AKAP12	6:151561133-151679692	q1	q2	OK	23.8342	24.3967	0.0336519	0.083073	0.90375	0.99995	no

ENSG00000131018	ENSG00000131018	SYNE1	6:151977825-152958936	q1	q2	OK	9.00644	8.25697	-0.125343	-0.13844	0.7379	0.99995	no

ENSG00000131019	ENSG00000131019	ULBP3	6:150384285-150390231	q1	q2	OK	3.6617	3.86128	0.0765652	0.0836198	0.90285	0.99995	no

ENSG00000131023	ENSG00000131023	LATS1	6:149979288-150039392	q1	q2	OK	11.4428	12.3378	0.108648	0.250536	0.7105	0.99995	no

ENSG00000131037	ENSG00000131037	EPS8L1	19:55583387-55599291	q1	q2	OK	12.0581	12.4927	0.0510717	0.0702733	0.92035	0.99995	no

ENSG00000131042	ENSG00000131042	LILRB2	19:54777674-54785039	q1	q2	NOTEST	0	0	0	0	1	1	no

ENSG00000131043	ENSG00000131043	AAR2	20:34824380-34858840	q1	q2	OK	19.9198	18.2737	-0.124432	-0.276602	0.6965	0.99995	no

ENSG00000131044	ENSG00000131044	TTLL9	20:30435439-30539895	q1	q2	NOTEST	0.424969	0.289541	-0.553592	0	1	1	no

ENSG00000131050	ENSG00000131050	BPIFA2	20:31749573-31769218	q1	q2	NOTEST	0	0	0	0	1	1	no

ENSG00000131051	ENSG00000131051	RBM39	20:34291530-34330234	q1	q2	OK	202.152	207.879	0.0403009	0.0700283	0.8926	0.99995	no

ENSG00000131055	ENSG00000131055	COX4I2	20:30225690-30232809	q1	q2	NOTEST	0	0	0	0	1	1	no

ENSG00000131059	ENSG00000131059	BPIFA3	20:31805115-31815564	q1	q2	NOTEST	0	0	0	0	1	1	no

ENSG00000131061	ENSG00000131061	ZNF341	20:32316736-32398903	q1	q2	OK	2.1946	2.23685	0.0275052	0.0383948	0.9553	0.99995	no

ENSG00000131067	ENSG00000131067	GGT7	20:33432522-33515769	q1	q2	OK	15.622	16.9717	0.119552	0.135593	0.7895	0.99995	no

ENSG00000131068	ENSG00000131068	DEFB118	20:29956420-29961726	q1	q2	NOTEST	0	0	0	0	1	1	no

ENSG00000131069	ENSG00000131069	ACSS2	20:33432522-33515769	q1	q2	OK	24.0065	26.7831	0.157897	0.151408	0.6883	0.99995	no

ENSG00000131080	ENSG00000131080	EDA2R	X:65815478-65859108	q1	q2	OK	6.6989	5.71783	-0.228456	-0.388872	0.53885	0.99995	no

ENSG00000131089	ENSG00000131089	ARHGEF9	X:62854846-63005426	q1	q2	NOTEST	0	0	0	0	1	1	no

ENSG00000131094	ENSG00000131094	C1QL1	17:43037060-43045439	q1	q2	OK	4.54697	2.70111	-0.751353	-0.946484	0.1843	0.99995	no

ENSG00000131095	ENSG00000131095	GFAP	17:42923720-42994305	q1	q2	NOTEST	0.0333477	0.212957	2.6749	0	1	1	no

ENSG00000131096	ENSG00000131096	PYY	17:42030105-42081837	q1	q2	NOTEST	0	0	0	0	1	1	no

ENSG00000131097	ENSG00000131097	HIGD1B	17:42923720-42994305	q1	q2	NOTEST	0	0.2472	inf	0	1	1	no

ENSG00000131100	ENSG00000131100	ATP6V1E1	22:18074901-18111584	q1	q2	OK	45.3183	45.0078	-0.00991962	-0.0224932	0.9739	0.99995	no

ENSG00000131115	ENSG00000131115	ZNF227	19:44711699-44809199	q1	q2	OK	16.8246	16.5771	-0.0213825	-0.0289688	0.95235	0.99995	no

ENSG00000131116	ENSG00000131116	ZNF428	19:44100543-44124026	q1	q2	OK	83.504	71.647	-0.220939	-0.465549	0.51105	0.99995	no

ENSG00000131126	ENSG00000131126	TEX101	19:43905647-43922763	q1	q2	NOTEST	0.0306291	0	#NAME?	0	1	1	no

ENSG00000131127	ENSG00000131127	ZNF141	4:331602-378653	q1	q2	NOTEST	0	0	0	0	1	1	no

ENSG00000131142	ENSG00000131142	CCL25	19:8117650-8127534	q1	q2	NOTEST	0.315907	0.154343	-1.03336	0	1	1	no

ENSG00000131143	ENSG00000131143	COX4I1	16:85805363-85840650	q1	q2	OK	282.281	262.091	-0.107067	-0.228328	0.74375	0.99995	no

ENSG00000131148	ENSG00000131148	EMC8	16:85805363-85840650	q1	q2	OK	21.5239	20.2267	-0.0896766	-0.0838818	0.8903	0.99995	no

ENSG00000131149	ENSG00000131149	GSE1	16:85645014-85723679	q1	q2	OK	23.1074	22.8675	-0.0150586	-0.0155921	0.9657	0.99995	no

ENSG00000131152	ENSG00000131152	RP11-178L8.4	16:87117167-87438385	q1	q2	NOTEST	0.30305	0	#NAME?	0	1	1	no

ENSG00000131153	ENSG00000131153	GINS2	16:85645014-85723679	q1	q2	OK	27.7307	26.9754	-0.0398368	-0.0573861	0.93395	0.99995	no

ENSG00000131165	ENSG00000131165	CHMP1A	16:89710838-89737680	q1	q2	OK	44.5723	40.6904	-0.131459	-0.235614	0.683	0.99995	no

ENSG00000131171	ENSG00000131171	SH3BGRL	X:80457441-80554046	q1	q2	OK	139.175	148.869	0.0971439	0.232259	0.7363	0.99995	no

ENSG00000131174	ENSG00000131174	COX7B	X:77154934-77162870	q1	q2	OK	1.32047	1.56323	0.243484	0.175415	0.8267	0.99995	no

ENSG00000131183	ENSG00000131183	SLC34A1	5:176806235-176825849	q1	q2	NOTEST	0.142895	0.0134532	-3.40894	0	1	1	no

ENSG00000131187	ENSG00000131187	F12	5:176829140-176883283	q1	q2	OK	0.856645	0.85538	-0.00213217	-0.000666376	0.99855	0.99995	no

ENSG00000131188	ENSG00000131188	PRR7	5:176829140-176883283	q1	q2	OK	5.89248	4.64506	-0.34318	-0.28398	0.68165	0.99995	no

ENSG00000131196	ENSG00000131196	NFATC1	18:77155855-77289325	q1	q2	OK	1.49477	1.74855	0.226237	0.217511	0.7038	0.99995	no

ENSG00000131203	ENSG00000131203	IDO1	8:39759793-39873910	q1	q2	NOTEST	0.0188933	0	#NAME?	0	1	1	no

ENSG00000131233	ENSG00000131233	GJA9	1:39325671-39407471	q1	q2	NOTEST	0.140025	0.225326	0.686329	0	1	1	no

ENSG00000131236	ENSG00000131236	CAP1	1:40505904-40538321	q1	q2	OK	105.955	122.544	0.209853	0.464835	0.45935	0.99995	no

ENSG00000131238	ENSG00000131238	PPT1	1:40538378-40563375	q1	q2	OK	47.4013	50.6767	0.0963957	0.219526	0.73995	0.99995	no

ENSG00000131242	ENSG00000131242	RAB11FIP4	17:29718641-29865236	q1	q2	OK	2.48064	3.06887	0.306993	0.242099	0.59155	0.99995	no

ENSG00000131263	ENSG00000131263	RLIM	X:73805051-73834452	q1	q2	OK	15.7263	16.1008	0.0339529	0.0816267	0.9057	0.99995	no

ENSG00000131264	ENSG00000131264	CDX4	X:72667089-72675081	q1	q2	NOTEST	0	0	0	0	1	1	no

ENSG00000131269	ENSG00000131269	ABCB7	X:74273114-74376567	q1	q2	OK	14.0105	13.8563	-0.0159627	-0.0287714	0.9627	0.99995	no

ENSG00000131323	ENSG00000131323	TRAF3	14:103243812-103377837	q1	q2	OK	10.353	9.78341	-0.0816352	-0.144779	0.81325	0.99995	no

ENSG00000131351	ENSG00000131351	HAUS8	19:17160538-17186435	q1	q2	OK	15.0394	14.2283	-0.0799798	-0.136487	0.8405	0.99995	no

ENSG00000131355	ENSG00000131355	EMR3	19:14729928-14889353	q1	q2	NOTEST	0.014131	0.0260061	0.879984	0	1	1	no

ENSG00000131368	ENSG00000131368	MRPS25	3:14860468-15106842	q1	q2	OK	26.5234	26.6631	0.00758069	0.0053166	0.9907	0.99995	no

ENSG00000131370	ENSG00000131370	SH3BP5	3:15295690-15382875	q1	q2	OK	3.24447	4.03001	0.312799	0.278598	0.69445	0.99995	no

ENSG00000131373	ENSG00000131373	HACL1	3:15602210-15687329	q1	q2	OK	6.35038	5.74473	-0.144604	-0.191901	0.7796	0.99995	no

ENSG00000131374	ENSG00000131374	TBC1D5	3:17198653-18959184	q1	q2	OK	17.3815	18.6156	0.0989562	0.081059	0.7876	0.99995	no

ENSG00000131375	ENSG00000131375	CAPN7	3:15247658-15294425	q1	q2	OK	13.5613	14.3902	0.0855997	0.145186	0.7994	0.99995	no

ENSG00000131378	ENSG00000131378	RFTN1	3:16306705-16555533	q1	q2	OK	5.71885	5.38465	-0.0868743	-0.0963957	0.8824	0.99995	no

ENSG00000131379	ENSG00000131379	C3orf20	3:14716605-14814541	q1	q2	NOTEST	0.0942817	0.00969683	-3.28139	0	1	1	no

ENSG00000131381	ENSG00000131381	ZFYVE20	3:15111579-15140670	q1	q2	OK	14.273	14.253	-0.00202061	-0.00349845	0.9949	0.99995	no

ENSG00000131386	ENSG00000131386	GALNT15	3:16216155-16273499	q1	q2	NOTEST	0.0733357	0.0942522	0.362011	0	1	1	no

ENSG00000131389	ENSG00000131389	SLC6A6	3:14444075-14583588	q1	q2	OK	9.52281	9.63401	0.0167497	0.0295611	0.96215	0.99995	no

ENSG00000131398	ENSG00000131398	KCNC3	19:50815193-50886239	q1	q2	OK	2.13105	1.56428	-0.446071	-0.401107	0.5925	0.99995	no

ENSG00000131400	ENSG00000131400	NAPSA	19:50815193-50886239	q1	q2	NOTEST	0.219363	0.103898	-1.07815	0	1	1	no

ENSG00000131401	ENSG00000131401	NAPSB	19:50815193-50886239	q1	q2	NOTEST	0.259294	0.147632	-0.812587	0	1	1	no

ENSG00000131408	ENSG00000131408	NR1H2	19:50815193-50886239	q1	q2	OK	13.451	10.3488	-0.378243	-0.608908	0.342	0.99995	no

ENSG00000131409	ENSG00000131409	LRRC4B	19:51020148-51071302	q1	q2	OK	38.6741	32.8496	-0.235495	-0.562531	0.40985	0.99995	no

ENSG00000131435	ENSG00000131435	PDLIM4	5:131520568-131731306	q1	q2	OK	4.89804	4.62826	-0.0817348	-0.055149	0.91955	0.99995	no

ENSG00000131437	ENSG00000131437	KIF3A	5:132024372-132073330	q1	q2	OK	15.7214	17.7159	0.172314	0.217667	0.648	0.99995	no

ENSG00000131446	ENSG00000131446	MGAT1	5:180217540-180242652	q1	q2	OK	37.6901	36.4649	-0.0476779	-0.0827078	0.8846	0.99995	no

ENSG00000131459	ENSG00000131459	GFPT2	5:179727689-179780387	q1	q2	OK	4.76054	3.79011	-0.328885	-0.48851	0.41185	0.99995	no

ENSG00000131462	ENSG00000131462	TUBG1	17:40731530-40767252	q1	q2	OK	34.0345	33.1098	-0.0397402	-0.0632236	0.9271	0.99995	no

ENSG00000131467	ENSG00000131467	PSME3	17:40950809-40995774	q1	q2	OK	71.1582	68.9264	-0.0459728	-0.0788863	0.906	0.99995	no

ENSG00000131469	ENSG00000131469	RPL27	17:41150289-41154976	q1	q2	OK	837.003	875.377	0.0646729	0.155175	0.82155	0.99995	no

ENSG00000131470	ENSG00000131470	PSMC3IP	17:40719085-40729849	q1	q2	OK	32.6805	35.2285	0.108314	0.166103	0.8129	0.99995	no

ENSG00000131471	ENSG00000131471	AOC3	17:41003200-41010147	q1	q2	NOTEST	0.457794	0.390349	-0.229935	0	1	1	no

ENSG00000131473	ENSG00000131473	ACLY	17:40023160-40086795	q1	q2	OK	2.02496	1.3782	-0.555113	-0.581531	0.28555	0.99995	no

ENSG00000131475	ENSG00000131475	VPS25	17:40925453-40931617	q1	q2	OK	51.677	47.0136	-0.136442	-0.302835	0.65835	0.99995	no

ENSG00000131477	ENSG00000131477	RAMP2	17:40905931-40915059	q1	q2	OK	5.62251	4.76341	-0.239219	-0.237726	0.73905	0.99995	no

ENSG00000131480	ENSG00000131480	AOC2	17:40996616-41002724	q1	q2	OK	1.08177	1.14436	0.08114	0.0940081	0.8944	0.99995	no

ENSG00000131482	ENSG00000131482	G6PC	17:41052813-41065386	q1	q2	NOTEST	0.00819377	0	#NAME?	0	1	1	no

ENSG00000131484	ENSG00000131484	RP11-798G7.5	17:43578684-43640596	q1	q2	OK	0.710881	0.349924	-1.02257	-0.135886	0.62235	0.99995	no

ENSG00000131495	ENSG00000131495	NDUFA2	5:140018324-140042064	q1	q2	OK	47.6643	46.3863	-0.0392094	-0.0393345	0.9558	0.99995	no

ENSG00000131503	ENSG00000131503	ANKHD1	5:139781398-139973337	q1	q2	OK	71.7388	75.2484	0.0689066	0.0784665	0.84985	0.99995	no

ENSG00000131504	ENSG00000131504	DIAPH1	5:140894582-141016437	q1	q2	OK	31.1824	34.9546	0.164752	0.199573	0.7192	0.99995	no

ENSG00000131507	ENSG00000131507	NDFIP1	5:141488069-141534005	q1	q2	OK	45.3444	48.6442	0.101344	0.235015	0.7136	0.99995	no

ENSG00000131508	ENSG00000131508	UBE2D2	5:138906015-139008018	q1	q2	OK	84.0437	87.4118	0.0566886	0.111498	0.8693	0.99995	no

ENSG00000131538	ENSG00000131538	TTTY6	Y:24585086-24631739	q1	q2	NOTEST	0	0	0	0	1	1	no

ENSG00000131548	ENSG00000131548	TTTY6B	Y:24246960-24293631	q1	q2	NOTEST	0	0	0	0	1	1	no

ENSG00000131558	ENSG00000131558	EXOC4	7:132937828-133751342	q1	q2	OK	32.7471	38.6149	0.23779	0.375331	0.41785	0.99995	no

ENSG00000131584	ENSG00000131584	ACAP3	1:1227755-1260071	q1	q2	OK	47.5321	40.7256	-0.222965	-0.208485	0.58705	0.99995	no

ENSG00000131591	ENSG00000131591	C1orf159	1:1017197-1051741	q1	q2	OK	12.8754	11.5947	-0.151149	-0.233873	0.68845	0.99995	no

ENSG00000131620	ENSG00000131620	ANO1	11:69924407-70035634	q1	q2	NOTEST	0.248537	0.286671	0.205938	0	1	1	no

ENSG00000131626	ENSG00000131626	PPFIA1	11:70116805-70282690	q1	q2	OK	25.3885	24.4422	-0.0548	-0.0481195	0.9007	0.99995	no

ENSG00000131634	ENSG00000131634	TMEM204	16:1543344-1662111	q1	q2	OK	1.59911	1.77319	0.149074	0.0515506	0.9455	0.99995	no

ENSG00000131650	ENSG00000131650	KREMEN2	16:3013944-3030540	q1	q2	OK	1.09689	0.98782	-0.151098	-0.0597041	0.94085	0.99995	no

ENSG00000131652	ENSG00000131652	THOC6	16:3072620-3089134	q1	q2	OK	24.1808	22.6912	-0.0917301	-0.163971	0.8154	0.99995	no

ENSG00000131653	ENSG00000131653	TRAF7	16:2205698-2246526	q1	q2	OK	23.118	21.6774	-0.0928255	-0.184371	0.76415	0.99995	no

ENSG00000131668	ENSG00000131668	BARX1	9:96713904-96717654	q1	q2	NOTEST	0.043429	0.0205647	-1.07849	0	1	1	no

ENSG00000131669	ENSG00000131669	NINJ1	9:95883770-95896570	q1	q2	OK	10.7925	9.13578	-0.240433	-0.391284	0.57015	0.99995	no

ENSG00000131686	ENSG00000131686	CA6	1:9005925-9035151	q1	q2	NOTEST	0	0	0	0	1	1	no

ENSG00000131697	ENSG00000131697	NPHP4	1:5922870-6161253	q1	q2	OK	7.84007	7.27419	-0.108079	-0.152184	0.7886	0.99995	no

ENSG00000131711	ENSG00000131711	MAP1B	5:71403060-71505395	q1	q2	OK	111.146	120.597	0.117737	0.212243	0.7597	0.99995	no

ENSG00000131721	ENSG00000131721	RHOXF2	X:119170200-119379122	q1	q2	NOTEST	0	0	0	0	1	1	no

ENSG00000131724	ENSG00000131724	IL13RA1	X:117861534-117928502	q1	q2	OK	7.59769	8.09216	0.0909639	0.180877	0.78905	0.99995	no

ENSG00000131725	ENSG00000131725	WDR44	X:117480035-117583924	q1	q2	OK	7.1256	8.84653	0.3121	0.627377	0.3538	0.99995	no

ENSG00000131730	ENSG00000131730	CKMT2	5:80243515-80597379	q1	q2	NOTEST	0.167804	0.0160471	-3.38639	0	1	1	no

ENSG00000131732	ENSG00000131732	ZCCHC9	5:80597408-80609116	q1	q2	OK	26.903	27.5948	0.0366313	0.070792	0.91515	0.99995	no

ENSG00000131737	ENSG00000131737	KRT34	17:39533901-39538655	q1	q2	NOTEST	0	0	0	0	1	1	no

ENSG00000131738	ENSG00000131738	KRT33B	17:39519745-39526052	q1	q2	NOTEST	0	0	0	0	1	1	no

ENSG00000131746	ENSG00000131746	TNS4	17:38632079-38657849	q1	q2	NOTEST	0	0	0	0	1	1	no

ENSG00000131747	ENSG00000131747	TOP2A	17:38544767-38574202	q1	q2	OK	142.513	155.456	0.125418	0.237172	0.69395	0.99995	no

ENSG00000131748	ENSG00000131748	STARD3	17:37793317-37819737	q1	q2	OK	29.6588	25.1942	-0.235365	-0.419077	0.475	0.99995	no

ENSG00000131759	ENSG00000131759	RARA	17:38465443-38513094	q1	q2	OK	9.896	11.1274	0.169198	0.233144	0.72235	0.99995	no

ENSG00000131771	ENSG00000131771	PPP1R1B	17:37782992-37792879	q1	q2	NOTEST	0.395768	0.303811	-0.381482	0	1	1	no

ENSG00000131773	ENSG00000131773	KHDRBS3	8:136469699-136668965	q1	q2	OK	31.6674	31.9711	0.0137729	0.0224446	0.974	0.99995	no

ENSG00000131778	ENSG00000131778	CHD1L	1:146644349-146767443	q1	q2	OK	0.797859	1.29418	0.697838	0.476909	0.44995	0.99995	no

ENSG00000131779	ENSG00000131779	PEX11B	1:145516251-145523730	q1	q2	OK	0.0987901	1.45738	3.88286	2.28258	0.00295	0.99995	no

ENSG00000131781	ENSG00000131781	FMO5	1:146644349-146767443	q1	q2	NOTEST	0	0.0820093	inf	0	1	1	no

ENSG00000131788	ENSG00000131788	PIAS3	1:145575232-145589439	q1	q2	OK	0.39341	1.03078	1.38963	1.05586	0.06815	0.99995	no

ENSG00000131791	ENSG00000131791	PRKAB2	1:146626684-146644129	q1	q2	OK	2.37849	0.925346	-1.36198	-0.991499	0.0978	0.99995	no

ENSG00000131795	ENSG00000131795	RBM8A	1:145475981-145516076	q1	q2	OK	2.02991	1.78063	-0.189025	-0.134283	0.8342	0.99995	no

ENSG00000131797	ENSG00000131797	CLUHP3	16:31711910-31723305	q1	q2	OK	4.48173	4.4683	-0.00432837	-0.00615472	0.993	0.99995	no

ENSG00000131808	ENSG00000131808	FSHB	11:30252562-30256808	q1	q2	NOTEST	0	0	0	0	1	1	no

ENSG00000131828	ENSG00000131828	PDHA1	X:19362010-19533379	q1	q2	OK	86.1799	86.3111	0.00219512	0.00357888	0.99525	0.99995	no

ENSG00000131831	ENSG00000131831	RAI2	X:17818168-17879457	q1	q2	OK	0.59851	0.64517	0.108305	0.100813	0.87135	0.99995	no

ENSG00000131844	ENSG00000131844	MCCC2	5:70883114-70954531	q1	q2	OK	11.28	12.4741	0.145178	0.246711	0.67275	0.99995	no

ENSG00000131845	ENSG00000131845	ZNF304	19:57862215-57871266	q1	q2	OK	6.96619	7.15429	0.0384395	0.0773882	0.91235	0.99995	no

ENSG00000131848	ENSG00000131848	ZSCAN5A	19:56732680-56904914	q1	q2	OK	7.28452	5.8844	-0.30794	-0.204772	0.6162	0.99995	no

ENSG00000131849	ENSG00000131849	ZNF132	19:58944180-58951589	q1	q2	OK	2.1522	2.80219	0.380741	0.525972	0.45355	0.99995	no

ENSG00000131864	ENSG00000131864	USP29	19:57630505-57643294	q1	q2	NOTEST	0	0	0	0	1	1	no

ENSG00000131871	ENSG00000131871	VIMP	15:101811021-101817705	q1	q2	OK	37.7595	37.3175	-0.0169848	-0.0350324	0.95895	0.99995	no

ENSG00000131873	ENSG00000131873	CHSY1	15:101715927-101792137	q1	q2	OK	38.0836	42.3169	0.152066	0.317557	0.6333	0.99995	no

ENSG00000131876	ENSG00000131876	SNRPA1	15:101821714-101835487	q1	q2	OK	63.9341	70.5668	0.142406	0.314317	0.6331	0.99995	no

ENSG00000131885	ENSG00000131885	KRT17P1	17:16744056-16749197	q1	q2	NOTEST	0	0	0	0	1	1	no

ENSG00000131899	ENSG00000131899	LLGL1	17:18128900-18162230	q1	q2	OK	44.691	40.5509	-0.140252	-0.273827	0.695	0.99995	no

ENSG00000131910	ENSG00000131910	NR0B2	1:27216978-27273353	q1	q2	NOTEST	0	0	0	0	1	1	no

ENSG00000131914	ENSG00000131914	LIN28A	1:26737268-26756213	q1	q2	OK	9.2899	14.2191	0.614096	1.16807	0.0994	0.99995	no

ENSG00000131931	ENSG00000131931	THAP1	8:42691816-42698468	q1	q2	OK	6.8604	7.04985	0.0393004	0.0675886	0.92405	0.99995	no

ENSG00000131941	ENSG00000131941	RHPN2	19:33469498-33555794	q1	q2	OK	3.31429	3.52653	0.0895495	0.0966912	0.85255	0.99995	no

ENSG00000131943	ENSG00000131943	C19orf12	19:30191720-30206364	q1	q2	OK	17.5066	17.7188	0.0173764	0.0328331	0.95815	0.99995	no

ENSG00000131944	ENSG00000131944	C19orf40	19:33463114-33469128	q1	q2	OK	5.03942	5.14643	0.030316	0.0363192	0.95875	0.99995	no

ENSG00000131951	ENSG00000131951	LRRC9	14:60386140-60558501	q1	q2	OK	0.737945	0.898692	0.284314	0.162342	0.81985	0.99995	no

ENSG00000131966	ENSG00000131966	ACTR10	14:58466452-58764857	q1	q2	OK	45.0809	47.2681	0.0683521	0.0541886	0.93135	0.99995	no

ENSG00000131969	ENSG00000131969	ABHD12B	14:51314839-51411454	q1	q2	NOTEST	0.131258	0.0988939	-0.408452	0	1	1	no

ENSG00000131979	ENSG00000131979	GCH1	14:55308725-55369570	q1	q2	OK	1.84744	2.20767	0.256996	0.326857	0.6212	0.99995	no

ENSG00000131981	ENSG00000131981	LGALS3	14:55590827-55612126	q1	q2	OK	12.2626	11.8175	-0.053343	-0.0775515	0.91815	0.99995	no

ENSG00000131982	ENSG00000131982	UBE2L2	12:20514665-20837315	q1	q2	NOTEST	0	0	0	0	1	1	no

ENSG00000132000	ENSG00000132000	PODNL1	19:14041999-14072254	q1	q2	OK	0.58025	0.414313	-0.485953	-0.105772	0.55765	0.99995	no

ENSG00000132002	ENSG00000132002	DNAJB1	19:14625581-14676792	q1	q2	OK	52.4724	49.8298	-0.0745497	-0.136433	0.83185	0.99995	no

ENSG00000132003	ENSG00000132003	ZSWIM4	19:13906273-13943044	q1	q2	OK	11.335	10.86	-0.0617686	-0.130843	0.8474	0.99995	no

ENSG00000132004	ENSG00000132004	FBXW9	19:12798866-12807457	q1	q2	OK	7.08137	6.39064	-0.14807	-0.243201	0.73025	0.99995	no

ENSG00000132005	ENSG00000132005	RFX1	19:14072349-14117851	q1	q2	OK	7.18722	4.84	-0.570426	-0.390822	0.1806	0.99995	no

ENSG00000132010	ENSG00000132010	ZNF20	19:12203077-12267546	q1	q2	OK	4.7427	5.316	0.164631	0.0960529	0.80925	0.99995	no

ENSG00000132016	ENSG00000132016	C19orf57	19:13993160-14041692	q1	q2	OK	5.24597	4.03711	-0.377889	-0.298357	0.6045	0.99995	no

ENSG00000132017	ENSG00000132017	DCAF15	19:14041999-14072254	q1	q2	OK	36.9351	37.2466	0.012116	0.0199636	0.9738	0.99995	no

ENSG00000132024	ENSG00000132024	CC2D1A	19:13993160-14041692	q1	q2	OK	13.1236	11.2629	-0.220582	-0.303142	0.6394	0.99995	no

ENSG00000132026	ENSG00000132026	RTBDN	19:12873816-12985765	q1	q2	NOTEST	0.0974273	0.193274	0.988247	0	1	1	no

ENSG00000132031	ENSG00000132031	MATN3	2:20189977-20212455	q1	q2	OK	4.04917	5.05104	0.318954	0.43348	0.49775	0.99995	no

ENSG00000132109	ENSG00000132109	TRIM21	11:4406126-4414926	q1	q2	OK	4.88585	4.45878	-0.131959	-0.198803	0.7718	0.99995	no

ENSG00000132122	ENSG00000132122	SPATA6	1:48761043-48937845	q1	q2	OK	8.42897	8.87625	0.0745939	0.106952	0.8393	0.99995	no

ENSG00000132128	ENSG00000132128	LRRC41	1:46713359-46769280	q1	q2	OK	31.8792	29.8675	-0.0940405	-0.180514	0.7947	0.99995	no

ENSG00000132130	ENSG00000132130	LHX1	17:35294083-35301917	q1	q2	OK	1.20095	1.67046	0.476067	0.399402	0.41625	0.99995	no

ENSG00000132139	ENSG00000132139	GAS2L2	17:34071529-34079897	q1	q2	NOTEST	0.00843255	0	#NAME?	0	1	1	no

ENSG00000132141	ENSG00000132141	CCT6B	17:33254877-33332083	q1	q2	OK	3.27905	2.56836	-0.352433	-0.158246	0.7456	0.99995	no

ENSG00000132142	ENSG00000132142	ACACA	17:35441922-35766909	q1	q2	OK	34.2246	37.1281	0.117478	0.165717	0.70635	0.99995	no

ENSG00000132153	ENSG00000132153	DHX30	3:47844398-47891685	q1	q2	OK	45.7706	43.2523	-0.0816431	-0.166586	0.7976	0.99995	no

ENSG00000132155	ENSG00000132155	RAF1	3:12525930-12705725	q1	q2	OK	80.8575	78.5892	-0.0410506	-0.0687794	0.90835	0.99995	no

ENSG00000132164	ENSG00000132164	SLC6A11	3:10857884-10982419	q1	q2	NOTEST	0.403847	0.337418	-0.259274	0	1	1	no

ENSG00000132170	ENSG00000132170	PPARG	3:12328866-12475855	q1	q2	NOTEST	0.164698	0.0937574	-0.812817	0	1	1	no

ENSG00000132182	ENSG00000132182	NUP210	3:13357736-13461809	q1	q2	OK	15.1547	15.0802	-0.00710927	-0.0161351	0.97915	0.99995	no

ENSG00000132185	ENSG00000132185	FCRLA	1:161676761-161684142	q1	q2	NOTEST	0.0164866	0	#NAME?	0	1	1	no

ENSG00000132196	ENSG00000132196	HSD17B7	1:162760491-162782607	q1	q2	OK	8.87593	9.95301	0.165235	0.234765	0.7138	0.99995	no

ENSG00000132199	ENSG00000132199	ENOSF1	18:596987-712676	q1	q2	OK	14.4699	15.6712	0.115057	0.0550706	0.84325	0.99995	no

ENSG00000132204	ENSG00000132204	LINC00470	18:1254383-1408345	q1	q2	NOTEST	0	0	0	0	1	1	no

ENSG00000132205	ENSG00000132205	EMILIN2	18:2847027-2915991	q1	q2	NOTEST	0.366252	0.32038	-0.193051	0	1	1	no

ENSG00000132207	ENSG00000132207	SLX1A	16:30204254-30215631	q1	q2	OK	1.53189	0.718736	-1.09178	-0.59933	0.2994	0.99995	no

ENSG00000132254	ENSG00000132254	ARFIP2	11:6496909-6502666	q1	q2	OK	27.4853	26.0474	-0.0775179	-0.152693	0.8148	0.99995	no

ENSG00000132256	ENSG00000132256	TRIM5	11:5684424-5959849	q1	q2	OK	22.7494	24.807	0.124923	0.229345	0.70175	0.99995	no

ENSG00000132259	ENSG00000132259	CNGA4	11:6255994-6265659	q1	q2	NOTEST	0.0293191	0.0721834	1.29983	0	1	1	no

ENSG00000132274	ENSG00000132274	TRIM22	11:5684424-5959849	q1	q2	OK	6.19756	4.77414	-0.376458	-0.300307	0.6175	0.99995	no

ENSG00000132275	ENSG00000132275	RRP8	11:6616304-6624850	q1	q2	OK	7.07632	8.68036	0.294756	0.261803	0.72165	0.99995	no

ENSG00000132286	ENSG00000132286	TIMM10B	11:6502676-6614988	q1	q2	OK	18.9597	18.6261	-0.0256084	-0.0429053	0.9409	0.99995	no

ENSG00000132294	ENSG00000132294	EFR3A	8:132916334-133025889	q1	q2	OK	10.4015	10.8226	0.0572613	0.101406	0.8522	0.99995	no

ENSG00000132297	ENSG00000132297	HHLA1	8:133036466-133123406	q1	q2	NOTEST	0	0	0	0	1	1	no

ENSG00000132300	ENSG00000132300	PTCD3	2:86333304-86369280	q1	q2	OK	41.1877	46.0076	0.159659	0.221462	0.7062	0.99995	no

ENSG00000132305	ENSG00000132305	IMMT	2:86371054-86423172	q1	q2	OK	60.0971	65.629	0.127038	0.278513	0.64265	0.99995	no

ENSG00000132313	ENSG00000132313	MRPL35	2:86426477-86440917	q1	q2	OK	16.3881	17.2391	0.0730405	0.137874	0.8207	0.99995	no

ENSG00000132321	ENSG00000132321	IQCA1	2:237232793-237416185	q1	q2	NOTEST	0.301839	0.217274	-0.474261	0	1	1	no

ENSG00000132323	ENSG00000132323	ILKAP	2:239079041-239112370	q1	q2	OK	29.8171	30.3352	0.0248532	0.0471533	0.94575	0.99995	no

ENSG00000132326	ENSG00000132326	PER2	2:239140324-239198743	q1	q2	OK	2.29861	2.3091	0.00657455	0.00366831	0.9945	0.99995	no

ENSG00000132329	ENSG00000132329	RAMP1	2:238767535-238820756	q1	q2	OK	1.07323	0.841607	-0.350743	-0.306238	0.6344	0.99995	no

ENSG00000132330	ENSG00000132330	SCLY	2:238875468-239008054	q1	q2	OK	13.1511	13.9964	0.0898754	0.0909966	0.8485	0.99995	no

ENSG00000132334	ENSG00000132334	PTPRE	10:129705324-129884119	q1	q2	NOTEST	0.425664	0.452014	0.0866522	0	1	1	no

ENSG00000132341	ENSG00000132341	RAN	12:131356423-131362223	q1	q2	OK	499.935	519.737	0.0560429	0.11593	0.8654	0.99995	no

ENSG00000132356	ENSG00000132356	PRKAA1	5:40759480-40798476	q1	q2	OK	22.8831	26.8383	0.23001	0.301305	0.58305	0.99995	no

ENSG00000132357	ENSG00000132357	CARD6	5:40841285-40860275	q1	q2	NOTEST	0.11375	0.0641058	-0.827342	0	1	1	no

ENSG00000132359	ENSG00000132359	RAP1GAP2	17:2680349-2941033	q1	q2	OK	0.440344	0.783491	0.831287	0.506878	0.23285	0.99995	no

ENSG00000132361	ENSG00000132361	CLUH	17:2592679-2616127	q1	q2	OK	12.5324	10.9803	-0.190734	-0.239446	0.58045	0.99995	no

ENSG00000132376	ENSG00000132376	INPP5K	17:1397864-1420182	q1	q2	OK	9.97791	8.67043	-0.202634	-0.240173	0.60565	0.99995	no

ENSG00000132382	ENSG00000132382	MYBBP1A	17:4400688-4458926	q1	q2	OK	11.5791	11.8018	0.0274869	0.0416381	0.9388	0.99995	no

ENSG00000132383	ENSG00000132383	RPA1	17:1682778-1803376	q1	q2	OK	60.873	58.4812	-0.0578275	-0.0841488	0.88635	0.99995	no

ENSG00000132386	ENSG00000132386	SERPINF1	17:1665252-1680868	q1	q2	OK	18.2188	16.5746	-0.136453	-0.171472	0.77635	0.99995	no

ENSG00000132388	ENSG00000132388	UBE2G1	17:4172553-4269923	q1	q2	OK	42.5777	44.6784	0.0694816	0.142739	0.82935	0.99995	no

ENSG00000132394	ENSG00000132394	EEFSEC	3:127770483-128127485	q1	q2	OK	7.94537	8.09258	0.0264844	0.0119278	0.98995	0.99995	no

ENSG00000132405	ENSG00000132405	TBC1D14	4:6910968-7059679	q1	q2	OK	30.0749	28.9273	-0.0561278	-0.107065	0.8643	0.99995	no

ENSG00000132406	ENSG00000132406	TMEM128	4:4237268-4249950	q1	q2	OK	28.1468	26.8029	-0.0705809	-0.137558	0.84115	0.99995	no

ENSG00000132423	ENSG00000132423	COQ3	6:99817275-99842080	q1	q2	OK	4.85681	4.77569	-0.0242994	-0.0331118	0.9608	0.99995	no

ENSG00000132424	ENSG00000132424	PNISR	6:99845926-99879249	q1	q2	OK	136.013	138.625	0.0274386	0.0577188	0.93265	0.99995	no

ENSG00000132429	ENSG00000132429	POPDC3	6:105544696-105627870	q1	q2	OK	1.50549	0.939691	-0.679976	-0.283728	0.66885	0.99995	no

ENSG00000132432	ENSG00000132432	SEC61G	7:54819942-54872621	q1	q2	OK	173.041	196.081	0.180332	0.399765	0.56945	0.99995	no

ENSG00000132434	ENSG00000132434	LANCL2	7:55433140-55501435	q1	q2	OK	10.6604	9.98428	-0.0945349	-0.134776	0.7928	0.99995	no

ENSG00000132436	ENSG00000132436	FIGNL1	7:50511830-50518088	q1	q2	OK	10.91	10.8086	-0.0134594	-0.0229721	0.9691	0.99995	no

ENSG00000132437	ENSG00000132437	DDC	7:50526133-50633154	q1	q2	OK	2.94395	4.26415	0.534504	0.6072	0.29785	0.99995	no

ENSG00000132446	ENSG00000132446	FTHL17	X:31089359-31090170	q1	q2	NOTEST	0	0	0	0	1	1	no

ENSG00000132463	ENSG00000132463	GRSF1	4:71681498-71705662	q1	q2	OK	58.1479	60.1899	0.0497937	0.113677	0.8684	0.99995	no

ENSG00000132464	ENSG00000132464	ENAM	4:71494460-71552533	q1	q2	NOTEST	0	0.00418877	inf	0	1	1	no

ENSG00000132465	ENSG00000132465	IGJ	4:71494460-71552533	q1	q2	NOTEST	0.262573	0.0984541	-1.41519	0	1	1	no

ENSG00000132466	ENSG00000132466	ANKRD17	4:73939092-74124515	q1	q2	OK	26.8018	27.4381	0.0338509	0.0575406	0.90675	0.99995	no

ENSG00000132467	ENSG00000132467	UTP3	4:71554195-71556267	q1	q2	OK	26.009	27.4601	0.078327	0.179376	0.7984	0.99995	no

ENSG00000132470	ENSG00000132470	ITGB4	17:73717407-73761792	q1	q2	NOTEST	0.0582057	0.00871139	-2.74019	0	1	1	no

ENSG00000132471	ENSG00000132471	WBP2	17:73841779-73852588	q1	q2	OK	26.9778	25.1128	-0.103348	-0.202851	0.73695	0.99995	no

ENSG00000132475	ENSG00000132475	H3F3B	17:73772514-73821886	q1	q2	OK	383.406	390.639	0.0269648	0.041454	0.9518	0.99995	no

ENSG00000132478	ENSG00000132478	UNK	17:73772514-73821886	q1	q2	OK	24.1995	22.8536	-0.0825504	-0.0519296	0.908	0.99995	no

ENSG00000132481	ENSG00000132481	TRIM47	17:73870241-73875627	q1	q2	OK	2.66	1.71783	-0.630835	-0.373981	0.4576	0.99995	no

ENSG00000132485	ENSG00000132485	ZRANB2	1:71514537-71546980	q1	q2	OK	79.1517	83.2501	0.0728323	0.146881	0.80905	0.99995	no

ENSG00000132498	ENSG00000132498	ANKRD20A3	9:43064965-43133544	q1	q2	OK	3.62069	3.5533	-0.0271086	-0.0330271	0.9608	0.99995	no

ENSG00000132507	ENSG00000132507	EIF5A	17:7210317-7232712	q1	q2	OK	206.025	210.923	0.0338951	0.0656336	0.9257	0.99995	no

ENSG00000132510	ENSG00000132510	KDM6B	17:7743221-7758114	q1	q2	OK	14.7428	15.0177	0.0266552	0.0396771	0.9262	0.99995	no

ENSG00000132514	ENSG00000132514	CLEC10A	17:6977855-6983626	q1	q2	NOTEST	0	0	0	0	1	1	no

ENSG00000132517	ENSG00000132517	SLC52A1	17:4935894-4955304	q1	q2	NOTEST	0.00896927	0.0164834	0.877954	0	1	1	no

ENSG00000132518	ENSG00000132518	GUCY2D	17:7905911-7923657	q1	q2	NOTEST	0	0	0	0	1	1	no

ENSG00000132522	ENSG00000132522	GPS2	17:7210317-7232712	q1	q2	OK	40.997	41.8718	0.0304595	0.0229817	0.967	0.99995	no

ENSG00000132530	ENSG00000132530	XAF1	17:6658765-6678966	q1	q2	NOTEST	0.342101	0.448647	0.391158	0	1	1	no

ENSG00000132535	ENSG00000132535	DLG4	17:7093208-7128592	q1	q2	OK	33.7917	30.7457	-0.136282	-0.176307	0.77485	0.99995	no

ENSG00000132541	ENSG00000132541	HRSP12	8:99114571-99129469	q1	q2	OK	20.2722	24.3018	0.261563	0.454711	0.4842	0.99995	no

ENSG00000132549	ENSG00000132549	VPS13B	8:100025493-100906290	q1	q2	OK	10.8042	10.5761	-0.0307745	-0.0411643	0.9495	0.99995	no

ENSG00000132554	ENSG00000132554	RGS22	8:100973163-101143496	q1	q2	NOTEST	0.0498524	0.0381789	-0.384885	0	1	1	no

ENSG00000132561	ENSG00000132561	MATN2	8:98881067-99058697	q1	q2	OK	3.95323	3.45437	-0.19461	-0.0567414	0.81485	0.99995	no

ENSG00000132563	ENSG00000132563	REEP2	5:137774705-137782658	q1	q2	OK	26.2028	20.606	-0.346653	-0.599833	0.31025	0.99995	no

ENSG00000132570	ENSG00000132570	PCBD2	5:134240595-134347392	q1	q2	OK	8.75793	9.02799	0.0438162	0.047702	0.9361	0.99995	no

ENSG00000132581	ENSG00000132581	SDF2	17:26975373-27029697	q1	q2	OK	41.6436	39.3129	-0.0830914	-0.0734149	0.8983	0.99995	no

ENSG00000132589	ENSG00000132589	FLOT2	17:27206352-27224697	q1	q2	OK	45.4922	44.6977	-0.0254192	-0.053138	0.9318	0.99995	no

ENSG00000132591	ENSG00000132591	ERAL1	17:27082995-27188085	q1	q2	OK	32.8729	30.303	-0.117437	-0.191776	0.77085	0.99995	no

ENSG00000132600	ENSG00000132600	PRMT7	16:68344876-68482591	q1	q2	OK	32.0223	30.2698	-0.0811983	-0.121436	0.8453	0.99995	no

ENSG00000132603	ENSG00000132603	NIP7	16:69139466-69442474	q1	q2	OK	12.9005	14.5204	0.170657	0.084048	0.8502	0.99995	no

ENSG00000132604	ENSG00000132604	TERF2	16:69139466-69442474	q1	q2	OK	21.1469	20.1344	-0.0707865	-0.0462007	0.9297	0.99995	no

ENSG00000132612	ENSG00000132612	VPS4A	16:69139466-69442474	q1	q2	OK	49.3556	48.3709	-0.0290741	-0.0311453	0.96665	0.99995	no

ENSG00000132613	ENSG00000132613	MTSS1L	16:70695106-70719969	q1	q2	OK	23.4421	22.6393	-0.0502778	-0.0555649	0.90415	0.99995	no

ENSG00000132622	ENSG00000132622	HSPA12B	20:3713313-3733758	q1	q2	OK	3.27231	3.03581	-0.108229	-0.154625	0.83205	0.99995	no

ENSG00000132623	ENSG00000132623	ANKEF1	20:9966735-10349424	q1	q2	OK	0.781332	0.915766	0.229043	0.157693	0.81955	0.99995	no

ENSG00000132631	ENSG00000132631	SCP2D1	20:18790371-18810797	q1	q2	NOTEST	0	0.0503357	inf	0	1	1	no

ENSG00000132635	ENSG00000132635	PCED1A	20:2815959-3019722	q1	q2	OK	25.6509	21.6279	-0.246116	-0.206184	0.7207	0.99995	no

ENSG00000132639	ENSG00000132639	SNAP25	20:9966735-10349424	q1	q2	OK	6.95083	7.29718	0.0701531	0.112255	0.87035	0.99995	no

ENSG00000132640	ENSG00000132640	BTBD3	20:11871370-11907257	q1	q2	OK	33.7841	36.397	0.107474	0.189192	0.69995	0.99995	no

ENSG00000132646	ENSG00000132646	PCNA	20:5095598-5107272	q1	q2	OK	271.956	269.706	-0.0119868	-0.02918	0.96365	0.99995	no

ENSG00000132661	ENSG00000132661	NXT1	20:23327577-23335414	q1	q2	OK	24.3553	22.1762	-0.135222	-0.255942	0.71705	0.99995	no

ENSG00000132664	ENSG00000132664	POLR3F	20:18364010-18465287	q1	q2	OK	9.98928	10.5433	0.0778762	0.120599	0.8408	0.99995	no

ENSG00000132669	ENSG00000132669	RIN2	20:19867164-19983101	q1	q2	OK	5.88697	5.47337	-0.105097	-0.134571	0.78895	0.99995	no

ENSG00000132670	ENSG00000132670	PTPRA	20:2815959-3019722	q1	q2	OK	65.2115	60.0321	-0.119391	-0.203033	0.7356	0.99995	no

ENSG00000132671	ENSG00000132671	SSTR4	20:23012178-23019873	q1	q2	NOTEST	0	0	0	0	1	1	no

ENSG00000132676	ENSG00000132676	DAP3	1:155579566-155829191	q1	q2	OK	63.3961	61.9411	-0.0334972	-0.0350571	0.9466	0.99995	no

ENSG00000132677	ENSG00000132677	RHBG	1:156339002-156355011	q1	q2	NOTEST	0.0193626	0	#NAME?	0	1	1	no

ENSG00000132680	ENSG00000132680	KIAA0907	1:155882833-155904191	q1	q2	OK	33.6362	34.2187	0.0247721	0.0430305	0.93515	0.99995	no

ENSG00000132681	ENSG00000132681	ATP1A4	1:160121359-160156767	q1	q2	NOTEST	0.0487946	0.054379	0.156327	0	1	1	no

ENSG00000132688	ENSG00000132688	NES	1:156638554-156647189	q1	q2	OK	352.88	342.354	-0.0436911	-0.073749	0.91765	0.99995	no

ENSG00000132692	ENSG00000132692	BCAN	1:156611181-156631216	q1	q2	OK	0.884809	0.899552	0.0238399	0.0216865	0.9685	0.99995	no

ENSG00000132693	ENSG00000132693	CRP	1:159682078-159684379	q1	q2	NOTEST	0	0	0	0	1	1	no

ENSG00000132694	ENSG00000132694	ARHGEF11	1:156904631-157015162	q1	q2	OK	14.5866	15.3946	0.0777795	0.120861	0.80115	0.99995	no

ENSG00000132698	ENSG00000132698	RAB25	1:156030950-156040295	q1	q2	OK	0.496484	0.688349	0.471391	0.366753	0.57415	0.99995	no

ENSG00000132702	ENSG00000132702	HAPLN2	1:156589085-156595517	q1	q2	NOTEST	0.485168	0.464257	-0.0635607	0	1	1	no

ENSG00000132703	ENSG00000132703	APCS	1:159557614-159558655	q1	q2	NOTEST	0	0	0	0	1	1	no

ENSG00000132704	ENSG00000132704	FCRL2	1:157715522-157746922	q1	q2	NOTEST	0	0.0481642	inf	0	1	1	no

ENSG00000132716	ENSG00000132716	DCAF8	1:160185504-160256138	q1	q2	OK	81.9742	72.1302	-0.184565	-0.245642	0.659	0.99995	no

ENSG00000132718	ENSG00000132718	SYT11	1:155829299-155854990	q1	q2	OK	30.2658	30.0079	-0.0123458	-0.0302536	0.9636	0.99995	no

ENSG00000132740	ENSG00000132740	IGHMBP2	11:68671309-68708070	q1	q2	OK	8.93299	9.69344	0.117866	0.12946	0.78605	0.99995	no

ENSG00000132744	ENSG00000132744	ACY3	11:67410025-67418130	q1	q2	NOTEST	0	0	0	0	1	1	no

ENSG00000132746	ENSG00000132746	ALDH3B2	11:67429632-67448671	q1	q2	NOTEST	0	0	0	0	1	1	no

ENSG00000132749	ENSG00000132749	MTL5	11:68474907-68519032	q1	q2	OK	0.508981	1.21062	1.25006	0.869485	0.10165	0.99995	no

ENSG00000132763	ENSG00000132763	MMACHC	1:45959537-45988719	q1	q2	OK	3.70919	3.50646	-0.0810914	-0.0429595	0.94735	0.99995	no

ENSG00000132768	ENSG00000132768	DPH2	1:44435671-44439041	q1	q2	OK	13.0618	12.5029	-0.0630912	-0.0987271	0.87055	0.99995	no

ENSG00000132773	ENSG00000132773	TOE1	1:45794834-45956872	q1	q2	OK	9.83207	10.0707	0.0345905	0.049468	0.94135	0.99995	no

ENSG00000132780	ENSG00000132780	NASP	1:46049517-46084566	q1	q2	OK	350.427	366.575	0.0649934	0.101802	0.86555	0.99995	no

ENSG00000132781	ENSG00000132781	MUTYH	1:45794834-45956872	q1	q2	OK	19.383	18.1089	-0.0980993	-0.133317	0.8303	0.99995	no

ENSG00000132792	ENSG00000132792	CTNNBL1	20:36322407-36500531	q1	q2	OK	32.5863	33.3789	0.0346702	0.0799569	0.9074	0.99995	no

ENSG00000132793	ENSG00000132793	LPIN3	20:39969559-39995467	q1	q2	NOTEST	0.354211	0.231877	-0.611252	0	1	1	no

ENSG00000132801	ENSG00000132801	ZSWIM3	20:44486255-44507761	q1	q2	OK	3.18952	3.21862	0.0131039	0.0199947	0.979	0.99995	no

ENSG00000132819	ENSG00000132819	RBM38	20:55959215-55984389	q1	q2	OK	16.2955	13.8639	-0.23314	-0.468655	0.46915	0.99995	no

ENSG00000132821	ENSG00000132821	VSTM2L	20:36531498-36573752	q1	q2	OK	4.60223	2.95948	-0.636987	-0.868012	0.19955	0.99995	no

ENSG00000132823	ENSG00000132823	OSER1	20:42825135-42839431	q1	q2	OK	19.7399	19.7671	0.00198907	0.00404326	0.9953	0.99995	no

ENSG00000132824	ENSG00000132824	SERINC3	20:43124861-43150750	q1	q2	OK	15.9649	15.7586	-0.0187561	-0.0395896	0.9518	0.99995	no

ENSG00000132825	ENSG00000132825	PPP1R3D	20:58508818-58523735	q1	q2	OK	1.84887	2.18803	0.242994	0.172625	0.8097	0.99995	no

ENSG00000132832	ENSG00000132832	RP11-445H22.3	20:43285091-43379675	q1	q2	NOTEST	0	0.0596383	inf	0	1	1	no

ENSG00000132837	ENSG00000132837	DMGDH	5:78293437-78531861	q1	q2	OK	0.534435	0.206862	-1.36935	-0.584781	0.09305	0.99995	no

ENSG00000132840	ENSG00000132840	BHMT2	5:78293437-78531861	q1	q2	NOTEST	0.14463	0.0657872	-1.13649	0	1	1	no

ENSG00000132842	ENSG00000132842	AP3B1	5:77296348-77590579	q1	q2	OK	24.5844	25.4926	0.0523366	0.111193	0.858	0.99995	no

ENSG00000132846	ENSG00000132846	ZBED3	5:76367896-76462734	q1	q2	OK	5.0873	5.01531	-0.020562	-0.0198458	0.95835	0.99995	no

ENSG00000132849	ENSG00000132849	INADL	1:62208148-62629592	q1	q2	OK	1.6087	1.15221	-0.48149	-0.242624	0.51205	0.99995	no

ENSG00000132854	ENSG00000132854	KANK4	1:62702650-62785085	q1	q2	OK	1.0329	0.954907	-0.11327	-0.121521	0.86845	0.99995	no

ENSG00000132855	ENSG00000132855	ANGPTL3	1:62920398-63153969	q1	q2	NOTEST	0.0455346	0.0687407	0.594201	0	1	1	no

ENSG00000132872	ENSG00000132872	SYT4	18:40847842-40857615	q1	q2	OK	8.58057	7.56053	-0.182585	-0.345654	0.58905	0.99995	no

ENSG00000132874	ENSG00000132874	SLC14A2	18:42792959-43332485	q1	q2	NOTEST	0.0163704	0.00578887	-1.49974	0	1	1	no

ENSG00000132879	ENSG00000132879	FBXO44	1:11708423-11723384	q1	q2	OK	7.48517	5.43673	-0.461296	-0.631278	0.33725	0.99995	no

ENSG00000132881	ENSG00000132881	RSG1	1:16558194-16563657	q1	q2	OK	4.51013	4.80428	0.0911529	0.065804	0.9149	0.99995	no

ENSG00000132906	ENSG00000132906	CASP9	1:15783222-15853029	q1	q2	OK	6.49371	5.45888	-0.250438	-0.370758	0.5666	0.99995	no

ENSG00000132911	ENSG00000132911	NMUR2	5:151771092-151812929	q1	q2	NOTEST	0.179445	0.141336	-0.344406	0	1	1	no

ENSG00000132912	ENSG00000132912	DCTN4	5:150088001-150138671	q1	q2	OK	42.8468	44.6577	0.0597216	0.110074	0.82445	0.99995	no

ENSG00000132915	ENSG00000132915	PDE6A	5:149237518-149324356	q1	q2	NOTEST	0.00871681	0	#NAME?	0	1	1	no

ENSG00000132932	ENSG00000132932	ATP8A2	13:25946208-26599989	q1	q2	OK	1.81845	1.58694	-0.196461	-0.189858	0.6791	0.99995	no

ENSG00000132938	ENSG00000132938	MTUS2	13:29598747-30077892	q1	q2	OK	0.894199	0.620997	-0.526009	-0.378242	0.4135	0.99995	no

ENSG00000132950	ENSG00000132950	ZMYM5	13:20397621-20437776	q1	q2	OK	9.81932	9.59839	-0.0328294	-0.0552162	0.93615	0.99995	no

ENSG00000132952	ENSG00000132952	USPL1	13:31191829-31233686	q1	q2	OK	12.1865	12.1455	-0.00485972	-0.00912254	0.9895	0.99995	no

ENSG00000132953	ENSG00000132953	XPO4	13:21351468-21477187	q1	q2	OK	8.17567	9.87002	0.271717	0.519167	0.35365	0.99995	no

ENSG00000132958	ENSG00000132958	TPTE2	13:19997016-20110903	q1	q2	NOTEST	0.208342	0.196954	-0.0810969	0	1	1	no

ENSG00000132963	ENSG00000132963	POMP	13:29233240-29253062	q1	q2	OK	75.0047	80.3394	0.0991279	0.237306	0.7313	0.99995	no

ENSG00000132964	ENSG00000132964	CDK8	13:26828275-26979375	q1	q2	OK	21.3364	23.4729	0.137685	0.283372	0.67345	0.99995	no

ENSG00000132965	ENSG00000132965	ALOX5AP	13:31309644-31338556	q1	q2	NOTEST	0.0340497	0.0332967	-0.0322644	0	1	1	no

ENSG00000132967	ENSG00000132967	HMGB1P5	3:22423309-22424420	q1	q2	OK	53.6953	63.3603	0.238783	0.476198	0.4981	0.99995	no

ENSG00000132970	ENSG00000132970	WASF3	13:27131839-27263085	q1	q2	OK	21.9883	22.3604	0.0242073	0.0585176	0.9348	0.99995	no

ENSG00000132972	ENSG00000132972	RNF17	13:25338289-25454059	q1	q2	NOTEST	0	0.0114902	inf	0	1	1	no

ENSG00000132975	ENSG00000132975	GPR12	13:27329340-27334922	q1	q2	NOTEST	0.0514357	0.0138461	-1.89329	0	1	1	no

ENSG00000133019	ENSG00000133019	CHRM3	1:239549864-240080255	q1	q2	NOTEST	0.438531	0.37838	-0.212841	0	1	1	no

ENSG00000133020	ENSG00000133020	MYH8	17:10286448-10527203	q1	q2	NOTEST	0.00428107	0	#NAME?	0	1	1	no

ENSG00000133026	ENSG00000133026	MYH10	17:8316448-8534079	q1	q2	OK	110.803	120.54	0.121514	0.160227	0.73055	0.99995	no

ENSG00000133027	ENSG00000133027	PEMT	17:17408876-17513799	q1	q2	OK	17.3021	16.8163	-0.0410852	-0.06535	0.91675	0.99995	no

ENSG00000133028	ENSG00000133028	SCO1	17:10583653-10718481	q1	q2	OK	16.8053	18.1124	0.108064	0.188745	0.765	0.99995	no

ENSG00000133030	ENSG00000133030	MPRIP	17:16945858-17140502	q1	q2	OK	76.6287	73.5338	-0.0594778	-0.0570708	0.89675	0.99995	no

ENSG00000133048	ENSG00000133048	CHI3L1	1:203148058-203155877	q1	q2	NOTEST	0.470181	0.206534	-1.18684	0	1	1	no

ENSG00000133055	ENSG00000133055	MYBPH	1:203136938-203144941	q1	q2	NOTEST	0.0155942	0	#NAME?	0	1	1	no

ENSG00000133056	ENSG00000133056	PIK3C2B	1:204346977-204463852	q1	q2	OK	21.9831	19.0465	-0.206874	-0.174511	0.68725	0.99995	no

ENSG00000133059	ENSG00000133059	DSTYK	1:205111631-205180727	q1	q2	OK	5.34707	5.19585	-0.0413891	-0.0902012	0.898	0.99995	no

ENSG00000133063	ENSG00000133063	CHIT1	1:203181954-203242769	q1	q2	NOTEST	0	0.0176275	inf	0	1	1	no

ENSG00000133065	ENSG00000133065	SLC41A1	1:205758220-205782876	q1	q2	OK	9.16458	9.18203	0.00274346	0.00498257	0.99345	0.99995	no

ENSG00000133067	ENSG00000133067	LGR6	1:202163028-202288909	q1	q2	NOTEST	0.19329	0.0581455	-1.73303	0	1	1	no

ENSG00000133069	ENSG00000133069	TMCC2	1:205197303-205242471	q1	q2	OK	4.03515	4.3228	0.0993423	0.168545	0.80625	0.99995	no

ENSG00000133083	ENSG00000133083	DCLK1	13:36345477-36705443	q1	q2	OK	19.0904	20.1955	0.0811858	0.181886	0.7905	0.99995	no

ENSG00000133101	ENSG00000133101	CCNA1	13:37005966-37017019	q1	q2	OK	0.690824	0.747823	0.114378	0.10661	0.8664	0.99995	no

ENSG00000133103	ENSG00000133103	COG6	13:40229763-40365802	q1	q2	OK	11.362	11.3765	0.00183133	0.00264455	0.99495	0.99995	no

ENSG00000133104	ENSG00000133104	SPG20	13:36875774-36944317	q1	q2	OK	42.839	47.518	0.149548	0.284941	0.6418	0.99995	no

ENSG00000133105	ENSG00000133105	RXFP2	13:32313673-32377009	q1	q2	NOTEST	0.00911613	0	#NAME?	0	1	1	no

ENSG00000133106	ENSG00000133106	EPSTI1	13:43460523-43566407	q1	q2	NOTEST	0.125508	0.117232	-0.0984087	0	1	1	no

ENSG00000133107	ENSG00000133107	TRPC4	13:38210772-38444562	q1	q2	OK	1.79274	1.7101	-0.0680846	-0.0870157	0.8924	0.99995	no

ENSG00000133110	ENSG00000133110	POSTN	13:38136719-38172981	q1	q2	OK	45.9407	46.6197	0.0211666	0.0347646	0.9545	0.99995	no

ENSG00000133111	ENSG00000133111	RFXAP	13:37393360-37403241	q1	q2	OK	6.56931	6.26989	-0.0673017	-0.115892	0.8674	0.99995	no

ENSG00000133112	ENSG00000133112	TPT1	13:45911007-45965872	q1	q2	OK	1520.41	1599.32	0.0729949	0.134762	0.84215	0.99995	no

ENSG00000133114	ENSG00000133114	GPALPP1	13:45513383-45657260	q1	q2	OK	17.189	17.5076	0.0264953	0.0412126	0.95075	0.99995	no

ENSG00000133115	ENSG00000133115	STOML3	13:39540061-39565203	q1	q2	NOTEST	0.0264086	0.136951	2.37458	0	1	1	no

ENSG00000133116	ENSG00000133116	KL	13:33590206-33640282	q1	q2	NOTEST	0.0477682	0.0249754	-0.935541	0	1	1	no

ENSG00000133119	ENSG00000133119	RFC3	13:34392185-34540695	q1	q2	OK	30.2325	35.0384	0.21284	0.498535	0.47395	0.99995	no

ENSG00000133121	ENSG00000133121	STARD13	13:33677271-33924767	q1	q2	OK	3.61339	3.80561	0.0747764	0.105756	0.83745	0.99995	no

ENSG00000133124	ENSG00000133124	IRS4	X:107963178-107982133	q1	q2	NOTEST	0.0524706	0.0518835	-0.0162317	0	1	1	no

ENSG00000133131	ENSG00000133131	MORC4	X:106045909-106243474	q1	q2	OK	6.60291	6.59529	-0.00166441	-0.00254843	0.99685	0.99995	no

ENSG00000133134	ENSG00000133134	BEX2	X:102564273-102565974	q1	q2	OK	14.4637	11.2842	-0.358135	-0.553522	0.4421	0.99995	no

ENSG00000133135	ENSG00000133135	RNF128	X:105937023-106040223	q1	q2	NOTEST	0	0.0482184	inf	0	1	1	no

ENSG00000133136	ENSG00000133136	GNG5P2	X:109437413-109699562	q1	q2	OK	0.078503	0.790668	3.33225	0.357877	0.3432	0.99995	no

ENSG00000133138	ENSG00000133138	TBC1D8B	X:106045909-106243474	q1	q2	OK	0.878637	0.838791	-0.0669563	-0.0469263	0.9442	0.99995	no

ENSG00000133142	ENSG00000133142	TCEAL4	X:102831158-102842657	q1	q2	OK	62.7495	65.5466	0.0629186	0.144701	0.83835	0.99995	no

ENSG00000133149	ENSG00000133149	TEX13A	X:103810995-105011822	q1	q2	NOTEST	0	0	0	0	1	1	no

ENSG00000133169	ENSG00000133169	BEX1	X:102317578-102319168	q1	q2	OK	67.8387	57.497	-0.238624	-0.527995	0.45515	0.99995	no

ENSG00000133193	ENSG00000133193	FAM104A	17:71161115-71258491	q1	q2	OK	11.4812	12.4217	0.113592	0.133431	0.84655	0.99995	no

ENSG00000133195	ENSG00000133195	SLC39A11	17:70642087-71088851	q1	q2	OK	4.0285	5.17642	0.36171	0.411596	0.38355	0.99995	no

ENSG00000133216	ENSG00000133216	EPHB2	1:23037331-23241818	q1	q2	OK	27.4113	24.3623	-0.170118	-0.385994	0.55785	0.99995	no

ENSG00000133226	ENSG00000133226	SRRM1	1:24958206-24999758	q1	q2	OK	56.025	56.0279	7.55E-05	0.0001226	0.99955	0.99995	no

ENSG00000133243	ENSG00000133243	BTBD2	19:1985446-2034880	q1	q2	OK	48.0739	41.038	-0.228292	-0.52288	0.4335	0.99995	no

ENSG00000133246	ENSG00000133246	PRAM1	19:8554939-8567996	q1	q2	NOTEST	0.288563	0.271367	-0.0886412	0	1	1	no

ENSG00000133247	ENSG00000133247	SUV420H2	19:55851220-55859488	q1	q2	OK	14.7772	13.1259	-0.170951	-0.261025	0.678	0.99995	no

ENSG00000133250	ENSG00000133250	ZNF414	19:8575461-8579048	q1	q2	OK	14.7268	15.3515	0.0599398	0.0713029	0.911	0.99995	no

ENSG00000133256	ENSG00000133256	PDE6B	4:619372-664571	q1	q2	OK	0.957881	0.859236	-0.156792	-0.0706199	0.85435	0.99995	no

ENSG00000133265	ENSG00000133265	HSPBP1	19:55773598-55791749	q1	q2	OK	28.8707	27.4255	-0.074086	-0.131961	0.85015	0.99995	no

ENSG00000133275	ENSG00000133275	CSNK1G2	19:1941187-1981337	q1	q2	OK	52.1454	48.4458	-0.106168	-0.198211	0.72655	0.99995	no

ENSG00000133302	ENSG00000133302	ANKRD32	5:93488670-94620279	q1	q2	OK	8.87382	10.838	0.288476	0.29069	0.6179	0.99995	no

ENSG00000133313	ENSG00000133313	CNDP2	18:72163050-72188366	q1	q2	OK	62.8495	69.17	0.138247	0.240332	0.6782	0.99995	no

ENSG00000133315	ENSG00000133315	MACROD1	11:63742078-63933578	q1	q2	OK	8.64074	9.53642	0.142293	0.0554385	0.90835	0.99995	no

ENSG00000133316	ENSG00000133316	WDR74	11:62599705-62609281	q1	q2	OK	20.2048	20.7149	0.0359698	0.0591575	0.9284	0.99995	no

ENSG00000133317	ENSG00000133317	LGALS12	11:63273555-63284246	q1	q2	NOTEST	0	0	0	0	1	1	no

ENSG00000133318	ENSG00000133318	RTN3	11:63448917-63527363	q1	q2	OK	152.585	136.936	-0.156112	-0.3675	0.5871	0.99995	no

ENSG00000133321	ENSG00000133321	RARRES3	11:63304280-63313934	q1	q2	NOTEST	0.213437	0.20678	-0.0457124	0	1	1	no

ENSG00000133328	ENSG00000133328	HRASLS2	11:63320241-63330855	q1	q2	NOTEST	0.0462936	0.0452974	-0.031384	0	1	1	no

ENSG00000133392	ENSG00000133392	MYH11	16:15737123-15950890	q1	q2	NOTEST	0.164243	0.215341	0.390789	0	1	1	no

ENSG00000133393	ENSG00000133393	FOPNL	16:15959576-15982482	q1	q2	OK	18.1595	19.2532	0.0843721	0.172964	0.7899	0.99995	no

ENSG00000133398	ENSG00000133398	MED10	5:6371993-6378707	q1	q2	OK	26.3601	28.2247	0.0986032	0.192708	0.7819	0.99995	no

ENSG00000133401	ENSG00000133401	PDZD2	5:31639516-32122047	q1	q2	OK	2.23229	3.28945	0.559323	0.52622	0.2192	0.99995	no

ENSG00000133422	ENSG00000133422	MORC2	22:31318294-31364284	q1	q2	OK	22.0589	23.3072	0.079411	0.114059	0.85775	0.99995	no

ENSG00000133424	ENSG00000133424	LARGE	22:33558211-34318829	q1	q2	OK	12.1422	11.7722	-0.044642	-0.089248	0.8846	0.99995	no

ENSG00000133433	ENSG00000133433	GSTT2B	22:24299600-24303373	q1	q2	NOTEST	0	0.0273783	inf	0	1	1	no

ENSG00000133454	ENSG00000133454	MYO18B	22:26138110-26427007	q1	q2	NOTEST	0.0427219	0.0302435	-0.498348	0	1	1	no

ENSG00000133460	ENSG00000133460	SLC2A11	22:24198613-24241117	q1	q2	OK	11.4511	10.7643	-0.0892402	-0.0667987	0.90165	0.99995	no

ENSG00000133466	ENSG00000133466	C1QTNF6	22:37521877-37595425	q1	q2	OK	7.79132	7.11367	-0.131275	-0.225707	0.72475	0.99995	no

ENSG00000133475	ENSG00000133475	GGT2	22:21562261-21581926	q1	q2	NOTEST	0	0	0	0	1	1	no

ENSG00000133477	ENSG00000133477	FAM83F	22:40390952-40440534	q1	q2	NOTEST	0.00463412	0	#NAME?	0	1	1	no

ENSG00000133488	ENSG00000133488	SEC14L4	22:30879340-30901698	q1	q2	NOTEST	0	0.0200067	inf	0	1	1	no

ENSG00000133519	ENSG00000133519	ZDHHC8P1	22:23732792-23744913	q1	q2	NOTEST	0.39894	0.146543	-1.44485	0	1	1	no

ENSG00000133561	ENSG00000133561	GIMAP6	7:150322462-150329473	q1	q2	NOTEST	0	0.00709135	inf	0	1	1	no

ENSG00000133574	ENSG00000133574	GIMAP4	7:150264364-150271041	q1	q2	NOTEST	0	0	0	0	1	1	no

ENSG00000133597	ENSG00000133597	ADCK2	7:140218219-140624564	q1	q2	OK	3.40824	2.78464	-0.291537	-0.143656	0.86285	0.99995	no

ENSG00000133606	ENSG00000133606	MKRN1	7:140152839-140179369	q1	q2	OK	49.0839	47.1007	-0.0595026	-0.109384	0.84665	0.99995	no

ENSG00000133612	ENSG00000133612	AGAP3	7:150782917-150841523	q1	q2	OK	39.9699	38.0934	-0.0693746	-0.117915	0.8084	0.99995	no

ENSG00000133619	ENSG00000133619	KRBA1	7:149411871-149431664	q1	q2	OK	7.85343	6.42281	-0.290116	-0.326461	0.5037	0.99995	no

ENSG00000133624	ENSG00000133624	ZNF767	7:149244244-149321843	q1	q2	OK	10.2899	11.0799	0.106717	0.143382	0.78945	0.99995	no

ENSG00000133627	ENSG00000133627	ACTR3B	7:152456833-152552463	q1	q2	OK	8.18573	8.58395	0.0685299	0.0986607	0.864	0.99995	no

ENSG00000133636	ENSG00000133636	NTS	12:86268072-86276770	q1	q2	OK	0.470904	0.747121	0.665909	0.559112	0.3818	0.99995	no

ENSG00000133639	ENSG00000133639	BTG1	12:92378755-92583436	q1	q2	OK	72.8904	67.3621	-0.113792	-0.257461	0.69595	0.99995	no

ENSG00000133640	ENSG00000133640	LRRIQ1	12:85430091-85657002	q1	q2	OK	3.87092	4.60645	0.250979	0.235079	0.63165	0.99995	no

ENSG00000133641	ENSG00000133641	C12orf29	12:88373815-88535993	q1	q2	OK	12.3007	11.8681	-0.051657	-0.0565651	0.92745	0.99995	no

ENSG00000133657	ENSG00000133657	ATP13A3	3:194123400-194239008	q1	q2	OK	27.5399	34.2554	0.31481	0.423837	0.32555	0.99995	no

ENSG00000133661	ENSG00000133661	SFTPD	10:81664653-81742370	q1	q2	NOTEST	0.0670735	0.0227835	-1.55775	0	1	1	no

ENSG00000133665	ENSG00000133665	DYDC2	10:82095860-82127829	q1	q2	OK	1.56105	1.81695	0.219004	0.130944	0.833	0.99995	no

ENSG00000133678	ENSG00000133678	TMEM254	10:81806615-81852313	q1	q2	OK	22.907	23.7062	0.049477	0.0958054	0.87595	0.99995	no

ENSG00000133687	ENSG00000133687	TMTC1	12:29653772-29937692	q1	q2	OK	1.94085	1.80263	-0.10658	-0.149747	0.8122	0.99995	no

ENSG00000133703	ENSG00000133703	KRAS	12:25348149-25403870	q1	q2	OK	22.6518	24.8997	0.1365	0.314288	0.6454	0.99995	no

ENSG00000133704	ENSG00000133704	IPO8	12:30781921-30848920	q1	q2	OK	17.7029	19.5855	0.145806	0.234583	0.66605	0.99995	no

ENSG00000133706	ENSG00000133706	LARS	5:145463948-145562223	q1	q2	OK	58.9243	59.9971	0.0260305	0.0432985	0.9266	0.99995	no

ENSG00000133710	ENSG00000133710	SPINK5	5:147405245-147516852	q1	q2	NOTEST	0	0	0	0	1	1	no

ENSG00000133731	ENSG00000133731	IMPA1	8:82570195-82598928	q1	q2	OK	17.3963	21.5624	0.309733	0.50597	0.4151	0.99995	no

ENSG00000133739	ENSG00000133739	LRRCC1	8:86019381-86058311	q1	q2	OK	10.4021	11.6467	0.163039	0.321245	0.6354	0.99995	no

ENSG00000133740	ENSG00000133740	E2F5	8:86089459-86132650	q1	q2	OK	25.031	31.6381	0.337952	0.547315	0.41605	0.99995	no

ENSG00000133742	ENSG00000133742	CA1	8:86239836-86393722	q1	q2	NOTEST	0	0.150694	inf	0	1	1	no

ENSG00000133773	ENSG00000133773	CCDC59	12:82617459-82873015	q1	q2	OK	39.5672	40.8531	0.0461409	0.075118	0.9106	0.99995	no

ENSG00000133789	ENSG00000133789	SWAP70	11:9685623-9774538	q1	q2	OK	15.5239	18.9511	0.287789	0.672756	0.34015	0.99995	no

ENSG00000133794	ENSG00000133794	ARNTL	11:13298198-13408813	q1	q2	OK	6.15756	7.65907	0.31481	0.392757	0.41185	0.99995	no

ENSG00000133800	ENSG00000133800	LYVE1	11:10562818-10715535	q1	q2	NOTEST	0.0146211	0.0316145	1.11253	0	1	1	no

ENSG00000133805	ENSG00000133805	AMPD3	11:10329859-10529126	q1	q2	OK	1.37324	0.779016	-0.817863	-0.500056	0.2319	0.99995	no

ENSG00000133808	ENSG00000133808	MICALCL	11:12297626-12380691	q1	q2	NOTEST	0.0250308	0	#NAME?	0	1	1	no

ENSG00000133812	ENSG00000133812	SBF2	11:9776316-10315754	q1	q2	OK	19.4871	18.7131	-0.0584688	-0.0752672	0.8734	0.99995	no

ENSG00000133816	ENSG00000133816	MICAL2	11:12115542-12285334	q1	q2	OK	0.673169	0.584043	-0.204893	-0.0739689	0.72415	0.99995	no

ENSG00000133818	ENSG00000133818	RRAS2	11:14299471-14386052	q1	q2	OK	1.29601	0.221954	-2.54574	-1.38355	0.00035	0.340103	no

ENSG00000133835	ENSG00000133835	HSD17B4	5:118788137-118972894	q1	q2	OK	67.8218	67.9378	0.00246515	0.00407992	0.99375	0.99995	no

ENSG00000133858	ENSG00000133858	ZFC3H1	12:72003251-72097836	q1	q2	OK	22.4411	20.9414	-0.0997878	-0.089215	0.8517	0.99995	no

ENSG00000133863	ENSG00000133863	TEX15	8:30689059-30748122	q1	q2	OK	1.06175	1.15947	0.127029	0.124159	0.80805	0.99995	no

ENSG00000133872	ENSG00000133872	TMEM66	8:29920527-29940983	q1	q2	OK	180.859	167.797	-0.108146	-0.251113	0.7086	0.99995	no

ENSG00000133874	ENSG00000133874	RNF122	8:33405272-33424643	q1	q2	OK	24.7725	22.9583	-0.109726	-0.240042	0.7355	0.99995	no

ENSG00000133878	ENSG00000133878	DUSP26	8:33448855-33457624	q1	q2	OK	2.02923	1.79527	-0.176732	-0.202994	0.76065	0.99995	no

ENSG00000133884	ENSG00000133884	DPF2	11:65101224-65120720	q1	q2	OK	49.9415	45.3471	-0.139228	-0.239819	0.654	0.99995	no

ENSG00000133895	ENSG00000133895	MEN1	11:64570981-64578766	q1	q2	OK	23.9309	21.6471	-0.144701	-0.282502	0.65535	0.99995	no

ENSG00000133935	ENSG00000133935	C14orf1	14:76041230-76550928	q1	q2	OK	26.7359	30.5843	0.194011	0.328195	0.6441	0.99995	no

ENSG00000133937	ENSG00000133937	GSC	14:95234552-95236562	q1	q2	NOTEST	0	0	0	0	1	1	no

ENSG00000133943	ENSG00000133943	C14orf159	14:91336798-91691976	q1	q2	OK	8.26191	7.86694	-0.0706737	-0.0849898	0.8662	0.99995	no

ENSG00000133958	ENSG00000133958	UNC79	14:93799564-94174222	q1	q2	OK	1.91529	1.93153	0.0121855	0.0200831	0.97545	0.99995	no

ENSG00000133961	ENSG00000133961	NUMB	14:73741814-73943880	q1	q2	OK	21.9548	22.022	0.00441197	0.0089167	0.9873	0.99995	no

ENSG00000133962	ENSG00000133962	CATSPERB	14:92047039-92333880	q1	q2	NOTEST	0	0	0	0	1	1	no

ENSG00000133980	ENSG00000133980	VRTN	14:74769771-74826711	q1	q2	NOTEST	0.00726547	0	#NAME?	0	1	1	no

ENSG00000133983	ENSG00000133983	COX16	14:70791797-70883778	q1	q2	OK	30.3745	32.0729	0.0784951	0.112996	0.86525	0.99995	no

ENSG00000133985	ENSG00000133985	TTC9	14:71108503-71142077	q1	q2	OK	4.53294	5.41721	0.257103	0.512687	0.4721	0.99995	no

ENSG00000133997	ENSG00000133997	MED6	14:71047973-71108015	q1	q2	OK	25.0261	29.0826	0.216725	0.41777	0.5564	0.99995	no

ENSG00000134001	ENSG00000134001	EIF2S1	14:67707825-67853233	q1	q2	OK	101.365	107.595	0.086041	0.0798066	0.92325	0.99995	no

ENSG00000134007	ENSG00000134007	ADAM20	14:70892528-71014181	q1	q2	NOTEST	0.0949152	0.0829829	-0.193824	0	1	1	no

ENSG00000134013	ENSG00000134013	LOXL2	8:23154701-23315208	q1	q2	OK	31.1245	28.2094	-0.141876	-0.152047	0.8094	0.99995	no

ENSG00000134014	ENSG00000134014	ELP3	8:27947189-28048673	q1	q2	OK	20.0216	18.7785	-0.0924745	-0.176413	0.78395	0.99995	no

ENSG00000134020	ENSG00000134020	PEBP4	8:22545171-22941132	q1	q2	NOTEST	0	0.0336388	inf	0	1	1	no

ENSG00000134028	ENSG00000134028	ADAMDEC1	8:24151552-24769586	q1	q2	NOTEST	0	0	0	0	1	1	no

ENSG00000134030	ENSG00000134030	CTIF	18:46065416-46389588	q1	q2	OK	11.6882	9.26792	-0.334733	-0.505766	0.3069	0.99995	no

ENSG00000134042	ENSG00000134042	MRO	18:48324573-48351772	q1	q2	NOTEST	0.208783	0.305234	0.547912	0	1	1	no

ENSG00000134046	ENSG00000134046	MBD2	18:51679078-51751158	q1	q2	OK	15.9925	19.6623	0.298039	0.398118	0.52625	0.99995	no

ENSG00000134049	ENSG00000134049	IER3IP1	18:44632316-44702745	q1	q2	OK	35.7157	39.4513	0.143515	0.223683	0.7489	0.99995	no

ENSG00000134056	ENSG00000134056	MRPS36	5:68513586-68525956	q1	q2	OK	10.6964	15.8779	0.569903	0.397421	0.5153	0.99995	no

ENSG00000134057	ENSG00000134057	CCNB1	5:68462836-68474072	q1	q2	OK	153.594	170.156	0.147739	0.318013	0.6288	0.99995	no

ENSG00000134058	ENSG00000134058	CDK7	5:68530667-68573250	q1	q2	OK	1.02398	0.551355	-0.893134	-0.620544	0.1447	0.99995	no

ENSG00000134061	ENSG00000134061	CD180	5:66478102-66492627	q1	q2	NOTEST	0	0	0	0	1	1	no

ENSG00000134070	ENSG00000134070	IRAK2	3:10206548-10285427	q1	q2	OK	0.41297	0.526744	0.351065	0.35508	0.6221	0.99995	no

ENSG00000134072	ENSG00000134072	CAMK1	3:9791627-9896822	q1	q2	OK	9.04324	8.4231	-0.102488	-0.0394445	0.9505	0.99995	no

ENSG00000134077	ENSG00000134077	THUMPD3	3:9022274-9520924	q1	q2	OK	32.9628	35.6643	0.11364	0.0530216	0.85905	0.99995	no

ENSG00000134086	ENSG00000134086	VHL	3:10182691-10193904	q1	q2	OK	45.6526	45.5843	-0.00216176	-0.00440001	0.9945	0.99995	no

ENSG00000134107	ENSG00000134107	BHLHE40	3:4938492-5027008	q1	q2	OK	2.61234	3.63103	0.475036	0.556414	0.30505	0.99995	no

ENSG00000134108	ENSG00000134108	ARL8B	3:5163904-5229014	q1	q2	OK	29.058	31.3093	0.107657	0.205462	0.73175	0.99995	no

ENSG00000134109	ENSG00000134109	EDEM1	3:5229226-5261642	q1	q2	OK	8.08683	9.00699	0.15547	0.191368	0.61785	0.99995	no

ENSG00000134115	ENSG00000134115	CNTN6	3:1134259-1445901	q1	q2	OK	6.69307	7.2656	0.118414	0.188824	0.7505	0.99995	no

ENSG00000134121	ENSG00000134121	CHL1	3:237440-451090	q1	q2	OK	7.18937	7.99337	0.152939	0.171022	0.63855	0.99995	no

ENSG00000134138	ENSG00000134138	MEIS2	15:37181405-37393504	q1	q2	OK	48.3855	45.8746	-0.0768786	-0.149298	0.78925	0.99995	no

ENSG00000134146	ENSG00000134146	DPH6	15:35473999-35838394	q1	q2	OK	6.99637	8.74116	0.321217	0.311492	0.65545	0.99995	no

ENSG00000134152	ENSG00000134152	KATNBL1	15:34432874-34502297	q1	q2	OK	39.381	42.5361	0.111185	0.200724	0.77315	0.99995	no

ENSG00000134153	ENSG00000134153	EMC7	15:34376217-34394149	q1	q2	OK	29.8388	28.2236	-0.0802861	-0.161555	0.8135	0.99995	no

ENSG00000134160	ENSG00000134160	TRPM1	15:31293263-31453476	q1	q2	NOTEST	0.0134867	0.0172726	0.356953	0	1	1	no

ENSG00000134183	ENSG00000134183	GNAT2	1:110145888-110155679	q1	q2	NOTEST	0.172433	0.0851549	-1.01787	0	1	1	no

ENSG00000134184	ENSG00000134184	GSTM1	1:110210643-110252171	q1	q2	OK	0.676507	0.368797	-0.875278	-0.229224	0.43815	0.99995	no

ENSG00000134186	ENSG00000134186	PRPF38B	1:109234944-109244425	q1	q2	OK	48.4233	51.7971	0.097171	0.204125	0.76325	0.99995	no

ENSG00000134193	ENSG00000134193	REG4	1:120336640-120354283	q1	q2	NOTEST	0.0576038	0	#NAME?	0	1	1	no

ENSG00000134198	ENSG00000134198	TSPAN2	1:115590631-115632121	q1	q2	OK	3.22352	3.28302	0.0263877	0.03383	0.9542	0.99995	no

ENSG00000134200	ENSG00000134200	TSHB	1:115572414-115576941	q1	q2	NOTEST	0.361399	0	#NAME?	0	1	1	no

ENSG00000134201	ENSG00000134201	GSTM5	1:110254863-110318050	q1	q2	NOTEST	0.00592037	0.0170213	1.52358	0	1	1	no

ENSG00000134202	ENSG00000134202	GSTM3	1:110254863-110318050	q1	q2	OK	30.4933	31.3354	0.0393014	0.0700609	0.9207	0.99995	no

ENSG00000134207	ENSG00000134207	SYT6	1:114631912-114696541	q1	q2	OK	3.84985	4.68217	0.282377	0.391075	0.505	0.99995	no

ENSG00000134215	ENSG00000134215	VAV3	1:108113781-108537229	q1	q2	OK	1.49467	1.76391	0.238955	0.258845	0.58595	0.99995	no

ENSG00000134216	ENSG00000134216	CHIA	1:111833483-111866697	q1	q2	NOTEST	0.0305424	0	#NAME?	0	1	1	no

ENSG00000134222	ENSG00000134222	PSRC1	1:109822177-109825808	q1	q2	OK	47.903	47.1563	-0.0226634	-0.0450751	0.94305	0.99995	no

ENSG00000134240	ENSG00000134240	HMGCS2	1:120290618-120311528	q1	q2	NOTEST	0	0	0	0	1	1	no

ENSG00000134242	ENSG00000134242	PTPN22	1:114304453-114456708	q1	q2	NOTEST	0.0576939	0.0450488	-0.356929	0	1	1	no

ENSG00000134243	ENSG00000134243	SORT1	1:109852191-109940573	q1	q2	OK	29.3361	30.4165	0.0521775	0.122292	0.86125	0.99995	no

ENSG00000134245	ENSG00000134245	WNT2B	1:113009162-113214241	q1	q2	OK	1.48498	1.55992	0.0710261	0.0328728	0.94315	0.99995	no

ENSG00000134247	ENSG00000134247	PTGFRN	1:117452678-117532980	q1	q2	OK	27.6177	27.3638	-0.0133255	-0.0133984	0.9758	0.99995	no

ENSG00000134248	ENSG00000134248	LAMTOR5	1:110905469-110974982	q1	q2	OK	101.367	109.1	0.106057	0.193029	0.78315	0.99995	no

ENSG00000134249	ENSG00000134249	ADAM30	1:120436155-120439118	q1	q2	NOTEST	0	0	0	0	1	1	no

ENSG00000134250	ENSG00000134250	NOTCH2	1:120454175-120612240	q1	q2	OK	11.6884	12.3862	0.0836521	0.183752	0.76525	0.99995	no

ENSG00000134253	ENSG00000134253	TRIM45	1:117653681-117665209	q1	q2	OK	5.89151	5.804	-0.0215919	-0.0335237	0.95755	0.99995	no

ENSG00000134255	ENSG00000134255	CEPT1	1:111659954-111727724	q1	q2	OK	15.4815	15.7619	0.0259014	0.0372354	0.9492	0.99995	no

ENSG00000134256	ENSG00000134256	CD101	1:117544381-117602112	q1	q2	OK	1.81089	1.5617	-0.213585	-0.144916	0.7468	0.99995	no

ENSG00000134258	ENSG00000134258	VTCN1	1:117686208-117753556	q1	q2	NOTEST	0.25543	0.190049	-0.426551	0	1	1	no

ENSG00000134259	ENSG00000134259	NGF	1:115825654-115910693	q1	q2	NOTEST	0.392262	0.247754	-0.662907	0	1	1	no

ENSG00000134262	ENSG00000134262	AP4B1	1:114304453-114456708	q1	q2	OK	13.5156	15.6202	0.208791	0.144687	0.7465	0.99995	no

ENSG00000134265	ENSG00000134265	NAPG	18:10525901-10552758	q1	q2	OK	13.3093	14.9537	0.168068	0.309893	0.5954	0.99995	no

ENSG00000134278	ENSG00000134278	SPIRE1	18:12438888-12725739	q1	q2	OK	20.8626	21.9984	0.0764774	0.103826	0.8346	0.99995	no

ENSG00000134283	ENSG00000134283	PPHLN1	12:42632248-42984157	q1	q2	OK	32.8811	36.2662	0.141369	0.202245	0.73285	0.99995	no

ENSG00000134285	ENSG00000134285	FKBP11	12:49297200-49351334	q1	q2	OK	16.0513	13.1975	-0.28243	-0.166397	0.62725	0.99995	no

ENSG00000134287	ENSG00000134287	ARF3	12:49297200-49351334	q1	q2	OK	67.4989	73.0625	0.114266	0.18262	0.7693	0.99995	no

ENSG00000134291	ENSG00000134291	TMEM106C	12:48357351-48362661	q1	q2	OK	81.6396	81.1948	-0.00788133	-0.0168902	0.9805	0.99995	no

ENSG00000134294	ENSG00000134294	SLC38A2	12:46751971-46767561	q1	q2	OK	98.7846	106.426	0.107491	0.173907	0.7016	0.99995	no

ENSG00000134297	ENSG00000134297	PLEKHA8P1	12:45566846-45834187	q1	q2	OK	6.55022	6.09117	-0.104823	-0.0287052	0.92835	0.99995	no

ENSG00000134308	ENSG00000134308	YWHAQ	2:9724100-9771143	q1	q2	OK	343.271	373.885	0.123246	0.267078	0.7002	0.99995	no

ENSG00000134313	ENSG00000134313	KIDINS220	2:8865407-8977760	q1	q2	OK	37.3787	38.447	0.0406511	0.0623723	0.8887	0.99995	no

ENSG00000134317	ENSG00000134317	GRHL1	2:10085340-10142411	q1	q2	OK	4.82225	3.58595	-0.42735	-0.623774	0.2982	0.99995	no

ENSG00000134318	ENSG00000134318	ROCK2	2:11319886-11488456	q1	q2	OK	21.9151	25.8631	0.238967	0.423575	0.38375	0.99995	no

ENSG00000134321	ENSG00000134321	RSAD2	2:6980700-7038370	q1	q2	OK	0.52077	0.687466	0.400643	0.154364	0.72055	0.99995	no

ENSG00000134323	ENSG00000134323	MYCN	2:16060520-16087129	q1	q2	OK	10.9729	10.606	-0.0490565	-0.0948732	0.896	0.99995	no

ENSG00000134324	ENSG00000134324	LPIN1	2:11817720-11967535	q1	q2	OK	11.9318	14.0891	0.239775	0.375388	0.44165	0.99995	no

ENSG00000134326	ENSG00000134326	CMPK2	2:6980700-7038370	q1	q2	OK	2.26704	2.56828	0.179994	0.19572	0.74405	0.99995	no

ENSG00000134330	ENSG00000134330	IAH1	2:9613786-9695921	q1	q2	OK	3.3104	2.42487	-0.449097	-0.152213	0.6301	0.99995	no

ENSG00000134333	ENSG00000134333	LDHA	11:18415934-18429972	q1	q2	OK	31.837	33.0908	0.0557283	0.126619	0.8552	0.99995	no

ENSG00000134339	ENSG00000134339	SAA2	11:18252895-18270190	q1	q2	NOTEST	0.122812	0.0948783	-0.372299	0	1	1	no

ENSG00000134343	ENSG00000134343	ANO3	11:26210669-26684835	q1	q2	NOTEST	0.0649812	0.0955826	0.556726	0	1	1	no

ENSG00000134352	ENSG00000134352	IL6ST	5:55230922-55290821	q1	q2	OK	16.2097	19.1576	0.241061	0.361097	0.44605	0.99995	no

ENSG00000134363	ENSG00000134363	FST	5:52776238-52782964	q1	q2	OK	21.2213	20.7222	-0.0343337	-0.0557685	0.93365	0.99995	no

ENSG00000134365	ENSG00000134365	CFHR4	1:196788886-196928356	q1	q2	NOTEST	0	0	0	0	1	1	no

ENSG00000134369	ENSG00000134369	NAV1	1:201592012-201853422	q1	q2	OK	9.57691	9.5214	-0.00838608	-0.00661429	0.9907	0.99995	no

ENSG00000134371	ENSG00000134371	CDC73	1:193091146-193223031	q1	q2	OK	14.0286	14.8992	0.0868654	0.188803	0.774	0.99995	no

ENSG00000134375	ENSG00000134375	TIMM17A	1:201924618-201939789	q1	q2	OK	49.1059	53.7779	0.131118	0.300084	0.6606	0.99995	no

ENSG00000134376	ENSG00000134376	CRB1	1:197170591-197447585	q1	q2	OK	2.59211	2.27888	-0.185797	-0.238889	0.7139	0.99995	no

ENSG00000134389	ENSG00000134389	CFHR5	1:196946666-196978804	q1	q2	NOTEST	0.0277371	0.0352218	0.344649	0	1	1	no

ENSG00000134398	ENSG00000134398	ERN2	16:23688976-23724821	q1	q2	NOTEST	0.0898499	0.0111546	-3.00988	0	1	1	no

ENSG00000134419	ENSG00000134419	RPS15A	16:18792616-18813000	q1	q2	OK	992.697	1003.76	0.01599	0.0204592	0.97715	0.99995	no

ENSG00000134438	ENSG00000134438	RAX	18:56934266-56941318	q1	q2	NOTEST	0.101025	0	#NAME?	0	1	1	no

ENSG00000134440	ENSG00000134440	NARS	18:55267887-55289445	q1	q2	OK	77.9523	82.0366	0.0736746	0.152426	0.8006	0.99995	no

ENSG00000134443	ENSG00000134443	GRP	18:56887399-56898006	q1	q2	OK	0.746346	0.358498	-1.05788	-0.577591	0.45835	0.99995	no

ENSG00000134444	ENSG00000134444	KIAA1468	18:59854490-59974355	q1	q2	OK	10.5458	11.725	0.152922	0.193458	0.69815	0.99995	no

ENSG00000134452	ENSG00000134452	FBXO18	10:5903579-5987863	q1	q2	OK	28.554	27.329	-0.0632636	-0.0838509	0.85515	0.99995	no

ENSG00000134453	ENSG00000134453	RBM17	10:6130949-6159420	q1	q2	OK	88.9498	87.6831	-0.0206931	-0.0367455	0.9567	0.99995	no

ENSG00000134460	ENSG00000134460	IL2RA	10:6052651-6104288	q1	q2	NOTEST	0	0	0	0	1	1	no

ENSG00000134461	ENSG00000134461	ANKRD16	10:5903579-5987863	q1	q2	OK	3.56183	3.81763	0.100059	0.0605992	0.9358	0.99995	no

ENSG00000134463	ENSG00000134463	ECHDC3	10:11784364-11806069	q1	q2	NOTEST	0.0163753	0.110615	2.75596	0	1	1	no

ENSG00000134470	ENSG00000134470	IL15RA	10:5990854-6020150	q1	q2	NOTEST	0	0	0	0	1	1	no

ENSG00000134480	ENSG00000134480	CCNH	5:86563704-86708836	q1	q2	OK	18.445	18.2602	-0.0145307	-0.0148152	0.9827	0.99995	no

ENSG00000134489	ENSG00000134489	HRH4	18:22040592-22059921	q1	q2	NOTEST	0.0483098	0.00711739	-2.7629	0	1	1	no

ENSG00000134490	ENSG00000134490	TMEM241	18:20714527-21017925	q1	q2	OK	2.22487	2.56446	0.204934	0.163747	0.72335	0.99995	no

ENSG00000134504	ENSG00000134504	KCTD1	18:24034873-24770662	q1	q2	OK	8.99811	9.36493	0.0576463	0.0858325	0.89555	0.99995	no

ENSG00000134508	ENSG00000134508	CABLES1	18:20714527-21017925	q1	q2	OK	3.86151	3.12321	-0.306134	-0.282689	0.54255	0.99995	no

ENSG00000134516	ENSG00000134516	DOCK2	5:169064250-169510386	q1	q2	NOTEST	0.169211	0.114288	-0.566156	0	1	1	no

ENSG00000134531	ENSG00000134531	EMP1	12:13349649-13369708	q1	q2	OK	15.8057	18.0042	0.187886	0.357938	0.59145	0.99995	no

ENSG00000134532	ENSG00000134532	SOX5	12:23682439-24103966	q1	q2	OK	7.85994	7.09492	-0.147732	-0.186611	0.6725	0.99995	no

ENSG00000134533	ENSG00000134533	RERG	12:15260716-15750333	q1	q2	NOTEST	0.205072	0.202451	-0.0185538	0	1	1	no

ENSG00000134538	ENSG00000134538	SLCO1B1	12:20963635-21392180	q1	q2	NOTEST	0	0	0	0	1	1	no

ENSG00000134539	ENSG00000134539	KLRD1	12:10378656-10469850	q1	q2	NOTEST	0.0313725	0	#NAME?	0	1	1	no

ENSG00000134545	ENSG00000134545	KLRC1	12:10564910-10607284	q1	q2	NOTEST	0	0.0712134	inf	0	1	1	no

ENSG00000134548	ENSG00000134548	C12orf39	12:21679240-21757781	q1	q2	OK	6.98176	9.10588	0.383207	0.622358	0.36225	0.99995	no

ENSG00000134551	ENSG00000134551	PRH2	12:10977558-11324212	q1	q2	NOTEST	0	0	0	0	1	1	no

ENSG00000134569	ENSG00000134569	LRP4	11:46867962-46940193	q1	q2	OK	15.6833	15.8737	0.0174123	0.0299489	0.95845	0.99995	no

ENSG00000134571	ENSG00000134571	MYBPC3	11:47352956-47374253	q1	q2	NOTEST	0.0118172	0	#NAME?	0	1	1	no

ENSG00000134574	ENSG00000134574	DDB2	11:47236492-47260767	q1	q2	OK	6.25157	5.26133	-0.248792	-0.368871	0.6012	0.99995	no

ENSG00000134575	ENSG00000134575	ACP2	11:47260852-47290396	q1	q2	OK	21.9795	19.4112	-0.179265	-0.326343	0.60525	0.99995	no

ENSG00000134588	ENSG00000134588	USP26	X:132158658-132231137	q1	q2	NOTEST	0	0.0157093	inf	0	1	1	no

ENSG00000134590	ENSG00000134590	FAM127A	X:134166332-134167576	q1	q2	OK	86.2017	78.8248	-0.129068	-0.306219	0.64905	0.99995	no

ENSG00000134594	ENSG00000134594	RAB33A	X:129305622-129318844	q1	q2	OK	5.84243	5.92585	0.0204545	0.0291024	0.96615	0.99995	no

ENSG00000134595	ENSG00000134595	SOX3	X:139585151-139587225	q1	q2	OK	107.83	81.2942	-0.407532	-0.982285	0.15745	0.99995	no

ENSG00000134597	ENSG00000134597	RBMX2	X:129535942-129547317	q1	q2	OK	19.8251	20.4456	0.0444612	0.0730179	0.90645	0.99995	no

ENSG00000134602	ENSG00000134602	MST4	X:131157292-131209971	q1	q2	OK	13.0819	14.4343	0.141935	0.307402	0.65615	0.99995	no

ENSG00000134612	ENSG00000134612	FOLH1B	11:89371682-89431886	q1	q2	OK	3.12724	2.58771	-0.273217	-0.323807	0.58515	0.99995	no

ENSG00000134627	ENSG00000134627	PIWIL4	11:94277005-94658686	q1	q2	OK	0.849638	0.617876	-0.45953	-0.0569406	0.69515	0.99995	no

ENSG00000134640	ENSG00000134640	MTNR1B	11:92702885-92718232	q1	q2	NOTEST	0	0	0	0	1	1	no

ENSG00000134644	ENSG00000134644	PUM1	1:31404352-31538838	q1	q2	OK	44.3054	41.6562	-0.0889507	-0.163434	0.7578	0.99995	no

ENSG00000134668	ENSG00000134668	SPOCD1	1:32254730-32281652	q1	q2	NOTEST	0.0136521	0	#NAME?	0	1	1	no

ENSG00000134684	ENSG00000134684	YARS	1:33240839-33324476	q1	q2	OK	60.2804	61.1352	0.0203155	0.0348579	0.9603	0.99995	no

ENSG00000134686	ENSG00000134686	PHC2	1:33772366-33896653	q1	q2	OK	106.98	100.43	-0.0911524	-0.183596	0.75225	0.99995	no

ENSG00000134690	ENSG00000134690	CDCA8	1:38158089-38175391	q1	q2	OK	33.2054	35.6435	0.102223	0.243004	0.7304	0.99995	no

ENSG00000134697	ENSG00000134697	GNL2	1:38022519-38061536	q1	q2	OK	38.4223	40.6487	0.0812663	0.124297	0.81065	0.99995	no

ENSG00000134698	ENSG00000134698	AGO4	1:36273772-36323491	q1	q2	OK	10.0522	10.2587	0.0293367	0.0388863	0.92315	0.99995	no

ENSG00000134709	ENSG00000134709	HOOK1	1:60280457-60342050	q1	q2	OK	0.538366	0.644446	0.259471	0.170322	0.66895	0.99995	no

ENSG00000134716	ENSG00000134716	CYP2J2	1:60358979-60392462	q1	q2	NOTEST	0	0	0	0	1	1	no

ENSG00000134717	ENSG00000134717	BTF3L4	1:52485802-52556388	q1	q2	OK	64.7065	69.1001	0.0947778	0.145521	0.83105	0.99995	no

ENSG00000134744	ENSG00000134744	ZCCHC11	1:52870235-53019159	q1	q2	OK	54.7437	53.483	-0.0336136	-0.0363138	0.94665	0.99995	no

ENSG00000134748	ENSG00000134748	PRPF38A	1:52870235-53019159	q1	q2	OK	29.5985	30.0873	0.0236298	0.0265641	0.96505	0.99995	no

ENSG00000134755	ENSG00000134755	DSC2	18:28645939-28742819	q1	q2	OK	4.20922	7.14505	0.763392	1.52093	0.0335	0.99995	no

ENSG00000134757	ENSG00000134757	DSG3	18:29027757-29058665	q1	q2	NOTEST	0	0.00862023	inf	0	1	1	no

ENSG00000134758	ENSG00000134758	RNF138	18:29598334-30050447	q1	q2	OK	29.9672	33.4025	0.156572	0.182895	0.735	0.99995	no

ENSG00000134759	ENSG00000134759	ELP2	18:33709406-33757909	q1	q2	OK	28.291	28.4031	0.00570624	0.00807674	0.98795	0.99995	no

ENSG00000134760	ENSG00000134760	DSG1	18:28898051-29006951	q1	q2	NOTEST	0	0	0	0	1	1	no

ENSG00000134762	ENSG00000134762	DSC3	18:28569973-28622781	q1	q2	NOTEST	0.112763	0.244637	1.11735	0	1	1	no

ENSG00000134765	ENSG00000134765	DSC1	18:28645939-28742819	q1	q2	NOTEST	0.0179931	0.028949	0.686068	0	1	1	no

ENSG00000134769	ENSG00000134769	DTNA	18:32073253-32471808	q1	q2	OK	21.4554	19.0889	-0.168609	-0.271737	0.6652	0.99995	no

ENSG00000134775	ENSG00000134775	FHOD3	18:33877676-34812135	q1	q2	OK	15.1598	15.0287	-0.0125235	-0.0114366	0.9872	0.99995	no

ENSG00000134779	ENSG00000134779	TPGS2	18:33877676-34812135	q1	q2	OK	119.298	121.238	0.0232799	0.0422509	0.95025	0.99995	no

ENSG00000134780	ENSG00000134780	DAGLA	11:61447904-61634826	q1	q2	OK	1.62507	1.63523	0.00899349	0.00157384	0.99545	0.99995	no

ENSG00000134802	ENSG00000134802	SLC43A3	11:57154266-57195053	q1	q2	OK	4.68624	4.0231	-0.220121	-0.224305	0.6067	0.99995	no

ENSG00000134809	ENSG00000134809	TIMM10	11:57295935-57298276	q1	q2	OK	39.2467	40.1012	0.0310767	0.0575341	0.9362	0.99995	no

ENSG00000134812	ENSG00000134812	GIF	11:59596740-59612974	q1	q2	NOTEST	0	0	0	0	1	1	no

ENSG00000134815	ENSG00000134815	DHX34	19:47852537-47885961	q1	q2	OK	8.45119	7.58906	-0.155233	-0.234306	0.6858	0.99995	no

ENSG00000134817	ENSG00000134817	APLNR	11:57001050-57004709	q1	q2	OK	4.76411	2.6612	-0.840131	-1.28149	0.082	0.99995	no

ENSG00000134824	ENSG00000134824	FADS2	11:61447904-61634826	q1	q2	OK	290.899	309.073	0.0874303	0.092994	0.86115	0.99995	no

ENSG00000134825	ENSG00000134825	TMEM258	11:61447904-61634826	q1	q2	OK	101.562	107.545	0.0825865	0.0291006	0.96655	0.99995	no

ENSG00000134827	ENSG00000134827	TCN1	11:59620272-59634048	q1	q2	NOTEST	0	0.0853502	inf	0	1	1	no

ENSG00000134830	ENSG00000134830	C5AR2	19:47835403-47846606	q1	q2	NOTEST	0	0	0	0	1	1	no

ENSG00000134851	ENSG00000134851	TMEM165	4:56262123-56414056	q1	q2	OK	41.1377	44.9412	0.127577	0.19587	0.7385	0.99995	no

ENSG00000134852	ENSG00000134852	CLOCK	4:56262123-56414056	q1	q2	OK	5.39291	7.22024	0.420982	0.272055	0.3588	0.99995	no

ENSG00000134853	ENSG00000134853	PDGFRA	4:54243809-55164414	q1	q2	OK	9.04347	10.6676	0.238284	0.196853	0.7985	0.99995	no

ENSG00000134864	ENSG00000134864	GGACT	13:101183809-101241782	q1	q2	OK	2.30115	1.84454	-0.319093	-0.182148	0.79535	0.99995	no

ENSG00000134871	ENSG00000134871	COL4A2	13:110801317-111165374	q1	q2	OK	89.5748	84.2416	-0.0885603	-0.11394	0.8297	0.99995	no

ENSG00000134873	ENSG00000134873	CLDN10	13:96085857-96296957	q1	q2	OK	0.627349	0.413641	-0.600887	-0.114654	0.68585	0.99995	no

ENSG00000134874	ENSG00000134874	DZIP1	13:96085857-96296957	q1	q2	OK	27.1554	26.0067	-0.0623593	-0.104698	0.8631	0.99995	no

ENSG00000134882	ENSG00000134882	UBAC2	13:99853027-100038688	q1	q2	OK	49.7875	46.7699	-0.0902012	-0.175564	0.7981	0.99995	no

ENSG00000134884	ENSG00000134884	ARGLU1	13:107194020-107220512	q1	q2	OK	277.364	322.89	0.219264	0.309731	0.61865	0.99995	no

ENSG00000134897	ENSG00000134897	BIVM	13:103451398-103528345	q1	q2	OK	15.4214	17.0455	0.144457	0.165767	0.75175	0.99995	no

ENSG00000134899	ENSG00000134899	ERCC5	13:103451398-103528345	q1	q2	OK	22.0986	22.5749	0.0307648	0.0277898	0.9661	0.99995	no

ENSG00000134900	ENSG00000134900	TPP2	13:103249352-103331521	q1	q2	OK	28.8873	28.1686	-0.0363435	-0.0574384	0.92915	0.99995	no

ENSG00000134901	ENSG00000134901	KDELC1	13:103436630-103451357	q1	q2	OK	15.7576	15.6777	-0.00733273	-0.0139181	0.98275	0.99995	no

ENSG00000134905	ENSG00000134905	CARS2	13:111293758-111373421	q1	q2	OK	29.6464	30.6294	0.0470635	0.0607513	0.9074	0.99995	no

ENSG00000134909	ENSG00000134909	ARHGAP32	11:128834954-129149219	q1	q2	OK	8.80429	9.48228	0.107029	0.195877	0.71595	0.99995	no

ENSG00000134910	ENSG00000134910	STT3A	11:125439111-125551018	q1	q2	OK	75.62	78.7214	0.057987	0.0781574	0.9071	0.99995	no

ENSG00000134917	ENSG00000134917	ADAMTS8	11:130274819-130298888	q1	q2	OK	1.11781	1.64682	0.559007	0.660269	0.34965	0.99995	no

ENSG00000134940	ENSG00000134940	ACRV1	11:125439111-125551018	q1	q2	OK	0.631432	0.663544	0.071565	0.00809482	0.95665	0.99995	no

ENSG00000134954	ENSG00000134954	ETS1	11:128328655-128457453	q1	q2	OK	24.9747	28.0779	0.168966	0.362651	0.57135	0.99995	no

ENSG00000134955	ENSG00000134955	SLC37A2	11:124932962-124960411	q1	q2	NOTEST	0.324269	0.213071	-0.605859	0	1	1	no

ENSG00000134962	ENSG00000134962	KLB	4:39408472-39453156	q1	q2	NOTEST	0.0907044	0.113392	0.322078	0	1	1	no

ENSG00000134970	ENSG00000134970	TMED7	5:114914338-114968689	q1	q2	OK	29.0348	30.7604	0.0832916	0.186455	0.77375	0.99995	no

ENSG00000134982	ENSG00000134982	APC	5:112043194-112258236	q1	q2	OK	14.1934	16.5354	0.220337	0.228473	0.60905	0.99995	no

ENSG00000134986	ENSG00000134986	NREP	5:110831730-111353006	q1	q2	OK	400.134	427.598	0.0957731	0.168069	0.7903	0.99995	no

ENSG00000134987	ENSG00000134987	WDR36	5:110405759-110466200	q1	q2	OK	10.1944	11.4224	0.164091	0.272679	0.57915	0.99995	no

ENSG00000134996	ENSG00000134996	OSTF1	9:77703458-77762181	q1	q2	OK	3.24215	4.02321	0.311397	0.384153	0.5807	0.99995	no

ENSG00000135002	ENSG00000135002	RFK	9:79000432-79009433	q1	q2	OK	11.3785	13.1278	0.206314	0.408654	0.5563	0.99995	no

ENSG00000135018	ENSG00000135018	UBQLN1	9:86274877-86328293	q1	q2	OK	59.1835	63.239	0.09562	0.19401	0.7485	0.99995	no

ENSG00000135040	ENSG00000135040	NAA35	9:88556060-88637213	q1	q2	OK	16.9629	18.1632	0.0986399	0.216253	0.76855	0.99995	no

ENSG00000135045	ENSG00000135045	C9orf40	9:77561496-77567802	q1	q2	OK	10.1399	9.83791	-0.0436205	-0.0833971	0.9073	0.99995	no

ENSG00000135046	ENSG00000135046	ANXA1	9:75766672-75785309	q1	q2	OK	37.8614	37.8832	0.000830074	0.00162867	0.9981	0.99995	no

ENSG00000135047	ENSG00000135047	CTSL	9:90340433-90346308	q1	q2	OK	30.1104	28.687	-0.0698637	-0.138201	0.84415	0.99995	no

ENSG00000135048	ENSG00000135048	TMEM2	9:74298281-74431606	q1	q2	OK	103.67	106.517	0.0390854	0.0703733	0.90985	0.99995	no

ENSG00000135049	ENSG00000135049	AGTPBP1	9:88161454-88356944	q1	q2	OK	16.1947	17.7999	0.136348	0.246363	0.6648	0.99995	no

ENSG00000135052	ENSG00000135052	GOLM1	9:88641060-88715088	q1	q2	OK	100.007	100.287	0.00402905	0.00788438	0.99065	0.99995	no

ENSG00000135063	ENSG00000135063	FAM189A2	9:71939487-72007371	q1	q2	NOTEST	0.427598	0.467755	0.1295	0	1	1	no

ENSG00000135069	ENSG00000135069	PSAT1	9:80912058-80945009	q1	q2	OK	121.127	130.279	0.105082	0.258207	0.70805	0.99995	no

ENSG00000135070	ENSG00000135070	ISCA1	9:88879460-88897676	q1	q2	OK	29.9478	29.5906	-0.0173104	-0.0384602	0.95605	0.99995	no

ENSG00000135074	ENSG00000135074	ADAM19	5:156687131-157002783	q1	q2	OK	11.4456	11.7091	0.0328425	0.03348	0.96395	0.99995	no

ENSG00000135077	ENSG00000135077	HAVCR2	5:156512842-156682201	q1	q2	NOTEST	0.373024	0.452344	0.278152	0	1	1	no

ENSG00000135083	ENSG00000135083	CCNJL	5:159678658-159772403	q1	q2	OK	16.332	17.0481	0.0619084	0.115237	0.8613	0.99995	no

ENSG00000135090	ENSG00000135090	TAOK3	12:118587605-118810750	q1	q2	OK	13.9228	13.7068	-0.0225623	-0.0185089	0.9712	0.99995	no

ENSG00000135093	ENSG00000135093	USP30	12:109460893-109525831	q1	q2	OK	8.94459	8.77261	-0.0280097	-0.0463618	0.93465	0.99995	no

ENSG00000135094	ENSG00000135094	SDS	12:113830249-113876081	q1	q2	NOTEST	0.0612586	0.0224115	-1.45067	0	1	1	no

ENSG00000135097	ENSG00000135097	MSI1	12:120779132-120806983	q1	q2	OK	46.5009	43.3895	-0.0999117	-0.171709	0.80335	0.99995	no

ENSG00000135100	ENSG00000135100	HNF1A	12:121406415-121454305	q1	q2	OK	0.695188	0.651166	-0.0943769	-0.039863	0.931	0.99995	no

ENSG00000135108	ENSG00000135108	FBXO21	12:117581145-117628336	q1	q2	OK	26.3712	25.3386	-0.0576287	-0.133047	0.8478	0.99995	no

ENSG00000135111	ENSG00000135111	TBX3	12:115108058-115121969	q1	q2	NOTEST	0.0308809	0.0171881	-0.845308	0	1	1	no

ENSG00000135114	ENSG00000135114	OASL	12:121458094-121477045	q1	q2	NOTEST	0	0	0	0	1	1	no

ENSG00000135116	ENSG00000135116	HRK	12:117293948-117319246	q1	q2	OK	1.56019	1.47692	-0.0791269	-0.105872	0.86005	0.99995	no

ENSG00000135119	ENSG00000135119	RNFT2	12:117176095-117291436	q1	q2	OK	12.829	12.607	-0.0251758	-0.041409	0.9502	0.99995	no

ENSG00000135124	ENSG00000135124	P2RX4	12:121647659-121671909	q1	q2	OK	4.65477	4.19092	-0.151442	-0.193188	0.75545	0.99995	no

ENSG00000135127	ENSG00000135127	CCDC64	12:120427672-120532298	q1	q2	OK	4.81963	4.14071	-0.219044	-0.207498	0.69605	0.99995	no

ENSG00000135144	ENSG00000135144	DTX1	12:113494513-113535833	q1	q2	OK	9.62055	9.04456	-0.0890692	-0.136906	0.83315	0.99995	no

ENSG00000135148	ENSG00000135148	TRAFD1	12:112563304-112591407	q1	q2	OK	26.7692	27.6731	0.0479113	0.0994228	0.873	0.99995	no

ENSG00000135164	ENSG00000135164	DMTF1	7:86780738-86849903	q1	q2	OK	93.383	94.3972	0.015584	0.0249145	0.96095	0.99995	no

ENSG00000135175	ENSG00000135175	OCM2	7:97613995-97620481	q1	q2	NOTEST	0	0	0	0	1	1	no

ENSG00000135185	ENSG00000135185	TMEM243	7:86780738-86849903	q1	q2	OK	13.6231	13.2226	-0.0430539	-0.0236322	0.9716	0.99995	no

ENSG00000135205	ENSG00000135205	CCDC146	7:76139744-77045717	q1	q2	OK	4.5794	4.94142	0.109766	0.102003	0.8529	0.99995	no

ENSG00000135211	ENSG00000135211	TMEM60	7:77423044-77427897	q1	q2	OK	21.0394	22.2558	0.0810835	0.148722	0.83265	0.99995	no

ENSG00000135213	ENSG00000135213	POM121C	7:74988447-75115548	q1	q2	OK	2.76486	4.05124	0.551161	0.326659	0.4342	0.99995	no

ENSG00000135218	ENSG00000135218	CD36	7:79959507-80308593	q1	q2	NOTEST	0.0672547	0.222607	1.72679	0	1	1	no

ENSG00000135220	ENSG00000135220	UGT2A3	4:69794180-69817509	q1	q2	NOTEST	0	0	0	0	1	1	no

ENSG00000135222	ENSG00000135222	CSN2	4:70820973-70826725	q1	q2	NOTEST	0	0	0	0	1	1	no

ENSG00000135226	ENSG00000135226	UGT2B28	4:70146216-70160768	q1	q2	NOTEST	0	0	0	0	1	1	no

ENSG00000135241	ENSG00000135241	PNPLA8	7:108110865-108215294	q1	q2	OK	13.4259	14.0781	0.0684403	0.092248	0.8941	0.99995	no

ENSG00000135245	ENSG00000135245	HILPDA	7:128095893-128146656	q1	q2	OK	22.1152	22.4816	0.0237099	0.0320141	0.96295	0.99995	no

ENSG00000135248	ENSG00000135248	FAM71F1	7:128349114-128371797	q1	q2	NOTEST	0.113326	0.160266	0.49999	0	1	1	no

ENSG00000135249	ENSG00000135249	RINT1	7:105172531-105241322	q1	q2	OK	11.154	13.8417	0.311458	0.416668	0.4035	0.99995	no

ENSG00000135250	ENSG00000135250	SRPK2	7:104653871-105039755	q1	q2	OK	32.1599	32.7204	0.0249278	0.0238211	0.9562	0.99995	no

ENSG00000135253	ENSG00000135253	KCP	7:128470430-128550773	q1	q2	NOTEST	0.118305	0.0224128	-2.40012	0	1	1	no

ENSG00000135269	ENSG00000135269	TES	7:115850546-116148595	q1	q2	OK	28.1844	36.4981	0.372927	0.723648	0.2124	0.99995	no

ENSG00000135272	ENSG00000135272	MDFIC	7:114562208-114659256	q1	q2	OK	6.41742	7.29064	0.184052	0.292485	0.57985	0.99995	no

ENSG00000135297	ENSG00000135297	MTO1	6:74171300-74218959	q1	q2	OK	10.3751	11.5817	0.158729	0.198603	0.7002	0.99995	no

ENSG00000135298	ENSG00000135298	BAI3	6:69345258-70099403	q1	q2	OK	3.55639	3.11663	-0.190425	-0.205771	0.71025	0.99995	no

ENSG00000135299	ENSG00000135299	ANKRD6	6:90142888-90348472	q1	q2	OK	19.103	16.8305	-0.182725	-0.158955	0.7098	0.99995	no

ENSG00000135312	ENSG00000135312	HTR1B	6:78171947-78173490	q1	q2	NOTEST	0.0539805	0.0710491	0.396377	0	1	1	no

ENSG00000135314	ENSG00000135314	KHDC1	6:73918805-74020088	q1	q2	OK	0.790335	0.539996	-0.549515	-0.280873	0.73165	0.99995	no

ENSG00000135315	ENSG00000135315	KIAA1009	6:84833959-84937353	q1	q2	OK	9.67705	11.1106	0.199299	0.3176	0.62585	0.99995	no

ENSG00000135316	ENSG00000135316	SYNCRIP	6:86215213-86353510	q1	q2	OK	84.4874	96.456	0.191135	0.360482	0.5722	0.99995	no

ENSG00000135317	ENSG00000135317	SNX14	6:86215213-86353510	q1	q2	OK	43.7172	47.8513	0.130357	0.0660294	0.84825	0.99995	no

ENSG00000135318	ENSG00000135318	NT5E	6:86159808-86205500	q1	q2	OK	7.64225	8.20128	0.101851	0.173277	0.78515	0.99995	no

ENSG00000135324	ENSG00000135324	MRAP2	6:84743474-84800600	q1	q2	NOTEST	0.364944	0.352746	-0.0490463	0	1	1	no

ENSG00000135333	ENSG00000135333	EPHA7	6:93949737-94129265	q1	q2	OK	6.171	6.56715	0.0897641	0.170146	0.7961	0.99995	no

ENSG00000135334	ENSG00000135334	AKIRIN2	6:88384789-88411927	q1	q2	OK	56.0767	55.9053	-0.00441681	-0.00815412	0.9885	0.99995	no

ENSG00000135336	ENSG00000135336	ORC3	6:88299838-88377169	q1	q2	OK	20.4281	21.6485	0.083714	0.153888	0.785	0.99995	no

ENSG00000135338	ENSG00000135338	LCA5	6:80194707-80247175	q1	q2	OK	2.42682	2.65882	0.131723	0.215993	0.7529	0.99995	no

ENSG00000135341	ENSG00000135341	MAP3K7	6:91223291-91296764	q1	q2	OK	30.105	32.5052	0.110664	0.249877	0.7225	0.99995	no

ENSG00000135346	ENSG00000135346	CGA	6:87795215-87804824	q1	q2	NOTEST	0	0	0	0	1	1	no

ENSG00000135355	ENSG00000135355	GJA10	6:90604187-90605839	q1	q2	NOTEST	0	0	0	0	1	1	no

ENSG00000135362	ENSG00000135362	PRR5L	11:36317837-36486754	q1	q2	OK	9.97025	12.044	0.272607	0.308082	0.5329	0.99995	no

ENSG00000135363	ENSG00000135363	LMO2	11:33880121-33913836	q1	q2	OK	4.32546	7.08799	0.712523	0.900077	0.18385	0.99995	no

ENSG00000135365	ENSG00000135365	PHF21A	11:45950870-46142985	q1	q2	OK	31.4971	31.7939	0.0135309	0.0177759	0.96665	0.99995	no

ENSG00000135372	ENSG00000135372	NAT10	11:34127148-34169217	q1	q2	OK	17.2573	17.4665	0.0173878	0.0287802	0.9517	0.99995	no

ENSG00000135373	ENSG00000135373	EHF	11:34642639-34682604	q1	q2	NOTEST	0.0312315	0.00836569	-1.90045	0	1	1	no

ENSG00000135374	ENSG00000135374	ELF5	11:34500339-34535352	q1	q2	NOTEST	0	0.0116283	inf	0	1	1	no

ENSG00000135378	ENSG00000135378	PRRG4	11:32851488-32879669	q1	q2	NOTEST	0.0138352	0.0351016	1.34319	0	1	1	no

ENSG00000135387	ENSG00000135387	CAPRIN1	11:34073229-34122703	q1	q2	OK	165.491	175.982	0.0886826	0.18263	0.77785	0.99995	no

ENSG00000135390	ENSG00000135390	ATP5G2	12:54026509-54071192	q1	q2	OK	323.058	281.295	-0.199709	-0.492978	0.4775	0.99995	no

ENSG00000135392	ENSG00000135392	DNAJC14	12:56137063-56236750	q1	q2	OK	18.3276	17.9246	-0.0320771	-0.0313935	0.95775	0.99995	no

ENSG00000135404	ENSG00000135404	CD63	12:56119106-56124467	q1	q2	OK	388.831	430.659	0.147403	0.332715	0.6351	0.99995	no

ENSG00000135406	ENSG00000135406	PRPH	12:49686413-49725514	q1	q2	OK	1.09442	1.21869	0.155166	0.0435264	0.90345	0.99995	no

ENSG00000135407	ENSG00000135407	AVIL	12:58156116-58212487	q1	q2	OK	2.35374	2.45861	0.0628865	0.0318143	0.9453	0.99995	no

ENSG00000135409	ENSG00000135409	AMHR2	12:53817638-53825318	q1	q2	NOTEST	0.100156	0.104156	0.0565012	0	1	1	no

ENSG00000135413	ENSG00000135413	LACRT	12:55024594-55028679	q1	q2	NOTEST	0	0	0	0	1	1	no

ENSG00000135414	ENSG00000135414	GDF11	12:56137063-56236750	q1	q2	OK	29.3188	29.8646	0.0266123	0.0371071	0.95765	0.99995	no

ENSG00000135423	ENSG00000135423	GLS2	12:56843285-56882198	q1	q2	OK	1.24827	1.34379	0.106373	0.0539436	0.91085	0.99995	no

ENSG00000135424	ENSG00000135424	ITGA7	12:56075329-56118489	q1	q2	OK	36.4599	37.3862	0.0361966	0.0460915	0.92905	0.99995	no

ENSG00000135426	ENSG00000135426	TESPA1	12:55341801-55378530	q1	q2	NOTEST	0.0699499	0	#NAME?	0	1	1	no

ENSG00000135436	ENSG00000135436	FAM186B	12:49962000-50158717	q1	q2	NOTEST	0.406438	0.447527	0.138939	0	1	1	no

ENSG00000135437	ENSG00000135437	RDH5	12:56075329-56118489	q1	q2	OK	4.67833	5.32665	0.187233	0.0626181	0.85435	0.99995	no

ENSG00000135439	ENSG00000135439	AGAP2	12:58117543-58154190	q1	q2	OK	15.0295	14.2528	-0.0765584	-0.0718516	0.9134	0.99995	no

ENSG00000135441	ENSG00000135441	BLOC1S1	12:56075329-56118489	q1	q2	OK	91.2811	85.4965	-0.0944509	-0.113321	0.8756	0.99995	no

ENSG00000135443	ENSG00000135443	KRT85	12:52753789-52761265	q1	q2	NOTEST	0	0	0	0	1	1	no

ENSG00000135446	ENSG00000135446	CDK4	12:58117543-58154190	q1	q2	OK	174.016	165.607	-0.071454	-0.106849	0.868	0.99995	no

ENSG00000135447	ENSG00000135447	PPP1R1A	12:54943133-54982443	q1	q2	OK	3.16933	2.9142	-0.12108	-0.127308	0.8417	0.99995	no

ENSG00000135451	ENSG00000135451	TROAP	12:49686413-49725514	q1	q2	OK	35.6705	36.4615	0.0316434	0.0552587	0.93015	0.99995	no

ENSG00000135452	ENSG00000135452	TSPAN31	12:58117543-58154190	q1	q2	OK	19.6952	20.1726	0.0345501	0.0209758	0.9685	0.99995	no

ENSG00000135454	ENSG00000135454	B4GALNT1	12:58003962-58027138	q1	q2	OK	25.8425	22.6347	-0.19121	-0.222493	0.6556	0.99995	no

ENSG00000135457	ENSG00000135457	TFCP2	12:51487445-51566926	q1	q2	OK	22.5945	22.2663	-0.0211051	-0.0470507	0.9437	0.99995	no

ENSG00000135469	ENSG00000135469	COQ10A	12:56660641-56664750	q1	q2	OK	15.1295	14.8423	-0.0276489	-0.0468002	0.94875	0.99995	no

ENSG00000135472	ENSG00000135472	FAIM2	12:50260678-50298000	q1	q2	OK	1.32238	0.965298	-0.454095	-0.364304	0.5428	0.99995	no

ENSG00000135473	ENSG00000135473	PAN2	12:56665482-56727837	q1	q2	OK	28.9649	27.2807	-0.0864215	-0.044393	0.88345	0.99995	no

ENSG00000135476	ENSG00000135476	ESPL1	12:53662082-53687427	q1	q2	OK	17.7928	17.0525	-0.0613065	-0.0893961	0.84895	0.99995	no

ENSG00000135477	ENSG00000135477	KRT121P	12:52626303-52702947	q1	q2	NOTEST	0	0	0	0	1	1	no

ENSG00000135480	ENSG00000135480	KRT7	12:52626303-52702947	q1	q2	NOTEST	0.0377706	0.134551	1.83281	0	1	1	no

ENSG00000135482	ENSG00000135482	ZC3H10	12:56511942-56538455	q1	q2	OK	7.69961	6.86203	-0.166149	-0.125073	0.83585	0.99995	no

ENSG00000135486	ENSG00000135486	HNRNPA1	12:54624723-54745633	q1	q2	OK	1914.77	1885.18	-0.022476	-0.0233514	0.97435	0.99995	no

ENSG00000135502	ENSG00000135502	SLC26A10	12:58003962-58027138	q1	q2	OK	13.2532	11.5649	-0.196592	-0.125278	0.8166	0.99995	no

ENSG00000135503	ENSG00000135503	ACVR1B	12:52345450-52390862	q1	q2	OK	14.739	13.9641	-0.0779245	-0.128281	0.799	0.99995	no

ENSG00000135506	ENSG00000135506	OS9	12:58087737-58115340	q1	q2	OK	78.9588	70.8592	-0.156145	-0.321555	0.6109	0.99995	no

ENSG00000135517	ENSG00000135517	MIP	12:56843285-56882198	q1	q2	NOTEST	0.0825937	0.0880622	0.0924896	0	1	1	no

ENSG00000135519	ENSG00000135519	KCNH3	12:49932939-49961936	q1	q2	NOTEST	0.356786	0.214372	-0.734944	0	1	1	no

ENSG00000135521	ENSG00000135521	LTV1	6:143857981-144184949	q1	q2	OK	22.6423	25.3069	0.160508	0.28416	0.6845	0.99995	no

ENSG00000135525	ENSG00000135525	MAP7	6:136663874-136873692	q1	q2	OK	2.87339	2.42584	-0.244266	-0.359909	0.61625	0.99995	no

ENSG00000135535	ENSG00000135535	CD164	6:109687716-109703762	q1	q2	OK	40.19	40.1377	-0.00188019	-0.0042543	0.99535	0.99995	no

ENSG00000135537	ENSG00000135537	LACE1	6:108616097-108847999	q1	q2	OK	1.7057	2.17104	0.348026	0.387397	0.4671	0.99995	no

ENSG00000135540	ENSG00000135540	NHSL1	6:138723721-139018425	q1	q2	OK	23.3781	23.376	-0.000130843	-0.000192263	0.99965	0.99995	no

ENSG00000135541	ENSG00000135541	AHI1	6:135604669-136037193	q1	q2	OK	12.9017	12.5574	-0.0390291	-0.0446354	0.9287	0.99995	no

ENSG00000135547	ENSG00000135547	HEY2	6:125899703-126082415	q1	q2	OK	4.86045	4.70479	-0.0469593	-0.0747481	0.915	0.99995	no

ENSG00000135549	ENSG00000135549	PKIB	6:122793075-123047518	q1	q2	NOTEST	0.285819	0.434951	0.605752	0	1	1	no

ENSG00000135569	ENSG00000135569	TAAR5	6:132909730-132910877	q1	q2	NOTEST	0.0264528	0	#NAME?	0	1	1	no

ENSG00000135577	ENSG00000135577	NMBR	6:142379466-142410356	q1	q2	NOTEST	0.12679	0.194694	0.618766	0	1	1	no

ENSG00000135587	ENSG00000135587	SMPD2	6:109711417-109765122	q1	q2	OK	3.23871	3.54185	0.129085	0.11859	0.8525	0.99995	no

ENSG00000135596	ENSG00000135596	MICAL1	6:109765264-109804440	q1	q2	OK	33.0182	30.75	-0.102679	-0.150928	0.7866	0.99995	no

ENSG00000135597	ENSG00000135597	REPS1	6:139117062-139309398	q1	q2	OK	29.2658	29.9998	0.0357396	0.053594	0.931	0.99995	no

ENSG00000135604	ENSG00000135604	STX11	6:144471662-144509507	q1	q2	NOTEST	0.0419967	0.0276301	-0.604034	0	1	1	no

ENSG00000135605	ENSG00000135605	TEC	4:48137799-48271881	q1	q2	NOTEST	0.0844761	0.0599041	-0.495888	0	1	1	no

ENSG00000135617	ENSG00000135617	PRADC1	2:73455133-73460366	q1	q2	OK	11.1487	9.72402	-0.197245	-0.300622	0.6553	0.99995	no

ENSG00000135622	ENSG00000135622	SEMA4F	2:74881354-74909186	q1	q2	OK	7.65287	6.61379	-0.210523	-0.312179	0.554	0.99995	no

ENSG00000135624	ENSG00000135624	CCT7	2:73460547-73480149	q1	q2	OK	227.861	230.006	0.0135128	0.0270852	0.9673	0.99995	no

ENSG00000135625	ENSG00000135625	EGR4	2:73518057-73520833	q1	q2	NOTEST	0	0	0	0	1	1	no

ENSG00000135631	ENSG00000135631	RAB11FIP5	2:73169164-73383849	q1	q2	OK	5.02425	3.77278	-0.413282	-0.307793	0.58185	0.99995	no

ENSG00000135632	ENSG00000135632	SMYD5	2:73441349-73454365	q1	q2	OK	17.7816	17.1826	-0.0494365	-0.0630884	0.8795	0.99995	no

ENSG00000135636	ENSG00000135636	DYSF	2:71680851-71913898	q1	q2	OK	2.93808	3.0214	0.0403453	0.0618135	0.92305	0.99995	no

ENSG00000135637	ENSG00000135637	CCDC142	2:74699084-74722013	q1	q2	OK	8.80706	8.15133	-0.111626	-0.0762306	0.8728	0.99995	no

ENSG00000135638	ENSG00000135638	EMX1	2:73143388-73162020	q1	q2	NOTEST	0	0.032403	inf	0	1	1	no

ENSG00000135643	ENSG00000135643	KCNMB4	12:70760055-70828072	q1	q2	OK	7.48777	6.38197	-0.230533	-0.283369	0.5564	0.99995	no

ENSG00000135655	ENSG00000135655	USP15	12:62102039-62811211	q1	q2	OK	21.8157	23.7092	0.120081	0.139084	0.81595	0.99995	no

ENSG00000135677	ENSG00000135677	GNS	12:65107224-65153227	q1	q2	OK	30.5208	30.9276	0.0190999	0.0426651	0.945	0.99995	no

ENSG00000135678	ENSG00000135678	CPM	12:69201955-69365350	q1	q2	OK	3.48947	4.19886	0.266993	0.0544875	0.84475	0.99995	no

ENSG00000135679	ENSG00000135679	MDM2	12:69201955-69365350	q1	q2	OK	46.5323	43.8821	-0.0846028	-0.0827651	0.85095	0.99995	no

ENSG00000135686	ENSG00000135686	KLHL36	16:84682130-84701292	q1	q2	OK	11.8	11.6545	-0.0178925	-0.0307359	0.9607	0.99995	no

ENSG00000135697	ENSG00000135697	BCMO1	16:81272052-81324747	q1	q2	NOTEST	0.0597293	0	#NAME?	0	1	1	no

ENSG00000135698	ENSG00000135698	MPHOSPH6	16:82181402-82203831	q1	q2	OK	38.0063	39.9777	0.0729576	0.139985	0.83215	0.99995	no

ENSG00000135702	ENSG00000135702	CHST5	16:75562429-75657198	q1	q2	OK	0.797584	0.468997	-0.766059	-0.144943	0.637	0.99995	no

ENSG00000135709	ENSG00000135709	KIAA0513	16:85061374-85127836	q1	q2	OK	6.84525	6.36067	-0.105924	-0.133325	0.8044	0.99995	no

ENSG00000135720	ENSG00000135720	DYNC1LI2	16:66754795-66788643	q1	q2	OK	93.015	94.3925	0.0212098	0.0318326	0.9443	0.99995	no

ENSG00000135722	ENSG00000135722	FBXL8	16:67188082-67203848	q1	q2	OK	0.583164	0.351789	-0.729191	-0.279972	0.52525	0.99995	no

ENSG00000135723	ENSG00000135723	FHOD1	16:67263289-67306093	q1	q2	OK	15.7585	13.5958	-0.212965	-0.253406	0.6573	0.99995	no

ENSG00000135736	ENSG00000135736	CCDC102A	16:57546089-57570511	q1	q2	OK	7.29553	5.44035	-0.423315	-0.54626	0.4302	0.99995	no

ENSG00000135740	ENSG00000135740	SLC9A5	16:67263289-67306093	q1	q2	OK	1.51665	1.04526	-0.537021	-0.330747	0.5576	0.99995	no

ENSG00000135744	ENSG00000135744	AGT	1:230838268-230937749	q1	q2	NOTEST	0.103991	0.0676121	-0.621105	0	1	1	no

ENSG00000135747	ENSG00000135747	ZNF670	1:247108848-247242113	q1	q2	OK	8.29854	9.26779	0.159367	0.247346	0.72215	0.99995	no

ENSG00000135749	ENSG00000135749	PCNXL2	1:233086350-233431473	q1	q2	OK	7.66235	8.00436	0.0629989	0.0470317	0.8785	0.99995	no

ENSG00000135750	ENSG00000135750	KCNK1	1:233749749-233808258	q1	q2	OK	0.689572	0.450244	-0.614995	-0.505662	0.30915	0.99995	no

ENSG00000135763	ENSG00000135763	URB2	1:229761980-229795946	q1	q2	OK	6.62542	7.5447	0.187452	0.297853	0.57605	0.99995	no

ENSG00000135766	ENSG00000135766	EGLN1	1:231499496-231560790	q1	q2	OK	13.7467	13.096	-0.0699674	-0.0850062	0.89435	0.99995	no

ENSG00000135773	ENSG00000135773	CAPN9	1:230838268-230937749	q1	q2	OK	2.21957	2.12752	-0.0611063	-0.0421232	0.95285	0.99995	no

ENSG00000135775	ENSG00000135775	COG2	1:230778234-230829728	q1	q2	OK	17.1127	18.0149	0.0741253	0.145637	0.81125	0.99995	no

ENSG00000135776	ENSG00000135776	ABCB10	1:229652328-229694442	q1	q2	OK	8.56458	9.40811	0.135522	0.26385	0.6874	0.99995	no

ENSG00000135778	ENSG00000135778	NTPCR	1:233086350-233431473	q1	q2	OK	39.3596	40.8638	0.0541084	0.0713568	0.9203	0.99995	no

ENSG00000135801	ENSG00000135801	TAF5L	1:229728857-229761794	q1	q2	OK	16.3354	16.1324	-0.0180451	-0.0360263	0.95095	0.99995	no

ENSG00000135821	ENSG00000135821	GLUL	1:182350838-182361341	q1	q2	OK	229.866	249.981	0.121022	0.173076	0.8071	0.99995	no

ENSG00000135823	ENSG00000135823	STX6	1:180882289-180992047	q1	q2	OK	15.7611	15.3168	-0.0412529	-0.0873449	0.8969	0.99995	no

ENSG00000135824	ENSG00000135824	RGS8	1:182615238-182653711	q1	q2	NOTEST	0.250405	0.294492	0.233966	0	1	1	no

ENSG00000135828	ENSG00000135828	RNASEL	1:182542768-182558391	q1	q2	NOTEST	0.481026	0.443253	-0.117986	0	1	1	no

ENSG00000135829	ENSG00000135829	DHX9	1:182808503-182856886	q1	q2	OK	152.45	160.591	0.0750552	0.0970178	0.81815	0.99995	no

ENSG00000135835	ENSG00000135835	KIAA1614	1:180882289-180992047	q1	q2	OK	0.662793	0.712947	0.105237	0.0410779	0.91265	0.99995	no

ENSG00000135837	ENSG00000135837	CEP350	1:179923872-180084015	q1	q2	OK	13.8146	16.2368	0.233073	0.270589	0.5557	0.99995	no

ENSG00000135838	ENSG00000135838	NPL	1:182758427-182799519	q1	q2	OK	1.39175	1.62343	0.222139	0.1824	0.7154	0.99995	no

ENSG00000135842	ENSG00000135842	FAM129A	1:184759857-184943682	q1	q2	OK	1.18538	1.15133	-0.0420491	-0.0584496	0.92635	0.99995	no

ENSG00000135845	ENSG00000135845	PIGC	1:171810620-172437971	q1	q2	OK	24.632	25.8835	0.0714977	0.133908	0.84605	0.99995	no

ENSG00000135847	ENSG00000135847	ACBD6	1:180199420-180472089	q1	q2	OK	34.4685	38.948	0.17627	0.249675	0.7173	0.99995	no

ENSG00000135862	ENSG00000135862	LAMC1	1:182992594-183114727	q1	q2	OK	144.826	160.663	0.149719	0.21171	0.70515	0.99995	no

ENSG00000135870	ENSG00000135870	RC3H1	1:173900351-173991435	q1	q2	OK	6.046	6.06911	0.00550567	0.00641213	0.9851	0.99995	no

ENSG00000135898	ENSG00000135898	GPR55	2:231772032-231825781	q1	q2	NOTEST	0.0192766	0.0332265	0.785483	0	1	1	no

ENSG00000135899	ENSG00000135899	SP110	2:230990025-231268447	q1	q2	OK	1.11231	1.10518	-0.00927849	-0.00508889	0.98855	0.99995	no

ENSG00000135900	ENSG00000135900	MRPL44	2:224822120-224832431	q1	q2	OK	25.9426	26.0252	0.00458789	0.00997567	0.98875	0.99995	no

ENSG00000135902	ENSG00000135902	CHRND	2:233390702-233401377	q1	q2	NOTEST	0	0.0174915	inf	0	1	1	no

ENSG00000135903	ENSG00000135903	PAX3	2:223064606-223169936	q1	q2	NOTEST	0.28648	0.25185	-0.185869	0	1	1	no

ENSG00000135905	ENSG00000135905	DOCK10	2:225629806-225907162	q1	q2	OK	3.0943	4.09801	0.405308	0.309751	0.4201	0.99995	no

ENSG00000135912	ENSG00000135912	TTLL4	2:219575567-219620139	q1	q2	OK	30.1201	29.5107	-0.0294906	-0.0354443	0.9589	0.99995	no

ENSG00000135913	ENSG00000135913	USP37	2:219283814-219433084	q1	q2	OK	8.64361	9.29594	0.104966	0.19334	0.7199	0.99995	no

ENSG00000135914	ENSG00000135914	HTR2B	2:231921577-232037541	q1	q2	NOTEST	0.108067	0.0691311	-0.644513	0	1	1	no

ENSG00000135916	ENSG00000135916	ITM2C	2:231729353-231743963	q1	q2	OK	136.142	126.489	-0.106103	-0.242145	0.7181	0.99995	no

ENSG00000135917	ENSG00000135917	SLC19A3	2:228549925-228582728	q1	q2	NOTEST	0.00816838	0.0632576	2.95312	0	1	1	no

ENSG00000135919	ENSG00000135919	SERPINE2	2:224839828-224904036	q1	q2	OK	38.8665	48.4513	0.318007	0.710747	0.28915	0.99995	no

ENSG00000135924	ENSG00000135924	DNAJB2	2:220143988-220151622	q1	q2	OK	26.1722	23.5831	-0.150278	-0.283747	0.64065	0.99995	no

ENSG00000135925	ENSG00000135925	WNT10A	2:219745084-219764303	q1	q2	NOTEST	0	0.00979971	inf	0	1	1	no

ENSG00000135926	ENSG00000135926	TMBIM1	2:219135114-219232822	q1	q2	OK	1.39906	1.16521	-0.263872	-0.123764	0.72145	0.99995	no

ENSG00000135929	ENSG00000135929	CYP27A1	2:219646471-219680016	q1	q2	OK	1.24434	1.03183	-0.270178	-0.256957	0.692	0.99995	no

ENSG00000135930	ENSG00000135930	EIF4E2	2:233412778-233547491	q1	q2	OK	56.1702	51.6902	-0.119914	-0.139618	0.77535	0.99995	no

ENSG00000135931	ENSG00000135931	ARMC9	2:232063259-232239548	q1	q2	OK	14.7057	13.0388	-0.173561	-0.246369	0.67855	0.99995	no

ENSG00000135932	ENSG00000135932	CAB39	2:231577559-231685790	q1	q2	OK	28.0844	29.2453	0.0584368	0.127364	0.8351	0.99995	no

ENSG00000135940	ENSG00000135940	COX5B	2:98262502-98264846	q1	q2	OK	94.7398	85.8197	-0.142663	-0.319806	0.641	0.99995	no

ENSG00000135945	ENSG00000135945	REV1	2:99613723-100106497	q1	q2	OK	27.8649	29.2737	0.0711538	0.0409278	0.89855	0.99995	no

ENSG00000135951	ENSG00000135951	TSGA10	2:99613723-100106497	q1	q2	OK	3.85939	3.73782	-0.0461752	-0.00497323	0.9503	0.99995	no

ENSG00000135953	ENSG00000135953	MFSD9	2:103332298-103353347	q1	q2	OK	6.43742	7.17713	0.156926	0.146289	0.6927	0.99995	no

ENSG00000135956	ENSG00000135956	TMEM127	2:96914253-96931732	q1	q2	OK	14.225	13.2118	-0.106602	-0.228747	0.7437	0.99995	no

ENSG00000135960	ENSG00000135960	EDAR	2:109510926-109605828	q1	q2	NOTEST	0.0495207	0.0356082	-0.475822	0	1	1	no

ENSG00000135966	ENSG00000135966	TGFBRAP1	2:105880870-105946986	q1	q2	OK	7.46654	7.38754	-0.0153459	-0.0338043	0.9598	0.99995	no

ENSG00000135968	ENSG00000135968	GCC2	2:109065016-109150652	q1	q2	OK	13.3119	13.6864	0.040023	0.0535943	0.9137	0.99995	no

ENSG00000135972	ENSG00000135972	MRPS9	2:105552868-105719402	q1	q2	OK	29.9407	30.405	0.0222041	0.0481018	0.9457	0.99995	no

ENSG00000135973	ENSG00000135973	GPR45	2:105858199-105859924	q1	q2	NOTEST	0.079509	0.0959384	0.27099	0	1	1	no

ENSG00000135974	ENSG00000135974	C2orf49	2:105950483-105965668	q1	q2	OK	8.96468	9.66608	0.108678	0.139159	0.8293	0.99995	no

ENSG00000135976	ENSG00000135976	ANKRD36	2:97779232-97930258	q1	q2	OK	3.6292	2.8836	-0.331782	-0.430904	0.47485	0.99995	no

ENSG00000135999	ENSG00000135999	EPC2	2:149402008-149545130	q1	q2	OK	19.7163	21.3701	0.116205	0.246354	0.69115	0.99995	no

ENSG00000136002	ENSG00000136002	ARHGEF4	2:131588550-131804836	q1	q2	OK	4.08127	3.98202	-0.0355208	-0.0358226	0.93965	0.99995	no

ENSG00000136003	ENSG00000136003	ISCU	12:108956357-108963160	q1	q2	OK	71.0201	60.8584	-0.22277	-0.480107	0.4853	0.99995	no

ENSG00000136010	ENSG00000136010	ALDH1L2	12:105380087-105478355	q1	q2	OK	2.13468	1.77306	-0.267783	-0.262839	0.60615	0.99995	no

ENSG00000136011	ENSG00000136011	STAB2	12:103981050-104162636	q1	q2	NOTEST	0.0840244	0	#NAME?	0	1	1	no

ENSG00000136014	ENSG00000136014	USP44	12:95910335-95945266	q1	q2	OK	9.63731	9.36227	-0.0417723	-0.0552043	0.9105	0.99995	no

ENSG00000136021	ENSG00000136021	SCYL2	12:100660917-100735502	q1	q2	OK	35.0581	38.8268	0.147305	0.225477	0.6348	0.99995	no

ENSG00000136026	ENSG00000136026	CKAP4	12:106631654-106740793	q1	q2	OK	71.1177	69.742	-0.0281808	-0.0689853	0.9195	0.99995	no

ENSG00000136040	ENSG00000136040	PLXNC1	12:94542498-94853764	q1	q2	OK	10.4911	9.78401	-0.100671	-0.118195	0.8052	0.99995	no

ENSG00000136044	ENSG00000136044	APPL2	12:105567073-105789875	q1	q2	OK	40.5023	39.3837	-0.0404053	-0.0498616	0.9275	0.99995	no

ENSG00000136045	ENSG00000136045	PWP1	12:108079508-108106944	q1	q2	OK	26.6381	29.4091	0.142771	0.319602	0.6443	0.99995	no

ENSG00000136048	ENSG00000136048	DRAM1	12:102271128-102405908	q1	q2	OK	2.86463	2.27126	-0.334858	-0.33264	0.47735	0.99995	no

ENSG00000136051	ENSG00000136051	KIAA1033	12:105501101-105562912	q1	q2	OK	19.1947	20.3994	0.0878177	0.116189	0.7745	0.99995	no

ENSG00000136052	ENSG00000136052	SLC41A2	12:105196330-105352522	q1	q2	OK	1.26142	1.02254	-0.302901	-0.176962	0.62595	0.99995	no

ENSG00000136059	ENSG00000136059	VILL	3:38029549-38048679	q1	q2	NOTEST	0.288597	0.171405	-0.75165	0	1	1	no

ENSG00000136068	ENSG00000136068	FLNB	3:57994126-58157982	q1	q2	OK	14.5877	12.9089	-0.176387	-0.246118	0.5681	0.99995	no

ENSG00000136098	ENSG00000136098	NEK3	13:52706774-52733996	q1	q2	OK	15.7362	17.7224	0.171484	0.24899	0.7108	0.99995	no

ENSG00000136099	ENSG00000136099	PCDH8	13:53418108-53422775	q1	q2	OK	23.4481	15.119	-0.633108	-1.44647	0.0387	0.99995	no

ENSG00000136100	ENSG00000136100	VPS36	13:52986736-53024763	q1	q2	OK	21.4564	22.6557	0.0784679	0.0879815	0.82905	0.99995	no

ENSG00000136104	ENSG00000136104	RNASEH2B	13:51450821-51544592	q1	q2	OK	14.8082	14.8971	0.00863543	0.0142754	0.98355	0.99995	no

ENSG00000136108	ENSG00000136108	CKAP2	13:53029563-53050763	q1	q2	OK	69.2187	77.6769	0.166326	0.365888	0.55235	0.99995	no

ENSG00000136110	ENSG00000136110	LECT1	13:53277398-53313947	q1	q2	NOTEST	0.217917	0.265273	0.283697	0	1	1	no

ENSG00000136111	ENSG00000136111	TBC1D4	13:75858807-76056250	q1	q2	OK	2.604	2.70671	0.0558084	0.0933583	0.88165	0.99995	no

ENSG00000136114	ENSG00000136114	THSD1	13:52951304-52980629	q1	q2	OK	1.83055	1.65857	-0.142331	-0.182547	0.7952	0.99995	no

ENSG00000136122	ENSG00000136122	BORA	13:73302060-73590591	q1	q2	OK	13.0882	13.4615	0.0405675	0.040658	0.95265	0.99995	no

ENSG00000136141	ENSG00000136141	LRCH1	13:47127302-47327175	q1	q2	OK	5.06631	5.77022	0.187691	0.364373	0.5986	0.99995	no

ENSG00000136143	ENSG00000136143	SUCLA2	13:48510621-48621358	q1	q2	OK	28.8315	33.8417	0.231154	0.369437	0.5325	0.99995	no

ENSG00000136144	ENSG00000136144	RCBTB1	13:50106081-50159719	q1	q2	OK	21.55	21.8979	0.0231014	0.0411455	0.93765	0.99995	no

ENSG00000136146	ENSG00000136146	MED4	13:48627458-48669267	q1	q2	OK	41.309	44.7696	0.116064	0.233597	0.7267	0.99995	no

ENSG00000136147	ENSG00000136147	PHF11	13:50069745-50103123	q1	q2	OK	3.44552	3.331	-0.0487655	-0.0485314	0.93945	0.99995	no

ENSG00000136149	ENSG00000136149	RPL13AP25	13:55014838-55015450	q1	q2	OK	10.3767	17.5214	0.755778	1.01083	0.15465	0.99995	no

ENSG00000136152	ENSG00000136152	COG3	13:46039059-46189874	q1	q2	OK	10.4939	11.032	0.0721533	0.143819	0.8085	0.99995	no

ENSG00000136153	ENSG00000136153	LMO7	13:76123618-76434004	q1	q2	OK	3.69166	4.50647	0.287729	0.214158	0.58675	0.99995	no

ENSG00000136155	ENSG00000136155	SCEL	13:78109808-78219398	q1	q2	NOTEST	0.0255601	0.0192417	-0.409657	0	1	1	no

ENSG00000136156	ENSG00000136156	ITM2B	13:48807293-48837063	q1	q2	OK	123.064	112.569	-0.128609	-0.265692	0.6842	0.99995	no

ENSG00000136158	ENSG00000136158	SPRY2	13:80910110-80915086	q1	q2	OK	170.375	194.181	0.188687	0.449082	0.5141	0.99995	no

ENSG00000136159	ENSG00000136159	NUDT15	13:48510621-48621358	q1	q2	OK	23.1094	27.0827	0.228894	0.359499	0.6149	0.99995	no

ENSG00000136160	ENSG00000136160	EDNRB	13:78469615-79233314	q1	q2	OK	17.7256	14.2758	-0.312271	-0.498833	0.4647	0.99995	no

ENSG00000136161	ENSG00000136161	RCBTB2	13:49063094-49107369	q1	q2	OK	12.4206	13.0796	0.0745894	0.141797	0.8181	0.99995	no

ENSG00000136167	ENSG00000136167	LCP1	13:46700054-46786006	q1	q2	NOTEST	0.469013	0.273076	-0.780325	0	1	1	no

ENSG00000136169	ENSG00000136169	SETDB2	13:49882785-50069138	q1	q2	OK	3.47239	3.43212	-0.0168292	-0.0178707	0.976	0.99995	no

ENSG00000136193	ENSG00000136193	SCRN1	7:29959718-30066300	q1	q2	OK	57.1594	61.6725	0.109637	0.245323	0.72535	0.99995	no

ENSG00000136197	ENSG00000136197	C7orf25	7:42948324-42988557	q1	q2	OK	2.51188	1.89917	-0.4034	-0.109986	0.7296	0.99995	no

ENSG00000136205	ENSG00000136205	TNS3	7:47314751-47622156	q1	q2	OK	5.54927	4.96601	-0.160212	-0.324259	0.6194	0.99995	no

ENSG00000136206	ENSG00000136206	SPDYE1	7:43966036-44058774	q1	q2	OK	0.425604	0.532143	0.322304	0.132095	0.62025	0.99995	no

ENSG00000136213	ENSG00000136213	CHST12	7:2443222-2474242	q1	q2	OK	6.72419	5.64174	-0.253221	-0.405157	0.568	0.99995	no

ENSG00000136231	ENSG00000136231	IGF2BP3	7:23338357-23510086	q1	q2	OK	55.9428	59.2305	0.0823883	0.127629	0.79215	0.99995	no

ENSG00000136235	ENSG00000136235	GPNMB	7:23275585-23314727	q1	q2	NOTEST	0.37041	0.470116	0.343894	0	1	1	no

ENSG00000136237	ENSG00000136237	RAPGEF5	7:22157855-22396763	q1	q2	OK	4.66511	4.82541	0.0487415	0.0839855	0.8854	0.99995	no

ENSG00000136238	ENSG00000136238	RAC1	7:6414153-6443608	q1	q2	OK	170.599	173.49	0.0242449	0.0539816	0.9338	0.99995	no

ENSG00000136240	ENSG00000136240	KDELR2	7:6448756-6524873	q1	q2	OK	75.0157	76.8059	0.0340247	0.070592	0.91015	0.99995	no

ENSG00000136243	ENSG00000136243	NUPL2	7:23145352-23240630	q1	q2	OK	27.9073	27.4187	-0.025483	-0.0218424	0.96965	0.99995	no

ENSG00000136244	ENSG00000136244	IL6	7:22765013-22771621	q1	q2	NOTEST	0	0.0434632	inf	0	1	1	no

ENSG00000136247	ENSG00000136247	ZDHHC4	7:6617064-6629005	q1	q2	OK	34.8187	34.4588	-0.0149917	-0.0282229	0.9665	0.99995	no

ENSG00000136250	ENSG00000136250	AOAH	7:36552455-36764154	q1	q2	NOTEST	0.25392	0.261967	0.0450112	0	1	1	no

ENSG00000136261	ENSG00000136261	BZW2	7:16685755-16759523	q1	q2	OK	55.9693	60.7001	0.117063	0.285949	0.68225	0.99995	no

ENSG00000136267	ENSG00000136267	DGKB	7:14184673-15014402	q1	q2	OK	0.796333	0.665295	-0.259378	-0.167288	0.65875	0.99995	no

ENSG00000136270	ENSG00000136270	TBRG4	7:45139698-45151646	q1	q2	OK	30.9205	31.1455	0.0104626	0.0175201	0.98	0.99995	no

ENSG00000136271	ENSG00000136271	DDX56	7:44605015-44614650	q1	q2	OK	39.55	37.8222	-0.0644444	-0.126404	0.82895	0.99995	no

ENSG00000136273	ENSG00000136273	HUS1	7:47735327-48019178	q1	q2	OK	7.91367	8.79244	0.151917	0.208813	0.7422	0.99995	no

ENSG00000136274	ENSG00000136274	NACAD	7:45120036-45128513	q1	q2	OK	4.73024	3.59743	-0.394944	-0.550904	0.299	0.99995	no

ENSG00000136275	ENSG00000136275	C7orf69	7:47735327-48019178	q1	q2	NOTEST	0	0	0	0	1	1	no

ENSG00000136279	ENSG00000136279	DBNL	7:44084238-44109055	q1	q2	OK	47.4414	43.2285	-0.134165	-0.27898	0.67265	0.99995	no

ENSG00000136280	ENSG00000136280	CCM2	7:45039073-45116068	q1	q2	OK	25.9515	23.5758	-0.13851	-0.278897	0.66515	0.99995	no

ENSG00000136286	ENSG00000136286	MYO1G	7:45002260-45018697	q1	q2	NOTEST	0.0363307	0.0234445	-0.631942	0	1	1	no

ENSG00000136295	ENSG00000136295	TTYH3	7:2671584-2704436	q1	q2	OK	104.754	99.7552	-0.0705367	-0.132207	0.80165	0.99995	no

ENSG00000136297	ENSG00000136297	MMD2	7:4945619-4998844	q1	q2	NOTEST	0.0234325	0.0454274	0.955051	0	1	1	no

ENSG00000136305	ENSG00000136305	CIDEB	14:24769067-24787242	q1	q2	NOTEST	0.333656	0.244066	-0.451088	0	1	1	no

ENSG00000136315	ENSG00000136315	RP11-84C10.2	14:21366143-21404414	q1	q2	NOTEST	0	0	0	0	1	1	no

ENSG00000136319	ENSG00000136319	TTC5	14:20724716-20774970	q1	q2	OK	11.4439	13.456	0.23367	0.329053	0.61985	0.99995	no

ENSG00000136327	ENSG00000136327	NKX2-8	14:37049783-37051812	q1	q2	NOTEST	0	0	0	0	1	1	no

ENSG00000136352	ENSG00000136352	NKX2-1	14:36942411-36992221	q1	q2	NOTEST	0	0	0	0	1	1	no

ENSG00000136367	ENSG00000136367	ZFHX2	14:23980968-24048009	q1	q2	OK	5.39584	3.49933	-0.624768	-0.43727	0.27365	0.99995	no

ENSG00000136371	ENSG00000136371	MTHFS	15:80125926-80217194	q1	q2	OK	5.83136	6.89168	0.241023	0.205256	0.7512	0.99995	no

ENSG00000136378	ENSG00000136378	ADAMTS7	15:79051544-79190475	q1	q2	OK	26.1054	23.894	-0.127703	-0.085849	0.86125	0.99995	no

ENSG00000136379	ENSG00000136379	ABHD17C	15:80972024-81047962	q1	q2	OK	26.1697	31.1363	0.250696	0.491294	0.47965	0.99995	no

ENSG00000136381	ENSG00000136381	IREB2	15:78729772-78793798	q1	q2	OK	30.8843	31.8676	0.0452155	0.0603975	0.87575	0.99995	no

ENSG00000136383	ENSG00000136383	ALPK3	15:85359910-85416713	q1	q2	NOTEST	0.142589	0.151482	0.0872841	0	1	1	no

ENSG00000136404	ENSG00000136404	TM6SF1	15:83776158-84108197	q1	q2	OK	5.25123	6.71938	0.355673	0.170924	0.74225	0.99995	no

ENSG00000136425	ENSG00000136425	CIB2	15:78370149-78538030	q1	q2	OK	21.561	16.9326	-0.348622	-0.415382	0.51055	0.99995	no

ENSG00000136436	ENSG00000136436	CALCOCO2	17:46908349-46943884	q1	q2	OK	22.813	20.1257	-0.180816	-0.282082	0.58525	0.99995	no

ENSG00000136444	ENSG00000136444	RSAD1	17:48556160-48563336	q1	q2	OK	18.2174	17.2369	-0.0798169	-0.128161	0.81955	0.99995	no

ENSG00000136448	ENSG00000136448	NMT1	17:43100707-43221548	q1	q2	OK	36.1625	35.8754	-0.0114965	-0.0113962	0.9737	0.99995	no

ENSG00000136449	ENSG00000136449	MYCBPAP	17:48585744-48608862	q1	q2	NOTEST	0.0789239	0.0404302	-0.965031	0	1	1	no

ENSG00000136450	ENSG00000136450	SRSF1	17:56066398-56084707	q1	q2	OK	261.381	285.245	0.126047	0.232675	0.7152	0.99995	no

ENSG00000136451	ENSG00000136451	VEZF1	17:56048909-56065620	q1	q2	OK	68.0321	71.9404	0.0805866	0.181834	0.78485	0.99995	no

ENSG00000136457	ENSG00000136457	CHAD	17:48503518-48552206	q1	q2	NOTEST	0.395614	0.326218	-0.278258	0	1	1	no

ENSG00000136463	ENSG00000136463	TACO1	17:61678230-61685725	q1	q2	OK	13.6487	14.177	0.0547882	0.100502	0.8847	0.99995	no

ENSG00000136478	ENSG00000136478	TEX2	17:62224586-62340661	q1	q2	OK	5.93479	6.55102	0.142524	0.243541	0.7154	0.99995	no

ENSG00000136485	ENSG00000136485	DCAF7	17:61627821-61671639	q1	q2	OK	60.8897	61.486	0.01406	0.0303969	0.959	0.99995	no

ENSG00000136487	ENSG00000136487	GH2	17:61957577-61959295	q1	q2	NOTEST	0	0	0	0	1	1	no

ENSG00000136488	ENSG00000136488	CSH1	17:61972274-61974021	q1	q2	NOTEST	0	0	0	0	1	1	no

ENSG00000136490	ENSG00000136490	LIMD2	17:61699774-61778532	q1	q2	OK	82.8968	75.1634	-0.141285	-0.262093	0.6901	0.99995	no

ENSG00000136492	ENSG00000136492	BRIP1	17:59758626-59940882	q1	q2	OK	15.9444	16.8122	0.0764656	0.152145	0.7962	0.99995	no

ENSG00000136504	ENSG00000136504	KAT7	17:47778304-47906458	q1	q2	OK	63.0103	65.4759	0.0553755	0.08292	0.89225	0.99995	no

ENSG00000136514	ENSG00000136514	RTP4	3:187086119-187089864	q1	q2	NOTEST	0	0	0	0	1	1	no

ENSG00000136518	ENSG00000136518	ACTL6A	3:179280667-179322442	q1	q2	OK	92.6511	104.276	0.170525	0.277045	0.6158	0.99995	no

ENSG00000136521	ENSG00000136521	NDUFB5	3:179322477-179345435	q1	q2	OK	71.5997	75.26	0.0719283	0.153961	0.82745	0.99995	no

ENSG00000136522	ENSG00000136522	MRPL47	3:179280667-179322442	q1	q2	OK	48.8379	52.7454	0.111043	0.13146	0.85565	0.99995	no

ENSG00000136527	ENSG00000136527	TRA2B	3:185633693-185655924	q1	q2	OK	161.714	173.775	0.103775	0.200935	0.75805	0.99995	no

ENSG00000136531	ENSG00000136531	SCN2A	2:166095911-166248818	q1	q2	NOTEST	0.353763	0.333236	-0.086239	0	1	1	no

ENSG00000136535	ENSG00000136535	TBR1	2:162272604-162841792	q1	q2	NOTEST	0.0350913	0.052556	0.582742	0	1	1	no

ENSG00000136536	ENSG00000136536	Mar-07	2:160568999-160761260	q1	q2	OK	73.0673	80.7364	0.143993	0.222048	0.7491	0.99995	no

ENSG00000136541	ENSG00000136541	ERMN	2:158175136-158184225	q1	q2	NOTEST	0.0351209	0.0713894	1.02338	0	1	1	no

ENSG00000136542	ENSG00000136542	GALNT5	2:158114109-158170723	q1	q2	NOTEST	0.0491847	0.0995871	1.01775	0	1	1	no

ENSG00000136546	ENSG00000136546	SCN7A	2:167260082-167350757	q1	q2	OK	1.55822	1.98305	0.347819	0.476229	0.409	0.99995	no

ENSG00000136560	ENSG00000136560	TANK	2:161952741-162111179	q1	q2	OK	21.3329	21.7948	0.0309066	0.0452124	0.93535	0.99995	no

ENSG00000136573	ENSG00000136573	BLK	8:11351509-11422113	q1	q2	NOTEST	0	0	0	0	1	1	no

ENSG00000136574	ENSG00000136574	GATA4	8:11534467-11617511	q1	q2	NOTEST	0	0	0	0	1	1	no

ENSG00000136603	ENSG00000136603	SKIL	3:170075465-170114623	q1	q2	OK	16.238	19.5384	0.266941	0.364805	0.41785	0.99995	no

ENSG00000136628	ENSG00000136628	EPRS	1:220141942-220220000	q1	q2	OK	40.3214	45.982	0.189522	0.329595	0.51255	0.99995	no

ENSG00000136630	ENSG00000136630	HLX	1:221006104-221058401	q1	q2	OK	0.693176	0.184929	-1.90625	-1.19524	0.0493	0.99995	no

ENSG00000136631	ENSG00000136631	VPS45	1:150039368-150117505	q1	q2	OK	6.11509	0.45676	-3.74287	-2.94962	5.00E-05	0.0795045	no

ENSG00000136634	ENSG00000136634	IL10	1:206940946-206945839	q1	q2	NOTEST	0	0	0	0	1	1	no

ENSG00000136636	ENSG00000136636	KCTD3	1:215740734-215795149	q1	q2	OK	25.7257	28.325	0.138868	0.283608	0.63755	0.99995	no

ENSG00000136643	ENSG00000136643	RPS6KC1	1:213224588-213448116	q1	q2	OK	9.46763	10.5265	0.152957	0.266184	0.65515	0.99995	no

ENSG00000136653	ENSG00000136653	RASSF5	1:206680878-206785904	q1	q2	NOTEST	0.0415063	0.109193	1.39548	0	1	1	no

ENSG00000136682	ENSG00000136682	CBWD2	2:114195267-114253766	q1	q2	OK	13.55	15.4643	0.190641	0.350222	0.6006	0.99995	no

ENSG00000136688	ENSG00000136688	IL36G	2:113730779-113743242	q1	q2	NOTEST	0.125368	0.0232349	-2.4318	0	1	1	no

ENSG00000136689	ENSG00000136689	IL1RN	2:113864790-113891593	q1	q2	NOTEST	0	0	0	0	1	1	no

ENSG00000136694	ENSG00000136694	IL36A	2:113763037-113765621	q1	q2	NOTEST	0	0	0	0	1	1	no

ENSG00000136695	ENSG00000136695	IL36RN	2:113816214-113822959	q1	q2	NOTEST	0	0	0	0	1	1	no

ENSG00000136696	ENSG00000136696	IL36B	2:113779667-113810444	q1	q2	NOTEST	0	0	0	0	1	1	no

ENSG00000136697	ENSG00000136697	IL1F10	2:113825546-113833427	q1	q2	NOTEST	0	0	0	0	1	1	no

ENSG00000136698	ENSG00000136698	CFC1	2:131350338-131357123	q1	q2	NOTEST	0.459796	0.473877	0.0435202	0	1	1	no

ENSG00000136699	ENSG00000136699	SMPD4	2:130908980-130948302	q1	q2	OK	35.5257	32.6604	-0.12132	-0.186582	0.72145	0.99995	no

ENSG00000136709	ENSG00000136709	WDR33	2:128458595-128568761	q1	q2	OK	23.3374	24.152	0.0494938	0.0916404	0.8824	0.99995	no

ENSG00000136710	ENSG00000136710	CCDC115	2:131095813-131105383	q1	q2	OK	18.4256	15.7656	-0.224927	-0.329202	0.65265	0.99995	no

ENSG00000136715	ENSG00000136715	SAP130	2:128698790-128785694	q1	q2	OK	17.9677	17.7413	-0.0182903	-0.0433934	0.9479	0.99995	no

ENSG00000136717	ENSG00000136717	BIN1	2:127805602-127864931	q1	q2	OK	28.5741	24.4808	-0.223054	-0.47063	0.489	0.99995	no

ENSG00000136718	ENSG00000136718	IMP4	2:131095813-131105383	q1	q2	OK	23.6189	24.052	0.0262162	0.0378246	0.9446	0.99995	no

ENSG00000136720	ENSG00000136720	HS6ST1	2:128994289-129076151	q1	q2	OK	31.6518	29.6343	-0.0950201	-0.227342	0.7363	0.99995	no

ENSG00000136731	ENSG00000136731	UGGT1	2:128848773-128953251	q1	q2	OK	20.8391	22.2265	0.0929842	0.118454	0.78085	0.99995	no

ENSG00000136732	ENSG00000136732	GYPC	2:127413508-127454246	q1	q2	OK	58.3822	55.5486	-0.0717783	-0.156391	0.8231	0.99995	no

ENSG00000136738	ENSG00000136738	STAM	10:17686123-17757913	q1	q2	OK	0.624903	3.69402	2.56349	1.64851	0.00365	0.99995	no

ENSG00000136750	ENSG00000136750	GAD2	10:26505235-26593487	q1	q2	OK	0.911388	0.826395	-0.141233	-0.0745419	0.9097	0.99995	no

ENSG00000136754	ENSG00000136754	ABI1	10:26986587-27150016	q1	q2	OK	17.5428	18.4109	0.0696748	0.14495	0.82555	0.99995	no

ENSG00000136758	ENSG00000136758	YME1L1	10:27399382-27475853	q1	q2	OK	78.4049	84.6735	0.110967	0.227569	0.74	0.99995	no

ENSG00000136770	ENSG00000136770	DNAJC1	10:22045465-22292698	q1	q2	OK	18.3581	18.0839	-0.0217115	-0.0463497	0.9476	0.99995	no

ENSG00000136783	ENSG00000136783	NIPSNAP3A	9:107509968-107522403	q1	q2	OK	8.5841	8.7043	0.0200606	0.0335038	0.9619	0.99995	no

ENSG00000136802	ENSG00000136802	LRRC8A	9:131595220-131680318	q1	q2	OK	19.4254	17.7351	-0.131337	-0.292545	0.6711	0.99995	no

ENSG00000136807	ENSG00000136807	CDK9	9:130547957-130553066	q1	q2	OK	33.1285	34.1699	0.0446522	0.0689025	0.91405	0.99995	no

ENSG00000136810	ENSG00000136810	TXN	9:113006090-113018920	q1	q2	OK	100.153	103.241	0.0438029	0.101961	0.88175	0.99995	no

ENSG00000136811	ENSG00000136811	ODF2	9:131217464-131263571	q1	q2	OK	44.9036	46.601	0.053531	0.104858	0.8707	0.99995	no

ENSG00000136813	ENSG00000136813	KIAA0368	9:114122971-114247025	q1	q2	OK	20.8885	23.0802	0.143947	0.248016	0.67555	0.99995	no

ENSG00000136816	ENSG00000136816	TOR1B	9:132565431-132573560	q1	q2	OK	12.4617	12.4249	-0.00426184	-0.00635754	0.9919	0.99995	no

ENSG00000136819	ENSG00000136819	C9orf78	9:132589568-132644107	q1	q2	OK	34.4117	34.5858	0.00728344	0.0115192	0.98615	0.99995	no

ENSG00000136824	ENSG00000136824	SMC2	9:106856540-106903698	q1	q2	OK	26.1707	29.4574	0.170679	0.391554	0.5461	0.99995	no

ENSG00000136826	ENSG00000136826	KLF4	9:110247132-110252763	q1	q2	OK	1.36874	1.55477	0.183853	0.200503	0.7604	0.99995	no

ENSG00000136827	ENSG00000136827	TOR1A	9:132575222-132586413	q1	q2	OK	25.4374	25.8207	0.0215748	0.0466122	0.9473	0.99995	no

ENSG00000136828	ENSG00000136828	RALGPS1	9:129677052-129985445	q1	q2	OK	6.18492	4.80746	-0.36348	-0.307085	0.48705	0.99995	no

ENSG00000136830	ENSG00000136830	FAM129B	9:130267617-130341268	q1	q2	OK	21.2711	18.9014	-0.170402	-0.314376	0.598	0.99995	no

ENSG00000136834	ENSG00000136834	OR1J1	9:125239219-125240235	q1	q2	NOTEST	0	0	0	0	1	1	no

ENSG00000136839	ENSG00000136839	OR13C9	9:107379528-107380485	q1	q2	NOTEST	0	0	0	0	1	1	no

ENSG00000136840	ENSG00000136840	ST6GALNAC4	9:130670164-130679317	q1	q2	OK	4.23169	3.69367	-0.196177	-0.235688	0.7002	0.99995	no

ENSG00000136842	ENSG00000136842	TMOD1	9:100263461-100436030	q1	q2	NOTEST	0.0936073	0.08809	-0.0876424	0	1	1	no

ENSG00000136848	ENSG00000136848	DAB2IP	9:124329335-124547809	q1	q2	OK	18.3167	16.0626	-0.189454	-0.38436	0.5025	0.99995	no

ENSG00000136854	ENSG00000136854	STXBP1	9:130374543-130497604	q1	q2	OK	12.6677	12.7032	0.00403807	0.00623992	0.9897	0.99995	no

ENSG00000136856	ENSG00000136856	SLC2A8	9:130159420-130170703	q1	q2	OK	8.82555	7.86018	-0.167123	-0.214281	0.6949	0.99995	no

ENSG00000136859	ENSG00000136859	ANGPTL2	9:129677052-129985445	q1	q2	OK	7.39348	8.40152	0.184397	0.216493	0.70085	0.99995	no

ENSG00000136861	ENSG00000136861	CDK5RAP2	9:123151146-123342448	q1	q2	OK	39.0873	39.4375	0.012867	0.0205321	0.97135	0.99995	no

ENSG00000136866	ENSG00000136866	ZFP37	9:115800659-115819039	q1	q2	OK	7.35073	7.33086	-0.00390484	-0.00691388	0.9924	0.99995	no

ENSG00000136867	ENSG00000136867	SLC31A2	9:115913221-115983641	q1	q2	OK	2.11637	1.7946	-0.237929	-0.128768	0.8588	0.99995	no

ENSG00000136868	ENSG00000136868	SLC31A1	9:115983807-116055185	q1	q2	OK	10.4848	12.4466	0.247458	0.269389	0.56045	0.99995	no

ENSG00000136869	ENSG00000136869	TLR4	9:120466609-120479149	q1	q2	OK	1.11147	1.80388	0.698637	0.849811	0.16905	0.99995	no

ENSG00000136870	ENSG00000136870	ZNF189	9:104161154-104172942	q1	q2	OK	17.0045	16.9814	-0.00196566	-0.00448525	0.99505	0.99995	no

ENSG00000136872	ENSG00000136872	ALDOB	9:104182859-104198105	q1	q2	NOTEST	0.195483	0.0978953	-0.997732	0	1	1	no

ENSG00000136874	ENSG00000136874	STX17	9:102668914-102732618	q1	q2	OK	5.85239	7.0097	0.260328	0.329383	0.5113	0.99995	no

ENSG00000136875	ENSG00000136875	PRPF4	9:115983807-116055185	q1	q2	OK	21.0717	21.1896	0.00804344	0.0132451	0.985	0.99995	no

ENSG00000136877	ENSG00000136877	FPGS	9:130556875-130576606	q1	q2	OK	22.1434	21.9382	-0.0134294	-0.0210654	0.9696	0.99995	no

ENSG00000136878	ENSG00000136878	USP20	9:132589568-132644107	q1	q2	OK	15.3176	11.6127	-0.399489	-0.422296	0.4479	0.99995	no

ENSG00000136881	ENSG00000136881	BAAT	9:104121213-104145801	q1	q2	NOTEST	0.250191	0.375679	0.586468	0	1	1	no

ENSG00000136883	ENSG00000136883	KIF12	9:116848405-116861507	q1	q2	NOTEST	0.0526452	0.0528961	0.00686019	0	1	1	no

ENSG00000136888	ENSG00000136888	ATP6V1G1	9:117350025-117360653	q1	q2	OK	116.554	123.132	0.0792144	0.193253	0.78115	0.99995	no

ENSG00000136891	ENSG00000136891	TEX10	9:103064358-103115221	q1	q2	OK	25.9323	28.3118	0.126653	0.289176	0.66725	0.99995	no

ENSG00000136895	ENSG00000136895	GARNL3	9:129986543-130155939	q1	q2	OK	3.61943	2.74982	-0.396422	-0.24749	0.4585	0.99995	no

ENSG00000136897	ENSG00000136897	MRPL50	9:104149914-104160896	q1	q2	OK	13.3339	14.8083	0.151309	0.311923	0.63825	0.99995	no

ENSG00000136908	ENSG00000136908	DPM2	9:130696781-130742792	q1	q2	OK	24.2495	24.2848	0.00209928	0.00281642	0.9967	0.99995	no

ENSG00000136918	ENSG00000136918	WDR38	9:127615754-127624260	q1	q2	NOTEST	0	0	0	0	1	1	no

ENSG00000136925	ENSG00000136925	TSTD2	9:100263461-100436030	q1	q2	OK	21.5964	20.9108	-0.0465486	-0.0585711	0.91915	0.99995	no

ENSG00000136928	ENSG00000136928	GABBR2	9:101050390-101471479	q1	q2	NOTEST	0.378217	0.273523	-0.467551	0	1	1	no

ENSG00000136929	ENSG00000136929	HEMGN	9:100689072-100707138	q1	q2	NOTEST	0	0	0	0	1	1	no

ENSG00000136930	ENSG00000136930	PSMB7	9:127115744-127177723	q1	q2	OK	134.392	137.399	0.0319241	0.076643	0.91015	0.99995	no

ENSG00000136931	ENSG00000136931	NR5A1	9:127243515-127269709	q1	q2	NOTEST	0.102216	0.049703	-1.04021	0	1	1	no

ENSG00000136932	ENSG00000136932	C9orf156	9:100666770-100684852	q1	q2	OK	6.11015	5.61086	-0.122984	-0.151309	0.8114	0.99995	no

ENSG00000136933	ENSG00000136933	RABEPK	9:127962820-127996437	q1	q2	OK	8.35959	8.0581	-0.0529913	-0.0865248	0.9023	0.99995	no

ENSG00000136935	ENSG00000136935	GOLGA1	9:127640645-127905785	q1	q2	OK	9.17692	8.53819	-0.104081	-0.144438	0.8134	0.99995	no

ENSG00000136936	ENSG00000136936	XPA	9:100437190-100459639	q1	q2	OK	12.9713	13.8049	0.0898544	0.15236	0.8225	0.99995	no

ENSG00000136937	ENSG00000136937	NCBP1	9:100263461-100436030	q1	q2	OK	23.2227	25.0779	0.110882	0.146919	0.76185	0.99995	no

ENSG00000136938	ENSG00000136938	ANP32B	9:100745642-100778225	q1	q2	OK	174.094	179.141	0.04123	0.09982	0.8816	0.99995	no

ENSG00000136939	ENSG00000136939	OR1L4	9:125486268-125487204	q1	q2	NOTEST	0	0	0	0	1	1	no

ENSG00000136940	ENSG00000136940	PDCL	9:125560667-125590910	q1	q2	OK	22.8989	23.9385	0.0640549	0.131684	0.82815	0.99995	no

ENSG00000136942	ENSG00000136942	RPL35	9:127615754-127624260	q1	q2	OK	872.944	807.698	-0.112074	-0.275123	0.6934	0.99995	no

ENSG00000136943	ENSG00000136943	CTSV	9:99794939-99801925	q1	q2	OK	10.9532	11.6729	0.0918079	0.159753	0.8195	0.99995	no

ENSG00000136944	ENSG00000136944	LMX1B	9:129376721-129463312	q1	q2	NOTEST	0.264623	0.256255	-0.0463576	0	1	1	no

ENSG00000136950	ENSG00000136950	ARPC5L	9:127624408-127640003	q1	q2	OK	31.5778	33.0909	0.0675239	0.145586	0.8292	0.99995	no

ENSG00000136960	ENSG00000136960	ENPP2	8:120569325-120685693	q1	q2	OK	55.3898	49.0345	-0.175822	-0.384801	0.57565	0.99995	no

ENSG00000136982	ENSG00000136982	DSCC1	8:120846215-120868250	q1	q2	OK	14.7578	18.7054	0.34198	0.715593	0.29475	0.99995	no

ENSG00000136986	ENSG00000136986	DERL1	8:124014799-124164393	q1	q2	OK	28.5151	30.3466	0.0898087	0.181152	0.78335	0.99995	no

ENSG00000136997	ENSG00000136997	MYC	8:128747679-128753674	q1	q2	OK	10.3218	9.69036	-0.0910697	-0.16492	0.8044	0.99995	no

ENSG00000136999	ENSG00000136999	NOV	8:120428545-120474590	q1	q2	OK	8.13552	12.7918	0.652914	1.12741	0.0749	0.99995	no

ENSG00000137033	ENSG00000137033	IL33	9:6215804-6257983	q1	q2	NOTEST	0.268278	0.226617	-0.243476	0	1	1	no

ENSG00000137038	ENSG00000137038	TMEM261	9:7796489-7888380	q1	q2	OK	22.9483	21.5768	-0.0889079	-0.0954231	0.8869	0.99995	no

ENSG00000137040	ENSG00000137040	RANBP6	9:6011042-6015618	q1	q2	OK	12.7901	13.8142	0.111122	0.238569	0.71105	0.99995	no

ENSG00000137054	ENSG00000137054	POLR1E	9:37485931-37503694	q1	q2	OK	13.0534	12.9887	-0.00716841	-0.0138629	0.98495	0.99995	no

ENSG00000137055	ENSG00000137055	PLAA	9:26904080-27062928	q1	q2	OK	21.8543	24.5674	0.168825	0.267653	0.6233	0.99995	no

ENSG00000137070	ENSG00000137070	IL11RA	9:34638129-34681295	q1	q2	OK	17.2423	17.2415	-7.31E-05	-7.67E-05	0.9998	0.99995	no

ENSG00000137073	ENSG00000137073	UBAP2	9:33921690-34048947	q1	q2	OK	15.0736	14.926	-0.0141984	-0.0229621	0.96555	0.99995	no

ENSG00000137074	ENSG00000137074	APTX	9:32972603-33025166	q1	q2	OK	21.5566	21.9036	0.0230383	0.0405736	0.9495	0.99995	no

ENSG00000137075	ENSG00000137075	RNF38	9:36336392-36487545	q1	q2	OK	24.5374	26.0056	0.0838375	0.187985	0.7699	0.99995	no

ENSG00000137076	ENSG00000137076	TLN1	9:35696944-35758572	q1	q2	OK	43.7863	36.8736	-0.247891	-0.162399	0.6245	0.99995	no

ENSG00000137077	ENSG00000137077	CCL21	9:34709001-34710121	q1	q2	NOTEST	0	0	0	0	1	1	no

ENSG00000137078	ENSG00000137078	SIT1	9:35649291-35650937	q1	q2	NOTEST	0	0	0	0	1	1	no

ENSG00000137080	ENSG00000137080	IFNA21	9:21165635-21166659	q1	q2	NOTEST	0.0864027	0.0597197	-0.532869	0	1	1	no

ENSG00000137090	ENSG00000137090	DMRT1	9:841689-969090	q1	q2	NOTEST	0	0.0194812	inf	0	1	1	no

ENSG00000137094	ENSG00000137094	DNAJB5	9:34989637-34998897	q1	q2	OK	19.1357	19.2301	0.00709956	0.0147726	0.9827	0.99995	no

ENSG00000137098	ENSG00000137098	SPAG8	9:35792150-35812269	q1	q2	OK	1.16543	0.968394	-0.267195	-0.12154	0.7964	0.99995	no

ENSG00000137100	ENSG00000137100	DCTN3	9:34613547-34620515	q1	q2	OK	80.8061	74.9775	-0.108008	-0.22967	0.7391	0.99995	no

ENSG00000137101	ENSG00000137101	CD72	9:35605366-35647096	q1	q2	OK	0.538265	0.591481	0.136015	0.0342408	0.87645	0.99995	no

ENSG00000137103	ENSG00000137103	TMEM8B	9:35812956-35854844	q1	q2	OK	8.95525	8.56063	-0.0650173	-0.0992009	0.8731	0.99995	no

ENSG00000137106	ENSG00000137106	GRHPR	9:37422662-37436987	q1	q2	OK	52.8919	49.3899	-0.0988302	-0.206728	0.7629	0.99995	no

ENSG00000137124	ENSG00000137124	ALDH1B1	9:38392660-38398658	q1	q2	OK	11.4023	12.9508	0.183712	0.394021	0.568	0.99995	no

ENSG00000137133	ENSG00000137133	HINT2	9:35812956-35854844	q1	q2	OK	39.5938	31.8814	-0.312557	-0.416202	0.56415	0.99995	no

ENSG00000137135	ENSG00000137135	ARHGEF39	9:35658300-35681156	q1	q2	OK	9.9584	8.05958	-0.305209	-0.357892	0.5843	0.99995	no

ENSG00000137142	ENSG00000137142	IGFBPL1	9:38408990-38424444	q1	q2	OK	33.1835	32.8271	-0.0155791	-0.0322452	0.96075	0.99995	no

ENSG00000137145	ENSG00000137145	DENND4C	9:19230432-19374139	q1	q2	OK	14.0149	17.1682	0.292776	0.428778	0.3621	0.99995	no

ENSG00000137154	ENSG00000137154	RPS6	9:19375448-19380252	q1	q2	OK	837.829	829.75	-0.0139794	-0.0298715	0.96495	0.99995	no

ENSG00000137161	ENSG00000137161	CNPY3	6:42896937-42907025	q1	q2	OK	34.5205	33.7529	-0.0324438	-0.0734684	0.91825	0.99995	no

ENSG00000137166	ENSG00000137166	FOXP4	6:41462590-41570122	q1	q2	OK	41.3639	37.2381	-0.151593	-0.352132	0.5958	0.99995	no

ENSG00000137168	ENSG00000137168	PPIL1	6:36822602-36896740	q1	q2	OK	66.9175	68.0028	0.0232107	0.0413984	0.95065	0.99995	no

ENSG00000137171	ENSG00000137171	KLC4	6:43005354-43042837	q1	q2	OK	19.2762	16.5031	-0.224088	-0.214049	0.64195	0.99995	no

ENSG00000137177	ENSG00000137177	KIF13A	6:17759413-17987854	q1	q2	OK	13.9169	15.3293	0.139457	0.268247	0.6622	0.99995	no

ENSG00000137185	ENSG00000137185	ZSCAN9	6:28192663-28201260	q1	q2	OK	22.34	22.9119	0.0364714	0.0748384	0.91065	0.99995	no

ENSG00000137193	ENSG00000137193	PIM1	6:37137978-37143202	q1	q2	OK	9.0838	8.76803	-0.0510429	-0.0859846	0.89675	0.99995	no

ENSG00000137198	ENSG00000137198	GMPR	6:16238810-16295780	q1	q2	OK	0.668993	1.05012	0.650496	0.461115	0.4565	0.99995	no

ENSG00000137200	ENSG00000137200	CMTR1	6:37400994-37450603	q1	q2	OK	20.9541	21.1478	0.0132792	0.0226285	0.9684	0.99995	no

ENSG00000137203	ENSG00000137203	TFAP2A	6:10393418-10419892	q1	q2	OK	0.714895	0.522723	-0.451684	-0.260362	0.48455	0.99995	no

ENSG00000137204	ENSG00000137204	SLC22A7	6:43263431-43337216	q1	q2	NOTEST	0.035358	0.0731009	1.04785	0	1	1	no

ENSG00000137207	ENSG00000137207	YIPF3	6:43474706-43543812	q1	q2	OK	73.8215	73.8831	0.00120389	0.00154787	0.9982	0.99995	no

ENSG00000137210	ENSG00000137210	TMEM14B	6:10747991-10979553	q1	q2	OK	53.3082	54.4902	0.0316407	0.0637729	0.92715	0.99995	no

ENSG00000137216	ENSG00000137216	TMEM63B	6:44081193-44123256	q1	q2	OK	16.7448	16.3099	-0.0379668	-0.055113	0.93125	0.99995	no

ENSG00000137218	ENSG00000137218	FRS3	6:41737913-41863099	q1	q2	OK	4.09924	3.31252	-0.307426	-0.121273	0.87005	0.99995	no

ENSG00000137221	ENSG00000137221	TJAP1	6:43445260-43474294	q1	q2	OK	22.2466	20.3216	-0.130567	-0.249457	0.6937	0.99995	no

ENSG00000137225	ENSG00000137225	CAPN11	6:44126547-44152139	q1	q2	NOTEST	0.047913	0.019548	-1.2934	0	1	1	no

ENSG00000137251	ENSG00000137251	TINAG	6:54172656-54254950	q1	q2	NOTEST	0	0	0	0	1	1	no

ENSG00000137252	ENSG00000137252	HCRTR2	6:55039049-55147418	q1	q2	NOTEST	0.015289	0.0893806	2.54747	0	1	1	no

ENSG00000137259	ENSG00000137259	HIST1H2AB	6:26033319-26033796	q1	q2	NOTEST	0	0	0	0	1	1	no

ENSG00000137261	ENSG00000137261	KIAA0319	6:24544331-24646383	q1	q2	OK	0.491622	0.33336	-0.560469	-0.641992	0.368	0.99995	no

ENSG00000137265	ENSG00000137265	IRF4	6:391738-411447	q1	q2	NOTEST	0.0426288	0.0364644	-0.225338	0	1	1	no

ENSG00000137266	ENSG00000137266	SLC22A23	6:3224494-3457256	q1	q2	OK	10.6909	10.813	0.0163842	0.0101945	0.9834	0.99995	no

ENSG00000137267	ENSG00000137267	TUBB2A	6:3153902-3157760	q1	q2	OK	93.932	98.8384	0.0734555	0.178718	0.79765	0.99995	no

ENSG00000137269	ENSG00000137269	LRRC1	6:53659294-53788919	q1	q2	OK	3.35773	2.97003	-0.177007	-0.206553	0.6989	0.99995	no

ENSG00000137270	ENSG00000137270	GCM1	6:52991761-53013627	q1	q2	NOTEST	0.0467579	0.0660263	0.497829	0	1	1	no

ENSG00000137273	ENSG00000137273	FOXF2	6:1390068-1395832	q1	q2	NOTEST	0.0484206	0.0239962	-1.01281	0	1	1	no

ENSG00000137274	ENSG00000137274	BPHL	6:3118607-3153812	q1	q2	OK	23.3079	24.3256	0.0616568	0.0909898	0.89655	0.99995	no

ENSG00000137275	ENSG00000137275	RIPK1	6:3064224-3115421	q1	q2	OK	8.78468	8.80392	0.00315624	0.00649627	0.99255	0.99995	no

ENSG00000137285	ENSG00000137285	TUBB2B	6:3224494-3457256	q1	q2	OK	205.253	208.714	0.024126	0.0524059	0.93795	0.99995	no

ENSG00000137288	ENSG00000137288	UQCC2	6:33588141-33679504	q1	q2	OK	69.3404	68.4988	-0.0176171	-0.0245421	0.9734	0.99995	no

ENSG00000137309	ENSG00000137309	HMGA1	6:34204649-34214008	q1	q2	OK	115.974	103.948	-0.157939	-0.37887	0.5915	0.99995	no

ENSG00000137310	ENSG00000137310	TCF19	6:31126318-31148508	q1	q2	OK	2.09357	2.18051	0.0587023	0.0503084	0.94185	0.99995	no

ENSG00000137312	ENSG00000137312	FLOT1	6:30695485-30710510	q1	q2	OK	0	5.30761	inf	#NAME?	5.00E-05	0.0795045	no

ENSG00000137331	ENSG00000137331	IER3	6:30710705-30712331	q1	q2	NOTEST	0.0239889	0	#NAME?	0	1	1	no

ENSG00000137337	ENSG00000137337	MDC1	6:30667583-30685666	q1	q2	OK	0.203236	1.88113	3.21037	1.01306	0.00775	0.99995	no

ENSG00000137338	ENSG00000137338	PGBD1	6:28249313-28270326	q1	q2	OK	9.57372	10.0806	0.0744318	0.1527	0.82785	0.99995	no

ENSG00000137343	ENSG00000137343	ATAT1	6:30594618-30614600	q1	q2	NOTEST	0	0	0	0	1	1	no

ENSG00000137364	ENSG00000137364	TPMT	6:18128541-18155305	q1	q2	OK	5.01292	5.9742	0.253093	0.455011	0.51315	0.99995	no

ENSG00000137392	ENSG00000137392	CLPS	6:35762758-35765088	q1	q2	NOTEST	0	0	0	0	1	1	no

ENSG00000137393	ENSG00000137393	RNF144B	6:18366966-18469105	q1	q2	OK	0.48511	0.628807	0.374304	0.226349	0.63975	0.99995	no

ENSG00000137404	ENSG00000137404	NRM	6:30655823-30659197	q1	q2	NOTEST	0	0	0	0	1	1	no

ENSG00000137409	ENSG00000137409	MTCH1	6:36935916-36954074	q1	q2	OK	95.3832	92.8499	-0.0388346	-0.090033	0.88435	0.99995	no

ENSG00000137411	ENSG00000137411	VARS2	6:30875960-30894236	q1	q2	NOTEST	0	0	0	0	1	1	no

ENSG00000137413	ENSG00000137413	TAF8	6:42018250-42055199	q1	q2	OK	11.3179	11.2361	-0.0104683	-0.0125855	0.98305	0.99995	no

ENSG00000137414	ENSG00000137414	FAM8A1	6:17600585-17611950	q1	q2	OK	11.2152	11.4837	0.034134	0.0781569	0.9081	0.99995	no

ENSG00000137434	ENSG00000137434	C6orf52	6:10671650-10710015	q1	q2	OK	4.39848	3.83768	-0.196769	-0.0883932	0.8793	0.99995	no

ENSG00000137440	ENSG00000137440	FGFBP1	4:15937191-15940363	q1	q2	NOTEST	0	0	0	0	1	1	no

ENSG00000137441	ENSG00000137441	FGFBP2	4:15961865-16086001	q1	q2	NOTEST	0.0490317	0.0747564	0.608481	0	1	1	no

ENSG00000137449	ENSG00000137449	CPEB2	4:15004297-15447790	q1	q2	OK	3.7019	3.56985	-0.0524033	-0.0788941	0.8893	0.99995	no

ENSG00000137460	ENSG00000137460	FHDC1	4:153857503-153900848	q1	q2	NOTEST	0.380995	0.463219	0.281921	0	1	1	no

ENSG00000137462	ENSG00000137462	TLR2	4:154622651-154626851	q1	q2	NOTEST	0.121847	0.118695	-0.0378016	0	1	1	no

ENSG00000137463	ENSG00000137463	MGARP	4:140187316-140397763	q1	q2	OK	0.600654	0.313589	-0.937661	-0.122891	0.62035	0.99995	no

ENSG00000137473	ENSG00000137473	TTC29	4:147627789-147867034	q1	q2	NOTEST	0.0348626	0.0503816	0.531217	0	1	1	no

ENSG00000137474	ENSG00000137474	MYO7A	11:76839309-76926284	q1	q2	NOTEST	0.0590695	0.0375757	-0.652613	0	1	1	no

ENSG00000137478	ENSG00000137478	FCHSD2	11:72525352-72853306	q1	q2	OK	10.4172	9.96671	-0.0637796	-0.0957396	0.8714	0.99995	no

ENSG00000137486	ENSG00000137486	ARRB1	11:74975225-75062873	q1	q2	OK	6.78517	6.12655	-0.147311	-0.207522	0.71715	0.99995	no

ENSG00000137491	ENSG00000137491	SLCO2B1	11:74810216-74917594	q1	q2	OK	0.605294	0.342673	-0.820802	-0.332249	0.21875	0.99995	no

ENSG00000137492	ENSG00000137492	PRKRIR	11:76060999-76092015	q1	q2	OK	47.2358	51.1622	0.115195	0.228124	0.6833	0.99995	no

ENSG00000137494	ENSG00000137494	ANKRD42	11:82868029-83134559	q1	q2	OK	5.04164	6.01444	0.254538	0.14308	0.80505	0.99995	no

ENSG00000137496	ENSG00000137496	IL18BP	11:71709586-71823826	q1	q2	OK	5.92518	5.41132	-0.130877	-0.0527925	0.91355	0.99995	no

ENSG00000137497	ENSG00000137497	NUMA1	11:71709586-71823826	q1	q2	OK	79.2764	69.4265	-0.191406	-0.270763	0.65375	0.99995	no

ENSG00000137500	ENSG00000137500	CCDC90B	11:82868029-83134559	q1	q2	OK	49.8095	56.289	0.176432	0.215806	0.74285	0.99995	no

ENSG00000137501	ENSG00000137501	SYTL2	11:85405266-85522184	q1	q2	OK	12.0007	11.6643	-0.0410224	-0.0525983	0.9297	0.99995	no

ENSG00000137502	ENSG00000137502	RAB30	11:82534543-82782965	q1	q2	OK	3.18383	3.31937	0.0601453	0.0366108	0.91485	0.99995	no

ENSG00000137504	ENSG00000137504	CREBZF	11:85370751-85393951	q1	q2	OK	51.9422	50.4741	-0.0413635	-0.089655	0.89075	0.99995	no

ENSG00000137507	ENSG00000137507	LRRC32	11:76368099-76381791	q1	q2	OK	0.748326	0.922892	0.302496	0.180639	0.6828	0.99995	no

ENSG00000137509	ENSG00000137509	PRCP	11:82534543-82782965	q1	q2	OK	24.996	24.3937	-0.0351866	-0.0557294	0.93015	0.99995	no

ENSG00000137513	ENSG00000137513	NARS2	11:77926342-78285919	q1	q2	OK	16.668	18.5718	0.156027	0.186187	0.76135	0.99995	no

ENSG00000137522	ENSG00000137522	RNF121	11:71639746-71708643	q1	q2	OK	20.4884	23.3071	0.185961	0.303431	0.654	0.99995	no

ENSG00000137547	ENSG00000137547	MRPL15	8:55047769-55060461	q1	q2	OK	49.4895	50.0301	0.0156748	0.0354402	0.9592	0.99995	no

ENSG00000137558	ENSG00000137558	PI15	8:75512009-75779174	q1	q2	OK	1.52237	1.15792	-0.394783	-0.456184	0.4513	0.99995	no

ENSG00000137561	ENSG00000137561	TTPA	8:63961111-63998612	q1	q2	NOTEST	0.170712	0.178396	0.0635165	0	1	1	no

ENSG00000137563	ENSG00000137563	GGH	8:63927637-63951730	q1	q2	OK	35.1456	41.8047	0.25032	0.496652	0.4522	0.99995	no

ENSG00000137571	ENSG00000137571	SLCO5A1	8:70579281-70767206	q1	q2	OK	0.944658	1.18035	0.321351	0.366682	0.4661	0.99995	no

ENSG00000137573	ENSG00000137573	SULF1	8:70378858-70573150	q1	q2	OK	7.1977	6.04613	-0.251524	-0.307911	0.50705	0.99995	no

ENSG00000137574	ENSG00000137574	TGS1	8:56608982-56738007	q1	q2	OK	19.8277	23.3795	0.237723	0.4473	0.52775	0.99995	no

ENSG00000137575	ENSG00000137575	SDCBP	8:59465482-59495419	q1	q2	OK	110.017	111.241	0.015971	0.0377788	0.9524	0.99995	no

ENSG00000137601	ENSG00000137601	NEK1	4:170314425-170533780	q1	q2	OK	5.46215	6.2244	0.188466	0.357465	0.5819	0.99995	no

ENSG00000137628	ENSG00000137628	DDX60	4:169137443-169239958	q1	q2	OK	0.687584	1.04611	0.605431	0.327344	0.4691	0.99995	no

ENSG00000137634	ENSG00000137634	NXPE4	11:114441312-114466484	q1	q2	NOTEST	0	0.0152005	inf	0	1	1	no

ENSG00000137642	ENSG00000137642	SORL1	11:121318039-121504402	q1	q2	OK	2.68075	2.47409	-0.115736	-0.16216	0.74185	0.99995	no

ENSG00000137648	ENSG00000137648	TMPRSS4	11:117886486-117992605	q1	q2	NOTEST	0	0	0	0	1	1	no

ENSG00000137656	ENSG00000137656	BUD13	11:116618885-116643704	q1	q2	OK	16.6064	18.747	0.174918	0.372936	0.5928	0.99995	no

ENSG00000137672	ENSG00000137672	TRPC6	11:101322294-101743293	q1	q2	NOTEST	0.248116	0.124066	-0.999907	0	1	1	no

ENSG00000137673	ENSG00000137673	MMP7	11:102391238-102401484	q1	q2	NOTEST	0	0.0250102	inf	0	1	1	no

ENSG00000137674	ENSG00000137674	MMP20	11:102447565-102498801	q1	q2	NOTEST	0	0	0	0	1	1	no

ENSG00000137675	ENSG00000137675	MMP27	11:102562217-102576537	q1	q2	NOTEST	0	0	0	0	1	1	no

ENSG00000137691	ENSG00000137691	C11orf70	11:101918173-101955291	q1	q2	OK	5.32688	4.72435	-0.173173	-0.194675	0.77525	0.99995	no

ENSG00000137692	ENSG00000137692	DCUN1D5	11:102932804-102962944	q1	q2	OK	28.9186	30.2652	0.0656601	0.138736	0.83765	0.99995	no

ENSG00000137693	ENSG00000137693	YAP1	11:101981191-102104154	q1	q2	OK	83.4635	89.8088	0.105712	0.219449	0.72175	0.99995	no

ENSG00000137699	ENSG00000137699	TRIM29	11:119981982-120056237	q1	q2	NOTEST	0.0603914	0	#NAME?	0	1	1	no

ENSG00000137700	ENSG00000137700	SLC37A4	11:118868851-118901616	q1	q2	NOTEST	0.0447813	0.00436111	-3.36013	0	1	1	no

ENSG00000137707	ENSG00000137707	BTG4	11:111338250-111383079	q1	q2	NOTEST	0	0	0	0	1	1	no

ENSG00000137709	ENSG00000137709	POU2F3	11:120107348-120190653	q1	q2	NOTEST	0	0	0	0	1	1	no

ENSG00000137710	ENSG00000137710	RDX	11:110045604-110167447	q1	q2	OK	81.7668	81.946	0.00315693	0.00531205	0.99375	0.99995	no

ENSG00000137713	ENSG00000137713	PPP2R1B	11:111473114-111637151	q1	q2	OK	9.95109	11.2476	0.176687	0.217201	0.6803	0.99995	no

ENSG00000137714	ENSG00000137714	FDX1	11:110300606-110335605	q1	q2	OK	7.74144	8.32136	0.104217	0.20541	0.77075	0.99995	no

ENSG00000137720	ENSG00000137720	C11orf1	11:111652918-111756699	q1	q2	NOTEST	0	0	0	0	1	1	no

ENSG00000137726	ENSG00000137726	FXYD6	11:117298488-117748201	q1	q2	OK	145.959	132.618	-0.138292	-0.307263	0.6391	0.99995	no

ENSG00000137727	ENSG00000137727	ARHGAP20	11:110447765-110583912	q1	q2	OK	1.1835	1.33764	0.176634	0.239117	0.7295	0.99995	no

ENSG00000137731	ENSG00000137731	FXYD2	11:117298488-117748201	q1	q2	NOTEST	0.438904	0.256819	-0.773154	0	1	1	no

ENSG00000137745	ENSG00000137745	MMP13	11:102813723-102826463	q1	q2	NOTEST	0	0	0	0	1	1	no

ENSG00000137747	ENSG00000137747	TMPRSS13	11:117771357-117800174	q1	q2	NOTEST	0.00815041	0	#NAME?	0	1	1	no

ENSG00000137752	ENSG00000137752	CASP1	11:104896169-104972158	q1	q2	NOTEST	0.026352	0.237657	3.1729	0	1	1	no

ENSG00000137757	ENSG00000137757	CASP5	11:104864961-104893895	q1	q2	NOTEST	0.0304474	0	#NAME?	0	1	1	no

ENSG00000137760	ENSG00000137760	ALKBH8	11:107373451-107436472	q1	q2	OK	3.88336	4.98561	0.360466	0.541516	0.34945	0.99995	no

ENSG00000137764	ENSG00000137764	MAP2K5	15:67835046-68099461	q1	q2	OK	8.69803	7.96746	-0.126568	-0.184604	0.75485	0.99995	no

ENSG00000137766	ENSG00000137766	UNC13C	15:54305100-54920806	q1	q2	NOTEST	0.0493597	0.270659	2.45507	0	1	1	no

ENSG00000137767	ENSG00000137767	SQRDL	15:45879320-45983492	q1	q2	OK	0.827263	0.879854	0.0889177	0.0168422	0.93535	0.99995	no

ENSG00000137770	ENSG00000137770	CTDSPL2	15:44719431-44829121	q1	q2	OK	26.6906	27.9503	0.0665327	0.101605	0.8557	0.99995	no

ENSG00000137776	ENSG00000137776	SLTM	15:59063390-59389618	q1	q2	OK	73.6775	82.0136	0.154639	0.204498	0.6899	0.99995	no

ENSG00000137801	ENSG00000137801	THBS1	15:39873279-39891667	q1	q2	OK	10.0702	9.52531	-0.0802551	-0.0745525	0.8156	0.99995	no

ENSG00000137802	ENSG00000137802	MAPKBP1	15:42066631-42120134	q1	q2	OK	11.1464	9.65708	-0.206917	-0.226698	0.57485	0.99995	no

ENSG00000137804	ENSG00000137804	NUSAP1	15:41624891-41673248	q1	q2	OK	101.867	103.812	0.0272865	0.0525144	0.9254	0.99995	no

ENSG00000137806	ENSG00000137806	NDUFAF1	15:41679550-41694717	q1	q2	OK	16.1585	16.2344	0.00675886	0.0113052	0.98765	0.99995	no

ENSG00000137807	ENSG00000137807	KIF23	15:69591285-69740764	q1	q2	OK	40.115	43.4744	0.116023	0.220199	0.71045	0.99995	no

ENSG00000137808	ENSG00000137808	RP11-809H16.2	15:69070873-69355083	q1	q2	NOTEST	0	0	0	0	1	1	no

ENSG00000137809	ENSG00000137809	ITGA11	15:68594049-68724501	q1	q2	OK	3.71498	2.94495	-0.335111	-0.398206	0.4666	0.99995	no

ENSG00000137812	ENSG00000137812	CASC5	15:40886217-40956540	q1	q2	OK	18.677	20.9259	0.164026	0.315577	0.5722	0.99995	no

ENSG00000137814	ENSG00000137814	HAUS2	15:42841007-42862192	q1	q2	OK	17.3184	18.8497	0.122234	0.177388	0.7217	0.99995	no

ENSG00000137815	ENSG00000137815	RTF1	15:41700605-41775761	q1	q2	OK	28.7793	28.5787	-0.010096	-0.0126092	0.9737	0.99995	no

ENSG00000137817	ENSG00000137817	PARP6	15:72533521-72671129	q1	q2	OK	35.8795	35.8341	-0.00182534	-0.00263854	0.99645	0.99995	no

ENSG00000137818	ENSG00000137818	RPLP1	15:69745122-69748255	q1	q2	OK	786.344	734.208	-0.0989714	-0.203955	0.7774	0.99995	no

ENSG00000137819	ENSG00000137819	PAQR5	15:69591285-69740764	q1	q2	NOTEST	0.0920564	0.100689	0.129317	0	1	1	no

ENSG00000137821	ENSG00000137821	LRRC49	15:71123862-71342414	q1	q2	OK	12.8885	13.7174	0.0899142	0.120733	0.83805	0.99995	no

ENSG00000137822	ENSG00000137822	TUBGCP4	15:43650369-43699293	q1	q2	OK	21.1626	22.9305	0.115746	0.145935	0.7634	0.99995	no

ENSG00000137824	ENSG00000137824	RMDN3	15:41028081-41048049	q1	q2	OK	15.8054	16.0888	0.0256449	0.0488152	0.93855	0.99995	no

ENSG00000137825	ENSG00000137825	ITPKA	15:41785590-41795747	q1	q2	OK	1.01972	0.869162	-0.230479	-0.200906	0.75195	0.99995	no

ENSG00000137831	ENSG00000137831	UACA	15:70946892-71055932	q1	q2	OK	13.9007	13.856	-0.0046419	-0.00648019	0.9875	0.99995	no

ENSG00000137834	ENSG00000137834	SMAD6	15:66994565-67074338	q1	q2	OK	1.29872	1.54708	0.252453	0.239696	0.69895	0.99995	no

ENSG00000137841	ENSG00000137841	PLCB2	15:40570376-40600136	q1	q2	OK	1.87616	2.12482	0.179555	0.144156	0.7776	0.99995	no

ENSG00000137842	ENSG00000137842	TMEM62	15:43398422-43513481	q1	q2	OK	6.16073	6.2393	0.0182822	0.016332	0.973	0.99995	no

ENSG00000137843	ENSG00000137843	PAK6	15:40453223-40569688	q1	q2	NOTEST	0.0860383	0.0734236	-0.228735	0	1	1	no

ENSG00000137845	ENSG00000137845	ADAM10	15:58887402-59042177	q1	q2	OK	38.6702	42.5197	0.136911	0.304132	0.6163	0.99995	no

ENSG00000137857	ENSG00000137857	DUOX1	15:45384847-45493373	q1	q2	OK	1.85419	2.11087	0.187051	0.0803916	0.82155	0.99995	no

ENSG00000137860	ENSG00000137860	SLC28A2	15:45543883-45571449	q1	q2	NOTEST	0	0.0517706	inf	0	1	1	no

ENSG00000137868	ENSG00000137868	STRA6	15:74471806-74628813	q1	q2	OK	4.57414	5.54612	0.27798	0.296862	0.5952	0.99995	no

ENSG00000137869	ENSG00000137869	CYP19A1	15:51329684-51630807	q1	q2	NOTEST	0.312591	0.0295816	-3.4015	0	1	1	no

ENSG00000137871	ENSG00000137871	ZNF280D	15:56922378-57210769	q1	q2	OK	11.2155	11.3267	0.0142229	0.0220161	0.9726	0.99995	no

ENSG00000137872	ENSG00000137872	SEMA6D	15:47476297-48066420	q1	q2	OK	7.98245	8.14884	0.0297639	0.0355068	0.9448	0.99995	no

ENSG00000137875	ENSG00000137875	BCL2L10	15:52401459-52404972	q1	q2	NOTEST	0.0464983	0.0469525	0.0140247	0	1	1	no

ENSG00000137876	ENSG00000137876	RSL24D1	15:55473003-55489265	q1	q2	OK	66.826	71.3225	0.0939491	0.230451	0.73775	0.99995	no

ENSG00000137877	ENSG00000137877	SPTBN5	15:42120282-42190773	q1	q2	OK	0.561235	0.356282	-0.655586	-0.190627	0.65965	0.99995	no

ENSG00000137878	ENSG00000137878	GCOM1	15:57884105-58074960	q1	q2	NOTEST	0.114738	0.0406872	-1.49569	0	1	1	no

ENSG00000137880	ENSG00000137880	GCHFR	15:41056217-41059906	q1	q2	OK	10.212	9.45124	-0.111689	-0.155039	0.834	0.99995	no

ENSG00000137936	ENSG00000137936	BCAR3	1:94027346-94312706	q1	q2	OK	10.9938	13.268	0.271256	0.493828	0.433	0.99995	no

ENSG00000137941	ENSG00000137941	TTLL7	1:84330710-84464833	q1	q2	OK	6.18664	5.34429	-0.211158	-0.246529	0.6391	0.99995	no

ENSG00000137942	ENSG00000137942	FNBP1L	1:93913687-94020218	q1	q2	OK	67.1898	73.3424	0.126404	0.270885	0.6557	0.99995	no

ENSG00000137944	ENSG00000137944	CCBL2	1:89401455-89458643	q1	q2	OK	27.6874	28.0833	0.0204816	0.0370686	0.95885	0.99995	no

ENSG00000137947	ENSG00000137947	GTF2B	1:89318614-89357627	q1	q2	OK	23.9372	23.8305	-0.00644952	-0.0126924	0.98415	0.99995	no

ENSG00000137948	ENSG00000137948	BRDT	1:92414927-92479983	q1	q2	NOTEST	0.0642445	0	#NAME?	0	1	1	no

ENSG00000137955	ENSG00000137955	RABGGTB	1:76190035-76260764	q1	q2	OK	37.1439	42.3389	0.188862	0.219396	0.70555	0.99995	no

ENSG00000137959	ENSG00000137959	IFI44L	1:79085606-79111830	q1	q2	OK	4.17806	3.81768	-0.130135	-0.128771	0.8261	0.99995	no

ENSG00000137960	ENSG00000137960	GIPC2	1:78444858-78604133	q1	q2	NOTEST	0.0270809	0.026133	-0.051404	0	1	1	no

ENSG00000137962	ENSG00000137962	ARHGAP29	1:94614543-94740624	q1	q2	OK	12.8943	19.4854	0.595653	1.35096	0.04505	0.99995	no

ENSG00000137965	ENSG00000137965	IFI44	1:79115480-79129763	q1	q2	OK	4.83076	4.51633	-0.0970987	-0.108859	0.8552	0.99995	no

ENSG00000137968	ENSG00000137968	SLC44A5	1:75667815-76076801	q1	q2	OK	9.25568	9.52172	0.0408838	0.0874975	0.90455	0.99995	no

ENSG00000137970	ENSG00000137970	RPL7P9	1:97144429-97145176	q1	q2	OK	148.431	167.842	0.177306	0.423843	0.5451	0.99995	no

ENSG00000137975	ENSG00000137975	CLCA2	1:86889768-86922241	q1	q2	NOTEST	0.0189459	0.012458	-0.604818	0	1	1	no

ENSG00000137976	ENSG00000137976	DNASE2B	1:84864214-84880701	q1	q2	NOTEST	0.0524517	0	#NAME?	0	1	1	no

ENSG00000137992	ENSG00000137992	DBT	1:100652474-100715390	q1	q2	OK	3.17336	3.97657	0.325513	0.477369	0.43635	0.99995	no

ENSG00000137996	ENSG00000137996	RTCA	1:100731762-100758325	q1	q2	OK	16.0796	17.676	0.136566	0.253924	0.7043	0.99995	no

ENSG00000138002	ENSG00000138002	IFT172	2:27665232-27712656	q1	q2	OK	11.838	9.46892	-0.322157	-0.326461	0.4036	0.99995	no

ENSG00000138018	ENSG00000138018	EPT1	2:26531040-26618759	q1	q2	OK	11.4541	14.4073	0.330926	0.668335	0.24295	0.99995	no

ENSG00000138028	ENSG00000138028	CGREF1	2:27309614-27341995	q1	q2	OK	0.505693	0.513489	0.0220699	0.0109719	0.98415	0.99995	no

ENSG00000138029	ENSG00000138029	HADHB	2:26413503-26513336	q1	q2	OK	35.6455	34.6976	-0.0388819	-0.051075	0.939	0.99995	no

ENSG00000138030	ENSG00000138030	KHK	2:27309614-27341995	q1	q2	OK	2.89466	2.62001	-0.143822	-0.138033	0.8313	0.99995	no

ENSG00000138031	ENSG00000138031	ADCY3	2:25012602-25142708	q1	q2	OK	10.7145	10.1857	-0.0730245	-0.0427053	0.9306	0.99995	no

ENSG00000138032	ENSG00000138032	PPM1B	2:44394763-44471523	q1	q2	OK	33.4846	35.2494	0.0740994	0.163521	0.80585	0.99995	no

ENSG00000138035	ENSG00000138035	PNPT1	2:55861399-55921045	q1	q2	OK	11.4708	13.1162	0.193373	0.349204	0.57035	0.99995	no

ENSG00000138036	ENSG00000138036	DYNC2LI1	2:44001177-44037149	q1	q2	OK	25.0757	22.2011	-0.175653	-0.311196	0.64145	0.99995	no

ENSG00000138039	ENSG00000138039	LHCGR	2:48756521-49003654	q1	q2	NOTEST	0	0.0124947	inf	0	1	1	no

ENSG00000138041	ENSG00000138041	SMEK2	2:55774427-55846015	q1	q2	OK	30.5712	32.9958	0.110107	0.226251	0.70525	0.99995	no

ENSG00000138050	ENSG00000138050	THUMPD2	2:39963199-40006407	q1	q2	OK	6.13118	7.0516	0.201786	0.278837	0.65075	0.99995	no

ENSG00000138061	ENSG00000138061	CYP1B1	2:38150329-38408997	q1	q2	OK	25.3946	29.8404	0.232745	0.459763	0.4907	0.99995	no

ENSG00000138068	ENSG00000138068	SULT6B1	2:37394962-37551951	q1	q2	NOTEST	0	0	0	0	1	1	no

ENSG00000138069	ENSG00000138069	RAB1A	2:65283499-65357240	q1	q2	OK	138.184	140.542	0.0244086	0.0473503	0.9455	0.99995	no

ENSG00000138071	ENSG00000138071	ACTR2	2:65454886-65498387	q1	q2	OK	136.586	156.191	0.193503	0.448203	0.52045	0.99995	no

ENSG00000138073	ENSG00000138073	PREB	2:27346681-27357543	q1	q2	OK	27.7238	24.7008	-0.166568	-0.342015	0.60115	0.99995	no

ENSG00000138074	ENSG00000138074	SLC5A6	2:27422454-27440046	q1	q2	OK	29.0551	29.6706	0.0302432	0.0421951	0.9389	0.99995	no

ENSG00000138075	ENSG00000138075	ABCG5	2:44039610-44066004	q1	q2	NOTEST	0.0349772	0.0340528	-0.0386434	0	1	1	no

ENSG00000138078	ENSG00000138078	PREPL	2:44502598-44589001	q1	q2	OK	26.9823	26.7365	-0.0132036	-0.0236412	0.96495	0.99995	no

ENSG00000138079	ENSG00000138079	SLC3A1	2:44502598-44589001	q1	q2	NOTEST	0.40083	0.133662	-1.5844	0	1	1	no

ENSG00000138080	ENSG00000138080	EMILIN1	2:27301434-27309271	q1	q2	OK	8.38085	7.69312	-0.123528	-0.228011	0.7376	0.99995	no

ENSG00000138081	ENSG00000138081	FBXO11	2:47922668-48134949	q1	q2	OK	49.4961	54.2369	0.131961	0.149598	0.83965	0.99995	no

ENSG00000138083	ENSG00000138083	SIX3	2:45167292-45173216	q1	q2	NOTEST	0	0.0100378	inf	0	1	1	no

ENSG00000138085	ENSG00000138085	ATRAID	2:27422454-27440046	q1	q2	OK	54.0595	46.931	-0.204006	-0.265808	0.65995	0.99995	no

ENSG00000138092	ENSG00000138092	CENPO	2:25012602-25142708	q1	q2	OK	31.8831	36.2338	0.184543	0.278672	0.6092	0.99995	no

ENSG00000138095	ENSG00000138095	LRPPRC	2:44113646-44223144	q1	q2	OK	37.3695	41.4593	0.149836	0.24299	0.5912	0.99995	no

ENSG00000138100	ENSG00000138100	TRIM54	2:27505259-27531313	q1	q2	NOTEST	0.0697892	0.0130285	-2.42134	0	1	1	no

ENSG00000138101	ENSG00000138101	DTNB	2:25600066-25896503	q1	q2	OK	7.24355	6.87665	-0.0749902	-0.104602	0.8446	0.99995	no

ENSG00000138107	ENSG00000138107	ACTR1A	10:104238985-104262482	q1	q2	OK	84.9882	80.0877	-0.0856814	-0.190444	0.7563	0.99995	no

ENSG00000138109	ENSG00000138109	CYP2C9	10:96698414-96749147	q1	q2	NOTEST	0.028816	0	#NAME?	0	1	1	no

ENSG00000138111	ENSG00000138111	TMEM180	10:104221148-104236802	q1	q2	OK	4.03355	3.39656	-0.247979	-0.234203	0.59875	0.99995	no

ENSG00000138115	ENSG00000138115	CYP2C8	10:96796529-96829254	q1	q2	NOTEST	0.0859457	0.170411	0.987518	0	1	1	no

ENSG00000138119	ENSG00000138119	MYOF	10:95066185-95242074	q1	q2	OK	3.80942	3.70604	-0.0396957	-0.0595993	0.9115	0.99995	no

ENSG00000138131	ENSG00000138131	LOXL4	10:100007446-100028007	q1	q2	NOTEST	0.035103	0.0342549	-0.0352812	0	1	1	no

ENSG00000138134	ENSG00000138134	STAMBPL1	10:90639490-90775542	q1	q2	OK	1.77119	2.10666	0.25024	0.12104	0.8747	0.99995	no

ENSG00000138135	ENSG00000138135	CH25H	10:90965693-90967071	q1	q2	OK	0.548361	0.667651	0.28397	0.235237	0.7279	0.99995	no

ENSG00000138136	ENSG00000138136	LBX1	10:102986732-103029905	q1	q2	NOTEST	0.214262	0.0496954	-2.10819	0	1	1	no

ENSG00000138138	ENSG00000138138	ATAD1	10:89511268-89605369	q1	q2	OK	25.6721	27.3238	0.0899585	0.203836	0.7634	0.99995	no

ENSG00000138152	ENSG00000138152	BTBD16	10:124030820-124097677	q1	q2	NOTEST	0	0.0615347	inf	0	1	1	no

ENSG00000138160	ENSG00000138160	KIF11	10:94353042-94415150	q1	q2	OK	38.6417	41.8202	0.114041	0.281217	0.6836	0.99995	no

ENSG00000138161	ENSG00000138161	CUZD1	10:124591664-124639157	q1	q2	NOTEST	0.0406345	0.0573041	0.495933	0	1	1	no

ENSG00000138162	ENSG00000138162	TACC2	10:123748688-124014060	q1	q2	OK	36.6434	36.4622	-0.0071526	-0.013137	0.9805	0.99995	no

ENSG00000138166	ENSG00000138166	DUSP5	10:112257595-112271302	q1	q2	OK	2.71595	3.37515	0.313494	0.448557	0.5089	0.99995	no

ENSG00000138172	ENSG00000138172	CALHM2	10:105206542-105222452	q1	q2	OK	1.92975	1.41477	-0.447843	-0.46139	0.4729	0.99995	no

ENSG00000138175	ENSG00000138175	ARL3	10:104433487-104474164	q1	q2	OK	20.3162	21.2295	0.0634458	0.150689	0.8298	0.99995	no

ENSG00000138180	ENSG00000138180	CEP55	10:95256388-95288849	q1	q2	OK	20.6474	24.4453	0.243591	0.546327	0.4129	0.99995	no

ENSG00000138182	ENSG00000138182	KIF20B	10:91461366-91534700	q1	q2	OK	12.9281	13.3107	0.0420829	0.0939119	0.8854	0.99995	no

ENSG00000138185	ENSG00000138185	ENTPD1	10:97471535-97849995	q1	q2	OK	6.50302	6.89128	0.0836631	0.10668	0.86325	0.99995	no

ENSG00000138190	ENSG00000138190	EXOC6	10:94590934-94819250	q1	q2	OK	11.0907	11.521	0.0549181	0.0779239	0.86925	0.99995	no

ENSG00000138193	ENSG00000138193	PLCE1	10:95753745-96122716	q1	q2	OK	1.76389	1.89709	0.105027	0.0723618	0.8628	0.99995	no

ENSG00000138207	ENSG00000138207	RBP4	10:95326421-95364237	q1	q2	NOTEST	0.110962	0.11359	0.0337742	0	1	1	no

ENSG00000138231	ENSG00000138231	DBR1	3:137879853-137893791	q1	q2	OK	14.4266	15.5647	0.109545	0.195404	0.7462	0.99995	no

ENSG00000138246	ENSG00000138246	DNAJC13	3:132136369-132257876	q1	q2	OK	15.6006	18.0579	0.211027	0.265301	0.46105	0.99995	no

ENSG00000138271	ENSG00000138271	GPR87	3:150803483-151176497	q1	q2	NOTEST	0.016855	0	#NAME?	0	1	1	no

ENSG00000138279	ENSG00000138279	ANXA7	10:75134858-75173834	q1	q2	OK	24.7616	23.124	-0.0987164	-0.198667	0.77785	0.99995	no

ENSG00000138286	ENSG00000138286	FAM149B1	10:74870216-75118617	q1	q2	OK	21.7508	23.2082	0.0935675	0.0702632	0.89515	0.99995	no

ENSG00000138293	ENSG00000138293	NCOA4	10:51565107-51590734	q1	q2	OK	6.86435	7.47012	0.122008	0.14939	0.8374	0.99995	no

ENSG00000138294	ENSG00000138294	MSMB	10:51549497-51562517	q1	q2	NOTEST	0	0	0	0	1	1	no

ENSG00000138297	ENSG00000138297	TIMM23	10:51592079-51623365	q1	q2	OK	1.9314	1.75629	-0.137114	-0.132918	0.84985	0.99995	no

ENSG00000138303	ENSG00000138303	ASCC1	10:73856277-73995618	q1	q2	OK	19.9166	20.3704	0.0325016	0.0426438	0.9411	0.99995	no

ENSG00000138308	ENSG00000138308	PLA2G12B	10:74694927-74714536	q1	q2	NOTEST	0.0255727	0	#NAME?	0	1	1	no

ENSG00000138311	ENSG00000138311	ZNF365	10:64133950-64431771	q1	q2	NOTEST	0.224372	0.18298	-0.294206	0	1	1	no

ENSG00000138315	ENSG00000138315	OIT3	10:74653338-74692791	q1	q2	NOTEST	0.0548026	0.0212538	-1.36652	0	1	1	no

ENSG00000138316	ENSG00000138316	ADAMTS14	10:72432558-72522197	q1	q2	OK	2.80016	2.53526	-0.143375	-0.219758	0.7568	0.99995	no

ENSG00000138326	ENSG00000138326	RPS24	10:79793517-79816570	q1	q2	OK	1687.04	1683.96	-0.00264036	-0.00608233	0.9924	0.99995	no

ENSG00000138336	ENSG00000138336	TET1	10:70320412-70454239	q1	q2	OK	7.29917	8.97284	0.297832	0.673165	0.3432	0.99995	no

ENSG00000138346	ENSG00000138346	DNA2	10:70173820-70231879	q1	q2	OK	16.5841	18.0969	0.125939	0.164239	0.75685	0.99995	no

ENSG00000138347	ENSG00000138347	MYPN	10:69865911-69971774	q1	q2	NOTEST	0.00977074	0.0373679	1.93526	0	1	1	no

ENSG00000138356	ENSG00000138356	AOX1	2:201450590-201541787	q1	q2	NOTEST	0.0910427	0.152073	0.74015	0	1	1	no

ENSG00000138363	ENSG00000138363	ATIC	2:216176539-216214487	q1	q2	OK	62.0905	66.0103	0.0883187	0.162018	0.80575	0.99995	no

ENSG00000138375	ENSG00000138375	SMARCAL1	2:217250010-217443903	q1	q2	OK	11.8938	12.3573	0.0551481	0.0289478	0.9759	0.99995	no

ENSG00000138376	ENSG00000138376	BARD1	2:215590369-215674428	q1	q2	OK	10.3938	11.6046	0.158976	0.202846	0.6904	0.99995	no

ENSG00000138378	ENSG00000138378	STAT4	2:191886251-192016322	q1	q2	OK	0.787067	0.575496	-0.451681	-0.290729	0.45185	0.99995	no

ENSG00000138379	ENSG00000138379	MSTN	2:190744334-191236391	q1	q2	NOTEST	0.316052	0.272055	-0.216266	0	1	1	no

ENSG00000138380	ENSG00000138380	CARF	2:203739504-203879521	q1	q2	OK	6.44936	5.97971	-0.109081	-0.107743	0.7984	0.99995	no

ENSG00000138381	ENSG00000138381	ASNSD1	2:190526110-190535557	q1	q2	OK	33.8133	34.7095	0.0377388	0.0695152	0.90275	0.99995	no

ENSG00000138382	ENSG00000138382	METTL5	2:170648442-170681441	q1	q2	OK	40.787	43.2914	0.0859695	0.0708467	0.91345	0.99995	no

ENSG00000138385	ENSG00000138385	SSB	2:170648442-170681441	q1	q2	OK	151.774	152.308	0.00507088	0.00964757	0.9862	0.99995	no

ENSG00000138386	ENSG00000138386	NAB1	2:191511471-191573442	q1	q2	OK	26.1282	28.6378	0.132314	0.243862	0.68485	0.99995	no

ENSG00000138395	ENSG00000138395	CDK15	2:202655183-202760273	q1	q2	NOTEST	0.325543	0.33596	0.0454413	0	1	1	no

ENSG00000138398	ENSG00000138398	PPIG	2:170440849-170497916	q1	q2	OK	49.214	53.6897	0.125577	0.248981	0.67855	0.99995	no

ENSG00000138399	ENSG00000138399	FASTKD1	2:170386258-170430385	q1	q2	OK	14.8414	14.6898	-0.0148133	-0.0220932	0.96595	0.99995	no

ENSG00000138400	ENSG00000138400	MDH1B	2:207602486-207657233	q1	q2	OK	1.64535	1.86657	0.182	0.0890738	0.88535	0.99995	no

ENSG00000138411	ENSG00000138411	HECW2	2:197059093-197458416	q1	q2	OK	1.24977	1.75156	0.486982	0.339241	0.35655	0.99995	no

ENSG00000138413	ENSG00000138413	IDH1	2:209100950-209130798	q1	q2	OK	169.528	180.724	0.0922635	0.210028	0.7561	0.99995	no

ENSG00000138430	ENSG00000138430	OLA1	2:174937174-175113426	q1	q2	OK	90.2242	95.9489	0.0887522	0.188912	0.779	0.99995	no

ENSG00000138433	ENSG00000138433	CIR1	2:175212749-175260443	q1	q2	OK	18.9221	18.7692	-0.011704	-0.0206391	0.9711	0.99995	no

ENSG00000138434	ENSG00000138434	SSFA2	2:182756559-182795465	q1	q2	OK	139.143	188.118	0.435071	0.657284	0.198	0.99995	no

ENSG00000138435	ENSG00000138435	CHRNA1	2:175411868-175639555	q1	q2	OK	0.422428	0.532562	0.334244	0.0609425	0.806	0.99995	no

ENSG00000138439	ENSG00000138439	FAM117B	2:203499900-203634480	q1	q2	OK	8.63676	8.29592	-0.0580883	-0.128126	0.8532	0.99995	no

ENSG00000138442	ENSG00000138442	WDR12	2:203739504-203879521	q1	q2	OK	24.1454	27.268	0.175462	0.13892	0.8544	0.99995	no

ENSG00000138443	ENSG00000138443	ABI2	2:204192941-204400133	q1	q2	OK	78.1894	86.0548	0.138283	0.197215	0.6564	0.99995	no

ENSG00000138448	ENSG00000138448	ITGAV	2:187454791-187630685	q1	q2	OK	26.1207	28.4097	0.121189	0.227323	0.68525	0.99995	no

ENSG00000138449	ENSG00000138449	SLC40A1	2:190425304-190448484	q1	q2	OK	1.70443	1.95401	0.197154	0.235499	0.6759	0.99995	no

ENSG00000138459	ENSG00000138459	SLC35A5	3:112251355-112304424	q1	q2	OK	30.0517	35.5378	0.241909	0.379865	0.53455	0.99995	no

ENSG00000138463	ENSG00000138463	DIRC2	3:122513641-122599986	q1	q2	OK	8.04086	7.14488	-0.170439	-0.294913	0.6751	0.99995	no

ENSG00000138468	ENSG00000138468	SENP7	3:101043048-101232085	q1	q2	OK	26.4779	29.6175	0.161663	0.286206	0.6642	0.99995	no

ENSG00000138472	ENSG00000138472	GUCA1C	3:108626638-108672742	q1	q2	NOTEST	0	0	0	0	1	1	no

ENSG00000138483	ENSG00000138483	CCDC54	3:106959538-107101007	q1	q2	NOTEST	0.132551	0	#NAME?	0	1	1	no

ENSG00000138495	ENSG00000138495	COX17	3:119355303-119396301	q1	q2	OK	23.7058	26.1985	0.144247	0.230822	0.7435	0.99995	no

ENSG00000138496	ENSG00000138496	PARP9	3:122246770-122294050	q1	q2	OK	1.02271	1.01662	-0.00860617	-0.00733653	0.99205	0.99995	no

ENSG00000138587	ENSG00000138587	MNS1	15:56536206-56757335	q1	q2	OK	1.76382	1.74339	-0.0168094	-0.0107338	0.98575	0.99995	no

ENSG00000138592	ENSG00000138592	USP8	15:50716576-50838905	q1	q2	OK	17.2161	18.1487	0.0761035	0.119819	0.80875	0.99995	no

ENSG00000138593	ENSG00000138593	SECISBP2L	15:49280672-49338760	q1	q2	OK	16.9878	18.1498	0.0954578	0.101988	0.7353	0.99995	no

ENSG00000138594	ENSG00000138594	TMOD3	15:52121824-52392720	q1	q2	OK	18.3637	20.6091	0.166425	0.139519	0.77015	0.99995	no

ENSG00000138600	ENSG00000138600	SPPL2A	15:50999505-51058005	q1	q2	OK	19.5469	20.0556	0.0370627	0.0534215	0.9322	0.99995	no

ENSG00000138604	ENSG00000138604	GLCE	15:69452922-69564556	q1	q2	OK	8.40829	8.6157	0.0351562	0.0515417	0.937	0.99995	no

ENSG00000138606	ENSG00000138606	SHF	15:45384847-45493373	q1	q2	OK	22.7977	21.056	-0.114656	-0.199628	0.76115	0.99995	no

ENSG00000138613	ENSG00000138613	APH1B	15:63568216-63601325	q1	q2	OK	8.09535	6.66056	-0.281449	-0.448954	0.3912	0.99995	no

ENSG00000138614	ENSG00000138614	VWA9	15:65871090-65903627	q1	q2	OK	31.3713	31.9027	0.0242323	0.0463019	0.93055	0.99995	no

ENSG00000138615	ENSG00000138615	CILP	15:65488336-65503826	q1	q2	OK	0.535327	0.38229	-0.485752	-0.49295	0.48955	0.99995	no

ENSG00000138617	ENSG00000138617	PARP16	15:65526797-65592956	q1	q2	OK	5.65634	5.92488	0.0669187	0.0958491	0.87865	0.99995	no

ENSG00000138621	ENSG00000138621	PPCDC	15:75315895-75409803	q1	q2	OK	9.01441	8.68393	-0.053885	-0.0685504	0.91685	0.99995	no

ENSG00000138622	ENSG00000138622	HCN4	15:73612199-73661605	q1	q2	OK	2.69373	2.55857	-0.0742706	-0.135849	0.84225	0.99995	no

ENSG00000138623	ENSG00000138623	SEMA7A	15:74701629-74726808	q1	q2	OK	4.61814	5.71268	0.306856	0.393971	0.48495	0.99995	no

ENSG00000138629	ENSG00000138629	UBL7	15:74738317-74753523	q1	q2	OK	27.2599	25.362	-0.104109	-0.197635	0.76715	0.99995	no

ENSG00000138639	ENSG00000138639	ARHGAP24	4:86396266-86923823	q1	q2	OK	3.28945	3.52043	0.0979068	0.109024	0.84315	0.99995	no

ENSG00000138640	ENSG00000138640	FAM13A	4:89630939-90032549	q1	q2	OK	7.67426	7.56084	-0.0214813	-0.0319378	0.95485	0.99995	no

ENSG00000138641	ENSG00000138641	HERC3	4:89442135-89629693	q1	q2	OK	2.69341	2.62227	-0.0386188	-0.0181134	0.95665	0.99995	no

ENSG00000138642	ENSG00000138642	HERC6	4:89299890-89364263	q1	q2	OK	0.524794	0.378401	-0.471837	-0.293631	0.5165	0.99995	no

ENSG00000138646	ENSG00000138646	HERC5	4:89378267-89427314	q1	q2	OK	0.98705	1.03025	0.0618007	0.0668234	0.9181	0.99995	no

ENSG00000138650	ENSG00000138650	PCDH10	4:134070469-134129356	q1	q2	OK	7.44397	6.19365	-0.265282	-0.244485	0.58775	0.99995	no

ENSG00000138653	ENSG00000138653	NDST4	4:115748918-116035032	q1	q2	OK	1.21266	1.39785	0.205037	0.246556	0.71065	0.99995	no

ENSG00000138658	ENSG00000138658	C4orf21	4:113434671-113578748	q1	q2	OK	13.5921	14.7253	0.115524	0.119612	0.8469	0.99995	no

ENSG00000138660	ENSG00000138660	AP1AR	4:113152892-113191203	q1	q2	OK	16.1158	19.5531	0.27892	0.474195	0.37725	0.99995	no

ENSG00000138663	ENSG00000138663	COPS4	4:83955599-83996971	q1	q2	OK	35.9832	38.5209	0.0983172	0.217787	0.73805	0.99995	no

ENSG00000138668	ENSG00000138668	HNRNPD	4:83273650-83305180	q1	q2	OK	514.746	556.71	0.113067	0.231255	0.73935	0.99995	no

ENSG00000138669	ENSG00000138669	PRKG2	4:82009836-82136218	q1	q2	NOTEST	0.12575	0.0546827	-1.2014	0	1	1	no

ENSG00000138670	ENSG00000138670	RASGEF1B	4:82347546-82965397	q1	q2	OK	1.19041	1.12069	-0.087073	-0.0885392	0.88095	0.99995	no

ENSG00000138674	ENSG00000138674	SEC31A	4:83739813-83934079	q1	q2	OK	72.7757	76.1995	0.0663244	0.0829389	0.87675	0.99995	no

ENSG00000138675	ENSG00000138675	FGF5	4:81187752-81884910	q1	q2	NOTEST	0.124536	0.120281	-0.0501493	0	1	1	no

ENSG00000138678	ENSG00000138678	AGPAT9	4:84457066-84527028	q1	q2	NOTEST	0.0580706	0.105034	0.854981	0	1	1	no

ENSG00000138684	ENSG00000138684	IL21	4:123533782-123610311	q1	q2	NOTEST	0	0	0	0	1	1	no

ENSG00000138685	ENSG00000138685	FGF2	4:123747862-123844123	q1	q2	OK	5.65112	6.227	0.14	0.27979	0.694	0.99995	no

ENSG00000138686	ENSG00000138686	BBS7	4:122745594-122791652	q1	q2	OK	10.5662	10.6917	0.0170412	0.0250132	0.9671	0.99995	no

ENSG00000138688	ENSG00000138688	KIAA1109	4:123073487-123283913	q1	q2	OK	22.9326	23.2273	0.0184218	0.0296262	0.95255	0.99995	no

ENSG00000138696	ENSG00000138696	BMPR1B	4:95679118-96079599	q1	q2	OK	7.62555	7.91239	0.0532728	0.093528	0.89385	0.99995	no

ENSG00000138698	ENSG00000138698	RAP1GDS1	4:99172838-99365012	q1	q2	OK	24.4418	25.8443	0.0804953	0.157448	0.78525	0.99995	no

ENSG00000138709	ENSG00000138709	LARP1B	4:128982422-129144086	q1	q2	OK	8.53376	8.6929	0.0266567	0.0461049	0.9442	0.99995	no

ENSG00000138722	ENSG00000138722	MMRN1	4:90800682-90875780	q1	q2	OK	13.4113	12.9064	-0.0553669	-0.105733	0.8691	0.99995	no

ENSG00000138735	ENSG00000138735	PDE5A	4:120375945-120550146	q1	q2	OK	5.77057	6.31055	0.129052	0.0883095	0.8355	0.99995	no

ENSG00000138738	ENSG00000138738	PRDM5	4:121606073-121844025	q1	q2	OK	3.61845	3.88667	0.103164	0.118267	0.8076	0.99995	no

ENSG00000138741	ENSG00000138741	TRPC3	4:122800181-122872909	q1	q2	OK	1.00174	0.913503	-0.133029	-0.153261	0.81015	0.99995	no

ENSG00000138744	ENSG00000138744	NAAA	4:76831808-76928641	q1	q2	OK	1.08754	1.26475	0.217775	0.0641836	0.80785	0.99995	no

ENSG00000138750	ENSG00000138750	NUP54	4:77035811-77069668	q1	q2	OK	35.0475	38.9625	0.152775	0.293795	0.60315	0.99995	no

ENSG00000138755	ENSG00000138755	CXCL9	4:76831808-76928641	q1	q2	NOTEST	0.0196985	0	#NAME?	0	1	1	no

ENSG00000138756	ENSG00000138756	BMP2K	4:79697495-79860592	q1	q2	OK	10.07	12.1787	0.274295	0.270856	0.58945	0.99995	no

ENSG00000138757	ENSG00000138757	G3BP2	4:76567965-76649709	q1	q2	OK	90.1158	94.0125	0.0610716	0.106418	0.8308	0.99995	no

ENSG00000138758	ENSG00000138758	Sep-11	4:77870855-77961537	q1	q2	OK	176.234	183.348	0.0570904	0.076665	0.8533	0.99995	no

ENSG00000138759	ENSG00000138759	FRAS1	4:78978723-79465423	q1	q2	OK	4.28766	5.20024	0.278389	0.507495	0.44045	0.99995	no

ENSG00000138760	ENSG00000138760	SCARB2	4:77079889-77135046	q1	q2	OK	43.1362	42.65	-0.0163528	-0.036201	0.9525	0.99995	no

ENSG00000138764	ENSG00000138764	CCNG2	4:78078303-78415440	q1	q2	OK	41.8212	38.7791	-0.108956	-0.204544	0.7615	0.99995	no

ENSG00000138767	ENSG00000138767	CNOT6L	4:78634540-78740769	q1	q2	OK	6.77089	6.88449	0.024005	0.0331782	0.94715	0.99995	no

ENSG00000138768	ENSG00000138768	USO1	4:76649776-76735382	q1	q2	OK	45.8661	49.409	0.107344	0.0940311	0.8196	0.99995	no

ENSG00000138769	ENSG00000138769	CDKL2	4:76503214-76555900	q1	q2	OK	0.489419	0.416927	-0.231274	-0.176124	0.7406	0.99995	no

ENSG00000138771	ENSG00000138771	SHROOM3	4:77356252-77723117	q1	q2	OK	19.7629	19.2595	-0.0372289	-0.0415944	0.9091	0.99995	no

ENSG00000138772	ENSG00000138772	ANXA3	4:79472672-79531597	q1	q2	NOTEST	0.271782	0.426482	0.650032	0	1	1	no

ENSG00000138777	ENSG00000138777	PPA2	4:106290233-106395238	q1	q2	OK	48.5647	55.1254	0.18281	0.375906	0.5932	0.99995	no

ENSG00000138778	ENSG00000138778	CENPE	4:104026962-104119566	q1	q2	OK	18.5077	20.3019	0.133491	0.144311	0.64395	0.99995	no

ENSG00000138780	ENSG00000138780	GSTCD	4:106473776-106943635	q1	q2	OK	11.743	12.9057	0.136207	0.217115	0.7154	0.99995	no

ENSG00000138785	ENSG00000138785	INTS12	4:106473776-106943635	q1	q2	OK	17.5861	16.4245	-0.098589	-0.114125	0.85325	0.99995	no

ENSG00000138792	ENSG00000138792	ENPEP	4:111286888-111486441	q1	q2	NOTEST	0.0363253	0.0969014	1.41554	0	1	1	no

ENSG00000138794	ENSG00000138794	CASP6	4:110481360-110624739	q1	q2	OK	16.6006	18.1147	0.125925	0.162573	0.81475	0.99995	no

ENSG00000138795	ENSG00000138795	LEF1	4:108968700-109177992	q1	q2	OK	10.2898	11.9763	0.218957	0.302576	0.64055	0.99995	no

ENSG00000138796	ENSG00000138796	HADH	4:108910869-108956331	q1	q2	OK	29.3982	28.6006	-0.0396863	-0.0755581	0.90235	0.99995	no

ENSG00000138798	ENSG00000138798	EGF	4:110834039-110933422	q1	q2	OK	0.899008	0.848819	-0.082877	-0.0475121	0.8996	0.99995	no

ENSG00000138801	ENSG00000138801	PAPSS1	4:108511432-108641608	q1	q2	OK	48.9833	51.2866	0.0662909	0.156742	0.8121	0.99995	no

ENSG00000138802	ENSG00000138802	SEC24B	4:110268630-110462052	q1	q2	OK	15.6581	15.8044	0.0134161	0.0307915	0.96435	0.99995	no

ENSG00000138813	ENSG00000138813	C4orf17	4:100432160-100463460	q1	q2	NOTEST	0	0	0	0	1	1	no

ENSG00000138814	ENSG00000138814	PPP3CA	4:101944565-102270040	q1	q2	OK	8.7945	8.8046	0.00165597	0.00293693	0.9964	0.99995	no

ENSG00000138821	ENSG00000138821	SLC39A8	4:103172197-103371167	q1	q2	OK	2.80818	2.56708	-0.129509	-0.168188	0.7739	0.99995	no

ENSG00000138823	ENSG00000138823	MTTP	4:100467865-100547070	q1	q2	OK	20.6211	19.5637	-0.0759438	-0.140529	0.8345	0.99995	no

ENSG00000138829	ENSG00000138829	FBN2	5:127593600-128369335	q1	q2	OK	32.5889	40.2504	0.30462	0.664254	0.297	0.99995	no

ENSG00000138834	ENSG00000138834	MAPK8IP3	16:1756183-1821731	q1	q2	OK	49.3193	45.8481	-0.105292	-0.134959	0.71965	0.99995	no

ENSG00000138835	ENSG00000138835	RGS3	9:116207010-116360018	q1	q2	OK	15.5444	17.1077	0.138252	0.223177	0.72635	0.99995	no

ENSG00000138867	ENSG00000138867	GUCD1	22:24936405-25024972	q1	q2	OK	39.823	38.2933	-0.056508	-0.0516066	0.9415	0.99995	no

ENSG00000138892	ENSG00000138892	TTLL8	22:50453551-50495364	q1	q2	NOTEST	0	0	0	0	1	1	no

ENSG00000138942	ENSG00000138942	RNF185	22:31556167-31603005	q1	q2	OK	20.1762	19.3726	-0.0586363	-0.128309	0.84875	0.99995	no

ENSG00000138944	ENSG00000138944	KIAA1644	22:44639546-44708731	q1	q2	OK	4.53357	3.77925	-0.262546	-0.514746	0.46045	0.99995	no

ENSG00000138964	ENSG00000138964	PARVG	22:44568835-44615413	q1	q2	NOTEST	0.0245254	0.0188404	-0.380447	0	1	1	no

ENSG00000139044	ENSG00000139044	B4GALNT3	12:569529-672675	q1	q2	OK	1.42974	1.44587	0.0161809	0.0139237	0.9807	0.99995	no

ENSG00000139053	ENSG00000139053	PDE6H	12:15125955-15134799	q1	q2	NOTEST	0.132647	0.0425773	-1.63944	0	1	1	no

ENSG00000139055	ENSG00000139055	ERP27	12:15066968-15092016	q1	q2	NOTEST	0.0316031	0.0242834	-0.380099	0	1	1	no

ENSG00000139083	ENSG00000139083	ETV6	12:11802787-12048336	q1	q2	OK	7.16384	7.44368	0.0552843	0.10478	0.86075	0.99995	no

ENSG00000139112	ENSG00000139112	GABARAPL1	12:10365056-10375727	q1	q2	OK	27.9188	24.0187	-0.217077	-0.457005	0.4905	0.99995	no

ENSG00000139116	ENSG00000139116	KIF21A	12:39687029-39837192	q1	q2	OK	52.4334	50.0966	-0.0657751	-0.117104	0.83975	0.99995	no

ENSG00000139117	ENSG00000139117	CPNE8	12:39040623-39303394	q1	q2	OK	2.24544	2.23751	-0.00510433	-0.00534817	0.99125	0.99995	no

ENSG00000139131	ENSG00000139131	YARS2	12:32832133-32908836	q1	q2	OK	15.968	15.9572	-0.000975759	-0.000538209	0.99915	0.99995	no

ENSG00000139132	ENSG00000139132	FGD4	12:32552462-32798984	q1	q2	OK	8.79218	9.55756	0.120421	0.25736	0.6758	0.99995	no

ENSG00000139133	ENSG00000139133	ALG10	12:34175215-34209675	q1	q2	OK	4.15948	5.02837	0.273687	0.390697	0.56255	0.99995	no

ENSG00000139144	ENSG00000139144	PIK3C2G	12:18233802-18801348	q1	q2	NOTEST	0	0	0	0	1	1	no

ENSG00000139146	ENSG00000139146	FAM60A	12:31433517-31479992	q1	q2	OK	74.5608	80.3251	0.107433	0.245234	0.7229	0.99995	no

ENSG00000139151	ENSG00000139151	PLCZ1	12:18836102-18890991	q1	q2	NOTEST	0	0	0	0	1	1	no

ENSG00000139154	ENSG00000139154	AEBP2	12:19556978-19873735	q1	q2	OK	14.8836	16.7165	0.167549	0.312394	0.64475	0.99995	no

ENSG00000139155	ENSG00000139155	SLCO1C1	12:20848288-20906320	q1	q2	OK	2.15802	1.03201	-1.06425	-0.867332	0.25695	0.99995	no

ENSG00000139160	ENSG00000139160	METTL20	12:31800093-31882108	q1	q2	OK	0.973487	0.94004	-0.0504397	-0.0306099	0.95245	0.99995	no

ENSG00000139163	ENSG00000139163	ETNK1	12:22778008-22843599	q1	q2	OK	26.348	33.2024	0.333596	0.403741	0.31975	0.99995	no

ENSG00000139168	ENSG00000139168	ZCRB1	12:42632248-42984157	q1	q2	OK	32.3622	38.7107	0.258421	0.305361	0.63325	0.99995	no

ENSG00000139173	ENSG00000139173	TMEM117	12:44229769-44783545	q1	q2	OK	4.67151	5.08065	0.121125	0.173184	0.78035	0.99995	no

ENSG00000139174	ENSG00000139174	PRICKLE1	12:42632248-42984157	q1	q2	OK	4.4531	4.49159	0.0124177	0.00726045	0.9899	0.99995	no

ENSG00000139178	ENSG00000139178	C1RL	12:7187512-7275097	q1	q2	OK	1.76827	1.31805	-0.423938	-0.251759	0.4562	0.99995	no

ENSG00000139180	ENSG00000139180	NDUFA9	12:4671369-4960277	q1	q2	OK	28.466	30.2288	0.0866853	0.103017	0.86685	0.99995	no

ENSG00000139182	ENSG00000139182	CLSTN3	12:7281674-7311541	q1	q2	OK	12.0403	11.7243	-0.038375	-0.0539689	0.9088	0.99995	no

ENSG00000139187	ENSG00000139187	KLRG1	12:9102639-9163356	q1	q2	OK	2.58917	3.54546	0.453484	0.44468	0.51825	0.99995	no

ENSG00000139190	ENSG00000139190	VAMP1	12:6548166-6580153	q1	q2	OK	4.30823	3.9549	-0.123452	-0.165133	0.7799	0.99995	no

ENSG00000139192	ENSG00000139192	TAPBPL	12:6548166-6580153	q1	q2	OK	0.652768	0.446455	-0.548057	-0.145709	0.62345	0.99995	no

ENSG00000139193	ENSG00000139193	CD27	12:6548166-6580153	q1	q2	NOTEST	0.190721	0.0669243	-1.51087	0	1	1	no

ENSG00000139194	ENSG00000139194	RBP5	12:7276279-7281538	q1	q2	NOTEST	0.284129	0.0993064	-1.51659	0	1	1	no

ENSG00000139197	ENSG00000139197	PEX5	12:7319269-7371170	q1	q2	OK	18.5144	17.9178	-0.0472537	-0.0865909	0.8701	0.99995	no

ENSG00000139200	ENSG00000139200	PIANP	12:6802957-6809966	q1	q2	OK	14.5516	13.1322	-0.148073	-0.284824	0.658	0.99995	no

ENSG00000139209	ENSG00000139209	SLC38A4	12:47158545-47226191	q1	q2	NOTEST	0.234858	0.266247	0.180976	0	1	1	no

ENSG00000139211	ENSG00000139211	AMIGO2	12:47469489-47630443	q1	q2	OK	2.36159	2.19328	-0.106664	-0.141201	0.84285	0.99995	no

ENSG00000139218	ENSG00000139218	SCAF11	12:46312913-46385903	q1	q2	OK	41.4936	46.9207	0.177335	0.243702	0.61005	0.99995	no

ENSG00000139219	ENSG00000139219	COL2A1	12:48366747-48398269	q1	q2	OK	57.7404	53.799	-0.102003	-0.135641	0.75335	0.99995	no

ENSG00000139220	ENSG00000139220	PPFIA2	12:81652044-82153332	q1	q2	OK	1.51058	1.25293	-0.269794	-0.189892	0.56925	0.99995	no

ENSG00000139223	ENSG00000139223	ANP32D	12:48866447-48866843	q1	q2	NOTEST	0	0	0	0	1	1	no

ENSG00000139233	ENSG00000139233	LLPH	12:66516841-66563852	q1	q2	OK	24.0126	26.6306	0.149296	0.221597	0.7559	0.99995	no

ENSG00000139239	ENSG00000139239	RPL14P1	12:63359094-63359764	q1	q2	OK	65.348	73.0276	0.160297	0.336573	0.6267	0.99995	no

ENSG00000139263	ENSG00000139263	LRIG3	12:59265930-59314303	q1	q2	OK	13.5577	14.7524	0.121834	0.172503	0.7412	0.99995	no

ENSG00000139266	ENSG00000139266	Mar-09	12:58117543-58154190	q1	q2	OK	16.8952	13.6185	-0.311048	-0.203324	0.7399	0.99995	no

ENSG00000139269	ENSG00000139269	INHBE	12:57846105-57853063	q1	q2	OK	0.966237	0.703431	-0.457968	-0.441332	0.4557	0.99995	no

ENSG00000139278	ENSG00000139278	GLIPR1	12:75874459-75905416	q1	q2	OK	1.27464	1.47367	0.209329	0.106326	0.82155	0.99995	no

ENSG00000139287	ENSG00000139287	TPH2	12:72332625-73059422	q1	q2	NOTEST	0.19497	0.144108	-0.436101	0	1	1	no

ENSG00000139289	ENSG00000139289	PHLDA1	12:76419226-76427712	q1	q2	OK	27.6033	33.5767	0.282619	0.684073	0.3324	0.99995	no

ENSG00000139291	ENSG00000139291	TMEM19	12:72003251-72097836	q1	q2	OK	13.0039	13.5928	0.0638974	0.0528066	0.902	0.99995	no

ENSG00000139292	ENSG00000139292	LGR5	12:71518864-71980090	q1	q2	OK	7.50215	8.16388	0.12195	0.201635	0.7207	0.99995	no

ENSG00000139304	ENSG00000139304	PTPRQ	12:80799773-81072802	q1	q2	NOTEST	0.115428	0.157378	0.447247	0	1	1	no

ENSG00000139318	ENSG00000139318	DUSP6	12:89741008-89747048	q1	q2	OK	166.29	233.108	0.487299	0.884774	0.2013	0.99995	no

ENSG00000139323	ENSG00000139323	POC1B	12:89813494-89934079	q1	q2	OK	9.10977	10.9766	0.268944	0.339411	0.5026	0.99995	no

ENSG00000139324	ENSG00000139324	TMTC3	12:88536072-88593664	q1	q2	OK	7.69086	8.44693	0.135282	0.286846	0.65525	0.99995	no

ENSG00000139329	ENSG00000139329	LUM	12:91496405-91505608	q1	q2	OK	177.501	201.012	0.179452	0.291569	0.67365	0.99995	no

ENSG00000139330	ENSG00000139330	KERA	12:91444267-91451760	q1	q2	OK	0.26806	0.485185	0.855982	0.683985	0.36055	0.99995	no

ENSG00000139343	ENSG00000139343	SNRPF	12:96252705-96336752	q1	q2	OK	60.1212	60.7853	0.0158498	0.0364917	0.9574	0.99995	no

ENSG00000139344	ENSG00000139344	AMDHD1	12:96337070-96362370	q1	q2	NOTEST	0.14287	0.176805	0.307452	0	1	1	no

ENSG00000139350	ENSG00000139350	NEDD1	12:97301000-97347129	q1	q2	OK	21.9383	24.3823	0.152384	0.323082	0.6074	0.99995	no

ENSG00000139351	ENSG00000139351	SYCP3	12:102090724-102137918	q1	q2	NOTEST	0.161784	0.347482	1.10286	0	1	1	no

ENSG00000139352	ENSG00000139352	ASCL1	12:103230662-103354294	q1	q2	OK	16.6418	14.5499	-0.193803	-0.412426	0.55655	0.99995	no

ENSG00000139354	ENSG00000139354	GAS2L3	12:100967460-101022064	q1	q2	OK	8.93787	9.34015	0.0635144	0.0826298	0.86685	0.99995	no

ENSG00000139364	ENSG00000139364	TMEM132B	12:125671381-126146917	q1	q2	OK	33.9577	31.1612	-0.123986	-0.163591	0.72685	0.99995	no

ENSG00000139370	ENSG00000139370	SLC15A4	12:129277738-129308528	q1	q2	OK	5.55062	5.85206	0.0762953	0.116873	0.8583	0.99995	no

ENSG00000139372	ENSG00000139372	TDG	12:104359581-104382652	q1	q2	OK	34.3943	38.4035	0.159069	0.336157	0.60545	0.99995	no

ENSG00000139405	ENSG00000139405	RITA1	12:113623330-113630173	q1	q2	OK	7.13933	6.75986	-0.0787955	-0.135384	0.8497	0.99995	no

ENSG00000139410	ENSG00000139410	SDSL	12:113830249-113876081	q1	q2	OK	3.18237	2.2209	-0.518956	-0.550814	0.3589	0.99995	no

ENSG00000139428	ENSG00000139428	MMAB	12:109991541-110035067	q1	q2	OK	33.8507	31.5459	-0.101734	-0.13175	0.8419	0.99995	no

ENSG00000139433	ENSG00000139433	GLTP	12:110288747-110318293	q1	q2	OK	13.193	13.2915	0.0107377	0.0211174	0.97605	0.99995	no

ENSG00000139436	ENSG00000139436	GIT2	12:110338068-110434194	q1	q2	OK	16.3386	17.3317	0.0851272	0.100032	0.81925	0.99995	no

ENSG00000139437	ENSG00000139437	TCHP	12:110338068-110434194	q1	q2	OK	13.5271	12.7426	-0.0861917	-0.110247	0.8642	0.99995	no

ENSG00000139438	ENSG00000139438	FAM222A	12:110152032-110211313	q1	q2	OK	7.46054	7.19896	-0.0514922	-0.0941183	0.8909	0.99995	no

ENSG00000139445	ENSG00000139445	FOXN4	12:109715783-109747025	q1	q2	OK	4.6706	5.16493	0.145143	0.215613	0.75145	0.99995	no

ENSG00000139496	ENSG00000139496	NUPL1	13:25875661-25923938	q1	q2	OK	32.7568	36.9529	0.173891	0.299849	0.55145	0.99995	no

ENSG00000139505	ENSG00000139505	MTMR6	13:25802306-25862147	q1	q2	OK	10.5318	12.6479	0.26415	0.613062	0.369	0.99995	no

ENSG00000139508	ENSG00000139508	SLC46A3	13:29274200-29293107	q1	q2	OK	0.987308	0.83286	-0.245425	-0.270247	0.6962	0.99995	no

ENSG00000139514	ENSG00000139514	SLC7A1	13:30083546-30169825	q1	q2	OK	15.1867	16.9051	0.154644	0.353982	0.58765	0.99995	no

ENSG00000139515	ENSG00000139515	PDX1	13:28407580-28500368	q1	q2	NOTEST	0	0	0	0	1	1	no

ENSG00000139517	ENSG00000139517	LNX2	13:28120049-28194541	q1	q2	OK	3.13859	3.29428	0.0698437	0.122104	0.8599	0.99995	no

ENSG00000139531	ENSG00000139531	SUOX	12:56390963-56400425	q1	q2	OK	15.8623	15.3103	-0.0510973	-0.088625	0.87605	0.99995	no

ENSG00000139537	ENSG00000139537	CCDC65	12:49297200-49351334	q1	q2	OK	2.16541	2.4467	0.176194	0.0377391	0.8856	0.99995	no

ENSG00000139540	ENSG00000139540	SLC39A5	12:56623832-56652175	q1	q2	NOTEST	0.345826	0.169162	-1.03164	0	1	1	no

ENSG00000139546	ENSG00000139546	TARBP2	12:53893945-53900215	q1	q2	OK	33.5029	29.6053	-0.17843	-0.317204	0.62635	0.99995	no

ENSG00000139547	ENSG00000139547	RDH16	12:57345218-57353158	q1	q2	OK	0.524114	0.431143	-0.281716	-0.279979	0.6944	0.99995	no

ENSG00000139549	ENSG00000139549	DHH	12:49483203-49488602	q1	q2	NOTEST	0.242833	0.113429	-1.09818	0	1	1	no

ENSG00000139567	ENSG00000139567	ACVRL1	12:52300691-52317145	q1	q2	OK	0.362888	0.596625	0.717298	0.282681	0.43065	0.99995	no

ENSG00000139572	ENSG00000139572	GPR84	12:54747444-54891472	q1	q2	NOTEST	0.0643857	0	#NAME?	0	1	1	no

ENSG00000139574	ENSG00000139574	NPFF	12:53900471-54020199	q1	q2	OK	1.41356	1.19787	-0.23886	-0.0614307	0.90875	0.99995	no

ENSG00000139579	ENSG00000139579	NABP2	12:56615798-56623638	q1	q2	OK	29.6358	27.0499	-0.13172	-0.273765	0.6834	0.99995	no

ENSG00000139597	ENSG00000139597	N4BP2L1	13:32974860-33112970	q1	q2	OK	2.90091	2.41078	-0.267004	-0.0996494	0.84045	0.99995	no

ENSG00000139610	ENSG00000139610	CELA1	12:51722226-51740463	q1	q2	NOTEST	0	0	0	0	1	1	no

ENSG00000139613	ENSG00000139613	SMARCC2	12:56544579-56584068	q1	q2	OK	55.1582	52.0956	-0.082414	-0.0593608	0.9107	0.99995	no

ENSG00000139618	ENSG00000139618	BRCA2	13:32889610-32973805	q1	q2	OK	9.4929	8.85915	-0.0996808	-0.0742135	0.8915	0.99995	no

ENSG00000139620	ENSG00000139620	KANSL2	12:49047183-49076021	q1	q2	OK	39.7113	40.395	0.0246266	0.0420241	0.9461	0.99995	no

ENSG00000139624	ENSG00000139624	CERS5	12:50505761-50561288	q1	q2	OK	43.5305	43.7433	0.00703816	0.0123337	0.98305	0.99995	no

ENSG00000139625	ENSG00000139625	MAP3K12	12:53835388-53893847	q1	q2	OK	24.2428	20.9151	-0.213011	-0.0834539	0.78925	0.99995	no

ENSG00000139626	ENSG00000139626	ITGB7	12:53585101-53601091	q1	q2	NOTEST	0.450253	0.343012	-0.392477	0	1	1	no

ENSG00000139629	ENSG00000139629	GALNT6	12:51745030-51902980	q1	q2	NOTEST	0.0841411	0.0526421	-0.676592	0	1	1	no

ENSG00000139631	ENSG00000139631	CSAD	12:53551446-53584596	q1	q2	OK	20.7782	19.7713	-0.0716653	-0.0570316	0.9192	0.99995	no

ENSG00000139636	ENSG00000139636	LMBR1L	12:49490918-49504683	q1	q2	OK	49.1898	51.8119	0.0749235	0.123253	0.85415	0.99995	no

ENSG00000139637	ENSG00000139637	C12orf10	12:53689074-53700961	q1	q2	OK	30.4715	27.6208	-0.141706	-0.139751	0.8154	0.99995	no

ENSG00000139641	ENSG00000139641	ESYT1	12:56511942-56538455	q1	q2	OK	29.586	29.3985	-0.00917133	-0.0129722	0.9764	0.99995	no

ENSG00000139644	ENSG00000139644	TMBIM6	12:49962000-50158717	q1	q2	OK	241.788	242.371	0.00347721	0.00705064	0.9921	0.99995	no

ENSG00000139645	ENSG00000139645	ANKRD52	12:56623832-56652175	q1	q2	OK	16.3176	16.3539	0.00320864	0.0062756	0.9898	0.99995	no

ENSG00000139648	ENSG00000139648	KRT71	12:52937692-52946931	q1	q2	NOTEST	0.0114913	0.0111437	-0.0443137	0	1	1	no

ENSG00000139651	ENSG00000139651	ZNF740	12:53551446-53584596	q1	q2	OK	22.5923	20.7723	-0.121165	-0.189025	0.7295	0.99995	no

ENSG00000139656	ENSG00000139656	SMIM2	13:44716681-44817328	q1	q2	NOTEST	0.0784762	0	#NAME?	0	1	1	no

ENSG00000139668	ENSG00000139668	WDFY2	13:52158643-52336171	q1	q2	OK	11.3171	12.1128	0.0980233	0.106411	0.82105	0.99995	no

ENSG00000139675	ENSG00000139675	HNRNPA1L2	13:53191604-53217919	q1	q2	OK	41.1269	43.074	0.0667332	0.150027	0.83515	0.99995	no

ENSG00000139679	ENSG00000139679	LPAR6	13:48877886-49056122	q1	q2	OK	15.0047	15.8493	0.0790024	0.0632664	0.9038	0.99995	no

ENSG00000139684	ENSG00000139684	ESD	13:47345390-47371367	q1	q2	OK	86.1012	89.6472	0.0582253	0.138323	0.84175	0.99995	no

ENSG00000139687	ENSG00000139687	RB1	13:48877886-49056122	q1	q2	OK	32.7483	38.3753	0.228758	0.427828	0.50715	0.99995	no

ENSG00000139697	ENSG00000139697	SBNO1	12:123773655-123850660	q1	q2	OK	13.2119	13.9335	0.0767241	0.169849	0.80895	0.99995	no

ENSG00000139714	ENSG00000139714	MORN3	12:122089023-122110537	q1	q2	NOTEST	0.178705	0.20917	0.227099	0	1	1	no

ENSG00000139718	ENSG00000139718	SETD1B	12:122242085-122270562	q1	q2	OK	6.65364	6.24494	-0.0914562	-0.205704	0.768	0.99995	no

ENSG00000139719	ENSG00000139719	VPS33A	12:122688089-122751068	q1	q2	OK	12.797	14.6902	0.19905	0.24065	0.6999	0.99995	no

ENSG00000139722	ENSG00000139722	VPS37B	12:123349881-123380991	q1	q2	OK	21.6727	19.04	-0.186846	-0.426082	0.5373	0.99995	no

ENSG00000139725	ENSG00000139725	RHOF	12:122150657-122241812	q1	q2	OK	6.98586	7.54798	0.111653	0.0833961	0.9056	0.99995	no

ENSG00000139726	ENSG00000139726	DENR	12:123237320-123255611	q1	q2	OK	48.9801	51.3113	0.06708	0.144756	0.81545	0.99995	no

ENSG00000139734	ENSG00000139734	DIAPH3	13:60239716-60738121	q1	q2	OK	5.23951	5.60418	0.0970718	0.183872	0.78745	0.99995	no

ENSG00000139737	ENSG00000139737	SLAIN1	13:78272022-78338377	q1	q2	OK	39.6666	43.4344	0.130915	0.258882	0.6973	0.99995	no

ENSG00000139746	ENSG00000139746	RBM26	13:79885961-79998463	q1	q2	OK	42.5102	46.7979	0.138635	0.33112	0.63335	0.99995	no

ENSG00000139767	ENSG00000139767	SRRM4	12:119419299-119600856	q1	q2	OK	4.72012	4.73318	0.00398499	0.00779152	0.9917	0.99995	no

ENSG00000139780	ENSG00000139780	METTL21C	13:103338093-103346854	q1	q2	NOTEST	0.0564288	0	#NAME?	0	1	1	no

ENSG00000139793	ENSG00000139793	MBNL2	13:97873687-98046374	q1	q2	OK	5.91039	5.3606	-0.140857	-0.149962	0.77455	0.99995	no

ENSG00000139797	ENSG00000139797	RNF113B	13:98794815-99102027	q1	q2	NOTEST	0.0201792	0	#NAME?	0	1	1	no

ENSG00000139800	ENSG00000139800	ZIC5	13:100615217-100624163	q1	q2	OK	1.14491	1.30946	0.193731	0.259091	0.71335	0.99995	no

ENSG00000139826	ENSG00000139826	ABHD13	13:108870726-108886603	q1	q2	OK	6.14088	6.98549	0.185916	0.394329	0.58055	0.99995	no

ENSG00000139832	ENSG00000139832	RAB20	13:111175416-111214080	q1	q2	OK	0.514613	0.561009	0.124536	0.119619	0.86935	0.99995	no

ENSG00000139835	ENSG00000139835	GRTP1	13:113978504-114018446	q1	q2	OK	3.58533	3.64435	0.0235582	0.0280065	0.9666	0.99995	no

ENSG00000139842	ENSG00000139842	CUL4A	13:113831890-113919399	q1	q2	OK	39.6567	38.3356	-0.0488808	-0.0804295	0.8848	0.99995	no

ENSG00000139865	ENSG00000139865	TTC6	14:38059188-38672128	q1	q2	NOTEST	0.0359997	0.0073306	-2.29598	0	1	1	no

ENSG00000139874	ENSG00000139874	SSTR1	14:38677203-38682272	q1	q2	NOTEST	0.0122146	0.00602854	-1.01873	0	1	1	no

ENSG00000139880	ENSG00000139880	CDH24	14:23516270-23526747	q1	q2	OK	15.1581	13.8094	-0.134441	-0.279899	0.6946	0.99995	no

ENSG00000139890	ENSG00000139890	REM2	14:23352373-23356895	q1	q2	OK	2.18485	1.67004	-0.387654	-0.467096	0.52325	0.99995	no

ENSG00000139899	ENSG00000139899	CBLN3	14:24895737-24912111	q1	q2	OK	0.8725	0.660378	-0.401863	-0.0832081	0.8137	0.99995	no

ENSG00000139908	ENSG00000139908	TSSK4	14:24641061-24708448	q1	q2	OK	4.10099	4.94839	0.270987	0.0797847	0.81045	0.99995	no

ENSG00000139910	ENSG00000139910	NOVA1	14:26912298-27066960	q1	q2	OK	24.53	23.5498	-0.0588351	-0.0982227	0.86615	0.99995	no

ENSG00000139914	ENSG00000139914	FITM1	14:24549315-24602058	q1	q2	NOTEST	0.123163	0.328503	1.41533	0	1	1	no

ENSG00000139915	ENSG00000139915	MDGA2	14:47308825-48144157	q1	q2	OK	1.72131	1.9958	0.213467	0.289709	0.6202	0.99995	no

ENSG00000139921	ENSG00000139921	TMX1	14:51706879-51722759	q1	q2	OK	49.4226	52.9753	0.100149	0.237982	0.72275	0.99995	no

ENSG00000139926	ENSG00000139926	FRMD6	14:51921229-52197445	q1	q2	OK	12.1329	12.7111	0.0671706	0.123375	0.8241	0.99995	no

ENSG00000139946	ENSG00000139946	PELI2	14:56584531-56768244	q1	q2	OK	6.61746	6.60725	-0.00222793	-0.00472108	0.9939	0.99995	no

ENSG00000139970	ENSG00000139970	RTN1	14:60062693-60337684	q1	q2	OK	12.1142	11.4154	-0.085712	-0.149746	0.82615	0.99995	no

ENSG00000139971	ENSG00000139971	C14orf37	14:58466452-58764857	q1	q2	OK	14.0465	12.2073	-0.202466	-0.133723	0.7797	0.99995	no

ENSG00000139973	ENSG00000139973	SYT16	14:62453802-62568431	q1	q2	NOTEST	0.100115	0.222476	1.15199	0	1	1	no

ENSG00000139974	ENSG00000139974	SLC38A6	14:61201459-61550451	q1	q2	OK	5.12484	5.35451	0.063246	0.0353551	0.93585	0.99995	no

ENSG00000139977	ENSG00000139977	NAA30	14:57857261-57882635	q1	q2	OK	8.72812	9.66645	0.147315	0.298913	0.6632	0.99995	no

ENSG00000139985	ENSG00000139985	ADAM21	14:70892528-71014181	q1	q2	NOTEST	0.175033	0.116975	-0.581422	0	1	1	no

ENSG00000139988	ENSG00000139988	RDH12	14:68168602-68283307	q1	q2	NOTEST	0.0934474	0.114162	0.288859	0	1	1	no

ENSG00000139990	ENSG00000139990	DCAF5	14:69517597-69619867	q1	q2	OK	24.714	25.6039	0.0510359	0.103041	0.8577	0.99995	no

ENSG00000139998	ENSG00000139998	RAB15	14:65381078-65569413	q1	q2	OK	18.1212	17.5328	-0.0476182	-0.0464414	0.9274	0.99995	no

ENSG00000140006	ENSG00000140006	WDR89	14:64063756-64108579	q1	q2	OK	10.1529	11.2563	0.148838	0.260888	0.70495	0.99995	no

ENSG00000140009	ENSG00000140009	ESR2	14:64319682-64805317	q1	q2	NOTEST	0.343244	0.338552	-0.0198545	0	1	1	no

ENSG00000140015	ENSG00000140015	KCNH5	14:63173286-63568755	q1	q2	NOTEST	0.0835414	0.0301066	-1.47241	0	1	1	no

ENSG00000140022	ENSG00000140022	STON2	14:81726999-81902809	q1	q2	OK	7.34446	9.35858	0.349635	0.379472	0.5704	0.99995	no

ENSG00000140025	ENSG00000140025	EFCAB11	14:89591214-90421121	q1	q2	OK	12.2064	10.7732	-0.180188	-0.104974	0.8814	0.99995	no

ENSG00000140030	ENSG00000140030	GPR65	14:88471467-88481955	q1	q2	NOTEST	0	0.0145282	inf	0	1	1	no

ENSG00000140043	ENSG00000140043	PTGR2	14:74318546-74551196	q1	q2	OK	2.91563	3.43756	0.237574	0.119892	0.7851	0.99995	no

ENSG00000140044	ENSG00000140044	JDP2	14:75890385-75940814	q1	q2	OK	6.8727	5.89807	-0.220635	-0.290454	0.65715	0.99995	no

ENSG00000140057	ENSG00000140057	AK7	14:96858447-96955764	q1	q2	OK	0.572102	0.719534	0.330788	0.258142	0.66505	0.99995	no

ENSG00000140067	ENSG00000140067	FAM181A	14:94371075-94395954	q1	q2	OK	23.9044	19.6226	-0.284761	-0.510398	0.4696	0.99995	no

ENSG00000140090	ENSG00000140090	SLC24A4	14:92788924-92962596	q1	q2	NOTEST	0.0241255	0.0356895	0.564942	0	1	1	no

ENSG00000140092	ENSG00000140092	FBLN5	14:92335755-92414331	q1	q2	OK	11.1877	9.07983	-0.301174	-0.437074	0.4509	0.99995	no

ENSG00000140093	ENSG00000140093	SERPINA10	14:94749649-94759608	q1	q2	NOTEST	0.0278038	0.0264881	-0.069935	0	1	1	no

ENSG00000140104	ENSG00000140104	C14orf79	14:105452111-105487485	q1	q2	OK	6.21821	5.21822	-0.252943	-0.23929	0.73625	0.99995	no

ENSG00000140105	ENSG00000140105	WARS	14:100800124-100996640	q1	q2	OK	66.5788	72.454	0.122001	0.191369	0.74575	0.99995	no

ENSG00000140107	ENSG00000140107	SLC25A47	14:100789673-100796715	q1	q2	NOTEST	0.0360667	0.0341173	-0.0801659	0	1	1	no

ENSG00000140153	ENSG00000140153	WDR20	14:102547074-102771537	q1	q2	OK	13.8619	13.5484	-0.033004	-0.00687	0.9692	0.99995	no

ENSG00000140157	ENSG00000140157	NIPA2	15:22892004-23034427	q1	q2	OK	32.2375	33.0742	0.0369703	0.042049	0.94395	0.99995	no

ENSG00000140181	ENSG00000140181	HERC2P2	15:23282280-23378228	q1	q2	OK	22.3393	21.2271	-0.0736733	-0.113456	0.8146	0.99995	no

ENSG00000140199	ENSG00000140199	SLC12A6	15:34525459-34630261	q1	q2	OK	5.26823	3.82116	-0.463305	-0.518068	0.25285	0.99995	no

ENSG00000140254	ENSG00000140254	DUOXA1	15:45384847-45493373	q1	q2	NOTEST	0.473635	0.266537	-0.829439	0	1	1	no

ENSG00000140259	ENSG00000140259	MFAP1	15:44096689-44117000	q1	q2	OK	37.1254	38.6765	0.0590504	0.139993	0.8392	0.99995	no

ENSG00000140262	ENSG00000140262	TCF12	15:57210820-57591479	q1	q2	OK	197.035	200.109	0.022331	0.0405765	0.94395	0.99995	no

ENSG00000140263	ENSG00000140263	SORD	15:45315301-45369383	q1	q2	OK	13.8268	14.0015	0.0181158	0.0302033	0.96495	0.99995	no

ENSG00000140264	ENSG00000140264	SERF2	15:44019115-44095241	q1	q2	OK	383.294	354.396	-0.113091	-0.112813	0.88185	0.99995	no

ENSG00000140265	ENSG00000140265	ZSCAN29	15:43650369-43699293	q1	q2	OK	23.5161	24.4651	0.0570761	0.061404	0.89545	0.99995	no

ENSG00000140274	ENSG00000140274	DUOXA2	15:45384847-45493373	q1	q2	NOTEST	0	0.0151236	inf	0	1	1	no

ENSG00000140279	ENSG00000140279	DUOX2	15:45384847-45493373	q1	q2	NOTEST	0.0462501	0.0147102	-1.65264	0	1	1	no

ENSG00000140280	ENSG00000140280	LYSMD2	15:52015207-52108565	q1	q2	OK	1.43717	1.09941	-0.386507	-0.147727	0.79795	0.99995	no

ENSG00000140284	ENSG00000140284	SLC27A2	15:50150434-50528592	q1	q2	NOTEST	0.164319	0.21255	0.371309	0	1	1	no

ENSG00000140285	ENSG00000140285	FGF7	15:49398267-49913128	q1	q2	NOTEST	0.131228	0.0943348	-0.476209	0	1	1	no

ENSG00000140287	ENSG00000140287	HDC	15:50534143-50558223	q1	q2	NOTEST	0.0535212	0.0124708	-2.10156	0	1	1	no

ENSG00000140297	ENSG00000140297	GCNT3	15:59887073-59981733	q1	q2	NOTEST	0.0596536	0.0584193	-0.0301634	0	1	1	no

ENSG00000140299	ENSG00000140299	BNIP2	15:59887073-59981733	q1	q2	OK	30.8994	33.81	0.129872	0.16364	0.77415	0.99995	no

ENSG00000140307	ENSG00000140307	GTF2A2	15:59887073-59981733	q1	q2	OK	78.7532	90.0192	0.192894	0.238949	0.7278	0.99995	no

ENSG00000140319	ENSG00000140319	SRP14	15:40327939-40331389	q1	q2	OK	267.987	272.347	0.0232798	0.0556858	0.93795	0.99995	no

ENSG00000140320	ENSG00000140320	BAHD1	15:40731919-40760441	q1	q2	OK	17.4496	17.9636	0.0418769	0.0646593	0.9062	0.99995	no

ENSG00000140323	ENSG00000140323	DISP2	15:40650435-40663257	q1	q2	OK	2.11751	1.38222	-0.615381	-0.427836	0.319	0.99995	no

ENSG00000140326	ENSG00000140326	CDAN1	15:43015756-43029324	q1	q2	OK	9.81287	9.3128	-0.0754603	-0.107165	0.8432	0.99995	no

ENSG00000140332	ENSG00000140332	TLE3	15:70340128-70390515	q1	q2	OK	12.502	14.1648	0.18015	0.156128	0.7107	0.99995	no

ENSG00000140350	ENSG00000140350	ANP32A	15:69070873-69355083	q1	q2	OK	273.042	299.667	0.134239	0.249072	0.72255	0.99995	no

ENSG00000140365	ENSG00000140365	COMMD4	15:75628231-75634268	q1	q2	OK	61.165	60.6905	-0.0112358	-0.0227652	0.97525	0.99995	no

ENSG00000140367	ENSG00000140367	UBE2Q2	15:76135621-76193419	q1	q2	OK	21.9759	23.8486	0.117983	0.261348	0.69455	0.99995	no

ENSG00000140368	ENSG00000140368	PSTPIP1	15:77285699-77329673	q1	q2	NOTEST	0.165886	0.016678	-3.31417	0	1	1	no

ENSG00000140374	ENSG00000140374	ETFA	15:76352177-76603813	q1	q2	OK	68.8916	71.0554	0.0446174	0.0952338	0.8919	0.99995	no

ENSG00000140379	ENSG00000140379	BCL2A1	15:80253230-80263788	q1	q2	NOTEST	0	0.0381778	inf	0	1	1	no

ENSG00000140382	ENSG00000140382	HMG20A	15:77712753-77777949	q1	q2	OK	29.1284	30.8243	0.0816443	0.145825	0.7831	0.99995	no

ENSG00000140386	ENSG00000140386	SCAPER	15:76640525-77197785	q1	q2	OK	5.52154	6.99752	0.341772	0.355191	0.40945	0.99995	no

ENSG00000140391	ENSG00000140391	TSPAN3	15:77336021-77376326	q1	q2	OK	176.045	167.896	-0.068382	-0.157947	0.81825	0.99995	no

ENSG00000140395	ENSG00000140395	WDR61	15:78542843-78592136	q1	q2	OK	39.6389	40.4244	0.0283116	0.0582421	0.9346	0.99995	no

ENSG00000140396	ENSG00000140396	NCOA2	8:71021996-71316040	q1	q2	OK	15.3507	15.6846	0.0310409	0.0379205	0.9145	0.99995	no

ENSG00000140398	ENSG00000140398	NEIL1	15:75639295-75648087	q1	q2	OK	7.44078	5.80067	-0.359234	-0.379689	0.50875	0.99995	no

ENSG00000140400	ENSG00000140400	MAN2C1	15:75648132-75748183	q1	q2	OK	85.2545	75.2999	-0.179127	-0.203764	0.66885	0.99995	no

ENSG00000140403	ENSG00000140403	DNAJA4	15:78542843-78592136	q1	q2	OK	0.76594	0.6848	-0.161548	-0.0599874	0.8949	0.99995	no

ENSG00000140406	ENSG00000140406	MESDC1	15:81293294-81296342	q1	q2	OK	14.7304	14.4789	-0.0248392	-0.0546522	0.93975	0.99995	no

ENSG00000140416	ENSG00000140416	TPM1	15:63334830-63364114	q1	q2	OK	239.578	256.352	0.0976316	0.183763	0.76785	0.99995	no

ENSG00000140443	ENSG00000140443	IGF1R	15:99192199-99675798	q1	q2	OK	36.6709	38.001	0.0513989	0.0733535	0.856	0.99995	no

ENSG00000140450	ENSG00000140450	ARRDC4	15:98462783-98517068	q1	q2	OK	2.97146	3.10054	0.0613477	0.101196	0.88635	0.99995	no

ENSG00000140451	ENSG00000140451	PIF1	15:65107830-65117867	q1	q2	OK	22.2835	21.1369	-0.0762107	-0.0912674	0.86955	0.99995	no

ENSG00000140455	ENSG00000140455	USP3	15:63796792-63894627	q1	q2	OK	34.7232	34.5564	-0.00694567	-0.0121032	0.9831	0.99995	no

ENSG00000140459	ENSG00000140459	CYP11A1	15:74630099-74660081	q1	q2	NOTEST	0.105044	0.0136926	-2.93953	0	1	1	no

ENSG00000140463	ENSG00000140463	BBS4	15:72978526-73030817	q1	q2	OK	22.1623	21.6376	-0.0345692	-0.0611041	0.9174	0.99995	no

ENSG00000140464	ENSG00000140464	PML	15:74287013-74340153	q1	q2	OK	9.54525	9.18039	-0.0562267	-0.0818607	0.8783	0.99995	no

ENSG00000140465	ENSG00000140465	CYP1A1	15:75011882-75017951	q1	q2	NOTEST	0.323558	0.210273	-0.62176	0	1	1	no

ENSG00000140470	ENSG00000140470	ADAMTS17	15:100510419-100882210	q1	q2	NOTEST	0.367485	0.364804	-0.0105655	0	1	1	no

ENSG00000140471	ENSG00000140471	LINS	15:101093733-101191910	q1	q2	OK	10.0361	9.29293	-0.110999	-0.122056	0.80965	0.99995	no

ENSG00000140474	ENSG00000140474	ULK3	15:75128456-75135687	q1	q2	OK	22.5548	18.696	-0.270704	-0.449476	0.4547	0.99995	no

ENSG00000140478	ENSG00000140478	GOLGA6D	15:75575175-75586816	q1	q2	NOTEST	0	0	0	0	1	1	no

ENSG00000140479	ENSG00000140479	PCSK6	15:101835623-102065405	q1	q2	OK	4.52994	4.96838	0.133284	0.105507	0.78935	0.99995	no

ENSG00000140481	ENSG00000140481	CCDC33	15:74471806-74628813	q1	q2	NOTEST	0.0409545	0.0523005	0.352805	0	1	1	no

ENSG00000140488	ENSG00000140488	CELF6	15:72533521-72671129	q1	q2	OK	0.728115	0.572291	-0.347419	-0.0948498	0.82195	0.99995	no

ENSG00000140497	ENSG00000140497	SCAMP2	15:75136070-75165706	q1	q2	OK	17.3396	15.0054	-0.208589	-0.367234	0.56705	0.99995	no

ENSG00000140505	ENSG00000140505	CYP1A2	15:75041184-75048543	q1	q2	NOTEST	0	0.00909561	inf	0	1	1	no

ENSG00000140506	ENSG00000140506	LMAN1L	15:75105056-75124141	q1	q2	NOTEST	0.0241039	0.0651259	1.43396	0	1	1	no

ENSG00000140511	ENSG00000140511	HAPLN3	15:89420518-89438857	q1	q2	OK	2.43288	2.79895	0.202221	0.242698	0.71025	0.99995	no

ENSG00000140519	ENSG00000140519	RHCG	15:89998679-90039844	q1	q2	NOTEST	0.0723061	0.0586825	-0.301187	0	1	1	no

ENSG00000140521	ENSG00000140521	POLG	15:89787179-89878092	q1	q2	OK	27.918	27.6447	-0.0141888	-0.0107141	0.9821	0.99995	no

ENSG00000140522	ENSG00000140522	RLBP1	15:89753099-89764982	q1	q2	NOTEST	0.178902	0.174298	-0.0376176	0	1	1	no

ENSG00000140525	ENSG00000140525	FANCI	15:89787179-89878092	q1	q2	OK	62.4608	64.1693	0.0389336	0.0393242	0.91315	0.99995	no

ENSG00000140526	ENSG00000140526	ABHD2	15:89630308-89745591	q1	q2	OK	8.10067	8.50595	0.0704315	0.129871	0.8129	0.99995	no

ENSG00000140527	ENSG00000140527	WDR93	15:90234027-90286869	q1	q2	NOTEST	0	0	0	0	1	1	no

ENSG00000140534	ENSG00000140534	TICRR	15:90048507-90198682	q1	q2	OK	12.9643	13.5145	0.0599643	0.106968	0.8792	0.99995	no

ENSG00000140538	ENSG00000140538	NTRK3	15:88418229-88814297	q1	q2	OK	1.4854	0.88567	-0.746009	-0.46935	0.13955	0.99995	no

ENSG00000140543	ENSG00000140543	DET1	15:89002706-89089906	q1	q2	OK	8.04658	7.91318	-0.0241183	-0.0250666	0.97225	0.99995	no

ENSG00000140545	ENSG00000140545	MFGE8	15:89441915-89456642	q1	q2	OK	82.4232	69.9425	-0.236883	-0.441325	0.4892	0.99995	no

ENSG00000140548	ENSG00000140548	ZNF710	15:90544623-90625439	q1	q2	OK	21.4464	21.7604	0.0209637	0.0394627	0.9434	0.99995	no

ENSG00000140553	ENSG00000140553	UNC45A	15:91473409-91506349	q1	q2	OK	29.159	25.5831	-0.188751	-0.333295	0.573	0.99995	no

ENSG00000140557	ENSG00000140557	ST8SIA2	15:92937057-93011958	q1	q2	OK	19.1206	20.9532	0.132044	0.21491	0.6975	0.99995	no

ENSG00000140563	ENSG00000140563	MCTP2	15:94774766-95023632	q1	q2	OK	0.646134	1.09413	0.75988	0.571168	0.31665	0.99995	no

ENSG00000140564	ENSG00000140564	FURIN	15:91411821-91426688	q1	q2	OK	6.18534	5.86278	-0.0772676	-0.122907	0.82885	0.99995	no

ENSG00000140575	ENSG00000140575	IQGAP1	15:90931449-91045475	q1	q2	OK	23.5917	22.782	-0.0503849	-0.0607968	0.89095	0.99995	no

ENSG00000140577	ENSG00000140577	CRTC3	15:91073156-91260372	q1	q2	OK	11.2077	12.5911	0.167912	0.233985	0.6078	0.99995	no

ENSG00000140598	ENSG00000140598	EFTUD1	15:82422570-82555104	q1	q2	OK	16.0573	12.1195	-0.405907	-0.760821	0.18515	0.99995	no

ENSG00000140600	ENSG00000140600	SH3GL3	15:84115979-84287495	q1	q2	OK	3.47496	2.81946	-0.301578	-0.376954	0.5694	0.99995	no

ENSG00000140612	ENSG00000140612	SEC11A	15:85212774-85259947	q1	q2	OK	132.94	134.397	0.0157244	0.0363792	0.9603	0.99995	no

ENSG00000140623	ENSG00000140623	Sep-12	16:4827669-4846533	q1	q2	NOTEST	0.0248234	0.0789891	1.66995	0	1	1	no

ENSG00000140632	ENSG00000140632	GLYR1	16:4853203-4932361	q1	q2	OK	52.5878	52.4654	-0.00336082	-0.00341127	0.993	0.99995	no

ENSG00000140650	ENSG00000140650	PMM2	16:8768421-8962866	q1	q2	OK	9.16085	9.4009	0.0373181	0.0163002	0.97435	0.99995	no

ENSG00000140675	ENSG00000140675	SLC5A2	16:31494322-31520630	q1	q2	NOTEST	0.17442	0.232997	0.417743	0	1	1	no

ENSG00000140678	ENSG00000140678	ITGAX	16:31366454-31394318	q1	q2	NOTEST	0	0	0	0	1	1	no

ENSG00000140682	ENSG00000140682	TGFB1I1	16:31482905-31489281	q1	q2	OK	20.8753	21.0002	0.00860167	0.0147894	0.98145	0.99995	no

ENSG00000140688	ENSG00000140688	C16orf58	16:31494322-31520630	q1	q2	OK	25.519	21.7823	-0.228416	-0.482357	0.45915	0.99995	no

ENSG00000140691	ENSG00000140691	ARMC5	16:31468031-31478487	q1	q2	OK	6.10667	5.33658	-0.194471	-0.271057	0.6805	0.99995	no

ENSG00000140694	ENSG00000140694	PARN	16:14529557-14726585	q1	q2	OK	13.2549	15.3103	0.207975	0.311176	0.5609	0.99995	no

ENSG00000140718	ENSG00000140718	FTO	16:53737874-54155853	q1	q2	OK	26.1331	28.52	0.126098	0.220716	0.7501	0.99995	no

ENSG00000140740	ENSG00000140740	UQCRC2	16:21963980-21994981	q1	q2	OK	92.2748	98.7734	0.0981868	0.170465	0.77175	0.99995	no

ENSG00000140743	ENSG00000140743	CDR2	16:22357256-22547842	q1	q2	OK	11.8795	12.4297	0.0653139	0.0549803	0.92845	0.99995	no

ENSG00000140749	ENSG00000140749	IGSF6	16:21608538-21668794	q1	q2	OK	0.831648	0.837409	0.00995783	0.00143495	0.9929	0.99995	no

ENSG00000140750	ENSG00000140750	ARHGAP17	16:24930705-25026987	q1	q2	OK	36.3476	38.2201	0.0724715	0.128888	0.81805	0.99995	no

ENSG00000140795	ENSG00000140795	MYLK3	16:46740890-46825952	q1	q2	NOTEST	0.180799	0.0695256	-1.37877	0	1	1	no

ENSG00000140798	ENSG00000140798	ABCC12	16:48116883-48189929	q1	q2	NOTEST	0	0	0	0	1	1	no

ENSG00000140807	ENSG00000140807	NKD1	16:50582240-50670647	q1	q2	OK	2.26931	2.6019	0.19731	0.228215	0.65855	0.99995	no

ENSG00000140829	ENSG00000140829	DHX38	16:72078187-72210777	q1	q2	OK	37.6272	32.8334	-0.196612	-0.261502	0.5921	0.99995	no

ENSG00000140830	ENSG00000140830	TXNL4B	16:72078187-72210777	q1	q2	OK	8.51757	7.31409	-0.219764	-0.171601	0.75615	0.99995	no

ENSG00000140832	ENSG00000140832	MARVELD3	16:71657610-71758604	q1	q2	OK	0.43797	0.715169	0.707451	0.204901	0.48435	0.99995	no

ENSG00000140835	ENSG00000140835	CHST4	16:71481499-71598992	q1	q2	NOTEST	0.0781196	0.0774012	-0.0133276	0	1	1	no

ENSG00000140836	ENSG00000140836	ZFHX3	16:72699068-73093597	q1	q2	OK	22.6078	23.3302	0.045379	0.065075	0.8786	0.99995	no

ENSG00000140839	ENSG00000140839	CLEC18B	16:74442528-74455368	q1	q2	OK	2.06357	1.03562	-0.994651	-0.851132	0.24765	0.99995	no

ENSG00000140848	ENSG00000140848	CPNE2	16:57126448-57181878	q1	q2	OK	34.4928	36.5896	0.08514	0.190496	0.7852	0.99995	no

ENSG00000140853	ENSG00000140853	NLRC5	16:57023396-57117443	q1	q2	NOTEST	0.466937	0.283843	-0.718136	0	1	1	no

ENSG00000140854	ENSG00000140854	KATNB1	16:57769641-57791162	q1	q2	OK	9.52891	9.0815	-0.0693808	-0.0718049	0.91985	0.99995	no

ENSG00000140859	ENSG00000140859	KIFC3	16:57792128-57896957	q1	q2	OK	1.22571	0.547529	-1.16261	-0.591695	0.02815	0.99995	no

ENSG00000140873	ENSG00000140873	ADAMTS18	16:77224731-77478233	q1	q2	NOTEST	0.360214	0.347143	-0.0533239	0	1	1	no

ENSG00000140876	ENSG00000140876	NUDT7	16:77756410-77776897	q1	q2	OK	3.9437	2.83622	-0.475581	-0.480385	0.4932	0.99995	no

ENSG00000140905	ENSG00000140905	GCSH	16:81087101-81130008	q1	q2	OK	27.5476	28.6916	0.0587036	0.0852631	0.89315	0.99995	no

ENSG00000140931	ENSG00000140931	CMTM3	16:66637776-66647795	q1	q2	OK	64.9241	63.4146	-0.0339387	-0.0789213	0.9107	0.99995	no

ENSG00000140932	ENSG00000140932	CMTM2	16:66613350-66622178	q1	q2	NOTEST	0.0981732	0.15619	0.6699	0	1	1	no

ENSG00000140937	ENSG00000140937	CDH11	16:64977655-65160015	q1	q2	OK	36.9358	33.4792	-0.141757	-0.182643	0.74465	0.99995	no

ENSG00000140939	ENSG00000140939	NOL3	16:67204056-67217943	q1	q2	OK	7.49789	4.18967	-0.839646	-0.387377	0.3421	0.99995	no

ENSG00000140941	ENSG00000140941	MAP1LC3B	16:87117167-87438385	q1	q2	OK	44.5163	44.1736	-0.0111497	-0.0241581	0.97165	0.99995	no

ENSG00000140943	ENSG00000140943	MBTPS1	16:84087367-84150511	q1	q2	OK	39.486	35.2185	-0.165006	-0.316313	0.5566	0.99995	no

ENSG00000140945	ENSG00000140945	CDH13	16:82660407-83841439	q1	q2	OK	0.697925	0.817772	0.228626	0.160234	0.65545	0.99995	no

ENSG00000140948	ENSG00000140948	ZCCHC14	16:87439851-87525651	q1	q2	OK	17.5544	17.5001	-0.00447121	-0.00943398	0.98775	0.99995	no

ENSG00000140950	ENSG00000140950	TLDC1	16:84511680-84587639	q1	q2	OK	12.3437	12.0767	-0.0315427	-0.0553839	0.9203	0.99995	no

ENSG00000140955	ENSG00000140955	ADAD2	16:84224743-84230774	q1	q2	NOTEST	0.0106101	0.0705613	2.73344	0	1	1	no

ENSG00000140961	ENSG00000140961	OSGIN1	16:83932730-83999937	q1	q2	NOTEST	0.390798	0.357727	-0.127563	0	1	1	no

ENSG00000140968	ENSG00000140968	IRF8	16:85932408-85956215	q1	q2	NOTEST	0.411219	0.134675	-1.61043	0	1	1	no

ENSG00000140983	ENSG00000140983	RHOT2	16:718085-724174	q1	q2	OK	48.2635	44.3868	-0.120801	-0.216273	0.7106	0.99995	no

ENSG00000140986	ENSG00000140986	RPL3L	16:1993974-2007607	q1	q2	NOTEST	0.0119726	0.0124443	0.0557557	0	1	1	no

ENSG00000140987	ENSG00000140987	ZSCAN32	16:3415098-3627401	q1	q2	OK	11.5173	11.4523	-0.00816654	-0.00801954	0.99145	0.99995	no

ENSG00000140988	ENSG00000140988	RPS2	16:2012052-2014861	q1	q2	OK	1112.39	967.844	-0.200816	-0.435071	0.52815	0.99995	no

ENSG00000140990	ENSG00000140990	NDUFB10	16:2009508-2011976	q1	q2	OK	91.2996	85.0433	-0.102412	-0.228144	0.7455	0.99995	no

ENSG00000140992	ENSG00000140992	PDPK1	16:2587964-2653189	q1	q2	OK	14.7738	15.089	0.0304557	0.0126953	0.9694	0.99995	no

ENSG00000140993	ENSG00000140993	TIGD7	16:3313742-3368852	q1	q2	OK	4.72781	5.61799	0.248883	0.164938	0.79015	0.99995	no

ENSG00000140995	ENSG00000140995	DEF8	16:90014332-90034468	q1	q2	OK	39.0143	32.6586	-0.256541	-0.380214	0.5283	0.99995	no

ENSG00000141002	ENSG00000141002	TCF25	16:89939999-90005169	q1	q2	OK	78.7313	66.7761	-0.237605	-0.16069	0.63535	0.99995	no

ENSG00000141012	ENSG00000141012	GALNS	16:88880141-88933068	q1	q2	OK	9.43108	8.16026	-0.208807	-0.20822	0.6646	0.99995	no

ENSG00000141013	ENSG00000141013	GAS8	16:90071272-90114181	q1	q2	OK	1.2782	1.14941	-0.153219	-0.0819687	0.8068	0.99995	no

ENSG00000141026	ENSG00000141026	MED9	17:17380299-17396540	q1	q2	OK	11.9057	11.1977	-0.0884557	-0.1642	0.79675	0.99995	no

ENSG00000141027	ENSG00000141027	NCOR1	17:15879873-16256970	q1	q2	OK	58.6132	54.9997	-0.0918031	-0.0589731	0.8158	0.99995	no

ENSG00000141028	ENSG00000141028	CDRT15P1	17:13659794-13972812	q1	q2	NOTEST	0.268419	0.325023	0.276054	0	1	1	no

ENSG00000141030	ENSG00000141030	COPS3	17:17150140-17184607	q1	q2	OK	66.9327	72.7402	0.120041	0.256889	0.6835	0.99995	no

ENSG00000141034	ENSG00000141034	GID4	17:17942605-17971718	q1	q2	OK	5.51539	4.81175	-0.1969	-0.316192	0.57675	0.99995	no

ENSG00000141040	ENSG00000141040	ZNF287	17:16454700-16472520	q1	q2	OK	7.03998	5.29721	-0.410338	-0.46523	0.36825	0.99995	no

ENSG00000141052	ENSG00000141052	MYOCD	17:12569206-12894960	q1	q2	NOTEST	0.043573	0.10984	1.3339	0	1	1	no

ENSG00000141068	ENSG00000141068	KSR1	17:25783669-25976586	q1	q2	OK	18.4256	17.4937	-0.0748773	-0.0702167	0.8577	0.99995	no

ENSG00000141076	ENSG00000141076	CIRH1A	16:69139466-69442474	q1	q2	OK	37.24	43.6957	0.230637	0.110795	0.80795	0.99995	no

ENSG00000141084	ENSG00000141084	RANBP10	16:67757004-67840555	q1	q2	OK	10.9508	10.0724	-0.120635	-0.237083	0.71485	0.99995	no

ENSG00000141086	ENSG00000141086	CTRL	16:67927174-67970990	q1	q2	OK	1.40315	1.25663	-0.159106	-0.0611746	0.88895	0.99995	no

ENSG00000141096	ENSG00000141096	DPEP3	16:68009565-68014732	q1	q2	NOTEST	0	0	0	0	1	1	no

ENSG00000141098	ENSG00000141098	GFOD2	16:67708433-67753324	q1	q2	OK	22.6034	24.1162	0.0934652	0.206568	0.7655	0.99995	no

ENSG00000141101	ENSG00000141101	NOB1	16:69760915-69788843	q1	q2	OK	22.4908	19.3818	-0.214631	-0.43052	0.51665	0.99995	no

ENSG00000141127	ENSG00000141127	PRPSAP2	17:18739019-18834581	q1	q2	OK	41.6659	44.5111	0.095301	0.207731	0.74035	0.99995	no

ENSG00000141140	ENSG00000141140	MYO19	17:34842472-34899284	q1	q2	NOTEST	0.0711753	0.0903796	0.344619	0	1	1	no

ENSG00000141141	ENSG00000141141	DDX52	17:35969786-36044255	q1	q2	OK	23.9651	24.8384	0.0516412	0.0970028	0.86485	0.99995	no

ENSG00000141150	ENSG00000141150	RASL10B	17:34058667-34070540	q1	q2	NOTEST	0.0811033	0.0705235	-0.201656	0	1	1	no

ENSG00000141161	ENSG00000141161	UNC45B	17:33474835-33518721	q1	q2	NOTEST	0.0137878	0	#NAME?	0	1	1	no

ENSG00000141179	ENSG00000141179	PCTP	17:53828339-53950300	q1	q2	OK	9.65465	9.7636	0.01619	0.0256791	0.9687	0.99995	no

ENSG00000141194	ENSG00000141194	OR4D1	17:56232493-56233517	q1	q2	NOTEST	0	0	0	0	1	1	no

ENSG00000141198	ENSG00000141198	TOM1L1	17:52976747-53241646	q1	q2	OK	5.33191	4.41614	-0.271867	-0.125956	0.69155	0.99995	no

ENSG00000141200	ENSG00000141200	KIF2B	17:51900238-51902573	q1	q2	NOTEST	0	0	0	0	1	1	no

ENSG00000141219	ENSG00000141219	C17orf80	17:71161115-71258491	q1	q2	OK	19.0567	19.5397	0.0361059	0.0600774	0.929	0.99995	no

ENSG00000141232	ENSG00000141232	TOB1	17:48939583-48987593	q1	q2	OK	14.6418	12.5669	-0.220467	-0.407153	0.55355	0.99995	no

ENSG00000141252	ENSG00000141252	VPS53	17:406410-624957	q1	q2	OK	7.02767	9.176	0.38482	0.458692	0.2948	0.99995	no

ENSG00000141255	ENSG00000141255	SPATA22	17:3343312-3461289	q1	q2	NOTEST	0.0739563	0	#NAME?	0	1	1	no

ENSG00000141258	ENSG00000141258	SGSM2	17:1963132-2284352	q1	q2	OK	66.5185	54.2764	-0.293432	-0.273584	0.5279	0.99995	no

ENSG00000141279	ENSG00000141279	NPEPPS	17:45600307-45726786	q1	q2	OK	54.7751	52.2437	-0.0682637	-0.118853	0.8155	0.99995	no

ENSG00000141293	ENSG00000141293	SKAP1	17:46210801-46507637	q1	q2	NOTEST	0	0.0399078	inf	0	1	1	no

ENSG00000141294	ENSG00000141294	LRRC46	17:45908992-45918699	q1	q2	OK	1.3871	1.19018	-0.220888	-0.113446	0.8683	0.99995	no

ENSG00000141295	ENSG00000141295	SCRN2	17:45908992-45918699	q1	q2	OK	16.9054	13.8702	-0.285495	-0.427822	0.53485	0.99995	no

ENSG00000141298	ENSG00000141298	SSH2	17:27887564-28513493	q1	q2	OK	12.2778	12	-0.0330225	-0.0164533	0.9493	0.99995	no

ENSG00000141314	ENSG00000141314	RHBDL3	17:30593194-30708905	q1	q2	OK	13.9929	13.7987	-0.0201634	-0.0141495	0.98455	0.99995	no

ENSG00000141316	ENSG00000141316	SPACA3	17:31297393-31324895	q1	q2	NOTEST	0.0384626	0	#NAME?	0	1	1	no

ENSG00000141337	ENSG00000141337	ARSG	17:66255322-66760023	q1	q2	OK	0.599934	0.73278	0.288577	0.057351	0.8705	0.99995	no

ENSG00000141338	ENSG00000141338	ABCA8	17:66863432-66951533	q1	q2	OK	3.20238	2.77018	-0.209164	-0.214835	0.68315	0.99995	no

ENSG00000141349	ENSG00000141349	G6PC3	17:42148102-42153709	q1	q2	OK	54.8416	47.7237	-0.200564	-0.424332	0.5416	0.99995	no

ENSG00000141367	ENSG00000141367	CLTC	17:57697218-57919616	q1	q2	OK	134.601	140.251	0.0593181	0.0737851	0.88975	0.99995	no

ENSG00000141371	ENSG00000141371	C17orf64	17:58256454-58508782	q1	q2	NOTEST	0	0	0	0	1	1	no

ENSG00000141376	ENSG00000141376	BCAS3	17:58754813-59488916	q1	q2	OK	6.33246	5.30263	-0.25606	-0.245593	0.6195	0.99995	no

ENSG00000141378	ENSG00000141378	PTRH2	17:57697218-57919616	q1	q2	OK	18.9589	18.9935	0.00263329	0.000810839	0.99845	0.99995	no

ENSG00000141380	ENSG00000141380	SS18	18:23596577-23671181	q1	q2	OK	49.1822	53.83	0.130274	0.272157	0.66155	0.99995	no

ENSG00000141384	ENSG00000141384	TAF4B	18:23805899-23971649	q1	q2	NOTEST	0.32986	0.254847	-0.372223	0	1	1	no

ENSG00000141385	ENSG00000141385	AFG3L2	18:12307667-12377313	q1	q2	OK	25.3539	26.7631	0.0780372	0.0963782	0.8729	0.99995	no

ENSG00000141391	ENSG00000141391	SLMO1	18:12407894-12432237	q1	q2	OK	4.89873	5.40952	0.143093	0.156653	0.80955	0.99995	no

ENSG00000141401	ENSG00000141401	IMPA2	18:11981023-12030876	q1	q2	OK	19.6946	20.8095	0.0794415	0.124342	0.84555	0.99995	no

ENSG00000141404	ENSG00000141404	GNAL	18:11688954-11909222	q1	q2	OK	9.33166	13.8876	0.573591	0.435053	0.3808	0.99995	no

ENSG00000141424	ENSG00000141424	SLC39A6	18:33688494-33709348	q1	q2	OK	76.5401	80.9205	0.0802904	0.194605	0.77165	0.99995	no

ENSG00000141425	ENSG00000141425	RPRD1A	18:33564349-33647539	q1	q2	OK	43.2223	44.4429	0.0401797	0.0843773	0.8988	0.99995	no

ENSG00000141428	ENSG00000141428	C18orf21	18:33552045-33559241	q1	q2	OK	21.6882	24.8287	0.195096	0.332884	0.6184	0.99995	no

ENSG00000141429	ENSG00000141429	GALNT1	18:33161080-33291798	q1	q2	OK	45.8674	49.5552	0.111568	0.230893	0.70735	0.99995	no

ENSG00000141431	ENSG00000141431	ASXL3	18:31158578-31331156	q1	q2	OK	2.97802	2.81381	-0.0818308	-0.0839249	0.80105	0.99995	no

ENSG00000141433	ENSG00000141433	ADCYAP1	18:894435-912173	q1	q2	NOTEST	0.130869	0.17451	0.41519	0	1	1	no

ENSG00000141434	ENSG00000141434	MEP1B	18:29598334-30050447	q1	q2	NOTEST	0.131116	0.0467684	-1.48724	0	1	1	no

ENSG00000141437	ENSG00000141437	SLC25A52	18:29339524-29340843	q1	q2	NOTEST	0	0.0554541	inf	0	1	1	no

ENSG00000141441	ENSG00000141441	GAREM	18:29598334-30050447	q1	q2	OK	5.78501	5.76222	-0.00569437	-0.00423473	0.9933	0.99995	no

ENSG00000141446	ENSG00000141446	ESCO1	18:19109241-19180845	q1	q2	OK	7.817	8.20882	0.0705599	0.145983	0.8293	0.99995	no

ENSG00000141447	ENSG00000141447	OSBPL1A	18:21718941-21977844	q1	q2	OK	12.4159	10.8348	-0.196522	-0.30506	0.5592	0.99995	no

ENSG00000141448	ENSG00000141448	GATA6	18:19749403-19785192	q1	q2	NOTEST	0.0939367	0.0651662	-0.527564	0	1	1	no

ENSG00000141449	ENSG00000141449	GREB1L	18:18822202-19105378	q1	q2	OK	23.881	21.5492	-0.148231	-0.180876	0.6409	0.99995	no

ENSG00000141452	ENSG00000141452	C18orf8	18:21083472-21166862	q1	q2	OK	10.1778	10.9904	0.110815	0.0911572	0.85505	0.99995	no

ENSG00000141456	ENSG00000141456	PELP1	17:4574678-4608824	q1	q2	OK	31.5173	30.6478	-0.0403621	-0.067134	0.89045	0.99995	no

ENSG00000141458	ENSG00000141458	NPC1	18:21083472-21166862	q1	q2	OK	18.6852	17.0022	-0.136181	-0.171401	0.6974	0.99995	no

ENSG00000141469	ENSG00000141469	SLC14A1	18:42792959-43332485	q1	q2	NOTEST	0	0.0762874	inf	0	1	1	no

ENSG00000141480	ENSG00000141480	ARRB2	17:4613783-4624794	q1	q2	OK	21.3355	18.9659	-0.169847	-0.284088	0.6429	0.99995	no

ENSG00000141485	ENSG00000141485	SLC13A5	17:6588031-6616886	q1	q2	OK	1.15752	1.42355	0.298458	0.272072	0.575	0.99995	no

ENSG00000141497	ENSG00000141497	ZMYND15	17:4643318-4649411	q1	q2	NOTEST	0.0340036	0.0230433	-0.561339	0	1	1	no

ENSG00000141499	ENSG00000141499	WRAP53	17:7565096-7606820	q1	q2	OK	15.8423	15.3208	-0.0482955	-0.0473281	0.93995	0.99995	no

ENSG00000141503	ENSG00000141503	MINK1	17:4736682-4806369	q1	q2	OK	28.4386	26.6295	-0.0948275	-0.129136	0.817	0.99995	no

ENSG00000141504	ENSG00000141504	SAT2	17:7465191-7536700	q1	q2	OK	91.3695	76.5423	-0.255456	-0.121706	0.7021	0.99995	no

ENSG00000141505	ENSG00000141505	ASGR1	17:7076749-7082883	q1	q2	OK	8.63085	9.15748	0.0854485	0.118731	0.87155	0.99995	no

ENSG00000141506	ENSG00000141506	PIK3R5	17:8782232-8869029	q1	q2	NOTEST	0.00576929	0.0115853	1.00583	0	1	1	no

ENSG00000141510	ENSG00000141510	TP53	17:7565096-7606820	q1	q2	OK	48.1652	45.1345	-0.0937601	-0.196766	0.7654	0.99995	no

ENSG00000141519	ENSG00000141519	CCDC40	17:78010434-78074412	q1	q2	OK	12.0251	11.2666	-0.0939962	-0.112284	0.8595	0.99995	no

ENSG00000141522	ENSG00000141522	ARHGDIA	17:79825596-79829282	q1	q2	OK	115.402	107.199	-0.10637	-0.240598	0.6909	0.99995	no

ENSG00000141524	ENSG00000141524	TMC6	17:76000248-76139049	q1	q2	OK	5.30895	5.65817	0.0919086	0.0531395	0.87315	0.99995	no

ENSG00000141526	ENSG00000141526	SLC16A3	17:80186272-80231607	q1	q2	OK	0.535938	0.702386	0.390197	0.055855	0.5916	0.99995	no

ENSG00000141527	ENSG00000141527	CARD14	17:78143790-78227299	q1	q2	NOTEST	0.375259	0.383826	0.032565	0	1	1	no

ENSG00000141540	ENSG00000141540	TTYH2	17:72209652-72258159	q1	q2	OK	10.7604	9.28099	-0.213374	-0.427069	0.50725	0.99995	no

ENSG00000141542	ENSG00000141542	RAB40B	17:80612848-80656604	q1	q2	OK	4.26594	3.36771	-0.341095	-0.405417	0.4637	0.99995	no

ENSG00000141543	ENSG00000141543	EIF4A3	17:78109012-78120982	q1	q2	OK	70.0735	67.7838	-0.0479284	-0.110358	0.86995	0.99995	no

ENSG00000141551	ENSG00000141551	CSNK1D	17:80186272-80231607	q1	q2	OK	64.0038	58.9974	-0.117506	-0.202512	0.67665	0.99995	no

ENSG00000141552	ENSG00000141552	ANAPC11	17:79845712-79869340	q1	q2	OK	123.754	114.308	-0.114556	-0.1442	0.8464	0.99995	no

ENSG00000141556	ENSG00000141556	TBCD	17:80709939-81009686	q1	q2	OK	38.3651	35.1036	-0.128177	-0.217585	0.67765	0.99995	no

ENSG00000141560	ENSG00000141560	FN3KRP	17:80674558-80692019	q1	q2	OK	20.672	19.3809	-0.0930359	-0.181062	0.77585	0.99995	no

ENSG00000141562	ENSG00000141562	NARF	17:80412148-80448413	q1	q2	OK	41.1598	36.9173	-0.156938	-0.28163	0.6202	0.99995	no

ENSG00000141564	ENSG00000141564	RPTOR	17:78518618-78945013	q1	q2	OK	8.8225	8.72959	-0.0152736	-0.0252814	0.9669	0.99995	no

ENSG00000141568	ENSG00000141568	FOXK2	17:80477588-80606429	q1	q2	OK	26.0821	29.0988	0.1579	0.180788	0.7031	0.99995	no

ENSG00000141569	ENSG00000141569	TRIM65	17:73876415-73893084	q1	q2	OK	8.40896	7.80821	-0.106936	-0.163807	0.76225	0.99995	no

ENSG00000141570	ENSG00000141570	CBX8	17:77765930-77776326	q1	q2	OK	7.64771	7.85037	0.0377327	0.0559114	0.92785	0.99995	no

ENSG00000141574	ENSG00000141574	SECTM1	17:80278899-80291950	q1	q2	NOTEST	0.128197	0.080184	-0.676976	0	1	1	no

ENSG00000141576	ENSG00000141576	RNF157	17:74132413-74236454	q1	q2	OK	18.4926	18.533	0.00314983	0.00476984	0.99185	0.99995	no

ENSG00000141577	ENSG00000141577	AZI1	17:79163392-79196799	q1	q2	OK	21.2402	18.4268	-0.204989	-0.268459	0.70065	0.99995	no

ENSG00000141579	ENSG00000141579	ZNF750	17:80709939-81009686	q1	q2	NOTEST	0.153634	0.110472	-0.475811	0	1	1	no

ENSG00000141580	ENSG00000141580	WDR45B	17:80477588-80606429	q1	q2	OK	42.0472	41.1143	-0.0323676	-0.050259	0.93985	0.99995	no

ENSG00000141582	ENSG00000141582	CBX4	17:77806954-77813228	q1	q2	OK	13.6378	12.5668	-0.117987	-0.190694	0.726	0.99995	no

ENSG00000141622	ENSG00000141622	RNF165	18:43906771-44043103	q1	q2	OK	19.336	20.754	0.102099	0.178862	0.7455	0.99995	no

ENSG00000141627	ENSG00000141627	DYM	18:46550072-46987717	q1	q2	OK	15.9995	15.9985	-9.11E-05	-0.000159604	0.9997	0.99995	no

ENSG00000141639	ENSG00000141639	MAPK4	18:48086447-48258194	q1	q2	OK	2.47206	2.20706	-0.163584	-0.259699	0.6989	0.99995	no

ENSG00000141642	ENSG00000141642	ELAC1	18:48494360-48611415	q1	q2	OK	10.4804	9.66693	-0.116564	-0.063552	0.90715	0.99995	no

ENSG00000141644	ENSG00000141644	MBD1	18:47793251-47808144	q1	q2	OK	25.0117	25.8645	0.0483715	0.0752816	0.8795	0.99995	no

ENSG00000141646	ENSG00000141646	SMAD4	18:48494360-48611415	q1	q2	OK	68.841	72.3295	0.0713153	0.0883557	0.88235	0.99995	no

ENSG00000141655	ENSG00000141655	TNFRSF11A	18:59992519-60058516	q1	q2	NOTEST	0.0649344	0.128952	0.989782	0	1	1	no

ENSG00000141664	ENSG00000141664	ZCCHC2	18:60190239-60254942	q1	q2	OK	4.44106	4.87748	0.135232	0.19273	0.7297	0.99995	no

ENSG00000141665	ENSG00000141665	FBXO15	18:71740587-71815100	q1	q2	OK	1.76347	1.1788	-0.5811	-0.484397	0.38115	0.99995	no

ENSG00000141668	ENSG00000141668	CBLN2	18:70203914-70305756	q1	q2	OK	1.61155	1.30246	-0.307209	-0.323061	0.5502	0.99995	no

ENSG00000141682	ENSG00000141682	PMAIP1	18:57567179-57571538	q1	q2	OK	13.8782	15.2997	0.14068	0.278065	0.69205	0.99995	no

ENSG00000141696	ENSG00000141696	LEPREL4	17:39958198-39968856	q1	q2	NOTEST	0.067718	0	#NAME?	0	1	1	no

ENSG00000141698	ENSG00000141698	NT5C3B	17:39981334-40004636	q1	q2	NOTEST	0	0	0	0	1	1	no

ENSG00000141699	ENSG00000141699	FAM134C	17:40731530-40767252	q1	q2	OK	18.3705	16.5327	-0.152071	-0.176933	0.6975	0.99995	no

ENSG00000141720	ENSG00000141720	PIP4K2B	17:36921941-36956379	q1	q2	OK	44.2584	45.4878	0.0395296	0.0793442	0.8904	0.99995	no

ENSG00000141736	ENSG00000141736	ERBB2	17:37827374-37887040	q1	q2	OK	34.4399	31.0375	-0.15007	-0.231525	0.66185	0.99995	no

ENSG00000141738	ENSG00000141738	GRB7	17:37894179-37903544	q1	q2	NOTEST	0.103967	0.12512	0.267181	0	1	1	no

ENSG00000141741	ENSG00000141741	MIEN1	17:37827374-37887040	q1	q2	OK	20.9546	18.5936	-0.17246	-0.122945	0.85295	0.99995	no

ENSG00000141744	ENSG00000141744	PNMT	17:37824233-37826728	q1	q2	OK	1.05628	0.739912	-0.513572	-0.448796	0.53075	0.99995	no

ENSG00000141748	ENSG00000141748	ARL5C	17:37313146-37323737	q1	q2	NOTEST	0.0768436	0.182143	1.24508	0	1	1	no

ENSG00000141750	ENSG00000141750	STAC2	17:37366788-37382125	q1	q2	OK	0.670487	0.540372	-0.311256	-0.339376	0.62115	0.99995	no

ENSG00000141753	ENSG00000141753	IGFBP4	17:38599701-38613983	q1	q2	OK	67.8299	68.7784	0.0200347	0.0465498	0.9446	0.99995	no

ENSG00000141756	ENSG00000141756	FKBP10	17:39968931-39979465	q1	q2	NOTEST	0	0	0	0	1	1	no

ENSG00000141759	ENSG00000141759	TXNL4A	18:77732866-77793949	q1	q2	OK	69.942	66.3499	-0.0760641	-0.144801	0.84085	0.99995	no

ENSG00000141837	ENSG00000141837	CACNA1A	19:13317255-13734804	q1	q2	OK	1.06791	0.868895	-0.297537	-0.149958	0.65405	0.99995	no

ENSG00000141854	ENSG00000141854	hsa-mir-1199	19:14183347-14185874	q1	q2	OK	1.07501	0.747567	-0.524074	-0.524376	0.4171	0.99995	no

ENSG00000141858	ENSG00000141858	SAMD1	19:14198651-14201848	q1	q2	OK	36.1885	30.4641	-0.248421	-0.459109	0.4979	0.99995	no

ENSG00000141867	ENSG00000141867	BRD4	19:15347646-15443356	q1	q2	OK	33.0043	31.6246	-0.0616064	-0.0874497	0.8359	0.99995	no

ENSG00000141873	ENSG00000141873	SLC39A3	19:2714564-2740150	q1	q2	OK	17.5499	17.0576	-0.0410447	-0.0523239	0.9334	0.99995	no

ENSG00000141905	ENSG00000141905	NFIC	19:3359560-3469215	q1	q2	OK	3.82533	3.86452	0.0147038	0.0141312	0.97955	0.99995	no

ENSG00000141933	ENSG00000141933	TPGS1	19:489175-519654	q1	q2	OK	9.24968	8.25094	-0.164846	-0.255927	0.7153	0.99995	no

ENSG00000141934	ENSG00000141934	PPAP2C	19:281039-291393	q1	q2	OK	0.612534	0.582146	-0.0734089	-0.0558494	0.92345	0.99995	no

ENSG00000141946	ENSG00000141946	ZIM3	19:57645417-57656570	q1	q2	NOTEST	0	0	0	0	1	1	no

ENSG00000141956	ENSG00000141956	PRDM15	21:43218384-43299591	q1	q2	OK	3.67951	3.99072	0.117135	0.191858	0.72355	0.99995	no

ENSG00000141959	ENSG00000141959	PFKL	21:45719933-45747259	q1	q2	OK	38.7552	32.0755	-0.27292	-0.620009	0.35665	0.99995	no

ENSG00000141965	ENSG00000141965	FEM1A	19:4791727-4795571	q1	q2	OK	10.6115	10.1314	-0.066803	-0.137318	0.8392	0.99995	no

ENSG00000141968	ENSG00000141968	VAV1	19:6772724-6857377	q1	q2	NOTEST	0.0436285	0	#NAME?	0	1	1	no

ENSG00000141971	ENSG00000141971	MVB12A	19:17516502-17544533	q1	q2	OK	22.1364	19.5919	-0.176166	-0.289743	0.6783	0.99995	no

ENSG00000141977	ENSG00000141977	CIB3	19:16272178-16284336	q1	q2	NOTEST	0.0940346	0	#NAME?	0	1	1	no

ENSG00000141979	ENSG00000141979	CTD-3222D19.2	19:16589867-16771253	q1	q2	NOTEST	0	0	0	0	1	1	no

ENSG00000141985	ENSG00000141985	SH3GL1	19:4360366-4400544	q1	q2	OK	39.5674	34.692	-0.189709	-0.448861	0.5124	0.99995	no

ENSG00000141994	ENSG00000141994	DUS3L	19:5782970-5791249	q1	q2	OK	11.1535	9.17097	-0.282354	-0.358018	0.51915	0.99995	no

ENSG00000142002	ENSG00000142002	DPP9	19:4675235-4724685	q1	q2	OK	13.6775	12.9833	-0.0751469	-0.118729	0.79395	0.99995	no

ENSG00000142025	ENSG00000142025	DMRTC2	19:42348805-42356401	q1	q2	NOTEST	0	0	0	0	1	1	no

ENSG00000142039	ENSG00000142039	CCDC97	19:41768400-41930910	q1	q2	OK	19.379	17.4171	-0.153989	-0.0634468	0.83505	0.99995	no

ENSG00000142046	ENSG00000142046	TMEM91	19:41768400-41930910	q1	q2	OK	6.43524	6.5621	0.0281638	0.00766162	0.9765	0.99995	no

ENSG00000142065	ENSG00000142065	ZFP14	19:36827161-36870101	q1	q2	OK	6.93165	6.23752	-0.152227	-0.218764	0.6714	0.99995	no

ENSG00000142082	ENSG00000142082	SIRT3	11:215457-252983	q1	q2	OK	14.765	14.2596	-0.0502454	-0.0301357	0.93675	0.99995	no

ENSG00000142089	ENSG00000142089	IFITM3	11:318639-330122	q1	q2	OK	114.989	91.678	-0.326848	-0.735086	0.2941	0.99995	no

ENSG00000142102	ENSG00000142102	ATHL1	11:289134-296107	q1	q2	OK	10.4935	9.35759	-0.165293	-0.260208	0.67595	0.99995	no

ENSG00000142149	ENSG00000142149	HUNK	21:33245627-33416946	q1	q2	OK	7.38873	8.8228	0.255911	0.468641	0.3929	0.99995	no

ENSG00000142156	ENSG00000142156	COL6A1	21:47401650-47424964	q1	q2	OK	78.0234	66.7852	-0.224379	-0.465403	0.4902	0.99995	no

ENSG00000142163	ENSG00000142163	OR1E3	17:3019716-3020660	q1	q2	NOTEST	0	0	0	0	1	1	no

ENSG00000142166	ENSG00000142166	IFNAR1	21:34696733-34732168	q1	q2	OK	14.0717	16.0459	0.189414	0.350045	0.61005	0.99995	no

ENSG00000142168	ENSG00000142168	SOD1	21:33031934-33041244	q1	q2	OK	153.319	139.357	-0.137752	-0.332476	0.63865	0.99995	no

ENSG00000142173	ENSG00000142173	COL6A2	21:47518010-47552763	q1	q2	OK	75.6463	64.1249	-0.238385	-0.394571	0.48405	0.99995	no

ENSG00000142178	ENSG00000142178	SIK1	21:44834394-44847008	q1	q2	OK	1.3491	1.17035	-0.205053	-0.26938	0.67145	0.99995	no

ENSG00000142182	ENSG00000142182	DNMT3L	21:45666221-45682099	q1	q2	NOTEST	0	0	0	0	1	1	no

ENSG00000142185	ENSG00000142185	TRPM2	21:45770045-45862964	q1	q2	NOTEST	0.0332401	0.102811	1.629	0	1	1	no

ENSG00000142186	ENSG00000142186	SCYL1	11:65292547-65306175	q1	q2	OK	26.2934	24.3001	-0.113741	-0.223996	0.72125	0.99995	no

ENSG00000142188	ENSG00000142188	TMEM50B	21:34775201-34852318	q1	q2	OK	0.474806	2.36726	2.31781	1.24419	0.0181	0.99995	no

ENSG00000142192	ENSG00000142192	APP	21:27252860-27589700	q1	q2	OK	206.248	213.051	0.0468198	0.0991078	0.87905	0.99995	no

ENSG00000142197	ENSG00000142197	DOPEY2	21:37529079-37666572	q1	q2	OK	5.47963	4.61214	-0.248641	-0.249126	0.54415	0.99995	no

ENSG00000142207	ENSG00000142207	URB1	21:33664123-33765335	q1	q2	OK	4.68714	5.10974	0.124544	0.256485	0.6741	0.99995	no

ENSG00000142208	ENSG00000142208	AKT1	14:105235685-105262088	q1	q2	OK	70.5296	66.0218	-0.0952868	-0.199308	0.7372	0.99995	no

ENSG00000142224	ENSG00000142224	IL19	1:206972214-207016324	q1	q2	NOTEST	0	0.0296449	inf	0	1	1	no

ENSG00000142227	ENSG00000142227	EMP3	19:48799713-48833810	q1	q2	OK	26.2307	24.7361	-0.084639	-0.121784	0.8563	0.99995	no

ENSG00000142230	ENSG00000142230	SAE1	19:47567443-47713886	q1	q2	OK	166.998	159.448	-0.0667413	-0.137594	0.8441	0.99995	no

ENSG00000142233	ENSG00000142233	NTN5	19:49141198-49184461	q1	q2	NOTEST	0.110904	0.0571567	-0.956313	0	1	1	no

ENSG00000142235	ENSG00000142235	LMTK3	19:48988527-49016446	q1	q2	OK	2.07769	1.65819	-0.325376	-0.50824	0.47175	0.99995	no

ENSG00000142252	ENSG00000142252	GEMIN7	19:45574757-45808541	q1	q2	OK	24.3416	25.0594	0.041928	0.0343891	0.9635	0.99995	no

ENSG00000142273	ENSG00000142273	CBLC	19:45281125-45303891	q1	q2	NOTEST	0	0.0397069	inf	0	1	1	no

ENSG00000142279	ENSG00000142279	WTIP	19:34971873-34997258	q1	q2	OK	5.57418	4.52448	-0.301006	-0.390745	0.52975	0.99995	no

ENSG00000142303	ENSG00000142303	ADAMTS10	19:8645125-8675620	q1	q2	NOTEST	0.0110802	0.165719	3.90268	0	1	1	no

ENSG00000142319	ENSG00000142319	SLC6A3	5:1392908-1445545	q1	q2	NOTEST	0.0496415	0.0649367	0.387487	0	1	1	no

ENSG00000142327	ENSG00000142327	RNPEPL1	2:241418838-241520789	q1	q2	OK	14.1625	13.0665	-0.116198	-0.160019	0.80005	0.99995	no

ENSG00000142330	ENSG00000142330	CAPN10	2:241526132-241570676	q1	q2	OK	18.9335	16.0165	-0.241383	-0.362981	0.52715	0.99995	no

ENSG00000142347	ENSG00000142347	MYO1F	19:8585673-8642461	q1	q2	OK	2.12749	1.2749	-0.738769	-0.474902	0.3749	0.99995	no

ENSG00000142396	ENSG00000142396	ERVK3-1	19:58739959-58827023	q1	q2	OK	39.3671	40.1529	0.0285139	0.0432177	0.94935	0.99995	no

ENSG00000142405	ENSG00000142405	NLRP12	19:54296856-54327648	q1	q2	NOTEST	0.0078958	0	#NAME?	0	1	1	no

ENSG00000142408	ENSG00000142408	CACNG8	19:54466293-54493469	q1	q2	OK	10.8421	11.4142	0.0741893	0.18007	0.7964	0.99995	no

ENSG00000142409	ENSG00000142409	ZNF787	19:56598731-56632649	q1	q2	OK	14.6278	14.2933	-0.0333773	-0.0650828	0.92245	0.99995	no

ENSG00000142444	ENSG00000142444	C19orf52	19:11039408-11044211	q1	q2	OK	13.8391	14.3746	0.0547695	0.0780588	0.9005	0.99995	no

ENSG00000142449	ENSG00000142449	FBN3	19:8130285-8214730	q1	q2	OK	6.41512	4.70602	-0.44697	-0.257077	0.4583	0.99995	no

ENSG00000142453	ENSG00000142453	CARM1	19:10982188-11039357	q1	q2	OK	44.7694	39.9324	-0.164953	-0.302357	0.6361	0.99995	no

ENSG00000142459	ENSG00000142459	EVI5L	19:7895118-7929862	q1	q2	OK	10.0658	8.44343	-0.253567	-0.503317	0.46525	0.99995	no

ENSG00000142484	ENSG00000142484	TM4SF5	17:4675178-4686506	q1	q2	NOTEST	0	0	0	0	1	1	no

ENSG00000142494	ENSG00000142494	SLC47A1	17:19398697-19501235	q1	q2	NOTEST	0.244825	0.19595	-0.321269	0	1	1	no

ENSG00000142507	ENSG00000142507	PSMB6	17:4699438-4701790	q1	q2	OK	129.088	126.799	-0.0258085	-0.061906	0.93045	0.99995	no

ENSG00000142511	ENSG00000142511	GPR32	19:51273720-51274989	q1	q2	NOTEST	0.0468563	0.0216131	-1.11633	0	1	1	no

ENSG00000142512	ENSG00000142512	SIGLEC10	19:51913274-51921057	q1	q2	OK	0.864526	0.829899	-0.0589723	-0.0332098	0.9466	0.99995	no

ENSG00000142513	ENSG00000142513	ACPT	19:51293671-51298481	q1	q2	NOTEST	0.0562051	0.069622	0.308841	0	1	1	no

ENSG00000142515	ENSG00000142515	KLK3	19:51358170-51364020	q1	q2	NOTEST	0	0	0	0	1	1	no

ENSG00000142528	ENSG00000142528	ZNF473	19:50479723-50570050	q1	q2	OK	8.20294	8.40232	0.0346454	0.0541902	0.9361	0.99995	no

ENSG00000142530	ENSG00000142530	FAM71E1	19:50970041-50986608	q1	q2	NOTEST	0.293479	0.33951	0.210194	0	1	1	no

ENSG00000142534	ENSG00000142534	RPS11	19:49999621-50002946	q1	q2	OK	1740.9	1694.41	-0.0390515	-0.0860412	0.90225	0.99995	no

ENSG00000142538	ENSG00000142538	PTH2	19:49925670-49926698	q1	q2	NOTEST	0	0	0	0	1	1	no

ENSG00000142539	ENSG00000142539	CTD-2545M3.6	19:50887460-50934570	q1	q2	NOTEST	0	0.0234912	inf	0	1	1	no

ENSG00000142541	ENSG00000142541	RPL13A	19:49949554-49995565	q1	q2	OK	755.71	671.159	-0.171177	-0.344138	0.61785	0.99995	no

ENSG00000142544	ENSG00000142544	CTU1	19:51600862-51611627	q1	q2	OK	1.76901	1.33233	-0.408993	-0.480785	0.5089	0.99995	no

ENSG00000142546	ENSG00000142546	NOSIP	19:50058967-50094272	q1	q2	OK	38.5392	33.3881	-0.206989	-0.422508	0.548	0.99995	no

ENSG00000142549	ENSG00000142549	IGLON5	19:51815101-51833608	q1	q2	OK	26.8633	23.7356	-0.178582	-0.415297	0.55405	0.99995	no

ENSG00000142552	ENSG00000142552	RCN3	19:50030874-50050219	q1	q2	OK	5.73225	4.37608	-0.389463	-0.452887	0.4369	0.99995	no

ENSG00000142556	ENSG00000142556	ZNF614	19:52516017-52533493	q1	q2	OK	5.58258	6.10945	0.13011	0.224423	0.7016	0.99995	no

ENSG00000142583	ENSG00000142583	SLC2A5	1:9095165-9148537	q1	q2	NOTEST	0.0190502	0.0356587	0.904447	0	1	1	no

ENSG00000142599	ENSG00000142599	RERE	1:8412456-8877702	q1	q2	OK	44.2041	44.1116	-0.00302132	-0.00203467	0.99405	0.99995	no

ENSG00000142606	ENSG00000142606	MMEL1	1:2517929-2718286	q1	q2	NOTEST	0	0.0103736	inf	0	1	1	no

ENSG00000142609	ENSG00000142609	C1orf222	1:1853395-1935276	q1	q2	NOTEST	0.128461	0.0342336	-1.90785	0	1	1	no

ENSG00000142611	ENSG00000142611	PRDM16	1:2985731-3355185	q1	q2	OK	0.67359	0.545272	-0.304893	-0.163976	0.6285	0.99995	no

ENSG00000142615	ENSG00000142615	CELA2A	1:15783222-15853029	q1	q2	NOTEST	0	0	0	0	1	1	no

ENSG00000142619	ENSG00000142619	PADI3	1:17575592-17610728	q1	q2	NOTEST	0	0.00768861	inf	0	1	1	no

ENSG00000142621	ENSG00000142621	FHAD1	1:15573767-15726779	q1	q2	OK	0.517837	0.472039	-0.133593	-0.0664579	0.8289	0.99995	no

ENSG00000142623	ENSG00000142623	PADI1	1:17531620-17572500	q1	q2	NOTEST	0	0	0	0	1	1	no

ENSG00000142627	ENSG00000142627	EPHA2	1:16450831-16483824	q1	q2	OK	14.8784	14.8094	-0.00670918	-0.0125283	0.9816	0.99995	no

ENSG00000142632	ENSG00000142632	ARHGEF19	1:16524348-16539104	q1	q2	OK	2.57443	2.61226	0.0210459	0.0215617	0.9731	0.99995	no

ENSG00000142634	ENSG00000142634	EFHD2	1:15736390-15756839	q1	q2	OK	10.1814	9.6444	-0.0781761	-0.150767	0.82435	0.99995	no

ENSG00000142655	ENSG00000142655	PEX14	1:10516578-10690815	q1	q2	OK	13.4944	11.0374	-0.289959	-0.290614	0.6488	0.99995	no

ENSG00000142657	ENSG00000142657	PGD	1:10458648-10480201	q1	q2	OK	232.005	223.804	-0.0519236	-0.117144	0.857	0.99995	no

ENSG00000142661	ENSG00000142661	MYOM3	1:24367324-24438665	q1	q2	NOTEST	0	0.016065	inf	0	1	1	no

ENSG00000142669	ENSG00000142669	SH3BGRL3	1:26605666-26647014	q1	q2	OK	71.4734	64.2719	-0.153218	-0.292191	0.6759	0.99995	no

ENSG00000142675	ENSG00000142675	CNKSR1	1:26503893-26516377	q1	q2	OK	1.14146	1.77106	0.633734	0.479036	0.2921	0.99995	no

ENSG00000142676	ENSG00000142676	RPL11	1:24018268-24022915	q1	q2	OK	1229.68	1183.79	-0.0548705	-0.126374	0.8548	0.99995	no

ENSG00000142677	ENSG00000142677	IL22RA1	1:24446260-24469611	q1	q2	NOTEST	0.0192584	0.00949764	-1.01984	0	1	1	no

ENSG00000142684	ENSG00000142684	ZNF593	1:26496361-26498551	q1	q2	OK	4.75062	3.73414	-0.347341	-0.500917	0.4709	0.99995	no

ENSG00000142686	ENSG00000142686	C1orf216	1:36179475-36185073	q1	q2	OK	9.98572	9.42184	-0.0838576	-0.141586	0.80635	0.99995	no

ENSG00000142687	ENSG00000142687	KIAA0319L	1:35899090-36032875	q1	q2	OK	24.6364	24.9019	0.0154591	0.015909	0.96205	0.99995	no

ENSG00000142694	ENSG00000142694	EVA1B	1:36771987-36794822	q1	q2	OK	21.3149	18.6651	-0.191519	-0.278089	0.7016	0.99995	no

ENSG00000142698	ENSG00000142698	C1orf94	1:34632483-34684732	q1	q2	NOTEST	0.0503616	0.0246262	-1.03213	0	1	1	no

ENSG00000142700	ENSG00000142700	DMRTA2	1:50883221-50889172	q1	q2	NOTEST	0.0184627	0.00870769	-1.08425	0	1	1	no

ENSG00000142731	ENSG00000142731	PLK4	4:128802015-128820350	q1	q2	OK	28.7222	31.6974	0.1422	0.212421	0.6969	0.99995	no

ENSG00000142733	ENSG00000142733	MAP3K6	1:27681674-27693383	q1	q2	OK	3.2561	2.88816	-0.172995	-0.134312	0.7905	0.99995	no

ENSG00000142748	ENSG00000142748	FCN3	1:27695602-27701315	q1	q2	NOTEST	0.0595458	0	#NAME?	0	1	1	no

ENSG00000142751	ENSG00000142751	GPN2	1:27202623-27216788	q1	q2	OK	10.3446	8.01808	-0.367549	-0.564246	0.319	0.99995	no

ENSG00000142765	ENSG00000142765	SYTL1	1:27668512-27680421	q1	q2	NOTEST	0.128431	0.0810177	-0.664687	0	1	1	no

ENSG00000142784	ENSG00000142784	WDTC1	1:27561006-27635110	q1	q2	OK	17.1116	14.8296	-0.206496	-0.469391	0.50905	0.99995	no

ENSG00000142789	ENSG00000142789	CELA3A	1:22328148-22339032	q1	q2	NOTEST	0	0	0	0	1	1	no

ENSG00000142794	ENSG00000142794	NBPF3	1:21766620-21811498	q1	q2	OK	4.15447	4.35894	0.069311	0.0855115	0.88625	0.99995	no

ENSG00000142798	ENSG00000142798	HSPG2	1:22138757-22263790	q1	q2	OK	10.1605	9.18064	-0.146308	-0.164958	0.70305	0.99995	no

ENSG00000142856	ENSG00000142856	ITGB3BP	1:63906440-64125916	q1	q2	OK	39.2043	44.3939	0.179351	0.240066	0.7273	0.99995	no

ENSG00000142864	ENSG00000142864	SERBP1	1:67873492-67896098	q1	q2	OK	192.696	198.685	0.0441602	0.0878131	0.8922	0.99995	no

ENSG00000142867	ENSG00000142867	BCL10	1:85731930-86043933	q1	q2	OK	6.2527	7.14768	0.192996	0.162465	0.8194	0.99995	no

ENSG00000142871	ENSG00000142871	CYR61	1:86046443-86049645	q1	q2	OK	29.95	27.4324	-0.126672	-0.276218	0.6741	0.99995	no

ENSG00000142875	ENSG00000142875	PRKACB	1:84543744-84704181	q1	q2	OK	15.1361	16.5217	0.126374	0.291523	0.6603	0.99995	no

ENSG00000142892	ENSG00000142892	PIGK	1:77554674-77685115	q1	q2	OK	10.3838	10.3841	4.61E-05	6.88E-05	0.9999	0.99995	no

ENSG00000142910	ENSG00000142910	TINAGL1	1:32042115-32053288	q1	q2	NOTEST	0.0443976	0.0329507	-0.430172	0	1	1	no

ENSG00000142920	ENSG00000142920	ADC	1:33546704-33586131	q1	q2	OK	3.65182	4.36304	0.256715	0.262622	0.5952	0.99995	no

ENSG00000142937	ENSG00000142937	RPS8	1:45205489-45244451	q1	q2	OK	736.663	732.75	-0.00768331	-0.015442	0.9833	0.99995	no

ENSG00000142945	ENSG00000142945	KIF2C	1:45205489-45244451	q1	q2	OK	43.0438	42.1161	-0.031433	-0.0236623	0.9714	0.99995	no

ENSG00000142949	ENSG00000142949	PTPRF	1:43990857-44089343	q1	q2	OK	34.1997	29.2036	-0.22784	-0.44865	0.44555	0.99995	no

ENSG00000142959	ENSG00000142959	BEST4	1:45249256-45253377	q1	q2	NOTEST	0.0777875	0.0249307	-1.64162	0	1	1	no

ENSG00000142961	ENSG00000142961	MOB3C	1:47004367-47082563	q1	q2	OK	2.40609	1.83135	-0.393785	-0.309122	0.6681	0.99995	no

ENSG00000142973	ENSG00000142973	CYP4B1	1:47223509-47285085	q1	q2	NOTEST	0	0	0	0	1	1	no

ENSG00000143001	ENSG00000143001	TMEM61	1:55446464-55457966	q1	q2	NOTEST	0.0279307	0	#NAME?	0	1	1	no

ENSG00000143006	ENSG00000143006	DMRTB1	1:53925071-53933161	q1	q2	NOTEST	0	0	0	0	1	1	no

ENSG00000143013	ENSG00000143013	LMO4	1:87794150-87814606	q1	q2	OK	53.9912	60.9304	0.174439	0.357082	0.60745	0.99995	no

ENSG00000143028	ENSG00000143028	SYPL2	1:110009179-110024759	q1	q2	OK	1.59408	1.61318	0.0171785	0.0145897	0.9762	0.99995	no

ENSG00000143032	ENSG00000143032	BARHL2	1:91177095-91182794	q1	q2	NOTEST	0.0266777	0	#NAME?	0	1	1	no

ENSG00000143033	ENSG00000143033	MTF2	1:93544791-93604638	q1	q2	OK	40.3796	42.2943	0.0668352	0.134546	0.8313	0.99995	no

ENSG00000143036	ENSG00000143036	SLC44A3	1:95285897-95360802	q1	q2	OK	0.540028	0.465357	-0.214696	-0.143087	0.7728	0.99995	no

ENSG00000143061	ENSG00000143061	IGSF3	1:117117030-117210375	q1	q2	OK	13.0963	12.6973	-0.0446448	-0.104868	0.87965	0.99995	no

ENSG00000143067	ENSG00000143067	ZNF697	1:120162044-120190396	q1	q2	OK	6.76265	7.82003	0.209585	0.449154	0.5127	0.99995	no

ENSG00000143079	ENSG00000143079	CTTNBP2NL	1:112938802-113006078	q1	q2	OK	14.3225	14.1717	-0.0152664	-0.033347	0.96	0.99995	no

ENSG00000143093	ENSG00000143093	STRIP1	1:110574198-110617263	q1	q2	OK	28.6615	26.0995	-0.135088	-0.199116	0.68965	0.99995	no

ENSG00000143105	ENSG00000143105	KCNA10	1:111059838-111061797	q1	q2	NOTEST	0	0	0	0	1	1	no

ENSG00000143106	ENSG00000143106	PSMA5	1:109941652-109969062	q1	q2	OK	79.6842	76.7646	-0.0538532	-0.119655	0.86825	0.99995	no

ENSG00000143107	ENSG00000143107	FNDC7	1:109255278-109285365	q1	q2	NOTEST	0	0.0414532	inf	0	1	1	no

ENSG00000143110	ENSG00000143110	C1orf162	1:112016413-112021134	q1	q2	OK	1.02732	1.09025	0.0857753	0.0761174	0.9127	0.99995	no

ENSG00000143119	ENSG00000143119	CD53	1:111415774-111442550	q1	q2	NOTEST	0.0451222	0.110465	1.29168	0	1	1	no

ENSG00000143125	ENSG00000143125	PROK1	1:110993821-110999976	q1	q2	NOTEST	0	0.0288736	inf	0	1	1	no

ENSG00000143126	ENSG00000143126	CELSR2	1:109792640-109818372	q1	q2	OK	44.112	39.4443	-0.161353	-0.272223	0.634	0.99995	no

ENSG00000143127	ENSG00000143127	ITGA10	1:145524890-145543868	q1	q2	NOTEST	0.0754426	0.168078	1.15568	0	1	1	no

ENSG00000143140	ENSG00000143140	GJA5	1:147228331-147245484	q1	q2	NOTEST	0	0.0117856	inf	0	1	1	no

ENSG00000143147	ENSG00000143147	GPR161	1:168053996-168106821	q1	q2	OK	15.2677	14.7151	-0.0531851	-0.112387	0.8709	0.99995	no

ENSG00000143149	ENSG00000143149	ALDH9A1	1:165631452-165679205	q1	q2	OK	23.8465	26.5395	0.154366	0.268839	0.65275	0.99995	no

ENSG00000143153	ENSG00000143153	ATP1B1	1:169074934-169337205	q1	q2	OK	29.1046	28.7105	-0.0196699	-0.0354357	0.96165	0.99995	no

ENSG00000143155	ENSG00000143155	TIPRL	1:168148170-168169950	q1	q2	OK	26.3574	30.1107	0.192069	0.421269	0.5448	0.99995	no

ENSG00000143156	ENSG00000143156	NME7	1:169074934-169337205	q1	q2	OK	22.8022	21.0304	-0.116693	-0.132337	0.81505	0.99995	no

ENSG00000143157	ENSG00000143157	POGK	1:166808680-166825581	q1	q2	OK	33.1807	31.8962	-0.0569583	-0.117409	0.8647	0.99995	no

ENSG00000143158	ENSG00000143158	MPC2	1:167885966-168045081	q1	q2	OK	25.8786	24.6584	-0.0696831	-0.0866811	0.9007	0.99995	no

ENSG00000143162	ENSG00000143162	CREG1	1:167498913-167523004	q1	q2	OK	11.132	11.4379	0.0391097	0.0729541	0.9189	0.99995	no

ENSG00000143164	ENSG00000143164	DCAF6	1:167885966-168045081	q1	q2	OK	14.8728	13.6632	-0.122377	-0.167125	0.7604	0.99995	no

ENSG00000143167	ENSG00000143167	GPA33	1:167021787-167059868	q1	q2	NOTEST	0.053253	0.0300838	-0.823873	0	1	1	no

ENSG00000143171	ENSG00000143171	RXRG	1:165370158-165414433	q1	q2	NOTEST	0.145699	0.336557	1.20786	0	1	1	no

ENSG00000143178	ENSG00000143178	TBX19	1:168250277-168283664	q1	q2	OK	1.08213	0.985252	-0.135315	-0.149423	0.83765	0.99995	no

ENSG00000143179	ENSG00000143179	UCK2	1:165696031-165880855	q1	q2	OK	25.8473	26.4951	0.0357133	0.048218	0.944	0.99995	no

ENSG00000143183	ENSG00000143183	TMCO1	1:165696031-165880855	q1	q2	OK	54.265	56.9744	0.0702915	0.116578	0.86395	0.99995	no

ENSG00000143184	ENSG00000143184	XCL1	1:168545710-168551315	q1	q2	NOTEST	0	0.0197843	inf	0	1	1	no

ENSG00000143185	ENSG00000143185	XCL2	1:168510002-168513235	q1	q2	NOTEST	0	0	0	0	1	1	no

ENSG00000143190	ENSG00000143190	POU2F1	1:167190065-167396582	q1	q2	OK	9.55895	10.4335	0.126299	0.195272	0.65315	0.99995	no

ENSG00000143194	ENSG00000143194	MAEL	1:166958345-166991451	q1	q2	OK	0.544977	0.157534	-1.79053	-0.880453	0.10185	0.99995	no

ENSG00000143195	ENSG00000143195	ILDR2	1:166882442-166944719	q1	q2	OK	13.6806	13.1443	-0.0577046	-0.129926	0.8411	0.99995	no

ENSG00000143196	ENSG00000143196	DPT	1:168664696-168698502	q1	q2	NOTEST	0.0149622	0	#NAME?	0	1	1	no

ENSG00000143198	ENSG00000143198	MGST3	1:165600097-165631033	q1	q2	OK	93.7817	95.2968	0.0231213	0.049243	0.94255	0.99995	no

ENSG00000143199	ENSG00000143199	ADCY10	1:167778624-167883453	q1	q2	NOTEST	0.135445	0.0252396	-2.42394	0	1	1	no

ENSG00000143207	ENSG00000143207	RFWD2	1:175913966-176176629	q1	q2	OK	28.0745	27.9556	-0.00612106	-0.0130878	0.98365	0.99995	no

ENSG00000143217	ENSG00000143217	PVRL4	1:161040784-161059389	q1	q2	NOTEST	0.0295224	0.124969	2.08169	0	1	1	no

ENSG00000143222	ENSG00000143222	UFC1	1:161122565-161129139	q1	q2	OK	62.1706	61.4894	-0.0158952	-0.0345944	0.9572	0.99995	no

ENSG00000143224	ENSG00000143224	PPOX	1:161136199-161147803	q1	q2	OK	18.4419	16.9527	-0.121471	-0.148325	0.8311	0.99995	no

ENSG00000143226	ENSG00000143226	FCGR2A	1:161475219-161648444	q1	q2	NOTEST	0.0893914	0.0887056	-0.0111116	0	1	1	no

ENSG00000143228	ENSG00000143228	NUF2	1:163080910-163325554	q1	q2	OK	39.3781	39.626	0.00905491	0.0123027	0.9808	0.99995	no

ENSG00000143248	ENSG00000143248	RGS5	1:163080910-163325554	q1	q2	OK	11.2014	11.3212	0.015351	0.0103102	0.98225	0.99995	no

ENSG00000143252	ENSG00000143252	SDHC	1:161284046-161332984	q1	q2	OK	83.4664	78.481	-0.0888518	-0.1661	0.78325	0.99995	no

ENSG00000143256	ENSG00000143256	PFDN2	1:161070345-161102478	q1	q2	OK	96.6788	107.537	0.153558	0.235551	0.73255	0.99995	no

ENSG00000143257	ENSG00000143257	NR1I3	1:161195792-161208092	q1	q2	OK	0.325461	0.558166	0.778209	0.182457	0.1209	0.99995	no

ENSG00000143258	ENSG00000143258	USP21	1:161129239-161135513	q1	q2	OK	26.2971	23.1891	-0.181453	-0.319305	0.63975	0.99995	no

ENSG00000143278	ENSG00000143278	F13B	1:197008320-197036397	q1	q2	NOTEST	0.0124057	0.0228202	0.87931	0	1	1	no

ENSG00000143294	ENSG00000143294	PRCC	1:156711898-156770607	q1	q2	OK	46.5266	42.1043	-0.144087	-0.108048	0.82765	0.99995	no

ENSG00000143297	ENSG00000143297	FCRL5	1:157483166-157522310	q1	q2	NOTEST	0.00550808	0.0203905	1.88827	0	1	1	no

ENSG00000143303	ENSG00000143303	RRNAD1	1:156691682-156706752	q1	q2	OK	16.2689	12.1058	-0.426418	-0.370314	0.5561	0.99995	no

ENSG00000143314	ENSG00000143314	MRPL24	1:156707094-156711382	q1	q2	OK	29.0117	26.462	-0.132711	-0.231966	0.7386	0.99995	no

ENSG00000143315	ENSG00000143315	PIGM	1:159997461-160001783	q1	q2	OK	9.06038	9.86749	0.123112	0.269224	0.69465	0.99995	no

ENSG00000143318	ENSG00000143318	CASQ1	1:160160284-160171676	q1	q2	NOTEST	0.330505	0.289728	-0.189975	0	1	1	no

ENSG00000143319	ENSG00000143319	ISG20L2	1:156691682-156706752	q1	q2	OK	21.9823	19.7237	-0.156408	-0.237545	0.69525	0.99995	no

ENSG00000143320	ENSG00000143320	CRABP2	1:156669397-156675608	q1	q2	OK	26.6367	23.6729	-0.170178	-0.299	0.6597	0.99995	no

ENSG00000143321	ENSG00000143321	HDGF	1:156711898-156770607	q1	q2	OK	211.086	203.907	-0.0499165	-0.0950634	0.8865	0.99995	no

ENSG00000143322	ENSG00000143322	ABL2	1:179068461-179198819	q1	q2	OK	6.58679	7.3039	0.149091	0.307313	0.60375	0.99995	no

ENSG00000143324	ENSG00000143324	XPR1	1:180601139-180859387	q1	q2	OK	31.7968	32.9749	0.0524876	0.0966513	0.85995	0.99995	no

ENSG00000143333	ENSG00000143333	RGS16	1:182567757-182573543	q1	q2	OK	0.963972	0.952862	-0.0167236	-0.0181516	0.9803	0.99995	no

ENSG00000143337	ENSG00000143337	TOR1AIP1	1:179850741-179894135	q1	q2	OK	14.9185	14.7113	-0.0201783	-0.0445597	0.9485	0.99995	no

ENSG00000143340	ENSG00000143340	FAM163A	1:179712297-179787326	q1	q2	NOTEST	0.0359875	0.0084859	-2.08436	0	1	1	no

ENSG00000143341	ENSG00000143341	HMCN1	1:185703682-186160085	q1	q2	OK	3.4306	3.35205	-0.0334168	-0.0211799	0.9644	0.99995	no

ENSG00000143344	ENSG00000143344	RGL1	1:183605219-183897665	q1	q2	OK	11.8089	12.6669	0.101196	0.22848	0.74615	0.99995	no

ENSG00000143353	ENSG00000143353	LYPLAL1	1:219259943-219386207	q1	q2	OK	8.12602	9.19816	0.178795	0.205789	0.77715	0.99995	no

ENSG00000143355	ENSG00000143355	LHX9	1:197881036-197904608	q1	q2	NOTEST	0.222188	0.215947	-0.0410976	0	1	1	no

ENSG00000143363	ENSG00000143363	PRUNE	1:150980895-151008189	q1	q2	OK	13.3048	13.4361	0.014161	0.026567	0.9645	0.99995	no

ENSG00000143365	ENSG00000143365	RORC	1:151772739-151804348	q1	q2	NOTEST	0.0119833	0.0694349	2.53464	0	1	1	no

ENSG00000143367	ENSG00000143367	TUFT1	1:151512780-151556059	q1	q2	OK	3.9929	4.27909	0.0998678	0.150021	0.8042	0.99995	no

ENSG00000143368	ENSG00000143368	SF3B4	1:149895208-149900236	q1	q2	NOTEST	0	0	0	0	1	1	no

ENSG00000143369	ENSG00000143369	ECM1	1:150480537-150486265	q1	q2	OK	8.69167	8.14516	-0.0936901	-0.147715	0.833	0.99995	no

ENSG00000143373	ENSG00000143373	ZNF687	1:151252499-151300191	q1	q2	OK	13.2455	11.847	-0.160973	-0.206987	0.70935	0.99995	no

ENSG00000143374	ENSG00000143374	TARS2	1:150459886-150480078	q1	q2	OK	11.6457	11.5137	-0.0164436	-0.0254645	0.9641	0.99995	no

ENSG00000143375	ENSG00000143375	CGN	1:151482985-151511168	q1	q2	OK	1.63781	1.44755	-0.178147	-0.159508	0.7342	0.99995	no

ENSG00000143376	ENSG00000143376	SNX27	1:151584513-151671567	q1	q2	OK	12.5216	13.8169	0.142023	0.31842	0.62275	0.99995	no

ENSG00000143379	ENSG00000143379	SETDB1	1:150898738-150948010	q1	q2	OK	33.9145	28.7587	-0.237901	-0.255261	0.56195	0.99995	no

ENSG00000143382	ENSG00000143382	ADAMTSL4	1:150521039-150533413	q1	q2	OK	0.580502	0.572641	-0.0196698	-0.0153746	0.98165	0.99995	no

ENSG00000143384	ENSG00000143384	MCL1	1:150547031-150552066	q1	q2	OK	95.3947	104.765	0.13517	0.299866	0.6478	0.99995	no

ENSG00000143387	ENSG00000143387	CTSK	1:150768683-150780799	q1	q2	OK	10.1048	12.3163	0.285519	0.459255	0.48825	0.99995	no

ENSG00000143390	ENSG00000143390	RFX5	1:151313115-151320503	q1	q2	OK	33.5904	31.7104	-0.0830902	-0.118892	0.81555	0.99995	no

ENSG00000143393	ENSG00000143393	PI4KB	1:151252499-151300191	q1	q2	OK	23.1002	20.5068	-0.171802	-0.232612	0.6914	0.99995	no

ENSG00000143398	ENSG00000143398	PIP5K1A	1:151170424-151222012	q1	q2	OK	41.9396	41.3723	-0.019649	-0.0339011	0.9434	0.99995	no

ENSG00000143401	ENSG00000143401	ANP32E	1:150190716-150208504	q1	q2	OK	10.0012	12.891	0.36619	0.417798	0.53585	0.99995	no

ENSG00000143409	ENSG00000143409	FAM63A	1:150969024-150980851	q1	q2	OK	7.54868	6.37293	-0.244269	-0.33156	0.55765	0.99995	no

ENSG00000143412	ENSG00000143412	ANXA9	1:150954492-150968110	q1	q2	OK	0.656513	0.862809	0.394217	0.397908	0.54865	0.99995	no

ENSG00000143416	ENSG00000143416	SELENBP1	1:151336777-151345209	q1	q2	OK	15.9474	14.4802	-0.13924	-0.204515	0.70025	0.99995	no

ENSG00000143418	ENSG00000143418	CERS2	1:150898738-150948010	q1	q2	OK	61.5324	65.2115	0.0837812	0.138487	0.82805	0.99995	no

ENSG00000143420	ENSG00000143420	ENSA	1:150573326-150602088	q1	q2	OK	114.11	115.641	0.0192205	0.0405428	0.95455	0.99995	no

ENSG00000143429	ENSG00000143429	AC027612.6	2:91777535-91847998	q1	q2	OK	3.72007	2.66291	-0.482328	-0.438643	0.5277	0.99995	no

ENSG00000143434	ENSG00000143434	SEMA6C	1:151104160-151119104	q1	q2	OK	19.5807	17.4935	-0.162617	-0.168924	0.6497	0.99995	no

ENSG00000143436	ENSG00000143436	MRPL9	1:151728016-151766878	q1	q2	OK	61.5773	61.8015	0.00524417	0.00924962	0.9871	0.99995	no

ENSG00000143437	ENSG00000143437	ARNT	1:150782180-150849244	q1	q2	OK	13.8056	14.943	0.114217	0.183409	0.73445	0.99995	no

ENSG00000143442	ENSG00000143442	POGZ	1:151375199-151431941	q1	q2	OK	55.4061	55.1408	-0.00692328	-0.00724702	0.9803	0.99995	no

ENSG00000143443	ENSG00000143443	C1orf56	1:151020215-151042801	q1	q2	OK	2.76831	2.07382	-0.416714	-0.0893889	0.8414	0.99995	no

ENSG00000143450	ENSG00000143450	OAZ3	1:151728016-151766878	q1	q2	OK	1.02381	1.07669	0.0726608	0.0178014	0.9501	0.99995	no

ENSG00000143452	ENSG00000143452	HORMAD1	1:150670535-150693364	q1	q2	NOTEST	0.049444	0.047201	-0.0669765	0	1	1	no

ENSG00000143457	ENSG00000143457	GOLPH3L	1:150618700-150669630	q1	q2	OK	10.836	10.9424	0.0141019	0.0204537	0.9734	0.99995	no

ENSG00000143458	ENSG00000143458	GABPB2	1:151043053-151098018	q1	q2	OK	1.24328	1.18904	-0.0643525	-0.0727217	0.88795	0.99995	no

ENSG00000143466	ENSG00000143466	IKBKE	1:206643790-206671061	q1	q2	NOTEST	0.0841338	0.104225	0.308939	0	1	1	no

ENSG00000143469	ENSG00000143469	SYT14	1:210111537-210337636	q1	q2	OK	1.36891	1.41877	0.051608	0.0669302	0.9199	0.99995	no

ENSG00000143473	ENSG00000143473	KCNH1	1:210856554-211307457	q1	q2	NOTEST	0.269866	0.138775	-0.959499	0	1	1	no

ENSG00000143476	ENSG00000143476	DTL	1:212208918-212280742	q1	q2	OK	30.5412	33.7671	0.144861	0.195282	0.71585	0.99995	no

ENSG00000143479	ENSG00000143479	DYRK3	1:206807993-206857764	q1	q2	NOTEST	0.0506205	0.024287	-1.05954	0	1	1	no

ENSG00000143486	ENSG00000143486	EIF2D	1:206680878-206785904	q1	q2	NOTEST	0.281828	0.170521	-0.724863	0	1	1	no

ENSG00000143493	ENSG00000143493	INTS7	1:212113740-212208884	q1	q2	OK	15.2086	16.0151	0.0745532	0.14249	0.81365	0.99995	no

ENSG00000143494	ENSG00000143494	VASH2	1:213123861-213165379	q1	q2	OK	10.3157	11.4117	0.145666	0.242447	0.6434	0.99995	no

ENSG00000143498	ENSG00000143498	TAF1A	1:222731243-222766374	q1	q2	OK	10.7154	13.1912	0.299892	0.472277	0.5185	0.99995	no

ENSG00000143499	ENSG00000143499	SMYD2	1:214454444-214510474	q1	q2	OK	32.6691	36.752	0.169894	0.333738	0.6024	0.99995	no

ENSG00000143502	ENSG00000143502	SUSD4	1:223394160-223537544	q1	q2	OK	0.681008	1.0145	0.575029	0.461437	0.31555	0.99995	no

ENSG00000143507	ENSG00000143507	DUSP10	1:221874765-221915518	q1	q2	OK	0.959317	0.58053	-0.724636	-0.721972	0.24255	0.99995	no

ENSG00000143512	ENSG00000143512	HHIPL2	1:222695601-222721445	q1	q2	NOTEST	0.342986	0.393652	0.198772	0	1	1	no

ENSG00000143514	ENSG00000143514	TP53BP2	1:223967600-224033674	q1	q2	OK	28.0777	27.513	-0.0293157	-0.0624906	0.9165	0.99995	no

ENSG00000143515	ENSG00000143515	ATP8B2	1:154298028-154323783	q1	q2	OK	13.1525	11.6644	-0.173227	-0.280843	0.57885	0.99995	no

ENSG00000143520	ENSG00000143520	FLG2	1:152140600-152339163	q1	q2	NOTEST	0	0	0	0	1	1	no

ENSG00000143536	ENSG00000143536	CRNN	1:152346429-152417932	q1	q2	NOTEST	0	0	0	0	1	1	no

ENSG00000143537	ENSG00000143537	ADAM15	1:155006299-155035252	q1	q2	OK	14.1004	10.7236	-0.394943	-0.511222	0.29005	0.99995	no

ENSG00000143543	ENSG00000143543	JTB	1:153931574-153950164	q1	q2	OK	43.794	41.4115	-0.0807013	-0.100838	0.8847	0.99995	no

ENSG00000143545	ENSG00000143545	RAB13	1:153954126-153958834	q1	q2	OK	81.169	78.1616	-0.0544704	-0.121686	0.8638	0.99995	no

ENSG00000143546	ENSG00000143546	S100A8	1:153362507-153363664	q1	q2	NOTEST	0	0	0	0	1	1	no

ENSG00000143549	ENSG00000143549	TPM3	1:154127783-154167124	q1	q2	OK	210.985	218.101	0.0478607	0.105954	0.87985	0.99995	no

ENSG00000143552	ENSG00000143552	NUP210L	1:153965160-154127592	q1	q2	NOTEST	0.0367594	0.0734118	0.997897	0	1	1	no

ENSG00000143553	ENSG00000143553	SNAPIN	1:153631129-153634306	q1	q2	OK	36.9683	37.2267	0.0100495	0.0208424	0.97845	0.99995	no

ENSG00000143554	ENSG00000143554	SLC27A3	1:153746829-153752633	q1	q2	OK	0.338272	1.73088	2.35525	1.33252	0.01335	0.99995	no

ENSG00000143556	ENSG00000143556	S100A7	1:153430219-153433177	q1	q2	NOTEST	0	0	0	0	1	1	no

ENSG00000143569	ENSG00000143569	UBAP2L	1:154179181-154243986	q1	q2	OK	97.7588	104.682	0.0987214	0.134714	0.8059	0.99995	no

ENSG00000143570	ENSG00000143570	SLC39A1	1:153931574-153950164	q1	q2	OK	54.1041	54.2498	0.00387942	0.00776882	0.9928	0.99995	no

ENSG00000143575	ENSG00000143575	HAX1	1:154244986-154248351	q1	q2	OK	48.482	46.1483	-0.0711717	-0.157422	0.82545	0.99995	no

ENSG00000143578	ENSG00000143578	CREB3L4	1:153931574-153950164	q1	q2	OK	9.53022	7.3231	-0.380055	-0.200135	0.63265	0.99995	no

ENSG00000143590	ENSG00000143590	EFNA3	1:155036206-155060014	q1	q2	OK	6.52097	6.33814	-0.0410263	-0.0440762	0.9444	0.99995	no

ENSG00000143595	ENSG00000143595	AQP10	1:154293565-154297801	q1	q2	NOTEST	0.0881844	0.0505275	-0.803453	0	1	1	no

ENSG00000143603	ENSG00000143603	KCNN3	1:154669930-154842756	q1	q2	OK	1.36244	1.20975	-0.171481	-0.253725	0.65325	0.99995	no

ENSG00000143612	ENSG00000143612	C1orf43	1:154179181-154243986	q1	q2	OK	102.414	100.547	-0.0265374	-0.037249	0.95755	0.99995	no

ENSG00000143614	ENSG00000143614	GATAD2B	1:153777200-153895451	q1	q2	OK	0.298755	0.504992	0.757298	0.905218	0.1922	0.99995	no

ENSG00000143621	ENSG00000143621	ILF2	1:153634511-153643524	q1	q2	OK	236.371	241.7	0.0321641	0.0768066	0.91295	0.99995	no

ENSG00000143622	ENSG00000143622	RIT1	1:155867598-155881195	q1	q2	OK	23.3409	21.9008	-0.0918773	-0.153565	0.8052	0.99995	no

ENSG00000143624	ENSG00000143624	INTS3	1:153700542-153746555	q1	q2	OK	3.69618	1.31843	-1.48722	-2.09702	0.0031	0.99995	no

ENSG00000143627	ENSG00000143627	PKLR	1:155232658-155271225	q1	q2	NOTEST	0	0	0	0	1	1	no

ENSG00000143630	ENSG00000143630	HCN3	1:155232658-155271225	q1	q2	NOTEST	0	0	0	0	1	1	no

ENSG00000143631	ENSG00000143631	FLG	1:152140600-152339163	q1	q2	NOTEST	0	0	0	0	1	1	no

ENSG00000143632	ENSG00000143632	ACTA1	1:229566991-229569845	q1	q2	NOTEST	0.256617	0.168138	-0.609972	0	1	1	no

ENSG00000143633	ENSG00000143633	C1orf131	1:231359508-231376933	q1	q2	OK	12.6774	13.3878	0.0786628	0.122303	0.8494	0.99995	no

ENSG00000143641	ENSG00000143641	GALNT2	1:230193535-230417870	q1	q2	OK	50.109	44.8117	-0.161196	-0.389856	0.5657	0.99995	no

ENSG00000143643	ENSG00000143643	TTC13	1:231041988-231114621	q1	q2	OK	12.1465	11.9696	-0.0211636	-0.028181	0.95685	0.99995	no

ENSG00000143653	ENSG00000143653	SCCPDH	1:246887348-246931439	q1	q2	OK	28.6825	30.7001	0.0980693	0.23251	0.73735	0.99995	no

ENSG00000143669	ENSG00000143669	LYST	1:235824340-236046940	q1	q2	OK	2.074	1.97162	-0.073032	-0.0583683	0.874	0.99995	no

ENSG00000143674	ENSG00000143674	MLK4	1:233463513-233520894	q1	q2	NOTEST	0.194458	0.317903	0.70913	0	1	1	no

ENSG00000143702	ENSG00000143702	CEP170	1:243287729-243418650	q1	q2	OK	49.0583	56.3183	0.199106	0.297311	0.52835	0.99995	no

ENSG00000143727	ENSG00000143727	ACP1	2:217729-278283	q1	q2	OK	115.048	121.969	0.0842728	0.175285	0.7817	0.99995	no

ENSG00000143740	ENSG00000143740	SNAP47	1:227916239-227968927	q1	q2	OK	20.2212	18.0284	-0.1656	-0.277237	0.6864	0.99995	no

ENSG00000143742	ENSG00000143742	SRP9	1:225965530-225978168	q1	q2	OK	359.488	390.559	0.119596	0.277577	0.6873	0.99995	no

ENSG00000143748	ENSG00000143748	NVL	1:224407314-224518089	q1	q2	OK	20.4431	20.4916	0.00341529	0.0053419	0.99145	0.99995	no

ENSG00000143751	ENSG00000143751	SDE2	1:226170402-226187032	q1	q2	OK	7.58139	8.4362	0.15413	0.318432	0.65325	0.99995	no

ENSG00000143753	ENSG00000143753	DEGS1	1:224363457-224381143	q1	q2	OK	31.055	32.2154	0.0529268	0.11726	0.85995	0.99995	no

ENSG00000143756	ENSG00000143756	FBXO28	1:224301788-224349749	q1	q2	OK	14.8888	15.2355	0.0332074	0.0553521	0.9207	0.99995	no

ENSG00000143761	ENSG00000143761	ARF1	1:228270360-228286912	q1	q2	OK	144.602	137.491	-0.0727502	-0.175146	0.79375	0.99995	no

ENSG00000143768	ENSG00000143768	LEFTY2	1:226124110-226129189	q1	q2	NOTEST	0.0711408	0.0122121	-2.54237	0	1	1	no

ENSG00000143771	ENSG00000143771	CNIH4	1:224544551-224567161	q1	q2	OK	36.3645	43.2265	0.249388	0.435951	0.52825	0.99995	no

ENSG00000143772	ENSG00000143772	ITPKB	1:226819390-226927024	q1	q2	OK	14.3822	17.3572	0.271248	0.62566	0.3885	0.99995	no

ENSG00000143774	ENSG00000143774	GUK1	1:228327662-228336685	q1	q2	OK	58.7911	51.1482	-0.200914	-0.410822	0.53015	0.99995	no

ENSG00000143776	ENSG00000143776	CDC42BPA	1:227177565-227506175	q1	q2	OK	14.4475	15.0352	0.0575263	0.0674324	0.84845	0.99995	no

ENSG00000143786	ENSG00000143786	CNIH3	1:224572844-224928251	q1	q2	OK	2.80348	2.60595	-0.105409	-0.0226155	0.89355	0.99995	no

ENSG00000143793	ENSG00000143793	C1orf35	1:228288426-228293112	q1	q2	OK	19.6488	16.6849	-0.235896	-0.432882	0.51935	0.99995	no

ENSG00000143797	ENSG00000143797	MBOAT2	2:8992819-9143942	q1	q2	OK	31.9827	36.9126	0.206821	0.230569	0.68345	0.99995	no

ENSG00000143799	ENSG00000143799	PARP1	1:226548391-226595780	q1	q2	OK	136.007	138.899	0.0303635	0.0446848	0.9229	0.99995	no

ENSG00000143801	ENSG00000143801	PSEN2	1:227057884-227083806	q1	q2	OK	6.09137	6.19127	0.0234698	0.0320141	0.9572	0.99995	no

ENSG00000143811	ENSG00000143811	PYCR2	1:226073981-226111978	q1	q2	OK	32.0623	28.1125	-0.189668	-0.317916	0.5939	0.99995	no

ENSG00000143815	ENSG00000143815	LBR	1:225589203-225616627	q1	q2	OK	106.897	116.923	0.129341	0.221186	0.6632	0.99995	no

ENSG00000143816	ENSG00000143816	WNT9A	1:228106356-228135599	q1	q2	OK	0.711719	0.829908	0.221644	0.265082	0.6974	0.99995	no

ENSG00000143819	ENSG00000143819	EPHX1	1:225997793-226070069	q1	q2	OK	8.59223	7.50725	-0.194749	-0.192321	0.76355	0.99995	no

ENSG00000143839	ENSG00000143839	REN	1:204123943-204135465	q1	q2	NOTEST	0	0	0	0	1	1	no

ENSG00000143842	ENSG00000143842	SOX13	1:204042242-204096863	q1	q2	OK	30.6269	29.7767	-0.0406133	-0.0722892	0.9023	0.99995	no

ENSG00000143845	ENSG00000143845	ETNK2	1:204100189-204121307	q1	q2	OK	8.26945	8.30747	0.00661711	0.0112379	0.98505	0.99995	no

ENSG00000143847	ENSG00000143847	PPFIA4	1:202995625-203047868	q1	q2	OK	0.921082	0.383653	-1.26353	-0.762838	0.0517	0.99995	no

ENSG00000143850	ENSG00000143850	PLEKHA6	1:204187978-204346793	q1	q2	OK	7.79223	7.8955	0.0189952	0.0225531	0.952	0.99995	no

ENSG00000143851	ENSG00000143851	PTPN7	1:202116140-202130716	q1	q2	NOTEST	0.0814288	0.0478703	-0.766408	0	1	1	no

ENSG00000143858	ENSG00000143858	SYT2	1:202317826-202679545	q1	q2	OK	3.99376	4.4947	0.170475	0.244355	0.7357	0.99995	no

ENSG00000143862	ENSG00000143862	ARL8A	1:202091985-202113869	q1	q2	OK	24.8292	22.4296	-0.146633	-0.260405	0.6506	0.99995	no

ENSG00000143867	ENSG00000143867	OSR1	2:19551245-19558414	q1	q2	OK	3.79063	3.04093	-0.317925	-0.416767	0.54515	0.99995	no

ENSG00000143869	ENSG00000143869	GDF7	2:20866423-20873418	q1	q2	NOTEST	0.0365813	0.0240538	-0.60484	0	1	1	no

ENSG00000143870	ENSG00000143870	PDIA6	2:10861774-10978103	q1	q2	OK	198.655	201.379	0.0196522	0.0459539	0.9468	0.99995	no

ENSG00000143878	ENSG00000143878	RHOB	2:20646834-20649200	q1	q2	OK	26.9267	27.8334	0.0477795	0.109534	0.87735	0.99995	no

ENSG00000143882	ENSG00000143882	ATP6V1C2	2:10861774-10978103	q1	q2	OK	1.67007	1.80788	0.11439	0.0290358	0.9573	0.99995	no

ENSG00000143889	ENSG00000143889	HNRNPLL	2:38789119-38830728	q1	q2	OK	40.9991	43.4459	0.083625	0.167213	0.78295	0.99995	no

ENSG00000143891	ENSG00000143891	GALM	2:38893051-38968379	q1	q2	OK	5.83906	4.78766	-0.286414	-0.409622	0.5173	0.99995	no

ENSG00000143919	ENSG00000143919	CAMKMT	2:44589088-44999731	q1	q2	OK	4.16146	3.61195	-0.204315	-0.240386	0.7009	0.99995	no

ENSG00000143921	ENSG00000143921	ABCG8	2:44066102-44105605	q1	q2	NOTEST	0.00962651	0.0100094	0.056267	0	1	1	no

ENSG00000143924	ENSG00000143924	EML4	2:42370377-42559688	q1	q2	OK	35.6438	39.1723	0.136182	0.281411	0.6173	0.99995	no

ENSG00000143933	ENSG00000143933	CALM2	2:47126413-47403740	q1	q2	OK	500.489	533.131	0.0911507	0.169625	0.8058	0.99995	no

ENSG00000143942	ENSG00000143942	CHAC2	2:53759809-54087297	q1	q2	OK	6.52153	7.91908	0.280123	0.1875	0.79665	0.99995	no

ENSG00000143947	ENSG00000143947	RPS27A	2:55401926-55462989	q1	q2	OK	623.396	623.118	-0.000645514	-0.00143421	0.99785	0.99995	no

ENSG00000143951	ENSG00000143951	WDPCP	2:63348517-64054977	q1	q2	OK	1.69754	2.47937	0.546524	0.168764	0.522	0.99995	no

ENSG00000143952	ENSG00000143952	VPS54	2:64119279-64246206	q1	q2	OK	14.1308	15.1885	0.104137	0.224229	0.7453	0.99995	no

ENSG00000143954	ENSG00000143954	REG3G	2:79252811-79255631	q1	q2	NOTEST	0	0	0	0	1	1	no

ENSG00000143970	ENSG00000143970	ASXL2	2:25956621-26101385	q1	q2	OK	5.38381	5.64768	0.0690303	0.156208	0.82095	0.99995	no

ENSG00000143971	ENSG00000143971	ETAA1	2:67624450-67637677	q1	q2	OK	12.8853	14.2523	0.145477	0.261028	0.64995	0.99995	no

ENSG00000143977	ENSG00000143977	SNRPG	2:70508493-70520903	q1	q2	OK	134.434	157.869	0.231831	0.540643	0.4371	0.99995	no

ENSG00000143994	ENSG00000143994	ABHD1	2:27346681-27357543	q1	q2	NOTEST	0	0.164998	inf	0	1	1	no

ENSG00000143995	ENSG00000143995	MEIS1	2:66653866-66801001	q1	q2	OK	47.6749	56.6844	0.249722	0.485057	0.42235	0.99995	no

ENSG00000144010	ENSG00000144010	TRIM43B	2:96142714-96150479	q1	q2	NOTEST	0.0438936	0	#NAME?	0	1	1	no

ENSG00000144015	ENSG00000144015	TRIM43	2:96257765-96265526	q1	q2	NOTEST	0	0.0166331	inf	0	1	1	no

ENSG00000144021	ENSG00000144021	CIAO1	2:96931869-96939087	q1	q2	OK	44.2441	42.0812	-0.0723098	-0.149446	0.81785	0.99995	no

ENSG00000144026	ENSG00000144026	ZNF514	2:95752951-95831158	q1	q2	OK	14.3626	14.7176	0.0352251	0.0343198	0.94925	0.99995	no

ENSG00000144028	ENSG00000144028	SNRNP200	2:96940073-96971297	q1	q2	OK	100.405	95.51	-0.072104	-0.12597	0.8393	0.99995	no

ENSG00000144029	ENSG00000144029	MRPS5	2:95752951-95831158	q1	q2	OK	43.698	46.2075	0.0805621	0.133108	0.8445	0.99995	no

ENSG00000144031	ENSG00000144031	ANKRD53	2:71163011-71306935	q1	q2	NOTEST	0	0	0	0	1	1	no

ENSG00000144034	ENSG00000144034	TPRKB	2:73956230-73964527	q1	q2	OK	51.3861	59.3414	0.20766	0.428736	0.5533	0.99995	no

ENSG00000144035	ENSG00000144035	NAT8	2:73867958-73869520	q1	q2	NOTEST	0.0671091	0.0658472	-0.0273858	0	1	1	no

ENSG00000144036	ENSG00000144036	EXOC6B	2:72403112-73053170	q1	q2	OK	10.4301	11.3486	0.121761	0.238029	0.6779	0.99995	no

ENSG00000144040	ENSG00000144040	SFXN5	2:73169164-73383849	q1	q2	OK	12.8633	11.4321	-0.170166	-0.238947	0.6129	0.99995	no

ENSG00000144043	ENSG00000144043	TEX261	2:71163011-71306935	q1	q2	NOTEST	0	0	0	0	1	1	no

ENSG00000144045	ENSG00000144045	DQX1	2:74745257-74753463	q1	q2	NOTEST	0.279997	0.156594	-0.838385	0	1	1	no

ENSG00000144048	ENSG00000144048	DUSP11	2:73989310-74007284	q1	q2	OK	11.9737	12.969	0.115195	0.211258	0.76335	0.99995	no

ENSG00000144057	ENSG00000144057	ST6GAL2	2:107418055-107503564	q1	q2	OK	3.07723	2.94253	-0.0645749	-0.115269	0.8605	0.99995	no

ENSG00000144061	ENSG00000144061	NPHP1	2:110879887-110962643	q1	q2	OK	5.91171	4.58083	-0.367965	-0.34604	0.59005	0.99995	no

ENSG00000144063	ENSG00000144063	MALL	2:110841446-110874143	q1	q2	NOTEST	0	0	0	0	1	1	no

ENSG00000144115	ENSG00000144115	THNSL2	2:88469834-88486146	q1	q2	OK	0.901354	0.627142	-0.523302	-0.428468	0.42555	0.99995	no

ENSG00000144118	ENSG00000144118	RALB	2:120997639-121052289	q1	q2	OK	39.7145	39.0932	-0.0227491	-0.0498864	0.94205	0.99995	no

ENSG00000144119	ENSG00000144119	C1QL2	2:119913818-119916465	q1	q2	NOTEST	0.0264538	0.0401435	0.601687	0	1	1	no

ENSG00000144120	ENSG00000144120	TMEM177	2:120436742-120444083	q1	q2	OK	10.4659	11.0842	0.0828159	0.131071	0.8569	0.99995	no

ENSG00000144130	ENSG00000144130	NT5DC4	2:113479062-113522254	q1	q2	NOTEST	0.0360624	0.034382	-0.0688439	0	1	1	no

ENSG00000144134	ENSG00000144134	RABL2A	2:114384805-114400973	q1	q2	OK	13.5092	13.5837	0.00794019	0.0124697	0.98515	0.99995	no

ENSG00000144136	ENSG00000144136	SLC20A1	2:113403433-113421404	q1	q2	OK	47.3976	54.9626	0.213636	0.433142	0.438	0.99995	no

ENSG00000144140	ENSG00000144140	RP11-143M1.7	9:52451-88826	q1	q2	NOTEST	0	0	0	0	1	1	no

ENSG00000144152	ENSG00000144152	FBLN7	2:112895961-112945791	q1	q2	OK	5.14649	4.23683	-0.280602	-0.397503	0.5593	0.99995	no

ENSG00000144158	ENSG00000144158	RP11-395L14.17	2:114415484-114416252	q1	q2	OK	10.7273	12.648	0.23763	0.344463	0.621	0.99995	no

ENSG00000144161	ENSG00000144161	ZC3H8	2:112969101-113012713	q1	q2	OK	8.3236	9.20535	0.145264	0.158374	0.75355	0.99995	no

ENSG00000144182	ENSG00000144182	LIPT1	2:99613723-100106497	q1	q2	OK	6.00779	5.849	-0.0386435	-0.0101318	0.9814	0.99995	no

ENSG00000144188	ENSG00000144188	TRIM43CP	2:97691717-97697373	q1	q2	NOTEST	0	0	0	0	1	1	no

ENSG00000144191	ENSG00000144191	CNGA3	2:98947851-99015064	q1	q2	OK	0.473538	0.602871	0.34837	0.301092	0.63125	0.99995	no

ENSG00000144199	ENSG00000144199	FAHD2B	2:97749319-97760619	q1	q2	OK	6.07775	5.76918	-0.0751721	-0.0998864	0.88265	0.99995	no

ENSG00000144214	ENSG00000144214	LYG1	2:99613723-100106497	q1	q2	OK	1.79458	1.53752	-0.223038	-0.0290767	0.895	0.99995	no

ENSG00000144218	ENSG00000144218	AFF3	2:100162322-100759201	q1	q2	OK	6.32928	8.40755	0.409643	0.453647	0.2189	0.99995	no

ENSG00000144224	ENSG00000144224	UBXN4	2:136499188-136542625	q1	q2	OK	73.6744	81.1748	0.139868	0.290537	0.64705	0.99995	no

ENSG00000144227	ENSG00000144227	NXPH2	2:139428341-139537918	q1	q2	OK	3.76568	4.67515	0.312101	0.381817	0.58075	0.99995	no

ENSG00000144228	ENSG00000144228	SPOPL	2:139259370-139332028	q1	q2	OK	6.01914	6.2165	0.0465444	0.0711724	0.8858	0.99995	no

ENSG00000144229	ENSG00000144229	THSD7B	2:137523114-138435287	q1	q2	OK	0.835078	0.958098	0.198262	0.183017	0.70615	0.99995	no

ENSG00000144230	ENSG00000144230	GPR17	2:128395955-128439360	q1	q2	OK	0.683178	0.773978	0.180032	0.0867617	0.87255	0.99995	no

ENSG00000144231	ENSG00000144231	POLR2D	2:128603839-128615731	q1	q2	OK	28.6466	29.9145	0.0624819	0.14144	0.84255	0.99995	no

ENSG00000144233	ENSG00000144233	AMMECR1L	2:128619203-128643496	q1	q2	OK	15.2239	15.6351	0.0384494	0.0914047	0.89625	0.99995	no

ENSG00000144278	ENSG00000144278	GALNT13	2:154728425-155313950	q1	q2	OK	3.25503	3.74296	0.201508	0.224944	0.641	0.99995	no

ENSG00000144283	ENSG00000144283	PKP4	2:159313475-159610287	q1	q2	OK	23.0834	25.5871	0.148559	0.224483	0.66445	0.99995	no

ENSG00000144285	ENSG00000144285	SCN1A	2:166813908-167232503	q1	q2	OK	2.51565	3.11944	0.310352	0.159592	0.6699	0.99995	no

ENSG00000144290	ENSG00000144290	SLC4A10	2:162272604-162841792	q1	q2	NOTEST	0.0459959	0.0260428	-0.820619	0	1	1	no

ENSG00000144306	ENSG00000144306	SCRN3	2:175260457-175294303	q1	q2	OK	21.7556	20.0667	-0.116586	-0.187478	0.7762	0.99995	no

ENSG00000144320	ENSG00000144320	KIAA1715	2:176788619-176867567	q1	q2	OK	16.0221	16.9307	0.0795768	0.111036	0.81905	0.99995	no

ENSG00000144331	ENSG00000144331	ZNF385B	2:180306708-180726232	q1	q2	NOTEST	0.349139	0.17827	-0.969743	0	1	1	no

ENSG00000144339	ENSG00000144339	TMEFF2	2:192699027-193060435	q1	q2	OK	2.90452	3.07354	0.0816027	0.0652089	0.88885	0.99995	no

ENSG00000144354	ENSG00000144354	CDCA7	2:174202946-174233725	q1	q2	OK	24.7993	28.7885	0.215194	0.49304	0.4722	0.99995	no

ENSG00000144355	ENSG00000144355	DLX1	2:172949467-172954405	q1	q2	OK	2.93272	3.24023	0.143858	0.169718	0.78145	0.99995	no

ENSG00000144357	ENSG00000144357	UBR3	2:170683967-170940641	q1	q2	OK	14.6856	15.2948	0.0586351	0.0877288	0.8569	0.99995	no

ENSG00000144362	ENSG00000144362	PHOSPHO2	2:170550974-170633499	q1	q2	OK	4.25642	5.11349	0.264668	0.13249	0.86845	0.99995	no

ENSG00000144366	ENSG00000144366	GULP1	2:189156395-189460653	q1	q2	OK	55.1114	62.4822	0.181094	0.306975	0.55705	0.99995	no

ENSG00000144369	ENSG00000144369	FAM171B	2:187454791-187630685	q1	q2	OK	5.54017	6.10187	0.139322	0.130032	0.8533	0.99995	no

ENSG00000144381	ENSG00000144381	HSPD1	2:198351304-198418423	q1	q2	OK	232.316	254.703	0.132727	0.215151	0.7067	0.99995	no

ENSG00000144395	ENSG00000144395	CCDC150	2:197504277-197664449	q1	q2	OK	17.3605	16.2634	-0.0941821	-0.0797973	0.8662	0.99995	no

ENSG00000144401	ENSG00000144401	METTL21A	2:208104373-208490652	q1	q2	OK	21.0825	20.9953	-0.00598128	-0.00536387	0.9945	0.99995	no

ENSG00000144406	ENSG00000144406	UNC80	2:210636716-210864024	q1	q2	OK	1.24271	1.1264	-0.141768	-0.165625	0.7545	0.99995	no

ENSG00000144407	ENSG00000144407	PTH2R	2:209224437-209719227	q1	q2	NOTEST	0.366423	0.243036	-0.592338	0	1	1	no

ENSG00000144410	ENSG00000144410	CPO	2:207804277-207834198	q1	q2	OK	1.60977	2.5121	0.642042	0.616044	0.39505	0.99995	no

ENSG00000144426	ENSG00000144426	NBEAL1	2:203879601-204091101	q1	q2	OK	3.36035	3.43838	0.0331182	0.0278028	0.94485	0.99995	no

ENSG00000144445	ENSG00000144445	KANSL1L	2:210867288-211036115	q1	q2	OK	10.3038	10.7678	0.0635554	0.0692527	0.8941	0.99995	no

ENSG00000144451	ENSG00000144451	SPAG16	2:214149112-215548970	q1	q2	OK	28.7177	25.9823	-0.144411	-0.254967	0.7091	0.99995	no

ENSG00000144452	ENSG00000144452	ABCA12	2:215674952-216003151	q1	q2	NOTEST	0.0382062	0.0372917	-0.0349512	0	1	1	no

ENSG00000144455	ENSG00000144455	SUMF1	3:3742497-4508965	q1	q2	OK	7.14275	6.66479	-0.0999203	-0.0270707	0.9539	0.99995	no

ENSG00000144460	ENSG00000144460	NYAP2	2:226265363-226518734	q1	q2	NOTEST	0.107569	0.0992996	-0.115399	0	1	1	no

ENSG00000144468	ENSG00000144468	RHBDD1	2:227700296-227863931	q1	q2	OK	4.63314	6.11415	0.400163	0.387041	0.34555	0.99995	no

ENSG00000144476	ENSG00000144476	ACKR3	2:237476429-237491001	q1	q2	OK	20.3138	17.5416	-0.211681	-0.400748	0.55945	0.99995	no

ENSG00000144481	ENSG00000144481	TRPM8	2:234826042-234928166	q1	q2	NOTEST	0.180998	0.129638	-0.481478	0	1	1	no

ENSG00000144485	ENSG00000144485	HES6	2:239140324-239198743	q1	q2	OK	101.351	90.0102	-0.171201	-0.369156	0.57015	0.99995	no

ENSG00000144488	ENSG00000144488	ESPNL	2:239008797-239041928	q1	q2	NOTEST	0.0827316	0.0893936	0.111733	0	1	1	no

ENSG00000144504	ENSG00000144504	ANKMY1	2:241418838-241520789	q1	q2	OK	3.37611	4.58367	0.441144	0.310821	0.45105	0.99995	no

ENSG00000144524	ENSG00000144524	COPS7B	2:232597134-232673963	q1	q2	OK	35.4169	33.562	-0.0776079	-0.131852	0.84015	0.99995	no

ENSG00000144535	ENSG00000144535	DIS3L2	2:232825954-233209060	q1	q2	OK	12.5175	11.3589	-0.140132	-0.216592	0.6979	0.99995	no

ENSG00000144550	ENSG00000144550	CPNE9	3:9745490-9771592	q1	q2	NOTEST	0.128728	0.0762885	-0.754787	0	1	1	no

ENSG00000144554	ENSG00000144554	FANCD2	3:10068097-10149915	q1	q2	OK	29.674	23.9069	-0.311777	-0.369684	0.4029	0.99995	no

ENSG00000144559	ENSG00000144559	TAMM41	3:11831915-11888393	q1	q2	OK	5.20988	5.48863	0.0751965	0.0946303	0.894	0.99995	no

ENSG00000144560	ENSG00000144560	VGLL4	3:11313994-11766453	q1	q2	OK	31.6197	33.1145	0.0666352	0.128108	0.84275	0.99995	no

ENSG00000144566	ENSG00000144566	RAB5A	3:19988570-20053822	q1	q2	OK	35.7856	33.7531	-0.0843565	-0.168584	0.79155	0.99995	no

ENSG00000144567	ENSG00000144567	FAM134A	2:220036618-220050201	q1	q2	OK	34.3789	30.3973	-0.177581	-0.266938	0.66805	0.99995	no

ENSG00000144579	ENSG00000144579	CTDSP1	2:219262978-219270664	q1	q2	OK	16.8836	13.4735	-0.325499	-0.558054	0.3922	0.99995	no

ENSG00000144580	ENSG00000144580	RQCD1	2:219433302-219461803	q1	q2	OK	46.3844	48.0095	0.0496794	0.0883818	0.87525	0.99995	no

ENSG00000144583	ENSG00000144583	Mar-04	2:217122587-217236750	q1	q2	NOTEST	0.264245	0.219794	-0.265726	0	1	1	no

ENSG00000144589	ENSG00000144589	STK11IP	2:220462581-220481173	q1	q2	OK	14.7282	13.2176	-0.156126	-0.208201	0.73135	0.99995	no

ENSG00000144591	ENSG00000144591	GMPPA	2:220299567-220403494	q1	q2	OK	14.9987	12.746	-0.234794	-0.264218	0.71115	0.99995	no

ENSG00000144596	ENSG00000144596	GRIP2	3:14444075-14583588	q1	q2	OK	1.00613	0.885494	-0.184268	-0.105589	0.90815	0.99995	no

ENSG00000144597	ENSG00000144597	EAF1	3:15422781-15563258	q1	q2	OK	12.0453	13.2686	0.139546	0.244443	0.6939	0.99995	no

ENSG00000144619	ENSG00000144619	CNTN4	3:2140496-3102829	q1	q2	OK	4.59347	5.1915	0.176566	0.239659	0.6569	0.99995	no

ENSG00000144635	ENSG00000144635	DYNC1LI1	3:32567462-32612366	q1	q2	OK	36.6964	40.3795	0.137982	0.290413	0.63755	0.99995	no

ENSG00000144642	ENSG00000144642	RBMS3	3:29305684-30051886	q1	q2	OK	3.51845	5.50209	0.645037	0.827577	0.12055	0.99995	no

ENSG00000144644	ENSG00000144644	GADL1	3:30767691-30936257	q1	q2	NOTEST	0	0	0	0	1	1	no

ENSG00000144645	ENSG00000144645	OSBPL10	3:31699381-32119072	q1	q2	OK	1.99946	1.66848	-0.26107	-0.174695	0.6201	0.99995	no

ENSG00000144647	ENSG00000144647	POMGNT2	3:43120723-43147568	q1	q2	OK	10.7381	9.07024	-0.243531	-0.47392	0.4937	0.99995	no

ENSG00000144648	ENSG00000144648	ACKR2	3:42846243-43101703	q1	q2	NOTEST	0.170853	0.128406	-0.412044	0	1	1	no

ENSG00000144649	ENSG00000144649	FAM198A	3:42846243-43101703	q1	q2	NOTEST	0.177817	0.312008	0.811193	0	1	1	no

ENSG00000144655	ENSG00000144655	CSRNP1	3:39183345-39196053	q1	q2	OK	1.58966	1.59413	0.00405844	0.00532942	0.9942	0.99995	no

ENSG00000144659	ENSG00000144659	SLC25A38	3:39424838-39438842	q1	q2	OK	14.9491	14.6566	-0.0285128	-0.058121	0.93015	0.99995	no

ENSG00000144668	ENSG00000144668	ITGA9	3:37493605-37903271	q1	q2	OK	1.17785	1.17846	0.000750504	0.000950531	0.9985	0.99995	no

ENSG00000144671	ENSG00000144671	SLC22A14	3:38323784-38360066	q1	q2	NOTEST	0.110474	0.0303072	-1.86597	0	1	1	no

ENSG00000144674	ENSG00000144674	GOLGA4	3:37283279-37408242	q1	q2	OK	20.7336	20.9499	0.0149725	0.025759	0.96125	0.99995	no

ENSG00000144677	ENSG00000144677	CTDSPL	3:37903450-38025960	q1	q2	OK	7.61629	7.41381	-0.0388735	-0.0632706	0.90715	0.99995	no

ENSG00000144681	ENSG00000144681	STAC	3:36421835-36589499	q1	q2	OK	0.498872	0.592881	0.249075	0.170168	0.6825	0.99995	no

ENSG00000144711	ENSG00000144711	IQSEC1	3:12938718-13114617	q1	q2	OK	15.7389	14.1319	-0.15538	-0.330199	0.59955	0.99995	no

ENSG00000144712	ENSG00000144712	CAND2	3:12837970-12913415	q1	q2	OK	2.90861	2.51226	-0.211346	-0.0745208	0.9317	0.99995	no

ENSG00000144713	ENSG00000144713	RPL32	3:12837970-12913415	q1	q2	OK	806.478	780.924	-0.0464528	-0.103173	0.88455	0.99995	no

ENSG00000144724	ENSG00000144724	PTPRG	3:61547242-62355005	q1	q2	OK	19.9868	22.0596	0.142355	0.265743	0.6719	0.99995	no

ENSG00000144730	ENSG00000144730	IL17RD	3:57124009-57204334	q1	q2	OK	30.8491	39.9463	0.372836	0.720913	0.1852	0.99995	no

ENSG00000144736	ENSG00000144736	SHQ1	3:72798427-72911065	q1	q2	OK	8.20412	8.63775	0.0743064	0.134036	0.83105	0.99995	no

ENSG00000144741	ENSG00000144741	SLC25A26	3:66119284-66551687	q1	q2	OK	6.87439	6.05711	-0.182602	-0.0655556	0.8708	0.99995	no

ENSG00000144744	ENSG00000144744	UBA3	3:69024364-69129559	q1	q2	OK	40.3408	44.661	0.146777	0.222999	0.70125	0.99995	no

ENSG00000144746	ENSG00000144746	ARL6IP5	3:69134094-69155217	q1	q2	OK	44.984	45.8293	0.0268568	0.0630439	0.9272	0.99995	no

ENSG00000144747	ENSG00000144747	TMF1	3:69024364-69129559	q1	q2	OK	9.25422	10.3546	0.162092	0.113109	0.75655	0.99995	no

ENSG00000144749	ENSG00000144749	LRIG1	3:66119284-66551687	q1	q2	OK	93.7236	91.5227	-0.0342823	-0.0545836	0.919	0.99995	no

ENSG00000144771	ENSG00000144771	LRTM1	3:54156573-55108584	q1	q2	NOTEST	0.0212263	0	#NAME?	0	1	1	no

ENSG00000144785	ENSG00000144785	RP11-977G19.10	12:56665482-56727837	q1	q2	OK	0	0.990125	inf	#NAME?	0.12895	0.99995	no

ENSG00000144791	ENSG00000144791	LIMD1	3:45596885-45786916	q1	q2	OK	7.50191	8.31424	0.148327	0.107573	0.7919	0.99995	no

ENSG00000144792	ENSG00000144792	ZNF660	3:44596684-44727145	q1	q2	OK	7.09354	7.06327	-0.00616816	-0.00472169	0.99155	0.99995	no

ENSG00000144802	ENSG00000144802	NFKBIZ	3:101498045-101579866	q1	q2	OK	1.34843	1.20911	-0.157338	-0.051954	0.89615	0.99995	no

ENSG00000144810	ENSG00000144810	COL8A1	3:99357318-99518070	q1	q2	OK	17.4703	15.3389	-0.187714	-0.306754	0.5478	0.99995	no

ENSG00000144815	ENSG00000144815	NXPE3	3:101498045-101579866	q1	q2	OK	11.5591	11.7171	0.0195857	0.0343578	0.9554	0.99995	no

ENSG00000144820	ENSG00000144820	GPR128	3:100328432-100414323	q1	q2	NOTEST	0.0139676	0.0137629	-0.0212908	0	1	1	no

ENSG00000144821	ENSG00000144821	MYH15	3:108099215-108248169	q1	q2	NOTEST	0.172096	0.0893457	-0.945741	0	1	1	no

ENSG00000144824	ENSG00000144824	PHLDB2	3:111393522-111695364	q1	q2	OK	43.1487	53.4157	0.307949	0.570599	0.32525	0.99995	no

ENSG00000144827	ENSG00000144827	ABHD10	3:111697856-111712210	q1	q2	OK	38.9104	41.9345	0.107979	0.221675	0.73565	0.99995	no

ENSG00000144834	ENSG00000144834	TAGLN3	3:111717510-111732734	q1	q2	OK	90.1742	94.7236	0.0710091	0.170141	0.81255	0.99995	no

ENSG00000144837	ENSG00000144837	PLA1A	3:119316688-119348658	q1	q2	NOTEST	0	0	0	0	1	1	no

ENSG00000144840	ENSG00000144840	RABL3	3:120405527-120501916	q1	q2	OK	7.71781	10.5204	0.446933	0.591333	0.32165	0.99995	no

ENSG00000144843	ENSG00000144843	ADPRH	3:119298058-119308792	q1	q2	NOTEST	0.242797	0.288726	0.249951	0	1	1	no

ENSG00000144847	ENSG00000144847	IGSF11	3:118619403-118864915	q1	q2	OK	0.938515	0.832699	-0.172584	-0.168053	0.74975	0.99995	no

ENSG00000144848	ENSG00000144848	ATG3	3:112251355-112304424	q1	q2	OK	23.3231	26.681	0.194049	0.270357	0.68585	0.99995	no

ENSG00000144852	ENSG00000144852	NR1I2	3:119499330-119537332	q1	q2	NOTEST	0	0.00916473	inf	0	1	1	no

ENSG00000144857	ENSG00000144857	BOC	3:112929849-113160457	q1	q2	OK	54.7258	46.6873	-0.22919	-0.353205	0.4607	0.99995	no

ENSG00000144867	ENSG00000144867	SRPRB	3:133502876-133614680	q1	q2	OK	21.8553	24.0681	0.139137	0.186392	0.7637	0.99995	no

ENSG00000144868	ENSG00000144868	TMEM108	3:132757234-133116636	q1	q2	OK	0.600516	0.888489	0.565152	0.39934	0.4038	0.99995	no

ENSG00000144891	ENSG00000144891	AGTR1	3:148415570-148460795	q1	q2	OK	1.09374	1.86012	0.76612	0.883471	0.18095	0.99995	no

ENSG00000144893	ENSG00000144893	MED12L	3:150803483-151176497	q1	q2	OK	1.55028	1.71894	0.148998	0.117694	0.74	0.99995	no

ENSG00000144895	ENSG00000144895	EIF2A	3:150259780-150348222	q1	q2	OK	50.5717	54.6753	0.112558	0.112667	0.85305	0.99995	no

ENSG00000144908	ENSG00000144908	ALDH1L1	3:125822411-125929012	q1	q2	NOTEST	0.340724	0.0911079	-1.90295	0	1	1	no

ENSG00000144909	ENSG00000144909	OSBPL11	3:125247701-125313934	q1	q2	OK	13.0809	14.4725	0.145849	0.337984	0.62765	0.99995	no

ENSG00000144935	ENSG00000144935	TRPC1	3:142442915-142526730	q1	q2	OK	7.30112	7.43236	0.0257022	0.0267627	0.9509	0.99995	no

ENSG00000144959	ENSG00000144959	NCEH1	3:172348038-172429008	q1	q2	OK	1.06004	1.14951	0.116895	0.112031	0.83475	0.99995	no

ENSG00000144962	ENSG00000144962	SPATA16	3:172607147-172859058	q1	q2	NOTEST	0	0	0	0	1	1	no

ENSG00000145002	ENSG00000145002	FAM86B2	8:12282912-12293915	q1	q2	NOTEST	0.103621	0.227831	1.13665	0	1	1	no

ENSG00000145012	ENSG00000145012	LPP	3:187868993-188608460	q1	q2	OK	4.14478	5.28053	0.349385	0.352914	0.23695	0.99995	no

ENSG00000145014	ENSG00000145014	TMEM44	3:194304739-194393206	q1	q2	OK	23.8182	23.8413	0.00139488	0.00180852	0.99795	0.99995	no

ENSG00000145016	ENSG00000145016	KIAA0226	3:197398263-197514467	q1	q2	OK	10.4477	9.36565	-0.15774	-0.0941435	0.76115	0.99995	no

ENSG00000145020	ENSG00000145020	AMT	3:49454210-49460186	q1	q2	OK	6.16758	5.42311	-0.185584	-0.204596	0.72665	0.99995	no

ENSG00000145022	ENSG00000145022	TCTA	3:49396577-49453908	q1	q2	OK	7.56725	6.97718	-0.117126	-0.0419698	0.964	0.99995	no

ENSG00000145029	ENSG00000145029	NICN1	3:49460378-49466759	q1	q2	OK	10.6882	10.7459	0.00776407	0.0133861	0.9846	0.99995	no

ENSG00000145040	ENSG00000145040	UCN2	3:48555116-48601206	q1	q2	NOTEST	0.338549	0.228383	-0.567913	0	1	1	no

ENSG00000145041	ENSG00000145041	VPRBP	3:51428730-51534010	q1	q2	NOTEST	0.227327	0.0290076	-2.97027	0	1	1	no

ENSG00000145050	ENSG00000145050	MANF	3:51422477-51426828	q1	q2	NOTEST	0.100139	0	#NAME?	0	1	1	no

ENSG00000145063	ENSG00000145063	AC062028.1	2:11239976-11272947	q1	q2	NOTEST	0	0	0	0	1	1	no

ENSG00000145075	ENSG00000145075	CCDC39	3:180319917-180700541	q1	q2	OK	1.31606	1.69965	0.369008	0.133656	0.7869	0.99995	no

ENSG00000145087	ENSG00000145087	STXBP5L	3:120626918-121143608	q1	q2	OK	2.42831	2.55744	0.0747439	0.11884	0.8569	0.99995	no

ENSG00000145088	ENSG00000145088	EAF2	3:121554029-121605373	q1	q2	OK	2.59527	2.63894	0.0240759	0.0259745	0.9705	0.99995	no

ENSG00000145103	ENSG00000145103	ILDR1	3:121706169-121741051	q1	q2	NOTEST	0	0.0100338	inf	0	1	1	no

ENSG00000145107	ENSG00000145107	TM4SF19	3:195938357-196065374	q1	q2	NOTEST	0.0867996	0.134474	0.631566	0	1	1	no

ENSG00000145113	ENSG00000145113	MUC4	3:195473635-195539148	q1	q2	NOTEST	0.0575846	0.039163	-0.55619	0	1	1	no

ENSG00000145147	ENSG00000145147	SLIT2	4:20254882-20622184	q1	q2	OK	34.628	43.0393	0.313717	0.63444	0.2639	0.99995	no

ENSG00000145191	ENSG00000145191	EIF2B5	3:183851806-184402546	q1	q2	OK	24.4095	25.1976	0.0458425	0.0113472	0.952	0.99995	no

ENSG00000145192	ENSG00000145192	AHSG	3:186330711-186339107	q1	q2	NOTEST	0.098941	0.0691153	-0.517562	0	1	1	no

ENSG00000145194	ENSG00000145194	ECE2	3:183851806-184402546	q1	q2	OK	2.10697	1.75946	-0.260037	-0.0240063	0.87735	0.99995	no

ENSG00000145198	ENSG00000145198	VWA5B2	3:183851806-184402546	q1	q2	OK	0.421531	0.853601	1.01792	0.0437141	0.2816	0.99995	no

ENSG00000145214	ENSG00000145214	DGKQ	4:952674-998316	q1	q2	OK	5.89328	4.84239	-0.283351	-0.371734	0.46435	0.99995	no

ENSG00000145216	ENSG00000145216	FIP1L1	4:54243809-55164414	q1	q2	OK	35.0538	36.5245	0.0592919	0.0549478	0.9342	0.99995	no

ENSG00000145217	ENSG00000145217	SLC26A1	4:952674-998316	q1	q2	OK	0.853093	0.446679	-0.933464	-0.369571	0.4598	0.99995	no

ENSG00000145220	ENSG00000145220	LYAR	4:4269427-4291896	q1	q2	OK	14.4686	17.6326	0.285317	0.519993	0.4536	0.99995	no

ENSG00000145241	ENSG00000145241	CENPC	4:68337520-68411324	q1	q2	OK	10.4585	11.253	0.105633	0.181729	0.76665	0.99995	no

ENSG00000145242	ENSG00000145242	EPHA5	4:66185280-66563747	q1	q2	NOTEST	0.319706	0.353725	0.145879	0	1	1	no

ENSG00000145244	ENSG00000145244	CORIN	4:47596014-47840123	q1	q2	NOTEST	0.156142	0.189317	0.277952	0	1	1	no

ENSG00000145246	ENSG00000145246	ATP10D	4:47487304-47595503	q1	q2	OK	4.2077	4.23602	0.00967759	0.00932694	0.9837	0.99995	no

ENSG00000145247	ENSG00000145247	OCIAD2	4:48887035-48908954	q1	q2	OK	1.77487	1.04126	-0.769389	-0.40904	0.3487	0.99995	no

ENSG00000145248	ENSG00000145248	SLC10A4	4:48485359-48491213	q1	q2	OK	2.15502	2.50933	0.219603	0.279136	0.6823	0.99995	no

ENSG00000145283	ENSG00000145283	SLC10A6	4:87744620-87770416	q1	q2	NOTEST	0	0	0	0	1	1	no

ENSG00000145284	ENSG00000145284	SCD5	4:83550691-83720010	q1	q2	OK	43.296	41.2752	-0.0689564	-0.169263	0.8042	0.99995	no

ENSG00000145287	ENSG00000145287	PLAC8	4:84011200-84058228	q1	q2	NOTEST	0.0939648	0.0939439	-0.000319891	0	1	1	no

ENSG00000145293	ENSG00000145293	ENOPH1	4:83351714-83382244	q1	q2	OK	38.7577	41.066	0.0834622	0.196351	0.7805	0.99995	no

ENSG00000145309	ENSG00000145309	CABS1	4:71200682-71202833	q1	q2	NOTEST	0.0742014	0.107455	0.53422	0	1	1	no

ENSG00000145321	ENSG00000145321	GC	4:72607409-72669758	q1	q2	NOTEST	0	0	0	0	1	1	no

ENSG00000145331	ENSG00000145331	TRMT10A	4:100467865-100547070	q1	q2	OK	8.98154	9.79163	0.124586	0.0782474	0.91055	0.99995	no

ENSG00000145332	ENSG00000145332	KLHL8	4:88081254-88161466	q1	q2	OK	9.08213	10.1564	0.161281	0.331629	0.6021	0.99995	no

ENSG00000145335	ENSG00000145335	SNCA	4:90472506-90763129	q1	q2	OK	7.51182	9.87019	0.393914	0.496337	0.4566	0.99995	no

ENSG00000145337	ENSG00000145337	PYURF	4:89442135-89629693	q1	q2	OK	53.9147	54.7115	0.0211655	0.0407532	0.95365	0.99995	no

ENSG00000145348	ENSG00000145348	TBCK	4:106962755-107270383	q1	q2	OK	16.9321	17.2511	0.0269336	0.0305252	0.95275	0.99995	no

ENSG00000145349	ENSG00000145349	CAMK2D	4:114372187-114683083	q1	q2	OK	27.7689	29.4413	0.0843697	0.174322	0.795	0.99995	no

ENSG00000145354	ENSG00000145354	CISD2	4:103790134-103940896	q1	q2	OK	27.5182	30.9445	0.169299	0.308109	0.65265	0.99995	no

ENSG00000145358	ENSG00000145358	DDIT4L	4:100869242-101136662	q1	q2	OK	0.656834	1.00255	0.610071	0.077801	0.76715	0.99995	no

ENSG00000145362	ENSG00000145362	ANK2	4:113739264-114304896	q1	q2	OK	23.8659	25.9538	0.120997	0.141236	0.7275	0.99995	no

ENSG00000145365	ENSG00000145365	TIFA	4:113195697-113363776	q1	q2	OK	3.4491	3.56563	0.0479354	0.0415092	0.9534	0.99995	no

ENSG00000145375	ENSG00000145375	SPATA5	4:123844228-124240605	q1	q2	OK	3.04665	3.42353	0.16826	0.267239	0.6937	0.99995	no

ENSG00000145384	ENSG00000145384	FABP2	4:120238404-120243545	q1	q2	NOTEST	0.010983	0	#NAME?	0	1	1	no

ENSG00000145386	ENSG00000145386	CCNA2	4:122722471-122745087	q1	q2	OK	54.5103	59.8924	0.135845	0.261909	0.71105	0.99995	no

ENSG00000145388	ENSG00000145388	METTL14	4:119606522-119636588	q1	q2	OK	15.026	15.8345	0.0756144	0.127336	0.84425	0.99995	no

ENSG00000145390	ENSG00000145390	USP53	4:120113927-120216672	q1	q2	OK	2.17787	2.72091	0.32117	0.366003	0.44865	0.99995	no

ENSG00000145391	ENSG00000145391	SETD7	4:140417094-140527853	q1	q2	OK	8.81268	9.93235	0.172555	0.294791	0.5726	0.99995	no

ENSG00000145414	ENSG00000145414	NAF1	4:164029936-164088073	q1	q2	OK	8.9697	10.0087	0.158121	0.251874	0.69475	0.99995	no

ENSG00000145416	ENSG00000145416	Mar-01	4:164445449-165305202	q1	q2	OK	2.84473	2.8927	0.0241241	0.0276088	0.95175	0.99995	no

ENSG00000145423	ENSG00000145423	SFRP2	4:154701743-154710272	q1	q2	OK	142.717	137.582	-0.0528677	-0.126701	0.8589	0.99995	no

ENSG00000145425	ENSG00000145425	RPS3A	4:152020724-152246784	q1	q2	OK	1239.37	1223.69	-0.0183678	-0.0372633	0.95365	0.99995	no

ENSG00000145428	ENSG00000145428	RNF175	4:154631276-154681387	q1	q2	OK	6.50302	5.75157	-0.177154	-0.198655	0.74875	0.99995	no

ENSG00000145431	ENSG00000145431	PDGFC	4:157681605-157892546	q1	q2	OK	33.6336	33.9274	0.0125456	0.0234005	0.97255	0.99995	no

ENSG00000145439	ENSG00000145439	CBR4	4:169277885-169931426	q1	q2	OK	15.6366	15.4687	-0.0155816	-0.0146155	0.9804	0.99995	no

ENSG00000145451	ENSG00000145451	GLRA3	4:175558064-175750465	q1	q2	NOTEST	0.085935	0.0887084	0.0458254	0	1	1	no

ENSG00000145476	ENSG00000145476	CYP4V2	4:187112673-187179625	q1	q2	OK	3.05644	3.76338	0.300179	0.430251	0.5316	0.99995	no

ENSG00000145491	ENSG00000145491	ROPN1L	5:10441401-10472141	q1	q2	NOTEST	0.265152	0.326948	0.302241	0	1	1	no

ENSG00000145494	ENSG00000145494	NDUFS6	5:1801513-1816719	q1	q2	OK	59.4037	53.526	-0.150313	-0.317248	0.64335	0.99995	no

ENSG00000145495	ENSG00000145495	Mar-06	5:10353814-10440500	q1	q2	OK	93.4188	97.8404	0.0667185	0.0889516	0.8703	0.99995	no

ENSG00000145506	ENSG00000145506	NKD2	5:1008943-1039058	q1	q2	OK	21.5762	22.2552	0.0447009	0.0713345	0.9138	0.99995	no

ENSG00000145526	ENSG00000145526	CDH18	5:19473059-20575982	q1	q2	NOTEST	0.31708	0.457549	0.529081	0	1	1	no

ENSG00000145536	ENSG00000145536	ADAMTS16	5:5140442-5320417	q1	q2	OK	1.33599	1.45224	0.120368	0.114819	0.84335	0.99995	no

ENSG00000145545	ENSG00000145545	SRD5A1	5:6633455-6669675	q1	q2	OK	12.197	12.3367	0.0164251	0.0318787	0.96275	0.99995	no

ENSG00000145555	ENSG00000145555	MYO10	5:16665394-16936372	q1	q2	OK	29.0747	27.0559	-0.103819	-0.205197	0.729	0.99995	no

ENSG00000145569	ENSG00000145569	FAM105A	5:14581883-14615116	q1	q2	OK	3.2451	2.47184	-0.392676	-0.369393	0.42	0.99995	no

ENSG00000145592	ENSG00000145592	RPL37	5:40825363-40835437	q1	q2	OK	723.176	718.489	-0.00937949	-0.0170117	0.9795	0.99995	no

ENSG00000145604	ENSG00000145604	SKP2	5:36152090-36184421	q1	q2	OK	27.5695	30.9518	0.166954	0.363314	0.56965	0.99995	no

ENSG00000145623	ENSG00000145623	OSMR	5:38845959-39074510	q1	q2	OK	1.59714	2.01496	0.335264	0.135796	0.7154	0.99995	no

ENSG00000145626	ENSG00000145626	UGT3A1	5:35951111-36001130	q1	q2	NOTEST	0	0.0158983	inf	0	1	1	no

ENSG00000145632	ENSG00000145632	PLK2	5:57749808-57756087	q1	q2	OK	37.4818	35.3412	-0.0848404	-0.115104	0.7796	0.99995	no

ENSG00000145642	ENSG00000145642	FAM159B	5:63986134-64013917	q1	q2	NOTEST	0.147123	0	#NAME?	0	1	1	no

ENSG00000145649	ENSG00000145649	GZMA	5:54398475-54406080	q1	q2	NOTEST	0.0341969	0.0334425	-0.0321855	0	1	1	no

ENSG00000145675	ENSG00000145675	PIK3R1	5:67511547-67597649	q1	q2	OK	12.1788	13.1275	0.108222	0.184305	0.70865	0.99995	no

ENSG00000145681	ENSG00000145681	HAPLN1	5:82933623-83017432	q1	q2	OK	0.598136	0.531239	-0.171112	-0.0869101	0.85005	0.99995	no

ENSG00000145685	ENSG00000145685	LHFPL2	5:77781037-78065844	q1	q2	OK	8.97999	8.53256	-0.0737352	-0.144904	0.8253	0.99995	no

ENSG00000145687	ENSG00000145687	SSBP2	5:80708839-81047616	q1	q2	OK	71.2753	70.8689	-0.00825062	-0.014398	0.9847	0.99995	no

ENSG00000145692	ENSG00000145692	BHMT	5:78293437-78531861	q1	q2	NOTEST	0	0	0	0	1	1	no

ENSG00000145700	ENSG00000145700	ANKRD31	5:74364099-74532703	q1	q2	NOTEST	0.163146	0.123844	-0.397642	0	1	1	no

ENSG00000145703	ENSG00000145703	IQGAP2	5:75699073-76031606	q1	q2	OK	14.387	15.0652	0.0664572	0.0621925	0.91075	0.99995	no

ENSG00000145708	ENSG00000145708	CRHBP	5:76248537-76276983	q1	q2	OK	1.59201	1.37532	-0.211086	-0.206347	0.72725	0.99995	no

ENSG00000145715	ENSG00000145715	RASA1	5:86563704-86708836	q1	q2	OK	25.8165	27.1719	0.0738221	0.158674	0.8189	0.99995	no

ENSG00000145721	ENSG00000145721	LIX1	5:96424778-96772921	q1	q2	OK	3.18065	2.22449	-0.515848	-0.401825	0.58755	0.99995	no

ENSG00000145723	ENSG00000145723	GIN1	5:102421703-102548500	q1	q2	NOTEST	0	0	0	0	1	1	no

ENSG00000145725	ENSG00000145725	PPIP5K2	5:102421703-102548500	q1	q2	OK	1.70926	3.32479	0.95989	0.689841	0.21085	0.99995	no

ENSG00000145730	ENSG00000145730	PAM	5:102089684-102366809	q1	q2	OK	41.6322	40.7404	-0.0312417	-0.0602758	0.919	0.99995	no

ENSG00000145734	ENSG00000145734	BDP1	5:70751441-70863649	q1	q2	NOTEST	0	0	0	0	1	1	no

ENSG00000145736	ENSG00000145736	GTF2H2	5:70330783-70363516	q1	q2	OK	2.42736	0.302028	-3.00663	-1.7168	0.00035	0.340103	no

ENSG00000145740	ENSG00000145740	SLC30A5	5:68389472-68432221	q1	q2	OK	19.3373	22.8236	0.239141	0.310655	0.61925	0.99995	no

ENSG00000145741	ENSG00000145741	BTF3	5:72794232-72801460	q1	q2	OK	422.332	423.952	0.0055225	0.0133622	0.98455	0.99995	no

ENSG00000145743	ENSG00000145743	FBXL17	5:107194735-107717799	q1	q2	OK	6.39345	8.41774	0.396839	0.523711	0.34255	0.99995	no

ENSG00000145757	ENSG00000145757	SPATA9	5:94982457-95034415	q1	q2	NOTEST	0.206311	0.392841	0.929129	0	1	1	no

ENSG00000145777	ENSG00000145777	TSLP	5:110405759-110466200	q1	q2	NOTEST	0.0470674	0.0550684	0.226497	0	1	1	no

ENSG00000145779	ENSG00000145779	TNFAIP8	5:118604386-118735383	q1	q2	OK	4.38439	5.51409	0.330747	0.417499	0.52985	0.99995	no

ENSG00000145780	ENSG00000145780	FEM1C	5:114856607-114880591	q1	q2	OK	8.20341	8.34204	0.0241761	0.0545238	0.93795	0.99995	no

ENSG00000145781	ENSG00000145781	COMMD10	5:115419503-115748459	q1	q2	OK	22.6061	25.9883	0.20115	0.400191	0.55235	0.99995	no

ENSG00000145782	ENSG00000145782	ATG12	5:115163892-115249778	q1	q2	OK	37.9412	39.3374	0.0521368	0.059719	0.91925	0.99995	no

ENSG00000145794	ENSG00000145794	MEGF10	5:126626522-126801429	q1	q2	OK	11.7899	10.821	-0.123723	-0.217427	0.6785	0.99995	no

ENSG00000145808	ENSG00000145808	ADAMTS19	5:128795251-129522327	q1	q2	OK	0.604541	0.894696	0.565558	0.286452	0.5451	0.99995	no

ENSG00000145817	ENSG00000145817	YIPF5	5:143537722-143550278	q1	q2	OK	23.0269	22.4482	-0.0367173	-0.0622262	0.92615	0.99995	no

ENSG00000145819	ENSG00000145819	ARHGAP26	5:142149948-142608576	q1	q2	OK	3.41584	4.41438	0.369973	0.416225	0.2673	0.99995	no

ENSG00000145824	ENSG00000145824	CXCL14	5:134895266-134970564	q1	q2	OK	1.4156	2.01497	0.509341	0.583602	0.3934	0.99995	no

ENSG00000145826	ENSG00000145826	LECT2	5:135257967-135290723	q1	q2	NOTEST	0.162034	0.328206	1.01831	0	1	1	no

ENSG00000145832	ENSG00000145832	SLC25A48	5:135170337-135224326	q1	q2	NOTEST	0.229544	0.446821	0.960924	0	1	1	no

ENSG00000145833	ENSG00000145833	DDX46	5:134094468-134195427	q1	q2	OK	53.0362	55.58	0.0675876	0.0674936	0.86785	0.99995	no

ENSG00000145835	ENSG00000145835	CDKN2AIPNLP2	2:27050036-27050388	q1	q2	NOTEST	0	0	0	0	1	1	no

ENSG00000145839	ENSG00000145839	IL9	5:135227934-135231516	q1	q2	NOTEST	0	0	0	0	1	1	no

ENSG00000145850	ENSG00000145850	TIMD4	5:156346292-156390266	q1	q2	NOTEST	0	0	0	0	1	1	no

ENSG00000145860	ENSG00000145860	RNF145	5:158584416-158675804	q1	q2	OK	78.2626	87.1231	0.154733	0.325514	0.61275	0.99995	no

ENSG00000145861	ENSG00000145861	C1QTNF2	5:159774757-159797648	q1	q2	OK	0.558272	0.576133	0.0454334	0.0467053	0.94675	0.99995	no

ENSG00000145863	ENSG00000145863	GABRA6	5:160715435-161129599	q1	q2	NOTEST	0.0671736	0	#NAME?	0	1	1	no

ENSG00000145864	ENSG00000145864	GABRB2	5:160715435-161129599	q1	q2	NOTEST	0.0996321	0.128855	0.37107	0	1	1	no

ENSG00000145868	ENSG00000145868	FBXO38	5:147763497-147822399	q1	q2	OK	17.4242	18.7285	0.104145	0.151643	0.7529	0.99995	no

ENSG00000145879	ENSG00000145879	SPINK7	5:147647742-147763346	q1	q2	NOTEST	0.0244358	0.0946456	1.95354	0	1	1	no

ENSG00000145882	ENSG00000145882	PCYOX1L	5:148737569-148749216	q1	q2	OK	10.1493	10.0166	-0.0189873	-0.0370216	0.95745	0.99995	no

ENSG00000145888	ENSG00000145888	GLRA1	5:151202073-151304403	q1	q2	NOTEST	0.14094	0.122717	-0.199743	0	1	1	no

ENSG00000145901	ENSG00000145901	TNIP1	5:150409505-150473138	q1	q2	OK	13.6405	11.6361	-0.229291	-0.4355	0.486	0.99995	no

ENSG00000145907	ENSG00000145907	G3BP1	5:151121876-151192346	q1	q2	OK	85.9321	91.0645	0.0836907	0.166512	0.8022	0.99995	no

ENSG00000145908	ENSG00000145908	ZNF300	5:150226084-150284545	q1	q2	OK	11.3787	13.0272	0.195191	0.423959	0.5535	0.99995	no

ENSG00000145911	ENSG00000145911	N4BP3	5:177540443-177553088	q1	q2	OK	1.6878	1.45021	-0.218884	-0.342028	0.62075	0.99995	no

ENSG00000145912	ENSG00000145912	NHP2	5:177557996-177580968	q1	q2	OK	98.2764	95.0454	-0.0482294	-0.086792	0.8987	0.99995	no

ENSG00000145916	ENSG00000145916	RMND5B	5:177557996-177580968	q1	q2	OK	25.2779	21.6294	-0.224882	-0.279177	0.6502	0.99995	no

ENSG00000145919	ENSG00000145919	BOD1	5:173034516-173043663	q1	q2	OK	91.3093	92.2294	0.0144656	0.0339909	0.96035	0.99995	no

ENSG00000145920	ENSG00000145920	CPLX2	5:175223312-175311023	q1	q2	OK	12.5103	12.897	0.043921	0.0636524	0.8945	0.99995	no

ENSG00000145934	ENSG00000145934	TENM2	5:166711803-167691162	q1	q2	OK	19.7633	16.9115	-0.224822	-0.469446	0.4336	0.99995	no

ENSG00000145936	ENSG00000145936	KCNMB1	5:169780490-170163636	q1	q2	NOTEST	0.143697	0.115457	-0.315675	0	1	1	no

ENSG00000145945	ENSG00000145945	FAM50B	6:3832166-3855971	q1	q2	NOTEST	0.016617	0.0334048	1.00739	0	1	1	no

ENSG00000145949	ENSG00000145949	MYLK4	6:2663862-2751200	q1	q2	NOTEST	0.19778	0.266245	0.428855	0	1	1	no

ENSG00000145965	ENSG00000145965	C6ORF50	6:3982908-3984372	q1	q2	OK	1.59031	2.02731	0.350264	0.403775	0.56025	0.99995	no

ENSG00000145975	ENSG00000145975	FAM217A	6:4021500-4157619	q1	q2	NOTEST	0.285043	0.297623	0.0623082	0	1	1	no

ENSG00000145979	ENSG00000145979	TBC1D7	6:12717892-13328815	q1	q2	OK	22.2199	22.0967	-0.00802168	-0.0120339	0.98605	0.99995	no

ENSG00000145982	ENSG00000145982	FARS2	6:5261276-5771813	q1	q2	OK	9.82582	9.36266	-0.0696592	-0.110416	0.8592	0.99995	no

ENSG00000145990	ENSG00000145990	GFOD1	6:13358061-13487894	q1	q2	OK	0.844213	0.715981	-0.237686	-0.248021	0.6613	0.99995	no

ENSG00000145996	ENSG00000145996	CDKAL1	6:20534687-21232635	q1	q2	OK	7.20039	8.47342	0.234869	0.456423	0.509	0.99995	no

ENSG00000146001	ENSG00000146001	PCDHB18	5:140613937-140617101	q1	q2	NOTEST	0.0125111	0.0115073	-0.120659	0	1	1	no

ENSG00000146005	ENSG00000146005	PSD2	5:139175405-139224051	q1	q2	OK	0.982521	0.735666	-0.417438	-0.507396	0.4705	0.99995	no

ENSG00000146006	ENSG00000146006	LRRTM2	5:137946655-138270723	q1	q2	OK	2.03662	1.62584	-0.324988	-0.0635831	0.85235	0.99995	no

ENSG00000146007	ENSG00000146007	ZMAT2	5:140044260-140086248	q1	q2	OK	71.1851	71.582	0.00802141	0.00942576	0.9881	0.99995	no

ENSG00000146013	ENSG00000146013	GFRA3	5:137588067-137610360	q1	q2	NOTEST	0.315884	0.174805	-0.853643	0	1	1	no

ENSG00000146021	ENSG00000146021	KLHL3	5:136953188-137071779	q1	q2	OK	1.77064	2.0165	0.187577	0.22752	0.6386	0.99995	no

ENSG00000146038	ENSG00000146038	DCDC2	6:24171983-24358512	q1	q2	NOTEST	0.179591	0.22118	0.300511	0	1	1	no

ENSG00000146039	ENSG00000146039	SLC17A4	6:25754926-25781419	q1	q2	NOTEST	0.00694643	0	#NAME?	0	1	1	no

ENSG00000146047	ENSG00000146047	HIST1H2BA	6:25727136-25727573	q1	q2	NOTEST	0	0	0	0	1	1	no

ENSG00000146049	ENSG00000146049	KAAG1	6:24171983-24358512	q1	q2	NOTEST	0	0	0	0	1	1	no

ENSG00000146054	ENSG00000146054	TRIM7	5:180618923-180632293	q1	q2	NOTEST	0.210241	0.38756	0.882375	0	1	1	no

ENSG00000146063	ENSG00000146063	TRIM41	5:180649498-180662809	q1	q2	OK	22.3762	19.1626	-0.223668	-0.505702	0.4757	0.99995	no

ENSG00000146066	ENSG00000146066	HIGD2A	5:175792470-175843570	q1	q2	OK	75.3777	71.8495	-0.06916	-0.0728442	0.9177	0.99995	no

ENSG00000146067	ENSG00000146067	FAM193B	5:176946788-176981542	q1	q2	OK	39.7208	38.0803	-0.060849	-0.0965757	0.8675	0.99995	no

ENSG00000146070	ENSG00000146070	PLA2G7	6:46620677-46703430	q1	q2	NOTEST	0.209203	0.235869	0.173082	0	1	1	no

ENSG00000146072	ENSG00000146072	TNFRSF21	6:47199267-47277641	q1	q2	OK	17.8534	19.2612	0.109496	0.238236	0.72975	0.99995	no

ENSG00000146083	ENSG00000146083	RNF44	5:175953697-175965026	q1	q2	OK	33.7078	31.6754	-0.0897173	-0.141082	0.7947	0.99995	no

ENSG00000146085	ENSG00000146085	MUT	6:49398072-49430904	q1	q2	OK	13.9887	13.9357	-0.00547306	-0.0125046	0.98705	0.99995	no

ENSG00000146090	ENSG00000146090	RASGEF1C	5:179527794-179636153	q1	q2	OK	0.630492	0.419422	-0.588075	-0.436119	0.40345	0.99995	no

ENSG00000146094	ENSG00000146094	DOK3	5:176910394-176938275	q1	q2	OK	5.00258	4.82745	-0.0514129	-0.027883	0.95815	0.99995	no

ENSG00000146109	ENSG00000146109	ABT1	6:26597179-26600278	q1	q2	NOTEST	0.237862	0.114396	-1.05608	0	1	1	no

ENSG00000146112	ENSG00000146112	PPP1R18	6:30644165-30655672	q1	q2	NOTEST	0	0	0	0	1	1	no

ENSG00000146122	ENSG00000146122	DAAM2	6:39760141-39902290	q1	q2	OK	4.61443	3.7648	-0.293578	-0.188783	0.5399	0.99995	no

ENSG00000146143	ENSG00000146143	PRIM2	6:57179602-57513375	q1	q2	OK	11.5882	12.5319	0.112946	0.21705	0.74385	0.99995	no

ENSG00000146147	ENSG00000146147	MLIP	6:53794779-54131078	q1	q2	NOTEST	0.178171	0.349806	0.973291	0	1	1	no

ENSG00000146151	ENSG00000146151	HMGCLL1	6:55299166-55444012	q1	q2	OK	0.696592	0.400008	-0.800286	-0.474736	0.2563	0.99995	no

ENSG00000146166	ENSG00000146166	LGSN	6:63985855-64029882	q1	q2	NOTEST	0.0276905	0.0234612	-0.239112	0	1	1	no

ENSG00000146192	ENSG00000146192	FGD2	6:36973421-36996846	q1	q2	NOTEST	0.0196355	0.0143266	-0.454762	0	1	1	no

ENSG00000146197	ENSG00000146197	SCUBE3	6:35182189-35220856	q1	q2	OK	15.3715	14.3071	-0.103526	-0.232072	0.7433	0.99995	no

ENSG00000146205	ENSG00000146205	ANO7	2:242127923-242164792	q1	q2	OK	0.819343	0.658904	-0.3144	-0.25474	0.61715	0.99995	no

ENSG00000146215	ENSG00000146215	CRIP3	6:43263431-43337216	q1	q2	OK	0.689916	0.980212	0.506674	0.0803448	0.6759	0.99995	no

ENSG00000146216	ENSG00000146216	TTBK1	6:43211417-43255997	q1	q2	OK	2.60861	1.74471	-0.580291	-0.85726	0.2257	0.99995	no

ENSG00000146221	ENSG00000146221	TCTE1	6:44238202-44346694	q1	q2	NOTEST	0.37733	0.329915	-0.193729	0	1	1	no

ENSG00000146223	ENSG00000146223	RPL7L1	6:42847355-42857663	q1	q2	OK	50.1005	51.0239	0.0263496	0.0473769	0.9274	0.99995	no

ENSG00000146232	ENSG00000146232	NFKBIE	6:44225902-44233500	q1	q2	OK	10.7516	10.4945	-0.0349096	-0.0488739	0.93085	0.99995	no

ENSG00000146233	ENSG00000146233	CYP39A1	6:46517540-46620523	q1	q2	OK	0.650232	0.584928	-0.152696	-0.110382	0.81765	0.99995	no

ENSG00000146242	ENSG00000146242	TPBG	6:83072922-83080545	q1	q2	OK	11.9239	12.5372	0.0723622	0.148136	0.83855	0.99995	no

ENSG00000146243	ENSG00000146243	IRAK1BP1	6:79577188-79787953	q1	q2	OK	6.16577	6.25824	0.0214753	0.0122651	0.98105	0.99995	no

ENSG00000146247	ENSG00000146247	PHIP	6:79577188-79787953	q1	q2	OK	29.9753	31.778	0.0842559	0.189264	0.78885	0.99995	no

ENSG00000146250	ENSG00000146250	PRSS35	6:84222193-84235423	q1	q2	OK	1.22723	1.15374	-0.089096	-0.10238	0.8852	0.99995	no

ENSG00000146263	ENSG00000146263	MMS22L	6:97590036-97731093	q1	q2	OK	11.2596	12.244	0.120913	0.135462	0.7558	0.99995	no

ENSG00000146267	ENSG00000146267	FAXC	6:99719044-99797938	q1	q2	OK	12.1805	13.3853	0.136073	0.163905	0.81455	0.99995	no

ENSG00000146276	ENSG00000146276	GABRR1	6:89887219-89940997	q1	q2	NOTEST	0.0095477	0.0151898	0.66988	0	1	1	no

ENSG00000146278	ENSG00000146278	PNRC1	6:89790469-89794879	q1	q2	OK	34.957	28.5713	-0.291012	-0.532311	0.447	0.99995	no

ENSG00000146281	ENSG00000146281	PM20D2	6:89855768-89875284	q1	q2	OK	5.2601	4.94054	-0.0904215	-0.176445	0.804	0.99995	no

ENSG00000146282	ENSG00000146282	RARS2	6:88224095-88299721	q1	q2	OK	20.3547	21.8315	0.10105	0.203668	0.7545	0.99995	no

ENSG00000146285	ENSG00000146285	SCML4	6:108025307-108145521	q1	q2	NOTEST	0.14734	0.0913435	-0.689774	0	1	1	no

ENSG00000146350	ENSG00000146350	TBC1D32	6:121400639-121655891	q1	q2	OK	3.53194	4.16568	0.238092	0.248014	0.61835	0.99995	no

ENSG00000146352	ENSG00000146352	CLVS2	6:123317115-123394072	q1	q2	OK	1.2935	1.3204	0.0296982	0.035665	0.9482	0.99995	no

ENSG00000146360	ENSG00000146360	GPR6	6:110299513-110301921	q1	q2	NOTEST	0.0166617	0.0161884	-0.0415705	0	1	1	no

ENSG00000146373	ENSG00000146373	RNF217	6:125230238-125413779	q1	q2	OK	2.07767	2.5618	0.30219	0.301892	0.5495	0.99995	no

ENSG00000146374	ENSG00000146374	RSPO3	6:127439748-127518910	q1	q2	NOTEST	0.271762	0.283446	0.0607277	0	1	1	no

ENSG00000146376	ENSG00000146376	ARHGAP18	6:129897276-130031370	q1	q2	OK	1.68602	2.08519	0.306558	0.310449	0.52635	0.99995	no

ENSG00000146378	ENSG00000146378	TAAR2	6:132938160-132945414	q1	q2	NOTEST	0.0261758	0.025725	-0.0250641	0	1	1	no

ENSG00000146383	ENSG00000146383	TAAR6	6:132891460-132892498	q1	q2	NOTEST	0.0299019	0.0587261	0.973763	0	1	1	no

ENSG00000146385	ENSG00000146385	TAAR8	6:132873831-132874860	q1	q2	NOTEST	0	0	0	0	1	1	no

ENSG00000146386	ENSG00000146386	ABRACL	6:139349818-139364439	q1	q2	OK	57.3569	67.4456	0.233758	0.511838	0.4555	0.99995	no

ENSG00000146399	ENSG00000146399	TAAR1	6:132966122-132967142	q1	q2	NOTEST	0	0	0	0	1	1	no

ENSG00000146409	ENSG00000146409	SLC18B1	6:133090508-133119701	q1	q2	OK	11.296	11.3264	0.00387052	0.00750275	0.9903	0.99995	no

ENSG00000146410	ENSG00000146410	MTFR2	6:136552161-136571473	q1	q2	OK	4.14722	4.45734	0.104042	0.134734	0.82505	0.99995	no

ENSG00000146411	ENSG00000146411	SLC2A12	6:134273307-134373774	q1	q2	OK	1.44936	0.848055	-0.77319	-0.443596	0.5486	0.99995	no

ENSG00000146414	ENSG00000146414	SHPRH	6:146136047-146285559	q1	q2	OK	9.17128	10.7476	0.228815	0.432096	0.45705	0.99995	no

ENSG00000146416	ENSG00000146416	AIG1	6:143381632-143661441	q1	q2	OK	9.96737	9.5745	-0.0580167	-0.0757173	0.90625	0.99995	no

ENSG00000146425	ENSG00000146425	DYNLT1	6:159057505-159065771	q1	q2	OK	123.094	133.482	0.116893	0.269061	0.7018	0.99995	no

ENSG00000146426	ENSG00000146426	TIAM2	6:155054458-155635627	q1	q2	OK	16.7019	17.5527	0.0716809	0.0509127	0.92275	0.99995	no

ENSG00000146433	ENSG00000146433	TMEM181	6:158957467-159056460	q1	q2	OK	14.1747	15.6864	0.146201	0.347816	0.6138	0.99995	no

ENSG00000146453	ENSG00000146453	PNLDC1	6:160221297-160241736	q1	q2	NOTEST	0.0301676	0.0285117	-0.0814424	0	1	1	no

ENSG00000146457	ENSG00000146457	WTAP	6:160090088-160210781	q1	q2	OK	70.2557	72.7831	0.0509877	0.0365885	0.9548	0.99995	no

ENSG00000146463	ENSG00000146463	ZMYM4	1:35734567-35887659	q1	q2	OK	36.4879	39.1836	0.10283	0.133741	0.73065	0.99995	no

ENSG00000146469	ENSG00000146469	VIP	6:153071932-153080900	q1	q2	NOTEST	0	0.0218597	inf	0	1	1	no

ENSG00000146476	ENSG00000146476	C6orf211	6:151773421-151791236	q1	q2	OK	19.3395	22.3898	0.211291	0.450515	0.51155	0.99995	no

ENSG00000146477	ENSG00000146477	SLC22A3	6:160769299-160932156	q1	q2	NOTEST	0.0352141	0.0663375	0.913671	0	1	1	no

ENSG00000146521	ENSG00000146521	C6orf123	6:168185216-168198927	q1	q2	NOTEST	0.0172397	0.0253035	0.553597	0	1	1	no

ENSG00000146530	ENSG00000146530	VWDE	7:12370510-12443567	q1	q2	NOTEST	0.0755589	0.315338	2.06123	0	1	1	no

ENSG00000146535	ENSG00000146535	GNA12	7:2719155-2883958	q1	q2	OK	31.6005	29.8495	-0.0822438	-0.193201	0.77955	0.99995	no

ENSG00000146540	ENSG00000146540	C7orf50	7:1036621-1177896	q1	q2	OK	48.7186	42.808	-0.186591	-0.3826	0.59195	0.99995	no

ENSG00000146555	ENSG00000146555	SDK1	7:3303663-4308632	q1	q2	OK	8.52966	6.27094	-0.443806	-0.616745	0.2054	0.99995	no

ENSG00000146556	ENSG00000146556	WASH2P	2:114341663-114359144	q1	q2	OK	6.69551	5.28608	-0.340996	-0.515364	0.4547	0.99995	no

ENSG00000146574	ENSG00000146574	CCZ1B	7:6793739-6866401	q1	q2	OK	18.4682	19.1565	0.0527935	0.0840051	0.88965	0.99995	no

ENSG00000146576	ENSG00000146576	C7orf26	7:6629647-6648357	q1	q2	OK	20.6436	19.6901	-0.0682278	-0.120932	0.83855	0.99995	no

ENSG00000146587	ENSG00000146587	RBAK	7:5013618-5112854	q1	q2	OK	9.05399	9.84809	0.12129	0.221552	0.74245	0.99995	no

ENSG00000146592	ENSG00000146592	CREB5	7:28338939-28865511	q1	q2	OK	8.06272	7.77246	-0.0528959	-0.0588024	0.8738	0.99995	no

ENSG00000146618	ENSG00000146618	FERD3L	7:19183915-19185876	q1	q2	NOTEST	0	0	0	0	1	1	no

ENSG00000146648	ENSG00000146648	EGFR	7:55086713-55324313	q1	q2	OK	3.76804	3.27073	-0.204201	-0.380753	0.5235	0.99995	no

ENSG00000146666	ENSG00000146666	LINC00525	7:47735327-48019178	q1	q2	NOTEST	0	0	0	0	1	1	no

ENSG00000146670	ENSG00000146670	CDCA5	11:64833771-64851636	q1	q2	OK	38.1926	34.458	-0.148456	-0.336174	0.6022	0.99995	no

ENSG00000146674	ENSG00000146674	IGFBP3	7:45951948-45961473	q1	q2	OK	27.8282	45.6521	0.714137	1.47566	0.0176	0.99995	no

ENSG00000146676	ENSG00000146676	PURB	7:44915895-44925992	q1	q2	OK	12.6303	13.4827	0.0942171	0.229702	0.7429	0.99995	no

ENSG00000146677	ENSG00000146677	AC004453.8	7:44418719-44530479	q1	q2	OK	59.2409	57.4839	-0.0434354	-0.0346416	0.9618	0.99995	no

ENSG00000146678	ENSG00000146678	IGFBP1	7:45927955-45933267	q1	q2	NOTEST	0	0	0	0	1	1	no

ENSG00000146700	ENSG00000146700	SRCRB4D	7:76018650-76071388	q1	q2	OK	0.488791	0.234465	-1.05985	-0.636085	0.5281	0.99995	no

ENSG00000146701	ENSG00000146701	MDH2	7:75677368-75696826	q1	q2	OK	13.1499	4.20037	-1.64646	-1.63292	0.0447	0.99995	no

ENSG00000146707	ENSG00000146707	POMZP3	7:76139744-77045717	q1	q2	OK	3.21806	3.57912	0.153412	0.115998	0.85655	0.99995	no

ENSG00000146722	ENSG00000146722	AC006014.8	7:74988447-75115548	q1	q2	NOTEST	0	0	0	0	1	1	no

ENSG00000146729	ENSG00000146729	GBAS	7:55954969-56067874	q1	q2	OK	46.8269	46.8301	9.81E-05	0.000177086	0.99975	0.99995	no

ENSG00000146731	ENSG00000146731	CCT6A	7:56119322-56131682	q1	q2	OK	159.435	168.046	0.0758868	0.151591	0.79315	0.99995	no

ENSG00000146733	ENSG00000146733	PSPH	7:56078743-56119297	q1	q2	OK	21.7192	23.7679	0.130044	0.217223	0.7424	0.99995	no

ENSG00000146755	ENSG00000146755	TRIM50	7:72726534-72742085	q1	q2	NOTEST	0	0	0	0	1	1	no

ENSG00000146757	ENSG00000146757	ZNF92	7:64838711-64866038	q1	q2	OK	20.3272	22.6265	0.154603	0.340171	0.59835	0.99995	no

ENSG00000146776	ENSG00000146776	ATXN7L1	7:105245513-105517050	q1	q2	OK	4.8314	4.99254	0.047333	0.05145	0.9214	0.99995	no

ENSG00000146802	ENSG00000146802	TMEM168	7:112402436-112430647	q1	q2	OK	9.72043	11.1358	0.196111	0.253923	0.68175	0.99995	no

ENSG00000146809	ENSG00000146809	ASB15	7:123207063-123277951	q1	q2	NOTEST	0	0	0	0	1	1	no

ENSG00000146826	ENSG00000146826	C7orf43	7:99746529-99756338	q1	q2	OK	12.451	10.9829	-0.181	-0.198118	0.75095	0.99995	no

ENSG00000146828	ENSG00000146828	SLC12A9	7:100400186-100464631	q1	q2	OK	11.9652	10.4297	-0.198139	-0.109543	0.70855	0.99995	no

ENSG00000146830	ENSG00000146830	GIGYF1	7:100277129-100287071	q1	q2	OK	23.0339	20.1708	-0.191487	-0.278514	0.5322	0.99995	no

ENSG00000146833	ENSG00000146833	TRIM4	7:99473609-99517223	q1	q2	OK	4.31987	4.37008	0.0166706	0.0240646	0.9665	0.99995	no

ENSG00000146834	ENSG00000146834	MEPCE	7:99998448-100031741	q1	q2	OK	25.1113	22.6297	-0.150118	-0.330189	0.6284	0.99995	no

ENSG00000146839	ENSG00000146839	ZAN	7:100331248-100395419	q1	q2	NOTEST	0.00302672	0	#NAME?	0	1	1	no

ENSG00000146842	ENSG00000146842	TMEM209	7:129804554-129847610	q1	q2	OK	13.6257	14.7903	0.118314	0.199342	0.71625	0.99995	no

ENSG00000146856	ENSG00000146856	AGBL3	7:134671258-134855547	q1	q2	OK	1.58793	2.76404	0.799633	0.277842	0.5069	0.99995	no

ENSG00000146857	ENSG00000146857	STRA8	7:134916730-134943244	q1	q2	NOTEST	0	0	0	0	1	1	no

ENSG00000146858	ENSG00000146858	ZC3HAV1L	7:138710451-138720775	q1	q2	OK	2.15615	2.67307	0.310039	0.390358	0.5702	0.99995	no

ENSG00000146859	ENSG00000146859	TMEM140	7:134671258-134855547	q1	q2	OK	6.98157	7.67078	0.135822	0.158334	0.81895	0.99995	no

ENSG00000146872	ENSG00000146872	TLK2	17:60536018-60692842	q1	q2	OK	31.8608	32.5111	0.0291495	0.0545378	0.91755	0.99995	no

ENSG00000146904	ENSG00000146904	EPHA1	7:143078172-143220542	q1	q2	OK	1.46136	1.65558	0.180027	0.0288185	0.9126	0.99995	no

ENSG00000146909	ENSG00000146909	NOM1	7:156742416-156765876	q1	q2	OK	11.3281	11.8767	0.0682321	0.0787374	0.8662	0.99995	no

ENSG00000146910	ENSG00000146910	CNPY1	7:155266900-155326557	q1	q2	OK	35.9567	43.3702	0.270445	0.40548	0.5575	0.99995	no

ENSG00000146918	ENSG00000146918	NCAPG2	7:158424002-158497520	q1	q2	OK	26.3305	28.6666	0.122639	0.272754	0.6784	0.99995	no

ENSG00000146926	ENSG00000146926	ASB10	7:150872784-150884919	q1	q2	NOTEST	0	0.015423	inf	0	1	1	no

ENSG00000146938	ENSG00000146938	NLGN4X	X:5758677-6146904	q1	q2	OK	9.7355	10.1562	0.0610371	0.110507	0.8538	0.99995	no

ENSG00000146950	ENSG00000146950	SHROOM2	X:9754495-9917483	q1	q2	OK	10.6893	10.1014	-0.08161	-0.166415	0.8055	0.99995	no

ENSG00000146955	ENSG00000146955	RAB19	7:139993492-140126050	q1	q2	NOTEST	0.0403199	0	#NAME?	0	1	1	no

ENSG00000146963	ENSG00000146963	C7orf55-LUC7L2	7:139024202-139108198	q1	q2	OK	63.3419	63.7051	0.00824977	0.0168319	0.97915	0.99995	no

ENSG00000146966	ENSG00000146966	DENND2A	7:140218219-140624564	q1	q2	OK	17.7985	17.0088	-0.0654733	-0.0885197	0.9044	0.99995	no

ENSG00000147003	ENSG00000147003	TMEM27	X:15645440-15683154	q1	q2	OK	0.952632	0.687812	-0.469905	-0.484378	0.48195	0.99995	no

ENSG00000147010	ENSG00000147010	SH3KBP1	X:19552092-19905719	q1	q2	OK	14.9425	13.671	-0.128299	-0.265291	0.70395	0.99995	no

ENSG00000147027	ENSG00000147027	TMEM47	X:34645180-34675405	q1	q2	OK	31.838	30.1635	-0.0779486	-0.186517	0.78815	0.99995	no

ENSG00000147036	ENSG00000147036	LANCL3	X:37208527-38548169	q1	q2	NOTEST	0	0	0	0	1	1	no

ENSG00000147041	ENSG00000147041	SYTL5	X:37208527-38548169	q1	q2	NOTEST	0.0268949	0.0313371	0.220542	0	1	1	no

ENSG00000147044	ENSG00000147044	CASK	X:41374186-41782716	q1	q2	OK	18.4584	21.233	0.20203	0.397879	0.5466	0.99995	no

ENSG00000147050	ENSG00000147050	KDM6A	X:44732756-44971847	q1	q2	OK	10.5886	10.9177	0.0441653	0.0643217	0.9011	0.99995	no

ENSG00000147059	ENSG00000147059	SPIN2A	X:57160962-57164058	q1	q2	OK	0.565444	0.638755	0.175878	0.162012	0.8271	0.99995	no

ENSG00000147065	ENSG00000147065	MSN	X:64808256-64961791	q1	q2	OK	90.012	91.4621	0.023056	0.054943	0.9373	0.99995	no

ENSG00000147081	ENSG00000147081	AKAP4	X:49955405-49965664	q1	q2	NOTEST	0	0	0	0	1	1	no

ENSG00000147082	ENSG00000147082	CCNB3	X:49967363-50094909	q1	q2	OK	0.677872	0.022687	-4.90107	-0.310005	0.2186	0.99995	no

ENSG00000147099	ENSG00000147099	HDAC8	X:71549365-71792953	q1	q2	NOTEST	0.035314	0.148887	2.0759	0	1	1	no

ENSG00000147100	ENSG00000147100	SLC16A2	X:73641084-73753752	q1	q2	OK	16.1625	16.1263	-0.00323131	-0.00732486	0.99155	0.99995	no

ENSG00000147113	ENSG00000147113	CXorf36	X:45007618-45193162	q1	q2	NOTEST	0.00561357	0.0106662	0.926051	0	1	1	no

ENSG00000147117	ENSG00000147117	ZNF157	X:47229981-47273704	q1	q2	NOTEST	0.478257	0.458034	-0.0623305	0	1	1	no

ENSG00000147118	ENSG00000147118	ZNF182	X:47696300-47931025	q1	q2	NOTEST	0.0678998	0	#NAME?	0	1	1	no

ENSG00000147119	ENSG00000147119	CHST7	X:46433218-46457843	q1	q2	OK	2.11709	2.10194	-0.0103632	-0.0133304	0.9845	0.99995	no

ENSG00000147121	ENSG00000147121	KRBOX4	X:46306291-46356857	q1	q2	OK	10.576	11.5089	0.121966	0.183784	0.7795	0.99995	no

ENSG00000147123	ENSG00000147123	NDUFB11	X:47001614-47046212	q1	q2	NOTEST	0	0	0	0	1	1	no

ENSG00000147124	ENSG00000147124	ZNF41	X:47305277-47342345	q1	q2	OK	4.86962	4.97218	0.0300688	0.0563837	0.93425	0.99995	no

ENSG00000147127	ENSG00000147127	RAB41	X:69501942-69504852	q1	q2	NOTEST	0.446396	0.277275	-0.687006	0	1	1	no

ENSG00000147130	ENSG00000147130	ZMYM3	X:70459473-70474996	q1	q2	OK	34.0649	32.7647	-0.0561435	-0.0995693	0.8417	0.99995	no

ENSG00000147133	ENSG00000147133	TAF1	X:70586113-70752224	q1	q2	OK	16.7356	16.6239	-0.00965467	-0.0105648	0.9828	0.99995	no

ENSG00000147138	ENSG00000147138	GPR174	X:78426468-78427726	q1	q2	NOTEST	0.0224301	0.0218397	-0.0384834	0	1	1	no

ENSG00000147140	ENSG00000147140	NONO	X:70503041-70521018	q1	q2	OK	430.766	421.276	-0.0321403	-0.0592026	0.92765	0.99995	no

ENSG00000147144	ENSG00000147144	CCDC120	X:48911100-48927509	q1	q2	OK	0.886032	0.689734	-0.36132	-0.291011	0.5776	0.99995	no

ENSG00000147145	ENSG00000147145	LPAR4	X:78003205-78012591	q1	q2	OK	11.8923	10.6667	-0.156918	-0.215832	0.7258	0.99995	no

ENSG00000147155	ENSG00000147155	EBP	X:48379545-48387104	q1	q2	NOTEST	0.0923484	0	#NAME?	0	1	1	no

ENSG00000147160	ENSG00000147160	AWAT2	X:69260391-69269788	q1	q2	NOTEST	0.0123335	0.0576681	2.22519	0	1	1	no

ENSG00000147162	ENSG00000147162	OGT	X:70752932-70795747	q1	q2	OK	70.8906	68.6793	-0.0457193	-0.0767781	0.87255	0.99995	no

ENSG00000147164	ENSG00000147164	SNX12	X:70279093-70288273	q1	q2	OK	30.6147	30.8943	0.0131178	0.0304841	0.9646	0.99995	no

ENSG00000147166	ENSG00000147166	ITGB1BP2	X:70521583-70525221	q1	q2	OK	2.43107	2.27281	-0.0971185	-0.0999903	0.8823	0.99995	no

ENSG00000147168	ENSG00000147168	IL2RG	X:70327253-70331958	q1	q2	NOTEST	0.0177886	0.0139241	-0.353367	0	1	1	no

ENSG00000147174	ENSG00000147174	ACRC	X:70798260-70833433	q1	q2	OK	3.75802	2.32506	-0.692707	-0.7645	0.2441	0.99995	no

ENSG00000147180	ENSG00000147180	ZNF711	X:84498996-84528368	q1	q2	OK	33.6596	35.779	0.0880923	0.212729	0.76405	0.99995	no

ENSG00000147183	ENSG00000147183	CPXCR1	X:88002225-88009786	q1	q2	NOTEST	0	0	0	0	1	1	no

ENSG00000147202	ENSG00000147202	DIAPH2	X:95939661-96897588	q1	q2	OK	1.31164	1.32582	0.0155129	0.0204732	0.97685	0.99995	no

ENSG00000147206	ENSG00000147206	NXF3	X:102330737-102348157	q1	q2	NOTEST	0	0.100259	inf	0	1	1	no

ENSG00000147223	ENSG00000147223	RIPPLY1	X:106045909-106243474	q1	q2	NOTEST	0.0672205	0	#NAME?	0	1	1	no

ENSG00000147224	ENSG00000147224	PRPS1	X:106871736-106894256	q1	q2	OK	33.2573	32.9923	-0.0115389	-0.0264347	0.9683	0.99995	no

ENSG00000147231	ENSG00000147231	CXorf57	X:105855159-105922672	q1	q2	OK	10.8904	12.1662	0.159814	0.202915	0.69885	0.99995	no

ENSG00000147234	ENSG00000147234	FRMPD3	X:106756212-106848481	q1	q2	OK	1.55735	1.38559	-0.168595	-0.260281	0.69925	0.99995	no

ENSG00000147246	ENSG00000147246	HTR2C	X:113818550-114144624	q1	q2	NOTEST	0	0	0	0	1	1	no

ENSG00000147251	ENSG00000147251	DOCK11	X:117629860-117820126	q1	q2	OK	4.6738	5.34141	0.192625	0.404706	0.5578	0.99995	no

ENSG00000147255	ENSG00000147255	IGSF1	X:130407479-130533677	q1	q2	NOTEST	0.388574	0.345075	-0.171276	0	1	1	no

ENSG00000147256	ENSG00000147256	ARHGAP36	X:130192215-130223857	q1	q2	NOTEST	0.0448484	0.010978	-2.03044	0	1	1	no

ENSG00000147257	ENSG00000147257	GPC3	X:132669772-133119922	q1	q2	OK	17.4597	19.758	0.178406	0.281652	0.69045	0.99995	no

ENSG00000147262	ENSG00000147262	GPR119	X:129518318-129519511	q1	q2	NOTEST	0	0	0	0	1	1	no

ENSG00000147274	ENSG00000147274	RBMX	X:135923089-135962923	q1	q2	OK	332.13	344.118	0.0511541	0.103016	0.8776	0.99995	no

ENSG00000147316	ENSG00000147316	MCPH1	8:6261071-6565730	q1	q2	OK	9.58386	10.1716	0.0858647	0.145482	0.819	0.99995	no

ENSG00000147324	ENSG00000147324	MFHAS1	8:8640863-8751155	q1	q2	OK	8.50457	9.80222	0.20487	0.209303	0.60445	0.99995	no

ENSG00000147364	ENSG00000147364	FBXO25	8:356427-421225	q1	q2	OK	8.23125	6.84176	-0.266745	-0.368581	0.5611	0.99995	no

ENSG00000147378	ENSG00000147378	FATE1	X:150884506-150891666	q1	q2	NOTEST	0	0	0	0	1	1	no

ENSG00000147380	ENSG00000147380	OPN1MW	X:153448106-153461633	q1	q2	NOTEST	0	0	0	0	1	1	no

ENSG00000147381	ENSG00000147381	MAGEA4	X:151072902-151093642	q1	q2	NOTEST	0	0	0	0	1	1	no

ENSG00000147382	ENSG00000147382	FAM58A	X:152853376-152869808	q1	q2	NOTEST	0	0	0	0	1	1	no

ENSG00000147383	ENSG00000147383	NSDHL	X:151999510-152038273	q1	q2	OK	2.1374	4.06868	0.928702	1.02559	0.1571	0.99995	no

ENSG00000147394	ENSG00000147394	ZNF185	X:152082985-152142024	q1	q2	NOTEST	0.00793298	0	#NAME?	0	1	1	no

ENSG00000147400	ENSG00000147400	CETN2	X:151995516-151999321	q1	q2	OK	2.87364	0.964686	-1.57475	-1.75042	0.01125	0.99995	no

ENSG00000147402	ENSG00000147402	GABRQ	X:151806636-151825999	q1	q2	NOTEST	0.00418573	0	#NAME?	0	1	1	no

ENSG00000147403	ENSG00000147403	RPL10	X:153618314-153650065	q1	q2	OK	32.5261	25.7272	-0.338306	-0.553554	0.42385	0.99995	no

ENSG00000147408	ENSG00000147408	CSGALNACT1	8:19261671-19615540	q1	q2	OK	1.42872	0.882565	-0.694947	-0.557958	0.14475	0.99995	no

ENSG00000147416	ENSG00000147416	ATP6V1B2	8:20054877-20084330	q1	q2	OK	60.2658	66.4496	0.140921	0.316575	0.6204	0.99995	no

ENSG00000147419	ENSG00000147419	CCDC25	8:27590834-27695612	q1	q2	OK	29.0581	27.6879	-0.0696861	-0.0866258	0.88405	0.99995	no

ENSG00000147421	ENSG00000147421	HMBOX1	8:28747910-28922281	q1	q2	OK	16.1288	14.7924	-0.124778	-0.143686	0.8258	0.99995	no

ENSG00000147432	ENSG00000147432	CHRNB3	8:42552518-42592550	q1	q2	NOTEST	0.0113322	0.0109887	-0.0443983	0	1	1	no

ENSG00000147434	ENSG00000147434	CHRNA6	8:42607762-42651535	q1	q2	NOTEST	0.0291604	0.0224265	-0.37881	0	1	1	no

ENSG00000147437	ENSG00000147437	GNRH1	8:25276775-25282170	q1	q2	OK	2.37653	2.89143	0.282932	0.332969	0.612	0.99995	no

ENSG00000147439	ENSG00000147439	BIN3	8:22462144-22526661	q1	q2	OK	11.4465	12.6151	0.140243	0.065893	0.9029	0.99995	no

ENSG00000147443	ENSG00000147443	DOK2	8:21766383-21771371	q1	q2	NOTEST	0	0.0292574	inf	0	1	1	no

ENSG00000147454	ENSG00000147454	SLC25A37	8:23386317-23432976	q1	q2	OK	14.242	15.137	0.0879273	0.163366	0.7908	0.99995	no

ENSG00000147457	ENSG00000147457	CHMP7	8:23101149-23119512	q1	q2	OK	23.1123	22.8195	-0.0183954	-0.0379458	0.9521	0.99995	no

ENSG00000147459	ENSG00000147459	DOCK5	8:25042237-25275598	q1	q2	OK	2.03759	2.19865	0.109751	0.114359	0.78465	0.99995	no

ENSG00000147465	ENSG00000147465	STAR	8:37962759-38070819	q1	q2	OK	1.65273	1.35619	-0.285295	-0.0912028	0.86895	0.99995	no

ENSG00000147471	ENSG00000147471	PROSC	8:37620110-37637283	q1	q2	OK	28.7424	30.6454	0.0924934	0.177704	0.76195	0.99995	no

ENSG00000147475	ENSG00000147475	ERLIN2	8:37592278-37616619	q1	q2	OK	18.399	18.3889	-0.000784917	-0.0012695	0.9977	0.99995	no

ENSG00000147481	ENSG00000147481	SNTG1	8:50822348-51706678	q1	q2	OK	1.35716	1.56851	0.208805	0.144196	0.76615	0.99995	no

ENSG00000147485	ENSG00000147485	PXDNL	8:52170247-52722005	q1	q2	NOTEST	0.0509245	0.0801157	0.653724	0	1	1	no

ENSG00000147488	ENSG00000147488	ST18	8:53023398-53373519	q1	q2	OK	7.92104	8.26234	0.0608607	0.0715542	0.84985	0.99995	no

ENSG00000147509	ENSG00000147509	RGS20	8:54764367-54871863	q1	q2	OK	32.3262	29.7206	-0.121243	-0.227907	0.73345	0.99995	no

ENSG00000147526	ENSG00000147526	TACC1	8:38585703-38710546	q1	q2	OK	33.1313	45.0195	0.442355	0.710165	0.15265	0.99995	no

ENSG00000147533	ENSG00000147533	GOLGA7	8:41347914-41368499	q1	q2	OK	43.2444	44.0785	0.0275602	0.063808	0.92585	0.99995	no

ENSG00000147535	ENSG00000147535	PPAPDC1B	8:38082735-38239790	q1	q2	OK	21.0624	22.2275	0.0776768	0.0382138	0.92925	0.99995	no

ENSG00000147536	ENSG00000147536	GINS4	8:41386724-41482520	q1	q2	OK	21.265	24.2516	0.189596	0.150077	0.72025	0.99995	no

ENSG00000147548	ENSG00000147548	WHSC1L1	8:38082735-38239790	q1	q2	OK	42.4802	47.4004	0.158111	0.179035	0.7383	0.99995	no

ENSG00000147570	ENSG00000147570	DNAJC5B	8:66933794-67012751	q1	q2	NOTEST	0.0202481	0.0210197	0.0539537	0	1	1	no

ENSG00000147571	ENSG00000147571	CRH	8:67088619-67090960	q1	q2	OK	1.48429	1.54113	0.0542124	0.0616502	0.9265	0.99995	no

ENSG00000147573	ENSG00000147573	TRIM55	8:67039130-67087720	q1	q2	OK	0.345065	0.570419	0.725153	0.464954	0.49215	0.99995	no

ENSG00000147576	ENSG00000147576	ADHFE1	8:67341262-67430759	q1	q2	OK	1.54739	1.46367	-0.0802517	-0.0234176	0.9231	0.99995	no

ENSG00000147586	ENSG00000147586	MRPS28	8:80830951-81143467	q1	q2	OK	41.6498	41.5926	-0.00198472	-0.00262813	0.99725	0.99995	no

ENSG00000147588	ENSG00000147588	PMP2	8:82351670-82445510	q1	q2	NOTEST	0.0235384	0	#NAME?	0	1	1	no

ENSG00000147592	ENSG00000147592	LACTB2	8:71520811-71581409	q1	q2	OK	8.41404	9.43784	0.165657	0.221776	0.7229	0.99995	no

ENSG00000147596	ENSG00000147596	PRDM14	8:70963885-70983928	q1	q2	NOTEST	0	0	0	0	1	1	no

ENSG00000147601	ENSG00000147601	TERF1	8:73921098-73960357	q1	q2	OK	28.9468	30.6232	0.0812211	0.177562	0.79895	0.99995	no

ENSG00000147604	ENSG00000147604	RPL7	8:74202505-74268696	q1	q2	OK	1260.84	1282.85	0.0249676	0.0448873	0.9467	0.99995	no

ENSG00000147606	ENSG00000147606	SLC26A7	8:92114059-92410378	q1	q2	NOTEST	0.0715634	0.0282805	-1.33942	0	1	1	no

ENSG00000147613	ENSG00000147613	PSKH2	8:86999551-87166457	q1	q2	NOTEST	0	0	0	0	1	1	no

ENSG00000147614	ENSG00000147614	ATP6V0D2	8:86999551-87166457	q1	q2	NOTEST	0	0	0	0	1	1	no

ENSG00000147642	ENSG00000147642	SYBU	8:110586206-110704020	q1	q2	OK	16.8093	14.7001	-0.193432	-0.349706	0.55615	0.99995	no

ENSG00000147647	ENSG00000147647	DPYS	8:105342551-105479281	q1	q2	NOTEST	0	0	0	0	1	1	no

ENSG00000147649	ENSG00000147649	MTDH	8:98656406-98740998	q1	q2	OK	33.9673	32.1779	-0.078074	-0.124773	0.80635	0.99995	no

ENSG00000147650	ENSG00000147650	LRP12	8:105501458-105601252	q1	q2	OK	18.2107	18.5322	0.0252481	0.0509735	0.9362	0.99995	no

ENSG00000147654	ENSG00000147654	EBAG9	8:110551939-110578225	q1	q2	OK	16.4907	16.9307	0.0379928	0.0683411	0.91975	0.99995	no

ENSG00000147655	ENSG00000147655	RSPO2	8:108911543-109095913	q1	q2	NOTEST	0.0316895	0.0723623	1.19124	0	1	1	no

ENSG00000147669	ENSG00000147669	POLR2K	8:101162811-101166230	q1	q2	OK	49.015	50.4149	0.0406282	0.0881758	0.89845	0.99995	no

ENSG00000147676	ENSG00000147676	MAL2	8:120177272-120259088	q1	q2	OK	2.16074	2.53439	0.230113	0.320778	0.63115	0.99995	no

ENSG00000147677	ENSG00000147677	EIF3H	8:117654368-117889107	q1	q2	OK	121.681	133.193	0.130418	0.122143	0.82565	0.99995	no

ENSG00000147679	ENSG00000147679	UTP23	8:117654368-117889107	q1	q2	OK	9.57328	10.5073	0.134301	0.0413599	0.9112	0.99995	no

ENSG00000147684	ENSG00000147684	NDUFB9	8:125500725-125740730	q1	q2	OK	129.56	127.382	-0.0244622	-0.0393568	0.95605	0.99995	no

ENSG00000147687	ENSG00000147687	TATDN1	8:125500725-125740730	q1	q2	OK	27.4992	27.2908	-0.0109719	-0.00923247	0.98955	0.99995	no

ENSG00000147689	ENSG00000147689	FAM83A	8:124191199-124222314	q1	q2	NOTEST	0.117751	0.0131905	-3.15816	0	1	1	no

ENSG00000147697	ENSG00000147697	GSDMC	8:130760441-130799134	q1	q2	NOTEST	0.00943887	0	#NAME?	0	1	1	no

ENSG00000147724	ENSG00000147724	FAM135B	8:139142265-139509065	q1	q2	OK	0.458807	0.48418	0.0776558	0.041948	0.89485	0.99995	no

ENSG00000147753	ENSG00000147753	TTTY7	Y:6317508-6325947	q1	q2	NOTEST	0	0	0	0	1	1	no

ENSG00000147761	ENSG00000147761	TTTY7B	Y:9544432-9552871	q1	q2	NOTEST	0	0	0	0	1	1	no

ENSG00000147789	ENSG00000147789	ZNF7	8:146052848-146127553	q1	q2	OK	18.5313	19.1555	0.0477987	0.0640816	0.9223	0.99995	no

ENSG00000147799	ENSG00000147799	ARHGAP39	8:145754562-145911194	q1	q2	OK	6.75132	5.60348	-0.268847	-0.540712	0.44545	0.99995	no

ENSG00000147804	ENSG00000147804	SLC39A4	8:145634875-145642279	q1	q2	NOTEST	0.00920454	0	#NAME?	0	1	1	no

ENSG00000147813	ENSG00000147813	NAPRT1	8:144655659-144660819	q1	q2	OK	5.49285	5.31247	-0.048172	-0.0549881	0.92725	0.99995	no

ENSG00000147852	ENSG00000147852	VLDLR	9:2422701-2660053	q1	q2	OK	18.8452	18.044	-0.062683	-0.100681	0.85515	0.99995	no

ENSG00000147853	ENSG00000147853	AK3	9:4711154-4742043	q1	q2	OK	36.4351	39.0127	0.0986166	0.233349	0.73265	0.99995	no

ENSG00000147854	ENSG00000147854	UHRF2	9:6413150-6507054	q1	q2	OK	31.022	33.3991	0.106518	0.213923	0.7057	0.99995	no

ENSG00000147862	ENSG00000147862	NFIB	9:14081841-14398982	q1	q2	OK	10.435	9.73519	-0.100152	-0.129127	0.7656	0.99995	no

ENSG00000147869	ENSG00000147869	CER1	9:14719721-14722715	q1	q2	OK	0.474093	0.588657	0.312257	0.280044	0.7	0.99995	no

ENSG00000147872	ENSG00000147872	PLIN2	9:19108372-19149288	q1	q2	OK	30.8464	30.7741	-0.00338426	-0.00758455	0.9911	0.99995	no

ENSG00000147873	ENSG00000147873	IFNA5	9:21304612-21305312	q1	q2	NOTEST	0.0474685	0.0981517	1.04804	0	1	1	no

ENSG00000147874	ENSG00000147874	HAUS6	9:19053140-19103117	q1	q2	OK	19.663	22.1205	0.169896	0.394252	0.5625	0.99995	no

ENSG00000147883	ENSG00000147883	CDKN2B	9:21802541-22121096	q1	q2	OK	0.82977	1.04573	0.333725	0.0865839	0.84235	0.99995	no

ENSG00000147885	ENSG00000147885	IFNA16	9:21216371-21217310	q1	q2	NOTEST	0.0339188	0.0332784	-0.0275004	0	1	1	no

ENSG00000147889	ENSG00000147889	CDKN2A	9:21802541-22121096	q1	q2	OK	3.05883	3.36636	0.138206	0.0462106	0.8803	0.99995	no

ENSG00000147894	ENSG00000147894	C9orf72	9:27546543-27573864	q1	q2	OK	22.2749	24.2186	0.120697	0.24314	0.70795	0.99995	no

ENSG00000147896	ENSG00000147896	IFNK	9:27325206-27529779	q1	q2	NOTEST	0.0266502	0	#NAME?	0	1	1	no

ENSG00000147905	ENSG00000147905	ZCCHC7	9:37120535-37358146	q1	q2	OK	16.7693	17.1979	0.0364081	0.0705697	0.9167	0.99995	no

ENSG00000147912	ENSG00000147912	FBXO10	9:37510888-38069210	q1	q2	OK	9.78667	9.49675	-0.0433845	-0.0360318	0.94995	0.99995	no

ENSG00000147926	ENSG00000147926	SPATA31A3	9:40700290-40713709	q1	q2	NOTEST	0	0	0	0	1	1	no

ENSG00000147955	ENSG00000147955	SIGMAR1	9:34634718-34637806	q1	q2	OK	39.0188	34.3414	-0.18422	-0.385083	0.552	0.99995	no

ENSG00000147996	ENSG00000147996	CBWD5	9:70432003-70497240	q1	q2	OK	7.55402	9.73776	0.366345	0.536498	0.38265	0.99995	no

ENSG00000148019	ENSG00000148019	CEP78	9:80850977-80894606	q1	q2	OK	26.4287	28.1499	0.0910238	0.141495	0.81125	0.99995	no

ENSG00000148053	ENSG00000148053	NTRK2	9:87283465-87638505	q1	q2	OK	6.68555	3.72172	-0.845075	-1.64299	0.017	0.99995	no

ENSG00000148057	ENSG00000148057	IDNK	9:86237963-86259045	q1	q2	OK	1.77183	1.6601	-0.0939764	-0.0904346	0.88805	0.99995	no

ENSG00000148082	ENSG00000148082	SHC3	9:91628059-91793682	q1	q2	OK	8.35129	7.64109	-0.12822	-0.20584	0.7577	0.99995	no

ENSG00000148090	ENSG00000148090	AUH	9:93976096-94124195	q1	q2	OK	7.00398	6.31161	-0.150166	-0.221018	0.74485	0.99995	no

ENSG00000148110	ENSG00000148110	HIATL1	9:97136832-97223324	q1	q2	OK	19.0538	18.5504	-0.0386275	-0.088623	0.9028	0.99995	no

ENSG00000148120	ENSG00000148120	C9orf3	9:97488982-97849441	q1	q2	OK	6.07608	5.83509	-0.0583864	-0.0680469	0.9153	0.99995	no

ENSG00000148123	ENSG00000148123	LPPR1	9:103790990-104087417	q1	q2	OK	4.58369	3.23347	-0.503428	-0.585841	0.32325	0.99995	no

ENSG00000148136	ENSG00000148136	OR13C4	9:107288533-107289490	q1	q2	NOTEST	0	0	0	0	1	1	no

ENSG00000148143	ENSG00000148143	ZNF462	9:109625377-109865269	q1	q2	OK	39.4848	39.2421	-0.00889554	-0.0128779	0.9798	0.99995	no

ENSG00000148153	ENSG00000148153	INIP	9:115446205-115480516	q1	q2	OK	17.6536	18.5158	0.068794	0.108319	0.8579	0.99995	no

ENSG00000148154	ENSG00000148154	UGCG	9:114659045-114697649	q1	q2	OK	8.26542	10.5979	0.358622	0.597606	0.25395	0.99995	no

ENSG00000148156	ENSG00000148156	ACTL7B	9:111616870-111619239	q1	q2	NOTEST	0	0.0113732	inf	0	1	1	no

ENSG00000148158	ENSG00000148158	SNX30	9:115513117-115653193	q1	q2	OK	6.60625	6.63588	0.00645711	0.0142722	0.9821	0.99995	no

ENSG00000148175	ENSG00000148175	STOM	9:124101354-124132531	q1	q2	OK	2.68987	2.23465	-0.267487	-0.302409	0.64775	0.99995	no

ENSG00000148180	ENSG00000148180	GSN	9:123940414-124095121	q1	q2	OK	25.8758	26.5273	0.0358732	0.0315535	0.96745	0.99995	no

ENSG00000148187	ENSG00000148187	MRRF	9:124999902-125085743	q1	q2	OK	15.958	17.8241	0.159549	0.208218	0.75975	0.99995	no

ENSG00000148200	ENSG00000148200	NR6A1	9:127279553-127535206	q1	q2	OK	7.55494	7.41406	-0.0271564	-0.0465895	0.9301	0.99995	no

ENSG00000148204	ENSG00000148204	CRB2	9:126118448-126692431	q1	q2	OK	41.5275	34.321	-0.274974	-0.558086	0.4296	0.99995	no

ENSG00000148215	ENSG00000148215	OR5C1	9:125551149-125552237	q1	q2	NOTEST	0	0	0	0	1	1	no

ENSG00000148218	ENSG00000148218	ALAD	9:116148596-116163613	q1	q2	OK	11.1364	10.975	-0.0210682	-0.0311321	0.9547	0.99995	no

ENSG00000148219	ENSG00000148219	ASTN2	9:119187503-120177348	q1	q2	OK	3.0743	3.12436	0.023305	0.0196537	0.98085	0.99995	no

ENSG00000148225	ENSG00000148225	WDR31	9:116075501-116102578	q1	q2	OK	1.42684	1.42159	-0.0053211	-0.00664618	0.9916	0.99995	no

ENSG00000148229	ENSG00000148229	POLE3	9:116169514-116191964	q1	q2	OK	43.1829	43.0966	-0.00288654	-0.00640906	0.99215	0.99995	no

ENSG00000148248	ENSG00000148248	SURF4	9:136228324-136242970	q1	q2	OK	1.18876	1.38512	0.220555	0.194866	0.72705	0.99995	no

ENSG00000148288	ENSG00000148288	GBGT1	9:135973106-136039332	q1	q2	OK	0.845977	0.806531	-0.0688892	-0.0194402	0.952	0.99995	no

ENSG00000148290	ENSG00000148290	SURF1	9:136218609-136228045	q1	q2	OK	0.613597	5.21773	3.08806	1.85049	0.055	0.99995	no

ENSG00000148291	ENSG00000148291	SURF2	9:136218609-136228045	q1	q2	OK	3.59978	3.3379	-0.10897	-0.0852584	0.8898	0.99995	no

ENSG00000148296	ENSG00000148296	SURF6	9:136197551-136203235	q1	q2	OK	2.57705	2.8308	0.135487	0.192499	0.77245	0.99995	no

ENSG00000148297	ENSG00000148297	MED22	9:136205159-136214986	q1	q2	OK	4.18486	4.35767	0.0583765	0.0524162	0.9251	0.99995	no

ENSG00000148300	ENSG00000148300	REXO4	9:136243116-136324508	q1	q2	OK	2.42643	2.15557	-0.17076	-0.134057	0.80205	0.99995	no

ENSG00000148303	ENSG00000148303	RPL7A	9:136215068-136218281	q1	q2	OK	0.290891	0.582194	1.00102	0.553044	0.15445	0.99995	no

ENSG00000148308	ENSG00000148308	GTF3C5	9:135906075-135933890	q1	q2	OK	53.6901	47.463	-0.177852	-0.351237	0.6031	0.99995	no

ENSG00000148331	ENSG00000148331	ASB6	9:132399170-132404444	q1	q2	OK	9.49884	9.53892	0.00607536	0.0114433	0.98715	0.99995	no

ENSG00000148334	ENSG00000148334	PTGES2	9:130882971-130891481	q1	q2	OK	16.4147	15.8567	-0.0499013	-0.0895129	0.8895	0.99995	no

ENSG00000148335	ENSG00000148335	NTMT1	9:132371162-132398209	q1	q2	OK	31.1341	27.4712	-0.180579	-0.329091	0.6509	0.99995	no

ENSG00000148337	ENSG00000148337	CIZ1	9:130928342-131017527	q1	q2	OK	65.4533	62.7242	-0.0614429	-0.0880982	0.87595	0.99995	no

ENSG00000148339	ENSG00000148339	SLC25A25	9:130823511-130871604	q1	q2	OK	9.62578	8.55067	-0.170865	-0.286304	0.6642	0.99995	no

ENSG00000148341	ENSG00000148341	SH3GLB2	9:131683173-131790582	q1	q2	OK	34.1124	29.2732	-0.220714	-0.21146	0.74455	0.99995	no

ENSG00000148343	ENSG00000148343	FAM73B	9:131798899-131834361	q1	q2	OK	12.7945	10.79	-0.245828	-0.301328	0.5304	0.99995	no

ENSG00000148344	ENSG00000148344	PTGES	9:132500609-132515326	q1	q2	OK	1.81286	1.33776	-0.438443	-0.407083	0.58495	0.99995	no

ENSG00000148346	ENSG00000148346	LCN2	9:130911349-130915734	q1	q2	NOTEST	0	0	0	0	1	1	no

ENSG00000148356	ENSG00000148356	LRSAM1	9:130213764-130265780	q1	q2	OK	7.19684	6.32857	-0.185483	-0.308789	0.62005	0.99995	no

ENSG00000148357	ENSG00000148357	HMCN2	9:133259835-133309510	q1	q2	NOTEST	0	0	0	0	1	1	no

ENSG00000148358	ENSG00000148358	GPR107	9:132815704-132902448	q1	q2	OK	8.58012	11.3242	0.400342	0.286715	0.3052	0.99995	no

ENSG00000148362	ENSG00000148362	C9orf142	9:139886869-139888436	q1	q2	OK	47.7984	41.0547	-0.219418	-0.43158	0.5532	0.99995	no

ENSG00000148377	ENSG00000148377	IDI2	10:1034337-1095110	q1	q2	NOTEST	0	0	0	0	1	1	no

ENSG00000148384	ENSG00000148384	INPP5E	9:139323070-139334274	q1	q2	OK	8.85069	8.47544	-0.0625015	-0.127311	0.8522	0.99995	no

ENSG00000148386	ENSG00000148386	LCN9	9:138555167-138558268	q1	q2	NOTEST	0.0419436	0	#NAME?	0	1	1	no

ENSG00000148396	ENSG00000148396	SEC16A	9:139334548-139372141	q1	q2	OK	39.5559	39.8087	0.00918788	0.015942	0.98055	0.99995	no

ENSG00000148399	ENSG00000148399	DPH7	9:140449355-140473387	q1	q2	OK	29.9578	28.548	-0.0695434	-0.133774	0.8462	0.99995	no

ENSG00000148400	ENSG00000148400	NOTCH1	9:139388895-139440314	q1	q2	OK	25.2186	25.7983	0.0327897	0.060253	0.90855	0.99995	no

ENSG00000148408	ENSG00000148408	CACNA1B	9:140513443-141019076	q1	q2	OK	0.737263	0.675945	-0.125275	-0.0348438	0.9169	0.99995	no

ENSG00000148411	ENSG00000148411	NACC2	9:138898382-138987131	q1	q2	OK	7.51241	6.67996	-0.169437	-0.348386	0.5897	0.99995	no

ENSG00000148426	ENSG00000148426	PROSER2	10:11865337-11936699	q1	q2	OK	0.591673	0.689796	0.22137	0.173204	0.73915	0.99995	no

ENSG00000148429	ENSG00000148429	USP6NL	10:11495944-11654226	q1	q2	OK	21.886	23.3995	0.0964656	0.212469	0.75855	0.99995	no

ENSG00000148444	ENSG00000148444	COMMD3	10:22604902-22620413	q1	q2	OK	28.1395	28.4584	0.0162604	0.0160192	0.98175	0.99995	no

ENSG00000148450	ENSG00000148450	MSRB2	10:23384434-23410942	q1	q2	OK	15.3652	13.8519	-0.149584	-0.222427	0.71365	0.99995	no

ENSG00000148459	ENSG00000148459	PDSS1	10:26986587-27150016	q1	q2	OK	7.87317	7.89813	0.00456559	0.00316385	0.996	0.99995	no

ENSG00000148468	ENSG00000148468	FAM171A1	10:15253641-15413061	q1	q2	OK	34.745	36.4167	0.0677952	0.146534	0.8037	0.99995	no

ENSG00000148481	ENSG00000148481	FAM188A	10:15820168-15902519	q1	q2	OK	14.9764	14.678	-0.0290398	-0.0434989	0.94305	0.99995	no

ENSG00000148482	ENSG00000148482	SLC39A12	10:18240767-18332221	q1	q2	NOTEST	0.0139585	0.0145042	0.0553273	0	1	1	no

ENSG00000148483	ENSG00000148483	TMEM236	10:17794250-17842867	q1	q2	NOTEST	0	0	0	0	1	1	no

ENSG00000148484	ENSG00000148484	RSU1	10:16632609-16859527	q1	q2	OK	35.3691	36.3337	0.038818	0.0676284	0.9228	0.99995	no

ENSG00000148488	ENSG00000148488	ST8SIA6	10:17360381-17496329	q1	q2	NOTEST	0.0349137	0.116062	1.73303	0	1	1	no

ENSG00000148498	ENSG00000148498	PARD3	10:34398487-35104253	q1	q2	OK	22.9072	23.5213	0.0381679	0.0629207	0.9116	0.99995	no

ENSG00000148513	ENSG00000148513	ANKRD30A	10:37414784-37673039	q1	q2	NOTEST	0	0	0	0	1	1	no

ENSG00000148516	ENSG00000148516	ZEB1	10:31476811-31818742	q1	q2	OK	41.55	43.9996	0.082643	0.147057	0.8033	0.99995	no

ENSG00000148541	ENSG00000148541	FAM13C	10:60936349-61122939	q1	q2	OK	2.7768	1.93965	-0.517626	-0.234115	0.4668	0.99995	no

ENSG00000148572	ENSG00000148572	NRBF2	10:64893049-64914783	q1	q2	OK	15.6164	14.5608	-0.100973	-0.200677	0.7761	0.99995	no

ENSG00000148584	ENSG00000148584	A1CF	10:52499077-52645435	q1	q2	NOTEST	0.0447706	0.0265971	-0.751283	0	1	1	no

ENSG00000148600	ENSG00000148600	CDHR1	10:85954409-85979377	q1	q2	OK	2.27164	2.29238	0.0131128	0.0193626	0.9787	0.99995	no

ENSG00000148602	ENSG00000148602	LRIT1	10:85991348-86001217	q1	q2	NOTEST	0	0	0	0	1	1	no

ENSG00000148604	ENSG00000148604	RGR	10:86004808-86019716	q1	q2	NOTEST	0.157309	0.112373	-0.485312	0	1	1	no

ENSG00000148606	ENSG00000148606	POLR3A	10:79734906-79789303	q1	q2	OK	9.67556	9.84662	0.0252832	0.0572217	0.92865	0.99995	no

ENSG00000148634	ENSG00000148634	HERC4	10:69681664-69835105	q1	q2	OK	19.9472	21.2862	0.0937313	0.148364	0.7858	0.99995	no

ENSG00000148655	ENSG00000148655	C10orf11	10:77360997-78320752	q1	q2	NOTEST	0	0.00616917	inf	0	1	1	no

ENSG00000148660	ENSG00000148660	CAMK2G	10:75572258-75634343	q1	q2	OK	15.5528	14.3564	-0.11548	-0.206297	0.7572	0.99995	no

ENSG00000148671	ENSG00000148671	ADIRF	10:88695296-88784489	q1	q2	NOTEST	0.0349639	9.15E-05	-8.57791	0	1	1	no

ENSG00000148672	ENSG00000148672	GLUD1	10:88810242-88951225	q1	q2	OK	47.2709	49.3404	0.0618156	0.124977	0.8423	0.99995	no

ENSG00000148677	ENSG00000148677	ANKRD1	10:92671852-92681033	q1	q2	OK	1.06037	1.42693	0.428345	0.430604	0.5326	0.99995	no

ENSG00000148680	ENSG00000148680	HTR7	10:92500577-92617671	q1	q2	NOTEST	0.0437356	0.0591197	0.43483	0	1	1	no

ENSG00000148688	ENSG00000148688	RPP30	10:92631472-92668312	q1	q2	OK	34.8352	35.1983	0.0149591	0.0246291	0.96925	0.99995	no

ENSG00000148690	ENSG00000148690	FRA10AC1	10:95427639-95462329	q1	q2	OK	18.7299	23.8999	0.351662	0.452177	0.38905	0.99995	no

ENSG00000148700	ENSG00000148700	ADD3	10:111756125-111895323	q1	q2	OK	67.9855	76.5832	0.171802	0.231537	0.56285	0.99995	no

ENSG00000148702	ENSG00000148702	HABP2	10:115310595-115423886	q1	q2	NOTEST	0	0.00892466	inf	0	1	1	no

ENSG00000148704	ENSG00000148704	VAX1	10:118888031-118897812	q1	q2	NOTEST	0.0311624	0	#NAME?	0	1	1	no

ENSG00000148719	ENSG00000148719	DNAJB12	10:74092587-74114988	q1	q2	OK	14.6474	13.4585	-0.122132	-0.225842	0.725	0.99995	no

ENSG00000148730	ENSG00000148730	EIF4EBP2	10:72164134-72188374	q1	q2	OK	58.7538	61.9285	0.075921	0.179057	0.79475	0.99995	no

ENSG00000148734	ENSG00000148734	NPFFR1	10:72007084-72043432	q1	q2	NOTEST	0.0169096	0.0246801	0.545511	0	1	1	no

ENSG00000148735	ENSG00000148735	PLEKHS1	10:115511212-115543188	q1	q2	NOTEST	0.0151821	0	#NAME?	0	1	1	no

ENSG00000148737	ENSG00000148737	TCF7L2	10:114710008-114927437	q1	q2	OK	25.4654	25.8934	0.0240468	0.0417964	0.932	0.99995	no

ENSG00000148773	ENSG00000148773	MKI67	10:129894922-129924649	q1	q2	OK	57.8739	57.4804	-0.00984353	-0.0135383	0.976	0.99995	no

ENSG00000148795	ENSG00000148795	CYP17A1	10:104590287-104597290	q1	q2	NOTEST	0.025899	0	#NAME?	0	1	1	no

ENSG00000148798	ENSG00000148798	INA	10:105036919-105050108	q1	q2	OK	35.8567	37.3598	0.0592444	0.144911	0.8331	0.99995	no

ENSG00000148803	ENSG00000148803	FUOM	10:135168657-135171529	q1	q2	OK	5.01733	3.92648	-0.353681	-0.360553	0.6343	0.99995	no

ENSG00000148814	ENSG00000148814	LRRC27	10:134145613-134195010	q1	q2	OK	25.114	24.4194	-0.0404629	-0.066649	0.9142	0.99995	no

ENSG00000148824	ENSG00000148824	MTG1	10:135192694-135382916	q1	q2	OK	50.9141	45.5172	-0.161654	-0.276339	0.6671	0.99995	no

ENSG00000148826	ENSG00000148826	NKX6-2	10:134598296-134599556	q1	q2	OK	2.48105	2.24591	-0.143651	-0.157916	0.81995	0.99995	no

ENSG00000148828	ENSG00000148828	CT476828.1	GL000228.1:30529-32226	q1	q2	NOTEST	0	0	0	0	1	1	no

ENSG00000148832	ENSG00000148832	PAOX	10:135192694-135382916	q1	q2	OK	3.28079	2.99227	-0.132804	-0.0739721	0.9341	0.99995	no

ENSG00000148834	ENSG00000148834	GSTO1	10:105995113-106027217	q1	q2	OK	46.1738	51.5085	0.157737	0.341973	0.62415	0.99995	no

ENSG00000148835	ENSG00000148835	TAF5	10:105127723-105156223	q1	q2	OK	8.2209	8.10309	-0.0208243	-0.027576	0.9703	0.99995	no

ENSG00000148840	ENSG00000148840	PPRC1	10:103892786-103910082	q1	q2	OK	16.0516	17.2653	0.105155	0.156834	0.7818	0.99995	no

ENSG00000148841	ENSG00000148841	ITPRIP	10:106071893-106098162	q1	q2	OK	10.6566	11.7138	0.136463	0.299391	0.6699	0.99995	no

ENSG00000148842	ENSG00000148842	CNNM2	10:104678049-104953056	q1	q2	OK	2.69464	2.27894	-0.24173	-0.138593	0.7812	0.99995	no

ENSG00000148843	ENSG00000148843	PDCD11	10:105156404-105206049	q1	q2	OK	17.1259	17.2472	0.0101829	0.0168089	0.9711	0.99995	no

ENSG00000148848	ENSG00000148848	ADAM12	10:127700949-128077024	q1	q2	OK	3.3576	2.92663	-0.198191	-0.249286	0.64455	0.99995	no

ENSG00000148908	ENSG00000148908	RGS10	10:121259339-121302220	q1	q2	OK	5.35338	6.63349	0.309317	0.359362	0.58615	0.99995	no

ENSG00000148925	ENSG00000148925	BTBD10	11:13409547-13484844	q1	q2	OK	24.1488	27.3422	0.179179	0.39706	0.5544	0.99995	no

ENSG00000148926	ENSG00000148926	ADM	11:10326226-10328944	q1	q2	OK	23.4229	31.6109	0.432503	0.728858	0.3078	0.99995	no

ENSG00000148935	ENSG00000148935	GAS2	11:22644078-22834601	q1	q2	OK	17.5324	19.6027	0.161031	0.19765	0.78955	0.99995	no

ENSG00000148942	ENSG00000148942	SLC5A12	11:26688565-26744974	q1	q2	NOTEST	0.0183715	0.0119219	-0.623851	0	1	1	no

ENSG00000148943	ENSG00000148943	LIN7C	11:27516122-27528320	q1	q2	OK	8.81955	9.95446	0.174639	0.382711	0.58675	0.99995	no

ENSG00000148948	ENSG00000148948	LRRC4C	11:40135752-41481323	q1	q2	OK	12.3103	11.8542	-0.054463	-0.0994695	0.8688	0.99995	no

ENSG00000148950	ENSG00000148950	IMMP1L	11:31453947-31531192	q1	q2	OK	43.0479	43.7572	0.0235752	0.0314362	0.9659	0.99995	no

ENSG00000148965	ENSG00000148965	SAA4	11:18252895-18270190	q1	q2	NOTEST	0.0450362	0.0438954	-0.0370149	0	1	1	no

ENSG00000148985	ENSG00000148985	PGAP2	11:3686816-3847601	q1	q2	OK	19.0043	18.1795	-0.0640157	-0.0504819	0.93245	0.99995	no

ENSG00000149016	ENSG00000149016	TUT1	11:62327072-62359649	q1	q2	OK	11.0305	10.0748	-0.130742	-0.0361952	0.9225	0.99995	no

ENSG00000149021	ENSG00000149021	SCGB1A1	11:62172574-62194196	q1	q2	NOTEST	0	0	0	0	1	1	no

ENSG00000149043	ENSG00000149043	SYT8	11:1848708-1858751	q1	q2	NOTEST	0.359747	0.258576	-0.476391	0	1	1	no

ENSG00000149050	ENSG00000149050	ZNF214	11:7020548-7041599	q1	q2	OK	2.74379	3.90114	0.507727	0.656978	0.28345	0.99995	no

ENSG00000149054	ENSG00000149054	ZNF215	11:6947634-7005863	q1	q2	OK	1.17246	1.33507	0.187376	0.204184	0.7395	0.99995	no

ENSG00000149084	ENSG00000149084	HSD17B12	11:43577985-43902276	q1	q2	OK	35.7472	39.9061	0.158778	0.35781	0.58145	0.99995	no

ENSG00000149089	ENSG00000149089	APIP	11:34874640-35042138	q1	q2	OK	21.3257	23.9966	0.170236	0.248276	0.7292	0.99995	no

ENSG00000149090	ENSG00000149090	PAMR1	11:35453369-35551848	q1	q2	OK	1.76441	1.24093	-0.507764	-0.201264	0.59605	0.99995	no

ENSG00000149091	ENSG00000149091	DGKZ	11:46354454-46402104	q1	q2	OK	19.8658	17.9397	-0.147132	-0.25327	0.6492	0.99995	no

ENSG00000149100	ENSG00000149100	EIF3M	11:32605343-32816204	q1	q2	OK	181.597	181.122	-0.00377924	-0.00804219	0.99085	0.99995	no

ENSG00000149115	ENSG00000149115	TNKS1BP1	11:57067111-57092426	q1	q2	OK	33.9997	32.591	-0.0610479	-0.104818	0.8525	0.99995	no

ENSG00000149124	ENSG00000149124	GLYAT	11:58407898-58499447	q1	q2	NOTEST	0	0.0471338	inf	0	1	1	no

ENSG00000149131	ENSG00000149131	SERPING1	11:57364859-57382326	q1	q2	OK	45.4541	42.6299	-0.0925445	-0.182349	0.78705	0.99995	no

ENSG00000149133	ENSG00000149133	OR5F1	11:55761156-55762101	q1	q2	NOTEST	0	0	0	0	1	1	no

ENSG00000149136	ENSG00000149136	SSRP1	11:57093076-57103351	q1	q2	OK	131.127	135.538	0.0477316	0.11399	0.8718	0.99995	no

ENSG00000149150	ENSG00000149150	SLC43A1	11:57252006-57283259	q1	q2	OK	1.43093	1.59916	0.160359	0.140243	0.78805	0.99995	no

ENSG00000149177	ENSG00000149177	PTPRJ	11:48002112-48189670	q1	q2	OK	5.06482	5.72604	0.177026	0.277526	0.59845	0.99995	no

ENSG00000149179	ENSG00000149179	C11orf49	11:46958239-47198676	q1	q2	OK	38.8452	39.1794	0.0123594	0.0156219	0.9785	0.99995	no

ENSG00000149182	ENSG00000149182	ARFGAP2	11:46958239-47198676	q1	q2	OK	45.7511	38.8789	-0.234819	-0.330775	0.4953	0.99995	no

ENSG00000149187	ENSG00000149187	CELF1	11:47487495-47606114	q1	q2	OK	55.954	56.1812	0.00584543	0.00576953	0.9875	0.99995	no

ENSG00000149196	ENSG00000149196	C11orf73	11:86013252-86056969	q1	q2	OK	42.4837	41.974	-0.0174129	-0.0375398	0.95965	0.99995	no

ENSG00000149201	ENSG00000149201	CCDC81	11:86085777-86134151	q1	q2	OK	1.02177	1.22662	0.263612	0.234209	0.6556	0.99995	no

ENSG00000149212	ENSG00000149212	SESN3	11:94883702-94967268	q1	q2	OK	22.3872	23.5318	0.0719375	0.163432	0.7969	0.99995	no

ENSG00000149218	ENSG00000149218	ENDOD1	11:94822973-94865809	q1	q2	OK	5.72935	5.94883	0.0542344	0.105746	0.87675	0.99995	no

ENSG00000149231	ENSG00000149231	CCDC82	11:96085932-96123087	q1	q2	OK	36.5326	48.2932	0.402636	0.644317	0.29365	0.99995	no

ENSG00000149243	ENSG00000149243	KLHL35	11:75133437-75141674	q1	q2	OK	3.03847	3.58653	0.239247	0.231438	0.68815	0.99995	no

ENSG00000149256	ENSG00000149256	TENM4	11:78363875-79151992	q1	q2	OK	11.8109	11.8723	0.00748398	0.00582531	0.9848	0.99995	no

ENSG00000149257	ENSG00000149257	SERPINH1	11:75273100-75283828	q1	q2	OK	231.442	237.173	0.0352867	0.0774082	0.91215	0.99995	no

ENSG00000149260	ENSG00000149260	CAPN5	11:76777978-76837201	q1	q2	NOTEST	0	0	0	0	1	1	no

ENSG00000149262	ENSG00000149262	INTS4	11:77532154-77705724	q1	q2	OK	15.3928	16.6061	0.109453	0.165876	0.768	0.99995	no

ENSG00000149269	ENSG00000149269	PAK1	11:77032751-77188074	q1	q2	OK	36.7706	37.5323	0.029579	0.0474071	0.9211	0.99995	no

ENSG00000149273	ENSG00000149273	RPS3	11:75110529-75133324	q1	q2	OK	1229.24	1133.25	-0.117301	-0.246957	0.72095	0.99995	no

ENSG00000149289	ENSG00000149289	ZC3H12C	11:109964086-110042566	q1	q2	OK	15.6701	14.4788	-0.114079	-0.252434	0.72045	0.99995	no

ENSG00000149292	ENSG00000149292	TTC12	11:113185250-113254266	q1	q2	OK	3.16468	2.97792	-0.0877556	-0.0816273	0.87625	0.99995	no

ENSG00000149294	ENSG00000149294	NCAM1	11:112830001-113149158	q1	q2	NOTEST	0	0.12755	inf	0	1	1	no

ENSG00000149295	ENSG00000149295	DRD2	11:113276042-113346413	q1	q2	OK	0.446194	0.546695	0.293065	0.208529	0.61875	0.99995	no

ENSG00000149300	ENSG00000149300	C11orf52	11:111779288-111797596	q1	q2	NOTEST	0	0	0	0	1	1	no

ENSG00000149305	ENSG00000149305	HTR3B	11:113775398-113817287	q1	q2	NOTEST	0.0577261	0.0136171	-2.0838	0	1	1	no

ENSG00000149308	ENSG00000149308	NPAT	11:108027941-108338258	q1	q2	OK	7.52382	7.2141	-0.0606453	-0.0484237	0.9234	0.99995	no

ENSG00000149311	ENSG00000149311	ATM	11:108027941-108338258	q1	q2	OK	24.703	26.1418	0.0816736	0.0748527	0.89255	0.99995	no

ENSG00000149313	ENSG00000149313	AASDHPPT	11:105921824-105972702	q1	q2	OK	40.8508	46.265	0.179558	0.431622	0.5398	0.99995	no

ENSG00000149328	ENSG00000149328	GLB1L2	11:134201767-134248235	q1	q2	OK	5.24193	4.66888	-0.167022	-0.240663	0.7082	0.99995	no

ENSG00000149346	ENSG00000149346	SLX4IP	20:10415950-10617477	q1	q2	OK	2.50012	2.49848	-0.000945952	-0.000995935	0.9959	0.99995	no

ENSG00000149357	ENSG00000149357	LAMTOR1	11:71709586-71823826	q1	q2	OK	56.8047	52.4955	-0.113816	-0.0758985	0.91005	0.99995	no

ENSG00000149380	ENSG00000149380	P4HA3	11:73723762-74035750	q1	q2	OK	0.671722	0.573836	-0.227224	-0.052318	0.9053	0.99995	no

ENSG00000149403	ENSG00000149403	GRIK4	11:120382467-120859613	q1	q2	OK	0.789167	0.653348	-0.272478	-0.279288	0.6477	0.99995	no

ENSG00000149418	ENSG00000149418	ST14	11:130029456-130080271	q1	q2	NOTEST	0.0359174	0.0206416	-0.79913	0	1	1	no

ENSG00000149428	ENSG00000149428	HYOU1	11:118914898-118927913	q1	q2	NOTEST	0.0220807	0.00727807	-1.60116	0	1	1	no

ENSG00000149435	ENSG00000149435	GGTLC1	20:23965689-23969416	q1	q2	NOTEST	0	0	0	0	1	1	no

ENSG00000149443	ENSG00000149443	C20orf78	20:18790371-18810797	q1	q2	NOTEST	0	0	0	0	1	1	no

ENSG00000149451	ENSG00000149451	ADAM33	20:3648611-3662893	q1	q2	OK	2.08108	1.64108	-0.342684	-0.426226	0.55435	0.99995	no

ENSG00000149452	ENSG00000149452	SLC22A8	11:62756625-62783311	q1	q2	NOTEST	0	0	0	0	1	1	no

ENSG00000149474	ENSG00000149474	CSRP2BP	20:18118516-18169031	q1	q2	OK	11.1334	10.1098	-0.139145	-0.233828	0.69135	0.99995	no

ENSG00000149476	ENSG00000149476	DAK	11:61066922-61136981	q1	q2	OK	18.0499	14.2345	-0.342592	-0.124861	0.6043	0.99995	no

ENSG00000149480	ENSG00000149480	MTA2	11:62360685-62369312	q1	q2	OK	74.3328	78.8074	0.0843308	0.157027	0.76955	0.99995	no

ENSG00000149483	ENSG00000149483	TMEM138	11:61066922-61136981	q1	q2	OK	19.8208	22.8895	0.207673	0.098271	0.78365	0.99995	no

ENSG00000149485	ENSG00000149485	FADS1	11:61447904-61634826	q1	q2	OK	80.0695	84.8953	0.0844331	0.0669228	0.90155	0.99995	no

ENSG00000149488	ENSG00000149488	TMC2	20:2517252-2622430	q1	q2	NOTEST	0.00842512	0.0673594	2.99911	0	1	1	no

ENSG00000149489	ENSG00000149489	ROM1	11:62369689-62382592	q1	q2	OK	1.32977	1.22989	-0.112645	-0.0409217	0.9333	0.99995	no

ENSG00000149499	ENSG00000149499	EML3	11:62369689-62382592	q1	q2	OK	15.413	13.6826	-0.171806	-0.213766	0.6843	0.99995	no

ENSG00000149503	ENSG00000149503	INCENP	11:61891444-61920635	q1	q2	OK	39.8477	41.2621	0.0503214	0.0637762	0.9236	0.99995	no

ENSG00000149506	ENSG00000149506	ZP1	11:60635034-60643166	q1	q2	NOTEST	0.0172836	0	#NAME?	0	1	1	no

ENSG00000149507	ENSG00000149507	OOSP2	11:59807747-59815517	q1	q2	NOTEST	0	0	0	0	1	1	no

ENSG00000149516	ENSG00000149516	MS4A3	11:59824059-59838601	q1	q2	NOTEST	0	0	0	0	1	1	no

ENSG00000149527	ENSG00000149527	PLCH2	1:2357418-2436969	q1	q2	NOTEST	0.481447	0.432108	-0.155984	0	1	1	no

ENSG00000149531	ENSG00000149531	FRG1B	20:29611856-29634010	q1	q2	OK	10.1939	7.44552	-0.453265	-0.548348	0.4101	0.99995	no

ENSG00000149532	ENSG00000149532	CPSF7	11:61170120-61258403	q1	q2	OK	61.2088	63.3508	0.0496231	0.0962114	0.86475	0.99995	no

ENSG00000149534	ENSG00000149534	MS4A2	11:59855733-59863444	q1	q2	NOTEST	0	0	0	0	1	1	no

ENSG00000149541	ENSG00000149541	B3GAT3	11:62382767-62389647	q1	q2	OK	19.5855	16.6279	-0.236181	-0.410801	0.5548	0.99995	no

ENSG00000149547	ENSG00000149547	EI24	11:125439111-125551018	q1	q2	OK	74.3351	70.8568	-0.0691379	-0.0853273	0.87995	0.99995	no

ENSG00000149548	ENSG00000149548	CCDC15	11:124824016-124911385	q1	q2	OK	2.80779	3.03801	0.113691	0.143885	0.79775	0.99995	no

ENSG00000149554	ENSG00000149554	CHEK1	11:125439111-125551018	q1	q2	OK	52.9118	53.4848	0.0155384	0.0134151	0.9802	0.99995	no

ENSG00000149557	ENSG00000149557	FEZ1	11:125315645-125369424	q1	q2	OK	134.175	132.402	-0.0191868	-0.0425828	0.9481	0.99995	no

ENSG00000149564	ENSG00000149564	ESAM	11:124617367-124635832	q1	q2	OK	1.76413	1.48471	-0.248774	-0.248961	0.72545	0.99995	no

ENSG00000149571	ENSG00000149571	KIRREL3	11:126225534-126875953	q1	q2	OK	0.909811	0.569823	-0.675053	-0.324207	0.44375	0.99995	no

ENSG00000149573	ENSG00000149573	MPZL2	11:118124117-118135251	q1	q2	NOTEST	0.0201436	0.12081	2.58435	0	1	1	no

ENSG00000149575	ENSG00000149575	SCN2B	11:118032665-118047388	q1	q2	NOTEST	0.191612	0.112616	-0.766775	0	1	1	no

ENSG00000149577	ENSG00000149577	SIDT2	11:117049448-117068160	q1	q2	OK	7.37586	7.05584	-0.0639952	-0.0996468	0.8465	0.99995	no

ENSG00000149582	ENSG00000149582	TMEM25	11:118307204-118473748	q1	q2	OK	0.513447	0.831596	0.695668	0.208201	0.29095	0.99995	no

ENSG00000149591	ENSG00000149591	TAGLN	11:117070036-117103241	q1	q2	OK	129.784	122.577	-0.0824288	-0.123154	0.8684	0.99995	no

ENSG00000149596	ENSG00000149596	JPH2	20:42740334-42816218	q1	q2	NOTEST	0.0528466	0.0419268	-0.333939	0	1	1	no

ENSG00000149599	ENSG00000149599	DUSP15	20:30435439-30539895	q1	q2	OK	2.84857	1.78588	-0.673603	-0.395879	0.5673	0.99995	no

ENSG00000149600	ENSG00000149600	COMMD7	20:31290492-31331803	q1	q2	OK	31.2652	29.4442	-0.0865734	-0.184011	0.7942	0.99995	no

ENSG00000149609	ENSG00000149609	C20orf144	20:32244892-32262269	q1	q2	NOTEST	0.379132	0.376232	-0.011079	0	1	1	no

ENSG00000149633	ENSG00000149633	KIAA1755	20:36838889-36965907	q1	q2	OK	5.60157	5.21365	-0.103539	-0.130277	0.84675	0.99995	no

ENSG00000149634	ENSG00000149634	SPATA25	20:44515127-44516274	q1	q2	OK	0.914269	0.740308	-0.304493	-0.262253	0.70435	0.99995	no

ENSG00000149635	ENSG00000149635	OCSTAMP	20:45169584-45179213	q1	q2	NOTEST	0.356776	0.378279	0.084432	0	1	1	no

ENSG00000149636	ENSG00000149636	DSN1	20:35380193-35402221	q1	q2	OK	19.576	19.2982	-0.0206198	-0.0443116	0.9464	0.99995	no

ENSG00000149639	ENSG00000149639	SOGA1	20:35405844-35492089	q1	q2	OK	14.0249	14.3639	0.0344607	0.0675778	0.9039	0.99995	no

ENSG00000149646	ENSG00000149646	CNBD2	20:34556511-34618622	q1	q2	NOTEST	0.144418	0.100997	-0.515939	0	1	1	no

ENSG00000149651	ENSG00000149651	SPINT4	20:44350989-44354469	q1	q2	NOTEST	0	0	0	0	1	1	no

ENSG00000149654	ENSG00000149654	CDH22	20:44802371-44937137	q1	q2	NOTEST	0.213976	0.161388	-0.406914	0	1	1	no

ENSG00000149656	ENSG00000149656	LINC00266-1	20:62887093-62944485	q1	q2	NOTEST	0.0553251	0.085355	0.625542	0	1	1	no

ENSG00000149657	ENSG00000149657	LSM14B	20:60697516-60710434	q1	q2	OK	46.4463	43.6704	-0.0889071	-0.194515	0.77055	0.99995	no

ENSG00000149658	ENSG00000149658	YTHDF1	20:61826780-61847586	q1	q2	OK	26.8117	27.541	0.0387186	0.0924645	0.8883	0.99995	no

ENSG00000149679	ENSG00000149679	CABLES2	20:60963687-60982341	q1	q2	OK	8.89724	8.58431	-0.0516551	-0.108316	0.87365	0.99995	no

ENSG00000149716	ENSG00000149716	ORAOV1	11:69455854-69490184	q1	q2	OK	14.7409	14.3926	-0.0344996	-0.018052	0.96655	0.99995	no

ENSG00000149735	ENSG00000149735	GPHA2	11:64685024-64703360	q1	q2	NOTEST	0	0	0	0	1	1	no

ENSG00000149742	ENSG00000149742	SLC22A9	11:63137260-63177766	q1	q2	NOTEST	0	0	0	0	1	1	no

ENSG00000149743	ENSG00000149743	TRPT1	11:63974149-64001824	q1	q2	OK	20.8121	19.9194	-0.063248	-0.0809697	0.9011	0.99995	no

ENSG00000149761	ENSG00000149761	NUDT22	11:63974149-64001824	q1	q2	OK	14.3627	12.5669	-0.192696	-0.178411	0.7723	0.99995	no

ENSG00000149781	ENSG00000149781	FERMT3	11:63974149-64001824	q1	q2	OK	1.04966	0.42119	-1.31737	-0.334423	0.16905	0.99995	no

ENSG00000149782	ENSG00000149782	PLCB3	11:64018994-64036622	q1	q2	OK	11.2425	11.5773	0.0423349	0.0688695	0.90015	0.99995	no

ENSG00000149792	ENSG00000149792	MRPL49	11:64888099-64902004	q1	q2	OK	26.7791	24.3213	-0.138885	-0.160251	0.80625	0.99995	no

ENSG00000149798	ENSG00000149798	CDC42EP2	11:65082288-65089900	q1	q2	OK	6.06261	5.15083	-0.235135	-0.361367	0.5985	0.99995	no

ENSG00000149806	ENSG00000149806	FAU	11:64888099-64902004	q1	q2	OK	253.923	228.408	-0.15278	-0.255828	0.71605	0.99995	no

ENSG00000149809	ENSG00000149809	TM7SF2	11:64851694-64883856	q1	q2	OK	41.8555	37.7302	-0.1497	-0.208483	0.73745	0.99995	no

ENSG00000149823	ENSG00000149823	VPS51	11:64851694-64883856	q1	q2	OK	45.2918	40.7888	-0.151077	-0.160358	0.76735	0.99995	no

ENSG00000149922	ENSG00000149922	TBX6	16:30097113-30103208	q1	q2	NOTEST	0.164033	0.104932	-0.644527	0	1	1	no

ENSG00000149923	ENSG00000149923	PPP4C	16:30087298-30096698	q1	q2	OK	88.4736	81.149	-0.124673	-0.29312	0.6783	0.99995	no

ENSG00000149925	ENSG00000149925	ALDOA	16:30064410-30081778	q1	q2	OK	295.792	280.295	-0.0776351	-0.1626	0.8174	0.99995	no

ENSG00000149926	ENSG00000149926	FAM57B	16:30034654-30064299	q1	q2	OK	10.0632	7.93497	-0.342796	-0.587887	0.39585	0.99995	no

ENSG00000149927	ENSG00000149927	DOC2A	16:30003644-30034591	q1	q2	OK	3.26701	3.00245	-0.12183	-0.0605147	0.90385	0.99995	no

ENSG00000149929	ENSG00000149929	HIRIP3	16:30003644-30034591	q1	q2	OK	23.1211	20.6707	-0.161623	-0.195323	0.75205	0.99995	no

ENSG00000149930	ENSG00000149930	TAOK2	16:29984961-30003582	q1	q2	OK	26.5846	24.3001	-0.129628	-0.276047	0.6933	0.99995	no

ENSG00000149932	ENSG00000149932	TMEM219	16:29952205-29984373	q1	q2	OK	37.7431	35.8215	-0.075387	-0.147087	0.839	0.99995	no

ENSG00000149948	ENSG00000149948	HMGA2	12:66151799-66360075	q1	q2	OK	67.037	84.7551	0.338343	0.798322	0.24405	0.99995	no

ENSG00000149968	ENSG00000149968	MMP3	11:102617698-102714534	q1	q2	NOTEST	0.0392012	0	#NAME?	0	1	1	no

ENSG00000149970	ENSG00000149970	CNKSR2	X:21392535-21672813	q1	q2	OK	3.92701	3.81225	-0.0427874	-0.052411	0.91635	0.99995	no

ENSG00000149972	ENSG00000149972	CNTN5	11:98891682-100229616	q1	q2	NOTEST	0.104015	0.0730143	-0.510542	0	1	1	no

ENSG00000150045	ENSG00000150045	KLRF1	12:9980076-9997606	q1	q2	NOTEST	0.0386958	0	#NAME?	0	1	1	no

ENSG00000150048	ENSG00000150048	CLEC1A	12:10222152-10264226	q1	q2	OK	1.15634	1.12728	-0.0367132	-0.0319313	0.95945	0.99995	no

ENSG00000150051	ENSG00000150051	MKX	10:27961803-28056723	q1	q2	OK	3.02638	2.93457	-0.0444414	-0.0670473	0.9183	0.99995	no

ENSG00000150054	ENSG00000150054	MPP7	10:28339921-28623415	q1	q2	OK	1.07337	0.92032	-0.221943	-0.20992	0.68245	0.99995	no

ENSG00000150076	ENSG00000150076	C10ORF68	10:32735067-33171802	q1	q2	NOTEST	0.11741	0.139967	0.253535	0	1	1	no

ENSG00000150093	ENSG00000150093	ITGB1	10:33189246-33371030	q1	q2	OK	162.359	199.702	0.298659	0.557117	0.3126	0.99995	no

ENSG00000150165	ENSG00000150165	ANXA8L1	10:47011752-47174093	q1	q2	NOTEST	0	0	0	0	1	1	no

ENSG00000150175	ENSG00000150175	FRMPD2P2	10:46657134-46681603	q1	q2	NOTEST	0	0	0	0	1	1	no

ENSG00000150201	ENSG00000150201	FXYD4	10:43867089-43871783	q1	q2	NOTEST	0	0	0	0	1	1	no

ENSG00000150244	ENSG00000150244	TRIM48	11:55029657-55038595	q1	q2	OK	0.695477	0.966946	0.475432	0.45839	0.51815	0.99995	no

ENSG00000150261	ENSG00000150261	OR8K1	11:56113420-56114507	q1	q2	NOTEST	0	0	0	0	1	1	no

ENSG00000150269	ENSG00000150269	OR5M9	11:56229944-56230877	q1	q2	NOTEST	0	0	0	0	1	1	no

ENSG00000150275	ENSG00000150275	PCDH15	10:55562530-57387702	q1	q2	OK	31.5947	35.3078	0.160304	0.229754	0.71925	0.99995	no

ENSG00000150276	ENSG00000150276	PPIAP26	13:53394631-53395153	q1	q2	NOTEST	0	0.0730115	inf	0	1	1	no

ENSG00000150281	ENSG00000150281	CTF1	16:30907927-30914881	q1	q2	OK	1.13012	0.766785	-0.559579	-0.565242	0.42575	0.99995	no

ENSG00000150316	ENSG00000150316	CWC15	11:94695786-94706776	q1	q2	NOTEST	0.178968	0.344407	0.944414	0	1	1	no

ENSG00000150337	ENSG00000150337	FCGR1A	1:149754226-149783928	q1	q2	NOTEST	0	0	0	0	1	1	no

ENSG00000150347	ENSG00000150347	ARID5B	10:63661058-63856703	q1	q2	OK	9.96442	7.83568	-0.346727	-0.767291	0.27785	0.99995	no

ENSG00000150361	ENSG00000150361	KLHL1	13:70274725-70713561	q1	q2	NOTEST	0.284899	0.279067	-0.0298407	0	1	1	no

ENSG00000150394	ENSG00000150394	CDH8	16:61681145-62070939	q1	q2	OK	6.42257	7.78284	0.277146	0.291156	0.5249	0.99995	no

ENSG00000150401	ENSG00000150401	DCUN1D2	13:114110133-114145267	q1	q2	OK	22.8555	21.6189	-0.0802502	-0.107274	0.8467	0.99995	no

ENSG00000150403	ENSG00000150403	TMCO3	13:114145309-114204542	q1	q2	OK	21.9992	23.0358	0.0664321	0.106059	0.83085	0.99995	no

ENSG00000150433	ENSG00000150433	TMEM218	11:124966397-124981659	q1	q2	OK	19.3333	21.5948	0.159597	0.255715	0.71565	0.99995	no

ENSG00000150455	ENSG00000150455	TIRAP	11:126152959-126215644	q1	q2	OK	4.93455	4.2994	-0.198783	-0.198439	0.72915	0.99995	no

ENSG00000150456	ENSG00000150456	N6AMT2	13:21302869-21348097	q1	q2	OK	5.17401	5.47789	0.0823355	0.0987271	0.88395	0.99995	no

ENSG00000150457	ENSG00000150457	LATS2	13:21547170-21635686	q1	q2	OK	7.09113	7.49845	0.0805773	0.172887	0.80315	0.99995	no

ENSG00000150459	ENSG00000150459	SAP18	13:21714652-21723221	q1	q2	OK	60.0227	67.6723	0.173057	0.316184	0.61715	0.99995	no

ENSG00000150471	ENSG00000150471	LPHN3	4:62066975-63031272	q1	q2	OK	19.24	18.7216	-0.0394011	-0.0731585	0.89765	0.99995	no

ENSG00000150477	ENSG00000150477	KIAA1328	18:33877676-34812135	q1	q2	OK	4.46872	3.9076	-0.193579	-0.0849172	0.82345	0.99995	no

ENSG00000150510	ENSG00000150510	FAM124A	13:51796502-51858377	q1	q2	OK	2.2406	1.73075	-0.372484	-0.405794	0.4161	0.99995	no

ENSG00000150526	ENSG00000150526	MIA2	14:39699434-39856306	q1	q2	NOTEST	0.070976	0	#NAME?	0	1	1	no

ENSG00000150527	ENSG00000150527	CTAGE5	14:39699434-39856306	q1	q2	OK	6.14729	6.06164	-0.0202432	-0.0325252	0.9598	0.99995	no

ENSG00000150540	ENSG00000150540	HNMT	2:138721589-138773930	q1	q2	OK	5.10536	5.17564	0.0197238	0.0221428	0.9694	0.99995	no

ENSG00000150551	ENSG00000150551	LYPD1	2:133174146-133429152	q1	q2	OK	126.653	125.224	-0.0163718	-0.0388256	0.95235	0.99995	no

ENSG00000150556	ENSG00000150556	LYPD6B	2:149894620-150071776	q1	q2	OK	0.550011	0.217181	-1.34056	-0.634791	0.0429	0.99995	no

ENSG00000150593	ENSG00000150593	PDCD4	10:112629500-112679032	q1	q2	OK	49.2472	39.2607	-0.326957	-0.446952	0.52465	0.99995	no

ENSG00000150594	ENSG00000150594	ADRA2A	10:112836789-112840658	q1	q2	NOTEST	0.431563	0.370393	-0.220515	0	1	1	no

ENSG00000150625	ENSG00000150625	GPM6A	4:176554084-176923815	q1	q2	OK	31.4478	30.1235	-0.0620694	-0.144091	0.8311	0.99995	no

ENSG00000150627	ENSG00000150627	WDR17	4:176986984-177103978	q1	q2	OK	5.32183	5.16108	-0.0442499	-0.0593262	0.90275	0.99995	no

ENSG00000150628	ENSG00000150628	SPATA4	4:177105788-177116822	q1	q2	NOTEST	0.262254	0.185355	-0.500676	0	1	1	no

ENSG00000150630	ENSG00000150630	VEGFC	4:177590771-177713881	q1	q2	OK	0.719857	0.854889	0.248027	0.258132	0.6911	0.99995	no

ENSG00000150636	ENSG00000150636	CCDC102B	18:66340924-66722426	q1	q2	OK	5.79425	5.27967	-0.134175	-0.0631101	0.87765	0.99995	no

ENSG00000150637	ENSG00000150637	CD226	18:67068290-67629039	q1	q2	NOTEST	0.272465	0.307854	0.176174	0	1	1	no

ENSG00000150656	ENSG00000150656	CNDP1	18:72201674-72254448	q1	q2	NOTEST	0.203873	0.140974	-0.532244	0	1	1	no

ENSG00000150667	ENSG00000150667	FSIP1	15:39892231-40213093	q1	q2	OK	3.03543	2.99011	-0.0217052	-0.0244678	0.96775	0.99995	no

ENSG00000150672	ENSG00000150672	DLG2	11:83166054-85338966	q1	q2	OK	3.67347	2.67125	-0.459631	-0.358931	0.37275	0.99995	no

ENSG00000150676	ENSG00000150676	CCDC83	11:85566143-85634725	q1	q2	NOTEST	0.0286269	0	#NAME?	0	1	1	no

ENSG00000150681	ENSG00000150681	RGS18	1:192127586-192154945	q1	q2	NOTEST	0	0	0	0	1	1	no

ENSG00000150687	ENSG00000150687	PRSS23	11:86502100-86666433	q1	q2	OK	45.0853	36.6627	-0.298343	-0.49049	0.45235	0.99995	no

ENSG00000150712	ENSG00000150712	MTMR12	5:32227099-32313115	q1	q2	OK	12.7529	14.2792	0.163096	0.305852	0.5758	0.99995	no

ENSG00000150722	ENSG00000150722	PPP1R1C	2:182818967-182996125	q1	q2	OK	1.20911	1.35932	0.16894	0.139265	0.80125	0.99995	no

ENSG00000150732	ENSG00000150732	RP11-134C1.1	1:188036428-188036656	q1	q2	NOTEST	0	0	0	0	1	1	no

ENSG00000150750	ENSG00000150750	C11orf53	11:111126706-111157126	q1	q2	NOTEST	0	0	0	0	1	1	no

ENSG00000150753	ENSG00000150753	CCT5	5:10250032-10267258	q1	q2	OK	230.134	258.397	0.167114	0.31698	0.62565	0.99995	no

ENSG00000150756	ENSG00000150756	FAM173B	5:10226441-10250027	q1	q2	OK	20.8644	18.9373	-0.139817	-0.21434	0.74805	0.99995	no

ENSG00000150760	ENSG00000150760	DOCK1	10:128593977-129250781	q1	q2	OK	23.8969	26.0609	0.125064	0.176127	0.6843	0.99995	no

ENSG00000150764	ENSG00000150764	DIXDC1	11:111797867-111893308	q1	q2	NOTEST	0.0428055	0	#NAME?	0	1	1	no

ENSG00000150768	ENSG00000150768	DLAT	11:111895537-112095422	q1	q2	NOTEST	0	0	0	0	1	1	no

ENSG00000150773	ENSG00000150773	PIH1D2	11:111895537-112095422	q1	q2	NOTEST	0	0	0	0	1	1	no

ENSG00000150776	ENSG00000150776	C11orf57	11:111895537-112095422	q1	q2	OK	23.9604	25.0312	0.0630765	0.093858	0.8769	0.99995	no

ENSG00000150779	ENSG00000150779	TIMM8B	11:111895537-112095422	q1	q2	OK	66.406	68.7264	0.04955	0.0605009	0.93455	0.99995	no

ENSG00000150782	ENSG00000150782	IL18	11:111895537-112095422	q1	q2	NOTEST	0	0	0	0	1	1	no

ENSG00000150783	ENSG00000150783	TEX12	11:111895537-112095422	q1	q2	NOTEST	0.209066	0.121934	-0.77786	0	1	1	no

ENSG00000150787	ENSG00000150787	PTS	11:112097087-112140678	q1	q2	OK	29.4923	33.2974	0.175073	0.318002	0.67	0.99995	no

ENSG00000150867	ENSG00000150867	PIP4K2A	10:22823777-23003484	q1	q2	OK	12.359	13.671	0.145552	0.265454	0.6409	0.99995	no

ENSG00000150873	ENSG00000150873	C2orf50	2:11273178-11286916	q1	q2	NOTEST	0.20183	0.192591	-0.0676	0	1	1	no

ENSG00000150893	ENSG00000150893	FREM2	13:39261265-39460074	q1	q2	OK	1.31842	1.59815	0.277591	0.509819	0.45345	0.99995	no

ENSG00000150907	ENSG00000150907	FOXO1	13:41129803-41240734	q1	q2	OK	8.19056	10.3963	0.344029	0.773974	0.2585	0.99995	no

ENSG00000150938	ENSG00000150938	CRIM1	2:36583068-36873230	q1	q2	OK	6.80784	7.05887	0.0522411	0.0506807	0.90925	0.99995	no

ENSG00000150961	ENSG00000150961	SEC24D	4:119643977-119759838	q1	q2	OK	18.3565	20.0872	0.129983	0.239804	0.67805	0.99995	no

ENSG00000150967	ENSG00000150967	ABCB9	12:123405497-123467456	q1	q2	OK	2.92785	2.67591	-0.129809	-0.0690296	0.84805	0.99995	no

ENSG00000150977	ENSG00000150977	RILPL2	12:123899935-123921264	q1	q2	OK	4.92675	4.62487	-0.0912252	-0.127858	0.85285	0.99995	no

ENSG00000150990	ENSG00000150990	DHX37	12:125431370-125473668	q1	q2	OK	5.08348	4.95845	-0.0359262	-0.0583916	0.92345	0.99995	no

ENSG00000150991	ENSG00000150991	UBC	12:125396149-125401914	q1	q2	OK	277.937	276.427	-0.00786372	-0.0125918	0.9823	0.99995	no

ENSG00000150995	ENSG00000150995	ITPR1	3:4535031-4928977	q1	q2	OK	3.18625	3.24907	0.0281664	0.0270619	0.95935	0.99995	no

ENSG00000151005	ENSG00000151005	TKTL2	4:164392256-164395047	q1	q2	NOTEST	0.0289657	0.018397	-0.654877	0	1	1	no

ENSG00000151006	ENSG00000151006	PRSS53	16:31085742-31107301	q1	q2	OK	1.28159	1.05782	-0.276834	-0.137705	0.79105	0.99995	no

ENSG00000151012	ENSG00000151012	SLC7A11	4:138948575-139163503	q1	q2	OK	12.1242	12.3429	0.0257881	0.062501	0.92935	0.99995	no

ENSG00000151014	ENSG00000151014	CCRN4L	4:139936942-140098372	q1	q2	OK	3.44701	4.02568	0.223887	0.121279	0.84385	0.99995	no

ENSG00000151023	ENSG00000151023	ENKUR	10:25270907-25305089	q1	q2	OK	7.23438	5.70454	-0.342761	-0.473963	0.4686	0.99995	no

ENSG00000151025	ENSG00000151025	GPR158	10:25401986-25891155	q1	q2	OK	1.60625	2.19851	0.452826	0.702994	0.31345	0.99995	no

ENSG00000151033	ENSG00000151033	LYZL2	10:30900717-30918691	q1	q2	NOTEST	0	0	0	0	1	1	no

ENSG00000151062	ENSG00000151062	CACNA2D4	12:1901122-2032033	q1	q2	NOTEST	0.0953351	0.159381	0.7414	0	1	1	no

ENSG00000151065	ENSG00000151065	DCP1B	12:2055219-2802108	q1	q2	OK	10.7858	10.2667	-0.0711601	-0.094483	0.8925	0.99995	no

ENSG00000151067	ENSG00000151067	CACNA1C	12:2055219-2802108	q1	q2	OK	2.30172	1.74198	-0.40198	-0.229707	0.55675	0.99995	no

ENSG00000151079	ENSG00000151079	KCNA6	12:4671369-4960277	q1	q2	OK	1.2143	1.20683	-0.00890476	-0.00654112	0.99175	0.99995	no

ENSG00000151090	ENSG00000151090	THRB	3:24158650-24541833	q1	q2	OK	1.19566	0.947218	-0.336036	-0.286285	0.45775	0.99995	no

ENSG00000151092	ENSG00000151092	NGLY1	3:25760434-25836025	q1	q2	OK	15.7627	16.8002	0.091962	0.17622	0.8013	0.99995	no

ENSG00000151093	ENSG00000151093	OXSM	3:25760434-25836025	q1	q2	OK	3.08815	2.47022	-0.322099	-0.164366	0.7543	0.99995	no

ENSG00000151116	ENSG00000151116	UEVLD	11:18551155-18610294	q1	q2	OK	7.36434	8.16532	0.148954	0.214539	0.66675	0.99995	no

ENSG00000151117	ENSG00000151117	TMEM86A	11:18714668-18814268	q1	q2	OK	1.23203	0.90245	-0.449121	-0.369577	0.47175	0.99995	no

ENSG00000151131	ENSG00000151131	C12orf45	12:105380087-105478355	q1	q2	OK	5.26644	5.23642	-0.0082491	-0.00528655	0.99255	0.99995	no

ENSG00000151135	ENSG00000151135	C12orf23	12:107349496-107380944	q1	q2	OK	29.6172	30.4957	0.0421716	0.0827366	0.9027	0.99995	no

ENSG00000151136	ENSG00000151136	BTBD11	12:107712189-108053419	q1	q2	OK	3.80063	3.93996	0.0519412	0.0884366	0.8884	0.99995	no

ENSG00000151148	ENSG00000151148	UBE3B	12:109785707-109974507	q1	q2	OK	13.1047	12.6208	-0.0542864	-0.0549115	0.9053	0.99995	no

ENSG00000151150	ENSG00000151150	ANK3	10:61786055-62493248	q1	q2	OK	22.8198	19.1893	-0.249988	-0.373079	0.4885	0.99995	no

ENSG00000151151	ENSG00000151151	IPMK	10:59951277-60027694	q1	q2	OK	3.72046	4.24066	0.188805	0.37063	0.59165	0.99995	no

ENSG00000151164	ENSG00000151164	RAD9B	12:110928901-111021125	q1	q2	NOTEST	0.361532	0.396996	0.134998	0	1	1	no

ENSG00000151176	ENSG00000151176	PLBD2	12:113736563-113827203	q1	q2	OK	8.76036	8.02778	-0.125989	-0.204533	0.76275	0.99995	no

ENSG00000151208	ENSG00000151208	DLG5	10:79542623-79686378	q1	q2	OK	40.0742	38.975	-0.040126	-0.0791454	0.8943	0.99995	no

ENSG00000151224	ENSG00000151224	MAT1A	10:82031575-82049440	q1	q2	NOTEST	0.0198073	0.00934381	-1.08395	0	1	1	no

ENSG00000151229	ENSG00000151229	SLC2A13	12:40019968-40499891	q1	q2	OK	2.68761	2.4876	-0.111568	-0.0943393	0.82015	0.99995	no

ENSG00000151233	ENSG00000151233	GXYLT1	12:42475646-42538681	q1	q2	OK	6.8255	7.27826	0.0926587	0.19727	0.7677	0.99995	no

ENSG00000151239	ENSG00000151239	TWF1	12:44187525-44200178	q1	q2	OK	27.2875	26.8144	-0.0252317	-0.0502579	0.92905	0.99995	no

ENSG00000151240	ENSG00000151240	DIP2C	10:320129-735683	q1	q2	OK	10.825	11.13	0.0400786	0.0665547	0.89505	0.99995	no

ENSG00000151247	ENSG00000151247	EIF4E	4:99792834-99916145	q1	q2	OK	51.3657	61.6091	0.262336	0.46004	0.49235	0.99995	no

ENSG00000151276	ENSG00000151276	MAGI1	3:65339199-66024509	q1	q2	OK	25.7578	30.93	0.263994	0.408298	0.36575	0.99995	no

ENSG00000151287	ENSG00000151287	TEX30	13:103418339-103426161	q1	q2	OK	10.8996	11.7844	0.112601	0.177046	0.80455	0.99995	no

ENSG00000151292	ENSG00000151292	CSNK1G3	5:122847792-122952739	q1	q2	OK	16.832	18.0121	0.0977628	0.198699	0.76235	0.99995	no

ENSG00000151303	ENSG00000151303	AGAP11	10:88695296-88784489	q1	q2	NOTEST	0	0.00889321	inf	0	1	1	no

ENSG00000151304	ENSG00000151304	SRFBP1	5:121297655-121413980	q1	q2	OK	4.93402	5.4751	0.150121	0.173908	0.7761	0.99995	no

ENSG00000151320	ENSG00000151320	AKAP6	14:32798478-33300567	q1	q2	OK	3.43161	3.78763	0.142409	0.130995	0.69675	0.99995	no

ENSG00000151322	ENSG00000151322	NPAS3	14:33404138-34273382	q1	q2	OK	13.4402	13.5651	0.0133454	0.0196026	0.96915	0.99995	no

ENSG00000151327	ENSG00000151327	FAM177A1	14:35514112-35786699	q1	q2	OK	22.9727	23.6865	0.0441425	0.0306373	0.958	0.99995	no

ENSG00000151332	ENSG00000151332	MBIP	14:36767769-36789882	q1	q2	OK	23.48	23.4923	0.000752587	0.00133453	0.99855	0.99995	no

ENSG00000151338	ENSG00000151338	MIPOL1	14:37667117-38021566	q1	q2	OK	2.83163	3.40611	0.266492	0.278125	0.62215	0.99995	no

ENSG00000151348	ENSG00000151348	EXT2	11:44117098-44266979	q1	q2	OK	31.5515	29.7073	-0.0868904	-0.193897	0.75715	0.99995	no

ENSG00000151353	ENSG00000151353	TMEM18	2:667334-697371	q1	q2	OK	20.7213	18.5398	-0.160493	-0.246669	0.73185	0.99995	no

ENSG00000151360	ENSG00000151360	ALLC	2:3705784-3836122	q1	q2	NOTEST	0	0	0	0	1	1	no

ENSG00000151364	ENSG00000151364	KCTD14	11:77726760-77791265	q1	q2	OK	1.36785	1.05115	-0.379937	-0.128767	0.8887	0.99995	no

ENSG00000151365	ENSG00000151365	THRSP	11:77726760-77791265	q1	q2	NOTEST	0	0.0252776	inf	0	1	1	no

ENSG00000151366	ENSG00000151366	NDUFC2	11:77726760-77791265	q1	q2	OK	164.465	166.346	0.0164026	0.032273	0.9618	0.99995	no

ENSG00000151376	ENSG00000151376	ME3	11:86142631-86383678	q1	q2	OK	1.58061	1.27437	-0.3107	-0.24317	0.59175	0.99995	no

ENSG00000151379	ENSG00000151379	MSGN1	2:17997762-17998368	q1	q2	NOTEST	0.0584542	0	#NAME?	0	1	1	no

ENSG00000151388	ENSG00000151388	ADAMTS12	5:33523639-33892297	q1	q2	OK	9.47367	11.6401	0.297104	0.518257	0.31105	0.99995	no

ENSG00000151413	ENSG00000151413	NUBPL	14:31959161-32330430	q1	q2	OK	7.11874	8.29232	0.220155	0.285205	0.62	0.99995	no

ENSG00000151414	ENSG00000151414	NEK7	1:198126092-198291550	q1	q2	OK	11.615	12.3712	0.0909888	0.170371	0.78065	0.99995	no

ENSG00000151418	ENSG00000151418	ATP6V1G3	1:198492351-198510075	q1	q2	NOTEST	0	0	0	0	1	1	no

ENSG00000151422	ENSG00000151422	FER	5:108083522-108532542	q1	q2	OK	5.57814	5.83549	0.0650703	0.110685	0.8208	0.99995	no

ENSG00000151445	ENSG00000151445	VIPAS39	14:77893017-77935817	q1	q2	OK	12.4887	11.4995	-0.11905	-0.128763	0.8326	0.99995	no

ENSG00000151458	ENSG00000151458	ANKRD50	4:125585206-125633887	q1	q2	OK	15.5055	16.811	0.116625	0.287825	0.6841	0.99995	no

ENSG00000151461	ENSG00000151461	UPF2	10:11962020-12085169	q1	q2	OK	13.8927	13.3611	-0.0562853	-0.120943	0.8445	0.99995	no

ENSG00000151465	ENSG00000151465	CDC123	10:12171635-12292588	q1	q2	OK	72.9844	75.72	0.0530854	0.079366	0.89875	0.99995	no

ENSG00000151466	ENSG00000151466	SCLT1	4:129730778-130037795	q1	q2	OK	8.13904	9.52862	0.227409	0.246531	0.7084	0.99995	no

ENSG00000151468	ENSG00000151468	CCDC3	10:12938626-13180291	q1	q2	OK	5.55033	4.52025	-0.29617	-0.341305	0.65845	0.99995	no

ENSG00000151470	ENSG00000151470	C4orf33	4:129730778-130037795	q1	q2	OK	6.3479	5.80831	-0.128161	-0.0705514	0.9047	0.99995	no

ENSG00000151474	ENSG00000151474	FRMD4A	10:13628926-14504141	q1	q2	OK	42.1965	37.6873	-0.163046	-0.205917	0.72755	0.99995	no

ENSG00000151475	ENSG00000151475	SLC25A31	4:128651532-128695448	q1	q2	NOTEST	0.0443353	0.0281809	-0.653738	0	1	1	no

ENSG00000151490	ENSG00000151490	PTPRO	12:15260716-15750333	q1	q2	OK	3.84311	3.88813	0.0168022	0.0193784	0.96895	0.99995	no

ENSG00000151491	ENSG00000151491	EPS8	12:15773091-16035263	q1	q2	OK	19.6528	19.04	-0.0457002	-0.073026	0.88215	0.99995	no

ENSG00000151498	ENSG00000151498	ACAD8	11:134123388-134135749	q1	q2	OK	19.812	19.2943	-0.0382063	-0.0717653	0.9053	0.99995	no

ENSG00000151500	ENSG00000151500	THYN1	11:134118172-134123264	q1	q2	OK	45.9429	46.9799	0.0322009	0.0667633	0.92455	0.99995	no

ENSG00000151502	ENSG00000151502	VPS26B	11:133938819-134117686	q1	q2	OK	24.7073	23.4628	-0.074558	-0.0570941	0.9056	0.99995	no

ENSG00000151503	ENSG00000151503	NCAPD3	11:133938819-134117686	q1	q2	OK	18.9488	17.9294	-0.0797812	-0.0437393	0.89815	0.99995	no

ENSG00000151532	ENSG00000151532	VTI1A	10:114206755-114578503	q1	q2	OK	10.5764	11.0008	0.0567709	0.085384	0.86615	0.99995	no

ENSG00000151552	ENSG00000151552	QDPR	4:17461883-17513857	q1	q2	OK	25.4492	26.1914	0.0414693	0.0841952	0.9021	0.99995	no

ENSG00000151553	ENSG00000151553	FAM160B1	10:116581502-116659591	q1	q2	OK	8.17114	7.83229	-0.0611021	-0.129761	0.8404	0.99995	no

ENSG00000151572	ENSG00000151572	ANO4	12:101111303-101522419	q1	q2	OK	0.735772	0.341749	-1.10632	-0.540232	0.2322	0.99995	no

ENSG00000151575	ENSG00000151575	TEX9	15:56536206-56757335	q1	q2	OK	9.03795	10.616	0.232172	0.295108	0.6583	0.99995	no

ENSG00000151576	ENSG00000151576	QTRTD1	3:113666747-113807269	q1	q2	OK	14.6214	15.1711	0.0532383	0.0683795	0.88275	0.99995	no

ENSG00000151577	ENSG00000151577	DRD3	3:113847498-113918254	q1	q2	NOTEST	0.0186534	0.0183775	-0.0214979	0	1	1	no

ENSG00000151611	ENSG00000151611	MMAA	4:146539414-146581187	q1	q2	OK	3.76779	3.07254	-0.294287	-0.296138	0.69475	0.99995	no

ENSG00000151612	ENSG00000151612	ZNF827	4:146601355-146859787	q1	q2	OK	12.0655	11.0146	-0.131464	-0.151517	0.70085	0.99995	no

ENSG00000151615	ENSG00000151615	POU4F2	4:147560044-147563626	q1	q2	OK	0.573613	0.46168	-0.313184	-0.343216	0.61165	0.99995	no

ENSG00000151617	ENSG00000151617	EDNRA	4:148402068-148466106	q1	q2	OK	26.2171	25.9007	-0.0175193	-0.0331363	0.96025	0.99995	no

ENSG00000151623	ENSG00000151623	NR3C2	4:148999912-149365850	q1	q2	NOTEST	0.220823	0.168089	-0.393669	0	1	1	no

ENSG00000151631	ENSG00000151631	AKR1C6P	10:4914092-4934167	q1	q2	NOTEST	0	0	0	0	1	1	no

ENSG00000151632	ENSG00000151632	AKR1C2	10:5029966-5060223	q1	q2	NOTEST	0.113188	0.077782	-0.541208	0	1	1	no

ENSG00000151640	ENSG00000151640	DPYSL4	10:134000403-134019280	q1	q2	OK	72.2207	65.7782	-0.134802	-0.242382	0.65565	0.99995	no

ENSG00000151650	ENSG00000151650	VENTX	10:135050907-135055433	q1	q2	NOTEST	0	0.00910227	inf	0	1	1	no

ENSG00000151651	ENSG00000151651	ADAM8	10:135075906-135090372	q1	q2	OK	1.13712	0.989268	-0.20095	-0.128637	0.7445	0.99995	no

ENSG00000151655	ENSG00000151655	ITIH2	10:7745231-7791483	q1	q2	NOTEST	0.183688	0.293381	0.675515	0	1	1	no

ENSG00000151657	ENSG00000151657	KIN	10:7792924-7829990	q1	q2	OK	14.7963	16.0899	0.120915	0.160491	0.818	0.99995	no

ENSG00000151665	ENSG00000151665	PIGF	2:46717888-46852881	q1	q2	OK	23.8003	23.5193	-0.0171325	-0.0183002	0.9804	0.99995	no

ENSG00000151687	ENSG00000151687	ANKAR	2:190539015-190630282	q1	q2	OK	0.708923	0.657904	-0.10775	-0.030313	0.909	0.99995	no

ENSG00000151689	ENSG00000151689	INPP1	2:190744334-191236391	q1	q2	OK	7.90378	9.6861	0.293373	0.234159	0.6751	0.99995	no

ENSG00000151690	ENSG00000151690	MFSD6	2:191273080-191399448	q1	q2	OK	1.17881	1.33705	0.18172	0.0929541	0.78735	0.99995	no

ENSG00000151692	ENSG00000151692	RNF144A	2:7052407-7218011	q1	q2	OK	18.0979	19.3154	0.0939299	0.198565	0.74945	0.99995	no

ENSG00000151693	ENSG00000151693	ASAP2	2:9346893-9563676	q1	q2	OK	20.177	22.264	0.141998	0.212177	0.69155	0.99995	no

ENSG00000151694	ENSG00000151694	ADAM17	2:9613786-9695921	q1	q2	OK	23.3648	24.9776	0.0963034	0.227707	0.7419	0.99995	no

ENSG00000151702	ENSG00000151702	FLI1	11:128499547-128683162	q1	q2	OK	3.65931	3.87409	0.0822853	0.0913928	0.88005	0.99995	no

ENSG00000151704	ENSG00000151704	KCNJ1	11:128706209-128737268	q1	q2	NOTEST	0.0836723	0.0261834	-1.6761	0	1	1	no

ENSG00000151715	ENSG00000151715	TMEM45B	11:129685713-129729898	q1	q2	NOTEST	0.139369	0.0686695	-1.02116	0	1	1	no

ENSG00000151718	ENSG00000151718	WWC2	4:184020445-184241930	q1	q2	OK	16.1507	15.0304	-0.103712	-0.160895	0.75305	0.99995	no

ENSG00000151725	ENSG00000151725	CENPU	4:185570766-185655287	q1	q2	OK	68.1072	67.294	-0.0173306	-0.0335598	0.9634	0.99995	no

ENSG00000151726	ENSG00000151726	ACSL1	4:185676748-185747972	q1	q2	OK	6.57299	6.82319	0.0538967	0.093467	0.89165	0.99995	no

ENSG00000151729	ENSG00000151729	SLC25A4	4:186064394-186071536	q1	q2	OK	16.2236	17.0882	0.0749093	0.14568	0.83285	0.99995	no

ENSG00000151743	ENSG00000151743	AMN1	12:31800093-31882108	q1	q2	OK	5.20307	5.26113	0.0160076	0.0170152	0.9759	0.99995	no

ENSG00000151746	ENSG00000151746	BICD1	12:32257050-32536567	q1	q2	OK	14.4361	13.8552	-0.0592483	-0.0717973	0.90175	0.99995	no

ENSG00000151748	ENSG00000151748	SAV1	14:50885218-51135049	q1	q2	OK	13.2095	13.5387	0.0355102	0.0352231	0.95315	0.99995	no

ENSG00000151773	ENSG00000151773	CCDC122	13:44398044-44468068	q1	q2	OK	0.580807	0.319499	-0.862248	-0.377755	0.57825	0.99995	no

ENSG00000151778	ENSG00000151778	SERP2	13:44947800-44971850	q1	q2	OK	1.28108	0.921596	-0.475156	-0.459659	0.52225	0.99995	no

ENSG00000151779	ENSG00000151779	NBAS	2:15307031-15701454	q1	q2	OK	15.0676	16.3317	0.116231	0.186358	0.7496	0.99995	no

ENSG00000151789	ENSG00000151789	ZNF385D	3:21459914-22414812	q1	q2	OK	2.26356	2.1377	-0.0825363	-0.0634039	0.8872	0.99995	no

ENSG00000151790	ENSG00000151790	TDO2	4:156750880-156841558	q1	q2	NOTEST	0	0	0	0	1	1	no

ENSG00000151806	ENSG00000151806	GUF1	4:44624085-44728612	q1	q2	OK	16.5466	19.7368	0.254358	0.446531	0.5096	0.99995	no

ENSG00000151812	ENSG00000151812	SLC35F4	14:58030639-58448912	q1	q2	NOTEST	0.102543	0.0709149	-0.532065	0	1	1	no

ENSG00000151834	ENSG00000151834	GABRA2	4:46250443-46477247	q1	q2	OK	0.533692	0.534045	0.000955395	0.000665637	0.9984	0.99995	no

ENSG00000151835	ENSG00000151835	SACS	13:23902964-24007841	q1	q2	OK	26.3017	31.7168	0.270092	0.511014	0.38835	0.99995	no

ENSG00000151838	ENSG00000151838	CCDC175	14:59895739-60043549	q1	q2	NOTEST	0	0	0	0	1	1	no

ENSG00000151846	ENSG00000151846	PABPC3	13:25670299-25673389	q1	q2	OK	0.608079	1.02082	0.747393	0.854764	0.2128	0.99995	no

ENSG00000151849	ENSG00000151849	CENPJ	13:25457170-25497018	q1	q2	OK	19.7535	24.625	0.318013	0.427049	0.43275	0.99995	no

ENSG00000151876	ENSG00000151876	FBXO4	5:41925355-41941845	q1	q2	OK	1.79423	2.18445	0.283904	0.268813	0.69925	0.99995	no

ENSG00000151881	ENSG00000151881	C5orf28	5:43444353-43483995	q1	q2	OK	6.36364	6.97661	0.132674	0.199026	0.7642	0.99995	no

ENSG00000151882	ENSG00000151882	CCL28	5:43376746-43412493	q1	q2	NOTEST	0.0180049	0	#NAME?	0	1	1	no

ENSG00000151883	ENSG00000151883	PARP8	5:49961732-50142356	q1	q2	OK	17.0155	18.9513	0.155448	0.215621	0.6313	0.99995	no

ENSG00000151892	ENSG00000151892	GFRA1	10:117816443-118032979	q1	q2	OK	17.8925	18.1863	0.0234932	0.0283065	0.93595	0.99995	no

ENSG00000151893	ENSG00000151893	CACUL1	10:120433678-120514761	q1	q2	OK	14.118	13.9209	-0.0202733	-0.0219296	0.9517	0.99995	no

ENSG00000151914	ENSG00000151914	DST	6:56322784-56819426	q1	q2	OK	42.4012	45.7608	0.110006	0.117277	0.84065	0.99995	no

ENSG00000151917	ENSG00000151917	BEND6	6:56819772-56892140	q1	q2	OK	0.847042	1.04221	0.299137	0.24805	0.65695	0.99995	no

ENSG00000151923	ENSG00000151923	TIAL1	10:121334198-121356541	q1	q2	OK	85.5069	91.3374	0.0951642	0.17808	0.75215	0.99995	no

ENSG00000151929	ENSG00000151929	BAG3	10:121410881-121437331	q1	q2	OK	14.8492	13.4863	-0.138895	-0.28153	0.6751	0.99995	no

ENSG00000151948	ENSG00000151948	GLT1D1	12:129337971-129469509	q1	q2	NOTEST	0.317987	0.282956	-0.168388	0	1	1	no

ENSG00000151952	ENSG00000151952	TMEM132D	12:129556269-130388211	q1	q2	OK	0.865572	0.779284	-0.151504	-0.192415	0.77365	0.99995	no

ENSG00000151962	ENSG00000151962	RBM46	4:155675907-155749965	q1	q2	NOTEST	0	0	0	0	1	1	no

ENSG00000151963	ENSG00000151963	RP11-775A3.1	10:38172521-38173037	q1	q2	NOTEST	0	0	0	0	1	1	no

ENSG00000151967	ENSG00000151967	SCHIP1	3:158680023-159615155	q1	q2	OK	44.1171	41.3053	-0.0950112	-0.137096	0.7858	0.99995	no

ENSG00000152022	ENSG00000152022	LIX1L	1:145475981-145516076	q1	q2	NOTEST	0.334994	0.383914	0.196649	0	1	1	no

ENSG00000152034	ENSG00000152034	MCHR2	6:100367785-100524289	q1	q2	NOTEST	0.0213598	0	#NAME?	0	1	1	no

ENSG00000152042	ENSG00000152042	NBPF11	1:146032646-146253110	q1	q2	OK	1.45901	1.75025	0.262577	0.269464	0.69775	0.99995	no

ENSG00000152049	ENSG00000152049	KCNE4	2:223916531-224063117	q1	q2	NOTEST	0.450827	0.402187	-0.164707	0	1	1	no

ENSG00000152056	ENSG00000152056	AP1S3	2:224616402-224702744	q1	q2	OK	1.82849	1.53089	-0.256282	-0.217974	0.7197	0.99995	no

ENSG00000152061	ENSG00000152061	RABGAP1L	1:174084437-174964445	q1	q2	OK	4.52514	4.76281	0.0738498	0.0829337	0.8443	0.99995	no

ENSG00000152076	ENSG00000152076	CCDC74B	2:130887193-130902707	q1	q2	OK	6.73801	4.67768	-0.526528	-0.650202	0.30415	0.99995	no

ENSG00000152078	ENSG00000152078	TMEM56	1:95582893-95729610	q1	q2	OK	0.671239	0.661324	-0.0214678	-0.01304	0.9786	0.99995	no

ENSG00000152082	ENSG00000152082	MZT2B	2:130908980-130948302	q1	q2	OK	93.1032	79.2963	-0.231578	-0.272301	0.67945	0.99995	no

ENSG00000152086	ENSG00000152086	TUBA3E	2:130949317-130956034	q1	q2	NOTEST	0.147516	0.0356042	-2.05075	0	1	1	no

ENSG00000152092	ENSG00000152092	ASTN1	1:176826437-177134109	q1	q2	OK	12.687	11.1428	-0.187243	-0.4399	0.5214	0.99995	no

ENSG00000152093	ENSG00000152093	CFC1B	2:131273569-131285579	q1	q2	NOTEST	0	0.0265767	inf	0	1	1	no

ENSG00000152102	ENSG00000152102	FAM168B	2:131805448-131851033	q1	q2	OK	76.5409	77.9108	0.0255907	0.0501995	0.93175	0.99995	no

ENSG00000152104	ENSG00000152104	PTPN14	1:214517514-214725792	q1	q2	OK	4.95196	5.29731	0.0972617	0.092864	0.78405	0.99995	no

ENSG00000152117	ENSG00000152117	AC093838.4	2:132250385-132279146	q1	q2	OK	15.101	12.7484	-0.24432	-0.488887	0.4686	0.99995	no

ENSG00000152127	ENSG00000152127	MGAT5	2:134877553-135212192	q1	q2	OK	19.0337	20.709	0.121701	0.159055	0.652	0.99995	no

ENSG00000152128	ENSG00000152128	TMEM163	2:135213329-135476570	q1	q2	OK	2.89386	2.00252	-0.53118	-0.66986	0.35825	0.99995	no

ENSG00000152133	ENSG00000152133	GPATCH11	2:37311593-37384208	q1	q2	OK	8.13856	7.92955	-0.0375353	-0.035981	0.94805	0.99995	no

ENSG00000152137	ENSG00000152137	HSPB8	12:119616446-119658936	q1	q2	NOTEST	0.459452	0.19815	-1.21332	0	1	1	no

ENSG00000152147	ENSG00000152147	GEMIN6	2:38978675-39012142	q1	q2	OK	7.86839	9.63343	0.291982	0.358138	0.6028	0.99995	no

ENSG00000152154	ENSG00000152154	TMEM178A	2:39476406-39945103	q1	q2	OK	9.89331	10.8519	0.133418	0.125672	0.8425	0.99995	no

ENSG00000152192	ENSG00000152192	POU4F1	13:78469615-79233314	q1	q2	NOTEST	0.193218	0.147391	-0.39058	0	1	1	no

ENSG00000152193	ENSG00000152193	RNF219	13:78469615-79233314	q1	q2	OK	19.0084	20.5685	0.113801	0.190095	0.7819	0.99995	no

ENSG00000152207	ENSG00000152207	CYSLTR2	13:49280950-49283498	q1	q2	NOTEST	0.115561	0.167407	0.534698	0	1	1	no

ENSG00000152208	ENSG00000152208	GRID2	4:93225549-94695707	q1	q2	NOTEST	0.233776	0.303932	0.378624	0	1	1	no

ENSG00000152213	ENSG00000152213	ARL11	13:50202434-50208008	q1	q2	NOTEST	0.00666544	0	#NAME?	0	1	1	no

ENSG00000152214	ENSG00000152214	RIT2	18:40323191-40695657	q1	q2	NOTEST	0.0748439	0.212903	1.50824	0	1	1	no

ENSG00000152217	ENSG00000152217	SETBP1	18:42260137-42648475	q1	q2	OK	9.79344	9.1194	-0.102878	-0.219313	0.72005	0.99995	no

ENSG00000152219	ENSG00000152219	ARL14EP	11:30344597-30359774	q1	q2	OK	23.09	27.6428	0.259633	0.251921	0.49975	0.99995	no

ENSG00000152223	ENSG00000152223	EPG5	18:43427573-43547240	q1	q2	OK	7.26527	7.2002	-0.01298	-0.0130979	0.9722	0.99995	no

ENSG00000152229	ENSG00000152229	PSTPIP2	18:43563501-43652238	q1	q2	OK	4.83072	5.84334	0.274555	0.315732	0.532	0.99995	no

ENSG00000152234	ENSG00000152234	ATP5A1	18:43664109-43708299	q1	q2	OK	307.125	307.583	0.00214947	0.00435475	0.99395	0.99995	no

ENSG00000152240	ENSG00000152240	HAUS1	18:43664109-43708299	q1	q2	OK	94.3304	96.7397	0.0363852	0.0296749	0.96525	0.99995	no

ENSG00000152242	ENSG00000152242	C18orf25	18:43753518-43846949	q1	q2	OK	8.09995	8.62947	0.0913592	0.144835	0.78685	0.99995	no

ENSG00000152253	ENSG00000152253	SPC25	2:169312371-169769881	q1	q2	OK	1.63112	2.02374	0.311164	0.102938	0.91155	0.99995	no

ENSG00000152254	ENSG00000152254	G6PC2	2:169312371-169769881	q1	q2	NOTEST	0	0	0	0	1	1	no

ENSG00000152256	ENSG00000152256	PDK1	2:173292081-173489823	q1	q2	OK	2.92134	3.60416	0.303033	0.196732	0.6759	0.99995	no

ENSG00000152266	ENSG00000152266	PTH	11:13513601-13517728	q1	q2	NOTEST	0	0	0	0	1	1	no

ENSG00000152268	ENSG00000152268	SPON1	11:13983913-14295237	q1	q2	NOTEST	0.269032	0.295633	0.136029	0	1	1	no

ENSG00000152270	ENSG00000152270	PDE3B	11:14665268-14892350	q1	q2	OK	4.66455	5.4866	0.234173	0.250842	0.6908	0.99995	no

ENSG00000152284	ENSG00000152284	TCF7L1	2:85360532-85537511	q1	q2	OK	42.3693	37.8268	-0.163612	-0.371652	0.5653	0.99995	no

ENSG00000152291	ENSG00000152291	TGOLN2	2:85545146-85555548	q1	q2	OK	24.8394	25.3461	0.0291375	0.0695664	0.91795	0.99995	no

ENSG00000152292	ENSG00000152292	SH2D6	2:85645843-85664152	q1	q2	NOTEST	0.0475632	0.0761301	0.678621	0	1	1	no

ENSG00000152315	ENSG00000152315	KCNK13	14:90528108-90652201	q1	q2	NOTEST	0.0209941	0	#NAME?	0	1	1	no

ENSG00000152332	ENSG00000152332	UHMK1	1:162467040-162499419	q1	q2	OK	8.74578	9.49758	0.118973	0.276885	0.69465	0.99995	no

ENSG00000152348	ENSG00000152348	ATG10	5:81267843-81574396	q1	q2	OK	9.30957	9.49843	0.0289752	0.00657194	0.9722	0.99995	no

ENSG00000152359	ENSG00000152359	POC5	5:74969948-75013313	q1	q2	OK	7.06637	8.6219	0.287037	0.388128	0.4767	0.99995	no

ENSG00000152377	ENSG00000152377	SPOCK1	5:136310986-136934068	q1	q2	OK	85.7851	82.0172	-0.0648018	-0.101755	0.83975	0.99995	no

ENSG00000152380	ENSG00000152380	FAM151B	5:79778111-79838382	q1	q2	OK	1.31486	0.782372	-0.748986	-0.550186	0.38205	0.99995	no

ENSG00000152382	ENSG00000152382	TADA1	1:166825746-166845564	q1	q2	OK	18.4219	19.9694	0.116371	0.252493	0.71785	0.99995	no

ENSG00000152402	ENSG00000152402	GUCY1A2	11:106544737-106889250	q1	q2	OK	1.5251	1.38787	-0.136031	-0.231684	0.72245	0.99995	no

ENSG00000152404	ENSG00000152404	CWF19L2	11:107197070-107328572	q1	q2	OK	9.92238	10.3483	0.06064	0.108857	0.8693	0.99995	no

ENSG00000152409	ENSG00000152409	JMY	5:78532011-78623038	q1	q2	OK	3.44943	2.94522	-0.227982	-0.435805	0.51775	0.99995	no

ENSG00000152413	ENSG00000152413	HOMER1	5:78668458-78810040	q1	q2	OK	14.5517	18.4054	0.338945	0.451344	0.42115	0.99995	no

ENSG00000152422	ENSG00000152422	XRCC4	5:82348664-82649606	q1	q2	OK	9.85001	12.3336	0.324401	0.202076	0.76805	0.99995	no

ENSG00000152430	ENSG00000152430	BOLL	2:198557829-198651486	q1	q2	NOTEST	0.00899355	0.00934047	0.0546052	0	1	1	no

ENSG00000152433	ENSG00000152433	ZNF547	19:57874844-57988938	q1	q2	OK	4.0576	3.52183	-0.204299	-0.0906394	0.82895	0.99995	no

ENSG00000152439	ENSG00000152439	ZNF773	19:57999078-58029772	q1	q2	OK	6.72983	6.69884	-0.00665828	-0.00632952	0.99135	0.99995	no

ENSG00000152443	ENSG00000152443	ZNF776	19:58193336-58269527	q1	q2	OK	14.1797	13.2898	-0.0935041	-0.101435	0.83315	0.99995	no

ENSG00000152454	ENSG00000152454	ZNF256	19:58452200-58459043	q1	q2	OK	4.65358	4.67059	0.00526386	0.00807478	0.992	0.99995	no

ENSG00000152455	ENSG00000152455	SUV39H2	10:14920818-14996431	q1	q2	OK	34.563	37.9551	0.135067	0.229475	0.74405	0.99995	no

ENSG00000152457	ENSG00000152457	DCLRE1C	10:14920818-14996431	q1	q2	OK	6.84286	7.258	0.0849734	0.0679629	0.8803	0.99995	no

ENSG00000152463	ENSG00000152463	OLAH	10:15057070-15130775	q1	q2	NOTEST	0.111602	0.0479652	-1.21831	0	1	1	no

ENSG00000152464	ENSG00000152464	RPP38	10:15137383-15210692	q1	q2	OK	14.097	12.4559	-0.178554	-0.210019	0.778	0.99995	no

ENSG00000152465	ENSG00000152465	NMT2	10:15137383-15210692	q1	q2	OK	14.7955	15.4178	0.0594374	0.0908207	0.88675	0.99995	no

ENSG00000152467	ENSG00000152467	ZSCAN1	19:58545399-58565999	q1	q2	NOTEST	0.0409127	0	#NAME?	0	1	1	no

ENSG00000152475	ENSG00000152475	ZNF837	19:58878984-58892427	q1	q2	OK	2.673	2.13845	-0.321895	-0.415195	0.56195	0.99995	no

ENSG00000152484	ENSG00000152484	USP12	13:27640292-27746033	q1	q2	OK	14.1926	15.7042	0.146015	0.341481	0.62475	0.99995	no

ENSG00000152487	ENSG00000152487	ARL5B-AS1	10:18429605-18948214	q1	q2	OK	1.81711	1.98181	0.12517	0.0416344	0.90735	0.99995	no

ENSG00000152492	ENSG00000152492	CCDC50	3:190984956-191116459	q1	q2	OK	19.8423	21.8803	0.141057	0.210873	0.6366	0.99995	no

ENSG00000152495	ENSG00000152495	CAMK4	5:110559350-110830584	q1	q2	OK	3.47519	2.78792	-0.317903	-0.365819	0.41825	0.99995	no

ENSG00000152503	ENSG00000152503	TRIM36	5:114460458-114516243	q1	q2	OK	7.18571	7.64343	0.0890885	0.128648	0.8186	0.99995	no

ENSG00000152518	ENSG00000152518	ZFP36L2	2:43393799-43823185	q1	q2	OK	13.04	12.0562	-0.113163	-0.157184	0.82995	0.99995	no

ENSG00000152520	ENSG00000152520	PAN3	13:28712642-28869475	q1	q2	OK	21.5778	21.8294	0.0167258	0.0391622	0.9544	0.99995	no

ENSG00000152527	ENSG00000152527	PLEKHH2	2:43864411-43995126	q1	q2	OK	3.23685	4.6111	0.510521	0.504333	0.21905	0.99995	no

ENSG00000152556	ENSG00000152556	PFKM	12:48436680-48540187	q1	q2	OK	51.8176	49.5623	-0.0641997	-0.0984312	0.8388	0.99995	no

ENSG00000152558	ENSG00000152558	TMEM123	11:102267062-102369532	q1	q2	OK	66.0993	73.9008	0.160956	0.323766	0.59145	0.99995	no

ENSG00000152578	ENSG00000152578	GRIA4	11:105480720-105852819	q1	q2	OK	9.78736	10.0145	0.0331014	0.0556787	0.92515	0.99995	no

ENSG00000152580	ENSG00000152580	IGSF10	3:150803483-151176497	q1	q2	NOTEST	0.0768131	0.0559593	-0.456976	0	1	1	no

ENSG00000152582	ENSG00000152582	SPEF2	5:35617945-35827120	q1	q2	OK	1.78965	2.03444	0.184955	0.123881	0.768	0.99995	no

ENSG00000152583	ENSG00000152583	SPARCL1	4:88394486-88452213	q1	q2	OK	110.077	128.241	0.220339	0.420351	0.54595	0.99995	no

ENSG00000152591	ENSG00000152591	DSPP	4:88489186-88655108	q1	q2	NOTEST	0.0058126	0.0112506	0.952751	0	1	1	no

ENSG00000152592	ENSG00000152592	DMP1	4:88489186-88655108	q1	q2	NOTEST	0.068765	0.0580594	-0.244144	0	1	1	no

ENSG00000152595	ENSG00000152595	MEPE	4:88742562-88767969	q1	q2	NOTEST	0	0	0	0	1	1	no

ENSG00000152601	ENSG00000152601	MBNL1	3:151961616-152214602	q1	q2	OK	12.4308	13.742	0.144682	0.278504	0.6403	0.99995	no

ENSG00000152611	ENSG00000152611	CAPSL	5:35904396-35938881	q1	q2	NOTEST	0	0	0	0	1	1	no

ENSG00000152620	ENSG00000152620	NADK2	5:36192693-36242381	q1	q2	OK	25.6238	20.4325	-0.326618	-0.688241	0.30015	0.99995	no

ENSG00000152642	ENSG00000152642	GPD1L	3:32147180-32210205	q1	q2	OK	3.17242	2.89515	-0.131947	-0.20889	0.74845	0.99995	no

ENSG00000152661	ENSG00000152661	GJA1	6:121756837-121770873	q1	q2	OK	9.22144	12.1754	0.400906	0.810146	0.249	0.99995	no

ENSG00000152669	ENSG00000152669	CCNO	5:54526979-54529508	q1	q2	NOTEST	0	0.0199827	inf	0	1	1	no

ENSG00000152670	ENSG00000152670	DDX4	5:54921672-55112985	q1	q2	NOTEST	0.106601	0.067437	-0.660614	0	1	1	no

ENSG00000152672	ENSG00000152672	CLEC4F	2:71035774-71047732	q1	q2	NOTEST	0	0	0	0	1	1	no

ENSG00000152683	ENSG00000152683	SLC30A6	2:32390932-32449448	q1	q2	OK	12.6839	13.4325	0.0827256	0.172637	0.78755	0.99995	no

ENSG00000152684	ENSG00000152684	PELO	5:51971026-52390609	q1	q2	OK	6.12339	6.91827	0.176079	0.143257	0.8023	0.99995	no

ENSG00000152689	ENSG00000152689	RASGRP3	2:33661390-33789817	q1	q2	NOTEST	0.15349	0.225045	0.552073	0	1	1	no

ENSG00000152700	ENSG00000152700	SAR1B	5:133936833-134063513	q1	q2	OK	20.6027	23.7586	0.205615	0.190276	0.7826	0.99995	no

ENSG00000152705	ENSG00000152705	CATSPER3	5:134240595-134347392	q1	q2	OK	0.86314	0.634793	-0.443309	-0.148682	0.8031	0.99995	no

ENSG00000152726	ENSG00000152726	FAM21B	10:47894022-47949412	q1	q2	OK	2.08095	2.31279	0.152395	0.229062	0.72695	0.99995	no

ENSG00000152749	ENSG00000152749	GPR180	13:95254156-95286899	q1	q2	OK	3.28733	3.72741	0.181259	0.375155	0.59245	0.99995	no

ENSG00000152760	ENSG00000152760	TCTEX1D1	1:67218141-67244470	q1	q2	NOTEST	0.169381	0.111996	-0.596819	0	1	1	no

ENSG00000152763	ENSG00000152763	WDR78	1:67278567-67390570	q1	q2	OK	2.06875	1.87526	-0.141672	-0.128109	0.7882	0.99995	no

ENSG00000152766	ENSG00000152766	ANKRD22	10:90581888-90611575	q1	q2	NOTEST	0	0	0	0	1	1	no

ENSG00000152767	ENSG00000152767	FARP1	13:98794815-99102027	q1	q2	OK	37.4185	37.6013	0.00702925	0.0125481	0.9783	0.99995	no

ENSG00000152778	ENSG00000152778	IFIT5	10:91174342-91180758	q1	q2	OK	8.33083	9.07837	0.123974	0.208951	0.76315	0.99995	no

ENSG00000152779	ENSG00000152779	SLC16A12	10:91190050-91316398	q1	q2	NOTEST	0.0873537	0.105856	0.277161	0	1	1	no

ENSG00000152782	ENSG00000152782	PANK1	10:91342744-91410579	q1	q2	OK	18.6942	21.2789	0.186833	0.356192	0.55185	0.99995	no

ENSG00000152784	ENSG00000152784	PRDM8	4:81104433-81125483	q1	q2	OK	7.17301	4.34216	-0.724164	-1.01759	0.07965	0.99995	no

ENSG00000152785	ENSG00000152785	BMP3	4:81952118-81978685	q1	q2	OK	1.88534	2.6637	0.498606	0.835662	0.2239	0.99995	no

ENSG00000152795	ENSG00000152795	HNRNPDL	4:83343716-83351294	q1	q2	OK	568.081	608.846	0.0999802	0.198341	0.7801	0.99995	no

ENSG00000152804	ENSG00000152804	HHEX	10:94447944-94455403	q1	q2	OK	1.78998	1.14883	-0.639778	-0.630388	0.398	0.99995	no

ENSG00000152818	ENSG00000152818	UTRN	6:144606836-145174170	q1	q2	OK	23.2056	23.0275	-0.0111127	-0.00711789	0.97895	0.99995	no

ENSG00000152822	ENSG00000152822	GRM1	6:146348781-146758734	q1	q2	NOTEST	0.265	0.229421	-0.207995	0	1	1	no

ENSG00000152894	ENSG00000152894	PTPRK	6:128289923-128841870	q1	q2	OK	7.89879	9.49813	0.266013	0.408913	0.41535	0.99995	no

ENSG00000152904	ENSG00000152904	GGPS1	1:235294497-235507847	q1	q2	OK	40.5947	42.7748	0.0754706	0.0947139	0.88775	0.99995	no

ENSG00000152910	ENSG00000152910	CNTNAP4	16:76311175-76692375	q1	q2	NOTEST	0.110983	0.0945365	-0.231397	0	1	1	no

ENSG00000152926	ENSG00000152926	ZNF117	7:64432153-64467121	q1	q2	OK	16.559	18.1021	0.128545	0.237451	0.7093	0.99995	no

ENSG00000152931	ENSG00000152931	PART1	5:58264864-59843484	q1	q2	NOTEST	0.0184705	0.0664731	1.84754	0	1	1	no

ENSG00000152932	ENSG00000152932	RAB3C	5:57878047-58159241	q1	q2	OK	7.61135	5.97834	-0.348408	-0.248664	0.6338	0.99995	no

ENSG00000152936	ENSG00000152936	IFLTD1	12:25562226-25801513	q1	q2	NOTEST	0	0.095663	inf	0	1	1	no

ENSG00000152939	ENSG00000152939	MARVELD2	5:68710938-68740157	q1	q2	NOTEST	0	0	0	0	1	1	no

ENSG00000152942	ENSG00000152942	RAD17	5:68646810-68710628	q1	q2	OK	6.88693	5.09895	-0.433662	-0.449819	0.55485	0.99995	no

ENSG00000152944	ENSG00000152944	MED21	12:27175478-27219276	q1	q2	OK	17.2715	18.1446	0.0711476	0.139621	0.83765	0.99995	no

ENSG00000152952	ENSG00000152952	PLOD2	3:145781828-145881440	q1	q2	OK	4.91941	5.45173	0.148229	0.209511	0.71545	0.99995	no

ENSG00000152953	ENSG00000152953	STK32B	4:5053168-5502725	q1	q2	OK	8.54629	9.02107	0.0779998	0.145098	0.82535	0.99995	no

ENSG00000152954	ENSG00000152954	NRSN1	6:24126349-24155128	q1	q2	OK	0.527191	0.455526	-0.210793	-0.148909	0.7594	0.99995	no

ENSG00000152969	ENSG00000152969	JAKMIP1	4:6027925-6202318	q1	q2	OK	1.19788	1.58804	0.406758	0.481071	0.4832	0.99995	no

ENSG00000152977	ENSG00000152977	ZIC1	3:147103832-147228080	q1	q2	OK	33.8428	30.729	-0.13925	-0.134222	0.8344	0.99995	no

ENSG00000152990	ENSG00000152990	GPR125	4:22346693-22517677	q1	q2	OK	35.8002	36.3109	0.0204369	0.0360155	0.9428	0.99995	no

ENSG00000153002	ENSG00000153002	CPB1	3:148508888-148677899	q1	q2	NOTEST	0.0981017	0.0636732	-0.623592	0	1	1	no

ENSG00000153006	ENSG00000153006	SREK1IP1	5:64013970-64064512	q1	q2	OK	9.64369	9.92174	0.0410081	0.0484737	0.90185	0.99995	no

ENSG00000153012	ENSG00000153012	LGI2	4:25000468-25032501	q1	q2	OK	7.5144	9.16312	0.28618	0.631282	0.35595	0.99995	no

ENSG00000153015	ENSG00000153015	CWC27	5:64064756-64314590	q1	q2	OK	30.8341	30.542	-0.0137334	-0.0288165	0.96705	0.99995	no

ENSG00000153029	ENSG00000153029	MR1	1:181003066-181031074	q1	q2	NOTEST	0.0521566	0.0133299	-1.96818	0	1	1	no

ENSG00000153037	ENSG00000153037	SRP19	5:112043194-112258236	q1	q2	OK	62.731	67.3085	0.10161	0.079454	0.91735	0.99995	no

ENSG00000153044	ENSG00000153044	CENPH	5:68485374-68506184	q1	q2	OK	45.0908	50.5316	0.164353	0.371179	0.58255	0.99995	no

ENSG00000153046	ENSG00000153046	CDYL	6:4706392-4955785	q1	q2	OK	22.7856	22.9407	0.00978916	0.0174297	0.98065	0.99995	no

ENSG00000153048	ENSG00000153048	CARHSP1	16:8768421-8962866	q1	q2	OK	101.721	101.56	-0.00229049	-0.00199555	0.99755	0.99995	no

ENSG00000153060	ENSG00000153060	TEKT5	16:10721357-10788802	q1	q2	NOTEST	0.0168062	0.0756216	2.16981	0	1	1	no

ENSG00000153064	ENSG00000153064	BANK1	4:102332442-102995969	q1	q2	NOTEST	0.188038	0.0675266	-1.47749	0	1	1	no

ENSG00000153066	ENSG00000153066	TXNDC11	16:11762269-11838362	q1	q2	OK	8.42321	7.94948	-0.083511	-0.041248	0.93485	0.99995	no

ENSG00000153071	ENSG00000153071	DAB2	5:39284363-39462402	q1	q2	OK	8.29821	8.26848	-0.00517815	-0.00783332	0.9875	0.99995	no

ENSG00000153086	ENSG00000153086	ACMSD	2:135493033-135716912	q1	q2	NOTEST	0.374354	0.298007	-0.329054	0	1	1	no

ENSG00000153093	ENSG00000153093	ACOXL	2:111490149-111875799	q1	q2	NOTEST	0.0109202	0.0219399	1.00656	0	1	1	no

ENSG00000153094	ENSG00000153094	BCL2L11	2:111876954-111926024	q1	q2	OK	8.6035	8.50782	-0.0161353	-0.0218333	0.9596	0.99995	no

ENSG00000153107	ENSG00000153107	ANAPC1	2:112523847-112642267	q1	q2	OK	10.6966	11.3084	0.0802463	0.0903886	0.8387	0.99995	no

ENSG00000153113	ENSG00000153113	CAST	5:95220801-96143803	q1	q2	OK	20.4112	20.8551	0.0310394	0.0398479	0.9514	0.99995	no

ENSG00000153130	ENSG00000153130	SCOC	4:141178439-141306880	q1	q2	OK	52.7612	55.9774	0.0853695	0.200657	0.77135	0.99995	no

ENSG00000153132	ENSG00000153132	CLGN	4:141309608-141349122	q1	q2	OK	11.2573	12.1682	0.112252	0.232657	0.74	0.99995	no

ENSG00000153140	ENSG00000153140	CETN3	5:89688077-89705603	q1	q2	OK	27.94	34.9128	0.321424	0.549515	0.4343	0.99995	no

ENSG00000153147	ENSG00000153147	SMARCA5	4:144434615-144478639	q1	q2	OK	60.9568	67.5329	0.147803	0.14492	0.65015	0.99995	no

ENSG00000153157	ENSG00000153157	SYCP2L	6:10747991-10979553	q1	q2	OK	1.33813	1.74157	0.380165	0.145452	0.76455	0.99995	no

ENSG00000153162	ENSG00000153162	BMP6	6:7727029-8102811	q1	q2	NOTEST	0.411248	0.391981	-0.0692233	0	1	1	no

ENSG00000153165	ENSG00000153165	RGPD3	2:107021445-107084832	q1	q2	NOTEST	0.122976	0.171967	0.483757	0	1	1	no

ENSG00000153179	ENSG00000153179	RASSF3	12:65004292-65091347	q1	q2	OK	2.83658	3.48566	0.297277	0.406911	0.48655	0.99995	no

ENSG00000153187	ENSG00000153187	HNRNPU	1:244998623-245027844	q1	q2	OK	297.774	322.987	0.117257	0.190132	0.7531	0.99995	no

ENSG00000153201	ENSG00000153201	RANBP2	2:109335936-109402267	q1	q2	OK	24.4062	26.6448	0.126606	0.201146	0.658	0.99995	no

ENSG00000153207	ENSG00000153207	AHCTF1	1:247002399-247095280	q1	q2	OK	20.7403	25.0948	0.274951	0.298842	0.4732	0.99995	no

ENSG00000153208	ENSG00000153208	MERTK	2:112656055-112787138	q1	q2	OK	4.24368	4.11437	-0.0446474	-0.0631748	0.91895	0.99995	no

ENSG00000153214	ENSG00000153214	TMEM87B	2:112812799-112876895	q1	q2	OK	4.00757	4.63466	0.209735	0.331176	0.55215	0.99995	no

ENSG00000153230	ENSG00000153230	OR14K1	1:247670359-247910364	q1	q2	NOTEST	0	0	0	0	1	1	no

ENSG00000153233	ENSG00000153233	PTPRR	12:71031852-71314623	q1	q2	NOTEST	0.0825023	0.0899277	0.124331	0	1	1	no

ENSG00000153234	ENSG00000153234	NR4A2	2:157180943-157198860	q1	q2	NOTEST	0.386683	0.324567	-0.252635	0	1	1	no

ENSG00000153237	ENSG00000153237	CCDC148	2:159023161-159313265	q1	q2	OK	0.917592	1.03288	0.170744	0.159866	0.79855	0.99995	no

ENSG00000153246	ENSG00000153246	PLA2R1	2:160788518-160919121	q1	q2	OK	0.749259	1.0535	0.491658	0.322128	0.49405	0.99995	no

ENSG00000153250	ENSG00000153250	RBMS1	2:161128661-161350305	q1	q2	OK	25.2967	26.4134	0.062319	0.137812	0.8323	0.99995	no

ENSG00000153253	ENSG00000153253	SCN3A	2:165848493-166060577	q1	q2	OK	9.35886	10.5956	0.17906	0.19754	0.68275	0.99995	no

ENSG00000153266	ENSG00000153266	FEZF2	3:62355355-62359999	q1	q2	NOTEST	0.0145644	0.0143508	-0.0213243	0	1	1	no

ENSG00000153283	ENSG00000153283	CD96	3:111011565-111384597	q1	q2	NOTEST	0.112912	0.0180547	-2.64475	0	1	1	no

ENSG00000153291	ENSG00000153291	SLC25A27	6:46620677-46703430	q1	q2	OK	4.99196	4.74043	-0.0745858	-0.0695775	0.91785	0.99995	no

ENSG00000153292	ENSG00000153292	GPR110	6:46965439-47010099	q1	q2	NOTEST	0.0959157	0.0631747	-0.602419	0	1	1	no

ENSG00000153294	ENSG00000153294	GPR115	6:47624171-47689757	q1	q2	NOTEST	0.0582298	0.170599	1.55078	0	1	1	no

ENSG00000153303	ENSG00000153303	FRMD1	6:168456424-168482237	q1	q2	NOTEST	0	0	0	0	1	1	no

ENSG00000153310	ENSG00000153310	FAM49B	8:130844546-131029375	q1	q2	OK	62.405	69.5898	0.157214	0.323492	0.58585	0.99995	no

ENSG00000153317	ENSG00000153317	ASAP1	8:131064352-131455906	q1	q2	OK	18.4202	21.7394	0.239023	0.406918	0.4109	0.99995	no

ENSG00000153339	ENSG00000153339	TRAPPC8	18:29409135-29533099	q1	q2	OK	15.1452	14.3331	-0.0795074	-0.117314	0.82985	0.99995	no

ENSG00000153347	ENSG00000153347	FAM81B	5:94727047-94786158	q1	q2	NOTEST	0.325778	0.311935	-0.0626422	0	1	1	no

ENSG00000153363	ENSG00000153363	LINC00467	1:211556144-211605878	q1	q2	OK	4.44931	4.69058	0.0761838	0.0847868	0.8983	0.99995	no

ENSG00000153391	ENSG00000153391	INO80C	18:33023832-33077955	q1	q2	OK	13.1413	14.0879	0.100341	0.13913	0.8207	0.99995	no

ENSG00000153395	ENSG00000153395	LPCAT1	5:1456594-1524092	q1	q2	OK	39.1664	40.2883	0.0407466	0.0926379	0.88635	0.99995	no

ENSG00000153404	ENSG00000153404	PLEKHG4B	5:140372-190085	q1	q2	OK	5.09673	4.67851	-0.123522	-0.135687	0.84625	0.99995	no

ENSG00000153406	ENSG00000153406	NMRAL1	16:4511680-4560348	q1	q2	OK	5.97065	5.90079	-0.0169797	-0.0143167	0.979	0.99995	no

ENSG00000153443	ENSG00000153443	UBALD1	16:4658883-4665028	q1	q2	OK	10.0043	7.72824	-0.372403	-0.563719	0.3772	0.99995	no

ENSG00000153446	ENSG00000153446	C16orf89	16:4932507-5147809	q1	q2	NOTEST	0.187217	0.0726845	-1.36499	0	1	1	no

ENSG00000153485	ENSG00000153485	TMEM251	14:93651295-93695561	q1	q2	OK	23.6146	24.4073	0.0476314	0.0453389	0.9473	0.99995	no

ENSG00000153487	ENSG00000153487	ING1	13:111293758-111373421	q1	q2	OK	18.8818	21.1796	0.165678	0.229433	0.738	0.99995	no

ENSG00000153495	ENSG00000153495	TEX29	13:111968530-111996596	q1	q2	NOTEST	0.297435	0.188046	-0.661489	0	1	1	no

ENSG00000153498	ENSG00000153498	SPACA7	13:113030632-113089003	q1	q2	NOTEST	0	0	0	0	1	1	no

ENSG00000153531	ENSG00000153531	ADPRHL1	13:114076259-114107839	q1	q2	OK	2.05236	1.47344	-0.478101	-0.540788	0.4365	0.99995	no

ENSG00000153551	ENSG00000153551	CMTM7	3:32433162-32544900	q1	q2	OK	22.7365	22.4599	-0.0176594	-0.0141508	0.9884	0.99995	no

ENSG00000153558	ENSG00000153558	FBXL2	3:33318516-33482863	q1	q2	OK	4.74677	4.33221	-0.131842	-0.074123	0.8698	0.99995	no

ENSG00000153560	ENSG00000153560	UBP1	3:33318516-33482863	q1	q2	OK	39.8309	39.7061	-0.00452747	-0.00807158	0.98825	0.99995	no

ENSG00000153561	ENSG00000153561	RMND5A	2:86730553-87005164	q1	q2	OK	16.9446	16.6844	-0.0223209	-0.0351876	0.9571	0.99995	no

ENSG00000153563	ENSG00000153563	CD8A	2:87011728-87035519	q1	q2	NOTEST	0.0986163	0.0310785	-1.66591	0	1	1	no

ENSG00000153574	ENSG00000153574	RPIA	2:88991161-89050452	q1	q2	OK	14.3745	15.3925	0.0987227	0.195068	0.78315	0.99995	no

ENSG00000153575	ENSG00000153575	TUBGCP5	15:22833394-22873892	q1	q2	OK	9.36925	10.4495	0.157424	0.240037	0.63865	0.99995	no

ENSG00000153666	ENSG00000153666	GOLGA8I	15:23255241-23268823	q1	q2	NOTEST	0.00493821	0.00906529	0.876366	0	1	1	no

ENSG00000153684	ENSG00000153684	GOLGA8F	15:28623766-28637170	q1	q2	NOTEST	0	0	0	0	1	1	no

ENSG00000153707	ENSG00000153707	PTPRD	9:8314245-10612723	q1	q2	OK	37.6259	38.2451	0.0235482	0.0225144	0.9485	0.99995	no

ENSG00000153714	ENSG00000153714	LURAP1L	9:12685438-12822130	q1	q2	OK	0.872881	0.578133	-0.594383	-0.56675	0.4036	0.99995	no

ENSG00000153721	ENSG00000153721	CNKSR3	6:154708637-154831793	q1	q2	OK	6.8554	8.71712	0.346611	0.532304	0.4377	0.99995	no

ENSG00000153767	ENSG00000153767	GTF2E1	3:120405527-120501916	q1	q2	OK	11.1681	12.0647	0.111412	0.147863	0.78745	0.99995	no

ENSG00000153774	ENSG00000153774	CFDP1	16:75327595-75499395	q1	q2	OK	91.0926	97.459	0.0974603	0.179477	0.78785	0.99995	no

ENSG00000153779	ENSG00000153779	TGIF2LX	X:89176880-89177882	q1	q2	NOTEST	0	0	0	0	1	1	no

ENSG00000153786	ENSG00000153786	ZDHHC7	16:85007786-85045141	q1	q2	OK	20.8857	20.9336	0.00329984	0.00545825	0.99185	0.99995	no

ENSG00000153789	ENSG00000153789	FAM92B	16:85131964-85146114	q1	q2	NOTEST	0.200355	0.056206	-1.83376	0	1	1	no

ENSG00000153790	ENSG00000153790	C7orf31	7:25174315-25219975	q1	q2	OK	3.48908	3.41514	-0.0309018	-0.0373922	0.95035	0.99995	no

ENSG00000153802	ENSG00000153802	TMPRSS11D	4:68566997-68995598	q1	q2	NOTEST	0.0105558	0.0438964	2.05606	0	1	1	no

ENSG00000153814	ENSG00000153814	JAZF1	7:27773013-28283536	q1	q2	OK	7.16497	7.26721	0.020441	0.0143324	0.9842	0.99995	no

ENSG00000153815	ENSG00000153815	CMIP	16:81478774-81745367	q1	q2	OK	16.0099	16.9708	0.0840963	0.179182	0.77255	0.99995	no

ENSG00000153820	ENSG00000153820	SPHKAP	2:228844665-229046361	q1	q2	NOTEST	0.00718403	0	#NAME?	0	1	1	no

ENSG00000153822	ENSG00000153822	KCNJ16	17:68013464-68131749	q1	q2	OK	0.546246	1.59241	1.54359	1.3604	0.0071	0.99995	no

ENSG00000153823	ENSG00000153823	PID1	2:229715241-230136001	q1	q2	OK	3.59728	3.69935	0.0403654	0.0605164	0.9263	0.99995	no

ENSG00000153827	ENSG00000153827	TRIP12	2:230628553-230877825	q1	q2	OK	61.7721	65.718	0.0893342	0.11024	0.8082	0.99995	no

ENSG00000153832	ENSG00000153832	FBXO36	2:230628553-230877825	q1	q2	OK	1.98072	2.15769	0.123464	0.0369507	0.9452	0.99995	no

ENSG00000153879	ENSG00000153879	CEBPG	19:33864235-33873592	q1	q2	OK	29.0808	31.2613	0.10431	0.255835	0.7102	0.99995	no

ENSG00000153885	ENSG00000153885	KCTD15	19:34286837-34306668	q1	q2	OK	27.4647	27.5153	0.00265781	0.00449797	0.9933	0.99995	no

ENSG00000153896	ENSG00000153896	ZNF599	19:35248980-35264134	q1	q2	OK	5.09162	4.28614	-0.248448	-0.301441	0.67035	0.99995	no

ENSG00000153898	ENSG00000153898	MCOLN2	1:85391267-85462796	q1	q2	NOTEST	0.18951	0.0208852	-3.18172	0	1	1	no

ENSG00000153902	ENSG00000153902	LGI4	19:35615416-35645204	q1	q2	OK	0.824891	0.783742	-0.0738255	-0.0506148	0.9334	0.99995	no

ENSG00000153904	ENSG00000153904	DDAH1	1:85731930-86043933	q1	q2	OK	36.8551	34.603	-0.090966	-0.20714	0.7628	0.99995	no

ENSG00000153914	ENSG00000153914	SREK1	5:65435798-65479443	q1	q2	OK	63.9303	68.3019	0.095425	0.149621	0.7646	0.99995	no

ENSG00000153922	ENSG00000153922	CHD1	5:98190907-98262240	q1	q2	OK	44.6969	50.1852	0.167086	0.260743	0.6376	0.99995	no

ENSG00000153923	ENSG00000153923	CLCA3P	1:87012760-87158886	q1	q2	NOTEST	0	0	0	0	1	1	no

ENSG00000153930	ENSG00000153930	ANKFN1	17:54188318-54589020	q1	q2	OK	0.450649	0.585611	0.377938	0.235895	0.5755	0.99995	no

ENSG00000153933	ENSG00000153933	DGKE	17:54869273-54946036	q1	q2	OK	2.15769	2.33759	0.115532	0.112701	0.81035	0.99995	no

ENSG00000153936	ENSG00000153936	HS2ST1	1:87380330-87634884	q1	q2	OK	32.9867	34.3325	0.0576908	0.110568	0.84995	0.99995	no

ENSG00000153944	ENSG00000153944	MSI2	17:55333211-55762046	q1	q2	OK	69.2961	70.5118	0.0250896	0.0356328	0.93635	0.99995	no

ENSG00000153956	ENSG00000153956	CACNA2D1	7:81575759-82073114	q1	q2	OK	3.2108	3.29789	0.0386123	0.0457321	0.9176	0.99995	no

ENSG00000153975	ENSG00000153975	ZUFSP	6:116956780-116989957	q1	q2	OK	12.3299	13.3616	0.115935	0.193172	0.7848	0.99995	no

ENSG00000153976	ENSG00000153976	HS3ST3A1	17:13399005-13505244	q1	q2	OK	2.29368	1.6464	-0.478356	-0.538649	0.4123	0.99995	no

ENSG00000153982	ENSG00000153982	GDPD1	17:57297827-57353328	q1	q2	OK	6.62415	7.13958	0.108105	0.160243	0.81185	0.99995	no

ENSG00000153989	ENSG00000153989	NUS1	6:117996664-118031803	q1	q2	OK	16.6013	17.263	0.0563814	0.133991	0.8489	0.99995	no

ENSG00000153993	ENSG00000153993	SEMA3D	7:84624868-84816171	q1	q2	OK	1.55574	2.62063	0.752309	1.03203	0.08795	0.99995	no

ENSG00000154001	ENSG00000154001	PPP2R5E	14:63838074-64010092	q1	q2	OK	25.6244	25.5651	-0.00334099	-0.0059952	0.9933	0.99995	no

ENSG00000154007	ENSG00000154007	ASB17	1:76384557-76398116	q1	q2	NOTEST	0	0	0	0	1	1	no

ENSG00000154016	ENSG00000154016	GRAP	17:18853657-18950950	q1	q2	OK	1.53039	1.41625	-0.111818	-0.111029	0.85505	0.99995	no

ENSG00000154025	ENSG00000154025	SLC5A10	17:18853657-18950950	q1	q2	NOTEST	0.0627417	0.0754578	0.266245	0	1	1	no

ENSG00000154027	ENSG00000154027	AK5	1:77747735-78025651	q1	q2	OK	2.70901	2.2915	-0.241473	-0.189466	0.74	0.99995	no

ENSG00000154035	ENSG00000154035	C17orf103	17:21142182-21156722	q1	q2	OK	4.22503	3.43783	-0.297465	-0.548229	0.42705	0.99995	no

ENSG00000154040	ENSG00000154040	CABYR	18:21718941-21977844	q1	q2	OK	1.60187	1.56432	-0.0342262	-0.0168125	0.9697	0.99995	no

ENSG00000154059	ENSG00000154059	IMPACT	18:22006579-22033499	q1	q2	OK	12.6963	13.4988	0.0884237	0.199344	0.7724	0.99995	no

ENSG00000154065	ENSG00000154065	ANKRD29	18:21178889-21242849	q1	q2	OK	0.944906	0.802351	-0.235938	-0.170333	0.7644	0.99995	no

ENSG00000154079	ENSG00000154079	C6orf57	6:71276619-71299272	q1	q2	OK	9.41385	9.57802	0.0249424	0.0311779	0.95345	0.99995	no

ENSG00000154080	ENSG00000154080	CHST9	18:24034873-24770662	q1	q2	OK	0.589089	0.749854	0.348124	0.160747	0.86605	0.99995	no

ENSG00000154096	ENSG00000154096	THY1	11:119252487-119397374	q1	q2	OK	150.468	174.934	0.217351	0.320329	0.646	0.99995	no

ENSG00000154099	ENSG00000154099	DNAAF1	16:84178864-84220669	q1	q2	OK	0.522622	0.411757	-0.343976	-0.0615386	0.7234	0.99995	no

ENSG00000154102	ENSG00000154102	C16orf74	16:85723689-85784735	q1	q2	OK	0.492357	0.449409	-0.131677	-0.0918034	0.86215	0.99995	no

ENSG00000154114	ENSG00000154114	TBCEL	11:120894780-120961484	q1	q2	OK	9.03167	9.61796	0.0907387	0.136793	0.78175	0.99995	no

ENSG00000154118	ENSG00000154118	JPH3	16:87634131-87799598	q1	q2	OK	3.03639	2.57475	-0.237927	-0.228516	0.73365	0.99995	no

ENSG00000154122	ENSG00000154122	ANKH	5:14704909-14871887	q1	q2	OK	4.46126	5.16256	0.210635	0.163353	0.71295	0.99995	no

ENSG00000154124	ENSG00000154124	FAM105B	5:14664772-14699820	q1	q2	OK	9.61672	8.58415	-0.16387	-0.154675	0.69105	0.99995	no

ENSG00000154127	ENSG00000154127	UBASH3B	11:122526382-122685181	q1	q2	OK	5.12941	4.93209	-0.0565932	-0.089281	0.8753	0.99995	no

ENSG00000154133	ENSG00000154133	ROBO4	11:124753586-124768396	q1	q2	NOTEST	0.0962308	0.271047	1.49397	0	1	1	no

ENSG00000154134	ENSG00000154134	ROBO3	11:124735281-124751366	q1	q2	OK	10.5975	7.97382	-0.410385	-0.504119	0.42755	0.99995	no

ENSG00000154143	ENSG00000154143	PANX3	11:124481385-124490252	q1	q2	NOTEST	0	0	0	0	1	1	no

ENSG00000154144	ENSG00000154144	TBRG1	11:124492731-124567414	q1	q2	OK	14.3651	14.34	-0.00252029	-0.00402832	0.9951	0.99995	no

ENSG00000154146	ENSG00000154146	NRGN	11:124609741-124617106	q1	q2	OK	56.9515	49.2511	-0.209578	-0.472879	0.49835	0.99995	no

ENSG00000154153	ENSG00000154153	FAM134B	5:16473146-16630078	q1	q2	OK	1.09311	1.30281	0.253192	0.222307	0.675	0.99995	no

ENSG00000154162	ENSG00000154162	CDH12	5:21616370-22853731	q1	q2	NOTEST	0.298104	0.460281	0.626702	0	1	1	no

ENSG00000154165	ENSG00000154165	GPR15	3:98216447-98312567	q1	q2	NOTEST	0.0712548	0.0242001	-1.55798	0	1	1	no

ENSG00000154174	ENSG00000154174	TOMM70A	3:100082274-100175163	q1	q2	OK	23.6916	26.4333	0.157983	0.306802	0.5928	0.99995	no

ENSG00000154175	ENSG00000154175	ABI3BP	3:100467999-100712359	q1	q2	OK	0.72122	0.894732	0.311016	0.140753	0.56385	0.99995	no

ENSG00000154188	ENSG00000154188	ANGPT1	8:108261720-108510283	q1	q2	OK	1.67107	2.05583	0.298947	0.411646	0.51455	0.99995	no

ENSG00000154198	ENSG00000154198	CYP4Z2P	1:47308766-47366109	q1	q2	NOTEST	0	0	0	0	1	1	no

ENSG00000154217	ENSG00000154217	PITPNC1	17:65373574-65693372	q1	q2	OK	10.1375	10.0498	-0.0125421	-0.017575	0.98005	0.99995	no

ENSG00000154222	ENSG00000154222	CC2D1B	1:52608045-52831865	q1	q2	OK	9.33728	10.9116	0.224789	0.183849	0.68015	0.99995	no

ENSG00000154227	ENSG00000154227	CERS3	15:100913143-101085200	q1	q2	NOTEST	0	0	0	0	1	1	no

ENSG00000154229	ENSG00000154229	PRKCA	17:64298753-64806861	q1	q2	OK	2.0171	2.09686	0.0559503	0.0907165	0.879	0.99995	no

ENSG00000154237	ENSG00000154237	LRRK1	15:101389765-101626271	q1	q2	OK	0.865231	1.11394	0.364512	0.2032	0.6698	0.99995	no

ENSG00000154240	ENSG00000154240	CEP112	17:63524680-64188202	q1	q2	OK	10.7959	10.2182	-0.0793433	-0.0693783	0.8982	0.99995	no

ENSG00000154252	ENSG00000154252	GAL3ST2	2:242716239-242743623	q1	q2	NOTEST	0.167859	0.0605218	-1.47172	0	1	1	no

ENSG00000154258	ENSG00000154258	ABCA9	17:66970628-67057205	q1	q2	OK	1.19551	1.64851	0.463534	0.505237	0.32795	0.99995	no

ENSG00000154262	ENSG00000154262	ABCA6	17:67074842-67138029	q1	q2	OK	0.90165	0.89924	-0.00386134	-0.00270671	0.99705	0.99995	no

ENSG00000154263	ENSG00000154263	ABCA10	17:67143354-67323385	q1	q2	OK	1.4076	0.796653	-0.821213	-0.22362	0.34465	0.99995	no

ENSG00000154265	ENSG00000154265	ABCA5	17:67143354-67323385	q1	q2	OK	23.5644	22.7011	-0.0538487	-0.0814783	0.90425	0.99995	no

ENSG00000154269	ENSG00000154269	ENPP3	6:131949581-132068553	q1	q2	NOTEST	0.111473	0.0776419	-0.521783	0	1	1	no

ENSG00000154274	ENSG00000154274	C4orf19	4:37455562-37687998	q1	q2	OK	1.17546	1.10076	-0.0947229	-0.0683131	0.92035	0.99995	no

ENSG00000154277	ENSG00000154277	UCHL1	4:41222090-41270472	q1	q2	OK	271.382	238.642	-0.185479	-0.431857	0.534	0.99995	no

ENSG00000154305	ENSG00000154305	MIA3	1:222791427-222841354	q1	q2	OK	26.4281	26.6227	0.010587	0.00903872	0.97835	0.99995	no

ENSG00000154309	ENSG00000154309	DISP1	1:222988363-223179337	q1	q2	OK	3.71472	4.04032	0.121217	0.183688	0.75335	0.99995	no

ENSG00000154310	ENSG00000154310	TNIK	3:170779127-171178197	q1	q2	OK	12.2124	13.2741	0.120266	0.126608	0.74385	0.99995	no

ENSG00000154316	ENSG00000154316	TDH	8:11197145-11332224	q1	q2	NOTEST	0.335731	0.306449	-0.131659	0	1	1	no

ENSG00000154319	ENSG00000154319	FAM167A	8:11197145-11332224	q1	q2	OK	4.1734	4.29728	0.0422031	0.0746721	0.9106	0.99995	no

ENSG00000154328	ENSG00000154328	NEIL2	8:11627147-11644855	q1	q2	OK	8.67791	8.49847	-0.030143	-0.0515166	0.9403	0.99995	no

ENSG00000154330	ENSG00000154330	PGM5	9:70921443-71145977	q1	q2	NOTEST	0.266175	0.297783	0.161886	0	1	1	no

ENSG00000154342	ENSG00000154342	WNT3A	1:228194751-228248961	q1	q2	NOTEST	0	0	0	0	1	1	no

ENSG00000154358	ENSG00000154358	OBSCN	1:228391206-228566577	q1	q2	OK	9.35421	8.93312	-0.0664506	-0.0674426	0.9053	0.99995	no

ENSG00000154359	ENSG00000154359	LONRF1	8:12579402-12613582	q1	q2	OK	22.8201	24.7183	0.115273	0.210506	0.7353	0.99995	no

ENSG00000154370	ENSG00000154370	TRIM11	1:228581373-228594541	q1	q2	OK	13.4189	14.1509	0.0766268	0.126082	0.83825	0.99995	no

ENSG00000154380	ENSG00000154380	ENAH	1:225674536-225840844	q1	q2	OK	91.3793	98.0502	0.101653	0.172981	0.78455	0.99995	no

ENSG00000154415	ENSG00000154415	PPP1R3A	7:113516831-113715975	q1	q2	NOTEST	0.00597734	0.040943	2.77604	0	1	1	no

ENSG00000154429	ENSG00000154429	CCSAP	1:229455149-229479041	q1	q2	OK	6.12113	6.9184	0.176641	0.368344	0.5962	0.99995	no

ENSG00000154438	ENSG00000154438	ASZ1	7:117003275-117068177	q1	q2	NOTEST	0	0	0	0	1	1	no

ENSG00000154447	ENSG00000154447	SH3RF1	4:170015406-170192256	q1	q2	OK	10.3161	11.5994	0.16915	0.332999	0.57355	0.99995	no

ENSG00000154451	ENSG00000154451	GBP5	1:89724632-89738544	q1	q2	NOTEST	0	0	0	0	1	1	no

ENSG00000154473	ENSG00000154473	BUB3	10:124913792-124924886	q1	q2	OK	105.589	112.001	0.0850429	0.187372	0.77715	0.99995	no

ENSG00000154478	ENSG00000154478	GPR26	10:125425870-125454123	q1	q2	NOTEST	0.00324426	0.00651313	1.00546	0	1	1	no

ENSG00000154479	ENSG00000154479	CCDC173	2:170501934-170550943	q1	q2	OK	1.20174	1.66887	0.473744	0.40458	0.44875	0.99995	no

ENSG00000154485	ENSG00000154485	MMP21	10:127455021-127464390	q1	q2	NOTEST	0.0559723	0.0545395	-0.0374118	0	1	1	no

ENSG00000154493	ENSG00000154493	C10orf90	10:128113565-128359079	q1	q2	NOTEST	0.0179451	0	#NAME?	0	1	1	no

ENSG00000154511	ENSG00000154511	FAM69A	1:93307723-93427057	q1	q2	OK	5.29009	5.15377	-0.0376628	-0.0632256	0.92995	0.99995	no

ENSG00000154518	ENSG00000154518	ATP5G3	2:176040985-176049335	q1	q2	OK	186.464	193	0.0497056	0.112854	0.87585	0.99995	no

ENSG00000154529	ENSG00000154529	CNTNAP3B	9:43684901-43924049	q1	q2	OK	0.903701	1.2915	0.515134	0.408734	0.4563	0.99995	no

ENSG00000154537	ENSG00000154537	FAM27C	9:44990313-44991483	q1	q2	NOTEST	0	0	0	0	1	1	no

ENSG00000154545	ENSG00000154545	MAGED4	X:51927918-51935364	q1	q2	NOTEST	0	0	0	0	1	1	no

ENSG00000154548	ENSG00000154548	SRSF12	6:89805677-89827800	q1	q2	OK	13.5643	14.3835	0.0845985	0.124563	0.8265	0.99995	no

ENSG00000154553	ENSG00000154553	PDLIM3	4:186422850-186456766	q1	q2	OK	4.52872	3.23805	-0.483981	-0.615817	0.32305	0.99995	no

ENSG00000154556	ENSG00000154556	SORBS2	4:186506597-186877806	q1	q2	OK	61.3817	59.2667	-0.0505864	-0.0675892	0.89575	0.99995	no

ENSG00000154582	ENSG00000154582	TCEB1	8:74851403-74884522	q1	q2	OK	78.3877	86.5056	0.142166	0.297147	0.67715	0.99995	no

ENSG00000154589	ENSG00000154589	LY96	8:74903586-74941322	q1	q2	NOTEST	0	0.102481	inf	0	1	1	no

ENSG00000154608	ENSG00000154608	CEP170P1	4:119388744-119476483	q1	q2	OK	0.658011	0.823292	0.323294	0.384797	0.58245	0.99995	no

ENSG00000154611	ENSG00000154611	PSMA8	18:23713815-23773319	q1	q2	NOTEST	0	0	0	0	1	1	no

ENSG00000154620	ENSG00000154620	TMSB4Y	Y:15815446-15817904	q1	q2	OK	3.33446	3.17047	-0.0727529	-0.0951401	0.89045	0.99995	no

ENSG00000154639	ENSG00000154639	CXADR	21:18884699-18965897	q1	q2	OK	26.5788	30.0887	0.178949	0.328734	0.56855	0.99995	no

ENSG00000154640	ENSG00000154640	BTG3	21:18965970-18985265	q1	q2	OK	23.5701	26.0918	0.146641	0.264506	0.66515	0.99995	no

ENSG00000154642	ENSG00000154642	C21orf91	21:19135631-19191703	q1	q2	OK	10.5567	11.7786	0.158002	0.296056	0.59935	0.99995	no

ENSG00000154645	ENSG00000154645	CHODL	21:19273579-19639690	q1	q2	OK	2.22888	1.80284	-0.30605	-0.403794	0.5699	0.99995	no

ENSG00000154646	ENSG00000154646	TMPRSS15	21:19641432-19858917	q1	q2	NOTEST	0.0256732	0.018847	-0.445924	0	1	1	no

ENSG00000154654	ENSG00000154654	NCAM2	21:22370632-22915650	q1	q2	OK	2.33208	2.58307	0.14747	0.104689	0.8304	0.99995	no

ENSG00000154655	ENSG00000154655	L3MBTL4	18:5954704-6415236	q1	q2	OK	0.600673	0.57024	-0.0750115	-0.0624559	0.8995	0.99995	no

ENSG00000154678	ENSG00000154678	PDE1C	7:31790792-32338941	q1	q2	OK	0.428636	0.49166	0.19791	0.123047	0.76145	0.99995	no

ENSG00000154710	ENSG00000154710	RABGEF1	7:66093867-66309813	q1	q2	OK	13.3085	11.7269	-0.182529	-0.237257	0.6998	0.99995	no

ENSG00000154719	ENSG00000154719	MRPL39	21:26957967-26979829	q1	q2	OK	23.536	25.1594	0.0962293	0.186672	0.78905	0.99995	no

ENSG00000154721	ENSG00000154721	JAM2	21:27011583-27144771	q1	q2	OK	44.026	39.0102	-0.174503	-0.206404	0.7216	0.99995	no

ENSG00000154723	ENSG00000154723	ATP5J	21:27011583-27144771	q1	q2	OK	123.447	130.916	0.0847515	0.0802416	0.91255	0.99995	no

ENSG00000154727	ENSG00000154727	GABPA	21:27011583-27144771	q1	q2	OK	15.2388	16.0759	0.0771539	0.0959155	0.89225	0.99995	no

ENSG00000154734	ENSG00000154734	ADAMTS1	21:28208065-28217728	q1	q2	OK	2.29395	2.67222	0.220209	0.299852	0.6002	0.99995	no

ENSG00000154736	ENSG00000154736	ADAMTS5	21:28261694-28338832	q1	q2	NOTEST	0.43494	0.398823	-0.125067	0	1	1	no

ENSG00000154743	ENSG00000154743	TSEN2	3:12525930-12705725	q1	q2	OK	12.298	12.8044	0.0582063	0.0301669	0.94395	0.99995	no

ENSG00000154760	ENSG00000154760	SLFN13	17:33762114-33775856	q1	q2	OK	1.11663	1.42718	0.354023	0.21309	0.63065	0.99995	no

ENSG00000154764	ENSG00000154764	WNT7A	3:13857754-13921618	q1	q2	OK	7.04425	6.01652	-0.227518	-0.43645	0.50905	0.99995	no

ENSG00000154767	ENSG00000154767	XPC	3:14166439-14242619	q1	q2	OK	26.0171	23.9202	-0.121232	-0.142474	0.7986	0.99995	no

ENSG00000154768	ENSG00000154768	C17orf50	17:34083267-34122711	q1	q2	NOTEST	0	0	0	0	1	1	no

ENSG00000154781	ENSG00000154781	CCDC174	3:14693270-14714166	q1	q2	OK	10.9733	12.2089	0.153927	0.207177	0.7087	0.99995	no

ENSG00000154783	ENSG00000154783	FGD5	3:14860468-15106842	q1	q2	NOTEST	0.401682	0.327244	-0.295687	0	1	1	no

ENSG00000154803	ENSG00000154803	FLCN	17:16945858-17140502	q1	q2	OK	22.0303	17.3656	-0.343253	-0.185121	0.5953	0.99995	no

ENSG00000154813	ENSG00000154813	DPH3	3:16299484-16306479	q1	q2	OK	21.7549	21.236	-0.0348235	-0.0802669	0.9047	0.99995	no

ENSG00000154814	ENSG00000154814	OXNAD1	3:16306705-16555533	q1	q2	OK	6.11921	7.05205	0.204698	0.214142	0.75775	0.99995	no

ENSG00000154822	ENSG00000154822	PLCL2	3:16844158-17132086	q1	q2	OK	5.58956	6.04124	0.112112	0.213513	0.7579	0.99995	no

ENSG00000154832	ENSG00000154832	CXXC1	18:47808712-47814674	q1	q2	OK	40.1524	33.1862	-0.274904	-0.46545	0.5027	0.99995	no

ENSG00000154839	ENSG00000154839	SKA1	18:47901364-47920543	q1	q2	NOTEST	0	0.00912243	inf	0	1	1	no

ENSG00000154845	ENSG00000154845	PPP4R1	18:9546788-9615238	q1	q2	OK	71.4117	73.7929	0.0473207	0.0679225	0.8612	0.99995	no

ENSG00000154856	ENSG00000154856	APCDD1	18:10454624-10489945	q1	q2	OK	15.5175	15.6465	0.0119417	0.0189606	0.97395	0.99995	no

ENSG00000154864	ENSG00000154864	PIEZO2	18:10661929-11148587	q1	q2	OK	9.91217	13.2776	0.421723	0.612777	0.25475	0.99995	no

ENSG00000154874	ENSG00000154874	CCDC144B	17:18414534-18528930	q1	q2	NOTEST	0.176479	0.219844	0.31698	0	1	1	no

ENSG00000154889	ENSG00000154889	MPPE1	18:11688954-11909222	q1	q2	OK	12.4711	12.3499	-0.0140894	-0.00842994	0.98605	0.99995	no

ENSG00000154898	ENSG00000154898	CCDC144CP	17:19912656-20306870	q1	q2	OK	1.25123	1.18345	-0.0803432	-0.0373905	0.96005	0.99995	no

ENSG00000154914	ENSG00000154914	USP43	17:9479943-9633008	q1	q2	OK	0.895659	0.922888	0.043206	0.0465147	0.94565	0.99995	no

ENSG00000154917	ENSG00000154917	RAB6B	3:133502876-133614680	q1	q2	OK	6.42421	6.44173	0.0039285	0.00383887	0.9935	0.99995	no

ENSG00000154920	ENSG00000154920	EME1	17:48450580-48475249	q1	q2	OK	20.2762	18.305	-0.147551	-0.114772	0.83045	0.99995	no

ENSG00000154928	ENSG00000154928	EPHB1	3:134316642-134979309	q1	q2	OK	10.3991	8.64293	-0.266869	-0.497651	0.3753	0.99995	no

ENSG00000154930	ENSG00000154930	ACSS1	20:24986867-25039616	q1	q2	OK	9.25758	8.35812	-0.147456	-0.304097	0.66285	0.99995	no

ENSG00000154945	ENSG00000154945	ANKRD40	17:48770550-48785285	q1	q2	OK	23.3927	24.192	0.0484671	0.105771	0.86915	0.99995	no

ENSG00000154957	ENSG00000154957	ZNF18	17:11501747-11900827	q1	q2	OK	4.94794	4.53734	-0.124982	-0.176226	0.7962	0.99995	no

ENSG00000154975	ENSG00000154975	CA10	17:49707673-50237377	q1	q2	OK	0.731551	0.0949195	-2.94618	-0.968991	0.0025	0.99995	no

ENSG00000154978	ENSG00000154978	VOPP1	7:55503748-55640681	q1	q2	OK	28.8375	29.4653	0.0310728	0.0706913	0.91635	0.99995	no

ENSG00000154997	ENSG00000154997	Sep-14	7:55861236-55930482	q1	q2	NOTEST	0.0132379	0.00640723	-1.0469	0	1	1	no

ENSG00000155008	ENSG00000155008	APOOL	X:84258831-84343069	q1	q2	OK	4.37124	5.65645	0.371855	0.498922	0.46725	0.99995	no

ENSG00000155011	ENSG00000155011	DKK2	4:107842958-108223027	q1	q2	OK	2.7995	3.00565	0.102512	0.153663	0.8139	0.99995	no

ENSG00000155016	ENSG00000155016	CYP2U1	4:108745718-108899955	q1	q2	OK	3.78878	3.63331	-0.0604486	-0.105206	0.87845	0.99995	no

ENSG00000155026	ENSG00000155026	RSPH10B	7:5938355-6010314	q1	q2	NOTEST	0.143458	0.118715	-0.273118	0	1	1	no

ENSG00000155034	ENSG00000155034	FBXL18	7:5470965-5553429	q1	q2	OK	3.76842	3.24292	-0.216667	-0.279243	0.7027	0.99995	no

ENSG00000155052	ENSG00000155052	CNTNAP5	2:124782863-125672864	q1	q2	NOTEST	0.287372	0.404618	0.49364	0	1	1	no

ENSG00000155066	ENSG00000155066	PROM2	2:95940200-95957056	q1	q2	OK	1.6002	1.19156	-0.425399	-0.270182	0.50145	0.99995	no

ENSG00000155070	ENSG00000155070	UNC93B2	7:6895115-6899461	q1	q2	NOTEST	0	0	0	0	1	1	no

ENSG00000155085	ENSG00000155085	AK9	6:109809108-110012420	q1	q2	OK	4.95015	4.37457	-0.178331	-0.21301	0.6906	0.99995	no

ENSG00000155087	ENSG00000155087	ODF1	8:103563799-103573245	q1	q2	NOTEST	0	0	0	0	1	1	no

ENSG00000155090	ENSG00000155090	KLF10	8:103661006-103668130	q1	q2	OK	6.04814	6.66582	0.140291	0.250903	0.727	0.99995	no

ENSG00000155093	ENSG00000155093	PTPRN2	7:157331749-158380480	q1	q2	OK	21.7894	21.5761	-0.0141911	-0.0332356	0.96305	0.99995	no

ENSG00000155096	ENSG00000155096	AZIN1	8:103838584-103990104	q1	q2	OK	64.6702	77.215	0.255781	0.539087	0.37315	0.99995	no

ENSG00000155097	ENSG00000155097	ATP6V1C1	8:104032414-104085279	q1	q2	OK	16.3778	16.6795	0.0263414	0.0523376	0.9373	0.99995	no

ENSG00000155099	ENSG00000155099	TMEM55A	8:92006023-92053292	q1	q2	OK	8.90261	10.4983	0.237861	0.409817	0.50275	0.99995	no

ENSG00000155100	ENSG00000155100	OTUD6B	8:92082423-92099323	q1	q2	OK	8.78779	9.45869	0.106141	0.209122	0.7665	0.99995	no

ENSG00000155111	ENSG00000155111	CDK19	6:110931180-111137161	q1	q2	OK	16.249	16.0973	-0.0135305	-0.0242806	0.96535	0.99995	no

ENSG00000155115	ENSG00000155115	GTF3C6	6:111279762-111289093	q1	q2	OK	83.9099	87.8439	0.0661011	0.153723	0.82645	0.99995	no

ENSG00000155130	ENSG00000155130	MARCKS	6:114178540-114184648	q1	q2	OK	275.826	287.215	0.0583682	0.118643	0.86335	0.99995	no

ENSG00000155158	ENSG00000155158	TTC39B	9:15163619-15307358	q1	q2	OK	0.9751	1.27083	0.382155	0.418389	0.4012	0.99995	no

ENSG00000155189	ENSG00000155189	AGPAT5	8:6565877-6617184	q1	q2	OK	26.3534	30.5987	0.215481	0.457686	0.43935	0.99995	no

ENSG00000155229	ENSG00000155229	MMS19	10:99218080-99258551	q1	q2	OK	32.893	31.2191	-0.075353	-0.12748	0.8684	0.99995	no

ENSG00000155249	ENSG00000155249	OR4K1	14:20403766-20404842	q1	q2	NOTEST	0	0	0	0	1	1	no

ENSG00000155252	ENSG00000155252	PI4K2A	10:99344079-99436191	q1	q2	OK	11.35	9.88265	-0.199722	-0.381572	0.58555	0.99995	no

ENSG00000155254	ENSG00000155254	MARVELD1	10:99472929-99477907	q1	q2	OK	16.9681	16.6954	-0.0233771	-0.0478374	0.9416	0.99995	no

ENSG00000155256	ENSG00000155256	ZFYVE27	10:99496877-99520664	q1	q2	OK	11.8205	11.1423	-0.0852373	-0.122409	0.802	0.99995	no

ENSG00000155265	ENSG00000155265	GOLGA7B	10:99609995-99790585	q1	q2	OK	1.66073	1.90029	0.194404	0.192573	0.7669	0.99995	no

ENSG00000155269	ENSG00000155269	GPR78	4:8560451-8621488	q1	q2	NOTEST	0.0416207	0	#NAME?	0	1	1	no

ENSG00000155275	ENSG00000155275	TRMT44	4:8368008-8514337	q1	q2	OK	4.98471	5.59285	0.166075	0.150141	0.77395	0.99995	no

ENSG00000155282	ENSG00000155282	RP11-195B21.3	9:67926760-67987998	q1	q2	OK	1.5511	0.843364	-0.879064	-0.521001	0.4107	0.99995	no

ENSG00000155287	ENSG00000155287	SLC25A28	10:101370281-101380366	q1	q2	OK	16.3828	12.6434	-0.373802	-0.572184	0.40905	0.99995	no

ENSG00000155304	ENSG00000155304	HSPA13	21:15743435-15755805	q1	q2	OK	42.9811	45.9371	0.0959576	0.229923	0.7328	0.99995	no

ENSG00000155307	ENSG00000155307	SAMSN1	21:15857548-16031142	q1	q2	NOTEST	0.117796	0.173736	0.560606	0	1	1	no

ENSG00000155313	ENSG00000155313	USP25	21:17102343-17252377	q1	q2	OK	14.2968	15.578	0.123818	0.230308	0.69695	0.99995	no

ENSG00000155324	ENSG00000155324	GRAMD3	5:125695823-125832186	q1	q2	OK	0.607828	0.794089	0.385638	0.245731	0.4575	0.99995	no

ENSG00000155329	ENSG00000155329	ZCCHC10	5:132332676-132362296	q1	q2	OK	19.4441	23.8836	0.296692	0.41744	0.4656	0.99995	no

ENSG00000155330	ENSG00000155330	C16orf87	16:46830518-46865323	q1	q2	OK	13.916	15.3777	0.144092	0.259159	0.7066	0.99995	no

ENSG00000155363	ENSG00000155363	MOV10	1:113215762-113243368	q1	q2	OK	33.3366	27.476	-0.278936	-0.307571	0.52115	0.99995	no

ENSG00000155366	ENSG00000155366	RHOC	1:113243727-113258099	q1	q2	OK	89.1807	87.3303	-0.0302506	-0.0665306	0.92485	0.99995	no

ENSG00000155367	ENSG00000155367	PPM1J	1:113243727-113258099	q1	q2	OK	0.736599	0.701948	-0.0695153	-0.0179313	0.9636	0.99995	no

ENSG00000155368	ENSG00000155368	DBI	2:120124496-120130126	q1	q2	OK	268.923	318.972	0.246235	0.605356	0.38935	0.99995	no

ENSG00000155380	ENSG00000155380	SLC16A1	1:113454468-113542118	q1	q2	OK	44.1624	48.7718	0.143227	0.312383	0.61425	0.99995	no

ENSG00000155393	ENSG00000155393	HEATR3	16:50080339-50155854	q1	q2	OK	8.73289	9.44777	0.113514	0.16224	0.75175	0.99995	no

ENSG00000155428	ENSG00000155428	TRIM74	7:72430015-72439997	q1	q2	NOTEST	0.0230578	0.0226762	-0.0240786	0	1	1	no

ENSG00000155438	ENSG00000155438	NIFK	2:122407225-122494499	q1	q2	OK	33.3651	41.7523	0.323517	0.573469	0.3474	0.99995	no

ENSG00000155463	ENSG00000155463	OXA1L	14:23171351-23241007	q1	q2	OK	61.2475	55.8022	-0.13433	-0.309713	0.6441	0.99995	no

ENSG00000155465	ENSG00000155465	SLC7A7	14:23242430-23299029	q1	q2	OK	0.528919	0.658515	0.316168	0.172541	0.55975	0.99995	no

ENSG00000155495	ENSG00000155495	MAGEC1	X:140991679-140997174	q1	q2	NOTEST	0	0	0	0	1	1	no

ENSG00000155506	ENSG00000155506	LARP1	5:154092461-154197167	q1	q2	OK	52.2352	54.9603	0.0733685	0.115689	0.7972	0.99995	no

ENSG00000155508	ENSG00000155508	CNOT8	5:154198050-154256353	q1	q2	OK	43.2412	46.8858	0.116742	0.230051	0.67725	0.99995	no

ENSG00000155511	ENSG00000155511	GRIA1	5:152869174-153193429	q1	q2	OK	15.9657	14.7968	-0.109688	-0.194265	0.70995	0.99995	no

ENSG00000155530	ENSG00000155530	LRGUK	7:133812051-133949343	q1	q2	OK	0.818429	0.669545	-0.289676	-0.3079	0.62775	0.99995	no

ENSG00000155542	ENSG00000155542	SETD9	5:56195867-56267502	q1	q2	OK	11.3932	12.8384	0.1723	0.117359	0.8664	0.99995	no

ENSG00000155545	ENSG00000155545	MIER3	5:56195867-56267502	q1	q2	OK	15.263	17.0266	0.157755	0.298811	0.60595	0.99995	no

ENSG00000155561	ENSG00000155561	NUP205	7:135242666-135333505	q1	q2	OK	40.0469	44.5395	0.153393	0.237539	0.6988	0.99995	no

ENSG00000155592	ENSG00000155592	ZKSCAN2	16:25247321-25269252	q1	q2	OK	8.31021	8.62445	0.0535481	0.123125	0.86245	0.99995	no

ENSG00000155621	ENSG00000155621	C9orf85	9:74526425-74600970	q1	q2	OK	7.85046	8.0405	0.0345082	0.0490563	0.94175	0.99995	no

ENSG00000155622	ENSG00000155622	XAGE2B	X:52112172-52118823	q1	q2	NOTEST	0	0	0	0	1	1	no

ENSG00000155629	ENSG00000155629	PIK3AP1	10:98353068-98480271	q1	q2	OK	0.598984	0.600774	0.00430457	0.00366545	0.99455	0.99995	no

ENSG00000155636	ENSG00000155636	RBM45	2:178977150-179003738	q1	q2	OK	10.2808	11.1521	0.117363	0.176687	0.7787	0.99995	no

ENSG00000155640	ENSG00000155640	C10orf12	10:98741040-98745582	q1	q2	OK	2.83508	3.22281	0.184931	0.312629	0.6495	0.99995	no

ENSG00000155657	ENSG00000155657	TTN	2:179385909-179914813	q1	q2	NOTEST	0.0742747	0.0749073	0.0122371	0	1	1	no

ENSG00000155659	ENSG00000155659	VSIG4	X:65241579-65259967	q1	q2	NOTEST	0	0	0	0	1	1	no

ENSG00000155660	ENSG00000155660	PDIA4	7:148700153-148725733	q1	q2	OK	112.502	105.541	-0.0921449	-0.205958	0.74725	0.99995	no

ENSG00000155666	ENSG00000155666	KDM8	16:27214806-27233089	q1	q2	OK	4.19216	4.04487	-0.0516022	-0.065427	0.9028	0.99995	no

ENSG00000155714	ENSG00000155714	PDZD9	16:21995185-22012433	q1	q2	NOTEST	0.171669	0.138394	-0.31085	0	1	1	no

ENSG00000155719	ENSG00000155719	OTOA	16:21689834-21772050	q1	q2	NOTEST	0.0520805	0.0200431	-1.37764	0	1	1	no

ENSG00000155729	ENSG00000155729	KCTD18	2:201353674-201448505	q1	q2	OK	4.52728	4.93816	0.125329	0.0889333	0.90285	0.99995	no

ENSG00000155744	ENSG00000155744	FAM126B	2:201773695-201950473	q1	q2	OK	3.8497	4.43494	0.204169	0.159918	0.67665	0.99995	no

ENSG00000155749	ENSG00000155749	ALS2CR12	2:202152993-202222121	q1	q2	OK	0.354777	0.533818	0.589435	0.318995	0.50825	0.99995	no

ENSG00000155754	ENSG00000155754	ALS2CR11	2:202352147-202483901	q1	q2	NOTEST	0.050821	0.049493	-0.038198	0	1	1	no

ENSG00000155755	ENSG00000155755	TMEM237	2:202484906-202508293	q1	q2	OK	36.4257	37.5757	0.0448452	0.0830986	0.8912	0.99995	no

ENSG00000155760	ENSG00000155760	FZD7	2:202899309-202903160	q1	q2	OK	54.9196	54.0458	-0.0231386	-0.0574064	0.934	0.99995	no

ENSG00000155761	ENSG00000155761	SPAG17	1:118472342-118727846	q1	q2	OK	0.574354	0.538224	-0.0937337	-0.0172273	0.89965	0.99995	no

ENSG00000155792	ENSG00000155792	DEPTOR	8:120879658-121063152	q1	q2	OK	1.00254	0.769431	-0.381789	-0.348743	0.5466	0.99995	no

ENSG00000155816	ENSG00000155816	FMN2	1:240177647-240643504	q1	q2	OK	16.3143	17.6842	0.116321	0.146382	0.82915	0.99995	no

ENSG00000155827	ENSG00000155827	RNF20	9:104296132-104325622	q1	q2	OK	26.739	27.3196	0.0309882	0.0588338	0.9121	0.99995	no

ENSG00000155833	ENSG00000155833	CYLC2	9:105757592-105780770	q1	q2	NOTEST	0	0	0	0	1	1	no

ENSG00000155846	ENSG00000155846	PPARGC1B	5:149109860-149234585	q1	q2	OK	0.640276	0.853407	0.41454	0.477681	0.4292	0.99995	no

ENSG00000155849	ENSG00000155849	ELMO1	7:36893960-37488852	q1	q2	OK	8.93179	8.31363	-0.103471	-0.15169	0.77215	0.99995	no

ENSG00000155850	ENSG00000155850	SLC26A2	5:149340299-149432386	q1	q2	OK	7.30618	9.23092	0.337358	0.562049	0.41505	0.99995	no

ENSG00000155858	ENSG00000155858	LSM11	5:157170702-157187717	q1	q2	OK	4.21808	4.73394	0.166456	0.337859	0.6302	0.99995	no

ENSG00000155868	ENSG00000155868	MED7	5:156512842-156682201	q1	q2	OK	11.9802	12.101	0.0144722	0.0254346	0.97055	0.99995	no

ENSG00000155875	ENSG00000155875	FAM154A	9:18927655-19033251	q1	q2	NOTEST	0	0.0161434	inf	0	1	1	no

ENSG00000155876	ENSG00000155876	RRAGA	9:19049371-19051019	q1	q2	OK	47.3315	47.473	0.00430466	0.0101812	0.9897	0.99995	no

ENSG00000155886	ENSG00000155886	SLC24A2	9:19507449-19786926	q1	q2	NOTEST	0.114069	0.0939879	-0.279357	0	1	1	no

ENSG00000155890	ENSG00000155890	TRIM42	3:140396880-140419992	q1	q2	NOTEST	0	0	0	0	1	1	no

ENSG00000155893	ENSG00000155893	ACPL2	3:140947567-141168634	q1	q2	OK	21.1693	23.1856	0.131258	0.216661	0.7117	0.99995	no

ENSG00000155897	ENSG00000155897	ADCY8	8:131792546-132054672	q1	q2	NOTEST	0.0777821	0.131816	0.761017	0	1	1	no

ENSG00000155903	ENSG00000155903	RASA2	3:141205888-141334184	q1	q2	OK	8.62261	9.3638	0.118969	0.263083	0.6916	0.99995	no

ENSG00000155906	ENSG00000155906	RMND1	6:151725988-151773259	q1	q2	OK	12.7265	14.1257	0.150484	0.277685	0.67715	0.99995	no

ENSG00000155918	ENSG00000155918	RAET1L	6:150339469-150346668	q1	q2	NOTEST	0	0	0	0	1	1	no

ENSG00000155926	ENSG00000155926	SLA	8:133879202-134147147	q1	q2	NOTEST	0.00770333	0.0942738	3.6133	0	1	1	no

ENSG00000155957	ENSG00000155957	TMBIM4	12:66516841-66563852	q1	q2	OK	29.8293	32.936	0.142933	0.179935	0.7718	0.99995	no

ENSG00000155959	ENSG00000155959	VBP1	X:154425283-154468098	q1	q2	OK	9.05058	9.99525	0.143232	0.245163	0.7252	0.99995	no

ENSG00000155961	ENSG00000155961	RAB39B	X:154487525-154493874	q1	q2	OK	0.512678	0.502102	-0.0300734	-0.0322195	0.9632	0.99995	no

ENSG00000155962	ENSG00000155962	CLIC2	X:154505499-154563966	q1	q2	NOTEST	0.188897	0.40734	1.10864	0	1	1	no

ENSG00000155966	ENSG00000155966	AFF2	X:147582138-148082193	q1	q2	NOTEST	0.276327	0.218985	-0.335544	0	1	1	no

ENSG00000155970	ENSG00000155970	MICU3	8:16884746-16980153	q1	q2	OK	4.95644	5.20189	0.0697324	0.0751999	0.8584	0.99995	no

ENSG00000155974	ENSG00000155974	GRIP1	12:66741210-67197966	q1	q2	OK	7.87543	8.12232	0.0445332	0.0551339	0.90535	0.99995	no

ENSG00000155975	ENSG00000155975	VPS37A	8:17086736-17271037	q1	q2	OK	12.4631	13.4955	0.114817	0.0752315	0.87185	0.99995	no

ENSG00000155980	ENSG00000155980	KIF5A	12:57943780-57980415	q1	q2	OK	11.1014	11.2312	0.0167627	0.0246382	0.9561	0.99995	no

ENSG00000155984	ENSG00000155984	TMEM185A	X:148678215-148713568	q1	q2	OK	0.730019	0.508668	-0.521209	-0.343055	0.47285	0.99995	no

ENSG00000156006	ENSG00000156006	NAT2	8:18248754-18258728	q1	q2	NOTEST	0.0423011	0.043907	0.0537573	0	1	1	no

ENSG00000156009	ENSG00000156009	MAGEA8	X:149009940-149014609	q1	q2	NOTEST	0	0	0	0	1	1	no

ENSG00000156011	ENSG00000156011	PSD3	8:18384810-18942240	q1	q2	OK	17.4356	17.192	-0.0202992	-0.0349536	0.9413	0.99995	no

ENSG00000156017	ENSG00000156017	C9orf41	9:77567883-77643339	q1	q2	OK	3.54307	4.15256	0.229001	0.394347	0.55055	0.99995	no

ENSG00000156026	ENSG00000156026	MCU	10:74451888-74647452	q1	q2	OK	13.6274	13.9378	0.032493	0.0583141	0.92185	0.99995	no

ENSG00000156030	ENSG00000156030	ELMSAN1	14:74181824-74269973	q1	q2	OK	7.6066	8.29923	0.125725	0.167584	0.70755	0.99995	no

ENSG00000156042	ENSG00000156042	TTC18	10:74870216-75118617	q1	q2	NOTEST	0.342525	0.282653	-0.277174	0	1	1	no

ENSG00000156049	ENSG00000156049	GNA14	9:80037994-80263223	q1	q2	NOTEST	0.149539	0.302615	1.01696	0	1	1	no

ENSG00000156050	ENSG00000156050	FAM161B	14:74318546-74551196	q1	q2	OK	2.74824	2.56517	-0.0994551	-0.0519254	0.93295	0.99995	no

ENSG00000156052	ENSG00000156052	GNAQ	9:80331002-80646374	q1	q2	OK	21.4399	24.5205	0.193692	0.471797	0.4943	0.99995	no

ENSG00000156076	ENSG00000156076	WIF1	12:65444405-65515346	q1	q2	OK	2.35352	3.69522	0.650838	0.836247	0.2123	0.99995	no

ENSG00000156096	ENSG00000156096	UGT2B4	4:70345882-70391732	q1	q2	NOTEST	0.0507976	0.0569467	0.164851	0	1	1	no

ENSG00000156097	ENSG00000156097	GPR61	1:110082493-110091028	q1	q2	NOTEST	0.227125	0.18178	-0.321293	0	1	1	no

ENSG00000156103	ENSG00000156103	MMP16	8:89044236-89749363	q1	q2	OK	7.78885	9.23828	0.246215	0.566997	0.3974	0.99995	no

ENSG00000156110	ENSG00000156110	ADK	10:75910959-76469061	q1	q2	OK	26.6232	26.3887	-0.0127674	-0.024828	0.97145	0.99995	no

ENSG00000156113	ENSG00000156113	KCNMA1	10:78629358-79398353	q1	q2	OK	0.673371	0.519165	-0.375209	-0.189476	0.59495	0.99995	no

ENSG00000156127	ENSG00000156127	BATF	14:75988767-76013358	q1	q2	NOTEST	0.0602276	0	#NAME?	0	1	1	no

ENSG00000156136	ENSG00000156136	DCK	4:71768042-71896631	q1	q2	OK	21.2585	24.9137	0.228898	0.283475	0.67575	0.99995	no

ENSG00000156140	ENSG00000156140	ADAMTS3	4:73146685-73434516	q1	q2	OK	2.51295	2.35487	-0.0937364	-0.147659	0.8126	0.99995	no

ENSG00000156150	ENSG00000156150	ALX3	1:110574198-110617263	q1	q2	NOTEST	0	0	0	0	1	1	no

ENSG00000156162	ENSG00000156162	DPY19L4	8:95731930-95806064	q1	q2	OK	19.8089	20.5375	0.0521096	0.0706823	0.87935	0.99995	no

ENSG00000156170	ENSG00000156170	NDUFAF6	8:95825538-96128683	q1	q2	OK	16.6861	14.3217	-0.220447	-0.128692	0.7926	0.99995	no

ENSG00000156171	ENSG00000156171	DRAM2	1:111659954-111727724	q1	q2	OK	17.2717	17.2287	-0.0035981	-0.00426202	0.9948	0.99995	no

ENSG00000156172	ENSG00000156172	C8orf37	8:96216683-96822364	q1	q2	OK	2.66567	2.53657	-0.0716161	-0.108858	0.8771	0.99995	no

ENSG00000156194	ENSG00000156194	PPEF2	4:76781019-76823724	q1	q2	NOTEST	0	0.0250767	inf	0	1	1	no

ENSG00000156206	ENSG00000156206	C15orf26	15:81299373-81441516	q1	q2	NOTEST	0.151654	0.0161116	-3.23461	0	1	1	no

ENSG00000156218	ENSG00000156218	ADAMTSL3	15:84322837-84708594	q1	q2	NOTEST	0.120327	0.111275	-0.112833	0	1	1	no

ENSG00000156219	ENSG00000156219	ART3	4:76932336-77033955	q1	q2	OK	0.564793	0.338206	-0.739818	-0.282854	0.32765	0.99995	no

ENSG00000156222	ENSG00000156222	SLC28A1	15:85427884-85518876	q1	q2	NOTEST	0.0215729	0.0101264	-1.0911	0	1	1	no

ENSG00000156232	ENSG00000156232	WHAMM	15:83478379-83503611	q1	q2	OK	2.68254	2.64086	-0.022588	-0.0341062	0.95815	0.99995	no

ENSG00000156234	ENSG00000156234	CXCL13	4:78432906-78532988	q1	q2	NOTEST	0.295737	0.233482	-0.341001	0	1	1	no

ENSG00000156239	ENSG00000156239	N6AMT1	21:30244512-30257693	q1	q2	OK	5.83518	6.43594	0.141374	0.238928	0.72035	0.99995	no

ENSG00000156253	ENSG00000156253	RWDD2B	21:30376704-30391699	q1	q2	OK	1.94975	2.14336	0.136584	0.146053	0.8239	0.99995	no

ENSG00000156256	ENSG00000156256	USP16	21:30396575-30426809	q1	q2	OK	23.3195	24.3695	0.0635398	0.142158	0.8405	0.99995	no

ENSG00000156261	ENSG00000156261	CCT8	21:30428125-30446118	q1	q2	OK	139.434	154.975	0.152455	0.359429	0.58615	0.99995	no

ENSG00000156265	ENSG00000156265	MAP3K7CL	21:30449791-30548210	q1	q2	NOTEST	0.180851	0.101504	-0.833269	0	1	1	no

ENSG00000156269	ENSG00000156269	NAA11	4:79892901-80247204	q1	q2	NOTEST	0	0.0294682	inf	0	1	1	no

ENSG00000156273	ENSG00000156273	BACH1	21:30565800-31312351	q1	q2	OK	17.0736	18.582	0.122135	0.219587	0.6727	0.99995	no

ENSG00000156282	ENSG00000156282	CLDN17	21:31538240-31538971	q1	q2	NOTEST	0	0	0	0	1	1	no

ENSG00000156284	ENSG00000156284	CLDN8	21:31586323-31588391	q1	q2	NOTEST	0	0	0	0	1	1	no

ENSG00000156298	ENSG00000156298	TSPAN7	X:37208527-38548169	q1	q2	OK	11.9428	10.1168	-0.239388	-0.33921	0.6302	0.99995	no

ENSG00000156299	ENSG00000156299	TIAM1	21:32490733-32932800	q1	q2	OK	13.9642	15.2879	0.130654	0.238743	0.67415	0.99995	no

ENSG00000156304	ENSG00000156304	SCAF4	21:33043345-33104388	q1	q2	OK	24.2752	25.0534	0.0455219	0.0963735	0.8771	0.99995	no

ENSG00000156313	ENSG00000156313	RPGR	X:37208527-38548169	q1	q2	OK	6.95339	7.10442	0.0309996	0.0233417	0.9644	0.99995	no

ENSG00000156345	ENSG00000156345	CDK20	9:90581355-90589668	q1	q2	OK	5.15856	5.64224	0.129299	0.178379	0.8003	0.99995	no

ENSG00000156374	ENSG00000156374	PCGF6	10:105062552-105110891	q1	q2	OK	12.0359	13.1973	0.132905	0.266293	0.70905	0.99995	no

ENSG00000156381	ENSG00000156381	ANKRD9	14:102973178-102976136	q1	q2	OK	9.88668	7.97372	-0.310232	-0.508155	0.47245	0.99995	no

ENSG00000156384	ENSG00000156384	SFR1	10:105881815-105886143	q1	q2	OK	7.66535	8.35766	0.124747	0.175103	0.7896	0.99995	no

ENSG00000156395	ENSG00000156395	SORCS3	10:106400858-107024993	q1	q2	NOTEST	0.4414	0.37871	-0.220994	0	1	1	no

ENSG00000156398	ENSG00000156398	SFXN2	10:104474294-104503249	q1	q2	OK	9.15577	9.40751	0.0391324	0.0497302	0.929	0.99995	no

ENSG00000156411	ENSG00000156411	C14orf2	14:104378624-104394606	q1	q2	OK	114.234	118.303	0.0504887	0.112402	0.8644	0.99995	no

ENSG00000156413	ENSG00000156413	FUT6	19:5830620-5839742	q1	q2	NOTEST	0	0.0114437	inf	0	1	1	no

ENSG00000156414	ENSG00000156414	TDRD9	14:104394798-104519004	q1	q2	NOTEST	0.00594815	0	#NAME?	0	1	1	no

ENSG00000156427	ENSG00000156427	FGF18	5:170846659-170884627	q1	q2	OK	0.330486	0.684979	1.05147	1.01856	0.14765	0.99995	no

ENSG00000156453	ENSG00000156453	PCDH1	5:141232937-141258811	q1	q2	OK	25.3681	32.9703	0.378153	0.856184	0.2294	0.99995	no

ENSG00000156463	ENSG00000156463	SH3RF2	5:145316141-145461354	q1	q2	OK	0.410501	0.590278	0.524009	0.327869	0.5345	0.99995	no

ENSG00000156466	ENSG00000156466	GDF6	8:97154561-97173020	q1	q2	NOTEST	0.291227	0.350678	0.268003	0	1	1	no

ENSG00000156467	ENSG00000156467	UQCRB	8:97238147-97250262	q1	q2	OK	154.728	171.582	0.149166	0.294044	0.66695	0.99995	no

ENSG00000156469	ENSG00000156469	MTERFD1	8:97251625-97273838	q1	q2	OK	17.5313	19.8787	0.181292	0.341238	0.61575	0.99995	no

ENSG00000156471	ENSG00000156471	PTDSS1	8:97273942-97349223	q1	q2	OK	35.8346	34.3565	-0.060771	-0.110031	0.8774	0.99995	no

ENSG00000156475	ENSG00000156475	PPP2R2B	5:145942788-146464347	q1	q2	OK	3.07089	2.2714	-0.435075	-0.476179	0.3451	0.99995	no

ENSG00000156482	ENSG00000156482	RPL30	8:98881067-99058697	q1	q2	OK	937.726	932.255	-0.00844199	-0.0197134	0.9779	0.99995	no

ENSG00000156486	ENSG00000156486	KCNS2	8:99413630-99955055	q1	q2	NOTEST	0.0356269	0.0587968	0.722769	0	1	1	no

ENSG00000156500	ENSG00000156500	FAM122C	X:133903595-133988640	q1	q2	OK	2.60566	2.90012	0.154465	0.102199	0.85015	0.99995	no

ENSG00000156502	ENSG00000156502	SUPV3L1	10:70939987-70968855	q1	q2	OK	23.2006	19.674	-0.23787	-0.366866	0.5815	0.99995	no

ENSG00000156504	ENSG00000156504	FAM122B	X:133903595-133988640	q1	q2	OK	14.9162	15.5344	0.0585922	0.102599	0.8643	0.99995	no

ENSG00000156508	ENSG00000156508	EEF1A1	6:74225472-74280319	q1	q2	OK	3847.76	3589.2	-0.100357	-0.0966874	0.8877	0.99995	no

ENSG00000156509	ENSG00000156509	FBXO43	8:101145587-101158028	q1	q2	OK	1.27197	1.29852	0.0297983	0.0358611	0.96205	0.99995	no

ENSG00000156510	ENSG00000156510	HKDC1	10:70975088-71027904	q1	q2	NOTEST	0.138942	0.143661	0.0481859	0	1	1	no

ENSG00000156515	ENSG00000156515	HK1	10:71029739-71161638	q1	q2	OK	33.9051	34.0964	0.00811734	0.0158642	0.9787	0.99995	no

ENSG00000156521	ENSG00000156521	TYSND1	10:71897736-71906432	q1	q2	OK	3.11771	2.41671	-0.367442	-0.567499	0.4179	0.99995	no

ENSG00000156531	ENSG00000156531	PHF6	X:133507282-133562820	q1	q2	OK	44.4475	48.9405	0.138925	0.327065	0.6317	0.99995	no

ENSG00000156535	ENSG00000156535	CD109	6:74403625-74538040	q1	q2	OK	1.36972	1.40967	0.0414782	0.0605002	0.92665	0.99995	no

ENSG00000156564	ENSG00000156564	LRFN2	6:40359324-40555204	q1	q2	NOTEST	0.0871149	0.117263	0.428751	0	1	1	no

ENSG00000156574	ENSG00000156574	NODAL	10:72192070-72207707	q1	q2	NOTEST	0.325738	0.448769	0.462262	0	1	1	no

ENSG00000156575	ENSG00000156575	PRG3	11:57144241-57148623	q1	q2	NOTEST	0	0	0	0	1	1	no

ENSG00000156587	ENSG00000156587	UBE2L6	11:57319128-57335757	q1	q2	OK	55.3725	49.7407	-0.154743	-0.359992	0.6047	0.99995	no

ENSG00000156599	ENSG00000156599	ZDHHC5	11:57435218-57468659	q1	q2	OK	16.0915	16.9391	0.0740577	0.156558	0.8015	0.99995	no

ENSG00000156603	ENSG00000156603	MED19	11:57471185-57479693	q1	q2	OK	22.5289	21.3312	-0.0788145	-0.147032	0.8382	0.99995	no

ENSG00000156639	ENSG00000156639	ZFAND3	6:37787274-38122400	q1	q2	OK	91.2833	97.0416	0.0882521	0.13463	0.84235	0.99995	no

ENSG00000156642	ENSG00000156642	NPTN	15:73735498-73926475	q1	q2	OK	49.4468	55.0643	0.155241	0.291734	0.64145	0.99995	no

ENSG00000156650	ENSG00000156650	KAT6B	10:76585339-76792380	q1	q2	OK	12.72	12.3603	-0.0413877	-0.0979679	0.889	0.99995	no

ENSG00000156671	ENSG00000156671	SAMD8	10:76854191-76941881	q1	q2	OK	8.10829	9.21929	0.185258	0.408025	0.5482	0.99995	no

ENSG00000156675	ENSG00000156675	RAB11FIP1	8:37716135-37756985	q1	q2	OK	1.25477	1.21674	-0.0444047	-0.0625798	0.9228	0.99995	no

ENSG00000156687	ENSG00000156687	UNC5D	8:35092974-35654068	q1	q2	OK	1.35764	2.01569	0.570175	0.677451	0.30095	0.99995	no

ENSG00000156689	ENSG00000156689	GLYATL2	11:58601541-58671688	q1	q2	OK	1.04038	1.2885	0.308577	0.252754	0.69305	0.99995	no

ENSG00000156697	ENSG00000156697	UTP14A	X:129003039-129091504	q1	q2	OK	10.6357	11.4907	0.11155	0.218047	0.757	0.99995	no

ENSG00000156709	ENSG00000156709	AIFM1	X:129263336-129299861	q1	q2	OK	22.5907	23.8032	0.0754243	0.163212	0.81765	0.99995	no

ENSG00000156711	ENSG00000156711	MAPK13	6:36095585-36107842	q1	q2	OK	1.04525	0.615943	-0.762976	-0.599305	0.32465	0.99995	no

ENSG00000156735	ENSG00000156735	BAG4	8:37962759-38070819	q1	q2	OK	8.88337	9.8771	0.15298	0.190258	0.7767	0.99995	no

ENSG00000156738	ENSG00000156738	MS4A1	11:60223224-60238233	q1	q2	NOTEST	0.179632	0.0423151	-2.0858	0	1	1	no

ENSG00000156750	ENSG00000156750	AQP7P3	9:42845767-42876505	q1	q2	NOTEST	0	0	0	0	1	1	no

ENSG00000156755	ENSG00000156755	IGKV1OR-2	9:69777098-69777953	q1	q2	NOTEST	0	0.168269	inf	0	1	1	no

ENSG00000156787	ENSG00000156787	TBC1D31	8:124014799-124164393	q1	q2	OK	8.67543	9.25271	0.0929412	0.0884327	0.88325	0.99995	no

ENSG00000156795	ENSG00000156795	WDYHV1	8:124428964-124479470	q1	q2	OK	13.177	15.78	0.260072	0.415833	0.54495	0.99995	no

ENSG00000156802	ENSG00000156802	ATAD2	8:124332089-124428590	q1	q2	OK	29.4431	31.6177	0.102805	0.229002	0.71985	0.99995	no

ENSG00000156804	ENSG00000156804	FBXO32	8:124510128-124553446	q1	q2	OK	0.799453	0.91768	0.198977	0.135988	0.79685	0.99995	no

ENSG00000156831	ENSG00000156831	NSMCE2	8:126036501-126379362	q1	q2	OK	24.9955	27.2155	0.122765	0.156638	0.81865	0.99995	no

ENSG00000156853	ENSG00000156853	ZNF689	16:30597227-30635333	q1	q2	OK	10.03	9.2797	-0.112166	-0.231925	0.7278	0.99995	no

ENSG00000156858	ENSG00000156858	PRR14	16:30662037-30667761	q1	q2	OK	25.5085	19.7563	-0.368664	-0.626175	0.3445	0.99995	no

ENSG00000156860	ENSG00000156860	FBRS	16:30669751-30682135	q1	q2	OK	25.0289	22.0992	-0.179603	-0.265203	0.574	0.99995	no

ENSG00000156869	ENSG00000156869	FRRS1	1:100174258-100232187	q1	q2	OK	0.699758	0.747667	0.0955392	0.0822738	0.8713	0.99995	no

ENSG00000156873	ENSG00000156873	PHKG2	16:30709029-30787628	q1	q2	OK	16.6744	17.1464	0.0402693	0.0197388	0.95555	0.99995	no

ENSG00000156875	ENSG00000156875	HIAT1	1:100503652-100598511	q1	q2	OK	33.6837	36.0446	0.0977309	0.198269	0.77225	0.99995	no

ENSG00000156876	ENSG00000156876	SASS6	1:100503652-100598511	q1	q2	OK	7.31093	8.59226	0.232983	0.280585	0.6991	0.99995	no

ENSG00000156885	ENSG00000156885	COX6A2	16:31439051-31439967	q1	q2	NOTEST	0	0	0	0	1	1	no

ENSG00000156886	ENSG00000156886	ITGAD	16:31404632-31437826	q1	q2	NOTEST	0	0.00618749	inf	0	1	1	no

ENSG00000156920	ENSG00000156920	GPR112	X:135383121-135519215	q1	q2	NOTEST	0.00276298	0.00287588	0.0577788	0	1	1	no

ENSG00000156925	ENSG00000156925	ZIC3	X:136648300-136659850	q1	q2	NOTEST	0.320444	0.417525	0.38179	0	1	1	no

ENSG00000156928	ENSG00000156928	MALSU1	7:23338357-23510086	q1	q2	OK	26.9073	23.8865	-0.171802	-0.0962087	0.857	0.99995	no

ENSG00000156931	ENSG00000156931	VPS8	3:184529930-184770402	q1	q2	OK	18.1964	18.7236	0.0412056	0.0563401	0.8911	0.99995	no

ENSG00000156958	ENSG00000156958	GALK2	15:49398267-49913128	q1	q2	OK	10.3389	9.71287	-0.0901194	-0.0475619	0.9166	0.99995	no

ENSG00000156959	ENSG00000156959	LHFPL4	3:9543480-9595486	q1	q2	NOTEST	0.100635	0.190531	0.920898	0	1	1	no

ENSG00000156966	ENSG00000156966	B3GNT7	2:232260253-232265875	q1	q2	NOTEST	0.118263	0.224909	0.927337	0	1	1	no

ENSG00000156968	ENSG00000156968	MPV17L	16:15489610-15737023	q1	q2	NOTEST	0.0609296	0.192127	1.65685	0	1	1	no

ENSG00000156970	ENSG00000156970	BUB1B	15:40453223-40569688	q1	q2	OK	44.5605	48.338	0.117392	0.18993	0.67455	0.99995	no

ENSG00000156973	ENSG00000156973	PDE6D	2:232597134-232673963	q1	q2	OK	24.2589	27.1834	0.164211	0.203179	0.76125	0.99995	no

ENSG00000156976	ENSG00000156976	EIF4A2	3:186499568-186524847	q1	q2	OK	231.645	248.315	0.100256	0.172681	0.7602	0.99995	no

ENSG00000156983	ENSG00000156983	BRPF1	3:9773412-9789702	q1	q2	OK	11.656	11.1637	-0.0622507	-0.0854888	0.8878	0.99995	no

ENSG00000156990	ENSG00000156990	RPUSD3	3:9791627-9896822	q1	q2	OK	25.9123	21.9946	-0.23649	-0.154066	0.82575	0.99995	no

ENSG00000157005	ENSG00000157005	SST	3:187386693-187388187	q1	q2	OK	0.574388	0.914169	0.670434	0.498012	0.47505	0.99995	no

ENSG00000157014	ENSG00000157014	TATDN2	3:10289706-10362862	q1	q2	OK	12.7222	13.6685	0.103499	0.12163	0.8294	0.99995	no

ENSG00000157017	ENSG00000157017	GHRL	3:10289706-10362862	q1	q2	OK	0.57831	0.265431	-1.12351	-0.113086	0.183	0.99995	no

ENSG00000157020	ENSG00000157020	SEC13	3:10289706-10362862	q1	q2	OK	73.8156	68.6854	-0.103923	-0.190033	0.7706	0.99995	no

ENSG00000157021	ENSG00000157021	FAM92A1P1	15:41455359-41456572	q1	q2	OK	4.4027	4.66867	0.0846207	0.102484	0.8854	0.99995	no

ENSG00000157036	ENSG00000157036	EXOG	3:38537617-38583652	q1	q2	OK	13.4403	15.286	0.185647	0.283243	0.62735	0.99995	no

ENSG00000157045	ENSG00000157045	NTAN1	16:15068447-15248421	q1	q2	OK	29.6613	31.0025	0.0638005	0.0490685	0.9364	0.99995	no

ENSG00000157060	ENSG00000157060	SHCBP1L	1:182868999-182922660	q1	q2	NOTEST	0	0	0	0	1	1	no

ENSG00000157064	ENSG00000157064	NMNAT2	1:183217371-183387737	q1	q2	OK	17.2634	13.6284	-0.341098	-0.747727	0.24005	0.99995	no

ENSG00000157077	ENSG00000157077	ZFYVE9	1:52608045-52831865	q1	q2	OK	11.8229	11.443	-0.0471192	-0.0817631	0.9027	0.99995	no

ENSG00000157087	ENSG00000157087	ATP2B2	3:10365706-10749716	q1	q2	NOTEST	0.356069	0.188811	-0.915218	0	1	1	no

ENSG00000157093	ENSG00000157093	LYZL4	3:42438569-42452092	q1	q2	NOTEST	0	0	0	0	1	1	no

ENSG00000157103	ENSG00000157103	SLC6A1	3:11034409-11080933	q1	q2	OK	2.10064	1.58	-0.410908	-0.454876	0.39145	0.99995	no

ENSG00000157106	ENSG00000157106	SMG1	16:18814404-18937776	q1	q2	OK	19.1872	19.9758	0.0581093	0.077323	0.84025	0.99995	no

ENSG00000157107	ENSG00000157107	FCHO2	5:72251807-72386349	q1	q2	OK	4.49272	5.20271	0.211675	0.368666	0.547	0.99995	no

ENSG00000157110	ENSG00000157110	RBPMS	8:30239634-30429778	q1	q2	OK	38.1349	44.946	0.23708	0.403389	0.5268	0.99995	no

ENSG00000157111	ENSG00000157111	TMEM171	5:72416118-72427644	q1	q2	NOTEST	0.214741	0.161746	-0.408868	0	1	1	no

ENSG00000157119	ENSG00000157119	KLHL40	3:42727010-42734036	q1	q2	NOTEST	0.010798	0	#NAME?	0	1	1	no

ENSG00000157131	ENSG00000157131	C8A	1:57320478-57383894	q1	q2	NOTEST	0.0116051	0	#NAME?	0	1	1	no

ENSG00000157150	ENSG00000157150	TIMP4	3:12045875-12232900	q1	q2	OK	6.39669	6.84709	0.0981647	0.147255	0.83715	0.99995	no

ENSG00000157152	ENSG00000157152	SYN2	3:12045875-12232900	q1	q2	NOTEST	0.46149	0.344904	-0.420104	0	1	1	no

ENSG00000157168	ENSG00000157168	NRG1	8:31496901-32622548	q1	q2	OK	2.86035	3.11485	0.122971	0.143591	0.81895	0.99995	no

ENSG00000157181	ENSG00000157181	C1orf27	1:186344889-186390510	q1	q2	OK	11.2198	10.7929	-0.0559541	-0.0760231	0.87075	0.99995	no

ENSG00000157184	ENSG00000157184	CPT2	1:53662100-53686289	q1	q2	OK	7.69831	7.93845	0.0443148	0.0580766	0.9298	0.99995	no

ENSG00000157191	ENSG00000157191	NECAP2	1:16767166-16786573	q1	q2	OK	23.602	23.0155	-0.0363045	-0.0638246	0.91345	0.99995	no

ENSG00000157193	ENSG00000157193	LRP8	1:53711216-53793742	q1	q2	OK	15.6568	17.2462	0.139495	0.237492	0.68505	0.99995	no

ENSG00000157211	ENSG00000157211	CDCP2	1:54598746-54879152	q1	q2	NOTEST	0.099137	0.00911369	-3.44332	0	1	1	no

ENSG00000157212	ENSG00000157212	PAXIP1	7:154720207-154794794	q1	q2	OK	11.026	12.0835	0.132123	0.249525	0.69635	0.99995	no

ENSG00000157214	ENSG00000157214	STEAP2	7:89511666-89867451	q1	q2	OK	0.671953	0.828517	0.302171	0.21457	0.61485	0.99995	no

ENSG00000157216	ENSG00000157216	SSBP3	1:54598746-54879152	q1	q2	OK	98.9309	90.6798	-0.125641	-0.217015	0.71805	0.99995	no

ENSG00000157219	ENSG00000157219	HTR5A	7:154858776-154877459	q1	q2	NOTEST	0	0	0	0	1	1	no

ENSG00000157224	ENSG00000157224	CLDN12	7:89964536-90839905	q1	q2	OK	20.0405	21.5498	0.104756	0.101085	0.82575	0.99995	no

ENSG00000157227	ENSG00000157227	MMP14	14:23305765-23318236	q1	q2	OK	61.3322	60.4741	-0.020326	-0.0354968	0.95085	0.99995	no

ENSG00000157240	ENSG00000157240	FZD1	7:90893782-90898123	q1	q2	OK	29.0867	24.8578	-0.22666	-0.549961	0.42905	0.99995	no

ENSG00000157259	ENSG00000157259	GATAD1	7:92076766-92157845	q1	q2	OK	23.2643	22.1133	-0.0731996	-0.114968	0.8612	0.99995	no

ENSG00000157303	ENSG00000157303	SUSD3	9:95820988-95847420	q1	q2	NOTEST	0.227456	0.0427356	-2.41208	0	1	1	no

ENSG00000157306	ENSG00000157306	RP11-66N24.4	14:23980968-24048009	q1	q2	OK	5.24289	6.05167	0.206971	0.0875751	0.9364	0.99995	no

ENSG00000157315	ENSG00000157315	TMED6	16:69139466-69442474	q1	q2	OK	1.02345	1.03431	0.0152191	0.00140942	0.99015	0.99995	no

ENSG00000157322	ENSG00000157322	CLEC18A	16:69984809-69998141	q1	q2	NOTEST	0.19354	0.0921481	-1.07061	0	1	1	no

ENSG00000157326	ENSG00000157326	DHRS4	14:24407939-24475617	q1	q2	OK	9.13923	10.0355	0.134973	0.116897	0.8628	0.99995	no

ENSG00000157330	ENSG00000157330	C1orf158	1:12806162-12823847	q1	q2	NOTEST	0	0	0	0	1	1	no

ENSG00000157335	ENSG00000157335	CLEC18C	16:70147528-70221264	q1	q2	NOTEST	0.139273	0.126528	-0.138462	0	1	1	no

ENSG00000157343	ENSG00000157343	ARMC12	6:35704808-35716856	q1	q2	OK	1.15487	1.07923	-0.0977191	-0.0932382	0.88325	0.99995	no

ENSG00000157349	ENSG00000157349	DDX19B	16:70323565-70407286	q1	q2	OK	21.346	18.063	-0.240925	-0.240486	0.71685	0.99995	no

ENSG00000157350	ENSG00000157350	ST3GAL2	16:70413337-70473140	q1	q2	OK	19.1581	19.4705	0.0233331	0.0367974	0.9488	0.99995	no

ENSG00000157353	ENSG00000157353	FUK	16:70488323-70514177	q1	q2	OK	3.57864	3.30161	-0.116242	-0.128769	0.808	0.99995	no

ENSG00000157358	ENSG00000157358	PRAMEF15	1:13641972-13648988	q1	q2	NOTEST	0	0.0138519	inf	0	1	1	no

ENSG00000157368	ENSG00000157368	IL34	16:70613797-70694585	q1	q2	OK	0.831101	1.24453	0.582507	0.49454	0.4387	0.99995	no

ENSG00000157379	ENSG00000157379	DHRS1	14:24759803-24769039	q1	q2	OK	8.91049	6.91918	-0.364903	-0.477799	0.4647	0.99995	no

ENSG00000157388	ENSG00000157388	CACNA1D	3:53528682-53880417	q1	q2	OK	0.661859	0.709987	0.101269	0.0638188	0.8942	0.99995	no

ENSG00000157399	ENSG00000157399	ARSE	X:2852698-2886286	q1	q2	OK	1.50763	1.23991	-0.282047	-0.272506	0.6753	0.99995	no

ENSG00000157404	ENSG00000157404	KIT	4:55524084-55606881	q1	q2	OK	5.63094	5.97628	0.085872	0.154111	0.80505	0.99995	no

ENSG00000157423	ENSG00000157423	HYDIN	16:70841280-71264625	q1	q2	OK	0.621996	1.11727	0.844995	0.585869	0.1119	0.99995	no

ENSG00000157426	ENSG00000157426	AASDH	4:57204455-57253666	q1	q2	OK	5.26883	5.34889	0.021755	0.0357301	0.9598	0.99995	no

ENSG00000157429	ENSG00000157429	ZNF19	16:71481499-71598992	q1	q2	OK	3.50649	3.55189	0.0185568	0.0131278	0.98195	0.99995	no

ENSG00000157445	ENSG00000157445	CACNA2D3	3:54156573-55108584	q1	q2	OK	0.419254	0.544548	0.377235	0.238902	0.58225	0.99995	no

ENSG00000157450	ENSG00000157450	RNF111	15:59063390-59389618	q1	q2	OK	22.8633	21.5507	-0.0853003	-0.0677779	0.8921	0.99995	no

ENSG00000157456	ENSG00000157456	CCNB2	15:59397276-59425449	q1	q2	OK	77.0426	79.0716	0.0375029	0.0886854	0.8918	0.99995	no

ENSG00000157470	ENSG00000157470	FAM81A	15:59427112-59815748	q1	q2	OK	3.22338	3.88546	0.269511	0.139996	0.65065	0.99995	no

ENSG00000157483	ENSG00000157483	MYO1E	15:59427112-59815748	q1	q2	OK	10.5264	11.4842	0.125647	0.141069	0.75315	0.99995	no

ENSG00000157500	ENSG00000157500	APPL1	3:57261764-57326710	q1	q2	OK	10.716	11.9611	0.158592	0.278984	0.5895	0.99995	no

ENSG00000157502	ENSG00000157502	MUM1L1	X:105412297-105452949	q1	q2	OK	1.63921	1.66081	0.0188806	0.0268538	0.97105	0.99995	no

ENSG00000157510	ENSG00000157510	AFAP1L1	5:148521045-148721365	q1	q2	OK	0.853305	0.993493	0.219449	0.189239	0.75	0.99995	no

ENSG00000157514	ENSG00000157514	TSC22D3	X:106956450-107020572	q1	q2	OK	21.4391	21.8311	0.026142	0.0535642	0.93165	0.99995	no

ENSG00000157538	ENSG00000157538	DSCR3	21:38580803-38640785	q1	q2	OK	23.6151	25.6625	0.119951	0.215015	0.71525	0.99995	no

ENSG00000157540	ENSG00000157540	DYRK1A	21:38737778-38889753	q1	q2	OK	28.3762	30.832	0.11975	0.162815	0.7171	0.99995	no

ENSG00000157542	ENSG00000157542	KCNJ6	21:38979677-39288749	q1	q2	OK	0.82182	0.898914	0.129361	0.0733289	0.90675	0.99995	no

ENSG00000157551	ENSG00000157551	KCNJ15	21:39493544-39679279	q1	q2	NOTEST	0.00304955	0.0282193	3.21001	0	1	1	no

ENSG00000157554	ENSG00000157554	ERG	21:39751948-40033704	q1	q2	NOTEST	0.0984886	0.262644	1.41508	0	1	1	no

ENSG00000157557	ENSG00000157557	ETS2	21:40177230-40196879	q1	q2	OK	5.04576	4.99014	-0.0159908	-0.0275839	0.965	0.99995	no

ENSG00000157570	ENSG00000157570	TSPAN18	11:44740941-44972840	q1	q2	OK	69.1608	57.0328	-0.278163	-0.421595	0.49015	0.99995	no

ENSG00000157578	ENSG00000157578	LCA5L	21:40752169-40817731	q1	q2	OK	1.0611	0.84906	-0.321623	-0.0998183	0.6622	0.99995	no

ENSG00000157593	ENSG00000157593	SLC35B2	6:44221832-44225291	q1	q2	OK	45.4406	44.5965	-0.0270488	-0.0650938	0.92665	0.99995	no

ENSG00000157600	ENSG00000157600	TMEM164	X:109245858-109425962	q1	q2	OK	10.5817	12.7743	0.271676	0.169922	0.49075	0.99995	no

ENSG00000157601	ENSG00000157601	MX1	21:42792230-42831141	q1	q2	OK	0.702291	0.745602	0.0863364	0.0546285	0.89155	0.99995	no

ENSG00000157613	ENSG00000157613	CREB3L1	11:46299211-46342972	q1	q2	OK	7.08498	6.20516	-0.191296	-0.26844	0.6568	0.99995	no

ENSG00000157617	ENSG00000157617	C2CD2	21:43305220-43373999	q1	q2	OK	4.0367	4.80545	0.251495	0.425562	0.50855	0.99995	no

ENSG00000157625	ENSG00000157625	TAB3	X:30845558-30993201	q1	q2	OK	8.14734	8.00883	-0.0247364	-0.055597	0.9336	0.99995	no

ENSG00000157637	ENSG00000157637	SLC38A10	17:79218799-79269347	q1	q2	OK	27.2699	22.5883	-0.271736	-0.521984	0.40575	0.99995	no

ENSG00000157653	ENSG00000157653	C9orf43	9:116169514-116191964	q1	q2	OK	0.510324	0.375278	-0.443451	-0.104685	0.81125	0.99995	no

ENSG00000157654	ENSG00000157654	PALM2-AKAP2	9:112403067-112934792	q1	q2	OK	0.378606	0.725798	0.938872	0.0734246	0.55095	0.99995	no

ENSG00000157657	ENSG00000157657	ZNF618	9:116638561-116818871	q1	q2	OK	26.383	25.9916	-0.0215582	-0.0420931	0.94005	0.99995	no

ENSG00000157680	ENSG00000157680	DGKI	7:137065782-137531838	q1	q2	OK	1.53258	1.60906	0.0702572	0.0651795	0.87065	0.99995	no

ENSG00000157693	ENSG00000157693	C9orf91	9:117373485-117408702	q1	q2	OK	3.94198	4.05136	0.0394882	0.052534	0.92885	0.99995	no

ENSG00000157703	ENSG00000157703	SVOPL	7:138279029-138386097	q1	q2	NOTEST	0.0618825	0.0942293	0.606644	0	1	1	no

ENSG00000157734	ENSG00000157734	SNX22	15:64443913-64455404	q1	q2	OK	2.54295	1.83399	-0.471519	-0.183807	0.7907	0.99995	no

ENSG00000157741	ENSG00000157741	UBN2	7:138915101-138992982	q1	q2	OK	6.0318	6.22351	0.045139	0.101762	0.87905	0.99995	no

ENSG00000157764	ENSG00000157764	BRAF	7:140218219-140624564	q1	q2	OK	11.7673	11.4481	-0.0396825	-0.0408123	0.9539	0.99995	no

ENSG00000157765	ENSG00000157765	SLC34A2	4:25656922-25680370	q1	q2	NOTEST	0.0248176	0.0121497	-1.03045	0	1	1	no

ENSG00000157766	ENSG00000157766	ACAN	15:89346673-89418585	q1	q2	NOTEST	0.279119	0.348237	0.319191	0	1	1	no

ENSG00000157778	ENSG00000157778	PSMG3	7:1606965-1629262	q1	q2	OK	32.2424	27.9796	-0.204584	-0.328088	0.64835	0.99995	no

ENSG00000157782	ENSG00000157782	CABP1	12:121067907-121105127	q1	q2	NOTEST	0.459746	0.45027	-0.030047	0	1	1	no

ENSG00000157796	ENSG00000157796	WDR19	4:39184023-39287430	q1	q2	OK	22.8837	23.1892	0.0191378	0.0310341	0.9547	0.99995	no

ENSG00000157800	ENSG00000157800	SLC37A3	7:139993492-140126050	q1	q2	OK	23.1077	23.6367	0.0326561	0.0551351	0.9131	0.99995	no

ENSG00000157823	ENSG00000157823	AP3S2	15:90373830-90456188	q1	q2	OK	28.8341	28.3609	-0.0238768	-0.0221084	0.9683	0.99995	no

ENSG00000157827	ENSG00000157827	FMNL2	2:153191750-153506348	q1	q2	OK	59.9369	64.5382	0.106707	0.236616	0.72065	0.99995	no

ENSG00000157828	ENSG00000157828	RPS4Y2	Y:22918049-22942918	q1	q2	NOTEST	0.0400903	0	#NAME?	0	1	1	no

ENSG00000157833	ENSG00000157833	GAREML	2:26395959-26412532	q1	q2	OK	11.4274	10.2902	-0.15123	-0.326995	0.63085	0.99995	no

ENSG00000157837	ENSG00000157837	SPPL3	12:121200312-121342174	q1	q2	OK	27.5943	25.8643	-0.0934039	-0.134045	0.83685	0.99995	no

ENSG00000157851	ENSG00000157851	DPYSL5	2:27070614-27250064	q1	q2	OK	134.867	126.628	-0.0909468	-0.17948	0.78525	0.99995	no

ENSG00000157856	ENSG00000157856	DRC1	2:26624783-26679579	q1	q2	NOTEST	0.331909	0.400843	0.272251	0	1	1	no

ENSG00000157869	ENSG00000157869	RAB28	4:13362977-13485989	q1	q2	OK	20.1319	23.7772	0.240096	0.477918	0.46885	0.99995	no

ENSG00000157870	ENSG00000157870	FAM213B	1:2517929-2718286	q1	q2	OK	19.502	16.4279	-0.247472	-0.487853	0.43435	0.99995	no

ENSG00000157873	ENSG00000157873	TNFRSF14	1:2481358-2496821	q1	q2	NOTEST	0.255754	0.0767918	-1.73574	0	1	1	no

ENSG00000157881	ENSG00000157881	PANK4	1:2439971-2458039	q1	q2	OK	15.2558	16.9837	0.154795	0.206613	0.7061	0.99995	no

ENSG00000157884	ENSG00000157884	CIB4	2:26804069-26864236	q1	q2	NOTEST	0	0	0	0	1	1	no

ENSG00000157890	ENSG00000157890	MEGF11	15:66187416-66546085	q1	q2	OK	1.03109	0.903067	-0.191271	-0.178239	0.67185	0.99995	no

ENSG00000157895	ENSG00000157895	C12orf43	12:121406415-121454305	q1	q2	OK	13.0376	12.6666	-0.0416545	-0.0669428	0.9162	0.99995	no

ENSG00000157911	ENSG00000157911	PEX10	1:2323266-2345236	q1	q2	OK	13.2009	12.171	-0.117195	-0.115089	0.85205	0.99995	no

ENSG00000157916	ENSG00000157916	RER1	1:2323266-2345236	q1	q2	OK	54.2855	57.5292	0.0837276	0.125364	0.84215	0.99995	no

ENSG00000157927	ENSG00000157927	RADIL	7:4836685-4923350	q1	q2	OK	3.90906	3.83748	-0.0266607	-0.0278369	0.95755	0.99995	no

ENSG00000157933	ENSG00000157933	SKI	1:2160133-2241558	q1	q2	OK	26.7891	25.5984	-0.065592	-0.127347	0.81415	0.99995	no

ENSG00000157950	ENSG00000157950	SSX2B	X:52780317-52790617	q1	q2	NOTEST	0	0	0	0	1	1	no

ENSG00000157954	ENSG00000157954	WIPI2	7:5229818-5273457	q1	q2	OK	31.1811	27.7698	-0.167153	-0.337339	0.59955	0.99995	no

ENSG00000157965	ENSG00000157965	SSX8	X:52651984-52662998	q1	q2	NOTEST	0	0	0	0	1	1	no

ENSG00000157978	ENSG00000157978	LDLRAP1	1:25870070-25895377	q1	q2	OK	3.35391	3.47644	0.0517691	0.0741562	0.90225	0.99995	no

ENSG00000157985	ENSG00000157985	AGAP1	2:236402732-237040444	q1	q2	OK	16.8859	15.6249	-0.111975	-0.187096	0.7463	0.99995	no

ENSG00000157992	ENSG00000157992	KRTCAP3	2:27665232-27712656	q1	q2	OK	3.84931	3.08322	-0.320163	-0.130763	0.8175	0.99995	no

ENSG00000157999	ENSG00000157999	ANKRD61	7:6048875-6098861	q1	q2	OK	1.11248	1.10713	-0.00694944	-0.00136171	0.98815	0.99995	no

ENSG00000158006	ENSG00000158006	PAFAH2	1:26286257-26324648	q1	q2	OK	4.25044	3.90322	-0.122948	-0.161445	0.7623	0.99995	no

ENSG00000158008	ENSG00000158008	EXTL1	1:26346374-26362955	q1	q2	OK	0.483759	0.516473	0.0944056	0.0674641	0.91235	0.99995	no

ENSG00000158014	ENSG00000158014	SLC30A2	1:26363742-26372624	q1	q2	NOTEST	0	0	0	0	1	1	no

ENSG00000158019	ENSG00000158019	BRE	2:27994583-28561768	q1	q2	OK	23.9506	23.082	-0.053291	-0.0868357	0.90105	0.99995	no

ENSG00000158022	ENSG00000158022	TRIM63	1:26377794-26394927	q1	q2	NOTEST	0.0151147	0.0358227	1.24492	0	1	1	no

ENSG00000158023	ENSG00000158023	WDR66	12:122326636-122441833	q1	q2	OK	0.599797	0.637121	0.0870935	0.0309227	0.918	0.99995	no

ENSG00000158042	ENSG00000158042	MRPL17	11:6702012-6704632	q1	q2	OK	17.6796	18.1388	0.0369945	0.0542474	0.93325	0.99995	no

ENSG00000158050	ENSG00000158050	DUSP2	2:96808904-96811179	q1	q2	NOTEST	0.320003	0.209602	-0.610432	0	1	1	no

ENSG00000158055	ENSG00000158055	GRHL3	1:24634045-24799466	q1	q2	NOTEST	0.058085	0.0967983	0.736815	0	1	1	no

ENSG00000158062	ENSG00000158062	UBXN11	1:26605666-26647014	q1	q2	OK	12.1808	9.41	-0.372346	-0.353817	0.5696	0.99995	no

ENSG00000158077	ENSG00000158077	NLRP14	11:7041676-7092539	q1	q2	NOTEST	0.0215148	0.0422043	0.97206	0	1	1	no

ENSG00000158079	ENSG00000158079	PTPDC1	9:96793075-96872138	q1	q2	OK	4.52209	5.41506	0.259989	0.41222	0.55115	0.99995	no

ENSG00000158089	ENSG00000158089	GALNT14	2:31133332-31378068	q1	q2	OK	0.792703	0.86488	0.12572	0.0951326	0.84005	0.99995	no

ENSG00000158092	ENSG00000158092	NCK1	3:136581049-136729927	q1	q2	OK	17.2901	19.0958	0.143306	0.249624	0.67385	0.99995	no

ENSG00000158104	ENSG00000158104	HPD	12:122277432-122301502	q1	q2	NOTEST	0.0605469	0.0391441	-0.629259	0	1	1	no

ENSG00000158106	ENSG00000158106	RHPN1	8:144451056-144466390	q1	q2	OK	11.8067	9.84935	-0.261505	-0.482108	0.49075	0.99995	no

ENSG00000158109	ENSG00000158109	TPRG1L	1:3541565-3546691	q1	q2	OK	11.0619	9.38368	-0.237372	-0.454961	0.51695	0.99995	no

ENSG00000158113	ENSG00000158113	LRRC43	12:122652284-122688018	q1	q2	OK	0.776932	0.501554	-0.631385	-0.431341	0.5126	0.99995	no

ENSG00000158122	ENSG00000158122	AAED1	9:99401858-99417585	q1	q2	OK	0.746186	1.07397	0.525351	0.556619	0.39175	0.99995	no

ENSG00000158125	ENSG00000158125	XDH	2:31557186-31637581	q1	q2	NOTEST	0	0	0	0	1	1	no

ENSG00000158156	ENSG00000158156	XKR8	1:28261503-28294607	q1	q2	OK	9.17788	8.46499	-0.116653	-0.196749	0.74115	0.99995	no

ENSG00000158158	ENSG00000158158	CNNM4	2:97426638-97477628	q1	q2	OK	4.3933	3.97898	-0.142905	-0.260182	0.69005	0.99995	no

ENSG00000158161	ENSG00000158161	EYA3	1:28296854-28415207	q1	q2	OK	9.46214	10.2	0.108328	0.196365	0.7355	0.99995	no

ENSG00000158163	ENSG00000158163	DZIP1L	3:137780831-137834660	q1	q2	OK	5.82217	5.84141	0.00475948	0.00460419	0.9937	0.99995	no

ENSG00000158164	ENSG00000158164	TMSB15A	X:101768603-101771712	q1	q2	NOTEST	0	0	0	0	1	1	no

ENSG00000158169	ENSG00000158169	FANCC	9:97861335-98079991	q1	q2	OK	10.8241	11.5232	0.0903052	0.137033	0.7921	0.99995	no

ENSG00000158186	ENSG00000158186	MRAS	3:138066538-138124375	q1	q2	OK	9.37586	9.33826	-0.00579698	-0.00385752	0.9901	0.99995	no

ENSG00000158195	ENSG00000158195	WASF2	1:27730729-27816669	q1	q2	OK	28.4564	28.4162	-0.00204365	-0.00498008	0.99455	0.99995	no

ENSG00000158201	ENSG00000158201	ABHD3	18:19230857-19284766	q1	q2	OK	11.45	10.9822	-0.0601841	-0.1022	0.87535	0.99995	no

ENSG00000158220	ENSG00000158220	ESYT3	3:138153427-138200528	q1	q2	NOTEST	0.258746	0.164968	-0.64935	0	1	1	no

ENSG00000158234	ENSG00000158234	FAIM	3:138327447-138352218	q1	q2	OK	6.47995	6.58431	0.0230493	0.0280663	0.96405	0.99995	no

ENSG00000158246	ENSG00000158246	FAM46B	1:27331510-27339327	q1	q2	OK	2.12323	2.12795	0.00320433	0.00407852	0.99495	0.99995	no

ENSG00000158258	ENSG00000158258	CLSTN2	3:139654026-140296239	q1	q2	OK	0.810937	0.968416	0.256037	0.402317	0.53495	0.99995	no

ENSG00000158270	ENSG00000158270	COLEC12	18:319360-500722	q1	q2	OK	11.2716	11.2331	-0.00493108	-0.0100541	0.98795	0.99995	no

ENSG00000158286	ENSG00000158286	RNF207	1:6241328-6296032	q1	q2	OK	1.84729	1.199	-0.623585	-0.120118	0.52015	0.99995	no

ENSG00000158290	ENSG00000158290	CUL4B	X:119658463-119709649	q1	q2	OK	24.4172	25.3303	0.052964	0.112902	0.8588	0.99995	no

ENSG00000158292	ENSG00000158292	GPR153	1:6307405-6321035	q1	q2	OK	8.46619	8.17183	-0.0510527	-0.105113	0.88175	0.99995	no

ENSG00000158296	ENSG00000158296	SLC13A3	20:45186462-45304714	q1	q2	OK	1.15035	1.00003	-0.202027	-0.18165	0.71755	0.99995	no

ENSG00000158301	ENSG00000158301	GPRASP2	X:101854095-101973607	q1	q2	OK	11.9114	9.70216	-0.295968	-0.306632	0.53585	0.99995	no

ENSG00000158315	ENSG00000158315	RHBDL2	1:39325671-39407471	q1	q2	OK	0.666072	0.453042	-0.556033	-0.153183	0.72155	0.99995	no

ENSG00000158321	ENSG00000158321	AUTS2	7:69063904-70258054	q1	q2	OK	75.4241	66.7988	-0.175202	-0.198939	0.625	0.99995	no

ENSG00000158352	ENSG00000158352	SHROOM4	X:50334646-50557302	q1	q2	NOTEST	0.0671973	0.0198384	-1.76011	0	1	1	no

ENSG00000158373	ENSG00000158373	HIST1H2BD	6:26158348-26171577	q1	q2	OK	9.08098	7.22117	-0.330615	-0.390127	0.5774	0.99995	no

ENSG00000158402	ENSG00000158402	CDC25C	5:137620953-137685416	q1	q2	OK	17.2559	16.8673	-0.032856	-0.0331234	0.96395	0.99995	no

ENSG00000158406	ENSG00000158406	HIST1H4H	6:26281282-26285762	q1	q2	OK	2.70955	1.8586	-0.543838	-0.442833	0.53045	0.99995	no

ENSG00000158411	ENSG00000158411	MITD1	2:99613723-100106497	q1	q2	OK	24.15	27.2281	0.173072	0.0792674	0.8776	0.99995	no

ENSG00000158417	ENSG00000158417	EIF5B	2:99613723-100106497	q1	q2	OK	44.6576	47.8705	0.100229	0.137033	0.79335	0.99995	no

ENSG00000158423	ENSG00000158423	RIBC1	X:53401069-53458059	q1	q2	NOTEST	0	0	0	0	1	1	no

ENSG00000158427	ENSG00000158427	TMSB15B	X:103172004-103229570	q1	q2	OK	1.01527	0.937325	-0.115245	-0.0803175	0.89695	0.99995	no

ENSG00000158428	ENSG00000158428	C2orf62	2:219135114-219232822	q1	q2	NOTEST	0.183252	0.067842	-1.43358	0	1	1	no

ENSG00000158435	ENSG00000158435	CNOT11	2:101436613-101886778	q1	q2	OK	24.2678	24.7356	0.0275448	0.0181146	0.97265	0.99995	no

ENSG00000158445	ENSG00000158445	KCNB1	20:47980413-48099184	q1	q2	OK	1.37051	1.39326	0.0237506	0.0426106	0.95275	0.99995	no

ENSG00000158457	ENSG00000158457	TSPAN33	7:128784711-128809538	q1	q2	OK	5.54711	4.67851	-0.245688	-0.363808	0.58275	0.99995	no

ENSG00000158458	ENSG00000158458	NRG2	5:139226363-139422884	q1	q2	OK	1.47006	1.02574	-0.519206	-0.466577	0.4887	0.99995	no

ENSG00000158467	ENSG00000158467	AHCYL2	7:128864863-129070052	q1	q2	OK	15.2658	14.8916	-0.0358019	-0.0677278	0.89855	0.99995	no

ENSG00000158470	ENSG00000158470	B4GALT5	20:48249481-48330415	q1	q2	OK	23	26.2036	0.188134	0.462113	0.5102	0.99995	no

ENSG00000158473	ENSG00000158473	CD1D	1:158149736-158154686	q1	q2	NOTEST	0.284328	0.209015	-0.443951	0	1	1	no

ENSG00000158477	ENSG00000158477	CD1A	1:158223926-158228059	q1	q2	NOTEST	0	0	0	0	1	1	no

ENSG00000158480	ENSG00000158480	SPATA2	20:48519927-48532080	q1	q2	OK	4.23444	4.54734	0.102852	0.180213	0.79625	0.99995	no

ENSG00000158481	ENSG00000158481	CD1C	1:158259575-158263420	q1	q2	OK	0.749459	1.83326	1.29049	1.20984	0.0855	0.99995	no

ENSG00000158482	ENSG00000158482	SNX29P1	16:21391312-21403412	q1	q2	NOTEST	0.0397537	0	#NAME?	0	1	1	no

ENSG00000158483	ENSG00000158483	FAM86C1	11:71498555-71532594	q1	q2	OK	3.92098	3.88378	-0.0137531	-0.0181664	0.9797	0.99995	no

ENSG00000158485	ENSG00000158485	CD1B	1:158297740-158301321	q1	q2	NOTEST	0	0	0	0	1	1	no

ENSG00000158486	ENSG00000158486	DNAH3	16:20944432-21191937	q1	q2	NOTEST	0.144286	0.0579282	-1.31659	0	1	1	no

ENSG00000158488	ENSG00000158488	CD1E	1:158323253-158327343	q1	q2	NOTEST	0	0.0367387	inf	0	1	1	no

ENSG00000158497	ENSG00000158497	HMHB1	5:142985192-143208337	q1	q2	NOTEST	0	0	0	0	1	1	no

ENSG00000158516	ENSG00000158516	CPA2	7:129906666-129929638	q1	q2	NOTEST	0.208179	0.335539	0.688653	0	1	1	no

ENSG00000158517	ENSG00000158517	NCF1	7:74071993-74306731	q1	q2	NOTEST	0	0	0	0	1	1	no

ENSG00000158525	ENSG00000158525	CPA5	7:129984629-130008571	q1	q2	NOTEST	0	0	0	0	1	1	no

ENSG00000158526	ENSG00000158526	TSR2	X:54466833-54522599	q1	q2	OK	27.4201	25.9508	-0.0794549	-0.128665	0.858	0.99995	no

ENSG00000158528	ENSG00000158528	PPP1R9A	7:94536513-94925727	q1	q2	OK	6.93408	7.64341	0.140512	0.236064	0.64535	0.99995	no

ENSG00000158545	ENSG00000158545	ZC3H18	16:88636788-88698374	q1	q2	OK	31.3564	29.9844	-0.0645479	-0.113544	0.82975	0.99995	no

ENSG00000158552	ENSG00000158552	ZFAND2B	2:220059958-220074373	q1	q2	OK	14.6009	14.2987	-0.0301737	-0.0483795	0.9423	0.99995	no

ENSG00000158553	ENSG00000158553	POM121L2	6:27253681-27279949	q1	q2	NOTEST	0.00920929	0.0266327	1.53204	0	1	1	no

ENSG00000158555	ENSG00000158555	GDPD5	11:75145684-75236948	q1	q2	OK	5.83596	5.15785	-0.1782	-0.195534	0.70685	0.99995	no

ENSG00000158560	ENSG00000158560	DYNC1I1	7:95401865-95739634	q1	q2	OK	1.72696	2.11123	0.289843	0.360721	0.5411	0.99995	no

ENSG00000158571	ENSG00000158571	PFKFB1	X:54959393-55024967	q1	q2	NOTEST	0.356322	0.360817	0.0180853	0	1	1	no

ENSG00000158578	ENSG00000158578	ALAS2	X:55026789-55057497	q1	q2	NOTEST	0	0	0	0	1	1	no

ENSG00000158604	ENSG00000158604	TMED4	7:44617492-44621886	q1	q2	OK	44.8161	45.4342	0.0197588	0.0469812	0.94525	0.99995	no

ENSG00000158615	ENSG00000158615	PPP1R15B	1:204346977-204463852	q1	q2	OK	18.5635	19.3472	0.059656	0.0799325	0.9005	0.99995	no

ENSG00000158623	ENSG00000158623	COPG2	7:130125882-130148500	q1	q2	NOTEST	0.0423635	0.117868	1.47628	0	1	1	no

ENSG00000158636	ENSG00000158636	C11orf30	11:76155966-76264069	q1	q2	OK	16.6466	17.1494	0.0429315	0.0608706	0.8943	0.99995	no

ENSG00000158639	ENSG00000158639	PAGE5	X:55246787-55250541	q1	q2	NOTEST	0	0	0	0	1	1	no

ENSG00000158669	ENSG00000158669	AGPAT6	8:41386724-41482520	q1	q2	OK	65.2295	60.8601	-0.100027	-0.132407	0.85685	0.99995	no

ENSG00000158683	ENSG00000158683	PKD1L1	7:47735327-48019178	q1	q2	NOTEST	0.067726	0.0972597	0.522133	0	1	1	no

ENSG00000158691	ENSG00000158691	ZSCAN12	6:28346731-28367511	q1	q2	OK	7.07511	7.68565	0.119414	0.244402	0.72775	0.99995	no

ENSG00000158710	ENSG00000158710	TAGLN2	1:159887896-159895522	q1	q2	OK	19.8189	18.658	-0.087087	-0.163199	0.8105	0.99995	no

ENSG00000158711	ENSG00000158711	ELK4	1:205577070-205601090	q1	q2	OK	6.54537	7.4456	0.185914	0.406029	0.53115	0.99995	no

ENSG00000158714	ENSG00000158714	SLAMF8	1:159796539-159869953	q1	q2	NOTEST	0.0469409	0.065507	0.480803	0	1	1	no

ENSG00000158715	ENSG00000158715	SLC45A3	1:205626978-205649587	q1	q2	OK	2.50283	2.47205	-0.0178506	-0.0256835	0.9728	0.99995	no

ENSG00000158716	ENSG00000158716	DUSP23	1:159750721-159752333	q1	q2	OK	2.67228	2.17175	-0.299216	-0.315578	0.66455	0.99995	no

ENSG00000158717	ENSG00000158717	RNF166	16:88762902-88772829	q1	q2	OK	17.5222	13.6019	-0.365372	-0.585696	0.3421	0.99995	no

ENSG00000158731	ENSG00000158731	OR10J6P	1:159568087-159569017	q1	q2	NOTEST	0	0	0	0	1	1	no

ENSG00000158747	ENSG00000158747	NBL1	1:19923472-19984945	q1	q2	OK	15.7148	11.597	-0.438373	-0.355809	0.64765	0.99995	no

ENSG00000158748	ENSG00000158748	HTR6	1:19991779-20006055	q1	q2	NOTEST	0.0273293	0	#NAME?	0	1	1	no

ENSG00000158764	ENSG00000158764	ITLN2	1:160901528-160924589	q1	q2	NOTEST	0.0526834	0.0258867	-1.02514	0	1	1	no

ENSG00000158769	ENSG00000158769	F11R	1:160965000-161008780	q1	q2	OK	4.11486	3.35301	-0.295387	-0.349493	0.461	0.99995	no

ENSG00000158773	ENSG00000158773	USF1	1:161009040-161015767	q1	q2	OK	51.7084	51.4829	-0.00630421	-0.0138124	0.98355	0.99995	no

ENSG00000158786	ENSG00000158786	PLA2G2F	1:20465818-20476879	q1	q2	NOTEST	0.0312153	0.0156246	-0.998437	0	1	1	no

ENSG00000158792	ENSG00000158792	SPATA2L	16:89747144-89768113	q1	q2	OK	5.10403	4.1917	-0.284102	-0.205126	0.728	0.99995	no

ENSG00000158793	ENSG00000158793	NIT1	1:161070345-161102478	q1	q2	OK	29.7657	26.5816	-0.163224	-0.189426	0.7891	0.99995	no

ENSG00000158796	ENSG00000158796	DEDD	1:161070345-161102478	q1	q2	OK	22.2458	21.2148	-0.068463	-0.0789531	0.8772	0.99995	no

ENSG00000158805	ENSG00000158805	ZNF276	16:89773541-89883065	q1	q2	OK	12.0382	9.31522	-0.369962	-0.289089	0.50935	0.99995	no

ENSG00000158806	ENSG00000158806	NPM2	8:21881635-21894408	q1	q2	OK	0.971939	0.929894	-0.0637992	-0.0406135	0.93455	0.99995	no

ENSG00000158813	ENSG00000158813	EDA	X:68835910-69259319	q1	q2	OK	0.569716	0.609589	0.0975938	0.0797554	0.84485	0.99995	no

ENSG00000158815	ENSG00000158815	FGF17	8:21899908-21906320	q1	q2	OK	0.816049	0.747687	-0.126222	-0.130026	0.85205	0.99995	no

ENSG00000158816	ENSG00000158816	VWA5B1	1:20617411-20681387	q1	q2	NOTEST	0.126487	0.0675624	-0.904692	0	1	1	no

ENSG00000158825	ENSG00000158825	CDA	1:20915440-20945401	q1	q2	NOTEST	0.213562	0.0414216	-2.3662	0	1	1	no

ENSG00000158828	ENSG00000158828	PINK1	1:20959947-20988000	q1	q2	OK	20.3768	16.2775	-0.324047	-0.30587	0.6996	0.99995	no

ENSG00000158850	ENSG00000158850	B4GALT3	1:161136199-161147803	q1	q2	OK	23.2642	20.9917	-0.148294	-0.191667	0.7108	0.99995	no

ENSG00000158856	ENSG00000158856	DMTN	8:21906505-21940038	q1	q2	OK	2.31975	2.21221	-0.0684786	-0.0656999	0.88885	0.99995	no

ENSG00000158859	ENSG00000158859	ADAMTS4	1:161154097-161184185	q1	q2	OK	1.17156	0.941729	-0.315053	-0.0956878	0.842	0.99995	no

ENSG00000158863	ENSG00000158863	FAM160B2	8:21946669-21962409	q1	q2	OK	24.4196	20.0804	-0.28225	-0.364503	0.5785	0.99995	no

ENSG00000158864	ENSG00000158864	NDUFS2	1:161154097-161184185	q1	q2	OK	79.5013	80.6588	0.0208534	0.0431272	0.94745	0.99995	no

ENSG00000158865	ENSG00000158865	SLC5A11	16:24857161-24922949	q1	q2	NOTEST	0.0952015	0.164606	0.789959	0	1	1	no

ENSG00000158869	ENSG00000158869	FCER1G	1:161185023-161193421	q1	q2	OK	1.32067	0.831631	-0.667251	-0.56448	0.39625	0.99995	no

ENSG00000158874	ENSG00000158874	APOA2	1:161185023-161193421	q1	q2	NOTEST	0	0	0	0	1	1	no

ENSG00000158882	ENSG00000158882	TOMM40L	1:161195792-161208092	q1	q2	OK	6.73347	5.79642	-0.216187	-0.339803	0.5684	0.99995	no

ENSG00000158887	ENSG00000158887	MPZ	1:161274524-161279762	q1	q2	OK	1.27863	0.66883	-0.934889	-0.675028	0.20735	0.99995	no

ENSG00000158901	ENSG00000158901	WFDC8	20:44179791-44207965	q1	q2	NOTEST	0	0.0216335	inf	0	1	1	no

ENSG00000158941	ENSG00000158941	CCAR2	8:22462144-22526661	q1	q2	OK	146.168	130.697	-0.161404	-0.182692	0.70625	0.99995	no

ENSG00000158955	ENSG00000158955	WNT9B	17:44910566-44964096	q1	q2	NOTEST	0.209275	0.119962	-0.802824	0	1	1	no

ENSG00000158966	ENSG00000158966	CACHD1	1:64936427-65158741	q1	q2	OK	33.1613	33.5521	0.0169035	0.0399297	0.9523	0.99995	no

ENSG00000158985	ENSG00000158985	CDC42SE2	5:130581185-130734140	q1	q2	OK	19.7299	19.7205	-0.000687712	-0.00136379	0.998	0.99995	no

ENSG00000158987	ENSG00000158987	RAPGEF6	5:130759613-131132710	q1	q2	OK	6.60864	6.9752	0.0778803	0.0950247	0.85865	0.99995	no

ENSG00000159023	ENSG00000159023	EPB41	1:29213602-29450447	q1	q2	OK	40.4208	42.0722	0.0577681	0.128934	0.8446	0.99995	no

ENSG00000159055	ENSG00000159055	MIS18A	21:33640529-33653299	q1	q2	OK	29.2371	31.0375	0.0862141	0.180327	0.7819	0.99995	no

ENSG00000159063	ENSG00000159063	ALG8	11:77811981-77850706	q1	q2	OK	36.9297	38.9865	0.0781948	0.152192	0.814	0.99995	no

ENSG00000159069	ENSG00000159069	FBXW5	9:139834886-139839148	q1	q2	OK	32.3884	28.5127	-0.183877	-0.389765	0.55515	0.99995	no

ENSG00000159079	ENSG00000159079	C21orf59	21:33948861-33985176	q1	q2	OK	24.5749	25.1971	0.0360731	0.0725403	0.91825	0.99995	no

ENSG00000159082	ENSG00000159082	SYNJ1	21:34001068-34100359	q1	q2	OK	6.78812	7.22097	0.0891824	0.134177	0.80105	0.99995	no

ENSG00000159086	ENSG00000159086	PAXBP1	21:34100425-34144169	q1	q2	OK	47.3464	53.939	0.188075	0.388712	0.5535	0.99995	no

ENSG00000159110	ENSG00000159110	IFNAR2	21:34602205-34669539	q1	q2	OK	8.80704	9.37456	0.0900944	0.0991802	0.89	0.99995	no

ENSG00000159111	ENSG00000159111	MRPL10	17:45900637-45908900	q1	q2	OK	29.8175	27.7913	-0.101528	-0.212131	0.75235	0.99995	no

ENSG00000159123	ENSG00000159123	DMRTC1	X:72091858-72163653	q1	q2	NOTEST	0	0	0	0	1	1	no

ENSG00000159128	ENSG00000159128	IFNGR2	21:34775201-34852318	q1	q2	OK	0.823368	0.456275	-0.851635	-0.486488	0.40135	0.99995	no

ENSG00000159131	ENSG00000159131	GART	21:34876237-35349992	q1	q2	OK	40.3424	45.5347	0.17467	0.0696936	0.8558	0.99995	no

ENSG00000159140	ENSG00000159140	SON	21:34876237-35349992	q1	q2	OK	169.797	170.054	0.00218698	0.00137633	0.9964	0.99995	no

ENSG00000159147	ENSG00000159147	DONSON	21:34876237-35349992	q1	q2	OK	29.6878	30.7549	0.050945	0.0163944	0.94475	0.99995	no

ENSG00000159164	ENSG00000159164	SV2A	1:149874869-149889434	q1	q2	NOTEST	0.0999783	0.104555	0.0645709	0	1	1	no

ENSG00000159166	ENSG00000159166	LAD1	1:201328135-201368736	q1	q2	NOTEST	0	0.0115483	inf	0	1	1	no

ENSG00000159167	ENSG00000159167	STC1	8:23699427-23712320	q1	q2	OK	1.8839	1.52281	-0.306987	-0.415535	0.54135	0.99995	no

ENSG00000159173	ENSG00000159173	TNNI1	1:201373624-201398994	q1	q2	NOTEST	0.0468321	0.0642721	0.456693	0	1	1	no

ENSG00000159176	ENSG00000159176	CSRP1	1:201452657-201502736	q1	q2	OK	5.60837	5.66346	0.014103	0.0194092	0.976	0.99995	no

ENSG00000159182	ENSG00000159182	PRAC1	17:46799083-46799884	q1	q2	NOTEST	0	0	0	0	1	1	no

ENSG00000159184	ENSG00000159184	HOXB13	17:46800529-46806540	q1	q2	NOTEST	0	0	0	0	1	1	no

ENSG00000159186	ENSG00000159186	AC007383.6	2:207100959-207101595	q1	q2	NOTEST	0	0	0	0	1	1	no

ENSG00000159189	ENSG00000159189	C1QC	1:22970122-22974603	q1	q2	NOTEST	0	0	0	0	1	1	no

ENSG00000159197	ENSG00000159197	KCNE2	21:35736322-35747647	q1	q2	OK	1.05972	0.581917	-0.864795	-0.72873	0.28795	0.99995	no

ENSG00000159199	ENSG00000159199	ATP5G1	17:46952221-47006418	q1	q2	OK	63.2397	65.9859	0.0613281	0.0621504	0.92555	0.99995	no

ENSG00000159200	ENSG00000159200	RCAN1	21:35885439-35987441	q1	q2	OK	12.769	14.1841	0.151629	0.297796	0.65365	0.99995	no

ENSG00000159202	ENSG00000159202	UBE2Z	17:46952221-47006418	q1	q2	OK	36.6336	36.3567	-0.0109474	-0.0162701	0.9737	0.99995	no

ENSG00000159208	ENSG00000159208	C1orf51	1:150254952-150259505	q1	q2	OK	1.78921	1.27685	-0.486732	-0.373295	0.7126	0.99995	no

ENSG00000159210	ENSG00000159210	SNF8	17:47006677-47022479	q1	q2	OK	84.2247	83.3422	-0.0151956	-0.0294036	0.9672	0.99995	no

ENSG00000159212	ENSG00000159212	CLIC6	21:36041687-36090525	q1	q2	NOTEST	0.0613481	0.0929722	0.599782	0	1	1	no

ENSG00000159214	ENSG00000159214	CCDC24	1:44457030-44497139	q1	q2	OK	2.72513	2.54249	-0.100082	-0.0567912	0.916	0.99995	no

ENSG00000159216	ENSG00000159216	RUNX1	21:36160097-37376965	q1	q2	OK	0.502572	0.531494	0.0807243	0.0442168	0.8841	0.99995	no

ENSG00000159217	ENSG00000159217	IGF2BP1	17:47072627-47133012	q1	q2	OK	73.6609	83.6886	0.184132	0.362859	0.56445	0.99995	no

ENSG00000159224	ENSG00000159224	GIP	17:47035915-47045958	q1	q2	NOTEST	0	0	0	0	1	1	no

ENSG00000159228	ENSG00000159228	CBR1	21:37402367-37498938	q1	q2	OK	5.28884	5.17666	-0.0309319	-0.0238384	0.97065	0.99995	no

ENSG00000159231	ENSG00000159231	CBR3	21:37502668-37528615	q1	q2	OK	1.62507	1.18196	-0.459321	-0.203796	0.8161	0.99995	no

ENSG00000159239	ENSG00000159239	C2orf81	2:74641303-74648718	q1	q2	OK	2.6761	2.75427	0.0415358	0.0475401	0.9369	0.99995	no

ENSG00000159247	ENSG00000159247	TUBBP5	9:141044564-141071821	q1	q2	NOTEST	0.0309187	0.0397724	0.363285	0	1	1	no

ENSG00000159248	ENSG00000159248	GJD2	15:35043232-35047166	q1	q2	NOTEST	0.0186061	0.00917664	-1.01974	0	1	1	no

ENSG00000159251	ENSG00000159251	ACTC1	15:35047284-35105124	q1	q2	NOTEST	0.381371	0.403935	0.0829273	0	1	1	no

ENSG00000159256	ENSG00000159256	MORC3	21:37692089-37791313	q1	q2	OK	17.3454	21.0544	0.279572	0.376012	0.45365	0.99995	no

ENSG00000159259	ENSG00000159259	CHAF1B	21:37692089-37791313	q1	q2	OK	10.5301	10.6154	0.0116414	0.0155948	0.9786	0.99995	no

ENSG00000159261	ENSG00000159261	CLDN14	21:37802657-37948867	q1	q2	NOTEST	0	0	0	0	1	1	no

ENSG00000159263	ENSG00000159263	SIM2	21:38071072-38122218	q1	q2	OK	0.54	0.614916	0.187431	0.207054	0.7411	0.99995	no

ENSG00000159267	ENSG00000159267	HLCS	21:38123188-38362536	q1	q2	OK	8.59067	9.36483	0.124482	0.181614	0.71785	0.99995	no

ENSG00000159289	ENSG00000159289	GOLGA6A	15:74362197-74374891	q1	q2	NOTEST	0.0163788	0.03203	0.967594	0	1	1	no

ENSG00000159307	ENSG00000159307	SCUBE1	22:43593288-43739394	q1	q2	OK	2.46775	2.49937	0.0183699	0.0209831	0.9587	0.99995	no

ENSG00000159314	ENSG00000159314	ARHGAP27	17:43471274-43511787	q1	q2	NOTEST	0.307859	0.132431	-1.21703	0	1	1	no

ENSG00000159322	ENSG00000159322	ADPGK	15:73043709-73090540	q1	q2	OK	22.1217	25.1547	0.185366	0.366134	0.5531	0.99995	no

ENSG00000159335	ENSG00000159335	PTMS	12:6857169-6880116	q1	q2	OK	459.726	455.314	-0.0139128	-0.0253322	0.9711	0.99995	no

ENSG00000159337	ENSG00000159337	PLA2G4D	15:42359206-42386752	q1	q2	NOTEST	0.00761441	0	#NAME?	0	1	1	no

ENSG00000159339	ENSG00000159339	PADI4	1:17634689-17690499	q1	q2	NOTEST	0	0	0	0	1	1	no

ENSG00000159346	ENSG00000159346	ADIPOR1	1:202909950-202927700	q1	q2	OK	23.7822	25.537	0.102704	0.235567	0.74215	0.99995	no

ENSG00000159348	ENSG00000159348	CYB5R1	1:202930996-202936408	q1	q2	OK	13.9892	11.4076	-0.294326	-0.498459	0.46545	0.99995	no

ENSG00000159352	ENSG00000159352	PSMD4	1:151227178-151239955	q1	q2	OK	143.891	135.003	-0.0919841	-0.212753	0.73295	0.99995	no

ENSG00000159363	ENSG00000159363	ATP13A2	1:17300996-17338423	q1	q2	OK	15.8851	15.3707	-0.0474881	-0.064166	0.90855	0.99995	no

ENSG00000159374	ENSG00000159374	M1AP	2:74785009-74875465	q1	q2	NOTEST	0.0192139	0	#NAME?	0	1	1	no

ENSG00000159377	ENSG00000159377	PSMB4	1:151372009-151374420	q1	q2	OK	190.218	179.463	-0.083966	-0.2066	0.769	0.99995	no

ENSG00000159387	ENSG00000159387	IRX6	16:55293880-55367500	q1	q2	NOTEST	0.157931	0.0941912	-0.745627	0	1	1	no

ENSG00000159388	ENSG00000159388	BTG2	1:203274618-203278730	q1	q2	OK	19.8954	18.2125	-0.127508	-0.282193	0.683	0.99995	no

ENSG00000159398	ENSG00000159398	CES5A	16:55880065-55989943	q1	q2	NOTEST	0	0	0	0	1	1	no

ENSG00000159399	ENSG00000159399	HK2	2:75059781-75120486	q1	q2	OK	11.1556	12.2994	0.140817	0.297012	0.6422	0.99995	no

ENSG00000159403	ENSG00000159403	C1R	12:7187512-7275097	q1	q2	OK	2.94731	2.47258	-0.253381	-0.200902	0.7803	0.99995	no

ENSG00000159409	ENSG00000159409	CELF3	1:151673501-151702281	q1	q2	OK	21.4807	18.1256	-0.245008	-0.418582	0.49985	0.99995	no

ENSG00000159423	ENSG00000159423	ALDH4A1	1:19166092-19283180	q1	q2	OK	6.19248	5.50066	-0.170911	-0.210919	0.7462	0.99995	no

ENSG00000159433	ENSG00000159433	STARD9	15:42867856-43013179	q1	q2	OK	7.42561	7.17961	-0.0486047	-0.0816919	0.8812	0.99995	no

ENSG00000159445	ENSG00000159445	THEM4	1:151846059-151882284	q1	q2	OK	5.56326	6.45009	0.213389	0.28514	0.65075	0.99995	no

ENSG00000159450	ENSG00000159450	TCHH	1:152078792-152086556	q1	q2	NOTEST	0.127383	0.0618746	-1.04176	0	1	1	no

ENSG00000159455	ENSG00000159455	LCE2B	1:152647770-152659877	q1	q2	NOTEST	0	0	0	0	1	1	no

ENSG00000159459	ENSG00000159459	UBR1	15:43235094-43398311	q1	q2	OK	7.69104	8.40579	0.128205	0.171079	0.68615	0.99995	no

ENSG00000159461	ENSG00000159461	AMFR	16:56395363-56459450	q1	q2	OK	36.6889	32.3962	-0.179521	-0.337931	0.59485	0.99995	no

ENSG00000159479	ENSG00000159479	MED8	1:43849587-43855479	q1	q2	OK	22.2785	22.9895	0.0453245	0.0892584	0.8955	0.99995	no

ENSG00000159495	ENSG00000159495	TGM7	15:43568477-43594970	q1	q2	NOTEST	0	0	0	0	1	1	no

ENSG00000159496	ENSG00000159496	RGL4	22:23950638-24059543	q1	q2	NOTEST	0.287817	0.177765	-0.695177	0	1	1	no

ENSG00000159516	ENSG00000159516	SPRR2G	1:153122057-153123345	q1	q2	NOTEST	0	0	0	0	1	1	no

ENSG00000159527	ENSG00000159527	PGLYRP3	1:153270337-153283194	q1	q2	NOTEST	0	0	0	0	1	1	no

ENSG00000159556	ENSG00000159556	ISL2	15:76629064-76634817	q1	q2	NOTEST	0.188924	0.137399	-0.459435	0	1	1	no

ENSG00000159579	ENSG00000159579	RSPRY1	16:57220048-57274387	q1	q2	OK	27.8673	29.327	0.0736583	0.14063	0.8075	0.99995	no

ENSG00000159588	ENSG00000159588	CCDC17	1:46085715-46089729	q1	q2	OK	0.768725	0.787393	0.0346171	0.0227396	0.9626	0.99995	no

ENSG00000159592	ENSG00000159592	GPBP1L1	1:46092975-46216322	q1	q2	OK	45.7408	45.7105	-0.000955468	-0.00124058	0.99785	0.99995	no

ENSG00000159593	ENSG00000159593	NAE1	16:66836777-66907159	q1	q2	OK	66.6812	73.1634	0.133841	0.278215	0.62915	0.99995	no

ENSG00000159596	ENSG00000159596	TMEM69	1:46092975-46216322	q1	q2	OK	24.7606	23.9711	-0.0467532	-0.039463	0.95235	0.99995	no

ENSG00000159618	ENSG00000159618	GPR114	16:57576332-57625593	q1	q2	NOTEST	0.0175941	0.00502576	-1.80768	0	1	1	no

ENSG00000159625	ENSG00000159625	CCDC135	16:57702098-57765717	q1	q2	NOTEST	0.27955	0.232249	-0.267435	0	1	1	no

ENSG00000159640	ENSG00000159640	ACE	17:61554421-61599209	q1	q2	NOTEST	0.0552986	0.139849	1.33856	0	1	1	no

ENSG00000159648	ENSG00000159648	TEPP	16:58010338-58022020	q1	q2	NOTEST	0.0780967	0.172908	1.14667	0	1	1	no

ENSG00000159650	ENSG00000159650	UROC1	3:126200123-126236616	q1	q2	NOTEST	0.0192283	0	#NAME?	0	1	1	no

ENSG00000159658	ENSG00000159658	EFCAB14	1:47098408-47184824	q1	q2	OK	14.1159	16.7099	0.243387	0.192552	0.57075	0.99995	no

ENSG00000159674	ENSG00000159674	SPON2	4:1160719-1202750	q1	q2	OK	7.27124	5.52941	-0.395076	-0.469152	0.42375	0.99995	no

ENSG00000159685	ENSG00000159685	CHCHD6	3:126423062-126679249	q1	q2	OK	20.9363	21.3515	0.028329	0.0513147	0.9403	0.99995	no

ENSG00000159692	ENSG00000159692	CTBP1	4:1205235-1282079	q1	q2	OK	104.361	86.3303	-0.273645	-0.484447	0.4393	0.99995	no

ENSG00000159708	ENSG00000159708	LRRC36	16:67360700-67419106	q1	q2	NOTEST	0.11426	0	#NAME?	0	1	1	no

ENSG00000159712	ENSG00000159712	ANKRD18CP	9:99918851-100140806	q1	q2	NOTEST	0.0359937	0.00887663	-2.01966	0	1	1	no

ENSG00000159713	ENSG00000159713	TPPP3	16:67423711-67427438	q1	q2	OK	4.47418	4.59606	0.0387748	0.049215	0.947	0.99995	no

ENSG00000159714	ENSG00000159714	ZDHHC1	16:67428321-67450736	q1	q2	OK	1.88677	1.61665	-0.222909	-0.191548	0.77715	0.99995	no

ENSG00000159720	ENSG00000159720	ATP6V0D1	16:67471916-67515140	q1	q2	OK	37.6136	33.9035	-0.149818	-0.305523	0.6421	0.99995	no

ENSG00000159723	ENSG00000159723	AGRP	16:67515216-67521153	q1	q2	NOTEST	0	0.16963	inf	0	1	1	no

ENSG00000159733	ENSG00000159733	ZFYVE28	4:2271308-2420390	q1	q2	NOTEST	0.321188	0.368329	0.19758	0	1	1	no

ENSG00000159753	ENSG00000159753	RLTPR	16:67678821-67694713	q1	q2	OK	0.646742	0.40307	-0.68216	-0.15826	0.4493	0.99995	no

ENSG00000159761	ENSG00000159761	C16orf86	16:67696847-67702661	q1	q2	OK	2.13626	0.978434	-1.12654	-0.543244	0.355	0.99995	no

ENSG00000159763	ENSG00000159763	PIP	7:142829169-142836839	q1	q2	NOTEST	0	0	0	0	1	1	no

ENSG00000159784	ENSG00000159784	FAM131B	7:143050492-143059863	q1	q2	OK	15.9254	15.6577	-0.0244497	-0.0507061	0.93695	0.99995	no

ENSG00000159788	ENSG00000159788	RGS12	4:3294754-3441640	q1	q2	OK	37.4753	34.8016	-0.106787	-0.133294	0.78485	0.99995	no

ENSG00000159792	ENSG00000159792	PSKH1	16:67927174-67970990	q1	q2	OK	14.777	13.5614	-0.123847	-0.247584	0.72585	0.99995	no

ENSG00000159840	ENSG00000159840	ZYX	7:143078172-143220542	q1	q2	OK	82.0747	80.7532	-0.0234171	-0.0502139	0.93945	0.99995	no

ENSG00000159842	ENSG00000159842	ABR	17:900356-1132315	q1	q2	OK	24.2462	23.27	-0.0592859	-0.075155	0.8484	0.99995	no

ENSG00000159860	ENSG00000159860	FAM115D	7:143318042-143534026	q1	q2	NOTEST	0.0106833	0	#NAME?	0	1	1	no

ENSG00000159871	ENSG00000159871	LYPD5	19:44300080-44331358	q1	q2	NOTEST	0.0705609	0.08143	0.206692	0	1	1	no

ENSG00000159873	ENSG00000159873	CCDC117	22:29168661-29185283	q1	q2	OK	15.8249	16.1036	0.0251868	0.0535167	0.9374	0.99995	no

ENSG00000159882	ENSG00000159882	ZNF230	19:44507099-44518078	q1	q2	OK	3.0121	3.21688	0.0948938	0.116504	0.8349	0.99995	no

ENSG00000159884	ENSG00000159884	CCDC107	9:35658300-35681156	q1	q2	OK	16.4609	13.9099	-0.242937	-0.243307	0.7352	0.99995	no

ENSG00000159885	ENSG00000159885	ZNF222	19:44529493-44593766	q1	q2	OK	8.28472	6.93345	-0.256881	-0.291542	0.6736	0.99995	no

ENSG00000159899	ENSG00000159899	NPR2	9:35792150-35812269	q1	q2	OK	6.06479	4.86876	-0.316905	-0.324653	0.62415	0.99995	no

ENSG00000159904	ENSG00000159904	ZNF890P	7:5160869-5184177	q1	q2	NOTEST	0.153081	0.0741351	-1.04606	0	1	1	no

ENSG00000159905	ENSG00000159905	ZNF221	19:44455374-44471861	q1	q2	OK	2.14901	2.55125	0.247533	0.307361	0.6403	0.99995	no

ENSG00000159915	ENSG00000159915	ZNF233	19:44711699-44809199	q1	q2	OK	1.6562	1.47687	-0.165333	-0.0797015	0.8926	0.99995	no

ENSG00000159917	ENSG00000159917	ZNF235	19:44711699-44809199	q1	q2	OK	3.73105	5.32034	0.511938	0.465951	0.41785	0.99995	no

ENSG00000159921	ENSG00000159921	GNE	9:36190852-36304921	q1	q2	OK	9.26738	10.7789	0.217976	0.25604	0.7204	0.99995	no

ENSG00000159958	ENSG00000159958	TNFRSF13C	22:42321044-42322822	q1	q2	OK	1.03911	0.39493	-1.39568	-1.22811	0.0932	0.99995	no

ENSG00000159961	ENSG00000159961	OR3A3	17:3323861-3324827	q1	q2	NOTEST	0	0	0	0	1	1	no

ENSG00000160007	ENSG00000160007	ARHGAP35	19:47421932-47508334	q1	q2	OK	30.1956	29.3888	-0.0390732	-0.0572286	0.8918	0.99995	no

ENSG00000160013	ENSG00000160013	PTGIR	19:47123724-47128375	q1	q2	OK	1.26181	1.20951	-0.0610702	-0.0402895	0.93835	0.99995	no

ENSG00000160014	ENSG00000160014	CALM3	19:46984052-47114050	q1	q2	OK	233.338	224.163	-0.0578706	-0.122536	0.84515	0.99995	no

ENSG00000160049	ENSG00000160049	DFFA	1:10516578-10690815	q1	q2	OK	20.7702	20.2937	-0.0334883	-0.0529687	0.93565	0.99995	no

ENSG00000160050	ENSG00000160050	CCDC28B	1:32665986-32674288	q1	q2	OK	18.0216	16.4968	-0.127538	-0.183899	0.7985	0.99995	no

ENSG00000160051	ENSG00000160051	IQCC	1:32665986-32674288	q1	q2	OK	4.44981	4.37669	-0.0239025	-0.0266252	0.96985	0.99995	no

ENSG00000160055	ENSG00000160055	TMEM234	1:32674694-32697205	q1	q2	OK	9.57628	8.27456	-0.210783	-0.140351	0.82895	0.99995	no

ENSG00000160058	ENSG00000160058	BSDC1	1:32830703-32860332	q1	q2	OK	24.7961	21.067	-0.23513	-0.472867	0.42355	0.99995	no

ENSG00000160062	ENSG00000160062	ZBTB8A	1:32930657-33116504	q1	q2	OK	5.75031	6.7292	0.226797	0.280819	0.67835	0.99995	no

ENSG00000160072	ENSG00000160072	ATAD3B	1:1407142-1433228	q1	q2	OK	16.2785	15.5359	-0.0673639	-0.0829255	0.873	0.99995	no

ENSG00000160075	ENSG00000160075	SSU72	1:1477052-1510249	q1	q2	OK	77.1882	73.547	-0.0697146	-0.159576	0.8236	0.99995	no

ENSG00000160087	ENSG00000160087	UBE2J2	1:1189288-1209265	q1	q2	OK	44.4141	42.0234	-0.0798258	-0.153681	0.8102	0.99995	no

ENSG00000160094	ENSG00000160094	ZNF362	1:33722145-33766320	q1	q2	OK	28.1018	25.5792	-0.13569	-0.253852	0.67475	0.99995	no

ENSG00000160097	ENSG00000160097	FNDC5	1:33327868-33338083	q1	q2	OK	10.5898	8.88048	-0.253964	-0.391509	0.54885	0.99995	no

ENSG00000160111	ENSG00000160111	CPAMD8	19:17003757-17137625	q1	q2	OK	8.80149	7.50671	-0.229567	-0.304118	0.54865	0.99995	no

ENSG00000160113	ENSG00000160113	NR2F6	19:17342691-17398455	q1	q2	OK	26.0761	23.881	-0.126861	-0.210638	0.76745	0.99995	no

ENSG00000160117	ENSG00000160117	ANKLE1	19:17342691-17398455	q1	q2	OK	2.13166	1.64933	-0.370099	-0.114079	0.7907	0.99995	no

ENSG00000160124	ENSG00000160124	CCDC58	3:122078437-122102078	q1	q2	OK	38.3943	42.198	0.136281	0.21766	0.77025	0.99995	no

ENSG00000160131	ENSG00000160131	VMA21	X:150564986-150577836	q1	q2	OK	19.0273	20.8513	0.132063	0.303227	0.6528	0.99995	no

ENSG00000160145	ENSG00000160145	KALRN	3:123798869-124445172	q1	q2	OK	19.6639	21.2508	0.111969	0.142853	0.7536	0.99995	no

ENSG00000160161	ENSG00000160161	CILP2	19:19649056-19657468	q1	q2	NOTEST	0.327186	0.363626	0.152344	0	1	1	no

ENSG00000160172	ENSG00000160172	FAM86C2P	11:67552743-67572807	q1	q2	OK	2.01996	2.40818	0.253618	0.307435	0.6506	0.99995	no

ENSG00000160179	ENSG00000160179	ABCG1	21:43619798-43717354	q1	q2	OK	4.67152	4.30294	-0.118567	-0.193461	0.7757	0.99995	no

ENSG00000160180	ENSG00000160180	TFF3	21:43731776-43735761	q1	q2	OK	0.800088	1.10608	0.467226	0.326877	0.61465	0.99995	no

ENSG00000160181	ENSG00000160181	TFF2	21:43766465-43771237	q1	q2	NOTEST	0	0	0	0	1	1	no

ENSG00000160182	ENSG00000160182	TFF1	21:43782390-43786703	q1	q2	NOTEST	0	0	0	0	1	1	no

ENSG00000160183	ENSG00000160183	TMPRSS3	21:43791998-43816955	q1	q2	NOTEST	0.0778639	0.0630836	-0.303691	0	1	1	no

ENSG00000160185	ENSG00000160185	UBASH3A	21:43824007-43867791	q1	q2	NOTEST	0	0	0	0	1	1	no

ENSG00000160188	ENSG00000160188	RSPH1	21:43892595-44001550	q1	q2	NOTEST	0.442048	0.251865	-0.811555	0	1	1	no

ENSG00000160190	ENSG00000160190	SLC37A1	21:43892595-44001550	q1	q2	OK	3.90547	2.7641	-0.498684	-0.562729	0.24755	0.99995	no

ENSG00000160191	ENSG00000160191	PDE9A	21:44073745-44195619	q1	q2	OK	23.2712	22.0554	-0.0774125	-0.140609	0.81725	0.99995	no

ENSG00000160193	ENSG00000160193	WDR4	21:44263203-44299678	q1	q2	OK	7.21693	7.44818	0.0455031	0.0658433	0.9172	0.99995	no

ENSG00000160194	ENSG00000160194	NDUFV3	21:44299753-44333414	q1	q2	OK	14.724	14.2026	-0.0520097	-0.0941002	0.8919	0.99995	no

ENSG00000160199	ENSG00000160199	PKNOX1	21:44394619-44453691	q1	q2	OK	9.22695	7.56281	-0.286932	-0.345381	0.437	0.99995	no

ENSG00000160200	ENSG00000160200	CBS	21:44473300-44497053	q1	q2	OK	58.0674	56.7891	-0.0321129	-0.0640087	0.921	0.99995	no

ENSG00000160201	ENSG00000160201	U2AF1	21:44513065-44527697	q1	q2	OK	224.905	242.22	0.107001	0.234402	0.73275	0.99995	no

ENSG00000160202	ENSG00000160202	CRYAA	21:44589117-44592915	q1	q2	NOTEST	0.0252731	0.0249083	-0.0209794	0	1	1	no

ENSG00000160207	ENSG00000160207	HSF2BP	21:44949071-45079374	q1	q2	OK	0.811491	0.603056	-0.428284	-0.422605	0.5344	0.99995	no

ENSG00000160208	ENSG00000160208	RRP1B	21:45079428-45115958	q1	q2	OK	18.5084	20.0443	0.115012	0.279343	0.6921	0.99995	no

ENSG00000160209	ENSG00000160209	PDXK	21:45138974-45182188	q1	q2	OK	21.2808	18.235	-0.222847	-0.300474	0.63085	0.99995	no

ENSG00000160211	ENSG00000160211	G6PD	X:153759605-153796782	q1	q2	OK	5.03415	4.45703	-0.175667	-0.190976	0.76815	0.99995	no

ENSG00000160213	ENSG00000160213	CSTB	21:45192392-45196326	q1	q2	OK	26.1794	28.5765	0.1264	0.256379	0.6978	0.99995	no

ENSG00000160214	ENSG00000160214	RRP1	21:45209393-45225174	q1	q2	OK	13.4716	13.8275	0.0376144	0.0565949	0.93695	0.99995	no

ENSG00000160216	ENSG00000160216	AGPAT3	21:45285066-45406417	q1	q2	OK	11.7424	11.553	-0.0234613	-0.0372491	0.943	0.99995	no

ENSG00000160218	ENSG00000160218	TRAPPC10	21:45432199-45526433	q1	q2	OK	13.7277	15.1208	0.139437	0.196903	0.63385	0.99995	no

ENSG00000160219	ENSG00000160219	GAB3	X:153903528-153979858	q1	q2	NOTEST	0.0293887	0.0516203	0.812675	0	1	1	no

ENSG00000160221	ENSG00000160221	C21orf33	21:45553486-45565605	q1	q2	OK	44.6885	40.6441	-0.136858	-0.297008	0.6333	0.99995	no

ENSG00000160223	ENSG00000160223	ICOSLG	21:45642873-45660849	q1	q2	OK	1.40476	1.04137	-0.431836	-0.478988	0.4612	0.99995	no

ENSG00000160224	ENSG00000160224	AIRE	21:45705720-45718531	q1	q2	NOTEST	0	0.0060124	inf	0	1	1	no

ENSG00000160226	ENSG00000160226	C21orf2	21:45748826-45760353	q1	q2	OK	12.1908	10.0958	-0.272037	-0.33136	0.5712	0.99995	no

ENSG00000160229	ENSG00000160229	ZNF66	19:20959109-20991922	q1	q2	OK	1.0466	1.2019	0.199609	0.17483	0.78635	0.99995	no

ENSG00000160233	ENSG00000160233	LRRC3	21:45875368-45878739	q1	q2	OK	3.52139	3.41182	-0.0456028	-0.069943	0.92	0.99995	no

ENSG00000160255	ENSG00000160255	ITGB2	21:46305867-46351904	q1	q2	NOTEST	0.0835757	0.0806645	-0.0511491	0	1	1	no

ENSG00000160256	ENSG00000160256	FAM207A	21:46359924-46396904	q1	q2	OK	15.1224	13.0394	-0.213804	-0.339312	0.61995	0.99995	no

ENSG00000160271	ENSG00000160271	RALGDS	9:135973106-136039332	q1	q2	OK	33.6449	29.412	-0.193983	-0.265894	0.55655	0.99995	no

ENSG00000160282	ENSG00000160282	FTCD	21:47556175-47575481	q1	q2	NOTEST	0.165887	0.148012	-0.164478	0	1	1	no

ENSG00000160284	ENSG00000160284	SPATC1L	21:47581061-47604390	q1	q2	OK	2.4995	2.64269	0.0803707	0.0916517	0.8958	0.99995	no

ENSG00000160285	ENSG00000160285	LSS	21:47604992-47648738	q1	q2	OK	70.5008	81.6467	0.211754	0.232816	0.48135	0.99995	no

ENSG00000160293	ENSG00000160293	VAV2	9:136627015-136857726	q1	q2	OK	16.9964	16.2138	-0.068007	-0.147633	0.82555	0.99995	no

ENSG00000160294	ENSG00000160294	MCM3AP	21:47649130-47706211	q1	q2	OK	46.2223	50.7025	0.133467	0.142925	0.7441	0.99995	no

ENSG00000160298	ENSG00000160298	C21orf58	21:47720094-47743789	q1	q2	OK	21.5617	17.0731	-0.336747	-0.655582	0.32855	0.99995	no

ENSG00000160299	ENSG00000160299	PCNT	21:47744035-47865682	q1	q2	OK	27.5727	25.1549	-0.132399	-0.140687	0.83405	0.99995	no

ENSG00000160305	ENSG00000160305	DIP2A	21:47878811-47989926	q1	q2	OK	29.2178	28.7226	-0.0246634	-0.0534937	0.936	0.99995	no

ENSG00000160307	ENSG00000160307	S100B	21:48018874-48025121	q1	q2	OK	21.0229	18.6352	-0.173928	-0.282386	0.6874	0.99995	no

ENSG00000160310	ENSG00000160310	PRMT2	21:48055078-48085036	q1	q2	OK	69.439	63.6002	-0.126714	-0.2682	0.6915	0.99995	no

ENSG00000160318	ENSG00000160318	CLDND2	19:51848422-51872257	q1	q2	OK	0.438019	1.23023	1.48986	0.285358	0.40845	0.99995	no

ENSG00000160321	ENSG00000160321	ZNF208	19:22115759-22193751	q1	q2	NOTEST	0	0	0	0	1	1	no

ENSG00000160323	ENSG00000160323	ADAMTS13	9:136243116-136324508	q1	q2	OK	0.500086	0.243411	-1.03878	-0.491152	0.41105	0.99995	no

ENSG00000160325	ENSG00000160325	CACFD1	9:136325088-136335970	q1	q2	NOTEST	0	0	0	0	1	1	no

ENSG00000160326	ENSG00000160326	SLC2A6	9:136336216-136344259	q1	q2	NOTEST	0	0	0	0	1	1	no

ENSG00000160336	ENSG00000160336	ZNF761	19:53935226-53961515	q1	q2	OK	7.05527	7.43355	0.0753491	0.130088	0.8445	0.99995	no

ENSG00000160339	ENSG00000160339	FCN2	9:137772653-137779924	q1	q2	NOTEST	0	0	0	0	1	1	no

ENSG00000160345	ENSG00000160345	C9orf116	9:138387026-138398293	q1	q2	OK	13.1414	11.5878	-0.181508	-0.153121	0.8254	0.99995	no

ENSG00000160349	ENSG00000160349	LCN1	9:138413283-138418378	q1	q2	NOTEST	0	0	0	0	1	1	no

ENSG00000160352	ENSG00000160352	ZNF714	19:21264964-21308073	q1	q2	OK	19.2298	21.9256	0.189276	0.278015	0.6151	0.99995	no

ENSG00000160360	ENSG00000160360	GPSM1	9:139221931-139268133	q1	q2	OK	18.7832	17.6441	-0.0902596	-0.177055	0.8038	0.99995	no

ENSG00000160392	ENSG00000160392	C19orf47	19:40825442-40896094	q1	q2	OK	7.57284	6.73703	-0.168722	-0.0724546	0.90415	0.99995	no

ENSG00000160396	ENSG00000160396	HIPK4	19:40825442-40896094	q1	q2	NOTEST	0.150956	0.106587	-0.502103	0	1	1	no

ENSG00000160401	ENSG00000160401	C9orf117	9:130374543-130497604	q1	q2	OK	0.738474	0.486616	-0.601765	-0.168127	0.5743	0.99995	no

ENSG00000160404	ENSG00000160404	TOR2A	9:130374543-130497604	q1	q2	OK	15.1695	12.114	-0.324495	-0.360993	0.5877	0.99995	no

ENSG00000160408	ENSG00000160408	ST6GALNAC6	9:130628758-130667687	q1	q2	OK	13.756	12.8691	-0.096148	-0.141641	0.8109	0.99995	no

ENSG00000160410	ENSG00000160410	SHKBP1	19:41082756-41097305	q1	q2	OK	25.3342	22.9311	-0.143783	-0.144593	0.66245	0.99995	no

ENSG00000160439	ENSG00000160439	RDH13	19:55517560-55582659	q1	q2	NOTEST	0	0	0	0	1	1	no

ENSG00000160445	ENSG00000160445	ZER1	9:131486723-131534693	q1	q2	OK	9.34912	7.65969	-0.287546	-0.492749	0.4068	0.99995	no

ENSG00000160446	ENSG00000160446	ZDHHC12	9:131464801-131486406	q1	q2	OK	4.60594	4.64012	0.0106662	0.00809253	0.9903	0.99995	no

ENSG00000160447	ENSG00000160447	PKN3	9:131464801-131486406	q1	q2	OK	7.25143	6.47525	-0.163329	-0.262718	0.68115	0.99995	no

ENSG00000160460	ENSG00000160460	SPTBN4	19:40972147-41082370	q1	q2	OK	0.891841	0.458877	-0.95868	-0.631883	0.2357	0.99995	no

ENSG00000160469	ENSG00000160469	BRSK1	19:55793439-55823901	q1	q2	OK	15.4439	12.6153	-0.291867	-0.43902	0.4645	0.99995	no

ENSG00000160471	ENSG00000160471	COX6B2	19:55860673-55874628	q1	q2	NOTEST	0.224395	0.222494	-0.0122745	0	1	1	no

ENSG00000160472	ENSG00000160472	TMEM190	19:55888203-55889612	q1	q2	NOTEST	0	0	0	0	1	1	no

ENSG00000160505	ENSG00000160505	NLRP4	19:56296769-56393220	q1	q2	NOTEST	0	0	0	0	1	1	no

ENSG00000160539	ENSG00000160539	PPAPDC3	9:134165080-134184649	q1	q2	OK	1.66647	1.14903	-0.536387	-0.590469	0.3833	0.99995	no

ENSG00000160551	ENSG00000160551	TAOK1	17:27679086-27878922	q1	q2	OK	22.3529	24.873	0.154116	0.255617	0.59285	0.99995	no

ENSG00000160563	ENSG00000160563	MED27	9:134735493-134955295	q1	q2	OK	22.5152	19.9544	-0.174189	-0.351275	0.60775	0.99995	no

ENSG00000160570	ENSG00000160570	DEDD2	19:42702749-42724292	q1	q2	OK	12.807	9.3203	-0.458485	-0.742606	0.24505	0.99995	no

ENSG00000160584	ENSG00000160584	SIK3	11:116706466-116969153	q1	q2	OK	9.45085	9.58229	0.0199267	0.0362343	0.95005	0.99995	no

ENSG00000160588	ENSG00000160588	MPZL3	11:118097408-118123065	q1	q2	NOTEST	0.213863	0.286222	0.420443	0	1	1	no

ENSG00000160593	ENSG00000160593	AMICA1	11:118064454-118095809	q1	q2	NOTEST	0.152916	0	#NAME?	0	1	1	no

ENSG00000160602	ENSG00000160602	NEK8	17:27046410-27070473	q1	q2	OK	1.83706	1.97653	0.105577	0.0211445	0.91965	0.99995	no

ENSG00000160606	ENSG00000160606	TLCD1	17:27046410-27070473	q1	q2	OK	3.49657	2.65694	-0.396177	-0.0556807	0.77395	0.99995	no

ENSG00000160613	ENSG00000160613	PCSK7	11:117070036-117103241	q1	q2	OK	14.7507	14.6013	-0.0146869	-0.0112084	0.98085	0.99995	no

ENSG00000160633	ENSG00000160633	SAFB	19:5587009-5668489	q1	q2	OK	85.6134	80.0455	-0.0970161	-0.146552	0.76885	0.99995	no

ENSG00000160654	ENSG00000160654	CD3G	11:118215058-118225876	q1	q2	NOTEST	0	0	0	0	1	1	no

ENSG00000160678	ENSG00000160678	S100A1	1:153591262-153618782	q1	q2	OK	0.765867	0.907271	0.244441	0.0338054	0.8484	0.99995	no

ENSG00000160679	ENSG00000160679	CHTOP	1:153591262-153618782	q1	q2	OK	104.927	101.876	-0.0425767	-0.0951508	0.8909	0.99995	no

ENSG00000160683	ENSG00000160683	CXCR5	11:118754474-118829269	q1	q2	OK	0.641757	0.247297	-1.37578	-0.495343	0.6651	0.99995	no

ENSG00000160685	ENSG00000160685	ZBTB7B	1:154975126-155006257	q1	q2	OK	2.53866	1.83583	-0.467629	-0.486375	0.37395	0.99995	no

ENSG00000160688	ENSG00000160688	FLAD1	1:154955813-154965587	q1	q2	OK	23.2078	20.7962	-0.158293	-0.271702	0.6467	0.99995	no

ENSG00000160691	ENSG00000160691	SHC1	1:154929501-154946871	q1	q2	OK	37.7467	35.4259	-0.0915445	-0.115576	0.85365	0.99995	no

ENSG00000160695	ENSG00000160695	VPS11	11:118938084-118952688	q1	q2	NOTEST	0	0.00760694	inf	0	1	1	no

ENSG00000160703	ENSG00000160703	NLRX1	11:119037276-119054725	q1	q2	OK	1.86762	1.47444	-0.341029	-0.330253	0.493	0.99995	no

ENSG00000160710	ENSG00000160710	ADAR	1:154554537-154600475	q1	q2	OK	68.0879	69.4177	0.0279046	0.0376663	0.93175	0.99995	no

ENSG00000160712	ENSG00000160712	IL6R	1:154374803-154441926	q1	q2	NOTEST	0.37116	0.233973	-0.665697	0	1	1	no

ENSG00000160714	ENSG00000160714	UBE2Q1	1:154521052-154531504	q1	q2	OK	30.8895	32.1728	0.0587256	0.0940727	0.84075	0.99995	no

ENSG00000160716	ENSG00000160716	CHRNB2	1:154540256-154552502	q1	q2	OK	2.9965	3.03278	0.0173625	0.031459	0.96385	0.99995	no

ENSG00000160741	ENSG00000160741	CRTC2	1:153920144-153931101	q1	q2	OK	16.0145	13.4714	-0.249483	-0.392278	0.54925	0.99995	no

ENSG00000160746	ENSG00000160746	ANO10	3:43328003-43775863	q1	q2	OK	12.0479	12.9596	0.105248	0.125328	0.8273	0.99995	no

ENSG00000160752	ENSG00000160752	FDPS	1:155278538-155300905	q1	q2	OK	316.157	350.806	0.150032	0.29421	0.67595	0.99995	no

ENSG00000160753	ENSG00000160753	RUSC1	1:155278538-155300905	q1	q2	OK	17.0736	17.3724	0.0250287	0.0165638	0.9778	0.99995	no

ENSG00000160766	ENSG00000160766	GBAP1	1:155183615-155197214	q1	q2	OK	5.20042	4.62059	-0.170553	-0.244241	0.7217	0.99995	no

ENSG00000160767	ENSG00000160767	FAM189B	1:155216995-155225274	q1	q2	NOTEST	0	0	0	0	1	1	no

ENSG00000160781	ENSG00000160781	PAQR6	1:156213205-156217881	q1	q2	OK	9.94207	7.57126	-0.393013	-0.669362	0.32755	0.99995	no

ENSG00000160783	ENSG00000160783	PMF1	1:156182772-156213123	q1	q2	OK	59.4844	55.9985	-0.0871234	-0.189927	0.78455	0.99995	no

ENSG00000160785	ENSG00000160785	SLC25A44	1:156163879-156182587	q1	q2	OK	12.492	12.2369	-0.0297567	-0.0414211	0.9316	0.99995	no

ENSG00000160789	ENSG00000160789	LMNA	1:156052363-156109880	q1	q2	OK	50.4814	46.3051	-0.124583	-0.238339	0.70245	0.99995	no

ENSG00000160791	ENSG00000160791	CCR5	3:46406445-46448550	q1	q2	NOTEST	0	0	0	0	1	1	no

ENSG00000160796	ENSG00000160796	NBEAL2	3:46963215-47051193	q1	q2	OK	6.25568	5.90543	-0.083124	-0.0687169	0.9045	0.99995	no

ENSG00000160799	ENSG00000160799	CCDC12	3:46963215-47051193	q1	q2	OK	37.9246	33.6939	-0.170648	-0.263511	0.6866	0.99995	no

ENSG00000160801	ENSG00000160801	PTH1R	3:46899361-46945287	q1	q2	NOTEST	0.435232	0.331881	-0.391119	0	1	1	no

ENSG00000160803	ENSG00000160803	UBQLN4	1:156005091-156023585	q1	q2	OK	33.8291	33.6685	-0.00686628	-0.0128087	0.98125	0.99995	no

ENSG00000160808	ENSG00000160808	MYL3	3:46899361-46945287	q1	q2	NOTEST	0.123487	0	#NAME?	0	1	1	no

ENSG00000160813	ENSG00000160813	PPP1R35	7:100032879-100036127	q1	q2	OK	13.1255	13.5117	0.0418398	0.060129	0.9331	0.99995	no

ENSG00000160818	ENSG00000160818	GPATCH4	1:156564278-156571288	q1	q2	OK	22.409	25.4838	0.185505	0.336588	0.55045	0.99995	no

ENSG00000160828	ENSG00000160828	STAG3L2	7:74071993-74306731	q1	q2	OK	2.05783	2.4522	0.252956	0.160816	0.7926	0.99995	no

ENSG00000160838	ENSG00000160838	LRRC71	1:156890441-156902886	q1	q2	NOTEST	0.23002	0.115184	-0.997817	0	1	1	no

ENSG00000160844	ENSG00000160844	GATS	7:99775185-99869855	q1	q2	OK	0.600385	0.567892	-0.0802716	-0.0299295	0.89825	0.99995	no

ENSG00000160856	ENSG00000160856	FCRL3	1:157644110-157670647	q1	q2	NOTEST	0	0.0054561	inf	0	1	1	no

ENSG00000160862	ENSG00000160862	AZGP1	7:99564342-99573780	q1	q2	NOTEST	0	0	0	0	1	1	no

ENSG00000160867	ENSG00000160867	FGFR4	5:176513886-176525145	q1	q2	OK	1.74805	1.5909	-0.135903	-0.119268	0.78715	0.99995	no

ENSG00000160868	ENSG00000160868	CYP3A4	7:99354603-99381888	q1	q2	NOTEST	0.0242995	0.0108637	-1.16141	0	1	1	no

ENSG00000160870	ENSG00000160870	CYP3A7	7:99302659-99332819	q1	q2	NOTEST	0.126223	0.159366	0.336368	0	1	1	no

ENSG00000160877	ENSG00000160877	NACC1	19:13228916-13251961	q1	q2	OK	22.6431	23.3019	0.0413726	0.0753706	0.8881	0.99995	no

ENSG00000160882	ENSG00000160882	CYP11B1	8:143915553-143999259	q1	q2	NOTEST	0	0	0	0	1	1	no

ENSG00000160883	ENSG00000160883	HK3	5:176237477-176326333	q1	q2	NOTEST	0.184197	0.013792	-3.73935	0	1	1	no

ENSG00000160886	ENSG00000160886	LY6K	8:143781528-143808391	q1	q2	NOTEST	0	0	0	0	1	1	no

ENSG00000160888	ENSG00000160888	IER2	19:13261228-13265722	q1	q2	OK	28.6675	32.9458	0.200675	0.44244	0.53575	0.99995	no

ENSG00000160908	ENSG00000160908	ZNF394	7:99070463-99101273	q1	q2	OK	8.20246	6.71349	-0.288994	-0.35699	0.60245	0.99995	no

ENSG00000160917	ENSG00000160917	CPSF4	7:98923520-99063954	q1	q2	OK	45.4574	41.4828	-0.132002	-0.0885727	0.88155	0.99995	no

ENSG00000160932	ENSG00000160932	LY6E	8:144063154-144105249	q1	q2	OK	13.2814	11.5439	-0.202286	-0.330514	0.6192	0.99995	no

ENSG00000160948	ENSG00000160948	VPS28	8:145648999-145653931	q1	q2	OK	21.7806	19.1889	-0.182778	-0.306372	0.6744	0.99995	no

ENSG00000160949	ENSG00000160949	TONSL	8:145654164-145669827	q1	q2	OK	6.99005	7.07424	0.0172714	0.0353136	0.95815	0.99995	no

ENSG00000160951	ENSG00000160951	PTGER1	19:14583277-14586174	q1	q2	NOTEST	0.0593776	0.0390814	-0.603436	0	1	1	no

ENSG00000160953	ENSG00000160953	MUM1	19:1285889-1378430	q1	q2	OK	37.2596	36.3179	-0.0369319	-0.0579694	0.9196	0.99995	no

ENSG00000160957	ENSG00000160957	RECQL4	8:145736666-145743229	q1	q2	OK	25.4417	22.8187	-0.156982	-0.226239	0.713	0.99995	no

ENSG00000160959	ENSG00000160959	LRRC14	8:145743375-145754516	q1	q2	OK	13.81	12.2219	-0.176249	-0.244304	0.64385	0.99995	no

ENSG00000160961	ENSG00000160961	ZNF333	19:14729928-14889353	q1	q2	OK	14.382	13.9223	-0.0468676	-0.0630669	0.91885	0.99995	no

ENSG00000160963	ENSG00000160963	COL26A1	7:101006100-101202304	q1	q2	OK	53.3851	45.9802	-0.215426	-0.458642	0.47675	0.99995	no

ENSG00000160972	ENSG00000160972	PPP1R16A	8:145703351-145732557	q1	q2	OK	16.4961	13.6414	-0.274134	-0.40233	0.4889	0.99995	no

ENSG00000160973	ENSG00000160973	FOXH1	8:145691425-145701718	q1	q2	NOTEST	0.11307	0.16413	0.537631	0	1	1	no

ENSG00000160991	ENSG00000160991	ORAI2	7:102067264-102105323	q1	q2	OK	20.8018	18.5247	-0.167259	-0.246304	0.6833	0.99995	no

ENSG00000160993	ENSG00000160993	ALKBH4	7:102067264-102105323	q1	q2	OK	8.30128	7.29424	-0.186576	-0.16235	0.81935	0.99995	no

ENSG00000160994	ENSG00000160994	CCDC105	19:15060845-15134081	q1	q2	NOTEST	0	0	0	0	1	1	no

ENSG00000160999	ENSG00000160999	SH2B2	7:101928404-101962178	q1	q2	NOTEST	0.114122	0.05619	-1.0222	0	1	1	no

ENSG00000161010	ENSG00000161010	C5orf45	5:179224596-179334859	q1	q2	OK	21.0445	20.3949	-0.0452339	-0.0293314	0.9413	0.99995	no

ENSG00000161011	ENSG00000161011	SQSTM1	5:179224596-179334859	q1	q2	OK	46.3225	36.0956	-0.359893	-0.388482	0.57285	0.99995	no

ENSG00000161013	ENSG00000161013	MGAT4B	5:179224596-179334859	q1	q2	OK	48.3397	40.9708	-0.238613	-0.218447	0.6453	0.99995	no

ENSG00000161016	ENSG00000161016	RPL8	8:146015149-146017972	q1	q2	OK	805.645	712.528	-0.177199	-0.383763	0.58035	0.99995	no

ENSG00000161021	ENSG00000161021	MAML1	5:179159850-179223648	q1	q2	OK	22.2214	21.6696	-0.0362781	-0.0866942	0.89795	0.99995	no

ENSG00000161031	ENSG00000161031	PGLYRP2	19:15579455-15609767	q1	q2	NOTEST	0	0	0	0	1	1	no

ENSG00000161036	ENSG00000161036	LRWD1	7:102105375-102119354	q1	q2	OK	20.1183	19.9536	-0.0118572	-0.0201616	0.976	0.99995	no

ENSG00000161040	ENSG00000161040	FBXL13	7:102453307-102715286	q1	q2	OK	0.700991	0.946232	0.432797	0.080332	0.64905	0.99995	no

ENSG00000161048	ENSG00000161048	NAPEPLD	7:102740222-102790007	q1	q2	OK	7.6083	8.49139	0.158426	0.28425	0.68045	0.99995	no

ENSG00000161055	ENSG00000161055	SCGB3A1	5:180017102-180018540	q1	q2	NOTEST	0	0	0	0	1	1	no

ENSG00000161057	ENSG00000161057	PSMC2	7:102937868-103629963	q1	q2	OK	79.6022	77.61	-0.0365653	-0.0594919	0.92875	0.99995	no

ENSG00000161082	ENSG00000161082	CELF5	19:3224700-3303001	q1	q2	OK	10.5447	9.2729	-0.185426	-0.335987	0.5883	0.99995	no

ENSG00000161091	ENSG00000161091	MFSD12	19:3506270-3579086	q1	q2	OK	20.0908	19.7399	-0.0254212	-0.0210755	0.9735	0.99995	no

ENSG00000161103	ENSG00000161103	AC008132.13	22:18834323-18848562	q1	q2	NOTEST	0	0.0123409	inf	0	1	1	no

ENSG00000161132	ENSG00000161132	XXbac-B444P24.10	22:20287282-20300739	q1	q2	NOTEST	0	0.0458997	inf	0	1	1	no

ENSG00000161133	ENSG00000161133	USP41	22:20692437-20745048	q1	q2	NOTEST	0.100739	0.0341519	-1.56058	0	1	1	no

ENSG00000161149	ENSG00000161149	TUBA3FP	22:21353392-21399306	q1	q2	OK	2.16607	2.33511	0.108413	0.0763657	0.92535	0.99995	no

ENSG00000161179	ENSG00000161179	YDJC	22:21982377-21984353	q1	q2	OK	6.46968	6.26308	-0.0468227	-0.0697887	0.92105	0.99995	no

ENSG00000161180	ENSG00000161180	CCDC116	22:21987004-21991616	q1	q2	OK	0.438682	0.511086	0.22039	0.189691	0.76055	0.99995	no

ENSG00000161202	ENSG00000161202	DVL3	3:183851806-184402546	q1	q2	OK	37.8904	34.9206	-0.117754	-0.0450967	0.90505	0.99995	no

ENSG00000161203	ENSG00000161203	AP2M1	3:183851806-184402546	q1	q2	OK	161.176	152.41	-0.0806768	-0.0525759	0.9191	0.99995	no

ENSG00000161204	ENSG00000161204	ABCF3	3:183851806-184402546	q1	q2	OK	25.354	22.0404	-0.202058	-0.0538968	0.8	0.99995	no

ENSG00000161217	ENSG00000161217	PCYT1A	3:195938357-196065374	q1	q2	OK	15.589	16.3507	0.0688227	0.0798071	0.84515	0.99995	no

ENSG00000161243	ENSG00000161243	FBXO27	19:39481353-39523425	q1	q2	NOTEST	0.440837	0.377094	-0.225322	0	1	1	no

ENSG00000161249	ENSG00000161249	DMKN	19:35988121-36004560	q1	q2	NOTEST	0.0176001	0	#NAME?	0	1	1	no

ENSG00000161265	ENSG00000161265	U2AF1L4	19:36230057-36245420	q1	q2	OK	9.66618	8.69829	-0.152214	-0.119476	0.8394	0.99995	no

ENSG00000161267	ENSG00000161267	BDH1	3:197236653-197300194	q1	q2	OK	1.42027	1.36506	-0.0571992	-0.0433727	0.9189	0.99995	no

ENSG00000161270	ENSG00000161270	NPHS1	19:36316865-36370693	q1	q2	NOTEST	0.401858	0.321558	-0.321607	0	1	1	no

ENSG00000161277	ENSG00000161277	THAP8	19:36505409-36545664	q1	q2	OK	5.0523	5.11446	0.0176415	0.0099052	0.9856	0.99995	no

ENSG00000161281	ENSG00000161281	COX7A1	19:36641823-36643771	q1	q2	NOTEST	0.086871	0.0414229	-1.06845	0	1	1	no

ENSG00000161298	ENSG00000161298	ZNF382	19:37001596-37119499	q1	q2	OK	5.82701	5.85473	0.00684692	0.0028058	0.9956	0.99995	no

ENSG00000161326	ENSG00000161326	DUSP14	17:35849936-35873603	q1	q2	OK	19.3466	17.8467	-0.116422	-0.232697	0.7456	0.99995	no

ENSG00000161328	ENSG00000161328	LRRC56	11:537526-564021	q1	q2	OK	1.00566	0.907871	-0.14759	-0.117054	0.86645	0.99995	no

ENSG00000161381	ENSG00000161381	PLXDC1	17:37213271-37310647	q1	q2	OK	3.32168	4.13792	0.316994	0.252717	0.5498	0.99995	no

ENSG00000161395	ENSG00000161395	PGAP3	17:37827374-37887040	q1	q2	OK	8.37328	6.70918	-0.319657	-0.216859	0.7458	0.99995	no

ENSG00000161405	ENSG00000161405	IKZF3	17:37921197-38020441	q1	q2	NOTEST	0.0226824	0.0199482	-0.185315	0	1	1	no

ENSG00000161509	ENSG00000161509	GRIN2C	17:72838161-72857627	q1	q2	NOTEST	0.191322	0.121543	-0.654539	0	1	1	no

ENSG00000161513	ENSG00000161513	FDXR	17:72858618-72869156	q1	q2	OK	11.4967	8.79884	-0.385829	-0.588163	0.33785	0.99995	no

ENSG00000161526	ENSG00000161526	SAP30BP	17:73584138-73704142	q1	q2	OK	49.3386	46.2164	-0.0943122	-0.152768	0.8038	0.99995	no

ENSG00000161533	ENSG00000161533	ACOX1	17:73937587-74023533	q1	q2	OK	9.03243	8.09469	-0.158139	-0.115657	0.71925	0.99995	no

ENSG00000161542	ENSG00000161542	PRPSAP1	17:74305566-74383941	q1	q2	OK	44.201	41.2174	-0.100828	-0.170634	0.7777	0.99995	no

ENSG00000161544	ENSG00000161544	CYGB	17:74523437-74553765	q1	q2	OK	0.618963	0.517889	-0.25721	-0.190199	0.73415	0.99995	no

ENSG00000161547	ENSG00000161547	SRSF2	17:74722911-74777531	q1	q2	OK	239.217	251.851	0.0742544	0.153783	0.8178	0.99995	no

ENSG00000161551	ENSG00000161551	ZNF577	19:52359054-52408293	q1	q2	OK	9.08452	8.02066	-0.179688	-0.125504	0.7768	0.99995	no

ENSG00000161558	ENSG00000161558	TMEM143	19:48835612-48867494	q1	q2	OK	4.94699	3.45073	-0.51965	-0.559959	0.30575	0.99995	no

ENSG00000161570	ENSG00000161570	CCL5	17:34195970-34212867	q1	q2	NOTEST	0	0	0	0	1	1	no

ENSG00000161572	ENSG00000161572	LYZL6	17:34261521-34270703	q1	q2	NOTEST	0	0	0	0	1	1	no

ENSG00000161573	ENSG00000161573	CCL16	17:34303528-34308532	q1	q2	NOTEST	0	0	0	0	1	1	no

ENSG00000161574	ENSG00000161574	CCL15-CCL14	17:34310326-34338658	q1	q2	NOTEST	0	0	0	0	1	1	no

ENSG00000161583	ENSG00000161583	TBC1D3G	17:34580901-34808104	q1	q2	NOTEST	0.181416	0.0244962	-2.88867	0	1	1	no

ENSG00000161594	ENSG00000161594	KLHL10	17:39981334-40004636	q1	q2	NOTEST	0	0	0	0	1	1	no

ENSG00000161609	ENSG00000161609	CCDC155	19:49891474-49921251	q1	q2	NOTEST	0.114461	0.130046	0.184164	0	1	1	no

ENSG00000161610	ENSG00000161610	HCRT	17:40336077-40337470	q1	q2	NOTEST	0.388744	0.0621895	-2.64408	0	1	1	no

ENSG00000161618	ENSG00000161618	ALDH16A1	19:49949554-49995565	q1	q2	OK	13.7444	11.6976	-0.232623	-0.104899	0.86045	0.99995	no

ENSG00000161634	ENSG00000161634	DCD	12:55038374-55042277	q1	q2	NOTEST	0	0	0	0	1	1	no

ENSG00000161638	ENSG00000161638	ITGA5	12:54747444-54891472	q1	q2	OK	33.0169	34.8251	0.0769201	0.10707	0.84035	0.99995	no

ENSG00000161640	ENSG00000161640	SIGLEC11	19:50392910-50464429	q1	q2	NOTEST	0.0109782	0.0371875	1.76017	0	1	1	no

ENSG00000161642	ENSG00000161642	ZNF385A	12:54747444-54891472	q1	q2	OK	14.1716	12.7938	-0.147561	-0.123154	0.8519	0.99995	no

ENSG00000161643	ENSG00000161643	SIGLEC16	19:50472856-50479071	q1	q2	NOTEST	0.0946855	0.0619118	-0.612928	0	1	1	no

ENSG00000161647	ENSG00000161647	MPP3	17:41878166-41910538	q1	q2	OK	4.07017	4.14585	0.026579	0.0204892	0.9673	0.99995	no

ENSG00000161649	ENSG00000161649	CD300LG	17:41924515-41940997	q1	q2	NOTEST	0	0	0	0	1	1	no

ENSG00000161652	ENSG00000161652	IZUMO2	19:50655804-50666452	q1	q2	NOTEST	0	0	0	0	1	1	no

ENSG00000161653	ENSG00000161653	NAGS	17:42081913-42086431	q1	q2	OK	0.650272	0.575084	-0.17727	-0.18265	0.7855	0.99995	no

ENSG00000161654	ENSG00000161654	LSM12	17:42112002-42144987	q1	q2	OK	17.9694	18.0209	0.00412451	0.0055081	0.99435	0.99995	no

ENSG00000161664	ENSG00000161664	ASB16	17:42247814-42264085	q1	q2	OK	1.36448	1.72366	0.337126	0.259121	0.70395	0.99995	no

ENSG00000161671	ENSG00000161671	EMC10	19:50970041-50986608	q1	q2	OK	31.9195	28.2375	-0.176827	-0.39041	0.57435	0.99995	no

ENSG00000161677	ENSG00000161677	JOSD2	19:51009254-51014610	q1	q2	OK	6.63777	6.41835	-0.0484962	-0.0517576	0.9392	0.99995	no

ENSG00000161681	ENSG00000161681	SHANK1	19:51124563-51222707	q1	q2	OK	3.20905	3.89665	0.280087	0.179368	0.6674	0.99995	no

ENSG00000161682	ENSG00000161682	FAM171A2	17:42430582-42441243	q1	q2	OK	18.0757	15.5663	-0.21562	-0.40401	0.5215	0.99995	no

ENSG00000161692	ENSG00000161692	DBF4B	17:42785975-42829632	q1	q2	OK	19.1957	18.2319	-0.0743128	-0.136115	0.82575	0.99995	no

ENSG00000161714	ENSG00000161714	PLCD3	17:43100707-43221548	q1	q2	OK	6.48723	6.77391	0.062385	0.0275591	0.9381	0.99995	no

ENSG00000161791	ENSG00000161791	FMNL3	12:49962000-50158717	q1	q2	OK	10.1622	10.8	0.08782	0.0477812	0.92665	0.99995	no

ENSG00000161798	ENSG00000161798	AQP5	12:50344523-50359464	q1	q2	NOTEST	0.137074	0.170059	0.311078	0	1	1	no

ENSG00000161800	ENSG00000161800	RACGAP1	12:50360976-50426919	q1	q2	OK	51.6645	56.5314	0.129877	0.229962	0.6338	0.99995	no

ENSG00000161807	ENSG00000161807	OR7G1	19:9225273-9226439	q1	q2	NOTEST	0	0	0	0	1	1	no

ENSG00000161813	ENSG00000161813	LARP4	12:50720012-50873787	q1	q2	OK	16.6644	18.8541	0.178108	0.321493	0.5238	0.99995	no

ENSG00000161835	ENSG00000161835	GRASP	12:52400723-52409673	q1	q2	OK	7.31342	7.15367	-0.0318628	-0.0364942	0.95365	0.99995	no

ENSG00000161847	ENSG00000161847	RAVER1	19:10415478-10444316	q1	q2	OK	19.2908	17.2126	-0.164451	-0.294019	0.64955	0.99995	no

ENSG00000161849	ENSG00000161849	KRT84	12:52771595-52800139	q1	q2	NOTEST	0	0	0	0	1	1	no

ENSG00000161850	ENSG00000161850	KRT82	12:52771595-52800139	q1	q2	NOTEST	0	0	0	0	1	1	no

ENSG00000161860	ENSG00000161860	SYCE2	19:13001839-13030090	q1	q2	OK	2.67086	1.85252	-0.527818	-0.224491	0.7369	0.99995	no

ENSG00000161888	ENSG00000161888	SPC24	19:11200037-11266484	q1	q2	OK	19.5162	18.4802	-0.0786874	-0.0578974	0.90635	0.99995	no

ENSG00000161896	ENSG00000161896	IP6K3	6:33689443-33714762	q1	q2	NOTEST	0.0207608	0.0102363	-1.02017	0	1	1	no

ENSG00000161904	ENSG00000161904	LEMD2	6:33738978-33756913	q1	q2	OK	42.077	38.3023	-0.135602	-0.263596	0.6646	0.99995	no

ENSG00000161905	ENSG00000161905	ALOX15	17:4534196-4545589	q1	q2	NOTEST	0.0776531	0	#NAME?	0	1	1	no

ENSG00000161911	ENSG00000161911	TREML1	6:41117079-41122075	q1	q2	NOTEST	0.0561717	0	#NAME?	0	1	1	no

ENSG00000161912	ENSG00000161912	ADCY10P1	6:41068760-41108571	q1	q2	OK	1.32022	2.01876	0.612687	0.389173	0.5826	0.99995	no

ENSG00000161914	ENSG00000161914	ZNF653	19:11562140-11639989	q1	q2	OK	5.68583	4.97515	-0.192634	-0.091503	0.84985	0.99995	no

ENSG00000161920	ENSG00000161920	MED11	17:4634722-4643217	q1	q2	OK	18.8595	15.9346	-0.243131	-0.388942	0.6041	0.99995	no

ENSG00000161921	ENSG00000161921	CXCL16	17:4634722-4643217	q1	q2	NOTEST	0.290327	0.310162	0.0953428	0	1	1	no

ENSG00000161929	ENSG00000161929	SCIMP	17:5095378-5151364	q1	q2	NOTEST	0.147275	0.219184	0.573631	0	1	1	no

ENSG00000161939	ENSG00000161939	RNASEK-C17orf49	17:6757179-6922978	q1	q2	OK	1.80228	1.76376	-0.0311679	-0.0112802	0.9841	0.99995	no

ENSG00000161940	ENSG00000161940	BCL6B	17:6926338-6933609	q1	q2	NOTEST	0	0	0	0	1	1	no

ENSG00000161944	ENSG00000161944	ASGR2	17:7004640-7019019	q1	q2	NOTEST	0.0460925	0.0461419	0.00154485	0	1	1	no

ENSG00000161955	ENSG00000161955	TNFSF13	17:7452207-7464925	q1	q2	OK	1.09886	0.844653	-0.379572	-0.260074	0.62575	0.99995	no

ENSG00000161956	ENSG00000161956	SENP3	17:7465191-7536700	q1	q2	OK	33.8343	33.2186	-0.026498	-0.0108196	0.9795	0.99995	no

ENSG00000161958	ENSG00000161958	FGF11	17:7323421-7361026	q1	q2	OK	7.63838	6.72746	-0.183204	-0.26199	0.68755	0.99995	no

ENSG00000161960	ENSG00000161960	EIF4A1	17:7465191-7536700	q1	q2	OK	457.632	490.78	0.100891	0.170103	0.77985	0.99995	no

ENSG00000161970	ENSG00000161970	RPL26	17:8268880-8286531	q1	q2	OK	1086.98	1069.07	-0.0239704	-0.0557585	0.93645	0.99995	no

ENSG00000161973	ENSG00000161973	CCDC42	17:8633251-8648537	q1	q2	NOTEST	0	0.0260917	inf	0	1	1	no

ENSG00000161980	ENSG00000161980	POLR3K	16:96406-107669	q1	q2	OK	15.9431	16.3052	0.0324012	0.0405467	0.9516	0.99995	no

ENSG00000161981	ENSG00000161981	SNRNP25	16:96406-107669	q1	q2	OK	46.6208	42.421	-0.136193	-0.259952	0.7082	0.99995	no

ENSG00000161992	ENSG00000161992	C16orf11	16:610421-615528	q1	q2	NOTEST	0.0117707	0.0358854	1.6082	0	1	1	no

ENSG00000161996	ENSG00000161996	WDR90	16:699310-717833	q1	q2	OK	24.7663	22.5137	-0.137572	-0.191265	0.7418	0.99995	no

ENSG00000161999	ENSG00000161999	JMJD8	16:730223-734529	q1	q2	OK	28.8554	26.0955	-0.145038	-0.203505	0.7708	0.99995	no

ENSG00000162004	ENSG00000162004	CCDC78	16:770580-791329	q1	q2	OK	1.80989	1.62801	-0.15279	-0.0747156	0.85435	0.99995	no

ENSG00000162006	ENSG00000162006	MSLNL	16:819427-833370	q1	q2	NOTEST	0	0	0	0	1	1	no

ENSG00000162009	ENSG00000162009	SSTR5	16:1114092-1131454	q1	q2	NOTEST	0.0101603	0	#NAME?	0	1	1	no

ENSG00000162032	ENSG00000162032	SPSB3	16:1823207-1844972	q1	q2	OK	20.272	15.6778	-0.37076	-0.391975	0.56945	0.99995	no

ENSG00000162039	ENSG00000162039	MEIOB	16:1845620-1934295	q1	q2	NOTEST	0.0327918	0.0503647	0.619076	0	1	1	no

ENSG00000162040	ENSG00000162040	HS3ST6	16:1961463-1968441	q1	q2	NOTEST	0	0	0	0	1	1	no

ENSG00000162062	ENSG00000162062	C16orf59	16:2510080-2518214	q1	q2	OK	16.358	14.9152	-0.133209	-0.195526	0.7477	0.99995	no

ENSG00000162063	ENSG00000162063	CCNF	16:2479394-2509980	q1	q2	OK	16.3791	16.4636	0.00742599	0.0167119	0.98035	0.99995	no

ENSG00000162065	ENSG00000162065	TBC1D24	16:2525146-2581423	q1	q2	OK	6.20729	6.09747	-0.0257539	-0.0245877	0.96515	0.99995	no

ENSG00000162066	ENSG00000162066	AMDHD2	16:2525146-2581423	q1	q2	OK	11.191	9.6571	-0.21268	-0.117192	0.755	0.99995	no

ENSG00000162068	ENSG00000162068	NTN3	16:2521499-2524146	q1	q2	NOTEST	0.342321	0.256855	-0.414395	0	1	1	no

ENSG00000162069	ENSG00000162069	CCDC64B	16:3072620-3089134	q1	q2	NOTEST	0	0.0389644	inf	0	1	1	no

ENSG00000162073	ENSG00000162073	PAQR4	16:3013944-3030540	q1	q2	OK	24.1301	22.6616	-0.0905868	-0.157709	0.8144	0.99995	no

ENSG00000162076	ENSG00000162076	FLYWCH2	16:2933186-2949383	q1	q2	OK	14.8786	11.8523	-0.328068	-0.517609	0.4426	0.99995	no

ENSG00000162078	ENSG00000162078	ZG16B	16:2880169-2888967	q1	q2	NOTEST	0.0772745	0	#NAME?	0	1	1	no

ENSG00000162086	ENSG00000162086	ZNF75A	16:3313742-3368852	q1	q2	OK	22.746	24.3005	0.0953731	0.101499	0.8539	0.99995	no

ENSG00000162104	ENSG00000162104	ADCY9	16:4003387-4166186	q1	q2	OK	2.37052	2.45074	0.0480142	0.0527754	0.9072	0.99995	no

ENSG00000162105	ENSG00000162105	SHANK2	11:70313960-70963623	q1	q2	NOTEST	0.00377596	0.0802561	4.4097	0	1	1	no

ENSG00000162129	ENSG00000162129	CLPB	11:72003468-72145692	q1	q2	OK	5.99275	5.5912	-0.100058	-0.116626	0.81675	0.99995	no

ENSG00000162139	ENSG00000162139	NEU3	11:74699178-74729938	q1	q2	OK	3.30525	3.31187	0.00288433	0.00347436	0.99495	0.99995	no

ENSG00000162144	ENSG00000162144	CYB561A3	11:61066922-61136981	q1	q2	OK	11.0838	10.002	-0.148162	-0.0738005	0.8726	0.99995	no

ENSG00000162148	ENSG00000162148	PPP1R32	11:61170120-61258403	q1	q2	OK	2.50639	3.17105	0.339352	0.0783825	0.7442	0.99995	no

ENSG00000162174	ENSG00000162174	ASRGL1	11:62104382-62160882	q1	q2	OK	31.0182	28.9605	-0.0990257	-0.180409	0.765	0.99995	no

ENSG00000162188	ENSG00000162188	GNG3	11:62457746-62494856	q1	q2	OK	9.97052	8.40417	-0.246563	-0.0760615	0.90585	0.99995	no

ENSG00000162191	ENSG00000162191	UBXN1	11:62443969-62446567	q1	q2	OK	64.8794	60.4018	-0.103168	-0.23105	0.74195	0.99995	no

ENSG00000162194	ENSG00000162194	C11orf48	11:62414319-62441159	q1	q2	OK	70.7939	73.816	0.0603092	0.0902712	0.89295	0.99995	no

ENSG00000162222	ENSG00000162222	TTC9C	11:62495540-62507765	q1	q2	OK	28.2288	27.5756	-0.0337746	-0.0480657	0.9352	0.99995	no

ENSG00000162227	ENSG00000162227	TAF6L	11:62538591-62559493	q1	q2	OK	26.4289	24.6911	-0.0981238	-0.10715	0.82875	0.99995	no

ENSG00000162231	ENSG00000162231	NXF1	11:62559594-62573774	q1	q2	OK	43.8236	41.4234	-0.0812614	-0.142911	0.784	0.99995	no

ENSG00000162236	ENSG00000162236	STX5	11:62574368-62599560	q1	q2	OK	18.8752	14.5266	-0.377789	-0.727453	0.2902	0.99995	no

ENSG00000162241	ENSG00000162241	SLC25A45	11:65134908-65151172	q1	q2	OK	0.639218	0.399209	-0.679164	-0.38472	0.32255	0.99995	no

ENSG00000162244	ENSG00000162244	RPL29	3:52027615-52029958	q1	q2	OK	589.429	523.052	-0.172363	-0.403523	0.5605	0.99995	no

ENSG00000162267	ENSG00000162267	ITIH3	3:52828783-52843025	q1	q2	NOTEST	0.171942	0.414249	1.26857	0	1	1	no

ENSG00000162290	ENSG00000162290	DCP1A	3:53317446-53381654	q1	q2	NOTEST	0.213177	0.230521	0.112844	0	1	1	no

ENSG00000162298	ENSG00000162298	SYVN1	11:64888099-64902004	q1	q2	OK	14.937	13.3041	-0.167014	-0.163322	0.7907	0.99995	no

ENSG00000162300	ENSG00000162300	ZFPL1	11:64851694-64883856	q1	q2	OK	21.8799	18.7558	-0.22227	-0.179123	0.7481	0.99995	no

ENSG00000162302	ENSG00000162302	RPS6KA4	11:64126619-64139687	q1	q2	OK	6.30839	6.04105	-0.0624716	-0.0984295	0.8749	0.99995	no

ENSG00000162337	ENSG00000162337	LRP5	11:68080076-68216743	q1	q2	OK	16.4275	14.3918	-0.190867	-0.285811	0.5162	0.99995	no

ENSG00000162341	ENSG00000162341	TPCN2	11:68816364-68858072	q1	q2	OK	4.63913	4.11342	-0.173519	-0.197748	0.70415	0.99995	no

ENSG00000162344	ENSG00000162344	FGF19	11:69512999-69519410	q1	q2	NOTEST	0.0322557	0.0848661	1.39563	0	1	1	no

ENSG00000162365	ENSG00000162365	CYP4A22	1:47533159-47646011	q1	q2	NOTEST	0	0	0	0	1	1	no

ENSG00000162366	ENSG00000162366	PDZK1IP1	1:47649264-47656716	q1	q2	NOTEST	0	0	0	0	1	1	no

ENSG00000162367	ENSG00000162367	TAL1	1:47681961-47697892	q1	q2	NOTEST	0.416916	0.466303	0.161511	0	1	1	no

ENSG00000162368	ENSG00000162368	CMPK1	1:47799468-47844511	q1	q2	OK	48.0191	51.3719	0.0973728	0.228502	0.7259	0.99995	no

ENSG00000162373	ENSG00000162373	BEND5	1:48998526-50489585	q1	q2	OK	11.0686	13.6221	0.299471	0.355176	0.54095	0.99995	no

ENSG00000162374	ENSG00000162374	ELAVL4	1:50513685-50669458	q1	q2	OK	15.3634	16.4418	0.0978719	0.187268	0.7587	0.99995	no

ENSG00000162377	ENSG00000162377	COA7	1:53152507-53164038	q1	q2	OK	7.73968	8.48828	0.133198	0.221615	0.7547	0.99995	no

ENSG00000162378	ENSG00000162378	ZYG11B	1:53192125-53293014	q1	q2	OK	10.7674	11.5774	0.104648	0.242634	0.72555	0.99995	no

ENSG00000162383	ENSG00000162383	SLC1A7	1:53552854-53608289	q1	q2	NOTEST	0.115649	0.0679273	-0.767689	0	1	1	no

ENSG00000162384	ENSG00000162384	C1orf123	1:53662100-53686289	q1	q2	OK	46.3357	43.2526	-0.0993374	-0.167481	0.8061	0.99995	no

ENSG00000162385	ENSG00000162385	MAGOH	1:53692563-53710212	q1	q2	OK	68.4135	78.5856	0.199983	0.309825	0.65575	0.99995	no

ENSG00000162390	ENSG00000162390	ACOT11	1:55007929-55104865	q1	q2	OK	0.403783	0.57485	0.509608	0.407572	0.5362	0.99995	no

ENSG00000162391	ENSG00000162391	FAM151A	1:55007929-55104865	q1	q2	NOTEST	0.0339274	0	#NAME?	0	1	1	no

ENSG00000162396	ENSG00000162396	PARS2	1:55222570-55230187	q1	q2	OK	2.90121	2.44099	-0.249188	-0.349539	0.60875	0.99995	no

ENSG00000162398	ENSG00000162398	C1orf177	1:55271735-55307925	q1	q2	NOTEST	0.0345014	0.0158861	-1.11888	0	1	1	no

ENSG00000162399	ENSG00000162399	BSND	1:55464605-55476556	q1	q2	NOTEST	0	0	0	0	1	1	no

ENSG00000162402	ENSG00000162402	USP24	1:55532031-55680786	q1	q2	OK	18.4849	22.4868	0.282731	0.322297	0.33805	0.99995	no

ENSG00000162407	ENSG00000162407	PPAP2B	1:56960418-57110974	q1	q2	OK	21.8681	17.0324	-0.360552	-0.821265	0.23485	0.99995	no

ENSG00000162408	ENSG00000162408	NOL9	1:6581406-6614595	q1	q2	OK	8.52367	7.85347	-0.118144	-0.153838	0.72255	0.99995	no

ENSG00000162409	ENSG00000162409	PRKAA2	1:57110994-57181008	q1	q2	OK	2.73371	2.84989	0.0600465	0.119605	0.8614	0.99995	no

ENSG00000162413	ENSG00000162413	KLHL21	1:6650783-6684093	q1	q2	OK	5.74897	5.88532	0.0338165	0.0418257	0.9507	0.99995	no

ENSG00000162415	ENSG00000162415	ZSWIM5	1:45482070-45771881	q1	q2	OK	4.9523	5.3014	0.0982753	0.170475	0.77915	0.99995	no

ENSG00000162419	ENSG00000162419	GMEB1	1:28995243-29045865	q1	q2	OK	9.55861	10.3445	0.113991	0.192366	0.78005	0.99995	no

ENSG00000162426	ENSG00000162426	SLC45A1	1:8377885-8404227	q1	q2	OK	1.8732	1.54648	-0.276512	-0.250361	0.712	0.99995	no

ENSG00000162430	ENSG00000162430	SEPN1	1:26126666-26185903	q1	q2	OK	48.1206	46.2751	-0.0564189	-0.11531	0.8683	0.99995	no

ENSG00000162433	ENSG00000162433	AK4	1:65613231-65697828	q1	q2	OK	25.4606	27.8445	0.129125	0.23793	0.68265	0.99995	no

ENSG00000162434	ENSG00000162434	JAK1	1:65210777-65432187	q1	q2	OK	19.9892	21.2258	0.0866041	0.1091	0.82475	0.99995	no

ENSG00000162437	ENSG00000162437	RAVER2	1:65210777-65432187	q1	q2	OK	12.3254	11.9517	-0.0444124	-0.0526175	0.9417	0.99995	no

ENSG00000162438	ENSG00000162438	CTRC	1:15764934-15775737	q1	q2	NOTEST	0.074134	0.00779851	-3.24886	0	1	1	no

ENSG00000162441	ENSG00000162441	LZIC	1:9982172-10045559	q1	q2	OK	25.4308	26.7253	0.0716318	0.112136	0.8671	0.99995	no

ENSG00000162444	ENSG00000162444	RBP7	1:10057263-10076078	q1	q2	NOTEST	0.166464	0.268146	0.687805	0	1	1	no

ENSG00000162456	ENSG00000162456	KNCN	1:47004367-47082563	q1	q2	NOTEST	0.0653775	0	#NAME?	0	1	1	no

ENSG00000162458	ENSG00000162458	FBLIM1	1:16083101-16113089	q1	q2	OK	20.4564	22.1436	0.114343	0.173514	0.7353	0.99995	no

ENSG00000162460	ENSG00000162460	TMEM82	1:16010826-16076391	q1	q2	NOTEST	0	0	0	0	1	1	no

ENSG00000162461	ENSG00000162461	SLC25A34	1:16010826-16076391	q1	q2	OK	2.23595	1.01469	-1.13984	-0.437932	0.4761	0.99995	no

ENSG00000162482	ENSG00000162482	AKR7A3	1:19609051-19615744	q1	q2	OK	1.03248	0.942365	-0.13176	-0.139641	0.8429	0.99995	no

ENSG00000162490	ENSG00000162490	DRAXIN	1:11751785-11785914	q1	q2	OK	5.13732	4.77269	-0.106212	-0.226521	0.7463	0.99995	no

ENSG00000162493	ENSG00000162493	PDPN	1:13909959-13944452	q1	q2	OK	46.8258	41.6147	-0.170212	-0.328943	0.6408	0.99995	no

ENSG00000162494	ENSG00000162494	LRRC38	1:13801444-13842765	q1	q2	NOTEST	0	0	0	0	1	1	no

ENSG00000162496	ENSG00000162496	DHRS3	1:12627938-12677737	q1	q2	OK	0.906521	0.48785	-0.893903	-0.782788	0.1965	0.99995	no

ENSG00000162510	ENSG00000162510	MATN1	1:31184123-31199674	q1	q2	NOTEST	0.264784	0.233577	-0.180918	0	1	1	no

ENSG00000162511	ENSG00000162511	LAPTM5	1:31205315-31230667	q1	q2	NOTEST	0.168691	0.106467	-0.663977	0	1	1	no

ENSG00000162512	ENSG00000162512	SDC3	1:31342313-31381608	q1	q2	OK	63.5	65.644	0.0479056	0.062238	0.90225	0.99995	no

ENSG00000162517	ENSG00000162517	PEF1	1:32083286-32169920	q1	q2	OK	39.849	37.9858	-0.0690841	-0.109047	0.87165	0.99995	no

ENSG00000162520	ENSG00000162520	SYNC	1:33116742-33169197	q1	q2	OK	1.90485	1.78194	-0.0962278	-0.0241921	0.96685	0.99995	no

ENSG00000162521	ENSG00000162521	RBBP4	1:33116742-33169197	q1	q2	OK	247.291	250.599	0.019166	0.032557	0.95615	0.99995	no

ENSG00000162522	ENSG00000162522	KIAA1522	1:33207485-33240571	q1	q2	OK	9.20512	8.11999	-0.180958	-0.313853	0.5734	0.99995	no

ENSG00000162526	ENSG00000162526	TSSK3	1:32817121-32829913	q1	q2	OK	2.05108	1.87076	-0.132758	-0.0738471	0.8976	0.99995	no

ENSG00000162542	ENSG00000162542	TMCO4	1:20008705-20126438	q1	q2	NOTEST	0.269962	0.289604	0.101322	0	1	1	no

ENSG00000162543	ENSG00000162543	UBXN10	1:20510734-20522541	q1	q2	NOTEST	0.224092	0.294991	0.396585	0	1	1	no

ENSG00000162545	ENSG00000162545	CAMK2N1	1:20808883-20812713	q1	q2	OK	109.787	103.283	-0.0880972	-0.197566	0.7651	0.99995	no

ENSG00000162551	ENSG00000162551	ALPL	1:21835857-21904905	q1	q2	OK	4.37601	3.89602	-0.167615	-0.240323	0.7219	0.99995	no

ENSG00000162552	ENSG00000162552	WNT4	1:22443797-22470462	q1	q2	NOTEST	0.2157	0.16234	-0.410003	0	1	1	no

ENSG00000162571	ENSG00000162571	TTLL10	1:1108435-1133315	q1	q2	NOTEST	0	0	0	0	1	1	no

ENSG00000162572	ENSG00000162572	SCNN1D	1:1215815-1227409	q1	q2	OK	3.40839	2.44515	-0.479168	-0.544373	0.4224	0.99995	no

ENSG00000162576	ENSG00000162576	MXRA8	1:1288068-1297157	q1	q2	OK	10.1863	7.95905	-0.355961	-0.383864	0.5068	0.99995	no

ENSG00000162585	ENSG00000162585	C1orf86	1:1981908-2152177	q1	q2	OK	36.2829	33.7666	-0.10369	-0.145336	0.8446	0.99995	no

ENSG00000162591	ENSG00000162591	MEGF6	1:3406483-3528059	q1	q2	OK	9.83703	8.90145	-0.144183	-0.139494	0.76625	0.99995	no

ENSG00000162592	ENSG00000162592	CCDC27	1:3668961-3688209	q1	q2	NOTEST	0	0	0	0	1	1	no

ENSG00000162594	ENSG00000162594	IL23R	1:67557847-67725662	q1	q2	NOTEST	0	0	0	0	1	1	no

ENSG00000162595	ENSG00000162595	DIRAS3	1:68167148-68698803	q1	q2	OK	1.49007	1.62018	0.120774	0.0271404	0.94305	0.99995	no

ENSG00000162598	ENSG00000162598	C1orf87	1:60452940-60539442	q1	q2	NOTEST	0	0	0	0	1	1	no

ENSG00000162599	ENSG00000162599	NFIA	1:61330930-61928465	q1	q2	OK	3.27657	3.53199	0.108298	0.134152	0.8176	0.99995	no

ENSG00000162600	ENSG00000162600	OMA1	1:57394882-59012474	q1	q2	OK	5.96714	5.97423	0.00171501	0.000882012	0.99865	0.99995	no

ENSG00000162601	ENSG00000162601	MYSM1	1:59120410-59165764	q1	q2	OK	14.4242	16.7135	0.212525	0.308967	0.4641	0.99995	no

ENSG00000162604	ENSG00000162604	TM2D1	1:62146717-62191095	q1	q2	OK	14.5731	14.6688	0.00944999	0.0132322	0.98585	0.99995	no

ENSG00000162607	ENSG00000162607	USP1	1:62901967-62917475	q1	q2	OK	51.6551	57.5966	0.157071	0.385825	0.57685	0.99995	no

ENSG00000162613	ENSG00000162613	FUBP1	1:78409739-78444794	q1	q2	OK	146.278	158.647	0.117105	0.222034	0.71145	0.99995	no

ENSG00000162614	ENSG00000162614	NEXN	1:78347032-78409580	q1	q2	OK	6.51694	6.81438	0.0643867	0.104734	0.8762	0.99995	no

ENSG00000162616	ENSG00000162616	DNAJB4	1:78444858-78604133	q1	q2	OK	18.2782	18.156	-0.00968236	-0.0159445	0.9813	0.99995	no

ENSG00000162618	ENSG00000162618	ELTD1	1:79355448-79472403	q1	q2	OK	1.28316	1.68096	0.38958	0.463169	0.4824	0.99995	no

ENSG00000162620	ENSG00000162620	LRRIQ3	1:74491698-74663871	q1	q2	NOTEST	0.143186	0.217616	0.603896	0	1	1	no

ENSG00000162621	ENSG00000162621	LRRC53	1:74663895-75010112	q1	q2	NOTEST	0	0	0	0	1	1	no

ENSG00000162623	ENSG00000162623	TYW3	1:75171169-75232362	q1	q2	OK	10.0722	11.2748	0.162724	0.20044	0.7281	0.99995	no

ENSG00000162624	ENSG00000162624	LHX8	1:75593514-75627218	q1	q2	NOTEST	0	0.0147333	inf	0	1	1	no

ENSG00000162627	ENSG00000162627	SNX7	1:99127235-99226056	q1	q2	OK	27.9376	28.7196	0.039826	0.0880724	0.9018	0.99995	no

ENSG00000162630	ENSG00000162630	B3GALT2	1:193091146-193223031	q1	q2	OK	2.15651	2.17829	0.0145007	0.00911108	0.9898	0.99995	no

ENSG00000162631	ENSG00000162631	NTNG1	1:107682628-108026080	q1	q2	OK	1.39958	1.55118	0.148372	0.180991	0.7675	0.99995	no

ENSG00000162636	ENSG00000162636	FAM102B	1:109102710-109187522	q1	q2	OK	6.68836	8.04655	0.266718	0.5797	0.39925	0.99995	no

ENSG00000162639	ENSG00000162639	HENMT1	1:109190911-109204148	q1	q2	NOTEST	0.402126	0.300442	-0.420562	0	1	1	no

ENSG00000162641	ENSG00000162641	AKNAD1	1:109358519-109506111	q1	q2	OK	1.59862	1.03618	-0.625555	-0.12549	0.46555	0.99995	no

ENSG00000162642	ENSG00000162642	C1orf52	1:85715638-85725355	q1	q2	OK	26.1666	28.0807	0.101852	0.174245	0.8041	0.99995	no

ENSG00000162643	ENSG00000162643	WDR63	1:85464829-85598821	q1	q2	OK	0.537601	0.397743	-0.434698	-0.216542	0.7295	0.99995	no

ENSG00000162645	ENSG00000162645	GBP2	1:89571814-89641723	q1	q2	NOTEST	0.246865	0.197204	-0.324038	0	1	1	no

ENSG00000162650	ENSG00000162650	ATXN7L2	1:110026100-110035426	q1	q2	OK	7.76766	7.1106	-0.127508	-0.196963	0.76665	0.99995	no

ENSG00000162654	ENSG00000162654	GBP4	1:89646830-89664615	q1	q2	NOTEST	0	0.00416624	inf	0	1	1	no

ENSG00000162664	ENSG00000162664	ZNF326	1:90460670-90501090	q1	q2	OK	45.8521	49.7277	0.11706	0.242191	0.7266	0.99995	no

ENSG00000162669	ENSG00000162669	HFM1	1:91726322-91870426	q1	q2	OK	4.68955	3.71741	-0.335152	-0.330535	0.4862	0.99995	no

ENSG00000162670	ENSG00000162670	BRINP3	1:190066791-190446759	q1	q2	NOTEST	0.0352982	0.00981165	-1.84703	0	1	1	no

ENSG00000162676	ENSG00000162676	GFI1	1:92940318-92952433	q1	q2	NOTEST	0.177526	0.118757	-0.580022	0	1	1	no

ENSG00000162685	ENSG00000162685	LSP1P3	5:28925706-28927696	q1	q2	NOTEST	0	0	0	0	1	1	no

ENSG00000162687	ENSG00000162687	KCNT2	1:196194908-196578355	q1	q2	OK	4.71692	5.08344	0.107958	0.205356	0.7615	0.99995	no

ENSG00000162688	ENSG00000162688	AGL	1:100315639-100389579	q1	q2	OK	6.40976	6.88171	0.102497	0.228196	0.74365	0.99995	no

ENSG00000162692	ENSG00000162692	VCAM1	1:101185297-101204601	q1	q2	OK	15.8666	8.11474	-0.967375	-1.9687	0.00835	0.99995	no

ENSG00000162694	ENSG00000162694	EXTL2	1:101337942-101361554	q1	q2	OK	45.9678	41.4389	-0.149639	-0.28543	0.61485	0.99995	no

ENSG00000162695	ENSG00000162695	SLC30A7	1:101361631-101447309	q1	q2	OK	6.65522	6.99614	0.0720728	0.12648	0.81725	0.99995	no

ENSG00000162699	ENSG00000162699	DNAJA1P5	1:102268129-102462586	q1	q2	NOTEST	0	0	0	0	1	1	no

ENSG00000162702	ENSG00000162702	ZNF281	1:200374067-200379184	q1	q2	OK	21.497	22.7948	0.0845695	0.200131	0.7775	0.99995	no

ENSG00000162704	ENSG00000162704	ARPC5	1:183592400-183604892	q1	q2	OK	88.9849	100.116	0.170033	0.41152	0.55975	0.99995	no

ENSG00000162706	ENSG00000162706	CADM3	1:159141398-159177763	q1	q2	OK	15.4166	12.9007	-0.257032	-0.540046	0.43815	0.99995	no

ENSG00000162711	ENSG00000162711	NLRP3	1:247579457-247612410	q1	q2	NOTEST	0.0907088	0.0386828	-1.22955	0	1	1	no

ENSG00000162714	ENSG00000162714	ZNF496	1:247460713-247495148	q1	q2	OK	24.8466	24.5212	-0.0190163	-0.0354272	0.9531	0.99995	no

ENSG00000162722	ENSG00000162722	TRIM58	1:248020500-248060449	q1	q2	OK	1.3619	1.87392	0.460443	0.592436	0.398	0.99995	no

ENSG00000162723	ENSG00000162723	SLAMF9	1:159921281-159924044	q1	q2	NOTEST	0.0526175	0	#NAME?	0	1	1	no

ENSG00000162727	ENSG00000162727	OR2M5	1:248308449-248309388	q1	q2	NOTEST	0	0	0	0	1	1	no

ENSG00000162728	ENSG00000162728	KCNJ9	1:160051359-160060353	q1	q2	NOTEST	0.296255	0.391171	0.400958	0	1	1	no

ENSG00000162729	ENSG00000162729	IGSF8	1:160061129-160068733	q1	q2	OK	22.4908	22.5329	0.00270024	0.00568226	0.99275	0.99995	no

ENSG00000162733	ENSG00000162733	DDR2	1:162601162-162757190	q1	q2	OK	7.62781	7.2221	-0.0788494	-0.0837952	0.8354	0.99995	no

ENSG00000162734	ENSG00000162734	PEA15	1:160171988-160185166	q1	q2	OK	137.096	129.304	-0.0844273	-0.170519	0.7948	0.99995	no

ENSG00000162735	ENSG00000162735	PEX19	1:160185504-160256138	q1	q2	OK	39.3472	40.9637	0.0580824	0.0799296	0.9032	0.99995	no

ENSG00000162736	ENSG00000162736	NCSTN	1:160259062-160328742	q1	q2	OK	57.2825	56.3221	-0.0243937	-0.0277268	0.9597	0.99995	no

ENSG00000162738	ENSG00000162738	VANGL2	1:160370375-160398468	q1	q2	OK	72.8859	72.7477	-0.00273765	-0.00651692	0.99155	0.99995	no

ENSG00000162739	ENSG00000162739	SLAMF6	1:160454819-160493052	q1	q2	NOTEST	0.00995058	0.00914487	-0.121817	0	1	1	no

ENSG00000162745	ENSG00000162745	OLFML2B	1:161952981-161993644	q1	q2	OK	4.36169	4.04425	-0.109014	-0.136487	0.8278	0.99995	no

ENSG00000162746	ENSG00000162746	FCRLB	1:161691352-161697933	q1	q2	NOTEST	0.272891	0.381098	0.48184	0	1	1	no

ENSG00000162747	ENSG00000162747	FCGR3B	1:161475219-161648444	q1	q2	NOTEST	0	0	0	0	1	1	no

ENSG00000162753	ENSG00000162753	SLC9C2	1:173469602-173639001	q1	q2	NOTEST	0.175723	0.0706618	-1.3143	0	1	1	no

ENSG00000162755	ENSG00000162755	KLHDC9	1:161068150-161070136	q1	q2	OK	3.94125	3.08071	-0.355393	-0.349378	0.6216	0.99995	no

ENSG00000162757	ENSG00000162757	C1orf74	1:209929376-209957904	q1	q2	OK	2.74721	2.32245	-0.24232	-0.26813	0.7085	0.99995	no

ENSG00000162761	ENSG00000162761	LMX1A	1:165171103-165325952	q1	q2	NOTEST	0.0463375	0	#NAME?	0	1	1	no

ENSG00000162763	ENSG00000162763	LRRC52	1:165446077-165551713	q1	q2	NOTEST	0	0	0	0	1	1	no

ENSG00000162769	ENSG00000162769	FLVCR1	1:213031596-213072705	q1	q2	OK	11.5762	12.4278	0.10241	0.121919	0.7798	0.99995	no

ENSG00000162771	ENSG00000162771	FAM71A	1:212797625-212800120	q1	q2	NOTEST	0	4.19E-05	inf	0	1	1	no

ENSG00000162772	ENSG00000162772	ATF3	1:212738675-212794119	q1	q2	OK	5.3133	4.51823	-0.233851	-0.337266	0.61635	0.99995	no

ENSG00000162775	ENSG00000162775	RBM15	1:110828996-110889299	q1	q2	OK	5.23172	5.28606	0.014906	0.0263099	0.972	0.99995	no

ENSG00000162777	ENSG00000162777	DENND2D	1:111729795-111786062	q1	q2	NOTEST	0	0.0478172	inf	0	1	1	no

ENSG00000162779	ENSG00000162779	AXDND1	1:179334854-179545087	q1	q2	NOTEST	0.265491	0.345118	0.378427	0	1	1	no

ENSG00000162782	ENSG00000162782	TDRD5	1:179560747-179660407	q1	q2	NOTEST	0.0370907	0.0153611	-1.27177	0	1	1	no

ENSG00000162783	ENSG00000162783	IER5	1:181057637-181059977	q1	q2	OK	13.8501	12.421	-0.157111	-0.315273	0.65395	0.99995	no

ENSG00000162804	ENSG00000162804	SNED1	2:241938254-242041747	q1	q2	OK	0.772147	0.865179	0.164123	0.0790186	0.85515	0.99995	no

ENSG00000162813	ENSG00000162813	BPNT1	1:220230823-220263804	q1	q2	OK	11.7709	13.2869	0.174773	0.269021	0.6631	0.99995	no

ENSG00000162814	ENSG00000162814	SPATA17	1:217804665-218045038	q1	q2	OK	2.79486	2.27371	-0.297725	-0.254978	0.7199	0.99995	no

ENSG00000162817	ENSG00000162817	C1orf115	1:220863186-220872499	q1	q2	OK	1.36312	1.36143	-0.00178501	-0.00227384	0.99415	0.99995	no

ENSG00000162819	ENSG00000162819	BROX	1:222841354-222908538	q1	q2	OK	17.3694	18.3724	0.0809922	0.124992	0.83355	0.99995	no

ENSG00000162825	ENSG00000162825	NBPF8	1:144146807-144224481	q1	q2	NOTEST	0.252604	0.388042	0.619336	0	1	1	no

ENSG00000162836	ENSG00000162836	ACP6	1:147101452-147142618	q1	q2	OK	0.724781	0.610757	-0.246946	-0.167552	0.84495	0.99995	no

ENSG00000162840	ENSG00000162840	MT2P1	4:69242040-69242226	q1	q2	NOTEST	0	0	0	0	1	1	no

ENSG00000162843	ENSG00000162843	WDR64	1:241695433-241965435	q1	q2	NOTEST	0.0112679	0.0306824	1.44519	0	1	1	no

ENSG00000162849	ENSG00000162849	KIF26B	1:245318286-245872733	q1	q2	OK	0.970575	0.990264	0.0289723	0.0333427	0.95225	0.99995	no

ENSG00000162851	ENSG00000162851	TFB2M	1:246703861-246729626	q1	q2	OK	6.77758	8.43558	0.315716	0.524167	0.44905	0.99995	no

ENSG00000162852	ENSG00000162852	CNST	1:246729745-246831886	q1	q2	OK	5.65859	6.03239	0.0922852	0.167075	0.78795	0.99995	no

ENSG00000162869	ENSG00000162869	PPP1R21	2:48667736-48742525	q1	q2	OK	8.443	8.88666	0.0738851	0.0904896	0.8663	0.99995	no

ENSG00000162873	ENSG00000162873	KLHDC8A	1:205305219-205326218	q1	q2	OK	36.5935	35.3373	-0.0503961	-0.0910876	0.8711	0.99995	no

ENSG00000162877	ENSG00000162877	PM20D1	1:205797149-205819260	q1	q2	NOTEST	0	0.0567348	inf	0	1	1	no

ENSG00000162878	ENSG00000162878	PKDCC	2:42275159-42285668	q1	q2	OK	39.8015	38.0913	-0.0633594	-0.10239	0.8788	0.99995	no

ENSG00000162881	ENSG00000162881	OXER1	2:42989641-42991401	q1	q2	OK	0.888104	1.00002	0.17123	0.162801	0.8163	0.99995	no

ENSG00000162882	ENSG00000162882	HAAO	2:42994228-43019733	q1	q2	OK	2.2513	2.49709	0.149487	0.139716	0.8422	0.99995	no

ENSG00000162885	ENSG00000162885	B3GALNT2	1:235613237-235667781	q1	q2	OK	10.3303	11.0985	0.103478	0.134836	0.79675	0.99995	no

ENSG00000162888	ENSG00000162888	C1orf147	1:206643790-206671061	q1	q2	NOTEST	0	0	0	0	1	1	no

ENSG00000162889	ENSG00000162889	MAPKAPK2	1:206858288-206907628	q1	q2	OK	5.22308	4.19713	-0.315497	-0.285802	0.6858	0.99995	no

ENSG00000162891	ENSG00000162891	IL20	1:207038698-207042568	q1	q2	NOTEST	0	0	0	0	1	1	no

ENSG00000162892	ENSG00000162892	IL24	1:207070787-207077484	q1	q2	NOTEST	0	0	0	0	1	1	no

ENSG00000162894	ENSG00000162894	FAIM3	1:207077730-207096592	q1	q2	NOTEST	0.0277565	0	#NAME?	0	1	1	no

ENSG00000162896	ENSG00000162896	PIGR	1:207101862-207119811	q1	q2	NOTEST	0.00581732	0	#NAME?	0	1	1	no

ENSG00000162897	ENSG00000162897	FCAMR	1:207131309-207143970	q1	q2	NOTEST	0	0.0102579	inf	0	1	1	no

ENSG00000162909	ENSG00000162909	CAPN2	1:223889294-223963720	q1	q2	OK	58.5239	57.7581	-0.0190042	-0.0349925	0.94885	0.99995	no

ENSG00000162910	ENSG00000162910	MRPL55	1:228294379-228297013	q1	q2	OK	31.5382	25.9537	-0.281158	-0.433878	0.52435	0.99995	no

ENSG00000162913	ENSG00000162913	C1orf145	1:228391206-228566577	q1	q2	OK	1.20146	0.820819	-0.549654	-0.163295	0.70625	0.99995	no

ENSG00000162923	ENSG00000162923	WDR26	1:224572844-224928251	q1	q2	OK	34.6634	37.5958	0.117157	0.22477	0.7156	0.99995	no

ENSG00000162924	ENSG00000162924	REL	2:61108655-61158745	q1	q2	OK	1.19099	1.28928	0.114398	0.136457	0.8426	0.99995	no

ENSG00000162927	ENSG00000162927	PUS10	2:61167356-61279125	q1	q2	OK	2.95943	2.54516	-0.217559	-0.128368	0.8264	0.99995	no

ENSG00000162928	ENSG00000162928	PEX13	2:61167356-61279125	q1	q2	OK	12.1378	12.7906	0.0755787	0.109867	0.86745	0.99995	no

ENSG00000162929	ENSG00000162929	KIAA1841	2:61293005-61391964	q1	q2	OK	33.6616	34.1611	0.0212487	0.0242152	0.9667	0.99995	no

ENSG00000162931	ENSG00000162931	TRIM17	1:228595081-228604562	q1	q2	OK	0.56507	0.575807	0.0271538	0.0226124	0.9668	0.99995	no

ENSG00000162944	ENSG00000162944	RFTN2	2:198432947-198540769	q1	q2	OK	13.0882	12.7603	-0.036601	-0.043565	0.941	0.99995	no

ENSG00000162946	ENSG00000162946	DISC1	1:231664398-232177018	q1	q2	OK	3.12345	3.59411	0.202497	0.183294	0.715	0.99995	no

ENSG00000162947	ENSG00000162947	AC007364.1	2:150426147-150715705	q1	q2	NOTEST	0.0290812	0.0584486	1.00708	0	1	1	no

ENSG00000162949	ENSG00000162949	CAPN13	2:30945636-31043408	q1	q2	NOTEST	0	0	0	0	1	1	no

ENSG00000162951	ENSG00000162951	LRRTM1	2:79384131-80875905	q1	q2	OK	0.518948	0.389534	-0.413842	-0.0795801	0.71835	0.99995	no

ENSG00000162959	ENSG00000162959	MEMO1	2:32090128-32264881	q1	q2	OK	21.1515	25.5429	0.27216	0.364688	0.5385	0.99995	no

ENSG00000162961	ENSG00000162961	DPY30	2:32090128-32264881	q1	q2	OK	65.0959	63.609	-0.0333352	-0.0543052	0.9359	0.99995	no

ENSG00000162971	ENSG00000162971	TYW5	2:200775978-200873263	q1	q2	OK	6.51571	7.24824	0.15371	0.181668	0.80145	0.99995	no

ENSG00000162972	ENSG00000162972	C2orf47	2:200775978-200873263	q1	q2	OK	14.1685	15.2547	0.106567	0.12656	0.8521	0.99995	no

ENSG00000162975	ENSG00000162975	KCNF1	2:11052062-11054350	q1	q2	OK	2.51216	3.32489	0.404376	0.552021	0.42915	0.99995	no

ENSG00000162976	ENSG00000162976	PQLC3	2:11295323-11319000	q1	q2	OK	4.33642	4.90221	0.176927	0.17147	0.74425	0.99995	no

ENSG00000162980	ENSG00000162980	ARL5A	2:152645497-152685006	q1	q2	OK	31.2412	35.3825	0.179587	0.226352	0.70505	0.99995	no

ENSG00000162981	ENSG00000162981	FAM84A	2:14772809-14790933	q1	q2	OK	2.46904	2.14423	-0.203491	-0.263049	0.69825	0.99995	no

ENSG00000162989	ENSG00000162989	KCNJ3	2:155554810-155714863	q1	q2	NOTEST	0.0798815	0.0896589	0.166585	0	1	1	no

ENSG00000162992	ENSG00000162992	NEUROD1	2:182401402-182545603	q1	q2	OK	3.97659	4.32343	0.120646	0.136632	0.84385	0.99995	no

ENSG00000162994	ENSG00000162994	CLHC1	2:55401926-55462989	q1	q2	OK	15.369	16.0306	0.060799	0.0207606	0.9368	0.99995	no

ENSG00000162997	ENSG00000162997	PRORSD1P	2:55509454-55511658	q1	q2	OK	0.93001	0.981154	0.0772332	0.0661474	0.90635	0.99995	no

ENSG00000162998	ENSG00000162998	FRZB	2:183698001-183731890	q1	q2	OK	31.5744	34.6574	0.134408	0.28173	0.67935	0.99995	no

ENSG00000162999	ENSG00000162999	DUSP19	2:183943286-183973247	q1	q2	OK	0.864375	1.02872	0.251117	0.231816	0.682	0.99995	no

ENSG00000163001	ENSG00000163001	CCDC104	2:55746739-55773015	q1	q2	OK	31.1224	31.5603	0.0201604	0.0393602	0.9559	0.99995	no

ENSG00000163002	ENSG00000163002	NUP35	2:183982240-184026408	q1	q2	OK	13.2736	17.3341	0.38505	0.725404	0.2732	0.99995	no

ENSG00000163006	ENSG00000163006	CCDC138	2:109403212-109501933	q1	q2	OK	13.2654	15.2891	0.204834	0.368873	0.53865	0.99995	no

ENSG00000163009	ENSG00000163009	C2orf48	2:10281508-10351851	q1	q2	OK	1.85355	1.87698	0.0181265	0.0222804	0.97385	0.99995	no

ENSG00000163012	ENSG00000163012	ZSWIM2	2:187692561-187713935	q1	q2	NOTEST	0	0	0	0	1	1	no

ENSG00000163013	ENSG00000163013	FBXO41	2:73481809-73511559	q1	q2	OK	2.17978	2.50848	0.202631	0.253488	0.6138	0.99995	no

ENSG00000163016	ENSG00000163016	ALMS1P	2:73872045-73912703	q1	q2	NOTEST	0.197695	0.301047	0.606717	0	1	1	no

ENSG00000163017	ENSG00000163017	ACTG2	2:74119440-74146992	q1	q2	OK	27.8624	30.6691	0.138467	0.241045	0.71475	0.99995	no

ENSG00000163026	ENSG00000163026	C2orf44	2:24232950-24286551	q1	q2	OK	10.6104	10.8751	0.0355599	0.0711088	0.9209	0.99995	no

ENSG00000163029	ENSG00000163029	SMC6	2:17845078-17981509	q1	q2	OK	18.8081	20.5903	0.130611	0.188644	0.74435	0.99995	no

ENSG00000163032	ENSG00000163032	VSNL1	2:17720392-17838285	q1	q2	OK	0.868249	1.17383	0.435045	0.361084	0.51945	0.99995	no

ENSG00000163040	ENSG00000163040	CCDC74A	2:132285247-132291239	q1	q2	OK	10.1278	8.12952	-0.317081	-0.444873	0.48045	0.99995	no

ENSG00000163041	ENSG00000163041	H3F3A	1:226248412-226259702	q1	q2	OK	571.398	530.388	-0.10745	-0.251606	0.727	0.99995	no

ENSG00000163046	ENSG00000163046	ANKRD30BL	2:132905163-133015542	q1	q2	NOTEST	0	0	0	0	1	1	no

ENSG00000163050	ENSG00000163050	ADCK3	1:227085236-227175246	q1	q2	OK	4.44076	2.50821	-0.824149	-0.908922	0.10265	0.99995	no

ENSG00000163053	ENSG00000163053	SLC16A14	2:230899697-230933715	q1	q2	OK	2.73384	2.78886	0.0287446	0.0422565	0.9442	0.99995	no

ENSG00000163060	ENSG00000163060	TEKT4	2:95533230-95613086	q1	q2	NOTEST	0	0	0	0	1	1	no

ENSG00000163064	ENSG00000163064	EN1	2:119599746-119605254	q1	q2	OK	22.9485	28.8574	0.330544	0.571433	0.32885	0.99995	no

ENSG00000163067	ENSG00000163067	ZNF2	2:95831176-95850065	q1	q2	OK	3.21206	3.44778	0.102171	0.149103	0.8286	0.99995	no

ENSG00000163069	ENSG00000163069	SGCB	4:52886871-52904648	q1	q2	OK	22.4514	26.2466	0.225325	0.442526	0.4563	0.99995	no

ENSG00000163071	ENSG00000163071	SPATA18	4:52917496-52963458	q1	q2	OK	3.92129	3.44647	-0.186208	-0.227289	0.6417	0.99995	no

ENSG00000163072	ENSG00000163072	NOSTRIN	2:169312371-169769881	q1	q2	OK	0.861697	0.780316	-0.143122	-0.017902	0.8938	0.99995	no

ENSG00000163075	ENSG00000163075	PCDP1	2:120302007-120419827	q1	q2	OK	2.18192	2.41198	0.144621	0.132932	0.83455	0.99995	no

ENSG00000163081	ENSG00000163081	CCDC140	2:223064606-223169936	q1	q2	NOTEST	0.0166985	0	#NAME?	0	1	1	no

ENSG00000163082	ENSG00000163082	SGPP2	2:223289235-223425667	q1	q2	OK	1.0111	1.03613	0.0352699	0.0435434	0.95005	0.99995	no

ENSG00000163083	ENSG00000163083	INHBB	2:121103718-121109384	q1	q2	OK	0.642427	0.471444	-0.446444	-0.479191	0.4904	0.99995	no

ENSG00000163092	ENSG00000163092	XIRP2	2:167744996-168116263	q1	q2	NOTEST	0.0328552	0.053752	0.710196	0	1	1	no

ENSG00000163093	ENSG00000163093	BBS5	2:170335687-170382772	q1	q2	OK	4.8057	4.04843	-0.247384	-0.338769	0.56475	0.99995	no

ENSG00000163098	ENSG00000163098	BIRC8	19:53792855-53794875	q1	q2	NOTEST	0	0	0	0	1	1	no

ENSG00000163104	ENSG00000163104	SMARCAD1	4:95128761-95212443	q1	q2	OK	34.2985	37.3629	0.123461	0.224543	0.6677	0.99995	no

ENSG00000163106	ENSG00000163106	HPGDS	4:95219685-95264027	q1	q2	NOTEST	0	0.0171646	inf	0	1	1	no

ENSG00000163110	ENSG00000163110	PDLIM5	4:95373036-95589377	q1	q2	OK	13.2353	13.4409	0.0222427	0.0358736	0.9378	0.99995	no

ENSG00000163113	ENSG00000163113	OTUD7B	1:149909704-149982625	q1	q2	NOTEST	0.0901717	0.0720651	-0.323375	0	1	1	no

ENSG00000163114	ENSG00000163114	PDHA2	4:96761238-96762625	q1	q2	NOTEST	0	0	0	0	1	1	no

ENSG00000163116	ENSG00000163116	STPG2	4:98105243-99064391	q1	q2	NOTEST	0.0401072	0.00573669	-2.80557	0	1	1	no

ENSG00000163121	ENSG00000163121	NEURL3	2:97163382-97173846	q1	q2	NOTEST	0	0.020789	inf	0	1	1	no

ENSG00000163125	ENSG00000163125	RPRD2	1:150335566-150449042	q1	q2	OK	10.8905	10.2333	-0.0897941	-0.206731	0.7686	0.99995	no

ENSG00000163126	ENSG00000163126	ANKRD23	2:97481981-97523832	q1	q2	OK	1.80247	1.70915	-0.0766979	-0.0361785	0.95315	0.99995	no

ENSG00000163131	ENSG00000163131	CTSS	1:150702671-150738433	q1	q2	OK	0.644844	0.866651	0.426502	0.297032	0.596	0.99995	no

ENSG00000163132	ENSG00000163132	MSX1	4:4861392-4865663	q1	q2	OK	2.08581	2.15752	0.0487642	0.0505377	0.9403	0.99995	no

ENSG00000163138	ENSG00000163138	PACRGL	4:20697904-21950422	q1	q2	OK	13.8625	15.0501	0.118577	0.164478	0.756	0.99995	no

ENSG00000163141	ENSG00000163141	BNIPL	1:151009045-151020076	q1	q2	OK	0.552217	0.848065	0.618938	0.409822	0.4653	0.99995	no

ENSG00000163145	ENSG00000163145	C1QTNF7	4:15004297-15447790	q1	q2	NOTEST	0.0187812	0.142384	2.92242	0	1	1	no

ENSG00000163154	ENSG00000163154	TNFAIP8L2	1:151129104-151167797	q1	q2	NOTEST	0.149633	0.0724596	-1.04618	0	1	1	no

ENSG00000163155	ENSG00000163155	LYSMD1	1:151129104-151167797	q1	q2	OK	6.41817	5.7181	-0.166626	-0.12689	0.8423	0.99995	no

ENSG00000163156	ENSG00000163156	SCNM1	1:151129104-151167797	q1	q2	OK	30.0883	30.3336	0.0117128	0.0114729	0.98905	0.99995	no

ENSG00000163157	ENSG00000163157	TMOD4	1:151129104-151167797	q1	q2	NOTEST	0.117951	0.432606	1.87486	0	1	1	no

ENSG00000163159	ENSG00000163159	VPS72	1:151129104-151167797	q1	q2	OK	61.9264	59.4394	-0.0591364	-0.0896845	0.88055	0.99995	no

ENSG00000163161	ENSG00000163161	ERCC3	2:128014865-128051752	q1	q2	OK	33.6225	34.6671	0.0441414	0.0840266	0.89085	0.99995	no

ENSG00000163162	ENSG00000163162	RNF149	2:101887680-101925163	q1	q2	OK	16.692	18.7394	0.16692	0.252108	0.69465	0.99995	no

ENSG00000163166	ENSG00000163166	IWS1	2:128193782-128284462	q1	q2	OK	37.2927	40.2075	0.10857	0.236428	0.7024	0.99995	no

ENSG00000163170	ENSG00000163170	BOLA3	2:74362524-74375121	q1	q2	OK	21.3153	24.9773	0.228728	0.323181	0.6473	0.99995	no

ENSG00000163171	ENSG00000163171	CDC42EP3	2:37827278-37965611	q1	q2	OK	23.8958	34.6862	0.537604	0.841635	0.20005	0.99995	no

ENSG00000163191	ENSG00000163191	S100A11	1:151967006-152020383	q1	q2	OK	91.7342	87.196	-0.0731982	-0.135694	0.8443	0.99995	no

ENSG00000163202	ENSG00000163202	LCE3D	1:152551856-152552980	q1	q2	NOTEST	0	0	0	0	1	1	no

ENSG00000163206	ENSG00000163206	SMCP	1:152850792-152857523	q1	q2	NOTEST	0	0	0	0	1	1	no

ENSG00000163207	ENSG00000163207	IVL	1:152881020-152884362	q1	q2	NOTEST	0	0	0	0	1	1	no

ENSG00000163209	ENSG00000163209	SPRR3	1:152974222-152976332	q1	q2	NOTEST	0	0	0	0	1	1	no

ENSG00000163214	ENSG00000163214	DHX57	2:39024870-39103075	q1	q2	OK	18.9895	17.8745	-0.0872983	-0.121676	0.78575	0.99995	no

ENSG00000163216	ENSG00000163216	SPRR2D	1:153012200-153014407	q1	q2	NOTEST	0	0	0	0	1	1	no

ENSG00000163217	ENSG00000163217	BMP10	2:69092612-69098649	q1	q2	NOTEST	0.0171462	0	#NAME?	0	1	1	no

ENSG00000163218	ENSG00000163218	PGLYRP4	1:153302595-153321316	q1	q2	NOTEST	0	0	0	0	1	1	no

ENSG00000163219	ENSG00000163219	ARHGAP25	2:68906732-69053965	q1	q2	NOTEST	0	0.0827445	inf	0	1	1	no

ENSG00000163220	ENSG00000163220	S100A9	1:153330329-153333503	q1	q2	NOTEST	0	0	0	0	1	1	no

ENSG00000163221	ENSG00000163221	S100A12	1:153346183-153348125	q1	q2	NOTEST	0	0	0	0	1	1	no

ENSG00000163235	ENSG00000163235	TGFA	2:70674411-70781325	q1	q2	NOTEST	0	0	0	0	1	1	no

ENSG00000163239	ENSG00000163239	TDRD10	1:154474694-154520623	q1	q2	NOTEST	0	0.0156432	inf	0	1	1	no

ENSG00000163249	ENSG00000163249	CCNYL1	2:208576263-208626563	q1	q2	OK	14.8622	16.6311	0.162236	0.33026	0.60115	0.99995	no

ENSG00000163251	ENSG00000163251	FZD5	2:208627309-208634287	q1	q2	OK	0.663983	0.643868	-0.044383	-0.0569438	0.9321	0.99995	no

ENSG00000163254	ENSG00000163254	CRYGC	2:208992860-208994554	q1	q2	OK	1.26533	0.610159	-1.05225	-0.849684	0.2572	0.99995	no

ENSG00000163257	ENSG00000163257	DCAF16	4:17802277-17812381	q1	q2	OK	17.3698	18.0807	0.0578716	0.0936065	0.85735	0.99995	no

ENSG00000163263	ENSG00000163263	C1orf189	1:154171847-154178809	q1	q2	NOTEST	0.12548	0	#NAME?	0	1	1	no

ENSG00000163273	ENSG00000163273	NPPC	2:232786529-232791113	q1	q2	NOTEST	0.457095	0.338463	-0.433494	0	1	1	no

ENSG00000163281	ENSG00000163281	GNPDA2	4:44624085-44728612	q1	q2	OK	10.1265	11.2617	0.153297	0.159995	0.797	0.99995	no

ENSG00000163283	ENSG00000163283	ALPP	2:233243243-233247599	q1	q2	NOTEST	0	0	0	0	1	1	no

ENSG00000163285	ENSG00000163285	GABRG1	4:46037785-46126098	q1	q2	NOTEST	0.026193	0.0251876	-0.0564673	0	1	1	no

ENSG00000163286	ENSG00000163286	ALPPL2	2:233271552-233275424	q1	q2	NOTEST	0	0	0	0	1	1	no

ENSG00000163288	ENSG00000163288	GABRB1	4:46920916-47428461	q1	q2	NOTEST	0.0628409	0.0839821	0.418378	0	1	1	no

ENSG00000163291	ENSG00000163291	PAQR3	4:79697495-79860592	q1	q2	OK	19.4819	17.5106	-0.153908	-0.188799	0.71525	0.99995	no

ENSG00000163293	ENSG00000163293	NIPAL1	4:47849256-48042188	q1	q2	NOTEST	0.174779	0.237888	0.444751	0	1	1	no

ENSG00000163295	ENSG00000163295	ALPI	2:233320832-233324742	q1	q2	NOTEST	0	0	0	0	1	1	no

ENSG00000163297	ENSG00000163297	ANTXR2	4:80822302-81046608	q1	q2	OK	32.3565	33.4838	0.0494084	0.0778767	0.91295	0.99995	no

ENSG00000163312	ENSG00000163312	HELQ	4:84328495-84377009	q1	q2	OK	5.97391	4.87576	-0.293049	-0.336096	0.5374	0.99995	no

ENSG00000163319	ENSG00000163319	MRPS18C	4:84377084-84444501	q1	q2	OK	32.8575	39.363	0.260616	0.320015	0.57925	0.99995	no

ENSG00000163320	ENSG00000163320	CGGBP1	3:88101093-88217879	q1	q2	OK	65.8877	72.5855	0.139672	0.227323	0.66105	0.99995	no

ENSG00000163322	ENSG00000163322	FAM175A	4:84377084-84444501	q1	q2	OK	9.1355	8.41002	-0.119374	-0.138552	0.82495	0.99995	no

ENSG00000163328	ENSG00000163328	GPR155	2:175296965-175351822	q1	q2	OK	1.1215	1.12652	0.00643716	0.00630946	0.98915	0.99995	no

ENSG00000163331	ENSG00000163331	DAPL1	2:159651828-159719797	q1	q2	NOTEST	0	0	0	0	1	1	no

ENSG00000163344	ENSG00000163344	PMVK	1:154897209-154909467	q1	q2	OK	26.1311	23.3633	-0.161523	-0.325989	0.64635	0.99995	no

ENSG00000163346	ENSG00000163346	PBXIP1	1:154916551-154928599	q1	q2	OK	25.1051	22.6813	-0.146479	-0.298066	0.6245	0.99995	no

ENSG00000163347	ENSG00000163347	CLDN1	3:190023489-190040264	q1	q2	NOTEST	0.236518	0.17486	-0.435748	0	1	1	no

ENSG00000163348	ENSG00000163348	PYGO2	1:154929501-154946871	q1	q2	OK	28.219	28.183	-0.00184269	-0.00243719	0.9972	0.99995	no

ENSG00000163349	ENSG00000163349	HIPK1	1:114466621-114520426	q1	q2	OK	18.6714	19.4711	0.0605062	0.14165	0.83495	0.99995	no

ENSG00000163352	ENSG00000163352	LENEP	1:154966061-154966791	q1	q2	NOTEST	0.212327	0	#NAME?	0	1	1	no

ENSG00000163354	ENSG00000163354	DCST2	1:154975126-155006257	q1	q2	NOTEST	0.391214	0.0994278	-1.97624	0	1	1	no

ENSG00000163357	ENSG00000163357	DCST1	1:155006299-155035252	q1	q2	NOTEST	0.172063	0.0733934	-1.22922	0	1	1	no

ENSG00000163359	ENSG00000163359	COL6A3	2:238232645-238323018	q1	q2	OK	101.835	105.67	0.0533335	0.0709286	0.90215	0.99995	no

ENSG00000163362	ENSG00000163362	C1orf106	1:200860175-200884863	q1	q2	OK	13.9908	13.6999	-0.0303154	-0.0459629	0.9255	0.99995	no

ENSG00000163364	ENSG00000163364	LINC01116	2:177494316-177520686	q1	q2	OK	0.78571	0.40419	-0.958963	-0.664605	0.29095	0.99995	no

ENSG00000163374	ENSG00000163374	YY1AP1	1:155579566-155829191	q1	q2	OK	32.6579	32.4498	-0.00922387	-0.0112412	0.985	0.99995	no

ENSG00000163376	ENSG00000163376	KBTBD8	3:67048726-67061634	q1	q2	OK	2.39994	2.85573	0.250858	0.295096	0.56005	0.99995	no

ENSG00000163377	ENSG00000163377	FAM19A4	3:68780916-68981761	q1	q2	OK	0.72696	0.854514	0.233229	0.205315	0.7428	0.99995	no

ENSG00000163378	ENSG00000163378	EOGT	3:69024364-69129559	q1	q2	OK	4.88184	6.11189	0.324192	0.226267	0.6874	0.99995	no

ENSG00000163380	ENSG00000163380	LMOD3	3:69156022-69172183	q1	q2	NOTEST	0.0369771	0.0418148	0.177382	0	1	1	no

ENSG00000163382	ENSG00000163382	APOA1BP	1:156561553-156564091	q1	q2	OK	32.0773	32.4691	0.0175114	0.0358631	0.9582	0.99995	no

ENSG00000163386	ENSG00000163386	NBPF10	1:145209118-145370303	q1	q2	OK	0.560766	0.619358	0.143376	0.11131	0.86735	0.99995	no

ENSG00000163389	ENSG00000163389	POGLUT1	3:119187784-119213555	q1	q2	OK	12.6723	14.0122	0.145003	0.193535	0.6844	0.99995	no

ENSG00000163393	ENSG00000163393	SLC22A15	1:116519118-116612675	q1	q2	OK	2.07419	2.40173	0.211528	0.282754	0.62685	0.99995	no

ENSG00000163394	ENSG00000163394	CCKAR	4:26483021-26492084	q1	q2	NOTEST	0.0164674	0.0151627	-0.119087	0	1	1	no

ENSG00000163395	ENSG00000163395	IGFN1	1:201159952-201198080	q1	q2	NOTEST	0.126795	0.0862663	-0.555628	0	1	1	no

ENSG00000163399	ENSG00000163399	ATP1A1	1:116915289-116961244	q1	q2	OK	61.0329	62.1853	0.026988	0.060683	0.9219	0.99995	no

ENSG00000163406	ENSG00000163406	SLC15A2	3:121612935-121662949	q1	q2	OK	1.06694	0.858115	-0.314238	-0.245238	0.5551	0.99995	no

ENSG00000163412	ENSG00000163412	EIF4E3	3:71724566-71805647	q1	q2	OK	4.87173	5.06512	0.0561618	0.0818731	0.89915	0.99995	no

ENSG00000163421	ENSG00000163421	PROK2	3:71820806-71834357	q1	q2	NOTEST	0.187925	0.125539	-0.582027	0	1	1	no

ENSG00000163424	ENSG00000163424	C3orf30	3:118864996-118924000	q1	q2	NOTEST	0	0	0	0	1	1	no

ENSG00000163428	ENSG00000163428	LRRC58	3:120043355-120068186	q1	q2	OK	7.7955	8.48779	0.122748	0.289019	0.68105	0.99995	no

ENSG00000163430	ENSG00000163430	FSTL1	3:120111139-120170100	q1	q2	OK	140.855	140.005	-0.00873668	-0.0110972	0.98215	0.99995	no

ENSG00000163431	ENSG00000163431	LMOD1	1:201862969-201915715	q1	q2	NOTEST	0.108484	0.074472	-0.542707	0	1	1	no

ENSG00000163435	ENSG00000163435	ELF3	1:201951499-201986316	q1	q2	NOTEST	0.193833	0.237876	0.295391	0	1	1	no

ENSG00000163440	ENSG00000163440	PDCL2	4:56422691-56458379	q1	q2	NOTEST	0.113801	0.214909	0.91721	0	1	1	no

ENSG00000163444	ENSG00000163444	TMEM183A	1:202976513-202993976	q1	q2	OK	33.832	32.9552	-0.037884	-0.0704472	0.91515	0.99995	no

ENSG00000163449	ENSG00000163449	TMEM169	2:216809212-216967506	q1	q2	OK	18.5876	20.8846	0.168099	0.305416	0.6397	0.99995	no

ENSG00000163453	ENSG00000163453	IGFBP7	4:57829535-58071676	q1	q2	OK	18.5193	11.7834	-0.652273	-0.283174	0.74035	0.99995	no

ENSG00000163462	ENSG00000163462	TRIM46	1:155141883-155162707	q1	q2	OK	8.03004	6.90156	-0.218485	-0.267521	0.6899	0.99995	no

ENSG00000163463	ENSG00000163463	KRTCAP2	1:155141883-155162707	q1	q2	OK	77.2785	75.5317	-0.0329852	-0.0506258	0.943	0.99995	no

ENSG00000163464	ENSG00000163464	CXCR1	2:219027567-219031718	q1	q2	NOTEST	0	0	0	0	1	1	no

ENSG00000163466	ENSG00000163466	ARPC2	2:219081816-219119079	q1	q2	OK	150.891	160.364	0.0878471	0.206895	0.7583	0.99995	no

ENSG00000163467	ENSG00000163467	TSACC	1:156278758-156337664	q1	q2	NOTEST	0.376433	0.343659	-0.131417	0	1	1	no

ENSG00000163468	ENSG00000163468	CCT3	1:156278758-156337664	q1	q2	OK	148.286	146.327	-0.0191864	-0.0453541	0.9459	0.99995	no

ENSG00000163472	ENSG00000163472	TMEM79	1:156252725-156265463	q1	q2	OK	3.56402	3.23785	-0.13847	-0.158242	0.81275	0.99995	no

ENSG00000163479	ENSG00000163479	SSR2	1:155978838-155990750	q1	q2	OK	199.943	189.265	-0.0791814	-0.188815	0.78565	0.99995	no

ENSG00000163481	ENSG00000163481	RNF25	2:219528586-219567439	q1	q2	OK	20.9004	18.5154	-0.174806	-0.161984	0.8016	0.99995	no

ENSG00000163482	ENSG00000163482	STK36	2:219528586-219567439	q1	q2	OK	42.3729	37.7188	-0.167855	-0.218398	0.73715	0.99995	no

ENSG00000163485	ENSG00000163485	ADORA1	1:203059781-203136533	q1	q2	OK	2.80075	1.96571	-0.510761	-0.59311	0.36905	0.99995	no

ENSG00000163486	ENSG00000163486	SRGAP2	1:206516199-206637783	q1	q2	OK	3.08526	3.44488	0.159062	0.267404	0.6627	0.99995	no

ENSG00000163491	ENSG00000163491	NEK10	3:27151575-27410951	q1	q2	OK	0.987917	0.674469	-0.550638	-0.45497	0.3174	0.99995	no

ENSG00000163492	ENSG00000163492	CCDC141	2:179385909-179914813	q1	q2	NOTEST	0.0150181	0.0201696	0.425475	0	1	1	no

ENSG00000163497	ENSG00000163497	FEV	2:219845808-219850379	q1	q2	NOTEST	0.0706045	0.136362	0.949608	0	1	1	no

ENSG00000163499	ENSG00000163499	CRYBA2	2:219854910-219858143	q1	q2	NOTEST	0	0	0	0	1	1	no

ENSG00000163501	ENSG00000163501	IHH	2:219919141-219925189	q1	q2	NOTEST	0.0816536	0.0523159	-0.642269	0	1	1	no

ENSG00000163507	ENSG00000163507	KIAA1524	3:108268715-108308491	q1	q2	OK	18.4335	22.1044	0.262008	0.554566	0.398	0.99995	no

ENSG00000163508	ENSG00000163508	EOMES	3:27757439-27764206	q1	q2	NOTEST	0.0132821	0	#NAME?	0	1	1	no

ENSG00000163510	ENSG00000163510	CWC22	2:180809602-180871840	q1	q2	OK	15.6408	17.1068	0.12926	0.286167	0.68205	0.99995	no

ENSG00000163512	ENSG00000163512	AZI2	3:28283074-28390618	q1	q2	OK	37.1139	37.5715	0.0176787	0.0223214	0.9707	0.99995	no

ENSG00000163513	ENSG00000163513	TGFBR2	3:30647993-30735634	q1	q2	OK	5.27892	6.55999	0.313451	0.605379	0.38795	0.99995	no

ENSG00000163515	ENSG00000163515	RETNLB	3:108462270-108476231	q1	q2	NOTEST	0	0	0	0	1	1	no

ENSG00000163516	ENSG00000163516	ANKZF1	2:220094478-220142892	q1	q2	OK	23.5275	20.3807	-0.20714	-0.261125	0.5808	0.99995	no

ENSG00000163517	ENSG00000163517	HDAC11	3:13518481-13547916	q1	q2	OK	6.34971	4.1218	-0.623417	-0.772376	0.143	0.99995	no

ENSG00000163518	ENSG00000163518	FCRL4	1:157543538-157567870	q1	q2	NOTEST	0	0	0	0	1	1	no

ENSG00000163519	ENSG00000163519	TRAT1	3:108541544-108573852	q1	q2	NOTEST	0	0.0341722	inf	0	1	1	no

ENSG00000163520	ENSG00000163520	FBLN2	3:13573823-13679922	q1	q2	OK	12.2162	11.2852	-0.114361	-0.216353	0.751	0.99995	no

ENSG00000163521	ENSG00000163521	GLB1L	2:220094478-220142892	q1	q2	OK	6.47226	5.36969	-0.269432	-0.169219	0.7194	0.99995	no

ENSG00000163527	ENSG00000163527	STT3B	3:31574129-31679112	q1	q2	OK	67.7313	69.2203	0.0313729	0.0580873	0.91135	0.99995	no

ENSG00000163528	ENSG00000163528	CHCHD4	3:14153579-14166370	q1	q2	OK	10.7406	12.7367	0.245928	0.426295	0.54055	0.99995	no

ENSG00000163530	ENSG00000163530	DPPA2	3:109012634-109035364	q1	q2	OK	0.571636	0.359125	-0.67061	-0.605674	0.3748	0.99995	no

ENSG00000163531	ENSG00000163531	NFASC	1:204797778-204991950	q1	q2	OK	6.29221	6.28668	-0.00126782	-0.00217629	0.9966	0.99995	no

ENSG00000163534	ENSG00000163534	FCRL1	1:157764192-157789895	q1	q2	NOTEST	0	0	0	0	1	1	no

ENSG00000163535	ENSG00000163535	SGOL2	2:201353674-201448505	q1	q2	OK	20.1583	23.173	0.201069	0.412107	0.50985	0.99995	no

ENSG00000163536	ENSG00000163536	SERPINI1	3:167453030-167543356	q1	q2	OK	1.96429	2.67543	0.445764	0.446425	0.4574	0.99995	no

ENSG00000163539	ENSG00000163539	CLASP2	3:33537736-33759848	q1	q2	OK	22.7647	23.5276	0.047556	0.0689602	0.8804	0.99995	no

ENSG00000163541	ENSG00000163541	SUCLG1	2:84650646-84687169	q1	q2	OK	41.0913	39.6076	-0.0530581	-0.108113	0.86975	0.99995	no

ENSG00000163545	ENSG00000163545	NUAK2	1:205271186-205290883	q1	q2	OK	9.39988	9.05001	-0.054723	-0.109501	0.8758	0.99995	no

ENSG00000163554	ENSG00000163554	SPTA1	1:158580495-158656488	q1	q2	NOTEST	0.0030425	0.036628	3.58962	0	1	1	no

ENSG00000163558	ENSG00000163558	PRKCI	3:169940152-170023769	q1	q2	OK	15.8986	17.0466	0.10059	0.103626	0.7583	0.99995	no

ENSG00000163563	ENSG00000163563	MNDA	1:158801106-158819296	q1	q2	NOTEST	0	0	0	0	1	1	no

ENSG00000163564	ENSG00000163564	PYHIN1	1:158900585-158946844	q1	q2	NOTEST	0	0	0	0	1	1	no

ENSG00000163565	ENSG00000163565	IFI16	1:158969757-159024945	q1	q2	OK	5.54873	7.55978	0.446186	0.609644	0.4108	0.99995	no

ENSG00000163568	ENSG00000163568	AIM2	1:159032273-159116886	q1	q2	OK	4.57962	4.78275	0.0626117	0.0764114	0.9069	0.99995	no

ENSG00000163576	ENSG00000163576	EFHB	3:19920963-19988517	q1	q2	OK	0.810266	0.216642	-1.90308	-0.985711	0.1149	0.99995	no

ENSG00000163577	ENSG00000163577	EIF5A2	3:170606203-170626482	q1	q2	OK	1.88776	1.08061	-0.804838	-0.560671	0.2212	0.99995	no

ENSG00000163581	ENSG00000163581	SLC2A2	3:170714136-170744539	q1	q2	NOTEST	0	0	0	0	1	1	no

ENSG00000163584	ENSG00000163584	RPL22L1	3:170582663-170588272	q1	q2	OK	72.1209	76.2371	0.0800754	0.149625	0.83225	0.99995	no

ENSG00000163586	ENSG00000163586	FABP1	2:88422509-88427635	q1	q2	NOTEST	0	0	0	0	1	1	no

ENSG00000163590	ENSG00000163590	PPM1L	3:160473389-160796695	q1	q2	OK	11.7056	12.0207	0.0383289	0.0676849	0.9074	0.99995	no

ENSG00000163596	ENSG00000163596	ICA1L	2:203640689-203736708	q1	q2	OK	6.15459	5.59018	-0.138766	-0.157339	0.69405	0.99995	no

ENSG00000163597	ENSG00000163597	SNHG16	17:74553847-74583038	q1	q2	OK	27.585	27.8864	0.0156808	0.0303045	0.96255	0.99995	no

ENSG00000163599	ENSG00000163599	CTLA4	2:204732508-204738683	q1	q2	OK	0.187719	0.518354	1.46536	0.478497	0.3967	0.99995	no

ENSG00000163600	ENSG00000163600	ICOS	2:204801470-204826300	q1	q2	NOTEST	0	0.00987777	inf	0	1	1	no

ENSG00000163602	ENSG00000163602	RYBP	3:72420975-72496069	q1	q2	OK	10.1702	11.2184	0.141513	0.339147	0.6237	0.99995	no

ENSG00000163605	ENSG00000163605	PPP4R2	3:72937223-73118350	q1	q2	OK	46.3502	52.2045	0.171599	0.308032	0.5741	0.99995	no

ENSG00000163606	ENSG00000163606	CD200R1	3:112640055-112693969	q1	q2	NOTEST	0.0393872	0.0653997	0.731555	0	1	1	no

ENSG00000163607	ENSG00000163607	GTPBP8	3:112709293-112902148	q1	q2	OK	16.3432	15.3775	-0.0878649	-0.0820378	0.9012	0.99995	no

ENSG00000163608	ENSG00000163608	C3orf17	3:112709293-112902148	q1	q2	OK	32.4094	32.7969	0.0171465	0.0254734	0.9639	0.99995	no

ENSG00000163611	ENSG00000163611	SPICE1	3:113161564-113234034	q1	q2	OK	6.16669	6.42889	0.0600738	0.0703271	0.8781	0.99995	no

ENSG00000163612	ENSG00000163612	FAM86KP	4:9155021-9167177	q1	q2	NOTEST	0	0	0	0	1	1	no

ENSG00000163617	ENSG00000163617	KIAA1407	3:113666747-113807269	q1	q2	OK	4.69678	3.4427	-0.448129	-0.389426	0.4635	0.99995	no

ENSG00000163618	ENSG00000163618	CADPS	3:62384021-62861054	q1	q2	OK	3.86894	4.49191	0.215391	0.214239	0.62905	0.99995	no

ENSG00000163623	ENSG00000163623	NKX6-1	4:85413139-85419603	q1	q2	OK	31.7015	28.065	-0.175779	-0.387388	0.57605	0.99995	no

ENSG00000163624	ENSG00000163624	CDS1	4:85504131-85572491	q1	q2	OK	0.424712	0.488262	0.201169	0.193079	0.7418	0.99995	no

ENSG00000163625	ENSG00000163625	WDFY3	4:85590703-85932430	q1	q2	OK	15.4442	15.0785	-0.0345719	-0.0316639	0.9303	0.99995	no

ENSG00000163626	ENSG00000163626	COX18	4:73921796-73935472	q1	q2	OK	8.11475	8.33214	0.0381402	0.0643281	0.9182	0.99995	no

ENSG00000163629	ENSG00000163629	PTPN13	4:87515467-87736324	q1	q2	OK	30.1878	28.7439	-0.0707097	-0.105057	0.81365	0.99995	no

ENSG00000163630	ENSG00000163630	SYNPR	3:63213990-63602597	q1	q2	NOTEST	0.0869567	0.0423749	-1.03709	0	1	1	no

ENSG00000163631	ENSG00000163631	ALB	4:74262830-74287129	q1	q2	OK	0.539089	0.558242	0.0503657	0.0228217	0.94075	0.99995	no

ENSG00000163632	ENSG00000163632	C3orf49	3:63727968-63849579	q1	q2	NOTEST	0.0217317	0.132732	2.61065	0	1	1	no

ENSG00000163633	ENSG00000163633	C4orf36	4:87797357-88062206	q1	q2	OK	1.64883	1.3359	-0.303627	-0.0912375	0.70595	0.99995	no

ENSG00000163634	ENSG00000163634	THOC7	3:63727968-63849579	q1	q2	OK	37.3278	41.9909	0.169827	0.352041	0.61885	0.99995	no

ENSG00000163635	ENSG00000163635	ATXN7	3:63850232-63989138	q1	q2	OK	6.21378	6.75185	0.119811	0.165149	0.70475	0.99995	no

ENSG00000163636	ENSG00000163636	PSMD6	3:63989697-64009658	q1	q2	OK	74.6589	80.0481	0.100553	0.229394	0.73345	0.99995	no

ENSG00000163637	ENSG00000163637	PRICKLE2	3:64053639-64440508	q1	q2	OK	3.2802	3.23743	-0.018932	-0.0293371	0.957	0.99995	no

ENSG00000163638	ENSG00000163638	ADAMTS9	3:64501332-64997143	q1	q2	OK	10.7598	12.4539	0.210946	0.311675	0.65475	0.99995	no

ENSG00000163644	ENSG00000163644	PPM1K	4:89178771-89205921	q1	q2	OK	9.1267	8.24217	-0.147069	-0.217765	0.63975	0.99995	no

ENSG00000163645	ENSG00000163645	FAM194A	3:150377671-150438623	q1	q2	NOTEST	0.0152597	0.0967169	2.66404	0	1	1	no

ENSG00000163646	ENSG00000163646	CLRN1	3:150570270-150798513	q1	q2	NOTEST	0.111409	0.258484	1.21421	0	1	1	no

ENSG00000163655	ENSG00000163655	GMPS	3:155588324-155661815	q1	q2	OK	49.5569	52.9329	0.0950797	0.173349	0.78505	0.99995	no

ENSG00000163659	ENSG00000163659	TIPARP	3:156389650-156424559	q1	q2	OK	5.69846	6.366	0.159814	0.293315	0.6782	0.99995	no

ENSG00000163660	ENSG00000163660	CCNL1	3:156864296-156878549	q1	q2	OK	55.8888	58.2686	0.0601613	0.100259	0.8438	0.99995	no

ENSG00000163661	ENSG00000163661	PTX3	3:156893011-157251408	q1	q2	OK	230.713	241.844	0.0679767	0.151608	0.82875	0.99995	no

ENSG00000163666	ENSG00000163666	HESX1	3:57231943-57260549	q1	q2	OK	2.5505	2.75977	0.113764	0.119714	0.8721	0.99995	no

ENSG00000163673	ENSG00000163673	DCLK3	3:36753912-36781352	q1	q2	NOTEST	0.316161	0.290977	-0.119753	0	1	1	no

ENSG00000163681	ENSG00000163681	SLMAP	3:57741176-57914895	q1	q2	OK	16.8675	16.2045	-0.0578535	-0.0942631	0.85135	0.99995	no

ENSG00000163682	ENSG00000163682	RPL9	4:39455743-39460568	q1	q2	OK	427.692	399.44	-0.0985933	-0.240191	0.74235	0.99995	no

ENSG00000163683	ENSG00000163683	SMIM14	4:39500374-39640710	q1	q2	OK	13.1817	11.0381	-0.256034	-0.188755	0.6639	0.99995	no

ENSG00000163684	ENSG00000163684	RPP14	3:58291973-58310422	q1	q2	OK	15.3233	16.0743	0.0690311	0.103076	0.85585	0.99995	no

ENSG00000163686	ENSG00000163686	ABHD6	3:58223232-58281420	q1	q2	OK	2.77314	2.92373	0.0762909	0.0987766	0.8661	0.99995	no

ENSG00000163687	ENSG00000163687	DNASE1L3	3:58177983-58200424	q1	q2	NOTEST	0	0	0	0	1	1	no

ENSG00000163689	ENSG00000163689	C3orf67	3:58703091-59035810	q1	q2	OK	0.572054	0.254683	-1.16745	-0.535098	0.11955	0.99995	no

ENSG00000163694	ENSG00000163694	RBM47	4:40425271-40632892	q1	q2	NOTEST	0.0659117	0.0834823	0.340935	0	1	1	no

ENSG00000163697	ENSG00000163697	APBB2	4:40812043-41218731	q1	q2	OK	29.9573	31.2293	0.0599917	0.0905514	0.86435	0.99995	no

ENSG00000163701	ENSG00000163701	IL17RE	3:9944295-9958086	q1	q2	OK	0.702796	0.491658	-0.51545	-0.294672	0.465	0.99995	no

ENSG00000163702	ENSG00000163702	IL17RC	3:9958757-9975314	q1	q2	OK	3.63918	2.96243	-0.296833	-0.289999	0.55275	0.99995	no

ENSG00000163703	ENSG00000163703	CRELD1	3:9975505-9987097	q1	q2	OK	14.924	10.395	-0.521755	-0.698313	0.2385	0.99995	no

ENSG00000163704	ENSG00000163704	PRRT3	3:9987225-9996471	q1	q2	OK	2.88521	2.18053	-0.403992	-0.563963	0.41675	0.99995	no

ENSG00000163705	ENSG00000163705	FANCD2OS	3:10068097-10149915	q1	q2	NOTEST	0.134114	0	#NAME?	0	1	1	no

ENSG00000163710	ENSG00000163710	PCOLCE2	3:142534763-142608045	q1	q2	OK	1.11239	1.85983	0.741511	0.573649	0.3037	0.99995	no

ENSG00000163714	ENSG00000163714	U2SURP	3:142668005-142779567	q1	q2	OK	66.4528	77.2892	0.217937	0.38699	0.49455	0.99995	no

ENSG00000163719	ENSG00000163719	MTMR14	3:9691116-9744077	q1	q2	OK	17.3605	15.5935	-0.154856	-0.293024	0.64385	0.99995	no

ENSG00000163728	ENSG00000163728	TTC14	3:180319917-180700541	q1	q2	OK	20.5166	20.4332	-0.00587023	-0.00553362	0.9936	0.99995	no

ENSG00000163734	ENSG00000163734	CXCL3	4:74902305-74904524	q1	q2	NOTEST	0.194584	0.210739	0.115064	0	1	1	no

ENSG00000163735	ENSG00000163735	CXCL5	4:74861358-74864496	q1	q2	NOTEST	0.156654	0.248252	0.664225	0	1	1	no

ENSG00000163736	ENSG00000163736	PPBP	4:74852754-74853914	q1	q2	NOTEST	0	0	0	0	1	1	no

ENSG00000163737	ENSG00000163737	PF4	4:74846793-74847841	q1	q2	NOTEST	0	0	0	0	1	1	no

ENSG00000163738	ENSG00000163738	MTHFD2L	4:74979890-75168816	q1	q2	OK	12.0166	13.5076	0.168743	0.291978	0.6444	0.99995	no

ENSG00000163739	ENSG00000163739	CXCL1	4:74735109-74736959	q1	q2	NOTEST	0.0543873	0.158073	1.53925	0	1	1	no

ENSG00000163743	ENSG00000163743	RCHY1	4:76404246-76475683	q1	q2	OK	19.1093	23.154	0.276985	0.401137	0.53795	0.99995	no

ENSG00000163746	ENSG00000163746	PLSCR2	3:146109149-146213778	q1	q2	NOTEST	0	0	0	0	1	1	no

ENSG00000163749	ENSG00000163749	CCDC158	4:77234153-77343021	q1	q2	NOTEST	0	0.0077394	inf	0	1	1	no

ENSG00000163751	ENSG00000163751	CPA3	3:148508888-148677899	q1	q2	NOTEST	0	0	0	0	1	1	no

ENSG00000163754	ENSG00000163754	GYG1	3:148709127-148745419	q1	q2	OK	19.2625	18.9828	-0.0211052	-0.0429273	0.9471	0.99995	no

ENSG00000163755	ENSG00000163755	HPS3	3:148847370-148939842	q1	q2	OK	9.29608	10.8352	0.221025	0.260607	0.6867	0.99995	no

ENSG00000163762	ENSG00000163762	TM4SF18	3:148947142-149052201	q1	q2	OK	0.973403	1.50408	0.627776	0.494053	0.38565	0.99995	no

ENSG00000163781	ENSG00000163781	TOPBP1	3:133317018-133380737	q1	q2	OK	29.7011	33.0968	0.156176	0.193921	0.64935	0.99995	no

ENSG00000163785	ENSG00000163785	RYK	3:133784146-133969689	q1	q2	OK	32.1338	34.4076	0.0986339	0.168705	0.7405	0.99995	no

ENSG00000163788	ENSG00000163788	SNRK	3:43328003-43775863	q1	q2	OK	6.9129	7.26608	0.0718862	0.0985419	0.865	0.99995	no

ENSG00000163792	ENSG00000163792	TCF23	2:27371871-27376378	q1	q2	NOTEST	0	0	0	0	1	1	no

ENSG00000163793	ENSG00000163793	DNAJC5G	2:27476551-27504367	q1	q2	NOTEST	0.0183782	0	#NAME?	0	1	1	no

ENSG00000163794	ENSG00000163794	UCN	2:27505259-27531313	q1	q2	OK	0.811369	0.682103	-0.250367	-0.215498	0.76315	0.99995	no

ENSG00000163795	ENSG00000163795	ZNF513	2:27600097-27603657	q1	q2	OK	13.2992	10.9719	-0.277522	-0.519516	0.4377	0.99995	no

ENSG00000163798	ENSG00000163798	SLC4A1AP	2:27805896-27917840	q1	q2	OK	11.8536	12.2259	0.0446088	0.0364312	0.95805	0.99995	no

ENSG00000163803	ENSG00000163803	PLB1	2:28680011-28887406	q1	q2	NOTEST	0.369834	0.356787	-0.0518166	0	1	1	no

ENSG00000163806	ENSG00000163806	SPDYA	2:28974505-29093167	q1	q2	OK	0.938915	1.90196	1.01842	0.135089	0.3423	0.99995	no

ENSG00000163807	ENSG00000163807	KIAA1143	3:44779152-44803154	q1	q2	OK	13.7782	13.6344	-0.0151364	-0.0308961	0.95975	0.99995	no

ENSG00000163808	ENSG00000163808	KIF15	3:44803208-44914868	q1	q2	OK	27.3902	29.4407	0.10415	0.162327	0.80525	0.99995	no

ENSG00000163810	ENSG00000163810	TGM4	3:44916099-44956482	q1	q2	NOTEST	0.0537137	0.0614001	0.192952	0	1	1	no

ENSG00000163811	ENSG00000163811	WDR43	2:29117508-29171088	q1	q2	OK	17.9327	20.5923	0.199512	0.393918	0.4948	0.99995	no

ENSG00000163812	ENSG00000163812	ZDHHC3	3:44956748-45077563	q1	q2	OK	27.1548	26.3495	-0.0434351	-0.0636815	0.89555	0.99995	no

ENSG00000163814	ENSG00000163814	CDCP1	3:45123769-45187914	q1	q2	NOTEST	0.0438754	0.012158	-1.85151	0	1	1	no

ENSG00000163815	ENSG00000163815	CLEC3B	3:44956748-45077563	q1	q2	NOTEST	0.290963	0.142764	-1.0272	0	1	1	no

ENSG00000163817	ENSG00000163817	SLC6A20	3:45796941-45838027	q1	q2	NOTEST	0.00495358	0.0434164	3.1317	0	1	1	no

ENSG00000163818	ENSG00000163818	LZTFL1	3:45864807-45957534	q1	q2	OK	11.898	11.2194	-0.0847319	-0.126513	0.82585	0.99995	no

ENSG00000163820	ENSG00000163820	FYCO1	3:45959395-46037316	q1	q2	OK	3.12516	3.13278	0.00351541	0.00444311	0.993	0.99995	no

ENSG00000163823	ENSG00000163823	CCR1	3:46205095-46308197	q1	q2	NOTEST	0.0286352	0.0194974	-0.554507	0	1	1	no

ENSG00000163825	ENSG00000163825	RTP3	3:46538980-46542439	q1	q2	NOTEST	0	0.0206026	inf	0	1	1	no

ENSG00000163827	ENSG00000163827	LRRC2	3:46556912-46668033	q1	q2	NOTEST	0.00510356	0.00503883	-0.0184131	0	1	1	no

ENSG00000163832	ENSG00000163832	ELP6	3:47537129-47555251	q1	q2	OK	33.5927	29.7978	-0.172942	-0.318982	0.62965	0.99995	no

ENSG00000163833	ENSG00000163833	FBXO40	3:121311965-121349139	q1	q2	NOTEST	0.0128782	0.0040078	-1.68405	0	1	1	no

ENSG00000163840	ENSG00000163840	DTX3L	3:122246770-122294050	q1	q2	OK	0.812998	0.851504	0.0667609	0.0740691	0.90995	0.99995	no

ENSG00000163848	ENSG00000163848	ZNF148	3:124801479-125094198	q1	q2	OK	11.3028	12.1174	0.100405	0.160686	0.80425	0.99995	no

ENSG00000163864	ENSG00000163864	NMNAT3	3:139108656-139396859	q1	q2	NOTEST	0.239817	0.208462	-0.202149	0	1	1	no

ENSG00000163866	ENSG00000163866	SMIM12	1:35178337-35325417	q1	q2	OK	32.6772	35.8955	0.135518	0.237102	0.74725	0.99995	no

ENSG00000163867	ENSG00000163867	ZMYM6	1:35439956-35497569	q1	q2	OK	8.27381	8.65989	0.0657965	0.0913643	0.8687	0.99995	no

ENSG00000163870	ENSG00000163870	TPRA1	3:127291911-127341276	q1	q2	OK	11.4322	10.1705	-0.168708	-0.0954575	0.88135	0.99995	no

ENSG00000163872	ENSG00000163872	YEATS2	3:183415605-183530413	q1	q2	OK	27.3339	28.0219	0.0358643	0.0478804	0.9035	0.99995	no

ENSG00000163873	ENSG00000163873	GRIK3	1:37261127-37499730	q1	q2	OK	0.64692	0.753726	0.220451	0.207186	0.6888	0.99995	no

ENSG00000163874	ENSG00000163874	ZC3H12A	1:37940152-37949980	q1	q2	OK	0.728912	0.919676	0.335381	0.283143	0.60975	0.99995	no

ENSG00000163875	ENSG00000163875	MEAF6	1:37958175-37980375	q1	q2	OK	56.3308	57.2266	0.022762	0.0519964	0.9421	0.99995	no

ENSG00000163877	ENSG00000163877	SNIP1	1:38000049-38019903	q1	q2	OK	5.17173	5.19586	0.00671513	0.0128874	0.9855	0.99995	no

ENSG00000163879	ENSG00000163879	DNALI1	1:38022519-38061536	q1	q2	OK	22.3863	17.5493	-0.351199	-0.391014	0.46705	0.99995	no

ENSG00000163882	ENSG00000163882	POLR2H	3:183851806-184402546	q1	q2	OK	50.351	50.3596	0.000244269	6.90E-05	0.99985	0.99995	no

ENSG00000163884	ENSG00000163884	KLF15	3:126061477-126076285	q1	q2	OK	3.71386	3.31869	-0.162304	-0.224528	0.71735	0.99995	no

ENSG00000163885	ENSG00000163885	CCDC37	3:126113781-126155399	q1	q2	NOTEST	0.365438	0.244297	-0.580994	0	1	1	no

ENSG00000163888	ENSG00000163888	CAMK2N2	3:183851806-184402546	q1	q2	OK	2.49366	2.61014	0.0658629	0.00590199	0.9682	0.99995	no

ENSG00000163898	ENSG00000163898	LIPH	3:185224049-185270401	q1	q2	NOTEST	0.0444196	0.0995293	1.16392	0	1	1	no

ENSG00000163900	ENSG00000163900	TMEM41A	3:185000728-185216845	q1	q2	OK	6.5317	6.2921	-0.0539184	-0.0529015	0.93375	0.99995	no

ENSG00000163902	ENSG00000163902	RPN1	3:128338816-128399918	q1	q2	OK	90.6177	83.494	-0.11812	-0.291116	0.67145	0.99995	no

ENSG00000163904	ENSG00000163904	SENP2	3:185300283-185351339	q1	q2	OK	23.4601	24.3835	0.0556957	0.0855458	0.86905	0.99995	no

ENSG00000163909	ENSG00000163909	HEYL	1:40089824-40105617	q1	q2	OK	2.16914	1.52802	-0.505462	-0.685533	0.32	0.99995	no

ENSG00000163913	ENSG00000163913	IFT122	3:129158967-129239198	q1	q2	OK	16.0129	16.6169	0.0534146	0.0821693	0.8572	0.99995	no

ENSG00000163914	ENSG00000163914	RHO	3:129247482-129254012	q1	q2	NOTEST	0	0	0	0	1	1	no

ENSG00000163915	ENSG00000163915	C3orf65	3:185361526-185542844	q1	q2	NOTEST	0.416256	0.218689	-0.928594	0	1	1	no

ENSG00000163918	ENSG00000163918	RFC4	3:186499568-186524847	q1	q2	OK	70.2038	72.6878	0.0501658	0.0371492	0.95115	0.99995	no

ENSG00000163923	ENSG00000163923	RPL39L	3:186838735-186898696	q1	q2	OK	9.48005	8.99245	-0.0761804	-0.103005	0.87975	0.99995	no

ENSG00000163930	ENSG00000163930	BAP1	3:52435028-52444366	q1	q2	OK	42.9357	35.7994	-0.26224	-0.504068	0.4252	0.99995	no

ENSG00000163931	ENSG00000163931	TKT	3:53258722-53290068	q1	q2	NOTEST	0.0571878	0	#NAME?	0	1	1	no

ENSG00000163932	ENSG00000163932	PRKCD	3:53190024-53226733	q1	q2	NOTEST	0	0	0	0	1	1	no

ENSG00000163933	ENSG00000163933	RFT1	3:52937587-53164478	q1	q2	OK	9.9527	10.8092	0.119094	0.141559	0.83005	0.99995	no

ENSG00000163935	ENSG00000163935	SFMBT1	3:52937587-53164478	q1	q2	OK	7.82202	7.49581	-0.0614576	-0.073823	0.8876	0.99995	no

ENSG00000163938	ENSG00000163938	GNL3	3:52529353-52742182	q1	q2	OK	58.9576	68.009	0.206047	0.137683	0.7761	0.99995	no

ENSG00000163939	ENSG00000163939	PBRM1	3:52529353-52742182	q1	q2	OK	37.677	43.3492	0.202321	0.171597	0.67365	0.99995	no

ENSG00000163945	ENSG00000163945	UVSSA	4:1341053-1381837	q1	q2	OK	8.68487	7.65121	-0.182816	-0.221644	0.66965	0.99995	no

ENSG00000163946	ENSG00000163946	FAM208A	3:56591188-56717265	q1	q2	OK	36.6647	40.4804	0.142833	0.181556	0.7649	0.99995	no

ENSG00000163947	ENSG00000163947	ARHGEF3	3:56761445-57114129	q1	q2	OK	1.98976	1.61886	-0.297618	-0.326638	0.51395	0.99995	no

ENSG00000163950	ENSG00000163950	SLBP	4:1694526-1714282	q1	q2	OK	34.8157	38.2764	0.136718	0.311855	0.6583	0.99995	no

ENSG00000163956	ENSG00000163956	LRPAP1	4:3508102-3534286	q1	q2	OK	36.4922	31.7255	-0.201948	-0.317701	0.6609	0.99995	no

ENSG00000163958	ENSG00000163958	ZDHHC19	3:195924319-195938308	q1	q2	NOTEST	0.052471	0	#NAME?	0	1	1	no

ENSG00000163959	ENSG00000163959	SLC51A	3:195938357-196065374	q1	q2	OK	2.09242	2.89684	0.46931	0.153633	0.5452	0.99995	no

ENSG00000163960	ENSG00000163960	UBXN7	3:196074532-196160242	q1	q2	OK	15.7022	17.1707	0.128989	0.273957	0.64065	0.99995	no

ENSG00000163961	ENSG00000163961	RNF168	3:196195653-196230639	q1	q2	OK	12.9783	13.8714	0.0960036	0.226664	0.7402	0.99995	no

ENSG00000163964	ENSG00000163964	PIGX	3:196366556-196462878	q1	q2	OK	16.3043	16.9348	0.0547328	0.0710867	0.9152	0.99995	no

ENSG00000163975	ENSG00000163975	MFI2	3:196715491-196756687	q1	q2	OK	1.1725	0.759294	-0.626851	-0.392107	0.47285	0.99995	no

ENSG00000163982	ENSG00000163982	OTOP1	4:4190529-4228616	q1	q2	NOTEST	0.0142608	0.0138436	-0.0428398	0	1	1	no

ENSG00000163993	ENSG00000163993	S100P	4:6694795-6698897	q1	q2	OK	0.224804	0.731495	1.70218	0.804221	0.2146	0.99995	no

ENSG00000163995	ENSG00000163995	ABLIM2	4:7967038-8160559	q1	q2	OK	4.11904	3.16258	-0.381206	-0.436316	0.4979	0.99995	no

ENSG00000164002	ENSG00000164002	EXO5	1:40974412-40982846	q1	q2	OK	2.04274	2.32729	0.188146	0.184266	0.7549	0.99995	no

ENSG00000164007	ENSG00000164007	CLDN19	1:43198763-43205925	q1	q2	NOTEST	0.323244	0.311751	-0.0522316	0	1	1	no

ENSG00000164008	ENSG00000164008	C1orf50	1:43232939-43263968	q1	q2	OK	14.0012	13.2053	-0.0844376	-0.125882	0.8498	0.99995	no

ENSG00000164010	ENSG00000164010	ERMAP	1:43272722-43312093	q1	q2	OK	3.54412	3.23177	-0.1331	-0.13564	0.82855	0.99995	no

ENSG00000164011	ENSG00000164011	ZNF691	1:43312279-43318148	q1	q2	OK	10.4023	10.0556	-0.0489047	-0.082525	0.90605	0.99995	no

ENSG00000164022	ENSG00000164022	AIMP1	4:106962755-107270383	q1	q2	OK	19.1485	19.2228	0.00558895	0.00882842	0.98935	0.99995	no

ENSG00000164023	ENSG00000164023	SGMS2	4:108745718-108899955	q1	q2	OK	0.531708	0.328824	-0.69332	-0.216228	0.465	0.99995	no

ENSG00000164024	ENSG00000164024	METAP1	4:99916770-99983964	q1	q2	OK	33.3111	35.8527	0.106077	0.209473	0.7185	0.99995	no

ENSG00000164031	ENSG00000164031	DNAJB14	4:100817404-100867883	q1	q2	OK	9.91386	11.9189	0.265737	0.50681	0.37155	0.99995	no

ENSG00000164032	ENSG00000164032	H2AFZ	4:100869242-101136662	q1	q2	OK	755.44	782.078	0.0499953	0.110177	0.87825	0.99995	no

ENSG00000164035	ENSG00000164035	EMCN	4:101316497-101801283	q1	q2	NOTEST	0.12549	0.15659	0.31942	0	1	1	no

ENSG00000164037	ENSG00000164037	SLC9B1	4:103790134-103940896	q1	q2	NOTEST	0.210521	0.102742	-1.03493	0	1	1	no

ENSG00000164038	ENSG00000164038	SLC9B2	4:103941024-104021040	q1	q2	OK	5.45466	5.62484	0.0443215	0.029615	0.94645	0.99995	no

ENSG00000164039	ENSG00000164039	BDH2	4:103941024-104021040	q1	q2	OK	49.7294	46.6767	-0.0913964	-0.18376	0.78785	0.99995	no

ENSG00000164040	ENSG00000164040	PGRMC2	4:129190396-129209984	q1	q2	OK	35.0318	37.6013	0.102116	0.185207	0.78965	0.99995	no

ENSG00000164045	ENSG00000164045	CDC25A	3:48198635-48229892	q1	q2	OK	23.5417	25.7295	0.128203	0.244735	0.6822	0.99995	no

ENSG00000164047	ENSG00000164047	CAMP	3:48264836-48266981	q1	q2	NOTEST	0	0	0	0	1	1	no

ENSG00000164048	ENSG00000164048	ZNF589	3:48282589-48343175	q1	q2	OK	10.3329	9.62429	-0.102489	-0.148608	0.8113	0.99995	no

ENSG00000164049	ENSG00000164049	FBXW12	3:48413708-48442666	q1	q2	OK	0.562845	0.436023	-0.368333	-0.34852	0.5794	0.99995	no

ENSG00000164050	ENSG00000164050	PLXNB1	3:48445260-48471594	q1	q2	OK	71.4892	72.2118	0.0145091	0.0186276	0.96535	0.99995	no

ENSG00000164051	ENSG00000164051	CCDC51	3:48473573-48488060	q1	q2	OK	9.75206	9.05752	-0.106592	-0.0859437	0.9207	0.99995	no

ENSG00000164053	ENSG00000164053	ATRIP	3:48488113-48509044	q1	q2	OK	5.95382	5.97845	0.00595706	0.00713912	0.9894	0.99995	no

ENSG00000164054	ENSG00000164054	SHISA5	3:48509196-48542259	q1	q2	OK	53.7172	48.2063	-0.156164	-0.359733	0.5917	0.99995	no

ENSG00000164056	ENSG00000164056	SPRY1	4:124317949-124324910	q1	q2	OK	237.271	264.455	0.156491	0.291377	0.6558	0.99995	no

ENSG00000164061	ENSG00000164061	BSN	3:49591921-49708978	q1	q2	OK	2.31381	2.2365	-0.0490303	-0.0826252	0.8829	0.99995	no

ENSG00000164062	ENSG00000164062	APEH	3:49711434-49761384	q1	q2	OK	49.826	45.1343	-0.142675	-0.191855	0.75195	0.99995	no

ENSG00000164066	ENSG00000164066	INTU	4:128544425-128647892	q1	q2	OK	6.81952	6.31875	-0.110029	-0.14296	0.7429	0.99995	no

ENSG00000164068	ENSG00000164068	RNF123	3:49711434-49761384	q1	q2	OK	10.6839	8.59428	-0.313983	-0.198455	0.6222	0.99995	no

ENSG00000164070	ENSG00000164070	HSPA4L	4:128702975-128765195	q1	q2	OK	5.53635	7.48158	0.434408	0.366457	0.50945	0.99995	no

ENSG00000164073	ENSG00000164073	MFSD8	4:128838959-128960866	q1	q2	OK	9.6158	8.33353	-0.206479	-0.22311	0.64145	0.99995	no

ENSG00000164074	ENSG00000164074	C4orf29	4:128838959-128960866	q1	q2	OK	5.3665	5.47393	0.0285969	0.0349238	0.96	0.99995	no

ENSG00000164076	ENSG00000164076	CAMKV	3:49895420-49907655	q1	q2	OK	31.1061	33.6121	0.111782	0.190756	0.755	0.99995	no

ENSG00000164077	ENSG00000164077	MON1A	3:49924434-49967606	q1	q2	OK	6.50268	4.76062	-0.449887	-0.704946	0.294	0.99995	no

ENSG00000164078	ENSG00000164078	MST1R	3:49924434-49967606	q1	q2	NOTEST	0.484185	0.300945	-0.68606	0	1	1	no

ENSG00000164080	ENSG00000164080	RAD54L2	3:51575159-51738339	q1	q2	OK	14.1735	14.6135	0.044106	0.0715044	0.8934	0.99995	no

ENSG00000164081	ENSG00000164081	TEX264	3:51575159-51738339	q1	q2	OK	14.673	12.9219	-0.183352	-0.120714	0.8519	0.99995	no

ENSG00000164082	ENSG00000164082	GRM2	3:51741085-51752629	q1	q2	OK	2.49377	2.48999	-0.00218872	-0.00262046	0.9959	0.99995	no

ENSG00000164086	ENSG00000164086	DUSP7	3:52082934-52090566	q1	q2	OK	6.45249	5.94948	-0.117094	-0.216029	0.76585	0.99995	no

ENSG00000164087	ENSG00000164087	POC1A	3:52109268-52188706	q1	q2	OK	10.3858	9.16131	-0.180994	-0.326274	0.6459	0.99995	no

ENSG00000164088	ENSG00000164088	PPM1M	3:52279840-52284613	q1	q2	OK	3.38658	3.16656	-0.0969145	-0.11939	0.8606	0.99995	no

ENSG00000164089	ENSG00000164089	ETNPPL	4:109663195-109684210	q1	q2	NOTEST	0.11022	0.0672559	-0.712653	0	1	1	no

ENSG00000164091	ENSG00000164091	WDR82	3:52288436-52333083	q1	q2	OK	59.2076	61.8942	0.0640232	0.145823	0.82795	0.99995	no

ENSG00000164093	ENSG00000164093	PITX2	4:111538578-111563279	q1	q2	OK	0.611702	0.717729	0.23061	0.197852	0.7302	0.99995	no

ENSG00000164096	ENSG00000164096	C4orf3	4:120218206-120225600	q1	q2	OK	48.1335	49.8553	0.0507063	0.110244	0.86775	0.99995	no

ENSG00000164099	ENSG00000164099	PRSS12	4:119201192-119274158	q1	q2	OK	1.22522	1.07517	-0.188474	-0.170786	0.7355	0.99995	no

ENSG00000164100	ENSG00000164100	NDST3	4:118954772-119179803	q1	q2	OK	4.23734	4.90554	0.211253	0.340146	0.62545	0.99995	no

ENSG00000164104	ENSG00000164104	HMGB2	4:174252845-174256276	q1	q2	OK	395.94	396.062	0.000446858	0.00101899	0.9988	0.99995	no

ENSG00000164105	ENSG00000164105	SAP30	4:174284930-174298683	q1	q2	OK	25.3183	25.6954	0.0213278	0.0405804	0.95115	0.99995	no

ENSG00000164106	ENSG00000164106	SCRG1	4:174305851-174327531	q1	q2	OK	4.84261	4.82447	-0.00541297	-0.00937201	0.9885	0.99995	no

ENSG00000164107	ENSG00000164107	HAND2	4:174446119-174512475	q1	q2	NOTEST	0.0926833	0	#NAME?	0	1	1	no

ENSG00000164109	ENSG00000164109	MAD2L1	4:120976762-121337921	q1	q2	OK	64.5926	73.3442	0.183315	0.351937	0.5904	0.99995	no

ENSG00000164111	ENSG00000164111	ANXA5	4:122589109-122618268	q1	q2	OK	118.629	118.667	0.000453315	0.00106058	0.99875	0.99995	no

ENSG00000164112	ENSG00000164112	TMEM155	4:122680087-122687962	q1	q2	NOTEST	0.292817	0.347095	0.24533	0	1	1	no

ENSG00000164113	ENSG00000164113	ADAD1	4:123300120-123350957	q1	q2	NOTEST	0	0	0	0	1	1	no

ENSG00000164114	ENSG00000164114	MAP9	4:156197840-156302498	q1	q2	OK	11.6328	11.8949	0.0321506	0.0434815	0.93365	0.99995	no

ENSG00000164116	ENSG00000164116	GUCY1A3	4:156587862-156653501	q1	q2	NOTEST	0.289279	0.461563	0.674063	0	1	1	no

ENSG00000164117	ENSG00000164117	FBXO8	4:175157808-175254531	q1	q2	OK	7.98967	7.98037	-0.00167991	-0.00174939	0.9977	0.99995	no

ENSG00000164118	ENSG00000164118	CEP44	4:175157808-175254531	q1	q2	OK	12.244	12.8436	0.0689667	0.0967931	0.8742	0.99995	no

ENSG00000164120	ENSG00000164120	HPGD	4:175411327-175444305	q1	q2	OK	0.498828	0.383933	-0.377686	-0.205359	0.5924	0.99995	no

ENSG00000164122	ENSG00000164122	ASB5	4:177134823-177198722	q1	q2	NOTEST	0	0.00871691	inf	0	1	1	no

ENSG00000164123	ENSG00000164123	C4orf45	4:159690289-159959912	q1	q2	NOTEST	0	0.216045	inf	0	1	1	no

ENSG00000164124	ENSG00000164124	TMEM144	4:159045625-159176563	q1	q2	NOTEST	0.203433	0.123757	-0.717045	0	1	1	no

ENSG00000164125	ENSG00000164125	FAM198B	4:159045625-159176563	q1	q2	OK	2.07133	2.20165	0.0880261	0.119242	0.84635	0.99995	no

ENSG00000164128	ENSG00000164128	NPY1R	4:164245112-164273086	q1	q2	OK	1.35902	0.886683	-0.616072	-0.449759	0.34265	0.99995	no

ENSG00000164129	ENSG00000164129	NPY5R	4:164245112-164273086	q1	q2	NOTEST	0.452139	0.421545	-0.10108	0	1	1	no

ENSG00000164134	ENSG00000164134	NAA15	4:140187316-140397763	q1	q2	OK	21.8064	24.2284	0.15195	0.216391	0.68865	0.99995	no

ENSG00000164136	ENSG00000164136	IL15	4:142557751-142655140	q1	q2	NOTEST	0.324827	0.278129	-0.223917	0	1	1	no

ENSG00000164142	ENSG00000164142	FAM160A1	4:152330367-152584784	q1	q2	NOTEST	0	0.00580443	inf	0	1	1	no

ENSG00000164144	ENSG00000164144	ARFIP1	4:153701088-153839615	q1	q2	OK	20.3795	22.4753	0.141225	0.28117	0.68005	0.99995	no

ENSG00000164151	ENSG00000164151	KIAA0947	5:5420776-5490347	q1	q2	OK	17.0843	17.5603	0.0396462	0.0769083	0.8958	0.99995	no

ENSG00000164161	ENSG00000164161	HHIP	4:145564073-145666423	q1	q2	OK	2.66628	2.63911	-0.0147748	-0.0120774	0.98085	0.99995	no

ENSG00000164162	ENSG00000164162	ANAPC10	4:145888263-146101313	q1	q2	OK	12.7259	15.537	0.287943	0.144484	0.77625	0.99995	no

ENSG00000164163	ENSG00000164163	ABCE1	4:145888263-146101313	q1	q2	OK	39.7245	44.8927	0.176452	0.257125	0.6098	0.99995	no

ENSG00000164164	ENSG00000164164	OTUD4	4:145888263-146101313	q1	q2	OK	11.5665	12.6258	0.126422	0.117791	0.7807	0.99995	no

ENSG00000164167	ENSG00000164167	LSM6	4:147096836-147121152	q1	q2	OK	51.4642	55.5637	0.110573	0.20256	0.77435	0.99995	no

ENSG00000164168	ENSG00000164168	TMEM184C	4:148538533-148605381	q1	q2	OK	16.0763	16.6745	0.0527047	0.0988671	0.8691	0.99995	no

ENSG00000164169	ENSG00000164169	PRMT10	4:148538533-148605381	q1	q2	OK	5.90311	6.06269	0.0384831	0.0280287	0.9538	0.99995	no

ENSG00000164171	ENSG00000164171	ITGA2	5:51971026-52390609	q1	q2	OK	0.961349	0.678217	-0.503314	-0.254893	0.6646	0.99995	no

ENSG00000164172	ENSG00000164172	MOCS2	5:52391508-52410956	q1	q2	OK	24.3415	24.9132	0.0334884	0.0645003	0.9283	0.99995	no

ENSG00000164175	ENSG00000164175	SLC45A2	5:33944720-33984835	q1	q2	NOTEST	0.15686	0.154768	-0.0193707	0	1	1	no

ENSG00000164176	ENSG00000164176	EDIL3	5:83236372-83786583	q1	q2	OK	3.83641	3.82549	-0.00411336	-0.00665104	0.9905	0.99995	no

ENSG00000164180	ENSG00000164180	TMEM161B	5:87485449-87794514	q1	q2	OK	7.66935	8.30202	0.114358	0.158163	0.7825	0.99995	no

ENSG00000164181	ENSG00000164181	ELOVL7	5:60047617-60140216	q1	q2	NOTEST	0.241714	0.187212	-0.368634	0	1	1	no

ENSG00000164182	ENSG00000164182	NDUFAF2	5:60240955-60448853	q1	q2	OK	28.0447	31.3971	0.162906	0.283917	0.68605	0.99995	no

ENSG00000164185	ENSG00000164185	ZNF474	5:121464456-121518353	q1	q2	NOTEST	0.21257	0.255972	0.268052	0	1	1	no

ENSG00000164187	ENSG00000164187	LMBRD2	5:36098513-36152063	q1	q2	OK	3.78372	4.0973	0.114867	0.224705	0.72995	0.99995	no

ENSG00000164188	ENSG00000164188	RANBP3L	5:36248535-36302216	q1	q2	NOTEST	0.346666	0.289803	-0.258475	0	1	1	no

ENSG00000164190	ENSG00000164190	NIPBL	5:36876860-37066515	q1	q2	OK	28.0759	26.8211	-0.0659632	-0.0988841	0.85025	0.99995	no

ENSG00000164197	ENSG00000164197	RNF180	5:63461670-63668696	q1	q2	OK	1.25321	1.06139	-0.239675	-0.260758	0.6702	0.99995	no

ENSG00000164199	ENSG00000164199	GPR98	5:89811427-90460038	q1	q2	OK	28.7428	28.407	-0.016951	-0.0159559	0.9719	0.99995	no

ENSG00000164209	ENSG00000164209	SLC25A46	5:109624933-110100857	q1	q2	OK	22.9969	25.1794	0.130806	0.269446	0.6873	0.99995	no

ENSG00000164211	ENSG00000164211	STARD4	5:110831730-111353006	q1	q2	OK	31.2613	39.4135	0.334311	0.190088	0.64235	0.99995	no

ENSG00000164219	ENSG00000164219	PGGT1B	5:114546526-114599145	q1	q2	OK	10.4988	10.6421	0.0195599	0.0330752	0.9605	0.99995	no

ENSG00000164220	ENSG00000164220	F2RL2	5:75699073-76031606	q1	q2	OK	6.00533	5.44607	-0.141027	-0.0696637	0.9393	0.99995	no

ENSG00000164221	ENSG00000164221	CCDC112	5:114602884-114632528	q1	q2	OK	10.9359	13.024	0.252105	0.455493	0.48535	0.99995	no

ENSG00000164236	ENSG00000164236	ANKRD33B	5:10564441-10650308	q1	q2	NOTEST	0.0365998	0.139496	1.93032	0	1	1	no

ENSG00000164237	ENSG00000164237	CMBL	5:10275986-10308138	q1	q2	OK	13.3497	11.7831	-0.180086	-0.344879	0.59375	0.99995	no

ENSG00000164241	ENSG00000164241	C5orf63	5:126378249-126409184	q1	q2	NOTEST	0.0176712	0.00888614	-0.991772	0	1	1	no

ENSG00000164244	ENSG00000164244	PRRC1	5:126853300-126890781	q1	q2	OK	29.7378	31.434	0.0800309	0.152831	0.8033	0.99995	no

ENSG00000164251	ENSG00000164251	F2RL1	5:76114757-76131140	q1	q2	OK	2.38207	2.09582	-0.1847	-0.25486	0.7009	0.99995	no

ENSG00000164252	ENSG00000164252	AGGF1	5:76325075-76361059	q1	q2	OK	12.4624	13.1455	0.0769818	0.176082	0.79455	0.99995	no

ENSG00000164253	ENSG00000164253	WDR41	5:76506273-76916436	q1	q2	OK	37.4108	40.7736	0.124179	0.190739	0.73345	0.99995	no

ENSG00000164256	ENSG00000164256	PRDM9	5:23507263-23528706	q1	q2	NOTEST	0	0	0	0	1	1	no

ENSG00000164258	ENSG00000164258	NDUFS4	5:52856462-52979168	q1	q2	OK	111.159	111.336	0.00228853	0.00519891	0.9934	0.99995	no

ENSG00000164265	ENSG00000164265	SCGB3A2	5:147250244-147286101	q1	q2	NOTEST	0	0	0	0	1	1	no

ENSG00000164266	ENSG00000164266	SPINK1	5:147204130-147211349	q1	q2	NOTEST	0	0	0	0	1	1	no

ENSG00000164270	ENSG00000164270	HTR4	5:147830594-148056798	q1	q2	NOTEST	0.0474044	0	#NAME?	0	1	1	no

ENSG00000164283	ENSG00000164283	ESM1	5:54273691-54338672	q1	q2	OK	0.442102	0.795977	0.848346	0.580994	0.29495	0.99995	no

ENSG00000164284	ENSG00000164284	GRPEL2	5:148724992-148737205	q1	q2	OK	8.86296	9.06573	0.0326338	0.0626884	0.9221	0.99995	no

ENSG00000164287	ENSG00000164287	CDC20B	5:54408798-54469005	q1	q2	NOTEST	0.0320895	0.025036	-0.358101	0	1	1	no

ENSG00000164291	ENSG00000164291	ARSK	5:94890777-94940768	q1	q2	OK	5.37905	5.95165	0.145938	0.251826	0.6958	0.99995	no

ENSG00000164292	ENSG00000164292	RHOBTB3	5:95036952-95160087	q1	q2	OK	41.0348	42.7583	0.0593545	0.0978695	0.82885	0.99995	no

ENSG00000164294	ENSG00000164294	GPX8	5:54408798-54469005	q1	q2	OK	42.7015	45.3685	0.0874038	0.181353	0.78675	0.99995	no

ENSG00000164296	ENSG00000164296	TIGD6	5:149340299-149432386	q1	q2	OK	3.59613	3.64749	0.0204588	0.0125917	0.98955	0.99995	no

ENSG00000164299	ENSG00000164299	SPZ1	5:79615443-79617661	q1	q2	NOTEST	0	0	0	0	1	1	no

ENSG00000164300	ENSG00000164300	SERINC5	5:79407049-79551898	q1	q2	OK	2.82739	4.17633	0.562766	0.718788	0.29925	0.99995	no

ENSG00000164303	ENSG00000164303	ENPP6	4:185009858-185142383	q1	q2	NOTEST	0.118903	0.102358	-0.216165	0	1	1	no

ENSG00000164304	ENSG00000164304	CAGE1	6:7268538-7418270	q1	q2	NOTEST	0.0698127	0.0155938	-2.16252	0	1	1	no

ENSG00000164305	ENSG00000164305	CASP3	4:185548849-185570663	q1	q2	OK	32.4008	35.7191	0.140663	0.327973	0.6376	0.99995	no

ENSG00000164306	ENSG00000164306	PRIMPOL	4:185570766-185655287	q1	q2	OK	13.9175	14.0429	0.0129443	0.00897927	0.9879	0.99995	no

ENSG00000164307	ENSG00000164307	ERAP1	5:95220801-96143803	q1	q2	OK	8.84753	8.61723	-0.0380505	-0.0449898	0.9378	0.99995	no

ENSG00000164308	ENSG00000164308	ERAP2	5:96149730-96373219	q1	q2	NOTEST	0.460114	0.133095	-1.78953	0	1	1	no

ENSG00000164309	ENSG00000164309	CMYA5	5:78985699-79112013	q1	q2	NOTEST	0.241363	0.21694	-0.153906	0	1	1	no

ENSG00000164318	ENSG00000164318	EGFLAM	5:38258510-38468441	q1	q2	OK	1.86763	2.5087	0.425734	0.41392	0.47025	0.99995	no

ENSG00000164323	ENSG00000164323	KIAA1430	4:186080818-186317053	q1	q2	OK	9.21088	10.4346	0.179963	0.317221	0.6181	0.99995	no

ENSG00000164325	ENSG00000164325	TMEM174	5:72469021-72470970	q1	q2	NOTEST	0	0	0	0	1	1	no

ENSG00000164326	ENSG00000164326	CARTPT	5:71014989-71016875	q1	q2	NOTEST	0.0331692	0	#NAME?	0	1	1	no

ENSG00000164327	ENSG00000164327	RICTOR	5:38845959-39074510	q1	q2	OK	7.77066	7.98343	0.0389703	0.0592906	0.8966	0.99995	no

ENSG00000164329	ENSG00000164329	PAPD4	5:78907942-78982471	q1	q2	OK	19.5252	20.067	0.039488	0.0546763	0.9049	0.99995	no

ENSG00000164330	ENSG00000164330	EBF1	5:158122927-158526769	q1	q2	OK	11.7042	12.4614	0.0904404	0.175668	0.77435	0.99995	no

ENSG00000164331	ENSG00000164331	ANKRA2	5:72848159-72877794	q1	q2	OK	12.6882	11.7161	-0.114992	-0.103629	0.86535	0.99995	no

ENSG00000164332	ENSG00000164332	UBLCP1	5:158690088-158713044	q1	q2	OK	32.9435	35.6471	0.113792	0.253986	0.7045	0.99995	no

ENSG00000164334	ENSG00000164334	FAM170A	5:118788137-118972894	q1	q2	NOTEST	0.0247258	0	#NAME?	0	1	1	no

ENSG00000164338	ENSG00000164338	UTP15	5:72848159-72877794	q1	q2	OK	11.5016	12.279	0.0943647	0.123171	0.82575	0.99995	no

ENSG00000164342	ENSG00000164342	TLR3	4:186990305-187009223	q1	q2	NOTEST	0.224983	0.269011	0.257853	0	1	1	no

ENSG00000164344	ENSG00000164344	KLKB1	4:187112673-187179625	q1	q2	NOTEST	0.163562	0.120982	-0.435042	0	1	1	no

ENSG00000164346	ENSG00000164346	NSA2	5:73923233-74072737	q1	q2	OK	58.507	63.8772	0.126691	0.123208	0.85155	0.99995	no

ENSG00000164347	ENSG00000164347	GFM2	5:73923233-74072737	q1	q2	OK	15.4122	18.4384	0.258645	0.141352	0.8084	0.99995	no

ENSG00000164362	ENSG00000164362	TERT	5:1253261-1295184	q1	q2	NOTEST	0.240127	0.321192	0.41964	0	1	1	no

ENSG00000164363	ENSG00000164363	SLC6A18	5:1225469-1246304	q1	q2	NOTEST	0	0.0128388	inf	0	1	1	no

ENSG00000164366	ENSG00000164366	CCDC127	5:196985-218330	q1	q2	OK	4.69616	4.28144	-0.133385	-0.227021	0.6747	0.99995	no

ENSG00000164379	ENSG00000164379	FOXQ1	6:1312674-1314992	q1	q2	NOTEST	0	0.0108637	inf	0	1	1	no

ENSG00000164385	ENSG00000164385	C6orf195	6:2622146-2634837	q1	q2	NOTEST	0.0218215	0.0102974	-1.08348	0	1	1	no

ENSG00000164393	ENSG00000164393	GPR111	6:47624171-47689757	q1	q2	NOTEST	0.0679488	0.206834	1.60596	0	1	1	no

ENSG00000164398	ENSG00000164398	ACSL6	5:131142682-131348892	q1	q2	NOTEST	0.405693	0.307112	-0.401624	0	1	1	no

ENSG00000164399	ENSG00000164399	IL3	5:131396221-131398897	q1	q2	NOTEST	0	0	0	0	1	1	no

ENSG00000164400	ENSG00000164400	CSF2	5:131409482-131411859	q1	q2	NOTEST	0	0	0	0	1	1	no

ENSG00000164402	ENSG00000164402	Sep-08	5:132083136-132142933	q1	q2	OK	31.3045	31.2534	-0.00235419	-0.00485787	0.99305	0.99995	no

ENSG00000164403	ENSG00000164403	SHROOM1	5:132157832-132166590	q1	q2	OK	2.3776	1.7869	-0.412051	-0.231561	0.54165	0.99995	no

ENSG00000164404	ENSG00000164404	GDF9	5:132196872-132203723	q1	q2	OK	0.609958	0.417391	-0.547309	-0.151834	0.68725	0.99995	no

ENSG00000164405	ENSG00000164405	UQCRQ	5:132196872-132203723	q1	q2	OK	160.779	146.654	-0.132667	-0.258908	0.6972	0.99995	no

ENSG00000164406	ENSG00000164406	LEAP2	5:132208013-132210738	q1	q2	OK	3.48431	3.44375	-0.0168954	-0.0192113	0.9761	0.99995	no

ENSG00000164411	ENSG00000164411	GJB7	6:87992695-88109467	q1	q2	NOTEST	0.0854629	0.124097	0.538099	0	1	1	no

ENSG00000164414	ENSG00000164414	SLC35A1	6:88117700-88222054	q1	q2	OK	14.8181	15.2474	0.0412019	0.0784927	0.91215	0.99995	no

ENSG00000164418	ENSG00000164418	GRIK2	6:101846663-102517958	q1	q2	OK	2.60985	3.2244	0.305065	0.295804	0.5584	0.99995	no

ENSG00000164430	ENSG00000164430	MB21D1	6:74078277-74161999	q1	q2	NOTEST	0.0879629	0.0656387	-0.422348	0	1	1	no

ENSG00000164434	ENSG00000164434	FABP7	6:123100619-123105219	q1	q2	OK	516.001	521.308	0.0147621	0.0344557	0.9597	0.99995	no

ENSG00000164438	ENSG00000164438	TLX3	5:170736287-170739138	q1	q2	NOTEST	0.019363	0.0190579	-0.0229172	0	1	1	no

ENSG00000164440	ENSG00000164440	TXLNB	6:139456248-139613276	q1	q2	OK	0.554225	0.737686	0.412535	0.17926	0.8133	0.99995	no

ENSG00000164442	ENSG00000164442	CITED2	6:139693392-139695757	q1	q2	OK	56.2702	55.2028	-0.027628	-0.0668827	0.9234	0.99995	no

ENSG00000164451	ENSG00000164451	FAM26D	6:116813459-116880031	q1	q2	NOTEST	0.0258172	0.0518356	1.00561	0	1	1	no

ENSG00000164458	ENSG00000164458	T	6:166571143-166582188	q1	q2	NOTEST	0	0	0	0	1	1	no

ENSG00000164463	ENSG00000164463	CREBRF	5:172483354-172566291	q1	q2	OK	2.30916	1.84869	-0.320861	-0.460691	0.40495	0.99995	no

ENSG00000164465	ENSG00000164465	DCBLD1	6:117609462-117923691	q1	q2	OK	5.04043	4.18716	-0.267573	-0.115013	0.7105	0.99995	no

ENSG00000164466	ENSG00000164466	SFXN1	5:174904064-174956745	q1	q2	OK	38.6085	38.3947	-0.00801183	-0.0129547	0.97875	0.99995	no

ENSG00000164483	ENSG00000164483	SAMD3	6:130334843-130686570	q1	q2	NOTEST	0.163954	0.029406	-2.47911	0	1	1	no

ENSG00000164484	ENSG00000164484	TMEM200A	6:130686878-130764208	q1	q2	OK	2.57546	2.60795	0.0180848	0.027478	0.96965	0.99995	no

ENSG00000164485	ENSG00000164485	IL22RA2	6:137464967-137494785	q1	q2	NOTEST	0	0	0	0	1	1	no

ENSG00000164488	ENSG00000164488	DACT2	6:168693509-168720434	q1	q2	NOTEST	0.18909	0.167473	-0.175142	0	1	1	no

ENSG00000164494	ENSG00000164494	PDSS2	6:107473760-107780768	q1	q2	OK	5.87005	8.27378	0.495173	0.331535	0.5127	0.99995	no

ENSG00000164500	ENSG00000164500	C7orf72	7:49813256-50199426	q1	q2	NOTEST	0	0	0	0	1	1	no

ENSG00000164506	ENSG00000164506	STXBP5	6:147163036-147706866	q1	q2	OK	3.2592	3.16729	-0.0412727	-0.0678667	0.91415	0.99995	no

ENSG00000164508	ENSG00000164508	HIST1H2AA	6:25726290-25726790	q1	q2	NOTEST	0	0	0	0	1	1	no

ENSG00000164509	ENSG00000164509	IL31RA	5:55147206-55218678	q1	q2	NOTEST	0.0868076	0.0896645	0.0467167	0	1	1	no

ENSG00000164512	ENSG00000164512	ANKRD55	5:55395506-55529186	q1	q2	NOTEST	0.304756	0.0798334	-1.93259	0	1	1	no

ENSG00000164520	ENSG00000164520	RAET1E	6:150139933-150244257	q1	q2	NOTEST	0	0	0	0	1	1	no

ENSG00000164530	ENSG00000164530	PI16	6:36922208-36932613	q1	q2	NOTEST	0.109919	0.0247888	-2.14868	0	1	1	no

ENSG00000164532	ENSG00000164532	TBX20	7:35242041-35293758	q1	q2	NOTEST	0	0	0	0	1	1	no

ENSG00000164535	ENSG00000164535	DAGLB	7:6448756-6524873	q1	q2	OK	11.8526	10.7385	-0.142414	-0.0902667	0.8683	0.99995	no

ENSG00000164542	ENSG00000164542	KIAA0895	7:36363829-36493400	q1	q2	OK	8.952	9.39262	0.0693169	0.054711	0.91115	0.99995	no

ENSG00000164543	ENSG00000164543	STK17A	7:43622356-43769316	q1	q2	OK	13.711	14.932	0.12307	0.156814	0.81735	0.99995	no

ENSG00000164548	ENSG00000164548	TRA2A	7:23544398-23571660	q1	q2	OK	84.5755	85.7628	0.020113	0.0462932	0.94265	0.99995	no

ENSG00000164556	ENSG00000164556	FAM183B	7:38724945-38726637	q1	q2	NOTEST	0.068044	0.0165301	-2.04137	0	1	1	no

ENSG00000164574	ENSG00000164574	GALNT10	5:153369687-153825410	q1	q2	OK	16.853	17.5203	0.0560203	0.0686329	0.917	0.99995	no

ENSG00000164576	ENSG00000164576	SAP30L	5:153825516-153840614	q1	q2	OK	20.4136	21.1395	0.0504108	0.0511865	0.8969	0.99995	no

ENSG00000164587	ENSG00000164587	RPS14	5:149822752-149829319	q1	q2	OK	979.553	952.685	-0.040124	-0.0831893	0.9097	0.99995	no

ENSG00000164588	ENSG00000164588	HCN1	5:45259348-45696253	q1	q2	OK	7.89438	8.55163	0.115373	0.255788	0.71255	0.99995	no

ENSG00000164591	ENSG00000164591	MYOZ3	5:150040435-150058927	q1	q2	OK	0.767183	0.485468	-0.660195	-0.412846	0.35335	0.99995	no

ENSG00000164597	ENSG00000164597	COG5	7:106809405-107218906	q1	q2	OK	10.548	12.063	0.193627	0.210719	0.69105	0.99995	no

ENSG00000164600	ENSG00000164600	NEUROD6	7:31377074-31380508	q1	q2	NOTEST	0.0122888	0	#NAME?	0	1	1	no

ENSG00000164603	ENSG00000164603	C7orf60	7:112459201-112579971	q1	q2	OK	12.2304	11.7887	-0.053063	-0.0828287	0.87715	0.99995	no

ENSG00000164604	ENSG00000164604	GPR85	7:112718385-112727833	q1	q2	OK	1.47113	0.899908	-0.709076	-0.629864	0.2493	0.99995	no

ENSG00000164609	ENSG00000164609	SLU7	5:159828647-159848718	q1	q2	OK	24.683	29.5998	0.262069	0.343127	0.6005	0.99995	no

ENSG00000164610	ENSG00000164610	RP9	7:33134408-33149013	q1	q2	OK	18.3018	19.5251	0.0933447	0.161047	0.8085	0.99995	no

ENSG00000164611	ENSG00000164611	PTTG1	5:159848828-159855748	q1	q2	OK	141.701	137.052	-0.0481216	-0.110222	0.87245	0.99995	no

ENSG00000164615	ENSG00000164615	CAMLG	5:134074190-134087847	q1	q2	OK	27.1422	25.8253	-0.0717524	-0.13484	0.8485	0.99995	no

ENSG00000164616	ENSG00000164616	FBXL21	5:135257967-135290723	q1	q2	OK	0.989416	1.4161	0.517275	0.403701	0.5292	0.99995	no

ENSG00000164619	ENSG00000164619	BMPER	7:33944522-34195484	q1	q2	OK	0.680311	0.0592272	-3.52186	-0.594452	0.22	0.99995	no

ENSG00000164620	ENSG00000164620	RELL2	5:141016516-141030986	q1	q2	OK	6.02423	5.8632	-0.0390873	-0.046208	0.94745	0.99995	no

ENSG00000164621	ENSG00000164621	SMAD5-AS1	5:135465195-135524435	q1	q2	FAIL	0.229877	0.216592	0	0	1	1	no

ENSG00000164626	ENSG00000164626	KCNK5	6:39156748-39197226	q1	q2	OK	11.241	17.4427	0.633857	1.41718	0.0432	0.99995	no

ENSG00000164627	ENSG00000164627	KIF6	6:39297765-39693181	q1	q2	OK	0.554997	0.315049	-0.816902	-0.551901	0.2577	0.99995	no

ENSG00000164631	ENSG00000164631	ZNF12	7:6703604-6748532	q1	q2	OK	10.9794	12.4234	0.178253	0.403502	0.5608	0.99995	no

ENSG00000164638	ENSG00000164638	SLC29A4	7:5313999-5465045	q1	q2	OK	15.5998	13.3741	-0.222089	-0.214942	0.75355	0.99995	no

ENSG00000164645	ENSG00000164645	C7orf62	7:88388681-88966346	q1	q2	NOTEST	0	0	0	0	1	1	no

ENSG00000164647	ENSG00000164647	STEAP1	7:89511666-89867451	q1	q2	OK	2.42222	3.14767	0.377954	0.345817	0.6056	0.99995	no

ENSG00000164649	ENSG00000164649	CDCA7L	7:21582832-21985702	q1	q2	OK	22.928	23.3181	0.0243349	0.0421242	0.9413	0.99995	no

ENSG00000164651	ENSG00000164651	SP8	7:20823905-20826505	q1	q2	OK	7.68577	6.10623	-0.331909	-0.51634	0.46795	0.99995	no

ENSG00000164654	ENSG00000164654	MIOS	7:7592092-7648560	q1	q2	OK	15.7796	16.2875	0.0457061	0.0807117	0.8833	0.99995	no

ENSG00000164659	ENSG00000164659	KIAA1324L	7:86506221-86689015	q1	q2	OK	14.0385	14.4602	0.042699	0.056517	0.9143	0.99995	no

ENSG00000164663	ENSG00000164663	USP49	6:41737913-41863099	q1	q2	OK	15.9039	15.087	-0.0760745	-0.0602525	0.90955	0.99995	no

ENSG00000164669	ENSG00000164669	INTS4L1	7:64601602-64694600	q1	q2	NOTEST	0.155157	0.375584	1.27541	0	1	1	no

ENSG00000164674	ENSG00000164674	SYTL3	6:159071045-159185908	q1	q2	NOTEST	0.249249	0.172495	-0.53103	0	1	1	no

ENSG00000164675	ENSG00000164675	IQUB	7:123092453-123175131	q1	q2	NOTEST	0.411294	0.307676	-0.418758	0	1	1	no

ENSG00000164683	ENSG00000164683	HEY1	8:80676244-80680098	q1	q2	OK	10.1021	10.7884	0.094832	0.157101	0.79835	0.99995	no

ENSG00000164684	ENSG00000164684	ZNF704	8:81540685-81787016	q1	q2	OK	10.1931	8.34211	-0.289115	-0.263035	0.51705	0.99995	no

ENSG00000164687	ENSG00000164687	FABP5	8:82192105-82197012	q1	q2	OK	56.8971	58.4809	0.0396121	0.0858273	0.9039	0.99995	no

ENSG00000164690	ENSG00000164690	SHH	7:155592679-155604967	q1	q2	NOTEST	0.0226129	0.141261	2.64314	0	1	1	no

ENSG00000164691	ENSG00000164691	TAGAP	6:159393902-159486305	q1	q2	NOTEST	0.0609414	0.144844	1.24901	0	1	1	no

ENSG00000164692	ENSG00000164692	COL1A2	7:94023872-94060544	q1	q2	OK	16.1107	15.6681	-0.0401938	-0.0614741	0.91625	0.99995	no

ENSG00000164694	ENSG00000164694	FNDC1	6:159586940-159693141	q1	q2	OK	1.01718	0.899389	-0.177557	-0.229379	0.73445	0.99995	no

ENSG00000164695	ENSG00000164695	CHMP4C	8:82613568-82671750	q1	q2	NOTEST	0	0	0	0	1	1	no

ENSG00000164707	ENSG00000164707	SLC13A4	7:135347243-135433594	q1	q2	OK	0.985879	1.05397	0.0963504	0.0371673	0.9422	0.99995	no

ENSG00000164708	ENSG00000164708	PGAM2	7:44084238-44109055	q1	q2	NOTEST	0.215278	0.171227	-0.330293	0	1	1	no

ENSG00000164713	ENSG00000164713	BRI3	7:97881690-98030380	q1	q2	OK	57.2797	50.4686	-0.182639	-0.288964	0.668	0.99995	no

ENSG00000164715	ENSG00000164715	LMTK2	7:97736196-97838945	q1	q2	OK	7.59274	7.78512	0.0360985	0.0851236	0.90275	0.99995	no

ENSG00000164729	ENSG00000164729	SLC35G3	17:33519538-33521412	q1	q2	NOTEST	0	0	0	0	1	1	no

ENSG00000164733	ENSG00000164733	CTSB	8:11700032-11726957	q1	q2	OK	56.0796	53.9037	-0.0570922	-0.117362	0.84995	0.99995	no

ENSG00000164736	ENSG00000164736	SOX17	8:55370494-55373448	q1	q2	NOTEST	0	0.0437407	inf	0	1	1	no

ENSG00000164740	ENSG00000164740	SEPT7P5	7:62956176-62962914	q1	q2	NOTEST	0	0	0	0	1	1	no

ENSG00000164741	ENSG00000164741	DLC1	8:12940869-13373167	q1	q2	OK	6.69938	7.99369	0.254833	0.275056	0.5098	0.99995	no

ENSG00000164742	ENSG00000164742	ADCY1	7:45613738-45762715	q1	q2	OK	3.73185	4.05555	0.120007	0.222751	0.6982	0.99995	no

ENSG00000164743	ENSG00000164743	C8orf48	8:13415165-13462119	q1	q2	OK	1.31382	1.23021	-0.0948612	-0.104802	0.8771	0.99995	no

ENSG00000164744	ENSG00000164744	SUN3	7:48026744-48068716	q1	q2	NOTEST	0	0.0847303	inf	0	1	1	no

ENSG00000164746	ENSG00000164746	C7orf57	7:48075107-48100901	q1	q2	NOTEST	0.157057	0.0256446	-2.61456	0	1	1	no

ENSG00000164749	ENSG00000164749	HNF4G	8:76320148-76479078	q1	q2	NOTEST	0.326205	0.307974	-0.0829708	0	1	1	no

ENSG00000164751	ENSG00000164751	PEX2	8:77892493-77913280	q1	q2	OK	29.2173	30.1181	0.0438067	0.0695516	0.91705	0.99995	no

ENSG00000164754	ENSG00000164754	RAD21	8:117654368-117889107	q1	q2	OK	125.132	133.423	0.0925511	0.156292	0.80115	0.99995	no

ENSG00000164756	ENSG00000164756	SLC30A8	8:117962511-118188953	q1	q2	NOTEST	0.246517	0.385338	0.644439	0	1	1	no

ENSG00000164758	ENSG00000164758	MED30	8:118532951-118552501	q1	q2	OK	15.0826	15.7063	0.0584513	0.0952478	0.8874	0.99995	no

ENSG00000164761	ENSG00000164761	TNFRSF11B	8:119935795-119964439	q1	q2	OK	1.03507	1.19876	0.211802	0.190616	0.7766	0.99995	no

ENSG00000164764	ENSG00000164764	SBSPON	8:73976774-74036323	q1	q2	OK	0.869991	1.15083	0.403607	0.497149	0.464	0.99995	no

ENSG00000164776	ENSG00000164776	PHKG1	7:56148439-56160689	q1	q2	NOTEST	0.230512	0.120994	-0.929903	0	1	1	no

ENSG00000164778	ENSG00000164778	EN2	7:155250823-155257526	q1	q2	OK	30.9472	39.0603	0.335894	0.822557	0.23595	0.99995	no

ENSG00000164794	ENSG00000164794	KCNV1	8:110975873-110988076	q1	q2	NOTEST	0.0899819	0.0792582	-0.183073	0	1	1	no

ENSG00000164796	ENSG00000164796	CSMD3	8:113235156-114449328	q1	q2	OK	1.18093	0.997059	-0.244173	-0.179307	0.75555	0.99995	no

ENSG00000164808	ENSG00000164808	SPIDR	8:48173166-48648868	q1	q2	OK	19.7129	20.3206	0.043806	0.0650561	0.9019	0.99995	no

ENSG00000164815	ENSG00000164815	ORC5	7:103766787-103848495	q1	q2	OK	14.3384	14.6786	0.0338297	0.0647047	0.9222	0.99995	no

ENSG00000164816	ENSG00000164816	DEFA5	8:6912830-6914256	q1	q2	NOTEST	0	0	0	0	1	1	no

ENSG00000164818	ENSG00000164818	HEATR2	7:588833-829190	q1	q2	OK	15.4184	14.6179	-0.0769175	-0.125929	0.82995	0.99995	no

ENSG00000164821	ENSG00000164821	DEFA4	8:6793343-6795860	q1	q2	NOTEST	0	0	0	0	1	1	no

ENSG00000164822	ENSG00000164822	DEFA6	8:6782214-6783598	q1	q2	NOTEST	0	0	0	0	1	1	no

ENSG00000164823	ENSG00000164823	OSGIN2	8:90914086-90940116	q1	q2	OK	3.44289	3.42575	-0.00720052	-0.0115273	0.98625	0.99995	no

ENSG00000164825	ENSG00000164825	DEFB1	8:6666037-6742798	q1	q2	NOTEST	0	0	0	0	1	1	no

ENSG00000164828	ENSG00000164828	SUN1	7:855527-936073	q1	q2	OK	42.4432	40.7899	-0.0573204	-0.0793132	0.87305	0.99995	no

ENSG00000164830	ENSG00000164830	OXR1	8:107282371-107764922	q1	q2	OK	16.844	16.7041	-0.0120362	-0.0224165	0.97305	0.99995	no

ENSG00000164841	ENSG00000164841	TMEM74	8:109593290-109799844	q1	q2	OK	0.775649	1.13446	0.548533	0.596669	0.3621	0.99995	no

ENSG00000164845	ENSG00000164845	FAM86FP	12:8385107-8395544	q1	q2	NOTEST	0.102914	0.19123	0.893872	0	1	1	no

ENSG00000164849	ENSG00000164849	GPR146	7:1036621-1177896	q1	q2	NOTEST	0.170727	0	#NAME?	0	1	1	no

ENSG00000164850	ENSG00000164850	GPER1	7:1036621-1177896	q1	q2	OK	0.856094	0.735605	-0.218838	-0.0941189	0.89375	0.99995	no

ENSG00000164853	ENSG00000164853	UNCX	7:1272542-1276954	q1	q2	NOTEST	0.327326	0.219774	-0.574708	0	1	1	no

ENSG00000164855	ENSG00000164855	TMEM184A	7:1570349-1600457	q1	q2	NOTEST	0.0535593	0.0628754	0.231361	0	1	1	no

ENSG00000164867	ENSG00000164867	NOS3	7:150688082-150721586	q1	q2	NOTEST	0.257197	0.320899	0.319247	0	1	1	no

ENSG00000164871	ENSG00000164871	SPAG11B	8:7300205-7321196	q1	q2	NOTEST	0	0	0	0	1	1	no

ENSG00000164877	ENSG00000164877	MICALL2	7:1468100-1499138	q1	q2	OK	5.94733	4.3703	-0.444509	-0.476852	0.3914	0.99995	no

ENSG00000164879	ENSG00000164879	CA3	8:86239836-86393722	q1	q2	NOTEST	0.394913	0.300918	-0.392167	0	1	1	no

ENSG00000164880	ENSG00000164880	INTS1	7:1509912-1545489	q1	q2	OK	21.0548	19.2453	-0.129649	-0.18815	0.65845	0.99995	no

ENSG00000164885	ENSG00000164885	CDK5	7:150750898-150773617	q1	q2	OK	12.5626	11.7946	-0.0910039	-0.0758499	0.9062	0.99995	no

ENSG00000164889	ENSG00000164889	SLC4A2	7:150750898-150773617	q1	q2	OK	39.3812	35.9094	-0.133145	-0.151619	0.752	0.99995	no

ENSG00000164893	ENSG00000164893	SLC7A13	8:87226280-87333375	q1	q2	NOTEST	0	0	0	0	1	1	no

ENSG00000164896	ENSG00000164896	FASTK	7:150773710-150777953	q1	q2	OK	35.5759	33.5222	-0.0857829	-0.146669	0.8041	0.99995	no

ENSG00000164897	ENSG00000164897	TMUB1	7:150778166-150780633	q1	q2	OK	18.9534	17.6599	-0.101979	-0.182873	0.7861	0.99995	no

ENSG00000164898	ENSG00000164898	C7orf55	7:139024202-139108198	q1	q2	OK	19.9556	18.062	-0.143837	-0.0669927	0.8781	0.99995	no

ENSG00000164900	ENSG00000164900	GBX1	7:150845675-150871832	q1	q2	OK	0.947234	0.625595	-0.598493	-0.524291	0.44595	0.99995	no

ENSG00000164902	ENSG00000164902	PHAX	5:125935959-125962944	q1	q2	OK	44.8857	48.6748	0.11692	0.20156	0.77305	0.99995	no

ENSG00000164904	ENSG00000164904	ALDH7A1	5:125877532-125931110	q1	q2	OK	101.171	96.9891	-0.0609047	-0.117215	0.85665	0.99995	no

ENSG00000164916	ENSG00000164916	FOXK1	7:4683387-4811074	q1	q2	OK	10.5414	9.27506	-0.184632	-0.309178	0.5224	0.99995	no

ENSG00000164919	ENSG00000164919	COX6C	8:100025493-100906290	q1	q2	OK	215.001	210.194	-0.0326153	-0.0487415	0.94425	0.99995	no

ENSG00000164920	ENSG00000164920	OSR2	8:99956630-99964332	q1	q2	OK	8.95809	8.93595	-0.00357068	-0.00535959	0.9946	0.99995	no

ENSG00000164924	ENSG00000164924	YWHAZ	8:101928752-101965616	q1	q2	OK	367.968	407.57	0.147467	0.256261	0.69125	0.99995	no

ENSG00000164929	ENSG00000164929	BAALC	8:104133259-104345094	q1	q2	OK	104.921	155.918	0.571491	0.998052	0.0691	0.99995	no

ENSG00000164930	ENSG00000164930	FZD6	8:104133259-104345094	q1	q2	OK	6.85793	9.53505	0.475466	0.266154	0.6966	0.99995	no

ENSG00000164932	ENSG00000164932	CTHRC1	8:104383742-104395225	q1	q2	OK	0.190244	0.514151	1.43434	0.728943	0.1529	0.99995	no

ENSG00000164933	ENSG00000164933	SLC25A32	8:104410862-104455681	q1	q2	OK	9.27295	12.3401	0.412249	0.459761	0.4689	0.99995	no

ENSG00000164934	ENSG00000164934	DCAF13	8:104410862-104455681	q1	q2	OK	41.4529	47.321	0.191007	0.288524	0.6572	0.99995	no

ENSG00000164935	ENSG00000164935	DCSTAMP	8:105342551-105479281	q1	q2	NOTEST	0.190709	0.0328358	-2.53803	0	1	1	no

ENSG00000164938	ENSG00000164938	TP53INP1	8:95825538-96128683	q1	q2	OK	22.3459	19.5564	-0.192368	-0.330602	0.63755	0.99995	no

ENSG00000164941	ENSG00000164941	INTS8	8:95825538-96128683	q1	q2	OK	33.1394	34.2665	0.048253	0.0394749	0.9128	0.99995	no

ENSG00000164944	ENSG00000164944	KIAA1429	8:95499920-95565757	q1	q2	OK	23.7842	25.1469	0.0803795	0.105634	0.8308	0.99995	no

ENSG00000164946	ENSG00000164946	FREM1	9:14734663-14910993	q1	q2	OK	19.5392	26.0539	0.415126	0.549716	0.3481	0.99995	no

ENSG00000164949	ENSG00000164949	GEM	8:95261480-95274578	q1	q2	OK	1.49193	1.61048	0.110308	0.116125	0.8491	0.99995	no

ENSG00000164951	ENSG00000164951	PDP1	8:94870034-94938294	q1	q2	OK	8.18547	8.42145	0.041005	0.0807647	0.90005	0.99995	no

ENSG00000164953	ENSG00000164953	TMEM67	8:94767071-94831462	q1	q2	OK	12.9816	12.3922	-0.0670346	-0.0705583	0.89185	0.99995	no

ENSG00000164961	ENSG00000164961	KIAA0196	8:126036501-126379362	q1	q2	OK	15.9202	17.2396	0.114868	0.11339	0.77905	0.99995	no

ENSG00000164967	ENSG00000164967	RPP25L	9:34610482-34612101	q1	q2	OK	17.5475	14.1025	-0.315314	-0.514028	0.467	0.99995	no

ENSG00000164970	ENSG00000164970	FAM219A	9:34398181-34520982	q1	q2	OK	18.634	15.9972	-0.220115	-0.492996	0.4757	0.99995	no

ENSG00000164972	ENSG00000164972	C9orf24	9:34379016-34397830	q1	q2	NOTEST	0.42295	0.327517	-0.368916	0	1	1	no

ENSG00000164975	ENSG00000164975	SNAPC3	9:15422701-15511017	q1	q2	OK	27.968	29.7354	0.0884065	0.090937	0.8965	0.99995	no

ENSG00000164976	ENSG00000164976	KIAA1161	9:34366667-34376851	q1	q2	OK	4.13877	3.34695	-0.306356	-0.581794	0.3986	0.99995	no

ENSG00000164978	ENSG00000164978	NUDT2	9:34329503-34343709	q1	q2	OK	23.3875	22.4938	-0.0562068	-0.101888	0.88445	0.99995	no

ENSG00000164983	ENSG00000164983	TMEM65	8:125324230-125384933	q1	q2	OK	15.644	18.2704	0.223897	0.509481	0.46775	0.99995	no

ENSG00000164985	ENSG00000164985	PSIP1	9:15422701-15511017	q1	q2	OK	123.462	127.527	0.046743	0.0574888	0.9055	0.99995	no

ENSG00000164989	ENSG00000164989	CCDC171	9:15552894-16061661	q1	q2	OK	2.72764	2.83756	0.0569992	0.0541913	0.92825	0.99995	no

ENSG00000165006	ENSG00000165006	UBAP1	9:34179002-34329198	q1	q2	OK	25.1115	24.7883	-0.0186901	-0.0343307	0.96155	0.99995	no

ENSG00000165023	ENSG00000165023	DIRAS2	9:93372113-93405386	q1	q2	OK	0.850618	0.552824	-0.621692	-0.750034	0.2779	0.99995	no

ENSG00000165025	ENSG00000165025	SYK	9:93564068-93660831	q1	q2	NOTEST	0.0206878	0.0149812	-0.465632	0	1	1	no

ENSG00000165028	ENSG00000165028	NIPSNAP3B	9:107526437-107539738	q1	q2	OK	0.585596	0.650668	0.152016	0.136243	0.84295	0.99995	no

ENSG00000165029	ENSG00000165029	ABCA1	9:107543282-107691173	q1	q2	OK	6.50036	5.54926	-0.228225	-0.51244	0.44585	0.99995	no

ENSG00000165030	ENSG00000165030	NFIL3	9:94171326-94186144	q1	q2	OK	7.99864	7.48254	-0.0962264	-0.169449	0.8093	0.99995	no

ENSG00000165046	ENSG00000165046	LETM2	8:38243724-38267045	q1	q2	OK	5.08138	5.67572	0.159585	0.222676	0.7229	0.99995	no

ENSG00000165055	ENSG00000165055	METTL2B	7:128095893-128146656	q1	q2	OK	15.0337	15.4862	0.0427802	0.0491879	0.92	0.99995	no

ENSG00000165059	ENSG00000165059	PRKACG	9:71627468-71629039	q1	q2	NOTEST	0.0182598	0.0168213	-0.118379	0	1	1	no

ENSG00000165060	ENSG00000165060	FXN	9:71650174-71715094	q1	q2	OK	13.0228	16.2634	0.320588	0.449964	0.50645	0.99995	no

ENSG00000165061	ENSG00000165061	ZMAT4	8:40388108-40755352	q1	q2	OK	7.55295	8.19668	0.118	0.179924	0.7492	0.99995	no

ENSG00000165066	ENSG00000165066	NKX6-3	8:41502696-41508855	q1	q2	NOTEST	0.0826396	0.0798737	-0.0491122	0	1	1	no

ENSG00000165071	ENSG00000165071	TMEM71	8:133697252-133772958	q1	q2	OK	1.45189	1.74843	0.268129	0.238225	0.63715	0.99995	no

ENSG00000165072	ENSG00000165072	MAMDC2	9:72658496-72873782	q1	q2	OK	1.44257	1.12131	-0.363465	-0.281379	0.7067	0.99995	no

ENSG00000165076	ENSG00000165076	PRSS37	7:141536085-141541287	q1	q2	NOTEST	0.139636	0.0734655	-0.926534	0	1	1	no

ENSG00000165078	ENSG00000165078	CPA6	8:68334359-68658620	q1	q2	NOTEST	0.0285731	0.0356568	0.319518	0	1	1	no

ENSG00000165084	ENSG00000165084	C8orf34	8:69215702-69731257	q1	q2	NOTEST	0.0452495	0.0821621	0.860572	0	1	1	no

ENSG00000165091	ENSG00000165091	TMC1	9:75136716-75451267	q1	q2	NOTEST	0.0449113	0.0681759	0.602186	0	1	1	no

ENSG00000165092	ENSG00000165092	ALDH1A1	9:75515577-75695358	q1	q2	NOTEST	0.139633	0.0379371	-1.87996	0	1	1	no

ENSG00000165097	ENSG00000165097	KDM1B	6:18155559-18224084	q1	q2	OK	6.67921	7.6605	0.197761	0.407769	0.5614	0.99995	no

ENSG00000165102	ENSG00000165102	HGSNAT	8:42995555-43057998	q1	q2	OK	22.3415	21.6133	-0.0478018	-0.0792028	0.88855	0.99995	no

ENSG00000165105	ENSG00000165105	RASEF	9:85594499-85678092	q1	q2	NOTEST	0.0875651	0.128313	0.551238	0	1	1	no

ENSG00000165113	ENSG00000165113	GKAP1	9:86354335-86444431	q1	q2	OK	8.20814	8.1906	-0.00308493	-0.00496251	0.99385	0.99995	no

ENSG00000165115	ENSG00000165115	KIF27	9:86444570-86536342	q1	q2	OK	2.51395	3.05558	0.281493	0.347879	0.57065	0.99995	no

ENSG00000165118	ENSG00000165118	C9orf64	9:86553225-86571901	q1	q2	NOTEST	0	0.0583418	inf	0	1	1	no

ENSG00000165119	ENSG00000165119	HNRNPK	9:86582997-86595569	q1	q2	OK	463.283	503.089	0.11892	0.21517	0.73475	0.99995	no

ENSG00000165120	ENSG00000165120	SSMEM1	7:129847699-129856683	q1	q2	NOTEST	0.024746	0	#NAME?	0	1	1	no

ENSG00000165121	ENSG00000165121	RP11-213G2.3	9:88430880-88464426	q1	q2	OK	1.31617	1.12867	-0.221725	-0.248362	0.71605	0.99995	no

ENSG00000165124	ENSG00000165124	SVEP1	9:113127530-113342160	q1	q2	OK	2.58327	2.68979	0.0582922	0.0921028	0.88085	0.99995	no

ENSG00000165125	ENSG00000165125	TRPV6	7:142568955-142590199	q1	q2	NOTEST	0.107241	0.0698281	-0.618974	0	1	1	no

ENSG00000165131	ENSG00000165131	C7orf34	7:142636439-142637955	q1	q2	NOTEST	0	0	0	0	1	1	no

ENSG00000165138	ENSG00000165138	ANKS6	9:101493610-101559247	q1	q2	OK	9.52433	8.58397	-0.149972	-0.18917	0.72355	0.99995	no

ENSG00000165140	ENSG00000165140	FBP1	9:97365414-97402531	q1	q2	NOTEST	0.0187948	0.018274	-0.0405434	0	1	1	no

ENSG00000165152	ENSG00000165152	TMEM246	9:104230720-104295819	q1	q2	OK	5.28168	5.38485	0.0279098	0.0535289	0.9384	0.99995	no

ENSG00000165156	ENSG00000165156	ZHX1	8:124232195-124287781	q1	q2	OK	17.9344	19.3583	0.110221	0.162404	0.79415	0.99995	no

ENSG00000165164	ENSG00000165164	CXorf22	X:35937850-36008269	q1	q2	NOTEST	0.0680954	0.0437453	-0.638429	0	1	1	no

ENSG00000165168	ENSG00000165168	CYBB	X:37208527-38548169	q1	q2	NOTEST	0.00575418	0.0393956	2.77535	0	1	1	no

ENSG00000165169	ENSG00000165169	DYNLT3	X:37208527-38548169	q1	q2	OK	0.80834	0.942232	0.22112	0.0847476	0.9141	0.99995	no

ENSG00000165171	ENSG00000165171	WBSCR27	7:73248919-73256865	q1	q2	NOTEST	0	0	0	0	1	1	no

ENSG00000165175	ENSG00000165175	MID1IP1	X:38660684-38665790	q1	q2	OK	13.5762	13.0709	-0.054721	-0.108924	0.8792	0.99995	no

ENSG00000165178	ENSG00000165178	NCF1C	7:74572444-74587848	q1	q2	NOTEST	0	0	0	0	1	1	no

ENSG00000165181	ENSG00000165181	C9orf84	9:114448452-114557288	q1	q2	NOTEST	0.0740453	0.0614623	-0.268706	0	1	1	no

ENSG00000165182	ENSG00000165182	CXorf58	X:23851469-23957624	q1	q2	NOTEST	0.095931	0.0356853	-1.42667	0	1	1	no

ENSG00000165185	ENSG00000165185	KIAA1958	9:115142216-115431677	q1	q2	OK	12.3167	11.2509	-0.130577	-0.172363	0.81555	0.99995	no

ENSG00000165186	ENSG00000165186	PTCHD1	X:23352132-23422489	q1	q2	NOTEST	0.0896259	0.0665285	-0.429943	0	1	1	no

ENSG00000165188	ENSG00000165188	RNF183	9:116059372-116065656	q1	q2	NOTEST	0.153249	0.120786	-0.343428	0	1	1	no

ENSG00000165192	ENSG00000165192	ASB11	X:15298052-15333778	q1	q2	NOTEST	0	0.0178789	inf	0	1	1	no

ENSG00000165194	ENSG00000165194	PCDH19	X:99546641-99665271	q1	q2	OK	31.9457	30.6458	-0.0599316	-0.141252	0.84235	0.99995	no

ENSG00000165195	ENSG00000165195	PIGA	X:15337572-15353676	q1	q2	OK	6.48435	6.59619	0.0246711	0.0368871	0.948	0.99995	no

ENSG00000165197	ENSG00000165197	FIGF	X:15363712-15402498	q1	q2	NOTEST	0.454169	0.244376	-0.894128	0	1	1	no

ENSG00000165202	ENSG00000165202	OR1Q1	9:125372193-125401964	q1	q2	NOTEST	0	0	0	0	1	1	no

ENSG00000165204	ENSG00000165204	OR1K1	9:125560667-125590910	q1	q2	NOTEST	0	0	0	0	1	1	no

ENSG00000165209	ENSG00000165209	STRBP	9:125871772-126030855	q1	q2	OK	25.5337	25.815	0.0158068	0.0271896	0.9653	0.99995	no

ENSG00000165215	ENSG00000165215	CLDN3	7:73183327-73184600	q1	q2	NOTEST	0	0	0	0	1	1	no

ENSG00000165219	ENSG00000165219	GAPVD1	9:128024072-128129486	q1	q2	OK	15.6481	16.2591	0.0552671	0.0580854	0.8655	0.99995	no

ENSG00000165233	ENSG00000165233	C9orf89	9:95858499-95875565	q1	q2	OK	12.0638	10.5069	-0.199341	-0.247538	0.7239	0.99995	no

ENSG00000165238	ENSG00000165238	WNK2	9:95947197-96108696	q1	q2	OK	10.1704	10.9597	0.10783	0.136576	0.79135	0.99995	no

ENSG00000165240	ENSG00000165240	ATP7A	X:77166193-77305892	q1	q2	NOTEST	0.0255015	0.00613181	-2.0562	0	1	1	no

ENSG00000165244	ENSG00000165244	ZNF367	9:99148222-99180611	q1	q2	OK	7.49194	7.60389	0.0213974	0.0426483	0.9503	0.99995	no

ENSG00000165246	ENSG00000165246	NLGN4Y	Y:16634517-16957530	q1	q2	OK	37.1262	44.4493	0.259724	0.358478	0.5593	0.99995	no

ENSG00000165259	ENSG00000165259	HDX	X:83572881-83757487	q1	q2	OK	5.13073	5.33964	0.057578	0.0882182	0.8937	0.99995	no

ENSG00000165264	ENSG00000165264	NDUFB6	9:32540541-32573160	q1	q2	OK	40.8824	43.3162	0.0834244	0.105654	0.87505	0.99995	no

ENSG00000165269	ENSG00000165269	AQP7	9:33384764-33402643	q1	q2	NOTEST	0	0	0	0	1	1	no

ENSG00000165271	ENSG00000165271	NOL6	9:33461438-33473928	q1	q2	OK	12.044	11.7081	-0.0408005	-0.0830652	0.90685	0.99995	no

ENSG00000165272	ENSG00000165272	AQP3	9:33441151-33447609	q1	q2	OK	0.510112	0.684725	0.424711	0.393689	0.52005	0.99995	no

ENSG00000165275	ENSG00000165275	TRMT10B	9:37510888-38069210	q1	q2	OK	7.76619	8.6568	0.156627	0.0790155	0.8546	0.99995	no

ENSG00000165280	ENSG00000165280	VCP	9:35056060-35073246	q1	q2	OK	151.548	147.096	-0.0430223	-0.0732484	0.8862	0.99995	no

ENSG00000165282	ENSG00000165282	PIGO	9:35088684-35098138	q1	q2	OK	9.02605	7.62012	-0.244281	-0.40323	0.5208	0.99995	no

ENSG00000165283	ENSG00000165283	STOML2	9:35099887-35103154	q1	q2	OK	71.3228	67.4911	-0.0796656	-0.175931	0.78925	0.99995	no

ENSG00000165288	ENSG00000165288	BRWD3	X:79926352-80065187	q1	q2	OK	6.38419	6.91189	0.114577	0.164526	0.69995	0.99995	no

ENSG00000165300	ENSG00000165300	SLITRK5	13:88324869-88331871	q1	q2	OK	5.40005	5.08028	-0.0880645	-0.164624	0.81485	0.99995	no

ENSG00000165304	ENSG00000165304	MELK	9:36572858-36677678	q1	q2	OK	40.4187	42.632	0.0769126	0.178922	0.7906	0.99995	no

ENSG00000165309	ENSG00000165309	ARMC3	10:23216952-23326518	q1	q2	OK	0.718367	0.444297	-0.693196	-0.292521	0.4081	0.99995	no

ENSG00000165312	ENSG00000165312	OTUD1	10:23728197-23731308	q1	q2	OK	1.55263	1.30543	-0.250192	-0.319171	0.658	0.99995	no

ENSG00000165322	ENSG00000165322	ARHGAP12	10:32094364-32217742	q1	q2	OK	25.0397	27.5521	0.137944	0.261896	0.68825	0.99995	no

ENSG00000165323	ENSG00000165323	FAT3	11:92085261-92629618	q1	q2	OK	24.659	20.7624	-0.248145	-0.287701	0.52755	0.99995	no

ENSG00000165325	ENSG00000165325	CCDC67	11:93063136-93171653	q1	q2	NOTEST	0.0428967	0.0727953	0.762979	0	1	1	no

ENSG00000165338	ENSG00000165338	HECTD2	10:93170095-93274586	q1	q2	OK	13.9829	14.5867	0.0609871	0.1395	0.8369	0.99995	no

ENSG00000165349	ENSG00000165349	SLC7A3	X:70145431-70150975	q1	q2	OK	8.36899	8.20841	-0.0279506	-0.0501956	0.9446	0.99995	no

ENSG00000165355	ENSG00000165355	FBXO33	14:39866872-39901704	q1	q2	OK	4.92709	4.51249	-0.126814	-0.179344	0.7533	0.99995	no

ENSG00000165359	ENSG00000165359	DDX26B	X:134654007-134716435	q1	q2	NOTEST	0.240006	0.345819	0.526949	0	1	1	no

ENSG00000165370	ENSG00000165370	GPR101	X:136112306-136113833	q1	q2	NOTEST	0	0.0359537	inf	0	1	1	no

ENSG00000165376	ENSG00000165376	CLDN2	X:106045909-106243474	q1	q2	NOTEST	0.0363204	0.0525801	0.533737	0	1	1	no

ENSG00000165379	ENSG00000165379	LRFN5	14:42076772-42373752	q1	q2	OK	2.3615	2.79335	0.242294	0.304228	0.6026	0.99995	no

ENSG00000165383	ENSG00000165383	LRRC18	10:49892920-50323554	q1	q2	NOTEST	0.250166	0.120077	-1.05892	0	1	1	no

ENSG00000165388	ENSG00000165388	ZNF488	10:48355023-48373866	q1	q2	NOTEST	0	0	0	0	1	1	no

ENSG00000165389	ENSG00000165389	SPTSSA	14:34393436-34931980	q1	q2	OK	18.954	18.9858	0.00241874	0.00539634	0.9945	0.99995	no

ENSG00000165390	ENSG00000165390	ANXA8	10:48247668-48279200	q1	q2	NOTEST	0	0	0	0	1	1	no

ENSG00000165392	ENSG00000165392	WRN	8:30891316-31034683	q1	q2	OK	11.1791	12.8386	0.199685	0.287812	0.6096	0.99995	no

ENSG00000165406	ENSG00000165406	Mar-08	10:45950034-46090354	q1	q2	OK	7.46522	8.58259	0.201229	0.213393	0.76305	0.99995	no

ENSG00000165409	ENSG00000165409	TSHR	14:80943329-81636758	q1	q2	NOTEST	0.0326493	0.0508323	0.638691	0	1	1	no

ENSG00000165410	ENSG00000165410	CFL2	14:35179592-35184029	q1	q2	OK	43.5225	44.3673	0.0277378	0.0506617	0.9402	0.99995	no

ENSG00000165416	ENSG00000165416	SUGT1	13:53226843-53275044	q1	q2	OK	50.5226	48.6945	-0.0531704	-0.0895357	0.8964	0.99995	no

ENSG00000165417	ENSG00000165417	GTF2A1	14:81641795-81687721	q1	q2	OK	17.9027	19.6244	0.132473	0.308188	0.63595	0.99995	no

ENSG00000165424	ENSG00000165424	ZCCHC24	10:81142080-81205383	q1	q2	OK	7.55606	7.98428	0.0795283	0.153953	0.81685	0.99995	no

ENSG00000165434	ENSG00000165434	PGM2L1	11:74041362-74197096	q1	q2	OK	6.09562	6.48435	0.0891888	0.203395	0.77065	0.99995	no

ENSG00000165443	ENSG00000165443	PHYHIPL	10:60936349-61122939	q1	q2	OK	18.7495	20.1578	0.104483	0.171863	0.75405	0.99995	no

ENSG00000165449	ENSG00000165449	SLC16A9	10:61410522-61495760	q1	q2	OK	10.4843	10.5884	0.0142652	0.0296792	0.96485	0.99995	no

ENSG00000165457	ENSG00000165457	FOLR2	11:71927644-71932994	q1	q2	NOTEST	0	0	0	0	1	1	no

ENSG00000165458	ENSG00000165458	INPPL1	11:71934744-71956708	q1	q2	OK	79.5499	68.0875	-0.224472	-0.291691	0.51175	0.99995	no

ENSG00000165462	ENSG00000165462	PHOX2A	11:71934744-71956708	q1	q2	NOTEST	0.0685956	0.0337788	-1.022	0	1	1	no

ENSG00000165471	ENSG00000165471	MBL2	10:54525139-54531460	q1	q2	NOTEST	0	0	0	0	1	1	no

ENSG00000165474	ENSG00000165474	GJB2	13:20761608-20767037	q1	q2	NOTEST	0	0.0112218	inf	0	1	1	no

ENSG00000165475	ENSG00000165475	CRYL1	13:20977805-21099996	q1	q2	OK	2.43921	2.37114	-0.0408291	-0.0481109	0.9412	0.99995	no

ENSG00000165476	ENSG00000165476	REEP3	10:65281122-65384883	q1	q2	OK	14.4706	17.9928	0.314298	0.741114	0.2964	0.99995	no

ENSG00000165478	ENSG00000165478	HEPACAM	11:124789088-124806308	q1	q2	NOTEST	0.0295726	0	#NAME?	0	1	1	no

ENSG00000165480	ENSG00000165480	SKA3	13:21727733-21750741	q1	q2	OK	18.2272	19.7937	0.118945	0.236693	0.7003	0.99995	no

ENSG00000165487	ENSG00000165487	MICU2	13:22066835-22178353	q1	q2	OK	37.8581	39.7719	0.0711472	0.158188	0.8136	0.99995	no

ENSG00000165490	ENSG00000165490	C11orf82	11:82534543-82782965	q1	q2	OK	11.6887	13.253	0.181202	0.218149	0.7104	0.99995	no

ENSG00000165494	ENSG00000165494	PCF11	11:82868029-83134559	q1	q2	OK	21.0166	21.512	0.0336106	0.0329567	0.9504	0.99995	no

ENSG00000165495	ENSG00000165495	PKNOX2	11:125034582-125303285	q1	q2	OK	11.4448	11.3575	-0.0110509	-0.0192187	0.97185	0.99995	no

ENSG00000165496	ENSG00000165496	RPL10L	14:47120221-47121028	q1	q2	NOTEST	0	0	0	0	1	1	no

ENSG00000165501	ENSG00000165501	LRR1	14:50043389-50081390	q1	q2	OK	22.5607	24.5068	0.119371	0.0935469	0.87455	0.99995	no

ENSG00000165502	ENSG00000165502	RPL36AL	14:50085236-50087403	q1	q2	OK	157.562	166.876	0.0828523	0.200215	0.7753	0.99995	no

ENSG00000165506	ENSG00000165506	DNAAF2	14:50091891-50101948	q1	q2	OK	13.4423	13.3132	-0.0139231	-0.0298642	0.96445	0.99995	no

ENSG00000165507	ENSG00000165507	C10orf10	10:45306471-45491339	q1	q2	NOTEST	0.406945	0.470147	0.208279	0	1	1	no

ENSG00000165509	ENSG00000165509	MAGEC3	X:140926101-140985618	q1	q2	NOTEST	0.0991566	0.0327265	-1.59925	0	1	1	no

ENSG00000165511	ENSG00000165511	C10orf25	10:45493145-45676875	q1	q2	NOTEST	0.356921	0.313363	-0.187771	0	1	1	no

ENSG00000165512	ENSG00000165512	ZNF22	10:45493145-45676875	q1	q2	OK	5.24775	5.99527	0.192124	0.30991	0.65795	0.99995	no

ENSG00000165516	ENSG00000165516	KLHDC2	14:50234325-50249909	q1	q2	OK	34.2552	32.9862	-0.0544615	-0.104568	0.8612	0.99995	no

ENSG00000165521	ENSG00000165521	EML5	14:89029252-89259096	q1	q2	OK	4.20744	4.80802	0.1925	0.147777	0.80435	0.99995	no

ENSG00000165525	ENSG00000165525	NEMF	14:50249996-50319921	q1	q2	OK	31.5453	34.4098	0.125395	0.20677	0.7059	0.99995	no

ENSG00000165526	ENSG00000165526	RPUSD4	11:126071992-126148026	q1	q2	OK	16.1461	16.9092	0.0666171	0.0589756	0.92315	0.99995	no

ENSG00000165527	ENSG00000165527	ARF6	14:50359809-50361490	q1	q2	OK	24.9045	25.4702	0.0324013	0.0693509	0.9224	0.99995	no

ENSG00000165533	ENSG00000165533	TTC8	14:89290496-89344335	q1	q2	OK	13.7951	14.0222	0.0235501	0.0428729	0.9489	0.99995	no

ENSG00000165548	ENSG00000165548	TMEM63C	14:77564439-77725838	q1	q2	OK	0.461962	0.752296	0.703524	0.116952	0.56185	0.99995	no

ENSG00000165553	ENSG00000165553	NGB	14:77731825-77737655	q1	q2	NOTEST	0	0.0283203	inf	0	1	1	no

ENSG00000165555	ENSG00000165555	NOXRED1	14:77860363-77889860	q1	q2	OK	1.05448	0.778967	-0.436902	-0.447022	0.49215	0.99995	no

ENSG00000165556	ENSG00000165556	CDX2	13:28536273-28545276	q1	q2	NOTEST	0	0	0	0	1	1	no

ENSG00000165566	ENSG00000165566	AMER2	13:25735821-25746426	q1	q2	OK	4.72521	4.68207	-0.0132331	-0.0270569	0.9681	0.99995	no

ENSG00000165568	ENSG00000165568	AKR1E2	10:4828820-4890254	q1	q2	NOTEST	0	0	0	0	1	1	no

ENSG00000165572	ENSG00000165572	KBTBD6	13:41701704-41706882	q1	q2	OK	8.92696	9.8202	0.137584	0.30763	0.6583	0.99995	no

ENSG00000165583	ENSG00000165583	SSX5	X:48045655-48056199	q1	q2	NOTEST	0	0	0	0	1	1	no

ENSG00000165584	ENSG00000165584	SSX3	X:48205862-48216142	q1	q2	NOTEST	0	0	0	0	1	1	no

ENSG00000165588	ENSG00000165588	OTX2	14:57267424-57277197	q1	q2	NOTEST	0.034511	0.0597857	0.792744	0	1	1	no

ENSG00000165591	ENSG00000165591	FAAH2	X:57313138-57515629	q1	q2	OK	0.883339	0.262361	-1.75141	-0.863396	0.16285	0.99995	no

ENSG00000165606	ENSG00000165606	DRGX	10:50572236-50603497	q1	q2	NOTEST	0.0192903	0.0188633	-0.0322871	0	1	1	no

ENSG00000165609	ENSG00000165609	NUDT5	10:12171635-12292588	q1	q2	OK	38.9306	41.1806	0.0810602	0.0920897	0.8767	0.99995	no

ENSG00000165617	ENSG00000165617	DACT1	14:59100684-59115039	q1	q2	OK	4.43965	4.89427	0.140647	0.205615	0.747	0.99995	no

ENSG00000165621	ENSG00000165621	OXGR1	13:97637972-97646984	q1	q2	NOTEST	0.0450723	0.1246	1.46699	0	1	1	no

ENSG00000165623	ENSG00000165623	UCMA	10:13263766-13276334	q1	q2	NOTEST	0	0	0	0	1	1	no

ENSG00000165626	ENSG00000165626	BEND7	10:13480483-13572262	q1	q2	OK	12.8858	15.298	0.247563	0.247587	0.6146	0.99995	no

ENSG00000165629	ENSG00000165629	ATP5C1	10:7830091-7849778	q1	q2	OK	125.262	131.277	0.0676648	0.163287	0.81645	0.99995	no

ENSG00000165630	ENSG00000165630	PRPF18	10:13628926-14504141	q1	q2	OK	11.656	12.6404	0.116967	0.0773006	0.90555	0.99995	no

ENSG00000165632	ENSG00000165632	TAF3	10:7860466-8058590	q1	q2	OK	5.46235	5.88874	0.108436	0.217736	0.75655	0.99995	no

ENSG00000165633	ENSG00000165633	VSTM4	10:49892920-50323554	q1	q2	OK	1.25793	0.951995	-0.40203	-0.491263	0.41475	0.99995	no

ENSG00000165637	ENSG00000165637	VDAC2	10:76969911-76991206	q1	q2	OK	95.7736	102.032	0.0913156	0.217158	0.7509	0.99995	no

ENSG00000165643	ENSG00000165643	SOHLH1	9:138585252-138591374	q1	q2	NOTEST	0	0	0	0	1	1	no

ENSG00000165644	ENSG00000165644	COMTD1	10:76993726-76995788	q1	q2	OK	1.28126	1.45867	0.18709	0.173875	0.79615	0.99995	no

ENSG00000165646	ENSG00000165646	SLC18A2	10:118999110-119038941	q1	q2	OK	2.04441	2.317	0.180571	0.271773	0.69165	0.99995	no

ENSG00000165650	ENSG00000165650	PDZD8	10:119039999-119134978	q1	q2	OK	8.64924	10.9315	0.337841	0.620939	0.322	0.99995	no

ENSG00000165655	ENSG00000165655	ZNF503	10:77029576-77171600	q1	q2	OK	6.04408	4.91702	-0.297738	-0.508779	0.48245	0.99995	no

ENSG00000165659	ENSG00000165659	DACH1	13:72012097-72441330	q1	q2	OK	14.3038	12.78	-0.162504	-0.368615	0.5981	0.99995	no

ENSG00000165660	ENSG00000165660	FAM175B	10:126490353-126525239	q1	q2	OK	10.5284	10.6506	0.0166476	0.0343695	0.9609	0.99995	no

ENSG00000165661	ENSG00000165661	QSOX2	9:139098178-139137687	q1	q2	OK	15.9023	16.9475	0.0918407	0.21135	0.75905	0.99995	no

ENSG00000165669	ENSG00000165669	FAM204A	10:120065400-120101840	q1	q2	OK	39.7798	45.2923	0.187231	0.2944	0.6758	0.99995	no

ENSG00000165671	ENSG00000165671	NSD1	5:176560025-176727216	q1	q2	OK	18.506	19.0684	0.043192	0.057646	0.8773	0.99995	no

ENSG00000165672	ENSG00000165672	PRDX3	10:120927214-120938345	q1	q2	OK	103.095	112.048	0.120141	0.27941	0.67275	0.99995	no

ENSG00000165675	ENSG00000165675	ENOX2	X:129757349-130037208	q1	q2	OK	7.30102	8.38063	0.198962	0.405562	0.5592	0.99995	no

ENSG00000165678	ENSG00000165678	GHITM	10:85898175-85913001	q1	q2	OK	76.3922	79.035	0.0490674	0.122141	0.8596	0.99995	no

ENSG00000165682	ENSG00000165682	CLEC1B	12:10100735-10183205	q1	q2	NOTEST	0	0	0	0	1	1	no

ENSG00000165684	ENSG00000165684	SNAPC4	9:139270028-139293249	q1	q2	OK	5.57356	5.15898	-0.111513	-0.221181	0.76035	0.99995	no

ENSG00000165685	ENSG00000165685	TMEM52B	12:10310901-10344400	q1	q2	NOTEST	0	0	0	0	1	1	no

ENSG00000165688	ENSG00000165688	PMPCA	9:139305109-139318213	q1	q2	OK	33.2899	29.4688	-0.175893	-0.386614	0.56515	0.99995	no

ENSG00000165689	ENSG00000165689	SDCCAG3	9:139296376-139305061	q1	q2	OK	35.2707	37.1099	0.0733331	0.122642	0.8476	0.99995	no

ENSG00000165694	ENSG00000165694	FRMD7	X:131211020-131262048	q1	q2	NOTEST	0.0933359	0.0121158	-2.94554	0	1	1	no

ENSG00000165695	ENSG00000165695	AK8	9:135600964-135765588	q1	q2	NOTEST	0.414343	0.380388	-0.123354	0	1	1	no

ENSG00000165698	ENSG00000165698	C9orf9	9:135600964-135765588	q1	q2	OK	3.16488	2.31458	-0.451401	-0.539559	0.45375	0.99995	no

ENSG00000165699	ENSG00000165699	TSC1	9:135766734-135820020	q1	q2	OK	13.6611	14.4539	0.0813801	0.121818	0.78095	0.99995	no

ENSG00000165702	ENSG00000165702	GFI1B	9:135820931-135867083	q1	q2	NOTEST	0.0163305	0	#NAME?	0	1	1	no

ENSG00000165704	ENSG00000165704	HPRT1	X:133594182-133654543	q1	q2	OK	9.86241	10.3961	0.0760357	0.127084	0.855	0.99995	no

ENSG00000165714	ENSG00000165714	LOH12CR1	12:12510012-12619840	q1	q2	OK	3.58843	4.90821	0.451842	0.408731	0.55645	0.99995	no

ENSG00000165716	ENSG00000165716	FAM69B	9:139607021-139622636	q1	q2	OK	25.4616	21.2075	-0.263748	-0.490181	0.4847	0.99995	no

ENSG00000165724	ENSG00000165724	ZMYND19	9:140476530-140484942	q1	q2	OK	29.1565	26.6079	-0.131965	-0.273608	0.7014	0.99995	no

ENSG00000165730	ENSG00000165730	STOX1	10:70587297-70655188	q1	q2	OK	4.51201	4.7078	0.0612821	0.100995	0.8816	0.99995	no

ENSG00000165731	ENSG00000165731	RET	10:43572474-43625799	q1	q2	NOTEST	0.223484	0.181296	-0.301826	0	1	1	no

ENSG00000165732	ENSG00000165732	DDX21	10:70715883-70744829	q1	q2	OK	30.7344	37.1836	0.274813	0.682292	0.33145	0.99995	no

ENSG00000165733	ENSG00000165733	BMS1	10:43278248-43330385	q1	q2	OK	15.0713	16.6451	0.143292	0.352019	0.60215	0.99995	no

ENSG00000165752	ENSG00000165752	STK32C	10:134020995-134145351	q1	q2	OK	10.0016	8.78818	-0.186598	-0.244175	0.7369	0.99995	no

ENSG00000165757	ENSG00000165757	KIAA1462	10:30301728-30404423	q1	q2	OK	3.06204	4.18728	0.451524	0.867945	0.19535	0.99995	no

ENSG00000165762	ENSG00000165762	OR4K2	14:20344390-20345468	q1	q2	NOTEST	0	0	0	0	1	1	no

ENSG00000165775	ENSG00000165775	FUNDC2	X:154064062-154288578	q1	q2	NOTEST	0.376	0	#NAME?	0	1	1	no

ENSG00000165782	ENSG00000165782	TMEM55B	14:20923349-20929771	q1	q2	OK	20.5128	21.2441	0.0505361	0.0390123	0.9581	0.99995	no

ENSG00000165792	ENSG00000165792	METTL17	14:21457928-21467322	q1	q2	OK	52.7405	50.6723	-0.0577145	-0.117351	0.8551	0.99995	no

ENSG00000165794	ENSG00000165794	SLC39A2	14:21467413-21470030	q1	q2	NOTEST	0.0908744	0.202112	1.15321	0	1	1	no

ENSG00000165795	ENSG00000165795	NDRG2	14:21484921-21572881	q1	q2	OK	14.0059	12.9799	-0.10976	-0.0500942	0.84835	0.99995	no

ENSG00000165799	ENSG00000165799	RNASE7	14:21484921-21572881	q1	q2	NOTEST	0	0	0	0	1	1	no

ENSG00000165801	ENSG00000165801	ARHGEF40	14:21484921-21572881	q1	q2	OK	49.3449	45.2986	-0.123434	-0.146426	0.80705	0.99995	no

ENSG00000165802	ENSG00000165802	NSMF	9:140342021-140353786	q1	q2	OK	28.0326	26.9405	-0.0573285	-0.11533	0.8673	0.99995	no

ENSG00000165804	ENSG00000165804	ZNF219	14:21484921-21572881	q1	q2	OK	64.0213	59.5386	-0.104726	-0.104395	0.83805	0.99995	no

ENSG00000165805	ENSG00000165805	C12orf50	12:88373815-88535993	q1	q2	NOTEST	0.147829	0.0468171	-1.65883	0	1	1	no

ENSG00000165806	ENSG00000165806	CASP7	10:115438941-115490662	q1	q2	OK	9.30066	8.72394	-0.0923529	-0.150625	0.8268	0.99995	no

ENSG00000165807	ENSG00000165807	PPP1R36	14:65016619-65063254	q1	q2	NOTEST	0.361814	0.479255	0.405545	0	1	1	no

ENSG00000165810	ENSG00000165810	BTNL9	5:180467224-180488523	q1	q2	OK	1.00429	1.03985	0.0501966	0.0374111	0.94225	0.99995	no

ENSG00000165813	ENSG00000165813	C10orf118	10:115880620-115933979	q1	q2	OK	4.89941	5.95801	0.282221	0.285252	0.48765	0.99995	no

ENSG00000165816	ENSG00000165816	VWA2	10:115999017-116051272	q1	q2	NOTEST	0.00914475	0.00886339	-0.0450855	0	1	1	no

ENSG00000165819	ENSG00000165819	METTL3	14:21927178-21979517	q1	q2	OK	52.4322	55.5232	0.0826374	0.12757	0.85515	0.99995	no

ENSG00000165821	ENSG00000165821	SALL2	14:21989231-22005350	q1	q2	OK	124.868	123.697	-0.0135936	-0.0242571	0.96495	0.99995	no

ENSG00000165828	ENSG00000165828	PRAP1	10:135093134-135166187	q1	q2	NOTEST	0.0456177	0.302941	2.73137	0	1	1	no

ENSG00000165832	ENSG00000165832	TRUB1	10:116697951-116737430	q1	q2	OK	12.6629	13.8385	0.128082	0.247408	0.6899	0.99995	no

ENSG00000165837	ENSG00000165837	FAM194B	13:46039059-46189874	q1	q2	OK	1.57165	1.54341	-0.0261576	-0.00717992	0.98865	0.99995	no

ENSG00000165841	ENSG00000165841	CYP2C19	10:96443250-96613017	q1	q2	NOTEST	0.0139501	0.0415773	1.57552	0	1	1	no

ENSG00000165861	ENSG00000165861	ZFYVE1	14:73436158-73493920	q1	q2	OK	9.77174	7.61602	-0.359577	-0.577766	0.30535	0.99995	no

ENSG00000165862	ENSG00000165862	PNLIPRP2	10:118380464-118404654	q1	q2	NOTEST	0	0	0	0	1	1	no

ENSG00000165863	ENSG00000165863	C10orf82	10:118423206-118502085	q1	q2	NOTEST	0	0	0	0	1	1	no

ENSG00000165868	ENSG00000165868	HSPA12A	10:118423206-118502085	q1	q2	NOTEST	0	0	0	0	1	1	no

ENSG00000165874	ENSG00000165874	FAM35BP	10:46897591-46971400	q1	q2	NOTEST	0.246642	0.318128	0.367186	0	1	1	no

ENSG00000165879	ENSG00000165879	FRAT1	10:99079021-99081672	q1	q2	OK	2.17492	1.63904	-0.408114	-0.518679	0.4434	0.99995	no

ENSG00000165886	ENSG00000165886	UBTD1	10:99258624-99330966	q1	q2	OK	6.87379	5.43836	-0.337934	-0.518589	0.4554	0.99995	no

ENSG00000165887	ENSG00000165887	ANKRD2	10:99332197-99343641	q1	q2	NOTEST	0	0	0	0	1	1	no

ENSG00000165891	ENSG00000165891	E2F7	12:77415026-77459360	q1	q2	OK	9.8044	10.275	0.0676374	0.132333	0.8252	0.99995	no

ENSG00000165895	ENSG00000165895	ARHGAP42	11:100554892-100862668	q1	q2	OK	5.07145	6.23875	0.298858	0.540403	0.36895	0.99995	no

ENSG00000165898	ENSG00000165898	ISCA2	14:74942894-74963809	q1	q2	OK	19.935	19.3864	-0.0402574	-0.0415044	0.95275	0.99995	no

ENSG00000165899	ENSG00000165899	OTOGL	12:80603232-80772870	q1	q2	NOTEST	0.212109	0.242715	0.194455	0	1	1	no

ENSG00000165905	ENSG00000165905	GYLTL1B	11:45943171-45950647	q1	q2	NOTEST	0.179812	0.0646743	-1.47523	0	1	1	no

ENSG00000165912	ENSG00000165912	PACSIN3	11:47199075-47207994	q1	q2	OK	4.22978	3.10043	-0.448113	-0.548523	0.36215	0.99995	no

ENSG00000165914	ENSG00000165914	TTC7B	14:91006931-91282823	q1	q2	OK	10.3559	9.89214	-0.0660998	-0.0896868	0.8434	0.99995	no

ENSG00000165915	ENSG00000165915	SLC39A13	11:47404698-47438047	q1	q2	OK	23.0081	20.1257	-0.1931	-0.291799	0.62635	0.99995	no

ENSG00000165916	ENSG00000165916	PSMC3	11:47440319-47447993	q1	q2	OK	144.63	157.31	0.12125	0.245379	0.72265	0.99995	no

ENSG00000165917	ENSG00000165917	RAPSN	11:47459307-47470730	q1	q2	NOTEST	0.0600053	0.0191078	-1.65093	0	1	1	no

ENSG00000165923	ENSG00000165923	AGBL2	11:47681142-47736941	q1	q2	NOTEST	0.0326444	0.0215961	-0.59606	0	1	1	no

ENSG00000165929	ENSG00000165929	TC2N	14:92047039-92333880	q1	q2	NOTEST	0.0454733	0.0356584	-0.350777	0	1	1	no

ENSG00000165934	ENSG00000165934	CPSF2	14:92588280-92630755	q1	q2	OK	29.1429	29.77	0.0307145	0.0499152	0.9315	0.99995	no

ENSG00000165935	ENSG00000165935	SMCO2	12:27619742-27655118	q1	q2	NOTEST	0.0794573	0.164503	1.04986	0	1	1	no

ENSG00000165943	ENSG00000165943	MOAP1	14:93648540-93651273	q1	q2	OK	17.0379	17.4954	0.0382283	0.0826495	0.9042	0.99995	no

ENSG00000165948	ENSG00000165948	IFI27L1	14:94547627-94570192	q1	q2	OK	17.7231	15.8034	-0.165402	-0.240406	0.72755	0.99995	no

ENSG00000165949	ENSG00000165949	IFI27	14:94571181-94583033	q1	q2	OK	2.94303	2.60432	-0.176392	-0.150807	0.76085	0.99995	no

ENSG00000165953	ENSG00000165953	SERPINA12	14:94953610-94984181	q1	q2	NOTEST	0	0	0	0	1	1	no

ENSG00000165959	ENSG00000165959	CLMN	14:95648276-95786243	q1	q2	OK	2.04126	1.53215	-0.413896	-0.456981	0.382	0.99995	no

ENSG00000165966	ENSG00000165966	PDZRN4	12:41582249-41968392	q1	q2	OK	2.40268	1.8048	-0.412806	-0.580782	0.4113	0.99995	no

ENSG00000165970	ENSG00000165970	SLC6A5	11:20620945-20680831	q1	q2	NOTEST	0.222741	0.245865	0.142498	0	1	1	no

ENSG00000165972	ENSG00000165972	CCDC38	12:96252705-96336752	q1	q2	NOTEST	0.00989137	0.0102844	0.0562097	0	1	1	no

ENSG00000165973	ENSG00000165973	NELL1	11:20691116-21597227	q1	q2	OK	10.5574	9.90647	-0.0918063	-0.140248	0.79675	0.99995	no

ENSG00000165983	ENSG00000165983	PTER	10:16478941-16555736	q1	q2	NOTEST	0.195755	0.169991	-0.20359	0	1	1	no

ENSG00000165985	ENSG00000165985	C1QL3	10:16555741-16564004	q1	q2	NOTEST	0.0206973	0.0207904	0.00647833	0	1	1	no

ENSG00000165995	ENSG00000165995	CACNB2	10:18429605-18948214	q1	q2	OK	2.45731	2.44449	-0.00754093	-0.00598759	0.9912	0.99995	no

ENSG00000165996	ENSG00000165996	PTPLA	10:17631957-17659376	q1	q2	OK	0.339301	1.97203	2.53904	1.627	0.0242	0.99995	no

ENSG00000165997	ENSG00000165997	ARL5B	10:18948333-18970568	q1	q2	OK	20.7523	24.0894	0.215123	0.531652	0.44965	0.99995	no

ENSG00000166002	ENSG00000166002	SMCO4	11:93211637-93276674	q1	q2	OK	6.7123	6.12404	-0.132325	-0.136502	0.83745	0.99995	no

ENSG00000166004	ENSG00000166004	KIAA1731	11:93394804-93547861	q1	q2	OK	14.7546	12.4481	-0.245236	-0.0993349	0.70065	0.99995	no

ENSG00000166006	ENSG00000166006	KCNC2	12:75414748-75603648	q1	q2	NOTEST	0.0798823	0.0490007	-0.705072	0	1	1	no

ENSG00000166007	ENSG00000166007	TRIM51HP	11:55059358-55065746	q1	q2	NOTEST	0.0308421	0	#NAME?	0	1	1	no

ENSG00000166008	ENSG00000166008	MAGEA9	X:148863583-148869397	q1	q2	NOTEST	0	0	0	0	1	1	no

ENSG00000166012	ENSG00000166012	TAF1D	11:93394804-93547861	q1	q2	OK	50.4425	68.9586	0.451089	0.505236	0.31985	0.99995	no

ENSG00000166013	ENSG00000166013	TRIM53BP	11:89575164-89584138	q1	q2	NOTEST	0	0	0	0	1	1	no

ENSG00000166016	ENSG00000166016	ABTB2	11:34172534-34379555	q1	q2	OK	24.96	27.4998	0.139806	0.329713	0.63415	0.99995	no

ENSG00000166024	ENSG00000166024	R3HCC1L	10:99894386-100004654	q1	q2	OK	9.91611	10.5219	0.0855438	0.178679	0.7999	0.99995	no

ENSG00000166025	ENSG00000166025	AMOTL1	11:94277005-94658686	q1	q2	OK	24.908	26.6924	0.0998219	0.203129	0.73775	0.99995	no

ENSG00000166033	ENSG00000166033	HTRA1	10:124221040-124274424	q1	q2	OK	11.2987	7.57579	-0.576685	-0.904921	0.19895	0.99995	no

ENSG00000166035	ENSG00000166035	LIPC	15:58245621-58861151	q1	q2	NOTEST	0.0385907	0	#NAME?	0	1	1	no

ENSG00000166037	ENSG00000166037	CEP57	11:95502105-95565857	q1	q2	OK	44.1674	46.6361	0.0784659	0.141577	0.8223	0.99995	no

ENSG00000166046	ENSG00000166046	TCP11L2	12:106631654-106740793	q1	q2	OK	1.62915	1.22024	-0.416949	-0.10036	0.74535	0.99995	no

ENSG00000166049	ENSG00000166049	PASD1	X:150732093-150845211	q1	q2	NOTEST	0	0.00775604	inf	0	1	1	no

ENSG00000166068	ENSG00000166068	SPRED1	15:38544526-38649450	q1	q2	OK	45.2226	48.738	0.108004	0.209142	0.71835	0.99995	no

ENSG00000166069	ENSG00000166069	TMCO5A	15:38214139-38259925	q1	q2	NOTEST	0.0143912	0	#NAME?	0	1	1	no

ENSG00000166073	ENSG00000166073	GPR176	15:39892231-40213093	q1	q2	OK	1.05493	0.812007	-0.377579	-0.265781	0.70765	0.99995	no

ENSG00000166086	ENSG00000166086	JAM3	11:133938819-134117686	q1	q2	OK	88.3543	84.1433	-0.0704509	-0.116378	0.85015	0.99995	no

ENSG00000166090	ENSG00000166090	IL25	14:23842017-23845612	q1	q2	NOTEST	0	0	0	0	1	1	no

ENSG00000166091	ENSG00000166091	CMTM5	14:23846016-23848981	q1	q2	NOTEST	0	0	0	0	1	1	no

ENSG00000166104	ENSG00000166104	hsa-mir-7162	15:62520094-62545434	q1	q2	NOTEST	0	0	0	0	1	1	no

ENSG00000166105	ENSG00000166105	GLB1L3	11:134144138-134189458	q1	q2	NOTEST	0	0.0136504	inf	0	1	1	no

ENSG00000166106	ENSG00000166106	ADAMTS15	11:130318868-130346532	q1	q2	OK	7.10992	5.21378	-0.447503	-0.905616	0.20325	0.99995	no

ENSG00000166111	ENSG00000166111	SVOP	12:109458638-109459045	q1	q2	OK	0.692972	1.56468	1.175	0.625633	0.11365	0.99995	no

ENSG00000166118	ENSG00000166118	SPATA19	11:133710525-133715433	q1	q2	NOTEST	0	0	0	0	1	1	no

ENSG00000166123	ENSG00000166123	GPT2	16:46918289-46965209	q1	q2	OK	30.0046	28.2801	-0.0853948	-0.207493	0.77015	0.99995	no

ENSG00000166126	ENSG00000166126	AMN	14:103388992-103523799	q1	q2	NOTEST	0.227755	0.155445	-0.551081	0	1	1	no

ENSG00000166128	ENSG00000166128	RAB8B	15:63481667-63559981	q1	q2	OK	32.2299	29.1966	-0.142601	-0.270861	0.63865	0.99995	no

ENSG00000166130	ENSG00000166130	IKBIP	12:99007182-99038891	q1	q2	OK	22.019	21.3297	-0.0458827	-0.0906654	0.8949	0.99995	no

ENSG00000166133	ENSG00000166133	RPUSD2	15:40861498-40866659	q1	q2	OK	6.3215	5.7759	-0.13022	-0.210828	0.75855	0.99995	no

ENSG00000166135	ENSG00000166135	HIF1AN	10:102246398-102319755	q1	q2	OK	16.0486	21.211	0.40237	0.281161	0.5223	0.99995	no

ENSG00000166136	ENSG00000166136	NDUFB8	10:102246398-102319755	q1	q2	OK	131.19	126.714	-0.0500836	-0.0650443	0.9283	0.99995	no

ENSG00000166140	ENSG00000166140	ZFYVE19	15:41060066-41106767	q1	q2	OK	9.70623	9.65974	-0.00692642	-0.00825873	0.98815	0.99995	no

ENSG00000166143	ENSG00000166143	PPP1R14D	15:41107649-41120907	q1	q2	NOTEST	0	0	0	0	1	1	no

ENSG00000166145	ENSG00000166145	SPINT1	15:41128005-41150405	q1	q2	NOTEST	0.391399	0.0823372	-2.24902	0	1	1	no

ENSG00000166147	ENSG00000166147	FBN1	15:48700502-48938046	q1	q2	OK	27.2778	25.4255	-0.101456	-0.169812	0.776	0.99995	no

ENSG00000166148	ENSG00000166148	AVPR1A	12:63539013-63544722	q1	q2	NOTEST	0.319607	0.216048	-0.564945	0	1	1	no

ENSG00000166152	ENSG00000166152	C16orf78	16:49407733-49433342	q1	q2	NOTEST	0	0.0283522	inf	0	1	1	no

ENSG00000166153	ENSG00000166153	DEPDC4	12:100550134-100660857	q1	q2	OK	2.60194	2.8163	0.114212	0.0512176	0.90245	0.99995	no

ENSG00000166157	ENSG00000166157	TPTE	21:10906200-11098980	q1	q2	NOTEST	0	0	0	0	1	1	no

ENSG00000166159	ENSG00000166159	LRTM2	12:1901122-2032033	q1	q2	NOTEST	0.0188421	0.0887856	2.23637	0	1	1	no

ENSG00000166160	ENSG00000166160	OPN1MW2	X:153485224-153498755	q1	q2	NOTEST	0	0	0	0	1	1	no

ENSG00000166164	ENSG00000166164	BRD7	16:50280047-50405391	q1	q2	OK	56.0082	61.4148	0.132949	0.276409	0.6628	0.99995	no

ENSG00000166165	ENSG00000166165	CKB	14:103985995-103989448	q1	q2	OK	591.884	534.92	-0.145991	-0.280692	0.659	0.99995	no

ENSG00000166166	ENSG00000166166	TRMT61A	14:103995520-104003410	q1	q2	OK	4.88159	4.60575	-0.0839166	-0.146396	0.83345	0.99995	no

ENSG00000166167	ENSG00000166167	BTRC	10:103113819-103317078	q1	q2	OK	13.4451	13.8983	0.0478215	0.0935449	0.86885	0.99995	no

ENSG00000166169	ENSG00000166169	POLL	10:103330316-103369425	q1	q2	OK	12.247	11.0613	-0.146913	-0.199046	0.79155	0.99995	no

ENSG00000166170	ENSG00000166170	BAG5	14:104019757-104181841	q1	q2	OK	17.1834	18.781	0.12826	0.123783	0.8537	0.99995	no

ENSG00000166171	ENSG00000166171	DPCD	10:103330316-103369425	q1	q2	OK	26.9322	25.0442	-0.104858	-0.146482	0.83385	0.99995	no

ENSG00000166173	ENSG00000166173	LARP6	15:71123862-71342414	q1	q2	OK	15.9422	13.5848	-0.230851	-0.192799	0.7731	0.99995	no

ENSG00000166181	ENSG00000166181	API5	11:43333512-43516483	q1	q2	OK	79.207	85.4872	0.110081	0.144685	0.7619	0.99995	no

ENSG00000166183	ENSG00000166183	ASPG	14:104552015-104579098	q1	q2	NOTEST	0.0127864	0	#NAME?	0	1	1	no

ENSG00000166188	ENSG00000166188	ZNF319	16:58028571-58055522	q1	q2	OK	4.92592	4.51926	-0.124308	-0.132549	0.8495	0.99995	no

ENSG00000166189	ENSG00000166189	HPS6	10:103825146-103827792	q1	q2	OK	7.28646	6.29239	-0.21161	-0.382974	0.58935	0.99995	no

ENSG00000166192	ENSG00000166192	SENP8	15:72110736-72447800	q1	q2	OK	3.62752	3.30875	-0.132697	-0.0783795	0.90145	0.99995	no

ENSG00000166197	ENSG00000166197	NOLC1	10:103911932-103923627	q1	q2	OK	48.0642	48.9308	0.0257777	0.0618548	0.92925	0.99995	no

ENSG00000166199	ENSG00000166199	ALKBH3	11:43902360-43942494	q1	q2	OK	12.8911	13.5077	0.0674068	0.0943011	0.89005	0.99995	no

ENSG00000166200	ENSG00000166200	COPS2	15:49398267-49913128	q1	q2	OK	55.4752	57.379	0.0486806	0.0922159	0.89445	0.99995	no

ENSG00000166206	ENSG00000166206	GABRB3	15:26788692-27194354	q1	q2	OK	9.29662	9.91884	0.0934657	0.170924	0.7672	0.99995	no

ENSG00000166211	ENSG00000166211	SPIC	12:101869198-101880775	q1	q2	NOTEST	0	0	0	0	1	1	no

ENSG00000166220	ENSG00000166220	TBATA	10:72530994-72545157	q1	q2	NOTEST	0	0	0	0	1	1	no

ENSG00000166224	ENSG00000166224	SGPL1	10:72575716-72640930	q1	q2	OK	24.3876	24.9732	0.0342324	0.0668607	0.90475	0.99995	no

ENSG00000166225	ENSG00000166225	FRS2	12:69864128-69973562	q1	q2	OK	12.2511	12.9843	0.0838508	0.123332	0.7926	0.99995	no

ENSG00000166226	ENSG00000166226	CCT2	12:69979113-69995350	q1	q2	OK	189.058	207.153	0.131866	0.271387	0.6277	0.99995	no

ENSG00000166228	ENSG00000166228	PCBD1	10:72642036-72648541	q1	q2	OK	14.9459	13.918	-0.102801	-0.164224	0.80695	0.99995	no

ENSG00000166233	ENSG00000166233	ARIH1	15:72690613-72879692	q1	q2	OK	21.1966	22.566	0.0903206	0.114441	0.78395	0.99995	no

ENSG00000166246	ENSG00000166246	C16orf71	16:4746512-4817625	q1	q2	OK	0.530364	0.144574	-1.87518	-0.415	0.22155	0.99995	no

ENSG00000166250	ENSG00000166250	CLMP	11:122943034-123098985	q1	q2	OK	2.36699	2.97718	0.330893	0.396386	0.5186	0.99995	no

ENSG00000166257	ENSG00000166257	SCN3B	11:123499894-123525952	q1	q2	OK	3.21609	2.835	-0.181958	-0.308654	0.6619	0.99995	no

ENSG00000166260	ENSG00000166260	COX11	17:52976747-53241646	q1	q2	OK	36.7371	38.5066	0.0678701	0.0782385	0.86625	0.99995	no

ENSG00000166261	ENSG00000166261	ZNF202	11:123594884-123612383	q1	q2	OK	11.3191	12.0171	0.0863253	0.135421	0.79275	0.99995	no

ENSG00000166262	ENSG00000166262	FAM227B	15:49398267-49913128	q1	q2	OK	5.46588	5.25966	-0.0554865	-0.0220369	0.9446	0.99995	no

ENSG00000166263	ENSG00000166263	STXBP4	17:52976747-53241646	q1	q2	OK	8.39963	8.64356	0.0412992	0.0296067	0.9424	0.99995	no

ENSG00000166265	ENSG00000166265	CYYR1	21:27750870-27945603	q1	q2	OK	7.2186	7.64878	0.0835107	0.147659	0.82555	0.99995	no

ENSG00000166266	ENSG00000166266	CUL5	11:107879458-108018503	q1	q2	OK	14.2869	15.6354	0.130122	0.190676	0.7127	0.99995	no

ENSG00000166268	ENSG00000166268	MYRFL	12:70132460-70748773	q1	q2	NOTEST	0.194799	0.223474	0.198126	0	1	1	no

ENSG00000166272	ENSG00000166272	WBP1L	10:104503726-104576021	q1	q2	OK	14.4033	13.5799	-0.0849353	-0.193978	0.7828	0.99995	no

ENSG00000166275	ENSG00000166275	C10orf32	10:104613979-104661656	q1	q2	OK	15.3715	16.1349	0.069927	0.0906202	0.87725	0.99995	no

ENSG00000166278	ENSG00000166278	C2	6:31865561-31919861	q1	q2	NOTEST	0	0	0	0	1	1	no

ENSG00000166289	ENSG00000166289	PLEKHF1	19:30155962-30166364	q1	q2	OK	1.65893	1.59016	-0.0610801	-0.0623772	0.917	0.99995	no

ENSG00000166292	ENSG00000166292	TMEM100	17:53796987-53809482	q1	q2	OK	9.32731	10.2057	0.129847	0.215665	0.7411	0.99995	no

ENSG00000166295	ENSG00000166295	ANAPC16	10:73856277-73995618	q1	q2	OK	45.245	47.4811	0.0695952	0.103166	0.88095	0.99995	no

ENSG00000166311	ENSG00000166311	SMPD1	11:6411654-6416228	q1	q2	OK	10.5508	8.89466	-0.246345	-0.354729	0.5698	0.99995	no

ENSG00000166313	ENSG00000166313	APBB1	11:6416354-6440644	q1	q2	OK	38.4999	34.1201	-0.174235	-0.364054	0.5683	0.99995	no

ENSG00000166317	ENSG00000166317	SYNPO2L	10:75404638-75490272	q1	q2	NOTEST	0.039239	0.100718	1.35997	0	1	1	no

ENSG00000166321	ENSG00000166321	NUDT13	10:74870216-75118617	q1	q2	OK	2.16902	1.88173	-0.204984	-0.052719	0.85125	0.99995	no

ENSG00000166323	ENSG00000166323	C11orf65	11:108027941-108338258	q1	q2	OK	0.497287	0.491582	-0.0166462	-0.00180023	0.9845	0.99995	no

ENSG00000166326	ENSG00000166326	TRIM44	11:35684352-35829775	q1	q2	OK	29.1229	33.4959	0.201829	0.411096	0.5061	0.99995	no

ENSG00000166329	ENSG00000166329	CCDC182	17:55821841-55822673	q1	q2	NOTEST	0.0375524	0.116646	1.63516	0	1	1	no

ENSG00000166333	ENSG00000166333	ILK	11:6624871-6633898	q1	q2	OK	83.7497	86.0501	0.0390942	0.0768969	0.90485	0.99995	no

ENSG00000166337	ENSG00000166337	TAF10	11:6624871-6633898	q1	q2	OK	38.5754	37.257	-0.0501695	-0.0368115	0.957	0.99995	no

ENSG00000166340	ENSG00000166340	TPP1	11:6633999-6640995	q1	q2	OK	19.7163	18.6266	-0.0820221	-0.122975	0.77635	0.99995	no

ENSG00000166341	ENSG00000166341	DCHS1	11:6642555-6677085	q1	q2	OK	25.0881	24.0496	-0.0609945	-0.148718	0.83455	0.99995	no

ENSG00000166342	ENSG00000166342	NETO1	18:70409548-70535381	q1	q2	OK	7.73299	5.7246	-0.43385	-0.352705	0.5132	0.99995	no

ENSG00000166343	ENSG00000166343	MSS51	10:75183338-75193319	q1	q2	OK	2.40431	2.17559	-0.144218	-0.192952	0.7832	0.99995	no

ENSG00000166347	ENSG00000166347	CYB5A	18:71920529-71959251	q1	q2	OK	25.6892	28.1741	0.133205	0.231608	0.74405	0.99995	no

ENSG00000166348	ENSG00000166348	USP54	10:75196185-75385711	q1	q2	OK	16.7426	16.5506	-0.0166358	-0.0138517	0.9722	0.99995	no

ENSG00000166349	ENSG00000166349	RAG1	11:36532258-36694823	q1	q2	NOTEST	0.311176	0.337718	0.118089	0	1	1	no

ENSG00000166351	ENSG00000166351	POTED	21:14982497-15013906	q1	q2	NOTEST	0	0	0	0	1	1	no

ENSG00000166352	ENSG00000166352	C11orf74	11:36532258-36694823	q1	q2	OK	39.6304	39.1268	-0.0184502	-0.032143	0.96435	0.99995	no

ENSG00000166359	ENSG00000166359	WDR88	19:33622995-33666701	q1	q2	NOTEST	0.111994	0.222066	0.987571	0	1	1	no

ENSG00000166363	ENSG00000166363	OR10A5	11:6866882-6867936	q1	q2	NOTEST	0	0.0290855	inf	0	1	1	no

ENSG00000166368	ENSG00000166368	OR2D2	11:6912720-6913830	q1	q2	NOTEST	0	0	0	0	1	1	no

ENSG00000166377	ENSG00000166377	ATP9B	18:76829284-77138283	q1	q2	OK	8.88247	9.25599	0.0594265	0.0714707	0.88395	0.99995	no

ENSG00000166387	ENSG00000166387	PPFIBP2	11:7534528-7678358	q1	q2	OK	5.36873	5.86294	0.127044	0.129741	0.775	0.99995	no

ENSG00000166391	ENSG00000166391	MOGAT2	11:75428863-75444003	q1	q2	NOTEST	0.00789917	0	#NAME?	0	1	1	no

ENSG00000166394	ENSG00000166394	CYB5R2	11:7686330-7698453	q1	q2	OK	1.63269	1.74849	0.0988635	0.0762552	0.8787	0.99995	no

ENSG00000166396	ENSG00000166396	SERPINB7	18:61420168-61472604	q1	q2	NOTEST	0	0.0129873	inf	0	1	1	no

ENSG00000166398	ENSG00000166398	KIAA0355	19:34745441-34850317	q1	q2	OK	6.60831	6.09879	-0.115758	-0.196832	0.75135	0.99995	no

ENSG00000166401	ENSG00000166401	SERPINB8	18:61616534-61672278	q1	q2	OK	3.05765	2.85536	-0.0987472	-0.115081	0.85985	0.99995	no

ENSG00000166402	ENSG00000166402	TUB	11:8040790-8190602	q1	q2	OK	9.53438	8.39401	-0.183779	-0.383746	0.57365	0.99995	no

ENSG00000166405	ENSG00000166405	RIC3	11:8040790-8190602	q1	q2	OK	7.04642	6.16436	-0.192939	-0.157493	0.7741	0.99995	no

ENSG00000166407	ENSG00000166407	LMO1	11:8245850-8290263	q1	q2	OK	0.743065	1.14072	0.618389	0.581415	0.4203	0.99995	no

ENSG00000166408	ENSG00000166408	OR5P1P	11:7710668-7927502	q1	q2	NOTEST	0	0	0	0	1	1	no

ENSG00000166411	ENSG00000166411	IDH3A	15:78370149-78538030	q1	q2	OK	13.9098	15.3368	0.140893	0.155624	0.74535	0.99995	no

ENSG00000166415	ENSG00000166415	WDR72	15:53805937-54055075	q1	q2	NOTEST	0.0836076	0.0885712	0.0832033	0	1	1	no

ENSG00000166426	ENSG00000166426	CRABP1	15:78632665-78640572	q1	q2	OK	357.838	329.299	-0.119905	-0.297736	0.66865	0.99995	no

ENSG00000166428	ENSG00000166428	PLD4	14:105391152-105399574	q1	q2	NOTEST	0.111561	0.106618	-0.0653809	0	1	1	no

ENSG00000166432	ENSG00000166432	ZMAT1	X:101137261-101187004	q1	q2	OK	6.4132	6.16945	-0.0559022	-0.106509	0.8777	0.99995	no

ENSG00000166435	ENSG00000166435	XRRA1	11:74459912-74660245	q1	q2	OK	2.67621	2.4186	-0.146021	-0.0640283	0.83395	0.99995	no

ENSG00000166436	ENSG00000166436	TRIM66	11:8633583-8693413	q1	q2	OK	4.20139	3.88985	-0.11115	-0.132531	0.7718	0.99995	no

ENSG00000166439	ENSG00000166439	RNF169	11:74459912-74660245	q1	q2	OK	7.48217	7.78668	0.0575517	0.121492	0.86135	0.99995	no

ENSG00000166441	ENSG00000166441	RPL27A	11:8703957-8932498	q1	q2	OK	702.297	657.247	-0.0956456	-0.177903	0.79705	0.99995	no

ENSG00000166444	ENSG00000166444	ST5	11:8703957-8932498	q1	q2	OK	26.0446	25.1353	-0.0512672	-0.0250669	0.94035	0.99995	no

ENSG00000166446	ENSG00000166446	CDYL2	16:80631802-80838226	q1	q2	OK	1.95665	1.95974	0.00227348	0.00307257	0.99505	0.99995	no

ENSG00000166448	ENSG00000166448	TMEM130	7:98444110-98468394	q1	q2	NOTEST	0	0	0	0	1	1	no

ENSG00000166450	ENSG00000166450	PRTG	15:55903743-56035288	q1	q2	OK	40.0665	47.7683	0.253655	0.320786	0.4601	0.99995	no

ENSG00000166451	ENSG00000166451	CENPN	16:81009697-81069364	q1	q2	OK	48.1859	46.19	-0.0610282	-0.0699737	0.906	0.99995	no

ENSG00000166452	ENSG00000166452	AKIP1	11:8932685-8954553	q1	q2	OK	18.3646	18.5391	0.0136387	0.0235865	0.9733	0.99995	no

ENSG00000166454	ENSG00000166454	ATMIN	16:81069451-81080963	q1	q2	OK	30.1586	32.5742	0.111158	0.222874	0.7087	0.99995	no

ENSG00000166455	ENSG00000166455	C16orf46	16:81087101-81130008	q1	q2	OK	2.59006	2.18623	-0.244538	-0.137738	0.81005	0.99995	no

ENSG00000166471	ENSG00000166471	TMEM41B	11:9302200-9336327	q1	q2	OK	21.3025	22.3544	0.069533	0.122442	0.8511	0.99995	no

ENSG00000166473	ENSG00000166473	PKD1L2	16:81134479-81253975	q1	q2	NOTEST	0.0966414	0.0375936	-1.36216	0	1	1	no

ENSG00000166477	ENSG00000166477	LEO1	15:52121824-52392720	q1	q2	OK	23.0939	25.4932	0.142599	0.122455	0.86	0.99995	no

ENSG00000166478	ENSG00000166478	ZNF143	11:9481746-9550071	q1	q2	OK	18.6952	18.6145	-0.00624346	-0.00876407	0.9893	0.99995	no

ENSG00000166479	ENSG00000166479	TMX3	18:66340924-66722426	q1	q2	OK	32.0185	37.0111	0.209053	0.278054	0.5349	0.99995	no

ENSG00000166482	ENSG00000166482	MFAP4	17:19240866-19290553	q1	q2	OK	11.3308	8.7151	-0.378663	-0.308805	0.7152	0.99995	no

ENSG00000166483	ENSG00000166483	WEE1	11:9595227-9615004	q1	q2	OK	49.9983	55.7143	0.156168	0.279832	0.66345	0.99995	no

ENSG00000166484	ENSG00000166484	MAPK7	17:19240866-19290553	q1	q2	OK	15.0508	15.4317	0.0360606	0.0428065	0.94	0.99995	no

ENSG00000166492	ENSG00000166492	FAM86GP	11:3431581-3443726	q1	q2	NOTEST	0	0	0	0	1	1	no

ENSG00000166501	ENSG00000166501	PRKCB	16:23847321-24231932	q1	q2	OK	1.05747	0.912756	-0.212314	-0.158218	0.6651	0.99995	no

ENSG00000166503	ENSG00000166503	HDGFRP3	15:83776158-84108197	q1	q2	OK	70.0739	73.9631	0.0779269	0.14772	0.8103	0.99995	no

ENSG00000166507	ENSG00000166507	NDST2	10:75545339-75571589	q1	q2	OK	12.4977	10.8199	-0.207968	-0.130058	0.7253	0.99995	no

ENSG00000166508	ENSG00000166508	MCM7	7:99690350-99726118	q1	q2	OK	179.392	175.832	-0.0289192	-0.0465462	0.94035	0.99995	no

ENSG00000166509	ENSG00000166509	CLEC3A	16:78048579-78100658	q1	q2	NOTEST	0.134347	0.066519	-1.01413	0	1	1	no

ENSG00000166510	ENSG00000166510	CCDC68	18:52568739-52626739	q1	q2	NOTEST	0.0313651	0.049404	0.655468	0	1	1	no

ENSG00000166523	ENSG00000166523	CLEC4E	12:8685900-8693559	q1	q2	NOTEST	0	0	0	0	1	1	no

ENSG00000166526	ENSG00000166526	ZNF3	7:99647389-99680171	q1	q2	OK	35.9333	35.3487	-0.0236641	-0.0406278	0.93855	0.99995	no

ENSG00000166527	ENSG00000166527	CLEC4D	12:8662070-8674962	q1	q2	NOTEST	0	0	0	0	1	1	no

ENSG00000166529	ENSG00000166529	ZSCAN21	7:99647389-99680171	q1	q2	OK	12.6101	13.2162	0.0677303	0.0724634	0.91455	0.99995	no

ENSG00000166530	ENSG00000166530	HSBP1P2	4:111173026-111173255	q1	q2	NOTEST	0	0	0	0	1	1	no

ENSG00000166532	ENSG00000166532	RIMKLB	12:8834195-9039597	q1	q2	OK	52.4243	54.1178	0.0458683	0.0874771	0.88725	0.99995	no

ENSG00000166535	ENSG00000166535	A2ML1	12:8834195-9039597	q1	q2	NOTEST	0.0267644	0.238375	3.15485	0	1	1	no

ENSG00000166546	ENSG00000166546	BEAN1	16:66461199-66586447	q1	q2	OK	0.968783	0.915138	-0.0821849	-0.0445217	0.94535	0.99995	no

ENSG00000166548	ENSG00000166548	TK2	16:66461199-66586447	q1	q2	OK	6.08969	5.44261	-0.16207	-0.219722	0.6922	0.99995	no

ENSG00000166557	ENSG00000166557	TMED3	15:79603403-79704334	q1	q2	OK	25.308	24.24	-0.0622041	-0.124606	0.86285	0.99995	no

ENSG00000166558	ENSG00000166558	SLC38A8	16:84043271-84076241	q1	q2	NOTEST	0.114665	0.118204	0.043851	0	1	1	no

ENSG00000166562	ENSG00000166562	SEC11C	18:56806708-56826068	q1	q2	OK	50.7676	53.1789	0.0669468	0.115863	0.86595	0.99995	no

ENSG00000166569	ENSG00000166569	CPLX4	18:56942387-56985881	q1	q2	NOTEST	0	0.0270034	inf	0	1	1	no

ENSG00000166573	ENSG00000166573	GALR1	18:74962504-74980858	q1	q2	NOTEST	0	0	0	0	1	1	no

ENSG00000166575	ENSG00000166575	TMEM135	11:86748885-87035401	q1	q2	OK	8.12955	10.9537	0.430164	0.288682	0.3523	0.99995	no

ENSG00000166578	ENSG00000166578	IQCD	12:113633245-113736390	q1	q2	OK	0.969121	0.772977	-0.326251	-0.144853	0.83605	0.99995	no

ENSG00000166579	ENSG00000166579	NDEL1	17:8316448-8534079	q1	q2	OK	15.572	19.7196	0.340677	0.1216	0.7745	0.99995	no

ENSG00000166582	ENSG00000166582	CENPV	17:15879873-16256970	q1	q2	OK	156.886	172.781	0.139231	0.122368	0.85585	0.99995	no

ENSG00000166589	ENSG00000166589	CDH16	16:66942024-66952887	q1	q2	NOTEST	0	0	0	0	1	1	no

ENSG00000166592	ENSG00000166592	RRAD	16:66955581-66959547	q1	q2	NOTEST	0.23224	0.226329	-0.0371919	0	1	1	no

ENSG00000166595	ENSG00000166595	FAM96B	16:66965958-66968326	q1	q2	OK	44.0589	38.8495	-0.18154	-0.342507	0.6351	0.99995	no

ENSG00000166596	ENSG00000166596	WDR16	17:9479943-9633008	q1	q2	NOTEST	0.243699	0.16915	-0.526799	0	1	1	no

ENSG00000166598	ENSG00000166598	HSP90B1	12:104235228-104359486	q1	q2	OK	473.224	456.668	-0.0513766	-0.0795966	0.8821	0.99995	no

ENSG00000166603	ENSG00000166603	MC4R	18:58038563-58040001	q1	q2	NOTEST	0.0575056	0.0199022	-1.53077	0	1	1	no

ENSG00000166619	ENSG00000166619	BLCAP	20:36120873-36156333	q1	q2	OK	38.8385	35.1991	-0.141948	-0.222909	0.7189	0.99995	no

ENSG00000166634	ENSG00000166634	SERPINB12	18:61223392-61234244	q1	q2	NOTEST	0	0.0456934	inf	0	1	1	no

ENSG00000166664	ENSG00000166664	CHRFAM7A	15:30653442-30686052	q1	q2	NOTEST	0.387311	0.485593	0.326256	0	1	1	no

ENSG00000166667	ENSG00000166667	SPDYE6	7:101986191-101996889	q1	q2	OK	0.581941	0.436402	-0.415215	-0.326224	0.65235	0.99995	no

ENSG00000166669	ENSG00000166669	ATF7IP2	16:10420290-10577495	q1	q2	OK	5.04584	5.97202	0.243126	0.242	0.63495	0.99995	no

ENSG00000166670	ENSG00000166670	MMP10	11:102617698-102714534	q1	q2	NOTEST	0.0947234	0.116117	0.293782	0	1	1	no

ENSG00000166676	ENSG00000166676	TVP23A	16:10837642-10912651	q1	q2	OK	0.717681	0.499485	-0.522901	-0.225591	0.64125	0.99995	no

ENSG00000166681	ENSG00000166681	NGFRAP1	X:102631267-102633005	q1	q2	OK	307.684	310.895	0.0149783	0.03637	0.9586	0.99995	no

ENSG00000166682	ENSG00000166682	TMPRSS5	11:113558271-113577095	q1	q2	OK	1.48604	1.39378	-0.0924695	-0.0850815	0.86295	0.99995	no

ENSG00000166685	ENSG00000166685	COG1	17:71161115-71258491	q1	q2	OK	15.6128	15.1225	-0.046034	-0.0372137	0.9347	0.99995	no

ENSG00000166689	ENSG00000166689	PLEKHA7	11:16799841-17074583	q1	q2	OK	6.95372	6.20435	-0.164506	-0.120431	0.76355	0.99995	no

ENSG00000166693	ENSG00000166693	OR5M13P	11:56364901-56365838	q1	q2	NOTEST	0	0	0	0	1	1	no

ENSG00000166704	ENSG00000166704	ZNF606	19:58488420-58522600	q1	q2	OK	17.6354	16.8337	-0.0671148	-0.0970791	0.86545	0.99995	no

ENSG00000166707	ENSG00000166707	ZCCHC18	X:103343897-103401708	q1	q2	NOTEST	0.127475	0.198891	0.641763	0	1	1	no

ENSG00000166710	ENSG00000166710	B2M	15:45003674-45011075	q1	q2	OK	206.116	209.791	0.0254969	0.0563644	0.93515	0.99995	no

ENSG00000166716	ENSG00000166716	ZNF592	15:85291865-85349659	q1	q2	OK	11.0203	11.1032	0.0108121	0.0239196	0.9717	0.99995	no

ENSG00000166734	ENSG00000166734	CASC4	15:44580926-44719342	q1	q2	OK	37.4641	37.0915	-0.0144195	-0.0312283	0.9596	0.99995	no

ENSG00000166736	ENSG00000166736	HTR3A	11:113845602-113861035	q1	q2	NOTEST	0	0	0	0	1	1	no

ENSG00000166741	ENSG00000166741	NNMT	11:113930314-114227293	q1	q2	OK	0.929356	0.772719	-0.266288	-0.0839803	0.82155	0.99995	no

ENSG00000166743	ENSG00000166743	ACSM1	16:20621564-20936328	q1	q2	NOTEST	0.0140082	0.102201	2.86706	0	1	1	no

ENSG00000166747	ENSG00000166747	AP1G1	16:71762912-71843104	q1	q2	OK	38.1184	41.1763	0.111326	0.147145	0.72785	0.99995	no

ENSG00000166748	ENSG00000166748	AGBL1	15:86685226-87572283	q1	q2	NOTEST	0	0.0168713	inf	0	1	1	no

ENSG00000166750	ENSG00000166750	SLFN5	17:33570054-33600674	q1	q2	OK	3.05597	3.20315	0.0678595	0.0788145	0.89425	0.99995	no

ENSG00000166762	ENSG00000166762	CATSPER2	15:43885251-44010458	q1	q2	OK	1.09207	1.04534	-0.0630861	-0.0310331	0.93815	0.99995	no

ENSG00000166763	ENSG00000166763	STRCP1	15:43885251-44010458	q1	q2	NOTEST	0.00462728	0.00481495	0.057357	0	1	1	no

ENSG00000166770	ENSG00000166770	ZNF667-AS1	19:56950693-57012035	q1	q2	OK	2.41221	2.75695	0.192722	0.160377	0.7775	0.99995	no

ENSG00000166780	ENSG00000166780	C16orf45	16:15489610-15737023	q1	q2	OK	39.4374	33.7566	-0.224391	-0.229654	0.7173	0.99995	no

ENSG00000166783	ENSG00000166783	KIAA0430	16:15489610-15737023	q1	q2	OK	20.3087	20.2867	-0.00156857	-0.00175608	0.99725	0.99995	no

ENSG00000166787	ENSG00000166787	SAA3P	11:18134018-18137679	q1	q2	NOTEST	0	0	0	0	1	1	no

ENSG00000166788	ENSG00000166788	SAAL1	11:18091481-18127638	q1	q2	OK	31.832	39.2026	0.300471	0.541717	0.39665	0.99995	no

ENSG00000166793	ENSG00000166793	YPEL4	11:57405496-57429340	q1	q2	OK	5.87037	5.04201	-0.219451	-0.229519	0.73785	0.99995	no

ENSG00000166794	ENSG00000166794	PPIB	15:64443913-64455404	q1	q2	OK	291.555	276.407	-0.0769776	-0.180764	0.8002	0.99995	no

ENSG00000166796	ENSG00000166796	LDHC	11:18433853-18473605	q1	q2	NOTEST	0	0	0	0	1	1	no

ENSG00000166797	ENSG00000166797	FAM96A	15:64364757-64386217	q1	q2	OK	46.2202	50.1062	0.116465	0.24284	0.72675	0.99995	no

ENSG00000166800	ENSG00000166800	LDHAL6A	11:18477370-18548779	q1	q2	NOTEST	0.113712	0.145309	0.353734	0	1	1	no

ENSG00000166801	ENSG00000166801	FAM111A	11:58897605-58922512	q1	q2	OK	13.2944	13.1455	-0.0162562	-0.0211027	0.9669	0.99995	no

ENSG00000166803	ENSG00000166803	KIAA0101	15:64457715-64679886	q1	q2	OK	84.9752	85.2998	0.00550103	0.00964076	0.9888	0.99995	no

ENSG00000166813	ENSG00000166813	KIF7	15:90048507-90198682	q1	q2	OK	5.73266	5.17045	-0.148916	-0.135307	0.8119	0.99995	no

ENSG00000166816	ENSG00000166816	LDHD	16:75145757-75150669	q1	q2	NOTEST	0.0417741	0.01304	-1.67967	0	1	1	no

ENSG00000166819	ENSG00000166819	PLIN1	15:90207595-90234014	q1	q2	NOTEST	0.0175727	0.0515293	1.55206	0	1	1	no

ENSG00000166821	ENSG00000166821	PEX11A	15:90207595-90234014	q1	q2	OK	1.10081	1.25262	0.186391	0.176372	0.77945	0.99995	no

ENSG00000166822	ENSG00000166822	TMEM170A	16:75327595-75499395	q1	q2	OK	18.5924	19.0866	0.0378537	0.0393512	0.9356	0.99995	no

ENSG00000166823	ENSG00000166823	MESP1	15:90291891-90294541	q1	q2	NOTEST	0.112379	0.352135	1.64776	0	1	1	no

ENSG00000166825	ENSG00000166825	ANPEP	15:90328119-90358633	q1	q2	OK	3.04526	3.79744	0.318462	0.260851	0.56035	0.99995	no

ENSG00000166828	ENSG00000166828	SCNN1G	16:23194035-23228204	q1	q2	NOTEST	0	0	0	0	1	1	no

ENSG00000166831	ENSG00000166831	RBPMS2	15:65032090-65067786	q1	q2	OK	20.9379	19.719	-0.0865327	-0.182839	0.79375	0.99995	no

ENSG00000166833	ENSG00000166833	NAV2	11:19372270-20143144	q1	q2	OK	12.8963	12.6919	-0.0230422	-0.0387818	0.93885	0.99995	no

ENSG00000166839	ENSG00000166839	ANKDD1A	15:65134081-65251042	q1	q2	OK	3.83119	3.35835	-0.19004	-0.173813	0.75955	0.99995	no

ENSG00000166840	ENSG00000166840	GLYATL1	11:58672870-58826132	q1	q2	OK	1.2077	1.06086	-0.187025	-0.0980494	0.8299	0.99995	no

ENSG00000166845	ENSG00000166845	C18orf54	18:51850727-51911588	q1	q2	OK	13.5082	15.4978	0.198223	0.460999	0.49865	0.99995	no

ENSG00000166847	ENSG00000166847	DCTN5	16:23652712-23681195	q1	q2	OK	35.285	37.0037	0.0686138	0.148663	0.8085	0.99995	no

ENSG00000166848	ENSG00000166848	TERF2IP	16:75661621-75795770	q1	q2	OK	56.0543	53.0366	-0.0798365	-0.12853	0.8585	0.99995	no

ENSG00000166851	ENSG00000166851	PLK1	16:23688976-23724821	q1	q2	OK	54.1506	53.6375	-0.0137334	-0.0303376	0.96115	0.99995	no

ENSG00000166855	ENSG00000166855	CLPX	15:65440556-65477680	q1	q2	OK	27.7934	25.8814	-0.10283	-0.145456	0.8167	0.99995	no

ENSG00000166856	ENSG00000166856	GPR182	12:57388229-57392225	q1	q2	NOTEST	0.0979143	0.0649276	-0.592688	0	1	1	no

ENSG00000166860	ENSG00000166860	ZBTB39	12:57392617-57400230	q1	q2	OK	10.7218	11.03	0.0408866	0.095782	0.888	0.99995	no

ENSG00000166862	ENSG00000166862	CACNG2	22:36959967-37099603	q1	q2	NOTEST	0.133928	0.209281	0.643979	0	1	1	no

ENSG00000166863	ENSG00000166863	TAC3	12:57403783-57444982	q1	q2	NOTEST	0.0770551	0.18022	1.2258	0	1	1	no

ENSG00000166866	ENSG00000166866	MYO1A	12:57403783-57444982	q1	q2	NOTEST	0.241477	0.081123	-1.5737	0	1	1	no

ENSG00000166869	ENSG00000166869	CHP2	16:23765947-23770272	q1	q2	NOTEST	0	0	0	0	1	1	no

ENSG00000166881	ENSG00000166881	TMEM194A	12:57449425-57481846	q1	q2	OK	20.909	21.1871	0.019058	0.0450859	0.9455	0.99995	no

ENSG00000166884	ENSG00000166884	OR4D6	11:59224410-59225464	q1	q2	NOTEST	0	0	0	0	1	1	no

ENSG00000166886	ENSG00000166886	NAB2	12:57482676-57607134	q1	q2	OK	38.2671	38.1045	-0.00614056	-0.00466683	0.99385	0.99995	no

ENSG00000166887	ENSG00000166887	VPS39	15:42450898-42500514	q1	q2	OK	27.1622	25.8255	-0.0728047	-0.123822	0.803	0.99995	no

ENSG00000166888	ENSG00000166888	STAT6	12:57482676-57607134	q1	q2	OK	3.36305	3.98639	0.245313	0.0380915	0.8007	0.99995	no

ENSG00000166889	ENSG00000166889	PATL1	11:59383901-59436453	q1	q2	OK	17.5208	18.3894	0.0698094	0.145557	0.80695	0.99995	no

ENSG00000166896	ENSG00000166896	XRCC6BP1	12:58335323-58351052	q1	q2	NOTEST	0	0	0	0	1	1	no

ENSG00000166897	ENSG00000166897	ELFN2	22:37735623-37823505	q1	q2	NOTEST	0.126433	0.138403	0.130504	0	1	1	no

ENSG00000166900	ENSG00000166900	STX3	11:59480295-59573354	q1	q2	OK	2.29115	2.3393	0.0300046	0.0288155	0.9585	0.99995	no

ENSG00000166902	ENSG00000166902	MRPL16	11:59573607-59578345	q1	q2	OK	31.2173	30.7484	-0.0218331	-0.0438904	0.94695	0.99995	no

ENSG00000166908	ENSG00000166908	PIP4K2C	12:57984956-57997198	q1	q2	OK	11.6725	11.7236	0.00630495	0.0119092	0.98295	0.99995	no

ENSG00000166912	ENSG00000166912	MTMR10	15:31196054-31283810	q1	q2	OK	9.41329	10.0156	0.0894737	0.12901	0.8318	0.99995	no

ENSG00000166913	ENSG00000166913	YWHAB	20:43514316-43537173	q1	q2	OK	115.406	126.873	0.136666	0.294809	0.6169	0.99995	no

ENSG00000166917	ENSG00000166917	MIR202HG	10:135059981-135061395	q1	q2	NOTEST	0	0	0	0	1	1	no

ENSG00000166920	ENSG00000166920	C15orf48	15:45722726-45878488	q1	q2	NOTEST	0.0724096	0.133736	0.885134	0	1	1	no

ENSG00000166922	ENSG00000166922	SCG5	15:32933876-32989299	q1	q2	OK	1.51636	1.40557	-0.109456	-0.106277	0.8534	0.99995	no

ENSG00000166923	ENSG00000166923	GREM1	15:33009470-33026870	q1	q2	NOTEST	0.300075	0.234148	-0.357898	0	1	1	no

ENSG00000166924	ENSG00000166924	NYAP1	7:100081549-100092422	q1	q2	OK	3.55373	3.24644	-0.130478	-0.2099	0.7562	0.99995	no

ENSG00000166925	ENSG00000166925	TSC22D4	7:100054237-100076902	q1	q2	OK	13.9762	12.4623	-0.165396	-0.300252	0.6608	0.99995	no

ENSG00000166926	ENSG00000166926	MS4A6E	11:60102303-60185161	q1	q2	NOTEST	0	0	0	0	1	1	no

ENSG00000166927	ENSG00000166927	MS4A7	11:60102303-60185161	q1	q2	NOTEST	0	0	0	0	1	1	no

ENSG00000166928	ENSG00000166928	MS4A14	11:60102303-60185161	q1	q2	NOTEST	0	0	0	0	1	1	no

ENSG00000166930	ENSG00000166930	MS4A5	11:60197061-60222687	q1	q2	NOTEST	0	0	0	0	1	1	no

ENSG00000166938	ENSG00000166938	DIS3L	15:66570835-66626236	q1	q2	OK	12.9398	15.4198	0.252969	0.337676	0.4424	0.99995	no

ENSG00000166946	ENSG00000166946	CCNDBP1	15:43398422-43513481	q1	q2	OK	20.0604	20.3591	0.0213175	0.0359381	0.96305	0.99995	no

ENSG00000166947	ENSG00000166947	EPB42	15:43398422-43513481	q1	q2	NOTEST	0.178259	0.11553	-0.625703	0	1	1	no

ENSG00000166948	ENSG00000166948	TGM6	20:2361553-2413399	q1	q2	NOTEST	0	0	0	0	1	1	no

ENSG00000166949	ENSG00000166949	SMAD3	15:67356100-67487533	q1	q2	OK	22.1699	20.8922	-0.0856429	-0.18738	0.7609	0.99995	no

ENSG00000166959	ENSG00000166959	MS4A8	11:60467046-60483284	q1	q2	NOTEST	0.0205818	0	#NAME?	0	1	1	no

ENSG00000166960	ENSG00000166960	CCDC178	18:30413416-31021065	q1	q2	NOTEST	0.32558	0.364014	0.16098	0	1	1	no

ENSG00000166961	ENSG00000166961	MS4A15	11:60524425-60544205	q1	q2	NOTEST	0	0	0	0	1	1	no

ENSG00000166963	ENSG00000166963	MAP1A	15:43803155-43823818	q1	q2	OK	11.2423	10.019	-0.166199	-0.411195	0.56075	0.99995	no

ENSG00000166965	ENSG00000166965	RCCD1	15:91473409-91506349	q1	q2	OK	5.49179	4.35467	-0.334713	-0.258344	0.70375	0.99995	no

ENSG00000166971	ENSG00000166971	AKTIP	16:53467888-53538323	q1	q2	OK	18.0563	17.1622	-0.0732636	-0.0730497	0.8971	0.99995	no

ENSG00000166974	ENSG00000166974	MAPRE2	18:32556891-32723434	q1	q2	OK	20.7553	21.1214	0.0252296	0.0573835	0.9296	0.99995	no

ENSG00000166979	ENSG00000166979	EVA1C	21:33784313-33887707	q1	q2	NOTEST	0.188915	0.0918861	-1.03982	0	1	1	no

ENSG00000166984	ENSG00000166984	TCP10L2	6:167559901-167610401	q1	q2	NOTEST	0	0	0	0	1	1	no

ENSG00000166986	ENSG00000166986	MARS	12:57853917-57914300	q1	q2	OK	122.369	119.474	-0.0345381	-0.0562643	0.91725	0.99995	no

ENSG00000166987	ENSG00000166987	MBD6	12:57914492-57941114	q1	q2	OK	20.0659	19.099	-0.0712514	-0.0581135	0.88655	0.99995	no

ENSG00000166997	ENSG00000166997	CNPY4	7:99690350-99726118	q1	q2	OK	24.8951	23.528	-0.0814863	-0.0362439	0.9673	0.99995	no

ENSG00000167004	ENSG00000167004	PDIA3	15:44019115-44095241	q1	q2	OK	245.823	246.942	0.00654997	0.0108439	0.9869	0.99995	no

ENSG00000167005	ENSG00000167005	NUDT21	16:56463044-56554195	q1	q2	OK	74.6439	80.3244	0.105814	0.186045	0.7604	0.99995	no

ENSG00000167011	ENSG00000167011	NAT16	7:100813773-100823557	q1	q2	OK	0.83168	0.681271	-0.287801	-0.24677	0.71435	0.99995	no

ENSG00000167014	ENSG00000167014	C15orf43	15:45248899-45271427	q1	q2	NOTEST	0	0	0	0	1	1	no

ENSG00000167034	ENSG00000167034	NKX3-1	8:23536205-23540440	q1	q2	NOTEST	0.33161	0.449892	0.440091	0	1	1	no

ENSG00000167037	ENSG00000167037	SGSM1	22:25202235-25323545	q1	q2	NOTEST	0.188263	0.15307	-0.298566	0	1	1	no

ENSG00000167046	ENSG00000167046	RP11-93B14.6	20:61272070-61317137	q1	q2	NOTEST	0	0	0	0	1	1	no

ENSG00000167065	ENSG00000167065	DUSP18	22:31031638-31065003	q1	q2	OK	3.40695	3.08701	-0.142269	-0.148848	0.80135	0.99995	no

ENSG00000167074	ENSG00000167074	TEF	22:41763336-41795330	q1	q2	OK	4.38549	4.65472	0.085955	0.159871	0.8155	0.99995	no

ENSG00000167077	ENSG00000167077	MEI1	22:42095502-42195460	q1	q2	NOTEST	0.107619	0.10447	-0.0428448	0	1	1	no

ENSG00000167080	ENSG00000167080	B4GALNT2	17:47209821-47247351	q1	q2	NOTEST	0	0	0	0	1	1	no

ENSG00000167081	ENSG00000167081	PBX3	9:128509623-128729656	q1	q2	OK	48.9894	47.6422	-0.0402278	-0.0835282	0.892	0.99995	no

ENSG00000167083	ENSG00000167083	GNGT2	17:47280152-47300587	q1	q2	NOTEST	0	0.124462	inf	0	1	1	no

ENSG00000167085	ENSG00000167085	PHB	17:47448101-47554350	q1	q2	OK	76.5544	80.9938	0.0813258	0.167182	0.8083	0.99995	no

ENSG00000167088	ENSG00000167088	SNRPD1	18:19192227-19213485	q1	q2	OK	74.7317	85.3021	0.190861	0.445942	0.5183	0.99995	no

ENSG00000167094	ENSG00000167094	TTC16	9:130374543-130497604	q1	q2	NOTEST	0.00506324	0.00464758	-0.123582	0	1	1	no

ENSG00000167098	ENSG00000167098	SUN5	20:31571578-31592239	q1	q2	NOTEST	0	0	0	0	1	1	no

ENSG00000167100	ENSG00000167100	SAMD14	17:48172100-48207246	q1	q2	OK	18.2127	17.3186	-0.0726214	-0.0959301	0.85515	0.99995	no

ENSG00000167103	ENSG00000167103	PIP5KL1	9:130683157-130693076	q1	q2	OK	0.851969	1.05229	0.304665	0.230442	0.64585	0.99995	no

ENSG00000167104	ENSG00000167104	BPIFB6	20:31619453-31631853	q1	q2	NOTEST	0	0	0	0	1	1	no

ENSG00000167105	ENSG00000167105	TMEM92	17:48348766-48358844	q1	q2	NOTEST	0.0922545	0.118499	0.361185	0	1	1	no

ENSG00000167106	ENSG00000167106	FAM102A	9:130696781-130742792	q1	q2	OK	13.1539	12.2521	-0.102461	-0.094508	0.80995	0.99995	no

ENSG00000167107	ENSG00000167107	ACSF2	17:48503518-48552206	q1	q2	OK	14.9776	12.5425	-0.255988	-0.333966	0.55415	0.99995	no

ENSG00000167110	ENSG00000167110	GOLGA2	9:131018107-131051269	q1	q2	OK	31.8845	32.3151	0.019353	0.0241595	0.9591	0.99995	no

ENSG00000167112	ENSG00000167112	TRUB2	9:131071381-131096351	q1	q2	OK	13.9628	13.2107	-0.0798901	-0.0990908	0.8778	0.99995	no

ENSG00000167113	ENSG00000167113	COQ4	9:131071381-131096351	q1	q2	OK	30.2726	26.4841	-0.192889	-0.289053	0.66985	0.99995	no

ENSG00000167114	ENSG00000167114	SLC27A4	9:131102924-131123749	q1	q2	OK	17.2022	15.4211	-0.157694	-0.310821	0.65415	0.99995	no

ENSG00000167117	ENSG00000167117	LINC00483	17:48838389-48844918	q1	q2	NOTEST	0	0	0	0	1	1	no

ENSG00000167118	ENSG00000167118	URM1	9:131133597-131153015	q1	q2	OK	51.0385	49.186	-0.0533395	-0.11227	0.8748	0.99995	no

ENSG00000167123	ENSG00000167123	CERCAM	9:131174029-131199626	q1	q2	OK	20.4993	16.9564	-0.27374	-0.436997	0.418	0.99995	no

ENSG00000167130	ENSG00000167130	DOLPP1	9:131843378-131852717	q1	q2	OK	18.5028	16.3729	-0.176435	-0.378023	0.59705	0.99995	no

ENSG00000167131	ENSG00000167131	CCDC103	17:42923720-42994305	q1	q2	OK	3.31774	3.09591	-0.0998366	-0.0359666	0.92945	0.99995	no

ENSG00000167136	ENSG00000167136	ENDOG	9:131580752-131592100	q1	q2	OK	8.24599	7.29472	-0.17684	-0.139219	0.84715	0.99995	no

ENSG00000167139	ENSG00000167139	TBC1D21	15:74165948-74181555	q1	q2	NOTEST	0	0	0	0	1	1	no

ENSG00000167157	ENSG00000167157	PRRX2	9:132427919-132484875	q1	q2	OK	2.95583	2.4023	-0.299144	-0.320103	0.64535	0.99995	no

ENSG00000167165	ENSG00000167165	UGT1A6	2:234526290-234681956	q1	q2	NOTEST	0.0303737	0.0484567	0.673874	0	1	1	no

ENSG00000167173	ENSG00000167173	C15orf39	15:75487983-75504510	q1	q2	OK	4.46647	4.22052	-0.0817131	-0.143334	0.8292	0.99995	no

ENSG00000167178	ENSG00000167178	ISLR2	15:74392651-74430881	q1	q2	OK	2.25187	2.66647	0.243808	0.282419	0.59165	0.99995	no

ENSG00000167182	ENSG00000167182	SP2	17:45973515-46018776	q1	q2	OK	10.2629	10.1634	-0.0140591	-0.028924	0.9659	0.99995	no

ENSG00000167183	ENSG00000167183	PRR15L	17:46018871-46073562	q1	q2	NOTEST	0	0	0	0	1	1	no

ENSG00000167186	ENSG00000167186	COQ7	16:18995255-19091417	q1	q2	OK	14.6126	14.2378	-0.0374871	-0.0551882	0.9255	0.99995	no

ENSG00000167191	ENSG00000167191	GPRC5B	16:19566561-19897489	q1	q2	OK	30.8897	26.4156	-0.225734	-0.297674	0.664	0.99995	no

ENSG00000167193	ENSG00000167193	CRK	17:1323982-1366456	q1	q2	OK	38.6951	39.5675	0.0321622	0.0790124	0.9092	0.99995	no

ENSG00000167194	ENSG00000167194	C16orf92	16:30034654-30064299	q1	q2	NOTEST	0	0.185647	inf	0	1	1	no

ENSG00000167195	ENSG00000167195	GOLGA6C	15:75550939-75562540	q1	q2	NOTEST	0	0	0	0	1	1	no

ENSG00000167196	ENSG00000167196	FBXO22	15:76196199-76227609	q1	q2	OK	29.2187	25.7934	-0.179891	-0.314834	0.6025	0.99995	no

ENSG00000167202	ENSG00000167202	TBC1D2B	15:78268383-78370066	q1	q2	OK	7.92952	6.22364	-0.349476	-0.358113	0.37715	0.99995	no

ENSG00000167207	ENSG00000167207	NOD2	16:50727513-50766988	q1	q2	NOTEST	0.159607	0.0656179	-1.28236	0	1	1	no

ENSG00000167208	ENSG00000167208	SNX20	16:50700121-50715264	q1	q2	NOTEST	0.0734623	4.19E-05	-10.7771	0	1	1	no

ENSG00000167210	ENSG00000167210	LOXHD1	18:44056934-44236996	q1	q2	NOTEST	0	0	0	0	1	1	no

ENSG00000167216	ENSG00000167216	KATNAL2	18:44388352-44627658	q1	q2	OK	3.32969	2.55348	-0.382928	-0.187225	0.7053	0.99995	no

ENSG00000167220	ENSG00000167220	HDHD2	18:44632316-44702745	q1	q2	OK	40.0067	41.822	0.0640188	0.100103	0.87055	0.99995	no

ENSG00000167230	ENSG00000167230	C17orf78	17:35441922-35766909	q1	q2	NOTEST	0.0500357	0.0992022	0.987413	0	1	1	no

ENSG00000167232	ENSG00000167232	ZNF91	19:23487792-23578362	q1	q2	OK	22.0564	22.8383	0.0502613	0.0796353	0.8923	0.99995	no

ENSG00000167236	ENSG00000167236	CCL23	17:34340095-34345005	q1	q2	NOTEST	0	0	0	0	1	1	no

ENSG00000167244	ENSG00000167244	IGF2	11:2150341-2182571	q1	q2	OK	88.5255	89.6762	0.0186319	0.0300659	0.96175	0.99995	no

ENSG00000167257	ENSG00000167257	RNF214	11:117103340-117283984	q1	q2	OK	17.1635	18.8227	0.133125	0.124593	0.8275	0.99995	no

ENSG00000167258	ENSG00000167258	CDK12	17:37617763-37721160	q1	q2	OK	20.7452	21.1038	0.0247283	0.0334623	0.9398	0.99995	no

ENSG00000167261	ENSG00000167261	DPEP2	16:68021296-68272005	q1	q2	NOTEST	0	0.0164668	inf	0	1	1	no

ENSG00000167264	ENSG00000167264	DUS2	16:68021296-68272005	q1	q2	OK	7.06403	6.91641	-0.0304692	-0.0179084	0.9749	0.99995	no

ENSG00000167272	ENSG00000167272	POP5	12:121016566-121019201	q1	q2	OK	36.5479	35.8402	-0.0282105	-0.0527167	0.94425	0.99995	no

ENSG00000167280	ENSG00000167280	ENGASE	17:77071020-77084681	q1	q2	OK	13.7646	12.1453	-0.180573	-0.181933	0.6474	0.99995	no

ENSG00000167281	ENSG00000167281	RBFOX3	17:77085426-77613550	q1	q2	NOTEST	0.458538	0.399887	-0.197449	0	1	1	no

ENSG00000167283	ENSG00000167283	ATP5L	11:118230299-118302211	q1	q2	OK	4.82726	4.43452	-0.122427	-0.062846	0.9283	0.99995	no

ENSG00000167286	ENSG00000167286	CD3D	11:118209668-118213459	q1	q2	NOTEST	0	0	0	0	1	1	no

ENSG00000167291	ENSG00000167291	TBC1D16	17:77906141-78009647	q1	q2	OK	28.5602	29.9227	0.0672322	0.115047	0.82405	0.99995	no

ENSG00000167302	ENSG00000167302	ENTHD2	17:79202076-79212891	q1	q2	OK	11.5062	9.35445	-0.298689	-0.343439	0.5524	0.99995	no

ENSG00000167306	ENSG00000167306	MYO5B	18:47309868-47721463	q1	q2	NOTEST	0.356575	0.354847	-0.00700982	0	1	1	no

ENSG00000167311	ENSG00000167311	ART5	11:3659732-3663546	q1	q2	OK	1.39408	0.766114	-0.863679	-0.789227	0.2359	0.99995	no

ENSG00000167315	ENSG00000167315	ACAA2	18:47309868-47721463	q1	q2	OK	53.7481	52.6387	-0.0300892	-0.0671291	0.92525	0.99995	no

ENSG00000167323	ENSG00000167323	STIM1	11:3875547-4114439	q1	q2	OK	15.0335	10.3264	-0.54184	-0.72082	0.14635	0.99995	no

ENSG00000167325	ENSG00000167325	RRM1	11:4115936-4160106	q1	q2	OK	91.5889	96.1423	0.0699994	0.157301	0.8063	0.99995	no

ENSG00000167332	ENSG00000167332	OR51E2	11:4701400-4719084	q1	q2	NOTEST	0	0.00956012	inf	0	1	1	no

ENSG00000167333	ENSG00000167333	TRIM68	11:4619901-4629489	q1	q2	OK	5.19608	5.35019	0.0421674	0.0515935	0.9237	0.99995	no

ENSG00000167346	ENSG00000167346	MMP26	11:4726156-5013659	q1	q2	NOTEST	0.0300775	0	#NAME?	0	1	1	no

ENSG00000167355	ENSG00000167355	AC104389.28	11:5274419-5667019	q1	q2	NOTEST	0.0246433	0.0240142	-0.0373034	0	1	1	no

ENSG00000167359	ENSG00000167359	OR51I1	11:5274419-5667019	q1	q2	NOTEST	0	0.0330116	inf	0	1	1	no

ENSG00000167360	ENSG00000167360	OR51Q1	11:5274419-5667019	q1	q2	NOTEST	0	0	0	0	1	1	no

ENSG00000167363	ENSG00000167363	FN3K	17:80693450-80709073	q1	q2	OK	7.5588	5.78774	-0.385156	-0.512979	0.41725	0.99995	no

ENSG00000167371	ENSG00000167371	PRRT2	16:29817426-29859355	q1	q2	OK	17.0205	13.6204	-0.321506	-0.230174	0.74695	0.99995	no

ENSG00000167377	ENSG00000167377	ZNF23	16:71481499-71598992	q1	q2	OK	10.7985	9.59932	-0.169823	-0.259411	0.696	0.99995	no

ENSG00000167378	ENSG00000167378	IRGQ	19:44047191-44100287	q1	q2	OK	12.5671	11.7403	-0.0981802	-0.153352	0.8242	0.99995	no

ENSG00000167380	ENSG00000167380	ZNF226	19:44669225-44682534	q1	q2	OK	14.7921	14.0374	-0.0755548	-0.111421	0.846	0.99995	no

ENSG00000167383	ENSG00000167383	ZNF229	19:44830707-44952766	q1	q2	OK	0.607219	1.07955	0.830135	0.353109	0.49885	0.99995	no

ENSG00000167384	ENSG00000167384	ZNF180	19:44979853-45004576	q1	q2	OK	7.72124	8.8552	0.197694	0.364831	0.5667	0.99995	no

ENSG00000167390	ENSG00000167390	POM121L3P	20:23969928-23971091	q1	q2	NOTEST	0	0	0	0	1	1	no

ENSG00000167393	ENSG00000167393	PPP2R3B	X:294697-347690	q1	q2	OK	26.1455	27.8683	0.092064	0.139519	0.82595	0.99995	no

ENSG00000167394	ENSG00000167394	ZNF668	16:31072163-31085641	q1	q2	OK	6.18108	5.70134	-0.116558	-0.193981	0.768	0.99995	no

ENSG00000167395	ENSG00000167395	ZNF646	16:31085742-31107301	q1	q2	OK	8.37418	7.51572	-0.156037	-0.158916	0.7326	0.99995	no

ENSG00000167397	ENSG00000167397	VKORC1	16:31085742-31107301	q1	q2	OK	35.6391	32.1125	-0.150326	-0.171807	0.79305	0.99995	no

ENSG00000167414	ENSG00000167414	GNG8	19:47137332-47137942	q1	q2	OK	2.95222	1.71543	-0.783231	-0.542364	0.60085	0.99995	no

ENSG00000167419	ENSG00000167419	LPO	17:56282802-56345879	q1	q2	NOTEST	0	0.0248399	inf	0	1	1	no

ENSG00000167434	ENSG00000167434	CA4	17:58227296-58248260	q1	q2	NOTEST	0.230464	0	#NAME?	0	1	1	no

ENSG00000167447	ENSG00000167447	SMG8	17:57232859-57292608	q1	q2	OK	11.1032	11.4578	0.0453538	0.0563184	0.9342	0.99995	no

ENSG00000167459	ENSG00000167459	LINC00905	19:16142116-16152926	q1	q2	NOTEST	0	0	0	0	1	1	no

ENSG00000167460	ENSG00000167460	TPM4	19:16177830-16213813	q1	q2	OK	311.036	358.431	0.204614	0.380319	0.5238	0.99995	no

ENSG00000167461	ENSG00000167461	RAB8A	19:16222438-16269386	q1	q2	OK	28.6044	31.2919	0.129553	0.214189	0.7339	0.99995	no

ENSG00000167468	ENSG00000167468	GPX4	19:1103935-1106787	q1	q2	OK	188.712	167.849	-0.169019	-0.381025	0.5523	0.99995	no

ENSG00000167470	ENSG00000167470	MIDN	19:1248551-1259142	q1	q2	OK	63.1641	65.115	0.0438846	0.0643187	0.8755	0.99995	no

ENSG00000167476	ENSG00000167476	JSRP1	19:2252250-2273487	q1	q2	OK	0.364148	0.691301	0.924787	0.0805801	0.5607	0.99995	no

ENSG00000167483	ENSG00000167483	FAM129C	19:17634109-17664648	q1	q2	NOTEST	0.257523	0.385795	0.583134	0	1	1	no

ENSG00000167487	ENSG00000167487	KLHL26	19:18747774-18781309	q1	q2	OK	3.69102	3.88984	0.0756884	0.105293	0.8571	0.99995	no

ENSG00000167491	ENSG00000167491	GATAD2A	19:19496634-19619740	q1	q2	OK	29.3921	29.6903	0.0145619	0.0223882	0.96555	0.99995	no

ENSG00000167494	ENSG00000167494	NOS2P2	17:18400885-18404348	q1	q2	NOTEST	0	0	0	0	1	1	no

ENSG00000167508	ENSG00000167508	MVD	16:88718342-88729569	q1	q2	OK	87.2621	95.376	0.128272	0.240382	0.66395	0.99995	no

ENSG00000167513	ENSG00000167513	CDT1	16:88869620-88875666	q1	q2	OK	19.1749	16.4389	-0.222105	-0.344468	0.5905	0.99995	no

ENSG00000167515	ENSG00000167515	TRAPPC2L	16:88880141-88933068	q1	q2	OK	38.4662	34.0403	-0.176349	-0.257411	0.7205	0.99995	no

ENSG00000167522	ENSG00000167522	ANKRD11	16:89334037-89556969	q1	q2	OK	63.5107	61.2955	-0.0512192	-0.0710919	0.8733	0.99995	no

ENSG00000167523	ENSG00000167523	SPATA33	16:89710838-89737680	q1	q2	OK	12.2069	11.7189	-0.0588481	-0.0551508	0.94975	0.99995	no

ENSG00000167524	ENSG00000167524	SGK494	17:26782769-26972472	q1	q2	OK	14.814	16.6128	0.165331	0.0548451	0.85535	0.99995	no

ENSG00000167525	ENSG00000167525	PROCA1	17:27030214-27038872	q1	q2	OK	9.41187	8.34915	-0.172851	-0.257559	0.69435	0.99995	no

ENSG00000167526	ENSG00000167526	RPL13	16:89627064-89630950	q1	q2	OK	908.769	758.492	-0.26078	-0.574867	0.41595	0.99995	no

ENSG00000167528	ENSG00000167528	ZNF641	12:48733790-48836730	q1	q2	OK	11.1066	11.089	-0.00229906	-0.00305602	0.99385	0.99995	no

ENSG00000167531	ENSG00000167531	LALBA	12:48961466-48963849	q1	q2	NOTEST	0	0	0	0	1	1	no

ENSG00000167535	ENSG00000167535	CACNB3	12:49207576-49222724	q1	q2	OK	26.2402	25.5289	-0.0396491	-0.0691909	0.90515	0.99995	no

ENSG00000167536	ENSG00000167536	DHRS13	17:27224755-27230089	q1	q2	OK	11.7864	10.9908	-0.100826	-0.137627	0.8268	0.99995	no

ENSG00000167543	ENSG00000167543	TP53I13	17:27887564-28513493	q1	q2	OK	22.2282	18.567	-0.259648	-0.157882	0.7692	0.99995	no

ENSG00000167548	ENSG00000167548	KMT2D	12:49388931-49453557	q1	q2	OK	25.5765	24.0316	-0.0898859	-0.0748353	0.9068	0.99995	no

ENSG00000167549	ENSG00000167549	CORO6	17:27887564-28513493	q1	q2	OK	3.17987	2.47307	-0.362662	-0.101416	0.6431	0.99995	no

ENSG00000167550	ENSG00000167550	RHEBL1	12:49458467-49463808	q1	q2	OK	0.810764	1.5207	0.907384	0.854524	0.20185	0.99995	no

ENSG00000167552	ENSG00000167552	TUBA1A	12:49578578-49667114	q1	q2	OK	2430.32	2370.46	-0.0359803	-0.0460754	0.94845	0.99995	no

ENSG00000167553	ENSG00000167553	TUBA1C	12:49578578-49667114	q1	q2	OK	40.7801	47.4987	0.220023	0.0308048	0.8279	0.99995	no

ENSG00000167554	ENSG00000167554	ZNF610	19:52772823-52871031	q1	q2	OK	5.22608	5.39908	0.0469849	0.0380101	0.9655	0.99995	no

ENSG00000167555	ENSG00000167555	ZNF528	19:52901101-52921665	q1	q2	OK	3.47678	3.05717	-0.185554	-0.162523	0.71355	0.99995	no

ENSG00000167562	ENSG00000167562	ZNF701	19:53030904-53090427	q1	q2	OK	3.24545	3.29556	0.0221061	0.0251611	0.9697	0.99995	no

ENSG00000167565	ENSG00000167565	SERTAD3	19:40946750-40950612	q1	q2	OK	10.4882	10.7149	0.0308512	0.0491258	0.94105	0.99995	no

ENSG00000167566	ENSG00000167566	NCKAP5L	12:50184928-50236912	q1	q2	OK	18.9285	17.4758	-0.115204	-0.237996	0.72395	0.99995	no

ENSG00000167578	ENSG00000167578	RAB4B	19:41277552-41406413	q1	q2	OK	13.0413	12.8451	-0.0218672	-0.0160285	0.9787	0.99995	no

ENSG00000167580	ENSG00000167580	AQP2	12:50344523-50359464	q1	q2	NOTEST	0	0	0	0	1	1	no

ENSG00000167588	ENSG00000167588	GPD1	12:50497601-50505102	q1	q2	NOTEST	0.169091	0.161241	-0.0685846	0	1	1	no

ENSG00000167595	ENSG00000167595	C19orf55	19:36249043-36261930	q1	q2	OK	16.7577	16.4666	-0.0252875	-0.0335542	0.96185	0.99995	no

ENSG00000167600	ENSG00000167600	CYP2S1	19:41698953-41713444	q1	q2	NOTEST	0.217178	0.0629848	-1.7858	0	1	1	no

ENSG00000167601	ENSG00000167601	AXL	19:41725107-41767671	q1	q2	OK	6.08329	5.42902	-0.16416	-0.322508	0.63855	0.99995	no

ENSG00000167604	ENSG00000167604	NFKBID	19:36378554-36393205	q1	q2	OK	2.45163	1.74804	-0.488001	-0.479366	0.42835	0.99995	no

ENSG00000167608	ENSG00000167608	TMC4	19:54663845-54676944	q1	q2	NOTEST	0	0	0	0	1	1	no

ENSG00000167612	ENSG00000167612	ANKRD33	12:52281743-52285448	q1	q2	NOTEST	0.352505	0.242211	-0.541379	0	1	1	no

ENSG00000167613	ENSG00000167613	LAIR1	19:54865361-54882165	q1	q2	NOTEST	0	0	0	0	1	1	no

ENSG00000167614	ENSG00000167614	TTYH1	19:54926392-54948080	q1	q2	OK	1.69718	2.37449	0.48448	0.385046	0.54505	0.99995	no

ENSG00000167615	ENSG00000167615	LENG8	19:54955990-54974894	q1	q2	OK	0.814976	1.80343	1.14591	0.680331	0.19685	0.99995	no

ENSG00000167617	ENSG00000167617	CDC42EP5	19:54976209-54984411	q1	q2	NOTEST	0.0357423	0.0699476	0.968644	0	1	1	no

ENSG00000167618	ENSG00000167618	LAIR2	19:55009099-55021897	q1	q2	NOTEST	0	0	0	0	1	1	no

ENSG00000167619	ENSG00000167619	TMEM145	19:42817476-42829214	q1	q2	OK	6.66081	4.37001	-0.60806	-0.611047	0.28445	0.99995	no

ENSG00000167625	ENSG00000167625	ZNF526	19:42724422-42732353	q1	q2	OK	4.65488	3.85915	-0.27046	-0.287227	0.5875	0.99995	no

ENSG00000167632	ENSG00000167632	TRAPPC9	8:140742585-141468678	q1	q2	OK	6.83107	7.06026	0.04761	0.072874	0.8982	0.99995	no

ENSG00000167633	ENSG00000167633	KIR3DL1	19:55235968-55378662	q1	q2	NOTEST	0	0	0	0	1	1	no

ENSG00000167634	ENSG00000167634	NLRP7	19:55434876-55512510	q1	q2	NOTEST	0	0	0	0	1	1	no

ENSG00000167635	ENSG00000167635	ZNF146	19:36673187-36737159	q1	q2	OK	80.4085	87.3999	0.120284	0.231298	0.68475	0.99995	no

ENSG00000167637	ENSG00000167637	ZNF283	19:44331443-44353307	q1	q2	OK	3.70075	4.11373	0.152629	0.18728	0.75385	0.99995	no

ENSG00000167641	ENSG00000167641	PPP1R14A	19:38734674-38795649	q1	q2	NOTEST	0.454405	0.165791	-1.45462	0	1	1	no

ENSG00000167642	ENSG00000167642	SPINT2	19:38734674-38795649	q1	q2	OK	3.87286	3.1504	-0.297866	-0.322655	0.56565	0.99995	no

ENSG00000167644	ENSG00000167644	C19orf33	19:38734674-38795649	q1	q2	NOTEST	0.095815	0	#NAME?	0	1	1	no

ENSG00000167645	ENSG00000167645	YIF1B	19:38795752-38807913	q1	q2	OK	22.6685	21.6023	-0.0695093	-0.129017	0.8597	0.99995	no

ENSG00000167646	ENSG00000167646	DNAAF3	19:55663136-55691809	q1	q2	OK	4.57	3.34734	-0.44918	-0.376086	0.4771	0.99995	no

ENSG00000167653	ENSG00000167653	PSCA	8:143738873-143764142	q1	q2	NOTEST	0	0	0	0	1	1	no

ENSG00000167654	ENSG00000167654	ATCAY	19:3879861-3928077	q1	q2	OK	14.1622	14.435	0.0275262	0.0375664	0.9407	0.99995	no

ENSG00000167656	ENSG00000167656	LY6D	8:143866295-143893536	q1	q2	NOTEST	0	0	0	0	1	1	no

ENSG00000167657	ENSG00000167657	DAPK3	19:3958450-3971121	q1	q2	OK	39.999	38.6484	-0.0495555	-0.0897844	0.89015	0.99995	no

ENSG00000167658	ENSG00000167658	EEF2	19:3976053-3985467	q1	q2	OK	485.411	503.614	0.053112	0.0645208	0.8943	0.99995	no

ENSG00000167664	ENSG00000167664	TMIGD2	19:4292228-4302428	q1	q2	NOTEST	0.14329	0.249851	0.802135	0	1	1	no

ENSG00000167670	ENSG00000167670	CHAF1A	19:4402658-4457819	q1	q2	OK	36.92	37.2428	0.0125591	0.0179857	0.9702	0.99995	no

ENSG00000167671	ENSG00000167671	UBXN6	19:4402658-4457819	q1	q2	OK	29.6718	24.6975	-0.264724	-0.297763	0.63695	0.99995	no

ENSG00000167674	ENSG00000167674	HDGFRP2	19:4472283-4517716	q1	q2	OK	56.415	49.5574	-0.18698	-0.395028	0.5131	0.99995	no

ENSG00000167676	ENSG00000167676	PLIN4	19:4472283-4517716	q1	q2	NOTEST	0.297698	0.255116	-0.222698	0	1	1	no

ENSG00000167680	ENSG00000167680	SEMA6B	19:4542599-4559820	q1	q2	OK	7.57891	6.2256	-0.283777	-0.5278	0.43175	0.99995	no

ENSG00000167685	ENSG00000167685	ZNF444	19:56643967-56672262	q1	q2	OK	24.1717	20.0027	-0.273123	-0.475941	0.40835	0.99995	no

ENSG00000167693	ENSG00000167693	NXN	17:702552-883010	q1	q2	OK	44.8493	41.067	-0.127107	-0.284145	0.6541	0.99995	no

ENSG00000167695	ENSG00000167695	FAM57A	17:635651-646206	q1	q2	OK	12.0854	12.7325	0.0752545	0.136992	0.83665	0.99995	no

ENSG00000167699	ENSG00000167699	GLOD4	17:660336-695749	q1	q2	OK	69.4324	75.3751	0.11848	0.241485	0.7014	0.99995	no

ENSG00000167700	ENSG00000167700	MFSD3	8:145734456-145736596	q1	q2	OK	7.17141	5.64273	-0.345865	-0.515637	0.4503	0.99995	no

ENSG00000167701	ENSG00000167701	GPT	8:145703351-145732557	q1	q2	OK	0.804662	0.646854	-0.314942	-0.0950611	0.77045	0.99995	no

ENSG00000167702	ENSG00000167702	KIFC2	8:145691425-145701718	q1	q2	OK	9.95341	7.10338	-0.486687	-0.51051	0.2871	0.99995	no

ENSG00000167703	ENSG00000167703	SLC43A2	17:1472560-1532180	q1	q2	OK	8.18133	6.75904	-0.275517	-0.424081	0.47805	0.99995	no

ENSG00000167705	ENSG00000167705	RILP	17:1549443-1553371	q1	q2	OK	1.41708	0.891078	-0.669294	-0.603695	0.4005	0.99995	no

ENSG00000167711	ENSG00000167711	SERPINF2	17:1646129-1658562	q1	q2	NOTEST	0.0616819	0.114718	0.895171	0	1	1	no

ENSG00000167716	ENSG00000167716	WDR81	17:1614804-1641893	q1	q2	OK	4.23869	3.63899	-0.22008	-0.171752	0.6567	0.99995	no

ENSG00000167720	ENSG00000167720	SRR	17:1963132-2284352	q1	q2	OK	22.1496	22.7625	0.0393781	0.0214467	0.95805	0.99995	no

ENSG00000167721	ENSG00000167721	TSR1	17:1963132-2284352	q1	q2	OK	19.2706	21.3654	0.148879	0.106742	0.7989	0.99995	no

ENSG00000167723	ENSG00000167723	TRPV3	17:3343312-3461289	q1	q2	NOTEST	0.300406	0.349388	0.217915	0	1	1	no

ENSG00000167733	ENSG00000167733	HSD11B1L	19:5674957-5720583	q1	q2	OK	14.913	11.481	-0.37732	-0.202861	0.60235	0.99995	no

ENSG00000167740	ENSG00000167740	CYB5D2	17:4046461-4167274	q1	q2	OK	10.8698	8.72229	-0.317548	-0.194104	0.7701	0.99995	no

ENSG00000167741	ENSG00000167741	GGT6	17:4460221-4464113	q1	q2	NOTEST	0	0	0	0	1	1	no

ENSG00000167747	ENSG00000167747	C19orf48	19:51300960-51308186	q1	q2	OK	53.7721	46.9995	-0.194214	-0.375076	0.5247	0.99995	no

ENSG00000167748	ENSG00000167748	KLK1	19:51322403-51327043	q1	q2	NOTEST	0.0704174	0	#NAME?	0	1	1	no

ENSG00000167749	ENSG00000167749	KLK4	19:51409607-51413994	q1	q2	NOTEST	0	0	0	0	1	1	no

ENSG00000167751	ENSG00000167751	KLK2	19:51364823-51383823	q1	q2	NOTEST	0.0089913	0	#NAME?	0	1	1	no

ENSG00000167754	ENSG00000167754	KLK5	19:51446558-51515385	q1	q2	NOTEST	0	0	0	0	1	1	no

ENSG00000167755	ENSG00000167755	KLK6	19:51446558-51515385	q1	q2	NOTEST	0.0600467	0.263121	2.13157	0	1	1	no

ENSG00000167757	ENSG00000167757	KLK11	19:51525471-51531295	q1	q2	NOTEST	0	0	0	0	1	1	no

ENSG00000167759	ENSG00000167759	KLK13	19:51559462-51568371	q1	q2	NOTEST	0	0	0	0	1	1	no

ENSG00000167765	ENSG00000167765	AC018755.1	19:52095035-52097630	q1	q2	OK	0.916635	0.780678	-0.23162	-0.23602	0.7475	0.99995	no

ENSG00000167766	ENSG00000167766	ZNF83	19:53091757-53193749	q1	q2	OK	28.033	28.4759	0.0226138	0.0341514	0.9565	0.99995	no

ENSG00000167767	ENSG00000167767	KRT80	12:52562779-52585784	q1	q2	NOTEST	0.0227173	0	#NAME?	0	1	1	no

ENSG00000167768	ENSG00000167768	KRT1	12:53068519-53074191	q1	q2	NOTEST	0	0	0	0	1	1	no

ENSG00000167769	ENSG00000167769	ACER1	19:6306152-6333640	q1	q2	NOTEST	0.0381215	0	#NAME?	0	1	1	no

ENSG00000167770	ENSG00000167770	OTUB1	11:63742078-63933578	q1	q2	OK	64.8004	62.9617	-0.0415271	-0.0661613	0.9231	0.99995	no

ENSG00000167771	ENSG00000167771	RCOR2	11:63678692-63684316	q1	q2	OK	42.3569	39.2634	-0.109409	-0.241952	0.7127	0.99995	no

ENSG00000167772	ENSG00000167772	ANGPTL4	19:8428172-8469318	q1	q2	OK	0.529641	0.485349	-0.125992	-0.0181773	0.9087	0.99995	no

ENSG00000167774	ENSG00000167774	NDUFA7	19:8367010-8408146	q1	q2	NOTEST	0	0	0	0	1	1	no

ENSG00000167775	ENSG00000167775	CD320	19:8367010-8408146	q1	q2	OK	28.9776	26.26	-0.142072	-0.122758	0.85085	0.99995	no

ENSG00000167778	ENSG00000167778	SPRYD3	12:53458387-53473204	q1	q2	OK	3.80173	3.40282	-0.159924	-0.229511	0.7236	0.99995	no

ENSG00000167779	ENSG00000167779	IGFBP6	12:53491219-53496129	q1	q2	OK	1.89471	1.90837	0.0103671	0.00762849	0.9903	0.99995	no

ENSG00000167780	ENSG00000167780	SOAT2	12:53497301-53518322	q1	q2	NOTEST	0	0	0	0	1	1	no

ENSG00000167785	ENSG00000167785	ZNF558	19:8920379-8943004	q1	q2	OK	3.11993	3.76064	0.269464	0.248716	0.71525	0.99995	no

ENSG00000167791	ENSG00000167791	CABP2	11:67286382-67290899	q1	q2	NOTEST	0.0425847	0	#NAME?	0	1	1	no

ENSG00000167792	ENSG00000167792	NDUFV1	11:67374322-67380006	q1	q2	OK	73.1279	65.6839	-0.154882	-0.313934	0.62695	0.99995	no

ENSG00000167797	ENSG00000167797	CDK2AP2	11:67273967-67276120	q1	q2	OK	25.1238	21.6645	-0.21372	-0.391957	0.58155	0.99995	no

ENSG00000167798	ENSG00000167798	C3P1	19:10148478-10184811	q1	q2	NOTEST	0	0	0	0	1	1	no

ENSG00000167799	ENSG00000167799	NUDT8	11:67395408-67397401	q1	q2	OK	5.03957	3.74363	-0.428864	-0.487482	0.5043	0.99995	no

ENSG00000167800	ENSG00000167800	TBX10	11:67398773-67407031	q1	q2	NOTEST	0	0	0	0	1	1	no

ENSG00000167807	ENSG00000167807	CTD-2369P2.10	19:10415478-10444316	q1	q2	NOTEST	0.132726	0	#NAME?	0	1	1	no

ENSG00000167815	ENSG00000167815	PRDX2	19:12873816-12985765	q1	q2	OK	249.592	232.489	-0.102411	-0.189378	0.77735	0.99995	no

ENSG00000167822	ENSG00000167822	OR8J3	11:55904246-55905194	q1	q2	NOTEST	0	0	0	0	1	1	no

ENSG00000167825	ENSG00000167825	OR5I1	11:55702931-55703876	q1	q2	NOTEST	0	0	0	0	1	1	no

ENSG00000167840	ENSG00000167840	ZNF232	17:5008835-5078329	q1	q2	OK	19.7779	17.3918	-0.185485	-0.299137	0.64395	0.99995	no

ENSG00000167842	ENSG00000167842	MIS12	17:5374570-5394134	q1	q2	OK	30.5256	29.7261	-0.0382892	-0.058045	0.92615	0.99995	no

ENSG00000167850	ENSG00000167850	CD300C	17:72537246-72542282	q1	q2	NOTEST	0	0	0	0	1	1	no

ENSG00000167851	ENSG00000167851	CD300A	17:72462554-72480933	q1	q2	NOTEST	0.0405978	0.014889	-1.44715	0	1	1	no

ENSG00000167858	ENSG00000167858	TEKT1	17:6679541-6735080	q1	q2	NOTEST	0.0482598	0.143713	1.57429	0	1	1	no

ENSG00000167861	ENSG00000167861	HID1	17:72946837-72971823	q1	q2	OK	5.51064	3.94847	-0.480926	-0.586728	0.28795	0.99995	no

ENSG00000167862	ENSG00000167862	ICT1	17:73008764-73017356	q1	q2	OK	24.3291	24.471	0.0083929	0.015185	0.9838	0.99995	no

ENSG00000167863	ENSG00000167863	ATP5H	17:73028669-73061984	q1	q2	OK	146.268	152.121	0.0566077	0.0927101	0.89705	0.99995	no

ENSG00000167874	ENSG00000167874	TMEM88	17:7758382-7759417	q1	q2	OK	5.69011	7.96048	0.484399	0.621271	0.366	0.99995	no

ENSG00000167880	ENSG00000167880	EVPL	17:73937587-74023533	q1	q2	NOTEST	0.00393966	0.00409987	0.057507	0	1	1	no

ENSG00000167881	ENSG00000167881	SRP68	17:74035183-74117657	q1	q2	OK	45.6635	44.8172	-0.0269904	-0.0322352	0.95915	0.99995	no

ENSG00000167889	ENSG00000167889	MGAT5B	17:74864537-74965238	q1	q2	OK	11.8072	11.6236	-0.0226152	-0.0395271	0.94945	0.99995	no

ENSG00000167895	ENSG00000167895	TMC8	17:76000248-76139049	q1	q2	NOTEST	0.189964	0.0445198	-2.0932	0	1	1	no

ENSG00000167900	ENSG00000167900	TK1	17:76170159-76183314	q1	q2	OK	32.5966	28.0357	-0.217456	-0.451319	0.50725	0.99995	no

ENSG00000167904	ENSG00000167904	TMEM68	8:56608982-56738007	q1	q2	OK	24.2925	25.6751	0.0798597	0.103604	0.85615	0.99995	no

ENSG00000167910	ENSG00000167910	CYP7A1	8:59402736-59412795	q1	q2	NOTEST	0	0.0250769	inf	0	1	1	no

ENSG00000167912	ENSG00000167912	RP11-25K19.1	8:59717976-60033905	q1	q2	OK	11.816	9.15908	-0.367462	-0.294267	0.67	0.99995	no

ENSG00000167914	ENSG00000167914	GSDMA	17:38119225-38134019	q1	q2	NOTEST	0	0.0117194	inf	0	1	1	no

ENSG00000167916	ENSG00000167916	KRT24	17:38854242-38860002	q1	q2	NOTEST	0	0	0	0	1	1	no

ENSG00000167920	ENSG00000167920	TMEM99	17:38974368-38992522	q1	q2	OK	11.2306	11.3202	0.011459	0.0120327	0.986	0.99995	no

ENSG00000167925	ENSG00000167925	GHDC	17:40340816-40346531	q1	q2	OK	1.78155	1.45481	-0.292303	-0.272953	0.5892	0.99995	no

ENSG00000167930	ENSG00000167930	ITFG3	16:284544-333003	q1	q2	OK	44.0082	34.97	-0.331656	-0.535877	0.309	0.99995	no

ENSG00000167941	ENSG00000167941	SOST	17:41831098-41836156	q1	q2	NOTEST	0.0464294	0.0688994	0.569454	0	1	1	no

ENSG00000167945	ENSG00000167945	PRR25	16:855442-863861	q1	q2	NOTEST	0.14764	0.0458194	-1.68805	0	1	1	no

ENSG00000167962	ENSG00000167962	ZNF598	16:2047654-2059824	q1	q2	OK	16.9399	14.7113	-0.203504	-0.332248	0.59735	0.99995	no

ENSG00000167964	ENSG00000167964	RAB26	16:2190803-2204166	q1	q2	OK	0.607472	0.80505	0.40626	0.324202	0.5947	0.99995	no

ENSG00000167965	ENSG00000167965	MLST8	16:2254248-2261951	q1	q2	OK	30.4111	25.4505	-0.256906	-0.439546	0.45915	0.99995	no

ENSG00000167967	ENSG00000167967	E4F1	16:2273566-2288712	q1	q2	OK	13.9763	12.4163	-0.170745	-0.24043	0.6389	0.99995	no

ENSG00000167968	ENSG00000167968	DNASE1L2	16:2273566-2288712	q1	q2	OK	1.27457	0.79538	-0.680299	-0.276425	0.6902	0.99995	no

ENSG00000167969	ENSG00000167969	ECI1	16:2289395-2302301	q1	q2	OK	31.9924	30.0552	-0.0901127	-0.170464	0.8057	0.99995	no

ENSG00000167970	ENSG00000167970	AC009065.1	16:2303116-2323419	q1	q2	NOTEST	0	0	0	0	1	1	no

ENSG00000167971	ENSG00000167971	CASKIN1	16:2205698-2246526	q1	q2	OK	3.79085	3.5391	-0.0991377	-0.046542	0.88385	0.99995	no

ENSG00000167972	ENSG00000167972	ABCA3	16:2325881-2476700	q1	q2	OK	8.27537	6.46232	-0.356772	-0.606669	0.27915	0.99995	no

ENSG00000167977	ENSG00000167977	KCTD5	16:2732475-2759031	q1	q2	OK	18.6717	16.9407	-0.140359	-0.305657	0.65905	0.99995	no

ENSG00000167978	ENSG00000167978	SRRM2	16:2787076-2827298	q1	q2	OK	275.554	246.021	-0.163549	-0.149307	0.7206	0.99995	no

ENSG00000167981	ENSG00000167981	ZNF597	16:3415098-3627401	q1	q2	OK	1.51089	1.77881	0.235515	0.0832971	0.86515	0.99995	no

ENSG00000167984	ENSG00000167984	NLRC3	16:3415098-3627401	q1	q2	NOTEST	0.329808	0.288789	-0.191611	0	1	1	no

ENSG00000167985	ENSG00000167985	SDHAF2	11:61170120-61258403	q1	q2	OK	22.5343	23.282	0.0470945	0.0310553	0.96155	0.99995	no

ENSG00000167986	ENSG00000167986	DDB1	11:61066922-61136981	q1	q2	OK	86.9172	85.8307	-0.0181476	-0.0328011	0.95135	0.99995	no

ENSG00000167987	ENSG00000167987	VPS37C	11:60897727-60929089	q1	q2	OK	11.2758	11.9399	0.0825563	0.148222	0.80755	0.99995	no

ENSG00000167992	ENSG00000167992	VWCE	11:61025761-61062896	q1	q2	OK	11.2494	10.2686	-0.131603	-0.177547	0.7741	0.99995	no

ENSG00000167994	ENSG00000167994	RAB3IL1	11:61664772-61687741	q1	q2	OK	3.3893	2.44524	-0.471013	-0.39166	0.45565	0.99995	no

ENSG00000167995	ENSG00000167995	BEST1	11:61717292-61735132	q1	q2	OK	0.745994	0.535417	-0.478501	-0.0498396	0.66265	0.99995	no

ENSG00000167996	ENSG00000167996	FTH1	11:61717292-61735132	q1	q2	OK	786.97	701.494	-0.165878	-0.351175	0.61505	0.99995	no

ENSG00000168000	ENSG00000168000	BSCL2	11:62457746-62494856	q1	q2	OK	34.5098	33.5294	-0.0415802	-0.0265333	0.95385	0.99995	no

ENSG00000168002	ENSG00000168002	POLR2G	11:62529015-62534182	q1	q2	OK	108.726	103.137	-0.0761351	-0.176636	0.79975	0.99995	no

ENSG00000168003	ENSG00000168003	SLC3A2	11:62623517-62656352	q1	q2	OK	73.4847	70.3546	-0.0627994	-0.127539	0.82845	0.99995	no

ENSG00000168004	ENSG00000168004	HRASLS5	11:63228875-63258666	q1	q2	NOTEST	0.0411763	0.0191726	-1.10277	0	1	1	no

ENSG00000168005	ENSG00000168005	C11orf84	11:63580859-63595190	q1	q2	OK	42.0329	40.9165	-0.0388348	-0.0819392	0.89685	0.99995	no

ENSG00000168010	ENSG00000168010	ATG16L2	11:72525352-72853306	q1	q2	OK	9.55219	7.92782	-0.268907	-0.278051	0.57685	0.99995	no

ENSG00000168014	ENSG00000168014	C2CD3	11:73723762-74035750	q1	q2	OK	14.9349	13.4008	-0.156368	-0.164212	0.73285	0.99995	no

ENSG00000168016	ENSG00000168016	TRANK1	3:36868310-36986548	q1	q2	OK	0.886838	0.782516	-0.180551	-0.10687	0.77365	0.99995	no

ENSG00000168026	ENSG00000168026	TTC21A	3:39093488-39180394	q1	q2	OK	3.56582	2.42107	-0.558593	-0.139843	0.44505	0.99995	no

ENSG00000168028	ENSG00000168028	RPSA	3:39448179-39454033	q1	q2	OK	827.697	787.979	-0.0709458	-0.152453	0.823	0.99995	no

ENSG00000168032	ENSG00000168032	ENTPD3	3:40355292-40494820	q1	q2	NOTEST	0.0372247	0.0597277	0.682141	0	1	1	no

ENSG00000168036	ENSG00000168036	CTNNB1	3:41236327-42003922	q1	q2	OK	243.963	244.822	0.00506725	0.0105207	0.98735	0.99995	no

ENSG00000168038	ENSG00000168038	ULK4	3:41236327-42003922	q1	q2	OK	2.89311	2.75498	-0.0705801	-0.00845737	0.9452	0.99995	no

ENSG00000168040	ENSG00000168040	FADD	11:70049268-70053496	q1	q2	OK	20.8789	18.8905	-0.144384	-0.30184	0.6717	0.99995	no

ENSG00000168056	ENSG00000168056	LTBP3	11:65306275-65326401	q1	q2	OK	19.0241	16.2616	-0.22636	-0.334937	0.5404	0.99995	no

ENSG00000168060	ENSG00000168060	NAALADL1	11:64808372-64826021	q1	q2	NOTEST	0.268073	0.269527	0.00780168	0	1	1	no

ENSG00000168061	ENSG00000168061	SAC3D1	11:64808372-64826021	q1	q2	OK	13.0273	10.8208	-0.267729	-0.453603	0.5242	0.99995	no

ENSG00000168062	ENSG00000168062	BATF2	11:64755414-64764517	q1	q2	NOTEST	0.051451	0.0474115	-0.11796	0	1	1	no

ENSG00000168065	ENSG00000168065	SLC22A11	11:64323097-64340347	q1	q2	NOTEST	0.0461866	0.0700215	0.600323	0	1	1	no

ENSG00000168066	ENSG00000168066	SF1	11:64532077-64546258	q1	q2	OK	197.991	215.619	0.123054	0.178987	0.79045	0.99995	no

ENSG00000168067	ENSG00000168067	MAP4K2	11:64556289-64570713	q1	q2	OK	5.85784	4.56247	-0.360552	-0.454582	0.4193	0.99995	no

ENSG00000168070	ENSG00000168070	C11orf85	11:64704988-64739557	q1	q2	NOTEST	0.0128547	0.0676669	2.39615	0	1	1	no

ENSG00000168071	ENSG00000168071	CCDC88B	11:64107694-64125006	q1	q2	NOTEST	0.239874	0.476698	0.990802	0	1	1	no

ENSG00000168077	ENSG00000168077	SCARA3	8:27491384-27534293	q1	q2	OK	17.116	14.7166	-0.217904	-0.479828	0.4965	0.99995	no

ENSG00000168078	ENSG00000168078	PBK	8:27590834-27695612	q1	q2	OK	39.5964	43.7874	0.145146	0.229875	0.7444	0.99995	no

ENSG00000168079	ENSG00000168079	SCARA5	8:27727398-27850244	q1	q2	NOTEST	0.0955049	0.0421654	-1.17952	0	1	1	no

ENSG00000168081	ENSG00000168081	PNOC	8:28107579-28200872	q1	q2	NOTEST	0	0.0690902	inf	0	1	1	no

ENSG00000168090	ENSG00000168090	COPS6	7:99686576-99689823	q1	q2	OK	83.9066	80.3478	-0.0625256	-0.141093	0.84515	0.99995	no

ENSG00000168092	ENSG00000168092	PAFAH1B2	11:117014982-117047610	q1	q2	OK	34.3314	38.2297	0.155163	0.351987	0.578	0.99995	no

ENSG00000168096	ENSG00000168096	ANKS3	16:4746512-4817625	q1	q2	OK	13.1872	11.798	-0.160597	-0.203642	0.7205	0.99995	no

ENSG00000168101	ENSG00000168101	NUDT16L1	16:4743694-4745860	q1	q2	OK	21.7976	20.2414	-0.106867	-0.208478	0.76535	0.99995	no

ENSG00000168116	ENSG00000168116	KIAA1586	6:56911346-56920023	q1	q2	OK	14.2578	16.449	0.206243	0.409218	0.53695	0.99995	no

ENSG00000168118	ENSG00000168118	RAB4A	1:229394027-229441641	q1	q2	OK	25.5546	26.2966	0.0412946	0.0619382	0.92165	0.99995	no

ENSG00000168122	ENSG00000168122	ZNF355P	21:14467625-14486111	q1	q2	NOTEST	0	0.0152391	inf	0	1	1	no

ENSG00000168124	ENSG00000168124	OR1F1	16:3238211-3286221	q1	q2	NOTEST	0.131534	0.0662889	-0.988597	0	1	1	no

ENSG00000168126	ENSG00000168126	OR2W6P	6:27905242-27906181	q1	q2	NOTEST	0.0642284	0.0313814	-1.0333	0	1	1	no

ENSG00000168129	ENSG00000168129	AC098817.5	2:83083926-83084885	q1	q2	NOTEST	0	0	0	0	1	1	no

ENSG00000168131	ENSG00000168131	OR2B2	6:27878962-27880174	q1	q2	NOTEST	0	0	0	0	1	1	no

ENSG00000168135	ENSG00000168135	KCNJ4	22:38820292-38851205	q1	q2	OK	3.32901	2.44838	-0.443265	-0.612413	0.36375	0.99995	no

ENSG00000168137	ENSG00000168137	SETD5	3:9022274-9520924	q1	q2	OK	78.8076	78.9563	0.00271991	0.00225601	0.99455	0.99995	no

ENSG00000168140	ENSG00000168140	VASN	16:4364761-4475706	q1	q2	OK	1.23624	0.969845	-0.350138	-0.184762	0.8154	0.99995	no

ENSG00000168143	ENSG00000168143	FAM83B	6:54711568-54806820	q1	q2	NOTEST	0	0	0	0	1	1	no

ENSG00000168148	ENSG00000168148	HIST3H3	1:228612545-228613026	q1	q2	NOTEST	0.0893829	0.0866954	-0.044042	0	1	1	no

ENSG00000168152	ENSG00000168152	THAP9	4:83739813-83934079	q1	q2	OK	6.46072	6.2724	-0.0426758	-0.0179781	0.9677	0.99995	no

ENSG00000168158	ENSG00000168158	OR2C1	16:3405888-3406924	q1	q2	NOTEST	0	0.0571343	inf	0	1	1	no

ENSG00000168159	ENSG00000168159	RNF187	1:228674761-228683467	q1	q2	OK	154.565	124.127	-0.316401	-0.524653	0.44935	0.99995	no

ENSG00000168172	ENSG00000168172	HOOK3	8:42704779-42940931	q1	q2	OK	14.1452	16.1307	0.189501	0.150967	0.66095	0.99995	no

ENSG00000168175	ENSG00000168175	MAPK1IP1L	14:55518348-55536910	q1	q2	OK	49.0553	49.358	0.00887522	0.0100281	0.9856	0.99995	no

ENSG00000168209	ENSG00000168209	DDIT4	10:74033677-74035794	q1	q2	OK	44.5006	38.3867	-0.213215	-0.483274	0.4731	0.99995	no

ENSG00000168214	ENSG00000168214	RBPJ	4:26165076-26436541	q1	q2	OK	42.2232	42.9796	0.0256153	0.0401175	0.9251	0.99995	no

ENSG00000168216	ENSG00000168216	LMBRD1	6:70385693-70507003	q1	q2	OK	14.5373	15.3657	0.0799583	0.161716	0.81155	0.99995	no

ENSG00000168228	ENSG00000168228	ZCCHC4	4:25314406-25372005	q1	q2	OK	6.07372	6.53241	0.105037	0.163301	0.8025	0.99995	no

ENSG00000168229	ENSG00000168229	PTGDR	14:52734430-52743442	q1	q2	NOTEST	0.00865398	0	#NAME?	0	1	1	no

ENSG00000168234	ENSG00000168234	TTC39C	18:21572736-21715574	q1	q2	OK	8.87558	9.05386	0.0286917	0.0339839	0.9504	0.99995	no

ENSG00000168237	ENSG00000168237	GLYCTK	3:52288436-52333083	q1	q2	OK	11.6692	10.5814	-0.141175	-0.0810335	0.904	0.99995	no

ENSG00000168242	ENSG00000168242	HIST1H2BI	6:26273143-26273622	q1	q2	NOTEST	0	0.0873028	inf	0	1	1	no

ENSG00000168243	ENSG00000168243	GNG4	1:235710986-235814054	q1	q2	OK	27.2642	26.6668	-0.0319635	-0.0635628	0.92895	0.99995	no

ENSG00000168246	ENSG00000168246	UBTD2	5:171636643-171711075	q1	q2	OK	59.1907	62.4771	0.0779555	0.194259	0.77715	0.99995	no

ENSG00000168255	ENSG00000168255	POLR2J3	7:102122891-102312088	q1	q2	OK	11.8655	12.2449	0.0454133	0.051229	0.93825	0.99995	no

ENSG00000168256	ENSG00000168256	NKIRAS2	17:40118758-40217383	q1	q2	OK	0.826316	0.765836	-0.109658	-0.0431534	0.91095	0.99995	no

ENSG00000168259	ENSG00000168259	DNAJC7	17:40118758-40217383	q1	q2	OK	9.8451	2.69319	-1.87009	-1.98996	0.00065	0.437275	no

ENSG00000168260	ENSG00000168260	C14orf183	14:50448429-50559361	q1	q2	NOTEST	0	0	0	0	1	1	no

ENSG00000168263	ENSG00000168263	KCNV2	9:2717501-2844241	q1	q2	NOTEST	0.157938	0.288932	0.871368	0	1	1	no

ENSG00000168264	ENSG00000168264	IRF2BP2	1:234740014-234745271	q1	q2	OK	30.8322	32.1331	0.0596199	0.0922199	0.86945	0.99995	no

ENSG00000168267	ENSG00000168267	PTF1A	10:23481255-23483181	q1	q2	NOTEST	0	0.0172519	inf	0	1	1	no

ENSG00000168268	ENSG00000168268	NT5DC2	3:52529353-52742182	q1	q2	OK	152.539	135.69	-0.168856	-0.171458	0.74155	0.99995	no

ENSG00000168269	ENSG00000168269	FOXI1	5:169532900-169536727	q1	q2	NOTEST	0	0	0	0	1	1	no

ENSG00000168273	ENSG00000168273	SMIM4	3:52529353-52742182	q1	q2	OK	19.2688	16.7871	-0.198916	-0.025914	0.8517	0.99995	no

ENSG00000168274	ENSG00000168274	HIST1H2AE	6:26217164-26217711	q1	q2	OK	1.4078	1.86339	0.40448	0.378965	0.6003	0.99995	no

ENSG00000168275	ENSG00000168275	COA6	1:234507931-234519795	q1	q2	OK	25.1893	24.1533	-0.0605892	-0.101552	0.884	0.99995	no

ENSG00000168280	ENSG00000168280	KIF5C	2:149624038-149883273	q1	q2	OK	26.6257	30.9053	0.215035	0.52127	0.45925	0.99995	no

ENSG00000168282	ENSG00000168282	MGAT2	14:50087488-50090198	q1	q2	OK	20.207	20.5593	0.0249318	0.0567441	0.93635	0.99995	no

ENSG00000168283	ENSG00000168283	BMI1	10:22604902-22620413	q1	q2	OK	25.6548	25.4425	-0.011985	-0.0155166	0.97115	0.99995	no

ENSG00000168286	ENSG00000168286	THAP11	16:67840667-67906470	q1	q2	OK	24.2915	23.488	-0.0485312	-0.0515322	0.94105	0.99995	no

ENSG00000168288	ENSG00000168288	MMADHC	2:150426147-150715705	q1	q2	OK	74.946	79.6535	0.0878855	0.212701	0.76	0.99995	no

ENSG00000168291	ENSG00000168291	PDHB	3:58413356-58419584	q1	q2	OK	46.1874	48.0323	0.0565044	0.128949	0.857	0.99995	no

ENSG00000168297	ENSG00000168297	PXK	3:58318606-58411748	q1	q2	OK	7.53202	8.25828	0.132804	0.226244	0.74115	0.99995	no

ENSG00000168298	ENSG00000168298	HIST1H1E	6:26156558-26157343	q1	q2	NOTEST	0.128637	0.119308	-0.108615	0	1	1	no

ENSG00000168300	ENSG00000168300	PCMTD1	8:52730139-52811735	q1	q2	OK	23.5755	23.8707	0.0179579	0.0315556	0.9647	0.99995	no

ENSG00000168301	ENSG00000168301	KCTD6	3:58477840-58488087	q1	q2	OK	13.5239	13.6743	0.0159546	0.0275876	0.96785	0.99995	no

ENSG00000168303	ENSG00000168303	MPLKIP	7:40165621-40174258	q1	q2	OK	4.05038	3.98422	-0.0237596	-0.0485968	0.9458	0.99995	no

ENSG00000168306	ENSG00000168306	ACOX2	3:58490862-58523046	q1	q2	OK	0.948123	0.868282	-0.12691	-0.0764644	0.8758	0.99995	no

ENSG00000168309	ENSG00000168309	FAM107A	3:58549843-58652575	q1	q2	OK	0.77401	0.391435	-0.983577	-0.83782	0.12245	0.99995	no

ENSG00000168310	ENSG00000168310	IRF2	4:185308866-185395734	q1	q2	OK	8.50801	8.52001	0.00203422	0.00284468	0.9955	0.99995	no

ENSG00000168314	ENSG00000168314	MOBP	3:39508688-39570970	q1	q2	NOTEST	0.0214298	0.00865515	-1.30799	0	1	1	no

ENSG00000168329	ENSG00000168329	CX3CR1	3:39304984-39323226	q1	q2	NOTEST	0.0253702	0.0163904	-0.630287	0	1	1	no

ENSG00000168333	ENSG00000168333	C8orf22	8:49966869-49988649	q1	q2	NOTEST	0	0	0	0	1	1	no

ENSG00000168334	ENSG00000168334	XIRP1	3:39224700-39234087	q1	q2	NOTEST	0	0	0	0	1	1	no

ENSG00000168348	ENSG00000168348	INSM2	14:36003247-36006260	q1	q2	NOTEST	0.331065	0.374568	0.178113	0	1	1	no

ENSG00000168350	ENSG00000168350	DEGS2	14:100612755-100626500	q1	q2	NOTEST	0.188323	0.220122	0.225096	0	1	1	no

ENSG00000168356	ENSG00000168356	SCN11A	3:38887259-38992052	q1	q2	OK	0.508501	0.306893	-0.728514	-0.839229	0.2509	0.99995	no

ENSG00000168367	ENSG00000168367	LINC00917	16:86365449-86383289	q1	q2	NOTEST	0	0	0	0	1	1	no

ENSG00000168374	ENSG00000168374	ARF4	3:57557089-57586654	q1	q2	OK	141.462	149.681	0.0814782	0.199671	0.7712	0.99995	no

ENSG00000168385	ENSG00000168385	Sep-02	2:242166678-242293442	q1	q2	OK	320.052	341.319	0.0928145	0.107236	0.8108	0.99995	no

ENSG00000168386	ENSG00000168386	FILIP1L	3:99536677-99897447	q1	q2	OK	6.82722	8.54067	0.323051	0.364831	0.547	0.99995	no

ENSG00000168389	ENSG00000168389	MFSD2A	1:40420801-40435638	q1	q2	OK	18.1221	17.7064	-0.0334817	-0.0618608	0.92435	0.99995	no

ENSG00000168393	ENSG00000168393	DTYMK	2:242615156-242626406	q1	q2	OK	53.9763	51.7645	-0.060364	-0.133372	0.84655	0.99995	no

ENSG00000168394	ENSG00000168394	TAP1	6:32808493-32827362	q1	q2	NOTEST	0	0	0	0	1	1	no

ENSG00000168395	ENSG00000168395	ING5	2:242641449-242668893	q1	q2	OK	17.5969	16.7039	-0.0751401	-0.104527	0.82605	0.99995	no

ENSG00000168397	ENSG00000168397	ATG4B	2:242523819-242613272	q1	q2	OK	51.684	48.567	-0.0897417	-0.125801	0.8045	0.99995	no

ENSG00000168398	ENSG00000168398	BDKRB2	14:96671015-96735304	q1	q2	NOTEST	0.045921	0.0491862	0.0990994	0	1	1	no

ENSG00000168404	ENSG00000168404	MLKL	16:74705752-74734858	q1	q2	NOTEST	0.0744637	0.121514	0.706519	0	1	1	no

ENSG00000168405	ENSG00000168405	CMAHP	6:25081547-25166793	q1	q2	NOTEST	0	0	0	0	1	1	no

ENSG00000168411	ENSG00000168411	RFWD3	16:74655291-74700779	q1	q2	OK	18.8619	22.2173	0.236211	0.434266	0.39725	0.99995	no

ENSG00000168412	ENSG00000168412	MTNR1A	4:187187098-187476721	q1	q2	NOTEST	0	0.0226167	inf	0	1	1	no

ENSG00000168418	ENSG00000168418	KCNG4	16:84255822-84273356	q1	q2	NOTEST	0	0	0	0	1	1	no

ENSG00000168421	ENSG00000168421	RHOH	4:40192672-40248587	q1	q2	OK	0.603974	0.438774	-0.461009	-0.210105	0.45095	0.99995	no

ENSG00000168427	ENSG00000168427	KLHL30	2:239047362-239061588	q1	q2	NOTEST	0	0.00707611	inf	0	1	1	no

ENSG00000168434	ENSG00000168434	COG7	16:23399813-23468927	q1	q2	OK	13.4986	11.1627	-0.274112	-0.375391	0.5372	0.99995	no

ENSG00000168438	ENSG00000168438	CDC40	6:110501343-110679475	q1	q2	OK	8.95225	8.89024	-0.0100272	-0.0202566	0.97285	0.99995	no

ENSG00000168439	ENSG00000168439	STIP1	11:63952743-63972015	q1	q2	OK	135.918	141.03	0.053269	0.116839	0.84955	0.99995	no

ENSG00000168447	ENSG00000168447	SCNN1B	16:23289551-23392620	q1	q2	NOTEST	0	0	0	0	1	1	no

ENSG00000168453	ENSG00000168453	HR	8:21971927-21990897	q1	q2	NOTEST	0.0925129	0.272296	1.55745	0	1	1	no

ENSG00000168454	ENSG00000168454	TXNDC2	18:9885762-9889272	q1	q2	NOTEST	0.174954	0.178281	0.0271789	0	1	1	no

ENSG00000168461	ENSG00000168461	RAB31	18:9708161-9862548	q1	q2	OK	49.4511	53.7797	0.121061	0.273559	0.6556	0.99995	no

ENSG00000168476	ENSG00000168476	REEP4	8:21995532-21999464	q1	q2	OK	17.2468	16.2269	-0.0879453	-0.167886	0.8038	0.99995	no

ENSG00000168477	ENSG00000168477	TNXB	6:32006041-32096030	q1	q2	NOTEST	0.00376915	0	#NAME?	0	1	1	no

ENSG00000168481	ENSG00000168481	LGI3	8:22004337-22021992	q1	q2	NOTEST	0.0167243	0.0242402	0.535454	0	1	1	no

ENSG00000168484	ENSG00000168484	SFTPC	8:22004337-22021992	q1	q2	NOTEST	0.0313259	0	#NAME?	0	1	1	no

ENSG00000168487	ENSG00000168487	BMP1	8:22022248-22069839	q1	q2	OK	19.7058	17.3701	-0.182021	-0.320473	0.60385	0.99995	no

ENSG00000168488	ENSG00000168488	ATXN2L	16:28834355-28848558	q1	q2	OK	82.8126	77.6446	-0.0929646	-0.144951	0.7754	0.99995	no

ENSG00000168490	ENSG00000168490	PHYHIP	8:22077221-22089854	q1	q2	NOTEST	0.39243	0.319289	-0.297573	0	1	1	no

ENSG00000168491	ENSG00000168491	CCDC110	4:186347393-186393417	q1	q2	NOTEST	0.305542	0.122072	-1.32363	0	1	1	no

ENSG00000168495	ENSG00000168495	POLR3D	8:22102616-22132675	q1	q2	OK	17.3912	16.5237	-0.0738172	-0.129838	0.8485	0.99995	no

ENSG00000168496	ENSG00000168496	FEN1	11:61447904-61634826	q1	q2	OK	63.299	60.9982	-0.0534153	-0.026956	0.9683	0.99995	no

ENSG00000168497	ENSG00000168497	SDPR	2:192699027-193060435	q1	q2	OK	0.595607	0.443933	-0.424019	-0.258261	0.7251	0.99995	no

ENSG00000168502	ENSG00000168502	SOGA2	18:8695853-8832776	q1	q2	OK	15.2009	16.9807	0.159733	0.261615	0.6139	0.99995	no

ENSG00000168505	ENSG00000168505	GBX2	2:237073878-237203570	q1	q2	OK	37.6493	46.1848	0.294793	0.610386	0.3707	0.99995	no

ENSG00000168509	ENSG00000168509	HFE2	1:145413094-145417545	q1	q2	NOTEST	0	0	0	0	1	1	no

ENSG00000168515	ENSG00000168515	SCGB1D1	11:61957687-61961011	q1	q2	NOTEST	0	0	0	0	1	1	no

ENSG00000168517	ENSG00000168517	HEXIM2	17:43224683-43248951	q1	q2	OK	6.07299	4.68023	-0.375829	-0.212275	0.7321	0.99995	no

ENSG00000168522	ENSG00000168522	FNTA	8:42704779-42940931	q1	q2	OK	45.2909	47.4835	0.0682048	0.0772212	0.90585	0.99995	no

ENSG00000168528	ENSG00000168528	SERINC2	1:31882411-31907525	q1	q2	NOTEST	0	0	0	0	1	1	no

ENSG00000168530	ENSG00000168530	MYL1	2:211154873-211179914	q1	q2	NOTEST	0.116141	0.0503553	-1.20566	0	1	1	no

ENSG00000168538	ENSG00000168538	TRAPPC11	4:184580419-184634745	q1	q2	OK	17.0939	18.9144	0.145999	0.271595	0.6349	0.99995	no

ENSG00000168539	ENSG00000168539	CHRM1	11:62676150-62689279	q1	q2	NOTEST	0	0.0083165	inf	0	1	1	no

ENSG00000168542	ENSG00000168542	COL3A1	2:189839045-189877472	q1	q2	OK	219.502	203.763	-0.107336	-0.153764	0.82385	0.99995	no

ENSG00000168546	ENSG00000168546	GFRA2	8:21547914-21669869	q1	q2	OK	1.93984	2.18978	0.174848	0.203807	0.7536	0.99995	no

ENSG00000168556	ENSG00000168556	ING2	4:184426146-184432249	q1	q2	OK	18.1301	20.2339	0.158386	0.268639	0.69475	0.99995	no

ENSG00000168564	ENSG00000168564	CDKN2AIP	4:184365743-184369351	q1	q2	OK	18.8684	19.4234	0.0418218	0.0884226	0.89235	0.99995	no

ENSG00000168566	ENSG00000168566	SNRNP48	6:7590431-7612200	q1	q2	OK	13.8567	14.9968	0.114071	0.255111	0.7127	0.99995	no

ENSG00000168569	ENSG00000168569	TMEM223	11:62538591-62559493	q1	q2	OK	11.6486	10.6005	-0.136017	-0.116103	0.8603	0.99995	no

ENSG00000168575	ENSG00000168575	SLC20A2	8:42273992-42409603	q1	q2	OK	17.5131	19.7623	0.174323	0.212724	0.6825	0.99995	no

ENSG00000168582	ENSG00000168582	CRYGA	2:209025463-209028300	q1	q2	NOTEST	0	0	0	0	1	1	no

ENSG00000168589	ENSG00000168589	DYNLRB2	16:80099732-80597032	q1	q2	OK	0.62677	0.641415	0.0333221	0.0163881	0.97585	0.99995	no

ENSG00000168591	ENSG00000168591	TMUB2	17:42264337-42269099	q1	q2	OK	18.885	17.0149	-0.150448	-0.293863	0.6824	0.99995	no

ENSG00000168594	ENSG00000168594	ADAM29	4:175750818-175900532	q1	q2	NOTEST	0.158046	0.0947927	-0.737494	0	1	1	no

ENSG00000168610	ENSG00000168610	STAT3	17:40465341-40540586	q1	q2	OK	25.2644	25.844	0.0327213	0.0556043	0.92065	0.99995	no

ENSG00000168612	ENSG00000168612	ZSWIM1	20:44509865-44513905	q1	q2	OK	5.76516	5.82907	0.015906	0.0262477	0.96945	0.99995	no

ENSG00000168614	ENSG00000168614	NBPF9	1:144811743-144830413	q1	q2	OK	2.02761	2.72832	0.428234	0.541687	0.3572	0.99995	no

ENSG00000168615	ENSG00000168615	ADAM9	8:38854387-38962663	q1	q2	OK	27.2727	26.1126	-0.0627151	-0.136183	0.8275	0.99995	no

ENSG00000168619	ENSG00000168619	ADAM18	8:39442007-39587583	q1	q2	NOTEST	0	0	0	0	1	1	no

ENSG00000168621	ENSG00000168621	GDNF	5:37812778-37839788	q1	q2	NOTEST	0.105923	0.101663	-0.0592251	0	1	1	no

ENSG00000168630	ENSG00000168630	SPINT5P	20:44376582-44420571	q1	q2	NOTEST	0	0	0	0	1	1	no

ENSG00000168631	ENSG00000168631	DPCR1	6:30908748-30922639	q1	q2	NOTEST	0	0	0	0	1	1	no

ENSG00000168634	ENSG00000168634	WFDC13	20:44313291-44337456	q1	q2	NOTEST	0	0	0	0	1	1	no

ENSG00000168646	ENSG00000168646	AXIN2	17:63524680-64188202	q1	q2	OK	8.68206	8.44549	-0.0398553	-0.0581992	0.91915	0.99995	no

ENSG00000168653	ENSG00000168653	NDUFS5	1:39491989-39500308	q1	q2	OK	315.635	321.086	0.0247014	0.0598977	0.9294	0.99995	no

ENSG00000168658	ENSG00000168658	VWA3B	2:98703578-98929762	q1	q2	NOTEST	0.14162	0.112083	-0.337455	0	1	1	no

ENSG00000168661	ENSG00000168661	ZNF30	19:35417806-35436074	q1	q2	OK	4.83488	4.17442	-0.211907	-0.305406	0.6341	0.99995	no

ENSG00000168671	ENSG00000168671	UGT3A2	5:36035118-36071460	q1	q2	NOTEST	0.0526551	0.0504669	-0.061237	0	1	1	no

ENSG00000168672	ENSG00000168672	FAM84B	8:127564686-127725660	q1	q2	OK	4.28465	3.59524	-0.253087	-0.255104	0.69945	0.99995	no

ENSG00000168675	ENSG00000168675	LDLRAD4	18:13217496-13652754	q1	q2	OK	2.20074	1.89134	-0.218579	-0.166086	0.6202	0.99995	no

ENSG00000168676	ENSG00000168676	KCTD19	16:67311412-67360666	q1	q2	NOTEST	0.0757609	0.0552502	-0.455476	0	1	1	no

ENSG00000168679	ENSG00000168679	SLC16A4	1:110905469-110974982	q1	q2	OK	1.99967	1.97434	-0.0183872	-0.00923827	0.9898	0.99995	no

ENSG00000168685	ENSG00000168685	IL7R	5:35852796-35879705	q1	q2	NOTEST	0.378408	0.18956	-0.997287	0	1	1	no

ENSG00000168701	ENSG00000168701	TMEM208	16:67261005-67263181	q1	q2	OK	22.422	23.2966	0.055205	0.0946225	0.89735	0.99995	no

ENSG00000168702	ENSG00000168702	LRP1B	2:140988991-142895399	q1	q2	OK	1.07466	1.22144	0.184693	0.207819	0.7487	0.99995	no

ENSG00000168703	ENSG00000168703	WFDC12	20:43752065-43753106	q1	q2	NOTEST	0	0	0	0	1	1	no

ENSG00000168710	ENSG00000168710	AHCYL1	1:110527307-110566357	q1	q2	OK	40.913	42.4931	0.0546695	0.122562	0.83665	0.99995	no

ENSG00000168724	ENSG00000168724	DNAJC21	5:34929697-34959069	q1	q2	OK	27.5192	30.9144	0.167841	0.311651	0.6142	0.99995	no

ENSG00000168734	ENSG00000168734	PKIG	20:43160425-43280874	q1	q2	OK	34.0304	32.8588	-0.0505455	-0.0701317	0.91025	0.99995	no

ENSG00000168743	ENSG00000168743	NPNT	4:106473776-106943635	q1	q2	OK	1.00215	0.720264	-0.4765	-0.054673	0.6973	0.99995	no

ENSG00000168746	ENSG00000168746	C20orf62	20:43080623-43093984	q1	q2	NOTEST	0	0	0	0	1	1	no

ENSG00000168748	ENSG00000168748	CA7	16:66836777-66907159	q1	q2	NOTEST	0	0	0	0	1	1	no

ENSG00000168754	ENSG00000168754	FAM178B	2:97541619-97684341	q1	q2	NOTEST	0.0312867	0.0583398	0.898931	0	1	1	no

ENSG00000168757	ENSG00000168757	TSPY2	Y:6114263-6117060	q1	q2	NOTEST	0	0	0	0	1	1	no

ENSG00000168758	ENSG00000168758	SEMA4C	2:97525452-97536494	q1	q2	OK	32.026	30.4621	-0.0722282	-0.14958	0.8098	0.99995	no

ENSG00000168763	ENSG00000168763	CNNM3	2:97481981-97523832	q1	q2	OK	11.8028	9.74624	-0.276207	-0.339117	0.4904	0.99995	no

ENSG00000168765	ENSG00000168765	GSTM4	1:110198702-110208118	q1	q2	OK	9.55412	7.95324	-0.264581	-0.408069	0.5397	0.99995	no

ENSG00000168769	ENSG00000168769	TET2	4:106067031-106200973	q1	q2	OK	9.29358	9.53687	0.037282	0.0845437	0.894	0.99995	no

ENSG00000168772	ENSG00000168772	CXXC4	4:105389468-106041157	q1	q2	OK	24.7312	21.2956	-0.215779	-0.324252	0.4775	0.99995	no

ENSG00000168778	ENSG00000168778	TCTN2	12:124155659-124192948	q1	q2	OK	10.7145	9.65882	-0.149645	-0.290751	0.6695	0.99995	no

ENSG00000168779	ENSG00000168779	SHOX2	3:157814947-158288855	q1	q2	OK	0.504786	0.521192	0.0461416	0.0173447	0.97685	0.99995	no

ENSG00000168781	ENSG00000168781	PPIP5K1	15:43825659-43882430	q1	q2	OK	2.58171	2.89329	0.164388	0.163418	0.6582	0.99995	no

ENSG00000168785	ENSG00000168785	TSPAN5	4:99391517-99579780	q1	q2	OK	17.8581	17.6074	-0.0203987	-0.0403116	0.9488	0.99995	no

ENSG00000168787	ENSG00000168787	OR12D2	6:29323006-29424848	q1	q2	NOTEST	0	0	0	0	1	1	no

ENSG00000168792	ENSG00000168792	ABHD15	17:27887564-28513493	q1	q2	OK	3.15805	2.31608	-0.44735	-0.194303	0.48595	0.99995	no

ENSG00000168795	ENSG00000168795	ZBTB5	9:37438110-37465396	q1	q2	OK	16.499	16.3292	-0.0149182	-0.0354923	0.9564	0.99995	no

ENSG00000168802	ENSG00000168802	CHTF8	16:69139466-69442474	q1	q2	OK	72.5672	65.9119	-0.138779	-0.127139	0.76495	0.99995	no

ENSG00000168803	ENSG00000168803	ADAL	15:43622871-43646096	q1	q2	OK	7.59748	7.96331	0.0678477	0.0824881	0.89145	0.99995	no

ENSG00000168806	ENSG00000168806	LCMT2	15:43619973-43622803	q1	q2	OK	9.1089	9.38856	0.0436261	0.0838629	0.90835	0.99995	no

ENSG00000168807	ENSG00000168807	SNTB2	16:69139466-69442474	q1	q2	OK	6.1822	6.8003	0.137478	0.0674234	0.85395	0.99995	no

ENSG00000168811	ENSG00000168811	IL12A	3:159631188-159943086	q1	q2	NOTEST	0.348827	0.262728	-0.408941	0	1	1	no

ENSG00000168813	ENSG00000168813	ZNF507	19:32836499-32878573	q1	q2	OK	10.7005	11.9417	0.158332	0.311103	0.61705	0.99995	no

ENSG00000168818	ENSG00000168818	STX18	4:4349866-4712665	q1	q2	OK	18.7203	24.5036	0.388389	0.514364	0.4233	0.99995	no

ENSG00000168824	ENSG00000168824	NSG1	4:4349866-4712665	q1	q2	OK	15.7586	14.8897	-0.0818287	-0.107287	0.8864	0.99995	no

ENSG00000168826	ENSG00000168826	ZBTB49	4:4291923-4323513	q1	q2	OK	3.35453	3.52008	0.0694962	0.07442	0.89285	0.99995	no

ENSG00000168827	ENSG00000168827	GFM1	3:158362066-158410364	q1	q2	OK	18.5832	19.3635	0.0593423	0.0929558	0.8887	0.99995	no

ENSG00000168828	ENSG00000168828	OR13J1	9:35869374-35870461	q1	q2	NOTEST	0.0550013	0	#NAME?	0	1	1	no

ENSG00000168830	ENSG00000168830	HTR1E	6:87647023-87726349	q1	q2	NOTEST	0.0680753	0.0255849	-1.41184	0	1	1	no

ENSG00000168843	ENSG00000168843	FSTL5	4:162300104-163085187	q1	q2	OK	0.585242	0.552494	-0.0830756	-0.0808206	0.8864	0.99995	no

ENSG00000168852	ENSG00000168852	TPTE2P5	13:41396431-41495910	q1	q2	OK	2.04871	0.93999	-1.124	-0.450567	0.5096	0.99995	no

ENSG00000168872	ENSG00000168872	DDX19A	16:70323565-70407286	q1	q2	OK	23.2762	22.0468	-0.0782859	-0.142427	0.8263	0.99995	no

ENSG00000168874	ENSG00000168874	ATOH8	2:85978466-86015189	q1	q2	OK	6.2032	5.70144	-0.121688	-0.172346	0.8025	0.99995	no

ENSG00000168875	ENSG00000168875	SOX14	3:137483578-137484396	q1	q2	OK	1.30652	0.55018	-1.24775	-1.06491	0.1354	0.99995	no

ENSG00000168876	ENSG00000168876	ANKRD49	11:94152894-94232749	q1	q2	OK	9.03353	11.0631	0.292397	0.310778	0.6182	0.99995	no

ENSG00000168878	ENSG00000168878	SFTPB	2:85884436-85895864	q1	q2	NOTEST	0.0290602	0.0892939	1.61952	0	1	1	no

ENSG00000168883	ENSG00000168883	USP39	2:85825670-85876403	q1	q2	OK	52.6637	52.2956	-0.010121	-0.0178703	0.9763	0.99995	no

ENSG00000168884	ENSG00000168884	TNIP2	4:2743374-2758103	q1	q2	OK	13.4393	10.8843	-0.304204	-0.4961	0.45755	0.99995	no

ENSG00000168887	ENSG00000168887	C2orf68	2:85825670-85876403	q1	q2	OK	24.5082	22.4678	-0.125403	-0.110146	0.83375	0.99995	no

ENSG00000168890	ENSG00000168890	TMEM150A	2:85825670-85876403	q1	q2	OK	6.09853	6.18265	0.0197656	0.0106416	0.98115	0.99995	no

ENSG00000168894	ENSG00000168894	RNF181	2:85822847-85824736	q1	q2	OK	48.0407	45.3436	-0.0833597	-0.151091	0.83155	0.99995	no

ENSG00000168899	ENSG00000168899	VAMP5	2:85811530-85820535	q1	q2	OK	0.739223	0.768572	0.0561706	0.0417808	0.95185	0.99995	no

ENSG00000168903	ENSG00000168903	BTNL3	5:180415844-180433727	q1	q2	NOTEST	0	0	0	0	1	1	no

ENSG00000168904	ENSG00000168904	LRRC28	15:99791566-99930934	q1	q2	OK	12.2151	8.81208	-0.471106	-0.587676	0.30775	0.99995	no

ENSG00000168906	ENSG00000168906	MAT2A	2:85766287-85788670	q1	q2	OK	182.51	194.425	0.0912398	0.172662	0.7665	0.99995	no

ENSG00000168907	ENSG00000168907	PLA2G4F	15:42433331-42448839	q1	q2	NOTEST	0	0.0846944	inf	0	1	1	no

ENSG00000168913	ENSG00000168913	ENHO	9:34521037-34524239	q1	q2	OK	10.7024	9.92033	-0.109475	-0.169862	0.80975	0.99995	no

ENSG00000168916	ENSG00000168916	ZNF608	5:123972607-124084500	q1	q2	OK	15.3977	13.8216	-0.155791	-0.275953	0.6636	0.99995	no

ENSG00000168917	ENSG00000168917	SLC35G2	3:136537488-136580896	q1	q2	OK	4.54655	3.97748	-0.192916	-0.219194	0.7767	0.99995	no

ENSG00000168918	ENSG00000168918	INPP5D	2:233924676-234116549	q1	q2	OK	0.928359	1.05628	0.186243	0.114671	0.7443	0.99995	no

ENSG00000168924	ENSG00000168924	LETM1	4:1813205-1857974	q1	q2	OK	12.7105	12.7369	0.00299867	0.004325	0.99255	0.99995	no

ENSG00000168925	ENSG00000168925	CTRB1	16:75252897-75258822	q1	q2	NOTEST	0	0	0	0	1	1	no

ENSG00000168928	ENSG00000168928	CTRB2	16:75237993-75241083	q1	q2	NOTEST	0	0.0112366	inf	0	1	1	no

ENSG00000168930	ENSG00000168930	TRIM49	11:89530822-89541743	q1	q2	NOTEST	0.058483	0.0368546	-0.666173	0	1	1	no

ENSG00000168936	ENSG00000168936	TMEM129	4:1717678-1723085	q1	q2	OK	18.9978	14.6726	-0.372703	-0.696523	0.2958	0.99995	no

ENSG00000168938	ENSG00000168938	PPIC	5:122179133-122390362	q1	q2	OK	11.004	11.2511	0.0320417	0.0287195	0.9717	0.99995	no

ENSG00000168939	ENSG00000168939	SPRY3	X:154997473-155012121	q1	q2	OK	1.09351	1.03325	-0.0817743	-0.12835	0.85025	0.99995	no

ENSG00000168944	ENSG00000168944	CEP120	5:122680578-122759286	q1	q2	OK	7.80678	8.77717	0.169029	0.317651	0.6041	0.99995	no

ENSG00000168952	ENSG00000168952	STXBP6	14:25278861-25519503	q1	q2	OK	4.87502	4.51786	-0.109769	-0.113627	0.8709	0.99995	no

ENSG00000168955	ENSG00000168955	TM4SF20	2:228226871-228246711	q1	q2	NOTEST	0	0.0345155	inf	0	1	1	no

ENSG00000168958	ENSG00000168958	MFF	2:228029280-228222550	q1	q2	OK	65.4358	66.6915	0.0274218	0.0582343	0.9301	0.99995	no

ENSG00000168959	ENSG00000168959	GRM5	11:88237743-88799113	q1	q2	NOTEST	0.0236845	0.0520468	1.13587	0	1	1	no

ENSG00000168961	ENSG00000168961	LGALS9	17:25783669-25976586	q1	q2	OK	1.82918	1.05388	-0.795483	-0.239007	0.3421	0.99995	no

ENSG00000168967	ENSG00000168967	PMCHL1	5:21616370-22853731	q1	q2	NOTEST	0	0	0	0	1	1	no

ENSG00000168970	ENSG00000168970	JMJD7-PLA2G4B	15:42120282-42190773	q1	q2	OK	3.93597	4.2704	0.117652	0.198016	0.76905	0.99995	no

ENSG00000168992	ENSG00000168992	OR7E102P	2:96212278-96213315	q1	q2	NOTEST	0	0	0	0	1	1	no

ENSG00000168993	ENSG00000168993	CPLX1	4:778744-819986	q1	q2	OK	1.66798	1.45152	-0.200546	-0.186857	0.7436	0.99995	no

ENSG00000168994	ENSG00000168994	PXDC1	6:3722847-3754105	q1	q2	OK	26.4415	28.8355	0.125044	0.244075	0.72075	0.99995	no

ENSG00000168995	ENSG00000168995	SIGLEC7	19:51645555-51685222	q1	q2	NOTEST	0	0	0	0	1	1	no

ENSG00000169006	ENSG00000169006	NTSR2	2:11798303-11810290	q1	q2	NOTEST	0.0356206	0.0348486	-0.031611	0	1	1	no

ENSG00000169016	ENSG00000169016	E2F6	2:11584500-11606297	q1	q2	OK	18.692	18.5105	-0.0140756	-0.0262159	0.9676	0.99995	no

ENSG00000169018	ENSG00000169018	FEM1B	15:68560129-68588203	q1	q2	OK	19.7376	22.7494	0.204877	0.425867	0.46755	0.99995	no

ENSG00000169019	ENSG00000169019	COMMD8	4:47452884-47465736	q1	q2	OK	11.1756	11.9057	0.0913081	0.153225	0.81045	0.99995	no

ENSG00000169020	ENSG00000169020	ATP5I	4:666224-683230	q1	q2	OK	190.235	199.464	0.0683451	0.135617	0.8448	0.99995	no

ENSG00000169021	ENSG00000169021	UQCRFS1	19:29698172-29706722	q1	q2	OK	32.406	31.7771	-0.0282749	-0.0626829	0.92785	0.99995	no

ENSG00000169026	ENSG00000169026	MFSD7	4:666224-683230	q1	q2	NOTEST	0.242412	0.090172	-1.42671	0	1	1	no

ENSG00000169031	ENSG00000169031	COL4A3	2:228029280-228222550	q1	q2	NOTEST	0.0512197	0.00893693	-2.51885	0	1	1	no

ENSG00000169032	ENSG00000169032	MAP2K1	15:66679154-66790151	q1	q2	OK	87.7384	108.14	0.301618	0.639822	0.3569	0.99995	no

ENSG00000169035	ENSG00000169035	KLK7	19:51446558-51515385	q1	q2	NOTEST	0	0	0	0	1	1	no

ENSG00000169040	ENSG00000169040	PMCHL2	5:70647681-70742396	q1	q2	NOTEST	0	0	0	0	1	1	no

ENSG00000169045	ENSG00000169045	HNRNPH1	5:179041178-179061785	q1	q2	OK	683.471	739.809	0.114273	0.116929	0.7812	0.99995	no

ENSG00000169047	ENSG00000169047	IRS1	2:227596032-227664475	q1	q2	OK	9.10737	8.79499	-0.0503521	-0.0992186	0.8628	0.99995	no

ENSG00000169057	ENSG00000169057	MECP2	X:153287023-153363212	q1	q2	OK	0.621879	1.00698	0.695327	0.405842	0.41185	0.99995	no

ENSG00000169059	ENSG00000169059	VCX3A	X:6451658-6453159	q1	q2	NOTEST	0	0.0582923	inf	0	1	1	no

ENSG00000169062	ENSG00000169062	UPF3A	13:115047058-115071283	q1	q2	OK	57.5587	54.7503	-0.0721678	-0.128499	0.83495	0.99995	no

ENSG00000169064	ENSG00000169064	ZBBX	3:166958074-167099134	q1	q2	OK	0.268308	0.690029	1.36276	0.63222	0.0608	0.99995	no

ENSG00000169067	ENSG00000169067	ACTBL2	5:56690886-56829251	q1	q2	NOTEST	0	0	0	0	1	1	no

ENSG00000169071	ENSG00000169071	ROR2	9:94325372-94712444	q1	q2	OK	3.93896	4.3277	0.135788	0.135145	0.73585	0.99995	no

ENSG00000169075	ENSG00000169075	BRD7P3	6:118781934-119031238	q1	q2	NOTEST	0.0763782	0.0192975	-1.98475	0	1	1	no

ENSG00000169083	ENSG00000169083	AR	X:66764464-66950461	q1	q2	OK	4.97509	5.02404	0.0141252	0.0318074	0.9604	0.99995	no

ENSG00000169084	ENSG00000169084	DHRSX	X:2137556-2420846	q1	q2	OK	19.0215	17.1666	-0.148023	-0.145026	0.82225	0.99995	no

ENSG00000169085	ENSG00000169085	C8orf46	8:67341262-67430759	q1	q2	OK	14.9705	16.749	0.161954	0.269319	0.65345	0.99995	no

ENSG00000169087	ENSG00000169087	HSPBAP1	3:122458845-122512671	q1	q2	OK	8.16833	7.20071	-0.181903	-0.29642	0.6664	0.99995	no

ENSG00000169093	ENSG00000169093	ASMTL	X:1520661-1572655	q1	q2	OK	12.3631	10.753	-0.201293	-0.326413	0.61265	0.99995	no

ENSG00000169100	ENSG00000169100	SLC25A6	X:1505044-1511617	q1	q2	OK	238.243	214.754	-0.149747	-0.349156	0.61235	0.99995	no

ENSG00000169105	ENSG00000169105	CHST14	15:40763159-40765353	q1	q2	OK	19.7044	16.5732	-0.249671	-0.533963	0.441	0.99995	no

ENSG00000169116	ENSG00000169116	PARM1	4:75858304-75975325	q1	q2	OK	0.973053	1.01146	0.0558486	0.0740914	0.91675	0.99995	no

ENSG00000169118	ENSG00000169118	CSNK1G1	15:64457715-64679886	q1	q2	OK	6.01488	6.22231	0.0489146	0.0655393	0.9133	0.99995	no

ENSG00000169122	ENSG00000169122	FAM110B	8:58907067-59116838	q1	q2	OK	12.5187	11.0948	-0.174193	-0.20919	0.74525	0.99995	no

ENSG00000169126	ENSG00000169126	ARMC4	10:28064114-28287977	q1	q2	OK	1.72638	2.26528	0.391941	0.457324	0.41425	0.99995	no

ENSG00000169129	ENSG00000169129	AFAP1L2	10:116054582-116164515	q1	q2	OK	8.59788	10.2262	0.250219	0.460749	0.50205	0.99995	no

ENSG00000169131	ENSG00000169131	ZNF354A	5:178138592-178157703	q1	q2	OK	11.9919	12.6201	0.0736615	0.140943	0.82605	0.99995	no

ENSG00000169136	ENSG00000169136	ATF5	19:50392910-50464429	q1	q2	OK	11.2757	10.7637	-0.067047	-0.0518945	0.9303	0.99995	no

ENSG00000169139	ENSG00000169139	UBE2V2	8:48920959-48977268	q1	q2	OK	77.1395	81.4608	0.078636	0.151452	0.8249	0.99995	no

ENSG00000169154	ENSG00000169154	GOT1L1	8:37791798-37797647	q1	q2	NOTEST	0	0	0	0	1	1	no

ENSG00000169155	ENSG00000169155	ZBTB43	9:129567284-129600489	q1	q2	OK	6.85428	5.4765	-0.323751	-0.155008	0.5106	0.99995	no

ENSG00000169164	ENSG00000169164	RP11-167P23.2	X:55681141-55683447	q1	q2	NOTEST	0	0	0	0	1	1	no

ENSG00000169169	ENSG00000169169	CPT1C	19:50194154-50216988	q1	q2	OK	49.6334	49.7117	0.00227422	0.00374514	0.9952	0.99995	no

ENSG00000169174	ENSG00000169174	PCSK9	1:55505220-55530525	q1	q2	OK	4.98089	5.5581	0.15819	0.292605	0.6772	0.99995	no

ENSG00000169180	ENSG00000169180	XPO6	16:28109299-28223241	q1	q2	OK	32.9739	32.8694	-0.00457643	-0.00588702	0.98675	0.99995	no

ENSG00000169181	ENSG00000169181	GSG1L	16:27798849-28074830	q1	q2	NOTEST	0.287364	0.144117	-0.995642	0	1	1	no

ENSG00000169184	ENSG00000169184	MN1	22:28144264-28197486	q1	q2	OK	25.5254	22.8001	-0.162893	-0.377308	0.5668	0.99995	no

ENSG00000169188	ENSG00000169188	APEX2	X:55026789-55057497	q1	q2	OK	6.53881	6.29355	-0.0551538	-0.0834767	0.8903	0.99995	no

ENSG00000169189	ENSG00000169189	NSMCE1	16:27236311-27301813	q1	q2	OK	17.9852	17.7369	-0.0200571	-0.0335852	0.95795	0.99995	no

ENSG00000169193	ENSG00000169193	CCDC126	7:23636997-23684327	q1	q2	OK	5.9367	7.6742	0.370355	0.515526	0.3935	0.99995	no

ENSG00000169194	ENSG00000169194	IL13	5:131877135-131999964	q1	q2	NOTEST	0.0348464	0.0169332	-1.04115	0	1	1	no

ENSG00000169202	ENSG00000169202	OR10G1P	14:22070048-22070976	q1	q2	NOTEST	0	0	0	0	1	1	no

ENSG00000169203	ENSG00000169203	RP11-231C14.4	16:29262828-29519817	q1	q2	OK	1.55745	1.38614	-0.16811	-0.0914201	0.83165	0.99995	no

ENSG00000169208	ENSG00000169208	OR10G3	14:22037933-22038875	q1	q2	NOTEST	0	0	0	0	1	1	no

ENSG00000169213	ENSG00000169213	RAB3B	1:52373627-52456436	q1	q2	OK	1.35824	1.41518	0.0592487	0.107557	0.8793	0.99995	no

ENSG00000169214	ENSG00000169214	OR6F1	1:247670359-247910364	q1	q2	NOTEST	0	0	0	0	1	1	no

ENSG00000169217	ENSG00000169217	CD2BP2	16:30362086-30366682	q1	q2	OK	38.5642	37.7328	-0.0314396	-0.0660492	0.91575	0.99995	no

ENSG00000169218	ENSG00000169218	RSPO1	1:38076950-38100595	q1	q2	NOTEST	0.107921	0.152206	0.496041	0	1	1	no

ENSG00000169220	ENSG00000169220	RGS14	5:176784837-176799602	q1	q2	OK	5.38959	4.70146	-0.197064	-0.172775	0.7641	0.99995	no

ENSG00000169221	ENSG00000169221	TBC1D10B	16:30368422-30381585	q1	q2	OK	19.745	16.9612	-0.219248	-0.381586	0.49495	0.99995	no

ENSG00000169223	ENSG00000169223	LMAN2	5:176758562-176778853	q1	q2	OK	53.549	48.6831	-0.137439	-0.247798	0.7042	0.99995	no

ENSG00000169224	ENSG00000169224	GCSAML	1:247670359-247910364	q1	q2	NOTEST	0.0212368	0	#NAME?	0	1	1	no

ENSG00000169228	ENSG00000169228	RAB24	5:176728198-176739758	q1	q2	OK	23.0745	20.4691	-0.172854	-0.168034	0.823	0.99995	no

ENSG00000169230	ENSG00000169230	PRELID1	5:176728198-176739758	q1	q2	OK	71.9971	67.5375	-0.0922505	-0.164928	0.805	0.99995	no

ENSG00000169231	ENSG00000169231	THBS3	1:155165378-155183615	q1	q2	OK	34.8304	30.6136	-0.186175	-0.262286	0.7154	0.99995	no

ENSG00000169239	ENSG00000169239	CA5B	X:15693054-15805747	q1	q2	OK	1.81803	2.02511	0.155623	0.114751	0.75455	0.99995	no

ENSG00000169241	ENSG00000169241	SLC50A1	1:155107819-155111329	q1	q2	OK	14.0704	12.6542	-0.153042	-0.226939	0.75085	0.99995	no

ENSG00000169242	ENSG00000169242	EFNA1	1:155099935-155107333	q1	q2	NOTEST	0.396488	0.451633	0.187874	0	1	1	no

ENSG00000169245	ENSG00000169245	CXCL10	4:76932336-77033955	q1	q2	NOTEST	0.0731701	0.0759105	0.0530441	0	1	1	no

ENSG00000169246	ENSG00000169246	NPIPB3	16:21413557-21531765	q1	q2	OK	7.36902	7.8298	0.0875034	0.0905895	0.8766	0.99995	no

ENSG00000169247	ENSG00000169247	SH3TC2	5:148303201-148442726	q1	q2	NOTEST	0.0248246	0.0500245	1.01086	0	1	1	no

ENSG00000169248	ENSG00000169248	CXCL11	4:76932336-77033955	q1	q2	NOTEST	0.0346828	0	#NAME?	0	1	1	no

ENSG00000169249	ENSG00000169249	ZRSR2	X:15808594-15841383	q1	q2	OK	7.59446	6.78314	-0.162995	-0.249374	0.70765	0.99995	no

ENSG00000169251	ENSG00000169251	NMD3	3:160801670-160971320	q1	q2	OK	19.9607	21.7616	0.12462	0.228512	0.71045	0.99995	no

ENSG00000169252	ENSG00000169252	ADRB2	5:148206155-148208196	q1	q2	NOTEST	0.251936	0.198266	-0.345619	0	1	1	no

ENSG00000169253	ENSG00000169253	RP11-220D10.1	9:111389112-111389430	q1	q2	OK	0.790577	0.386226	-1.03346	-0.428984	0.42895	0.99995	no

ENSG00000169255	ENSG00000169255	B3GALNT1	3:160801670-160971320	q1	q2	OK	1.88945	2.07859	0.137643	0.0826253	0.86525	0.99995	no

ENSG00000169258	ENSG00000169258	GPRIN1	5:175969511-176037134	q1	q2	OK	12.9507	12.8581	-0.0103454	-0.0229755	0.97495	0.99995	no

ENSG00000169271	ENSG00000169271	HSPB3	5:53751444-53752207	q1	q2	NOTEST	0	0	0	0	1	1	no

ENSG00000169282	ENSG00000169282	KCNAB1	3:155755489-156256545	q1	q2	OK	0.474651	0.635649	0.421363	0.248099	0.45985	0.99995	no

ENSG00000169288	ENSG00000169288	MRPL1	4:78783673-78873944	q1	q2	OK	24.2954	30.0468	0.306528	0.5906	0.3653	0.99995	no

ENSG00000169291	ENSG00000169291	SHE	1:154442247-154474589	q1	q2	NOTEST	0.0253596	0.0288649	0.186785	0	1	1	no

ENSG00000169297	ENSG00000169297	NR0B1	X:30322322-30327715	q1	q2	OK	0.674591	0.600804	-0.167118	-0.133791	0.8274	0.99995	no

ENSG00000169299	ENSG00000169299	PGM2	4:37828254-37864558	q1	q2	OK	13.9253	15.8702	0.188618	0.383639	0.5865	0.99995	no

ENSG00000169302	ENSG00000169302	STK32A	5:146614525-146767415	q1	q2	NOTEST	0.183759	0.367309	0.999174	0	1	1	no

ENSG00000169306	ENSG00000169306	IL1RAPL1	X:28605515-29974840	q1	q2	NOTEST	0.230189	0.332904	0.532286	0	1	1	no

ENSG00000169313	ENSG00000169313	P2RY12	3:150803483-151176497	q1	q2	NOTEST	0.0311387	0.0462591	0.571028	0	1	1	no

ENSG00000169314	ENSG00000169314	C22orf15	22:24105207-24126503	q1	q2	OK	0.571561	0.983922	0.783637	0.293134	0.57095	0.99995	no

ENSG00000169325	ENSG00000169325	GUSBP12	7:57268437-57271304	q1	q2	NOTEST	0	0	0	0	1	1	no

ENSG00000169327	ENSG00000169327	OR5AU1	14:21623025-21624222	q1	q2	NOTEST	0.0238027	0	#NAME?	0	1	1	no

ENSG00000169330	ENSG00000169330	KIAA1024	15:79724857-79764632	q1	q2	OK	1.28763	1.51728	0.236766	0.371305	0.59995	0.99995	no

ENSG00000169340	ENSG00000169340	PDILT	16:20370491-20416059	q1	q2	NOTEST	0	0	0	0	1	1	no

ENSG00000169344	ENSG00000169344	UMOD	16:20344373-20367623	q1	q2	NOTEST	0	0	0	0	1	1	no

ENSG00000169347	ENSG00000169347	GP2	16:20320893-20339130	q1	q2	NOTEST	0	0	0	0	1	1	no

ENSG00000169359	ENSG00000169359	SLC33A1	3:155538812-155572218	q1	q2	OK	9.34219	8.98248	-0.0566462	-0.0715391	0.9078	0.99995	no

ENSG00000169371	ENSG00000169371	SNUPN	15:75759461-75918810	q1	q2	OK	15.8856	14.6635	-0.115493	-0.113231	0.85705	0.99995	no

ENSG00000169372	ENSG00000169372	CRADD	12:94071150-94288616	q1	q2	OK	10.8899	9.90083	-0.137374	-0.218626	0.74195	0.99995	no

ENSG00000169375	ENSG00000169375	SIN3A	15:75648132-75748183	q1	q2	OK	25.8273	25.812	-0.000852419	-0.00125654	0.9982	0.99995	no

ENSG00000169379	ENSG00000169379	ARL13B	3:93698982-93847389	q1	q2	OK	12.5868	13.9236	0.145614	0.215493	0.71925	0.99995	no

ENSG00000169385	ENSG00000169385	RNASE2	14:21423610-21424595	q1	q2	NOTEST	0.0427636	0	#NAME?	0	1	1	no

ENSG00000169393	ENSG00000169393	ELSPBP1	19:48497907-48528410	q1	q2	NOTEST	0	0	0	0	1	1	no

ENSG00000169397	ENSG00000169397	RNASE3	14:21359557-21360507	q1	q2	NOTEST	0	0	0	0	1	1	no

ENSG00000169398	ENSG00000169398	PTK2	8:141667998-142012315	q1	q2	OK	82.0761	83.3872	0.0228636	0.0351936	0.9352	0.99995	no

ENSG00000169402	ENSG00000169402	RSPH10B2	7:6793739-6866401	q1	q2	NOTEST	0.0768092	0.0494714	-0.634682	0	1	1	no

ENSG00000169403	ENSG00000169403	PTAFR	1:28473676-28520447	q1	q2	NOTEST	0.0792169	0.142008	0.842091	0	1	1	no

ENSG00000169410	ENSG00000169410	PTPN9	15:75759461-75918810	q1	q2	OK	20.9258	22.1697	0.0833071	0.156868	0.788	0.99995	no

ENSG00000169413	ENSG00000169413	RNASE6	14:21249209-21250626	q1	q2	OK	0.228195	0.637063	1.48117	1.18073	0.12485	0.99995	no

ENSG00000169418	ENSG00000169418	NPR1	1:153651112-153666468	q1	q2	NOTEST	0.369136	0.364735	-0.0173021	0	1	1	no

ENSG00000169427	ENSG00000169427	KCNK9	8:140613080-140715299	q1	q2	NOTEST	0.111279	0.0686413	-0.697031	0	1	1	no

ENSG00000169429	ENSG00000169429	IL8	4:74606222-74609433	q1	q2	NOTEST	0.265133	0.0630294	-2.07262	0	1	1	no

ENSG00000169432	ENSG00000169432	SCN9A	2:166813908-167232503	q1	q2	OK	1.40935	1.49932	0.0892772	0.125804	0.85305	0.99995	no

ENSG00000169435	ENSG00000169435	RASSF6	4:74437266-74486348	q1	q2	NOTEST	0.00409538	0	#NAME?	0	1	1	no

ENSG00000169436	ENSG00000169436	COL22A1	8:139600477-139926249	q1	q2	OK	0.8267	0.761829	-0.117896	-0.062344	0.88035	0.99995	no

ENSG00000169439	ENSG00000169439	SDC2	8:97505578-97624000	q1	q2	OK	149.26	135.68	-0.137621	-0.225209	0.7442	0.99995	no

ENSG00000169442	ENSG00000169442	CD52	1:26605666-26647014	q1	q2	NOTEST	0	0	0	0	1	1	no

ENSG00000169446	ENSG00000169446	MMGT1	X:135044228-135056222	q1	q2	NOTEST	0.0793711	0.0984304	0.310489	0	1	1	no

ENSG00000169469	ENSG00000169469	SPRR1B	1:153003677-153005376	q1	q2	NOTEST	0	0	0	0	1	1	no

ENSG00000169474	ENSG00000169474	SPRR1A	1:152956556-152958289	q1	q2	NOTEST	0	0	0	0	1	1	no

ENSG00000169484	ENSG00000169484	OR4K14	14:20482418-20483352	q1	q2	NOTEST	0	0	0	0	1	1	no

ENSG00000169488	ENSG00000169488	OR4K15	14:20443602-20444818	q1	q2	NOTEST	0	0	0	0	1	1	no

ENSG00000169490	ENSG00000169490	TM2D2	8:38846326-38854343	q1	q2	OK	18.9238	19.5585	0.0475964	0.0743411	0.8972	0.99995	no

ENSG00000169495	ENSG00000169495	HTRA4	8:38831682-38846181	q1	q2	NOTEST	0	0	0	0	1	1	no

ENSG00000169499	ENSG00000169499	PLEKHA2	8:38758752-38831428	q1	q2	OK	5.56383	6.14939	0.144366	0.192857	0.663	0.99995	no

ENSG00000169504	ENSG00000169504	CLIC4	1:25071847-25170815	q1	q2	OK	106.344	119.969	0.173922	0.405597	0.5561	0.99995	no

ENSG00000169507	ENSG00000169507	SLC38A11	2:165752695-165812035	q1	q2	NOTEST	0.00707137	0.120686	4.09312	0	1	1	no

ENSG00000169508	ENSG00000169508	GPR183	13:99853027-100038688	q1	q2	NOTEST	0.0817029	0.127201	0.638656	0	1	1	no

ENSG00000169509	ENSG00000169509	CRCT1	1:152486977-152488486	q1	q2	NOTEST	0	0	0	0	1	1	no

ENSG00000169515	ENSG00000169515	CCDC8	19:46913628-46916841	q1	q2	OK	18.0651	16.8763	-0.0982119	-0.224457	0.7465	0.99995	no

ENSG00000169519	ENSG00000169519	METTL15	11:28042166-28355054	q1	q2	OK	5.29176	5.68167	0.102568	0.0982486	0.8577	0.99995	no

ENSG00000169548	ENSG00000169548	ZNF280A	22:22868059-22874613	q1	q2	NOTEST	0	0	0	0	1	1	no

ENSG00000169550	ENSG00000169550	MUC15	11:26210669-26684835	q1	q2	NOTEST	0	0	0	0	1	1	no

ENSG00000169551	ENSG00000169551	CT55	X:134290460-134305322	q1	q2	NOTEST	0	0	0	0	1	1	no

ENSG00000169554	ENSG00000169554	ZEB2	2:145141647-145282147	q1	q2	OK	38.84	35.9973	-0.109657	-0.151394	0.7206	0.99995	no

ENSG00000169562	ENSG00000169562	GJB1	X:70435043-70445366	q1	q2	NOTEST	0.0180446	0	#NAME?	0	1	1	no

ENSG00000169564	ENSG00000169564	PCBP1	2:70187225-70316332	q1	q2	OK	187.546	195.475	0.0597349	0.139823	0.84555	0.99995	no

ENSG00000169567	ENSG00000169567	HINT1	5:130494719-130541119	q1	q2	OK	204.59	203.198	-0.0098507	-0.0213286	0.97595	0.99995	no

ENSG00000169570	ENSG00000169570	DTWD2	5:118173016-118324240	q1	q2	OK	2.44109	2.38465	-0.0337504	-0.040073	0.9519	0.99995	no

ENSG00000169575	ENSG00000169575	VPREB1	22:22599086-22599927	q1	q2	NOTEST	0	0	0	0	1	1	no

ENSG00000169583	ENSG00000169583	CLIC3	9:139889086-139891255	q1	q2	NOTEST	0	0	0	0	1	1	no

ENSG00000169592	ENSG00000169592	INO80E	16:30003644-30034591	q1	q2	OK	33.7547	32.1681	-0.0694554	-0.0787165	0.89905	0.99995	no

ENSG00000169594	ENSG00000169594	BNC1	15:83776158-84108197	q1	q2	NOTEST	0.398172	0.473051	0.248604	0	1	1	no

ENSG00000169598	ENSG00000169598	DFFB	1:3773844-3801993	q1	q2	OK	5.00795	4.69087	-0.0943629	-0.117456	0.8355	0.99995	no

ENSG00000169599	ENSG00000169599	NFU1	2:69622881-69664760	q1	q2	OK	34.1821	33.8571	-0.0137799	-0.0221903	0.97495	0.99995	no

ENSG00000169604	ENSG00000169604	ANTXR1	2:69240309-69476459	q1	q2	OK	43.0321	42.2781	-0.0255041	-0.0554542	0.9277	0.99995	no

ENSG00000169605	ENSG00000169605	GKN1	2:69201704-69208106	q1	q2	NOTEST	0	0	0	0	1	1	no

ENSG00000169607	ENSG00000169607	CKAP2L	2:113479062-113522254	q1	q2	OK	16.4784	16.0771	-0.0355738	-0.0675654	0.903	0.99995	no

ENSG00000169609	ENSG00000169609	C15orf40	15:83509837-83772195	q1	q2	OK	10.2624	9.16833	-0.16264	-0.0832317	0.86385	0.99995	no

ENSG00000169612	ENSG00000169612	FAM103A1	15:83509837-83772195	q1	q2	OK	13.94	14.0493	0.011267	0.0102089	0.98835	0.99995	no

ENSG00000169618	ENSG00000169618	PROKR1	2:68870720-68882708	q1	q2	NOTEST	0.127024	0.0587052	-1.11354	0	1	1	no

ENSG00000169621	ENSG00000169621	APLF	2:68694692-68858004	q1	q2	OK	2.31129	2.48546	0.10482	0.150069	0.8228	0.99995	no

ENSG00000169627	ENSG00000169627	BOLA2B	16:30204254-30215631	q1	q2	OK	1.34727	1.51121	0.165666	0.056162	0.92965	0.99995	no

ENSG00000169629	ENSG00000169629	RGPD8	2:113127668-113192065	q1	q2	OK	0.470299	0.612727	0.381666	0.384655	0.46905	0.99995	no

ENSG00000169635	ENSG00000169635	HIC2	22:21771692-21805752	q1	q2	OK	11.8653	12.2855	0.0502113	0.119103	0.86125	0.99995	no

ENSG00000169641	ENSG00000169641	LUZP1	1:23345940-23504301	q1	q2	OK	11.0988	12.0416	0.117634	0.102653	0.85975	0.99995	no

ENSG00000169660	ENSG00000169660	HEXDC	17:80347098-80408705	q1	q2	OK	23.4606	20.0927	-0.223567	-0.212215	0.72055	0.99995	no

ENSG00000169662	ENSG00000169662	BCRP6	22:21645048-21648875	q1	q2	NOTEST	0	0.0541349	inf	0	1	1	no

ENSG00000169668	ENSG00000169668	BCRP2	22:21457304-21482352	q1	q2	NOTEST	0.0152424	0.0731071	2.26192	0	1	1	no

ENSG00000169676	ENSG00000169676	DRD5	4:9772776-10056560	q1	q2	NOTEST	0.0333239	0.0211557	-0.655512	0	1	1	no

ENSG00000169679	ENSG00000169679	BUB1	2:111395274-111435691	q1	q2	OK	39.7513	44.0568	0.148364	0.314961	0.59825	0.99995	no

ENSG00000169682	ENSG00000169682	SPNS1	16:28985541-29002104	q1	q2	OK	21.8339	18.205	-0.262234	-0.507835	0.4605	0.99995	no

ENSG00000169683	ENSG00000169683	LRRC45	17:79976577-79989027	q1	q2	OK	15.3767	12.8798	-0.255631	-0.307404	0.622	0.99995	no

ENSG00000169684	ENSG00000169684	CHRNA5	15:78857861-78913637	q1	q2	OK	11.6944	12.6995	0.118955	0.129166	0.85645	0.99995	no

ENSG00000169688	ENSG00000169688	MT1B	16:56682159-56687116	q1	q2	NOTEST	0	0	0	0	1	1	no

ENSG00000169689	ENSG00000169689	STRA13	17:79976577-79989027	q1	q2	OK	49.3218	49.1002	-0.00649728	-0.0106466	0.989	0.99995	no

ENSG00000169692	ENSG00000169692	AGPAT2	9:139567594-139581875	q1	q2	OK	10.2031	7.90357	-0.36843	-0.586034	0.38315	0.99995	no

ENSG00000169696	ENSG00000169696	ASPSCR1	17:79934682-79975282	q1	q2	OK	14.7008	12.4245	-0.242707	-0.362085	0.55255	0.99995	no

ENSG00000169704	ENSG00000169704	GP9	3:128779609-128781249	q1	q2	NOTEST	0.0344451	0	#NAME?	0	1	1	no

ENSG00000169710	ENSG00000169710	FASN	17:80036213-80056208	q1	q2	OK	114.344	111.586	-0.0352226	-0.0413983	0.93825	0.99995	no

ENSG00000169714	ENSG00000169714	CNBP	3:128888326-128902765	q1	q2	OK	191.143	197.955	0.0505206	0.120482	0.85835	0.99995	no

ENSG00000169715	ENSG00000169715	MT1E	16:56659386-56661024	q1	q2	NOTEST	0.0470918	0	#NAME?	0	1	1	no

ENSG00000169717	ENSG00000169717	ACTRT2	1:2938045-2939465	q1	q2	NOTEST	0.0205236	0	#NAME?	0	1	1	no

ENSG00000169718	ENSG00000169718	DUS1L	17:80015381-80023763	q1	q2	OK	34.4846	31.5254	-0.129437	-0.230284	0.73545	0.99995	no

ENSG00000169727	ENSG00000169727	GPS1	17:80005777-80015346	q1	q2	OK	58.6794	51.8827	-0.1776	-0.326323	0.57695	0.99995	no

ENSG00000169733	ENSG00000169733	RFNG	17:80005777-80015346	q1	q2	OK	17.3806	15.2778	-0.186035	-0.191251	0.76545	0.99995	no

ENSG00000169738	ENSG00000169738	DCXR	17:79993011-79995608	q1	q2	OK	46.4612	45.6971	-0.0239236	-0.0484265	0.94605	0.99995	no

ENSG00000169740	ENSG00000169740	ZNF32	10:44124264-44170151	q1	q2	OK	33.3141	35.2914	0.0831827	0.168774	0.8108	0.99995	no

ENSG00000169744	ENSG00000169744	LDB2	4:16402052-16900432	q1	q2	OK	22.9355	23.0959	0.0100538	0.0199789	0.975	0.99995	no

ENSG00000169750	ENSG00000169750	RAC3	17:79989499-79992080	q1	q2	OK	25.7168	24.0699	-0.0954802	-0.18083	0.79145	0.99995	no

ENSG00000169752	ENSG00000169752	NRG4	15:76228309-76352136	q1	q2	OK	4.61512	4.61896	0.00120023	0.0011635	0.99805	0.99995	no

ENSG00000169756	ENSG00000169756	LIMS1	2:109150856-109303702	q1	q2	OK	21.4186	25.2613	0.238068	0.349481	0.55435	0.99995	no

ENSG00000169758	ENSG00000169758	C15orf27	15:76352177-76603813	q1	q2	NOTEST	0.349064	0.360515	0.0465646	0	1	1	no

ENSG00000169760	ENSG00000169760	NLGN1	3:173114073-174004434	q1	q2	OK	2.79781	2.85675	0.0300759	0.0267894	0.94225	0.99995	no

ENSG00000169762	ENSG00000169762	TAPT1	4:16162127-16321763	q1	q2	OK	14.1905	16.1371	0.18545	0.193508	0.71815	0.99995	no

ENSG00000169763	ENSG00000169763	PRYP3	Y:25827586-25840726	q1	q2	NOTEST	0	0	0	0	1	1	no

ENSG00000169764	ENSG00000169764	UGP2	2:64068073-64118696	q1	q2	OK	52.3031	55.5382	0.0865846	0.200222	0.75455	0.99995	no

ENSG00000169777	ENSG00000169777	TAS2R1	5:9621488-9903938	q1	q2	NOTEST	0.137665	0	#NAME?	0	1	1	no

ENSG00000169783	ENSG00000169783	LINGO1	15:77905368-78113242	q1	q2	OK	14.1185	13.8022	-0.0326827	-0.0530078	0.92965	0.99995	no

ENSG00000169789	ENSG00000169789	PRY	Y:24636543-24660784	q1	q2	NOTEST	0	0	0	0	1	1	no

ENSG00000169800	ENSG00000169800	RBMY1F	Y:24314688-24329129	q1	q2	NOTEST	0	0	0	0	1	1	no

ENSG00000169807	ENSG00000169807	PRY2	Y:24217902-24242154	q1	q2	NOTEST	0	0	0	0	1	1	no

ENSG00000169811	ENSG00000169811	RBMY1HP	Y:23655404-23663854	q1	q2	NOTEST	0	0	0	0	1	1	no

ENSG00000169813	ENSG00000169813	HNRNPF	10:43881064-43904614	q1	q2	OK	154.206	152.766	-0.0135355	-0.0300275	0.9643	0.99995	no

ENSG00000169814	ENSG00000169814	BTD	3:15602210-15687329	q1	q2	OK	3.91122	3.33098	-0.231673	-0.187359	0.72075	0.99995	no

ENSG00000169826	ENSG00000169826	CSGALNACT2	10:43633933-43680756	q1	q2	OK	13.7749	15.5426	0.174188	0.39846	0.56305	0.99995	no

ENSG00000169836	ENSG00000169836	TACR3	4:104469901-104640973	q1	q2	NOTEST	0.0984359	0.095132	-0.0492539	0	1	1	no

ENSG00000169840	ENSG00000169840	GSX1	13:28366779-28368905	q1	q2	NOTEST	0.447733	0.241814	-0.888743	0	1	1	no

ENSG00000169849	ENSG00000169849	TSPY14P	Y:23630043-23632569	q1	q2	NOTEST	0	0	0	0	1	1	no

ENSG00000169851	ENSG00000169851	PCDH7	4:30722036-31148422	q1	q2	OK	6.49859	6.95297	0.0975025	0.0974507	0.7905	0.99995	no

ENSG00000169855	ENSG00000169855	ROBO1	3:78646389-79816965	q1	q2	OK	71.4618	74.1637	0.0535405	0.0538544	0.92345	0.99995	no

ENSG00000169856	ENSG00000169856	ONECUT1	15:53049636-53083273	q1	q2	OK	0.841967	1.86828	1.14988	0.904751	0.20015	0.99995	no

ENSG00000169857	ENSG00000169857	AVEN	15:34158427-34357291	q1	q2	OK	12.9883	13.1131	0.0137917	0.0251497	0.96825	0.99995	no

ENSG00000169860	ENSG00000169860	P2RY1	3:152552735-152555841	q1	q2	OK	1.77935	1.76717	-0.00991268	-0.0130804	0.9848	0.99995	no

ENSG00000169861	ENSG00000169861	IGHV1OR21-1	21:10862621-10863067	q1	q2	NOTEST	0	0	0	0	1	1	no

ENSG00000169862	ENSG00000169862	CTNND2	5:10971951-11904155	q1	q2	OK	44.8949	44.3538	-0.0174966	-0.036946	0.95335	0.99995	no

ENSG00000169871	ENSG00000169871	TRIM56	7:100728719-100735017	q1	q2	OK	4.95063	6.04895	0.289071	0.392794	0.4908	0.99995	no

ENSG00000169876	ENSG00000169876	MUC17	7:100663352-100702020	q1	q2	NOTEST	0.00872205	0.00520441	-0.744934	0	1	1	no

ENSG00000169877	ENSG00000169877	AHSP	16:31539184-31540124	q1	q2	NOTEST	0	0	0	0	1	1	no

ENSG00000169884	ENSG00000169884	WNT10B	12:49359122-49365546	q1	q2	NOTEST	0.295873	0.19552	-0.597665	0	1	1	no

ENSG00000169885	ENSG00000169885	CALML6	1:1846265-1848735	q1	q2	NOTEST	0.142665	0.133387	-0.097011	0	1	1	no

ENSG00000169891	ENSG00000169891	REPS2	X:16964813-17171395	q1	q2	OK	0.553724	0.527778	-0.0692345	-0.0347434	0.93635	0.99995	no

ENSG00000169894	ENSG00000169894	MUC3A	7:100547186-100611410	q1	q2	NOTEST	0.265314	0.363097	0.452657	0	1	1	no

ENSG00000169895	ENSG00000169895	SYAP1	X:16737754-16783459	q1	q2	OK	8.74242	9.71596	0.152324	0.294244	0.61125	0.99995	no

ENSG00000169896	ENSG00000169896	ITGAM	16:31271310-31344213	q1	q2	NOTEST	0.0295608	0.0208034	-0.506865	0	1	1	no

ENSG00000169900	ENSG00000169900	PYDC1	16:31225341-31236510	q1	q2	NOTEST	0	0.0379261	inf	0	1	1	no

ENSG00000169902	ENSG00000169902	TPST1	7:65670185-65955095	q1	q2	OK	12.2319	12.7328	0.0579039	0.0676276	0.89995	0.99995	no

ENSG00000169903	ENSG00000169903	TM4SF4	3:149191760-149221068	q1	q2	NOTEST	0	0	0	0	1	1	no

ENSG00000169905	ENSG00000169905	TOR1AIP2	1:179809101-179846938	q1	q2	OK	16.8032	17.9948	0.0988422	0.148255	0.8232	0.99995	no

ENSG00000169906	ENSG00000169906	S100G	X:16606125-16731059	q1	q2	NOTEST	0	0	0	0	1	1	no

ENSG00000169908	ENSG00000169908	TM4SF1	3:149086808-149104370	q1	q2	OK	3.95879	4.6493	0.231954	0.305585	0.64795	0.99995	no

ENSG00000169914	ENSG00000169914	OTUD3	1:20209005-20239438	q1	q2	OK	9.44378	10.8944	0.206157	0.21134	0.49085	0.99995	no

ENSG00000169918	ENSG00000169918	OTUD7A	15:31775328-32162992	q1	q2	OK	1.21499	1.16963	-0.0549033	-0.0436435	0.95165	0.99995	no

ENSG00000169919	ENSG00000169919	GUSB	7:65425670-65447301	q1	q2	OK	26.5059	21.3536	-0.311833	-0.562634	0.3271	0.99995	no

ENSG00000169925	ENSG00000169925	BRD3	9:136890560-136933657	q1	q2	OK	28.1658	26.4496	-0.0906987	-0.174679	0.7694	0.99995	no

ENSG00000169926	ENSG00000169926	KLF13	15:31619057-31727868	q1	q2	OK	20.2501	22.7003	0.164784	0.184488	0.67335	0.99995	no

ENSG00000169933	ENSG00000169933	FRMPD4	X:12156584-12742642	q1	q2	NOTEST	0.0207136	0.011527	-0.845569	0	1	1	no

ENSG00000169946	ENSG00000169946	ZFPM2	8:105602960-107072752	q1	q2	OK	2.91108	2.90913	-0.000962836	-0.00148151	0.99795	0.99995	no

ENSG00000169951	ENSG00000169951	ZNF764	16:30535324-30569819	q1	q2	OK	7.17188	6.37269	-0.170449	-0.15775	0.80965	0.99995	no

ENSG00000169953	ENSG00000169953	HSFY2	Y:20835918-20991373	q1	q2	NOTEST	0.0380103	0.0179831	-1.07975	0	1	1	no

ENSG00000169955	ENSG00000169955	ZNF747	16:30535324-30569819	q1	q2	OK	1.19732	0.940262	-0.348671	-0.147285	0.87135	0.99995	no

ENSG00000169957	ENSG00000169957	ZNF768	16:30535324-30569819	q1	q2	OK	43.894	38.9152	-0.17369	-0.372506	0.587	0.99995	no

ENSG00000169962	ENSG00000169962	TAS1R3	1:1266693-1284730	q1	q2	NOTEST	0.256695	0.199266	-0.365358	0	1	1	no

ENSG00000169964	ENSG00000169964	TMEM42	3:44803208-44914868	q1	q2	OK	3.72729	2.82061	-0.402119	-0.192462	0.8462	0.99995	no

ENSG00000169967	ENSG00000169967	MAP3K2	2:128056305-128146041	q1	q2	OK	7.27854	7.99224	0.13495	0.172798	0.6846	0.99995	no

ENSG00000169972	ENSG00000169972	PUSL1	1:1227755-1260071	q1	q2	OK	10.512	10.0332	-0.0672601	-0.0386184	0.94335	0.99995	no

ENSG00000169976	ENSG00000169976	SF3B5	6:144416017-144416754	q1	q2	OK	97.2739	89.936	-0.113154	-0.259173	0.7107	0.99995	no

ENSG00000169981	ENSG00000169981	ZNF35	3:44596684-44727145	q1	q2	OK	9.93855	9.4724	-0.069305	-0.0570438	0.9311	0.99995	no

ENSG00000169989	ENSG00000169989	TIGD4	4:153690505-153700916	q1	q2	OK	2.50541	2.62022	0.0646421	0.0892329	0.89865	0.99995	no

ENSG00000169991	ENSG00000169991	IFFO2	1:19166092-19283180	q1	q2	OK	4.41009	3.95961	-0.15545	-0.206315	0.7321	0.99995	no

ENSG00000169992	ENSG00000169992	NLGN2	17:7308192-7323179	q1	q2	OK	57.2058	50.5062	-0.179702	-0.384582	0.5232	0.99995	no

ENSG00000169994	ENSG00000169994	MYO7B	2:128293377-128395304	q1	q2	NOTEST	0.0523926	0.0110608	-2.24391	0	1	1	no

ENSG00000170004	ENSG00000170004	CHD3	17:7760002-7816078	q1	q2	OK	55.0207	52.1185	-0.0781804	-0.11613	0.82595	0.99995	no

ENSG00000170006	ENSG00000170006	TMEM154	4:153539783-153601317	q1	q2	NOTEST	0.190219	0.239774	0.334015	0	1	1	no

ENSG00000170011	ENSG00000170011	MYRIP	3:39850404-40301812	q1	q2	NOTEST	0.294711	0.210978	-0.482212	0	1	1	no

ENSG00000170017	ENSG00000170017	ALCAM	3:105085752-105295744	q1	q2	OK	5.28502	4.30216	-0.296849	-0.505062	0.4205	0.99995	no

ENSG00000170027	ENSG00000170027	YWHAG	7:75956115-75988348	q1	q2	OK	113.423	118.238	0.0599831	0.143197	0.8372	0.99995	no

ENSG00000170035	ENSG00000170035	UBE2E3	2:181831974-182264286	q1	q2	OK	286.113	294.311	0.0407586	0.0759727	0.90865	0.99995	no

ENSG00000170037	ENSG00000170037	CNTROB	17:7825176-7853236	q1	q2	OK	27.911	22.3476	-0.320715	-0.409575	0.4663	0.99995	no

ENSG00000170043	ENSG00000170043	TRAPPC1	17:7825176-7853236	q1	q2	OK	82.1946	81.7202	-0.00835066	-0.0127091	0.987	0.99995	no

ENSG00000170044	ENSG00000170044	ZPLD1	3:101818087-102198685	q1	q2	NOTEST	0.0138807	0.0345959	1.31753	0	1	1	no

ENSG00000170049	ENSG00000170049	KCNAB3	17:7825176-7853236	q1	q2	OK	5.35265	5.56884	0.0571238	0.0287255	0.9491	0.99995	no

ENSG00000170054	ENSG00000170054	SERPINA9	14:94896969-94946026	q1	q2	NOTEST	0	0	0	0	1	1	no

ENSG00000170074	ENSG00000170074	FAM153A	5:177109294-177221397	q1	q2	NOTEST	0	0.026576	inf	0	1	1	no

ENSG00000170075	ENSG00000170075	GPR37L1	1:202091985-202113869	q1	q2	NOTEST	0.119461	0.101651	-0.232913	0	1	1	no

ENSG00000170085	ENSG00000170085	SIMC1	5:175665364-175772994	q1	q2	OK	16.2366	16.2968	0.00533318	0.0100865	0.9863	0.99995	no

ENSG00000170088	ENSG00000170088	TMEM192	4:165995573-166244308	q1	q2	OK	4.1071	4.14636	0.0137244	0.0140515	0.98245	0.99995	no

ENSG00000170089	ENSG00000170089	RP11-423H2.1	5:177236407-177410055	q1	q2	OK	13.4907	13.8349	0.0363397	0.0620824	0.92465	0.99995	no

ENSG00000170091	ENSG00000170091	NSG2	5:173472606-173670504	q1	q2	OK	6.62467	6.38017	-0.0542536	-0.0776727	0.89125	0.99995	no

ENSG00000170092	ENSG00000170092	AC018720.10	7:75123400-75157478	q1	q2	NOTEST	0.304602	0.239352	-0.347795	0	1	1	no

ENSG00000170099	ENSG00000170099	SERPINA6	14:94770584-94789731	q1	q2	NOTEST	0	0	0	0	1	1	no

ENSG00000170100	ENSG00000170100	ZNF778	16:89284117-89295363	q1	q2	OK	6.17095	6.87248	0.155337	0.134434	0.7328	0.99995	no

ENSG00000170113	ENSG00000170113	NIPA1	15:23043276-23115256	q1	q2	OK	8.97259	9.71311	0.11441	0.178552	0.6959	0.99995	no

ENSG00000170122	ENSG00000170122	FOXD4	9:116236-118417	q1	q2	OK	0.967852	0.873941	-0.14725	-0.165448	0.809	0.99995	no

ENSG00000170128	ENSG00000170128	GPR25	1:200842082-200843306	q1	q2	NOTEST	0.04635	0	#NAME?	0	1	1	no

ENSG00000170142	ENSG00000170142	UBE2E1	3:23845514-23932807	q1	q2	OK	102.893	124.607	0.276232	0.646263	0.32735	0.99995	no

ENSG00000170144	ENSG00000170144	HNRNPA3	2:177855235-178088686	q1	q2	OK	343.689	365.188	0.0875328	0.137498	0.82175	0.99995	no

ENSG00000170145	ENSG00000170145	SIK2	11:111473114-111637151	q1	q2	OK	9.15552	8.82605	-0.0528729	-0.100701	0.886	0.99995	no

ENSG00000170152	ENSG00000170152	RP11-374M1.7	9:44997776-44999352	q1	q2	NOTEST	0	0	0	0	1	1	no

ENSG00000170153	ENSG00000170153	RNF150	4:141780960-142134031	q1	q2	OK	6.94165	7.22358	0.0574359	0.13069	0.847	0.99995	no

ENSG00000170160	ENSG00000170160	CCDC144A	17:16592850-16719854	q1	q2	NOTEST	0.0795686	0.0585447	-0.44266	0	1	1	no

ENSG00000170161	ENSG00000170161	RP11-262H14.4	9:66513031-66555928	q1	q2	OK	2.01182	1.91999	-0.0673973	-0.0440598	0.9233	0.99995	no

ENSG00000170162	ENSG00000170162	VGLL2	6:117586720-117594728	q1	q2	NOTEST	0.0687642	0.0305473	-1.17061	0	1	1	no

ENSG00000170165	ENSG00000170165	CR848007.2	9:44051023-44069881	q1	q2	OK	2.66631	2.49637	-0.0950138	-0.1215	0.8551	0.99995	no

ENSG00000170166	ENSG00000170166	HOXD4	2:176986338-177037830	q1	q2	OK	1.43663	1.52336	0.0845678	0.046009	0.94085	0.99995	no

ENSG00000170175	ENSG00000170175	CHRNB1	17:7323421-7361026	q1	q2	OK	6.46525	4.93391	-0.389973	-0.32959	0.63735	0.99995	no

ENSG00000170178	ENSG00000170178	HOXD12	2:176964457-176966408	q1	q2	NOTEST	0	0	0	0	1	1	no

ENSG00000170180	ENSG00000170180	GYPA	4:144833483-145061904	q1	q2	NOTEST	0.00965	0	#NAME?	0	1	1	no

ENSG00000170185	ENSG00000170185	USP38	4:144106069-144144983	q1	q2	OK	7.89005	8.68052	0.137747	0.298415	0.67075	0.99995	no

ENSG00000170190	ENSG00000170190	SLC16A5	17:73083821-73102257	q1	q2	NOTEST	0.183308	0.0869746	-1.07561	0	1	1	no

ENSG00000170191	ENSG00000170191	NANP	20:25593570-25677477	q1	q2	OK	3.02594	3.4226	0.177707	0.176566	0.8007	0.99995	no

ENSG00000170209	ENSG00000170209	ANKK1	11:113258512-113271140	q1	q2	NOTEST	0.0421621	0.0210596	-1.00147	0	1	1	no

ENSG00000170214	ENSG00000170214	ADRA1B	5:159343789-159399551	q1	q2	NOTEST	0.190222	0.166916	-0.188563	0	1	1	no

ENSG00000170215	ENSG00000170215	FAM27B	9:67792166-67794649	q1	q2	NOTEST	0.0786549	0	#NAME?	0	1	1	no

ENSG00000170217	ENSG00000170217	RP11-12A20.2	9:67784943-67786684	q1	q2	NOTEST	0	0	0	0	1	1	no

ENSG00000170222	ENSG00000170222	ADPRM	17:10583653-10718481	q1	q2	OK	5.22124	5.51693	0.0794714	0.0684377	0.93855	0.99995	no

ENSG00000170231	ENSG00000170231	FABP6	5:159614373-159665742	q1	q2	NOTEST	0.102525	0.100891	-0.0231739	0	1	1	no

ENSG00000170234	ENSG00000170234	PWWP2A	5:159436119-159546430	q1	q2	OK	16.2301	16.3445	0.0101324	0.014115	0.97945	0.99995	no

ENSG00000170236	ENSG00000170236	USP50	15:50716576-50838905	q1	q2	NOTEST	0.124895	0.0911479	-0.454439	0	1	1	no

ENSG00000170242	ENSG00000170242	USP47	11:11862969-11980870	q1	q2	OK	53.6077	51.7985	-0.0495297	-0.0958671	0.87685	0.99995	no

ENSG00000170248	ENSG00000170248	PDCD6IP	3:33839843-33911194	q1	q2	OK	54.6139	59.2026	0.116394	0.16298	0.80265	0.99995	no

ENSG00000170255	ENSG00000170255	MRGPRX1	11:18955359-18961268	q1	q2	NOTEST	0	0	0	0	1	1	no

ENSG00000170260	ENSG00000170260	ZNF212	7:148936741-148952700	q1	q2	OK	10.1152	9.00072	-0.168414	-0.311511	0.62465	0.99995	no

ENSG00000170262	ENSG00000170262	MRAP	21:33664123-33765335	q1	q2	NOTEST	0	0	0	0	1	1	no

ENSG00000170264	ENSG00000170264	FAM161A	2:62051988-62081278	q1	q2	OK	11.5511	11.339	-0.0267424	-0.0371365	0.94865	0.99995	no

ENSG00000170265	ENSG00000170265	ZNF282	7:148892576-148923339	q1	q2	OK	12.6355	9.31524	-0.439815	-0.456157	0.19585	0.99995	no

ENSG00000170266	ENSG00000170266	GLB1	3:33038099-33138722	q1	q2	OK	18.3305	16.879	-0.11902	-0.235334	0.7027	0.99995	no

ENSG00000170270	ENSG00000170270	C14orf142	14:93651295-93695561	q1	q2	OK	13.2534	14.572	0.136831	0.102408	0.8835	0.99995	no

ENSG00000170271	ENSG00000170271	FAXDC2	5:154198050-154256353	q1	q2	OK	4.14707	3.43922	-0.270011	-0.139395	0.7771	0.99995	no

ENSG00000170275	ENSG00000170275	CRTAP	3:33155470-33189265	q1	q2	OK	51.2568	51.5315	0.00770862	0.010772	0.98675	0.99995	no

ENSG00000170276	ENSG00000170276	HSPB2	11:111779288-111797596	q1	q2	NOTEST	0	0	0	0	1	1	no

ENSG00000170279	ENSG00000170279	C7orf33	7:148287656-148312952	q1	q2	NOTEST	0	0	0	0	1	1	no

ENSG00000170289	ENSG00000170289	CNGB3	8:87345502-87777251	q1	q2	NOTEST	0.0114439	0	#NAME?	0	1	1	no

ENSG00000170290	ENSG00000170290	SLN	11:107578103-107595135	q1	q2	NOTEST	0	0.133951	inf	0	1	1	no

ENSG00000170291	ENSG00000170291	ELP5	17:7143332-7167302	q1	q2	OK	43.9936	38.4745	-0.193391	-0.146268	0.7858	0.99995	no

ENSG00000170293	ENSG00000170293	CMTM8	3:32280170-32411817	q1	q2	OK	1.39759	1.62067	0.213651	0.236335	0.7284	0.99995	no

ENSG00000170296	ENSG00000170296	GABARAP	17:7143332-7167302	q1	q2	OK	410.421	385.493	-0.0904006	-0.148898	0.8253	0.99995	no

ENSG00000170298	ENSG00000170298	LGALS9B	17:20352707-20370852	q1	q2	NOTEST	0.0156842	0	#NAME?	0	1	1	no

ENSG00000170310	ENSG00000170310	STX8	17:9153787-9479908	q1	q2	OK	38.1897	38.9711	0.0292223	0.0466798	0.94605	0.99995	no

ENSG00000170312	ENSG00000170312	CDK1	10:62538088-62554610	q1	q2	OK	107.582	116.461	0.114413	0.252148	0.7027	0.99995	no

ENSG00000170315	ENSG00000170315	UBB	17:16284111-16286059	q1	q2	OK	312.986	303.735	-0.0432877	-0.0963924	0.88735	0.99995	no

ENSG00000170322	ENSG00000170322	NFRKB	11:129733530-129765485	q1	q2	OK	16.7017	17.7699	0.089446	0.133141	0.7742	0.99995	no

ENSG00000170323	ENSG00000170323	FABP4	8:82351670-82445510	q1	q2	NOTEST	0	0	0	0	1	1	no

ENSG00000170324	ENSG00000170324	FRMPD2	10:49364600-49482941	q1	q2	NOTEST	0.114802	0.253832	1.14473	0	1	1	no

ENSG00000170325	ENSG00000170325	PRDM10	11:129769600-129872730	q1	q2	OK	6.46016	6.76249	0.0659865	0.141546	0.83605	0.99995	no

ENSG00000170340	ENSG00000170340	B3GNT2	2:62423247-62451866	q1	q2	OK	4.38855	4.33711	-0.0170099	-0.0274266	0.9691	0.99995	no

ENSG00000170345	ENSG00000170345	FOS	14:75745476-75748933	q1	q2	OK	2.49122	2.96228	0.249855	0.286553	0.625	0.99995	no

ENSG00000170348	ENSG00000170348	TMED10	14:75548821-75643334	q1	q2	OK	85.4131	84.2414	-0.0199292	-0.0283349	0.96645	0.99995	no

ENSG00000170356	ENSG00000170356	OR2A20P	7:143883175-144077725	q1	q2	NOTEST	0	0	0	0	1	1	no

ENSG00000170364	ENSG00000170364	SETMAR	3:3742497-4508965	q1	q2	OK	15.187	17.276	0.18593	0.0827367	0.90425	0.99995	no

ENSG00000170365	ENSG00000170365	SMAD1	4:146402345-146479231	q1	q2	OK	20.0019	19.3476	-0.0479853	-0.0879599	0.88295	0.99995	no

ENSG00000170367	ENSG00000170367	CST5	20:23856571-23860387	q1	q2	NOTEST	0	0	0	0	1	1	no

ENSG00000170369	ENSG00000170369	CST2	20:23804405-23807368	q1	q2	NOTEST	0	0	0	0	1	1	no

ENSG00000170370	ENSG00000170370	EMX2	10:119184704-119309056	q1	q2	NOTEST	0.238324	0.356527	0.581088	0	1	1	no

ENSG00000170373	ENSG00000170373	CST1	20:23728189-23731905	q1	q2	NOTEST	0	0.0415732	inf	0	1	1	no

ENSG00000170374	ENSG00000170374	SP7	12:53720361-53739099	q1	q2	NOTEST	0.0428157	0	#NAME?	0	1	1	no

ENSG00000170379	ENSG00000170379	FAM115C	7:143318042-143534026	q1	q2	NOTEST	0.0407911	0.065549	0.684318	0	1	1	no

ENSG00000170381	ENSG00000170381	SEMA3E	7:82993221-83278326	q1	q2	NOTEST	0.337237	0.258471	-0.38376	0	1	1	no

ENSG00000170382	ENSG00000170382	LRRN2	1:204586297-204654861	q1	q2	OK	5.04119	3.17777	-0.665749	-0.826263	0.13575	0.99995	no

ENSG00000170385	ENSG00000170385	SLC30A1	1:211744909-211752084	q1	q2	OK	7.15375	7.69066	0.104407	0.22643	0.75065	0.99995	no

ENSG00000170390	ENSG00000170390	DCLK2	4:150999425-151178609	q1	q2	OK	29.7148	30.0366	0.0155429	0.037704	0.95635	0.99995	no

ENSG00000170396	ENSG00000170396	ZNF804A	2:185463092-185804219	q1	q2	NOTEST	0.233801	0.261339	0.160638	0	1	1	no

ENSG00000170409	ENSG00000170409	CTA-313A17.2	7:101967673-101969945	q1	q2	NOTEST	0	0	0	0	1	1	no

ENSG00000170412	ENSG00000170412	GPRC5C	17:72420989-72447792	q1	q2	OK	5.97596	4.69385	-0.348397	-0.46432	0.50145	0.99995	no

ENSG00000170417	ENSG00000170417	TMEM182	2:103353366-103460352	q1	q2	OK	2.51706	3.053	0.278486	0.289983	0.66005	0.99995	no

ENSG00000170419	ENSG00000170419	VSTM2A	7:54610017-54639419	q1	q2	OK	1.71513	1.41613	-0.276359	-0.256516	0.6356	0.99995	no

ENSG00000170421	ENSG00000170421	KRT8	12:53290976-53346686	q1	q2	OK	0.830959	0.402034	-1.04746	-0.414308	0.16305	0.99995	no

ENSG00000170423	ENSG00000170423	KRT78	12:53231587-53242876	q1	q2	NOTEST	0	0	0	0	1	1	no

ENSG00000170425	ENSG00000170425	ADORA2B	17:15848230-15879060	q1	q2	OK	3.01265	2.49319	-0.273039	-0.333654	0.64235	0.99995	no

ENSG00000170426	ENSG00000170426	SDR9C7	12:57316937-57328189	q1	q2	NOTEST	0.107802	0.07465	-0.530166	0	1	1	no

ENSG00000170430	ENSG00000170430	MGMT	10:131265447-131566271	q1	q2	OK	1.1245	0.831828	-0.434923	-0.413348	0.5008	0.99995	no

ENSG00000170439	ENSG00000170439	METTL7B	12:56075329-56118489	q1	q2	NOTEST	0.185702	0.0554016	-1.74499	0	1	1	no

ENSG00000170442	ENSG00000170442	KRT86	12:52626303-52702947	q1	q2	NOTEST	0	0	0	0	1	1	no

ENSG00000170445	ENSG00000170445	HARS	5:140044260-140086248	q1	q2	OK	51.0469	58.4236	0.194728	0.229667	0.71775	0.99995	no

ENSG00000170448	ENSG00000170448	NFXL1	4:47849256-48042188	q1	q2	OK	8.04714	8.8451	0.136402	0.154039	0.77035	0.99995	no

ENSG00000170454	ENSG00000170454	KRT75	12:52801363-52828309	q1	q2	NOTEST	0	0	0	0	1	1	no

ENSG00000170456	ENSG00000170456	DENND5B	12:31535156-31768600	q1	q2	OK	14.2956	14.9375	0.0633626	0.109192	0.8467	0.99995	no

ENSG00000170458	ENSG00000170458	CD14	5:140011312-140013286	q1	q2	NOTEST	0.276984	0.329541	0.250658	0	1	1	no

ENSG00000170464	ENSG00000170464	DNAJC18	5:138744278-138780180	q1	q2	OK	15.4474	14.3365	-0.107673	-0.121485	0.8056	0.99995	no

ENSG00000170465	ENSG00000170465	KRT6C	12:52862299-52867569	q1	q2	NOTEST	0	0	0	0	1	1	no

ENSG00000170468	ENSG00000170468	AC005280.1	14:73945188-74025651	q1	q2	OK	3.41482	5.18619	0.602866	0.573931	0.37135	0.99995	no

ENSG00000170469	ENSG00000170469	SPATA24	5:138732251-138739777	q1	q2	OK	6.0455	6.12498	0.0188437	0.0220519	0.97545	0.99995	no

ENSG00000170471	ENSG00000170471	RALGAPB	20:37101458-37207504	q1	q2	OK	18.8267	19.6291	0.0602148	0.105396	0.84675	0.99995	no

ENSG00000170473	ENSG00000170473	WIBG	12:56295196-56347811	q1	q2	OK	28.3479	27.3035	-0.0541554	-0.0871081	0.9032	0.99995	no

ENSG00000170476	ENSG00000170476	MZB1	5:138723168-138725770	q1	q2	NOTEST	0.0424302	0.395686	3.22119	0	1	1	no

ENSG00000170477	ENSG00000170477	KRT4	12:53200332-53208335	q1	q2	NOTEST	0	0	0	0	1	1	no

ENSG00000170482	ENSG00000170482	SLC23A1	5:138677275-138720242	q1	q2	NOTEST	0.408199	0.360708	-0.178442	0	1	1	no

ENSG00000170484	ENSG00000170484	KRT74	12:52959565-52967609	q1	q2	NOTEST	0	0	0	0	1	1	no

ENSG00000170485	ENSG00000170485	NPAS2	2:101436613-101886778	q1	q2	OK	1.58509	2.20749	0.477847	0.0900978	0.6696	0.99995	no

ENSG00000170486	ENSG00000170486	KRT72	12:52979372-53012343	q1	q2	NOTEST	0	0	0	0	1	1	no

ENSG00000170498	ENSG00000170498	KISS1	1:204159468-204165614	q1	q2	NOTEST	0	0.0482141	inf	0	1	1	no

ENSG00000170500	ENSG00000170500	LONRF2	2:100889752-100939195	q1	q2	OK	1.54708	2.00082	0.37104	0.718783	0.29195	0.99995	no

ENSG00000170502	ENSG00000170502	NUDT9	4:88343733-88380606	q1	q2	OK	21.0045	23.2604	0.147182	0.269649	0.67515	0.99995	no

ENSG00000170509	ENSG00000170509	HSD17B13	4:88224940-88312538	q1	q2	NOTEST	0.139947	0.160095	0.194045	0	1	1	no

ENSG00000170515	ENSG00000170515	PA2G4	12:56473640-56507691	q1	q2	OK	76.259	88.6104	0.216569	0.437998	0.4857	0.99995	no

ENSG00000170516	ENSG00000170516	COX7B2	4:46736843-46911252	q1	q2	NOTEST	0	0.0843416	inf	0	1	1	no

ENSG00000170522	ENSG00000170522	ELOVL6	4:110967001-111120355	q1	q2	OK	45.1893	54.5616	0.271905	0.476789	0.36485	0.99995	no

ENSG00000170523	ENSG00000170523	KRT83	12:52708084-52715182	q1	q2	NOTEST	0	0	0	0	1	1	no

ENSG00000170525	ENSG00000170525	PFKFB3	10:6186880-6277495	q1	q2	OK	18.4056	19.412	0.0768092	0.157154	0.79405	0.99995	no

ENSG00000170537	ENSG00000170537	TMC7	16:18995255-19091417	q1	q2	OK	1.17343	1.27852	0.123735	0.0893665	0.89585	0.99995	no

ENSG00000170540	ENSG00000170540	ARL6IP1	16:18792616-18813000	q1	q2	OK	210.854	220.879	0.0670166	0.103587	0.8787	0.99995	no

ENSG00000170542	ENSG00000170542	SERPINB9	6:2887499-2903514	q1	q2	OK	0.39611	0.611617	0.626729	0.681724	0.33595	0.99995	no

ENSG00000170545	ENSG00000170545	SMAGP	12:51632075-51665146	q1	q2	OK	3.3899	4.10706	0.276862	0.0860012	0.8005	0.99995	no

ENSG00000170549	ENSG00000170549	IRX1	5:3596167-3601517	q1	q2	OK	22.4667	19.5126	-0.203385	-0.434831	0.53565	0.99995	no

ENSG00000170558	ENSG00000170558	CDH2	18:25530929-25757410	q1	q2	OK	151.013	141.321	-0.0957001	-0.198289	0.76455	0.99995	no

ENSG00000170561	ENSG00000170561	IRX2	5:2745958-2755508	q1	q2	OK	54.4109	47.1192	-0.20758	-0.466429	0.49005	0.99995	no

ENSG00000170571	ENSG00000170571	EMB	5:49692025-49739082	q1	q2	OK	17.5086	19.967	0.189555	0.430359	0.5339	0.99995	no

ENSG00000170577	ENSG00000170577	SIX2	2:45232299-45236569	q1	q2	OK	2.46233	2.86748	0.219756	0.293374	0.6807	0.99995	no

ENSG00000170579	ENSG00000170579	DLGAP1	18:3496029-4455335	q1	q2	OK	3.45084	2.21737	-0.638097	-0.383857	0.2631	0.99995	no

ENSG00000170581	ENSG00000170581	STAT2	12:56735380-56753939	q1	q2	OK	32.21	29.8482	-0.109865	-0.193944	0.7436	0.99995	no

ENSG00000170584	ENSG00000170584	NUDCD2	5:162864574-162887146	q1	q2	OK	22.1037	22.6315	0.0340502	0.0151757	0.968	0.99995	no

ENSG00000170590	ENSG00000170590	LINC00266-3	6:131909-145083	q1	q2	NOTEST	0	0	0	0	1	1	no

ENSG00000170604	ENSG00000170604	IRF2BP1	19:46386865-46389376	q1	q2	OK	14.0822	11.1935	-0.33121	-0.681777	0.32085	0.99995	no

ENSG00000170605	ENSG00000170605	OR9K2	12:55523464-55524586	q1	q2	NOTEST	0	0	0	0	1	1	no

ENSG00000170606	ENSG00000170606	HSPA4	5:132387653-132442141	q1	q2	OK	79.6364	88.328	0.149443	0.273353	0.62655	0.99995	no

ENSG00000170608	ENSG00000170608	FOXA3	19:46367246-46377055	q1	q2	NOTEST	0.0132643	0.0684019	2.36649	0	1	1	no

ENSG00000170613	ENSG00000170613	FAM71B	5:156512842-156682201	q1	q2	NOTEST	0	0	0	0	1	1	no

ENSG00000170615	ENSG00000170615	SLC26A5	7:102937868-103629963	q1	q2	NOTEST	0.0405601	0	#NAME?	0	1	1	no

ENSG00000170616	ENSG00000170616	SCRT1	8:145554227-145559943	q1	q2	NOTEST	0.128515	0.167201	0.379646	0	1	1	no

ENSG00000170619	ENSG00000170619	COMMD5	8:146052848-146127553	q1	q2	OK	16.7273	15.0149	-0.15581	-0.17137	0.81175	0.99995	no

ENSG00000170624	ENSG00000170624	SGCD	5:155297353-156194799	q1	q2	OK	2.42879	2.90246	0.257038	0.301784	0.5695	0.99995	no

ENSG00000170627	ENSG00000170627	GTSF1	12:54747444-54891472	q1	q2	NOTEST	0	0	0	0	1	1	no

ENSG00000170629	ENSG00000170629	DPY19L2P2	7:102815579-102920857	q1	q2	OK	2.75935	2.89646	0.069962	0.105985	0.87985	0.99995	no

ENSG00000170631	ENSG00000170631	ZNF16	8:146155743-146176274	q1	q2	OK	5.32098	5.04492	-0.0768611	-0.126046	0.847	0.99995	no

ENSG00000170632	ENSG00000170632	ARMC10	7:102715327-102740205	q1	q2	OK	13.0458	15.6211	0.259913	0.46999	0.4788	0.99995	no

ENSG00000170633	ENSG00000170633	RNF34	12:121837843-122031409	q1	q2	OK	0.538164	0.840176	0.642647	0.266299	0.5174	0.99995	no

ENSG00000170634	ENSG00000170634	ACYP2	2:54091203-54532437	q1	q2	OK	12.2928	11.6056	-0.0829877	-0.0441912	0.9394	0.99995	no

ENSG00000170638	ENSG00000170638	TRABD	22:50624343-50638976	q1	q2	OK	16.367	14.5864	-0.166167	-0.339887	0.62605	0.99995	no

ENSG00000170647	ENSG00000170647	TMEM133	11:100862810-100864663	q1	q2	OK	4.76117	5.48192	0.203364	0.311458	0.66115	0.99995	no

ENSG00000170653	ENSG00000170653	ATF7	12:53900471-54020199	q1	q2	OK	11.9133	13.3686	0.16628	0.193	0.70665	0.99995	no

ENSG00000170667	ENSG00000170667	RASA4B	7:102122891-102312088	q1	q2	OK	1.81641	1.4991	-0.276988	-0.175136	0.83655	0.99995	no

ENSG00000170677	ENSG00000170677	SOCS6	18:67956136-67997436	q1	q2	OK	11.7083	12.1255	0.0505021	0.106635	0.8775	0.99995	no

ENSG00000170681	ENSG00000170681	MURC	9:103340360-103350188	q1	q2	OK	6.70086	7.18249	0.100139	0.179924	0.7976	0.99995	no

ENSG00000170683	ENSG00000170683	OR10A3	11:7960080-7961141	q1	q2	NOTEST	0	0	0	0	1	1	no

ENSG00000170684	ENSG00000170684	ZNF296	19:45574757-45808541	q1	q2	NOTEST	0.410545	0.368626	-0.155382	0	1	1	no

ENSG00000170688	ENSG00000170688	OR5E1P	11:7710668-7927502	q1	q2	NOTEST	0	0.190771	inf	0	1	1	no

ENSG00000170689	ENSG00000170689	HOXB9	17:46684593-46712294	q1	q2	OK	0.308031	0.535511	0.797841	0.532054	0.58075	0.99995	no

ENSG00000170703	ENSG00000170703	TTLL6	17:46839596-46894576	q1	q2	NOTEST	0.325769	0.0963613	-1.75733	0	1	1	no

ENSG00000170727	ENSG00000170727	BOP1	8:145486054-145515082	q1	q2	OK	4.24697	5.13227	0.273163	0.294439	0.6475	0.99995	no

ENSG00000170734	ENSG00000170734	POLH	6:43543886-43596899	q1	q2	OK	6.15278	5.6322	-0.127542	-0.138126	0.832	0.99995	no

ENSG00000170743	ENSG00000170743	SYT9	11:7260008-7490273	q1	q2	OK	0.925541	0.902048	-0.0370925	-0.0387594	0.9532	0.99995	no

ENSG00000170745	ENSG00000170745	KCNS3	2:18059113-18542882	q1	q2	OK	6.79073	6.22367	-0.125802	-0.209159	0.74605	0.99995	no

ENSG00000170748	ENSG00000170748	RBMXL2	11:7110164-7112379	q1	q2	NOTEST	0.0117765	0	#NAME?	0	1	1	no

ENSG00000170759	ENSG00000170759	KIF5B	10:32297937-32345359	q1	q2	OK	70.3908	81.2555	0.207079	0.475068	0.47535	0.99995	no

ENSG00000170775	ENSG00000170775	GPR37	7:124386050-124405681	q1	q2	OK	0.690707	0.635088	-0.121116	-0.136734	0.84065	0.99995	no

ENSG00000170776	ENSG00000170776	AKAP13	15:85923801-86293512	q1	q2	OK	26.0211	28.2777	0.11998	0.152016	0.6913	0.99995	no

ENSG00000170777	ENSG00000170777	TPD52L3	9:6328348-6331900	q1	q2	NOTEST	0.010004	0	#NAME?	0	1	1	no

ENSG00000170779	ENSG00000170779	CDCA4	14:105452111-105487485	q1	q2	OK	21.9531	22.1266	0.0113549	0.0227122	0.9738	0.99995	no

ENSG00000170782	ENSG00000170782	OR10A4	11:6897855-6898850	q1	q2	NOTEST	0	0	0	0	1	1	no

ENSG00000170786	ENSG00000170786	SDR16C5	8:57212568-57233335	q1	q2	NOTEST	0	0	0	0	1	1	no

ENSG00000170788	ENSG00000170788	DYDC1	10:82095860-82127829	q1	q2	NOTEST	0	0.059464	inf	0	1	1	no

ENSG00000170790	ENSG00000170790	OR10A2	11:6890923-6891976	q1	q2	NOTEST	0.0278216	0.225674	3.01997	0	1	1	no

ENSG00000170791	ENSG00000170791	CHCHD7	8:57124244-57131357	q1	q2	OK	26.9828	27.5741	0.0312751	0.0533337	0.93215	0.99995	no

ENSG00000170801	ENSG00000170801	HTRA3	4:8271491-8308838	q1	q2	NOTEST	0.309582	0.223411	-0.470617	0	1	1	no

ENSG00000170802	ENSG00000170802	FOXN2	2:48541775-48606433	q1	q2	OK	7.55052	8.64783	0.195762	0.328572	0.57615	0.99995	no

ENSG00000170803	ENSG00000170803	OR2AG1	11:6806247-6807245	q1	q2	NOTEST	0.0297394	0	#NAME?	0	1	1	no

ENSG00000170807	ENSG00000170807	LMOD2	7:123295860-123304344	q1	q2	NOTEST	0.0110576	0	#NAME?	0	1	1	no

ENSG00000170819	ENSG00000170819	BFSP2	3:133118838-133194066	q1	q2	NOTEST	0	0	0	0	1	1	no

ENSG00000170820	ENSG00000170820	FSHR	2:49189295-49381676	q1	q2	NOTEST	0	0	0	0	1	1	no

ENSG00000170827	ENSG00000170827	CELP	9:135957925-135962742	q1	q2	NOTEST	0	0.0440574	inf	0	1	1	no

ENSG00000170832	ENSG00000170832	USP32	17:58256454-58508782	q1	q2	OK	14.3994	14.6087	0.0208207	0.0335975	0.94605	0.99995	no

ENSG00000170835	ENSG00000170835	CEL	9:135937364-135947248	q1	q2	OK	1.36833	1.11405	-0.2966	-0.337771	0.62885	0.99995	no

ENSG00000170836	ENSG00000170836	PPM1D	17:58677543-58741849	q1	q2	OK	19.0576	17.6941	-0.107097	-0.239639	0.72395	0.99995	no

ENSG00000170837	ENSG00000170837	GPR27	3:71724566-71805647	q1	q2	OK	3.92971	3.92608	-0.00133002	-0.00145207	0.99765	0.99995	no

ENSG00000170846	ENSG00000170846	AC093323.3	4:6672451-6677774	q1	q2	OK	11.3099	11.2856	-0.00309859	-0.00603292	0.9939	0.99995	no

ENSG00000170848	ENSG00000170848	PSG6	19:43406230-43423715	q1	q2	NOTEST	0	0	0	0	1	1	no

ENSG00000170852	ENSG00000170852	KBTBD2	7:32535037-33102409	q1	q2	OK	35.4485	38.8312	0.13149	0.163916	0.7626	0.99995	no

ENSG00000170854	ENSG00000170854	MINA	3:97595818-97691301	q1	q2	OK	6.5743	8.28013	0.332816	0.331526	0.5998	0.99995	no

ENSG00000170855	ENSG00000170855	TRIAP1	12:120875892-120899389	q1	q2	OK	44.1097	41.4492	-0.0897528	-0.108138	0.88045	0.99995	no

ENSG00000170858	ENSG00000170858	LILRP2	19:55219699-55224955	q1	q2	NOTEST	0	0	0	0	1	1	no

ENSG00000170860	ENSG00000170860	LSM3	3:14166439-14242619	q1	q2	OK	15.2956	17.1304	0.163442	0.196257	0.7798	0.99995	no

ENSG00000170866	ENSG00000170866	LILRA3	19:54799853-54809952	q1	q2	NOTEST	0	0	0	0	1	1	no

ENSG00000170871	ENSG00000170871	KIAA0232	4:6783101-6885897	q1	q2	OK	19.8513	21.2149	0.0958418	0.230763	0.7248	0.99995	no

ENSG00000170873	ENSG00000170873	MTSS1	8:125500725-125740730	q1	q2	OK	16.0364	18.3124	0.191473	0.212495	0.65535	0.99995	no

ENSG00000170876	ENSG00000170876	TMEM43	3:14166439-14242619	q1	q2	OK	30.1941	30.4389	0.0116508	0.0178021	0.97695	0.99995	no

ENSG00000170881	ENSG00000170881	RNF139	8:125486978-125500155	q1	q2	OK	16.9692	17.3206	0.0295656	0.0656222	0.92545	0.99995	no

ENSG00000170889	ENSG00000170889	RPS9	19:54704609-54752862	q1	q2	NOTEST	0.0217303	0.0331244	0.608186	0	1	1	no

ENSG00000170890	ENSG00000170890	PLA2G1B	12:120759913-120765592	q1	q2	NOTEST	0.0654787	0.105678	0.690576	0	1	1	no

ENSG00000170891	ENSG00000170891	CYTL1	4:5016312-5021199	q1	q2	OK	2.75378	2.66904	-0.0450962	-0.0451528	0.94455	0.99995	no

ENSG00000170892	ENSG00000170892	TSEN34	19:54693788-54697585	q1	q2	NOTEST	0.0217928	0.0200934	-0.117133	0	1	1	no

ENSG00000170893	ENSG00000170893	TRH	3:129693147-129696781	q1	q2	OK	0.810306	0.882242	0.122707	0.130215	0.85865	0.99995	no

ENSG00000170899	ENSG00000170899	GSTA4	6:52842750-52860176	q1	q2	OK	61.3832	65.1539	0.086008	0.203341	0.7712	0.99995	no

ENSG00000170903	ENSG00000170903	MSANTD4	11:105866349-105893130	q1	q2	OK	18.5062	19.7663	0.0950325	0.165871	0.7807	0.99995	no

ENSG00000170906	ENSG00000170906	NDUFA3	19:54606035-54635140	q1	q2	NOTEST	0	0	0	0	1	1	no

ENSG00000170909	ENSG00000170909	OSCAR	19:54597932-54606000	q1	q2	NOTEST	0	0	0	0	1	1	no

ENSG00000170915	ENSG00000170915	PAQR8	6:52226218-52272575	q1	q2	OK	7.25883	7.29858	0.0078806	0.0167277	0.98085	0.99995	no

ENSG00000170917	ENSG00000170917	NUDT6	4:123747862-123844123	q1	q2	OK	6.5562	6.82727	0.0584494	0.0436247	0.94915	0.99995	no

ENSG00000170919	ENSG00000170919	TPT1-AS1	13:45911007-45965872	q1	q2	OK	17.6667	16.2564	-0.120022	-0.0236316	0.819	0.99995	no

ENSG00000170920	ENSG00000170920	OR7G3	19:9236687-9237626	q1	q2	NOTEST	0	0	0	0	1	1	no

ENSG00000170921	ENSG00000170921	TANC2	17:61086916-61509572	q1	q2	OK	17.0432	14.4991	-0.233237	-0.137933	0.55955	0.99995	no

ENSG00000170923	ENSG00000170923	OR7G2	19:9212944-9213982	q1	q2	NOTEST	0	0.0276345	inf	0	1	1	no

ENSG00000170925	ENSG00000170925	TEX13B	X:107224093-107225600	q1	q2	NOTEST	0	0	0	0	1	1	no

ENSG00000170927	ENSG00000170927	PKHD1	6:51464520-51952423	q1	q2	NOTEST	0	0	0	0	1	1	no

ENSG00000170929	ENSG00000170929	OR1M1	19:9203854-9204889	q1	q2	NOTEST	0	0	0	0	1	1	no

ENSG00000170935	ENSG00000170935	NCBP2L	X:107020962-107037912	q1	q2	NOTEST	0	0	0	0	1	1	no

ENSG00000170946	ENSG00000170946	DNAJC24	11:31391386-31453396	q1	q2	OK	18.2208	19.3936	0.089998	0.138537	0.8125	0.99995	no

ENSG00000170948	ENSG00000170948	MBD3L1	19:8943073-8954016	q1	q2	NOTEST	0	0	0	0	1	1	no

ENSG00000170949	ENSG00000170949	ZNF160	19:53569858-53606689	q1	q2	OK	12.897	14.0895	0.127588	0.209882	0.68235	0.99995	no

ENSG00000170950	ENSG00000170950	PGK2	6:49753365-49755053	q1	q2	NOTEST	0	0	0	0	1	1	no

ENSG00000170953	ENSG00000170953	OR8B12	11:124412577-124413575	q1	q2	NOTEST	0	0	0	0	1	1	no

ENSG00000170954	ENSG00000170954	ZNF415	19:53611131-53662328	q1	q2	OK	6.43383	6.74664	0.0684915	0.0685847	0.8836	0.99995	no

ENSG00000170955	ENSG00000170955	PRKCDBP	11:6340175-6341877	q1	q2	OK	1.07806	0.718453	-0.585477	-0.475601	0.43745	0.99995	no

ENSG00000170956	ENSG00000170956	CEACAM3	19:42300368-42315591	q1	q2	NOTEST	0	0	0	0	1	1	no

ENSG00000170959	ENSG00000170959	DCDC1	11:30851915-31391357	q1	q2	OK	1.09442	0.64286	-0.767594	-0.602872	0.2654	0.99995	no

ENSG00000170961	ENSG00000170961	HAS2	8:122624355-122656933	q1	q2	OK	7.35324	9.23756	0.329132	0.599373	0.3783	0.99995	no

ENSG00000170962	ENSG00000170962	PDGFD	11:103777913-104035107	q1	q2	OK	3.8114	4.49761	0.238838	0.420171	0.549	0.99995	no

ENSG00000170965	ENSG00000170965	PLAC1	X:133699867-133898352	q1	q2	NOTEST	0.155533	0.162707	0.0650612	0	1	1	no

ENSG00000170967	ENSG00000170967	DDI1	11:103777913-104035107	q1	q2	NOTEST	0	0.00952276	inf	0	1	1	no

ENSG00000170983	ENSG00000170983	LINC00208	8:11433821-11438851	q1	q2	NOTEST	0	0	0	0	1	1	no

ENSG00000170989	ENSG00000170989	S1PR1	1:101702443-101707074	q1	q2	OK	5.85911	3.8248	-0.615295	-0.92883	0.1468	0.99995	no

ENSG00000171004	ENSG00000171004	HS6ST2	X:131760043-132095423	q1	q2	OK	9.75007	10.6626	0.12908	0.274909	0.6823	0.99995	no

ENSG00000171014	ENSG00000171014	OR4D5	11:123810249-123811344	q1	q2	NOTEST	0	0	0	0	1	1	no

ENSG00000171016	ENSG00000171016	PYGO1	15:55831087-55881145	q1	q2	OK	7.34916	8.50172	0.210176	0.416874	0.54535	0.99995	no

ENSG00000171017	ENSG00000171017	LRRC8E	19:7953389-7968427	q1	q2	NOTEST	0.0762635	0.0696295	-0.131295	0	1	1	no

ENSG00000171033	ENSG00000171033	PKIA	8:79338337-79517502	q1	q2	OK	46.0867	48.1214	0.0623276	0.137649	0.84435	0.99995	no

ENSG00000171044	ENSG00000171044	XKR6	8:10753554-11058875	q1	q2	OK	4.95524	4.14598	-0.257242	-0.164156	0.88805	0.99995	no

ENSG00000171045	ENSG00000171045	TSNARE1	8:143293440-143484601	q1	q2	OK	5.18009	4.72516	-0.132613	-0.179567	0.7795	0.99995	no

ENSG00000171049	ENSG00000171049	FPR2	19:52248424-52329442	q1	q2	NOTEST	0	0.010191	inf	0	1	1	no

ENSG00000171051	ENSG00000171051	FPR1	19:52248424-52329442	q1	q2	NOTEST	0	0.12893	inf	0	1	1	no

ENSG00000171053	ENSG00000171053	PATE1	11:125616187-125619762	q1	q2	NOTEST	0.0178828	0	#NAME?	0	1	1	no

ENSG00000171054	ENSG00000171054	OR13H1	X:130677949-130679030	q1	q2	NOTEST	0	0	0	0	1	1	no

ENSG00000171055	ENSG00000171055	FEZ2	2:36583068-36873230	q1	q2	OK	37.3264	40.5492	0.119475	0.204754	0.75745	0.99995	no

ENSG00000171056	ENSG00000171056	SOX7	8:10581277-10697394	q1	q2	OK	4.44197	5.66314	0.350402	0.420377	0.57825	0.99995	no

ENSG00000171060	ENSG00000171060	C8orf74	8:10463858-10569697	q1	q2	NOTEST	0	0	0	0	1	1	no

ENSG00000171067	ENSG00000171067	C11orf24	11:68028802-68039469	q1	q2	OK	17.3988	16.6312	-0.0650991	-0.111304	0.85525	0.99995	no

ENSG00000171084	ENSG00000171084	FAM86JP	3:125635462-125655882	q1	q2	OK	0.474008	0.558333	0.236212	0.22603	0.7349	0.99995	no

ENSG00000171094	ENSG00000171094	ALK	2:29415639-30144432	q1	q2	OK	2.93069	2.84983	-0.040363	-0.0656987	0.91535	0.99995	no

ENSG00000171097	ENSG00000171097	CCBL1	9:131595220-131680318	q1	q2	OK	5.98153	4.62443	-0.371239	-0.138023	0.73695	0.99995	no

ENSG00000171100	ENSG00000171100	MTM1	X:149737068-149841795	q1	q2	OK	1.363	0.611788	-1.15568	-0.852404	0.1488	0.99995	no

ENSG00000171101	ENSG00000171101	SIGLEC17P	19:51645555-51685222	q1	q2	NOTEST	0	0	0	0	1	1	no

ENSG00000171102	ENSG00000171102	OBP2B	9:136080663-136084630	q1	q2	NOTEST	0	0	0	0	1	1	no

ENSG00000171103	ENSG00000171103	TRMT61B	2:28974505-29093167	q1	q2	OK	11.7919	14.2042	0.268521	0.125239	0.7712	0.99995	no

ENSG00000171105	ENSG00000171105	INSR	19:7112265-7294045	q1	q2	OK	5.13536	4.49821	-0.191116	-0.28706	0.54805	0.99995	no

ENSG00000171109	ENSG00000171109	MFN1	3:179065479-179112719	q1	q2	OK	23.0437	25.1091	0.123833	0.14814	0.72055	0.99995	no

ENSG00000171115	ENSG00000171115	GIMAP8	7:150147717-150176480	q1	q2	NOTEST	0.0119121	0.0230564	0.952745	0	1	1	no

ENSG00000171116	ENSG00000171116	HSFX1	X:148855725-148858525	q1	q2	NOTEST	0	0	0	0	1	1	no

ENSG00000171119	ENSG00000171119	NRTN	19:5823812-5828335	q1	q2	OK	0.646929	0.448966	-0.527001	-0.474286	0.501	0.99995	no

ENSG00000171121	ENSG00000171121	KCNMB3	3:178865901-178984790	q1	q2	OK	0.65788	0.613082	-0.101743	-0.0258282	0.9277	0.99995	no

ENSG00000171124	ENSG00000171124	FUT3	19:5842898-5858250	q1	q2	NOTEST	0.0621017	0	#NAME?	0	1	1	no

ENSG00000171126	ENSG00000171126	KCNG3	2:42669156-42721237	q1	q2	NOTEST	0.0209665	0.0332337	0.664559	0	1	1	no

ENSG00000171129	ENSG00000171129	HSFX2	X:148674171-148676974	q1	q2	NOTEST	0	0	0	0	1	1	no

ENSG00000171130	ENSG00000171130	ATP6V0E2	7:149564785-149577784	q1	q2	OK	35.4447	30.1457	-0.233619	-0.460683	0.4768	0.99995	no

ENSG00000171132	ENSG00000171132	PRKCE	2:45878483-46415129	q1	q2	OK	3.18951	3.38036	0.0838421	0.10257	0.8123	0.99995	no

ENSG00000171133	ENSG00000171133	OR2K2	9:114089762-114090800	q1	q2	NOTEST	0	0	0	0	1	1	no

ENSG00000171135	ENSG00000171135	JAGN1	3:9932237-9936033	q1	q2	OK	23.8578	20.7608	-0.200601	-0.40146	0.5373	0.99995	no

ENSG00000171136	ENSG00000171136	RLN3	19:14138959-14142369	q1	q2	NOTEST	0	0	0	0	1	1	no

ENSG00000171148	ENSG00000171148	TADA3	3:9791627-9896822	q1	q2	OK	53.3871	47.8249	-0.158731	-0.219593	0.74975	0.99995	no

ENSG00000171150	ENSG00000171150	SOCS5	2:46926090-46990268	q1	q2	OK	8.823	8.96774	0.0234751	0.0508497	0.9412	0.99995	no

ENSG00000171155	ENSG00000171155	C1GALT1C1	X:119759647-119764005	q1	q2	OK	12.4718	13.6301	0.128128	0.236962	0.73295	0.99995	no

ENSG00000171159	ENSG00000171159	C9orf16	9:130922538-130926207	q1	q2	OK	70.2046	60.4701	-0.215343	-0.449586	0.5216	0.99995	no

ENSG00000171160	ENSG00000171160	MORN4	10:99344079-99436191	q1	q2	OK	15.2071	12.5327	-0.279051	-0.239461	0.64315	0.99995	no

ENSG00000171161	ENSG00000171161	ZNF672	1:249132408-249143716	q1	q2	OK	14.1949	12.6972	-0.160861	-0.321234	0.60935	0.99995	no

ENSG00000171163	ENSG00000171163	ZNF692	1:249144204-249153343	q1	q2	OK	32.1347	28.3969	-0.178403	-0.320471	0.6305	0.99995	no

ENSG00000171169	ENSG00000171169	NAIF1	9:130823511-130871604	q1	q2	OK	6.46816	5.82547	-0.150981	-0.137175	0.84535	0.99995	no

ENSG00000171174	ENSG00000171174	RBKS	2:27994583-28561768	q1	q2	OK	0.680576	0.748263	0.136789	0.0416121	0.9126	0.99995	no

ENSG00000171180	ENSG00000171180	OR2M4	1:248402230-248403166	q1	q2	NOTEST	0.0322451	0	#NAME?	0	1	1	no

ENSG00000171189	ENSG00000171189	GRIK1	21:30565800-31312351	q1	q2	OK	0.51876	0.611513	0.237317	0.078826	0.8342	0.99995	no

ENSG00000171195	ENSG00000171195	MUC7	4:71296208-71348714	q1	q2	NOTEST	0	0	0	0	1	1	no

ENSG00000171199	ENSG00000171199	PROL1	4:71263598-71275911	q1	q2	NOTEST	0	0	0	0	1	1	no

ENSG00000171201	ENSG00000171201	SMR3B	4:71235809-71255961	q1	q2	NOTEST	0	0	0	0	1	1	no

ENSG00000171202	ENSG00000171202	TMEM126A	11:85359010-85367591	q1	q2	OK	33.6331	38.7839	0.205578	0.384527	0.5954	0.99995	no

ENSG00000171204	ENSG00000171204	TMEM126B	11:85339628-85347580	q1	q2	OK	37.7971	44.179	0.225083	0.44959	0.51105	0.99995	no

ENSG00000171206	ENSG00000171206	TRIM8	10:104404252-104418164	q1	q2	OK	22.5329	21.1573	-0.0908807	-0.175984	0.78575	0.99995	no

ENSG00000171208	ENSG00000171208	NETO2	16:47111613-47177908	q1	q2	OK	62.9176	60.3787	-0.0594242	-0.11325	0.85785	0.99995	no

ENSG00000171209	ENSG00000171209	CSN3	4:71108304-71117145	q1	q2	NOTEST	0	0	0	0	1	1	no

ENSG00000171217	ENSG00000171217	CLDN20	6:155054458-155635627	q1	q2	OK	0.661119	0.425842	-0.634591	-0.0829592	0.7469	0.99995	no

ENSG00000171219	ENSG00000171219	CDC42BPG	11:64590858-64612041	q1	q2	NOTEST	0.2482	0.435393	0.810813	0	1	1	no

ENSG00000171222	ENSG00000171222	SCAND1	20:34541538-34547394	q1	q2	OK	54.5142	44.5144	-0.292361	-0.613465	0.3815	0.99995	no

ENSG00000171223	ENSG00000171223	JUNB	19:12873816-12985765	q1	q2	OK	6.22755	6.24281	0.00353198	0.00157607	0.99105	0.99995	no

ENSG00000171224	ENSG00000171224	C10orf35	10:71390006-71393352	q1	q2	OK	19.803	19.7758	-0.0019879	-0.00355455	0.99585	0.99995	no

ENSG00000171227	ENSG00000171227	TMEM37	2:120187476-120196096	q1	q2	OK	3.31245	4.36237	0.397212	0.515138	0.4285	0.99995	no

ENSG00000171234	ENSG00000171234	UGT2B7	4:69917080-69978705	q1	q2	NOTEST	0	0.0945084	inf	0	1	1	no

ENSG00000171236	ENSG00000171236	LRG1	19:4522542-4540486	q1	q2	NOTEST	0.00882068	0.0255079	1.53198	0	1	1	no

ENSG00000171241	ENSG00000171241	SHCBP1	16:46614465-46655538	q1	q2	OK	14.4999	14.4284	-0.00713467	-0.0102558	0.9829	0.99995	no

ENSG00000171243	ENSG00000171243	SOSTDC1	7:16501105-16621193	q1	q2	OK	1.52788	1.8605	0.284158	0.337131	0.62335	0.99995	no

ENSG00000171246	ENSG00000171246	NPTX1	17:78440947-78451643	q1	q2	NOTEST	0.368215	0.18203	-1.01637	0	1	1	no

ENSG00000171262	ENSG00000171262	FAM98B	15:38746327-38779911	q1	q2	OK	36.524	45.3514	0.312305	0.27913	0.4293	0.99995	no

ENSG00000171282	ENSG00000171282	RP11-1055B8.7	17:79369274-79433357	q1	q2	OK	2.09871	0.480366	-2.1273	-1.25522	0.0616	0.99995	no

ENSG00000171291	ENSG00000171291	ZNF439	19:11959540-11994565	q1	q2	OK	0.553365	0.544695	-0.0227836	-0.0184031	0.97205	0.99995	no

ENSG00000171295	ENSG00000171295	ZNF440	19:11925098-11946016	q1	q2	OK	11.2915	13.1692	0.221934	0.388444	0.5068	0.99995	no

ENSG00000171298	ENSG00000171298	GAA	17:78075354-78093678	q1	q2	OK	16.5023	11.3915	-0.534713	-0.772301	0.14485	0.99995	no

ENSG00000171302	ENSG00000171302	CANT1	17:76987798-77005949	q1	q2	OK	28.8222	28.0036	-0.0415715	-0.0719667	0.88485	0.99995	no

ENSG00000171303	ENSG00000171303	KCNK3	2:26915618-26956288	q1	q2	NOTEST	0.341814	0.219598	-0.638345	0	1	1	no

ENSG00000171307	ENSG00000171307	ZDHHC16	10:99205926-99217127	q1	q2	OK	26.3541	24.4163	-0.110181	-0.208612	0.75585	0.99995	no

ENSG00000171310	ENSG00000171310	CHST11	12:104849072-105155792	q1	q2	OK	8.93564	9.615	0.105716	0.169883	0.7607	0.99995	no

ENSG00000171311	ENSG00000171311	EXOSC1	10:99195898-99205774	q1	q2	OK	21.4527	20.4022	-0.0724346	-0.120691	0.85765	0.99995	no

ENSG00000171314	ENSG00000171314	PGAM1	10:99185916-99193198	q1	q2	OK	91.8373	98.5222	0.101369	0.245181	0.7235	0.99995	no

ENSG00000171316	ENSG00000171316	CHD7	8:61591336-61779465	q1	q2	OK	51.4055	45.3539	-0.180697	-0.182631	0.54525	0.99995	no

ENSG00000171320	ENSG00000171320	ESCO2	8:27590834-27695612	q1	q2	OK	12.0216	12.0593	0.00451061	0.00338604	0.99415	0.99995	no

ENSG00000171345	ENSG00000171345	KRT19	17:39679868-39684560	q1	q2	NOTEST	0.0210419	0	#NAME?	0	1	1	no

ENSG00000171346	ENSG00000171346	KRT15	17:39669994-39678781	q1	q2	NOTEST	0	0	0	0	1	1	no

ENSG00000171357	ENSG00000171357	LURAP1	1:46654353-46686933	q1	q2	OK	2.34803	1.72897	-0.441541	-0.224511	0.7703	0.99995	no

ENSG00000171360	ENSG00000171360	KRT38	17:39592697-39597173	q1	q2	NOTEST	0	0	0	0	1	1	no

ENSG00000171365	ENSG00000171365	CLCN5	X:49687224-49863892	q1	q2	NOTEST	0.377957	0.241226	-0.647836	0	1	1	no

ENSG00000171368	ENSG00000171368	TPPP	5:612386-693510	q1	q2	OK	2.36105	2.01423	-0.2292	-0.295957	0.67455	0.99995	no

ENSG00000171385	ENSG00000171385	KCND3	1:112313283-112531777	q1	q2	OK	5.52932	3.99915	-0.46741	-0.950466	0.1596	0.99995	no

ENSG00000171388	ENSG00000171388	APLN	X:128779239-128788933	q1	q2	OK	3.81896	5.49372	0.524603	0.857897	0.20505	0.99995	no

ENSG00000171396	ENSG00000171396	KRTAP4-4	17:39315909-39316983	q1	q2	NOTEST	0	0	0	0	1	1	no

ENSG00000171401	ENSG00000171401	KRT13	17:39657232-39661957	q1	q2	NOTEST	0	0	0	0	1	1	no

ENSG00000171402	ENSG00000171402	XAGE3	X:52891556-52897099	q1	q2	NOTEST	0	0	0	0	1	1	no

ENSG00000171403	ENSG00000171403	KRT9	17:39722095-39728310	q1	q2	NOTEST	0.0367059	0	#NAME?	0	1	1	no

ENSG00000171405	ENSG00000171405	XAGE5	X:52841227-52847325	q1	q2	NOTEST	0	0	0	0	1	1	no

ENSG00000171408	ENSG00000171408	PDE7B	6:136172833-136546733	q1	q2	OK	3.5953	3.47083	-0.0508323	-0.0896814	0.89015	0.99995	no

ENSG00000171421	ENSG00000171421	MRPL36	5:1798499-1801480	q1	q2	OK	41.2322	39.4799	-0.0626555	-0.114066	0.8696	0.99995	no

ENSG00000171425	ENSG00000171425	ZNF581	19:56146381-56164527	q1	q2	OK	13.5654	13.2975	-0.0287852	-0.0321312	0.9664	0.99995	no

ENSG00000171428	ENSG00000171428	NAT1	8:18027985-18081198	q1	q2	OK	1.60111	1.80468	0.172673	0.181028	0.76575	0.99995	no

ENSG00000171431	ENSG00000171431	KRT20	17:39032192-39041479	q1	q2	NOTEST	0	0	0	0	1	1	no

ENSG00000171433	ENSG00000171433	GLOD5	X:48620153-48632064	q1	q2	NOTEST	0	0	0	0	1	1	no

ENSG00000171435	ENSG00000171435	KSR2	12:117890816-118406788	q1	q2	NOTEST	0.0519404	0.0529502	0.0277777	0	1	1	no

ENSG00000171443	ENSG00000171443	ZNF524	19:56102745-56114504	q1	q2	OK	3.483	3.00457	-0.213175	-0.144189	0.8678	0.99995	no

ENSG00000171444	ENSG00000171444	MCC	5:112357795-112824527	q1	q2	OK	4.90099	4.96543	0.0188464	0.0324031	0.95625	0.99995	no

ENSG00000171446	ENSG00000171446	KRT27	17:38933059-38938786	q1	q2	NOTEST	0	0	0	0	1	1	no

ENSG00000171448	ENSG00000171448	ZBTB26	9:125677844-125693779	q1	q2	OK	9.33938	9.70979	0.0561145	0.122508	0.8615	0.99995	no

ENSG00000171450	ENSG00000171450	CDK5R2	2:219809350-219826876	q1	q2	OK	0.59234	0.737626	0.316465	0.335291	0.6264	0.99995	no

ENSG00000171451	ENSG00000171451	DSEL	18:65173818-65566856	q1	q2	NOTEST	0	0	0	0	1	1	no

ENSG00000171453	ENSG00000171453	POLR1C	6:43474706-43543812	q1	q2	OK	31.2542	30.0009	-0.059048	-0.0377755	0.9416	0.99995	no

ENSG00000171456	ENSG00000171456	ASXL1	20:30946154-31027122	q1	q2	OK	38.4903	38.185	-0.0114894	-0.0174397	0.97035	0.99995	no

ENSG00000171459	ENSG00000171459	OR1L6	9:125512018-125513062	q1	q2	NOTEST	0	0	0	0	1	1	no

ENSG00000171462	ENSG00000171462	DLK2	6:43395103-43424370	q1	q2	OK	0.901064	0.792125	-0.185901	-0.0714795	0.92385	0.99995	no

ENSG00000171466	ENSG00000171466	ZNF562	19:9759329-9785776	q1	q2	OK	7.85747	8.95529	0.188676	0.252162	0.60145	0.99995	no

ENSG00000171467	ENSG00000171467	ZNF318	6:43263431-43337216	q1	q2	OK	11.963	11.6911	-0.0331633	-0.058337	0.90525	0.99995	no

ENSG00000171469	ENSG00000171469	ZNF561	19:9715355-9756572	q1	q2	OK	40.6016	45.5393	0.165574	0.220028	0.6438	0.99995	no

ENSG00000171471	ENSG00000171471	MAP1LC3B2	12:116997185-117014425	q1	q2	OK	5.14228	6.43348	0.323191	0.39328	0.58735	0.99995	no

ENSG00000171475	ENSG00000171475	WIPF2	17:38375555-38440388	q1	q2	OK	18.1238	17.1947	-0.0759216	-0.122255	0.81965	0.99995	no

ENSG00000171476	ENSG00000171476	HOPX	4:57514154-57548065	q1	q2	NOTEST	0.330312	0.401409	0.281244	0	1	1	no

ENSG00000171478	ENSG00000171478	SPACA5B	X:47990038-47991995	q1	q2	NOTEST	0	0	0	0	1	1	no

ENSG00000171481	ENSG00000171481	OR1L3	9:125437314-125438432	q1	q2	NOTEST	0	0	0	0	1	1	no

ENSG00000171483	ENSG00000171483	SSX6	X:47967366-47980068	q1	q2	NOTEST	0	0	0	0	1	1	no

ENSG00000171484	ENSG00000171484	OR1B1	9:125372193-125401964	q1	q2	NOTEST	0	0	0	0	1	1	no

ENSG00000171487	ENSG00000171487	NLRP5	19:56511091-56573179	q1	q2	NOTEST	0	0.00622785	inf	0	1	1	no

ENSG00000171488	ENSG00000171488	LRRC8C	1:89990394-90402170	q1	q2	OK	1.47425	1.49103	0.0163249	0.011869	0.9865	0.99995	no

ENSG00000171489	ENSG00000171489	SPACA5	X:47696300-47931025	q1	q2	NOTEST	0	0	0	0	1	1	no

ENSG00000171490	ENSG00000171490	RSL1D1	16:11929055-11945442	q1	q2	OK	77.5743	85.9685	0.148229	0.263709	0.67535	0.99995	no

ENSG00000171492	ENSG00000171492	LRRC8D	1:89990394-90402170	q1	q2	OK	15.8644	16.1146	0.0225722	0.0372201	0.95405	0.99995	no

ENSG00000171495	ENSG00000171495	MROH2B	5:40998118-41071444	q1	q2	NOTEST	0	0	0	0	1	1	no

ENSG00000171496	ENSG00000171496	OR1L8	9:125329777-125330838	q1	q2	NOTEST	0.0857656	0	#NAME?	0	1	1	no

ENSG00000171497	ENSG00000171497	PPID	4:159587830-159644548	q1	q2	OK	28.7398	31.1135	0.114488	0.157824	0.81325	0.99995	no

ENSG00000171501	ENSG00000171501	OR1N2	9:125315390-125316493	q1	q2	NOTEST	0	0	0	0	1	1	no

ENSG00000171502	ENSG00000171502	COL24A1	1:86194915-86622626	q1	q2	OK	0.889655	0.709843	-0.325746	-0.214756	0.6592	0.99995	no

ENSG00000171503	ENSG00000171503	ETFDH	4:159587830-159644548	q1	q2	OK	11.5191	11.5112	-0.000991731	-0.000798007	0.99895	0.99995	no

ENSG00000171505	ENSG00000171505	OR1N1	9:125288636-125289572	q1	q2	NOTEST	0	0	0	0	1	1	no

ENSG00000171509	ENSG00000171509	RXFP1	4:159236462-159574524	q1	q2	NOTEST	0	0.00709962	inf	0	1	1	no

ENSG00000171517	ENSG00000171517	LPAR3	1:85277284-85358896	q1	q2	NOTEST	0.0335413	0.051717	0.624703	0	1	1	no

ENSG00000171522	ENSG00000171522	PTGER4	5:40679599-40693837	q1	q2	OK	0.787141	0.728195	-0.112298	-0.0624431	0.88135	0.99995	no

ENSG00000171530	ENSG00000171530	TBCA	5:76986990-77164604	q1	q2	OK	151.596	182.701	0.269247	0.621984	0.3692	0.99995	no

ENSG00000171532	ENSG00000171532	NEUROD2	17:37759788-37766030	q1	q2	NOTEST	0.00413861	0.00408699	-0.0181078	0	1	1	no

ENSG00000171533	ENSG00000171533	MAP6	11:75294240-75380165	q1	q2	OK	36.1097	33.7288	-0.0984049	-0.179784	0.7642	0.99995	no

ENSG00000171540	ENSG00000171540	OTP	5:76924537-76935513	q1	q2	NOTEST	0.220243	0.132041	-0.738109	0	1	1	no

ENSG00000171551	ENSG00000171551	ECEL1	2:233344536-233352538	q1	q2	OK	1.00719	1.10156	0.129213	0.123843	0.84955	0.99995	no

ENSG00000171552	ENSG00000171552	BCL2L1	20:30252254-30311792	q1	q2	OK	38.2044	37.2184	-0.0377208	-0.0765384	0.9083	0.99995	no

ENSG00000171557	ENSG00000171557	FGG	4:155525285-155534119	q1	q2	NOTEST	0	0	0	0	1	1	no

ENSG00000171560	ENSG00000171560	FGA	4:155504277-155511918	q1	q2	NOTEST	0	0	0	0	1	1	no

ENSG00000171561	ENSG00000171561	OR2AT4	11:74799757-74800799	q1	q2	NOTEST	0.0297595	0	#NAME?	0	1	1	no

ENSG00000171564	ENSG00000171564	FGB	4:155484107-155492238	q1	q2	NOTEST	0	0.0527413	inf	0	1	1	no

ENSG00000171566	ENSG00000171566	PLRG1	4:155456157-155471587	q1	q2	OK	46.0267	48.8452	0.0857454	0.155704	0.78575	0.99995	no

ENSG00000171570	ENSG00000171570	RAB4B-EGLN2	19:41277552-41406413	q1	q2	OK	0.588305	0.526858	-0.159152	-0.0164922	0.9263	0.99995	no

ENSG00000171574	ENSG00000171574	ZNF584	19:58912870-58929694	q1	q2	OK	11.0762	10.3652	-0.0957114	-0.135044	0.81485	0.99995	no

ENSG00000171587	ENSG00000171587	DSCAM	21:41382925-42219065	q1	q2	NOTEST	0	0.00443604	inf	0	1	1	no

ENSG00000171595	ENSG00000171595	DNAI2	17:72270385-72311023	q1	q2	NOTEST	0.108173	0.0275141	-1.9751	0	1	1	no

ENSG00000171596	ENSG00000171596	NMUR1	2:232387870-232395206	q1	q2	NOTEST	0.038712	0.0153838	-1.33137	0	1	1	no

ENSG00000171603	ENSG00000171603	CLSTN1	1:9711789-9884584	q1	q2	OK	70.4986	63.9324	-0.141048	-0.326525	0.63055	0.99995	no

ENSG00000171604	ENSG00000171604	CXXC5	5:139026883-139063467	q1	q2	OK	30.7752	32.1046	0.0610113	0.128877	0.83645	0.99995	no

ENSG00000171606	ENSG00000171606	ZNF274	19:58694395-58724928	q1	q2	OK	11.6545	12.7405	0.128538	0.222999	0.7102	0.99995	no

ENSG00000171608	ENSG00000171608	PIK3CD	1:9711789-9884584	q1	q2	OK	1.02436	0.86989	-0.23582	-0.0546714	0.87145	0.99995	no

ENSG00000171611	ENSG00000171611	PTCRA	6:42883726-42893573	q1	q2	NOTEST	0	0	0	0	1	1	no

ENSG00000171612	ENSG00000171612	SLC25A33	1:9599540-9645237	q1	q2	OK	5.88662	6.20329	0.075594	0.145733	0.8364	0.99995	no

ENSG00000171617	ENSG00000171617	ENC1	5:73923233-74072737	q1	q2	OK	43.7591	40.4601	-0.11308	-0.195825	0.7709	0.99995	no

ENSG00000171621	ENSG00000171621	SPSB1	1:9352938-9429591	q1	q2	OK	1.82032	2.13115	0.227439	0.26575	0.6709	0.99995	no

ENSG00000171631	ENSG00000171631	P2RY6	11:72975549-73009662	q1	q2	NOTEST	0.0204368	0.0112233	-0.864668	0	1	1	no

ENSG00000171634	ENSG00000171634	BPTF	17:65821639-65980494	q1	q2	OK	29.2687	31.9297	0.125544	0.179704	0.73125	0.99995	no

ENSG00000171643	ENSG00000171643	S100Z	5:76145825-76217475	q1	q2	NOTEST	0	0	0	0	1	1	no

ENSG00000171649	ENSG00000171649	ZIK1	19:58082933-58105145	q1	q2	OK	10.7651	12.3745	0.201007	0.294938	0.5893	0.99995	no

ENSG00000171657	ENSG00000171657	GPR82	X:41374186-41782716	q1	q2	NOTEST	0.0234264	0.0933491	1.9945	0	1	1	no

ENSG00000171658	ENSG00000171658	RP11-443P15.2	3:185677731-185698658	q1	q2	NOTEST	0.203174	0.0145006	-3.80853	0	1	1	no

ENSG00000171659	ENSG00000171659	GPR34	X:41374186-41782716	q1	q2	NOTEST	0.0440856	0.0295366	-0.577805	0	1	1	no

ENSG00000171671	ENSG00000171671	SHANK2-AS3	11:70313960-70963623	q1	q2	NOTEST	0	0	0	0	1	1	no

ENSG00000171680	ENSG00000171680	PLEKHG5	1:6484847-6580121	q1	q2	OK	10.7937	9.76128	-0.145047	-0.255346	0.72985	0.99995	no

ENSG00000171681	ENSG00000171681	ATF7IP	12:14518609-14651697	q1	q2	OK	29.7666	31.7875	0.0947642	0.173028	0.76095	0.99995	no

ENSG00000171695	ENSG00000171695	C20orf201	20:62711525-62731996	q1	q2	NOTEST	0.159682	0.104285	-0.614675	0	1	1	no

ENSG00000171700	ENSG00000171700	RGS19	20:62704533-62711323	q1	q2	OK	5.58384	4.6479	-0.264679	-0.351978	0.6136	0.99995	no

ENSG00000171703	ENSG00000171703	TCEA2	20:62681188-62703700	q1	q2	OK	43.571	40.5433	-0.103903	-0.186657	0.77615	0.99995	no

ENSG00000171711	ENSG00000171711	DEFB4A	8:7752150-7754233	q1	q2	NOTEST	0	0	0	0	1	1	no

ENSG00000171714	ENSG00000171714	ANO5	11:22214721-22304903	q1	q2	OK	1.57458	1.39192	-0.177888	-0.262624	0.68375	0.99995	no

ENSG00000171720	ENSG00000171720	HDAC3	5:140894582-141016437	q1	q2	OK	63.1102	64.3384	0.0278054	0.0349659	0.9507	0.99995	no

ENSG00000171722	ENSG00000171722	C1orf111	1:162039563-162356600	q1	q2	OK	0.58847	0.945097	0.683494	0.117694	0.66465	0.99995	no

ENSG00000171723	ENSG00000171723	GPHN	14:66679527-67648520	q1	q2	OK	23.6943	23.6075	-0.00529262	-0.00591469	0.9898	0.99995	no

ENSG00000171724	ENSG00000171724	VAT1L	16:77822426-78014004	q1	q2	OK	9.16142	8.92795	-0.0372431	-0.0716531	0.91375	0.99995	no

ENSG00000171729	ENSG00000171729	TMEM51	1:15479027-15546976	q1	q2	OK	4.55713	4.87099	0.0960888	0.141343	0.8432	0.99995	no

ENSG00000171735	ENSG00000171735	CAMTA1	1:6845383-7829766	q1	q2	OK	115.774	110.669	-0.065062	-0.120995	0.8654	0.99995	no

ENSG00000171747	ENSG00000171747	LGALS4	19:39292310-39304004	q1	q2	NOTEST	0.372752	0.236285	-0.65769	0	1	1	no

ENSG00000171757	ENSG00000171757	LRRC34	3:169511215-169530774	q1	q2	OK	3.53768	3.36076	-0.0740178	-0.0676896	0.8954	0.99995	no

ENSG00000171759	ENSG00000171759	PAH	12:103230662-103354294	q1	q2	NOTEST	0.0616739	0	#NAME?	0	1	1	no

ENSG00000171763	ENSG00000171763	SPATA5L1	15:45694528-45713617	q1	q2	OK	7.40322	10.2442	0.468587	0.550739	0.31715	0.99995	no

ENSG00000171766	ENSG00000171766	GATM	15:45653321-45694525	q1	q2	OK	6.67227	6.49675	-0.0384596	-0.0599494	0.92145	0.99995	no

ENSG00000171772	ENSG00000171772	SYCE1	10:135192694-135382916	q1	q2	NOTEST	0.00583801	0	#NAME?	0	1	1	no

ENSG00000171773	ENSG00000171773	NXNL1	19:17546317-17571763	q1	q2	NOTEST	0	0	0	0	1	1	no

ENSG00000171777	ENSG00000171777	RASGRP4	19:38893774-38916945	q1	q2	NOTEST	0.103552	0.0581958	-0.831369	0	1	1	no

ENSG00000171786	ENSG00000171786	NHLH1	1:160336856-160342638	q1	q2	OK	6.82516	6.97486	0.0313021	0.0564701	0.93395	0.99995	no

ENSG00000171790	ENSG00000171790	SLFNL1	1:41481268-41488909	q1	q2	OK	0.484201	0.389093	-0.315491	-0.302497	0.63685	0.99995	no

ENSG00000171791	ENSG00000171791	BCL2	18:60790578-60987361	q1	q2	OK	5.93562	6.19913	0.0626666	0.105825	0.85635	0.99995	no

ENSG00000171792	ENSG00000171792	RHNO1	12:2904118-3050306	q1	q2	OK	37.7072	36.2557	-0.0566322	-0.046949	0.941	0.99995	no

ENSG00000171793	ENSG00000171793	CTPS1	1:41445006-41478235	q1	q2	OK	47.5967	50.1759	0.0761322	0.126778	0.80725	0.99995	no

ENSG00000171794	ENSG00000171794	UTF1	10:135043777-135045062	q1	q2	NOTEST	0.0247977	0	#NAME?	0	1	1	no

ENSG00000171798	ENSG00000171798	KNDC1	10:134973950-135039916	q1	q2	OK	2.0561	1.47311	-0.481045	-0.409379	0.33095	0.99995	no

ENSG00000171804	ENSG00000171804	WDR87	19:38375462-38397317	q1	q2	NOTEST	0.0027426	0	#NAME?	0	1	1	no

ENSG00000171806	ENSG00000171806	METTL18	1:169631244-169863408	q1	q2	OK	9.3382	9.56949	0.0352972	0.033934	0.96235	0.99995	no

ENSG00000171811	ENSG00000171811	TTC40	10:134621895-134756327	q1	q2	OK	1.90514	1.75914	-0.115027	-0.103707	0.87335	0.99995	no

ENSG00000171812	ENSG00000171812	COL8A2	1:36560836-36590821	q1	q2	NOTEST	0.124055	0.111247	-0.157213	0	1	1	no

ENSG00000171813	ENSG00000171813	PWWP2B	10:134210671-134231367	q1	q2	OK	4.77956	4.3055	-0.150697	-0.244578	0.72835	0.99995	no

ENSG00000171815	ENSG00000171815	PCDHB1	5:140430978-140433512	q1	q2	NOTEST	0	0	0	0	1	1	no

ENSG00000171817	ENSG00000171817	ZNF540	19:37988062-38210691	q1	q2	OK	1.16549	0.956524	-0.28506	-0.0823809	0.8182	0.99995	no

ENSG00000171819	ENSG00000171819	ANGPTL7	1:11166591-11322564	q1	q2	NOTEST	0.0245555	0.0817331	1.73488	0	1	1	no

ENSG00000171823	ENSG00000171823	FBXL14	12:1639056-1756409	q1	q2	OK	13.6161	12.9377	-0.0737268	-0.113311	0.85375	0.99995	no

ENSG00000171824	ENSG00000171824	EXOSC10	1:11126674-11162157	q1	q2	OK	49.1375	46.2798	-0.0864418	-0.137033	0.76745	0.99995	no

ENSG00000171827	ENSG00000171827	ZNF570	19:37902056-37976260	q1	q2	OK	3.27322	3.89656	0.251489	0.21275	0.72585	0.99995	no

ENSG00000171840	ENSG00000171840	NINJ2	12:673461-772945	q1	q2	NOTEST	0.168293	0.140786	-0.257471	0	1	1	no

ENSG00000171843	ENSG00000171843	MLLT3	9:20341662-20622542	q1	q2	OK	18.4165	15.6778	-0.232275	-0.293493	0.6536	0.99995	no

ENSG00000171847	ENSG00000171847	FAM90A1	12:8373855-8380214	q1	q2	NOTEST	0.0344376	0.043738	0.344904	0	1	1	no

ENSG00000171848	ENSG00000171848	RRM2	2:10262454-10271545	q1	q2	OK	85.5142	93.7105	0.132047	0.24145	0.64325	0.99995	no

ENSG00000171853	ENSG00000171853	TRAPPC12	2:3383445-3488865	q1	q2	OK	28.5896	24.878	-0.20062	-0.291209	0.62915	0.99995	no

ENSG00000171855	ENSG00000171855	IFNB1	9:21077103-21077943	q1	q2	NOTEST	0	0	0	0	1	1	no

ENSG00000171858	ENSG00000171858	RPS21	20:60962171-60963576	q1	q2	OK	696.084	680.92	-0.0317766	-0.0768911	0.9111	0.99995	no

ENSG00000171860	ENSG00000171860	C3AR1	12:8210897-8219067	q1	q2	NOTEST	0.0397627	0.0121934	-1.70532	0	1	1	no

ENSG00000171861	ENSG00000171861	RNMTL1	17:660336-695749	q1	q2	OK	9.27657	9.66851	0.0597019	0.041615	0.9422	0.99995	no

ENSG00000171862	ENSG00000171862	PTEN	10:89618917-89731687	q1	q2	OK	22.6942	22.035	-0.0425244	-0.0418164	0.93165	0.99995	no

ENSG00000171863	ENSG00000171863	RPS7	2:3622794-3628509	q1	q2	OK	673.75	631.891	-0.092539	-0.218771	0.7587	0.99995	no

ENSG00000171864	ENSG00000171864	PRND	20:4702555-4709106	q1	q2	NOTEST	0.0819464	0.0981238	0.259923	0	1	1	no

ENSG00000171865	ENSG00000171865	RNASEH1	2:3579402-3609324	q1	q2	OK	11.442	11.7479	0.0380607	0.0614144	0.92615	0.99995	no

ENSG00000171867	ENSG00000171867	PRNP	20:4666881-4682236	q1	q2	OK	33.998	33.4395	-0.0238981	-0.0547721	0.9363	0.99995	no

ENSG00000171872	ENSG00000171872	KLF17	1:44584521-44600812	q1	q2	NOTEST	0.0161918	0.0235236	0.538844	0	1	1	no

ENSG00000171873	ENSG00000171873	ADRA1D	20:4201328-4229721	q1	q2	NOTEST	0.362777	0.434695	0.260922	0	1	1	no

ENSG00000171877	ENSG00000171877	FRMD5	15:44162961-44487450	q1	q2	OK	2.27207	2.52828	0.154146	0.189877	0.71265	0.99995	no

ENSG00000171885	ENSG00000171885	AQP4	18:24034873-24770662	q1	q2	NOTEST	0.20888	0.198884	-0.0707453	0	1	1	no

ENSG00000171889	ENSG00000171889	MIR31HG	9:21455482-21559668	q1	q2	NOTEST	0.0460378	0	#NAME?	0	1	1	no

ENSG00000171903	ENSG00000171903	CYP4F11	19:16023176-16045677	q1	q2	NOTEST	0	0	0	0	1	1	no

ENSG00000171914	ENSG00000171914	TLN2	15:62682724-63176233	q1	q2	OK	5.9118	6.07276	0.0387544	0.0367705	0.9073	0.99995	no

ENSG00000171916	ENSG00000171916	LGALS9C	17:18380050-18398259	q1	q2	NOTEST	0	0	0	0	1	1	no

ENSG00000171928	ENSG00000171928	TVP23B	17:18684307-18710027	q1	q2	OK	15.3687	17.054	0.150115	0.241059	0.6818	0.99995	no

ENSG00000171931	ENSG00000171931	FBXW10	17:18647325-18682662	q1	q2	NOTEST	0.080518	0.0151861	-2.40656	0	1	1	no

ENSG00000171936	ENSG00000171936	OR10H3	19:15852202-15853153	q1	q2	NOTEST	0	0	0	0	1	1	no

ENSG00000171940	ENSG00000171940	ZNF217	20:52169308-52251129	q1	q2	OK	15.6452	19.8156	0.340919	0.678413	0.25685	0.99995	no

ENSG00000171942	ENSG00000171942	OR10H2	19:15838833-15839862	q1	q2	NOTEST	0	0	0	0	1	1	no

ENSG00000171943	ENSG00000171943	SRGAP2C	1:121102016-121204983	q1	q2	NOTEST	0.032369	0.0987236	1.60878	0	1	1	no

ENSG00000171944	ENSG00000171944	OR52A5	11:5152921-5153872	q1	q2	NOTEST	0	0	0	0	1	1	no

ENSG00000171951	ENSG00000171951	SCG2	2:224461657-224467221	q1	q2	OK	9.23414	8.69676	-0.0865002	-0.153275	0.81915	0.99995	no

ENSG00000171953	ENSG00000171953	ATPAF2	17:17876126-17942523	q1	q2	OK	13.9944	12.2369	-0.19361	-0.264603	0.6636	0.99995	no

ENSG00000171954	ENSG00000171954	CYP4F22	19:15619303-15663128	q1	q2	NOTEST	0.0111434	0	#NAME?	0	1	1	no

ENSG00000171956	ENSG00000171956	FOXB1	15:60296420-60353929	q1	q2	OK	1.52183	1.07917	-0.49589	-0.510654	0.41885	0.99995	no

ENSG00000171960	ENSG00000171960	PPIH	1:43124095-43142429	q1	q2	OK	30.6066	30.0811	-0.0249854	-0.0443626	0.9455	0.99995	no

ENSG00000171962	ENSG00000171962	LRRC48	17:17876126-17942523	q1	q2	OK	2.16799	2.11914	-0.0328806	-0.0190688	0.95475	0.99995	no

ENSG00000171970	ENSG00000171970	ZNF57	19:2900895-2926805	q1	q2	OK	2.6565	3.21194	0.273918	0.337148	0.60035	0.99995	no

ENSG00000171984	ENSG00000171984	C20orf196	20:5731038-5844558	q1	q2	OK	5.92709	5.71695	-0.0520802	-0.0660217	0.91945	0.99995	no

ENSG00000171987	ENSG00000171987	C11orf40	11:4592652-4599050	q1	q2	NOTEST	0	0	0	0	1	1	no

ENSG00000171988	ENSG00000171988	JMJD1C	10:64926980-65226323	q1	q2	OK	37.2306	39.2115	0.0747887	0.103573	0.8731	0.99995	no

ENSG00000171989	ENSG00000171989	LDHAL6B	15:59427112-59815748	q1	q2	NOTEST	0.233153	0.09666	-1.27029	0	1	1	no

ENSG00000171992	ENSG00000171992	SYNPO	5:149980641-150038782	q1	q2	OK	3.65882	4.5501	0.31452	0.463069	0.48675	0.99995	no

ENSG00000171999	ENSG00000171999	OR52P2P	11:4452552-4453507	q1	q2	NOTEST	0	0	0	0	1	1	no

ENSG00000172000	ENSG00000172000	ZNF556	19:2867332-2878515	q1	q2	NOTEST	0.0994157	0.0160683	-2.62925	0	1	1	no

ENSG00000172005	ENSG00000172005	MAL	2:95690937-95719737	q1	q2	NOTEST	0.250763	0.319711	0.350442	0	1	1	no

ENSG00000172006	ENSG00000172006	ZNF554	19:2819871-2835771	q1	q2	OK	3.14648	3.01637	-0.0609257	-0.0889572	0.897	0.99995	no

ENSG00000172007	ENSG00000172007	RAB33B	4:140187316-140397763	q1	q2	OK	4.29552	4.33778	0.0141248	0.00867835	0.98655	0.99995	no

ENSG00000172009	ENSG00000172009	THOP1	19:2785457-2813599	q1	q2	OK	32.2988	29.5228	-0.129648	-0.232499	0.6738	0.99995	no

ENSG00000172014	ENSG00000172014	ANKRD20A4	9:69381811-69425560	q1	q2	OK	1.03647	1.46728	0.501463	0.662097	0.32615	0.99995	no

ENSG00000172016	ENSG00000172016	REG3A	2:79384131-80875905	q1	q2	NOTEST	0	0	0	0	1	1	no

ENSG00000172020	ENSG00000172020	GAP43	3:115342170-115440337	q1	q2	OK	50.3981	57.8138	0.198045	0.43442	0.5357	0.99995	no

ENSG00000172023	ENSG00000172023	REG1B	2:79312155-79315145	q1	q2	NOTEST	0	0	0	0	1	1	no

ENSG00000172031	ENSG00000172031	EPHX4	1:92495538-92529093	q1	q2	OK	1.0518	1.04657	-0.00719264	-0.00745336	0.9895	0.99995	no

ENSG00000172037	ENSG00000172037	LAMB2	3:49158546-49170551	q1	q2	OK	83.9203	63.8307	-0.394768	-0.495615	0.3723	0.99995	no

ENSG00000172046	ENSG00000172046	USP19	3:49145478-49158371	q1	q2	OK	19.5256	18.3772	-0.0874526	-0.138797	0.79625	0.99995	no

ENSG00000172053	ENSG00000172053	QARS	3:49133364-49142553	q1	q2	OK	102.804	90.0579	-0.190971	-0.332619	0.5916	0.99995	no

ENSG00000172057	ENSG00000172057	ORMDL3	17:38077293-38083854	q1	q2	OK	29.6186	28.9108	-0.0348992	-0.0775776	0.90545	0.99995	no

ENSG00000172058	ENSG00000172058	SERF1A	5:70196491-70214357	q1	q2	NOTEST	0.0494233	0.108934	1.14019	0	1	1	no

ENSG00000172059	ENSG00000172059	KLF11	2:10182975-10194993	q1	q2	OK	3.17156	3.43154	0.113664	0.163598	0.786	0.99995	no

ENSG00000172061	ENSG00000172061	LRRC15	3:194075975-194090472	q1	q2	NOTEST	0.0208808	0	#NAME?	0	1	1	no

ENSG00000172062	ENSG00000172062	SMN1	5:70220767-70249769	q1	q2	NOTEST	0.274247	0.194639	-0.494677	0	1	1	no

ENSG00000172071	ENSG00000172071	EIF2AK3	2:88838237-88931339	q1	q2	OK	10.8911	11.0966	0.0269671	0.038677	0.93975	0.99995	no

ENSG00000172073	ENSG00000172073	TEX37	2:88824168-88829102	q1	q2	NOTEST	0.0421558	0	#NAME?	0	1	1	no

ENSG00000172081	ENSG00000172081	MOB3A	19:2071036-2099592	q1	q2	OK	15.3711	14.4174	-0.0924084	-0.140507	0.7742	0.99995	no

ENSG00000172086	ENSG00000172086	KRCC1	2:88326723-88355248	q1	q2	OK	17.8588	19.2656	0.109396	0.222917	0.7443	0.99995	no

ENSG00000172113	ENSG00000172113	NME6	3:48282589-48343175	q1	q2	OK	19.8898	19.5158	-0.0273904	-0.0340206	0.9533	0.99995	no

ENSG00000172115	ENSG00000172115	CYCS	7:25159709-25164980	q1	q2	OK	98.4804	117.509	0.254868	0.473631	0.49335	0.99995	no

ENSG00000172116	ENSG00000172116	CD8B	2:87042461-87092827	q1	q2	NOTEST	0	0	0	0	1	1	no

ENSG00000172123	ENSG00000172123	SLFN12	17:33738078-33760302	q1	q2	OK	3.48712	4.47454	0.359704	0.427689	0.51965	0.99995	no

ENSG00000172137	ENSG00000172137	CALB2	16:71392615-71424341	q1	q2	OK	1.35819	1.51377	0.156464	0.158744	0.7934	0.99995	no

ENSG00000172139	ENSG00000172139	SLC9C1	3:111859733-112013105	q1	q2	NOTEST	0.00936786	0	#NAME?	0	1	1	no

ENSG00000172146	ENSG00000172146	OR1A1	17:3118914-3119844	q1	q2	NOTEST	0	0	0	0	1	1	no

ENSG00000172148	ENSG00000172148	AC005255.6	19:14974597-14975527	q1	q2	NOTEST	0	0	0	0	1	1	no

ENSG00000172150	ENSG00000172150	OR1A2	17:3100812-3101742	q1	q2	NOTEST	0	0	0	0	1	1	no

ENSG00000172154	ENSG00000172154	OR8I2	11:55860752-55861762	q1	q2	NOTEST	0	0	0	0	1	1	no

ENSG00000172155	ENSG00000172155	LCE1D	1:152769226-152770657	q1	q2	NOTEST	0	0	0	0	1	1	no

ENSG00000172156	ENSG00000172156	CCL11	17:32612686-32615353	q1	q2	NOTEST	0.0269985	0.0806636	1.57904	0	1	1	no

ENSG00000172159	ENSG00000172159	FRMD3	9:85857904-86153461	q1	q2	OK	0.576069	0.774751	0.427491	0.279156	0.48835	0.99995	no

ENSG00000172164	ENSG00000172164	SNTB1	8:121392999-121825599	q1	q2	OK	1.15828	1.10994	-0.0615033	-0.0338189	0.9511	0.99995	no

ENSG00000172167	ENSG00000172167	MTBP	8:121392999-121825599	q1	q2	OK	8.07291	8.91192	0.142647	0.177545	0.76875	0.99995	no

ENSG00000172171	ENSG00000172171	TEFM	17:29224353-29286340	q1	q2	OK	13.2019	13.0281	-0.0191164	-0.0272362	0.96905	0.99995	no

ENSG00000172172	ENSG00000172172	MRPL13	8:121392999-121825599	q1	q2	OK	35.4456	39.0874	0.141096	0.23996	0.7326	0.99995	no

ENSG00000172175	ENSG00000172175	MALT1	18:56337711-56421709	q1	q2	OK	7.86472	9.06803	0.205394	0.357319	0.57005	0.99995	no

ENSG00000172179	ENSG00000172179	PRL	6:22260652-22318027	q1	q2	NOTEST	0.0216994	0.0426931	0.976346	0	1	1	no

ENSG00000172183	ENSG00000172183	ISG20	15:89179383-89199714	q1	q2	OK	0.531298	0.717747	0.433955	0.229988	0.56345	0.99995	no

ENSG00000172186	ENSG00000172186	HMGN1P35	X:68393966-68394560	q1	q2	NOTEST	0	0.0594577	inf	0	1	1	no

ENSG00000172188	ENSG00000172188	OR4C11	11:55370829-55371874	q1	q2	NOTEST	0	0	0	0	1	1	no

ENSG00000172197	ENSG00000172197	MBOAT1	6:20100934-20317970	q1	q2	OK	4.98047	5.4251	0.123367	0.191538	0.7833	0.99995	no

ENSG00000172199	ENSG00000172199	OR8U1	11:56143099-56144029	q1	q2	NOTEST	0	0	0	0	1	1	no

ENSG00000172201	ENSG00000172201	ID4	6:19535174-19840915	q1	q2	OK	86.4489	79.6975	-0.117313	-0.271019	0.6936	0.99995	no

ENSG00000172208	ENSG00000172208	OR4X2	11:48266607-48267622	q1	q2	NOTEST	0	0	0	0	1	1	no

ENSG00000172209	ENSG00000172209	GPR22	7:106809405-107218906	q1	q2	NOTEST	0.0879291	0.177784	1.01572	0	1	1	no

ENSG00000172215	ENSG00000172215	CXCR6	3:45959395-46037316	q1	q2	NOTEST	0.0848609	0.0335078	-1.3406	0	1	1	no

ENSG00000172216	ENSG00000172216	CEBPB	20:48807375-48809212	q1	q2	OK	4.22437	3.65985	-0.20695	-0.297802	0.6595	0.99995	no

ENSG00000172232	ENSG00000172232	AZU1	19:825096-832017	q1	q2	OK	1.46767	0.763298	-0.94321	-0.887269	0.1995	0.99995	no

ENSG00000172236	ENSG00000172236	TPSAB1	16:1290696-1292555	q1	q2	NOTEST	0	0.0323099	inf	0	1	1	no

ENSG00000172238	ENSG00000172238	ATOH1	4:94750041-94751221	q1	q2	NOTEST	0.0511204	0.0502467	-0.0248703	0	1	1	no

ENSG00000172239	ENSG00000172239	PAIP1	5:43526368-43557860	q1	q2	OK	84.5322	89.3984	0.0807479	0.17609	0.79285	0.99995	no

ENSG00000172243	ENSG00000172243	CLEC7A	12:10269375-10282857	q1	q2	NOTEST	0	0	0	0	1	1	no

ENSG00000172244	ENSG00000172244	C5orf34	5:43484060-43525412	q1	q2	OK	6.95383	6.72928	-0.0473571	-0.053186	0.9398	0.99995	no

ENSG00000172247	ENSG00000172247	C1QTNF4	11:47611215-47616211	q1	q2	OK	2.66569	2.49848	-0.0934544	-0.116868	0.86335	0.99995	no

ENSG00000172250	ENSG00000172250	SERHL	22:42896584-42915808	q1	q2	OK	0.954584	0.75067	-0.346694	-0.0979166	0.8573	0.99995	no

ENSG00000172260	ENSG00000172260	NEGR1	1:71547006-72748417	q1	q2	OK	2.6649	2.98388	0.163109	0.150966	0.80435	0.99995	no

ENSG00000172262	ENSG00000172262	ZNF131	5:43014515-43192123	q1	q2	OK	21.6704	24.5845	0.18202	0.30577	0.5562	0.99995	no

ENSG00000172264	ENSG00000172264	MACROD2	20:13829892-16033842	q1	q2	OK	3.88848	4.34987	0.161769	0.0632224	0.82615	0.99995	no

ENSG00000172269	ENSG00000172269	DPAGT1	11:118967212-118989252	q1	q2	OK	23.1791	22.1901	-0.0629065	-0.0925328	0.88035	0.99995	no

ENSG00000172270	ENSG00000172270	BSG	19:567211-583493	q1	q2	OK	222.824	207.627	-0.101909	-0.239527	0.71945	0.99995	no

ENSG00000172273	ENSG00000172273	HINFP	11:118992296-119006752	q1	q2	OK	15.6556	16.6494	0.0887936	0.128842	0.82865	0.99995	no

ENSG00000172283	ENSG00000172283	PRYP4	Y:28121659-28134216	q1	q2	NOTEST	0	0	0	0	1	1	no

ENSG00000172288	ENSG00000172288	CDY1	Y:27768263-27771049	q1	q2	NOTEST	0	0	0	0	1	1	no

ENSG00000172289	ENSG00000172289	OR10V1	11:59480295-59573354	q1	q2	NOTEST	0	0	0	0	1	1	no

ENSG00000172292	ENSG00000172292	CERS6	2:169312371-169769881	q1	q2	OK	14.0418	15.9777	0.186333	0.413689	0.55665	0.99995	no

ENSG00000172294	ENSG00000172294	CSPG4P4Y	Y:27624415-27633809	q1	q2	NOTEST	0	0	0	0	1	1	no

ENSG00000172296	ENSG00000172296	SPTLC3	20:12989626-13147411	q1	q2	NOTEST	0.0781084	0.0771607	-0.017612	0	1	1	no

ENSG00000172297	ENSG00000172297	GOLGA2P3Y	Y:27600707-27606719	q1	q2	NOTEST	0	0	0	0	1	1	no

ENSG00000172301	ENSG00000172301	COPRS	17:30178882-30186356	q1	q2	OK	32.2591	33.2236	0.0425031	0.0825566	0.90735	0.99995	no

ENSG00000172315	ENSG00000172315	TP53RK	20:45313003-45318928	q1	q2	OK	12.3756	13.3398	0.108238	0.221031	0.75475	0.99995	no

ENSG00000172318	ENSG00000172318	B3GALT1	2:168671283-168797654	q1	q2	OK	2.61939	2.70919	0.0486303	0.0710874	0.9196	0.99995	no

ENSG00000172320	ENSG00000172320	OR5A1	11:59210616-59211667	q1	q2	NOTEST	0	0	0	0	1	1	no

ENSG00000172322	ENSG00000172322	CLEC12A	12:10100735-10183205	q1	q2	NOTEST	0	0	0	0	1	1	no

ENSG00000172324	ENSG00000172324	OR5A2	11:59189415-59190449	q1	q2	NOTEST	0	0	0	0	1	1	no

ENSG00000172331	ENSG00000172331	BPGM	7:134331559-134364565	q1	q2	OK	15.1774	16.191	0.0932649	0.184958	0.7885	0.99995	no

ENSG00000172332	ENSG00000172332	AC012005.2	Y:26355713-26361728	q1	q2	NOTEST	0	0	0	0	1	1	no

ENSG00000172336	ENSG00000172336	POP7	7:100303675-100305118	q1	q2	OK	32.8282	29.4601	-0.156174	-0.303813	0.66245	0.99995	no

ENSG00000172339	ENSG00000172339	ALG14	1:95439962-95538501	q1	q2	OK	1.77524	2.99351	0.753825	0.373297	0.43685	0.99995	no

ENSG00000172340	ENSG00000172340	SUCLG2	3:67410883-67705038	q1	q2	OK	20.0368	20.8786	0.0593753	0.132231	0.84215	0.99995	no

ENSG00000172342	ENSG00000172342	CSPG4P3Y	Y:26325462-26338017	q1	q2	NOTEST	0	0	0	0	1	1	no

ENSG00000172345	ENSG00000172345	STARD5	15:81451915-81616524	q1	q2	OK	1.16298	0.897067	-0.374544	-0.303073	0.57145	0.99995	no

ENSG00000172346	ENSG00000172346	CSDC2	22:41956766-41985894	q1	q2	OK	1.67907	1.35456	-0.309839	-0.240388	0.6919	0.99995	no

ENSG00000172348	ENSG00000172348	RCAN2	6:46188474-46459709	q1	q2	OK	4.47955	4.70716	0.0715021	0.124921	0.85775	0.99995	no

ENSG00000172349	ENSG00000172349	IL16	15:81451915-81616524	q1	q2	NOTEST	0.284545	0.044452	-2.67834	0	1	1	no

ENSG00000172350	ENSG00000172350	ABCG4	11:119019721-119033360	q1	q2	OK	0.607726	0.323061	-0.911613	-0.802679	0.1705	0.99995	no

ENSG00000172352	ENSG00000172352	CDY1B	Y:26191375-26194166	q1	q2	NOTEST	0	0	0	0	1	1	no

ENSG00000172354	ENSG00000172354	GNB2	7:100271153-100276797	q1	q2	OK	124.734	114.17	-0.127669	-0.306902	0.65355	0.99995	no

ENSG00000172361	ENSG00000172361	CCDC11	18:47753562-47792892	q1	q2	OK	0.639268	0.416412	-0.61841	-0.619346	0.40435	0.99995	no

ENSG00000172362	ENSG00000172362	OR5B12	11:58206592-58207646	q1	q2	NOTEST	0	0	0	0	1	1	no

ENSG00000172365	ENSG00000172365	OR5B2	11:58189737-58190786	q1	q2	NOTEST	0	0	0	0	1	1	no

ENSG00000172366	ENSG00000172366	FAM195A	16:691812-699200	q1	q2	OK	5.65737	4.59549	-0.299913	-0.371867	0.59495	0.99995	no

ENSG00000172367	ENSG00000172367	PDZD3	11:119056165-119067479	q1	q2	NOTEST	0.0169752	0.00937855	-0.855992	0	1	1	no

ENSG00000172375	ENSG00000172375	C2CD2L	11:118967212-118989252	q1	q2	OK	4.3755	3.8704	-0.176963	-0.164459	0.77435	0.99995	no

ENSG00000172377	ENSG00000172377	OR9I1	11:57791352-57949088	q1	q2	NOTEST	0	0	0	0	1	1	no

ENSG00000172379	ENSG00000172379	ARNT2	15:80696691-80890278	q1	q2	OK	25.4902	24.6425	-0.0487939	-0.113726	0.8665	0.99995	no

ENSG00000172380	ENSG00000172380	GNG12	1:68167148-68698803	q1	q2	OK	19.4727	19.7084	0.0173556	0.0215581	0.97455	0.99995	no

ENSG00000172381	ENSG00000172381	OR6Q1	11:57791352-57949088	q1	q2	NOTEST	0	0	0	0	1	1	no

ENSG00000172382	ENSG00000172382	PRSS27	16:2762418-2770552	q1	q2	OK	3.85879	2.90919	-0.40753	-0.474624	0.50685	0.99995	no

ENSG00000172399	ENSG00000172399	MYOZ2	4:120056938-120108944	q1	q2	NOTEST	0	0.0102929	inf	0	1	1	no

ENSG00000172403	ENSG00000172403	SYNPO2	4:119809995-119982402	q1	q2	NOTEST	0.0954878	0.0940946	-0.0212056	0	1	1	no

ENSG00000172404	ENSG00000172404	DNAJB7	22:41253080-41369313	q1	q2	NOTEST	0.334516	0.246587	-0.439979	0	1	1	no

ENSG00000172409	ENSG00000172409	CLP1	11:57405496-57429340	q1	q2	OK	15.3119	14.0829	-0.120707	-0.161451	0.81475	0.99995	no

ENSG00000172410	ENSG00000172410	INSL5	1:67263423-67266939	q1	q2	NOTEST	0.284447	0	#NAME?	0	1	1	no

ENSG00000172421	ENSG00000172421	EFCAB3	17:60447578-60493837	q1	q2	NOTEST	0.0186533	0	#NAME?	0	1	1	no

ENSG00000172425	ENSG00000172425	TTC36	11:118307204-118473748	q1	q2	NOTEST	0	0	0	0	1	1	no

ENSG00000172426	ENSG00000172426	RSPH9	6:43612782-43655528	q1	q2	OK	0.493883	0.500079	0.0179857	0.00804872	0.91555	0.99995	no

ENSG00000172428	ENSG00000172428	MYEOV2	2:241065979-241076224	q1	q2	OK	129.735	124.759	-0.0564239	-0.115376	0.86435	0.99995	no

ENSG00000172432	ENSG00000172432	GTPBP2	6:43543886-43596899	q1	q2	OK	35.1817	34.4645	-0.0297154	-0.0449239	0.9476	0.99995	no

ENSG00000172456	ENSG00000172456	FGGY	1:59762309-60254854	q1	q2	OK	25.456	28.6089	0.16846	0.26844	0.7046	0.99995	no

ENSG00000172457	ENSG00000172457	OR9G4	11:56510302-56511287	q1	q2	NOTEST	0	0	0	0	1	1	no

ENSG00000172458	ENSG00000172458	IL17D	13:21276265-21297237	q1	q2	OK	7.57243	6.74068	-0.167861	-0.244428	0.732	0.99995	no

ENSG00000172459	ENSG00000172459	OR5AR1	11:56431137-56432163	q1	q2	NOTEST	0	0	0	0	1	1	no

ENSG00000172460	ENSG00000172460	PRSS30P	16:2889568-2892745	q1	q2	NOTEST	0	0	0	0	1	1	no

ENSG00000172461	ENSG00000172461	FUT9	6:96463859-96663488	q1	q2	OK	3.98901	4.31888	0.114626	0.235308	0.7307	0.99995	no

ENSG00000172464	ENSG00000172464	OR5AP2	11:56408941-56409947	q1	q2	NOTEST	0	0	0	0	1	1	no

ENSG00000172465	ENSG00000172465	TCEAL1	X:102862378-102885881	q1	q2	OK	10.6421	10.6003	-0.00567768	-0.00700311	0.9928	0.99995	no

ENSG00000172466	ENSG00000172466	ZNF24	18:32912175-32925446	q1	q2	OK	40.5405	45.1969	0.156859	0.385237	0.5858	0.99995	no

ENSG00000172468	ENSG00000172468	HSFY1	Y:20652800-20790963	q1	q2	NOTEST	0	0	0	0	1	1	no

ENSG00000172469	ENSG00000172469	MANEA	6:96025418-96057333	q1	q2	OK	10.1055	12.1162	0.261799	0.518943	0.404	0.99995	no

ENSG00000172476	ENSG00000172476	RAB40A	X:102752450-102774417	q1	q2	OK	1.86807	1.90733	0.0300119	0.0395837	0.95715	0.99995	no

ENSG00000172478	ENSG00000172478	C2orf54	2:241825464-241836306	q1	q2	NOTEST	0	0	0	0	1	1	no

ENSG00000172482	ENSG00000172482	AGXT	2:241807895-241819919	q1	q2	NOTEST	0	0	0	0	1	1	no

ENSG00000172487	ENSG00000172487	OR8J1	11:56127690-56128764	q1	q2	NOTEST	0	0	0	0	1	1	no

ENSG00000172489	ENSG00000172489	OR5T3	11:56019675-56020698	q1	q2	NOTEST	0	0	0	0	1	1	no

ENSG00000172493	ENSG00000172493	AFF1	4:87797357-88062206	q1	q2	OK	8.14062	8.35348	0.0372381	0.0718575	0.9016	0.99995	no

ENSG00000172497	ENSG00000172497	ACOT12	5:80625823-80689998	q1	q2	NOTEST	0.0251641	0	#NAME?	0	1	1	no

ENSG00000172500	ENSG00000172500	FIBP	11:65647279-65656010	q1	q2	OK	44.4914	38.762	-0.198885	-0.391387	0.55115	0.99995	no

ENSG00000172508	ENSG00000172508	CARNS1	11:67159175-67193078	q1	q2	NOTEST	0.186982	0.167939	-0.154964	0	1	1	no

ENSG00000172519	ENSG00000172519	OR10H5	19:15904760-15905892	q1	q2	NOTEST	0	0	0	0	1	1	no

ENSG00000172530	ENSG00000172530	BANP	16:87982849-88110924	q1	q2	OK	10.0673	9.95265	-0.0165212	-0.0241709	0.9655	0.99995	no

ENSG00000172531	ENSG00000172531	PPP1CA	11:67159175-67193078	q1	q2	OK	108.27	104.771	-0.0473937	-0.107001	0.8731	0.99995	no

ENSG00000172534	ENSG00000172534	HCFC1	X:153213003-153237258	q1	q2	OK	1.74211	1.29537	-0.427471	-0.509792	0.50765	0.99995	no

ENSG00000172538	ENSG00000172538	FAM170B	10:50329883-50359592	q1	q2	NOTEST	0	0	0	0	1	1	no

ENSG00000172543	ENSG00000172543	CTSW	11:65647279-65656010	q1	q2	NOTEST	0.0219473	0.193279	3.13857	0	1	1	no

ENSG00000172548	ENSG00000172548	NIPAL4	5:156687131-157002783	q1	q2	NOTEST	0.101086	0.109175	0.111062	0	1	1	no

ENSG00000172551	ENSG00000172551	MUCL1	12:55224302-55252177	q1	q2	NOTEST	0	0	0	0	1	1	no

ENSG00000172554	ENSG00000172554	SNTG2	2:946553-1371385	q1	q2	NOTEST	0.0608084	0.237837	1.96763	0	1	1	no

ENSG00000172568	ENSG00000172568	FNDC9	5:156687131-157002783	q1	q2	NOTEST	0.167805	0.136665	-0.296144	0	1	1	no

ENSG00000172572	ENSG00000172572	PDE3A	12:20514665-20837315	q1	q2	OK	2.38649	1.96218	-0.282431	-0.341324	0.6194	0.99995	no

ENSG00000172575	ENSG00000172575	RASGRP1	15:38780303-38857776	q1	q2	OK	0.514941	0.701053	0.445117	0.347576	0.3996	0.99995	no

ENSG00000172578	ENSG00000172578	KLHL6	3:183205318-183273477	q1	q2	NOTEST	0.0342058	0.0571868	0.741443	0	1	1	no

ENSG00000172586	ENSG00000172586	CHCHD1	10:75541804-75543410	q1	q2	OK	39.3572	40.2504	0.0323765	0.0649897	0.92605	0.99995	no

ENSG00000172590	ENSG00000172590	MRPL52	14:23299087-23304246	q1	q2	OK	56.3726	53.0573	-0.0874431	-0.131999	0.84535	0.99995	no

ENSG00000172594	ENSG00000172594	SMPDL3A	6:123110314-123130865	q1	q2	OK	5.71693	5.14904	-0.150935	-0.217152	0.7605	0.99995	no

ENSG00000172602	ENSG00000172602	RND1	12:49250927-49259681	q1	q2	OK	7.87066	6.2721	-0.327536	-0.471564	0.4508	0.99995	no

ENSG00000172613	ENSG00000172613	RAD9A	11:67159175-67193078	q1	q2	OK	13.1761	9.80487	-0.426351	-0.290488	0.5512	0.99995	no

ENSG00000172638	ENSG00000172638	EFEMP2	11:65590492-65641063	q1	q2	OK	14.3731	12.9798	-0.147101	-0.0346981	0.8445	0.99995	no

ENSG00000172640	ENSG00000172640	OR10AD1	12:48596080-48597170	q1	q2	NOTEST	0.0266648	0	#NAME?	0	1	1	no

ENSG00000172650	ENSG00000172650	AGAP5	10:75404638-75490272	q1	q2	OK	3.06677	2.90489	-0.0782338	-0.0891471	0.89885	0.99995	no

ENSG00000172653	ENSG00000172653	C17orf66	17:34136458-34195895	q1	q2	NOTEST	0	0	0	0	1	1	no

ENSG00000172660	ENSG00000172660	TAF15	17:34136458-34195895	q1	q2	OK	41.5875	52.6068	0.3391	0.562632	0.4126	0.99995	no

ENSG00000172661	ENSG00000172661	FAM21C	10:46201878-46288409	q1	q2	OK	0.672467	1.13831	0.759361	0.764112	0.16465	0.99995	no

ENSG00000172663	ENSG00000172663	TMEM134	11:67231823-67236743	q1	q2	OK	15.0253	13.74	-0.129015	-0.17566	0.80245	0.99995	no

ENSG00000172667	ENSG00000172667	ZMAT3	3:178735010-178790067	q1	q2	OK	7.16708	6.31934	-0.181612	-0.3423	0.56385	0.99995	no

ENSG00000172671	ENSG00000172671	ZFAND4	10:46111042-46168228	q1	q2	OK	2.59782	2.61785	0.0110836	0.0111045	0.98655	0.99995	no

ENSG00000172673	ENSG00000172673	THEMIS	6:128029216-128239776	q1	q2	NOTEST	0.00688294	0.00679293	-0.0189913	0	1	1	no

ENSG00000172680	ENSG00000172680	MOS	8:57025500-57026541	q1	q2	NOTEST	0	0	0	0	1	1	no

ENSG00000172687	ENSG00000172687	ZNF738	19:21541731-21562104	q1	q2	OK	43.244	47.2511	0.127846	0.260277	0.70335	0.99995	no

ENSG00000172689	ENSG00000172689	MS4A10	11:60552820-60568778	q1	q2	NOTEST	0	0	0	0	1	1	no

ENSG00000172716	ENSG00000172716	SLFN11	17:33677323-33700720	q1	q2	NOTEST	0.290962	0.419221	0.526879	0	1	1	no

ENSG00000172717	ENSG00000172717	FAM71D	14:67656109-67695267	q1	q2	NOTEST	0.230966	0.168224	-0.457298	0	1	1	no

ENSG00000172724	ENSG00000172724	CCL19	9:34689563-34691274	q1	q2	NOTEST	0	0	0	0	1	1	no

ENSG00000172725	ENSG00000172725	CORO1B	11:67202980-67211292	q1	q2	OK	31.6941	28.6546	-0.145445	-0.19648	0.6586	0.99995	no

ENSG00000172728	ENSG00000172728	FUT10	8:33228341-33371119	q1	q2	OK	4.25677	4.27499	0.00616265	0.00451945	0.99455	0.99995	no

ENSG00000172731	ENSG00000172731	LRRC20	10:72058728-72142382	q1	q2	OK	16.4486	16.1433	-0.0270255	-0.0574566	0.934	0.99995	no

ENSG00000172732	ENSG00000172732	MUS81	11:65590492-65641063	q1	q2	OK	35.8257	35.2019	-0.0253411	-0.00909793	0.9697	0.99995	no

ENSG00000172733	ENSG00000172733	PURG	8:30853320-30891231	q1	q2	OK	7.35053	7.04281	-0.061698	-0.083124	0.88925	0.99995	no

ENSG00000172738	ENSG00000172738	TMEM217	6:37179955-37300746	q1	q2	OK	0.811954	0.89972	0.148079	0.0374273	0.8967	0.99995	no

ENSG00000172742	ENSG00000172742	OR4D9	11:59282385-59283330	q1	q2	NOTEST	0	0	0	0	1	1	no

ENSG00000172746	ENSG00000172746	RP11-344H11.4	1:27306655-27307466	q1	q2	NOTEST	0	0.0792498	inf	0	1	1	no

ENSG00000172748	ENSG00000172748	ZNF596	8:163185-197342	q1	q2	OK	5.46401	4.85327	-0.171004	-0.160119	0.74695	0.99995	no

ENSG00000172752	ENSG00000172752	COL6A5	3:130064358-130203688	q1	q2	NOTEST	0.00525779	0	#NAME?	0	1	1	no

ENSG00000172757	ENSG00000172757	CFL1	11:65590492-65641063	q1	q2	OK	960.75	960.712	-5.72E-05	-0.000103909	0.99995	0.99995	no

ENSG00000172765	ENSG00000172765	TMCC1	3:129366634-129612419	q1	q2	OK	11.8654	13.2407	0.158211	0.167994	0.73855	0.99995	no

ENSG00000172766	ENSG00000172766	NAA16	13:41885340-41951166	q1	q2	OK	27.1467	28.9684	0.0937028	0.153797	0.8167	0.99995	no

ENSG00000172769	ENSG00000172769	OR5B3	11:58169936-58170882	q1	q2	NOTEST	0	0	0	0	1	1	no

ENSG00000172771	ENSG00000172771	EFCAB12	3:129120163-129147494	q1	q2	OK	1.561	1.37513	-0.182902	-0.20325	0.7581	0.99995	no

ENSG00000172772	ENSG00000172772	OR10W1	11:58034263-58035732	q1	q2	NOTEST	0	0	0	0	1	1	no

ENSG00000172774	ENSG00000172774	OR1S1	11:57982216-57983195	q1	q2	NOTEST	0	0	0	0	1	1	no

ENSG00000172775	ENSG00000172775	FAM192A	16:57186377-57220028	q1	q2	OK	60.9654	63.7169	0.0636843	0.111526	0.83715	0.99995	no

ENSG00000172780	ENSG00000172780	RAB43	3:128806411-128880136	q1	q2	OK	2.20286	1.39955	-0.654414	-0.424361	0.60375	0.99995	no

ENSG00000172782	ENSG00000172782	FADS6	17:72873427-72889708	q1	q2	NOTEST	0	0	0	0	1	1	no

ENSG00000172785	ENSG00000172785	CBWD1	9:121040-188979	q1	q2	OK	21.9257	21.7807	-0.00957185	-0.0179285	0.9772	0.99995	no

ENSG00000172789	ENSG00000172789	HOXC5	12:54375292-54449814	q1	q2	NOTEST	0	0	0	0	1	1	no

ENSG00000172794	ENSG00000172794	RAB37	17:72666716-72765492	q1	q2	NOTEST	0.144665	0.040582	-1.83381	0	1	1	no

ENSG00000172795	ENSG00000172795	DCP2	5:112312398-112356667	q1	q2	OK	17.2963	19.9743	0.20768	0.321231	0.4863	0.99995	no

ENSG00000172799	ENSG00000172799	ZBTB8OSP2	2:232298633-232299137	q1	q2	NOTEST	0	0.160529	inf	0	1	1	no

ENSG00000172803	ENSG00000172803	SNX32	11:65590492-65641063	q1	q2	OK	1.55444	1.2931	-0.265556	-0.0243885	0.77415	0.99995	no

ENSG00000172809	ENSG00000172809	RPL38	17:72199720-72209481	q1	q2	OK	718.569	757.687	0.0764744	0.173306	0.81035	0.99995	no

ENSG00000172817	ENSG00000172817	CYP7B1	8:65500319-65711318	q1	q2	OK	1.34631	1.63689	0.281944	0.304917	0.6434	0.99995	no

ENSG00000172818	ENSG00000172818	OVOL1	11:65554492-65564690	q1	q2	NOTEST	0.0334979	0.0439188	0.390767	0	1	1	no

ENSG00000172819	ENSG00000172819	RARG	12:53604353-53626764	q1	q2	OK	3.2834	3.06376	-0.0998842	-0.123724	0.83065	0.99995	no

ENSG00000172824	ENSG00000172824	CES4A	16:67022491-67043661	q1	q2	OK	1.27334	0.858785	-0.568249	-0.488998	0.4333	0.99995	no

ENSG00000172828	ENSG00000172828	CES3	16:66968346-67009051	q1	q2	OK	0.566998	0.210028	-1.43276	-0.352929	0.20785	0.99995	no

ENSG00000172830	ENSG00000172830	SSH3	11:67070918-67080078	q1	q2	OK	0.951434	0.921445	-0.0462059	-0.0239794	0.9503	0.99995	no

ENSG00000172831	ENSG00000172831	CES2	16:66968346-67009051	q1	q2	OK	17.0372	14.9836	-0.185301	-0.282466	0.5499	0.99995	no

ENSG00000172840	ENSG00000172840	PDP2	16:66912491-66929657	q1	q2	OK	4.68413	4.91331	0.0689149	0.0598603	0.86795	0.99995	no

ENSG00000172845	ENSG00000172845	SP3	2:174771186-174830430	q1	q2	OK	38.6917	43.0245	0.153133	0.368141	0.59485	0.99995	no

ENSG00000172867	ENSG00000172867	KRT2	12:53038341-53045948	q1	q2	NOTEST	0	0	0	0	1	1	no

ENSG00000172869	ENSG00000172869	DMXL1	5:118342041-118584833	q1	q2	OK	7.56039	10.9095	0.529056	0.551028	0.27385	0.99995	no

ENSG00000172878	ENSG00000172878	METAP1D	2:172640879-172947158	q1	q2	OK	5.9813	6.5458	0.13011	0.0809361	0.8559	0.99995	no

ENSG00000172888	ENSG00000172888	ZNF621	3:40566368-40616176	q1	q2	OK	7.90049	8.48385	0.102778	0.174849	0.77485	0.99995	no

ENSG00000172889	ENSG00000172889	EGFL7	9:139543061-139567130	q1	q2	OK	8.36123	7.81961	-0.096618	-0.147319	0.8376	0.99995	no

ENSG00000172890	ENSG00000172890	NADSYN1	11:71164154-71235153	q1	q2	OK	14.3038	11.4729	-0.318165	-0.463041	0.3865	0.99995	no

ENSG00000172893	ENSG00000172893	DHCR7	11:71139238-71163914	q1	q2	OK	50.2913	54.2969	0.110561	0.228148	0.6988	0.99995	no

ENSG00000172900	ENSG00000172900	AP002387.1	11:71093646-71134469	q1	q2	NOTEST	0	0	0	0	1	1	no

ENSG00000172901	ENSG00000172901	AQPEP	5:115298150-115395815	q1	q2	NOTEST	0.252146	0.349746	0.472053	0	1	1	no

ENSG00000172912	ENSG00000172912	COX6B1P3	22:40742506-41032706	q1	q2	NOTEST	0	0	0	0	1	1	no

ENSG00000172915	ENSG00000172915	NBEA	13:35516423-36247159	q1	q2	OK	5.41555	5.40734	-0.00219024	-0.00364445	0.9954	0.99995	no

ENSG00000172922	ENSG00000172922	RNASEH2C	11:65479466-65488418	q1	q2	OK	32.7124	27.6286	-0.243674	-0.361633	0.58095	0.99995	no

ENSG00000172927	ENSG00000172927	MYEOV	11:69061604-69182494	q1	q2	NOTEST	0.0851208	0.0256414	-1.73103	0	1	1	no

ENSG00000172932	ENSG00000172932	ANKRD13D	11:67056017-67069956	q1	q2	OK	19.2936	18.7021	-0.0449253	-0.0756927	0.90095	0.99995	no

ENSG00000172935	ENSG00000172935	MRGPRF	11:68771861-68785915	q1	q2	NOTEST	0.264835	0.257786	-0.0389185	0	1	1	no

ENSG00000172936	ENSG00000172936	MYD88	3:38179968-38184513	q1	q2	OK	12.1539	12.614	0.0535997	0.110737	0.87045	0.99995	no

ENSG00000172938	ENSG00000172938	MRGPRD	11:68747489-68748455	q1	q2	NOTEST	0	0.0321106	inf	0	1	1	no

ENSG00000172939	ENSG00000172939	OXSR1	3:38206579-38296979	q1	q2	OK	23.5814	24.8246	0.0741209	0.142297	0.7834	0.99995	no

ENSG00000172940	ENSG00000172940	SLC22A13	3:38307302-38319806	q1	q2	NOTEST	0.0385801	0.0726236	0.91258	0	1	1	no

ENSG00000172943	ENSG00000172943	PHF8	X:53963108-54075391	q1	q2	OK	1.32476	0.625471	-1.08272	-0.643853	0.08785	0.99995	no

ENSG00000172954	ENSG00000172954	LCLAT1	2:30670091-30867091	q1	q2	OK	3.71983	5.02051	0.432596	0.494944	0.28325	0.99995	no

ENSG00000172955	ENSG00000172955	ADH6	4:100010007-100222513	q1	q2	OK	0.571957	1.4494	1.34147	0.411064	0.43505	0.99995	no

ENSG00000172965	ENSG00000172965	MIR4435-1HG	2:111953539-112268567	q1	q2	OK	20.6965	22.9471	0.148925	0.194054	0.78145	0.99995	no

ENSG00000172967	ENSG00000172967	XKR3	22:17264301-17302589	q1	q2	NOTEST	0	0	0	0	1	1	no

ENSG00000172969	ENSG00000172969	FRG2C	3:75713480-75716371	q1	q2	NOTEST	0	0	0	0	1	1	no

ENSG00000172971	ENSG00000172971	UNC93B3	3:75668656-75673000	q1	q2	NOTEST	0	0	0	0	1	1	no

ENSG00000172974	ENSG00000172974	AC007318.5	2:65432136-65433396	q1	q2	OK	41.9845	42.7359	0.0255917	0.0535611	0.93915	0.99995	no

ENSG00000172977	ENSG00000172977	KAT5	11:65479466-65488418	q1	q2	OK	26.4957	25.4882	-0.0559294	-0.0741912	0.90275	0.99995	no

ENSG00000172985	ENSG00000172985	SH3RF3	2:109745803-110262207	q1	q2	OK	2.16308	1.99928	-0.113605	-0.153096	0.8282	0.99995	no

ENSG00000172986	ENSG00000172986	GXYLT2	3:72937223-73118350	q1	q2	OK	1.66913	1.78498	0.0968128	0.0289877	0.95975	0.99995	no

ENSG00000172987	ENSG00000172987	HPSE2	10:100218874-100995619	q1	q2	NOTEST	0	0.0185654	inf	0	1	1	no

ENSG00000172992	ENSG00000172992	DCAKD	17:43100707-43221548	q1	q2	OK	26.1912	23.5419	-0.153854	-0.154794	0.8026	0.99995	no

ENSG00000172995	ENSG00000172995	ARPP21	3:35680436-35835988	q1	q2	OK	0.735438	0.511404	-0.52414	-0.219045	0.3039	0.99995	no

ENSG00000173011	ENSG00000173011	TADA2B	4:6910968-7059679	q1	q2	OK	9.15375	8.7361	-0.0673746	-0.0658011	0.9154	0.99995	no

ENSG00000173013	ENSG00000173013	CCDC96	4:6910968-7059679	q1	q2	OK	2.49749	2.19478	-0.186403	-0.079526	0.62205	0.99995	no

ENSG00000173020	ENSG00000173020	ADRBK1	11:67033880-67054027	q1	q2	OK	25.8524	23.9964	-0.107475	-0.194847	0.7084	0.99995	no

ENSG00000173039	ENSG00000173039	RELA	11:65421066-65430565	q1	q2	OK	20.9724	19.9502	-0.0720889	-0.131082	0.81445	0.99995	no

ENSG00000173040	ENSG00000173040	EVC2	4:5544498-5711275	q1	q2	NOTEST	0.188413	0.20837	0.14525	0	1	1	no

ENSG00000173041	ENSG00000173041	ZNF680	7:63980261-64023484	q1	q2	OK	5.06049	5.64904	0.158727	0.249095	0.684	0.99995	no

ENSG00000173064	ENSG00000173064	HECTD4	12:112597991-112819896	q1	q2	OK	19.3349	18.1436	-0.0917503	-0.0815127	0.8341	0.99995	no

ENSG00000173065	ENSG00000173065	FAM222B	17:27082995-27188085	q1	q2	OK	13.967	13.7711	-0.0203775	-0.0230154	0.96005	0.99995	no

ENSG00000173068	ENSG00000173068	BNC2	9:16409500-16870841	q1	q2	OK	2.68332	2.91831	0.121116	0.0954128	0.81645	0.99995	no

ENSG00000173077	ENSG00000173077	Dec-01	9:117904096-118164923	q1	q2	NOTEST	0.177739	0.0840029	-1.08125	0	1	1	no

ENSG00000173080	ENSG00000173080	RXFP4	1:155911479-155912625	q1	q2	NOTEST	0.0264808	0.026023	-0.0251602	0	1	1	no

ENSG00000173083	ENSG00000173083	HPSE	4:84213613-84256306	q1	q2	NOTEST	0.368559	0.30124	-0.290981	0	1	1	no

ENSG00000173085	ENSG00000173085	COQ2	4:84182688-84206067	q1	q2	OK	9.42949	10.1604	0.107701	0.182189	0.79175	0.99995	no

ENSG00000173088	ENSG00000173088	C10orf131	10:97471535-97849995	q1	q2	NOTEST	0.143094	0.0695115	-1.04164	0	1	1	no

ENSG00000173093	ENSG00000173093	CCDC63	12:111284572-111345339	q1	q2	NOTEST	0.0148353	0	#NAME?	0	1	1	no

ENSG00000173110	ENSG00000173110	HSPA6	1:161475219-161648444	q1	q2	NOTEST	0.0689626	0.0577806	-0.25523	0	1	1	no

ENSG00000173113	ENSG00000173113	TRMT112	11:64073043-64085556	q1	q2	OK	100.165	100.951	0.0112738	0.0208853	0.9751	0.99995	no

ENSG00000173114	ENSG00000173114	LRRN3	7:110303109-111202573	q1	q2	OK	26.7961	23.8368	-0.168834	-0.3849	0.5873	0.99995	no

ENSG00000173120	ENSG00000173120	KDM2A	11:66886739-67025558	q1	q2	OK	24.7349	23.6623	-0.0639601	-0.111073	0.8294	0.99995	no

ENSG00000173124	ENSG00000173124	C10orf129	10:96953956-96990901	q1	q2	NOTEST	0	0	0	0	1	1	no

ENSG00000173137	ENSG00000173137	ADCK5	8:145596789-145634753	q1	q2	NOTEST	0.137896	0.177691	0.365791	0	1	1	no

ENSG00000173141	ENSG00000173141	MRP63	13:21750783-21753223	q1	q2	OK	18.247	16.3735	-0.156292	-0.3337	0.6411	0.99995	no

ENSG00000173145	ENSG00000173145	NOC3L	10:95753745-96122716	q1	q2	OK	8.17178	10.0023	0.291609	0.448099	0.4766	0.99995	no

ENSG00000173153	ENSG00000173153	ESRRA	11:64073043-64085556	q1	q2	OK	12.8233	12.5211	-0.0344008	-0.0361225	0.9529	0.99995	no

ENSG00000173156	ENSG00000173156	RHOD	11:66824288-66839484	q1	q2	OK	0.827202	0.337539	-1.29319	-0.906266	0.14635	0.99995	no

ENSG00000173157	ENSG00000173157	ADAMTS20	12:43747668-43945724	q1	q2	OK	0.74628	0.856564	0.198844	0.219819	0.7635	0.99995	no

ENSG00000173163	ENSG00000173163	COMMD1	2:62095223-62374382	q1	q2	OK	37.2142	36.8906	-0.0126012	-0.00834759	0.9904	0.99995	no

ENSG00000173166	ENSG00000173166	RAPH1	2:204192941-204400133	q1	q2	OK	9.0383	9.09262	0.00864521	0.00665024	0.9913	0.99995	no

ENSG00000173171	ENSG00000173171	MTX1	1:155165378-155183615	q1	q2	OK	10.0129	9.96101	-0.00750172	-0.00723212	0.99105	0.99995	no

ENSG00000173175	ENSG00000173175	ADCY5	3:123001142-123168605	q1	q2	OK	2.18204	1.63517	-0.416234	-0.39267	0.37965	0.99995	no

ENSG00000173193	ENSG00000173193	PARP14	3:122399464-122449687	q1	q2	OK	2.40788	1.97758	-0.284028	-0.321712	0.51125	0.99995	no

ENSG00000173198	ENSG00000173198	CYSLTR1	X:77526960-77583048	q1	q2	NOTEST	0.0205168	0.0193593	-0.0837766	0	1	1	no

ENSG00000173200	ENSG00000173200	PARP15	3:122296448-122357894	q1	q2	NOTEST	0	0.0112406	inf	0	1	1	no

ENSG00000173207	ENSG00000173207	CKS1B	1:154947128-154951725	q1	q2	OK	110.759	114.488	0.0477736	0.111724	0.87215	0.99995	no

ENSG00000173208	ENSG00000173208	ABCD2	12:39943834-40013553	q1	q2	NOTEST	0.150692	0.211547	0.48937	0	1	1	no

ENSG00000173209	ENSG00000173209	AHSA2	2:61404552-61697904	q1	q2	OK	43.9247	46.7195	0.0889906	0.0624144	0.86685	0.99995	no

ENSG00000173210	ENSG00000173210	ABLIM3	5:148521045-148721365	q1	q2	NOTEST	0.146338	0.154293	0.0763742	0	1	1	no

ENSG00000173212	ENSG00000173212	MAB21L3	1:116654375-116677861	q1	q2	NOTEST	0	0.0151736	inf	0	1	1	no

ENSG00000173213	ENSG00000173213	RP11-683L23.1	18:47224-73545	q1	q2	NOTEST	0.0209079	0	#NAME?	0	1	1	no

ENSG00000173214	ENSG00000173214	KIAA1919	6:111580550-111592370	q1	q2	OK	2.80817	2.9916	0.0912874	0.165152	0.8103	0.99995	no

ENSG00000173218	ENSG00000173218	VANGL1	1:116184573-116240845	q1	q2	OK	2.92532	2.49809	-0.227773	-0.310839	0.6134	0.99995	no

ENSG00000173221	ENSG00000173221	GLRX	5:95036952-95160087	q1	q2	OK	9.16088	9.87357	0.108087	0.0454927	0.93855	0.99995	no

ENSG00000173226	ENSG00000173226	IQCB1	3:121488609-121553926	q1	q2	OK	22.7195	24.7969	0.126228	0.21352	0.7242	0.99995	no

ENSG00000173227	ENSG00000173227	SYT12	11:66774248-66818334	q1	q2	NOTEST	0.183422	0.247174	0.43036	0	1	1	no

ENSG00000173230	ENSG00000173230	GOLGB1	3:121382045-121468602	q1	q2	OK	22.6399	20.4455	-0.147085	-0.23928	0.6296	0.99995	no

ENSG00000173231	ENSG00000173231	TERF1P1	21:15148406-15149587	q1	q2	NOTEST	0	0	0	0	1	1	no

ENSG00000173237	ENSG00000173237	C11orf86	11:66742747-66744475	q1	q2	NOTEST	0.0240439	0	#NAME?	0	1	1	no

ENSG00000173239	ENSG00000173239	LIPM	10:90562486-90580303	q1	q2	NOTEST	0	0.0384729	inf	0	1	1	no

ENSG00000173250	ENSG00000173250	GPR151	5:145892665-145895753	q1	q2	NOTEST	0.0259848	0.0341803	0.395499	0	1	1	no

ENSG00000173253	ENSG00000173253	DMRT2	9:1050353-1057552	q1	q2	NOTEST	0.0821345	0.128344	0.643957	0	1	1	no

ENSG00000173258	ENSG00000173258	ZNF483	9:114287438-114393569	q1	q2	OK	1.42243	1.4505	0.0281896	0.0189929	0.9742	0.99995	no

ENSG00000173261	ENSG00000173261	PLAC8L1	5:145463948-145562223	q1	q2	OK	2.11131	1.42407	-0.56812	-0.0845349	0.7479	0.99995	no

ENSG00000173262	ENSG00000173262	SLC2A14	12:7965107-8043744	q1	q2	OK	0.652259	1.35433	1.05406	0.770068	0.03645	0.99995	no

ENSG00000173264	ENSG00000173264	GPR137	11:64037301-64056972	q1	q2	OK	28.244	22.5997	-0.321643	-0.438442	0.5018	0.99995	no

ENSG00000173267	ENSG00000173267	SNCG	10:88695296-88784489	q1	q2	OK	4.38884	4.01236	-0.129389	-0.105339	0.88245	0.99995	no

ENSG00000173269	ENSG00000173269	MMRN2	10:88695296-88784489	q1	q2	OK	0.514005	0.812851	0.661211	0.27381	0.39335	0.99995	no

ENSG00000173272	ENSG00000173272	MZT2A	2:132222472-132250316	q1	q2	OK	26.0644	23.6627	-0.139462	-0.219624	0.75355	0.99995	no

ENSG00000173273	ENSG00000173273	TNKS	8:9412655-9639856	q1	q2	OK	25.2382	27.1026	0.102821	0.150737	0.71745	0.99995	no

ENSG00000173275	ENSG00000173275	ZNF449	X:134478720-134497077	q1	q2	NOTEST	0.103925	0.0746562	-0.477202	0	1	1	no

ENSG00000173276	ENSG00000173276	ZBTB21	21:43406939-43445060	q1	q2	OK	4.69442	5.10622	0.121309	0.203244	0.71445	0.99995	no

ENSG00000173281	ENSG00000173281	PPP1R3B	8:8993764-9009084	q1	q2	OK	2.6459	3.07696	0.217748	0.356567	0.61235	0.99995	no

ENSG00000173285	ENSG00000173285	OR10K1	1:158435271-158436344	q1	q2	NOTEST	0	0	0	0	1	1	no

ENSG00000173295	ENSG00000173295	FAM86B3P	8:8086116-8102387	q1	q2	OK	1.60922	1.42945	-0.170899	-0.138673	0.78275	0.99995	no

ENSG00000173302	ENSG00000173302	GPR148	2:131486642-131487909	q1	q2	NOTEST	0.0234721	0	#NAME?	0	1	1	no

ENSG00000173320	ENSG00000173320	STOX2	4:184774583-184944679	q1	q2	OK	14.1705	13.6786	-0.0509636	-0.0877834	0.8626	0.99995	no

ENSG00000173327	ENSG00000173327	MAP3K11	11:65365225-65382853	q1	q2	OK	17.3939	14.3762	-0.274891	-0.476205	0.3819	0.99995	no

ENSG00000173334	ENSG00000173334	TRIB1	8:126442562-126450647	q1	q2	OK	4.6929	4.87697	0.0555045	0.0899532	0.8891	0.99995	no

ENSG00000173335	ENSG00000173335	CST9	20:23583046-23586513	q1	q2	NOTEST	0	0	0	0	1	1	no

ENSG00000173338	ENSG00000173338	KCNK7	11:65360325-65363467	q1	q2	NOTEST	0.215675	0.32147	0.575825	0	1	1	no

ENSG00000173349	ENSG00000173349	SFT2D3	2:128458595-128568761	q1	q2	NOTEST	0	0	0	0	1	1	no

ENSG00000173357	ENSG00000173357	AC007967.3	Y:8774264-8784114	q1	q2	NOTEST	0	0	0	0	1	1	no

ENSG00000173366	ENSG00000173366	TLR9	3:52255095-52275113	q1	q2	NOTEST	0.00019056	0	#NAME?	0	1	1	no

ENSG00000173369	ENSG00000173369	C1QB	1:22979254-22988031	q1	q2	NOTEST	0	0	0	0	1	1	no

ENSG00000173372	ENSG00000173372	C1QA	1:22962998-22966101	q1	q2	NOTEST	0	0	0	0	1	1	no

ENSG00000173376	ENSG00000173376	NDNF	4:121956767-122001631	q1	q2	OK	6.4587	5.68038	-0.185255	-0.313002	0.63535	0.99995	no

ENSG00000173389	ENSG00000173389	IQCF1	3:51928891-51937351	q1	q2	NOTEST	0	0	0	0	1	1	no

ENSG00000173391	ENSG00000173391	OLR1	12:10310901-10344400	q1	q2	NOTEST	0	0	0	0	1	1	no

ENSG00000173401	ENSG00000173401	GLIPR1L1	12:75628519-75784708	q1	q2	NOTEST	0	0.0338876	inf	0	1	1	no

ENSG00000173402	ENSG00000173402	DAG1	3:49506145-49573048	q1	q2	OK	25.7216	23.9335	-0.103951	-0.20982	0.7066	0.99995	no

ENSG00000173404	ENSG00000173404	INSM1	20:20348764-20351590	q1	q2	OK	14.4651	14.1172	-0.0351283	-0.0761812	0.91305	0.99995	no

ENSG00000173406	ENSG00000173406	DAB1	1:57394882-59012474	q1	q2	OK	13.0967	13.7083	0.0658513	0.103181	0.8444	0.99995	no

ENSG00000173409	ENSG00000173409	ARV1	1:231114726-231136341	q1	q2	OK	13.7336	13.5798	-0.016248	-0.0284979	0.96575	0.99995	no

ENSG00000173418	ENSG00000173418	NAA20	20:19997759-20014299	q1	q2	OK	36.7168	38.0795	0.052572	0.109434	0.87845	0.99995	no

ENSG00000173421	ENSG00000173421	CCDC36	3:49235860-49295537	q1	q2	NOTEST	0.078695	0.112299	0.513007	0	1	1	no

ENSG00000173431	ENSG00000173431	RNASE8	14:21484921-21572881	q1	q2	NOTEST	0.057861	0	#NAME?	0	1	1	no

ENSG00000173432	ENSG00000173432	SAA1	11:18287720-18291524	q1	q2	NOTEST	0	0	0	0	1	1	no

ENSG00000173436	ENSG00000173436	MINOS1	1:19923472-19984945	q1	q2	OK	119.645	129.486	0.114047	0.138327	0.84195	0.99995	no

ENSG00000173442	ENSG00000173442	EHBP1L1	11:65343508-65360121	q1	q2	OK	6.97668	5.90072	-0.241649	-0.285197	0.62015	0.99995	no

ENSG00000173451	ENSG00000173451	THAP2	12:72003251-72097836	q1	q2	OK	7.30145	8.2297	0.172657	0.0890957	0.82745	0.99995	no

ENSG00000173452	ENSG00000173452	TMEM196	7:19758932-19813221	q1	q2	NOTEST	0.146922	0.0766429	-0.938822	0	1	1	no

ENSG00000173456	ENSG00000173456	RNF26	11:119205236-119208023	q1	q2	OK	25.2336	23.4157	-0.10787	-0.251141	0.72545	0.99995	no

ENSG00000173457	ENSG00000173457	PPP1R14B	11:64011955-64016966	q1	q2	OK	70.3448	62.7405	-0.165047	-0.368724	0.5958	0.99995	no

ENSG00000173464	ENSG00000173464	RNASE11	14:21051050-21078043	q1	q2	NOTEST	0	0	0	0	1	1	no

ENSG00000173465	ENSG00000173465	SSSCA1	11:65337900-65341669	q1	q2	OK	27.3559	25.1847	-0.119304	-0.137694	0.8486	0.99995	no

ENSG00000173467	ENSG00000173467	AGR3	7:16899028-16921611	q1	q2	NOTEST	0	0	0	0	1	1	no

ENSG00000173473	ENSG00000173473	SMARCC1	3:47626761-47823596	q1	q2	OK	59.835	65.679	0.134443	0.27138	0.63525	0.99995	no

ENSG00000173480	ENSG00000173480	ZNF417	19:58281022-58427978	q1	q2	OK	7.75877	8.50146	0.131882	0.0584981	0.86035	0.99995	no

ENSG00000173482	ENSG00000173482	PTPRM	18:7566779-8406951	q1	q2	OK	9.69252	10.7534	0.149844	0.190247	0.67305	0.99995	no

ENSG00000173486	ENSG00000173486	FKBP2	11:64008474-64011604	q1	q2	OK	43.1807	38.3319	-0.171839	-0.318061	0.65495	0.99995	no

ENSG00000173511	ENSG00000173511	VEGFB	11:64002009-64006259	q1	q2	OK	34.4404	30.5473	-0.173055	-0.306815	0.659	0.99995	no

ENSG00000173517	ENSG00000173517	PEAK1	15:77400470-77712486	q1	q2	OK	13.1091	13.7051	0.0641475	0.0607924	0.8299	0.99995	no

ENSG00000173530	ENSG00000173530	TNFRSF10D	8:22993100-23021543	q1	q2	OK	5.63016	4.67904	-0.266964	-0.4634	0.51285	0.99995	no

ENSG00000173531	ENSG00000173531	MST1	3:49711434-49761384	q1	q2	OK	9.87736	7.3404	-0.428266	-0.180472	0.52115	0.99995	no

ENSG00000173535	ENSG00000173535	TNFRSF10C	8:22941867-22974950	q1	q2	NOTEST	0.434329	0.474014	0.126142	0	1	1	no

ENSG00000173540	ENSG00000173540	GMPPB	3:49711434-49761384	q1	q2	OK	10.6431	9.16374	-0.215909	-0.161972	0.79545	0.99995	no

ENSG00000173542	ENSG00000173542	MOB1B	4:71768042-71896631	q1	q2	OK	21.3199	22.6976	0.0903341	0.168479	0.7924	0.99995	no

ENSG00000173545	ENSG00000173545	ZNF622	5:16451627-16465901	q1	q2	OK	14.2575	13.1488	-0.11679	-0.221922	0.7549	0.99995	no

ENSG00000173546	ENSG00000173546	CSPG4	15:75966662-76005189	q1	q2	OK	2.83889	2.59309	-0.130654	-0.229957	0.7508	0.99995	no

ENSG00000173548	ENSG00000173548	SNX33	15:75928479-75954642	q1	q2	OK	10.0468	9.15178	-0.134615	-0.193208	0.7699	0.99995	no

ENSG00000173557	ENSG00000173557	C2orf70	2:26785449-26802400	q1	q2	NOTEST	0	0	0	0	1	1	no

ENSG00000173559	ENSG00000173559	NABP1	2:192542793-192561385	q1	q2	OK	2.2509	2.23909	-0.00759329	-0.00725967	0.99135	0.99995	no

ENSG00000173566	ENSG00000173566	NUDT18	8:21964382-21966932	q1	q2	OK	1.08639	0.921187	-0.237969	-0.251905	0.71625	0.99995	no

ENSG00000173567	ENSG00000173567	GPR113	2:26531040-26618759	q1	q2	NOTEST	0.23824	0.195324	-0.286545	0	1	1	no

ENSG00000173572	ENSG00000173572	NLRP13	19:56403064-56443702	q1	q2	NOTEST	0.00809264	0	#NAME?	0	1	1	no

ENSG00000173575	ENSG00000173575	CHD2	15:93425936-93571237	q1	q2	OK	31.6949	30.725	-0.0448374	-0.0411064	0.88745	0.99995	no

ENSG00000173578	ENSG00000173578	XCR1	3:46058515-46069234	q1	q2	NOTEST	0.00492168	0.00903491	0.876359	0	1	1	no

ENSG00000173581	ENSG00000173581	CCDC106	19:56146381-56164527	q1	q2	OK	14.8392	11.7583	-0.335732	-0.391171	0.53205	0.99995	no

ENSG00000173585	ENSG00000173585	CCR9	3:45864807-45957534	q1	q2	NOTEST	0.0108168	0	#NAME?	0	1	1	no

ENSG00000173588	ENSG00000173588	CCDC41	12:94542498-94853764	q1	q2	OK	13.2667	15.7025	0.24318	0.183371	0.73255	0.99995	no

ENSG00000173597	ENSG00000173597	SULT1B1	4:70586879-70653679	q1	q2	NOTEST	0.0102944	0.0529854	2.36374	0	1	1	no

ENSG00000173598	ENSG00000173598	NUDT4	12:93771658-93797024	q1	q2	OK	9.08809	9.91783	0.126048	0.229588	0.70555	0.99995	no

ENSG00000173599	ENSG00000173599	PC	11:66615703-66725847	q1	q2	OK	10.9191	10.3517	-0.0769811	-0.0572658	0.8841	0.99995	no

ENSG00000173610	ENSG00000173610	UGT2A1	4:70454134-70518967	q1	q2	NOTEST	0.0634476	0	#NAME?	0	1	1	no

ENSG00000173611	ENSG00000173611	SCAI	9:127640645-127905785	q1	q2	OK	3.1667	3.93356	0.312856	0.297241	0.44655	0.99995	no

ENSG00000173612	ENSG00000173612	GPRC6A	6:117113247-117150200	q1	q2	NOTEST	0	0	0	0	1	1	no

ENSG00000173614	ENSG00000173614	NMNAT1	1:9982172-10045559	q1	q2	OK	7.27875	7.35693	0.0154117	0.016289	0.97865	0.99995	no

ENSG00000173621	ENSG00000173621	LRFN4	11:66615703-66725847	q1	q2	OK	12.3012	11.0255	-0.157957	-0.104689	0.757	0.99995	no

ENSG00000173626	ENSG00000173626	TRAPPC3L	6:116813459-116880031	q1	q2	NOTEST	0.0219493	0.0629223	1.5194	0	1	1	no

ENSG00000173627	ENSG00000173627	APOBEC4	1:183605219-183897665	q1	q2	NOTEST	0	0.0100788	inf	0	1	1	no

ENSG00000173638	ENSG00000173638	SLC19A1	21:46825051-46964325	q1	q2	OK	6.9025	5.71381	-0.272667	-0.176522	0.76375	0.99995	no

ENSG00000173641	ENSG00000173641	HSPB7	1:16340522-16360545	q1	q2	NOTEST	0.00881473	0.0247299	1.48827	0	1	1	no

ENSG00000173653	ENSG00000173653	RCE1	11:66511921-66614017	q1	q2	OK	13.4344	12.957	-0.0522076	-0.0627682	0.91365	0.99995	no

ENSG00000173660	ENSG00000173660	UQCRH	1:46769302-46782448	q1	q2	OK	265.501	268.248	0.014852	0.0362526	0.95855	0.99995	no

ENSG00000173662	ENSG00000173662	TAS1R1	1:6615240-6639817	q1	q2	NOTEST	0.0297365	0.0142974	-1.05648	0	1	1	no

ENSG00000173673	ENSG00000173673	HES3	1:6304251-6305638	q1	q2	OK	0.491283	1.10874	1.1743	0.956141	0.19845	0.99995	no

ENSG00000173674	ENSG00000173674	EIF1AX	X:20142635-20159962	q1	q2	OK	32.1337	34.6777	0.109918	0.266524	0.70055	0.99995	no

ENSG00000173678	ENSG00000173678	SPDYE2B	7:102122891-102312088	q1	q2	OK	0.429984	0.685934	0.673788	0.193686	0.7101	0.99995	no

ENSG00000173679	ENSG00000173679	OR1L1	9:125423844-125424927	q1	q2	NOTEST	0	0	0	0	1	1	no

ENSG00000173681	ENSG00000173681	CXorf23	X:19930977-19988416	q1	q2	OK	6.20538	6.77507	0.126718	0.186618	0.71815	0.99995	no

ENSG00000173692	ENSG00000173692	PSMD1	2:231921577-232037541	q1	q2	OK	73.5518	74.9638	0.0274336	0.0563897	0.9207	0.99995	no

ENSG00000173698	ENSG00000173698	GPR64	X:19007426-19140755	q1	q2	OK	2.01747	2.5454	0.335343	0.539559	0.4518	0.99995	no

ENSG00000173699	ENSG00000173699	SPATA3	2:231860835-231889770	q1	q2	NOTEST	0	0	0	0	1	1	no

ENSG00000173702	ENSG00000173702	MUC13	3:124624288-124672663	q1	q2	NOTEST	0.062072	0.0171818	-1.85306	0	1	1	no

ENSG00000173705	ENSG00000173705	SUSD5	3:33191536-33260707	q1	q2	OK	1.04646	1.08824	0.0564896	0.0694693	0.9113	0.99995	no

ENSG00000173706	ENSG00000173706	HEG1	3:124684553-124774802	q1	q2	OK	11.481	11.5838	0.0128559	0.0183788	0.976	0.99995	no

ENSG00000173714	ENSG00000173714	WFIKKN2	17:48912010-48919709	q1	q2	NOTEST	0.0461253	0.0565533	0.294051	0	1	1	no

ENSG00000173715	ENSG00000173715	C11orf80	11:66511921-66614017	q1	q2	OK	16.5096	17.6088	0.092992	0.115154	0.8532	0.99995	no

ENSG00000173726	ENSG00000173726	TOMM20	1:235272650-235292251	q1	q2	OK	78.4018	82.4595	0.0727984	0.157915	0.7963	0.99995	no

ENSG00000173727	ENSG00000173727	AP000769.1	11:65222727-65234028	q1	q2	NOTEST	0.0509128	0.0473238	-0.10546	0	1	1	no

ENSG00000173728	ENSG00000173728	C1orf100	1:244515936-244615436	q1	q2	NOTEST	0.150196	0.112924	-0.411494	0	1	1	no

ENSG00000173744	ENSG00000173744	AGFG1	2:228336847-228425930	q1	q2	OK	38.7994	37.0429	-0.0668373	-0.0929261	0.8915	0.99995	no

ENSG00000173757	ENSG00000173757	STAT5B	17:40351185-40428725	q1	q2	OK	28.7073	28.3362	-0.0187722	-0.0325304	0.95115	0.99995	no

ENSG00000173762	ENSG00000173762	CD7	17:80272743-80275478	q1	q2	NOTEST	0	0	0	0	1	1	no

ENSG00000173769	ENSG00000173769	TOPAZ1	3:44283377-44373590	q1	q2	NOTEST	0	0	0	0	1	1	no

ENSG00000173786	ENSG00000173786	CNP	17:40118758-40217383	q1	q2	OK	0.616847	0.599784	-0.0404697	-0.0165698	0.96145	0.99995	no

ENSG00000173801	ENSG00000173801	JUP	17:39775688-39943183	q1	q2	NOTEST	0	0.0075889	inf	0	1	1	no

ENSG00000173805	ENSG00000173805	HAP1	17:39775688-39943183	q1	q2	NOTEST	0	0	0	0	1	1	no

ENSG00000173809	ENSG00000173809	TDRD12	19:33210658-33320483	q1	q2	NOTEST	0.0554518	0.0718547	0.373848	0	1	1	no

ENSG00000173810	ENSG00000173810	PPIAP7	1:101736430-101736919	q1	q2	NOTEST	0	0	0	0	1	1	no

ENSG00000173811	ENSG00000173811	CCDC13-AS1	3:42734154-42846023	q1	q2	NOTEST	0.307649	0.216358	-0.507866	0	1	1	no

ENSG00000173812	ENSG00000173812	EIF1	17:39775688-39943183	q1	q2	OK	481.742	456.62	-0.0772656	-0.165024	0.81295	0.99995	no

ENSG00000173818	ENSG00000173818	ENDOV	17:78234664-78411886	q1	q2	OK	18.0867	16.0203	-0.175028	-0.10639	0.7609	0.99995	no

ENSG00000173821	ENSG00000173821	RNF213	17:78234664-78411886	q1	q2	OK	25.4862	25.1979	-0.0164085	-0.0146223	0.9642	0.99995	no

ENSG00000173825	ENSG00000173825	TIGD3	11:65122237-65125084	q1	q2	OK	2.3947	2.13438	-0.166026	-0.212398	0.7606	0.99995	no

ENSG00000173826	ENSG00000173826	KCNH6	17:61600694-61626338	q1	q2	NOTEST	0.0356094	0.025006	-0.509983	0	1	1	no

ENSG00000173838	ENSG00000173838	Mar-10	17:60776604-60885705	q1	q2	NOTEST	0.29543	0.281335	-0.0705281	0	1	1	no

ENSG00000173846	ENSG00000173846	PLK3	1:45265896-45272957	q1	q2	OK	14.4244	12.1932	-0.242436	-0.369124	0.5927	0.99995	no

ENSG00000173848	ENSG00000173848	NET1	10:5454513-5500426	q1	q2	OK	25.058	27.466	0.132378	0.302235	0.6495	0.99995	no

ENSG00000173852	ENSG00000173852	DPY19L1	7:34968487-35077883	q1	q2	OK	16.4989	17.5583	0.0897843	0.170701	0.75215	0.99995	no

ENSG00000173862	ENSG00000173862	RP11-89N17.1	7:33765592-33768068	q1	q2	NOTEST	0.0584065	0	#NAME?	0	1	1	no

ENSG00000173867	ENSG00000173867	RP11-97O12.7	15:89002706-89089906	q1	q2	OK	0.468064	0.785906	0.74765	0.112202	0.6337	0.99995	no

ENSG00000173868	ENSG00000173868	PHOSPHO1	17:47300723-47308128	q1	q2	NOTEST	0.0648766	0.0332513	-0.964289	0	1	1	no

ENSG00000173875	ENSG00000173875	ZNF791	19:12490002-12750912	q1	q2	OK	8.29177	9.85641	0.249382	0.266566	0.6652	0.99995	no

ENSG00000173876	ENSG00000173876	TUBB8	10:92827-120103	q1	q2	NOTEST	0	0	0	0	1	1	no

ENSG00000173889	ENSG00000173889	PHC3	3:169804519-169899537	q1	q2	OK	5.59998	6.08739	0.120404	0.162945	0.69925	0.99995	no

ENSG00000173890	ENSG00000173890	GPR160	3:169755716-169803191	q1	q2	OK	0.528229	0.379152	-0.478387	-0.274218	0.51365	0.99995	no

ENSG00000173894	ENSG00000173894	CBX2	17:77751930-77761782	q1	q2	OK	0.753444	0.149715	-2.33128	-1.46197	0.0075	0.99995	no

ENSG00000173898	ENSG00000173898	SPTBN2	11:66452718-66496697	q1	q2	OK	16.1514	14.9182	-0.11459	-0.112095	0.8315	0.99995	no

ENSG00000173905	ENSG00000173905	GOLIM4	3:167726464-167813763	q1	q2	OK	22.5908	23.006	0.0262744	0.0585378	0.9332	0.99995	no

ENSG00000173908	ENSG00000173908	KRT28	17:38948454-38956211	q1	q2	NOTEST	0	0	0	0	1	1	no

ENSG00000173914	ENSG00000173914	RBM4B	11:66384052-66445392	q1	q2	OK	47.6176	45.5921	-0.0627094	-0.0490829	0.9244	0.99995	no

ENSG00000173915	ENSG00000173915	USMG5	10:105127723-105156223	q1	q2	OK	243.688	283.887	0.220282	0.408657	0.566	0.99995	no

ENSG00000173917	ENSG00000173917	HOXB2	17:46618255-46683776	q1	q2	OK	23.6043	21.8706	-0.110055	-0.141716	0.81185	0.99995	no

ENSG00000173918	ENSG00000173918	C1QTNF1	17:77015290-77045870	q1	q2	OK	1.97461	1.62683	-0.279507	-0.260314	0.6299	0.99995	no

ENSG00000173926	ENSG00000173926	Mar-03	5:126203405-126366500	q1	q2	OK	2.85297	3.36823	0.239524	0.210076	0.7567	0.99995	no

ENSG00000173928	ENSG00000173928	SWSAP1	19:11478798-11487627	q1	q2	OK	1.69513	1.60312	-0.080517	-0.0889191	0.8966	0.99995	no

ENSG00000173930	ENSG00000173930	SLCO4C1	5:101569689-101632253	q1	q2	NOTEST	0.0784434	0.154747	0.980188	0	1	1	no

ENSG00000173933	ENSG00000173933	RBM4	11:66384052-66445392	q1	q2	OK	157.283	152.328	-0.0461804	-0.0723272	0.9142	0.99995	no

ENSG00000173947	ENSG00000173947	PIFO	1:111888909-111895635	q1	q2	OK	1.31875	0.962367	-0.454513	-0.351518	0.6512	0.99995	no

ENSG00000173950	ENSG00000173950	XXYLT1	3:194789007-194991896	q1	q2	OK	30.8923	28.787	-0.101826	-0.227771	0.7372	0.99995	no

ENSG00000173954	ENSG00000173954	SNURFL	X:138444198-138444541	q1	q2	NOTEST	0	0	0	0	1	1	no

ENSG00000173960	ENSG00000173960	UBXN2A	2:24150154-24227779	q1	q2	OK	16.5427	18.1116	0.130717	0.24461	0.7066	0.99995	no

ENSG00000173966	ENSG00000173966	RP11-18M17.1	4:54243809-55164414	q1	q2	NOTEST	0	0	0	0	1	1	no

ENSG00000173976	ENSG00000173976	RAX2	19:3769086-3772233	q1	q2	NOTEST	0	0	0	0	1	1	no

ENSG00000173988	ENSG00000173988	LRRC63	13:46786082-46851501	q1	q2	NOTEST	0	0	0	0	1	1	no

ENSG00000173991	ENSG00000173991	TCAP	17:37820439-37822808	q1	q2	OK	1.51846	1.09701	-0.469029	-0.479456	0.45865	0.99995	no

ENSG00000173992	ENSG00000173992	CCS	11:66357639-66373490	q1	q2	OK	10.2456	10.5648	0.0442673	0.0653782	0.92645	0.99995	no

ENSG00000174004	ENSG00000174004	NRROS	3:196366556-196462878	q1	q2	NOTEST	0.0737486	0.079954	0.116555	0	1	1	no

ENSG00000174007	ENSG00000174007	CEP19	3:196366556-196462878	q1	q2	OK	5.32817	4.49193	-0.246304	-0.263096	0.70315	0.99995	no

ENSG00000174010	ENSG00000174010	KLHL15	X:24001836-24045303	q1	q2	OK	3.44006	3.33616	-0.0442476	-0.0842209	0.9013	0.99995	no

ENSG00000174013	ENSG00000174013	FBXO45	3:196281055-196315930	q1	q2	OK	10.2816	12.0402	0.22779	0.467137	0.5053	0.99995	no

ENSG00000174015	ENSG00000174015	SPERT	13:46276445-46288694	q1	q2	NOTEST	0	0	0	0	1	1	no

ENSG00000174016	ENSG00000174016	FAM46D	X:79591002-79700810	q1	q2	NOTEST	0	0	0	0	1	1	no

ENSG00000174021	ENSG00000174021	GNG5	1:84964007-85040163	q1	q2	OK	140.306	141.884	0.0161362	0.0378864	0.9588	0.99995	no

ENSG00000174028	ENSG00000174028	FAM3C2	X:22277913-23311263	q1	q2	OK	9.76223	13.5246	0.470305	0.654185	0.34945	0.99995	no

ENSG00000174032	ENSG00000174032	SLC25A30	13:45967450-45994506	q1	q2	OK	2.6325	2.83831	0.1086	0.142505	0.79665	0.99995	no

ENSG00000174038	ENSG00000174038	C9orf131	9:35041091-35045988	q1	q2	NOTEST	0.372768	0.390738	0.0679215	0	1	1	no

ENSG00000174059	ENSG00000174059	CD34	1:208057593-208084747	q1	q2	OK	4.71681	4.44982	-0.0840661	-0.128192	0.8508	0.99995	no

ENSG00000174080	ENSG00000174080	CTSF	11:66330933-66336312	q1	q2	OK	7.21596	5.9252	-0.284328	-0.387086	0.51005	0.99995	no

ENSG00000174083	ENSG00000174083	PIK3R6	17:8706040-8770994	q1	q2	NOTEST	0.0204771	0	#NAME?	0	1	1	no

ENSG00000174093	ENSG00000174093	RP11-1407O15.2	17:36337710-36421858	q1	q2	OK	5.55378	5.05724	-0.135121	-0.191762	0.78465	0.99995	no

ENSG00000174099	ENSG00000174099	MSRB3	12:65672422-66036152	q1	q2	OK	8.70128	9.19596	0.0797715	0.144905	0.7923	0.99995	no

ENSG00000174100	ENSG00000174100	MRPL45	17:36452988-36479101	q1	q2	NOTEST	0	0	0	0	1	1	no

ENSG00000174106	ENSG00000174106	LEMD3	12:65563350-65642107	q1	q2	OK	14.998	15.1967	0.0189885	0.0279451	0.9478	0.99995	no

ENSG00000174109	ENSG00000174109	C16orf91	16:1469744-1479345	q1	q2	OK	10.4768	9.05855	-0.209849	-0.314015	0.65195	0.99995	no

ENSG00000174111	ENSG00000174111	SOCS7	17:36508110-36556019	q1	q2	NOTEST	0.250697	0.141972	-0.820338	0	1	1	no

ENSG00000174123	ENSG00000174123	TLR10	4:38773859-38784611	q1	q2	NOTEST	0.0196431	0.0166698	-0.236783	0	1	1	no

ENSG00000174125	ENSG00000174125	TLR1	4:38792297-38858438	q1	q2	NOTEST	0.147415	0.0363057	-2.02162	0	1	1	no

ENSG00000174130	ENSG00000174130	TLR6	4:38792297-38858438	q1	q2	NOTEST	0.0669677	0.0649634	-0.0438371	0	1	1	no

ENSG00000174132	ENSG00000174132	FAM174A	5:99871008-99922445	q1	q2	OK	3.30696	3.64384	0.139956	0.177248	0.7967	0.99995	no

ENSG00000174136	ENSG00000174136	RGMB	5:98104353-98134347	q1	q2	OK	64.3414	66.8231	0.0546002	0.126565	0.8545	0.99995	no

ENSG00000174137	ENSG00000174137	FAM53A	4:1619641-1686029	q1	q2	OK	0.594202	0.429146	-0.469483	-0.338912	0.5902	0.99995	no

ENSG00000174145	ENSG00000174145	KIAA1239	4:37245841-37451087	q1	q2	NOTEST	0.0181502	0.0505917	1.47892	0	1	1	no

ENSG00000174151	ENSG00000174151	CYB561D1	1:110036673-110045554	q1	q2	OK	3.72816	3.83955	0.0424702	0.0604768	0.9144	0.99995	no

ENSG00000174156	ENSG00000174156	GSTA3	6:52761436-52774483	q1	q2	NOTEST	0	0	0	0	1	1	no

ENSG00000174165	ENSG00000174165	ZDHHC24	11:66234215-66313709	q1	q2	OK	2.83691	2.33906	-0.27839	-0.13189	0.86475	0.99995	no

ENSG00000174171	ENSG00000174171	RP11-23P13.6	15:42120282-42190773	q1	q2	OK	1.41265	1.63855	0.214015	0.0506453	0.91795	0.99995	no

ENSG00000174173	ENSG00000174173	TRMT10C	3:101280705-101285290	q1	q2	OK	25.5643	25.8726	0.0172985	0.0381433	0.9545	0.99995	no

ENSG00000174175	ENSG00000174175	SELP	1:169558086-169599431	q1	q2	NOTEST	0.0113526	0	#NAME?	0	1	1	no

ENSG00000174177	ENSG00000174177	CTU2	16:88772870-88851619	q1	q2	OK	9.05228	9.20194	0.0236567	0.0213795	0.97125	0.99995	no

ENSG00000174194	ENSG00000174194	AGAP8	10:51224680-51371338	q1	q2	OK	1.87547	1.89265	0.0131505	0.0155223	0.9797	0.99995	no

ENSG00000174196	ENSG00000174196	FAM21D	10:51187937-51192032	q1	q2	NOTEST	0	0	0	0	1	1	no

ENSG00000174197	ENSG00000174197	MGA	15:41913421-42062141	q1	q2	OK	10.0837	11.2623	0.159466	0.245762	0.6022	0.99995	no

ENSG00000174206	ENSG00000174206	C12orf66	12:64580095-64616076	q1	q2	OK	1.50588	1.68387	0.161167	0.180829	0.7776	0.99995	no

ENSG00000174225	ENSG00000174225	ARL13A	X:100224696-100245818	q1	q2	NOTEST	0.0355512	0.0184599	-0.945508	0	1	1	no

ENSG00000174226	ENSG00000174226	SNX31	8:101585115-101675643	q1	q2	NOTEST	0.0111149	0	#NAME?	0	1	1	no

ENSG00000174227	ENSG00000174227	PIGG	4:492988-533985	q1	q2	OK	30.2942	27.6208	-0.133287	-0.228404	0.67335	0.99995	no

ENSG00000174231	ENSG00000174231	PRPF8	17:1553922-1588176	q1	q2	OK	90.7585	89.3336	-0.0228309	-0.0305119	0.9406	0.99995	no

ENSG00000174233	ENSG00000174233	ADCY6	12:49159974-49182820	q1	q2	OK	32.0922	32.4337	0.01527	0.0190833	0.9616	0.99995	no

ENSG00000174236	ENSG00000174236	REP15	12:27849320-27863703	q1	q2	NOTEST	0.348729	0.463434	0.410257	0	1	1	no

ENSG00000174238	ENSG00000174238	PITPNA	17:1420224-1466110	q1	q2	OK	32.4845	32.5859	0.00449633	0.00866059	0.9868	0.99995	no

ENSG00000174243	ENSG00000174243	DDX23	12:49223546-49246625	q1	q2	OK	47.8529	47.3491	-0.0152676	-0.0258658	0.9573	0.99995	no

ENSG00000174255	ENSG00000174255	ZNF80	3:113933159-113956425	q1	q2	NOTEST	0.0231802	0.0256967	0.148688	0	1	1	no

ENSG00000174276	ENSG00000174276	ZNHIT2	11:64883874-64885170	q1	q2	OK	7.0121	6.96911	-0.00887233	-0.0133825	0.9848	0.99995	no

ENSG00000174279	ENSG00000174279	EVX2	2:176942199-176948641	q1	q2	NOTEST	0.00624775	0.0176404	1.49747	0	1	1	no

ENSG00000174282	ENSG00000174282	ZBTB4	17:7362684-7387582	q1	q2	OK	7.96902	7.11353	-0.163836	-0.361133	0.6165	0.99995	no

ENSG00000174292	ENSG00000174292	TNK1	17:7283852-7307456	q1	q2	OK	0.763993	0.890604	0.221226	0.0547327	0.8194	0.99995	no

ENSG00000174306	ENSG00000174306	ZHX3	20:39657457-39946312	q1	q2	OK	6.51332	6.89546	0.0822539	0.0173825	0.93745	0.99995	no

ENSG00000174307	ENSG00000174307	PHLDA3	1:201434619-201438365	q1	q2	OK	19.9625	13.9231	-0.519809	-0.950013	0.1528	0.99995	no

ENSG00000174325	ENSG00000174325	DIRC1	2:189463517-189704171	q1	q2	NOTEST	0	0.029398	inf	0	1	1	no

ENSG00000174326	ENSG00000174326	SLC16A11	17:6944948-6947242	q1	q2	NOTEST	0.199231	0.0775936	-1.36043	0	1	1	no

ENSG00000174327	ENSG00000174327	SLC16A13	17:6939393-6943440	q1	q2	OK	3.38201	2.91232	-0.215709	-0.274251	0.7028	0.99995	no

ENSG00000174332	ENSG00000174332	GLIS1	1:53971909-54199877	q1	q2	NOTEST	0.0817624	0.0622594	-0.393146	0	1	1	no

ENSG00000174339	ENSG00000174339	OR2Y1	5:180166041-180167099	q1	q2	NOTEST	0	0	0	0	1	1	no

ENSG00000174343	ENSG00000174343	CHRNA9	4:40337345-40357234	q1	q2	NOTEST	0.0469664	0.0783884	0.739011	0	1	1	no

ENSG00000174348	ENSG00000174348	PODN	1:53527853-53551174	q1	q2	OK	1.2565	1.09166	-0.202893	-0.201472	0.75465	0.99995	no

ENSG00000174353	ENSG00000174353	STAG3L3	7:72440226-72555749	q1	q2	NOTEST	0.193117	0.295163	0.612037	0	1	1	no

ENSG00000174358	ENSG00000174358	SLC6A19	5:1201709-1225232	q1	q2	NOTEST	0	0	0	0	1	1	no

ENSG00000174365	ENSG00000174365	SNHG11	20:37075220-37079564	q1	q2	OK	12.4906	9.69893	-0.364951	-0.548553	0.48565	0.99995	no

ENSG00000174368	ENSG00000174368	PMS2P2	7:74880904-74988262	q1	q2	NOTEST	0.10764	0	#NAME?	0	1	1	no

ENSG00000174370	ENSG00000174370	C11orf45	11:128761250-128790930	q1	q2	NOTEST	0.166477	0.165556	-0.0079985	0	1	1	no

ENSG00000174371	ENSG00000174371	EXO1	1:242011268-242058450	q1	q2	OK	16.2855	16.4661	0.0159125	0.0297379	0.96045	0.99995	no

ENSG00000174373	ENSG00000174373	RALGAPA1	14:36007557-36278510	q1	q2	OK	11.2204	10.4236	-0.106262	-0.157889	0.7695	0.99995	no

ENSG00000174374	ENSG00000174374	WBSCR16	7:74441225-74490064	q1	q2	OK	2.81042	1.73535	-0.695563	-0.907711	0.1638	0.99995	no

ENSG00000174384	ENSG00000174384	RP11-313P13.4	7:72440226-72555749	q1	q2	NOTEST	0	0	0	0	1	1	no

ENSG00000174403	ENSG00000174403	C20orf166-AS1	20:61141549-61167971	q1	q2	NOTEST	0	0	0	0	1	1	no

ENSG00000174405	ENSG00000174405	LIG4	13:108859786-108870716	q1	q2	OK	10.411	12.2319	0.232548	0.520499	0.4556	0.99995	no

ENSG00000174407	ENSG00000174407	C20orf166	20:61141549-61167971	q1	q2	NOTEST	0	0	0	0	1	1	no

ENSG00000174408	ENSG00000174408	PPIAP24	13:107516163-107516634	q1	q2	NOTEST	0	0	0	0	1	1	no

ENSG00000174417	ENSG00000174417	TRHR	8:110098849-110131813	q1	q2	NOTEST	0.0206071	0.059551	1.53098	0	1	1	no

ENSG00000174418	ENSG00000174418	RPL35P9	13:107078120-107078470	q1	q2	NOTEST	0	0	0	0	1	1	no

ENSG00000174428	ENSG00000174428	GTF2IRD2B	7:74508363-74565623	q1	q2	NOTEST	0.199945	0.256594	0.359888	0	1	1	no

ENSG00000174429	ENSG00000174429	ABRA	8:107771710-107782473	q1	q2	NOTEST	0	0.00965727	inf	0	1	1	no

ENSG00000174437	ENSG00000174437	ATP2A2	12:110718560-110788898	q1	q2	OK	100.114	106.588	0.090401	0.169668	0.76325	0.99995	no

ENSG00000174442	ENSG00000174442	ZWILCH	15:66790354-66858317	q1	q2	OK	23.3722	25.2227	0.109929	0.0368511	0.94815	0.99995	no

ENSG00000174444	ENSG00000174444	RPL4	15:66790354-66858317	q1	q2	OK	1183.74	1172.18	-0.0141675	-0.0214717	0.97325	0.99995	no

ENSG00000174446	ENSG00000174446	SNAPC5	15:66679154-66790151	q1	q2	OK	15.868	14.7828	-0.102207	-0.0566082	0.9351	0.99995	no

ENSG00000174448	ENSG00000174448	STARD6	18:51850727-51911588	q1	q2	NOTEST	0.265489	0.291562	0.135149	0	1	1	no

ENSG00000174450	ENSG00000174450	GOLGA6L2	15:23684644-23692381	q1	q2	NOTEST	0.0547685	0.0304473	-0.847032	0	1	1	no

ENSG00000174453	ENSG00000174453	VWC2L	2:214149112-215548970	q1	q2	NOTEST	0.345645	0.197636	-0.806441	0	1	1	no

ENSG00000174456	ENSG00000174456	C12orf76	12:110436990-110511491	q1	q2	OK	17.6388	14.4969	-0.283007	-0.25126	0.68045	0.99995	no

ENSG00000174460	ENSG00000174460	ZCCHC12	X:117957752-117960931	q1	q2	OK	3.77774	2.88827	-0.387316	-0.556953	0.4222	0.99995	no

ENSG00000174469	ENSG00000174469	CNTNAP2	7:145813452-148118090	q1	q2	OK	8.19543	7.64182	-0.100905	-0.190428	0.7775	0.99995	no

ENSG00000174473	ENSG00000174473	GALNTL6	4:172733404-173962710	q1	q2	OK	0.555837	0.493755	-0.170866	-0.156332	0.8217	0.99995	no

ENSG00000174482	ENSG00000174482	LINGO2	9:27948075-28670283	q1	q2	NOTEST	0.464607	0.357041	-0.37992	0	1	1	no

ENSG00000174483	ENSG00000174483	BBS1	11:66234215-66313709	q1	q2	OK	22.6496	23.3483	0.0438329	0.0410911	0.9291	0.99995	no

ENSG00000174485	ENSG00000174485	DENND4A	15:65903703-66184329	q1	q2	OK	4.36909	4.71205	0.109023	0.0373521	0.9076	0.99995	no

ENSG00000174495	ENSG00000174495	AC005017.2	7:28318829-28319319	q1	q2	NOTEST	0	0	0	0	1	1	no

ENSG00000174498	ENSG00000174498	IGDCC3	15:65619464-65670378	q1	q2	OK	62.3848	57.5475	-0.116441	-0.224138	0.70815	0.99995	no

ENSG00000174500	ENSG00000174500	GCSAM	3:111805181-111852152	q1	q2	NOTEST	0.186761	0.177506	-0.0733274	0	1	1	no

ENSG00000174501	ENSG00000174501	ANKRD36C	2:96514586-96657541	q1	q2	OK	4.07512	3.73985	-0.123862	-0.19872	0.77485	0.99995	no

ENSG00000174502	ENSG00000174502	SLC26A9	1:205882175-205912588	q1	q2	NOTEST	0.031044	0.116008	1.90184	0	1	1	no

ENSG00000174514	ENSG00000174514	MFSD4	1:205538012-205572046	q1	q2	OK	0.649976	0.482969	-0.428456	-0.271978	0.53585	0.99995	no

ENSG00000174516	ENSG00000174516	PELI3	11:66234215-66313709	q1	q2	OK	4.63136	4.09274	-0.17837	-0.112895	0.88515	0.99995	no

ENSG00000174521	ENSG00000174521	TTC9B	19:40721964-40724306	q1	q2	OK	0.971887	0.69958	-0.4743	-0.412219	0.58535	0.99995	no

ENSG00000174527	ENSG00000174527	MYO1H	12:109785707-109974507	q1	q2	NOTEST	0.011609	0.291409	4.64973	0	1	1	no

ENSG00000174529	ENSG00000174529	TMEM81	1:205052257-205053645	q1	q2	OK	4.22967	4.37439	0.0485361	0.0663612	0.92285	0.99995	no

ENSG00000174547	ENSG00000174547	MRPL11	11:66202545-66234209	q1	q2	OK	40.1722	37.3614	-0.104646	-0.20249	0.77165	0.99995	no

ENSG00000174562	ENSG00000174562	KLK15	19:51328544-51354345	q1	q2	NOTEST	0	0	0	0	1	1	no

ENSG00000174564	ENSG00000174564	IL20RB	3:136581049-136729927	q1	q2	OK	0.855747	0.763268	-0.164994	-0.0709434	0.9104	0.99995	no

ENSG00000174567	ENSG00000174567	GOLT1A	1:204167287-204183220	q1	q2	NOTEST	0.0367081	0.252553	2.78241	0	1	1	no

ENSG00000174572	ENSG00000174572	RP11-209A2.1	6:27179022-27179692	q1	q2	NOTEST	0.0534137	0.0521845	-0.0335885	0	1	1	no

ENSG00000174574	ENSG00000174574	AKIRIN1	1:39456894-39471731	q1	q2	OK	79.7482	87.0582	0.126528	0.289138	0.6803	0.99995	no

ENSG00000174576	ENSG00000174576	NPAS4	11:66188474-66194178	q1	q2	OK	0.595847	0.486846	-0.291476	-0.235255	0.689	0.99995	no

ENSG00000174579	ENSG00000174579	MSL2	3:135867763-135916083	q1	q2	OK	19.2951	18.4688	-0.0631489	-0.0973101	0.85005	0.99995	no

ENSG00000174586	ENSG00000174586	ZNF497	19:58856543-58877958	q1	q2	OK	5.7372	4.15084	-0.466942	-0.528041	0.38635	0.99995	no

ENSG00000174595	ENSG00000174595	KLF14	7:130417400-130418888	q1	q2	NOTEST	0	0	0	0	1	1	no

ENSG00000174599	ENSG00000174599	TRAM1L1	4:118004717-118006736	q1	q2	OK	2.7344	2.89277	0.081228	0.110984	0.87555	0.99995	no

ENSG00000174600	ENSG00000174600	CMKLR1	12:108681820-108733118	q1	q2	OK	6.31642	6.61976	0.0676721	0.102955	0.88025	0.99995	no

ENSG00000174606	ENSG00000174606	ANGEL2	1:213165523-213189168	q1	q2	OK	19.5767	21.5012	0.13528	0.180331	0.70755	0.99995	no

ENSG00000174607	ENSG00000174607	UGT8	4:115519610-115599380	q1	q2	OK	1.64473	1.55083	-0.0848118	-0.109853	0.8676	0.99995	no

ENSG00000174611	ENSG00000174611	KY	3:134316642-134979309	q1	q2	NOTEST	0.0517796	0.0401252	-0.367876	0	1	1	no

ENSG00000174628	ENSG00000174628	IQCK	16:19566561-19897489	q1	q2	OK	10.9341	11.7196	0.100092	0.0549799	0.91595	0.99995	no

ENSG00000174640	ENSG00000174640	SLCO2A1	3:133651539-133771028	q1	q2	NOTEST	0.023913	0.0429063	0.843396	0	1	1	no

ENSG00000174652	ENSG00000174652	ZNF266	19:9523271-9546254	q1	q2	OK	25.4435	23.8118	-0.0956199	-0.164134	0.79415	0.99995	no

ENSG00000174667	ENSG00000174667	OR7D4	19:9324525-9325542	q1	q2	NOTEST	0	0	0	0	1	1	no

ENSG00000174669	ENSG00000174669	SLC29A2	11:66115420-66139685	q1	q2	OK	5.22081	5.25682	0.00991523	0.0159062	0.9825	0.99995	no

ENSG00000174672	ENSG00000174672	BRSK2	11:1411128-1483919	q1	q2	OK	29.2332	26.4978	-0.141735	-0.270268	0.6731	0.99995	no

ENSG00000174677	ENSG00000174677	VN1R6P	19:53818389-53819296	q1	q2	NOTEST	0.033568	0	#NAME?	0	1	1	no

ENSG00000174678	ENSG00000174678	FAM47DP	X:37208527-38548169	q1	q2	NOTEST	0	0	0	0	1	1	no

ENSG00000174680	ENSG00000174680	GRIK1-AS1	21:30565800-31312351	q1	q2	NOTEST	0.284789	0.404869	0.507562	0	1	1	no

ENSG00000174684	ENSG00000174684	B3GNT1	11:66112842-66115163	q1	q2	OK	26.3356	23.1274	-0.187409	-0.422506	0.5411	0.99995	no

ENSG00000174695	ENSG00000174695	TMEM167A	5:82348664-82649606	q1	q2	OK	50.1921	54.5997	0.121431	0.192971	0.69445	0.99995	no

ENSG00000174697	ENSG00000174697	LEP	7:127881336-127897681	q1	q2	NOTEST	0	0	0	0	1	1	no

ENSG00000174705	ENSG00000174705	SH3PXD2B	5:171752184-171881527	q1	q2	OK	28.7667	29.4051	0.0316687	0.0724707	0.911	0.99995	no

ENSG00000174715	ENSG00000174715	RP11-79L9.2	3:138362715-138363536	q1	q2	NOTEST	0.23397	0.160529	-0.54349	0	1	1	no

ENSG00000174718	ENSG00000174718	KIAA1551	12:32112303-32146039	q1	q2	OK	16.6246	17.0941	0.0401838	0.0427147	0.9338	0.99995	no

ENSG00000174720	ENSG00000174720	LARP7	4:113434671-113578748	q1	q2	OK	50.8304	52.9628	0.0592883	0.0906478	0.86745	0.99995	no

ENSG00000174721	ENSG00000174721	FGFBP3	10:93666345-93669240	q1	q2	OK	137.554	136.344	-0.0127502	-0.0301993	0.9645	0.99995	no

ENSG00000174738	ENSG00000174738	NR1D2	3:23933150-24022109	q1	q2	OK	9.21711	8.82611	-0.0625376	-0.0271248	0.9538	0.99995	no

ENSG00000174740	ENSG00000174740	PABPC5	X:90669876-90693583	q1	q2	OK	3.86825	3.21025	-0.268994	-0.411821	0.54175	0.99995	no

ENSG00000174744	ENSG00000174744	BRMS1	11:66097712-66112596	q1	q2	OK	20.4946	20.6652	0.011958	0.0223398	0.97385	0.99995	no

ENSG00000174748	ENSG00000174748	RPL15	3:23933150-24022109	q1	q2	OK	639.965	576.547	-0.150556	-0.256696	0.7062	0.99995	no

ENSG00000174749	ENSG00000174749	C4orf32	4:113066552-113116412	q1	q2	NOTEST	0.177317	0.148021	-0.260528	0	1	1	no

ENSG00000174775	ENSG00000174775	HRAS	11:532241-537287	q1	q2	OK	43.7802	43.9512	0.00562485	0.0107299	0.98745	0.99995	no

ENSG00000174776	ENSG00000174776	WDR49	3:167159576-167371771	q1	q2	OK	2.13362	3.06691	0.52348	0.394025	0.4716	0.99995	no

ENSG00000174780	ENSG00000174780	SRP72	4:57333080-57369839	q1	q2	OK	81.4163	87.2649	0.100084	0.219846	0.73855	0.99995	no

ENSG00000174788	ENSG00000174788	PCP2	19:7694622-7712759	q1	q2	NOTEST	0.0412111	0	#NAME?	0	1	1	no

ENSG00000174791	ENSG00000174791	RIN1	11:66097712-66112596	q1	q2	OK	2.24931	2.02417	-0.152156	-0.0886768	0.86575	0.99995	no

ENSG00000174792	ENSG00000174792	C4orf26	4:76481257-76491095	q1	q2	NOTEST	0.0745325	0.222116	1.57537	0	1	1	no

ENSG00000174796	ENSG00000174796	THAP6	4:76404246-76475683	q1	q2	OK	8.74811	8.02673	-0.124159	-0.116713	0.8294	0.99995	no

ENSG00000174799	ENSG00000174799	CEP135	4:56815036-56899529	q1	q2	OK	10.7528	11.4169	0.0864666	0.168992	0.8081	0.99995	no

ENSG00000174804	ENSG00000174804	FZD4	11:86502100-86666433	q1	q2	OK	2.67632	3.05191	0.18946	0.184187	0.802	0.99995	no

ENSG00000174807	ENSG00000174807	CD248	11:66080323-66086708	q1	q2	OK	8.4637	6.35502	-0.413392	-0.710012	0.30935	0.99995	no

ENSG00000174808	ENSG00000174808	BTC	4:75669968-75719896	q1	q2	NOTEST	0	0	0	0	1	1	no

ENSG00000174827	ENSG00000174827	PDZK1	1:145726917-145764074	q1	q2	NOTEST	0.0292981	0	#NAME?	0	1	1	no

ENSG00000174837	ENSG00000174837	EMR1	19:6887576-6940470	q1	q2	NOTEST	0.0100235	0	#NAME?	0	1	1	no

ENSG00000174839	ENSG00000174839	DENND6A	3:57611183-57678816	q1	q2	OK	11.6166	12.441	0.0989161	0.153093	0.7399	0.99995	no

ENSG00000174840	ENSG00000174840	PDE12	3:57542002-57552571	q1	q2	OK	6.8834	6.69641	-0.0397335	-0.074426	0.91495	0.99995	no

ENSG00000174842	ENSG00000174842	GLMN	1:92711958-92867613	q1	q2	OK	17.9187	21.0334	0.231217	0.257097	0.71465	0.99995	no

ENSG00000174844	ENSG00000174844	DNAH12	3:57327726-57530071	q1	q2	NOTEST	0.10057	0.0975903	-0.0433928	0	1	1	no

ENSG00000174851	ENSG00000174851	YIF1A	11:66036003-66056641	q1	q2	OK	32.2227	29.4449	-0.130062	-0.10942	0.8396	0.99995	no

ENSG00000174871	ENSG00000174871	CNIH2	11:66036003-66056641	q1	q2	OK	32.1792	25.8345	-0.316832	-0.29289	0.66825	0.99995	no

ENSG00000174876	ENSG00000174876	AMY1B	1:104230036-104239302	q1	q2	NOTEST	0	0	0	0	1	1	no

ENSG00000174885	ENSG00000174885	NLRP6	11:278364-285359	q1	q2	NOTEST	0.00957127	0	#NAME?	0	1	1	no

ENSG00000174886	ENSG00000174886	NDUFA11	19:5865836-5904017	q1	q2	OK	134.353	122.086	-0.138125	-0.29027	0.6766	0.99995	no

ENSG00000174891	ENSG00000174891	RSRC1	3:157814947-158288855	q1	q2	OK	21.9184	24.0959	0.136647	0.222086	0.7496	0.99995	no

ENSG00000174898	ENSG00000174898	CATSPERD	19:5720687-5778745	q1	q2	NOTEST	0	0	0	0	1	1	no

ENSG00000174899	ENSG00000174899	C3orf55	3:157261034-157395538	q1	q2	NOTEST	0.0238019	0	#NAME?	0	1	1	no

ENSG00000174903	ENSG00000174903	RAB1B	11:66036003-66056641	q1	q2	OK	73.9926	69.221	-0.0961712	-0.198183	0.7732	0.99995	no

ENSG00000174912	ENSG00000174912	METTL15P1	3:156431672-156432717	q1	q2	OK	1.96634	1.59567	-0.301354	-0.284594	0.6815	0.99995	no

ENSG00000174914	ENSG00000174914	OR9G1	11:56467863-56468781	q1	q2	NOTEST	0	0	0	0	1	1	no

ENSG00000174915	ENSG00000174915	PTDSS2	11:448267-491393	q1	q2	OK	16.963	14.1133	-0.265335	-0.401926	0.4612	0.99995	no

ENSG00000174917	ENSG00000174917	C19orf70	19:5674957-5720583	q1	q2	OK	14.7102	13.2694	-0.148719	-0.0714512	0.91145	0.99995	no

ENSG00000174928	ENSG00000174928	C3orf33	3:155480400-155524140	q1	q2	OK	2.17964	2.3245	0.0928279	0.116991	0.86245	0.99995	no

ENSG00000174930	ENSG00000174930	VN2R1P	3:155737434-155755475	q1	q2	NOTEST	0	0	0	0	1	1	no

ENSG00000174937	ENSG00000174937	OR5M3	11:56236963-56238014	q1	q2	NOTEST	0	0	0	0	1	1	no

ENSG00000174938	ENSG00000174938	SEZ6L2	16:29882479-29910868	q1	q2	OK	2.74883	2.05275	-0.421259	-0.569296	0.36515	0.99995	no

ENSG00000174939	ENSG00000174939	ASPHD1	16:29911695-29940254	q1	q2	OK	1.45138	0.983939	-0.560788	-0.283476	0.76485	0.99995	no

ENSG00000174942	ENSG00000174942	OR5R1	11:56184733-56185708	q1	q2	NOTEST	0	0	0	0	1	1	no

ENSG00000174943	ENSG00000174943	KCTD13	16:29911695-29940254	q1	q2	OK	9.38319	8.59304	-0.126909	-0.129477	0.81325	0.99995	no

ENSG00000174944	ENSG00000174944	P2RY14	3:150803483-151176497	q1	q2	NOTEST	0.0308626	0.0291677	-0.0814896	0	1	1	no

ENSG00000174945	ENSG00000174945	AMZ1	7:2719155-2883958	q1	q2	NOTEST	0.204434	0.255382	0.321021	0	1	1	no

ENSG00000174946	ENSG00000174946	GPR171	3:150803483-151176497	q1	q2	NOTEST	0	0	0	0	1	1	no

ENSG00000174948	ENSG00000174948	GPR149	3:154055460-154147504	q1	q2	NOTEST	0	0	0	0	1	1	no

ENSG00000174950	ENSG00000174950	CD164L2	1:27705665-27709870	q1	q2	NOTEST	0	0	0	0	1	1	no

ENSG00000174951	ENSG00000174951	FUT1	19:49251267-49258647	q1	q2	NOTEST	0.0351886	0.077754	1.14381	0	1	1	no

ENSG00000174953	ENSG00000174953	DHX36	3:153990334-154042286	q1	q2	OK	55.1283	55.2604	0.00345187	0.00510591	0.9935	0.99995	no

ENSG00000174957	ENSG00000174957	OR5J2	11:55944093-55945032	q1	q2	NOTEST	0	0	0	0	1	1	no

ENSG00000174963	ENSG00000174963	ZIC4	3:147103832-147228080	q1	q2	OK	3.14599	2.54803	-0.304134	-0.136576	0.68445	0.99995	no

ENSG00000174970	ENSG00000174970	OR10AG1	11:55734974-55735990	q1	q2	NOTEST	0	0	0	0	1	1	no

ENSG00000174977	ENSG00000174977	AC026271.5	17:18538318-18585575	q1	q2	OK	3.20008	4.05925	0.343107	0.231232	0.75555	0.99995	no

ENSG00000174982	ENSG00000174982	OR4S2	11:55418379-55419315	q1	q2	NOTEST	0	0	0	0	1	1	no

ENSG00000174989	ENSG00000174989	FBXW8	12:117348760-117468953	q1	q2	OK	9.25851	9.66668	0.062241	0.129318	0.84985	0.99995	no

ENSG00000174990	ENSG00000174990	CA5A	16:87921624-87970135	q1	q2	NOTEST	0.0774839	0	#NAME?	0	1	1	no

ENSG00000174992	ENSG00000174992	ZG16	16:29789560-29793096	q1	q2	NOTEST	0	0	0	0	1	1	no

ENSG00000174996	ENSG00000174996	KLC2	11:66024764-66035600	q1	q2	OK	25.8171	25.2881	-0.0298695	-0.0491642	0.92235	0.99995	no

ENSG00000175003	ENSG00000175003	SLC22A1	6:160542820-160579750	q1	q2	OK	0.433951	0.780535	0.84693	0.632803	0.40995	0.99995	no

ENSG00000175018	ENSG00000175018	TEX36	10:127262939-127371713	q1	q2	NOTEST	0.166208	0.119391	-0.477287	0	1	1	no

ENSG00000175029	ENSG00000175029	CTBP2	10:126605711-126849739	q1	q2	OK	77.0337	71.8054	-0.101397	-0.158124	0.775	0.99995	no

ENSG00000175040	ENSG00000175040	CHST2	3:142838172-142841800	q1	q2	OK	10.3133	8.6643	-0.251353	-0.48987	0.4775	0.99995	no

ENSG00000175048	ENSG00000175048	ZDHHC14	6:157802164-158099178	q1	q2	OK	2.36732	2.84194	0.263622	0.250338	0.6835	0.99995	no

ENSG00000175054	ENSG00000175054	ATR	3:142168076-142297668	q1	q2	OK	11.946	13.4785	0.174132	0.175076	0.7118	0.99995	no

ENSG00000175061	ENSG00000175061	FAM211A-AS1	17:16342135-16395467	q1	q2	OK	294.754	287.2	-0.0374574	-0.0859784	0.9013	0.99995	no

ENSG00000175063	ENSG00000175063	UBE2C	20:44441214-44445596	q1	q2	OK	127.114	132.263	0.0572889	0.134253	0.8451	0.99995	no

ENSG00000175065	ENSG00000175065	DSG4	18:28898051-29006951	q1	q2	NOTEST	0	0	0	0	1	1	no

ENSG00000175066	ENSG00000175066	GK5	3:141882413-141944449	q1	q2	OK	5.51935	5.6577	0.0357169	0.0449277	0.93285	0.99995	no

ENSG00000175073	ENSG00000175073	VCPIP1	8:67540721-67579452	q1	q2	OK	3.55386	3.83731	0.110709	0.236451	0.7429	0.99995	no

ENSG00000175077	ENSG00000175077	RTP1	3:186914877-186925417	q1	q2	NOTEST	0.0842763	0.0831454	-0.0194912	0	1	1	no

ENSG00000175084	ENSG00000175084	DES	2:220283098-220291461	q1	q2	NOTEST	0.095815	0.0581269	-0.721046	0	1	1	no

ENSG00000175087	ENSG00000175087	PDIK1L	1:26437663-26452034	q1	q2	OK	9.06036	8.71855	-0.0554796	-0.102267	0.87365	0.99995	no

ENSG00000175093	ENSG00000175093	SPSB4	3:140770243-140867453	q1	q2	OK	37.7808	33.3617	-0.17946	-0.389942	0.5622	0.99995	no

ENSG00000175097	ENSG00000175097	RAG2	11:36532258-36694823	q1	q2	NOTEST	0.0968407	0.222764	1.20183	0	1	1	no

ENSG00000175104	ENSG00000175104	TRAF6	11:36508576-36531822	q1	q2	OK	6.05813	6.46317	0.0933695	0.163346	0.8037	0.99995	no

ENSG00000175105	ENSG00000175105	ZNF654	3:88101093-88217879	q1	q2	OK	4.42138	4.78436	0.113829	0.05908	0.9347	0.99995	no

ENSG00000175106	ENSG00000175106	TVP23C	17:15339331-15466909	q1	q2	OK	3.75571	6.44719	0.779586	0.608338	0.28355	0.99995	no

ENSG00000175110	ENSG00000175110	MRPS22	3:138722803-139108574	q1	q2	OK	35.1487	35.3318	0.00749623	0.00663708	0.99175	0.99995	no

ENSG00000175115	ENSG00000175115	PACS1	11:65837833-66012218	q1	q2	OK	16.4055	14.1468	-0.213705	-0.353008	0.45895	0.99995	no

ENSG00000175121	ENSG00000175121	WFDC5	20:43738092-43743813	q1	q2	NOTEST	0	0	0	0	1	1	no

ENSG00000175130	ENSG00000175130	MARCKSL1	1:32799432-32801980	q1	q2	OK	650.378	643.138	-0.0161516	-0.0339635	0.96155	0.99995	no

ENSG00000175137	ENSG00000175137	SH3BP5L	1:249104647-249120832	q1	q2	OK	13.5624	12.5873	-0.107636	-0.199395	0.7378	0.99995	no

ENSG00000175143	ENSG00000175143	OR2T1	1:248569295-248570405	q1	q2	NOTEST	0	0	0	0	1	1	no

ENSG00000175147	ENSG00000175147	TMEM51-AS1	1:14925199-15478960	q1	q2	OK	0.584314	0	#NAME?	#NAME?	0.1407	0.99995	no

ENSG00000175155	ENSG00000175155	YPEL2	17:57409049-57480664	q1	q2	OK	3.1078	2.81614	-0.142173	-0.159466	0.71805	0.99995	no

ENSG00000175161	ENSG00000175161	CADM2	3:85008131-86123579	q1	q2	OK	7.33465	5.95454	-0.300737	-0.377087	0.5691	0.99995	no

ENSG00000175164	ENSG00000175164	ABO	9:136125787-136150617	q1	q2	NOTEST	7.13E-07	7.89E-07	0.145386	0	1	1	no

ENSG00000175166	ENSG00000175166	PSMD2	3:183851806-184402546	q1	q2	OK	171.743	165.414	-0.0541663	-0.0336765	0.9351	0.99995	no

ENSG00000175170	ENSG00000175170	FAM182B	20:25744101-25848861	q1	q2	OK	1.49631	1.73324	0.212062	0.170083	0.73895	0.99995	no

ENSG00000175175	ENSG00000175175	PPM1E	17:56833229-57058983	q1	q2	OK	9.73259	11.6998	0.265589	0.53713	0.4441	0.99995	no

ENSG00000175182	ENSG00000175182	FAM131A	3:183851806-184402546	q1	q2	OK	11.5303	10.7589	-0.099905	-0.0214466	0.9136	0.99995	no

ENSG00000175183	ENSG00000175183	CSRP2	12:77252494-77274132	q1	q2	OK	136.792	146.008	0.0940631	0.213039	0.7552	0.99995	no

ENSG00000175189	ENSG00000175189	INHBC	12:57828542-57844611	q1	q2	NOTEST	0.0830675	0.0651606	-0.350285	0	1	1	no

ENSG00000175193	ENSG00000175193	PARL	3:183547172-183606237	q1	q2	OK	39.5099	39.3246	-0.00678227	-0.0134778	0.98425	0.99995	no

ENSG00000175197	ENSG00000175197	DDIT3	12:57853917-57914300	q1	q2	OK	19.3836	15.1811	-0.352554	-0.193037	0.81455	0.99995	no

ENSG00000175198	ENSG00000175198	PCCA	13:100741268-101182686	q1	q2	OK	15.165	13.7593	-0.140339	-0.209984	0.7035	0.99995	no

ENSG00000175202	ENSG00000175202	HIGD2B	15:72968123-72978490	q1	q2	NOTEST	0	0.0268721	inf	0	1	1	no

ENSG00000175203	ENSG00000175203	DCTN2	12:57914492-57941114	q1	q2	OK	142.664	140.403	-0.0230469	-0.033114	0.9562	0.99995	no

ENSG00000175206	ENSG00000175206	NPPA	1:11821843-11908402	q1	q2	OK	0.411186	0.621983	0.597085	0.0676919	0.6897	0.99995	no

ENSG00000175213	ENSG00000175213	ZNF408	11:46722367-46727462	q1	q2	OK	5.15869	5.73318	0.152329	0.18791	0.7287	0.99995	no

ENSG00000175215	ENSG00000175215	CTDSP2	12:58213709-58240522	q1	q2	OK	60.3082	56.7523	-0.087675	-0.145351	0.7693	0.99995	no

ENSG00000175216	ENSG00000175216	CKAP5	11:46764597-46867847	q1	q2	OK	95.0826	106.801	0.167665	0.234367	0.61505	0.99995	no

ENSG00000175220	ENSG00000175220	ARHGAP1	11:46698629-46722165	q1	q2	OK	15.5939	12.999	-0.262581	-0.516126	0.41655	0.99995	no

ENSG00000175221	ENSG00000175221	MED16	19:867961-893218	q1	q2	OK	23.1947	20.1409	-0.203664	-0.440858	0.49865	0.99995	no

ENSG00000175224	ENSG00000175224	ATG13	11:46624410-46696368	q1	q2	OK	28.0063	24.9349	-0.167584	-0.232881	0.66115	0.99995	no

ENSG00000175229	ENSG00000175229	GAL3ST3	11:65809422-65816651	q1	q2	OK	3.5619	3.38191	-0.074809	-0.100475	0.8751	0.99995	no

ENSG00000175262	ENSG00000175262	C1orf127	1:11006527-11042094	q1	q2	NOTEST	0.0114889	0.0119445	0.056107	0	1	1	no

ENSG00000175264	ENSG00000175264	CHST1	11:45670426-45687172	q1	q2	OK	4.79661	5.24418	0.128702	0.202538	0.7699	0.99995	no

ENSG00000175265	ENSG00000175265	GOLGA8A	15:34671268-34880704	q1	q2	OK	31.1576	31.1808	0.00107789	0.00125692	0.99815	0.99995	no

ENSG00000175267	ENSG00000175267	VWA3A	16:22103858-22168287	q1	q2	NOTEST	0.184029	0.0551997	-1.7372	0	1	1	no

ENSG00000175274	ENSG00000175274	TP53I11	11:44740941-44972840	q1	q2	OK	45.5188	38.7563	-0.232031	-0.186062	0.7409	0.99995	no

ENSG00000175279	ENSG00000175279	APITD1	1:10489937-10512210	q1	q2	OK	25.8536	25.549	-0.0170951	-0.0301591	0.9648	0.99995	no

ENSG00000175283	ENSG00000175283	DOLK	9:131683173-131790582	q1	q2	OK	11.5788	9.81341	-0.23866	-0.178684	0.78805	0.99995	no

ENSG00000175287	ENSG00000175287	PHYHD1	9:131683173-131790582	q1	q2	OK	0.775959	0.831124	0.0990823	0.0120187	0.9189	0.99995	no

ENSG00000175294	ENSG00000175294	CATSPER1	11:65784222-65793988	q1	q2	NOTEST	0	0.047201	inf	0	1	1	no

ENSG00000175302	ENSG00000175302	ANKRD30BP1	21:14756569-14800094	q1	q2	NOTEST	0	0	0	0	1	1	no

ENSG00000175305	ENSG00000175305	CCNE2	8:95825538-96128683	q1	q2	OK	9.79838	10.4507	0.0929783	0.0539397	0.91635	0.99995	no

ENSG00000175309	ENSG00000175309	PHYKPL	5:177631507-177659792	q1	q2	OK	20.5705	18.7179	-0.136162	-0.0629548	0.8073	0.99995	no

ENSG00000175311	ENSG00000175311	ANKS4B	16:21244985-21329912	q1	q2	NOTEST	0.017348	0	#NAME?	0	1	1	no

ENSG00000175315	ENSG00000175315	CST6	11:65779311-65780976	q1	q2	NOTEST	0	0	0	0	1	1	no

ENSG00000175318	ENSG00000175318	GRAMD2	15:72452147-72490126	q1	q2	NOTEST	0.318307	0.352434	0.146936	0	1	1	no

ENSG00000175319	ENSG00000175319	NF1P5	18:14152997-14157341	q1	q2	NOTEST	0	0	0	0	1	1	no

ENSG00000175322	ENSG00000175322	ZNF519	18:14057455-14132489	q1	q2	OK	20.081	22.1032	0.138419	0.211979	0.73335	0.99995	no

ENSG00000175324	ENSG00000175324	LSM1	8:37962759-38070819	q1	q2	OK	37.3821	34.5051	-0.115536	-0.125598	0.8609	0.99995	no

ENSG00000175325	ENSG00000175325	PROP1	5:177419235-177423243	q1	q2	NOTEST	0.0385822	0.0365106	-0.0796187	0	1	1	no

ENSG00000175329	ENSG00000175329	ISX	22:35318598-35483380	q1	q2	NOTEST	0	0	0	0	1	1	no

ENSG00000175334	ENSG00000175334	BANF1	11:65764015-65771620	q1	q2	OK	162.536	159.341	-0.0286457	-0.0629872	0.93045	0.99995	no

ENSG00000175336	ENSG00000175336	APOF	12:56754352-56756607	q1	q2	NOTEST	0.0480303	0	#NAME?	0	1	1	no

ENSG00000175344	ENSG00000175344	CHRNA7	15:32322690-32464722	q1	q2	OK	1.35043	1.52994	0.180054	0.204724	0.76445	0.99995	no

ENSG00000175348	ENSG00000175348	TMEM9B	11:8968840-8999074	q1	q2	OK	18.6948	19.9483	0.0936246	0.184221	0.78825	0.99995	no

ENSG00000175352	ENSG00000175352	NRIP3	11:9002122-9025596	q1	q2	OK	1.38423	1.02352	-0.43555	-0.416819	0.41245	0.99995	no

ENSG00000175354	ENSG00000175354	PTPN2	18:12785476-12929642	q1	q2	OK	16.754	16.8316	0.00666571	0.0122243	0.9846	0.99995	no

ENSG00000175356	ENSG00000175356	SCUBE2	11:9025708-9159661	q1	q2	OK	2.145	1.94115	-0.144064	-0.0858213	0.83715	0.99995	no

ENSG00000175376	ENSG00000175376	EIF1AD	11:65764015-65771620	q1	q2	OK	17.604	19.4183	0.14151	0.107855	0.7884	0.99995	no

ENSG00000175387	ENSG00000175387	SMAD2	18:45357921-45457515	q1	q2	OK	36.6923	40.5156	0.143001	0.219995	0.7329	0.99995	no

ENSG00000175390	ENSG00000175390	EIF3F	11:7991797-8023409	q1	q2	OK	135.215	121.932	-0.149181	-0.304952	0.6479	0.99995	no

ENSG00000175393	ENSG00000175393	OR10A6	11:7949179-7950209	q1	q2	NOTEST	0	0	0	0	1	1	no

ENSG00000175395	ENSG00000175395	ZNF25	10:38238499-38265561	q1	q2	OK	7.73735	8.09003	0.0643054	0.12608	0.85065	0.99995	no

ENSG00000175398	ENSG00000175398	OR10P1	12:56030643-56031638	q1	q2	NOTEST	0	0	0	0	1	1	no

ENSG00000175414	ENSG00000175414	ARL10	5:175792470-175843570	q1	q2	OK	10.8461	11.3189	0.0615551	0.0479226	0.87585	0.99995	no

ENSG00000175416	ENSG00000175416	CLTB	5:175792470-175843570	q1	q2	OK	24.2973	21.8459	-0.153432	-0.125065	0.85735	0.99995	no

ENSG00000175426	ENSG00000175426	PCSK1	5:95220801-96143803	q1	q2	OK	0.582863	0.69551	0.254915	0.0947329	0.9073	0.99995	no

ENSG00000175445	ENSG00000175445	LPL	8:19759227-19824769	q1	q2	OK	123.612	142.45	0.204636	0.403367	0.51665	0.99995	no

ENSG00000175449	ENSG00000175449	RFESD	5:94982457-95034415	q1	q2	OK	0.62887	0.641015	0.0275961	0.0167266	0.9779	0.99995	no

ENSG00000175455	ENSG00000175455	CCDC14	3:123616151-123680564	q1	q2	OK	73.0811	80.8588	0.145907	0.214799	0.6789	0.99995	no

ENSG00000175463	ENSG00000175463	TBC1D10C	11:67159175-67193078	q1	q2	OK	1.27507	1.04055	-0.293225	-0.0699899	0.75985	0.99995	no

ENSG00000175467	ENSG00000175467	SART1	11:65729159-65747299	q1	q2	OK	36.0424	27.4237	-0.394273	-0.564509	0.23045	0.99995	no

ENSG00000175470	ENSG00000175470	PPP2R2D	10:133747954-133773331	q1	q2	OK	6.31811	2.30252	-1.45628	-1.34434	0.0477	0.99995	no

ENSG00000175471	ENSG00000175471	MCTP1	5:93488670-94620279	q1	q2	OK	11.4669	12.8246	0.161441	0.135939	0.8107	0.99995	no

ENSG00000175482	ENSG00000175482	POLD4	11:67118247-67141648	q1	q2	OK	10.2394	8.33467	-0.296937	-0.341927	0.67055	0.99995	no

ENSG00000175485	ENSG00000175485	OR52W1	11:6220375-6221489	q1	q2	NOTEST	0.05482	0	#NAME?	0	1	1	no

ENSG00000175489	ENSG00000175489	LRRC25	19:18501953-18508427	q1	q2	NOTEST	0.0549735	0.0350748	-0.648302	0	1	1	no

ENSG00000175497	ENSG00000175497	DPP10	2:115199875-116603328	q1	q2	OK	4.50409	4.74478	0.0751067	0.0889094	0.8617	0.99995	no

ENSG00000175505	ENSG00000175505	CLCF1	11:67118247-67141648	q1	q2	OK	3.01522	2.35696	-0.35534	-0.304266	0.65495	0.99995	no

ENSG00000175509	ENSG00000175509	RP11-395L14.13	2:114425054-114426198	q1	q2	NOTEST	0	0	0	0	1	1	no

ENSG00000175513	ENSG00000175513	TSGA10IP	11:65712915-65727434	q1	q2	OK	2.65241	1.62738	-0.704754	-0.68209	0.27745	0.99995	no

ENSG00000175514	ENSG00000175514	GPR152	11:67218771-67226699	q1	q2	NOTEST	0	0	0	0	1	1	no

ENSG00000175518	ENSG00000175518	UBQLNL	11:5274419-5667019	q1	q2	NOTEST	0.276862	0.282437	0.028767	0	1	1	no

ENSG00000175520	ENSG00000175520	UBQLN3	11:5274419-5667019	q1	q2	NOTEST	0	0	0	0	1	1	no

ENSG00000175535	ENSG00000175535	PNLIP	10:118305442-118327367	q1	q2	NOTEST	0	0	0	0	1	1	no

ENSG00000175536	ENSG00000175536	LIPT2	11:74202756-74380162	q1	q2	OK	3.05223	3.0105	-0.0198579	-0.00721686	0.9899	0.99995	no

ENSG00000175538	ENSG00000175538	KCNE3	11:74041362-74197096	q1	q2	NOTEST	0.00900631	0.0622989	2.7902	0	1	1	no

ENSG00000175544	ENSG00000175544	CABP4	11:67218771-67226699	q1	q2	NOTEST	0.0215916	0	#NAME?	0	1	1	no

ENSG00000175548	ENSG00000175548	ALG10B	12:38710379-38717784	q1	q2	OK	3.52686	3.76082	0.0926612	0.104426	0.8738	0.99995	no

ENSG00000175550	ENSG00000175550	DRAP1	11:65686727-65689032	q1	q2	OK	65.4618	58.0232	-0.174023	-0.376743	0.5837	0.99995	no

ENSG00000175556	ENSG00000175556	LONRF3	X:118108580-118152318	q1	q2	NOTEST	0.117428	0.042275	-1.4739	0	1	1	no

ENSG00000175564	ENSG00000175564	UCP3	11:73711325-73720480	q1	q2	OK	2.09221	2.01901	-0.0513793	-0.0447196	0.94215	0.99995	no

ENSG00000175567	ENSG00000175567	UCP2	11:73685711-73694352	q1	q2	OK	24.869	22.6548	-0.134537	-0.211168	0.74345	0.99995	no

ENSG00000175573	ENSG00000175573	C11orf68	11:65684278-65686588	q1	q2	OK	18.9167	15.696	-0.269271	-0.521897	0.4568	0.99995	no

ENSG00000175575	ENSG00000175575	PAAF1	11:73583711-73638790	q1	q2	OK	14.8654	14.5071	-0.0352002	-0.045446	0.94505	0.99995	no

ENSG00000175581	ENSG00000175581	MRPL48	11:73498360-73576178	q1	q2	OK	30.7749	30.6311	-0.00676068	-0.0110272	0.9859	0.99995	no

ENSG00000175582	ENSG00000175582	RAB6A	11:73386682-73472182	q1	q2	OK	42.3025	48.3068	0.191481	0.442377	0.5195	0.99995	no

ENSG00000175591	ENSG00000175591	P2RY2	11:72929342-72947397	q1	q2	NOTEST	0.0386799	0.0249975	-0.629801	0	1	1	no

ENSG00000175592	ENSG00000175592	FOSL1	11:65659519-65668044	q1	q2	OK	1.45267	1.43698	-0.0156635	-0.015887	0.98045	0.99995	no

ENSG00000175595	ENSG00000175595	ERCC4	16:14014013-14046202	q1	q2	OK	4.65858	5.33128	0.194593	0.223407	0.59975	0.99995	no

ENSG00000175600	ENSG00000175600	SUGCT	7:40174574-40900362	q1	q2	OK	7.57667	4.91538	-0.624263	-0.63079	0.3495	0.99995	no

ENSG00000175602	ENSG00000175602	CCDC85B	11:65657874-65659105	q1	q2	OK	19.1739	16.2492	-0.238773	-0.44151	0.52335	0.99995	no

ENSG00000175604	ENSG00000175604	RP11-276H1.3	16:12070593-12704901	q1	q2	NOTEST	0.0423208	0.0129809	-1.70498	0	1	1	no

ENSG00000175606	ENSG00000175606	TMEM70	8:74884671-74895018	q1	q2	OK	18.9516	19.5991	0.0484721	0.0832294	0.8996	0.99995	no

ENSG00000175611	ENSG00000175611	LINC00476	9:98521512-98776842	q1	q2	OK	4.71526	5.16638	0.131819	0.0789672	0.9218	0.99995	no

ENSG00000175619	ENSG00000175619	OR4B1	11:48238343-48239314	q1	q2	NOTEST	0	0	0	0	1	1	no

ENSG00000175634	ENSG00000175634	RPS6KB2	11:67195930-67202872	q1	q2	OK	20.4995	19.2505	-0.0906954	-0.158869	0.8073	0.99995	no

ENSG00000175643	ENSG00000175643	RMI2	16:11343475-11445619	q1	q2	OK	22.0305	19.2326	-0.19595	-0.358383	0.6113	0.99995	no

ENSG00000175646	ENSG00000175646	PRM1	16:11343475-11445619	q1	q2	NOTEST	0	0	0	0	1	1	no

ENSG00000175658	ENSG00000175658	DRD5P2	1:148900614-148903715	q1	q2	NOTEST	0	0	0	0	1	1	no

ENSG00000175662	ENSG00000175662	TOM1L2	17:17746827-17875736	q1	q2	OK	8.18505	6.86181	-0.254403	-0.313288	0.5152	0.99995	no

ENSG00000175664	ENSG00000175664	TEX26	13:31456069-31551760	q1	q2	NOTEST	0.149217	0	#NAME?	0	1	1	no

ENSG00000175676	ENSG00000175676	GOLGA8EP	15:23435095-23448420	q1	q2	NOTEST	0	0	0	0	1	1	no

ENSG00000175691	ENSG00000175691	ZNF77	19:2933215-2944969	q1	q2	OK	4.4989	4.46931	-0.00952269	-0.0143669	0.98175	0.99995	no

ENSG00000175697	ENSG00000175697	GPR156	3:119883491-120003941	q1	q2	NOTEST	0.210221	0.167265	-0.329771	0	1	1	no

ENSG00000175699	ENSG00000175699	LINC00521	14:94463615-94478040	q1	q2	NOTEST	0	0	0	0	1	1	no

ENSG00000175701	ENSG00000175701	LINC00116	2:110969105-111043433	q1	q2	OK	18.4098	14.5757	-0.336912	-0.437156	0.5236	0.99995	no

ENSG00000175707	ENSG00000175707	C1orf172	1:27276052-27286897	q1	q2	NOTEST	0.0148965	0.0154766	0.0551188	0	1	1	no

ENSG00000175711	ENSG00000175711	B3GNTL1	17:80709939-81009686	q1	q2	OK	3.09366	2.79705	-0.145409	-0.0647996	0.88425	0.99995	no

ENSG00000175718	ENSG00000175718	RBMXL3	X:114345184-114468635	q1	q2	NOTEST	0	0	0	0	1	1	no

ENSG00000175727	ENSG00000175727	MLXIP	12:122516627-122631894	q1	q2	OK	10.6124	10.2683	-0.0475625	-0.0750746	0.87185	0.99995	no

ENSG00000175728	ENSG00000175728	C11orf44	11:130542850-130587247	q1	q2	NOTEST	0	0	0	0	1	1	no

ENSG00000175730	ENSG00000175730	BAK1P1	20:31276722-31278816	q1	q2	OK	0.976795	1.27444	0.383738	0.428326	0.52435	0.99995	no

ENSG00000175741	ENSG00000175741	RWDD4P2	7:39856147-39907739	q1	q2	OK	4.2758	3.56484	-0.262356	-0.14962	0.83665	0.99995	no

ENSG00000175745	ENSG00000175745	NR2F1	5:92745064-92930321	q1	q2	OK	39.8704	28.2807	-0.495498	-0.890588	0.12635	0.99995	no

ENSG00000175746	ENSG00000175746	C15orf54	15:39157522-39719396	q1	q2	NOTEST	0.0402087	0.0399685	-0.00864362	0	1	1	no

ENSG00000175749	ENSG00000175749	EIF3KP1	5:102368079-102368735	q1	q2	NOTEST	0.107108	0.105009	-0.0285589	0	1	1	no

ENSG00000175756	ENSG00000175756	AURKAIP1	1:1309109-1310875	q1	q2	OK	62.1892	54.8405	-0.181422	-0.389332	0.5821	0.99995	no

ENSG00000175764	ENSG00000175764	TTLL11	9:124584206-124855885	q1	q2	OK	3.8838	2.60521	-0.57607	-0.64693	0.3493	0.99995	no

ENSG00000175766	ENSG00000175766	EIF4E1B	5:176057682-176073642	q1	q2	NOTEST	0	0	0	0	1	1	no

ENSG00000175768	ENSG00000175768	TOMM5	9:37510888-38069210	q1	q2	OK	109.144	118.978	0.124468	0.160699	0.8164	0.99995	no

ENSG00000175772	ENSG00000175772	LINC01106	2:111132714-111142219	q1	q2	NOTEST	0.0869357	0.104044	0.259175	0	1	1	no

ENSG00000175773	ENSG00000175773	RP11-121M22.1	11:130184887-130263688	q1	q2	NOTEST	0	0.154654	inf	0	1	1	no

ENSG00000175779	ENSG00000175779	C15orf53	15:38988798-38992239	q1	q2	NOTEST	0	0	0	0	1	1	no

ENSG00000175782	ENSG00000175782	SLC35E3	12:69139885-69187743	q1	q2	OK	5.20476	5.64159	0.116272	0.104677	0.8024	0.99995	no

ENSG00000175785	ENSG00000175785	PRIMA1	14:94184643-94254827	q1	q2	OK	1.01488	1.03755	0.0318651	0.037558	0.9535	0.99995	no

ENSG00000175787	ENSG00000175787	ZNF169	9:97021592-97063736	q1	q2	OK	3.01109	2.80507	-0.102246	-0.101257	0.8472	0.99995	no

ENSG00000175792	ENSG00000175792	RUVBL1	3:127770483-128127485	q1	q2	OK	54.0742	55.6239	0.0407639	0.0408376	0.95145	0.99995	no

ENSG00000175793	ENSG00000175793	SFN	1:27189632-27190947	q1	q2	NOTEST	0.0212833	0	#NAME?	0	1	1	no

ENSG00000175800	ENSG00000175800	OR52B3P	11:4399511-4400457	q1	q2	NOTEST	0.0325862	0	#NAME?	0	1	1	no

ENSG00000175806	ENSG00000175806	MSRA	8:9911777-10286401	q1	q2	OK	9.17817	8.94978	-0.0363539	-0.0563545	0.93285	0.99995	no

ENSG00000175809	ENSG00000175809	ZNF645	X:22277913-23311263	q1	q2	NOTEST	0	0	0	0	1	1	no

ENSG00000175820	ENSG00000175820	CCDC168	13:103381800-103389159	q1	q2	NOTEST	0.0441576	0.0675221	0.612696	0	1	1	no

ENSG00000175826	ENSG00000175826	CTDNEP1	17:7143332-7167302	q1	q2	OK	75.053	70.1102	-0.0982851	-0.109087	0.86805	0.99995	no

ENSG00000175827	ENSG00000175827	AP001266.1	11:65543363-65548273	q1	q2	OK	0.700268	0.71971	0.0395079	0.0215716	0.97755	0.99995	no

ENSG00000175832	ENSG00000175832	ETV4	17:41561232-41687706	q1	q2	OK	84.5621	91.3999	0.112181	0.196132	0.76255	0.99995	no

ENSG00000175841	ENSG00000175841	FAM172BP	3:101237710-101242731	q1	q2	NOTEST	0	0	0	0	1	1	no

ENSG00000175854	ENSG00000175854	SWI5	9:131018107-131051269	q1	q2	OK	17.1597	14.4283	-0.250127	-0.186774	0.79005	0.99995	no

ENSG00000175857	ENSG00000175857	GAPT	5:57787261-57792917	q1	q2	NOTEST	0	0.0119952	inf	0	1	1	no

ENSG00000175866	ENSG00000175866	BAIAP2	17:79008947-79156964	q1	q2	OK	18.0022	17.2607	-0.060684	-0.0920992	0.8654	0.99995	no

ENSG00000175868	ENSG00000175868	CALCB	11:14926542-15103888	q1	q2	OK	0.375086	0.703631	0.907598	0.78527	0.2569	0.99995	no

ENSG00000175873	ENSG00000175873	AC004840.9	7:2477397-2487485	q1	q2	OK	1.01127	0.819057	-0.304128	-0.322614	0.6387	0.99995	no

ENSG00000175874	ENSG00000175874	CREG2	2:101962012-102004057	q1	q2	NOTEST	0.0870391	0.0353792	-1.29876	0	1	1	no

ENSG00000175877	ENSG00000175877	WBSCR28	7:73275488-73280223	q1	q2	NOTEST	0	0	0	0	1	1	no

ENSG00000175879	ENSG00000175879	HOXD8	2:176986338-177037830	q1	q2	OK	1.33139	1.54574	0.215371	0.130574	0.8548	0.99995	no

ENSG00000175886	ENSG00000175886	RPL7AP66	18:36786887-37380282	q1	q2	OK	2.50431	4.88683	0.964485	0.568085	0.4426	0.99995	no

ENSG00000175893	ENSG00000175893	ZDHHC21	9:14611068-14693469	q1	q2	OK	7.23994	8.41911	0.21769	0.400277	0.4735	0.99995	no

ENSG00000175894	ENSG00000175894	TSPEAR	21:45917774-46131495	q1	q2	NOTEST	0	0	0	0	1	1	no

ENSG00000175895	ENSG00000175895	PLEKHF2	8:96146031-96168912	q1	q2	OK	5.61906	5.39926	-0.057569	-0.100569	0.88255	0.99995	no

ENSG00000175898	ENSG00000175898	CTD-2369P2.2	19:10244020-10341962	q1	q2	OK	8.33777	7.73607	-0.108062	-0.0430856	0.9566	0.99995	no

ENSG00000175899	ENSG00000175899	A2M	12:9217772-9268825	q1	q2	OK	30.6771	31.3215	0.0299926	0.066721	0.9146	0.99995	no

ENSG00000175906	ENSG00000175906	ARL4D	17:41476326-41478505	q1	q2	OK	4.46925	4.43998	-0.00947873	-0.0135853	0.98445	0.99995	no

ENSG00000175911	ENSG00000175911	AC127496.1	17:78965397-78983317	q1	q2	OK	0.884863	0.742131	-0.253781	-0.11874	0.88065	0.99995	no

ENSG00000175920	ENSG00000175920	DOK7	4:3465032-3503200	q1	q2	NOTEST	0.285178	0.361477	0.342042	0	1	1	no

ENSG00000175928	ENSG00000175928	LRRN1	3:3742497-4508965	q1	q2	OK	161.902	138.539	-0.22483	-0.366226	0.5041	0.99995	no

ENSG00000175931	ENSG00000175931	UBE2O	17:74385531-74449288	q1	q2	OK	22.1849	20.2719	-0.130099	-0.228697	0.6704	0.99995	no

ENSG00000175938	ENSG00000175938	ORAI3	16:30930639-30967832	q1	q2	OK	5.97408	4.59772	-0.377799	-0.227853	0.72975	0.99995	no

ENSG00000175946	ENSG00000175946	KLHL38	8:124626464-124670810	q1	q2	NOTEST	0	0	0	0	1	1	no

ENSG00000175967	ENSG00000175967	RP11-544L8__B.4	6:114225301-114242806	q1	q2	NOTEST	0.179497	0.147991	-0.278449	0	1	1	no

ENSG00000175970	ENSG00000175970	UNC119B	12:121148237-121161443	q1	q2	OK	40.2122	39.8799	-0.0119719	-0.0281295	0.9662	0.99995	no

ENSG00000175984	ENSG00000175984	DENND2C	1:115125468-115213043	q1	q2	OK	0.903132	1.09468	0.277503	0.276949	0.67845	0.99995	no

ENSG00000175985	ENSG00000175985	PLEKHD1	14:69951408-69995215	q1	q2	NOTEST	0.104377	0.0709703	-0.556516	0	1	1	no

ENSG00000176007	ENSG00000176007	FAM220BP	9:38527093-38527909	q1	q2	NOTEST	0	0	0	0	1	1	no

ENSG00000176009	ENSG00000176009	ASCL3	11:8959118-8964580	q1	q2	NOTEST	0.0579951	0.113193	0.964776	0	1	1	no

ENSG00000176014	ENSG00000176014	TUBB6	18:12307667-12377313	q1	q2	OK	57.6118	51.4259	-0.163871	-0.252242	0.67235	0.99995	no

ENSG00000176018	ENSG00000176018	LYSMD3	5:89811427-90460038	q1	q2	OK	8.82044	9.41037	0.0934018	0.0595581	0.921	0.99995	no

ENSG00000176020	ENSG00000176020	AMIGO3	3:49711434-49761384	q1	q2	OK	5.37594	5.06455	-0.0860832	-0.0897439	0.90055	0.99995	no

ENSG00000176022	ENSG00000176022	B3GALT6	1:1167628-1170421	q1	q2	OK	22.9527	19.548	-0.231641	-0.535389	0.4398	0.99995	no

ENSG00000176024	ENSG00000176024	ZNF613	19:52430399-52452012	q1	q2	OK	4.12094	4.01304	-0.0382783	-0.0497048	0.9327	0.99995	no

ENSG00000176029	ENSG00000176029	C11orf16	11:8932685-8954553	q1	q2	NOTEST	0.069467	0.0484012	-0.521285	0	1	1	no

ENSG00000176034	ENSG00000176034	CHDC2	X:36053907-36163187	q1	q2	NOTEST	0.0285027	0.041199	0.531509	0	1	1	no

ENSG00000176040	ENSG00000176040	TMPRSS7	3:111753689-111800116	q1	q2	NOTEST	0.0975763	0.18452	0.919171	0	1	1	no

ENSG00000176043	ENSG00000176043	RP1-146I3.1	14:73393039-73426411	q1	q2	NOTEST	0	0	0	0	1	1	no

ENSG00000176046	ENSG00000176046	NUPR1	16:28548605-28550495	q1	q2	OK	15.7732	11.3432	-0.475642	-0.661977	0.3435	0.99995	no

ENSG00000176049	ENSG00000176049	JAKMIP2	5:146967989-147162338	q1	q2	OK	13.9575	14.0281	0.00728167	0.0147496	0.9815	0.99995	no

ENSG00000176054	ENSG00000176054	RPL23P2	21:30369933-30370354	q1	q2	OK	3.08761	2.91529	-0.0828503	-0.0795386	0.91175	0.99995	no

ENSG00000176055	ENSG00000176055	MBLAC2	5:89754019-89810370	q1	q2	OK	2.84128	2.48342	-0.194213	-0.261298	0.71505	0.99995	no

ENSG00000176058	ENSG00000176058	TPRN	9:140086068-140100090	q1	q2	OK	6.69351	6.17599	-0.116092	-0.129148	0.8502	0.99995	no

ENSG00000176075	ENSG00000176075	LINC00302	1:152627952-152629280	q1	q2	NOTEST	0	0	0	0	1	1	no

ENSG00000176076	ENSG00000176076	KCNE1L	X:108866928-108976632	q1	q2	NOTEST	0.189659	0.150877	-0.330034	0	1	1	no

ENSG00000176082	ENSG00000176082	AL357153.1	14:70892528-71014181	q1	q2	NOTEST	0.198621	0.182934	-0.118693	0	1	1	no

ENSG00000176083	ENSG00000176083	ZNF683	1:26688124-26701013	q1	q2	NOTEST	0	0	0	0	1	1	no

ENSG00000176087	ENSG00000176087	SLC35A4	5:139781398-139973337	q1	q2	OK	31.1673	29.3849	-0.0849595	-0.0749126	0.90185	0.99995	no

ENSG00000176092	ENSG00000176092	AIM1L	1:26648349-26680621	q1	q2	NOTEST	0.0222568	0.09892	2.15202	0	1	1	no

ENSG00000176095	ENSG00000176095	IP6K1	3:49761726-49823975	q1	q2	OK	23.8629	23.0857	-0.0477679	-0.102973	0.8656	0.99995	no

ENSG00000176101	ENSG00000176101	SSNA1	9:140083098-140084822	q1	q2	OK	40.3559	39.1842	-0.0425068	-0.0869789	0.89905	0.99995	no

ENSG00000176102	ENSG00000176102	CSTF3	11:33060962-33213144	q1	q2	OK	48.4389	53.6165	0.146512	0.221149	0.72505	0.99995	no

ENSG00000176105	ENSG00000176105	YES1	18:721587-812547	q1	q2	OK	49.8147	63.6093	0.352665	0.563274	0.26625	0.99995	no

ENSG00000176108	ENSG00000176108	CHMP6	17:78965397-78983317	q1	q2	OK	16.5722	13.9008	-0.253602	-0.450958	0.49085	0.99995	no

ENSG00000176115	ENSG00000176115	AQP7P4	9:67032330-67048373	q1	q2	NOTEST	0	0	0	0	1	1	no

ENSG00000176124	ENSG00000176124	DLEU1	13:50601268-51423190	q1	q2	OK	10.455	9.86379	-0.0839731	-0.0908848	0.8832	0.99995	no

ENSG00000176125	ENSG00000176125	UFSP1	7:100486345-100487339	q1	q2	NOTEST	0.333804	0.446747	0.420457	0	1	1	no

ENSG00000176134	ENSG00000176134	AL445665.1	9:69650262-69660358	q1	q2	NOTEST	0	0	0	0	1	1	no

ENSG00000176136	ENSG00000176136	MC5R	18:13824153-13826861	q1	q2	NOTEST	0.101768	0.103198	0.0201408	0	1	1	no

ENSG00000176142	ENSG00000176142	TMEM39A	3:119148346-119187677	q1	q2	OK	14.7626	14.5406	-0.0218592	-0.0312305	0.9552	0.99995	no

ENSG00000176148	ENSG00000176148	TCP11L1	11:33060962-33213144	q1	q2	OK	6.11428	6.17733	0.014801	0.011724	0.98335	0.99995	no

ENSG00000176153	ENSG00000176153	GPX2	14:65381078-65569413	q1	q2	NOTEST	0.183001	0.24081	0.396043	0	1	1	no

ENSG00000176155	ENSG00000176155	CCDC57	17:80059335-80170706	q1	q2	OK	27.6511	24.3376	-0.184146	-0.246645	0.6606	0.99995	no

ENSG00000176160	ENSG00000176160	HSF5	17:56497527-56565745	q1	q2	NOTEST	0.0128455	0.024916	0.955812	0	1	1	no

ENSG00000176165	ENSG00000176165	FOXG1	14:29235049-29238870	q1	q2	NOTEST	0.0104928	0	#NAME?	0	1	1	no

ENSG00000176170	ENSG00000176170	SPHK1	17:74305566-74383941	q1	q2	OK	3.60707	2.8365	-0.346717	-0.237929	0.76785	0.99995	no

ENSG00000176171	ENSG00000176171	BNIP3	10:133781577-133795435	q1	q2	OK	19.427	18.1939	-0.0946117	-0.174725	0.7972	0.99995	no

ENSG00000176177	ENSG00000176177	ENTHD1	22:40139048-40289868	q1	q2	NOTEST	0.0398682	0.00916106	-2.12165	0	1	1	no

ENSG00000176182	ENSG00000176182	MYPOP	19:46393277-46405862	q1	q2	OK	5.70898	4.92594	-0.212835	-0.3336	0.63	0.99995	no

ENSG00000176183	ENSG00000176183	CH17-12M21.1	5:97674447-97675536	q1	q2	NOTEST	0	0	0	0	1	1	no

ENSG00000176194	ENSG00000176194	CIDEA	18:12254317-12277594	q1	q2	NOTEST	0	0	0	0	1	1	no

ENSG00000176198	ENSG00000176198	OR11H4	14:20710897-20711979	q1	q2	NOTEST	0	0.0262419	inf	0	1	1	no

ENSG00000176200	ENSG00000176200	OR4D11	11:59271048-59271984	q1	q2	NOTEST	0	0	0	0	1	1	no

ENSG00000176204	ENSG00000176204	LRRTM4	2:76974844-77820445	q1	q2	OK	2.78548	3.02978	0.121288	0.178764	0.78205	0.99995	no

ENSG00000176208	ENSG00000176208	ATAD5	17:29158987-29222887	q1	q2	OK	8.53664	9.27224	0.119249	0.264135	0.7001	0.99995	no

ENSG00000176209	ENSG00000176209	SMIM19	8:42273992-42409603	q1	q2	OK	23.4835	23.0061	-0.0296269	-0.0332662	0.96395	0.99995	no

ENSG00000176219	ENSG00000176219	OR11H6	14:20691790-20692964	q1	q2	NOTEST	0	0	0	0	1	1	no

ENSG00000176222	ENSG00000176222	ZNF404	19:44376514-44405957	q1	q2	OK	3.92339	3.38646	-0.212323	-0.257835	0.708	0.99995	no

ENSG00000176225	ENSG00000176225	RTTN	18:67671028-67873181	q1	q2	OK	10.9408	9.87937	-0.147231	-0.155222	0.791	0.99995	no

ENSG00000176227	ENSG00000176227	CTAGE15	7:143268818-143271480	q1	q2	NOTEST	0	0	0	0	1	1	no

ENSG00000176230	ENSG00000176230	OR4K17	14:20585565-20586597	q1	q2	NOTEST	0	0	0	0	1	1	no

ENSG00000176231	ENSG00000176231	OR10H4	19:16059817-16060768	q1	q2	NOTEST	0	0	0	0	1	1	no

ENSG00000176232	ENSG00000176232	RP5-1193P9.4	7:63440762-63441855	q1	q2	NOTEST	0	0	0	0	1	1	no

ENSG00000176236	ENSG00000176236	C10orf111	10:15137383-15210692	q1	q2	OK	0.520039	0.611421	0.233546	0.0734021	0.89675	0.99995	no

ENSG00000176239	ENSG00000176239	OR51B6	11:5274419-5667019	q1	q2	NOTEST	0	0	0	0	1	1	no

ENSG00000176243	ENSG00000176243	CDV3P1	3:96495788-96496527	q1	q2	NOTEST	0	0	0	0	1	1	no

ENSG00000176244	ENSG00000176244	ACBD7	10:15057070-15130775	q1	q2	OK	9.29749	8.47102	-0.134305	-0.211972	0.75585	0.99995	no

ENSG00000176246	ENSG00000176246	OR4L1	14:20528203-20529142	q1	q2	NOTEST	0	0	0	0	1	1	no

ENSG00000176248	ENSG00000176248	ANAPC2	9:140069235-140082989	q1	q2	OK	19.4174	17.9562	-0.112869	-0.203401	0.76005	0.99995	no

ENSG00000176253	ENSG00000176253	OR4K13	14:20502002-20502919	q1	q2	NOTEST	0	0	0	0	1	1	no

ENSG00000176256	ENSG00000176256	HMGB4	1:33979608-34631443	q1	q2	NOTEST	0	0	0	0	1	1	no

ENSG00000176261	ENSG00000176261	ZBTB8OS	1:32930657-33116504	q1	q2	OK	33.0874	35.5448	0.103355	0.112397	0.87175	0.99995	no

ENSG00000176268	ENSG00000176268	CYCSP34	13:41396431-41495910	q1	q2	OK	1.10815	1.71647	0.631287	0.305204	0.64415	0.99995	no

ENSG00000176269	ENSG00000176269	OR4F21	8:116048-117043	q1	q2	NOTEST	0	0	0	0	1	1	no

ENSG00000176273	ENSG00000176273	SLC35G1	10:95653729-95715819	q1	q2	OK	4.55101	5.03705	0.146391	0.198634	0.74305	0.99995	no

ENSG00000176274	ENSG00000176274	SLC25A53	X:103343897-103401708	q1	q2	NOTEST	0	0.00380925	inf	0	1	1	no

ENSG00000176281	ENSG00000176281	OR4K5	14:20388740-20389818	q1	q2	NOTEST	0	0	0	0	1	1	no

ENSG00000176289	ENSG00000176289	IDSP1	X:148558520-148632055	q1	q2	NOTEST	0	0	0	0	1	1	no

ENSG00000176290	ENSG00000176290	OR4K3	14:20336400-20337302	q1	q2	NOTEST	0	0	0	0	1	1	no

ENSG00000176293	ENSG00000176293	ZNF135	19:58570606-58629794	q1	q2	NOTEST	0.154442	0.247444	0.680035	0	1	1	no

ENSG00000176294	ENSG00000176294	OR4N2	14:20187173-20296531	q1	q2	NOTEST	0	0	0	0	1	1	no

ENSG00000176299	ENSG00000176299	OR4M1	14:20187173-20296531	q1	q2	NOTEST	0	0	0	0	1	1	no

ENSG00000176302	ENSG00000176302	FOXR1	11:118842416-118852001	q1	q2	NOTEST	0	0	0	0	1	1	no

ENSG00000176305	ENSG00000176305	AC087269.1	8:8559447-8561616	q1	q2	NOTEST	0.0181921	0.13753	2.91836	0	1	1	no

ENSG00000176312	ENSG00000176312	OR4H12P	14:20187173-20296531	q1	q2	NOTEST	0	0	0	0	1	1	no

ENSG00000176318	ENSG00000176318	FOXN3P1	X:101801661-101803141	q1	q2	NOTEST	0	0	0	0	1	1	no

ENSG00000176320	ENSG00000176320	RP11-404O13.5	1:158167711-158173667	q1	q2	NOTEST	0.0410119	0.0950464	1.21259	0	1	1	no

ENSG00000176340	ENSG00000176340	COX8A	11:63742078-63933578	q1	q2	OK	291.157	279.21	-0.0604511	-0.0951962	0.8946	0.99995	no

ENSG00000176343	ENSG00000176343	RPL37AP8	11:111759922-111760198	q1	q2	NOTEST	0	0	0	0	1	1	no

ENSG00000176349	ENSG00000176349	AC110781.3	7:1855428-2272878	q1	q2	NOTEST	0.0176165	0.0261466	0.569699	0	1	1	no

ENSG00000176354	ENSG00000176354	AC097359.1	3:37283279-37408242	q1	q2	NOTEST	0.243975	0.180327	-0.436119	0	1	1	no

ENSG00000176358	ENSG00000176358	TAC4	17:47915670-47926199	q1	q2	NOTEST	0.0651768	0	#NAME?	0	1	1	no

ENSG00000176371	ENSG00000176371	ZSCAN2	15:85144216-85171027	q1	q2	OK	10.192	9.72034	-0.0683609	-0.0859935	0.88565	0.99995	no

ENSG00000176378	ENSG00000176378	PFN1P10	1:21766620-21811498	q1	q2	NOTEST	0	0	0	0	1	1	no

ENSG00000176381	ENSG00000176381	PRR18	6:166719167-166721936	q1	q2	NOTEST	0.251697	0.113737	-1.14598	0	1	1	no

ENSG00000176383	ENSG00000176383	B3GNT4	12:122688089-122751068	q1	q2	OK	0.797312	0.823912	0.0473454	0.00777022	0.9654	0.99995	no

ENSG00000176386	ENSG00000176386	CDC26	9:115983807-116055185	q1	q2	OK	15.1706	15.5477	0.0354197	0.0230663	0.9716	0.99995	no

ENSG00000176387	ENSG00000176387	HSD11B2	16:67464554-67471456	q1	q2	OK	1.84591	1.4798	-0.318924	-0.259812	0.6259	0.99995	no

ENSG00000176390	ENSG00000176390	CRLF3	17:29036316-29151794	q1	q2	OK	7.7517	8.61308	0.152016	0.179708	0.73045	0.99995	no

ENSG00000176393	ENSG00000176393	RNPEP	1:201951499-201986316	q1	q2	OK	28.3742	25.0739	-0.178393	-0.334985	0.57325	0.99995	no

ENSG00000176395	ENSG00000176395	CEACAM20	19:45005728-45033811	q1	q2	NOTEST	0	0	0	0	1	1	no

ENSG00000176396	ENSG00000176396	EID2	19:40028889-40030870	q1	q2	OK	19.2245	19.2105	-0.00105278	-0.00225237	0.99745	0.99995	no

ENSG00000176399	ENSG00000176399	DMRTA1	9:22446839-22455739	q1	q2	NOTEST	0.232538	0.369129	0.666656	0	1	1	no

ENSG00000176401	ENSG00000176401	EID2B	19:40021629-40025062	q1	q2	OK	4.95495	4.4973	-0.139809	-0.210555	0.75475	0.99995	no

ENSG00000176402	ENSG00000176402	GJC3	7:99520891-99546243	q1	q2	NOTEST	0.0791656	0.0773888	-0.0327488	0	1	1	no

ENSG00000176406	ENSG00000176406	RIMS2	8:104495625-105268322	q1	q2	OK	4.10386	5.30407	0.370119	0.422587	0.3596	0.99995	no

ENSG00000176407	ENSG00000176407	KCMF1	2:85198215-85286595	q1	q2	OK	24.0621	25.5564	0.0869205	0.0903197	0.8956	0.99995	no

ENSG00000176410	ENSG00000176410	DNAJC30	7:73095298-73134002	q1	q2	NOTEST	0.0426686	0.0316603	-0.4305	0	1	1	no

ENSG00000176422	ENSG00000176422	SPRYD4	12:56843285-56882198	q1	q2	OK	3.98697	4.16209	0.0620138	0.0895435	0.9017	0.99995	no

ENSG00000176428	ENSG00000176428	VPS37D	7:73082154-73086442	q1	q2	OK	0.168476	0.571739	1.76282	1.35846	0.0408	0.99995	no

ENSG00000176435	ENSG00000176435	CLEC14A	14:38723307-38725574	q1	q2	NOTEST	0	0	0	0	1	1	no

ENSG00000176438	ENSG00000176438	SYNE3	14:95883830-95942173	q1	q2	NOTEST	0.147423	0.136445	-0.111643	0	1	1	no

ENSG00000176444	ENSG00000176444	CLK2	1:155232658-155271225	q1	q2	NOTEST	0	0.0152521	inf	0	1	1	no

ENSG00000176454	ENSG00000176454	LPCAT4	15:34651105-34659479	q1	q2	OK	31.8367	33.1514	0.0583805	0.102607	0.8839	0.99995	no

ENSG00000176463	ENSG00000176463	SLCO3A1	15:92396924-92874267	q1	q2	OK	4.08704	3.66043	-0.159043	-0.233759	0.71795	0.99995	no

ENSG00000176472	ENSG00000176472	ZNF575	19:44010870-44040282	q1	q2	OK	0.730154	0.761943	0.0614822	0.0239276	0.95825	0.99995	no

ENSG00000176473	ENSG00000176473	WDR25	14:100800124-100996640	q1	q2	OK	6.95858	7.86603	0.176841	0.118256	0.85465	0.99995	no

ENSG00000176476	ENSG00000176476	CCDC101	16:28565235-28603111	q1	q2	OK	14.5809	14.2811	-0.0299761	-0.0497734	0.93935	0.99995	no

ENSG00000176485	ENSG00000176485	PLA2G16	11:63340666-63389143	q1	q2	OK	6.29774	4.96713	-0.342422	-0.453105	0.5039	0.99995	no

ENSG00000176490	ENSG00000176490	DIRAS1	19:2714564-2740150	q1	q2	OK	11.8737	10.4249	-0.187727	-0.31646	0.63045	0.99995	no

ENSG00000176495	ENSG00000176495	OR5AN1	11:59131884-59132921	q1	q2	NOTEST	0	0	0	0	1	1	no

ENSG00000176510	ENSG00000176510	OR10AC1P	7:143078172-143220542	q1	q2	NOTEST	0.0304649	0	#NAME?	0	1	1	no

ENSG00000176515	ENSG00000176515	AL033381.1	6:1080163-1105181	q1	q2	NOTEST	0	0	0	0	1	1	no

ENSG00000176531	ENSG00000176531	PHLDB3	19:43979105-44009087	q1	q2	OK	2.47496	2.17786	-0.184499	-0.17258	0.7925	0.99995	no

ENSG00000176532	ENSG00000176532	PRR15	7:29603426-29606911	q1	q2	NOTEST	0.0160556	0.0322742	1.0073	0	1	1	no

ENSG00000176533	ENSG00000176533	GNG7	19:2511216-2702707	q1	q2	OK	4.43373	4.13992	-0.0989171	-0.174651	0.79715	0.99995	no

ENSG00000176540	ENSG00000176540	OR4C5	11:48387036-48388017	q1	q2	NOTEST	0	0	0	0	1	1	no

ENSG00000176542	ENSG00000176542	KIAA2018	3:113367231-113415493	q1	q2	OK	6.09014	6.23624	0.0342015	0.043433	0.94995	0.99995	no

ENSG00000176547	ENSG00000176547	OR4C3	11:48346471-48347507	q1	q2	NOTEST	0	0	0	0	1	1	no

ENSG00000176555	ENSG00000176555	OR4S1	11:48327774-48328704	q1	q2	NOTEST	0	0	0	0	1	1	no

ENSG00000176563	ENSG00000176563	CNTD1	17:40950809-40995774	q1	q2	OK	1.22929	1.06423	-0.208019	-0.0551831	0.91515	0.99995	no

ENSG00000176566	ENSG00000176566	DCAF4L2	8:88882972-88886296	q1	q2	NOTEST	0.00759574	0	#NAME?	0	1	1	no

ENSG00000176567	ENSG00000176567	OR4X1	11:48285412-48286330	q1	q2	NOTEST	0	0	0	0	1	1	no

ENSG00000176571	ENSG00000176571	CNBD1	8:87878669-88767946	q1	q2	NOTEST	0	0	0	0	1	1	no

ENSG00000176584	ENSG00000176584	DMBT1P1	10:124516209-124558696	q1	q2	NOTEST	0	0	0	0	1	1	no

ENSG00000176593	ENSG00000176593	CTD-2368P22.1	19:58488420-58522600	q1	q2	OK	7.32214	7.20401	-0.023466	-0.0229845	0.97105	0.99995	no

ENSG00000176595	ENSG00000176595	KBTBD11	8:1919562-1955102	q1	q2	OK	8.21654	8.86624	0.109792	0.250517	0.72145	0.99995	no

ENSG00000176597	ENSG00000176597	B3GNT5	3:182895830-183146566	q1	q2	OK	17.0555	19.27	0.17612	0.364387	0.54845	0.99995	no

ENSG00000176601	ENSG00000176601	MAP3K19	2:135722060-135805038	q1	q2	NOTEST	0.0394891	0.0123633	-1.6754	0	1	1	no

ENSG00000176605	ENSG00000176605	C14orf177	14:99177949-99184098	q1	q2	NOTEST	0	0	0	0	1	1	no

ENSG00000176619	ENSG00000176619	LMNB2	19:2389768-2456994	q1	q2	OK	65.2882	62.6594	-0.0592908	-0.122824	0.8426	0.99995	no

ENSG00000176623	ENSG00000176623	RMDN1	8:87345502-87777251	q1	q2	OK	27.4469	27.4248	-0.00116441	-0.000880284	0.99795	0.99995	no

ENSG00000176624	ENSG00000176624	MEX3C	18:48700919-48744674	q1	q2	OK	33.4761	36.6647	0.13126	0.23891	0.7052	0.99995	no

ENSG00000176635	ENSG00000176635	HORMAD2	22:30279143-30573064	q1	q2	NOTEST	0.024783	0.19182	2.95233	0	1	1	no

ENSG00000176641	ENSG00000176641	RNF152	18:59475295-59561480	q1	q2	OK	2.24725	2.5425	0.178083	0.207072	0.62095	0.99995	no

ENSG00000176654	ENSG00000176654	NANOGP1	12:8045171-8052736	q1	q2	NOTEST	0.0149622	0.0310169	1.05173	0	1	1	no

ENSG00000176658	ENSG00000176658	MYO1D	17:30819539-31205655	q1	q2	OK	2.15893	1.26388	-0.772452	-0.685474	0.1212	0.99995	no

ENSG00000176659	ENSG00000176659	C20orf197	20:58630979-58648008	q1	q2	NOTEST	0	0.0279072	inf	0	1	1	no

ENSG00000176678	ENSG00000176678	FOXL1	16:86609973-86615303	q1	q2	NOTEST	0.27078	0.185663	-0.544435	0	1	1	no

ENSG00000176679	ENSG00000176679	TGIF2LY	Y:3447081-3448082	q1	q2	NOTEST	0	0	0	0	1	1	no

ENSG00000176681	ENSG00000176681	LRRC37A	17:44352149-44439130	q1	q2	NOTEST	0.258686	0.295616	0.192523	0	1	1	no

ENSG00000176692	ENSG00000176692	FOXC2	16:86598750-86602539	q1	q2	NOTEST	0.432918	0.267472	-0.694705	0	1	1	no

ENSG00000176695	ENSG00000176695	OR4F17	19:107470-111696	q1	q2	NOTEST	0	0	0	0	1	1	no

ENSG00000176697	ENSG00000176697	BDNF	11:27528384-27899195	q1	q2	OK	0.728603	0.676008	-0.108093	-0.0786284	0.92395	0.99995	no

ENSG00000176700	ENSG00000176700	SCAND2P	15:85174681-85185695	q1	q2	OK	9.12665	8.7849	-0.0550594	-0.0628805	0.89065	0.99995	no

ENSG00000176714	ENSG00000176714	CCDC121	2:27805896-27917840	q1	q2	OK	5.39411	4.89263	-0.140776	-0.0472291	0.93385	0.99995	no

ENSG00000176715	ENSG00000176715	ACSF3	16:89154782-89222254	q1	q2	OK	7.30854	7.52416	0.0419468	0.052872	0.9135	0.99995	no

ENSG00000176716	ENSG00000176716	OR10AB1P	11:7710668-7927502	q1	q2	NOTEST	0.0661174	0.0961717	0.540583	0	1	1	no

ENSG00000176720	ENSG00000176720	BOK	2:242483817-242513546	q1	q2	OK	5.7642	4.38746	-0.393734	-0.655676	0.3532	0.99995	no

ENSG00000176723	ENSG00000176723	ZNF843	16:31445568-31454346	q1	q2	NOTEST	0.253488	0.292305	0.205559	0	1	1	no

ENSG00000176728	ENSG00000176728	TTTY14	Y:21034386-21239302	q1	q2	OK	10.0997	8.92244	-0.178797	-0.196335	0.7971	0.99995	no

ENSG00000176731	ENSG00000176731	C8orf59	8:86089459-86132650	q1	q2	OK	76.0827	85.4209	0.16702	0.145476	0.83065	0.99995	no

ENSG00000176732	ENSG00000176732	PFN4	2:24299395-24424567	q1	q2	OK	0.344811	0.5529	0.681213	0.103038	0.62015	0.99995	no

ENSG00000176734	ENSG00000176734	TRIL	7:28992973-28998961	q1	q2	OK	12.653	10.7651	-0.23311	-0.411372	0.55115	0.99995	no

ENSG00000176742	ENSG00000176742	OR51V1	11:5220964-5221930	q1	q2	NOTEST	0	0	0	0	1	1	no

ENSG00000176746	ENSG00000176746	MAGEB6	X:26210556-26213763	q1	q2	NOTEST	0	0	0	0	1	1	no

ENSG00000176748	ENSG00000176748	OR52Z1	11:5172238-5207612	q1	q2	NOTEST	0	0	0	0	1	1	no

ENSG00000176749	ENSG00000176749	CDK5R1	17:30813636-30818274	q1	q2	OK	10.9062	11.2784	0.0484162	0.104398	0.87275	0.99995	no

ENSG00000176752	ENSG00000176752	OR51A1P	11:5172238-5207612	q1	q2	NOTEST	0	0	0	0	1	1	no

ENSG00000176753	ENSG00000176753	C15orf56	15:40453223-40569688	q1	q2	NOTEST	0.024632	0	#NAME?	0	1	1	no

ENSG00000176754	ENSG00000176754	LINC00303	1:204001574-204010393	q1	q2	NOTEST	0.0140331	0	#NAME?	0	1	1	no

ENSG00000176761	ENSG00000176761	ZNF285B	19:44971857-44977444	q1	q2	OK	1.3979	0.699222	-0.999436	-0.999515	0.15655	0.99995	no

ENSG00000176769	ENSG00000176769	TCERG1L	10:132890653-133109984	q1	q2	NOTEST	0.188037	0.255001	0.439492	0	1	1	no

ENSG00000176771	ENSG00000176771	NCKAP5	2:133429373-134326034	q1	q2	OK	9.73443	10.8482	0.156289	0.237647	0.619	0.99995	no

ENSG00000176774	ENSG00000176774	MAGEB18	X:26156459-26158852	q1	q2	NOTEST	0	0	0	0	1	1	no

ENSG00000176782	ENSG00000176782	DEFB104A	8:7693992-7698764	q1	q2	NOTEST	0	0	0	0	1	1	no

ENSG00000176783	ENSG00000176783	RUFY1	5:178977558-179037027	q1	q2	OK	26.2233	25.0042	-0.0686754	-0.130601	0.8341	0.99995	no

ENSG00000176787	ENSG00000176787	OR52E2	11:5079879-5080857	q1	q2	NOTEST	0	0	0	0	1	1	no

ENSG00000176788	ENSG00000176788	BASP1	5:17065706-17276943	q1	q2	OK	179.655	181.535	0.015014	0.0362116	0.9614	0.99995	no

ENSG00000176797	ENSG00000176797	DEFB103A	8:7738725-7740186	q1	q2	NOTEST	0	0	0	0	1	1	no

ENSG00000176798	ENSG00000176798	OR51L1	11:5020212-5021160	q1	q2	NOTEST	0	0	0	0	1	1	no

ENSG00000176809	ENSG00000176809	LRRC37A3	17:62850429-62915598	q1	q2	OK	4.61203	4.41163	-0.0640902	-0.083234	0.8814	0.99995	no

ENSG00000176812	ENSG00000176812	RP11-629O4.1	X:25212497-25213324	q1	q2	NOTEST	0	0	0	0	1	1	no

ENSG00000176824	ENSG00000176824	AC009951.1	2:145141647-145282147	q1	q2	NOTEST	0.0514547	0.0944362	0.876037	0	1	1	no

ENSG00000176826	ENSG00000176826	FKBP9L	7:55748766-55780945	q1	q2	OK	1.39851	1.61121	0.204249	0.247179	0.7146	0.99995	no

ENSG00000176834	ENSG00000176834	VSIG10	12:118501397-118583389	q1	q2	OK	11.7859	9.73115	-0.276374	-0.11214	0.65495	0.99995	no

ENSG00000176840	ENSG00000176840	MIR7-3HG	19:4769116-4772545	q1	q2	OK	0.735814	0.339362	-1.11652	-0.473748	0.1443	0.99995	no

ENSG00000176842	ENSG00000176842	IRX5	16:54964773-54968397	q1	q2	OK	9.6756	8.23534	-0.232523	-0.41602	0.5564	0.99995	no

ENSG00000176845	ENSG00000176845	METRNL	17:81037566-81052864	q1	q2	OK	13.1171	11.5076	-0.188866	-0.30038	0.6587	0.99995	no

ENSG00000176853	ENSG00000176853	FAM91A1	8:124780695-124827692	q1	q2	OK	11.8985	11.8393	-0.00719426	-0.0112546	0.985	0.99995	no

ENSG00000176855	ENSG00000176855	KRT18P28	1:182928208-182929540	q1	q2	NOTEST	0	0	0	0	1	1	no

ENSG00000176857	ENSG00000176857	GJA1P1	5:108083522-108532542	q1	q2	NOTEST	0.0781828	0.0244906	-1.67462	0	1	1	no

ENSG00000176868	ENSG00000176868	AL358781.1	9:134269479-134375584	q1	q2	OK	32.1254	33.0419	0.0405821	0.0156481	0.8419	0.99995	no

ENSG00000176871	ENSG00000176871	WSB2	12:118451392-118500235	q1	q2	OK	36.2312	39.0172	0.106876	0.192519	0.76975	0.99995	no

ENSG00000176879	ENSG00000176879	OR51G1	11:4726156-5013659	q1	q2	NOTEST	0	0	0	0	1	1	no

ENSG00000176882	ENSG00000176882	RP11-432M24.4	13:50194449-50194784	q1	q2	NOTEST	0	0	0	0	1	1	no

ENSG00000176884	ENSG00000176884	GRIN1	9:140032841-140063207	q1	q2	NOTEST	0.190137	0.434095	1.19097	0	1	1	no

ENSG00000176887	ENSG00000176887	SOX11	2:5832798-5848227	q1	q2	OK	115.245	129.232	0.165265	0.339494	0.62445	0.99995	no

ENSG00000176890	ENSG00000176890	TYMS	18:596987-712676	q1	q2	OK	229.601	220.056	-0.0612573	-0.128081	0.8509	0.99995	no

ENSG00000176893	ENSG00000176893	OR51G2	11:4726156-5013659	q1	q2	NOTEST	0	0	0	0	1	1	no

ENSG00000176894	ENSG00000176894	PXMP2	12:133264191-133299228	q1	q2	OK	27.0497	27.3035	0.0134737	0.017176	0.9792	0.99995	no

ENSG00000176895	ENSG00000176895	OR51A7	11:4726156-5013659	q1	q2	NOTEST	0	0	0	0	1	1	no

ENSG00000176896	ENSG00000176896	TCEANC	X:13671224-13700083	q1	q2	OK	2.28499	2.08454	-0.132453	-0.169918	0.79775	0.99995	no

ENSG00000176900	ENSG00000176900	OR51T1	11:4726156-5013659	q1	q2	NOTEST	0	0	0	0	1	1	no

ENSG00000176903	ENSG00000176903	PNMA1	14:74178493-74181128	q1	q2	OK	59.6782	61.1263	0.0345892	0.0859171	0.9018	0.99995	no

ENSG00000176904	ENSG00000176904	OR51H1P	11:4726156-5013659	q1	q2	NOTEST	0	0	0	0	1	1	no

ENSG00000176907	ENSG00000176907	C8orf4	8:40010988-40012821	q1	q2	OK	8.59121	10.2925	0.260659	0.447765	0.5132	0.99995	no

ENSG00000176909	ENSG00000176909	MAMSTR	19:49215998-49222978	q1	q2	OK	0.869367	0.995805	0.195899	0.164884	0.76805	0.99995	no

ENSG00000176912	ENSG00000176912	C18orf56	18:596987-712676	q1	q2	OK	4.09272	3.48459	-0.232071	-0.0515365	0.9086	0.99995	no

ENSG00000176915	ENSG00000176915	ANKLE2	12:133302253-133338474	q1	q2	OK	44.6787	46.5906	0.0604524	0.106999	0.86205	0.99995	no

ENSG00000176919	ENSG00000176919	C8G	9:139839697-139841426	q1	q2	NOTEST	0.177135	0.232173	0.390351	0	1	1	no

ENSG00000176920	ENSG00000176920	FUT2	19:49199227-49209207	q1	q2	NOTEST	0.164709	0.49048	1.57428	0	1	1	no

ENSG00000176922	ENSG00000176922	OR51S1	11:4726156-5013659	q1	q2	NOTEST	0	0	0	0	1	1	no

ENSG00000176923	ENSG00000176923	OR7A15P	19:15037869-15038836	q1	q2	NOTEST	0	0	0	0	1	1	no

ENSG00000176925	ENSG00000176925	OR51F2	11:4726156-5013659	q1	q2	NOTEST	0	0	0	0	1	1	no

ENSG00000176927	ENSG00000176927	EFCAB5	17:27887564-28513493	q1	q2	NOTEST	0.444706	0.489305	0.137883	0	1	1	no

ENSG00000176928	ENSG00000176928	GCNT4	5:74323288-74326724	q1	q2	OK	0.438395	0.519143	0.243898	0.251741	0.7253	0.99995	no

ENSG00000176933	ENSG00000176933	TOB2P1	6:28185420-28186655	q1	q2	NOTEST	0.164242	0.274978	0.743498	0	1	1	no

ENSG00000176937	ENSG00000176937	OR52R1	11:4726156-5013659	q1	q2	NOTEST	0	0	0	0	1	1	no

ENSG00000176945	ENSG00000176945	MUC20	3:195384932-195467994	q1	q2	OK	0.707298	0.81844	0.21056	0.0714353	0.8331	0.99995	no

ENSG00000176946	ENSG00000176946	THAP4	2:242523819-242613272	q1	q2	OK	17.5916	17.3158	-0.0227979	-0.0263163	0.97075	0.99995	no

ENSG00000176951	ENSG00000176951	OR51N1P	11:4726156-5013659	q1	q2	NOTEST	0	0	0	0	1	1	no

ENSG00000176953	ENSG00000176953	NFATC2IP	16:28962127-28978418	q1	q2	OK	39.0893	36.8324	-0.085799	-0.142213	0.82145	0.99995	no

ENSG00000176956	ENSG00000176956	LY6H	8:144239330-144242128	q1	q2	OK	5.28558	3.89378	-0.440889	-0.547367	0.4478	0.99995	no

ENSG00000176970	ENSG00000176970	RPL7L1P11	X:147546227-147546948	q1	q2	NOTEST	0	0	0	0	1	1	no

ENSG00000176971	ENSG00000176971	FIBIN	11:27015627-27018630	q1	q2	OK	5.17964	3.7745	-0.456567	-0.663474	0.35435	0.99995	no

ENSG00000176973	ENSG00000176973	FAM89B	11:65337900-65341669	q1	q2	OK	37.114	32.8824	-0.174645	-0.336851	0.6329	0.99995	no

ENSG00000176974	ENSG00000176974	SHMT1	17:18231186-18266856	q1	q2	OK	16.7103	14.8023	-0.174915	-0.232984	0.6541	0.99995	no

ENSG00000176978	ENSG00000176978	DPP7	9:140004993-140009629	q1	q2	OK	8.29293	6.78171	-0.290233	-0.323871	0.6499	0.99995	no

ENSG00000176979	ENSG00000176979	TRIM60	4:165953104-165962896	q1	q2	NOTEST	0	0	0	0	1	1	no

ENSG00000176984	ENSG00000176984	AP000679.2	11:119981982-120056237	q1	q2	NOTEST	0.0133036	0	#NAME?	0	1	1	no

ENSG00000176986	ENSG00000176986	SEC24C	10:75504119-75531919	q1	q2	OK	42.7272	38.2134	-0.161076	-0.346274	0.59165	0.99995	no

ENSG00000176988	ENSG00000176988	FMR1NB	X:147062848-147108187	q1	q2	NOTEST	0	0	0	0	1	1	no

ENSG00000176994	ENSG00000176994	SMCR8	17:18218623-18226517	q1	q2	OK	4.24237	4.45904	0.0718626	0.123637	0.865	0.99995	no

ENSG00000176998	ENSG00000176998	HCG4	6:29758730-29765588	q1	q2	NOTEST	0	0	0	0	1	1	no

ENSG00000177000	ENSG00000177000	MTHFR	1:11821843-11908402	q1	q2	OK	9.23433	8.37072	-0.141655	-0.108031	0.7898	0.99995	no

ENSG00000177023	ENSG00000177023	DEFB104B	8:7327829-7332604	q1	q2	NOTEST	0	0	0	0	1	1	no

ENSG00000177025	ENSG00000177025	C19orf18	19:58469804-58485902	q1	q2	NOTEST	0.372589	0.460097	0.304355	0	1	1	no

ENSG00000177030	ENSG00000177030	DEAF1	11:644232-727727	q1	q2	OK	24.8388	21.6578	-0.197709	-0.285527	0.5869	0.99995	no

ENSG00000177034	ENSG00000177034	MTX3	5:79275583-79287082	q1	q2	OK	7.40178	8.31374	0.167626	0.292442	0.6676	0.99995	no

ENSG00000177042	ENSG00000177042	TMEM80	11:644232-727727	q1	q2	OK	18.9265	17.9636	-0.0753253	-0.0780433	0.91395	0.99995	no

ENSG00000177045	ENSG00000177045	SIX5	19:46268042-46296060	q1	q2	OK	14.334	12.3445	-0.215574	-0.214547	0.75665	0.99995	no

ENSG00000177047	ENSG00000177047	IFNW1	9:21140630-21142144	q1	q2	NOTEST	0	0.12409	inf	0	1	1	no

ENSG00000177051	ENSG00000177051	FBXO46	19:46213886-46234162	q1	q2	OK	12.9853	12.7001	-0.0320397	-0.0456151	0.9418	0.99995	no

ENSG00000177054	ENSG00000177054	ZDHHC13	11:19138645-19197969	q1	q2	OK	7.70918	8.65952	0.167709	0.277814	0.6712	0.99995	no

ENSG00000177058	ENSG00000177058	SLC38A9	5:54921672-55112985	q1	q2	OK	10.2549	10.1758	-0.011178	-0.0146696	0.97545	0.99995	no

ENSG00000177076	ENSG00000177076	ACER2	9:19408924-19452018	q1	q2	OK	3.03334	2.93399	-0.0480433	-0.0566305	0.9269	0.99995	no

ENSG00000177082	ENSG00000177082	WDR73	15:85185998-85197574	q1	q2	OK	29.2322	27.3394	-0.0965805	-0.183148	0.77615	0.99995	no

ENSG00000177084	ENSG00000177084	POLE	12:133200347-133263951	q1	q2	OK	54.4649	50.4407	-0.11074	-0.113821	0.7846	0.99995	no

ENSG00000177096	ENSG00000177096	FAM109B	22:42470254-42475445	q1	q2	NOTEST	0.0234388	0	#NAME?	0	1	1	no

ENSG00000177098	ENSG00000177098	SCN4B	11:118004091-118023603	q1	q2	OK	1.71707	1.18822	-0.531147	-0.600816	0.3146	0.99995	no

ENSG00000177103	ENSG00000177103	DSCAML1	11:117298488-117748201	q1	q2	NOTEST	0.0243519	0.0206353	-0.238919	0	1	1	no

ENSG00000177105	ENSG00000177105	RHOG	11:3848207-3862213	q1	q2	OK	10.1786	9.86789	-0.0447285	-0.0709084	0.91865	0.99995	no

ENSG00000177106	ENSG00000177106	EPS8L2	11:644232-727727	q1	q2	OK	0.582518	0.687439	0.238929	0.0471596	0.632	0.99995	no

ENSG00000177108	ENSG00000177108	ZDHHC22	14:77564439-77725838	q1	q2	OK	20.0168	16.041	-0.319446	-0.48629	0.4483	0.99995	no

ENSG00000177112	ENSG00000177112	MRVI1-AS1	11:10562818-10715535	q1	q2	NOTEST	0	0.0632783	inf	0	1	1	no

ENSG00000177119	ENSG00000177119	ANO6	12:45566846-45834187	q1	q2	OK	19.0615	19.988	0.0684693	0.161995	0.8084	0.99995	no

ENSG00000177125	ENSG00000177125	ZBTB34	9:129622943-129648157	q1	q2	OK	6.7138	6.88685	0.0367141	0.0823951	0.9074	0.99995	no

ENSG00000177133	ENSG00000177133	LINC00982	1:2976178-2985001	q1	q2	NOTEST	0.45631	0.276748	-0.721437	0	1	1	no

ENSG00000177138	ENSG00000177138	FAM9B	X:8992272-9132680	q1	q2	NOTEST	0	0	0	0	1	1	no

ENSG00000177143	ENSG00000177143	CETN1	18:580366-581524	q1	q2	NOTEST	0	0	0	0	1	1	no

ENSG00000177144	ENSG00000177144	NUDT4P1	1:145139024-145139569	q1	q2	NOTEST	0.212664	0.206536	-0.0421838	0	1	1	no

ENSG00000177150	ENSG00000177150	FAM210A	18:13663345-13764557	q1	q2	OK	5.1389	5.32955	0.0525545	0.0370431	0.95045	0.99995	no

ENSG00000177151	ENSG00000177151	OR2T35	1:248801587-248802559	q1	q2	NOTEST	0	0	0	0	1	1	no

ENSG00000177156	ENSG00000177156	TALDO1	11:747328-765024	q1	q2	OK	79.4767	72.736	-0.127864	-0.299531	0.65945	0.99995	no

ENSG00000177169	ENSG00000177169	ULK1	12:132379195-132407712	q1	q2	OK	25.9204	20.372	-0.347502	-0.666232	0.2889	0.99995	no

ENSG00000177173	ENSG00000177173	NAP1L4P1	1:117057156-117113661	q1	q2	NOTEST	0.224559	0.321955	0.519764	0	1	1	no

ENSG00000177174	ENSG00000177174	OR14C36	1:248512076-248513015	q1	q2	NOTEST	0	0	0	0	1	1	no

ENSG00000177181	ENSG00000177181	RIMKLA	1:42846465-42888249	q1	q2	OK	1.80315	1.47474	-0.29006	-0.487086	0.47985	0.99995	no

ENSG00000177182	ENSG00000177182	CLVS1	8:61969716-62627155	q1	q2	OK	3.20689	3.16652	-0.0182779	-0.0065056	0.9879	0.99995	no

ENSG00000177186	ENSG00000177186	OR2M7	1:248486893-248487899	q1	q2	NOTEST	0	0	0	0	1	1	no

ENSG00000177189	ENSG00000177189	RPS6KA3	X:20168028-20285523	q1	q2	OK	11.2632	13.2348	0.232716	0.453228	0.4946	0.99995	no

ENSG00000177191	ENSG00000177191	B3GNT8	19:41931263-41934635	q1	q2	NOTEST	0.301512	0.38772	0.362799	0	1	1	no

ENSG00000177192	ENSG00000177192	PUS1	12:132409280-132428406	q1	q2	OK	10.0551	10.5005	0.0625338	0.102867	0.8763	0.99995	no

ENSG00000177197	ENSG00000177197	PCNPP5	13:48877886-49056122	q1	q2	OK	1.29516	1.70513	0.396743	0.0487454	0.83435	0.99995	no

ENSG00000177200	ENSG00000177200	CHD9	16:53088944-53363062	q1	q2	OK	36.6563	39.1487	0.0949012	0.099511	0.78865	0.99995	no

ENSG00000177201	ENSG00000177201	OR2T12	1:248457917-248458880	q1	q2	NOTEST	0	0	0	0	1	1	no

ENSG00000177202	ENSG00000177202	SPACA4	19:49104066-49118111	q1	q2	NOTEST	0.251427	0.0300638	-3.06404	0	1	1	no

ENSG00000177212	ENSG00000177212	OR2T33	1:248436072-248437138	q1	q2	NOTEST	0.0274039	0.0551492	1.00896	0	1	1	no

ENSG00000177225	ENSG00000177225	PDDC1	11:767219-777488	q1	q2	OK	27.0007	24.6915	-0.128983	-0.138798	0.7313	0.99995	no

ENSG00000177233	ENSG00000177233	OR2M1P	1:248285233-248286184	q1	q2	NOTEST	0	0	0	0	1	1	no

ENSG00000177234	ENSG00000177234	C10orf85	10:122357720-122359629	q1	q2	NOTEST	0	0.0423336	inf	0	1	1	no

ENSG00000177236	ENSG00000177236	AP006621.1	11:777577-784297	q1	q2	OK	1.62429	1.4844	-0.129931	-0.0722566	0.90255	0.99995	no

ENSG00000177238	ENSG00000177238	TRIM72	16:31225341-31236510	q1	q2	NOTEST	0.0243132	0.024427	0.00673857	0	1	1	no

ENSG00000177239	ENSG00000177239	MAN1B1	9:139981378-140003635	q1	q2	OK	25.2134	22.0804	-0.191422	-0.408147	0.5455	0.99995	no

ENSG00000177243	ENSG00000177243	DEFB103B	8:7286409-7287870	q1	q2	NOTEST	0	0	0	0	1	1	no

ENSG00000177257	ENSG00000177257	DEFB4B	8:7272381-7274385	q1	q2	NOTEST	0	0	0	0	1	1	no

ENSG00000177261	ENSG00000177261	ENSAP3	2:215562450-215562788	q1	q2	NOTEST	0	0	0	0	1	1	no

ENSG00000177272	ENSG00000177272	KCNA3	1:111214309-111217655	q1	q2	OK	1.09513	1.07557	-0.0260042	-0.0324901	0.9613	0.99995	no

ENSG00000177275	ENSG00000177275	OR2AJ1	1:248097070-248098057	q1	q2	NOTEST	0	0	0	0	1	1	no

ENSG00000177283	ENSG00000177283	FZD8	10:35927176-35930362	q1	q2	OK	1.36797	1.36301	-0.00524166	-0.00663326	0.994	0.99995	no

ENSG00000177291	ENSG00000177291	GJD4	10:35894337-35897863	q1	q2	NOTEST	0	0.0178594	inf	0	1	1	no

ENSG00000177294	ENSG00000177294	FBXO39	17:6679541-6735080	q1	q2	NOTEST	0.0491652	0.111023	1.17515	0	1	1	no

ENSG00000177300	ENSG00000177300	CLDN22	4:184020445-184241930	q1	q2	NOTEST	0	0	0	0	1	1	no

ENSG00000177301	ENSG00000177301	KCNA2	1:111136201-111174096	q1	q2	OK	0.718897	1.10229	0.616651	0.550294	0.37145	0.99995	no

ENSG00000177302	ENSG00000177302	TOP3A	17:18174741-18218321	q1	q2	OK	29.3418	29.2791	-0.00308575	-0.00400456	0.99395	0.99995	no

ENSG00000177303	ENSG00000177303	CASKIN2	17:73496341-73511664	q1	q2	OK	9.37221	7.93887	-0.239455	-0.283935	0.50045	0.99995	no

ENSG00000177306	ENSG00000177306	OR7E125P	8:7104247-7105874	q1	q2	NOTEST	0	0	0	0	1	1	no

ENSG00000177311	ENSG00000177311	ZBTB38	3:140947567-141168634	q1	q2	OK	3.86711	4.69689	0.280451	0.176382	0.5744	0.99995	no

ENSG00000177324	ENSG00000177324	BEND2	X:18181050-18239024	q1	q2	NOTEST	0	0.0120917	inf	0	1	1	no

ENSG00000177335	ENSG00000177335	C8orf31	8:144120625-144141359	q1	q2	NOTEST	0.128498	0.226312	0.81657	0	1	1	no

ENSG00000177337	ENSG00000177337	DLGAP1-AS1	18:3496029-4455335	q1	q2	OK	1.68353	2.45064	0.541672	0.202538	0.56515	0.99995	no

ENSG00000177338	ENSG00000177338	LINC00469	17:71733992-71824659	q1	q2	NOTEST	0	0	0	0	1	1	no

ENSG00000177340	ENSG00000177340	AC024940.1	12:31433517-31479992	q1	q2	NOTEST	0.116306	0.148447	0.352027	0	1	1	no

ENSG00000177350	ENSG00000177350	RPL13AP3	14:56214925-56239921	q1	q2	NOTEST	0.0186414	0.0496547	1.41342	0	1	1	no

ENSG00000177352	ENSG00000177352	CCDC71	3:49199967-49203754	q1	q2	OK	12.9207	12.5866	-0.0377936	-0.0712933	0.9184	0.99995	no

ENSG00000177354	ENSG00000177354	C10orf71	10:50507186-50535537	q1	q2	NOTEST	0	0	0	0	1	1	no

ENSG00000177359	ENSG00000177359	RP11-551L14.1	12:31264585-31359088	q1	q2	OK	1.61061	2.92096	0.858832	0.627353	0.39645	0.99995	no

ENSG00000177363	ENSG00000177363	LRRN4CL	11:62453873-62457371	q1	q2	OK	1.83804	2.48318	0.434019	0.550598	0.42705	0.99995	no

ENSG00000177369	ENSG00000177369	RP1-62O9.3	17:47325600-47336024	q1	q2	NOTEST	0	0.0449134	inf	0	1	1	no

ENSG00000177370	ENSG00000177370	TIMM22	17:900356-1132315	q1	q2	OK	11.0925	10.8314	-0.0343588	-0.0420542	0.9541	0.99995	no

ENSG00000177374	ENSG00000177374	HIC1	17:1957447-1962981	q1	q2	OK	7.80373	5.91824	-0.398996	-0.421386	0.44065	0.99995	no

ENSG00000177380	ENSG00000177380	PPFIA3	19:49622645-49654283	q1	q2	OK	8.89919	7.4161	-0.263013	-0.333814	0.63005	0.99995	no

ENSG00000177383	ENSG00000177383	MAGEF1	3:184428155-184429836	q1	q2	OK	79.772	80.9074	0.0203896	0.0503216	0.94345	0.99995	no

ENSG00000177398	ENSG00000177398	UMODL1	21:43483067-43563563	q1	q2	NOTEST	0.00979743	0	#NAME?	0	1	1	no

ENSG00000177400	ENSG00000177400	OR7E8P	8:12541662-12542662	q1	q2	NOTEST	0	0	0	0	1	1	no

ENSG00000177406	ENSG00000177406	RP11-218M22.1	12:673461-772945	q1	q2	NOTEST	0.0369967	0.0247377	-0.580686	0	1	1	no

ENSG00000177409	ENSG00000177409	SAMD9L	7:92759367-92777682	q1	q2	NOTEST	0.146975	0.272579	0.891106	0	1	1	no

ENSG00000177410	ENSG00000177410	ZFAS1	20:47835883-47905797	q1	q2	OK	109.795	110.002	0.00271774	0.0037367	0.99555	0.99995	no

ENSG00000177414	ENSG00000177414	UBE2U	1:64669309-64733051	q1	q2	NOTEST	0	0	0	0	1	1	no

ENSG00000177418	ENSG00000177418	AC006001.1	7:66010633-66057373	q1	q2	NOTEST	0.0745862	0	#NAME?	0	1	1	no

ENSG00000177425	ENSG00000177425	PAWR	12:79968758-80334448	q1	q2	OK	12.8985	12.0304	-0.100519	-0.0618812	0.88415	0.99995	no

ENSG00000177426	ENSG00000177426	TGIF1	18:3411605-3458409	q1	q2	OK	38.3746	38.4391	0.00242632	0.00474919	0.99485	0.99995	no

ENSG00000177427	ENSG00000177427	MIEF2	17:18163847-18169866	q1	q2	OK	5.68524	3.90433	-0.542146	-0.654301	0.2611	0.99995	no

ENSG00000177432	ENSG00000177432	NAP1L5	4:89442135-89629693	q1	q2	OK	12.4116	12.8388	0.0488197	0.061458	0.93425	0.99995	no

ENSG00000177447	ENSG00000177447	CBX3P1	11:27528384-27899195	q1	q2	OK	1.16706	1.14112	-0.0324342	-0.0103536	0.90515	0.99995	no

ENSG00000177452	ENSG00000177452	RP4-597J3.1	1:64239692-64647181	q1	q2	NOTEST	0	0	0	0	1	1	no

ENSG00000177453	ENSG00000177453	NIM1K	5:43192172-43280952	q1	q2	OK	0.497388	0.505842	0.0243146	0.0203141	0.9678	0.99995	no

ENSG00000177455	ENSG00000177455	CD19	16:28889725-28950667	q1	q2	NOTEST	0.0144452	0	#NAME?	0	1	1	no

ENSG00000177459	ENSG00000177459	C8orf47	8:99076538-99105838	q1	q2	NOTEST	0.0464381	0.120956	1.3811	0	1	1	no

ENSG00000177462	ENSG00000177462	OR2T8	1:248084319-248085258	q1	q2	NOTEST	0.0321142	0	#NAME?	0	1	1	no

ENSG00000177463	ENSG00000177463	NR2C2	3:14860468-15106842	q1	q2	OK	10.3691	11.1469	0.10436	0.0501626	0.92505	0.99995	no

ENSG00000177464	ENSG00000177464	GPR4	19:46030684-46105470	q1	q2	NOTEST	0.284187	0.26871	-0.080787	0	1	1	no

ENSG00000177465	ENSG00000177465	ACOT4	14:74058409-74063200	q1	q2	NOTEST	0.0664515	0.0212834	-1.64257	0	1	1	no

ENSG00000177468	ENSG00000177468	OLIG3	6:137813335-137815531	q1	q2	NOTEST	0.410042	0.307236	-0.416423	0	1	1	no

ENSG00000177469	ENSG00000177469	PTRF	17:40554469-40575535	q1	q2	OK	20.1443	19.4414	-0.0512406	-0.103455	0.88365	0.99995	no

ENSG00000177476	ENSG00000177476	OR2G3	1:247670359-247910364	q1	q2	NOTEST	0	0	0	0	1	1	no

ENSG00000177479	ENSG00000177479	ARIH2	3:48955220-49060928	q1	q2	OK	48.7926	46.116	-0.0813971	-0.0537968	0.8718	0.99995	no

ENSG00000177483	ENSG00000177483	RBM44	2:238536218-238751451	q1	q2	OK	0.625186	0.628849	0.00842842	0.00312421	0.9963	0.99995	no

ENSG00000177485	ENSG00000177485	ZBTB33	X:119384606-119392253	q1	q2	NOTEST	0.209487	0.145467	-0.526168	0	1	1	no

ENSG00000177489	ENSG00000177489	OR2G2	1:247670359-247910364	q1	q2	NOTEST	0	0	0	0	1	1	no

ENSG00000177494	ENSG00000177494	ZBED2	3:111011565-111384597	q1	q2	NOTEST	0	0.0108999	inf	0	1	1	no

ENSG00000177504	ENSG00000177504	VCX2	X:8137988-8139308	q1	q2	NOTEST	0	0	0	0	1	1	no

ENSG00000177508	ENSG00000177508	IRX3	16:54317215-54320675	q1	q2	OK	13.7366	11.1392	-0.302382	-0.494358	0.41705	0.99995	no

ENSG00000177511	ENSG00000177511	ST8SIA3	18:55018043-55038962	q1	q2	NOTEST	0.278024	0.164235	-0.759443	0	1	1	no

ENSG00000177519	ENSG00000177519	RPRM	2:154277190-154449801	q1	q2	OK	3.20112	2.98299	-0.101815	-0.127715	0.85995	0.99995	no

ENSG00000177535	ENSG00000177535	OR2B11	1:247614254-247615308	q1	q2	NOTEST	0	0	0	0	1	1	no

ENSG00000177542	ENSG00000177542	SLC25A22	11:790474-798316	q1	q2	OK	23.7361	21.8501	-0.119444	-0.186933	0.7339	0.99995	no

ENSG00000177548	ENSG00000177548	RABEP2	16:28889725-28950667	q1	q2	OK	10.6428	8.77236	-0.278847	-0.396113	0.48725	0.99995	no

ENSG00000177551	ENSG00000177551	NHLH2	1:116378997-116386538	q1	q2	OK	0.652482	0.64522	-0.0161468	-0.0158924	0.98045	0.99995	no

ENSG00000177553	ENSG00000177553	RP11-56N19.5	1:11821843-11908402	q1	q2	NOTEST	0	0	0	0	1	1	no

ENSG00000177556	ENSG00000177556	ATOX1	5:151121876-151192346	q1	q2	OK	51.922	44.6733	-0.216932	-0.103871	0.91845	0.99995	no

ENSG00000177558	ENSG00000177558	FAM187B	19:35715702-35719632	q1	q2	NOTEST	0	0	0	0	1	1	no

ENSG00000177565	ENSG00000177565	TBL1XR1	3:176737142-176915261	q1	q2	OK	28.1897	30.9896	0.136617	0.200403	0.6519	0.99995	no

ENSG00000177570	ENSG00000177570	SAMD12	8:119201697-119634234	q1	q2	OK	0.537588	0.477471	-0.171087	-0.0813859	0.7955	0.99995	no

ENSG00000177575	ENSG00000177575	CD163	12:7499280-7656489	q1	q2	NOTEST	0	0.00677493	inf	0	1	1	no

ENSG00000177576	ENSG00000177576	C18orf32	18:47008027-47018906	q1	q2	OK	70.372	78.9931	0.166725	0.138228	0.8444	0.99995	no

ENSG00000177586	ENSG00000177586	OR7E149P	12:8589998-8591021	q1	q2	NOTEST	0	0	0	0	1	1	no

ENSG00000177590	ENSG00000177590	RP5-1051J4.6	7:150413644-150487837	q1	q2	NOTEST	0	0	0	0	1	1	no

ENSG00000177595	ENSG00000177595	PIDD	11:799178-812880	q1	q2	OK	10.9917	8.07569	-0.444751	-0.336667	0.6369	0.99995	no

ENSG00000177596	ENSG00000177596	AL355390.1	13:74987093-74993252	q1	q2	NOTEST	0	0	0	0	1	1	no

ENSG00000177599	ENSG00000177599	ZNF491	19:11906898-11920437	q1	q2	OK	4.2575	4.2442	-0.00451358	-0.0055642	0.9913	0.99995	no

ENSG00000177600	ENSG00000177600	RPLP2	11:799178-812880	q1	q2	OK	434.359	368.426	-0.237511	-0.380835	0.57255	0.99995	no

ENSG00000177602	ENSG00000177602	GSG2	17:3617921-3704537	q1	q2	OK	6.0096	5.85233	-0.0382581	-0.0518603	0.9418	0.99995	no

ENSG00000177606	ENSG00000177606	JUN	1:59246464-59249785	q1	q2	OK	30.9653	30.1944	-0.0363713	-0.0875262	0.89675	0.99995	no

ENSG00000177613	ENSG00000177613	CSTF2T	10:52750944-54073888	q1	q2	OK	17.688	18.4529	0.0610736	0.110078	0.8778	0.99995	no

ENSG00000177614	ENSG00000177614	PGBD5	1:230457391-230561475	q1	q2	OK	22.0987	23.257	0.0737046	0.172542	0.8071	0.99995	no

ENSG00000177627	ENSG00000177627	C12orf54	12:48876285-48894744	q1	q2	NOTEST	0	0.0811669	inf	0	1	1	no

ENSG00000177628	ENSG00000177628	GBA	1:155204242-155214490	q1	q2	OK	3.42434	1.97877	-0.791223	-0.947099	0.1381	0.99995	no

ENSG00000177640	ENSG00000177640	CASC2	10:119764426-119969663	q1	q2	OK	1.5425	2.33799	0.599994	0.310959	0.4851	0.99995	no

ENSG00000177646	ENSG00000177646	ACAD9	3:128598438-128759585	q1	q2	OK	24.4462	24.9106	0.0271503	0.0434376	0.94075	0.99995	no

ENSG00000177663	ENSG00000177663	IL17RA	22:17565843-17596583	q1	q2	OK	3.07124	3.2912	0.0997906	0.13067	0.78825	0.99995	no

ENSG00000177666	ENSG00000177666	PNPLA2	11:818901-842545	q1	q2	OK	12.0979	12.2935	0.0231399	0.0151998	0.9717	0.99995	no

ENSG00000177669	ENSG00000177669	MBOAT4	8:29952913-30041156	q1	q2	OK	0.708215	0.803641	0.182364	0.0270478	0.9189	0.99995	no

ENSG00000177673	ENSG00000177673	C2orf57	2:232457574-232458994	q1	q2	NOTEST	0	0	0	0	1	1	no

ENSG00000177674	ENSG00000177674	AGTRAP	1:11796140-11814859	q1	q2	OK	17.1787	14.4901	-0.245558	-0.420966	0.54325	0.99995	no

ENSG00000177675	ENSG00000177675	CD163L1	12:7499280-7656489	q1	q2	NOTEST	0.00553894	0	#NAME?	0	1	1	no

ENSG00000177679	ENSG00000177679	SRRM3	7:75831215-75916605	q1	q2	NOTEST	0.0432252	0.215101	2.31507	0	1	1	no

ENSG00000177683	ENSG00000177683	THAP5	7:108110865-108215294	q1	q2	OK	16.9936	18.2941	0.106384	0.121254	0.8215	0.99995	no

ENSG00000177684	ENSG00000177684	DEFB114	6:49927961-49931818	q1	q2	NOTEST	0	0	0	0	1	1	no

ENSG00000177685	ENSG00000177685	EFCAB4A	11:818901-842545	q1	q2	OK	1.77853	1.28742	-0.466202	-0.123167	0.53955	0.99995	no

ENSG00000177688	ENSG00000177688	SUMO4	6:149539061-149732749	q1	q2	OK	1.06029	1.43099	0.432549	0.0729288	0.82255	0.99995	no

ENSG00000177689	ENSG00000177689	MAGEB10	X:27826106-27841131	q1	q2	NOTEST	0	0	0	0	1	1	no

ENSG00000177692	ENSG00000177692	DNAJC28	21:34860496-34864027	q1	q2	NOTEST	0	0.0200076	inf	0	1	1	no

ENSG00000177693	ENSG00000177693	OR4F4	15:102462244-102463298	q1	q2	NOTEST	0	0	0	0	1	1	no

ENSG00000177694	ENSG00000177694	NAALADL2	3:174156362-175523428	q1	q2	OK	0.748417	0.919896	0.297628	0.123233	0.73815	0.99995	no

ENSG00000177697	ENSG00000177697	CD151	11:818901-842545	q1	q2	OK	56.1402	51.1008	-0.135689	-0.233297	0.7243	0.99995	no

ENSG00000177699	ENSG00000177699	RP11-16K12.1	15:79252288-79383115	q1	q2	NOTEST	0.0139588	0.0135489	-0.0430006	0	1	1	no

ENSG00000177700	ENSG00000177700	POLR2L	11:818901-842545	q1	q2	OK	78.6649	74.1359	-0.0855477	-0.107079	0.8792	0.99995	no

ENSG00000177706	ENSG00000177706	FAM20C	7:182934-300711	q1	q2	OK	10.6151	8.79153	-0.271931	-0.410359	0.4723	0.99995	no

ENSG00000177707	ENSG00000177707	PVRL3	3:110612554-110994410	q1	q2	OK	22.4583	23.8884	0.0890612	0.180225	0.7813	0.99995	no

ENSG00000177710	ENSG00000177710	SLC35G5	8:11188396-11189717	q1	q2	NOTEST	0.257543	0.252713	-0.0273142	0	1	1	no

ENSG00000177721	ENSG00000177721	ANXA2R	5:43014515-43192123	q1	q2	NOTEST	0.239048	0.210974	-0.180237	0	1	1	no

ENSG00000177725	ENSG00000177725	AC105206.1	8:22022248-22069839	q1	q2	NOTEST	0.112175	0.0673492	-0.736013	0	1	1	no

ENSG00000177728	ENSG00000177728	KIAA0195	17:73437239-73496171	q1	q2	OK	45.6642	37.8926	-0.269148	-0.334358	0.4068	0.99995	no

ENSG00000177731	ENSG00000177731	FLII	17:18128900-18162230	q1	q2	OK	59.641	50.9698	-0.226662	-0.202498	0.6571	0.99995	no

ENSG00000177732	ENSG00000177732	SOX12	20:300956-340304	q1	q2	OK	44.4755	42.8653	-0.0531993	-0.108898	0.87875	0.99995	no

ENSG00000177733	ENSG00000177733	HNRNPA0	5:137087074-137090039	q1	q2	OK	172.454	173.451	0.00831545	0.0194833	0.97875	0.99995	no

ENSG00000177736	ENSG00000177736	RP11-474P12.3	9:127640645-127905785	q1	q2	NOTEST	0.0683708	0.320971	2.23099	0	1	1	no

ENSG00000177738	ENSG00000177738	CTD-2201E18.3	5:43014515-43192123	q1	q2	OK	0.610439	0.826415	0.437019	0.122914	0.75275	0.99995	no

ENSG00000177752	ENSG00000177752	YIPF7	4:44624085-44728612	q1	q2	NOTEST	0.294608	0.112576	-1.3879	0	1	1	no

ENSG00000177757	ENSG00000177757	FAM87B	1:714149-755214	q1	q2	NOTEST	0.0702629	0.0537555	-0.38635	0	1	1	no

ENSG00000177764	ENSG00000177764	ZCCHC3	20:277736-280965	q1	q2	OK	13.4296	12.9864	-0.0484157	-0.0681935	0.92555	0.99995	no

ENSG00000177770	ENSG00000177770	CDKN2AIPNLP1	1:226680888-226681190	q1	q2	NOTEST	0	0	0	0	1	1	no

ENSG00000177776	ENSG00000177776	RPS2P2	14:74844520-74845121	q1	q2	NOTEST	0	0	0	0	1	1	no

ENSG00000177788	ENSG00000177788	RP5-1061H20.4	1:229394027-229441641	q1	q2	OK	1.65255	1.41406	-0.224845	-0.0317214	0.91185	0.99995	no

ENSG00000177791	ENSG00000177791	MYOZ1	10:75391411-75401515	q1	q2	NOTEST	0.0707843	0	#NAME?	0	1	1	no

ENSG00000177800	ENSG00000177800	TMEM78	1:229385382-229387557	q1	q2	NOTEST	0.012015	0.0116538	-0.0440351	0	1	1	no

ENSG00000177803	ENSG00000177803	RP11-606P2.1	4:164445449-165305202	q1	q2	OK	0.210744	0.518848	1.29982	0.223545	0.63525	0.99995	no

ENSG00000177807	ENSG00000177807	KCNJ10	1:160007256-160049611	q1	q2	OK	1.76929	1.38888	-0.349252	-0.509052	0.43545	0.99995	no

ENSG00000177820	ENSG00000177820	AC004917.1	7:106012696-106410653	q1	q2	NOTEST	0	0	0	0	1	1	no

ENSG00000177822	ENSG00000177822	AC108142.1	4:182795590-183724177	q1	q2	OK	3.07407	3.50719	0.190166	0.05068	0.8743	0.99995	no

ENSG00000177830	ENSG00000177830	CHID1	11:867356-915058	q1	q2	OK	33.5894	30.3599	-0.145839	-0.280859	0.6555	0.99995	no

ENSG00000177839	ENSG00000177839	PCDHB9	5:140566892-140571111	q1	q2	NOTEST	0.0429227	0.01757	-1.28862	0	1	1	no

ENSG00000177842	ENSG00000177842	ZNF620	3:40547482-40560227	q1	q2	OK	5.90721	6.12109	0.0513105	0.0792236	0.8992	0.99995	no

ENSG00000177853	ENSG00000177853	ZNF518A	10:97889471-98031344	q1	q2	NOTEST	0	0.0146411	inf	0	1	1	no

ENSG00000177854	ENSG00000177854	TMEM187	X:153237777-153248646	q1	q2	NOTEST	0	0	0	0	1	1	no

ENSG00000177855	ENSG00000177855	CACYBPP2	2:184472169-184472847	q1	q2	OK	1.73975	2.7207	0.645099	0.640352	0.3635	0.99995	no

ENSG00000177868	ENSG00000177868	CCDC23	1:43272722-43312093	q1	q2	OK	56.4685	54.3228	-0.0558872	-0.0921278	0.8945	0.99995	no

ENSG00000177873	ENSG00000177873	ZNF619	3:40518603-40531727	q1	q2	OK	1.94323	2.05906	0.0835245	0.107834	0.87185	0.99995	no

ENSG00000177875	ENSG00000177875	C12orf68	12:48577365-48579709	q1	q2	OK	17.4488	16.0985	-0.116204	-0.249073	0.72365	0.99995	no

ENSG00000177879	ENSG00000177879	AP3S1	5:115163892-115249778	q1	q2	OK	55.1601	56.365	0.0311752	0.0491733	0.94205	0.99995	no

ENSG00000177885	ENSG00000177885	GRB2	17:73314156-73401790	q1	q2	OK	35.5843	34.5601	-0.0421356	-0.0753234	0.89255	0.99995	no

ENSG00000177888	ENSG00000177888	ZBTB41	1:197122809-197169672	q1	q2	OK	4.72056	5.24839	0.152918	0.336547	0.6344	0.99995	no

ENSG00000177889	ENSG00000177889	UBE2N	12:93799448-93836038	q1	q2	OK	101.077	104.151	0.043234	0.0750158	0.91105	0.99995	no

ENSG00000177910	ENSG00000177910	SPATA31C2	9:90744219-90753361	q1	q2	NOTEST	0.00664876	0.0133525	1.00595	0	1	1	no

ENSG00000177917	ENSG00000177917	ARL6IP6	2:153508106-153617767	q1	q2	OK	47.2374	53.7702	0.186878	0.196742	0.75885	0.99995	no

ENSG00000177932	ENSG00000177932	ZNF354C	5:178487415-178510538	q1	q2	OK	1.19041	1.65496	0.475346	0.706607	0.30625	0.99995	no

ENSG00000177938	ENSG00000177938	CAPZA3	12:18891044-18892121	q1	q2	NOTEST	0	0	0	0	1	1	no

ENSG00000177943	ENSG00000177943	MAMDC4	9:139743175-139755249	q1	q2	OK	9.95848	7.32896	-0.442317	-0.322967	0.5059	0.99995	no

ENSG00000177946	ENSG00000177946	CENPBD1	16:90036198-90038952	q1	q2	OK	4.85356	4.45446	-0.123792	-0.205246	0.7624	0.99995	no

ENSG00000177947	ENSG00000177947	ODF3	11:127114-207428	q1	q2	NOTEST	0	0	0	0	1	1	no

ENSG00000177951	ENSG00000177951	BET1L	11:127114-207428	q1	q2	OK	16.426	15.2362	-0.108479	-0.207289	0.7375	0.99995	no

ENSG00000177954	ENSG00000177954	RPS27	1:153963234-153964626	q1	q2	OK	2206.01	2100.06	-0.0710093	-0.171089	0.8054	0.99995	no

ENSG00000177963	ENSG00000177963	RIC8A	11:207510-215113	q1	q2	OK	36.9481	34.8963	-0.0824287	-0.135925	0.76835	0.99995	no

ENSG00000177971	ENSG00000177971	IMP3	15:75928479-75954642	q1	q2	OK	31.3442	29.2616	-0.0991906	-0.13683	0.84525	0.99995	no

ENSG00000177981	ENSG00000177981	ASB8	12:48541570-48574996	q1	q2	OK	16.366	14.3809	-0.186549	-0.297724	0.59665	0.99995	no

ENSG00000177984	ENSG00000177984	LCN15	9:139654085-139660707	q1	q2	NOTEST	0.00931694	0	#NAME?	0	1	1	no

ENSG00000177989	ENSG00000177989	ODF3B	22:50961996-50971009	q1	q2	NOTEST	0.484249	0.406217	-0.253498	0	1	1	no

ENSG00000177990	ENSG00000177990	DPY19L2	12:63952692-64217675	q1	q2	OK	5.0688	6.46911	0.351924	0.337965	0.5941	0.99995	no

ENSG00000177992	ENSG00000177992	SPATA31E1	9:90497740-90503814	q1	q2	NOTEST	0	0	0	0	1	1	no

ENSG00000177993	ENSG00000177993	ZNRF3-AS1	22:29279579-29453475	q1	q2	NOTEST	0.0996737	0.0484071	-1.04199	0	1	1	no

ENSG00000177994	ENSG00000177994	C2orf73	2:54557170-54610879	q1	q2	NOTEST	0.161191	0.0152322	-3.40358	0	1	1	no

ENSG00000178015	ENSG00000178015	GPR150	5:94955781-94957846	q1	q2	NOTEST	0	0.0255681	inf	0	1	1	no

ENSG00000178021	ENSG00000178021	TSPYL6	2:54091203-54532437	q1	q2	NOTEST	0.0163932	0.0244025	0.57393	0	1	1	no

ENSG00000178026	ENSG00000178026	FAM211B	22:24936405-25024972	q1	q2	OK	10.3858	8.10992	-0.35685	-0.215565	0.75345	0.99995	no

ENSG00000178028	ENSG00000178028	DMAP1	1:44679126-44686353	q1	q2	OK	32.3288	30.2127	-0.097664	-0.186366	0.7763	0.99995	no

ENSG00000178031	ENSG00000178031	ADAMTSL1	9:18473891-18910948	q1	q2	OK	0.988648	0.866692	-0.189938	-0.127894	0.80025	0.99995	no

ENSG00000178033	ENSG00000178033	FAM26E	6:116813459-116880031	q1	q2	OK	1.79094	2.33152	0.380554	0.705604	0.3081	0.99995	no

ENSG00000178035	ENSG00000178035	IMPDH2	3:49061757-49067139	q1	q2	OK	86.8338	85.8293	-0.0167869	-0.0381632	0.9515	0.99995	no

ENSG00000178038	ENSG00000178038	ALS2CL	3:46710486-46735194	q1	q2	NOTEST	0.117164	0.157009	0.422315	0	1	1	no

ENSG00000178053	ENSG00000178053	MLF1	3:158288951-158325041	q1	q2	OK	17.9437	17.3864	-0.0455176	-0.0804352	0.90445	0.99995	no

ENSG00000178055	ENSG00000178055	PRSS42	3:46871031-46875585	q1	q2	NOTEST	0	0.0213912	inf	0	1	1	no

ENSG00000178057	ENSG00000178057	NDUFAF3	3:48955220-49060928	q1	q2	OK	24.3357	23.2055	-0.0686081	-0.0301718	0.95595	0.99995	no

ENSG00000178074	ENSG00000178074	C2orf69	2:200775978-200873263	q1	q2	OK	7.30798	8.98963	0.298788	0.448302	0.5128	0.99995	no

ENSG00000178075	ENSG00000178075	GRAMD1C	3:113547028-113666417	q1	q2	OK	2.67291	2.97914	0.156483	0.137455	0.7718	0.99995	no

ENSG00000178078	ENSG00000178078	STAP2	19:4324039-4343488	q1	q2	OK	2.40484	3.11913	0.375201	0.39829	0.5202	0.99995	no

ENSG00000178081	ENSG00000178081	ULK4P3	15:30395922-30423960	q1	q2	OK	0.533842	0.520412	-0.0367602	-0.0230092	0.96655	0.99995	no

ENSG00000178082	ENSG00000178082	TWF1P1	17:27530440-27531492	q1	q2	OK	3.0051	3.34786	0.155825	0.18233	0.7934	0.99995	no

ENSG00000178084	ENSG00000178084	HTR3C	3:183770834-183778459	q1	q2	NOTEST	0	0	0	0	1	1	no

ENSG00000178093	ENSG00000178093	TSSK6	19:19623226-19648390	q1	q2	OK	1.16743	0.861	-0.439258	-0.15175	0.8558	0.99995	no

ENSG00000178096	ENSG00000178096	BOLA1	1:149859018-149872351	q1	q2	NOTEST	0	0	0	0	1	1	no

ENSG00000178104	ENSG00000178104	PDE4DIP	1:144836156-145076186	q1	q2	OK	0.556029	0.577404	0.0544212	0.0228215	0.8753	0.99995	no

ENSG00000178105	ENSG00000178105	DDX10	11:108535751-108811657	q1	q2	OK	13.7715	14.8863	0.112302	0.17039	0.7935	0.99995	no

ENSG00000178107	ENSG00000178107	RP11-998D10.4	14:21576201-21584862	q1	q2	NOTEST	0	0	0	0	1	1	no

ENSG00000178115	ENSG00000178115	GOLGA8Q	15:30844182-30854704	q1	q2	NOTEST	0.0713619	0.0135569	-2.39613	0	1	1	no

ENSG00000178125	ENSG00000178125	PPP1R42	8:67876333-68255912	q1	q2	OK	0.117633	0.76701	2.70495	0.142465	0.0479	0.99995	no

ENSG00000178127	ENSG00000178127	NDUFV2	18:9102627-9285983	q1	q2	OK	99.8521	106.987	0.099571	0.157538	0.81845	0.99995	no

ENSG00000178130	ENSG00000178130	FAM27L	17:21825296-21826499	q1	q2	NOTEST	0	0	0	0	1	1	no

ENSG00000178146	ENSG00000178146	RP1-232L22__B.1	X:101033941-101035511	q1	q2	OK	0.778322	0.828167	0.0895544	0.0905491	0.89725	0.99995	no

ENSG00000178149	ENSG00000178149	DALRD3	3:48955220-49060928	q1	q2	OK	19.6996	16.8019	-0.229548	-0.107906	0.7367	0.99995	no

ENSG00000178150	ENSG00000178150	ZNF114	19:48673948-48790865	q1	q2	OK	6.22471	6.21296	-0.00272809	-0.00161737	0.9975	0.99995	no

ENSG00000178162	ENSG00000178162	FAR2P2	2:131174327-131199048	q1	q2	NOTEST	0.293205	0.471403	0.685049	0	1	1	no

ENSG00000178163	ENSG00000178163	ZNF518B	4:10441497-10459034	q1	q2	OK	10.7741	13.9355	0.371199	0.619854	0.23685	0.99995	no

ENSG00000178171	ENSG00000178171	AMER3	2:131501110-131525707	q1	q2	NOTEST	0.363227	0.332375	-0.128059	0	1	1	no

ENSG00000178172	ENSG00000178172	SPINK6	5:147582356-147594700	q1	q2	NOTEST	0	0	0	0	1	1	no

ENSG00000178175	ENSG00000178175	ZNF366	5:71738478-71803554	q1	q2	NOTEST	0.0199639	0.0131277	-0.604783	0	1	1	no

ENSG00000178177	ENSG00000178177	LCORL	4:17812524-18023499	q1	q2	OK	14.0817	16.3913	0.219106	0.180448	0.70935	0.99995	no

ENSG00000178184	ENSG00000178184	PARD6G	18:77905806-78005429	q1	q2	OK	15.8306	14.5644	-0.120273	-0.205267	0.7099	0.99995	no

ENSG00000178187	ENSG00000178187	ZNF454	5:178368191-178393434	q1	q2	NOTEST	0.112641	0.0634999	-0.826909	0	1	1	no

ENSG00000178188	ENSG00000178188	SH2B1	16:28857920-28885526	q1	q2	OK	26.0771	19.8034	-0.397033	-0.542445	0.2512	0.99995	no

ENSG00000178193	ENSG00000178193	RP4-682C21.5	1:76175687-76189696	q1	q2	NOTEST	0	0	0	0	1	1	no

ENSG00000178199	ENSG00000178199	ZC3H12D	6:149768793-149806197	q1	q2	NOTEST	0.357991	0.278134	-0.364145	0	1	1	no

ENSG00000178201	ENSG00000178201	VN1R1	19:57874844-57988938	q1	q2	OK	1.13994	1.1015	-0.0494865	-0.0149155	0.9555	0.99995	no

ENSG00000178202	ENSG00000178202	KDELC2	11:108342831-108369159	q1	q2	OK	17.7157	18.9474	0.096971	0.205793	0.74485	0.99995	no

ENSG00000178206	ENSG00000178206	CYP4F31P	2:131862419-132111282	q1	q2	NOTEST	0.0597974	0.0240762	-1.31247	0	1	1	no

ENSG00000178209	ENSG00000178209	PLEC	8:144989320-145050902	q1	q2	OK	3.27113	2.00051	-0.709423	-0.431568	0.52175	0.99995	no

ENSG00000178217	ENSG00000178217	SH2D4B	10:82297657-82406316	q1	q2	NOTEST	0.187813	0.234411	0.319743	0	1	1	no

ENSG00000178222	ENSG00000178222	RNF212	4:1050037-1107350	q1	q2	NOTEST	0.0304808	0	#NAME?	0	1	1	no

ENSG00000178226	ENSG00000178226	PRSS36	16:31150245-31161415	q1	q2	NOTEST	0.0386844	0.373692	3.27203	0	1	1	no

ENSG00000178229	ENSG00000178229	ZNF543	19:57831876-57842144	q1	q2	OK	2.6111	2.71713	0.0574278	0.0888449	0.9003	0.99995	no

ENSG00000178233	ENSG00000178233	TMEM151B	6:44238202-44346694	q1	q2	OK	19.2679	19.675	0.0301635	0.0602951	0.92775	0.99995	no

ENSG00000178234	ENSG00000178234	GALNT11	7:151722758-151819425	q1	q2	OK	18.2887	18.5388	0.0195895	0.0366345	0.9519	0.99995	no

ENSG00000178235	ENSG00000178235	SLITRK1	13:84451343-84456528	q1	q2	OK	1.83246	2.25447	0.299009	0.476805	0.49345	0.99995	no

ENSG00000178243	ENSG00000178243	C9orf62	9:138235094-138238408	q1	q2	NOTEST	0	0	0	0	1	1	no

ENSG00000178248	ENSG00000178248	AP000345.1	22:23804272-23829167	q1	q2	NOTEST	0.0181669	0.0178853	-0.0225399	0	1	1	no

ENSG00000178252	ENSG00000178252	WDR6	3:48955220-49060928	q1	q2	OK	114.713	108.337	-0.0825068	-0.0863962	0.84885	0.99995	no

ENSG00000178257	ENSG00000178257	PRM3	16:11343475-11445619	q1	q2	NOTEST	0	0	0	0	1	1	no

ENSG00000178279	ENSG00000178279	TNP2	16:11343475-11445619	q1	q2	NOTEST	0	0	0	0	1	1	no

ENSG00000178287	ENSG00000178287	SPAG11A	8:7705397-7726389	q1	q2	NOTEST	0	0	0	0	1	1	no

ENSG00000178295	ENSG00000178295	GEN1	2:17845078-17981509	q1	q2	OK	14.701	15.873	0.11066	0.114128	0.78835	0.99995	no

ENSG00000178297	ENSG00000178297	TMPRSS9	19:2389768-2456994	q1	q2	NOTEST	0.196121	0.0238227	-3.04134	0	1	1	no

ENSG00000178301	ENSG00000178301	AQP11	11:77225980-77348850	q1	q2	OK	1.28062	1.24073	-0.0456587	-0.016538	0.935	0.99995	no

ENSG00000178307	ENSG00000178307	TMEM11	17:21100592-21133348	q1	q2	OK	15.4208	14.9223	-0.0474004	-0.0839808	0.90275	0.99995	no

ENSG00000178338	ENSG00000178338	ZNF354B	5:178286953-178315123	q1	q2	OK	8.86438	9.25178	0.0617116	0.0915045	0.89175	0.99995	no

ENSG00000178342	ENSG00000178342	KCNG2	18:77623667-77660184	q1	q2	NOTEST	0.144997	0.233354	0.686492	0	1	1	no

ENSG00000178343	ENSG00000178343	SHISA3	4:42399855-42404504	q1	q2	OK	11.6379	6.05486	-0.942669	-1.49486	0.0316	0.99995	no

ENSG00000178358	ENSG00000178358	OR2D3	11:6942204-6943264	q1	q2	NOTEST	0	0.0269202	inf	0	1	1	no

ENSG00000178363	ENSG00000178363	CALML3	10:5556206-5568225	q1	q2	NOTEST	0	0	0	0	1	1	no

ENSG00000178372	ENSG00000178372	CALML5	10:5540657-5541533	q1	q2	NOTEST	0	0	0	0	1	1	no

ENSG00000178381	ENSG00000178381	ZFAND2A	7:1191706-1204903	q1	q2	OK	8.58357	7.04732	-0.284504	-0.340161	0.63925	0.99995	no

ENSG00000178385	ENSG00000178385	PLEKHM3	2:208693026-208890284	q1	q2	OK	1.22644	1.50358	0.293926	0.359867	0.6059	0.99995	no

ENSG00000178386	ENSG00000178386	ZNF223	19:44529493-44593766	q1	q2	OK	5.11616	4.90743	-0.0600937	-0.0601806	0.91685	0.99995	no

ENSG00000178394	ENSG00000178394	HTR1A	5:63253719-63276870	q1	q2	NOTEST	0	0	0	0	1	1	no

ENSG00000178395	ENSG00000178395	C1orf65	1:223566714-223568812	q1	q2	NOTEST	0	0	0	0	1	1	no

ENSG00000178397	ENSG00000178397	FAM220A	7:6369039-6388612	q1	q2	OK	12.8854	11.8499	-0.12086	-0.219608	0.73275	0.99995	no

ENSG00000178401	ENSG00000178401	DNAJC22	12:49740699-49751309	q1	q2	OK	0.602409	0.464006	-0.376599	-0.235167	0.6364	0.99995	no

ENSG00000178403	ENSG00000178403	NEUROG2	4:113434671-113578748	q1	q2	OK	0.640608	0.402568	-0.670211	-0.145871	0.77945	0.99995	no

ENSG00000178404	ENSG00000178404	DDC8	17:76849058-76921469	q1	q2	NOTEST	0.143588	0.323776	1.17306	0	1	1	no

ENSG00000178409	ENSG00000178409	BEND3	6:107386385-107436473	q1	q2	NOTEST	0	0	0	0	1	1	no

ENSG00000178412	ENSG00000178412	RP11-567M16.3	18:77398927-77439745	q1	q2	NOTEST	0.02605	0	#NAME?	0	1	1	no

ENSG00000178425	ENSG00000178425	NT5DC1	6:116422011-116570660	q1	q2	OK	10.0918	11.8159	0.227535	0.22432	0.75945	0.99995	no

ENSG00000178429	ENSG00000178429	RPS3AP5	10:86320198-86321019	q1	q2	OK	9.96225	12.6288	0.342169	0.493524	0.4802	0.99995	no

ENSG00000178430	ENSG00000178430	AC006111.1	16:3661728-3767598	q1	q2	OK	0.54104	0.470033	-0.202974	-0.0421513	0.91785	0.99995	no

ENSG00000178440	ENSG00000178440	LINC00843	10:51623416-51741813	q1	q2	NOTEST	0	0	0	0	1	1	no

ENSG00000178445	ENSG00000178445	GLDC	9:6532463-6645650	q1	q2	OK	25.2196	22.1355	-0.188182	-0.331444	0.52335	0.99995	no

ENSG00000178449	ENSG00000178449	COX14	12:50505761-50561288	q1	q2	OK	14.8596	14.5083	-0.0345229	-0.0259196	0.9724	0.99995	no

ENSG00000178457	ENSG00000178457	LINC00314	21:29385681-29395552	q1	q2	NOTEST	0	0	0	0	1	1	no

ENSG00000178458	ENSG00000178458	H3F3AP6	4:140586921-141075338	q1	q2	OK	9.9617	19.5963	0.976121	0.527646	0.55245	0.99995	no

ENSG00000178460	ENSG00000178460	MCMDC2	8:67782983-67838633	q1	q2	NOTEST	0.321688	0.380303	0.24149	0	1	1	no

ENSG00000178462	ENSG00000178462	TUBAL3	10:5435060-5446793	q1	q2	NOTEST	0	0	0	0	1	1	no

ENSG00000178464	ENSG00000178464	CTD-2192J16.15	19:12754088-12792716	q1	q2	OK	98.9353	101.487	0.0367438	0.0415763	0.9533	0.99995	no

ENSG00000178467	ENSG00000178467	P4HTM	3:48955220-49060928	q1	q2	OK	5.59194	4.91068	-0.187426	-0.0600263	0.8427	0.99995	no

ENSG00000178473	ENSG00000178473	UCN3	10:5406971-5416169	q1	q2	NOTEST	0	0	0	0	1	1	no

ENSG00000178498	ENSG00000178498	DTX3	12:57998404-58003587	q1	q2	OK	44.3981	40.5473	-0.130892	-0.260782	0.70675	0.99995	no

ENSG00000178502	ENSG00000178502	KLHL11	17:40009796-40021684	q1	q2	NOTEST	0.0223316	0	#NAME?	0	1	1	no

ENSG00000178503	ENSG00000178503	NECAP1P1	7:37625102-37625932	q1	q2	NOTEST	0	0	0	0	1	1	no

ENSG00000178522	ENSG00000178522	AMBN	4:71457972-71473005	q1	q2	NOTEST	0	0	0	0	1	1	no

ENSG00000178531	ENSG00000178531	CTXN1	19:7989371-8008805	q1	q2	OK	71.2405	59.8239	-0.251977	-0.493964	0.48875	0.99995	no

ENSG00000178537	ENSG00000178537	SLC25A20	3:48894368-48936426	q1	q2	OK	7.93998	6.50524	-0.287533	-0.355783	0.6122	0.99995	no

ENSG00000178538	ENSG00000178538	CA8	8:61099905-61193971	q1	q2	OK	1.51428	1.21909	-0.312828	-0.331259	0.6194	0.99995	no

ENSG00000178550	ENSG00000178550	AC010170.1	3:45265957-45267770	q1	q2	NOTEST	0	0	0	0	1	1	no

ENSG00000178556	ENSG00000178556	CKS1BP6	X:30635570-30635810	q1	q2	OK	0	2.8231	inf	#NAME?	0.0082	0.99995	no

ENSG00000178562	ENSG00000178562	CD28	2:204571197-204603635	q1	q2	NOTEST	0.0141453	0	#NAME?	0	1	1	no

ENSG00000178567	ENSG00000178567	EPM2AIP1	3:37027356-37034795	q1	q2	OK	18.954	19.6421	0.0514491	0.12637	0.85605	0.99995	no

ENSG00000178568	ENSG00000178568	ERBB4	2:212240445-213403565	q1	q2	OK	1.16131	0.824538	-0.494094	-0.442835	0.3212	0.99995	no

ENSG00000178573	ENSG00000178573	MAF	16:79619739-79634611	q1	q2	OK	7.24363	6.75092	-0.101629	-0.199403	0.76805	0.99995	no

ENSG00000178585	ENSG00000178585	CTNNBIP1	1:9908333-9970394	q1	q2	OK	27.9293	27.1934	-0.0385197	-0.0760742	0.90715	0.99995	no

ENSG00000178586	ENSG00000178586	OR6B3	2:240984493-240985489	q1	q2	NOTEST	0	0	0	0	1	1	no

ENSG00000178591	ENSG00000178591	DEFB125	20:67890-77735	q1	q2	NOTEST	0	0	0	0	1	1	no

ENSG00000178596	ENSG00000178596	GAPDHP29	1:94767593-94768578	q1	q2	NOTEST	0	0	0	0	1	1	no

ENSG00000178597	ENSG00000178597	PSAPL1	4:7194264-7744554	q1	q2	NOTEST	0	0	0	0	1	1	no

ENSG00000178602	ENSG00000178602	OTOS	2:241078445-241083979	q1	q2	NOTEST	0.0648273	0	#NAME?	0	1	1	no

ENSG00000178605	ENSG00000178605	GTPBP6	X:220024-230886	q1	q2	OK	28.1776	24.3693	-0.209487	-0.428873	0.5489	0.99995	no

ENSG00000178607	ENSG00000178607	ERN1	17:62116501-62208179	q1	q2	OK	1.88445	1.92284	0.0290944	0.033312	0.9499	0.99995	no

ENSG00000178623	ENSG00000178623	GPR35	2:241526132-241570676	q1	q2	OK	0.711805	0.430599	-0.725138	-0.264693	0.62235	0.99995	no

ENSG00000178631	ENSG00000178631	ACTG1P1	3:139108656-139396859	q1	q2	OK	0.600338	0.465783	-0.366117	-0.0825132	0.8682	0.99995	no

ENSG00000178636	ENSG00000178636	RP11-455G16.1	4:120113927-120216672	q1	q2	NOTEST	0.0956915	0.0130009	-2.87978	0	1	1	no

ENSG00000178642	ENSG00000178642	AL513477.1	1:2252691-2323146	q1	q2	NOTEST	0.235765	0.231397	-0.0269799	0	1	1	no

ENSG00000178645	ENSG00000178645	C10orf53	10:50817140-50918307	q1	q2	NOTEST	0.0541614	0.0353237	-0.616629	0	1	1	no

ENSG00000178654	ENSG00000178654	RP11-366I21.1	9:7597822-7598377	q1	q2	NOTEST	0.0707758	0	#NAME?	0	1	1	no

ENSG00000178660	ENSG00000178660	ARMC10P1	3:94225609-94226464	q1	q2	OK	0.387999	0.677839	0.804891	0.62614	0.3744	0.99995	no

ENSG00000178662	ENSG00000178662	CSRNP3	2:166326156-166545917	q1	q2	OK	5.64942	6.1252	0.116654	0.201559	0.701	0.99995	no

ENSG00000178665	ENSG00000178665	ZNF713	7:55954969-56067874	q1	q2	OK	7.01986	7.04442	0.00503838	0.00292751	0.99535	0.99995	no

ENSG00000178685	ENSG00000178685	PARP10	8:145051320-145101933	q1	q2	NOTEST	0.211433	0.0789135	-1.42186	0	1	1	no

ENSG00000178690	ENSG00000178690	DYNAP	18:52254987-52266724	q1	q2	NOTEST	0	0.0134981	inf	0	1	1	no

ENSG00000178691	ENSG00000178691	SUZ12	17:30264036-30328064	q1	q2	OK	22.58	23.9663	0.0859566	0.204379	0.7576	0.99995	no

ENSG00000178694	ENSG00000178694	NSUN3	3:93698982-93847389	q1	q2	OK	5.96046	5.95604	-0.00107111	-0.000792077	0.998	0.99995	no

ENSG00000178695	ENSG00000178695	KCTD12	13:77454311-77460540	q1	q2	OK	28.1775	27.7599	-0.0215415	-0.0442637	0.9485	0.99995	no

ENSG00000178700	ENSG00000178700	DHFRL1	3:93698982-93847389	q1	q2	OK	3.21161	3.00691	-0.0950112	-0.0923399	0.89225	0.99995	no

ENSG00000178715	ENSG00000178715	RP11-169K16.8	1:16154723-16155167	q1	q2	OK	2.35054	3.13899	0.417305	0.400086	0.5658	0.99995	no

ENSG00000178718	ENSG00000178718	RPP25	15:75246756-75313837	q1	q2	OK	2.29121	1.54851	-0.565225	-0.289501	0.7248	0.99995	no

ENSG00000178719	ENSG00000178719	GRINA	8:145051320-145101933	q1	q2	OK	3.42477	2.34627	-0.545636	-0.549289	0.3918	0.99995	no

ENSG00000178722	ENSG00000178722	C5orf64	5:60933534-61047590	q1	q2	OK	2.49708	1.88452	-0.406041	-0.326484	0.5375	0.99995	no

ENSG00000178723	ENSG00000178723	GLULP4	9:34917171-34918293	q1	q2	NOTEST	0.24168	0.339045	0.488379	0	1	1	no

ENSG00000178726	ENSG00000178726	THBD	20:23026269-23030378	q1	q2	NOTEST	0.0242813	0.0591897	1.2855	0	1	1	no

ENSG00000178732	ENSG00000178732	GP5	3:194115549-194119995	q1	q2	NOTEST	0.00720032	0.0139479	0.953914	0	1	1	no

ENSG00000178734	ENSG00000178734	C13orf45	13:76445186-76457947	q1	q2	NOTEST	0.103651	0.0258056	-2.00597	0	1	1	no

ENSG00000178741	ENSG00000178741	COX5A	15:75212131-75230509	q1	q2	OK	162.682	153.699	-0.0819422	-0.182059	0.7963	0.99995	no

ENSG00000178750	ENSG00000178750	STX19	3:93698982-93847389	q1	q2	NOTEST	0.103235	0.0256982	-2.0062	0	1	1	no

ENSG00000178752	ENSG00000178752	FAM132B	2:239067622-239077541	q1	q2	OK	3.28066	2.67492	-0.29449	-0.312785	0.6404	0.99995	no

ENSG00000178761	ENSG00000178761	FAM219B	15:75192327-75199462	q1	q2	OK	43.4524	43.0813	-0.0123741	-0.0245741	0.9631	0.99995	no

ENSG00000178762	ENSG00000178762	HIST1H2BPS1	6:25732009-25732390	q1	q2	NOTEST	0	0	0	0	1	1	no

ENSG00000178764	ENSG00000178764	ZHX2	8:123793632-123986750	q1	q2	OK	9.40333	9.16153	-0.037583	-0.0734615	0.90485	0.99995	no

ENSG00000178772	ENSG00000178772	CPN2	3:194060493-194072057	q1	q2	NOTEST	0	0	0	0	1	1	no

ENSG00000178773	ENSG00000178773	CPNE7	16:89642175-89663654	q1	q2	NOTEST	0.378503	0.31762	-0.253	0	1	1	no

ENSG00000178776	ENSG00000178776	C5orf46	5:147250244-147286101	q1	q2	NOTEST	0	0	0	0	1	1	no

ENSG00000178789	ENSG00000178789	CD300LB	17:72517312-72527613	q1	q2	NOTEST	0.0113756	0.0118249	0.0558858	0	1	1	no

ENSG00000178795	ENSG00000178795	GDPD4	11:76927602-77012732	q1	q2	NOTEST	0.0658896	0	#NAME?	0	1	1	no

ENSG00000178796	ENSG00000178796	RIIAD1	1:151673501-151702281	q1	q2	OK	1.33501	1.64346	0.299893	0.0680884	0.80125	0.99995	no

ENSG00000178802	ENSG00000178802	MPI	15:75182345-75191798	q1	q2	OK	17.5734	16.3827	-0.101218	-0.171894	0.7898	0.99995	no

ENSG00000178803	ENSG00000178803	ADORA2A-AS1	22:24666785-24924358	q1	q2	OK	0.646925	0.386556	-0.742921	-0.143089	0.6583	0.99995	no

ENSG00000178804	ENSG00000178804	H1FOO	3:129262056-129270310	q1	q2	NOTEST	0	0	0	0	1	1	no

ENSG00000178809	ENSG00000178809	TRIM73	7:74988447-75115548	q1	q2	NOTEST	0.17219	0.0508151	-1.76068	0	1	1	no

ENSG00000178814	ENSG00000178814	OPLAH	8:145104029-145118735	q1	q2	NOTEST	0	0	0	0	1	1	no

ENSG00000178821	ENSG00000178821	TMEM52	1:1849028-1850712	q1	q2	OK	0.821806	0.901039	0.132792	0.110336	0.85485	0.99995	no

ENSG00000178826	ENSG00000178826	TMEM139	7:142941185-142985141	q1	q2	OK	0.4149	0.663875	0.67815	0.182803	0.57635	0.99995	no

ENSG00000178828	ENSG00000178828	RNF186	1:20140522-20145995	q1	q2	NOTEST	0.0226226	0	#NAME?	0	1	1	no

ENSG00000178836	ENSG00000178836	AC114812.5	2:234526290-234681956	q1	q2	NOTEST	0	0	0	0	1	1	no

ENSG00000178852	ENSG00000178852	EFCAB13	17:45331211-45518678	q1	q2	OK	1.56539	1.50597	-0.0558262	-0.0285104	0.93095	0.99995	no

ENSG00000178860	ENSG00000178860	MSC	8:72740401-73030628	q1	q2	OK	4.86514	4.20309	-0.211029	-0.158667	0.78615	0.99995	no

ENSG00000178863	ENSG00000178863	CEBPA-AS1	19:33793762-33800293	q1	q2	NOTEST	0.0379845	0.206619	2.44349	0	1	1	no

ENSG00000178878	ENSG00000178878	APOLD1	12:12878850-12982915	q1	q2	OK	3.21484	3.48164	0.115021	0.0601063	0.88065	0.99995	no

ENSG00000178882	ENSG00000178882	FAM101A	12:124247041-124800570	q1	q2	OK	2.79214	2.51298	-0.151974	-0.0217497	0.89475	0.99995	no

ENSG00000178894	ENSG00000178894	AC073416.1	2:90137997-90138233	q1	q2	NOTEST	0	0	0	0	1	1	no

ENSG00000178896	ENSG00000178896	EXOSC4	8:145132904-145135550	q1	q2	NOTEST	0	0	0	0	1	1	no

ENSG00000178904	ENSG00000178904	DPY19L3	19:32880955-32976801	q1	q2	OK	31.8254	33.8589	0.0893537	0.161999	0.74815	0.99995	no

ENSG00000178913	ENSG00000178913	TAF7	5:140698056-140700339	q1	q2	OK	73.6568	87.8795	0.254709	0.62859	0.37145	0.99995	no

ENSG00000178917	ENSG00000178917	ZNF852	3:44540461-44552128	q1	q2	OK	2.68209	3.22554	0.266185	0.330929	0.6354	0.99995	no

ENSG00000178919	ENSG00000178919	FOXE1	9:100615535-100618986	q1	q2	NOTEST	0.0757539	0.0217265	-1.80186	0	1	1	no

ENSG00000178921	ENSG00000178921	PFAS	17:8130190-8173809	q1	q2	OK	26.4641	26.2233	-0.0131877	-0.0156902	0.9725	0.99995	no

ENSG00000178922	ENSG00000178922	HYI	1:43855552-43922666	q1	q2	OK	10.1906	10.455	0.0369483	0.0331666	0.94505	0.99995	no

ENSG00000178927	ENSG00000178927	C17orf62	17:80347098-80408705	q1	q2	OK	42.8829	41.8013	-0.0368561	-0.0452738	0.94115	0.99995	no

ENSG00000178928	ENSG00000178928	TPRX1	19:48304499-48322308	q1	q2	NOTEST	0	0.0143011	inf	0	1	1	no

ENSG00000178934	ENSG00000178934	LGALS7B	19:39279850-39282389	q1	q2	NOTEST	0	0	0	0	1	1	no

ENSG00000178935	ENSG00000178935	ZNF552	19:58281022-58427978	q1	q2	OK	4.32629	3.88398	-0.155591	-0.0684834	0.91845	0.99995	no

ENSG00000178947	ENSG00000178947	LINC00086	X:134555867-134561999	q1	q2	NOTEST	0.0141523	0.0185987	0.394166	0	1	1	no

ENSG00000178950	ENSG00000178950	GAK	4:843063-926161	q1	q2	OK	27.8948	22.9496	-0.281528	-0.390571	0.407	0.99995	no

ENSG00000178951	ENSG00000178951	ZBTB7A	19:4044361-4066943	q1	q2	OK	4.12197	3.49985	-0.236042	-0.428493	0.5353	0.99995	no

ENSG00000178952	ENSG00000178952	TUFM	16:28853731-28857729	q1	q2	OK	81.021	76.1069	-0.0902684	-0.16466	0.77565	0.99995	no

ENSG00000178965	ENSG00000178965	C1orf173	1:75033794-75139422	q1	q2	NOTEST	0.00973276	0	#NAME?	0	1	1	no

ENSG00000178966	ENSG00000178966	RMI1	9:86595625-86618985	q1	q2	OK	24.59	26.0804	0.0848919	0.183456	0.79105	0.99995	no

ENSG00000178971	ENSG00000178971	CTC1	17:8130190-8173809	q1	q2	OK	11.546	10.8502	-0.0896674	-0.0574679	0.8744	0.99995	no

ENSG00000178972	ENSG00000178972	AC097658.1	4:144256417-144395721	q1	q2	OK	0.485627	0.510336	0.071599	0.0224467	0.94145	0.99995	no

ENSG00000178974	ENSG00000178974	FBXO34	14:55729484-55828636	q1	q2	OK	10.6709	10.1789	-0.0680905	-0.143722	0.837	0.99995	no

ENSG00000178977	ENSG00000178977	LINC00324	17:8123959-8127361	q1	q2	OK	0.593759	0.838756	0.498372	0.510507	0.48355	0.99995	no

ENSG00000178980	ENSG00000178980	SEPW1	19:48281828-48287943	q1	q2	OK	100.528	93.7083	-0.101353	-0.223218	0.7448	0.99995	no

ENSG00000178982	ENSG00000178982	EIF3K	19:39109734-39127595	q1	q2	OK	119.417	106.002	-0.171916	-0.381747	0.57745	0.99995	no

ENSG00000178988	ENSG00000178988	MRFAP1L1	4:6709427-6711607	q1	q2	OK	72.9895	73.2762	0.00565419	0.013777	0.9828	0.99995	no

ENSG00000178996	ENSG00000178996	SNX18	5:53813588-53842415	q1	q2	OK	5.11479	5.57577	0.124496	0.251921	0.71555	0.99995	no

ENSG00000178997	ENSG00000178997	EXD1	15:41474922-41522941	q1	q2	NOTEST	0.0113372	0.0179991	0.66686	0	1	1	no

ENSG00000178999	ENSG00000178999	AURKB	17:8108055-8113918	q1	q2	OK	69.6499	67.6224	-0.0426194	-0.0873	0.88875	0.99995	no

ENSG00000179002	ENSG00000179002	TAS1R2	1:19166092-19283180	q1	q2	NOTEST	0	0	0	0	1	1	no

ENSG00000179008	ENSG00000179008	C14orf39	14:60863186-61021634	q1	q2	NOTEST	0.306094	0.323364	0.0791833	0	1	1	no

ENSG00000179010	ENSG00000179010	MRFAP1	4:6641817-6644472	q1	q2	OK	286.56	299.472	0.0635827	0.151384	0.82945	0.99995	no

ENSG00000179021	ENSG00000179021	C3orf38	3:88101093-88217879	q1	q2	OK	20.1659	22.5147	0.158949	0.129709	0.83555	0.99995	no

ENSG00000179023	ENSG00000179023	KLHDC7A	1:18807423-18812478	q1	q2	NOTEST	0	0	0	0	1	1	no

ENSG00000179028	ENSG00000179028	RP11-363G10.2	X:51942962-51946358	q1	q2	NOTEST	0	0	0	0	1	1	no

ENSG00000179029	ENSG00000179029	TMEM107	17:8076554-8079717	q1	q2	OK	9.65166	9.95763	0.0450252	0.0647876	0.92535	0.99995	no

ENSG00000179031	ENSG00000179031	LL0XNC01-131B10.2	X:100165700-100166478	q1	q2	NOTEST	0	0.0424928	inf	0	1	1	no

ENSG00000179038	ENSG00000179038	AP001885.1	11:66886739-67025558	q1	q2	NOTEST	0.119492	0.346684	1.5367	0	1	1	no

ENSG00000179041	ENSG00000179041	RRS1	8:67341262-67430759	q1	q2	OK	16.3147	17.1923	0.0755902	0.09878	0.88925	0.99995	no

ENSG00000179044	ENSG00000179044	EXOC3L1	16:67218268-67224107	q1	q2	OK	0.524546	0.241144	-1.12118	-0.699566	0.17965	0.99995	no

ENSG00000179046	ENSG00000179046	TRIML2	4:189012426-189030757	q1	q2	NOTEST	0.0294433	0	#NAME?	0	1	1	no

ENSG00000179051	ENSG00000179051	RCC2	1:17733255-17766220	q1	q2	OK	71.7154	74.9154	0.062978	0.150271	0.82595	0.99995	no

ENSG00000179055	ENSG00000179055	OR13D1	9:107456659-107457766	q1	q2	NOTEST	0.027622	0	#NAME?	0	1	1	no

ENSG00000179057	ENSG00000179057	IGSF22	11:18714668-18814268	q1	q2	NOTEST	0.162642	0.165936	0.0289344	0	1	1	no

ENSG00000179058	ENSG00000179058	C9orf50	9:132371162-132398209	q1	q2	NOTEST	0.101186	0.0520726	-0.958415	0	1	1	no

ENSG00000179059	ENSG00000179059	ZFP42	4:188916924-188926204	q1	q2	NOTEST	0	0	0	0	1	1	no

ENSG00000179061	ENSG00000179061	AC012074.1	2:25592041-25598714	q1	q2	OK	0.425385	0.561886	0.401509	0.191883	0.7949	0.99995	no

ENSG00000179066	ENSG00000179066	AC020907.1	19:35531409-35598141	q1	q2	NOTEST	0	0	0	0	1	1	no

ENSG00000179071	ENSG00000179071	CCDC89	11:85394892-85397320	q1	q2	NOTEST	0.0437607	0.0523733	0.259197	0	1	1	no

ENSG00000179073	ENSG00000179073	TAAR3	6:132929363-132930441	q1	q2	OK	1.77305	2.00642	0.178391	0.165016	0.81595	0.99995	no

ENSG00000179082	ENSG00000179082	C9orf106	9:132083294-132087184	q1	q2	NOTEST	0	0.00780382	inf	0	1	1	no

ENSG00000179083	ENSG00000179083	FAM133A	X:92929011-92967273	q1	q2	NOTEST	0.255804	0.259206	0.0190613	0	1	1	no

ENSG00000179085	ENSG00000179085	DPM3	1:155112366-155113071	q1	q2	OK	46.5903	36.1498	-0.366043	-0.570956	0.42935	0.99995	no

ENSG00000179088	ENSG00000179088	C12orf42	12:103631368-103889749	q1	q2	NOTEST	0.0220421	0.0420088	0.930431	0	1	1	no

ENSG00000179091	ENSG00000179091	CYC1	8:145149929-145152428	q1	q2	OK	60.213	54.8349	-0.134981	-0.312636	0.6473	0.99995	no

ENSG00000179094	ENSG00000179094	PER1	17:8043789-8066864	q1	q2	OK	10.6776	9.3527	-0.191135	-0.133225	0.6961	0.99995	no

ENSG00000179097	ENSG00000179097	HTR1F	3:88039845-88042919	q1	q2	NOTEST	0.0169603	0.00858665	-0.981995	0	1	1	no

ENSG00000179101	ENSG00000179101	RP11-349N19.2	X:122648323-122648723	q1	q2	OK	11.5803	12.2321	0.0789985	0.0921023	0.89725	0.99995	no

ENSG00000179104	ENSG00000179104	TMTC2	12:83080658-83528649	q1	q2	OK	25.438	22.1236	-0.2014	-0.44899	0.51395	0.99995	no

ENSG00000179111	ENSG00000179111	HES7	17:8023907-8027410	q1	q2	NOTEST	0.19306	0.145278	-0.410229	0	1	1	no

ENSG00000179115	ENSG00000179115	FARSA	19:13033292-13044851	q1	q2	OK	31.3596	26.5354	-0.24099	-0.5337	0.4335	0.99995	no

ENSG00000179119	ENSG00000179119	SPTY2D1	11:18621333-18656338	q1	q2	OK	7.09723	6.62154	-0.100091	-0.167082	0.75555	0.99995	no

ENSG00000179131	ENSG00000179131	RP11-458F8.3	7:66379567-66379930	q1	q2	NOTEST	0.303628	0	#NAME?	0	1	1	no

ENSG00000179133	ENSG00000179133	C10orf67	10:23556123-23634110	q1	q2	NOTEST	0.263985	0.394804	0.580681	0	1	1	no

ENSG00000179134	ENSG00000179134	SAMD4B	19:39833035-39876350	q1	q2	OK	60.0801	53.4269	-0.169319	-0.239865	0.7133	0.99995	no

ENSG00000179136	ENSG00000179136	LINC00670	17:12453098-12546171	q1	q2	NOTEST	0	0	0	0	1	1	no

ENSG00000179141	ENSG00000179141	MTUS2-AS1	13:29598747-30077892	q1	q2	NOTEST	0.0644124	0.0816568	0.342235	0	1	1	no

ENSG00000179142	ENSG00000179142	CYP11B2	8:143915553-143999259	q1	q2	NOTEST	0	0	0	0	1	1	no

ENSG00000179144	ENSG00000179144	GIMAP7	7:150211917-150218161	q1	q2	NOTEST	0	0	0	0	1	1	no

ENSG00000179148	ENSG00000179148	ALOXE3	17:7999217-8022365	q1	q2	NOTEST	0.0541385	0	#NAME?	0	1	1	no

ENSG00000179151	ENSG00000179151	EDC3	15:74890840-74988633	q1	q2	OK	25.6582	26.4013	0.0411883	0.0515133	0.91675	0.99995	no

ENSG00000179152	ENSG00000179152	TCAIM	3:44379432-44450943	q1	q2	OK	12.4144	13.5923	0.130778	0.24821	0.71005	0.99995	no

ENSG00000179157	ENSG00000179157	RPS2P28	6:43263431-43337216	q1	q2	NOTEST	0.0438708	0.0453813	0.0488373	0	1	1	no

ENSG00000179163	ENSG00000179163	FUCA1	1:24171566-24194784	q1	q2	OK	5.47528	5.05596	-0.114949	-0.181382	0.7963	0.99995	no

ENSG00000179165	ENSG00000179165	PXT1	6:36322418-36458920	q1	q2	NOTEST	0.152636	0.173217	0.182481	0	1	1	no

ENSG00000179168	ENSG00000179168	GGN	19:38874904-38878722	q1	q2	OK	0.936033	1.16745	0.318727	0.243826	0.66585	0.99995	no

ENSG00000179170	ENSG00000179170	OR7E97P	3:125465926-125466941	q1	q2	NOTEST	0	0	0	0	1	1	no

ENSG00000179172	ENSG00000179172	HNRNPCL1	1:12907260-12908578	q1	q2	NOTEST	0	0.101932	inf	0	1	1	no

ENSG00000179178	ENSG00000179178	TMEM125	1:43629845-43739673	q1	q2	NOTEST	0	0	0	0	1	1	no

ENSG00000179195	ENSG00000179195	ZNF664	12:124247041-124800570	q1	q2	OK	120.342	126.361	0.0704123	0.119456	0.82445	0.99995	no

ENSG00000179213	ENSG00000179213	SIGLECL1	19:51749602-51774433	q1	q2	NOTEST	0	0.016347	inf	0	1	1	no

ENSG00000179218	ENSG00000179218	CALR	19:13049391-13055303	q1	q2	OK	430.85	415.885	-0.0510037	-0.103761	0.87545	0.99995	no

ENSG00000179219	ENSG00000179219	LINC00311	16:85316563-85319569	q1	q2	NOTEST	0	0	0	0	1	1	no

ENSG00000179222	ENSG00000179222	MAGED1	X:51546102-51645453	q1	q2	NOTEST	0.00948242	0.00918848	-0.0454304	0	1	1	no

ENSG00000179240	ENSG00000179240	RP11-111M22.2	11:76092356-76125663	q1	q2	OK	6.10788	6.91727	0.179531	0.224033	0.7293	0.99995	no

ENSG00000179241	ENSG00000179241	LDLRAD3	11:35965530-36253686	q1	q2	OK	23.8488	25.0504	0.070918	0.157222	0.80665	0.99995	no

ENSG00000179242	ENSG00000179242	CDH4	20:59827481-60515673	q1	q2	OK	14.8386	11.9095	-0.317245	-0.752992	0.2738	0.99995	no

ENSG00000179253	ENSG00000179253	RP11-429E11.3	20:59827481-60515673	q1	q2	NOTEST	0	0	0	0	1	1	no

ENSG00000179256	ENSG00000179256	SMCO3	12:14956505-15059520	q1	q2	NOTEST	0.0629973	0.0388597	-0.697014	0	1	1	no

ENSG00000179262	ENSG00000179262	RAD23A	19:13056668-13064456	q1	q2	OK	41.2231	42.0231	0.0277301	0.0604592	0.92825	0.99995	no

ENSG00000179270	ENSG00000179270	C2orf71	2:29179476-29297127	q1	q2	NOTEST	0.00346712	0	#NAME?	0	1	1	no

ENSG00000179271	ENSG00000179271	GADD45GIP1	19:13064971-13068050	q1	q2	OK	56.2866	54.0547	-0.0583707	-0.120709	0.86135	0.99995	no

ENSG00000179277	ENSG00000179277	MEIS3P1	17:15689931-15690889	q1	q2	OK	0.962116	0.534853	-0.847068	-0.78032	0.2931	0.99995	no

ENSG00000179284	ENSG00000179284	DAND5	19:13075972-13085567	q1	q2	NOTEST	0	0.0147821	inf	0	1	1	no

ENSG00000179292	ENSG00000179292	TMEM151A	11:66059340-66064135	q1	q2	NOTEST	0.142832	0.0800219	-0.83585	0	1	1	no

ENSG00000179294	ENSG00000179294	C17orf96	17:36827960-36831187	q1	q2	OK	5.53362	4.83706	-0.194093	-0.346935	0.61715	0.99995	no

ENSG00000179295	ENSG00000179295	PTPN11	12:112842993-112947717	q1	q2	OK	37.6473	42.191	0.16439	0.164631	0.8164	0.99995	no

ENSG00000179296	ENSG00000179296	CTGLF12P	10:49217897-49239729	q1	q2	NOTEST	0.178829	0.228394	0.352946	0	1	1	no

ENSG00000179299	ENSG00000179299	NSUN7	4:40751913-40812002	q1	q2	OK	0.683611	0.597424	-0.194421	-0.13948	0.79045	0.99995	no

ENSG00000179300	ENSG00000179300	ZCCHC5	X:77911565-77914825	q1	q2	NOTEST	0	0	0	0	1	1	no

ENSG00000179304	ENSG00000179304	FAM156B	X:52920335-52937587	q1	q2	NOTEST	0.187061	0.0851984	-1.13461	0	1	1	no

ENSG00000179314	ENSG00000179314	WSCD1	17:5675553-6027747	q1	q2	OK	29.6527	30.6054	0.0456231	0.0987979	0.8713	0.99995	no

ENSG00000179331	ENSG00000179331	RAB39A	11:107799228-107834208	q1	q2	OK	2.72527	3.25034	0.25419	0.332525	0.6361	0.99995	no

ENSG00000179335	ENSG00000179335	CLK3	15:74890840-74988633	q1	q2	OK	35.776	37.9864	0.0864912	0.101112	0.85875	0.99995	no

ENSG00000179342	ENSG00000179342	GS1-124K5.9	7:65970141-65971210	q1	q2	NOTEST	0.112284	0.106554	-0.0755659	0	1	1	no

ENSG00000179344	ENSG00000179344	HLA-DQB1	6:32627243-32636160	q1	q2	NOTEST	0.103576	0	#NAME?	0	1	1	no

ENSG00000179348	ENSG00000179348	GATA2	3:128198269-128221191	q1	q2	OK	5.17979	3.92406	-0.400547	-0.593722	0.3769	0.99995	no

ENSG00000179361	ENSG00000179361	ARID3B	15:74833517-74890472	q1	q2	OK	15.8737	17.6937	0.156598	0.279264	0.6224	0.99995	no

ENSG00000179362	ENSG00000179362	HMGN2P46	15:45722726-45878488	q1	q2	NOTEST	0.322693	0.338524	0.069093	0	1	1	no

ENSG00000179363	ENSG00000179363	TMEM31	X:102962151-102983583	q1	q2	NOTEST	0.322381	0.406638	0.33498	0	1	1	no

ENSG00000179364	ENSG00000179364	PACS2	14:105675622-105864484	q1	q2	OK	3.12138	2.8403	-0.136138	-0.0580881	0.9169	0.99995	no

ENSG00000179381	ENSG00000179381	OR4K11P	21:14916602-14917506	q1	q2	NOTEST	0	0	0	0	1	1	no

ENSG00000179387	ENSG00000179387	ELMOD2	4:141445311-141474924	q1	q2	OK	18.2263	20.3682	0.160299	0.28652	0.59635	0.99995	no

ENSG00000179388	ENSG00000179388	EGR3	8:22545171-22941132	q1	q2	OK	0.431524	0.773591	0.842129	0.148631	0.6719	0.99995	no

ENSG00000179397	ENSG00000179397	C1orf101	1:244617678-244804479	q1	q2	OK	0.114257	0.579693	2.34301	0.676712	0.01515	0.99995	no

ENSG00000179399	ENSG00000179399	GPC5	13:92050928-93519490	q1	q2	NOTEST	0.240451	0.0610994	-1.97651	0	1	1	no

ENSG00000179403	ENSG00000179403	VWA1	1:1370240-1378262	q1	q2	OK	5.84	4.61617	-0.339272	-0.405966	0.49405	0.99995	no

ENSG00000179406	ENSG00000179406	LINC00174	7:65670185-65955095	q1	q2	OK	3.57829	3.46982	-0.0444091	-0.0326147	0.93315	0.99995	no

ENSG00000179407	ENSG00000179407	DNAJB8	3:128181281-128191160	q1	q2	NOTEST	0.0325684	0.00725986	-2.16546	0	1	1	no

ENSG00000179409	ENSG00000179409	GEMIN4	17:647653-658576	q1	q2	OK	12.1397	11.9279	-0.0253928	-0.0544891	0.93525	0.99995	no

ENSG00000179412	ENSG00000179412	HNRNPCP5	1:13182959-13184326	q1	q2	NOTEST	0	0.0341392	inf	0	1	1	no

ENSG00000179420	ENSG00000179420	OR6W1P	7:142759426-142760735	q1	q2	NOTEST	0	0	0	0	1	1	no

ENSG00000179428	ENSG00000179428	AC073072.5	7:22765013-22771621	q1	q2	NOTEST	0	0	0	0	1	1	no

ENSG00000179431	ENSG00000179431	FJX1	11:35639734-35642419	q1	q2	OK	17.5846	15.9334	-0.142257	-0.296389	0.6611	0.99995	no

ENSG00000179443	ENSG00000179443	OR13C6P	9:35991332-35992282	q1	q2	NOTEST	0	0	0	0	1	1	no

ENSG00000179447	ENSG00000179447	RP5-1027G4.3	20:19193289-19703581	q1	q2	NOTEST	0	0	0	0	1	1	no

ENSG00000179449	ENSG00000179449	AC124309.1	15:23888690-23891175	q1	q2	OK	3.92056	7.23346	0.883627	0.88446	0.2206	0.99995	no

ENSG00000179452	ENSG00000179452	RP11-380B22.1	1:181205523-181207740	q1	q2	NOTEST	0	0	0	0	1	1	no

ENSG00000179454	ENSG00000179454	KLHL28	14:45393521-45543634	q1	q2	OK	6.23349	6.27427	0.00940662	0.0132764	0.97975	0.99995	no

ENSG00000179455	ENSG00000179455	MKRN3	15:23810453-23873064	q1	q2	OK	5.62212	4.7659	-0.238365	-0.351285	0.57105	0.99995	no

ENSG00000179456	ENSG00000179456	ZBTB18	1:244214584-244220778	q1	q2	OK	18.2331	17.2624	-0.0789233	-0.184851	0.78845	0.99995	no

ENSG00000179460	ENSG00000179460	EEF1A1P27	7:20621694-20622829	q1	q2	NOTEST	0	0	0	0	1	1	no

ENSG00000179467	ENSG00000179467	AL109806.1	20:55043646-55101198	q1	q2	NOTEST	0.0973099	0.199731	1.0374	0	1	1	no

ENSG00000179468	ENSG00000179468	OR9A2	7:142723244-142724282	q1	q2	NOTEST	0	0	0	0	1	1	no

ENSG00000179476	ENSG00000179476	C14orf28	14:45366497-45381066	q1	q2	OK	1.76777	1.80613	0.0309679	0.0383197	0.95095	0.99995	no

ENSG00000179477	ENSG00000179477	ALOX12B	17:7975953-7991021	q1	q2	NOTEST	0.11808	0.0834958	-0.499984	0	1	1	no

ENSG00000179520	ENSG00000179520	SLC17A8	12:100750856-100815837	q1	q2	OK	2.30639	1.33072	-0.793433	-0.993035	0.1145	0.99995	no

ENSG00000179523	ENSG00000179523	EIF3J-AS1	15:44719431-44829121	q1	q2	OK	9.06048	6.99396	-0.373477	-0.203233	0.6585	0.99995	no

ENSG00000179526	ENSG00000179526	SHARPIN	8:145153535-145173218	q1	q2	OK	19.5143	14.4993	-0.428545	-0.383556	0.4567	0.99995	no

ENSG00000179528	ENSG00000179528	LBX2	2:74724643-74731805	q1	q2	NOTEST	0.0345936	0.0873577	1.33643	0	1	1	no

ENSG00000179532	ENSG00000179532	DNHD1	11:6502676-6614988	q1	q2	OK	5.66821	4.70696	-0.268098	-0.224132	0.67505	0.99995	no

ENSG00000179542	ENSG00000179542	SLITRK4	X:142710595-142723596	q1	q2	NOTEST	0.042891	0.0681404	0.667835	0	1	1	no

ENSG00000179546	ENSG00000179546	HTR1D	1:23516992-23521222	q1	q2	NOTEST	0.0699933	0.0529895	-0.401509	0	1	1	no

ENSG00000179562	ENSG00000179562	GCC1	7:127220671-127242198	q1	q2	OK	9.2738	8.91471	-0.0569728	-0.0701116	0.91935	0.99995	no

ENSG00000179564	ENSG00000179564	LSMEM2	3:50316457-50336899	q1	q2	NOTEST	0	0	0	0	1	1	no

ENSG00000179571	ENSG00000179571	NBPF23	1:149089874-149130302	q1	q2	NOTEST	0	0	0	0	1	1	no

ENSG00000179577	ENSG00000179577	AP003471.1	8:105342551-105479281	q1	q2	NOTEST	0	0	0	0	1	1	no

ENSG00000179580	ENSG00000179580	RNF151	16:2016823-2018976	q1	q2	NOTEST	0.0834144	0.0795095	-0.0691693	0	1	1	no

ENSG00000179583	ENSG00000179583	CIITA	16:10958770-11036878	q1	q2	NOTEST	0.338468	0.365478	0.110765	0	1	1	no

ENSG00000179588	ENSG00000179588	ZFPM1	16:88519724-88603424	q1	q2	OK	1.98967	1.18869	-0.743157	-0.596604	0.1416	0.99995	no

ENSG00000179593	ENSG00000179593	ALOX15B	17:7942334-7952452	q1	q2	NOTEST	0.00986483	0	#NAME?	0	1	1	no

ENSG00000179598	ENSG00000179598	PLD6	17:16945858-17140502	q1	q2	NOTEST	0.225497	0.311324	0.465307	0	1	1	no

ENSG00000179600	ENSG00000179600	GPHB5	14:63779548-63785593	q1	q2	NOTEST	0	0	0	0	1	1	no

ENSG00000179603	ENSG00000179603	GRM8	7:126078651-126893348	q1	q2	OK	1.37717	0.959822	-0.520872	-0.452036	0.3438	0.99995	no

ENSG00000179604	ENSG00000179604	CDC42EP4	17:71279762-71308314	q1	q2	OK	37.2942	34.0901	-0.129596	-0.303386	0.64925	0.99995	no

ENSG00000179611	ENSG00000179611	DGKZP1	13:44482849-44604597	q1	q2	OK	4.72773	4.53026	-0.0615522	-0.102359	0.88425	0.99995	no

ENSG00000179615	ENSG00000179615	OR2AP1	12:55828520-55979255	q1	q2	NOTEST	0	0	0	0	1	1	no

ENSG00000179626	ENSG00000179626	OR6C4	12:55828520-55979255	q1	q2	NOTEST	0	0	0	0	1	1	no

ENSG00000179627	ENSG00000179627	ZBTB42	14:105266932-105271049	q1	q2	OK	1.96706	1.65689	-0.247564	-0.345882	0.61085	0.99995	no

ENSG00000179630	ENSG00000179630	LACC1	13:44398044-44468068	q1	q2	NOTEST	0.399062	0.472076	0.242407	0	1	1	no

ENSG00000179632	ENSG00000179632	MAF1	8:145153535-145173218	q1	q2	OK	49.2116	40.5557	-0.279094	-0.557898	0.4102	0.99995	no

ENSG00000179636	ENSG00000179636	TPPP2	14:21484921-21572881	q1	q2	NOTEST	0	0	0	0	1	1	no

ENSG00000179639	ENSG00000179639	FCER1A	1:159259503-159278014	q1	q2	NOTEST	0	0	0	0	1	1	no

ENSG00000179673	ENSG00000179673	RPRML	17:45000482-45177689	q1	q2	OK	7.3732	6.72467	-0.132827	-0.101614	0.89035	0.99995	no

ENSG00000179674	ENSG00000179674	ARL14	3:160394947-160396233	q1	q2	NOTEST	0.0230607	0	#NAME?	0	1	1	no

ENSG00000179676	ENSG00000179676	LINC00305	18:61747242-62090836	q1	q2	NOTEST	0	0	0	0	1	1	no

ENSG00000179695	ENSG00000179695	OR6C2	12:55828520-55979255	q1	q2	NOTEST	0	0	0	0	1	1	no

ENSG00000179698	ENSG00000179698	KIAA1875	8:145153535-145173218	q1	q2	OK	0.596911	0.379554	-0.653212	-0.23225	0.59595	0.99995	no

ENSG00000179709	ENSG00000179709	NLRP8	19:56459197-56499995	q1	q2	NOTEST	0	0	0	0	1	1	no

ENSG00000179715	ENSG00000179715	PCED1B	12:47469489-47630443	q1	q2	NOTEST	0.274843	0.180283	-0.608343	0	1	1	no

ENSG00000179743	ENSG00000179743	RP11-169K16.9	1:16160559-16266955	q1	q2	OK	6.08588	5.26933	-0.207844	-0.114275	0.8549	0.99995	no

ENSG00000179750	ENSG00000179750	APOBEC3B	22:39378351-39394214	q1	q2	NOTEST	0	0	0	0	1	1	no

ENSG00000179751	ENSG00000179751	SYCN	19:39693470-39694906	q1	q2	NOTEST	0	0	0	0	1	1	no

ENSG00000179755	ENSG00000179755	CTD-2144E22.5	16:34256506-34258621	q1	q2	NOTEST	0	0	0	0	1	1	no

ENSG00000179761	ENSG00000179761	PIPOX	17:27232267-27384234	q1	q2	OK	10.3814	10.7902	0.0557116	0.0429053	0.93905	0.99995	no

ENSG00000179766	ENSG00000179766	ATP8B5P	9:35406751-35483026	q1	q2	NOTEST	0.0735642	0.047018	-0.645792	0	1	1	no

ENSG00000179772	ENSG00000179772	FOXS1	20:30432102-30433420	q1	q2	OK	0.63796	0.746446	0.226572	0.183843	0.7964	0.99995	no

ENSG00000179774	ENSG00000179774	ATOH7	10:69990385-69991871	q1	q2	NOTEST	0.244967	0.313538	0.356053	0	1	1	no

ENSG00000179776	ENSG00000179776	CDH5	16:66400532-66438686	q1	q2	NOTEST	0.393449	0.477274	0.27864	0	1	1	no

ENSG00000179796	ENSG00000179796	LRRC3B	3:26664296-26752267	q1	q2	NOTEST	0.415383	0.477698	0.201654	0	1	1	no

ENSG00000179799	ENSG00000179799	OR7E22P	3:75405666-75406654	q1	q2	NOTEST	0.0318042	0.0332943	0.0660607	0	1	1	no

ENSG00000179813	ENSG00000179813	FAM216B	13:43355685-43365683	q1	q2	NOTEST	0.00779881	0	#NAME?	0	1	1	no

ENSG00000179817	ENSG00000179817	MRGPRX4	11:18161732-18211168	q1	q2	NOTEST	0	0	0	0	1	1	no

ENSG00000179818	ENSG00000179818	PCBP1-AS1	2:70187225-70316332	q1	q2	OK	20.3487	20.5671	0.0153967	0.00771538	0.97165	0.99995	no

ENSG00000179820	ENSG00000179820	MYADM	19:54357834-54379691	q1	q2	OK	13.9203	17.1897	0.30435	0.580163	0.39075	0.99995	no

ENSG00000179826	ENSG00000179826	MRGPRX3	11:18142501-18160027	q1	q2	NOTEST	0	0	0	0	1	1	no

ENSG00000179832	ENSG00000179832	MROH1	8:145202918-145316843	q1	q2	OK	1.92888	1.95929	0.0225701	0.0236349	0.97025	0.99995	no

ENSG00000179833	ENSG00000179833	SERTAD2	2:64858754-64978139	q1	q2	OK	7.51784	8.34711	0.15096	0.220028	0.63255	0.99995	no

ENSG00000179837	ENSG00000179837	RBM15B	3:51428730-51534010	q1	q2	NOTEST	0	0	0	0	1	1	no

ENSG00000179840	ENSG00000179840	C1orf200	1:9711789-9884584	q1	q2	NOTEST	0	0	0	0	1	1	no

ENSG00000179841	ENSG00000179841	AKAP5	14:64854748-65009955	q1	q2	OK	1.31409	1.68695	0.360349	0.174973	0.7739	0.99995	no

ENSG00000179846	ENSG00000179846	NKPD1	19:45574757-45808541	q1	q2	NOTEST	0.164291	0.089575	-0.875085	0	1	1	no

ENSG00000179855	ENSG00000179855	GIPC3	19:3585550-3593539	q1	q2	NOTEST	0.163776	0.129808	-0.335337	0	1	1	no

ENSG00000179859	ENSG00000179859	AC025335.1	17:7816641-7819271	q1	q2	OK	1.67559	1.65868	-0.0146275	-0.0188144	0.9781	0.99995	no

ENSG00000179862	ENSG00000179862	CITED4	1:41326728-41328018	q1	q2	OK	1.7911	1.61039	-0.153443	-0.172802	0.7967	0.99995	no

ENSG00000179869	ENSG00000179869	ABCA13	7:48211054-48687092	q1	q2	NOTEST	0.144619	0.137785	-0.0698434	0	1	1	no

ENSG00000179873	ENSG00000179873	NLRP11	19:56296769-56393220	q1	q2	NOTEST	0	0.0165634	inf	0	1	1	no

ENSG00000179886	ENSG00000179886	TIGD5	8:144661805-144682485	q1	q2	OK	5.87106	4.74233	-0.308024	-0.170837	0.80075	0.99995	no

ENSG00000179889	ENSG00000179889	PDXDC1	16:15068447-15248421	q1	q2	OK	22.3415	21.2827	-0.0700489	-0.0767539	0.8735	0.99995	no

ENSG00000179899	ENSG00000179899	PHC1P1	12:55804262-55808633	q1	q2	OK	5.09741	5.18761	0.0253065	0.0392562	0.95625	0.99995	no

ENSG00000179902	ENSG00000179902	C1orf194	1:109648572-109749401	q1	q2	OK	4.59759	3.70407	-0.311766	-0.222489	0.7917	0.99995	no

ENSG00000179909	ENSG00000179909	ZNF154	19:58193336-58269527	q1	q2	OK	5.6981	5.98491	0.0708489	0.0787832	0.91145	0.99995	no

ENSG00000179912	ENSG00000179912	R3HDM2	12:57637235-57824788	q1	q2	OK	16.4894	18.6866	0.180462	0.267187	0.60195	0.99995	no

ENSG00000179913	ENSG00000179913	B3GNT3	19:17905636-17923891	q1	q2	NOTEST	0	0	0	0	1	1	no

ENSG00000179914	ENSG00000179914	ITLN1	1:160846328-160854960	q1	q2	NOTEST	0	0	0	0	1	1	no

ENSG00000179915	ENSG00000179915	NRXN1	2:50145642-51259674	q1	q2	OK	1.82601	1.36987	-0.414662	-0.355188	0.48655	0.99995	no

ENSG00000179918	ENSG00000179918	SEPHS2	16:30454951-30457502	q1	q2	OK	24.2367	24.0494	-0.0111885	-0.0252538	0.972	0.99995	no

ENSG00000179919	ENSG00000179919	OR10A7	12:55614808-55615759	q1	q2	NOTEST	0	0	0	0	1	1	no

ENSG00000179921	ENSG00000179921	GPBAR1	2:219124218-219128582	q1	q2	NOTEST	0.182572	0.175515	-0.056865	0	1	1	no

ENSG00000179922	ENSG00000179922	ZNF784	19:56132107-56135967	q1	q2	OK	6.60278	6.71734	0.024816	0.0416347	0.95465	0.99995	no

ENSG00000179930	ENSG00000179930	ZNF648	1:182023704-182030847	q1	q2	NOTEST	0	0.0143131	inf	0	1	1	no

ENSG00000179933	ENSG00000179933	C14orf119	14:23527772-23569665	q1	q2	OK	37.963	33.7911	-0.16795	-0.105137	0.9024	0.99995	no

ENSG00000179934	ENSG00000179934	CCR8	3:39371196-39375171	q1	q2	NOTEST	0.0225366	0	#NAME?	0	1	1	no

ENSG00000179935	ENSG00000179935	LINC00652	20:18768294-18774979	q1	q2	NOTEST	0.313386	0.474341	0.597986	0	1	1	no

ENSG00000179938	ENSG00000179938	GOLGA8J	15:30375157-30385702	q1	q2	NOTEST	0.0698342	0.0135561	-2.365	0	1	1	no

ENSG00000179941	ENSG00000179941	BBS10	12:76738253-76742222	q1	q2	OK	8.18692	9.05309	0.14509	0.297674	0.6778	0.99995	no

ENSG00000179943	ENSG00000179943	FIZ1	19:56102745-56114504	q1	q2	OK	16.0564	16.1034	0.00421885	0.0057913	0.9932	0.99995	no

ENSG00000179950	ENSG00000179950	PUF60	8:144898513-144912029	q1	q2	OK	12.4367	4.02581	-1.62725	-1.64725	0.01715	0.99995	no

ENSG00000179954	ENSG00000179954	SSC5D	19:55999770-56030465	q1	q2	OK	0.65802	0.481264	-0.451302	-0.325693	0.51665	0.99995	no

ENSG00000179958	ENSG00000179958	DCTPP1	16:30418617-30441396	q1	q2	OK	25.8464	26.3747	0.0291909	0.0515838	0.93985	0.99995	no

ENSG00000179965	ENSG00000179965	ZNF771	16:30418617-30441396	q1	q2	OK	7.8025	6.64987	-0.23061	-0.241484	0.74135	0.99995	no

ENSG00000179967	ENSG00000179967	PPP1R14BP3	4:139936942-140098372	q1	q2	OK	28.0935	27.2409	-0.0444643	-0.0336839	0.96305	0.99995	no

ENSG00000179978	ENSG00000179978	RP11-1319K7.1	5:69390201-69424261	q1	q2	NOTEST	0.0448785	0.0590483	0.395873	0	1	1	no

ENSG00000179979	ENSG00000179979	CRIPAK	4:1385339-1389780	q1	q2	OK	4.56903	4.36986	-0.0643011	-0.119615	0.8628	0.99995	no

ENSG00000179981	ENSG00000179981	TSHZ1	18:72922709-73001905	q1	q2	OK	7.36397	8.12675	0.142194	0.298663	0.6664	0.99995	no

ENSG00000179988	ENSG00000179988	PSTK	10:124690418-124768333	q1	q2	OK	6.2957	6.18333	-0.0259827	-0.0194544	0.9799	0.99995	no

ENSG00000179994	ENSG00000179994	SPDYE7P	7:72333320-72341076	q1	q2	NOTEST	0	0	0	0	1	1	no

ENSG00000179997	ENSG00000179997	AC010894.4	2:175366679-175367043	q1	q2	NOTEST	0	0	0	0	1	1	no

ENSG00000180008	ENSG00000180008	SOCS4	14:55493947-55516206	q1	q2	OK	13.6878	16.5317	0.272344	0.644292	0.35235	0.99995	no

ENSG00000180011	ENSG00000180011	ZADH2	18:72907062-72921303	q1	q2	OK	14.8855	15.4316	0.05198	0.0717702	0.8989	0.99995	no

ENSG00000180015	ENSG00000180015	RP11-756P10.3	4:189659505-189660648	q1	q2	OK	0.865084	0.78424	-0.141544	-0.137462	0.8439	0.99995	no

ENSG00000180016	ENSG00000180016	OR1E1	17:3300397-3301704	q1	q2	NOTEST	0.0214372	0	#NAME?	0	1	1	no

ENSG00000180019	ENSG00000180019	AC079741.2	7:112063022-112131117	q1	q2	NOTEST	0	0	0	0	1	1	no

ENSG00000180035	ENSG00000180035	ZNF48	16:30389426-30411429	q1	q2	OK	45.1762	43.4555	-0.0560257	-0.0824024	0.8844	0.99995	no

ENSG00000180042	ENSG00000180042	OR1R1P	17:3181192-3290132	q1	q2	NOTEST	0	0	0	0	1	1	no

ENSG00000180043	ENSG00000180043	FAM71E2	19:55860673-55874628	q1	q2	NOTEST	0	0	0	0	1	1	no

ENSG00000180044	ENSG00000180044	C3orf80	3:159943422-160203561	q1	q2	NOTEST	0.246764	0.267575	0.116809	0	1	1	no

ENSG00000180053	ENSG00000180053	NKX2-6	8:23559963-23564111	q1	q2	NOTEST	0	0	0	0	1	1	no

ENSG00000180061	ENSG00000180061	TMEM150B	19:55824168-55845416	q1	q2	NOTEST	0.0376655	0	#NAME?	0	1	1	no

ENSG00000180066	ENSG00000180066	C10orf91	10:134257830-134262912	q1	q2	NOTEST	0	0.0232988	inf	0	1	1	no

ENSG00000180068	ENSG00000180068	OR3A4P	17:3181192-3290132	q1	q2	NOTEST	0	0	0	0	1	1	no

ENSG00000180071	ENSG00000180071	ANKRD18A	9:38540563-38624987	q1	q2	OK	0.83126	1.71587	1.04557	0.369949	0.2975	0.99995	no

ENSG00000180081	ENSG00000180081	CTC-451A6.4	19:22934984-22937677	q1	q2	NOTEST	0	0	0	0	1	1	no

ENSG00000180083	ENSG00000180083	WFDC11	20:44277201-44298909	q1	q2	NOTEST	0	0	0	0	1	1	no

ENSG00000180089	ENSG00000180089	TMEM86B	19:55738006-55770363	q1	q2	OK	2.64687	1.94084	-0.447612	-0.225425	0.8526	0.99995	no

ENSG00000180090	ENSG00000180090	OR3A1	17:3181192-3290132	q1	q2	NOTEST	0	0	0	0	1	1	no

ENSG00000180096	ENSG00000180096	Sep-01	16:30389426-30411429	q1	q2	OK	2.19523	1.45498	-0.593373	-0.131146	0.57245	0.99995	no

ENSG00000180098	ENSG00000180098	TRNAU1AP	1:28879596-28909495	q1	q2	OK	21.4046	22.5487	0.0751225	0.108419	0.87405	0.99995	no

ENSG00000180104	ENSG00000180104	EXOC3	5:443272-473213	q1	q2	OK	27.5384	21.7525	-0.340265	-0.559152	0.35405	0.99995	no

ENSG00000180105	ENSG00000180105	AC017081.2	2:207274810-207275633	q1	q2	NOTEST	0.114285	0.11619	0.0238443	0	1	1	no

ENSG00000180113	ENSG00000180113	TDRD6	6:46620677-46703430	q1	q2	NOTEST	0.102339	0.0557618	-0.876001	0	1	1	no

ENSG00000180116	ENSG00000180116	C12orf40	12:40019968-40499891	q1	q2	NOTEST	0	0	0	0	1	1	no

ENSG00000180138	ENSG00000180138	CSNK1A1L	13:37677397-37679803	q1	q2	NOTEST	0.0340255	0.137834	2.01824	0	1	1	no

ENSG00000180139	ENSG00000180139	ACTA2-AS1	10:90639490-90775542	q1	q2	NOTEST	0.473778	0.401805	-0.237717	0	1	1	no

ENSG00000180150	ENSG00000180150	HMGN2P9	22:43911996-44208217	q1	q2	NOTEST	0	0	0	0	1	1	no

ENSG00000180152	ENSG00000180152	AC079753.4	2:113610904-113611699	q1	q2	NOTEST	0.0398881	0.0803766	1.01082	0	1	1	no

ENSG00000180155	ENSG00000180155	LYNX1	8:143844533-143859640	q1	q2	OK	0.596239	0.523003	-0.189072	-0.125505	0.7314	0.99995	no

ENSG00000180172	ENSG00000180172	RPS12P23	13:21668456-21668853	q1	q2	NOTEST	0	0.239417	inf	0	1	1	no

ENSG00000180176	ENSG00000180176	TH	11:2185158-2193107	q1	q2	NOTEST	0.348988	0.122809	-1.50676	0	1	1	no

ENSG00000180178	ENSG00000180178	FAR2P1	2:130748028-130808704	q1	q2	NOTEST	0.265449	0.0399567	-2.73192	0	1	1	no

ENSG00000180182	ENSG00000180182	MED14	X:40507557-40597950	q1	q2	OK	22.9584	24.3448	0.0845924	0.0962477	0.8261	0.99995	no

ENSG00000180185	ENSG00000180185	FAHD1	16:1845620-1934295	q1	q2	OK	26.1983	25.4133	-0.0438931	-0.0809453	0.9103	0.99995	no

ENSG00000180189	ENSG00000180189	HMGB1P14	14:58466452-58764857	q1	q2	OK	0.524836	0.552826	0.0749594	0.0046226	0.95425	0.99995	no

ENSG00000180190	ENSG00000180190	TDRP	8:439802-495781	q1	q2	OK	15.8479	15.2299	-0.057389	-0.0992019	0.85595	0.99995	no

ENSG00000180198	ENSG00000180198	RCC1	1:28832454-28865812	q1	q2	OK	44.1522	42.4745	-0.0558901	-0.108969	0.86715	0.99995	no

ENSG00000180205	ENSG00000180205	WFDC9	20:44236577-44259907	q1	q2	NOTEST	0	0	0	0	1	1	no

ENSG00000180209	ENSG00000180209	MYLPF	16:30382254-30389312	q1	q2	NOTEST	0.0623586	0.111525	0.838705	0	1	1	no

ENSG00000180210	ENSG00000180210	F2	11:46740729-46761056	q1	q2	NOTEST	0.0301542	0.0270554	-0.156445	0	1	1	no

ENSG00000180211	ENSG00000180211	RP1-278E11.3	6:39926152-39926572	q1	q2	OK	2.72713	8.39077	1.62142	1.48693	0.05365	0.99995	no

ENSG00000180219	ENSG00000180219	FAM71C	12:99038918-100378432	q1	q2	NOTEST	0	0	0	0	1	1	no

ENSG00000180221	ENSG00000180221	TPT1P10	10:59951277-60027694	q1	q2	NOTEST	0	0.241434	inf	0	1	1	no

ENSG00000180228	ENSG00000180228	PRKRA	2:179278665-179326117	q1	q2	OK	53.7868	54.6365	0.0226151	0.043937	0.9464	0.99995	no

ENSG00000180229	ENSG00000180229	HERC2P3	15:20587868-20711433	q1	q2	OK	0.640394	0.661673	0.047159	0.0257751	0.9603	0.99995	no

ENSG00000180230	ENSG00000180230	NACAP2	10:121756597-121757221	q1	q2	NOTEST	0	0	0	0	1	1	no

ENSG00000180233	ENSG00000180233	ZNRF2	7:30323922-30452118	q1	q2	OK	1.66485	1.26845	-0.392332	-0.358948	0.513	0.99995	no

ENSG00000180245	ENSG00000180245	RRH	4:110749149-110765760	q1	q2	NOTEST	0.155436	0.26739	0.782619	0	1	1	no

ENSG00000180251	ENSG00000180251	SLC9A4	2:103089761-103150431	q1	q2	NOTEST	0.0120512	0.00626867	-0.942948	0	1	1	no

ENSG00000180257	ENSG00000180257	ZNF816	19:53430387-53541151	q1	q2	OK	5.86145	7.37526	0.331438	0.355432	0.5354	0.99995	no

ENSG00000180259	ENSG00000180259	PRNT	20:4711927-4721314	q1	q2	NOTEST	0	0	0	0	1	1	no

ENSG00000180263	ENSG00000180263	FGD6	12:95470524-95611258	q1	q2	OK	5.15349	5.47045	0.086109	0.14561	0.78775	0.99995	no

ENSG00000180264	ENSG00000180264	GPR144	9:127213422-127239379	q1	q2	NOTEST	0	0	0	0	1	1	no

ENSG00000180269	ENSG00000180269	GPR139	16:20042806-20085239	q1	q2	NOTEST	0.0210763	0.0174433	-0.272943	0	1	1	no

ENSG00000180279	ENSG00000180279	CTD-2568A17.5	19:51320936-51322134	q1	q2	NOTEST	0.0340574	0.077439	1.18509	0	1	1	no

ENSG00000180284	ENSG00000180284	RP5-1055C14.6	X:102906662-102907034	q1	q2	NOTEST	0	0	0	0	1	1	no

ENSG00000180287	ENSG00000180287	PLD5	1:242246287-242687998	q1	q2	OK	1.88238	1.58819	-0.245176	-0.187466	0.7147	0.99995	no

ENSG00000180304	ENSG00000180304	OAZ2	15:64979771-65011801	q1	q2	OK	64.016	64.3403	0.00729068	0.0165448	0.98015	0.99995	no

ENSG00000180305	ENSG00000180305	WFDC10A	20:44236577-44259907	q1	q2	NOTEST	0	0	0	0	1	1	no

ENSG00000180316	ENSG00000180316	PNPLA1	6:36210979-36276372	q1	q2	NOTEST	0.0741265	0.0735894	-0.0104909	0	1	1	no

ENSG00000180318	ENSG00000180318	ALX1	12:85673884-85695562	q1	q2	OK	0.532491	0.47469	-0.165771	-0.157393	0.82635	0.99995	no

ENSG00000180329	ENSG00000180329	CCDC43	17:42733761-42767676	q1	q2	OK	18.4502	19.4445	0.0757248	0.134476	0.8398	0.99995	no

ENSG00000180332	ENSG00000180332	KCTD4	13:45694649-45858237	q1	q2	NOTEST	0.0743705	0.0592317	-0.32836	0	1	1	no

ENSG00000180336	ENSG00000180336	C17orf104	17:42733761-42767676	q1	q2	OK	2.00471	2.02948	0.017713	0.00686325	0.98895	0.99995	no

ENSG00000180340	ENSG00000180340	FZD2	17:42634924-42636907	q1	q2	OK	34.3991	30.2036	-0.187651	-0.4352	0.5294	0.99995	no

ENSG00000180344	ENSG00000180344	AL035460.1	20:2673523-2740753	q1	q2	NOTEST	0.104472	0	#NAME?	0	1	1	no

ENSG00000180346	ENSG00000180346	TIGD2	4:90033967-90036050	q1	q2	OK	3.50751	4.54423	0.37359	0.561343	0.4289	0.99995	no

ENSG00000180347	ENSG00000180347	CCDC129	7:31553703-31698334	q1	q2	NOTEST	0.0389614	0.0517581	0.40974	0	1	1	no

ENSG00000180353	ENSG00000180353	HCLS1	3:121350245-121379774	q1	q2	NOTEST	0.254484	0.36217	0.50909	0	1	1	no

ENSG00000180354	ENSG00000180354	MTURN	7:30174425-30202378	q1	q2	OK	4.43612	3.70031	-0.261651	-0.425566	0.48885	0.99995	no

ENSG00000180357	ENSG00000180357	ZNF609	15:64752940-64978264	q1	q2	OK	19.4415	19.1613	-0.020949	-0.0507374	0.9396	0.99995	no

ENSG00000180370	ENSG00000180370	PAK2	3:196466727-196559518	q1	q2	OK	45.7185	50.525	0.144219	0.279234	0.61375	0.99995	no

ENSG00000180376	ENSG00000180376	CCDC66	3:56591188-56717265	q1	q2	OK	20.1319	21.9528	0.124928	0.0998564	0.8016	0.99995	no

ENSG00000180383	ENSG00000180383	DEFB124	20:30053308-30072708	q1	q2	NOTEST	0.0639596	0	#NAME?	0	1	1	no

ENSG00000180385	ENSG00000180385	EMC3-AS1	3:10000400-10052893	q1	q2	OK	5.13722	4.27284	-0.265793	-0.193795	0.7032	0.99995	no

ENSG00000180386	ENSG00000180386	KRTAP9-7	17:39431910-39432949	q1	q2	NOTEST	0	0	0	0	1	1	no

ENSG00000180389	ENSG00000180389	ATP5EP2	13:28519342-28519727	q1	q2	OK	0.388165	2.41181	2.63537	1.68815	0.11005	0.99995	no

ENSG00000180398	ENSG00000180398	MCFD2	2:47126413-47403740	q1	q2	OK	37.8616	37.8534	-0.000313798	-0.000234463	0.9997	0.99995	no

ENSG00000180409	ENSG00000180409	OR10AA1P	1:158778188-158779125	q1	q2	NOTEST	0	0	0	0	1	1	no

ENSG00000180422	ENSG00000180422	LINC00304	16:89225553-89230653	q1	q2	OK	1.21063	1.29728	0.0997381	0.1062	0.88245	0.99995	no

ENSG00000180423	ENSG00000180423	HARBI1	11:46624410-46696368	q1	q2	OK	4.54704	4.7838	0.0732301	0.0405585	0.94245	0.99995	no

ENSG00000180424	ENSG00000180424	DEFB123	20:30028321-30038060	q1	q2	NOTEST	0	0	0	0	1	1	no

ENSG00000180425	ENSG00000180425	C11orf71	11:114262164-114321001	q1	q2	OK	1.09284	1.43384	0.391812	0.0888763	0.8688	0.99995	no

ENSG00000180432	ENSG00000180432	CYP8B1	3:42846243-43101703	q1	q2	NOTEST	0.0129974	0	#NAME?	0	1	1	no

ENSG00000180433	ENSG00000180433	OR6K6	1:158724509-158725681	q1	q2	NOTEST	0	0	0	0	1	1	no

ENSG00000180437	ENSG00000180437	OR6K4P	1:158693902-158694902	q1	q2	NOTEST	0	0	0	0	1	1	no

ENSG00000180438	ENSG00000180438	TPRXL	3:13974552-14124311	q1	q2	NOTEST	0	0.0332436	inf	0	1	1	no

ENSG00000180440	ENSG00000180440	SERTM1	13:37248048-37271976	q1	q2	NOTEST	0.398637	0.436715	0.131616	0	1	1	no

ENSG00000180447	ENSG00000180447	GAS1	9:89559278-89562104	q1	q2	OK	12.4475	11.8346	-0.0728461	-0.149355	0.83535	0.99995	no

ENSG00000180448	ENSG00000180448	HMHA1	19:1065921-1095598	q1	q2	OK	6.24572	4.34682	-0.522909	-0.324114	0.6234	0.99995	no

ENSG00000180458	ENSG00000180458	CTD-3064H18.4	19:37988062-38210691	q1	q2	NOTEST	0.073428	0.127227	0.793	0	1	1	no

ENSG00000180475	ENSG00000180475	OR10Q1	11:57995353-57996390	q1	q2	NOTEST	0	0	0	0	1	1	no

ENSG00000180479	ENSG00000180479	ZNF571	19:37988062-38210691	q1	q2	OK	4.05564	3.51435	-0.206673	-0.0809469	0.87355	0.99995	no

ENSG00000180481	ENSG00000180481	GLIPR1L2	12:75784849-75826468	q1	q2	NOTEST	0.100886	0.206297	1.03199	0	1	1	no

ENSG00000180483	ENSG00000180483	DEFB119	20:29964966-29978406	q1	q2	NOTEST	0	0	0	0	1	1	no

ENSG00000180488	ENSG00000180488	FAM73A	1:78245308-78344106	q1	q2	OK	3.225	3.28787	0.027853	0.0333165	0.95445	0.99995	no

ENSG00000180509	ENSG00000180509	KCNE1	21:35818987-35884573	q1	q2	NOTEST	0.140844	0.0595749	-1.24132	0	1	1	no

ENSG00000180525	ENSG00000180525	PRR26	10:320129-735683	q1	q2	NOTEST	0.18693	0.24842	0.410285	0	1	1	no

ENSG00000180530	ENSG00000180530	NRIP1	21:16333555-16437321	q1	q2	OK	18.7454	20.2755	0.113199	0.260455	0.7132	0.99995	no

ENSG00000180532	ENSG00000180532	ZSCAN4	19:58180302-58190520	q1	q2	NOTEST	0.0115981	0.0112477	-0.0442569	0	1	1	no

ENSG00000180535	ENSG00000180535	BHLHA15	7:97840738-97842291	q1	q2	NOTEST	0.099197	0.0460846	-1.10601	0	1	1	no

ENSG00000180537	ENSG00000180537	RNF182	6:13924676-13980533	q1	q2	OK	5.92119	5.8386	-0.0202635	-0.0319899	0.95965	0.99995	no

ENSG00000180539	ENSG00000180539	C9orf139	9:139901685-139931234	q1	q2	NOTEST	0.0503656	0.0439264	-0.197349	0	1	1	no

ENSG00000180543	ENSG00000180543	TSPYL5	8:98285716-98290176	q1	q2	NOTEST	0	0	0	0	1	1	no

ENSG00000180549	ENSG00000180549	FUT7	9:139901685-139931234	q1	q2	NOTEST	0.0452348	0.0237067	-0.93214	0	1	1	no

ENSG00000180573	ENSG00000180573	HIST1H2AC	6:26124372-26139344	q1	q2	OK	3.34344	4.42473	0.404255	0.516571	0.44325	0.99995	no

ENSG00000180574	ENSG00000180574	EIF2S3L	12:10658200-10675734	q1	q2	OK	4.73307	5.3076	0.165284	0.243584	0.71865	0.99995	no

ENSG00000180581	ENSG00000180581	SRP9P1	10:93558068-93625033	q1	q2	OK	19.0298	28.7609	0.595853	0.159211	0.7504	0.99995	no

ENSG00000180592	ENSG00000180592	SKIDA1	10:21802406-21814611	q1	q2	OK	18.3734	16.0163	-0.198071	-0.389445	0.56795	0.99995	no

ENSG00000180596	ENSG00000180596	HIST1H2BC	6:26115100-26124154	q1	q2	OK	0.425994	0.669223	0.651654	0.446509	0.5004	0.99995	no

ENSG00000180610	ENSG00000180610	ZBTB12P1	4:39699663-39784412	q1	q2	NOTEST	0.332348	0.445714	0.423423	0	1	1	no

ENSG00000180611	ENSG00000180611	MB21D2	3:192514603-192635950	q1	q2	OK	2.98909	2.83196	-0.0779038	-0.122394	0.8585	0.99995	no

ENSG00000180613	ENSG00000180613	GSX2	4:54243809-55164414	q1	q2	NOTEST	0	0	0	0	1	1	no

ENSG00000180616	ENSG00000180616	SSTR2	17:71161115-71258491	q1	q2	OK	0.784047	0.841143	0.10141	0.026418	0.9568	0.99995	no

ENSG00000180626	ENSG00000180626	ZNF594	17:5082830-5095178	q1	q2	OK	6.84918	7.33042	0.0979649	0.144482	0.7938	0.99995	no

ENSG00000180628	ENSG00000180628	PCGF5	10:92979907-93044088	q1	q2	OK	6.69716	5.80548	-0.206134	-0.227959	0.71995	0.99995	no

ENSG00000180636	ENSG00000180636	OR4E1	14:22138202-22139150	q1	q2	NOTEST	0	0	0	0	1	1	no

ENSG00000180638	ENSG00000180638	SLC47A2	17:19581600-19622292	q1	q2	NOTEST	0.0773373	0.0580797	-0.41313	0	1	1	no

ENSG00000180644	ENSG00000180644	PRF1	10:72357103-72362531	q1	q2	NOTEST	0.0103468	0.0523574	2.3392	0	1	1	no

ENSG00000180658	ENSG00000180658	OR2A4	6:131949581-132068553	q1	q2	NOTEST	0	0	0	0	1	1	no

ENSG00000180660	ENSG00000180660	MAB21L1	13:35516423-36247159	q1	q2	OK	0.80699	0.607547	-0.409555	-0.172946	0.84965	0.99995	no

ENSG00000180662	ENSG00000180662	RPL21P8	14:65877309-66210839	q1	q2	NOTEST	0	0.145979	inf	0	1	1	no

ENSG00000180663	ENSG00000180663	VN1R3	16:31814267-31820165	q1	q2	NOTEST	0.0330076	0	#NAME?	0	1	1	no

ENSG00000180667	ENSG00000180667	YOD1	1:207217193-207254369	q1	q2	OK	6.64571	6.95422	0.0654667	0.128006	0.86065	0.99995	no

ENSG00000180672	ENSG00000180672	AC007362.1	2:206546713-206662857	q1	q2	OK	12.4444	17.1533	0.462983	0.340854	0.62695	0.99995	no

ENSG00000180673	ENSG00000180673	EXOC5P1	4:63682543-63684512	q1	q2	OK	0.675614	1.02542	0.60194	0.622204	0.3906	0.99995	no

ENSG00000180694	ENSG00000180694	TMEM64	8:91634222-91997485	q1	q2	OK	4.25361	4.18019	-0.0251167	-0.0369883	0.9513	0.99995	no

ENSG00000180697	ENSG00000180697	C3orf22	3:126243125-126277808	q1	q2	NOTEST	0	0.0307515	inf	0	1	1	no

ENSG00000180708	ENSG00000180708	OR10K2	1:158389717-158390656	q1	q2	NOTEST	0	0	0	0	1	1	no

ENSG00000180712	ENSG00000180712	RP11-290F5.2	4:185261908-185275130	q1	q2	NOTEST	0.174741	0.0362002	-2.27115	0	1	1	no

ENSG00000180714	ENSG00000180714	OR5AZ1P	11:57684768-57685711	q1	q2	NOTEST	0	0	0	0	1	1	no

ENSG00000180720	ENSG00000180720	CHRM4	11:46406639-46408107	q1	q2	NOTEST	0.246386	0.224544	-0.133919	0	1	1	no

ENSG00000180723	ENSG00000180723	OR51A9P	11:4682082-4682993	q1	q2	NOTEST	0	0	0	0	1	1	no

ENSG00000180725	ENSG00000180725	AC015871.1	15:80282246-80282948	q1	q2	NOTEST	0	0	0	0	1	1	no

ENSG00000180730	ENSG00000180730	SHISA2	13:26618734-26625169	q1	q2	OK	16.3611	17.8674	0.127057	0.260012	0.7095	0.99995	no

ENSG00000180739	ENSG00000180739	S1PR5	19:10623622-10628607	q1	q2	OK	1.03867	0.894356	-0.215815	-0.245819	0.7182	0.99995	no

ENSG00000180745	ENSG00000180745	CLRN3	10:129676104-129691211	q1	q2	NOTEST	0	0	0	0	1	1	no

ENSG00000180747	ENSG00000180747	CTD-2547E10.2	16:21413557-21531765	q1	q2	OK	3.51343	4.04334	0.202665	0.13261	0.8249	0.99995	no

ENSG00000180758	ENSG00000180758	GPR157	1:9160363-9189229	q1	q2	OK	0.399584	0.584307	0.548229	0.301056	0.5015	0.99995	no

ENSG00000180764	ENSG00000180764	PIPSL	10:95717947-95721297	q1	q2	OK	0.673381	0.821993	0.287702	0.274415	0.6974	0.99995	no

ENSG00000180767	ENSG00000180767	CHST13	3:126243125-126277808	q1	q2	NOTEST	0.0306854	0	#NAME?	0	1	1	no

ENSG00000180769	ENSG00000180769	WDFY3-AS2	4:85590703-85932430	q1	q2	OK	1.45452	1.75043	0.267169	0.107867	0.8728	0.99995	no

ENSG00000180770	ENSG00000180770	OR7E129P	3:129740395-129741363	q1	q2	NOTEST	0	0	0	0	1	1	no

ENSG00000180771	ENSG00000180771	SRSF8	11:94800055-94804388	q1	q2	NOTEST	0	0	0	0	1	1	no

ENSG00000180772	ENSG00000180772	AGTR2	X:115301974-115306225	q1	q2	NOTEST	0	0	0	0	1	1	no

ENSG00000180773	ENSG00000180773	SLC36A4	11:92877340-92931130	q1	q2	OK	18.9628	21.0542	0.150931	0.220548	0.7341	0.99995	no

ENSG00000180776	ENSG00000180776	ZDHHC20	13:21950262-22033509	q1	q2	OK	23.5157	25.6089	0.123021	0.180234	0.78775	0.99995	no

ENSG00000180777	ENSG00000180777	ANKRD30B	18:14728270-14852737	q1	q2	NOTEST	0	0	0	0	1	1	no

ENSG00000180785	ENSG00000180785	OR51E1	11:4664649-4676718	q1	q2	NOTEST	0.00695457	0	#NAME?	0	1	1	no

ENSG00000180787	ENSG00000180787	ZFP3	17:4981542-4999669	q1	q2	NOTEST	0.0636638	0.0853103	0.422245	0	1	1	no

ENSG00000180801	ENSG00000180801	ARSJ	4:114821439-114900883	q1	q2	OK	0.714727	0.648652	-0.139947	-0.137275	0.8444	0.99995	no

ENSG00000180803	ENSG00000180803	RP11-467D10.2	13:25946208-26599989	q1	q2	NOTEST	0	0	0	0	1	1	no

ENSG00000180806	ENSG00000180806	HOXC9	12:54375292-54449814	q1	q2	NOTEST	0	0	0	0	1	1	no

ENSG00000180815	ENSG00000180815	MAP3K15	X:19362010-19533379	q1	q2	OK	0.598196	0.855779	0.516619	0.211559	0.7289	0.99995	no

ENSG00000180817	ENSG00000180817	PPA1	10:71962585-71993667	q1	q2	OK	85.521	94.6803	0.146786	0.357388	0.607	0.99995	no

ENSG00000180818	ENSG00000180818	HOXC10	12:54375292-54449814	q1	q2	NOTEST	0	0	0	0	1	1	no

ENSG00000180822	ENSG00000180822	PSMG4	6:3224494-3457256	q1	q2	OK	11.7021	10.9113	-0.100952	-0.0334929	0.90275	0.99995	no

ENSG00000180828	ENSG00000180828	BHLHE22	8:65486862-65496181	q1	q2	OK	0.976195	1.23977	0.344826	0.4132	0.54885	0.99995	no

ENSG00000180834	ENSG00000180834	MAP6D1	3:183533663-183543382	q1	q2	OK	4.50885	4.03526	-0.160097	-0.231954	0.7217	0.99995	no

ENSG00000180846	ENSG00000180846	CSNK1G2-AS1	19:1941187-1981337	q1	q2	NOTEST	0.208152	0.318045	0.611592	0	1	1	no

ENSG00000180855	ENSG00000180855	ZNF443	19:12490002-12750912	q1	q2	OK	5.17476	6.0897	0.234881	0.209546	0.764	0.99995	no

ENSG00000180861	ENSG00000180861	C12orf36	12:13524022-13540101	q1	q2	NOTEST	0	0.0288524	inf	0	1	1	no

ENSG00000180867	ENSG00000180867	PDIA3P1	1:146644349-146767443	q1	q2	NOTEST	0	0	0	0	1	1	no

ENSG00000180869	ENSG00000180869	C1orf180	1:85093912-85100703	q1	q2	NOTEST	0.00963002	0	#NAME?	0	1	1	no

ENSG00000180871	ENSG00000180871	CXCR2	2:218990011-219001976	q1	q2	NOTEST	0	0.0270029	inf	0	1	1	no

ENSG00000180872	ENSG00000180872	DEFB112	6:50011287-50016364	q1	q2	NOTEST	0	0	0	0	1	1	no

ENSG00000180875	ENSG00000180875	GREM2	1:240652872-240775449	q1	q2	OK	2.2127	2.2685	0.0359277	0.0545681	0.9375	0.99995	no

ENSG00000180878	ENSG00000180878	C11orf42	11:6226795-6232362	q1	q2	OK	0.524866	0.127509	-2.04135	-1.4341	0.08235	0.99995	no

ENSG00000180879	ENSG00000180879	SSR4	X:153051220-153063960	q1	q2	OK	10.6719	42.3421	1.98827	2.30571	0.0052	0.99995	no

ENSG00000180881	ENSG00000180881	CAPS2	12:75628519-75784708	q1	q2	OK	1.18181	0.447693	-1.40042	-0.917004	0.04635	0.99995	no

ENSG00000180884	ENSG00000180884	ZNF792	19:35447257-35454953	q1	q2	OK	1.66965	1.65778	-0.0102994	-0.0143538	0.9838	0.99995	no

ENSG00000180891	ENSG00000180891	CUEDC1	17:55938603-56032684	q1	q2	OK	37.6886	36.6432	-0.0405842	-0.0686685	0.9063	0.99995	no

ENSG00000180900	ENSG00000180900	SCRIB	8:144873089-144897549	q1	q2	OK	12.1721	5.60525	-1.11873	-1.05236	0.0865	0.99995	no

ENSG00000180901	ENSG00000180901	KCTD2	17:73028669-73061984	q1	q2	OK	18.4252	17.7402	-0.0546566	-0.0850176	0.89985	0.99995	no

ENSG00000180902	ENSG00000180902	D2HGDH	2:242673993-242708231	q1	q2	OK	5.99058	6.02475	0.0082047	0.011685	0.98365	0.99995	no

ENSG00000180909	ENSG00000180909	OR52B1P	11:6128913-6206806	q1	q2	NOTEST	0	0	0	0	1	1	no

ENSG00000180910	ENSG00000180910	TTTY11	Y:8651350-8685423	q1	q2	NOTEST	0	0	0	0	1	1	no

ENSG00000180913	ENSG00000180913	OR56B3P	11:6128913-6206806	q1	q2	NOTEST	0	0	0	0	1	1	no

ENSG00000180914	ENSG00000180914	OXTR	3:8661085-9005457	q1	q2	OK	1.9691	1.90867	-0.044964	-0.0285722	0.95665	0.99995	no

ENSG00000180917	ENSG00000180917	CMTR2	16:71315291-71323618	q1	q2	OK	19.3473	21.5613	0.156316	0.339012	0.59975	0.99995	no

ENSG00000180919	ENSG00000180919	OR56B4	11:6128913-6206806	q1	q2	NOTEST	0	0.0258596	inf	0	1	1	no

ENSG00000180921	ENSG00000180921	FAM83H	8:144806102-144815971	q1	q2	NOTEST	0.0132863	0	#NAME?	0	1	1	no

ENSG00000180926	ENSG00000180926	OR7E18P	19:9371690-9372392	q1	q2	NOTEST	0	0	0	0	1	1	no

ENSG00000180929	ENSG00000180929	GPR62	3:51989329-52023213	q1	q2	NOTEST	0.162985	0.147818	-0.140918	0	1	1	no

ENSG00000180934	ENSG00000180934	OR56A1	11:6047900-6048971	q1	q2	NOTEST	0	0	0	0	1	1	no

ENSG00000180938	ENSG00000180938	ZNF572	8:125985539-125991631	q1	q2	OK	0.726037	0.837214	0.205553	0.236692	0.72095	0.99995	no

ENSG00000180953	ENSG00000180953	ST20	15:80125926-80217194	q1	q2	OK	6.22065	5.64843	-0.139214	-0.110556	0.91275	0.99995	no

ENSG00000180957	ENSG00000180957	PITPNB	22:28247656-29075853	q1	q2	OK	40.666	46.3581	0.188998	0.143019	0.87255	0.99995	no

ENSG00000180964	ENSG00000180964	TCEAL8	X:102507922-102510131	q1	q2	OK	60.5391	64.6637	0.0950893	0.219789	0.74995	0.99995	no

ENSG00000180974	ENSG00000180974	OR52E4	11:5684424-5959849	q1	q2	NOTEST	0	0	0	0	1	1	no

ENSG00000180979	ENSG00000180979	LRRC57	15:42783430-42841000	q1	q2	OK	8.50221	9.47384	0.156113	0.182138	0.79145	0.99995	no

ENSG00000180987	ENSG00000180987	AC123768.1	15:32885656-32896179	q1	q2	NOTEST	0.320076	0	#NAME?	0	1	1	no

ENSG00000180988	ENSG00000180988	OR52N2	11:5684424-5959849	q1	q2	NOTEST	0	0	0	0	1	1	no

ENSG00000180992	ENSG00000180992	MRPL14	6:44081193-44123256	q1	q2	OK	35.5222	34.0212	-0.0622842	-0.094558	0.8936	0.99995	no

ENSG00000180998	ENSG00000180998	GPR137C	14:53019865-53104431	q1	q2	OK	6.07184	6.05118	-0.00491729	-0.00941654	0.9886	0.99995	no

ENSG00000180999	ENSG00000180999	C1orf105	1:171810620-172437971	q1	q2	NOTEST	0	0.0337379	inf	0	1	1	no

ENSG00000181001	ENSG00000181001	OR52N1	11:5684424-5959849	q1	q2	NOTEST	0	0	0	0	1	1	no

ENSG00000181004	ENSG00000181004	BBS12	4:123653856-123666098	q1	q2	OK	2.45184	2.52778	0.044005	0.0660187	0.9239	0.99995	no

ENSG00000181007	ENSG00000181007	ZFP82	19:36874021-36909558	q1	q2	OK	9.25465	11.9104	0.363966	0.587034	0.3905	0.99995	no

ENSG00000181009	ENSG00000181009	OR52N5	11:5684424-5959849	q1	q2	NOTEST	0	0	0	0	1	1	no

ENSG00000181013	ENSG00000181013	C17orf47	17:56597197-56769416	q1	q2	NOTEST	0	0	0	0	1	1	no

ENSG00000181016	ENSG00000181016	LSMEM1	7:112063022-112131117	q1	q2	OK	3.25282	1.47349	-1.14245	-0.334776	0.43835	0.99995	no

ENSG00000181017	ENSG00000181017	OR56B2P	11:5684424-5959849	q1	q2	NOTEST	0	0	0	0	1	1	no

ENSG00000181019	ENSG00000181019	NQO1	16:69740898-69760854	q1	q2	OK	57.7869	44.8203	-0.366591	-0.662391	0.3415	0.99995	no

ENSG00000181023	ENSG00000181023	OR56B1	11:5684424-5959849	q1	q2	NOTEST	0	0	0	0	1	1	no

ENSG00000181026	ENSG00000181026	AEN	15:89164526-89175513	q1	q2	OK	27.9485	24.3147	-0.200942	-0.401148	0.49185	0.99995	no

ENSG00000181027	ENSG00000181027	FKRP	19:47222763-47291851	q1	q2	OK	20.5567	18.2933	-0.16829	-0.10043	0.77265	0.99995	no

ENSG00000181029	ENSG00000181029	TRAPPC5	19:7741513-7747744	q1	q2	OK	35.1575	28.1031	-0.3231	-0.562707	0.4254	0.99995	no

ENSG00000181031	ENSG00000181031	RPH3AL	17:62292-236045	q1	q2	NOTEST	0	0	0	0	1	1	no

ENSG00000181035	ENSG00000181035	SLC25A42	19:19174807-19223697	q1	q2	OK	4.77209	4.36654	-0.128131	-0.12622	0.78315	0.99995	no

ENSG00000181036	ENSG00000181036	FCRL6	1:159770300-159786041	q1	q2	NOTEST	0	0	0	0	1	1	no

ENSG00000181038	ENSG00000181038	METTL23	17:74722911-74777531	q1	q2	OK	21.393	20.3409	-0.0727585	-0.0275596	0.93065	0.99995	no

ENSG00000181039	ENSG00000181039	ANKRD34A	1:145470507-145475646	q1	q2	NOTEST	0	0.0072362	inf	0	1	1	no

ENSG00000181045	ENSG00000181045	SLC26A11	17:78143790-78227299	q1	q2	OK	9.97732	9.10182	-0.132497	-0.147727	0.76775	0.99995	no

ENSG00000181061	ENSG00000181061	HIGD1A	3:42734154-42846023	q1	q2	OK	108.25	115.487	0.0933576	0.222024	0.74885	0.99995	no

ENSG00000181072	ENSG00000181072	CHRM2	7:136370305-136866854	q1	q2	NOTEST	0.124495	0.0565561	-1.13833	0	1	1	no

ENSG00000181074	ENSG00000181074	OR52N4	11:5684424-5959849	q1	q2	NOTEST	0.0299439	0	#NAME?	0	1	1	no

ENSG00000181085	ENSG00000181085	MAPK15	8:144798428-144804628	q1	q2	NOTEST	0.322455	0.0360108	-3.1626	0	1	1	no

ENSG00000181090	ENSG00000181090	EHMT1	9:140513443-141019076	q1	q2	OK	45.9347	45.3284	-0.0191704	-0.0238996	0.9583	0.99995	no

ENSG00000181092	ENSG00000181092	ADIPOQ	3:186560462-186576252	q1	q2	NOTEST	0	0	0	0	1	1	no

ENSG00000181097	ENSG00000181097	RP11-429J17.2	8:144766621-144796068	q1	q2	NOTEST	0	0.0130624	inf	0	1	1	no

ENSG00000181101	ENSG00000181101	SDCCAG3P2	1:175013761-175014784	q1	q2	NOTEST	0.274034	0.415039	0.598889	0	1	1	no

ENSG00000181104	ENSG00000181104	F2R	5:75699073-76031606	q1	q2	OK	43.3527	44.5004	0.0376958	0.0727792	0.9144	0.99995	no

ENSG00000181109	ENSG00000181109	OR52P1P	11:5684424-5959849	q1	q2	NOTEST	0	0	0	0	1	1	no

ENSG00000181123	ENSG00000181123	RP4-539M6.14	22:30879340-30901698	q1	q2	NOTEST	0	0	0	0	1	1	no

ENSG00000181126	ENSG00000181126	HLA-V	6:29758730-29765588	q1	q2	NOTEST	0	0	0	0	1	1	no

ENSG00000181135	ENSG00000181135	ZNF707	8:144766621-144796068	q1	q2	OK	1.34634	0.49963	-1.43011	-0.776579	0.0106	0.99995	no

ENSG00000181143	ENSG00000181143	MUC16	19:8959519-9092018	q1	q2	NOTEST	0.000573143	0.000525447	-0.125352	0	1	1	no

ENSG00000181163	ENSG00000181163	NPM1	5:170814119-170838141	q1	q2	OK	1287.15	1357.1	0.0763462	0.0809647	0.86135	0.99995	no

ENSG00000181171	ENSG00000181171	FER1L6-AS1	8:124864226-125183763	q1	q2	NOTEST	0	0	0	0	1	1	no

ENSG00000181191	ENSG00000181191	PJA1	X:68380693-68385636	q1	q2	OK	39.8134	42.5411	0.0956024	0.217972	0.745	0.99995	no

ENSG00000181192	ENSG00000181192	DHTKD1	10:12110970-12165224	q1	q2	OK	11.4731	11.0988	-0.0478391	-0.0650321	0.87745	0.99995	no

ENSG00000181195	ENSG00000181195	PENK	8:57349232-57472382	q1	q2	OK	13.8289	16.3132	0.23835	0.339687	0.63395	0.99995	no

ENSG00000181201	ENSG00000181201	HIST3H2BA	1:228651803-228652327	q1	q2	NOTEST	0	0.223338	inf	0	1	1	no

ENSG00000181211	ENSG00000181211	HECW1-IT1	7:43152197-43605600	q1	q2	NOTEST	0	0	0	0	1	1	no

ENSG00000181214	ENSG00000181214	OR8G2P	11:124095436-124096365	q1	q2	NOTEST	0	0	0	0	1	1	no

ENSG00000181215	ENSG00000181215	C4orf50	4:5958844-5991545	q1	q2	NOTEST	0.0309276	0.0724721	1.22853	0	1	1	no

ENSG00000181218	ENSG00000181218	HIST3H2A	1:228645064-228645560	q1	q2	OK	8.98767	7.93662	-0.179423	-0.210779	0.7612	0.99995	no

ENSG00000181220	ENSG00000181220	ZNF746	7:149169884-149194908	q1	q2	OK	9.05775	8.91641	-0.0226893	-0.0418655	0.9461	0.99995	no

ENSG00000181222	ENSG00000181222	POLR2A	17:7387684-7417933	q1	q2	OK	67.0352	67.2622	0.00487749	0.00614873	0.98555	0.99995	no

ENSG00000181227	ENSG00000181227	RP4-682C21.2	1:76190035-76260764	q1	q2	OK	1.39679	1.36056	-0.0379173	-0.00978403	0.98505	0.99995	no

ENSG00000181234	ENSG00000181234	TMEM132C	12:128751947-129192460	q1	q2	OK	0.572307	0.609243	0.0902267	0.0951447	0.89015	0.99995	no

ENSG00000181240	ENSG00000181240	SLC25A41	19:6426047-6433790	q1	q2	NOTEST	0.165234	0.301276	0.866579	0	1	1	no

ENSG00000181260	ENSG00000181260	MTHFD2P7	3:179182133-179183116	q1	q2	NOTEST	0.0303092	0.0906121	1.57995	0	1	1	no

ENSG00000181264	ENSG00000181264	TMEM136	11:120195837-120204391	q1	q2	OK	12.2034	12.4387	0.0275455	0.0557386	0.93285	0.99995	no

ENSG00000181273	ENSG00000181273	OR5AK2	11:56756346-56757342	q1	q2	NOTEST	0.183894	0.0582179	-1.65934	0	1	1	no

ENSG00000181274	ENSG00000181274	FRAT2	10:99092254-99102770	q1	q2	OK	5.66031	4.00934	-0.497516	-0.788587	0.2547	0.99995	no

ENSG00000181282	ENSG00000181282	OR5AK3P	11:56738324-56739621	q1	q2	NOTEST	0	0	0	0	1	1	no

ENSG00000181284	ENSG00000181284	TMEM102	17:7323421-7361026	q1	q2	OK	0.605639	0.700241	0.209393	0.0892654	0.92305	0.99995	no

ENSG00000181291	ENSG00000181291	TMEM132E	17:32907767-32966337	q1	q2	OK	0.523034	0.473794	-0.142643	-0.134464	0.8202	0.99995	no

ENSG00000181296	ENSG00000181296	OR5G1P	11:56542736-56543682	q1	q2	NOTEST	0	0	0	0	1	1	no

ENSG00000181315	ENSG00000181315	ZNF322	6:26634610-26659980	q1	q2	NOTEST	0.328467	0.469405	0.515084	0	1	1	no

ENSG00000181322	ENSG00000181322	NME9	3:137906108-138048728	q1	q2	OK	1.19405	1.22506	0.0369944	0.0139388	0.97265	0.99995	no

ENSG00000181323	ENSG00000181323	SPEM1	17:7323421-7361026	q1	q2	NOTEST	0.156844	0.122191	-0.360187	0	1	1	no

ENSG00000181325	ENSG00000181325	OR9G3P	11:56507654-56508572	q1	q2	NOTEST	0	0	0	0	1	1	no

ENSG00000181333	ENSG00000181333	HEPHL1	11:93754526-93846917	q1	q2	NOTEST	0.0427501	0.0239953	-0.83318	0	1	1	no

ENSG00000181350	ENSG00000181350	FAM211A	17:16342135-16395467	q1	q2	OK	4.93833	4.46053	-0.146807	-0.0827309	0.8768	0.99995	no

ENSG00000181355	ENSG00000181355	OFCC1	6:9596342-10211841	q1	q2	NOTEST	0	0	0	0	1	1	no

ENSG00000181358	ENSG00000181358	CTAGE10P	13:50464726-50467134	q1	q2	NOTEST	0.0107471	0.0104191	-0.0447095	0	1	1	no

ENSG00000181359	ENSG00000181359	HSP90AA6P	4:171502620-171526601	q1	q2	NOTEST	0	0.479029	inf	0	1	1	no

ENSG00000181371	ENSG00000181371	OR5M8	11:56257824-56258871	q1	q2	NOTEST	0	0	0	0	1	1	no

ENSG00000181374	ENSG00000181374	CCL13	17:32683470-32685629	q1	q2	NOTEST	0	0	0	0	1	1	no

ENSG00000181378	ENSG00000181378	CCDC108	2:219866936-219906249	q1	q2	OK	0.463927	0.593483	0.355307	0.24364	0.59505	0.99995	no

ENSG00000181381	ENSG00000181381	DDX60L	4:169277885-169931426	q1	q2	OK	1.0551	1.04319	-0.0163835	-0.00343333	0.98625	0.99995	no

ENSG00000181392	ENSG00000181392	SYNE4	19:36494208-36505141	q1	q2	OK	0.322419	0.517005	0.68124	0.252475	0.61015	0.99995	no

ENSG00000181395	ENSG00000181395	OR5AL1	11:56180171-56181156	q1	q2	NOTEST	0	0	0	0	1	1	no

ENSG00000181396	ENSG00000181396	OGFOD3	17:80347098-80408705	q1	q2	OK	11.2109	10.2423	-0.130365	-0.11422	0.82625	0.99995	no

ENSG00000181404	ENSG00000181404	XXyac-YRM2039.2	9:11986-30891	q1	q2	OK	5.10024	4.25195	-0.262441	-0.373764	0.59455	0.99995	no

ENSG00000181408	ENSG00000181408	UTS2R	17:80332152-80333462	q1	q2	NOTEST	0.0641377	0.0429985	-0.576886	0	1	1	no

ENSG00000181409	ENSG00000181409	AATK	17:79008947-79156964	q1	q2	OK	1.07201	1.14827	0.0991517	0.0631082	0.9069	0.99995	no

ENSG00000181418	ENSG00000181418	DDN	12:49388931-49453557	q1	q2	NOTEST	0.279873	0.171678	-0.705063	0	1	1	no

ENSG00000181433	ENSG00000181433	SAGE1	X:134971373-134995220	q1	q2	NOTEST	0	0	0	0	1	1	no

ENSG00000181444	ENSG00000181444	ZNF467	7:149461270-149470568	q1	q2	OK	7.95005	7.42818	-0.0979553	-0.144164	0.8331	0.99995	no

ENSG00000181449	ENSG00000181449	SOX2	3:180701496-181554668	q1	q2	OK	139.286	127.063	-0.132502	-0.253334	0.71995	0.99995	no

ENSG00000181450	ENSG00000181450	ZNF678	1:227751243-227865144	q1	q2	OK	6.96316	7.96351	0.193663	0.288217	0.6303	0.99995	no

ENSG00000181458	ENSG00000181458	TMEM45A	3:100211462-100296288	q1	q2	OK	8.758	8.00571	-0.129572	-0.169818	0.76895	0.99995	no

ENSG00000181464	ENSG00000181464	CDRT1	17:15468796-15587625	q1	q2	NOTEST	0	0	0	0	1	1	no

ENSG00000181467	ENSG00000181467	RAP2B	3:152880028-152886265	q1	q2	OK	7.4321	6.88469	-0.110378	-0.244682	0.72775	0.99995	no

ENSG00000181472	ENSG00000181472	ZBTB2	6:151685251-151712683	q1	q2	OK	9.02104	10.3271	0.195067	0.39443	0.57915	0.99995	no

ENSG00000181481	ENSG00000181481	RNF135	17:29295802-29326929	q1	q2	OK	1.44068	1.32438	-0.121432	-0.119332	0.8509	0.99995	no

ENSG00000181495	ENSG00000181495	AC026703.1	5:32689175-32791819	q1	q2	OK	0.430393	0.567301	0.398461	0.228233	0.74045	0.99995	no

ENSG00000181499	ENSG00000181499	OR6T1	11:123813491-123814580	q1	q2	NOTEST	0	0	0	0	1	1	no

ENSG00000181511	ENSG00000181511	RP4-655L22.4	X:119866947-119867250	q1	q2	NOTEST	0	0	0	0	1	1	no

ENSG00000181513	ENSG00000181513	ACBD4	17:43100707-43221548	q1	q2	OK	1.12519	0.743056	-0.598628	-0.107777	0.44195	0.99995	no

ENSG00000181514	ENSG00000181514	RP3-474G15.1	6:73308178-73309362	q1	q2	NOTEST	0	0	0	0	1	1	no

ENSG00000181518	ENSG00000181518	OR8D4	11:123777108-123778183	q1	q2	NOTEST	0	0	0	0	1	1	no

ENSG00000181523	ENSG00000181523	SGSH	17:78143790-78227299	q1	q2	OK	13.5942	11.3262	-0.263323	-0.269476	0.5708	0.99995	no

ENSG00000181524	ENSG00000181524	RPL24P4	6:42924082-42924503	q1	q2	OK	81.1589	80.038	-0.020063	-0.0369394	0.9583	0.99995	no

ENSG00000181541	ENSG00000181541	MAB21L2	4:151185593-151936879	q1	q2	NOTEST	0.314582	0.261278	-0.267847	0	1	1	no

ENSG00000181544	ENSG00000181544	FANCB	X:14861528-14891191	q1	q2	OK	3.0566	3.53843	0.211183	0.23903	0.66215	0.99995	no

ENSG00000181552	ENSG00000181552	EDDM3B	14:21236585-21239107	q1	q2	NOTEST	0.0701191	0.0353242	-0.989151	0	1	1	no

ENSG00000181555	ENSG00000181555	SETD2	3:47057918-47205457	q1	q2	OK	21.5894	21.9803	0.0258932	0.0467325	0.92445	0.99995	no

ENSG00000181562	ENSG00000181562	EDDM3A	14:21214050-21216539	q1	q2	NOTEST	0.0344802	0	#NAME?	0	1	1	no

ENSG00000181577	ENSG00000181577	C6orf223	6:43963459-44045689	q1	q2	NOTEST	0.0572139	0.0543454	-0.0742086	0	1	1	no

ENSG00000181585	ENSG00000181585	TMIE	3:46742822-46752376	q1	q2	NOTEST	0.0446357	0.0151818	-1.55586	0	1	1	no

ENSG00000181588	ENSG00000181588	MEX3D	19:1554667-1568057	q1	q2	OK	22.9626	23.7268	0.0472339	0.108329	0.8785	0.99995	no

ENSG00000181609	ENSG00000181609	OR52D1	11:5274419-5667019	q1	q2	NOTEST	0	0	0	0	1	1	no

ENSG00000181610	ENSG00000181610	MRPS23	17:55916841-55927417	q1	q2	OK	43.9976	45.6394	0.0528547	0.111118	0.87125	0.99995	no

ENSG00000181616	ENSG00000181616	OR52H1	11:5274419-5667019	q1	q2	NOTEST	0	0	0	0	1	1	no

ENSG00000181617	ENSG00000181617	FDCSP	4:71091787-71100969	q1	q2	NOTEST	0	0	0	0	1	1	no

ENSG00000181619	ENSG00000181619	GPR135	14:59895739-60043549	q1	q2	OK	1.59929	1.44663	-0.14474	-0.0823878	0.8994	0.99995	no

ENSG00000181625	ENSG00000181625	SLX1B	16:29262828-29519817	q1	q2	OK	0.412828	0.511512	0.309225	0.0866234	0.82415	0.99995	no

ENSG00000181626	ENSG00000181626	ANKRD62	18:12093851-12129748	q1	q2	NOTEST	0.00872698	0	#NAME?	0	1	1	no

ENSG00000181631	ENSG00000181631	P2RY13	3:150803483-151176497	q1	q2	NOTEST	0	0	0	0	1	1	no

ENSG00000181634	ENSG00000181634	TNFSF15	9:117546914-117568406	q1	q2	NOTEST	0.102814	0.0579409	-0.827378	0	1	1	no

ENSG00000181638	ENSG00000181638	ZFP41	8:144328990-144359101	q1	q2	OK	5.59104	5.57545	-0.00402824	-0.00318773	0.99465	0.99995	no

ENSG00000181649	ENSG00000181649	PHLDA2	11:2949502-2950685	q1	q2	OK	1.7638	0.8366	-1.07608	-0.925498	0.1921	0.99995	no

ENSG00000181652	ENSG00000181652	ATG9B	7:150688082-150721586	q1	q2	OK	0.724782	0.932215	0.363116	0.182617	0.69195	0.99995	no

ENSG00000181656	ENSG00000181656	GPR88	1:101003692-101007574	q1	q2	NOTEST	0.473747	0.15929	-1.57246	0	1	1	no

ENSG00000181666	ENSG00000181666	HKR1	19:37803738-37860267	q1	q2	OK	25.7458	23.8533	-0.110145	-0.18137	0.7333	0.99995	no

ENSG00000181689	ENSG00000181689	OR8K3	11:56085782-56086721	q1	q2	NOTEST	0	0	0	0	1	1	no

ENSG00000181690	ENSG00000181690	PLAG1	8:57073462-57123883	q1	q2	OK	7.72097	7.32452	-0.0760473	-0.146545	0.80655	0.99995	no

ENSG00000181693	ENSG00000181693	OR8H1	11:56057528-56058566	q1	q2	NOTEST	0	0	0	0	1	1	no

ENSG00000181698	ENSG00000181698	OR5T1	11:56043028-56044146	q1	q2	NOTEST	0	0	0	0	1	1	no

ENSG00000181704	ENSG00000181704	YIPF6	X:67718164-67757127	q1	q2	OK	15.1904	16.9326	0.156642	0.261916	0.60735	0.99995	no

ENSG00000181705	ENSG00000181705	RP11-11L12.2	6:81208912-81209109	q1	q2	NOTEST	0	0	0	0	1	1	no

ENSG00000181718	ENSG00000181718	OR5T2	11:55999489-56000737	q1	q2	NOTEST	0	0	0	0	1	1	no

ENSG00000181722	ENSG00000181722	ZBTB20	3:114056940-114866118	q1	q2	OK	2.72533	2.50601	-0.121039	-0.0942383	0.8409	0.99995	no

ENSG00000181733	ENSG00000181733	OR2Z1	19:8841315-8842371	q1	q2	NOTEST	0	0	0	0	1	1	no

ENSG00000181741	ENSG00000181741	FDX1P1	20:32951040-33099198	q1	q2	OK	0.135191	0.810965	2.58464	0.226264	0.38245	0.99995	no

ENSG00000181744	ENSG00000181744	C3orf58	3:143690639-143767561	q1	q2	OK	14.2246	12.2715	-0.213077	-0.449033	0.49355	0.99995	no

ENSG00000181751	ENSG00000181751	C5orf30	5:102594402-102614361	q1	q2	NOTEST	0.197348	0.150942	-0.386744	0	1	1	no

ENSG00000181752	ENSG00000181752	OR8K5	11:55926869-55927793	q1	q2	NOTEST	0	0	0	0	1	1	no

ENSG00000181754	ENSG00000181754	AMIGO1	1:110046796-110052360	q1	q2	OK	2.37408	2.01711	-0.235081	-0.379079	0.577	0.99995	no

ENSG00000181761	ENSG00000181761	OR8H3	11:55889848-55890787	q1	q2	NOTEST	0	0	0	0	1	1	no

ENSG00000181767	ENSG00000181767	OR8H2	11:55872518-55873457	q1	q2	NOTEST	0	0	0	0	1	1	no

ENSG00000181773	ENSG00000181773	GPR3	1:27719147-27722318	q1	q2	OK	1.52279	2.02631	0.412138	0.504655	0.4797	0.99995	no

ENSG00000181778	ENSG00000181778	TMEM252	9:71151495-71155783	q1	q2	NOTEST	0.0706437	0.0471316	-0.583865	0	1	1	no

ENSG00000181780	ENSG00000181780	OR5J1P	11:55838505-55839525	q1	q2	NOTEST	0	0	0	0	1	1	no

ENSG00000181781	ENSG00000181781	ODF3L2	19:463345-474983	q1	q2	NOTEST	0.116644	0.118192	0.0190177	0	1	1	no

ENSG00000181785	ENSG00000181785	OR5AS1	11:55797894-55798869	q1	q2	NOTEST	0	0	0	0	1	1	no

ENSG00000181786	ENSG00000181786	ACTL9	19:8807750-8809172	q1	q2	NOTEST	0	0	0	0	1	1	no

ENSG00000181788	ENSG00000181788	SIAH2	3:150458913-150481264	q1	q2	OK	8.47156	7.46105	-0.183249	-0.33096	0.6233	0.99995	no

ENSG00000181789	ENSG00000181789	COPG1	3:128968448-128996614	q1	q2	OK	69.7624	67.1583	-0.0548844	-0.0792524	0.90555	0.99995	no

ENSG00000181790	ENSG00000181790	BAI1	8:143530790-143626370	q1	q2	OK	10.214	10.2783	0.00906477	0.00911051	0.98955	0.99995	no

ENSG00000181791	ENSG00000181791	AC009041.1	16:1114092-1131454	q1	q2	NOTEST	0	0	0	0	1	1	no

ENSG00000181798	ENSG00000181798	LINC00471	2:232373136-232379050	q1	q2	NOTEST	0.469078	0.402669	-0.220234	0	1	1	no

ENSG00000181800	ENSG00000181800	CELF2-AS1	10:11047258-11378674	q1	q2	NOTEST	0.228267	0.234894	0.0412842	0	1	1	no

ENSG00000181803	ENSG00000181803	OR6S1	14:21108854-21109850	q1	q2	NOTEST	0	0	0	0	1	1	no

ENSG00000181804	ENSG00000181804	SLC9A9	3:142984063-143567373	q1	q2	OK	1.07621	0.850558	-0.339479	-0.280293	0.5759	0.99995	no

ENSG00000181817	ENSG00000181817	LSM10	1:36856838-36863493	q1	q2	OK	26.2383	22.4048	-0.227869	-0.40505	0.5521	0.99995	no

ENSG00000181819	ENSG00000181819	KCTD9P2	X:103810995-105011822	q1	q2	NOTEST	0.241691	0.379259	0.65002	0	1	1	no

ENSG00000181826	ENSG00000181826	RELL1	4:37455562-37687998	q1	q2	OK	3.78702	3.90325	0.043616	0.0510505	0.93755	0.99995	no

ENSG00000181827	ENSG00000181827	RFX7	15:56379477-56535483	q1	q2	OK	18.1882	19.5917	0.107235	0.261645	0.71085	0.99995	no

ENSG00000181830	ENSG00000181830	SLC35C1	11:45825622-45834566	q1	q2	OK	5.71385	5.82252	0.0271807	0.0497685	0.9427	0.99995	no

ENSG00000181837	ENSG00000181837	OR5D17P	11:55522451-55523433	q1	q2	NOTEST	0	0	0	0	1	1	no

ENSG00000181847	ENSG00000181847	TIGIT	3:113995759-114029135	q1	q2	NOTEST	0.0310364	0.00830624	-1.9017	0	1	1	no

ENSG00000181852	ENSG00000181852	RNF41	12:56598284-56615717	q1	q2	OK	22.1524	20.3075	-0.125451	-0.237902	0.70315	0.99995	no

ENSG00000181856	ENSG00000181856	SLC2A4	17:7184985-7197934	q1	q2	NOTEST	0.339435	0.253571	-0.420747	0	1	1	no

ENSG00000181867	ENSG00000181867	FTMT	5:121187649-121188519	q1	q2	NOTEST	0	0	0	0	1	1	no

ENSG00000181873	ENSG00000181873	IBA57	1:228353515-228369958	q1	q2	OK	1.95849	1.65004	-0.24724	-0.415852	0.5462	0.99995	no

ENSG00000181885	ENSG00000181885	CLDN7	17:7143332-7167302	q1	q2	NOTEST	0.109578	0.164264	0.58406	0	1	1	no

ENSG00000181894	ENSG00000181894	ZNF329	19:58637618-58666477	q1	q2	OK	7.4295	6.77043	-0.134018	-0.239896	0.70975	0.99995	no

ENSG00000181896	ENSG00000181896	ZNF101	19:19779604-19794318	q1	q2	OK	7.00108	8.14812	0.21889	0.263139	0.67495	0.99995	no

ENSG00000181903	ENSG00000181903	OR4C6	11:55432613-55433671	q1	q2	NOTEST	0	0	0	0	1	1	no

ENSG00000181904	ENSG00000181904	C5orf24	5:134094468-134195427	q1	q2	OK	31.6704	34.7547	0.134073	0.172415	0.7691	0.99995	no

ENSG00000181908	ENSG00000181908	AP003774.4	11:64216545-64219126	q1	q2	NOTEST	0	0.0661533	inf	0	1	1	no

ENSG00000181915	ENSG00000181915	ADO	10:64553321-64568238	q1	q2	OK	28.9452	29.7382	0.0389929	0.0935573	0.89205	0.99995	no

ENSG00000181924	ENSG00000181924	COA4	11:73583711-73638790	q1	q2	OK	34.8146	33.5212	-0.0546198	-0.0878513	0.89985	0.99995	no

ENSG00000181927	ENSG00000181927	OR4P4	11:55405833-55406772	q1	q2	NOTEST	0	0	0	0	1	1	no

ENSG00000181929	ENSG00000181929	PRKAG1	12:49388931-49453557	q1	q2	OK	39.0694	34.5308	-0.178157	-0.147615	0.81875	0.99995	no

ENSG00000181935	ENSG00000181935	OR4C16	11:55339603-55340536	q1	q2	NOTEST	0	0	0	0	1	1	no

ENSG00000181938	ENSG00000181938	GINS3	16:58328983-58440048	q1	q2	OK	16.1979	17.1596	0.0832121	0.166175	0.801	0.99995	no

ENSG00000181939	ENSG00000181939	OR4C15	11:55321782-55322895	q1	q2	NOTEST	0	0	0	0	1	1	no

ENSG00000181943	ENSG00000181943	OR4A21P	11:55258627-55259618	q1	q2	NOTEST	0	0	0	0	1	1	no

ENSG00000181950	ENSG00000181950	OR4A13P	11:55234246-55235188	q1	q2	NOTEST	0	0	0	0	1	1	no

ENSG00000181958	ENSG00000181958	OR4A15	11:55135359-55136394	q1	q2	NOTEST	0	0	0	0	1	1	no

ENSG00000181961	ENSG00000181961	OR4A16	11:55110626-55111707	q1	q2	OK	0.615147	0.65548	0.0916214	0.0817682	0.90605	0.99995	no

ENSG00000181963	ENSG00000181963	OR52K2	11:4470524-4471591	q1	q2	NOTEST	0	0	0	0	1	1	no

ENSG00000181965	ENSG00000181965	NEUROG1	5:134869990-134871639	q1	q2	OK	2.84498	2.49591	-0.188853	-0.239787	0.7272	0.99995	no

ENSG00000181977	ENSG00000181977	PYY3	X:49920806-49921019	q1	q2	NOTEST	0	0	0	0	1	1	no

ENSG00000181982	ENSG00000181982	CCDC149	4:24807738-24981826	q1	q2	OK	3.95422	3.93954	-0.00536536	-0.00629019	0.9895	0.99995	no

ENSG00000181984	ENSG00000181984	GOLGA8CP	15:20767699-20781019	q1	q2	NOTEST	0	0	0	0	1	1	no

ENSG00000181991	ENSG00000181991	MRPS11	15:89002706-89089906	q1	q2	OK	28.1598	31.6109	0.166786	0.195762	0.74175	0.99995	no

ENSG00000181995	ENSG00000181995	LINC00301	11:60383208-60454622	q1	q2	NOTEST	0	0	0	0	1	1	no

ENSG00000181997	ENSG00000181997	AQP7P2	9:69633977-69650004	q1	q2	NOTEST	0	0.113587	inf	0	1	1	no

ENSG00000182000	ENSG00000182000	RP11-651P23.2	3:149478891-149942977	q1	q2	NOTEST	0	0.118668	inf	0	1	1	no

ENSG00000182004	ENSG00000182004	SNRPE	1:203830730-203839678	q1	q2	OK	202.557	212.716	0.0705944	0.120835	0.85245	0.99995	no

ENSG00000182010	ENSG00000182010	RTKN2	10:63942793-64028466	q1	q2	OK	16.3794	17.8074	0.120591	0.23594	0.7317	0.99995	no

ENSG00000182013	ENSG00000182013	PNMAL1	19:46969747-46974820	q1	q2	OK	24.6361	24.8856	0.0145351	0.0351664	0.9609	0.99995	no

ENSG00000182021	ENSG00000182021	RP11-381O7.3	9:67017399-67032072	q1	q2	NOTEST	0.0698945	0.0395478	-0.821581	0	1	1	no

ENSG00000182022	ENSG00000182022	CHST15	10:125767183-125853206	q1	q2	OK	4.83975	4.54514	-0.090609	-0.128151	0.82255	0.99995	no

ENSG00000182035	ENSG00000182035	ADIG	20:37209837-37217106	q1	q2	NOTEST	0	0.0676643	inf	0	1	1	no

ENSG00000182040	ENSG00000182040	USH1G	17:72912175-72919351	q1	q2	NOTEST	0.0551936	0	#NAME?	0	1	1	no

ENSG00000182048	ENSG00000182048	TRPC2	11:3631130-3658789	q1	q2	NOTEST	0	0.0233772	inf	0	1	1	no

ENSG00000182050	ENSG00000182050	MGAT4C	12:86372515-87232775	q1	q2	OK	1.91237	1.7187	-0.154039	-0.140475	0.81495	0.99995	no

ENSG00000182053	ENSG00000182053	TRIM49B	11:49050503-49059579	q1	q2	OK	0.581706	0.479032	-0.280167	-0.254661	0.70665	0.99995	no

ENSG00000182054	ENSG00000182054	IDH2	15:90626276-90645736	q1	q2	OK	109.523	98.8397	-0.148068	-0.345964	0.62785	0.99995	no

ENSG00000182057	ENSG00000182057	Z83851.3	22:42556018-42739622	q1	q2	NOTEST	0.258496	0.400183	0.630521	0	1	1	no

ENSG00000182070	ENSG00000182070	OR52A1	11:5172238-5207612	q1	q2	NOTEST	0	0	0	0	1	1	no

ENSG00000182077	ENSG00000182077	PTCHD3	10:27687115-27703297	q1	q2	NOTEST	0	0	0	0	1	1	no

ENSG00000182083	ENSG00000182083	OR6B2	2:240968840-240969906	q1	q2	NOTEST	0	0	0	0	1	1	no

ENSG00000182087	ENSG00000182087	TMEM259	19:999600-1021117	q1	q2	OK	69.0478	58.0286	-0.250831	-0.512613	0.41675	0.99995	no

ENSG00000182093	ENSG00000182093	WRB	21:40752169-40817731	q1	q2	OK	44.6203	45.8973	0.0407097	0.0860636	0.88835	0.99995	no

ENSG00000182095	ENSG00000182095	TNRC18	7:5313999-5465045	q1	q2	OK	34.7817	34.5125	-0.011209	-0.0111207	0.9838	0.99995	no

ENSG00000182103	ENSG00000182103	FAM181B	11:82443052-82444906	q1	q2	OK	1.64117	1.17145	-0.486427	-0.560707	0.411	0.99995	no

ENSG00000182107	ENSG00000182107	TMEM30B	14:61654276-62125414	q1	q2	NOTEST	0.0844226	0.0862022	0.0300947	0	1	1	no

ENSG00000182108	ENSG00000182108	DEXI	16:10958770-11036878	q1	q2	OK	15.3887	13.7179	-0.165817	-0.279371	0.6794	0.99995	no

ENSG00000182109	ENSG00000182109	RP11-69E11.4	1:39957317-40024379	q1	q2	OK	1.73085	1.25679	-0.461737	-0.335218	0.53575	0.99995	no

ENSG00000182111	ENSG00000182111	ZNF716	7:57509882-57533252	q1	q2	NOTEST	0	0	0	0	1	1	no

ENSG00000182117	ENSG00000182117	NOP10	15:34633916-34635378	q1	q2	OK	141.029	150.486	0.0936392	0.214564	0.75715	0.99995	no

ENSG00000182118	ENSG00000182118	FAM89A	1:231154703-231175992	q1	q2	OK	9.17116	8.90478	-0.0425238	-0.0692963	0.92125	0.99995	no

ENSG00000182132	ENSG00000182132	KCNIP1	5:169780490-170163636	q1	q2	OK	4.72596	3.15097	-0.584814	-0.795586	0.2354	0.99995	no

ENSG00000182134	ENSG00000182134	TDRKH	1:151728016-151766878	q1	q2	OK	9.12196	9.6571	0.0822471	0.0814891	0.88385	0.99995	no

ENSG00000182141	ENSG00000182141	ZNF708	19:21473961-21512227	q1	q2	OK	11.449	12.6767	0.146951	0.252539	0.64325	0.99995	no

ENSG00000182149	ENSG00000182149	IST1	16:71879893-71962913	q1	q2	OK	139.632	138.122	-0.0156843	-0.0232098	0.9683	0.99995	no

ENSG00000182150	ENSG00000182150	ERCC6L2	9:98521512-98776842	q1	q2	OK	26.4421	31.494	0.252237	0.334888	0.5792	0.99995	no

ENSG00000182154	ENSG00000182154	MRPL41	9:140445650-140447007	q1	q2	OK	20.6482	17.1017	-0.271878	-0.507186	0.4772	0.99995	no

ENSG00000182156	ENSG00000182156	ENPP7	17:77704680-77716021	q1	q2	NOTEST	0	0	0	0	1	1	no

ENSG00000182158	ENSG00000182158	CREB3L2	7:137559724-137686813	q1	q2	OK	9.19248	9.58241	0.0599345	0.126912	0.8347	0.99995	no

ENSG00000182162	ENSG00000182162	P2RY8	X:1581464-1656000	q1	q2	NOTEST	0.0237407	0.0176624	-0.426679	0	1	1	no

ENSG00000182165	ENSG00000182165	TP53TG1	7:86954540-86974831	q1	q2	OK	9.37254	8.78109	-0.0940407	-0.11133	0.8674	0.99995	no

ENSG00000182168	ENSG00000182168	UNC5C	4:96083654-96473608	q1	q2	OK	3.19383	3.09178	-0.0468481	-0.0826543	0.8984	0.99995	no

ENSG00000182170	ENSG00000182170	MRGPRG	11:3239173-3244361	q1	q2	NOTEST	0	0	0	0	1	1	no

ENSG00000182173	ENSG00000182173	TSEN54	17:73512140-73520820	q1	q2	OK	20.0568	17.788	-0.173184	-0.232995	0.7113	0.99995	no

ENSG00000182175	ENSG00000182175	RGMA	15:93586635-93632433	q1	q2	OK	77.4796	57.4324	-0.431951	-0.974974	0.1251	0.99995	no

ENSG00000182177	ENSG00000182177	ASB18	2:237073878-237203570	q1	q2	NOTEST	0.155101	0.233617	0.590932	0	1	1	no

ENSG00000182179	ENSG00000182179	UBA7	3:49842639-49851379	q1	q2	OK	5.33219	3.92176	-0.443227	-0.395552	0.48805	0.99995	no

ENSG00000182180	ENSG00000182180	MRPS16	10:74870216-75118617	q1	q2	OK	40.3685	42.6203	0.0783104	0.0840502	0.8723	0.99995	no

ENSG00000182183	ENSG00000182183	FAM159A	1:53099015-53135355	q1	q2	NOTEST	0	0	0	0	1	1	no

ENSG00000182185	ENSG00000182185	RAD51B	14:68286495-69196935	q1	q2	OK	4.73653	5.67841	0.261656	0.225304	0.632	0.99995	no

ENSG00000182187	ENSG00000182187	CRYGB	2:209007296-209010892	q1	q2	NOTEST	0	0	0	0	1	1	no

ENSG00000182195	ENSG00000182195	LDOC1	X:140269933-140271310	q1	q2	NOTEST	0	0	0	0	1	1	no

ENSG00000182196	ENSG00000182196	ARL6IP4	12:123405497-123467456	q1	q2	OK	96.0261	91.0106	-0.0773918	-0.14776	0.83665	0.99995	no

ENSG00000182197	ENSG00000182197	EXT1	8:118806728-119124092	q1	q2	OK	22.4533	25.0384	0.157212	0.189688	0.7812	0.99995	no

ENSG00000182199	ENSG00000182199	SHMT2	12:57623109-57634498	q1	q2	OK	110.525	101.904	-0.117168	-0.253413	0.6842	0.99995	no

ENSG00000182208	ENSG00000182208	MOB2	11:1490686-1522477	q1	q2	OK	12.6668	9.54392	-0.408398	-0.522803	0.43615	0.99995	no

ENSG00000182217	ENSG00000182217	HIST2H4B	1:149824180-149832714	q1	q2	NOTEST	0	0	0	0	1	1	no

ENSG00000182218	ENSG00000182218	HHIPL1	14:100111446-100146906	q1	q2	NOTEST	0.227678	0.193515	-0.234555	0	1	1	no

ENSG00000182220	ENSG00000182220	ATP6AP2	X:40440145-40465889	q1	q2	OK	54.8806	55.8881	0.0262435	0.059874	0.92565	0.99995	no

ENSG00000182223	ENSG00000182223	ZAR1	4:48492268-48496406	q1	q2	NOTEST	0.0983177	0.058667	-0.744903	0	1	1	no

ENSG00000182224	ENSG00000182224	CYB5D1	17:7760002-7816078	q1	q2	OK	9.34664	10.2673	0.13553	0.0663635	0.88215	0.99995	no

ENSG00000182230	ENSG00000182230	FAM153B	5:175476680-175559261	q1	q2	NOTEST	0	0	0	0	1	1	no

ENSG00000182240	ENSG00000182240	BACE2	21:42539727-42654445	q1	q2	OK	6.45469	6.5337	0.0175536	0.0219313	0.97135	0.99995	no

ENSG00000182242	ENSG00000182242	TEX28P1	X:153461811-153482755	q1	q2	NOTEST	0	0	0	0	1	1	no

ENSG00000182247	ENSG00000182247	UBE2E2	3:23244510-23633284	q1	q2	OK	16.079	18.4538	0.198741	0.360922	0.6016	0.99995	no

ENSG00000182253	ENSG00000182253	SYNM	15:99192199-99675798	q1	q2	NOTEST	0.0255628	0.05453	1.093	0	1	1	no

ENSG00000182255	ENSG00000182255	KCNA4	11:30031287-30038570	q1	q2	NOTEST	0.323227	0.187465	-0.785929	0	1	1	no

ENSG00000182256	ENSG00000182256	GABRG3	15:27216428-27787137	q1	q2	OK	0.925168	0.774824	-0.255846	-0.20333	0.70185	0.99995	no

ENSG00000182257	ENSG00000182257	C22orf26	22:46445357-46509808	q1	q2	NOTEST	0	0	0	0	1	1	no

ENSG00000182261	ENSG00000182261	NLRP10	11:7980970-7986973	q1	q2	NOTEST	0	0	0	0	1	1	no

ENSG00000182263	ENSG00000182263	FIGN	2:164449905-164592522	q1	q2	OK	9.98196	10.0833	0.0145706	0.0192604	0.96375	0.99995	no

ENSG00000182264	ENSG00000182264	IZUMO1	19:49244108-49250166	q1	q2	NOTEST	0.011759	0.0350316	1.57489	0	1	1	no

ENSG00000182271	ENSG00000182271	TMIGD1	17:28643350-28661077	q1	q2	NOTEST	0	0	0	0	1	1	no

ENSG00000182272	ENSG00000182272	B4GALNT4	11:369795-382116	q1	q2	OK	63.2464	52.4554	-0.269893	-0.349075	0.47305	0.99995	no

ENSG00000182287	ENSG00000182287	AP1S2	X:15843928-15873054	q1	q2	OK	41.1983	38.0517	-0.114624	-0.225728	0.70815	0.99995	no

ENSG00000182307	ENSG00000182307	C8orf33	8:146277763-146281416	q1	q2	OK	29.0126	28.8438	-0.00841868	-0.0197538	0.9778	0.99995	no

ENSG00000182308	ENSG00000182308	DCAF4L1	4:41983712-41988476	q1	q2	NOTEST	0.223409	0.39054	0.805784	0	1	1	no

ENSG00000182310	ENSG00000182310	SPACA6P	19:52188615-52215640	q1	q2	OK	9.87676	7.97982	-0.30768	-0.343899	0.6013	0.99995	no

ENSG00000182315	ENSG00000182315	MBD3L3	19:7056219-7058651	q1	q2	NOTEST	0	0	0	0	1	1	no

ENSG00000182318	ENSG00000182318	ZSCAN22	19:58838384-58853698	q1	q2	OK	2.13236	1.9845	-0.103678	-0.16294	0.8186	0.99995	no

ENSG00000182319	ENSG00000182319	SGK223	8:8175257-8244008	q1	q2	OK	6.75171	5.74205	-0.233689	-0.47983	0.49805	0.99995	no

ENSG00000182324	ENSG00000182324	KCNJ14	19:48949029-48985571	q1	q2	OK	0.774358	0.886305	0.194802	0.0374087	0.91945	0.99995	no

ENSG00000182325	ENSG00000182325	FBXL6	8:145575877-145584932	q1	q2	OK	1.97059	1.42675	-0.465898	-0.499681	0.3996	0.99995	no

ENSG00000182326	ENSG00000182326	C1S	12:7072407-7178336	q1	q2	NOTEST	0	0	0	0	1	1	no

ENSG00000182327	ENSG00000182327	GLTPD2	17:4692253-4693685	q1	q2	OK	0.589617	0.436173	-0.434877	-0.378916	0.5936	0.99995	no

ENSG00000182329	ENSG00000182329	AC079354.1	2:202937977-203062585	q1	q2	NOTEST	0.138682	0.0899025	-0.625345	0	1	1	no

ENSG00000182330	ENSG00000182330	PRAMEF8	1:13386645-13390765	q1	q2	NOTEST	0	0	0	0	1	1	no

ENSG00000182333	ENSG00000182333	LIPF	10:90424197-90438571	q1	q2	NOTEST	0	0	0	0	1	1	no

ENSG00000182334	ENSG00000182334	OR5P3	11:7710668-7927502	q1	q2	NOTEST	0	0	0	0	1	1	no

ENSG00000182346	ENSG00000182346	DAOA	13:106111403-106158030	q1	q2	NOTEST	0	0	0	0	1	1	no

ENSG00000182347	ENSG00000182347	RP11-39K24.9	9:4985032-5128183	q1	q2	NOTEST	0	0.0775668	inf	0	1	1	no

ENSG00000182348	ENSG00000182348	ZNF804B	7:88388681-88966346	q1	q2	NOTEST	0.00560853	0.00514886	-0.12337	0	1	1	no

ENSG00000182351	ENSG00000182351	CRIP1P4	22:25108258-25108489	q1	q2	NOTEST	0	0	0	0	1	1	no

ENSG00000182352	ENSG00000182352	C17orf77	17:72575503-72590348	q1	q2	NOTEST	0.0220393	0	#NAME?	0	1	1	no

ENSG00000182359	ENSG00000182359	KBTBD3	11:105921824-105972702	q1	q2	OK	1.16573	2.03559	0.804212	0.219258	0.4662	0.99995	no

ENSG00000182362	ENSG00000182362	YBEY	21:47706250-47717665	q1	q2	OK	15.6265	16.7576	0.100817	0.152834	0.82805	0.99995	no

ENSG00000182365	ENSG00000182365	OR5F2P	11:55782492-55783433	q1	q2	NOTEST	0	0	0	0	1	1	no

ENSG00000182366	ENSG00000182366	FAM87A	8:325930-333174	q1	q2	NOTEST	0.0531944	0.0371888	-0.516406	0	1	1	no

ENSG00000182368	ENSG00000182368	FAM27A	9:45727106-45728274	q1	q2	NOTEST	0.0603734	0.112513	0.898105	0	1	1	no

ENSG00000182372	ENSG00000182372	CLN8	8:1703943-1734738	q1	q2	OK	5.72613	5.46741	-0.0667036	-0.0605975	0.88575	0.99995	no

ENSG00000182376	ENSG00000182376	RP5-1142A6.8	16:88772870-88851619	q1	q2	NOTEST	0.0959104	0.0280569	-1.77333	0	1	1	no

ENSG00000182378	ENSG00000182378	PLCXD1	X:192988-220023	q1	q2	OK	20.0609	19.2636	-0.0585077	-0.089597	0.8715	0.99995	no

ENSG00000182379	ENSG00000182379	NXPH4	12:57610577-57620232	q1	q2	OK	3.93138	3.08245	-0.35096	-0.460258	0.49575	0.99995	no

ENSG00000182383	ENSG00000182383	RPL27AP5	5:74213550-74319753	q1	q2	NOTEST	0	0.0946657	inf	0	1	1	no

ENSG00000182389	ENSG00000182389	CACNB4	2:152689289-152965716	q1	q2	OK	2.73274	2.95622	0.113402	0.129044	0.7652	0.99995	no

ENSG00000182393	ENSG00000182393	IFNL1	19:39786964-39789313	q1	q2	NOTEST	0	0	0	0	1	1	no

ENSG00000182397	ENSG00000182397	DNM1P46	15:100330360-100347132	q1	q2	NOTEST	0.200803	0.179801	-0.159383	0	1	1	no

ENSG00000182400	ENSG00000182400	TRAPPC6B	14:39617014-39639736	q1	q2	OK	12.4871	13.4016	0.101974	0.200864	0.7543	0.99995	no

ENSG00000182404	ENSG00000182404	RP1-86D1.3	6:128289923-128841870	q1	q2	NOTEST	0	0	0	0	1	1	no

ENSG00000182405	ENSG00000182405	PGBD4	15:34394273-34396591	q1	q2	OK	1.55458	1.55634	0.00163165	0.00202569	0.99215	0.99995	no

ENSG00000182415	ENSG00000182415	CDY2A	Y:20137666-20140477	q1	q2	NOTEST	0	0	0	0	1	1	no

ENSG00000182446	ENSG00000182446	NPLOC4	17:79523912-79615779	q1	q2	OK	32.9553	29.5143	-0.159094	-0.300147	0.55685	0.99995	no

ENSG00000182447	ENSG00000182447	OTOL1	3:161214595-161221730	q1	q2	NOTEST	0.311929	0.30303	-0.0417597	0	1	1	no

ENSG00000182450	ENSG00000182450	KCNK4	11:64058773-64072242	q1	q2	NOTEST	0.0308649	0.0981774	1.66942	0	1	1	no

ENSG00000182459	ENSG00000182459	TEX19	17:80317122-80321652	q1	q2	NOTEST	0.164032	0.125963	-0.380975	0	1	1	no

ENSG00000182463	ENSG00000182463	TSHZ2	20:51588945-52120853	q1	q2	OK	1.62899	1.58136	-0.0428103	-0.0688029	0.92085	0.99995	no

ENSG00000182472	ENSG00000182472	CAPN12	19:39138288-39260544	q1	q2	NOTEST	0.0201781	0.0307579	0.60817	0	1	1	no

ENSG00000182473	ENSG00000182473	EXOC7	17:74035183-74117657	q1	q2	OK	47.6211	43.7633	-0.121882	-0.160603	0.7976	0.99995	no

ENSG00000182477	ENSG00000182477	OR2B8P	6:28021005-28021943	q1	q2	NOTEST	0.0661226	0.0628488	-0.0732575	0	1	1	no

ENSG00000182481	ENSG00000182481	KPNA2	17:66031634-66042958	q1	q2	OK	12.8126	7.48755	-0.774994	-0.87492	0.20715	0.99995	no

ENSG00000182484	ENSG00000182484	WASH6P	X:155227245-155257848	q1	q2	OK	7.10186	5.97631	-0.248941	-0.267986	0.6874	0.99995	no

ENSG00000182487	ENSG00000182487	NCF1B	7:72634610-72649979	q1	q2	NOTEST	0	0	0	0	1	1	no

ENSG00000182489	ENSG00000182489	XKRX	X:100168430-100184422	q1	q2	NOTEST	0.142799	0.12657	-0.174043	0	1	1	no

ENSG00000182492	ENSG00000182492	BGN	X:152710177-152775012	q1	q2	NOTEST	0	0	0	0	1	1	no

ENSG00000182500	ENSG00000182500	ORAI1	12:122064454-122080583	q1	q2	NOTEST	0	0	0	0	1	1	no

ENSG00000182502	ENSG00000182502	ABHD17AP5	22:22469235-22472374	q1	q2	NOTEST	0.0339037	0.0331521	-0.0323427	0	1	1	no

ENSG00000182504	ENSG00000182504	CEP97	3:101442768-101489406	q1	q2	OK	8.94182	9.31894	0.0595983	0.121326	0.85305	0.99995	no

ENSG00000182508	ENSG00000182508	LHFPL1	X:111873875-111923375	q1	q2	OK	0.6573	0.813968	0.30842	0.297043	0.64125	0.99995	no

ENSG00000182511	ENSG00000182511	FES	15:91426924-91439006	q1	q2	NOTEST	0.195818	0.394607	1.01091	0	1	1	no

ENSG00000182512	ENSG00000182512	GLRX5	14:95998633-96011061	q1	q2	OK	49.5387	48.0989	-0.0425525	-0.0878127	0.90265	0.99995	no

ENSG00000182518	ENSG00000182518	FAM104B	X:55169534-55187743	q1	q2	OK	14.6153	15.8376	0.115872	0.182807	0.79885	0.99995	no

ENSG00000182521	ENSG00000182521	TBPL2	14:55880258-55923444	q1	q2	NOTEST	0	0	0	0	1	1	no

ENSG00000182531	ENSG00000182531	OR7E115P	10:15049859-15050883	q1	q2	NOTEST	0.0288009	0.0298599	0.0520937	0	1	1	no

ENSG00000182533	ENSG00000182533	CAV3	3:8661085-9005457	q1	q2	NOTEST	0.213379	0.209092	-0.0292799	0	1	1	no

ENSG00000182534	ENSG00000182534	MXRA7	17:74668023-74707098	q1	q2	OK	53.258	52.0096	-0.0342219	-0.0524159	0.94005	0.99995	no

ENSG00000182541	ENSG00000182541	LIMK2	22:31608224-31676066	q1	q2	OK	16.0944	14.5517	-0.145366	-0.238757	0.67395	0.99995	no

ENSG00000182544	ENSG00000182544	MFSD5	12:53645034-53648189	q1	q2	OK	12.7578	11.8819	-0.102612	-0.190738	0.77755	0.99995	no

ENSG00000182545	ENSG00000182545	RNASE10	14:20973695-20979328	q1	q2	NOTEST	0	0.0253294	inf	0	1	1	no

ENSG00000182551	ENSG00000182551	ADI1	2:3500726-3526968	q1	q2	OK	28.5653	28.7495	0.00927766	0.0212697	0.97505	0.99995	no

ENSG00000182552	ENSG00000182552	RWDD4	4:184560787-184580378	q1	q2	OK	7.21936	9.42422	0.384502	0.571962	0.3419	0.99995	no

ENSG00000182557	ENSG00000182557	SPNS3	17:4336982-4391503	q1	q2	NOTEST	0	0	0	0	1	1	no

ENSG00000182565	ENSG00000182565	OR4C2P	11:48441761-48442825	q1	q2	NOTEST	0	0	0	0	1	1	no

ENSG00000182566	ENSG00000182566	CLEC4G	19:7793842-7798792	q1	q2	NOTEST	0	0	0	0	1	1	no

ENSG00000182568	ENSG00000182568	SATB1	3:17198653-18959184	q1	q2	OK	14.708	15.1138	0.0392662	0.0408906	0.92865	0.99995	no

ENSG00000182572	ENSG00000182572	HIST1H3I	6:27839622-27840099	q1	q2	NOTEST	0	0.0879186	inf	0	1	1	no

ENSG00000182574	ENSG00000182574	RP3-341D10.1	X:100211581-100212528	q1	q2	NOTEST	0.0972252	0.0640509	-0.602112	0	1	1	no

ENSG00000182575	ENSG00000182575	NXPH3	17:47572654-47661189	q1	q2	OK	0.568225	0.695947	0.292515	0.0498124	0.81935	0.99995	no

ENSG00000182578	ENSG00000182578	CSF1R	5:149432853-149492935	q1	q2	NOTEST	0.104065	0.0131377	-2.9857	0	1	1	no

ENSG00000182580	ENSG00000182580	EPHB3	3:183851806-184402546	q1	q2	OK	33.516	31.5179	-0.088679	-0.0257397	0.93975	0.99995	no

ENSG00000182583	ENSG00000182583	VCX	X:7810302-7812184	q1	q2	NOTEST	0.0326773	0.163236	2.3206	0	1	1	no

ENSG00000182584	ENSG00000182584	ACTL10	20:32244892-32262269	q1	q2	NOTEST	0.130525	0.166428	0.350569	0	1	1	no

ENSG00000182585	ENSG00000182585	EPGN	4:75174189-75181024	q1	q2	NOTEST	0.0728134	0.0456318	-0.674166	0	1	1	no

ENSG00000182586	ENSG00000182586	LINC00334	21:46654254-46678645	q1	q2	NOTEST	0.0718788	0.290511	2.01495	0	1	1	no

ENSG00000182591	ENSG00000182591	KRTAP11-1	21:32252965-32253874	q1	q2	NOTEST	0	0	0	0	1	1	no

ENSG00000182600	ENSG00000182600	C2orf82	2:233562008-233880595	q1	q2	OK	0.686911	0.46035	-0.577393	-0.0956164	0.5903	0.99995	no

ENSG00000182601	ENSG00000182601	HS3ST4	16:25703346-26149009	q1	q2	OK	4.46425	4.5147	0.016211	0.022875	0.97235	0.99995	no

ENSG00000182606	ENSG00000182606	TRAK1	3:42055293-42267381	q1	q2	OK	46.1035	44.2471	-0.0592944	-0.136688	0.83295	0.99995	no

ENSG00000182611	ENSG00000182611	HIST1H2AJ	6:27782111-27782607	q1	q2	NOTEST	0.0848732	0	#NAME?	0	1	1	no

ENSG00000182612	ENSG00000182612	TSPAN10	17:79523912-79615779	q1	q2	OK	1.85171	1.8217	-0.0235742	-0.00700891	0.9822	0.99995	no

ENSG00000182613	ENSG00000182613	OR2V2	5:180581942-180582890	q1	q2	NOTEST	0.0335096	0	#NAME?	0	1	1	no

ENSG00000182621	ENSG00000182621	PLCB1	20:8112823-8949003	q1	q2	OK	2.8509	3.31421	0.21725	0.145567	0.73175	0.99995	no

ENSG00000182625	ENSG00000182625	RP11-123C21.1	15:58887402-59042177	q1	q2	NOTEST	0	0	0	0	1	1	no

ENSG00000182628	ENSG00000182628	SKA2	17:57059998-57232630	q1	q2	OK	145.427	159.948	0.137306	0.185105	0.7801	0.99995	no

ENSG00000182631	ENSG00000182631	RXFP3	5:33936490-33939023	q1	q2	NOTEST	0.0519614	0.0408672	-0.346497	0	1	1	no

ENSG00000182632	ENSG00000182632	CCNYL2	10:42903615-42967688	q1	q2	NOTEST	0	0	0	0	1	1	no

ENSG00000182634	ENSG00000182634	OR10G7	11:123908772-123909717	q1	q2	NOTEST	0	0	0	0	1	1	no

ENSG00000182636	ENSG00000182636	NDN	15:23930564-23932450	q1	q2	OK	67.1817	67.1793	-5.08E-05	-0.000125468	0.9996	0.99995	no

ENSG00000182645	ENSG00000182645	CCDC172	10:118083939-118139541	q1	q2	NOTEST	0	0	0	0	1	1	no

ENSG00000182646	ENSG00000182646	FAM156A	X:52976461-53024651	q1	q2	NOTEST	0.00911314	0	#NAME?	0	1	1	no

ENSG00000182648	ENSG00000182648	LINC01006	7:156264889-156685924	q1	q2	OK	1.32837	1.69986	0.355764	0.0640008	0.7627	0.99995	no

ENSG00000182652	ENSG00000182652	OR4Q3	14:20187173-20296531	q1	q2	NOTEST	0	0	0	0	1	1	no

ENSG00000182667	ENSG00000182667	NTM	11:131240372-132206716	q1	q2	OK	20.3807	18.7831	-0.117764	-0.163278	0.76345	0.99995	no

ENSG00000182670	ENSG00000182670	TTC3	21:38445525-38575413	q1	q2	OK	116.996	112.591	-0.0553708	-0.0753844	0.8545	0.99995	no

ENSG00000182674	ENSG00000182674	KCNB2	8:73449625-73850584	q1	q2	OK	0.569081	0.5692	0.000300801	0.000322837	0.98855	0.99995	no

ENSG00000182676	ENSG00000182676	PPP1R27	17:79791367-79792926	q1	q2	NOTEST	0.212193	0.11786	-0.848305	0	1	1	no

ENSG00000182685	ENSG00000182685	BRICD5	16:2254248-2261951	q1	q2	OK	12.4235	7.85035	-0.662247	-0.371421	0.3053	0.99995	no

ENSG00000182687	ENSG00000182687	GALR2	17:74035183-74117657	q1	q2	NOTEST	0.0622936	0.0820735	0.397833	0	1	1	no

ENSG00000182698	ENSG00000182698	RESP18	2:220192130-220197899	q1	q2	NOTEST	0	0	0	0	1	1	no

ENSG00000182700	ENSG00000182700	IGIP	5:139505520-139508391	q1	q2	OK	1.66239	1.50234	-0.146051	-0.186272	0.7883	0.99995	no

ENSG00000182704	ENSG00000182704	TSKU	11:76493294-76509198	q1	q2	OK	17.23	15.4421	-0.158056	-0.33444	0.62835	0.99995	no

ENSG00000182707	ENSG00000182707	FXYD6P3	X:73094902-73095470	q1	q2	NOTEST	0	0	0	0	1	1	no

ENSG00000182712	ENSG00000182712	CMC4	X:154289896-154384778	q1	q2	NOTEST	0	0	0	0	1	1	no

ENSG00000182718	ENSG00000182718	ANXA2	15:60639332-60695082	q1	q2	OK	98.1973	95.6235	-0.0383181	-0.0777772	0.90215	0.99995	no

ENSG00000182722	ENSG00000182722	SEPHS1P1	7:64312774-64313732	q1	q2	NOTEST	0.0643754	0	#NAME?	0	1	1	no

ENSG00000182732	ENSG00000182732	RGS6	14:72399155-73061833	q1	q2	OK	1.1014	0.616035	-0.838252	-0.676553	0.1536	0.99995	no

ENSG00000182742	ENSG00000182742	HOXB4	17:46618255-46683776	q1	q2	NOTEST	0.276404	0.281057	0.0240823	0	1	1	no

ENSG00000182747	ENSG00000182747	SLC35D3	6:137243401-137246777	q1	q2	NOTEST	0.101311	0.0890985	-0.185316	0	1	1	no

ENSG00000182749	ENSG00000182749	PAQR7	1:26186103-26197744	q1	q2	OK	4.09633	3.66308	-0.161273	-0.269451	0.69965	0.99995	no

ENSG00000182752	ENSG00000182752	PAPPA	9:118916082-119164601	q1	q2	OK	1.22645	1.99465	0.701647	0.883662	0.09275	0.99995	no

ENSG00000182759	ENSG00000182759	MAFA	8:144501351-144512576	q1	q2	NOTEST	0.130787	0.0329222	-1.99008	0	1	1	no

ENSG00000182768	ENSG00000182768	NGRN	15:90792761-90816463	q1	q2	OK	148.087	146	-0.0204783	-0.0468408	0.9471	0.99995	no

ENSG00000182771	ENSG00000182771	GRID1	10:87359311-88126250	q1	q2	OK	4.0661	3.04154	-0.418843	-0.606536	0.38595	0.99995	no

ENSG00000182774	ENSG00000182774	RPS17L	15:83205503-83378666	q1	q2	OK	28.1528	79.4315	1.49644	1.59316	0.06455	0.99995	no

ENSG00000182776	ENSG00000182776	RP11-114H20.1	X:51793955-51797351	q1	q2	NOTEST	0	0	0	0	1	1	no

ENSG00000182782	ENSG00000182782	HCAR2	12:123011792-123215390	q1	q2	NOTEST	0.195797	0.0764	-1.35772	0	1	1	no

ENSG00000182783	ENSG00000182783	OR2T29	1:248712056-248727140	q1	q2	NOTEST	0	0	0	0	1	1	no

ENSG00000182791	ENSG00000182791	CCDC87	11:66357639-66373490	q1	q2	NOTEST	0.105961	0.0805563	-0.395459	0	1	1	no

ENSG00000182793	ENSG00000182793	GSTA5	6:52696452-52710893	q1	q2	NOTEST	0	0	0	0	1	1	no

ENSG00000182795	ENSG00000182795	C1orf116	1:207191865-207206101	q1	q2	NOTEST	0.0139358	0.0254532	0.869054	0	1	1	no

ENSG00000182796	ENSG00000182796	TMEM198B	12:56137063-56236750	q1	q2	OK	13.4068	10.4904	-0.353895	-0.240589	0.7534	0.99995	no

ENSG00000182798	ENSG00000182798	MAGEB17	X:16141678-16189587	q1	q2	NOTEST	0	0.122605	inf	0	1	1	no

ENSG00000182809	ENSG00000182809	CRIP2	14:105939298-105946499	q1	q2	OK	5.16539	1.86583	-1.46906	-1.45439	0.0267	0.99995	no

ENSG00000182810	ENSG00000182810	DDX28	16:68021296-68272005	q1	q2	OK	5.62085	4.57978	-0.29551	-0.212065	0.7487	0.99995	no

ENSG00000182814	ENSG00000182814	FUNDC2P2	2:84517805-84519326	q1	q2	OK	0.0808955	0.48782	2.59222	0.966898	0.2818	0.99995	no

ENSG00000182816	ENSG00000182816	KRTAP13-2	21:31743708-31744557	q1	q2	NOTEST	0	0	0	0	1	1	no

ENSG00000182824	ENSG00000182824	AC008132.1	22:18721426-18745407	q1	q2	NOTEST	0	0	0	0	1	1	no

ENSG00000182827	ENSG00000182827	ACBD3	1:226332379-226374431	q1	q2	OK	14.4752	16.2106	0.163348	0.334433	0.589	0.99995	no

ENSG00000182831	ENSG00000182831	C16orf72	16:9185504-9215497	q1	q2	OK	11.9325	12.588	0.0771503	0.120114	0.8651	0.99995	no

ENSG00000182836	ENSG00000182836	PLCXD3	5:41307055-41510730	q1	q2	NOTEST	0.0304905	0.0252469	-0.272258	0	1	1	no

ENSG00000182841	ENSG00000182841	RRP7B	22:42949622-42978044	q1	q2	OK	16.3498	16.4747	0.0109816	0.0183651	0.9785	0.99995	no

ENSG00000182851	ENSG00000182851	GPIHBP1	8:144295067-144299044	q1	q2	NOTEST	0	0	0	0	1	1	no

ENSG00000182853	ENSG00000182853	VMO1	17:4688579-4689728	q1	q2	OK	0.585663	0.502606	-0.220643	-0.157305	0.79345	0.99995	no

ENSG00000182854	ENSG00000182854	OR4F15	15:102358365-102359350	q1	q2	NOTEST	0	0	0	0	1	1	no

ENSG00000182858	ENSG00000182858	ALG12	22:50293876-50321188	q1	q2	OK	5.70217	5.55496	-0.0377363	-0.0277787	0.953	0.99995	no

ENSG00000182866	ENSG00000182866	LCK	1:32716839-32751766	q1	q2	NOTEST	0	0.011988	inf	0	1	1	no

ENSG00000182870	ENSG00000182870	GALNT9	12:132680923-132905935	q1	q2	OK	0.521538	0.311347	-0.744248	-0.487428	0.27225	0.99995	no

ENSG00000182871	ENSG00000182871	COL18A1	21:46825051-46964325	q1	q2	OK	17.7598	14.6982	-0.272973	-0.472649	0.53185	0.99995	no

ENSG00000182872	ENSG00000182872	RBM10	X:47001614-47046212	q1	q2	NOTEST	0	0	0	0	1	1	no

ENSG00000182873	ENSG00000182873	RP11-181G12.2	1:1981908-2152177	q1	q2	OK	4.06613	4.36197	0.101323	0.0556427	0.92115	0.99995	no

ENSG00000182885	ENSG00000182885	GPR97	16:57702098-57765717	q1	q2	NOTEST	0.0282717	0	#NAME?	0	1	1	no

ENSG00000182888	ENSG00000182888	RP11-558O12.1	X:3635189-3635641	q1	q2	NOTEST	0	0	0	0	1	1	no

ENSG00000182890	ENSG00000182890	GLUD2	X:120181461-120183794	q1	q2	OK	0.63995	0.931775	0.542024	0.601282	0.36575	0.99995	no

ENSG00000182896	ENSG00000182896	TMEM95	17:7258441-7260538	q1	q2	NOTEST	0	0	0	0	1	1	no

ENSG00000182898	ENSG00000182898	TCHHL1	1:152056619-152061540	q1	q2	NOTEST	0	0	0	0	1	1	no

ENSG00000182899	ENSG00000182899	RPL35A	3:197615945-197687013	q1	q2	OK	750.109	756.405	0.0120605	0.0281347	0.96855	0.99995	no

ENSG00000182901	ENSG00000182901	RGS7	1:240928856-241520530	q1	q2	OK	0.886234	0.806337	-0.136304	-0.136895	0.8318	0.99995	no

ENSG00000182902	ENSG00000182902	SLC25A18	22:18043138-18073760	q1	q2	OK	1.07663	0.777936	-0.468805	-0.457879	0.47835	0.99995	no

ENSG00000182903	ENSG00000182903	ZNF721	4:419223-492945	q1	q2	NOTEST	0.0511502	0.00831031	-2.62177	0	1	1	no

ENSG00000182909	ENSG00000182909	LENG9	19:54955990-54974894	q1	q2	NOTEST	0	0	0	0	1	1	no

ENSG00000182912	ENSG00000182912	C21orf90	21:45917774-46131495	q1	q2	NOTEST	0	0	0	0	1	1	no

ENSG00000182916	ENSG00000182916	TCEAL7	X:102585123-102587254	q1	q2	OK	4.4019	4.45625	0.0177015	0.0210191	0.9764	0.99995	no

ENSG00000182919	ENSG00000182919	C11orf54	11:93394804-93547861	q1	q2	OK	22.1735	24.3851	0.137161	0.111275	0.8566	0.99995	no

ENSG00000182921	ENSG00000182921	CCDC75P1	3:73231651-73232442	q1	q2	NOTEST	0	0	0	0	1	1	no

ENSG00000182923	ENSG00000182923	CEP63	3:134196547-134293859	q1	q2	OK	20.287	21.2983	0.07018	0.0783019	0.8889	0.99995	no

ENSG00000182931	ENSG00000182931	WFDC10B	20:44313291-44337456	q1	q2	NOTEST	0	0	0	0	1	1	no

ENSG00000182934	ENSG00000182934	SRPR	11:126071992-126148026	q1	q2	OK	34.9641	34.5301	-0.0180169	-0.0242558	0.96325	0.99995	no

ENSG00000182938	ENSG00000182938	OTOP3	17:72931813-72946087	q1	q2	NOTEST	0	0	0	0	1	1	no

ENSG00000182944	ENSG00000182944	EWSR1	22:29655840-29696515	q1	q2	OK	266.452	271.309	0.0260656	0.0543615	0.9362	0.99995	no

ENSG00000182950	ENSG00000182950	ODF3L1	15:76016317-76055662	q1	q2	NOTEST	0.121777	0.0696342	-0.806376	0	1	1	no

ENSG00000182952	ENSG00000182952	HMGN4	6:26538632-26546482	q1	q2	OK	35.7633	37.6007	0.0722808	0.156597	0.8216	0.99995	no

ENSG00000182957	ENSG00000182957	SPATA13	13:24553838-24896673	q1	q2	OK	22.454	24.4972	0.125646	0.216383	0.69245	0.99995	no

ENSG00000182963	ENSG00000182963	GJC1	17:42875815-42908184	q1	q2	OK	41.4379	43.1876	0.0596662	0.114021	0.8587	0.99995	no

ENSG00000182965	ENSG00000182965	NPM1P14	7:112160542-112161725	q1	q2	NOTEST	0	0	0	0	1	1	no

ENSG00000182968	ENSG00000182968	SOX1	13:112721912-112726020	q1	q2	OK	36.586	37.4724	0.0345365	0.0851967	0.90345	0.99995	no

ENSG00000182973	ENSG00000182973	CNOT10	3:32726636-32815367	q1	q2	OK	22.7795	22.6373	-0.00903765	-0.0129299	0.979	0.99995	no

ENSG00000182974	ENSG00000182974	OR4M2	15:22278009-22413497	q1	q2	NOTEST	0	0	0	0	1	1	no

ENSG00000182979	ENSG00000182979	MTA1	14:105864915-105937066	q1	q2	OK	6.93535	12.6695	0.869319	0.999456	0.10525	0.99995	no

ENSG00000182983	ENSG00000182983	ZNF662	3:42846243-43101703	q1	q2	NOTEST	0.0158938	0.0246651	0.634008	0	1	1	no

ENSG00000182985	ENSG00000182985	CADM1	11:115039937-115375675	q1	q2	OK	30.7545	31.2088	0.0211552	0.0389039	0.94255	0.99995	no

ENSG00000182986	ENSG00000182986	ZNF320	19:53367042-53400946	q1	q2	OK	18.784	22.0439	0.230874	0.267343	0.4837	0.99995	no

ENSG00000182993	ENSG00000182993	C12orf60	12:14956505-15059520	q1	q2	OK	1.8923	2.0866	0.141011	0.0368819	0.92965	0.99995	no

ENSG00000183010	ENSG00000183010	PYCR1	17:79890259-79905477	q1	q2	OK	27.7541	22.7512	-0.286761	-0.590776	0.3728	0.99995	no

ENSG00000183011	ENSG00000183011	LSMD1	17:7760002-7816078	q1	q2	OK	81.6704	76.6671	-0.0912043	-0.0611667	0.921	0.99995	no

ENSG00000183016	ENSG00000183016	IQCA1P1	7:150888066-150902582	q1	q2	NOTEST	0.0105184	0	#NAME?	0	1	1	no

ENSG00000183018	ENSG00000183018	SPNS2	17:4400688-4458926	q1	q2	OK	1.55131	1.68254	0.117156	0.0576911	0.9261	0.99995	no

ENSG00000183019	ENSG00000183019	C19orf59	19:7741513-7747744	q1	q2	NOTEST	0.0741266	0.0161142	-2.20166	0	1	1	no

ENSG00000183020	ENSG00000183020	AP2A2	11:924893-1012239	q1	q2	OK	30.2221	28.7031	-0.0743987	-0.106618	0.7992	0.99995	no

ENSG00000183022	ENSG00000183022	TPM3P8	2:231280656-231444721	q1	q2	NOTEST	0.113363	0.0553397	-1.03456	0	1	1	no

ENSG00000183023	ENSG00000183023	SLC8A1	2:40013592-40838193	q1	q2	OK	1.26404	1.15377	-0.131684	-0.131228	0.7782	0.99995	no

ENSG00000183024	ENSG00000183024	OR1G1	17:3029877-3030875	q1	q2	NOTEST	0	0	0	0	1	1	no

ENSG00000183032	ENSG00000183032	SLC25A21	14:37116287-37643016	q1	q2	OK	2.49296	2.6372	0.081145	0.0306352	0.9359	0.99995	no

ENSG00000183034	ENSG00000183034	OTOP2	17:72920369-72930007	q1	q2	NOTEST	0	0	0	0	1	1	no

ENSG00000183035	ENSG00000183035	CYLC1	X:83116153-83141705	q1	q2	NOTEST	0	0	0	0	1	1	no

ENSG00000183036	ENSG00000183036	PCP4	21:41239242-41301322	q1	q2	OK	1.57146	1.62732	0.0503941	0.0469732	0.9399	0.99995	no

ENSG00000183038	ENSG00000183038	GGTLC3	22:20366205-20368028	q1	q2	NOTEST	0	0	0	0	1	1	no

ENSG00000183044	ENSG00000183044	ABAT	16:8768421-8962866	q1	q2	OK	63.344	54.7429	-0.210537	-0.321896	0.54965	0.99995	no

ENSG00000183048	ENSG00000183048	SLC25A10	17:79670386-79688042	q1	q2	OK	9.51208	8.27262	-0.201417	-0.222667	0.70865	0.99995	no

ENSG00000183049	ENSG00000183049	CAMK1D	10:12391480-12877545	q1	q2	OK	7.58561	7.1178	-0.0918334	-0.15078	0.82585	0.99995	no

ENSG00000183054	ENSG00000183054	RGPD6	2:111271388-111334762	q1	q2	OK	2.20725	1.20951	-0.867828	-0.517037	0.4061	0.99995	no

ENSG00000183055	ENSG00000183055	FAM133CP	10:60272899-60591195	q1	q2	OK	0.523421	0.562527	0.103951	0.0171975	0.94425	0.99995	no

ENSG00000183060	ENSG00000183060	LYSMD4	15:100017369-100273766	q1	q2	OK	6.14408	6.12438	-0.00463097	-0.00463157	0.9947	0.99995	no

ENSG00000183066	ENSG00000183066	WBP2NL	22:42394728-42466846	q1	q2	NOTEST	0.0213985	0.0148868	-0.523477	0	1	1	no

ENSG00000183067	ENSG00000183067	IGSF5	21:41117333-41174023	q1	q2	OK	1.50538	1.20311	-0.323359	-0.351815	0.6045	0.99995	no

ENSG00000183072	ENSG00000183072	NKX2-5	5:172659111-172662360	q1	q2	OK	10.0706	11.2638	0.16154	0.278472	0.6906	0.99995	no

ENSG00000183077	ENSG00000183077	AFMID	17:76183397-76203782	q1	q2	OK	17.0775	16.336	-0.0640363	-0.087725	0.9096	0.99995	no

ENSG00000183086	ENSG00000183086	GATSL1	7:74379082-74438803	q1	q2	NOTEST	0.0317234	0.0311394	-0.02681	0	1	1	no

ENSG00000183087	ENSG00000183087	GAS6	13:114518602-114567046	q1	q2	OK	4.84254	3.89449	-0.314327	-0.489363	0.5075	0.99995	no

ENSG00000183090	ENSG00000183090	FREM3	4:144480624-144786225	q1	q2	NOTEST	0	0	0	0	1	1	no

ENSG00000183091	ENSG00000183091	NEB	2:152266396-152591001	q1	q2	OK	1.09466	1.11721	0.0294152	0.00618776	0.97125	0.99995	no

ENSG00000183092	ENSG00000183092	BEGAIN	14:101003485-101053750	q1	q2	OK	1.91331	2.06288	0.108589	0.0966894	0.84895	0.99995	no

ENSG00000183098	ENSG00000183098	GPC6	13:93879094-95059655	q1	q2	OK	5.17848	4.51543	-0.197665	-0.402169	0.56965	0.99995	no

ENSG00000183103	ENSG00000183103	LYPD4	19:42341147-42348760	q1	q2	NOTEST	0	0	0	0	1	1	no

ENSG00000183111	ENSG00000183111	ARHGEF37	5:148931509-149014531	q1	q2	OK	0.46913	0.589584	0.329709	0.21435	0.66025	0.99995	no

ENSG00000183114	ENSG00000183114	FAM43B	1:20878931-20881512	q1	q2	OK	1.28357	1.04496	-0.296706	-0.343908	0.61205	0.99995	no

ENSG00000183117	ENSG00000183117	CSMD1	8:2792874-4852494	q1	q2	NOTEST	0.109733	0.0984288	-0.156848	0	1	1	no

ENSG00000183122	ENSG00000183122	OR2A3P	7:143854318-143855271	q1	q2	NOTEST	0	0	0	0	1	1	no

ENSG00000183128	ENSG00000183128	CALHM3	10:105232560-105238997	q1	q2	NOTEST	0	0	0	0	1	1	no

ENSG00000183130	ENSG00000183130	OR2T11	1:248789419-248790491	q1	q2	NOTEST	0	0	0	0	1	1	no

ENSG00000183134	ENSG00000183134	PTGDR2	11:60603468-60623444	q1	q2	NOTEST	0.288418	0.218479	-0.400664	0	1	1	no

ENSG00000183137	ENSG00000183137	CEP57L1	6:109416312-109485135	q1	q2	OK	10.9165	11.0502	0.0175652	0.0249012	0.96625	0.99995	no

ENSG00000183145	ENSG00000183145	RIPPLY3	21:38378449-38391959	q1	q2	OK	0.769739	0.835644	0.118519	0.127525	0.8616	0.99995	no

ENSG00000183146	ENSG00000183146	CYorf17	Y:23544839-23548246	q1	q2	NOTEST	0	0	0	0	1	1	no

ENSG00000183148	ENSG00000183148	ANKRD20A2	9:42368302-42474239	q1	q2	OK	1.98406	1.74883	-0.182069	-0.198866	0.777	0.99995	no

ENSG00000183150	ENSG00000183150	GPR19	12:12813824-12849141	q1	q2	OK	11.2962	10.8144	-0.0628834	-0.112481	0.86975	0.99995	no

ENSG00000183153	ENSG00000183153	GJD3	17:38516906-38520945	q1	q2	NOTEST	0.114435	0	#NAME?	0	1	1	no

ENSG00000183154	ENSG00000183154	RP11-863K10.7	8:37592278-37616619	q1	q2	NOTEST	0.0384324	0.0261532	-0.555333	0	1	1	no

ENSG00000183155	ENSG00000183155	RABIF	1:202848084-202858263	q1	q2	OK	9.69596	10.6123	0.130277	0.254044	0.7178	0.99995	no

ENSG00000183160	ENSG00000183160	TMEM119	12:108983621-108992096	q1	q2	OK	3.241	3.10296	-0.0627942	-0.0827215	0.89205	0.99995	no

ENSG00000183161	ENSG00000183161	FANCF	11:22644078-22834601	q1	q2	OK	8.05291	8.7049	0.112316	0.107837	0.86815	0.99995	no

ENSG00000183166	ENSG00000183166	CALN1	7:71244475-71912136	q1	q2	NOTEST	0.26787	0.195764	-0.452418	0	1	1	no

ENSG00000183169	ENSG00000183169	POM121L1P	22:22981647-22986944	q1	q2	NOTEST	0	0	0	0	1	1	no

ENSG00000183171	ENSG00000183171	RP11-49C9.2	X:149097744-149392815	q1	q2	NOTEST	0.0373045	0.0473866	0.345129	0	1	1	no

ENSG00000183172	ENSG00000183172	SMDT1	22:42475694-42480288	q1	q2	NOTEST	0.298392	0.47533	0.671722	0	1	1	no

ENSG00000183185	ENSG00000183185	GABRR3	3:97705516-97754148	q1	q2	NOTEST	0	0	0	0	1	1	no

ENSG00000183186	ENSG00000183186	C2CD4C	19:405437-409139	q1	q2	OK	5.47704	5.11249	-0.0993709	-0.176022	0.8032	0.99995	no

ENSG00000183196	ENSG00000183196	CHST6	16:75509793-75529305	q1	q2	OK	0.973884	0.787719	-0.306069	-0.34041	0.638	0.99995	no

ENSG00000183199	ENSG00000183199	HSP90AB3P	4:88812994-88815167	q1	q2	OK	3.17685	5.40735	0.767325	1.16499	0.0916	0.99995	no

ENSG00000183206	ENSG00000183206	POTEC	18:14507337-14543584	q1	q2	NOTEST	0	0	0	0	1	1	no

ENSG00000183207	ENSG00000183207	RUVBL2	19:49496704-49520338	q1	q2	OK	46.5605	40.648	-0.195923	-0.440984	0.5281	0.99995	no

ENSG00000183208	ENSG00000183208	GDPGP1	15:90773206-90785315	q1	q2	OK	1.6457	1.41309	-0.219849	-0.101874	0.9016	0.99995	no

ENSG00000183230	ENSG00000183230	CTNNA3	10:67672275-69455927	q1	q2	NOTEST	0.157053	0.202013	0.363194	0	1	1	no

ENSG00000183239	ENSG00000183239	RP5-1120P11.4	6:44056978-44057433	q1	q2	NOTEST	0.295255	0.0953	-1.63141	0	1	1	no

ENSG00000183242	ENSG00000183242	WT1-AS	11:32409320-32480315	q1	q2	NOTEST	0	0.0126682	inf	0	1	1	no

ENSG00000183246	ENSG00000183246	RIMBP3C	22:21899632-21978323	q1	q2	NOTEST	0.0921467	0.0896578	-0.0395037	0	1	1	no

ENSG00000183248	ENSG00000183248	CTD-3193O13.9	19:7933604-7939326	q1	q2	OK	15.0151	12.3318	-0.284035	-0.633693	0.36115	0.99995	no

ENSG00000183249	ENSG00000183249	NF1P3	21:15373247-15377600	q1	q2	NOTEST	0	0	0	0	1	1	no

ENSG00000183250	ENSG00000183250	C21orf67	21:46352728-46359828	q1	q2	OK	1.67133	1.42189	-0.233187	-0.215265	0.75045	0.99995	no

ENSG00000183251	ENSG00000183251	OR51B4	11:5274419-5667019	q1	q2	NOTEST	0.0320079	0.12021	1.90906	0	1	1	no

ENSG00000183255	ENSG00000183255	PTTG1IP	21:46269499-46293752	q1	q2	OK	114.22	110.329	-0.0499978	-0.113775	0.8529	0.99995	no

ENSG00000183258	ENSG00000183258	DDX41	5:176938577-176944470	q1	q2	OK	30.7411	29.3282	-0.0678779	-0.12402	0.84065	0.99995	no

ENSG00000183260	ENSG00000183260	ABHD16B	20:62492565-62494341	q1	q2	OK	2.54841	1.7922	-0.507866	-0.607325	0.39015	0.99995	no

ENSG00000183269	ENSG00000183269	OR52E8	11:5684424-5959849	q1	q2	NOTEST	0	0	0	0	1	1	no

ENSG00000183273	ENSG00000183273	CCDC60	12:119772516-120119435	q1	q2	NOTEST	0.0659865	0.103512	0.649551	0	1	1	no

ENSG00000183281	ENSG00000183281	PLGLB1	2:87098423-87248975	q1	q2	OK	2.07058	1.6629	-0.316336	-0.252796	0.70995	0.99995	no

ENSG00000183283	ENSG00000183283	DAZAP2	12:51632075-51665146	q1	q2	OK	100.466	103.631	0.0447508	0.090885	0.8938	0.99995	no

ENSG00000183287	ENSG00000183287	CCBE1	18:57098171-57364644	q1	q2	NOTEST	0.177972	0.182346	0.035034	0	1	1	no

ENSG00000183291	ENSG00000183291	Sep-15	1:87328131-87380107	q1	q2	OK	114.277	115.794	0.0190311	0.0427557	0.95025	0.99995	no

ENSG00000183292	ENSG00000183292	AC140481.2	2:131328401-131348036	q1	q2	NOTEST	0	0	0	0	1	1	no

ENSG00000183298	ENSG00000183298	RP11-556K13.1	1:102251891-102254059	q1	q2	OK	12.6043	12.3572	-0.0285677	-0.0435428	0.9494	0.99995	no

ENSG00000183303	ENSG00000183303	OR5P2	11:7710668-7927502	q1	q2	NOTEST	0	0	0	0	1	1	no

ENSG00000183304	ENSG00000183304	FAM9A	X:8758835-8769424	q1	q2	NOTEST	0	0	0	0	1	1	no

ENSG00000183305	ENSG00000183305	MAGEA2B	X:151883081-151887096	q1	q2	NOTEST	0	0	0	0	1	1	no

ENSG00000183307	ENSG00000183307	CECR6	22:17597188-17602257	q1	q2	OK	8.20651	7.56449	-0.117526	-0.244168	0.72505	0.99995	no

ENSG00000183308	ENSG00000183308	AC005037.3	2:201773695-201950473	q1	q2	OK	1.12807	0.926502	-0.283996	-0.0364146	0.86375	0.99995	no

ENSG00000183309	ENSG00000183309	ZNF623	8:144718182-144735900	q1	q2	OK	13.5511	13.8227	0.0286295	0.0611051	0.93125	0.99995	no

ENSG00000183310	ENSG00000183310	OR2T34	1:248737021-248738080	q1	q2	NOTEST	0	0	0	0	1	1	no

ENSG00000183313	ENSG00000183313	OR52L1	11:6007121-6008215	q1	q2	NOTEST	0	0	0	0	1	1	no

ENSG00000183317	ENSG00000183317	EPHA10	1:38179551-38230805	q1	q2	NOTEST	0.163106	0.274847	0.752817	0	1	1	no

ENSG00000183318	ENSG00000183318	SPDYE4	17:8656423-8661877	q1	q2	NOTEST	0	0	0	0	1	1	no

ENSG00000183323	ENSG00000183323	CCDC125	5:68576001-68628636	q1	q2	NOTEST	0.100523	0.245152	1.28615	0	1	1	no

ENSG00000183324	ENSG00000183324	C15orf60	15:73735498-73926475	q1	q2	NOTEST	0	0.0957172	inf	0	1	1	no

ENSG00000183336	ENSG00000183336	BOLA2	16:29262828-29519817	q1	q2	NOTEST	0	0	0	0	1	1	no

ENSG00000183337	ENSG00000183337	BCOR	X:39909067-40036582	q1	q2	OK	20.051	20.8762	0.0581896	0.0694786	0.882	0.99995	no

ENSG00000183340	ENSG00000183340	JRKL	11:96123152-96240738	q1	q2	OK	8.5595	9.17839	0.100715	0.199219	0.76915	0.99995	no

ENSG00000183346	ENSG00000183346	C10orf107	10:63422718-63526524	q1	q2	NOTEST	0.0496921	0.29587	2.57387	0	1	1	no

ENSG00000183347	ENSG00000183347	GBP6	1:89829616-89852020	q1	q2	NOTEST	0	0	0	0	1	1	no

ENSG00000183354	ENSG00000183354	KIAA2026	9:5881595-6007904	q1	q2	OK	11.1271	10.6856	-0.0584089	-0.113827	0.8512	0.99995	no

ENSG00000183378	ENSG00000183378	OVCH2	11:7710668-7927502	q1	q2	NOTEST	0	0.0483881	inf	0	1	1	no

ENSG00000183379	ENSG00000183379	SYNDIG1L	14:74872595-74892805	q1	q2	NOTEST	0.0546049	0.011111	-2.29704	0	1	1	no

ENSG00000183385	ENSG00000183385	TTTY8	Y:9528708-9531566	q1	q2	NOTEST	0	0	0	0	1	1	no

ENSG00000183386	ENSG00000183386	FHL3	1:38462441-38471278	q1	q2	OK	27.3225	27.1218	-0.0106348	-0.0206233	0.97745	0.99995	no

ENSG00000183389	ENSG00000183389	OR56A4	11:6023215-6024424	q1	q2	NOTEST	0	0	0	0	1	1	no

ENSG00000183395	ENSG00000183395	PMCH	12:102513955-102591623	q1	q2	NOTEST	0.0682839	0.160884	1.2364	0	1	1	no

ENSG00000183396	ENSG00000183396	TMEM89	3:48658191-48659288	q1	q2	NOTEST	0	0.154196	inf	0	1	1	no

ENSG00000183397	ENSG00000183397	C19orf71	19:3506270-3579086	q1	q2	OK	0.986523	0.813036	-0.279034	-0.036623	0.88345	0.99995	no

ENSG00000183401	ENSG00000183401	CCDC159	19:11406823-11465620	q1	q2	OK	6.29798	4.58235	-0.458803	-0.250396	0.51845	0.99995	no

ENSG00000183405	ENSG00000183405	RPS7P1	17:26782769-26972472	q1	q2	NOTEST	0	0	0	0	1	1	no

ENSG00000183421	ENSG00000183421	RIPK4	21:43159528-43187266	q1	q2	NOTEST	0.139593	0.0515345	-1.43762	0	1	1	no

ENSG00000183423	ENSG00000183423	LRIT3	4:110769357-110793471	q1	q2	NOTEST	0.197958	0.272424	0.460657	0	1	1	no

ENSG00000183426	ENSG00000183426	NPIPA1	16:15005407-15045915	q1	q2	OK	12.7223	13.18	0.0509898	0.0629607	0.92605	0.99995	no

ENSG00000183431	ENSG00000183431	SF3A3	1:38422646-38456593	q1	q2	OK	91.6104	97.2427	0.0860791	0.126805	0.83945	0.99995	no

ENSG00000183432	ENSG00000183432	ZBTB8OSP1	4:151185593-151936879	q1	q2	NOTEST	0	0.0789804	inf	0	1	1	no

ENSG00000183434	ENSG00000183434	TFDP3	X:132350696-132352376	q1	q2	NOTEST	0	0	0	0	1	1	no

ENSG00000183439	ENSG00000183439	TRIM61	4:165869230-165899980	q1	q2	NOTEST	0.187393	0.0705113	-1.41014	0	1	1	no

ENSG00000183444	ENSG00000183444	OR7E38P	7:97595401-97596386	q1	q2	OK	3.15169	2.67414	-0.237051	-0.274688	0.6897	0.99995	no

ENSG00000183454	ENSG00000183454	GRIN2A	16:9852375-10276611	q1	q2	OK	0.30781	0.510639	0.730265	0.263886	0.39695	0.99995	no

ENSG00000183458	ENSG00000183458	RP11-958N24.1	16:15005407-15045915	q1	q2	OK	3.22434	3.78796	0.232417	0.185307	0.7494	0.99995	no

ENSG00000183461	ENSG00000183461	XAGE1C	X:52511760-52516905	q1	q2	NOTEST	0	0	0	0	1	1	no

ENSG00000183463	ENSG00000183463	URAD	13:28551850-28562791	q1	q2	NOTEST	0	0	0	0	1	1	no

ENSG00000183470	ENSG00000183470	AC009365.3	7:132333552-132413528	q1	q2	NOTEST	0	0	0	0	1	1	no

ENSG00000183473	ENSG00000183473	SSTR3	22:37600277-37608362	q1	q2	NOTEST	0.111521	0.0978015	-0.189384	0	1	1	no

ENSG00000183474	ENSG00000183474	GTF2H2C	5:68773298-69006341	q1	q2	OK	1.3539	1.27586	-0.0856467	-0.0515423	0.92915	0.99995	no

ENSG00000183475	ENSG00000183475	ASB7	15:101093733-101191910	q1	q2	OK	4.94059	4.957	0.00478249	0.00690426	0.99045	0.99995	no

ENSG00000183476	ENSG00000183476	SH2D7	15:78370149-78538030	q1	q2	NOTEST	0.353995	0.238433	-0.570142	0	1	1	no

ENSG00000183479	ENSG00000183479	TREX2	X:152710177-152775012	q1	q2	NOTEST	0	0	0	0	1	1	no

ENSG00000183484	ENSG00000183484	GPR132	14:105515727-105531782	q1	q2	NOTEST	0.0272718	0.0259448	-0.0719656	0	1	1	no

ENSG00000183486	ENSG00000183486	MX2	21:42733869-42781317	q1	q2	NOTEST	0.00888403	0.0190989	1.1042	0	1	1	no

ENSG00000183495	ENSG00000183495	EP400	12:132434507-132565005	q1	q2	OK	14.8153	14.4198	-0.0390341	-0.0958866	0.89025	0.99995	no

ENSG00000183496	ENSG00000183496	MEX3B	15:82334118-82338482	q1	q2	OK	25.3361	24.977	-0.0205923	-0.0495522	0.9436	0.99995	no

ENSG00000183504	ENSG00000183504	AL132868.1	4:1873150-1983934	q1	q2	OK	0.980046	0.322244	-1.6047	-0.0583529	0.48115	0.99995	no

ENSG00000183506	ENSG00000183506	PI4KAP2	22:21827288-21871822	q1	q2	OK	24.2148	25.5932	0.0798718	0.162326	0.80525	0.99995	no

ENSG00000183508	ENSG00000183508	FAM46C	1:118148555-118170994	q1	q2	NOTEST	0.134125	0.0966597	-0.472597	0	1	1	no

ENSG00000183513	ENSG00000183513	COA5	2:99215772-99224978	q1	q2	OK	31.0183	28.5652	-0.11886	-0.230671	0.73365	0.99995	no

ENSG00000183514	ENSG00000183514	TDGF1P2	2:228734798-228735360	q1	q2	NOTEST	0	0	0	0	1	1	no

ENSG00000183520	ENSG00000183520	UTP11L	1:38474929-38490496	q1	q2	OK	33.7344	40.4965	0.263579	0.423892	0.541	0.99995	no

ENSG00000183527	ENSG00000183527	PSMG1	21:40546694-40555777	q1	q2	OK	36.0617	39.8887	0.145515	0.270258	0.7009	0.99995	no

ENSG00000183530	ENSG00000183530	PRR14L	22:32072241-32146126	q1	q2	OK	9.09131	9.9889	0.135837	0.220503	0.6901	0.99995	no

ENSG00000183531	ENSG00000183531	Z98256.1	22:32908538-33454358	q1	q2	OK	11.894	8.15458	-0.54455	-0.150709	0.84565	0.99995	no

ENSG00000183535	ENSG00000183535	COL18A1-AS1	21:46825051-46964325	q1	q2	NOTEST	0.0116875	0	#NAME?	0	1	1	no

ENSG00000183542	ENSG00000183542	KLRC4	12:10516367-10562745	q1	q2	NOTEST	0.0325995	0.0337788	0.0512679	0	1	1	no

ENSG00000183549	ENSG00000183549	ACSM5	16:20420855-20458322	q1	q2	NOTEST	0	0	0	0	1	1	no

ENSG00000183558	ENSG00000183558	HIST2H2AA3	1:149813504-149814478	q1	q2	NOTEST	0	0	0	0	1	1	no

ENSG00000183559	ENSG00000183559	C10orf120	10:124457224-124459338	q1	q2	NOTEST	0.0844513	0.028483	-1.56802	0	1	1	no

ENSG00000183560	ENSG00000183560	FOLR4	11:94038758-94040887	q1	q2	NOTEST	0	0	0	0	1	1	no

ENSG00000183562	ENSG00000183562	AC131971.1	11:2965666-3013607	q1	q2	NOTEST	0.442821	0.47728	0.108115	0	1	1	no

ENSG00000183566	ENSG00000183566	BPIFA4P	20:31781390-31798268	q1	q2	NOTEST	0	0	0	0	1	1	no

ENSG00000183569	ENSG00000183569	SERHL2	22:42949622-42978044	q1	q2	OK	2.74095	2.40011	-0.191572	-0.106163	0.8513	0.99995	no

ENSG00000183570	ENSG00000183570	PCBP3	21:47063607-47362368	q1	q2	OK	3.06863	1.81896	-0.754481	-0.661092	0.14025	0.99995	no

ENSG00000183571	ENSG00000183571	PGPEP1L	15:99192199-99675798	q1	q2	NOTEST	0	0.0309444	inf	0	1	1	no

ENSG00000183576	ENSG00000183576	SETD3	14:99864082-99947216	q1	q2	OK	16.8859	17.8326	0.0786956	0.171664	0.80075	0.99995	no

ENSG00000183578	ENSG00000183578	TNFAIP8L3	15:51329684-51630807	q1	q2	OK	2.58196	2.32777	-0.149516	-0.181249	0.7944	0.99995	no

ENSG00000183579	ENSG00000183579	ZNRF3	22:29279579-29453475	q1	q2	OK	4.12541	4.1551	0.0103437	0.0208085	0.97505	0.99995	no

ENSG00000183580	ENSG00000183580	FBXL7	5:15500304-15939900	q1	q2	OK	16.0132	15.4901	-0.0479203	-0.113261	0.8741	0.99995	no

ENSG00000183586	ENSG00000183586	HMGN3P1	1:152346429-152417932	q1	q2	NOTEST	0	0	0	0	1	1	no

ENSG00000183597	ENSG00000183597	TANGO2	22:20004536-20058066	q1	q2	OK	7.65915	6.79379	-0.172967	-0.180336	0.71695	0.99995	no

ENSG00000183598	ENSG00000183598	HIST2H3D	1:149784825-149785236	q1	q2	NOTEST	0.11854	0	#NAME?	0	1	1	no

ENSG00000183604	ENSG00000183604	RP11-347C12.2	16:30234229-30346695	q1	q2	OK	1.29868	1.33498	0.0397764	0.0412693	0.95405	0.99995	no

ENSG00000183605	ENSG00000183605	SFXN4	10:120900278-120925179	q1	q2	OK	4.58285	4.59417	0.00355873	0.0044399	0.9949	0.99995	no

ENSG00000183607	ENSG00000183607	GKN2	2:69172363-69180102	q1	q2	NOTEST	0	0	0	0	1	1	no

ENSG00000183615	ENSG00000183615	FAM167B	1:32712833-32714457	q1	q2	NOTEST	0.331513	0.358034	0.111034	0	1	1	no

ENSG00000183617	ENSG00000183617	MRPL54	19:3762661-3768573	q1	q2	OK	31.2861	29.8125	-0.0696053	-0.118421	0.8645	0.99995	no

ENSG00000183621	ENSG00000183621	ZNF438	10:31109135-31320866	q1	q2	OK	2.22588	1.8905	-0.235607	-0.314631	0.67145	0.99995	no

ENSG00000183624	ENSG00000183624	HMCES	3:128997670-129025029	q1	q2	OK	30.8774	32.2013	0.0605662	0.134749	0.845	0.99995	no

ENSG00000183625	ENSG00000183625	CCR3	3:46205095-46308197	q1	q2	NOTEST	0	0.0230938	inf	0	1	1	no

ENSG00000183628	ENSG00000183628	DGCR6	22:18893540-18924066	q1	q2	OK	4.13487	3.15459	-0.390387	-0.431043	0.52555	0.99995	no

ENSG00000183629	ENSG00000183629	GOLGA8G	15:28764756-28778160	q1	q2	NOTEST	0	0	0	0	1	1	no

ENSG00000183631	ENSG00000183631	CXorf64	X:125953746-125955769	q1	q2	NOTEST	0	0	0	0	1	1	no

ENSG00000183632	ENSG00000183632	TP53TG3	16:32684851-32690152	q1	q2	NOTEST	0	0	0	0	1	1	no

ENSG00000183638	ENSG00000183638	RP1L1	8:10463858-10569697	q1	q2	NOTEST	0.0911524	0.0948772	0.0577802	0	1	1	no

ENSG00000183640	ENSG00000183640	KRTAP8-1	21:32185015-32185570	q1	q2	NOTEST	0	0	0	0	1	1	no

ENSG00000183643	ENSG00000183643	C15orf32	15:93014883-93044347	q1	q2	NOTEST	0.0774373	0.0467083	-0.72935	0	1	1	no

ENSG00000183644	ENSG00000183644	C11orf88	11:111384767-111407756	q1	q2	NOTEST	0.00818529	0	#NAME?	0	1	1	no

ENSG00000183647	ENSG00000183647	ZNF530	19:58111252-58124090	q1	q2	OK	4.98177	4.51879	-0.140723	-0.150284	0.75735	0.99995	no

ENSG00000183648	ENSG00000183648	NDUFB1	14:92582465-92588261	q1	q2	OK	113.364	131.96	0.219138	0.372784	0.60105	0.99995	no

ENSG00000183654	ENSG00000183654	Mar-11	5:16067247-16185694	q1	q2	NOTEST	0.351664	0.369272	0.0704874	0	1	1	no

ENSG00000183655	ENSG00000183655	KLHL25	15:86302553-86338261	q1	q2	OK	13.2067	14.6096	0.145654	0.310743	0.63495	0.99995	no

ENSG00000183657	ENSG00000183657	PP13439	3:171318194-171528740	q1	q2	NOTEST	0.0267828	0.00849534	-1.65657	0	1	1	no

ENSG00000183662	ENSG00000183662	FAM19A1	3:68053358-68594776	q1	q2	NOTEST	0.0713636	0.042131	-0.760305	0	1	1	no

ENSG00000183663	ENSG00000183663	RP11-44N17.1	8:97080956-97081909	q1	q2	NOTEST	0	0	0	0	1	1	no

ENSG00000183665	ENSG00000183665	TRMT12	8:125463047-125474391	q1	q2	OK	9.62836	8.28597	-0.21662	-0.343874	0.59605	0.99995	no

ENSG00000183666	ENSG00000183666	GUSBP1	5:21341941-21589481	q1	q2	OK	11.4741	10.579	-0.117182	-0.147794	0.83645	0.99995	no

ENSG00000183668	ENSG00000183668	PSG9	19:43715942-43773682	q1	q2	NOTEST	0	0	0	0	1	1	no

ENSG00000183671	ENSG00000183671	GPR1	2:207040039-207082771	q1	q2	OK	1.9946	1.46978	-0.440502	-0.296348	0.5808	0.99995	no

ENSG00000183674	ENSG00000183674	LINC00518	6:10429487-10457014	q1	q2	NOTEST	0	0	0	0	1	1	no

ENSG00000183675	ENSG00000183675	PTPN20B	10:48736873-48827966	q1	q2	NOTEST	0	0	0	0	1	1	no

ENSG00000183678	ENSG00000183678	CTAG1A	X:153813406-153815075	q1	q2	NOTEST	0	0	0	0	1	1	no

ENSG00000183682	ENSG00000183682	BMP8A	1:39957317-40024379	q1	q2	NOTEST	0.178443	0.0804637	-1.14905	0	1	1	no

ENSG00000183684	ENSG00000183684	ALYREF	17:79845712-79869340	q1	q2	OK	87.2948	83.436	-0.0652266	-0.102797	0.87765	0.99995	no

ENSG00000183688	ENSG00000183688	FAM101B	17:289768-295730	q1	q2	NOTEST	0	0	0	0	1	1	no

ENSG00000183690	ENSG00000183690	EFHC2	X:44007127-44202918	q1	q2	NOTEST	0.0524061	0.104027	0.989153	0	1	1	no

ENSG00000183691	ENSG00000183691	NOG	17:54671059-54672951	q1	q2	NOTEST	0.373078	0.266329	-0.486266	0	1	1	no

ENSG00000183695	ENSG00000183695	MRGPRX2	11:19076001-19082228	q1	q2	NOTEST	0	0	0	0	1	1	no

ENSG00000183696	ENSG00000183696	UPP1	7:48128224-48148330	q1	q2	OK	16.1514	14.0979	-0.196183	-0.319996	0.64475	0.99995	no

ENSG00000183704	ENSG00000183704	SLC9B1P1	Y:13496240-13524717	q1	q2	NOTEST	0	0	0	0	1	1	no

ENSG00000183706	ENSG00000183706	OR4N4	15:22278009-22413497	q1	q2	NOTEST	0	0	0	0	1	1	no

ENSG00000183709	ENSG00000183709	IFNL2	19:39759153-39760732	q1	q2	NOTEST	0	0	0	0	1	1	no

ENSG00000183715	ENSG00000183715	OPCML	11:132284870-133402414	q1	q2	OK	1.57655	2.1049	0.416983	0.258619	0.4367	0.99995	no

ENSG00000183718	ENSG00000183718	TRIM52	5:180663908-180699308	q1	q2	OK	10.8926	10.9949	0.0134904	0.00432823	0.9926	0.99995	no

ENSG00000183722	ENSG00000183722	LHFP	13:39917028-40177665	q1	q2	OK	72.0841	68.6923	-0.0695333	-0.171642	0.80135	0.99995	no

ENSG00000183723	ENSG00000183723	CMTM4	16:66648652-66730610	q1	q2	OK	10.5334	11.4711	0.123033	0.209171	0.7423	0.99995	no

ENSG00000183726	ENSG00000183726	TMEM50A	1:25568727-25756683	q1	q2	OK	52.9224	55.0329	0.0564161	0.0846791	0.8949	0.99995	no

ENSG00000183729	ENSG00000183729	NPBWR1	8:53850990-53853677	q1	q2	NOTEST	0.02966	0.0184922	-0.681602	0	1	1	no

ENSG00000183733	ENSG00000183733	FIGLA	2:71004441-71017775	q1	q2	NOTEST	0	0	0	0	1	1	no

ENSG00000183734	ENSG00000183734	ASCL2	11:2289724-2292182	q1	q2	NOTEST	0.071822	0.056759	-0.339577	0	1	1	no

ENSG00000183735	ENSG00000183735	TBK1	12:64845659-64895888	q1	q2	OK	12.903	14.5553	0.173832	0.220396	0.63545	0.99995	no

ENSG00000183741	ENSG00000183741	CBX6	22:39257454-39268319	q1	q2	OK	12.7641	11.0472	-0.20841	-0.474231	0.50695	0.99995	no

ENSG00000183742	ENSG00000183742	MACC1	7:19958603-20257027	q1	q2	NOTEST	0.00279085	0.0163445	2.55003	0	1	1	no

ENSG00000183747	ENSG00000183747	ACSM2A	16:20462782-20498991	q1	q2	NOTEST	0	0.0230402	inf	0	1	1	no

ENSG00000183748	ENSG00000183748	MRC1L1	10:17851361-17953178	q1	q2	NOTEST	0	0	0	0	1	1	no

ENSG00000183751	ENSG00000183751	TBL3	16:2022037-2034193	q1	q2	OK	14.4458	11.3286	-0.350676	-0.513246	0.4535	0.99995	no

ENSG00000183753	ENSG00000183753	BPY2	Y:25119965-25151612	q1	q2	NOTEST	0	0	0	0	1	1	no

ENSG00000183760	ENSG00000183760	PAPL	19:39574552-39602133	q1	q2	NOTEST	0	0	0	0	1	1	no

ENSG00000183762	ENSG00000183762	KREMEN1	22:29469065-29564321	q1	q2	OK	2.31818	2.4915	0.10402	0.158847	0.79455	0.99995	no

ENSG00000183763	ENSG00000183763	TRAIP	3:49866033-49894007	q1	q2	OK	14.873	11.9613	-0.314325	-0.4012	0.4452	0.99995	no

ENSG00000183765	ENSG00000183765	CHEK2	22:29083730-29153503	q1	q2	OK	14.032	13.9125	-0.0123408	-0.0183621	0.9737	0.99995	no

ENSG00000183770	ENSG00000183770	FOXL2	3:138663065-138665982	q1	q2	OK	1.48335	1.60191	0.110941	0.137353	0.84615	0.99995	no

ENSG00000183773	ENSG00000183773	AIFM3	22:21319395-21353327	q1	q2	NOTEST	0.161301	0.185959	0.20523	0	1	1	no

ENSG00000183775	ENSG00000183775	KCTD16	5:143550395-143865249	q1	q2	NOTEST	0.366877	0.422502	0.203663	0	1	1	no

ENSG00000183778	ENSG00000183778	B3GALT5	21:40928368-41098012	q1	q2	OK	3.02414	3.79289	0.326773	0.475694	0.3373	0.99995	no

ENSG00000183779	ENSG00000183779	ZNF703	8:37553268-37557537	q1	q2	OK	21.1426	17.8881	-0.24116	-0.550147	0.432	0.99995	no

ENSG00000183780	ENSG00000183780	SLC35F3	1:234040678-234460262	q1	q2	NOTEST	0.269011	0.173249	-0.634822	0	1	1	no

ENSG00000183783	ENSG00000183783	KCTD8	4:44175925-44450824	q1	q2	NOTEST	0.362199	0.256831	-0.495963	0	1	1	no

ENSG00000183784	ENSG00000183784	C9orf66	9:213107-746105	q1	q2	NOTEST	0.00917631	0.0177818	0.954417	0	1	1	no

ENSG00000183785	ENSG00000183785	TUBA8	22:18559292-18629321	q1	q2	OK	1.16526	1.05033	-0.149804	-0.0512402	0.9178	0.99995	no

ENSG00000183791	ENSG00000183791	TCEB3C	18:44388352-44627658	q1	q2	NOTEST	0	0.0147093	inf	0	1	1	no

ENSG00000183793	ENSG00000183793	NPIPA5	16:15457515-15474904	q1	q2	OK	0.965883	2.29235	1.24691	1.26201	0.08615	0.99995	no

ENSG00000183795	ENSG00000183795	BPY2B	Y:26753706-26785354	q1	q2	NOTEST	0	0	0	0	1	1	no

ENSG00000183798	ENSG00000183798	EMILIN3	20:39969559-39995467	q1	q2	OK	15.1975	14.8416	-0.0341939	-0.069359	0.923	0.99995	no

ENSG00000183801	ENSG00000183801	OLFML1	11:7490917-7533746	q1	q2	OK	4.69874	3.97393	-0.241706	-0.302353	0.66935	0.99995	no

ENSG00000183807	ENSG00000183807	FAM162B	6:117073362-117086886	q1	q2	NOTEST	0.117143	0.231099	0.980241	0	1	1	no

ENSG00000183808	ENSG00000183808	RBM12B	8:94225530-94753245	q1	q2	OK	14.1393	15.8276	0.162731	0.14312	0.70015	0.99995	no

ENSG00000183813	ENSG00000183813	CCR4	3:32993065-32997841	q1	q2	NOTEST	0.118749	0.056744	-1.06537	0	1	1	no

ENSG00000183814	ENSG00000183814	LIN9	1:226418849-226497570	q1	q2	OK	8.65697	8.77793	0.020019	0.0373564	0.9562	0.99995	no

ENSG00000183822	ENSG00000183822	CTA-833B7.2	22:37243414-37274057	q1	q2	NOTEST	0	0	0	0	1	1	no

ENSG00000183826	ENSG00000183826	BTBD9	6:38136226-38607924	q1	q2	OK	4.35594	4.56177	0.0666083	0.0697165	0.8872	0.99995	no

ENSG00000183828	ENSG00000183828	NUDT14	14:105607317-105647660	q1	q2	OK	12.3304	9.1876	-0.424458	-0.46348	0.4949	0.99995	no

ENSG00000183831	ENSG00000183831	ANKRD45	1:173469602-173639001	q1	q2	OK	1.55936	1.7371	0.15573	0.168731	0.805	0.99995	no

ENSG00000183833	ENSG00000183833	MAATS1	3:119421868-119485949	q1	q2	OK	0.899494	1.08896	0.275768	0.174365	0.63885	0.99995	no

ENSG00000183837	ENSG00000183837	PNMA3	X:152224765-152228827	q1	q2	NOTEST	0.118131	0.121288	0.0380542	0	1	1	no

ENSG00000183840	ENSG00000183840	GPR39	2:133174146-133429152	q1	q2	OK	1.43397	1.24704	-0.201503	-0.0583308	0.88315	0.99995	no

ENSG00000183844	ENSG00000183844	FAM3B	21:42676138-42729358	q1	q2	NOTEST	0	0.0997227	inf	0	1	1	no

ENSG00000183850	ENSG00000183850	ZNF730	19:23258002-23330021	q1	q2	OK	12.9841	15.3747	0.243815	0.411756	0.5231	0.99995	no

ENSG00000183853	ENSG00000183853	KIRREL	1:157963062-158070052	q1	q2	OK	23.8522	26.532	0.15361	0.337763	0.6228	0.99995	no

ENSG00000183856	ENSG00000183856	IQGAP3	1:156495196-156542396	q1	q2	OK	12.9468	13.3458	0.043793	0.0530975	0.90955	0.99995	no

ENSG00000183862	ENSG00000183862	CNGA2	X:150906922-150913776	q1	q2	NOTEST	0	0	0	0	1	1	no

ENSG00000183864	ENSG00000183864	TOB2	22:41829495-41843027	q1	q2	OK	18.0113	17.8875	-0.00995377	-0.0212587	0.9743	0.99995	no

ENSG00000183873	ENSG00000183873	SCN5A	3:38589547-38691164	q1	q2	NOTEST	0.226913	0.354341	0.642998	0	1	1	no

ENSG00000183876	ENSG00000183876	ARSI	5:149675905-149718870	q1	q2	OK	1.30771	1.07422	-0.283751	-0.321938	0.6125	0.99995	no

ENSG00000183878	ENSG00000183878	UTY	Y:15360258-15592553	q1	q2	OK	6.59843	6.0504	-0.125092	-0.221541	0.72125	0.99995	no

ENSG00000183888	ENSG00000183888	C1orf64	1:16330730-16335302	q1	q2	NOTEST	0	0	0	0	1	1	no

ENSG00000183889	ENSG00000183889	NPIPA7	16:16404197-16444447	q1	q2	OK	3.46957	2.96174	-0.228315	-0.234086	0.66715	0.99995	no

ENSG00000183891	ENSG00000183891	TTC32	2:20096403-20101747	q1	q2	OK	9.42616	11.077	0.23282	0.322255	0.6518	0.99995	no

ENSG00000183900	ENSG00000183900	AC099522.1	5:72740653-72744352	q1	q2	OK	1.39612	1.31263	-0.0889583	-0.0867321	0.9072	0.99995	no

ENSG00000183908	ENSG00000183908	LRRC55	11:56949220-56959191	q1	q2	OK	8.06185	7.99867	-0.011352	-0.0248923	0.9716	0.99995	no

ENSG00000183909	ENSG00000183909	OR4G2P	15:102467007-102467910	q1	q2	NOTEST	0	0	0	0	1	1	no

ENSG00000183911	ENSG00000183911	RPL21P132	X:100594904-100595922	q1	q2	NOTEST	0	0	0	0	1	1	no

ENSG00000183914	ENSG00000183914	DNAH2	17:7620671-7737062	q1	q2	NOTEST	0.0272605	0.0484372	0.8293	0	1	1	no

ENSG00000183918	ENSG00000183918	SH2D1A	X:123093082-124097666	q1	q2	NOTEST	0.259422	0.147577	-0.813833	0	1	1	no

ENSG00000183921	ENSG00000183921	SDR42E2	16:22177617-22203075	q1	q2	NOTEST	0.0305792	0	#NAME?	0	1	1	no

ENSG00000183929	ENSG00000183929	DUSP5P1	1:228744884-228788150	q1	q2	NOTEST	0.216601	0.249141	0.201926	0	1	1	no

ENSG00000183935	ENSG00000183935	HTR7P1	12:13080659-13193613	q1	q2	OK	0.803524	0.886832	0.142321	0.0608453	0.9237	0.99995	no

ENSG00000183938	ENSG00000183938	TRBV21OR9-2	9:33629118-33629584	q1	q2	NOTEST	0	0	0	0	1	1	no

ENSG00000183941	ENSG00000183941	HIST2H4A	1:149804220-149812765	q1	q2	NOTEST	0	0	0	0	1	1	no

ENSG00000183943	ENSG00000183943	PRKX	X:3522410-3631649	q1	q2	OK	13.8641	14.5806	0.0727061	0.0829807	0.8424	0.99995	no

ENSG00000183955	ENSG00000183955	SETD8	12:123868319-123893905	q1	q2	OK	27.1665	28.1193	0.0497311	0.0884447	0.87325	0.99995	no

ENSG00000183960	ENSG00000183960	KCNH8	3:19189945-19577138	q1	q2	OK	4.76815	4.73439	-0.0102496	-0.0195874	0.97675	0.99995	no

ENSG00000183963	ENSG00000183963	SMTN	22:31460090-31500743	q1	q2	OK	10.1386	9.26453	-0.130065	-0.191412	0.72925	0.99995	no

ENSG00000183971	ENSG00000183971	NPW	16:2059926-2070756	q1	q2	NOTEST	0.391694	0.473904	0.274868	0	1	1	no

ENSG00000183977	ENSG00000183977	PP2D1	3:19988570-20053822	q1	q2	NOTEST	0.204224	0.332631	0.703772	0	1	1	no

ENSG00000183978	ENSG00000183978	COA3	17:40932695-40950722	q1	q2	OK	73.0192	65.7303	-0.151717	-0.329419	0.6309	0.99995	no

ENSG00000183979	ENSG00000183979	NPB	17:79845712-79869340	q1	q2	NOTEST	0.387402	0.386313	-0.00406091	0	1	1	no

ENSG00000183981	ENSG00000183981	MAGEA13P	X:137564902-137565955	q1	q2	NOTEST	0	0	0	0	1	1	no

ENSG00000184005	ENSG00000184005	ST6GALNAC3	1:76540403-77100286	q1	q2	OK	16.3011	17.1596	0.0740499	0.106854	0.8723	0.99995	no

ENSG00000184007	ENSG00000184007	PTP4A2	1:32307619-32410457	q1	q2	OK	102.928	109.621	0.0908872	0.127501	0.8065	0.99995	no

ENSG00000184009	ENSG00000184009	ACTG1	17:79476996-79494802	q1	q2	OK	120.278	105.09	-0.19474	-0.229655	0.75845	0.99995	no

ENSG00000184012	ENSG00000184012	TMPRSS2	21:42836477-42903043	q1	q2	NOTEST	0.0473726	0.0940177	0.988881	0	1	1	no

ENSG00000184014	ENSG00000184014	DENND5A	11:9160371-9286937	q1	q2	OK	42.1889	41.6037	-0.0201503	-0.0319255	0.94935	0.99995	no

ENSG00000184022	ENSG00000184022	OR2T10	1:248756051-248757100	q1	q2	NOTEST	0	0	0	0	1	1	no

ENSG00000184029	ENSG00000184029	DSCR4	21:39323727-39493454	q1	q2	NOTEST	0	0	0	0	1	1	no

ENSG00000184032	ENSG00000184032	KRTAP20-2	21:32007554-32007938	q1	q2	NOTEST	0	0	0	0	1	1	no

ENSG00000184033	ENSG00000184033	CTAG1B	X:153845864-153847533	q1	q2	NOTEST	0	0	0	0	1	1	no

ENSG00000184040	ENSG00000184040	TMEM236	10:18041217-18089855	q1	q2	NOTEST	0.00925648	0.00435931	-1.08636	0	1	1	no

ENSG00000184047	ENSG00000184047	DIABLO	12:122688089-122751068	q1	q2	OK	73.0171	68.8754	-0.0842462	-0.127527	0.8508	0.99995	no

ENSG00000184055	ENSG00000184055	OR7E87P	11:71292900-71319086	q1	q2	NOTEST	0	0.06555	inf	0	1	1	no

ENSG00000184056	ENSG00000184056	VPS33B	15:91541645-91565833	q1	q2	OK	9.50073	10.5709	0.153986	0.213274	0.726	0.99995	no

ENSG00000184058	ENSG00000184058	TBX1	22:19744225-19842462	q1	q2	OK	0.895479	0.909437	0.0223132	0.0059484	0.9868	0.99995	no

ENSG00000184060	ENSG00000184060	ADAP2	17:29224353-29286340	q1	q2	NOTEST	0.19658	0.138101	-0.509395	0	1	1	no

ENSG00000184068	ENSG00000184068	RP5-821D11.7	22:42227218-42303312	q1	q2	OK	2.64	2.75635	0.0622234	0.020206	0.9439	0.99995	no

ENSG00000184076	ENSG00000184076	UQCR10	22:30163357-30166402	q1	q2	OK	95.6975	90.7699	-0.0762676	-0.168379	0.8051	0.99995	no

ENSG00000184083	ENSG00000184083	FAM120C	X:54094756-54209714	q1	q2	NOTEST	0.098088	0.127141	0.374283	0	1	1	no

ENSG00000184084	ENSG00000184084	CTD-2372A4.1	5:72921982-73237818	q1	q2	NOTEST	0	0	0	0	1	1	no

ENSG00000184100	ENSG00000184100	BRD7P2	3:159631188-159943086	q1	q2	OK	2.41185	2.69206	0.158574	0.198548	0.7846	0.99995	no

ENSG00000184106	ENSG00000184106	TREML3P	6:41176291-41190382	q1	q2	NOTEST	0	0	0	0	1	1	no

ENSG00000184108	ENSG00000184108	TRIML1	4:189060572-189068897	q1	q2	NOTEST	0.0483382	0	#NAME?	0	1	1	no

ENSG00000184110	ENSG00000184110	EIF3C	16:28671016-28752102	q1	q2	OK	7.39934	7.3465	-0.0103393	-0.0187384	0.9776	0.99995	no

ENSG00000184111	ENSG00000184111	RP11-366I13.3	10:94353042-94415150	q1	q2	NOTEST	0	0	0	0	1	1	no

ENSG00000184113	ENSG00000184113	CLDN5	22:19510546-19515068	q1	q2	NOTEST	0.3274	0.259191	-0.337035	0	1	1	no

ENSG00000184115	ENSG00000184115	RP13-1039J1.2	2:111143953-111230652	q1	q2	NOTEST	0.00920375	0	#NAME?	0	1	1	no

ENSG00000184117	ENSG00000184117	NIPSNAP1	22:29901867-29977326	q1	q2	OK	75.6857	74.0521	-0.0314803	-0.0650187	0.9197	0.99995	no

ENSG00000184139	ENSG00000184139	RPL7AP28	4:132778497-132779068	q1	q2	NOTEST	0	0	0	0	1	1	no

ENSG00000184140	ENSG00000184140	OR4F6	15:102345901-102346876	q1	q2	NOTEST	0	0	0	0	1	1	no

ENSG00000184144	ENSG00000184144	CNTN2	1:205012324-205047627	q1	q2	OK	1.4998	1.34704	-0.15498	-0.125501	0.7307	0.99995	no

ENSG00000184148	ENSG00000184148	SPRR4	1:152943141-152945050	q1	q2	NOTEST	0	0	0	0	1	1	no

ENSG00000184154	ENSG00000184154	LRTOMT	11:71709586-71823826	q1	q2	OK	9.60442	8.7448	-0.135274	-0.0269026	0.84595	0.99995	no

ENSG00000184155	ENSG00000184155	OR10J5	1:159504792-159505842	q1	q2	NOTEST	0	0	0	0	1	1	no

ENSG00000184156	ENSG00000184156	KCNQ3	8:133133107-133493200	q1	q2	NOTEST	0.482052	0.477864	-0.0125878	0	1	1	no

ENSG00000184160	ENSG00000184160	ADRA2C	4:3768074-3770251	q1	q2	OK	1.21373	1.54439	0.347594	0.400983	0.56655	0.99995	no

ENSG00000184162	ENSG00000184162	NR2C2AP	19:19256375-19314233	q1	q2	OK	5.75439	6.36321	0.145091	0.0843196	0.89325	0.99995	no

ENSG00000184163	ENSG00000184163	FAM132A	1:1177825-1182102	q1	q2	OK	1.90513	1.65603	-0.202161	-0.184331	0.81065	0.99995	no

ENSG00000184164	ENSG00000184164	CRELD2	22:50293876-50321188	q1	q2	OK	29.1402	24.2686	-0.263924	-0.445586	0.5306	0.99995	no

ENSG00000184166	ENSG00000184166	OR1D2	17:2995351-2996290	q1	q2	NOTEST	0	0	0	0	1	1	no

ENSG00000184178	ENSG00000184178	SCFD2	4:53739148-54232242	q1	q2	OK	4.56154	4.61039	0.0153683	0.0250105	0.9708	0.99995	no

ENSG00000184182	ENSG00000184182	UBE2F	2:238875468-239008054	q1	q2	OK	29.6175	30.52	0.0433072	0.0699267	0.9169	0.99995	no

ENSG00000184185	ENSG00000184185	KCNJ12	17:21279508-21323179	q1	q2	NOTEST	0	0	0	0	1	1	no

ENSG00000184188	ENSG00000184188	CTC-512J14.7	5:79595496-79598666	q1	q2	NOTEST	0	0	0	0	1	1	no

ENSG00000184194	ENSG00000184194	GPR173	X:53078272-53109797	q1	q2	NOTEST	0	0	0	0	1	1	no

ENSG00000184203	ENSG00000184203	PPP1R2	3:195241220-195270209	q1	q2	OK	14.4582	15.1608	0.0684519	0.107853	0.8684	0.99995	no

ENSG00000184205	ENSG00000184205	TSPYL2	X:53111548-53117722	q1	q2	NOTEST	0	0	0	0	1	1	no

ENSG00000184206	ENSG00000184206	GOLGA6L4	15:84904524-84914120	q1	q2	NOTEST	0.27788	0.452503	0.703464	0	1	1	no

ENSG00000184207	ENSG00000184207	PGP	16:2261997-2264808	q1	q2	OK	14.3074	14.0131	-0.0299843	-0.0606979	0.92765	0.99995	no

ENSG00000184208	ENSG00000184208	C22orf46	22:42069933-42094140	q1	q2	OK	9.04464	8.25313	-0.132122	-0.116373	0.82655	0.99995	no

ENSG00000184209	ENSG00000184209	SNRNP35	12:123942187-124018265	q1	q2	OK	21.6146	20.7628	-0.0580108	-0.0723619	0.90295	0.99995	no

ENSG00000184210	ENSG00000184210	DGAT2L6	X:69397332-69425395	q1	q2	NOTEST	0.0175391	0.0352615	1.00752	0	1	1	no

ENSG00000184216	ENSG00000184216	IRAK1	X:153275950-153285440	q1	q2	NOTEST	0.039386	0.00736519	-2.41889	0	1	1	no

ENSG00000184220	ENSG00000184220	CMSS1	3:99536677-99897447	q1	q2	OK	23.4314	27.6707	0.239917	0.332503	0.61875	0.99995	no

ENSG00000184221	ENSG00000184221	OLIG1	21:34285956-34444726	q1	q2	OK	5.64518	8.9034	0.657337	0.810675	0.1889	0.99995	no

ENSG00000184224	ENSG00000184224	C11orf72	11:67370350-67374177	q1	q2	NOTEST	0.0112146	0.0360404	1.68423	0	1	1	no

ENSG00000184226	ENSG00000184226	PCDH9	13:66876966-67804468	q1	q2	OK	6.33447	5.36337	-0.240083	-0.478025	0.4993	0.99995	no

ENSG00000184227	ENSG00000184227	ACOT1	14:73945188-74025651	q1	q2	NOTEST	0.303157	0.414478	0.451233	0	1	1	no

ENSG00000184232	ENSG00000184232	OAF	11:120081474-120101041	q1	q2	OK	59.7412	51.3662	-0.217908	-0.349425	0.59375	0.99995	no

ENSG00000184247	ENSG00000184247	AC100791.1	17:77906141-78009647	q1	q2	NOTEST	0	0	0	0	1	1	no

ENSG00000184254	ENSG00000184254	ALDH1A3	15:101389765-101626271	q1	q2	OK	2.72207	3.08798	0.181958	0.2014	0.72595	0.99995	no

ENSG00000184258	ENSG00000184258	CDR1	X:139865424-139866723	q1	q2	OK	2.12181	2.17149	0.0333894	0.0387233	0.95565	0.99995	no

ENSG00000184260	ENSG00000184260	HIST2H2AC	1:149858524-149858961	q1	q2	NOTEST	0.199451	0	#NAME?	0	1	1	no

ENSG00000184261	ENSG00000184261	KCNK12	2:47630107-47798078	q1	q2	OK	1.46472	1.05044	-0.479635	-0.189357	0.82405	0.99995	no

ENSG00000184270	ENSG00000184270	HIST2H2AB	1:149859018-149872351	q1	q2	NOTEST	0	0	0	0	1	1	no

ENSG00000184271	ENSG00000184271	POU6F1	12:51580718-51611477	q1	q2	OK	6.41787	5.71493	-0.167359	-0.151873	0.73485	0.99995	no

ENSG00000184274	ENSG00000184274	LINC00315	21:46720159-46725172	q1	q2	NOTEST	0.0341404	0.0332952	-0.036166	0	1	1	no

ENSG00000184276	ENSG00000184276	DEFB108B	11:71544245-71548756	q1	q2	NOTEST	0	0	0	0	1	1	no

ENSG00000184277	ENSG00000184277	TM2D3	15:102161846-102192594	q1	q2	OK	25.4503	25.5406	0.00510835	0.00962159	0.9889	0.99995	no

ENSG00000184281	ENSG00000184281	TSSC4	11:2421717-2425106	q1	q2	OK	23.3108	18.7425	-0.314685	-0.605566	0.39885	0.99995	no

ENSG00000184292	ENSG00000184292	TACSTD2	1:59041098-59043166	q1	q2	NOTEST	0	0	0	0	1	1	no

ENSG00000184293	ENSG00000184293	CLECL1	12:9868455-9885895	q1	q2	NOTEST	0.188887	0.16042	-0.23567	0	1	1	no

ENSG00000184302	ENSG00000184302	SIX6	14:60863186-61021634	q1	q2	OK	1.98332	1.7387	-0.189911	-0.231655	0.74615	0.99995	no

ENSG00000184303	ENSG00000184303	DRD5P1	2:91872472-91902771	q1	q2	NOTEST	0	0	0	0	1	1	no

ENSG00000184304	ENSG00000184304	PRKD1	14:30045686-30766249	q1	q2	OK	5.07325	5.6848	0.164198	0.226673	0.68705	0.99995	no

ENSG00000184305	ENSG00000184305	CCSER1	4:91048685-92523064	q1	q2	NOTEST	0.254728	0.319424	0.326517	0	1	1	no

ENSG00000184307	ENSG00000184307	ZDHHC23	3:113666747-113807269	q1	q2	OK	0.452367	0.684922	0.598446	0.115794	0.65845	0.99995	no

ENSG00000184313	ENSG00000184313	MROH7	1:55107458-55208330	q1	q2	NOTEST	0.017594	0.0837084	2.25029	0	1	1	no

ENSG00000184319	ENSG00000184319	RPL23AP82	22:51195375-51239737	q1	q2	NOTEST	0.250413	0.342767	0.452921	0	1	1	no

ENSG00000184321	ENSG00000184321	OR51J1	11:5274419-5667019	q1	q2	NOTEST	0	0.0636231	inf	0	1	1	no

ENSG00000184324	ENSG00000184324	CSAG2	X:151922444-151928738	q1	q2	NOTEST	0	0	0	0	1	1	no

ENSG00000184330	ENSG00000184330	S100A7A	1:153388999-153395701	q1	q2	NOTEST	0.00613129	0	#NAME?	0	1	1	no

ENSG00000184343	ENSG00000184343	SRPK3	X:152990322-153051187	q1	q2	NOTEST	0.00584463	0	#NAME?	0	1	1	no

ENSG00000184344	ENSG00000184344	GDF3	12:7842377-7848372	q1	q2	OK	0.640796	0.492253	-0.380463	-0.302552	0.6762	0.99995	no

ENSG00000184345	ENSG00000184345	IQCF2	3:51895644-51897440	q1	q2	NOTEST	0	0	0	0	1	1	no

ENSG00000184347	ENSG00000184347	SLIT3	5:168081517-168728133	q1	q2	OK	2.20534	2.20668	0.00087834	0.00114459	0.99855	0.99995	no

ENSG00000184348	ENSG00000184348	HIST1H2AK	6:27805657-27806117	q1	q2	OK	1.22758	1.11072	-0.144325	-0.112215	0.8803	0.99995	no

ENSG00000184349	ENSG00000184349	EFNA5	5:106712589-107006596	q1	q2	OK	9.72768	10.4746	0.106722	0.160082	0.7558	0.99995	no

ENSG00000184350	ENSG00000184350	MRGPRE	11:3247290-3254068	q1	q2	NOTEST	0	0	0	0	1	1	no

ENSG00000184351	ENSG00000184351	KRTAP19-1	21:31852017-31852663	q1	q2	NOTEST	0	0	0	0	1	1	no

ENSG00000184357	ENSG00000184357	HIST1H1B	6:27834569-27835359	q1	q2	NOTEST	0.0804524	0.157709	0.971052	0	1	1	no

ENSG00000184361	ENSG00000184361	SPATA32	17:43268297-43394414	q1	q2	NOTEST	0	0.0279381	inf	0	1	1	no

ENSG00000184363	ENSG00000184363	PKP3	11:392613-404908	q1	q2	NOTEST	0.0181903	0	#NAME?	0	1	1	no

ENSG00000184368	ENSG00000184368	MAP7D2	X:20024830-20135035	q1	q2	OK	1.27588	1.12942	-0.175906	-0.192352	0.7336	0.99995	no

ENSG00000184371	ENSG00000184371	CSF1	1:110452863-110473614	q1	q2	OK	2.7671	2.31645	-0.256461	-0.31284	0.55165	0.99995	no

ENSG00000184374	ENSG00000184374	COLEC10	8:120007690-120118821	q1	q2	NOTEST	0.0469884	0.202213	2.1055	0	1	1	no

ENSG00000184378	ENSG00000184378	ACTRT3	3:169484708-169489831	q1	q2	NOTEST	0.240947	0.259664	0.107931	0	1	1	no

ENSG00000184381	ENSG00000184381	PLA2G6	22:38507501-38612518	q1	q2	OK	6.32023	5.31079	-0.25105	-0.212062	0.57405	0.99995	no

ENSG00000184384	ENSG00000184384	MAML2	11:95709761-96076344	q1	q2	OK	10.9156	11.1299	0.0280463	0.0667419	0.92085	0.99995	no

ENSG00000184385	ENSG00000184385	C21orf128	21:43483067-43563563	q1	q2	NOTEST	0	0	0	0	1	1	no

ENSG00000184388	ENSG00000184388	PABPC1L2B	X:72223351-72225551	q1	q2	NOTEST	0	0	0	0	1	1	no

ENSG00000184389	ENSG00000184389	A3GALT2	1:33772366-33896653	q1	q2	NOTEST	0.074285	0.179824	1.27544	0	1	1	no

ENSG00000184394	ENSG00000184394	OR4N5	14:20611894-20612821	q1	q2	NOTEST	0	0	0	0	1	1	no

ENSG00000184402	ENSG00000184402	SS18L1	20:60718821-60757540	q1	q2	OK	18.0386	18.9974	0.0747192	0.128217	0.79835	0.99995	no

ENSG00000184408	ENSG00000184408	KCND2	7:119913721-120392568	q1	q2	OK	1.64206	1.99461	0.280601	0.259839	0.6048	0.99995	no

ENSG00000184414	ENSG00000184414	RP11-44M6.3	7:100167753-100168759	q1	q2	NOTEST	0.0588884	0.116755	0.987432	0	1	1	no

ENSG00000184423	ENSG00000184423	AC090043.1	3:1637460-1637929	q1	q2	NOTEST	0	0	0	0	1	1	no

ENSG00000184428	ENSG00000184428	TOP1MT	8:144386553-144442149	q1	q2	OK	7.71368	8.62957	0.161869	0.23877	0.6896	0.99995	no

ENSG00000184432	ENSG00000184432	COPB2	3:138722803-139108574	q1	q2	OK	69.1333	71.8455	0.0555165	0.097606	0.8511	0.99995	no

ENSG00000184434	ENSG00000184434	LRRC19	9:26904080-27062928	q1	q2	NOTEST	0.340327	0.159991	-1.08893	0	1	1	no

ENSG00000184436	ENSG00000184436	THAP7	22:21353392-21399306	q1	q2	OK	16.4511	15.7653	-0.0614305	-0.0940702	0.8901	0.99995	no

ENSG00000184441	ENSG00000184441	AP001062.7	21:45748826-45760353	q1	q2	OK	1.64123	1.45165	-0.177086	-0.170899	0.81075	0.99995	no

ENSG00000184445	ENSG00000184445	KNTC1	12:123011792-123215390	q1	q2	OK	43.5685	43.6442	0.00250282	0.00360316	0.9953	0.99995	no

ENSG00000184451	ENSG00000184451	CCR10	17:40830906-40851832	q1	q2	OK	0.60149	0.587062	-0.0350271	-0.0131973	0.9841	0.99995	no

ENSG00000184454	ENSG00000184454	NCMAP	1:24882601-24935819	q1	q2	NOTEST	0.0126431	0.0244767	0.953059	0	1	1	no

ENSG00000184459	ENSG00000184459	BPIFC	22:32809833-32860471	q1	q2	NOTEST	0.0184383	0	#NAME?	0	1	1	no

ENSG00000184465	ENSG00000184465	WDR27	6:169857306-170102159	q1	q2	OK	34.2794	34.7247	0.0186209	0.0267292	0.962	0.99995	no

ENSG00000184470	ENSG00000184470	TXNRD2	22:19863039-20004331	q1	q2	OK	6.28571	6.04942	-0.0552789	-0.0365912	0.94795	0.99995	no

ENSG00000184471	ENSG00000184471	C1QTNF8	16:1140004-1146244	q1	q2	NOTEST	0.033259	0	#NAME?	0	1	1	no

ENSG00000184478	ENSG00000184478	OR56A3	11:5968569-5969591	q1	q2	NOTEST	0	0	0	0	1	1	no

ENSG00000184481	ENSG00000184481	FOXO4	X:70316046-70323385	q1	q2	OK	3.9609	2.91738	-0.441155	-0.521769	0.3579	0.99995	no

ENSG00000184486	ENSG00000184486	POU3F2	6:99282579-99286660	q1	q2	OK	141.344	141.326	-0.000184422	-0.000417712	0.99965	0.99995	no

ENSG00000184489	ENSG00000184489	PTP4A3	8:142400038-142441620	q1	q2	OK	9.45055	9.7041	0.0381957	0.0616125	0.93155	0.99995	no

ENSG00000184490	ENSG00000184490	AP000351.9	22:24366749-24370882	q1	q2	NOTEST	0	0	0	0	1	1	no

ENSG00000184492	ENSG00000184492	FOXD4L1	2:114256660-114258728	q1	q2	NOTEST	0.232817	0.205093	-0.182918	0	1	1	no

ENSG00000184497	ENSG00000184497	TMEM255B	13:114462215-114514926	q1	q2	OK	1.50782	0.907538	-0.732435	-0.638304	0.30505	0.99995	no

ENSG00000184500	ENSG00000184500	PROS1	3:93591880-93692910	q1	q2	OK	11.7503	12.157	0.0490866	0.0903012	0.88065	0.99995	no

ENSG00000184502	ENSG00000184502	GAST	17:39775688-39943183	q1	q2	NOTEST	0	0	0	0	1	1	no

ENSG00000184507	ENSG00000184507	NUTM1	15:34635515-34649938	q1	q2	NOTEST	0.0274684	0.0452787	0.721063	0	1	1	no

ENSG00000184508	ENSG00000184508	HDDC3	15:91473409-91506349	q1	q2	OK	6.19754	6.77588	0.128712	0.0784173	0.91595	0.99995	no

ENSG00000184515	ENSG00000184515	BEX5	X:101408679-101411029	q1	q2	NOTEST	0	0	0	0	1	1	no

ENSG00000184517	ENSG00000184517	ZFP1	16:75182389-75206134	q1	q2	OK	14.3968	17.695	0.297594	0.417243	0.3842	0.99995	no

ENSG00000184523	ENSG00000184523	PTGER4P2	9:66499742-66499994	q1	q2	NOTEST	0.378268	0	#NAME?	0	1	1	no

ENSG00000184524	ENSG00000184524	CEND1	11:787103-790123	q1	q2	OK	1.80742	1.60304	-0.173116	-0.184729	0.77935	0.99995	no

ENSG00000184530	ENSG00000184530	C6orf58	6:127840599-127912962	q1	q2	NOTEST	0.0474625	0.072363	0.608463	0	1	1	no

ENSG00000184544	ENSG00000184544	DHRS7C	17:9674750-9725388	q1	q2	NOTEST	0	0	0	0	1	1	no

ENSG00000184545	ENSG00000184545	DUSP8	11:1575273-1620414	q1	q2	OK	2.36048	1.97088	-0.260243	-0.372765	0.58735	0.99995	no

ENSG00000184551	ENSG00000184551	AC132872.1	17:80186272-80231607	q1	q2	OK	1.19168	0.99589	-0.258936	-0.0818698	0.89515	0.99995	no

ENSG00000184557	ENSG00000184557	SOCS3	17:76352863-76356158	q1	q2	OK	4.06477	4.31692	0.0868271	0.135027	0.84605	0.99995	no

ENSG00000184560	ENSG00000184560	C17orf74	17:7323421-7361026	q1	q2	NOTEST	0.155105	0.15419	-0.00854019	0	1	1	no

ENSG00000184564	ENSG00000184564	SLITRK6	13:86366924-86373623	q1	q2	NOTEST	0.101378	0.0762586	-0.410778	0	1	1	no

ENSG00000184566	ENSG00000184566	AC132216.1	11:33880121-33913836	q1	q2	NOTEST	0	0.0243459	inf	0	1	1	no

ENSG00000184571	ENSG00000184571	PIWIL3	22:25115000-25170687	q1	q2	NOTEST	0.0143638	0.0161656	0.170486	0	1	1	no

ENSG00000184574	ENSG00000184574	LPAR5	12:6728000-6745613	q1	q2	NOTEST	0.17429	0.12451	-0.485227	0	1	1	no

ENSG00000184575	ENSG00000184575	XPOT	12:64798129-64844907	q1	q2	OK	43.3162	44.4827	0.0383389	0.0626371	0.9178	0.99995	no

ENSG00000184584	ENSG00000184584	TMEM173	5:138855118-138862520	q1	q2	OK	2.7001	2.13099	-0.341488	-0.312788	0.5832	0.99995	no

ENSG00000184586	ENSG00000184586	KRTAP7-1	21:32201356-32202078	q1	q2	NOTEST	0.000118637	0	#NAME?	0	1	1	no

ENSG00000184588	ENSG00000184588	PDE4B	1:66258196-66840259	q1	q2	OK	43.386	47.9563	0.144488	0.317356	0.6277	0.99995	no

ENSG00000184596	ENSG00000184596	AF207550.1	X:48779304-48815648	q1	q2	NOTEST	0	0	0	0	1	1	no

ENSG00000184599	ENSG00000184599	FAM19A3	1:113263040-113269857	q1	q2	NOTEST	0.178538	0.100198	-0.833378	0	1	1	no

ENSG00000184601	ENSG00000184601	C14orf180	14:104941014-105071984	q1	q2	NOTEST	0.0100933	0	#NAME?	0	1	1	no

ENSG00000184602	ENSG00000184602	SNN	16:11762269-11838362	q1	q2	OK	80.3845	82.789	0.0425219	0.100073	0.89025	0.99995	no

ENSG00000184608	ENSG00000184608	C8orf12	8:11197145-11332224	q1	q2	NOTEST	0	0.06798	inf	0	1	1	no

ENSG00000184611	ENSG00000184611	KCNH7	2:163175349-163695240	q1	q2	OK	0.822501	0.989575	0.266792	0.174931	0.8467	0.99995	no

ENSG00000184612	ENSG00000184612	RPL7P26	6:17393446-17558023	q1	q2	NOTEST	0.26684	0.212462	-0.328772	0	1	1	no

ENSG00000184613	ENSG00000184613	NELL2	12:44902057-45315631	q1	q2	OK	43.0257	38.4692	-0.161494	-0.285506	0.5905	0.99995	no

ENSG00000184616	ENSG00000184616	AC004166.6	7:74306893-74366314	q1	q2	NOTEST	0	0	0	0	1	1	no

ENSG00000184617	ENSG00000184617	ZNF840	20:45113099-45121279	q1	q2	NOTEST	0.369729	0.334825	-0.143061	0	1	1	no

ENSG00000184619	ENSG00000184619	KRBA2	17:8268880-8286531	q1	q2	OK	3.48248	3.69615	0.0859087	0.0310787	0.9701	0.99995	no

ENSG00000184624	ENSG00000184624	ZNF72P	22:16634897-16651926	q1	q2	NOTEST	0	0	0	0	1	1	no

ENSG00000184634	ENSG00000184634	MED12	X:70338405-70362303	q1	q2	OK	16.3716	16.4281	0.0049747	0.00466381	0.9889	0.99995	no

ENSG00000184635	ENSG00000184635	ZNF93	19:20011721-20074273	q1	q2	OK	21.7494	21.2254	-0.0351816	-0.0619194	0.91405	0.99995	no

ENSG00000184640	ENSG00000184640	Sep-09	17:75253818-75496678	q1	q2	OK	250.687	285.554	0.187876	0.313753	0.54985	0.99995	no

ENSG00000184647	ENSG00000184647	PRSS55	8:10340387-10411676	q1	q2	NOTEST	0.0495368	0.0504999	0.0277787	0	1	1	no

ENSG00000184650	ENSG00000184650	ODF4	17:8243181-8249364	q1	q2	NOTEST	0	0	0	0	1	1	no

ENSG00000184659	ENSG00000184659	FOXD4L4	9:70426622-70429731	q1	q2	NOTEST	0.0412276	0.0830863	1.011	0	1	1	no

ENSG00000184661	ENSG00000184661	CDCA2	8:25316512-25365436	q1	q2	OK	15.3148	16.6169	0.117726	0.248144	0.70995	0.99995	no

ENSG00000184669	ENSG00000184669	OR7E14P	11:16799841-17074583	q1	q2	OK	3.70823	2.08821	-0.828462	-0.169448	0.5838	0.99995	no

ENSG00000184672	ENSG00000184672	RALYL	8:85095021-85834079	q1	q2	OK	2.11004	2.28522	0.115063	0.115423	0.85055	0.99995	no

ENSG00000184674	ENSG00000184674	GSTT1	22:24375561-24384680	q1	q2	NOTEST	0	0.0123378	inf	0	1	1	no

ENSG00000184675	ENSG00000184675	AMER1	X:63404996-63425624	q1	q2	OK	3.5902	3.7421	0.0597839	0.123392	0.8536	0.99995	no

ENSG00000184677	ENSG00000184677	ZBTB40	1:22778343-22857650	q1	q2	OK	9.76863	9.53626	-0.0347328	-0.0832017	0.9037	0.99995	no

ENSG00000184678	ENSG00000184678	HIST2H2BE	1:149856009-149858232	q1	q2	NOTEST	0.0618443	0.0365777	-0.757675	0	1	1	no

ENSG00000184682	ENSG00000184682	C11orf89	11:1874199-1913497	q1	q2	NOTEST	0.0637227	0.0305275	-1.0617	0	1	1	no

ENSG00000184697	ENSG00000184697	CLDN6	16:3064712-3072384	q1	q2	NOTEST	0.102622	0.160651	0.646588	0	1	1	no

ENSG00000184698	ENSG00000184698	OR51M1	11:5274419-5667019	q1	q2	NOTEST	0	0	0	0	1	1	no

ENSG00000184702	ENSG00000184702	Sep-05	22:19701986-19712295	q1	q2	OK	39.1471	36.8955	-0.0854584	-0.140454	0.82725	0.99995	no

ENSG00000184708	ENSG00000184708	EIF4ENIF1	22:31795508-32058418	q1	q2	OK	19.1951	19.9926	0.0587269	0.0535015	0.90915	0.99995	no

ENSG00000184709	ENSG00000184709	LRRC26	9:140063209-140066861	q1	q2	NOTEST	0.291366	0.20079	-0.537149	0	1	1	no

ENSG00000184716	ENSG00000184716	SERINC4	15:44019115-44095241	q1	q2	NOTEST	0.280291	0.244056	-0.199717	0	1	1	no

ENSG00000184719	ENSG00000184719	RNLS	10:90033620-90344287	q1	q2	NOTEST	0.407568	0.351328	-0.214222	0	1	1	no

ENSG00000184724	ENSG00000184724	KRTAP6-1	21:31985749-31986249	q1	q2	NOTEST	0	0.0784469	inf	0	1	1	no

ENSG00000184730	ENSG00000184730	APOBR	16:28467692-28510291	q1	q2	NOTEST	0.0744771	0.0602607	-0.30558	0	1	1	no

ENSG00000184731	ENSG00000184731	FAM110C	2:38813-46870	q1	q2	NOTEST	0.136901	0.0664485	-1.04282	0	1	1	no

ENSG00000184735	ENSG00000184735	DDX53	X:22277913-23311263	q1	q2	NOTEST	0	0	0	0	1	1	no

ENSG00000184741	ENSG00000184741	OR5AQ1P	11:55821839-55822779	q1	q2	NOTEST	0.0339885	0.0333464	-0.0275171	0	1	1	no

ENSG00000184743	ENSG00000184743	ATL3	11:63391558-63439393	q1	q2	OK	3.27636	3.10237	-0.0787272	-0.129548	0.83015	0.99995	no

ENSG00000184750	ENSG00000184750	MAGEA2	X:151918386-151922364	q1	q2	NOTEST	0	0	0	0	1	1	no

ENSG00000184752	ENSG00000184752	NDUFA12	12:95290830-95397546	q1	q2	OK	98.0484	103.976	0.0846856	0.181709	0.7893	0.99995	no

ENSG00000184761	ENSG00000184761	AC013269.5	2:131296667-131307456	q1	q2	NOTEST	0	0	0	0	1	1	no

ENSG00000184774	ENSG00000184774	RP11-480I12.4	1:202789393-202796353	q1	q2	NOTEST	0.210202	0.250269	0.251699	0	1	1	no

ENSG00000184779	ENSG00000184779	RPS17	15:82821157-82824972	q1	q2	OK	85.4503	194.081	1.1835	2.57273	0.00025	0.291517	no

ENSG00000184785	ENSG00000184785	SMIM10	X:134124967-134126501	q1	q2	NOTEST	0.341902	0.29513	-0.212235	0	1	1	no

ENSG00000184786	ENSG00000184786	TCTE3	6:170140209-170151655	q1	q2	NOTEST	0.426623	0.206782	-1.04485	0	1	1	no

ENSG00000184787	ENSG00000184787	UBE2G2	21:46188954-46221934	q1	q2	NOTEST	0	0	0	0	1	1	no

ENSG00000184788	ENSG00000184788	SATL1	X:84347292-84364054	q1	q2	OK	0.801671	0.69525	-0.205479	-0.228628	0.7395	0.99995	no

ENSG00000184789	ENSG00000184789	OR4C10P	11:48453768-48454699	q1	q2	NOTEST	0	0	0	0	1	1	no

ENSG00000184792	ENSG00000184792	OSBP2	22:31089768-31303811	q1	q2	OK	2.11771	2.29129	0.113655	0.106598	0.8283	0.99995	no

ENSG00000184795	ENSG00000184795	UNC93B5	11:67479172-67483476	q1	q2	NOTEST	0.100597	0.098347	-0.0326295	0	1	1	no

ENSG00000184809	ENSG00000184809	C21orf88	21:40928368-41098012	q1	q2	OK	2.13712	2.1982	0.0406554	0.0193448	0.98135	0.99995	no

ENSG00000184811	ENSG00000184811	TUSC5	17:1182956-1204281	q1	q2	NOTEST	0.0643973	0.0914654	0.506227	0	1	1	no

ENSG00000184814	ENSG00000184814	PRR23B	3:138722803-139108574	q1	q2	NOTEST	0	0	0	0	1	1	no

ENSG00000184825	ENSG00000184825	HIST1H2AH	6:27114860-27115317	q1	q2	OK	0.862239	0.277995	-1.63303	-0.957652	0.1994	0.99995	no

ENSG00000184828	ENSG00000184828	ZBTB7C	18:45553043-45937123	q1	q2	NOTEST	0.386502	0.20964	-0.882563	0	1	1	no

ENSG00000184831	ENSG00000184831	APOO	X:23851469-23957624	q1	q2	OK	24.7779	24.5805	-0.0115361	-0.0117471	0.9816	0.99995	no

ENSG00000184838	ENSG00000184838	PRR16	5:119799972-120023027	q1	q2	OK	0.500911	0.374609	-0.419169	-0.298668	0.59155	0.99995	no

ENSG00000184840	ENSG00000184840	TMED9	5:177019158-177023125	q1	q2	OK	103.917	96.4474	-0.107618	-0.261863	0.6989	0.99995	no

ENSG00000184844	ENSG00000184844	CYCSP45	X:153106705-153107020	q1	q2	NOTEST	0	0	0	0	1	1	no

ENSG00000184845	ENSG00000184845	DRD1	5:174867041-174871211	q1	q2	NOTEST	0.0560775	0.0362001	-0.631427	0	1	1	no

ENSG00000184856	ENSG00000184856	LINC00308	21:23470935-23488847	q1	q2	NOTEST	0	0	0	0	1	1	no

ENSG00000184857	ENSG00000184857	TMEM186	16:8768421-8962866	q1	q2	OK	10.3756	10.2856	-0.01257	-0.00423446	0.99375	0.99995	no

ENSG00000184860	ENSG00000184860	SDR42E1	16:82031220-82045093	q1	q2	NOTEST	0	0.00882584	inf	0	1	1	no

ENSG00000184863	ENSG00000184863	RBM33	7:155403924-155574179	q1	q2	OK	21.5198	19.7175	-0.126187	-0.205524	0.71105	0.99995	no

ENSG00000184867	ENSG00000184867	ARMCX2	X:100910266-100914876	q1	q2	OK	1.28408	1.30187	0.0198536	0.0185624	0.9702	0.99995	no

ENSG00000184879	ENSG00000184879	RP11-7G23.8	9:45733440-45734896	q1	q2	NOTEST	0	0	0	0	1	1	no

ENSG00000184881	ENSG00000184881	OR51B2	11:5274419-5667019	q1	q2	NOTEST	0	0	0	0	1	1	no

ENSG00000184886	ENSG00000184886	PIGW	17:34842472-34899284	q1	q2	NOTEST	0.0638729	0.123934	0.956294	0	1	1	no

ENSG00000184887	ENSG00000184887	BTBD6	14:105675622-105864484	q1	q2	OK	17.3524	16.7855	-0.0479205	-0.0711425	0.9212	0.99995	no

ENSG00000184895	ENSG00000184895	SRY	Y:2654895-2655740	q1	q2	OK	1.17257	1.1705	-0.0025512	-0.00203255	0.99645	0.99995	no

ENSG00000184897	ENSG00000184897	H1FX	3:129033614-129045068	q1	q2	OK	135.594	117.333	-0.208679	-0.50289	0.47035	0.99995	no

ENSG00000184898	ENSG00000184898	RBM43	2:152104453-152118393	q1	q2	OK	2.18539	2.14444	-0.0272866	-0.0346255	0.95305	0.99995	no

ENSG00000184900	ENSG00000184900	SUMO3	21:46225531-46238694	q1	q2	OK	59.41	72.2824	0.282939	0.678809	0.3328	0.99995	no

ENSG00000184903	ENSG00000184903	IMMP2L	7:110303109-111202573	q1	q2	OK	4.60008	5.18626	0.173036	0.0788114	0.89145	0.99995	no

ENSG00000184905	ENSG00000184905	TCEAL2	X:101380659-101382683	q1	q2	OK	0.546465	0.77495	0.503974	0.449291	0.4817	0.99995	no

ENSG00000184906	ENSG00000184906	AC129778.2	9:42845767-42876505	q1	q2	NOTEST	0	0.249019	inf	0	1	1	no

ENSG00000184908	ENSG00000184908	CLCNKB	1:16370271-16383803	q1	q2	NOTEST	0.12767	0.136594	0.0974795	0	1	1	no

ENSG00000184909	ENSG00000184909	C1ORF220	1:178482211-178518024	q1	q2	NOTEST	0.102983	0	#NAME?	0	1	1	no

ENSG00000184911	ENSG00000184911	DMRTC1B	X:71996832-72068636	q1	q2	NOTEST	0	0	0	0	1	1	no

ENSG00000184916	ENSG00000184916	JAG2	14:105607317-105647660	q1	q2	OK	3.68742	4.43427	0.266084	0.277957	0.651	0.99995	no

ENSG00000184922	ENSG00000184922	FMNL1	17:43268297-43394414	q1	q2	NOTEST	0.311724	0.346697	0.153405	0	1	1	no

ENSG00000184923	ENSG00000184923	NUTM2A	10:88963631-89102369	q1	q2	OK	0.85902	0.780728	-0.137872	-0.0827885	0.93585	0.99995	no

ENSG00000184924	ENSG00000184924	PTRHD1	2:25012602-25142708	q1	q2	OK	28.4459	25.3718	-0.164995	-0.119667	0.8381	0.99995	no

ENSG00000184925	ENSG00000184925	LCN12	9:139844002-139849949	q1	q2	OK	0.541564	0.433574	-0.320854	-0.150985	0.62425	0.99995	no

ENSG00000184933	ENSG00000184933	OR6A2	11:6815755-6817128	q1	q2	NOTEST	0	0.0210018	inf	0	1	1	no

ENSG00000184937	ENSG00000184937	WT1	11:32409320-32480315	q1	q2	NOTEST	0.0106784	0	#NAME?	0	1	1	no

ENSG00000184939	ENSG00000184939	ZFP90	16:68563992-68623415	q1	q2	OK	22.06	22.184	0.00808838	0.0120291	0.9766	0.99995	no

ENSG00000184945	ENSG00000184945	AQP12A	2:241631261-241637900	q1	q2	NOTEST	0	0	0	0	1	1	no

ENSG00000184949	ENSG00000184949	FAM227A	22:38974124-39052634	q1	q2	OK	4.09594	4.25076	0.0535272	0.0555652	0.87785	0.99995	no

ENSG00000184954	ENSG00000184954	OR6C70	12:55828520-55979255	q1	q2	NOTEST	0	0	0	0	1	1	no

ENSG00000184956	ENSG00000184956	MUC6	11:1012820-1036706	q1	q2	NOTEST	0.2554	0.143059	-0.836151	0	1	1	no

ENSG00000184961	ENSG00000184961	AL772307.1	9:42128838-42129419	q1	q2	NOTEST	0	0	0	0	1	1	no

ENSG00000184967	ENSG00000184967	NOC4L	12:132628992-132637013	q1	q2	OK	12.4773	10.6786	-0.224584	-0.389237	0.56715	0.99995	no

ENSG00000184979	ENSG00000184979	USP18	22:18632665-18660164	q1	q2	OK	1.22142	1.11405	-0.132751	-0.153665	0.82905	0.99995	no

ENSG00000184983	ENSG00000184983	NDUFA6	22:42481528-42535938	q1	q2	OK	0.596562	0.648005	0.119331	0.0541211	0.9296	0.99995	no

ENSG00000184984	ENSG00000184984	CHRM5	15:34158427-34357291	q1	q2	NOTEST	0.0783975	0.0865843	0.143298	0	1	1	no

ENSG00000184985	ENSG00000184985	SORCS2	4:7194264-7744554	q1	q2	OK	2.29999	1.11506	-1.04451	-0.827697	0.08145	0.99995	no

ENSG00000184986	ENSG00000184986	TMEM121	14:105992939-105996539	q1	q2	NOTEST	0	0.0548158	inf	0	1	1	no

ENSG00000184988	ENSG00000184988	TMEM106A	17:41363853-41383338	q1	q2	NOTEST	0.397521	0.408139	0.0380304	0	1	1	no

ENSG00000184990	ENSG00000184990	SIVA1	14:105219436-105234831	q1	q2	OK	69.8314	65.385	-0.0949161	-0.179291	0.79805	0.99995	no

ENSG00000184991	ENSG00000184991	TTTY13	Y:23745485-23756552	q1	q2	NOTEST	0	0.050894	inf	0	1	1	no

ENSG00000184992	ENSG00000184992	BRI3BP	12:125478245-125515777	q1	q2	OK	5.86693	6.51398	0.150935	0.330687	0.6389	0.99995	no

ENSG00000184995	ENSG00000184995	IFNE	9:21455482-21559668	q1	q2	NOTEST	0.0196832	0.0956269	2.28045	0	1	1	no

ENSG00000184999	ENSG00000184999	SLC22A10	11:62847411-63137190	q1	q2	NOTEST	0.0129955	0	#NAME?	0	1	1	no

ENSG00000185000	ENSG00000185000	DGAT1	8:145539953-145550573	q1	q2	OK	2.8402	5.4217	0.93275	0.700735	0.21435	0.99995	no

ENSG00000185002	ENSG00000185002	RFX6	6:117198374-117253326	q1	q2	NOTEST	0	0.013367	inf	0	1	1	no

ENSG00000185008	ENSG00000185008	ROBO2	3:75955845-77699115	q1	q2	OK	4.47536	3.9957	-0.163554	-0.151543	0.75945	0.99995	no

ENSG00000185009	ENSG00000185009	AP3M1	10:75881523-75910821	q1	q2	OK	34.4728	37.4311	0.118778	0.27172	0.682	0.99995	no

ENSG00000185010	ENSG00000185010	F8	X:154064062-154288578	q1	q2	NOTEST	0	0	0	0	1	1	no

ENSG00000185013	ENSG00000185013	NT5C1B	2:18735988-18770838	q1	q2	NOTEST	0	0.0816278	inf	0	1	1	no

ENSG00000185015	ENSG00000185015	CA13	8:86132815-86196302	q1	q2	OK	1.14613	1.02903	-0.15549	-0.116272	0.84205	0.99995	no

ENSG00000185019	ENSG00000185019	UBOX5	20:3087558-3140842	q1	q2	OK	3.56627	3.1403	-0.183515	-0.231191	0.73445	0.99995	no

ENSG00000185020	ENSG00000185020	RP11-111G23.1	9:40293024-40339528	q1	q2	OK	0.766726	0.793971	0.0503751	0.058063	0.9311	0.99995	no

ENSG00000185022	ENSG00000185022	MAFF	22:38507501-38612518	q1	q2	OK	3.16288	3.05413	-0.0504761	-0.0534125	0.9343	0.99995	no

ENSG00000185024	ENSG00000185024	BRF1	14:105675622-105864484	q1	q2	OK	15.8329	13.4724	-0.232924	-0.322805	0.6124	0.99995	no

ENSG00000185028	ENSG00000185028	LRRC14B	5:191625-195468	q1	q2	NOTEST	0.0364663	0.0347469	-0.0696795	0	1	1	no

ENSG00000185031	ENSG00000185031	SLC2A3P2	1:65437907-65468159	q1	q2	NOTEST	0.130797	0.127404	-0.037917	0	1	1	no

ENSG00000185033	ENSG00000185033	SEMA4B	15:90703835-90772911	q1	q2	OK	9.00275	7.23955	-0.314465	-0.41716	0.33795	0.99995	no

ENSG00000185037	ENSG00000185037	ZNF733P	7:62751895-62764434	q1	q2	NOTEST	0	0	0	0	1	1	no

ENSG00000185038	ENSG00000185038	MROH2A	2:234684369-234763212	q1	q2	NOTEST	0	0.0780691	inf	0	1	1	no

ENSG00000185040	ENSG00000185040	AC004980.11	7:76139744-77045717	q1	q2	NOTEST	0	0	0	0	1	1	no

ENSG00000185041	ENSG00000185041	RP11-321E2.6	5:17498333-17498936	q1	q2	NOTEST	0	0	0	0	1	1	no

ENSG00000185043	ENSG00000185043	CIB1	15:90773206-90785315	q1	q2	OK	15.2608	13.7016	-0.15548	-0.266946	0.70825	0.99995	no

ENSG00000185044	ENSG00000185044	RP11-435B5.4	1:143347957-143467632	q1	q2	NOTEST	0.0613712	0.0517219	-0.246786	0	1	1	no

ENSG00000185046	ENSG00000185046	ANKS1B	12:99038918-100378432	q1	q2	OK	25.8885	25.5647	-0.0181597	-0.0180747	0.97035	0.99995	no

ENSG00000185049	ENSG00000185049	NELFA	4:1984440-2043630	q1	q2	OK	22.9705	21.313	-0.108048	-0.207446	0.74165	0.99995	no

ENSG00000185052	ENSG00000185052	SLC24A3	20:19193289-19703581	q1	q2	OK	0.575786	0.566877	-0.022498	-0.025667	0.9688	0.99995	no

ENSG00000185053	ENSG00000185053	SGCZ	8:13947372-15095848	q1	q2	NOTEST	0.0751827	0	#NAME?	0	1	1	no

ENSG00000185055	ENSG00000185055	EFCAB10	7:105172531-105241322	q1	q2	OK	0.841025	0.6184	-0.443608	-0.118509	0.70405	0.99995	no

ENSG00000185056	ENSG00000185056	C5orf47	5:173400781-173433143	q1	q2	NOTEST	0.0102194	0	#NAME?	0	1	1	no

ENSG00000185065	ENSG00000185065	AC000068.5	22:19318220-19466738	q1	q2	OK	1.06505	0.907459	-0.231022	-0.0587703	0.9154	0.99995	no

ENSG00000185069	ENSG00000185069	KRT76	12:53161938-53171129	q1	q2	NOTEST	0	0	0	0	1	1	no

ENSG00000185070	ENSG00000185070	FLRT2	14:85996487-86095034	q1	q2	OK	15.4836	24.7237	0.67515	0.794769	0.03675	0.99995	no

ENSG00000185074	ENSG00000185074	OR7E31P	9:92978320-92979206	q1	q2	NOTEST	0	0	0	0	1	1	no

ENSG00000185078	ENSG00000185078	AC093698.1	2:208896106-208896677	q1	q2	NOTEST	0	0	0	0	1	1	no

ENSG00000185085	ENSG00000185085	INTS5	11:62414319-62441159	q1	q2	OK	9.56473	8.08409	-0.242639	-0.338499	0.6246	0.99995	no

ENSG00000185087	ENSG00000185087	FAM169B	15:98980390-99057611	q1	q2	NOTEST	0.00809998	0.00784536	-0.0460796	0	1	1	no

ENSG00000185088	ENSG00000185088	RPS27L	15:63413998-63450220	q1	q2	OK	140.269	124.182	-0.175743	-0.32893	0.63775	0.99995	no

ENSG00000185090	ENSG00000185090	MANEAL	1:38259473-38266809	q1	q2	OK	19.2306	16.6871	-0.204678	-0.426251	0.53675	0.99995	no

ENSG00000185097	ENSG00000185097	OR4F16	1:536815-660283	q1	q2	NOTEST	0	0	0	0	1	1	no

ENSG00000185100	ENSG00000185100	ADSSL1	14:105190522-105213662	q1	q2	OK	3.14879	1.96297	-0.681756	-0.718036	0.2165	0.99995	no

ENSG00000185101	ENSG00000185101	ANO9	11:417932-442011	q1	q2	NOTEST	0.0336148	0.0117552	-1.5158	0	1	1	no

ENSG00000185104	ENSG00000185104	FAF1	1:50905149-51425935	q1	q2	OK	19.01	20.1404	0.0833303	0.144396	0.8243	0.99995	no

ENSG00000185105	ENSG00000185105	MYADML2	17:79890259-79905477	q1	q2	NOTEST	0.197062	0.19742	0.00261472	0	1	1	no

ENSG00000185112	ENSG00000185112	FAM43A	3:194406621-194409762	q1	q2	OK	3.07063	2.26289	-0.440369	-0.624974	0.36645	0.99995	no

ENSG00000185115	ENSG00000185115	NDNL2	15:29412456-29862927	q1	q2	OK	21.405	20.9055	-0.0340655	-0.0647257	0.92545	0.99995	no

ENSG00000185122	ENSG00000185122	HSF1	8:145515279-145538801	q1	q2	OK	8.7925	1.23817	-2.82807	-2.52697	0.00095	0.553882	no

ENSG00000185127	ENSG00000185127	C6orf120	6:170102232-170124151	q1	q2	OK	18.8345	19.7342	0.067315	0.0826016	0.9071	0.99995	no

ENSG00000185128	ENSG00000185128	TBC1D3F	17:36283970-36294915	q1	q2	OK	3.96104	3.54242	-0.161145	-0.241143	0.7426	0.99995	no

ENSG00000185129	ENSG00000185129	PURA	5:139452441-139496321	q1	q2	OK	6.04985	6.25401	0.0478828	0.0717793	0.90355	0.99995	no

ENSG00000185130	ENSG00000185130	HIST1H2BL	6:27775256-27775709	q1	q2	OK	0.782177	0.381259	-1.03672	-0.577727	0.3731	0.99995	no

ENSG00000185133	ENSG00000185133	INPP5J	22:31518716-31530682	q1	q2	OK	2.88819	2.13098	-0.438647	-0.571343	0.365	0.99995	no

ENSG00000185149	ENSG00000185149	NPY2R	4:156127680-156138230	q1	q2	NOTEST	0.107274	0.122853	0.19564	0	1	1	no

ENSG00000185155	ENSG00000185155	MIXL1	1:226411318-226414755	q1	q2	NOTEST	0.305489	0.21912	-0.479396	0	1	1	no

ENSG00000185156	ENSG00000185156	MFSD6L	17:8700435-8702667	q1	q2	NOTEST	0	0	0	0	1	1	no

ENSG00000185158	ENSG00000185158	LRRC37B	17:30334890-30380523	q1	q2	OK	16.5623	17.6602	0.092599	0.128698	0.84515	0.99995	no

ENSG00000185162	ENSG00000185162	AP001324.1	11:74407473-74442430	q1	q2	NOTEST	0	0	0	0	1	1	no

ENSG00000185163	ENSG00000185163	DDX51	12:132621138-132628880	q1	q2	OK	11.3695	9.52259	-0.255742	-0.410483	0.5036	0.99995	no

ENSG00000185164	ENSG00000185164	NOMO2	16:18511181-18573533	q1	q2	OK	7.48523	8.3594	0.159354	0.309814	0.6279	0.99995	no

ENSG00000185168	ENSG00000185168	LINC00482	17:79277570-79283048	q1	q2	NOTEST	0.155785	0.0514184	-1.5992	0	1	1	no

ENSG00000185176	ENSG00000185176	AQP12B	2:241615834-241622323	q1	q2	NOTEST	0	0	0	0	1	1	no

ENSG00000185177	ENSG00000185177	ZNF479	7:57187320-57207571	q1	q2	NOTEST	0	0	0	0	1	1	no

ENSG00000185182	ENSG00000185182	GOLGA8DP	15:22704720-22715604	q1	q2	NOTEST	0	0	0	0	1	1	no

ENSG00000185186	ENSG00000185186	LINC00313	21:44881973-44899414	q1	q2	NOTEST	0.0972269	0.0618553	-0.652457	0	1	1	no

ENSG00000185187	ENSG00000185187	SIGIRR	11:405715-417455	q1	q2	OK	1.59348	2.12679	0.416494	0.363232	0.5108	0.99995	no

ENSG00000185189	ENSG00000185189	NRBP2	8:144915439-144924200	q1	q2	NOTEST	0.157698	0.215787	0.452442	0	1	1	no

ENSG00000185198	ENSG00000185198	PRSS57	19:685520-695460	q1	q2	NOTEST	0	0	0	0	1	1	no

ENSG00000185201	ENSG00000185201	IFITM2	11:307630-315272	q1	q2	OK	42.4867	40.8886	-0.0553118	-0.0847255	0.89915	0.99995	no

ENSG00000185203	ENSG00000185203	WASIR1	X:155227245-155257848	q1	q2	NOTEST	0	0	0	0	1	1	no

ENSG00000185215	ENSG00000185215	TNFAIP2	14:103589778-103603776	q1	q2	OK	6.64606	3.8392	-0.791695	-0.71567	0.2054	0.99995	no

ENSG00000185219	ENSG00000185219	ZNF445	3:44481261-44519162	q1	q2	OK	12.8256	12.7491	-0.00863211	-0.0176592	0.9758	0.99995	no

ENSG00000185220	ENSG00000185220	PGBD2	1:249200394-249214145	q1	q2	OK	5.89067	6.28718	0.0939798	0.153187	0.8226	0.99995	no

ENSG00000185221	ENSG00000185221	GAPDHP24	1:215044076-215045059	q1	q2	NOTEST	0	0	0	0	1	1	no

ENSG00000185222	ENSG00000185222	WBP5	X:102611372-102613397	q1	q2	OK	89.0997	93.8735	0.0752974	0.177467	0.8001	0.99995	no

ENSG00000185231	ENSG00000185231	MC2R	18:13882042-13915706	q1	q2	NOTEST	0	0	0	0	1	1	no

ENSG00000185236	ENSG00000185236	RAB11B	19:8428172-8469318	q1	q2	OK	88.1114	78.8474	-0.160266	-0.357705	0.60585	0.99995	no

ENSG00000185238	ENSG00000185238	PRMT3	11:20409075-20530840	q1	q2	OK	9.76409	9.55665	-0.0309808	-0.0587082	0.9296	0.99995	no

ENSG00000185245	ENSG00000185245	GP1BA	17:4835591-4838325	q1	q2	OK	0.455215	0.541571	0.250603	0.25293	0.7257	0.99995	no

ENSG00000185246	ENSG00000185246	PRPF39	14:45553301-45604522	q1	q2	OK	29.8716	31.8195	0.0911358	0.110156	0.85405	0.99995	no

ENSG00000185247	ENSG00000185247	MAGEA11	X:148769893-148798926	q1	q2	NOTEST	0	0	0	0	1	1	no

ENSG00000185250	ENSG00000185250	PPIL6	6:109711417-109765122	q1	q2	OK	1.23932	1.8198	0.554223	0.314835	0.53635	0.99995	no

ENSG00000185252	ENSG00000185252	ZNF74	22:20748404-20762745	q1	q2	OK	18.9446	18.0902	-0.0665776	-0.150462	0.82765	0.99995	no

ENSG00000185254	ENSG00000185254	TEX28	X:153498929-153523796	q1	q2	NOTEST	0	0	0	0	1	1	no

ENSG00000185261	ENSG00000185261	KIAA0825	5:93488670-94620279	q1	q2	OK	1.19725	1.23227	0.0415923	0.020157	0.9827	0.99995	no

ENSG00000185262	ENSG00000185262	UBALD2	17:74261282-74267375	q1	q2	OK	21.6797	16.9644	-0.353833	-0.575298	0.33585	0.99995	no

ENSG00000185264	ENSG00000185264	TEX33	22:37387162-37403882	q1	q2	NOTEST	0.0913209	0	#NAME?	0	1	1	no

ENSG00000185267	ENSG00000185267	CDNF	10:14861248-14913740	q1	q2	OK	1.42194	0.674084	-1.07686	-0.303539	0.4007	0.99995	no

ENSG00000185269	ENSG00000185269	NOTUM	17:79910387-79919716	q1	q2	NOTEST	0	0.0108329	inf	0	1	1	no

ENSG00000185271	ENSG00000185271	KLHL33	14:20896969-20903801	q1	q2	NOTEST	0	0.0138519	inf	0	1	1	no

ENSG00000185272	ENSG00000185272	RBM11	21:15588450-15600693	q1	q2	OK	1.54526	1.39973	-0.142693	-0.131838	0.8406	0.99995	no

ENSG00000185274	ENSG00000185274	WBSCR17	7:70597154-71178585	q1	q2	OK	4.05521	3.48237	-0.21971	-0.366232	0.5943	0.99995	no

ENSG00000185275	ENSG00000185275	CD24P4	Y:21034386-21239302	q1	q2	OK	15.8373	14.8786	-0.090083	-0.0474598	0.9465	0.99995	no

ENSG00000185278	ENSG00000185278	ZBTB37	1:173832385-173872687	q1	q2	OK	3.94191	4.25133	0.109021	0.120588	0.8106	0.99995	no

ENSG00000185290	ENSG00000185290	NUPR1L	7:56182373-56184093	q1	q2	NOTEST	0	0	0	0	1	1	no

ENSG00000185291	ENSG00000185291	IL3RA	X:1455508-1501578	q1	q2	OK	0.612496	0.638507	0.0600017	0.0508643	0.9361	0.99995	no

ENSG00000185294	ENSG00000185294	SPPL2C	17:43921016-44105700	q1	q2	NOTEST	0	0	0	0	1	1	no

ENSG00000185296	ENSG00000185296	CTD-2139B15.1	5:17306489-17307069	q1	q2	NOTEST	0	0	0	0	1	1	no

ENSG00000185298	ENSG00000185298	CCDC137	17:79632065-79640934	q1	q2	OK	15.5732	14.6015	-0.0929501	-0.0814023	0.8219	0.99995	no

ENSG00000185303	ENSG00000185303	SFTPA2	10:81315607-81320153	q1	q2	NOTEST	0.0204394	0	#NAME?	0	1	1	no

ENSG00000185304	ENSG00000185304	RGPD2	2:88047605-88285309	q1	q2	NOTEST	0.173943	0.111323	-0.643858	0	1	1	no

ENSG00000185305	ENSG00000185305	ARL15	5:53179774-53606412	q1	q2	OK	11.646	13.7985	0.244684	0.303676	0.64475	0.99995	no

ENSG00000185306	ENSG00000185306	C12orf56	12:64616116-64790845	q1	q2	NOTEST	0.0082228	0.0556785	2.75942	0	1	1	no

ENSG00000185313	ENSG00000185313	SCN10A	3:38738292-38835501	q1	q2	NOTEST	0	0	0	0	1	1	no

ENSG00000185319	ENSG00000185319	SSXP4	X:52627543-52632339	q1	q2	NOTEST	0	0	0	0	1	1	no

ENSG00000185324	ENSG00000185324	CDK10	16:89747144-89768113	q1	q2	OK	42.8604	39.5679	-0.115315	-0.185057	0.7525	0.99995	no

ENSG00000185332	ENSG00000185332	TMEM105	17:79285073-79304474	q1	q2	NOTEST	0.0312752	0.0613796	0.972738	0	1	1	no

ENSG00000185338	ENSG00000185338	SOCS1	16:11343475-11445619	q1	q2	OK	2.06953	1.55651	-0.410986	-0.209014	0.77445	0.99995	no

ENSG00000185339	ENSG00000185339	TCN2	22:30972611-31023265	q1	q2	OK	14.328	14.0075	-0.0326367	-0.0330987	0.95865	0.99995	no

ENSG00000185340	ENSG00000185340	GAS2L1	22:29702571-29708774	q1	q2	OK	6.83816	5.68361	-0.2668	-0.413498	0.5308	0.99995	no

ENSG00000185344	ENSG00000185344	ATP6V0A2	12:124196864-124246302	q1	q2	OK	15.2037	16.8078	0.144714	0.218648	0.7217	0.99995	no

ENSG00000185345	ENSG00000185345	PARK2	6:161768451-163736524	q1	q2	OK	0.510981	0.571915	0.162533	0.147587	0.80785	0.99995	no

ENSG00000185347	ENSG00000185347	C14orf80	14:105952653-105965912	q1	q2	OK	2.28272	0.850835	-1.4238	-1.11341	0.08545	0.99995	no

ENSG00000185352	ENSG00000185352	HS6ST3	13:96743092-97485671	q1	q2	NOTEST	0.150843	0.228867	0.601454	0	1	1	no

ENSG00000185359	ENSG00000185359	HGS	17:79648203-79670330	q1	q2	OK	34.8374	34.0459	-0.0331585	-0.0406971	0.9235	0.99995	no

ENSG00000185361	ENSG00000185361	TNFAIP8L1	19:4639529-4670382	q1	q2	OK	5.74298	5.15077	-0.157013	-0.187641	0.78155	0.99995	no

ENSG00000185372	ENSG00000185372	OR2V1	5:180551356-180552304	q1	q2	NOTEST	0	0.0328798	inf	0	1	1	no

ENSG00000185379	ENSG00000185379	RAD51D	17:33332106-33448541	q1	q2	OK	6.63372	7.11744	0.101542	0.0862465	0.8884	0.99995	no

ENSG00000185385	ENSG00000185385	OR7A17	19:14991137-14992264	q1	q2	NOTEST	0	0	0	0	1	1	no

ENSG00000185386	ENSG00000185386	MAPK11	22:50702141-50709196	q1	q2	OK	22.7002	22.4545	-0.0157042	-0.0321722	0.96	0.99995	no

ENSG00000185390	ENSG00000185390	FGF7P2	21:14721585-14722969	q1	q2	NOTEST	0	0	0	0	1	1	no

ENSG00000185404	ENSG00000185404	SP140L	2:230990025-231268447	q1	q2	OK	1.24315	0.770118	-0.690846	-0.483019	0.3811	0.99995	no

ENSG00000185414	ENSG00000185414	MRPL30	2:99613723-100106497	q1	q2	OK	1.36328	4.90892	1.84833	0.332117	0.1773	0.99995	no

ENSG00000185418	ENSG00000185418	TARSL2	15:102193800-102264807	q1	q2	OK	8.18757	8.85261	0.112668	0.173478	0.75925	0.99995	no

ENSG00000185420	ENSG00000185420	SMYD3	1:245912641-246670614	q1	q2	OK	14.4225	13.6874	-0.0754687	-0.116927	0.8601	0.99995	no

ENSG00000185432	ENSG00000185432	METTL7A	12:51317254-51326300	q1	q2	OK	4.14067	4.07204	-0.0241118	-0.0345042	0.95745	0.99995	no

ENSG00000185433	ENSG00000185433	LINC00158	21:26758132-26804013	q1	q2	NOTEST	0.223222	0.288016	0.367667	0	1	1	no

ENSG00000185436	ENSG00000185436	IFNLR1	1:24480646-24514449	q1	q2	NOTEST	0.0278694	0.0113535	-1.29555	0	1	1	no

ENSG00000185437	ENSG00000185437	SH3BGR	21:40817780-40887433	q1	q2	OK	2.34619	2.33097	-0.00939284	-0.00862308	0.98995	0.99995	no

ENSG00000185442	ENSG00000185442	FAM174B	15:93146139-93353114	q1	q2	OK	6.08364	3.49532	-0.799509	-0.529807	0.23675	0.99995	no

ENSG00000185448	ENSG00000185448	FAM47A	X:34147868-34150447	q1	q2	NOTEST	0	0	0	0	1	1	no

ENSG00000185453	ENSG00000185453	C19orf68	19:48673948-48790865	q1	q2	OK	4.00888	4.01025	0.000491764	0.000364156	0.9994	0.99995	no

ENSG00000185467	ENSG00000185467	KPNA7	7:98771196-98805129	q1	q2	NOTEST	0.0155608	0	#NAME?	0	1	1	no

ENSG00000185475	ENSG00000185475	TMEM179B	11:62538591-62559493	q1	q2	OK	15.2624	14.7628	-0.0480081	-0.0486962	0.95805	0.99995	no

ENSG00000185477	ENSG00000185477	GPRIN3	4:90157536-90229161	q1	q2	OK	0.456009	0.546166	0.260278	0.383194	0.5779	0.99995	no

ENSG00000185479	ENSG00000185479	KRT6B	12:52840434-52845910	q1	q2	NOTEST	0	0.0118476	inf	0	1	1	no

ENSG00000185480	ENSG00000185480	PARPBP	12:102513955-102591623	q1	q2	OK	17.3121	19.0769	0.140053	0.262262	0.70485	0.99995	no

ENSG00000185482	ENSG00000185482	STAC3	12:57637235-57824788	q1	q2	OK	1.44813	1.59708	0.141239	0.0481003	0.8886	0.99995	no

ENSG00000185483	ENSG00000185483	ROR1	1:64239692-64647181	q1	q2	OK	4.30086	5.01763	0.22238	0.382957	0.59285	0.99995	no

ENSG00000185485	ENSG00000185485	SDHAP1	3:195686618-195717189	q1	q2	OK	20.6543	17.1054	-0.27199	-0.361236	0.62885	0.99995	no

ENSG00000185495	ENSG00000185495	RP11-504P24.4	1:224183596-224198314	q1	q2	OK	1.67815	1.66165	-0.0142558	-0.0154148	0.9788	0.99995	no

ENSG00000185499	ENSG00000185499	MUC1	1:155141883-155162707	q1	q2	OK	6.14838	4.44168	-0.469101	-0.272638	0.53385	0.99995	no

ENSG00000185504	ENSG00000185504	C17orf70	17:79506910-79520987	q1	q2	OK	15.9218	14.6785	-0.1173	-0.228419	0.73945	0.99995	no

ENSG00000185507	ENSG00000185507	IRF7	11:612552-615999	q1	q2	OK	5.66493	4.36894	-0.374776	-0.456766	0.4745	0.99995	no

ENSG00000185513	ENSG00000185513	L3MBTL1	20:42136319-42179590	q1	q2	OK	8.68446	7.03285	-0.304325	-0.323818	0.4669	0.99995	no

ENSG00000185515	ENSG00000185515	BRCC3	X:154289896-154384778	q1	q2	NOTEST	0.108301	0.106002	-0.0309617	0	1	1	no

ENSG00000185518	ENSG00000185518	SV2B	15:91643179-91844539	q1	q2	NOTEST	0.0122226	0.0280545	1.19868	0	1	1	no

ENSG00000185519	ENSG00000185519	FAM131C	1:16384263-16400127	q1	q2	NOTEST	0.116332	0.127626	0.133675	0	1	1	no

ENSG00000185522	ENSG00000185522	C11orf35	11:537526-564021	q1	q2	OK	0.698736	0.283429	-1.30176	-0.384348	0.33595	0.99995	no

ENSG00000185523	ENSG00000185523	C1orf227	1:213003482-213020991	q1	q2	NOTEST	0.392043	0.153775	-1.35019	0	1	1	no

ENSG00000185527	ENSG00000185527	PDE6G	17:79617488-79630142	q1	q2	NOTEST	0.321716	0.343525	0.0946302	0	1	1	no

ENSG00000185532	ENSG00000185532	PRKG1	10:52750944-54073888	q1	q2	OK	4.45508	5.31208	0.253824	0.241337	0.71455	0.99995	no

ENSG00000185551	ENSG00000185551	NR2F2	15:96670597-96883492	q1	q2	OK	41.3921	41.1605	-0.00809622	-0.0174211	0.9806	0.99995	no

ENSG00000185554	ENSG00000185554	NXF2	X:101470279-101581634	q1	q2	NOTEST	0	0	0	0	1	1	no

ENSG00000185559	ENSG00000185559	DLK1	14:101192041-101201539	q1	q2	OK	1.23375	1.05409	-0.227052	-0.215155	0.7143	0.99995	no

ENSG00000185561	ENSG00000185561	TLCD2	17:1611063-1613732	q1	q2	NOTEST	0.275066	0.181186	-0.602307	0	1	1	no

ENSG00000185565	ENSG00000185565	LSAMP	3:115521234-117716439	q1	q2	OK	15.6891	15.6106	-0.00723559	-0.0110116	0.98295	0.99995	no

ENSG00000185567	ENSG00000185567	AHNAK2	14:105403580-105444694	q1	q2	OK	1.43657	1.06868	-0.426804	-0.467669	0.50785	0.99995	no

ENSG00000185585	ENSG00000185585	OLFML2A	9:127539436-127577164	q1	q2	OK	3.79426	3.15449	-0.266413	-0.404733	0.50725	0.99995	no

ENSG00000185591	ENSG00000185591	SP1	12:53773959-53810230	q1	q2	OK	22.5814	25.6469	0.183648	0.330186	0.51365	0.99995	no

ENSG00000185594	ENSG00000185594	SPATA8	15:97326618-97328845	q1	q2	NOTEST	0.163872	0.125626	-0.383439	0	1	1	no

ENSG00000185596	ENSG00000185596	WASH3P	15:102500050-102519298	q1	q2	OK	8.68968	7.28268	-0.254834	-0.348141	0.5552	0.99995	no

ENSG00000185607	ENSG00000185607	ACTBP7	15:44162961-44487450	q1	q2	NOTEST	0.131276	0	#NAME?	0	1	1	no

ENSG00000185608	ENSG00000185608	MRPL40	22:19318220-19466738	q1	q2	OK	20.7914	20.2151	-0.0405556	-0.0305973	0.9598	0.99995	no

ENSG00000185610	ENSG00000185610	DBX2	12:45408454-45496890	q1	q2	NOTEST	0.0854085	0.0624032	-0.452761	0	1	1	no

ENSG00000185614	ENSG00000185614	FAM212A	3:49840686-49842463	q1	q2	OK	4.06216	4.31617	0.0875041	0.099301	0.8875	0.99995	no

ENSG00000185615	ENSG00000185615	PDIA2	16:333151-337215	q1	q2	OK	0.699163	0.539477	-0.374068	-0.246145	0.70855	0.99995	no

ENSG00000185619	ENSG00000185619	PCGF3	4:699536-775637	q1	q2	OK	35.8945	35.2504	-0.0261232	-0.0477793	0.93595	0.99995	no

ENSG00000185621	ENSG00000185621	LMLN	3:197687070-197770591	q1	q2	OK	3.90226	4.43829	0.185695	0.252864	0.5891	0.99995	no

ENSG00000185624	ENSG00000185624	P4HB	17:79801034-79818570	q1	q2	OK	217.134	194.735	-0.157071	-0.320945	0.59045	0.99995	no

ENSG00000185627	ENSG00000185627	PSMD13	11:215457-252983	q1	q2	OK	89.7218	87.1231	-0.0424048	-0.0861788	0.8962	0.99995	no

ENSG00000185630	ENSG00000185630	PBX1	1:164524820-164868533	q1	q2	OK	51.8361	52.1598	0.00898184	0.0166602	0.9768	0.99995	no

ENSG00000185631	ENSG00000185631	AC017079.3	2:128960934-128961349	q1	q2	NOTEST	0.116389	0.112346	-0.0510098	0	1	1	no

ENSG00000185633	ENSG00000185633	NDUFA4L2	12:57623109-57634498	q1	q2	OK	1.63174	1.04621	-0.64123	-0.12932	0.5909	0.99995	no

ENSG00000185634	ENSG00000185634	SHC4	15:49115931-49255641	q1	q2	OK	1.23564	1.53596	0.313877	0.0638006	0.777	0.99995	no

ENSG00000185638	ENSG00000185638	FTH1P14	X:34165152-34165703	q1	q2	NOTEST	0	0	0	0	1	1	no

ENSG00000185640	ENSG00000185640	KRT79	12:53215193-53228079	q1	q2	NOTEST	0	0	0	0	1	1	no

ENSG00000185641	ENSG00000185641	CTD-2287O16.1	5:115298150-115395815	q1	q2	OK	99.4303	97.1759	-0.0330868	-0.0600985	0.9347	0.99995	no

ENSG00000185650	ENSG00000185650	ZFP36L1	14:69254376-69263190	q1	q2	OK	113.131	100.016	-0.177775	-0.426941	0.5366	0.99995	no

ENSG00000185651	ENSG00000185651	UBE2L3	22:21899632-21978323	q1	q2	OK	67.6005	68.6505	0.0222364	0.04903	0.94195	0.99995	no

ENSG00000185652	ENSG00000185652	NTF3	12:5541277-5630702	q1	q2	OK	0.508761	0.263892	-0.947044	-0.500911	0.2911	0.99995	no

ENSG00000185658	ENSG00000185658	BRWD1	21:40556101-40695144	q1	q2	OK	18.4163	18.6345	0.0169964	0.0265384	0.96645	0.99995	no

ENSG00000185662	ENSG00000185662	SMIM23	5:171212875-171221602	q1	q2	NOTEST	0	0	0	0	1	1	no

ENSG00000185664	ENSG00000185664	PMEL	12:56347888-56367101	q1	q2	OK	7.18239	6.16023	-0.221479	-0.111276	0.7728	0.99995	no

ENSG00000185666	ENSG00000185666	SYN3	22:32908538-33454358	q1	q2	OK	0.499489	0.571229	0.193615	0.0303908	0.89425	0.99995	no

ENSG00000185668	ENSG00000185668	POU3F1	1:38509522-38512450	q1	q2	OK	1.62786	1.55341	-0.0675349	-0.0870008	0.9023	0.99995	no

ENSG00000185669	ENSG00000185669	SNAI3	16:88729705-88753594	q1	q2	OK	0.843865	1.07677	0.351624	0.192332	0.7872	0.99995	no

ENSG00000185670	ENSG00000185670	ZBTB3	11:62515790-62521660	q1	q2	OK	4.13284	3.90671	-0.0811814	-0.0666969	0.91525	0.99995	no

ENSG00000185674	ENSG00000185674	LYG2	2:99613723-100106497	q1	q2	NOTEST	0.219224	0.255793	0.222576	0	1	1	no

ENSG00000185681	ENSG00000185681	MORN5	9:124922189-124962367	q1	q2	NOTEST	0.149095	0.247478	0.731065	0	1	1	no

ENSG00000185684	ENSG00000185684	EP400NL	12:132568827-132613029	q1	q2	OK	7.98267	6.58837	-0.27695	-0.322398	0.6147	0.99995	no

ENSG00000185686	ENSG00000185686	PRAME	22:22890122-22909007	q1	q2	NOTEST	0	0.0116157	inf	0	1	1	no

ENSG00000185689	ENSG00000185689	C6orf201	6:4021500-4157619	q1	q2	NOTEST	0.431776	0.286638	-0.591052	0	1	1	no

ENSG00000185697	ENSG00000185697	MYBL1	8:67474409-67526482	q1	q2	OK	6.88913	7.92721	0.202492	0.357995	0.57495	0.99995	no

ENSG00000185700	ENSG00000185700	TTTY8B	Y:6338813-6341671	q1	q2	NOTEST	0	0	0	0	1	1	no

ENSG00000185701	ENSG00000185701	OR5M5P	11:56294043-56294979	q1	q2	NOTEST	0	0	0	0	1	1	no

ENSG00000185710	ENSG00000185710	RP11-645C24.2	16:21845889-21930477	q1	q2	OK	0.652426	1.16377	0.83492	0.386563	0.4233	0.99995	no

ENSG00000185716	ENSG00000185716	C16orf52	16:22018958-22103552	q1	q2	OK	7.99351	10.2179	0.354195	0.495207	0.3939	0.99995	no

ENSG00000185721	ENSG00000185721	DRG1	22:31795508-32058418	q1	q2	OK	50.8103	50.3297	-0.0137134	-0.0194333	0.9761	0.99995	no

ENSG00000185722	ENSG00000185722	ANKFY1	17:4046461-4167274	q1	q2	OK	29.1537	32.0334	0.135895	0.181093	0.65605	0.99995	no

ENSG00000185728	ENSG00000185728	YTHDF3	8:64081111-64125346	q1	q2	OK	35.0789	37.897	0.111479	0.221297	0.68805	0.99995	no

ENSG00000185730	ENSG00000185730	ZNF696	8:144371845-144380231	q1	q2	OK	6.50552	6.80213	0.0643227	0.0984512	0.8629	0.99995	no

ENSG00000185736	ENSG00000185736	ADARB2	10:1228072-1779670	q1	q2	OK	1.16804	1.57505	0.43131	0.45679	0.45195	0.99995	no

ENSG00000185737	ENSG00000185737	NRG3	10:83635069-84746935	q1	q2	OK	3.86342	3.20759	-0.268391	-0.400162	0.5541	0.99995	no

ENSG00000185739	ENSG00000185739	SRL	16:4239374-4292081	q1	q2	NOTEST	0.0209331	0.0685076	1.71048	0	1	1	no

ENSG00000185742	ENSG00000185742	C11orf87	11:109225811-109454633	q1	q2	NOTEST	0.44373	0.467327	0.0747492	0	1	1	no

ENSG00000185745	ENSG00000185745	IFIT1	10:90973325-91174314	q1	q2	OK	0.721161	0.612963	-0.234522	-0.0813105	0.92175	0.99995	no

ENSG00000185751	ENSG00000185751	XAGE2	X:52380350-52387001	q1	q2	NOTEST	0	0	0	0	1	1	no

ENSG00000185753	ENSG00000185753	CXorf38	X:40488284-40506819	q1	q2	OK	3.13292	3.63471	0.21433	0.283774	0.6526	0.99995	no

ENSG00000185758	ENSG00000185758	CLDN24	4:184242916-184243579	q1	q2	NOTEST	0	0.0589974	inf	0	1	1	no

ENSG00000185760	ENSG00000185760	KCNQ5	6:73331519-73908574	q1	q2	NOTEST	0.434157	0.434715	0.00185326	0	1	1	no

ENSG00000185761	ENSG00000185761	ADAMTSL5	19:1505016-1513603	q1	q2	NOTEST	0.360014	0.0586914	-2.61683	0	1	1	no

ENSG00000185774	ENSG00000185774	KCNIP4	4:20697904-21950422	q1	q2	OK	0.840283	0.820216	-0.0348705	-0.0170072	0.97215	0.99995	no

ENSG00000185775	ENSG00000185775	SPATA31A6	9:43624506-43630730	q1	q2	NOTEST	0.0059189	0	#NAME?	0	1	1	no

ENSG00000185787	ENSG00000185787	MORF4L1	15:79051544-79190475	q1	q2	OK	351.367	366.261	0.059894	0.106604	0.8678	0.99995	no

ENSG00000185792	ENSG00000185792	NLRP9	19:56186591-56249768	q1	q2	NOTEST	0.105961	0.083549	-0.342842	0	1	1	no

ENSG00000185798	ENSG00000185798	WDR53	3:196281055-196315930	q1	q2	OK	10.3517	10.7861	0.0593114	0.0481363	0.9445	0.99995	no

ENSG00000185800	ENSG00000185800	DMWD	19:46268042-46296060	q1	q2	OK	28.5023	29.802	0.0643326	0.0617575	0.88975	0.99995	no

ENSG00000185803	ENSG00000185803	SLC52A2	8:145575877-145584932	q1	q2	NOTEST	0.0652868	0.158524	1.27983	0	1	1	no

ENSG00000185808	ENSG00000185808	PIGP	21:38431469-38445470	q1	q2	OK	20.7969	20.4406	-0.0249323	-0.0369928	0.95965	0.99995	no

ENSG00000185811	ENSG00000185811	IKZF1	7:50343719-50472799	q1	q2	OK	1.89989	2.05427	0.11271	0.118922	0.7993	0.99995	no

ENSG00000185813	ENSG00000185813	PCYT2	17:79845712-79869340	q1	q2	OK	18.5252	20.8161	0.168212	0.145031	0.795	0.99995	no

ENSG00000185818	ENSG00000185818	NAT8L	4:2061238-2070816	q1	q2	OK	14.659	12.5505	-0.224038	-0.538504	0.4423	0.99995	no

ENSG00000185821	ENSG00000185821	OR6C76	12:55820037-55820976	q1	q2	NOTEST	0	0	0	0	1	1	no

ENSG00000185823	ENSG00000185823	NPAP1	15:24920540-24928593	q1	q2	NOTEST	0.177426	0.149175	-0.250206	0	1	1	no

ENSG00000185825	ENSG00000185825	BCAP31	X:152965946-152990152	q1	q2	OK	1.2264	1.15892	-0.0816445	-0.0738788	0.89405	0.99995	no

ENSG00000185829	ENSG00000185829	ARL17A	17:44588876-44657088	q1	q2	OK	0.398632	0.552706	0.471456	0.192989	0.68125	0.99995	no

ENSG00000185834	ENSG00000185834	RPL12P4	20:53691154-53691685	q1	q2	OK	13.5574	15.0329	0.149042	0.191708	0.79205	0.99995	no

ENSG00000185837	ENSG00000185837	CECR5-AS1	22:17618400-17646335	q1	q2	NOTEST	0	0	0	0	1	1	no

ENSG00000185838	ENSG00000185838	GNB1L	22:19744225-19842462	q1	q2	OK	2.88476	2.71545	-0.0872582	-0.0569252	0.92765	0.99995	no

ENSG00000185839	ENSG00000185839	RP4-592A1.2	1:59096512-59097202	q1	q2	OK	6.23633	8.81422	0.499137	0.539755	0.4496	0.99995	no

ENSG00000185842	ENSG00000185842	DNAH14	1:225083963-225586996	q1	q2	OK	6.68311	8.71071	0.382269	0.42238	0.5253	0.99995	no

ENSG00000185847	ENSG00000185847	RP1-46F2.2	12:111374388-111396012	q1	q2	OK	0.301886	0.62087	1.04029	0.55902	0.4199	0.99995	no

ENSG00000185860	ENSG00000185860	C1orf110	1:162794247-162838605	q1	q2	NOTEST	0	0	0	0	1	1	no

ENSG00000185862	ENSG00000185862	EVI2B	17:29335514-29709134	q1	q2	NOTEST	0.117398	0.0789703	-0.572029	0	1	1	no

ENSG00000185863	ENSG00000185863	TMEM210	9:140063209-140066861	q1	q2	NOTEST	0	0	0	0	1	1	no

ENSG00000185864	ENSG00000185864	NPIPB4	16:21845889-21930477	q1	q2	OK	13.0046	13.9018	0.096252	0.104915	0.8699	0.99995	no

ENSG00000185869	ENSG00000185869	ZNF829	19:37288419-37407193	q1	q2	OK	1.30614	1.33324	0.0296213	0.0246172	0.977	0.99995	no

ENSG00000185873	ENSG00000185873	TMPRSS11B	4:69048009-69111438	q1	q2	NOTEST	0	0.0234489	inf	0	1	1	no

ENSG00000185875	ENSG00000185875	THNSL1	10:25305586-25315593	q1	q2	OK	4.6996	4.81628	0.0353809	0.0644827	0.92715	0.99995	no

ENSG00000185880	ENSG00000185880	TRIM69	15:45021185-45060027	q1	q2	OK	10.1051	9.29694	-0.12025	-0.194021	0.73655	0.99995	no

ENSG00000185883	ENSG00000185883	ATP6V0C	16:2525146-2581423	q1	q2	OK	97.221	84.9325	-0.194951	-0.36853	0.5975	0.99995	no

ENSG00000185885	ENSG00000185885	IFITM1	11:307630-315272	q1	q2	OK	10.8118	8.58913	-0.332018	-0.241991	0.7502	0.99995	no

ENSG00000185888	ENSG00000185888	PRSS38	1:228003393-228034171	q1	q2	NOTEST	0	0	0	0	1	1	no

ENSG00000185894	ENSG00000185894	BPY2C	Y:27177047-27208695	q1	q2	NOTEST	0	0	0	0	1	1	no

ENSG00000185896	ENSG00000185896	LAMP1	13:113951555-113977987	q1	q2	OK	78.5853	70.8736	-0.149011	-0.342303	0.6197	0.99995	no

ENSG00000185897	ENSG00000185897	FFAR3	19:35849361-35851387	q1	q2	NOTEST	0	0	0	0	1	1	no

ENSG00000185899	ENSG00000185899	TAS2R60	7:143078172-143220542	q1	q2	NOTEST	0.0331101	0	#NAME?	0	1	1	no

ENSG00000185900	ENSG00000185900	POMK	8:42948657-42978577	q1	q2	OK	2.72061	3.26597	0.263581	0.343568	0.6223	0.99995	no

ENSG00000185903	ENSG00000185903	OR11N1P	X:130407479-130533677	q1	q2	NOTEST	0	0	0	0	1	1	no

ENSG00000185904	ENSG00000185904	LINC00839	10:42970990-42990784	q1	q2	NOTEST	0	0	0	0	1	1	no

ENSG00000185905	ENSG00000185905	C16orf54	16:29753783-29759620	q1	q2	NOTEST	0	0	0	0	1	1	no

ENSG00000185909	ENSG00000185909	KLHDC8B	3:49209043-49213917	q1	q2	OK	38.6529	35.8656	-0.107977	-0.209403	0.77005	0.99995	no

ENSG00000185915	ENSG00000185915	KLHL34	X:21673608-21676448	q1	q2	NOTEST	0.036441	0.0435422	0.256853	0	1	1	no

ENSG00000185917	ENSG00000185917	SETD4	21:37402367-37498938	q1	q2	OK	13.8464	13.4756	-0.0391589	-0.0569409	0.92175	0.99995	no

ENSG00000185920	ENSG00000185920	PTCH1	9:98205261-98279339	q1	q2	OK	29.3736	24.3073	-0.273133	-0.407876	0.4065	0.99995	no

ENSG00000185924	ENSG00000185924	RTN4RL1	17:1837970-1928639	q1	q2	OK	0.550697	0.390806	-0.494806	-0.497117	0.4817	0.99995	no

ENSG00000185926	ENSG00000185926	OR4C46	11:51515281-51516211	q1	q2	NOTEST	0	0	0	0	1	1	no

ENSG00000185928	ENSG00000185928	PAGR1	16:29817426-29859355	q1	q2	OK	19.1856	17.3094	-0.148472	-0.0965479	0.87975	0.99995	no

ENSG00000185933	ENSG00000185933	CALHM1	10:105206542-105222452	q1	q2	NOTEST	0	0.00864978	inf	0	1	1	no

ENSG00000185940	ENSG00000185940	KRTAP5-5	11:1651032-1652160	q1	q2	NOTEST	0	0	0	0	1	1	no

ENSG00000185942	ENSG00000185942	NKAIN3	8:63161149-63912211	q1	q2	OK	1.86951	2.74589	0.554612	0.495997	0.50725	0.99995	no

ENSG00000185945	ENSG00000185945	NXF2B	X:101615315-101726732	q1	q2	NOTEST	0	0	0	0	1	1	no

ENSG00000185946	ENSG00000185946	RNPC3	1:104068312-104122156	q1	q2	OK	13.2161	13.2451	0.0031551	0.00416386	0.99365	0.99995	no

ENSG00000185947	ENSG00000185947	ZNF267	16:31885078-31928668	q1	q2	OK	5.84696	5.78862	-0.0144694	-0.0224627	0.96715	0.99995	no

ENSG00000185950	ENSG00000185950	IRS2	13:110406183-110438915	q1	q2	OK	3.09534	2.51801	-0.297816	-0.54843	0.4356	0.99995	no

ENSG00000185955	ENSG00000185955	C7orf61	7:100054237-100076902	q1	q2	OK	0.560321	0.693034	0.306674	0.0667139	0.8573	0.99995	no

ENSG00000185958	ENSG00000185958	FAM186A	12:50720012-50873787	q1	q2	NOTEST	0.162496	0.109413	-0.570613	0	1	1	no

ENSG00000185960	ENSG00000185960	SHOX	X:585078-620146	q1	q2	NOTEST	0.0147043	0.00693948	-1.08334	0	1	1	no

ENSG00000185962	ENSG00000185962	LCE3A	1:152595309-152595579	q1	q2	NOTEST	0	0	0	0	1	1	no

ENSG00000185963	ENSG00000185963	BICD2	9:95473644-95527094	q1	q2	OK	12.7427	13.0698	0.0365646	0.0824859	0.9087	0.99995	no

ENSG00000185966	ENSG00000185966	LCE3E	1:152538129-152539248	q1	q2	NOTEST	0	0	0	0	1	1	no

ENSG00000185972	ENSG00000185972	CCIN	9:36169388-36171329	q1	q2	NOTEST	0.0408901	0.0415553	0.0232804	0	1	1	no

ENSG00000185973	ENSG00000185973	TMLHE	X:154695630-154899605	q1	q2	NOTEST	0.159167	0.12393	-0.361013	0	1	1	no

ENSG00000185974	ENSG00000185974	GRK1	13:114321469-114440708	q1	q2	NOTEST	0.0115863	0.0230769	0.994026	0	1	1	no

ENSG00000185978	ENSG00000185978	H2AFB3	X:154689079-154689596	q1	q2	NOTEST	0	0.0770294	inf	0	1	1	no

ENSG00000185982	ENSG00000185982	DEFB128	20:168493-170322	q1	q2	NOTEST	0	0	0	0	1	1	no

ENSG00000185985	ENSG00000185985	SLITRK2	X:144899349-144907360	q1	q2	NOTEST	0	0	0	0	1	1	no

ENSG00000185986	ENSG00000185986	SDHAP3	5:1568636-1594735	q1	q2	OK	9.71154	11.0954	0.192192	0.284767	0.69895	0.99995	no

ENSG00000185988	ENSG00000185988	PLK5	19:1524072-1535455	q1	q2	NOTEST	0.0328075	0.0484117	0.561331	0	1	1	no

ENSG00000185989	ENSG00000185989	RASA3	13:114747193-114898086	q1	q2	OK	17.8365	17.8534	0.00136671	0.00322948	0.9965	0.99995	no

ENSG00000185990	ENSG00000185990	F8A3	X:154686575-154688276	q1	q2	NOTEST	0	0.0153558	inf	0	1	1	no

ENSG00000186001	ENSG00000186001	LRCH3	3:197518096-197615307	q1	q2	OK	36.0313	38.3494	0.0899526	0.0721569	0.8019	0.99995	no

ENSG00000186007	ENSG00000186007	LEMD1	1:205342379-205438152	q1	q2	OK	1.32388	1.4886	0.169183	0.112641	0.8467	0.99995	no

ENSG00000186008	ENSG00000186008	RPS4XP21	19:34583444-34584215	q1	q2	NOTEST	0.04314	0	#NAME?	0	1	1	no

ENSG00000186009	ENSG00000186009	ATP4B	13:114303118-114312501	q1	q2	NOTEST	0.0184243	0.0549573	1.5767	0	1	1	no

ENSG00000186010	ENSG00000186010	NDUFA13	19:19623226-19648390	q1	q2	OK	159	147.071	-0.112516	-0.245848	0.72435	0.99995	no

ENSG00000186017	ENSG00000186017	ZNF566	19:36936020-36981942	q1	q2	OK	6.78877	7.27861	0.100511	0.130689	0.78985	0.99995	no

ENSG00000186019	ENSG00000186019	AC084219.4	19:44598491-44637027	q1	q2	OK	7.36221	6.67169	-0.142086	-0.203888	0.76865	0.99995	no

ENSG00000186020	ENSG00000186020	ZNF529	19:37001596-37119499	q1	q2	OK	10.9804	10.5206	-0.0617106	-0.0561598	0.92135	0.99995	no

ENSG00000186026	ENSG00000186026	ZNF284	19:44529493-44593766	q1	q2	OK	1.8629	1.95341	0.0684474	0.0692897	0.9213	0.99995	no

ENSG00000186038	ENSG00000186038	HTR3E	3:183812905-183824783	q1	q2	NOTEST	0	0	0	0	1	1	no

ENSG00000186047	ENSG00000186047	DLEU7	13:50601268-51423190	q1	q2	OK	0.469392	0.527376	0.168039	0.050825	0.9364	0.99995	no

ENSG00000186049	ENSG00000186049	KRT73	12:52979372-53012343	q1	q2	NOTEST	0	0	0	0	1	1	no

ENSG00000186051	ENSG00000186051	TAL2	9:108424737-108425367	q1	q2	OK	0.798375	1.01031	0.339665	0.281883	0.68705	0.99995	no

ENSG00000186056	ENSG00000186056	MATN1-AS1	1:31184123-31199674	q1	q2	NOTEST	0.101372	0.0862952	-0.232306	0	1	1	no

ENSG00000186063	ENSG00000186063	AIDA	1:222841354-222908538	q1	q2	OK	23.6858	25.8118	0.124013	0.17215	0.8047	0.99995	no

ENSG00000186073	ENSG00000186073	C15orf41	15:36871811-37110660	q1	q2	OK	7.22521	6.81991	-0.0832868	-0.11365	0.8608	0.99995	no

ENSG00000186074	ENSG00000186074	CD300LF	17:72666716-72765492	q1	q2	NOTEST	0	0	0	0	1	1	no

ENSG00000186075	ENSG00000186075	ZPBP2	17:38024416-38034149	q1	q2	NOTEST	0	0.0124758	inf	0	1	1	no

ENSG00000186076	ENSG00000186076	RP11-887P2.3	12:94034597-94035362	q1	q2	OK	7.54653	10.2257	0.438317	0.468269	0.49805	0.99995	no

ENSG00000186081	ENSG00000186081	KRT5	12:52908358-52914471	q1	q2	NOTEST	0	0.00986723	inf	0	1	1	no

ENSG00000186082	ENSG00000186082	KRT18P14	11:35881798-35883048	q1	q2	NOTEST	0.0238529	0	#NAME?	0	1	1	no

ENSG00000186086	ENSG00000186086	NBPF6	1:108803817-109013624	q1	q2	NOTEST	0	0.0127257	inf	0	1	1	no

ENSG00000186088	ENSG00000186088	GSAP	7:76139744-77045717	q1	q2	NOTEST	0.123575	0.272687	1.14185	0	1	1	no

ENSG00000186090	ENSG00000186090	HTR3D	3:183749331-183757157	q1	q2	NOTEST	0	0	0	0	1	1	no

ENSG00000186092	ENSG00000186092	OR4F5	1:69090-70008	q1	q2	NOTEST	0	0	0	0	1	1	no

ENSG00000186094	ENSG00000186094	AGBL4	1:48998526-50489585	q1	q2	OK	0.488715	0.238244	-1.03655	-0.340961	0.38505	0.99995	no

ENSG00000186103	ENSG00000186103	ARGFX	3:121289550-121305481	q1	q2	NOTEST	0	0	0	0	1	1	no

ENSG00000186104	ENSG00000186104	CYP2R1	11:14899552-14913798	q1	q2	NOTEST	0	0	0	0	1	1	no

ENSG00000186105	ENSG00000186105	LRRC70	5:61601988-61924409	q1	q2	OK	0.691775	0.966048	0.481791	0.0899982	0.7743	0.99995	no

ENSG00000186106	ENSG00000186106	ANKRD46	8:101521979-101572012	q1	q2	OK	16.2186	17.0796	0.0746269	0.14361	0.81775	0.99995	no

ENSG00000186111	ENSG00000186111	PIP5K1C	19:3630180-3700477	q1	q2	OK	15.6058	14.141	-0.142199	-0.32791	0.6315	0.99995	no

ENSG00000186113	ENSG00000186113	OR5D14	11:55563031-55563976	q1	q2	NOTEST	0	0	0	0	1	1	no

ENSG00000186115	ENSG00000186115	CYP4F2	19:15988832-16008930	q1	q2	NOTEST	0.0115512	0.0548298	2.24692	0	1	1	no

ENSG00000186117	ENSG00000186117	OR5L1	11:55578853-55579952	q1	q2	NOTEST	0	0	0	0	1	1	no

ENSG00000186118	ENSG00000186118	TEX38	1:47098408-47184824	q1	q2	NOTEST	0.0712698	0.0668528	-0.0923039	0	1	1	no

ENSG00000186119	ENSG00000186119	OR5D18	11:55587085-55588073	q1	q2	NOTEST	0	0	0	0	1	1	no

ENSG00000186124	ENSG00000186124	OR9M1P	11:55623067-55623988	q1	q2	NOTEST	0	0	0	0	1	1	no

ENSG00000186130	ENSG00000186130	ZBTB6	9:125670334-125675609	q1	q2	OK	6.08253	6.58198	0.113851	0.226357	0.7469	0.99995	no

ENSG00000186132	ENSG00000186132	C2orf76	2:120059800-120124404	q1	q2	OK	7.24664	6.51315	-0.153957	-0.185206	0.7895	0.99995	no

ENSG00000186136	ENSG00000186136	TAS2R42	12:11338598-11339543	q1	q2	NOTEST	0.166435	0.157514	-0.0794789	0	1	1	no

ENSG00000186141	ENSG00000186141	POLR3C	1:145592604-145611025	q1	q2	OK	1.96081	1.9175	-0.032225	-0.0320496	0.96255	0.99995	no

ENSG00000186143	ENSG00000186143	C2orf53	2:27359722-27362278	q1	q2	NOTEST	0	0	0	0	1	1	no

ENSG00000186146	ENSG00000186146	DEFB131	4:9446259-9452240	q1	q2	NOTEST	0	0	0	0	1	1	no

ENSG00000186148	ENSG00000186148	AC013271.3	2:110690737-110742667	q1	q2	NOTEST	0.0447769	0	#NAME?	0	1	1	no

ENSG00000186150	ENSG00000186150	UBL4B	1:110655061-110656569	q1	q2	NOTEST	0.0181426	0	#NAME?	0	1	1	no

ENSG00000186152	ENSG00000186152	LILRP1	19:55208382-55212683	q1	q2	NOTEST	0	0	0	0	1	1	no

ENSG00000186153	ENSG00000186153	WWOX	16:78133309-79246564	q1	q2	OK	10.2325	10.0294	-0.0289238	-0.0382819	0.95145	0.99995	no

ENSG00000186160	ENSG00000186160	CYP4Z1	1:47533159-47646011	q1	q2	NOTEST	0.0139097	0	#NAME?	0	1	1	no

ENSG00000186162	ENSG00000186162	CIDECP	3:10055931-10068049	q1	q2	OK	5.65947	6.7985	0.26455	0.34493	0.641	0.99995	no

ENSG00000186163	ENSG00000186163	U66059.29	7:141965649-141972054	q1	q2	NOTEST	0	0	0	0	1	1	no

ENSG00000186166	ENSG00000186166	CCDC84	11:118868851-118901616	q1	q2	NOTEST	0	0.0179972	inf	0	1	1	no

ENSG00000186174	ENSG00000186174	BCL9L	11:118754474-118829269	q1	q2	OK	5.7026	5.59566	-0.027313	-0.0341423	0.9378	0.99995	no

ENSG00000186184	ENSG00000186184	POLR1D	13:28194902-28241548	q1	q2	OK	55.0994	58.4153	0.0843094	0.159728	0.81975	0.99995	no

ENSG00000186185	ENSG00000186185	KIF18B	17:43002076-43025082	q1	q2	OK	22.3316	21.3888	-0.0622294	-0.0884996	0.8723	0.99995	no

ENSG00000186187	ENSG00000186187	ZNRF1	16:74907467-75144892	q1	q2	OK	25.1978	26.6649	0.0816481	0.0989155	0.88795	0.99995	no

ENSG00000186188	ENSG00000186188	FFAR4	10:95326421-95364237	q1	q2	NOTEST	0	0.00674189	inf	0	1	1	no

ENSG00000186190	ENSG00000186190	BPIFB3	20:31643229-31661434	q1	q2	NOTEST	0	0	0	0	1	1	no

ENSG00000186191	ENSG00000186191	BPIFB4	20:31667449-31699557	q1	q2	NOTEST	0	0	0	0	1	1	no

ENSG00000186193	ENSG00000186193	SAPCD2	9:139952114-139965040	q1	q2	OK	12.3374	10.7084	-0.204291	-0.442289	0.5246	0.99995	no

ENSG00000186197	ENSG00000186197	EDARADD	1:236511561-236648214	q1	q2	OK	0.397077	0.598231	0.591283	0.449819	0.42685	0.99995	no

ENSG00000186198	ENSG00000186198	SLC51B	15:65337707-65369028	q1	q2	NOTEST	0.292242	0.160483	-0.864741	0	1	1	no

ENSG00000186204	ENSG00000186204	CYP4F12	19:15783566-15807984	q1	q2	NOTEST	0	0	0	0	1	1	no

ENSG00000186205	ENSG00000186205	Mar-01	1:220960100-220987735	q1	q2	OK	7.39412	6.91884	-0.0958487	-0.153583	0.8151	0.99995	no

ENSG00000186207	ENSG00000186207	LCE5A	1:152483319-152484653	q1	q2	NOTEST	0	0	0	0	1	1	no

ENSG00000186212	ENSG00000186212	SOWAHB	4:77816081-77819002	q1	q2	NOTEST	0.133176	0.139517	0.0671121	0	1	1	no

ENSG00000186222	ENSG00000186222	BLOC1S4	4:6717841-6719387	q1	q2	OK	13.2808	13.6872	0.0434879	0.0801338	0.9092	0.99995	no

ENSG00000186223	ENSG00000186223	SSU72P4	11:4308731-4309313	q1	q2	NOTEST	0	0	0	0	1	1	no

ENSG00000186226	ENSG00000186226	LCE1E	1:152758689-152760902	q1	q2	NOTEST	0	0	0	0	1	1	no

ENSG00000186230	ENSG00000186230	ZNF749	19:57874844-57988938	q1	q2	OK	6.67882	7.62034	0.19026	0.124811	0.8038	0.99995	no

ENSG00000186231	ENSG00000186231	KLHL32	6:97372604-97588630	q1	q2	OK	0.688417	0.352325	-0.966377	-0.713201	0.2039	0.99995	no

ENSG00000186232	ENSG00000186232	SSU72P3	11:4351094-4351679	q1	q2	NOTEST	0	0	0	0	1	1	no

ENSG00000186234	ENSG00000186234	FAM86MP	4:9694118-9704578	q1	q2	NOTEST	0	0	0	0	1	1	no

ENSG00000186235	ENSG00000186235	AC016757.3	2:239133192-239140318	q1	q2	NOTEST	0.11578	0.204093	0.817836	0	1	1	no

ENSG00000186244	ENSG00000186244	RP11-1079K10.1	17:47448101-47554350	q1	q2	NOTEST	0	0	0	0	1	1	no

ENSG00000186260	ENSG00000186260	MKL2	16:14165177-14360630	q1	q2	OK	15.0564	16.6173	0.142308	0.142273	0.703	0.99995	no

ENSG00000186265	ENSG00000186265	BTLA	3:112182814-112218408	q1	q2	NOTEST	0.00787558	0.00762718	-0.0462356	0	1	1	no

ENSG00000186268	ENSG00000186268	OR10D4P	11:123964288-123965239	q1	q2	NOTEST	0.0316009	0	#NAME?	0	1	1	no

ENSG00000186272	ENSG00000186272	ZNF17	19:57874844-57988938	q1	q2	OK	4.77528	4.82462	0.014832	0.0100028	0.98355	0.99995	no

ENSG00000186275	ENSG00000186275	NBPF12	1:146373545-146467744	q1	q2	OK	1.03922	3.40172	1.71076	1.12895	0.0968	0.99995	no

ENSG00000186280	ENSG00000186280	KDM4D	11:94706844-94732682	q1	q2	NOTEST	0	0	0	0	1	1	no

ENSG00000186281	ENSG00000186281	GPAT2	2:96676298-96705199	q1	q2	NOTEST	0.0397335	0.0319132	-0.316202	0	1	1	no

ENSG00000186283	ENSG00000186283	TOR3A	1:179050511-179067158	q1	q2	OK	12.2222	11.9839	-0.0284029	-0.050621	0.93555	0.99995	no

ENSG00000186288	ENSG00000186288	PABPC1L2A	X:72297114-72299351	q1	q2	NOTEST	0	0	0	0	1	1	no

ENSG00000186297	ENSG00000186297	GABRA5	15:26788692-27194354	q1	q2	OK	0.671947	0.668793	-0.00678711	-0.00168042	0.9954	0.99995	no

ENSG00000186298	ENSG00000186298	PPP1CC	12:111157484-111180744	q1	q2	OK	149.098	158.791	0.0908656	0.205171	0.76355	0.99995	no

ENSG00000186300	ENSG00000186300	ZNF555	19:2841432-2860482	q1	q2	OK	4.00456	4.00633	0.000639086	0.000910922	0.99895	0.99995	no

ENSG00000186301	ENSG00000186301	MST1P2	1:16972068-16976914	q1	q2	OK	0.81967	0.77821	-0.0748827	-0.0727773	0.92085	0.99995	no

ENSG00000186306	ENSG00000186306	OR10T2	1:158368311-158369256	q1	q2	NOTEST	0.0336449	0	#NAME?	0	1	1	no

ENSG00000186310	ENSG00000186310	NAP1L3	X:92925928-92928567	q1	q2	OK	12.0101	11.5524	-0.0560499	-0.115194	0.86665	0.99995	no

ENSG00000186312	ENSG00000186312	CA5BP1	X:15693054-15805747	q1	q2	OK	6.74274	6.65338	-0.0192479	-0.0201898	0.97925	0.99995	no

ENSG00000186314	ENSG00000186314	PRELID2	5:144851361-145214932	q1	q2	OK	0.70845	1.24044	0.808113	0.617268	0.22125	0.99995	no

ENSG00000186318	ENSG00000186318	BACE1	11:117103340-117283984	q1	q2	OK	16.576	16.769	0.0166968	0.0187861	0.9668	0.99995	no

ENSG00000186322	ENSG00000186322	GOLGA6L17P	15:82804002-82810874	q1	q2	NOTEST	0.295154	0.481159	0.705043	0	1	1	no

ENSG00000186326	ENSG00000186326	RGS9BP	19:33087912-33169206	q1	q2	NOTEST	0.0269026	0.0255999	-0.0716079	0	1	1	no

ENSG00000186328	ENSG00000186328	RP11-140K17.2	6:34683389-34683717	q1	q2	NOTEST	0	0	0	0	1	1	no

ENSG00000186329	ENSG00000186329	TMEM212	3:171561138-171656505	q1	q2	NOTEST	0	0	0	0	1	1	no

ENSG00000186334	ENSG00000186334	SLC36A3	5:150656322-150683327	q1	q2	NOTEST	0	0	0	0	1	1	no

ENSG00000186335	ENSG00000186335	SLC36A2	5:150694538-150727151	q1	q2	NOTEST	0	0.0123819	inf	0	1	1	no

ENSG00000186340	ENSG00000186340	THBS2	6:169613348-169654139	q1	q2	OK	1.79769	1.59824	-0.169663	-0.22831	0.70795	0.99995	no

ENSG00000186350	ENSG00000186350	RXRA	9:137208943-137332431	q1	q2	OK	17.8852	15.0437	-0.249603	-0.500458	0.4765	0.99995	no

ENSG00000186352	ENSG00000186352	ANKRD37	4:186317174-186347139	q1	q2	OK	1.56068	1.13219	-0.463063	-0.153585	0.6694	0.99995	no

ENSG00000186354	ENSG00000186354	C9orf47	9:91605777-91619925	q1	q2	OK	0.901193	0.881075	-0.0325719	-0.0120042	0.98485	0.99995	no

ENSG00000186364	ENSG00000186364	NUDT17	1:145575232-145589439	q1	q2	NOTEST	0	0	0	0	1	1	no

ENSG00000186367	ENSG00000186367	KIAA1024L	5:128795251-129522327	q1	q2	NOTEST	0.232035	0.0549497	-2.07816	0	1	1	no

ENSG00000186369	ENSG00000186369	LINC00643	14:62570095-62596352	q1	q2	NOTEST	0.26584	0.238732	-0.155164	0	1	1	no

ENSG00000186376	ENSG00000186376	ZNF75D	X:134382866-134478012	q1	q2	NOTEST	0.0136761	0.0228968	0.743485	0	1	1	no

ENSG00000186377	ENSG00000186377	CYP4X1	1:47427035-47516423	q1	q2	NOTEST	0.130774	0.0899614	-0.539694	0	1	1	no

ENSG00000186393	ENSG00000186393	KRT26	17:38922489-38928414	q1	q2	NOTEST	0	0	0	0	1	1	no

ENSG00000186395	ENSG00000186395	KRT10	17:38974368-38992522	q1	q2	OK	9.73421	10.6046	0.123548	0.206581	0.7661	0.99995	no

ENSG00000186399	ENSG00000186399	GOLGA8R	15:30695942-30706463	q1	q2	NOTEST	0.0419763	0.0295978	-0.504086	0	1	1	no

ENSG00000186400	ENSG00000186400	OR10X1	1:158548708-158549689	q1	q2	NOTEST	0	0	0	0	1	1	no

ENSG00000186407	ENSG00000186407	CD300E	17:72606025-72619897	q1	q2	NOTEST	0	0	0	0	1	1	no

ENSG00000186409	ENSG00000186409	CCDC30	1:42895999-43120335	q1	q2	OK	2.05567	1.87998	-0.128894	-0.0780552	0.8535	0.99995	no

ENSG00000186416	ENSG00000186416	NKRF	X:118722299-118739858	q1	q2	OK	8.035	8.57054	0.0930882	0.185167	0.7906	0.99995	no

ENSG00000186417	ENSG00000186417	GLDN	15:51633825-51700210	q1	q2	NOTEST	0.155818	0.156507	0.00636788	0	1	1	no

ENSG00000186431	ENSG00000186431	FCAR	19:55385703-55401838	q1	q2	NOTEST	0	0	0	0	1	1	no

ENSG00000186432	ENSG00000186432	KPNA4	3:160212782-160287036	q1	q2	OK	15.9407	16.3807	0.0392884	0.0874668	0.88735	0.99995	no

ENSG00000186439	ENSG00000186439	TRDN	6:123537482-123958238	q1	q2	NOTEST	0.0227996	0	#NAME?	0	1	1	no

ENSG00000186440	ENSG00000186440	OR6P1	1:158532440-158533394	q1	q2	NOTEST	0	0	0	0	1	1	no

ENSG00000186442	ENSG00000186442	KRT3	12:53183468-53189901	q1	q2	NOTEST	0	0	0	0	1	1	no

ENSG00000186446	ENSG00000186446	ZNF501	3:44771087-44778575	q1	q2	OK	2.29987	2.5071	0.124471	0.117457	0.81795	0.99995	no

ENSG00000186448	ENSG00000186448	ZNF197	3:44596684-44727145	q1	q2	OK	25.8123	26.6196	0.04443	0.0576328	0.9185	0.99995	no

ENSG00000186451	ENSG00000186451	SPATA12	3:56761445-57114129	q1	q2	NOTEST	0.0762258	0.0869234	0.189464	0	1	1	no

ENSG00000186452	ENSG00000186452	TMPRSS12	12:51236702-51281667	q1	q2	NOTEST	0.0227028	0.0146544	-0.631539	0	1	1	no

ENSG00000186453	ENSG00000186453	FAM228A	2:24299395-24424567	q1	q2	NOTEST	0.233124	0.311557	0.418397	0	1	1	no

ENSG00000186458	ENSG00000186458	DEFB132	20:238376-241737	q1	q2	NOTEST	0	0	0	0	1	1	no

ENSG00000186462	ENSG00000186462	NAP1L2	X:72432134-72434684	q1	q2	OK	4.16828	3.8089	-0.130076	-0.206592	0.76275	0.99995	no

ENSG00000186466	ENSG00000186466	AQP7P1	9:67272037-67298632	q1	q2	NOTEST	0.272584	0.121844	-1.16166	0	1	1	no

ENSG00000186468	ENSG00000186468	RPS23	5:81267843-81574396	q1	q2	OK	1545.8	1537.99	-0.00731068	-0.0132412	0.9847	0.99995	no

ENSG00000186469	ENSG00000186469	GNG2	14:52292912-52446060	q1	q2	OK	136.037	157.327	0.209767	0.468604	0.48795	0.99995	no

ENSG00000186470	ENSG00000186470	BTN3A2	6:26365386-26378546	q1	q2	OK	4.7493	3.92508	-0.274993	-0.34718	0.4997	0.99995	no

ENSG00000186471	ENSG00000186471	AKAP14	X:119029799-119054679	q1	q2	NOTEST	0	0.253928	inf	0	1	1	no

ENSG00000186472	ENSG00000186472	PCLO	7:82383328-82792246	q1	q2	OK	1.41033	0.709003	-0.992165	-0.583187	0.23125	0.99995	no

ENSG00000186474	ENSG00000186474	KLK12	19:51532347-51538486	q1	q2	NOTEST	0	0	0	0	1	1	no

ENSG00000186479	ENSG00000186479	RGS7BP	5:63802083-63908139	q1	q2	OK	0.380899	0.484147	0.346037	0.253844	0.6152	0.99995	no

ENSG00000186480	ENSG00000186480	INSIG1	7:155089485-155101945	q1	q2	OK	34.8101	42.2345	0.278916	0.606647	0.32675	0.99995	no

ENSG00000186481	ENSG00000186481	ANKRD20A5P	18:14179095-14252139	q1	q2	NOTEST	0	0	0	0	1	1	no

ENSG00000186487	ENSG00000186487	MYT1L	2:1792884-2335032	q1	q2	OK	1.94248	2.00597	0.0464011	0.0390555	0.9261	0.99995	no

ENSG00000186493	ENSG00000186493	C5orf38	5:2745958-2755508	q1	q2	OK	21.7952	15.9311	-0.45217	-0.227497	0.6966	0.99995	no

ENSG00000186496	ENSG00000186496	ZNF396	18:32946660-32957301	q1	q2	OK	1.8051	1.80537	0.000211472	0.000230407	0.99965	0.99995	no

ENSG00000186501	ENSG00000186501	TMEM222	1:27648650-27662891	q1	q2	OK	25.5573	21.8494	-0.226142	-0.462113	0.49745	0.99995	no

ENSG00000186508	ENSG00000186508	OR9I2P	11:57791352-57949088	q1	q2	NOTEST	0	0	0	0	1	1	no

ENSG00000186509	ENSG00000186509	OR9Q1	11:57791352-57949088	q1	q2	NOTEST	0	0	0	0	1	1	no

ENSG00000186510	ENSG00000186510	CLCNKA	1:16340522-16360545	q1	q2	NOTEST	0.0321703	0	#NAME?	0	1	1	no

ENSG00000186513	ENSG00000186513	OR9Q2	11:57957905-57958990	q1	q2	NOTEST	0	0	0	0	1	1	no

ENSG00000186517	ENSG00000186517	ARHGAP30	1:161016735-161039760	q1	q2	NOTEST	0.35431	0.299493	-0.24249	0	1	1	no

ENSG00000186522	ENSG00000186522	Sep-10	2:110300558-110371783	q1	q2	OK	57.9991	65.0056	0.164534	0.34587	0.5785	0.99995	no

ENSG00000186523	ENSG00000186523	FAM86B1	8:12036205-12051642	q1	q2	OK	0.553569	0.710939	0.360961	0.265227	0.4748	0.99995	no

ENSG00000186526	ENSG00000186526	CYP4F8	19:15726028-15741448	q1	q2	NOTEST	0	0	0	0	1	1	no

ENSG00000186529	ENSG00000186529	CYP4F3	19:15751706-15773635	q1	q2	NOTEST	0.00489252	0.0826332	4.07807	0	1	1	no

ENSG00000186530	ENSG00000186530	XKR5	8:6666037-6742798	q1	q2	NOTEST	0.424229	0.457644	0.109385	0	1	1	no

ENSG00000186532	ENSG00000186532	SMYD4	17:1682778-1803376	q1	q2	OK	8.55603	8.49085	-0.0110333	-0.00932537	0.9894	0.99995	no

ENSG00000186543	ENSG00000186543	CROCCP5	1:21760810-21764051	q1	q2	NOTEST	0	0	0	0	1	1	no

ENSG00000186562	ENSG00000186562	DEFB105A	8:7679487-7681411	q1	q2	NOTEST	0	0	0	0	1	1	no

ENSG00000186564	ENSG00000186564	FOXD2	1:47901688-47906363	q1	q2	NOTEST	0.010605	0.016551	0.642172	0	1	1	no

ENSG00000186566	ENSG00000186566	GPATCH8	17:42472651-42580798	q1	q2	OK	22.2903	23.4876	0.075483	0.0981488	0.81495	0.99995	no

ENSG00000186567	ENSG00000186567	CEACAM19	19:45041044-45222031	q1	q2	OK	2.06633	2.14161	0.0516223	0.0351127	0.9547	0.99995	no

ENSG00000186572	ENSG00000186572	DEFB107A	8:7669241-7673238	q1	q2	NOTEST	0	0	0	0	1	1	no

ENSG00000186575	ENSG00000186575	NF2	22:29999544-30094587	q1	q2	OK	8.23191	8.33588	0.0181075	0.0308957	0.96505	0.99995	no

ENSG00000186577	ENSG00000186577	C6orf1	6:34214156-34217247	q1	q2	OK	7.32465	6.22606	-0.23444	-0.321422	0.68015	0.99995	no

ENSG00000186579	ENSG00000186579	DEFB106A	8:7682693-7686575	q1	q2	NOTEST	0	0	0	0	1	1	no

ENSG00000186583	ENSG00000186583	SPATC1	8:145051320-145101933	q1	q2	NOTEST	0	0	0	0	1	1	no

ENSG00000186591	ENSG00000186591	UBE2H	7:129470571-129592789	q1	q2	OK	32.6503	31.8549	-0.0355804	-0.0627851	0.8959	0.99995	no

ENSG00000186594	ENSG00000186594	MIR22HG	17:1614804-1641893	q1	q2	OK	5.65639	4.66404	-0.2783	-0.265052	0.6094	0.99995	no

ENSG00000186599	ENSG00000186599	DEFB105B	8:7345190-7347115	q1	q2	NOTEST	0	0	0	0	1	1	no

ENSG00000186603	ENSG00000186603	HPDL	1:45792544-45794347	q1	q2	OK	0.674851	0.692387	0.037011	0.0375767	0.95385	0.99995	no

ENSG00000186615	ENSG00000186615	KTN1-AS1	14:55965995-56168244	q1	q2	OK	0.838365	1.35622	0.693946	0.148779	0.63145	0.99995	no

ENSG00000186625	ENSG00000186625	KATNA1	6:149916008-149970108	q1	q2	OK	11.8335	12.1993	0.0439194	0.076997	0.9023	0.99995	no

ENSG00000186628	ENSG00000186628	FSD2	15:83419315-83474822	q1	q2	NOTEST	0.0680051	0.0732656	0.107493	0	1	1	no

ENSG00000186635	ENSG00000186635	ARAP1	11:72396113-72504726	q1	q2	OK	11.4732	8.76159	-0.389	-0.482233	0.3469	0.99995	no

ENSG00000186638	ENSG00000186638	KIF24	9:34179002-34329198	q1	q2	OK	5.60652	5.22767	-0.100937	-0.131603	0.8592	0.99995	no

ENSG00000186642	ENSG00000186642	PDE2A	11:72287184-72385635	q1	q2	OK	1.13263	1.26612	0.160735	0.0955261	0.74945	0.99995	no

ENSG00000186645	ENSG00000186645	SPDYE9P	7:76139744-77045717	q1	q2	NOTEST	0.00884148	0.0275811	1.64132	0	1	1	no

ENSG00000186648	ENSG00000186648	LRRC16B	14:24521205-24538937	q1	q2	OK	2.68921	2.44891	-0.135039	-0.117444	0.82365	0.99995	no

ENSG00000186652	ENSG00000186652	PRG2	11:57154266-57195053	q1	q2	NOTEST	0.0527388	0.047864	-0.139925	0	1	1	no

ENSG00000186654	ENSG00000186654	PRR5	22:45064592-45258665	q1	q2	OK	4.37616	3.50303	-0.32106	-0.406444	0.52695	0.99995	no

ENSG00000186660	ENSG00000186660	ZFP91	11:58346583-58393198	q1	q2	OK	28.5716	29.9715	0.0690068	0.168817	0.80575	0.99995	no

ENSG00000186665	ENSG00000186665	C17orf58	17:65987216-65989765	q1	q2	NOTEST	0.249715	0.133439	-0.904104	0	1	1	no

ENSG00000186666	ENSG00000186666	BCDIN3D	12:50184928-50236912	q1	q2	OK	7.2929	6.99786	-0.0595796	-0.045166	0.9452	0.99995	no

ENSG00000186675	ENSG00000186675	MAGEE2	X:75002822-75005079	q1	q2	NOTEST	0.0595647	0.0687318	0.20652	0	1	1	no

ENSG00000186676	ENSG00000186676	EEF1GP1	7:124462439-125019375	q1	q2	NOTEST	0	0	0	0	1	1	no

ENSG00000186678	ENSG00000186678	RP11-266I3.7	X:55199149-55202459	q1	q2	NOTEST	0	0	0	0	1	1	no

ENSG00000186684	ENSG00000186684	CYP27C1	2:127941695-127977654	q1	q2	OK	7.74053	7.40399	-0.0641299	-0.133797	0.85185	0.99995	no

ENSG00000186687	ENSG00000186687	LYRM7	5:130494719-130541119	q1	q2	OK	4.29415	4.63033	0.108741	0.06558	0.8855	0.99995	no

ENSG00000186704	ENSG00000186704	DTX2P1	7:76139744-77045717	q1	q2	OK	1.03747	0.823847	-0.332627	-0.147795	0.8434	0.99995	no

ENSG00000186710	ENSG00000186710	CCDC42B	12:113587662-113623284	q1	q2	NOTEST	0.265667	0.195191	-0.444737	0	1	1	no

ENSG00000186714	ENSG00000186714	CCDC73	11:32605343-32816204	q1	q2	NOTEST	0.0430313	0.056988	0.405271	0	1	1	no

ENSG00000186715	ENSG00000186715	MST1L	1:17066767-17299474	q1	q2	OK	0.68679	0.973048	0.502643	0.157417	0.74045	0.99995	no

ENSG00000186716	ENSG00000186716	BCR	22:23521890-23660224	q1	q2	OK	25.0596	19.0115	-0.398487	-0.483082	0.2637	0.99995	no

ENSG00000186723	ENSG00000186723	OR10H1	19:15917816-15918936	q1	q2	NOTEST	0	0	0	0	1	1	no

ENSG00000186732	ENSG00000186732	MPPED1	22:43807201-43903728	q1	q2	OK	0.579268	0.92568	0.676283	0.573802	0.25475	0.99995	no

ENSG00000186743	ENSG00000186743	TPI1P3	6:116252311-116381921	q1	q2	NOTEST	0	0	0	0	1	1	no

ENSG00000186765	ENSG00000186765	FSCN2	17:79495421-79504156	q1	q2	OK	0.403726	0.533537	0.402213	0.296181	0.65315	0.99995	no

ENSG00000186766	ENSG00000186766	FOXI2	10:129535498-129539450	q1	q2	NOTEST	0	0.0152341	inf	0	1	1	no

ENSG00000186767	ENSG00000186767	SPIN4	X:62563105-62780951	q1	q2	NOTEST	0.0810944	0.0969442	0.257552	0	1	1	no

ENSG00000186777	ENSG00000186777	ZNF732	4:264463-299110	q1	q2	NOTEST	0	0	0	0	1	1	no

ENSG00000186787	ENSG00000186787	SPIN2B	X:57144983-57147980	q1	q2	OK	3.613	3.14007	-0.202401	-0.20821	0.7175	0.99995	no

ENSG00000186788	ENSG00000186788	SPATA31D3	9:84542935-84592004	q1	q2	NOTEST	0	0	0	0	1	1	no

ENSG00000186790	ENSG00000186790	FOXE3	1:47881743-47883723	q1	q2	NOTEST	0.0133314	0.0794552	2.57532	0	1	1	no

ENSG00000186792	ENSG00000186792	HYAL3	3:50316457-50336899	q1	q2	NOTEST	0.315323	0.109849	-1.52131	0	1	1	no

ENSG00000186795	ENSG00000186795	KCNK18	10:118956999-118969810	q1	q2	NOTEST	0	0	0	0	1	1	no

ENSG00000186803	ENSG00000186803	IFNA10	9:21206179-21207142	q1	q2	NOTEST	0	0	0	0	1	1	no

ENSG00000186806	ENSG00000186806	VSIG10L	19:51834789-51847371	q1	q2	OK	2.88347	2.59399	-0.152632	-0.217854	0.7594	0.99995	no

ENSG00000186807	ENSG00000186807	ANXA8L2	10:47740329-47770871	q1	q2	NOTEST	0	0	0	0	1	1	no

ENSG00000186810	ENSG00000186810	CXCR3	X:70835765-70838367	q1	q2	NOTEST	0	0.04588	inf	0	1	1	no

ENSG00000186812	ENSG00000186812	ZNF397	18:32820993-32870196	q1	q2	OK	20.8635	21.3691	0.0345441	0.0473013	0.93585	0.99995	no

ENSG00000186814	ENSG00000186814	ZSCAN30	18:32820993-32870196	q1	q2	OK	13.2363	13.8491	0.0652964	0.0780718	0.88005	0.99995	no

ENSG00000186815	ENSG00000186815	TPCN1	12:113633245-113736390	q1	q2	OK	4.89882	4.8721	-0.00789088	-0.00860756	0.98295	0.99995	no

ENSG00000186818	ENSG00000186818	LILRB4	19:55173578-55181810	q1	q2	NOTEST	0	0	0	0	1	1	no

ENSG00000186825	ENSG00000186825	C2orf27B	2:132552533-132559234	q1	q2	NOTEST	0.0210941	0.0410575	0.960803	0	1	1	no

ENSG00000186827	ENSG00000186827	TNFRSF4	1:1146705-1149518	q1	q2	NOTEST	0	0.0152943	inf	0	1	1	no

ENSG00000186831	ENSG00000186831	KRT17P2	17:18330174-18335162	q1	q2	NOTEST	0	0	0	0	1	1	no

ENSG00000186832	ENSG00000186832	KRT16	17:39766029-39772151	q1	q2	NOTEST	0	0	0	0	1	1	no

ENSG00000186834	ENSG00000186834	HEXIM1	17:43224683-43248951	q1	q2	OK	17.2405	16.0997	-0.0987628	-0.229395	0.7452	0.99995	no

ENSG00000186838	ENSG00000186838	SELV	19:40005752-40011326	q1	q2	NOTEST	0.0533347	0.0600763	0.171721	0	1	1	no

ENSG00000186842	ENSG00000186842	LINC00846	21:33944547-33948191	q1	q2	NOTEST	0.32074	0.104258	-1.62124	0	1	1	no

ENSG00000186844	ENSG00000186844	LCE1A	1:152799948-152800573	q1	q2	NOTEST	0	0	0	0	1	1	no

ENSG00000186847	ENSG00000186847	KRT14	17:39738530-39743173	q1	q2	NOTEST	0	0	0	0	1	1	no

ENSG00000186854	ENSG00000186854	TRABD2A	2:85048773-85134132	q1	q2	NOTEST	0.424915	0.303842	-0.483853	0	1	1	no

ENSG00000186860	ENSG00000186860	KRTAP17-1	17:39471172-39471947	q1	q2	NOTEST	0	0	0	0	1	1	no

ENSG00000186862	ENSG00000186862	PDZD7	10:102756374-102790890	q1	q2	NOTEST	0.216944	0.248497	0.195909	0	1	1	no

ENSG00000186866	ENSG00000186866	POFUT2	21:46683842-46707813	q1	q2	OK	20.3875	19.6595	-0.0524605	-0.0891547	0.873	0.99995	no

ENSG00000186867	ENSG00000186867	QRFPR	4:122250466-122302214	q1	q2	NOTEST	0.0230395	0.122534	2.411	0	1	1	no

ENSG00000186868	ENSG00000186868	MAPT	17:43921016-44105700	q1	q2	OK	2.17275	2.29679	0.0800942	0.0800314	0.87795	0.99995	no

ENSG00000186871	ENSG00000186871	ERCC6L	X:71401202-71527037	q1	q2	OK	7.67397	7.55594	-0.0223611	-0.010911	0.99245	0.99995	no

ENSG00000186881	ENSG00000186881	OR13F1	9:107266454-107267547	q1	q2	NOTEST	0	0	0	0	1	1	no

ENSG00000186886	ENSG00000186886	OR5D3P	11:55493593-55494634	q1	q2	NOTEST	0	0	0	0	1	1	no

ENSG00000186889	ENSG00000186889	TMEM17	2:62727355-62739029	q1	q2	OK	2.586	2.68608	0.0547757	0.0725242	0.91825	0.99995	no

ENSG00000186891	ENSG00000186891	TNFRSF18	1:1138887-1142071	q1	q2	NOTEST	0	0	0	0	1	1	no

ENSG00000186895	ENSG00000186895	FGF3	11:69624991-69633792	q1	q2	NOTEST	0	0	0	0	1	1	no

ENSG00000186897	ENSG00000186897	C1QL4	12:49726199-49730971	q1	q2	OK	4.70826	4.38903	-0.101293	-0.155031	0.82685	0.99995	no

ENSG00000186907	ENSG00000186907	RTN4RL2	11:57228021-57245007	q1	q2	OK	8.808	7.62556	-0.207972	-0.26967	0.67675	0.99995	no

ENSG00000186908	ENSG00000186908	ZDHHC17	12:77157367-77247476	q1	q2	OK	32.6964	35.8614	0.1333	0.214783	0.64765	0.99995	no

ENSG00000186910	ENSG00000186910	SERPINA11	14:94896969-94946026	q1	q2	NOTEST	0	0	0	0	1	1	no

ENSG00000186912	ENSG00000186912	P2RY4	X:69478015-69479654	q1	q2	NOTEST	0.10166	0.0845372	-0.266088	0	1	1	no

ENSG00000186918	ENSG00000186918	ZNF395	8:28203101-28347835	q1	q2	OK	9.91384	8.29482	-0.257232	-0.325523	0.4575	0.99995	no

ENSG00000186919	ENSG00000186919	ZACN	17:74035183-74117657	q1	q2	OK	20.2777	16.0052	-0.341355	-0.263808	0.7132	0.99995	no

ENSG00000186924	ENSG00000186924	KRTAP22-1	21:31973413-31973612	q1	q2	NOTEST	0	0	0	0	1	1	no

ENSG00000186925	ENSG00000186925	KRTAP19-6	21:31913853-31914183	q1	q2	NOTEST	0	0	0	0	1	1	no

ENSG00000186930	ENSG00000186930	KRTAP6-2	21:31970908-31971219	q1	q2	NOTEST	0	0	0	0	1	1	no

ENSG00000186940	ENSG00000186940	CHCHD2P9	9:82006218-82006674	q1	q2	NOTEST	0	0	0	0	1	1	no

ENSG00000186943	ENSG00000186943	OR13C8	9:107331448-107332411	q1	q2	NOTEST	0	0	0	0	1	1	no

ENSG00000186951	ENSG00000186951	PPARA	22:46546423-46639653	q1	q2	OK	2.77604	2.8177	0.0214894	0.021183	0.95795	0.99995	no

ENSG00000186952	ENSG00000186952	TMEM232	5:109624933-110100857	q1	q2	OK	2.38752	1.80245	-0.405552	-0.097636	0.65365	0.99995	no

ENSG00000186960	ENSG00000186960	C14orf23	14:29241909-29299410	q1	q2	NOTEST	0	0	0	0	1	1	no

ENSG00000186965	ENSG00000186965	KRTAP19-2	21:31859361-31859755	q1	q2	NOTEST	0	0	0	0	1	1	no

ENSG00000186967	ENSG00000186967	KRTAP19-4	21:31869141-31869451	q1	q2	NOTEST	0	0	0	0	1	1	no

ENSG00000186970	ENSG00000186970	KRTAP15-1	21:31812596-31813070	q1	q2	NOTEST	0	0	0	0	1	1	no

ENSG00000186971	ENSG00000186971	KRTAP13-4	21:31802571-31803216	q1	q2	NOTEST	0	0	0	0	1	1	no

ENSG00000186973	ENSG00000186973	FAM183A	1:43610823-43622067	q1	q2	NOTEST	0	0.0817574	inf	0	1	1	no

ENSG00000186976	ENSG00000186976	EFCAB6	22:43911996-44208217	q1	q2	OK	0.637532	0.49356	-0.369271	-0.251571	0.58845	0.99995	no

ENSG00000186977	ENSG00000186977	KRTAP19-5	21:31873974-31874435	q1	q2	NOTEST	0	0	0	0	1	1	no

ENSG00000186980	ENSG00000186980	KRTAP23-1	21:31720716-31720924	q1	q2	NOTEST	0	0	0	0	1	1	no

ENSG00000186994	ENSG00000186994	KANK3	19:8367010-8408146	q1	q2	NOTEST	0.405787	0.268772	-0.594341	0	1	1	no

ENSG00000186998	ENSG00000186998	EMID1	22:29601839-29655586	q1	q2	OK	4.2971	3.68316	-0.222419	-0.280501	0.61485	0.99995	no

ENSG00000187003	ENSG00000187003	ACTL7A	9:111624602-111626035	q1	q2	NOTEST	0	0.0187176	inf	0	1	1	no

ENSG00000187005	ENSG00000187005	KRTAP21-1	21:32127438-32127746	q1	q2	NOTEST	0	0	0	0	1	1	no

ENSG00000187008	ENSG00000187008	PKD1L3	16:71963440-72033877	q1	q2	NOTEST	0.00500645	0.0236722	2.24134	0	1	1	no

ENSG00000187010	ENSG00000187010	RHD	1:25568727-25756683	q1	q2	NOTEST	0.073708	0	#NAME?	0	1	1	no

ENSG00000187012	ENSG00000187012	LINC00207	22:44965218-44968221	q1	q2	NOTEST	0	0	0	0	1	1	no

ENSG00000187013	ENSG00000187013	C17orf82	17:59489111-59490641	q1	q2	OK	0.512343	0.506957	-0.0152464	-0.0146069	0.97045	0.99995	no

ENSG00000187017	ENSG00000187017	ESPN	1:6484847-6580121	q1	q2	NOTEST	0.300897	0.159995	-0.911237	0	1	1	no

ENSG00000187021	ENSG00000187021	PNLIPRP1	10:118349896-118368687	q1	q2	NOTEST	0	0	0	0	1	1	no

ENSG00000187024	ENSG00000187024	PTRH1	9:130374543-130497604	q1	q2	OK	3.56686	3.28981	-0.116651	-0.050247	0.94545	0.99995	no

ENSG00000187026	ENSG00000187026	KRTAP21-2	21:32119111-32119551	q1	q2	NOTEST	0	0	0	0	1	1	no

ENSG00000187033	ENSG00000187033	SAMD7	3:169629359-169656963	q1	q2	NOTEST	0.0168657	0	#NAME?	0	1	1	no

ENSG00000187037	ENSG00000187037	GPR141	7:37723398-38065297	q1	q2	NOTEST	0	0	0	0	1	1	no

ENSG00000187045	ENSG00000187045	TMPRSS6	22:37461475-37505603	q1	q2	NOTEST	0.100521	0.187281	0.897699	0	1	1	no

ENSG00000187048	ENSG00000187048	CYP4A11	1:47394848-47407156	q1	q2	NOTEST	0	0.0156525	inf	0	1	1	no

ENSG00000187049	ENSG00000187049	TMEM216	11:61159158-61166335	q1	q2	OK	5.31987	6.47254	0.282941	0.345036	0.60825	0.99995	no

ENSG00000187051	ENSG00000187051	RPS19BP1	22:39925097-39928860	q1	q2	OK	53.3961	47.6587	-0.163996	-0.347907	0.61955	0.99995	no

ENSG00000187054	ENSG00000187054	TMPRSS11A	4:68566997-68995598	q1	q2	NOTEST	0.0271124	0.0554008	1.03095	0	1	1	no

ENSG00000187060	ENSG00000187060	RP11-187C18.3	9:45562978-45563576	q1	q2	NOTEST	0.185853	0.235396	0.34093	0	1	1	no

ENSG00000187066	ENSG00000187066	TMEM262	11:64851694-64883856	q1	q2	OK	0.725535	0.693712	-0.0647077	-0.0224982	0.9694	0.99995	no

ENSG00000187068	ENSG00000187068	C3orf70	3:184795837-184870802	q1	q2	OK	4.21826	4.51081	0.0967377	0.186915	0.79945	0.99995	no

ENSG00000187079	ENSG00000187079	TEAD1	11:12695968-12966298	q1	q2	OK	32.8171	32.986	0.00740255	0.010599	0.9808	0.99995	no

ENSG00000187080	ENSG00000187080	OR2AK2	1:248099362-248264224	q1	q2	NOTEST	0	0	0	0	1	1	no

ENSG00000187082	ENSG00000187082	DEFB106B	8:7340025-7343922	q1	q2	NOTEST	0	0	0	0	1	1	no

ENSG00000187091	ENSG00000187091	PLCD1	3:38048986-38071253	q1	q2	OK	20.43	16.5694	-0.302171	-0.503888	0.4285	0.99995	no

ENSG00000187094	ENSG00000187094	CCK	3:42299316-42307699	q1	q2	NOTEST	0.0458676	0	#NAME?	0	1	1	no

ENSG00000187097	ENSG00000187097	ENTPD5	14:74318546-74551196	q1	q2	OK	4.19114	3.64895	-0.199861	-0.114894	0.78555	0.99995	no

ENSG00000187098	ENSG00000187098	MITF	3:69788585-70017488	q1	q2	OK	0.65462	0.611972	-0.0971922	-0.0762553	0.8368	0.99995	no

ENSG00000187103	ENSG00000187103	FUNDC2P3	6:146348781-146758734	q1	q2	NOTEST	0	0	0	0	1	1	no

ENSG00000187105	ENSG00000187105	HEATR4	14:73945188-74025651	q1	q2	NOTEST	0.156743	0.0419284	-1.9024	0	1	1	no

ENSG00000187109	ENSG00000187109	NAP1L1	12:76438669-76478813	q1	q2	OK	471.733	479.468	0.0234653	0.0411809	0.95065	0.99995	no

ENSG00000187116	ENSG00000187116	LILRA5	19:54818352-54850421	q1	q2	NOTEST	0	0	0	0	1	1	no

ENSG00000187118	ENSG00000187118	CMC1	3:28283074-28390618	q1	q2	OK	22.8173	23.4342	0.0384878	0.0295352	0.97235	0.99995	no

ENSG00000187122	ENSG00000187122	SLIT1	10:98757794-99052413	q1	q2	OK	6.96956	7.22228	0.0513856	0.0733269	0.9093	0.99995	no

ENSG00000187123	ENSG00000187123	LYPD6	2:150186498-150330662	q1	q2	OK	4.62832	4.51613	-0.0354007	-0.0608329	0.92505	0.99995	no

ENSG00000187134	ENSG00000187134	AKR1C1	10:4934795-5025475	q1	q2	OK	4.58697	3.61221	-0.344657	-0.374187	0.58575	0.99995	no

ENSG00000187135	ENSG00000187135	VSTM2B	19:29777917-30055386	q1	q2	NOTEST	0.0747113	0.0179128	-2.06034	0	1	1	no

ENSG00000187140	ENSG00000187140	FOXD3	1:63786554-63790797	q1	q2	NOTEST	0.0910021	0	#NAME?	0	1	1	no

ENSG00000187144	ENSG00000187144	SPATA21	1:16679069-16763919	q1	q2	NOTEST	0.0342226	0	#NAME?	0	1	1	no

ENSG00000187145	ENSG00000187145	MRPS21	1:150266288-150281414	q1	q2	OK	32.9837	28.6146	-0.205004	-0.415866	0.5458	0.99995	no

ENSG00000187147	ENSG00000187147	RNF220	1:44870865-45140227	q1	q2	OK	74.3271	69.4514	-0.0978832	-0.165748	0.75035	0.99995	no

ENSG00000187151	ENSG00000187151	ANGPTL5	11:101761404-101871789	q1	q2	NOTEST	0.0109454	0	#NAME?	0	1	1	no

ENSG00000187156	ENSG00000187156	LINC00221	14:106938444-106977000	q1	q2	NOTEST	0	0	0	0	1	1	no

ENSG00000187164	ENSG00000187164	KIAA1598	10:118592511-118886097	q1	q2	OK	5.74309	6.33901	0.142432	0.258375	0.70825	0.99995	no

ENSG00000187166	ENSG00000187166	H1FNT	12:48722762-48724062	q1	q2	NOTEST	0.107868	0.0433903	-1.31382	0	1	1	no

ENSG00000187170	ENSG00000187170	LCE4A	1:152680635-152681967	q1	q2	NOTEST	0	0	0	0	1	1	no

ENSG00000187172	ENSG00000187172	BAGE2	21:10906200-11098980	q1	q2	NOTEST	0	0	0	0	1	1	no

ENSG00000187173	ENSG00000187173	LCE2A	1:152670839-152671918	q1	q2	NOTEST	0	0	0	0	1	1	no

ENSG00000187175	ENSG00000187175	KRTAP12-1	21:45917774-46131495	q1	q2	NOTEST	0	0	0	0	1	1	no

ENSG00000187180	ENSG00000187180	LCE2C	1:152647770-152659877	q1	q2	NOTEST	0	0	0	0	1	1	no

ENSG00000187185	ENSG00000187185	CTD-2600O9.1	16:57792128-57896957	q1	q2	NOTEST	0	0	0	0	1	1	no

ENSG00000187186	ENSG00000187186	RP11-195F19.5	9:34638129-34681295	q1	q2	OK	1.99567	2.13281	0.0958824	0.0195301	0.94445	0.99995	no

ENSG00000187187	ENSG00000187187	ZNF546	19:40490040-40523514	q1	q2	OK	3.25209	2.69224	-0.272559	-0.230409	0.5307	0.99995	no

ENSG00000187189	ENSG00000187189	TSPYL4	6:116571150-116575261	q1	q2	OK	32.4247	33.9464	0.0661662	0.162735	0.8127	0.99995	no

ENSG00000187191	ENSG00000187191	DAZ3	Y:26909215-26959626	q1	q2	NOTEST	0	0	0	0	1	1	no

ENSG00000187193	ENSG00000187193	MT1X	16:56716335-56721964	q1	q2	OK	15.0226	13.6682	-0.136319	-0.163289	0.8197	0.99995	no

ENSG00000187210	ENSG00000187210	GCNT1	9:79034751-79122332	q1	q2	NOTEST	0.182039	0.171072	-0.0896378	0	1	1	no

ENSG00000187223	ENSG00000187223	LCE2D	1:152635871-152637135	q1	q2	NOTEST	0	0	0	0	1	1	no

ENSG00000187229	ENSG00000187229	RP11-161I2.1	8:18384810-18942240	q1	q2	NOTEST	0.0134976	0.0133625	-0.0145051	0	1	1	no

ENSG00000187231	ENSG00000187231	SESTD1	2:179966418-180129517	q1	q2	OK	32.4855	36.6483	0.173949	0.215016	0.69505	0.99995	no

ENSG00000187238	ENSG00000187238	LCE3B	1:152586286-152586574	q1	q2	NOTEST	0	0	0	0	1	1	no

ENSG00000187239	ENSG00000187239	FNBP1	9:132649465-132805473	q1	q2	OK	21.8241	22.3995	0.0375504	0.065158	0.90525	0.99995	no

ENSG00000187240	ENSG00000187240	DYNC2H1	11:102980159-103350591	q1	q2	OK	8.92049	10.197	0.19295	0.190818	0.7239	0.99995	no

ENSG00000187242	ENSG00000187242	KRT12	17:39007051-39023462	q1	q2	NOTEST	0	0	0	0	1	1	no

ENSG00000187243	ENSG00000187243	MAGED4B	X:51804922-51812368	q1	q2	NOTEST	0	0.140692	inf	0	1	1	no

ENSG00000187244	ENSG00000187244	BCAM	19:45312327-45324673	q1	q2	OK	12.6912	10.8813	-0.221975	-0.360746	0.55635	0.99995	no

ENSG00000187257	ENSG00000187257	RSBN1L	7:77325759-77412339	q1	q2	OK	16.4178	17.6336	0.103066	0.168175	0.75425	0.99995	no

ENSG00000187258	ENSG00000187258	NPSR1	7:34386123-34917944	q1	q2	NOTEST	0	0	0	0	1	1	no

ENSG00000187260	ENSG00000187260	WDR86	7:151038784-151110440	q1	q2	OK	15.0143	12.6026	-0.25262	-0.235098	0.73995	0.99995	no

ENSG00000187266	ENSG00000187266	EPOR	19:11488235-11530018	q1	q2	OK	3.64323	2.81106	-0.374104	-0.428284	0.53745	0.99995	no

ENSG00000187268	ENSG00000187268	FAM9C	X:13053736-13062801	q1	q2	NOTEST	0.0610335	0.0442928	-0.46253	0	1	1	no

ENSG00000187272	ENSG00000187272	KRTAP9-8	17:39394296-39395256	q1	q2	NOTEST	0	0	0	0	1	1	no

ENSG00000187288	ENSG00000187288	CIDEC	3:9908397-9921938	q1	q2	NOTEST	0.10132	0.0766336	-0.402874	0	1	1	no

ENSG00000187323	ENSG00000187323	DCC	18:49866541-51094653	q1	q2	OK	8.74606	12.3241	0.494779	0.583255	0.14795	0.99995	no

ENSG00000187325	ENSG00000187325	TAF9B	X:77385244-77395203	q1	q2	NOTEST	0	0	0	0	1	1	no

ENSG00000187372	ENSG00000187372	PCDHB13	5:140593508-140596993	q1	q2	NOTEST	0.00761416	0	#NAME?	0	1	1	no

ENSG00000187391	ENSG00000187391	MAGI2	7:77646392-79100524	q1	q2	OK	6.83531	6.20235	-0.140192	-0.166743	0.7961	0.99995	no

ENSG00000187398	ENSG00000187398	LUZP2	11:24518515-25104150	q1	q2	OK	0.557842	0.459278	-0.280492	-0.174806	0.6779	0.99995	no

ENSG00000187416	ENSG00000187416	LHFPL3	7:103969103-104549001	q1	q2	OK	0.763016	0.801009	0.0701058	0.0414775	0.9493	0.99995	no

ENSG00000187446	ENSG00000187446	CHP1	15:41523036-41574043	q1	q2	OK	21.6478	21.3498	-0.0200033	-0.0448715	0.9461	0.99995	no

ENSG00000187456	ENSG00000187456	RDM1	17:34245069-34257777	q1	q2	OK	2.21239	2.07187	-0.0946731	-0.0860687	0.8809	0.99995	no

ENSG00000187461	ENSG00000187461	AL583828.1	6:13358061-13487894	q1	q2	NOTEST	0	0	0	0	1	1	no

ENSG00000187472	ENSG00000187472	RP11-67P15.1	6:99228132-99241868	q1	q2	NOTEST	0	0	0	0	1	1	no

ENSG00000187474	ENSG00000187474	FPR3	19:52248424-52329442	q1	q2	NOTEST	0.0123744	0.0101952	-0.279459	0	1	1	no

ENSG00000187475	ENSG00000187475	HIST1H1T	6:26107639-26108364	q1	q2	NOTEST	0	0	0	0	1	1	no

ENSG00000187479	ENSG00000187479	C11orf96	11:43942608-44022707	q1	q2	OK	0.600195	0.493229	-0.283174	-0.258884	0.704	0.99995	no

ENSG00000187481	ENSG00000187481	HSD3BP1	1:120009843-120016426	q1	q2	NOTEST	0	0	0	0	1	1	no

ENSG00000187483	ENSG00000187483	SERPINA13P	14:95107061-95113331	q1	q2	NOTEST	0	0	0	0	1	1	no

ENSG00000187486	ENSG00000187486	KCNJ11	11:17407405-17410878	q1	q2	OK	0.69666	0.49782	-0.484829	-0.336937	0.48185	0.99995	no

ENSG00000187492	ENSG00000187492	CDHR4	3:49828164-49837268	q1	q2	NOTEST	0	0.023401	inf	0	1	1	no

ENSG00000187498	ENSG00000187498	COL4A1	13:110801317-111165374	q1	q2	OK	51.5989	51.2887	-0.00869886	-0.0108107	0.98795	0.99995	no

ENSG00000187504	ENSG00000187504	RPL7P48	17:49579848-49580594	q1	q2	NOTEST	0.0459532	0.0876242	0.931165	0	1	1	no

ENSG00000187510	ENSG00000187510	PLEKHG7	12:93115280-93323107	q1	q2	NOTEST	0	0	0	0	1	1	no

ENSG00000187513	ENSG00000187513	GJA4	1:35178337-35325417	q1	q2	NOTEST	0.303031	0.423943	0.484404	0	1	1	no

ENSG00000187514	ENSG00000187514	PTMA	2:232571516-232578251	q1	q2	OK	765.532	776.434	0.0204	0.0433877	0.9493	0.99995	no

ENSG00000187516	ENSG00000187516	CXorf27	X:37208527-38548169	q1	q2	NOTEST	0	0	0	0	1	1	no

ENSG00000187522	ENSG00000187522	HSPA14	10:14861248-14913740	q1	q2	OK	32.0203	34.6866	0.115394	0.247872	0.71405	0.99995	no

ENSG00000187527	ENSG00000187527	ATP13A5	3:192958913-193096632	q1	q2	NOTEST	0.0114772	0	#NAME?	0	1	1	no

ENSG00000187531	ENSG00000187531	SIRT7	17:79869814-79885590	q1	q2	OK	11.2133	10.6634	-0.072547	-0.0626447	0.89085	0.99995	no

ENSG00000187533	ENSG00000187533	C4orf40	4:70999332-71042516	q1	q2	NOTEST	0	0	0	0	1	1	no

ENSG00000187534	ENSG00000187534	CTC-471F3.4	19:40448562-40449634	q1	q2	OK	1.89166	2.72363	0.525878	0.443263	0.5445	0.99995	no

ENSG00000187535	ENSG00000187535	IFT140	16:1543344-1662111	q1	q2	OK	15.578	12.7874	-0.284783	-0.348161	0.49995	0.99995	no

ENSG00000187536	ENSG00000187536	TPM3P7	2:25956621-26101385	q1	q2	NOTEST	0	0	0	0	1	1	no

ENSG00000187537	ENSG00000187537	POTEM	14:19983558-20020272	q1	q2	NOTEST	0	0	0	0	1	1	no

ENSG00000187545	ENSG00000187545	PRAMEF10	1:12952726-12958101	q1	q2	NOTEST	0	0.0185988	inf	0	1	1	no

ENSG00000187546	ENSG00000187546	AGMO	7:15239942-15601640	q1	q2	NOTEST	0.221309	0.240897	0.122351	0	1	1	no

ENSG00000187550	ENSG00000187550	SBK2	19:56041099-56048456	q1	q2	NOTEST	0.0295777	0	#NAME?	0	1	1	no

ENSG00000187553	ENSG00000187553	CYP26C1	10:94821020-94828454	q1	q2	NOTEST	0.0182865	0.0180026	-0.0225776	0	1	1	no

ENSG00000187554	ENSG00000187554	TLR5	1:223282747-223316624	q1	q2	NOTEST	0.181316	0.183316	0.0158264	0	1	1	no

ENSG00000187555	ENSG00000187555	USP7	16:8985950-9060847	q1	q2	OK	64.6982	61.3066	-0.0776832	-0.106113	0.78385	0.99995	no

ENSG00000187556	ENSG00000187556	NANOS3	19:13972876-13991571	q1	q2	OK	1.85797	1.63775	-0.182013	-0.142814	0.82715	0.99995	no

ENSG00000187559	ENSG00000187559	FOXD4L3	9:70917275-70920000	q1	q2	NOTEST	0	0	0	0	1	1	no

ENSG00000187566	ENSG00000187566	NHLRC1	6:18120717-18122851	q1	q2	NOTEST	0.365187	0.455686	0.319404	0	1	1	no

ENSG00000187569	ENSG00000187569	DPPA3	12:7864049-7870155	q1	q2	NOTEST	0	0.0279988	inf	0	1	1	no

ENSG00000187581	ENSG00000187581	COX8C	14:93799564-94174222	q1	q2	NOTEST	0	0.0705613	inf	0	1	1	no

ENSG00000187583	ENSG00000187583	PLEKHN1	1:901876-917497	q1	q2	NOTEST	0	0.0224824	inf	0	1	1	no

ENSG00000187589	ENSG00000187589	TERF1P2	18:14377544-14378756	q1	q2	NOTEST	0.109381	0.186552	0.770218	0	1	1	no

ENSG00000187595	ENSG00000187595	ZNF385C	17:40118758-40217383	q1	q2	NOTEST	0.040477	0.0257413	-0.653016	0	1	1	no

ENSG00000187600	ENSG00000187600	TMEM247	2:46656328-46711564	q1	q2	NOTEST	0	0	0	0	1	1	no

ENSG00000187601	ENSG00000187601	MAGEH1	X:55478537-55479998	q1	q2	OK	21.2859	22.1263	0.0558628	0.113108	0.8722	0.99995	no

ENSG00000187605	ENSG00000187605	TET3	2:74229839-74335303	q1	q2	OK	15.5482	17.09	0.136404	0.315161	0.62295	0.99995	no

ENSG00000187607	ENSG00000187607	ZNF286A	17:15602890-15652629	q1	q2	OK	43.2369	45.6141	0.0772156	0.0943901	0.883	0.99995	no

ENSG00000187608	ENSG00000187608	ISG15	1:948802-949920	q1	q2	OK	10.7818	10.3406	-0.060279	-0.0826795	0.9079	0.99995	no

ENSG00000187609	ENSG00000187609	EXD3	9:140201347-140317714	q1	q2	OK	6.15271	5.36238	-0.198347	-0.258637	0.73045	0.99995	no

ENSG00000187612	ENSG00000187612	OR5W2	11:55681125-55682058	q1	q2	NOTEST	0	0	0	0	1	1	no

ENSG00000187616	ENSG00000187616	TMEM8C	9:136379707-136393734	q1	q2	NOTEST	0	0	0	0	1	1	no

ENSG00000187621	ENSG00000187621	TCL6	14:96116834-96158980	q1	q2	NOTEST	0	0	0	0	1	1	no

ENSG00000187624	ENSG00000187624	C17orf97	17:254325-273510	q1	q2	NOTEST	0	0	0	0	1	1	no

ENSG00000187626	ENSG00000187626	ZKSCAN4	6:28212400-28227011	q1	q2	OK	5.55532	5.0141	-0.147882	-0.221959	0.754	0.99995	no

ENSG00000187627	ENSG00000187627	RGPD1	2:87098423-87248975	q1	q2	NOTEST	0.0909295	0.0608124	-0.580383	0	1	1	no

ENSG00000187630	ENSG00000187630	DHRS4L2	14:24407939-24475617	q1	q2	OK	4.71735	4.2995	-0.133809	-0.0962863	0.8831	0.99995	no

ENSG00000187634	ENSG00000187634	SAMD11	1:860259-894689	q1	q2	OK	0.5633	0.398617	-0.498898	-0.0748987	0.61855	0.99995	no

ENSG00000187642	ENSG00000187642	C1orf170	1:901876-917497	q1	q2	NOTEST	0	0.0649663	inf	0	1	1	no

ENSG00000187650	ENSG00000187650	VMAC	19:5904851-5915888	q1	q2	OK	1.09306	1.20866	0.145037	0.11733	0.8592	0.99995	no

ENSG00000187653	ENSG00000187653	TMSB4XP8	4:91048685-92523064	q1	q2	OK	202.315	232.614	0.201331	0.426395	0.50535	0.99995	no

ENSG00000187657	ENSG00000187657	TSPY13P	Y:9743203-9745748	q1	q2	NOTEST	0	0	0	0	1	1	no

ENSG00000187658	ENSG00000187658	C5orf52	5:157098560-157107151	q1	q2	NOTEST	0	0	0	0	1	1	no

ENSG00000187664	ENSG00000187664	HAPLN4	19:19366449-19384200	q1	q2	OK	0.761378	0.967442	0.345563	0.277322	0.63705	0.99995	no

ENSG00000187667	ENSG00000187667	WHAMMP3	15:23187727-23208737	q1	q2	NOTEST	0.330345	0.208668	-0.662765	0	1	1	no

ENSG00000187672	ENSG00000187672	ERC2	3:55542335-56502391	q1	q2	OK	2.69454	3.60377	0.419471	0.419143	0.40945	0.99995	no

ENSG00000187676	ENSG00000187676	B3GALTL	13:31774072-31906413	q1	q2	OK	8.5759	8.45198	-0.0209991	-0.044445	0.9494	0.99995	no

ENSG00000187678	ENSG00000187678	SPRY4	5:141689991-142077617	q1	q2	OK	40.2336	49.3205	0.293785	0.560896	0.3466	0.99995	no

ENSG00000187682	ENSG00000187682	ERAS	X:48687282-48688548	q1	q2	NOTEST	0	0	0	0	1	1	no

ENSG00000187686	ENSG00000187686	KRT18P59	11:124983056-124996677	q1	q2	OK	0.401575	0.484115	0.269681	0.21952	0.7632	0.99995	no

ENSG00000187688	ENSG00000187688	TRPV2	17:16317837-16340317	q1	q2	NOTEST	0.284591	0.0502385	-2.50203	0	1	1	no

ENSG00000187689	ENSG00000187689	AMTN	4:71384256-71398459	q1	q2	NOTEST	0	0	0	0	1	1	no

ENSG00000187690	ENSG00000187690	CXorf67	X:51139362-51208513	q1	q2	NOTEST	0	0	0	0	1	1	no

ENSG00000187695	ENSG00000187695	RP11-723O4.6	3:128598438-128759585	q1	q2	OK	0.700041	0.913694	0.384271	0.0865973	0.8301	0.99995	no

ENSG00000187699	ENSG00000187699	C2orf88	2:190744334-191236391	q1	q2	OK	0.82035	0.720235	-0.187772	-0.0717586	0.87995	0.99995	no

ENSG00000187701	ENSG00000187701	OR2T27	1:248813231-248814185	q1	q2	NOTEST	0	0	0	0	1	1	no

ENSG00000187713	ENSG00000187713	TMEM203	9:140086068-140100090	q1	q2	OK	29.0794	27.9075	-0.0593468	-0.115896	0.8677	0.99995	no

ENSG00000187714	ENSG00000187714	SLC18A3	10:50817140-50918307	q1	q2	OK	13.6972	12.8162	-0.095912	-0.194044	0.7797	0.99995	no

ENSG00000187715	ENSG00000187715	KBTBD12	3:127634074-127706514	q1	q2	NOTEST	0.0127557	0	#NAME?	0	1	1	no

ENSG00000187720	ENSG00000187720	THSD4	15:71389290-72075722	q1	q2	OK	1.54626	1.20034	-0.365338	-0.277934	0.6163	0.99995	no

ENSG00000187721	ENSG00000187721	GTF2IP3	13:19520894-19636858	q1	q2	NOTEST	0	0	0	0	1	1	no

ENSG00000187726	ENSG00000187726	DNAJB13	11:73661363-73681411	q1	q2	NOTEST	0.0475554	0.0815051	0.777282	0	1	1	no

ENSG00000187730	ENSG00000187730	GABRD	1:1950779-1962192	q1	q2	NOTEST	0.0561684	0.0412856	-0.444119	0	1	1	no

ENSG00000187733	ENSG00000187733	AMY1C	1:104293027-104301312	q1	q2	NOTEST	0.0174809	0.0172124	-0.0223235	0	1	1	no

ENSG00000187735	ENSG00000187735	TCEA1	8:54879111-54935089	q1	q2	OK	67.5935	70.2401	0.0554103	0.1297	0.8418	0.99995	no

ENSG00000187736	ENSG00000187736	NHEJ1	2:219940038-220035549	q1	q2	OK	13.3639	14.0046	0.0675544	0.124001	0.8439	0.99995	no

ENSG00000187741	ENSG00000187741	FANCA	16:89773541-89883065	q1	q2	OK	32.9224	34.9699	0.087045	0.092551	0.84635	0.99995	no

ENSG00000187742	ENSG00000187742	SECISBP2	9:91933420-91974557	q1	q2	OK	38.5243	43.081	0.161283	0.284212	0.644	0.99995	no

ENSG00000187747	ENSG00000187747	OR52B6	11:5274419-5667019	q1	q2	NOTEST	0.093045	0.0860156	-0.113329	0	1	1	no

ENSG00000187753	ENSG00000187753	C9orf153	9:88835179-88874572	q1	q2	NOTEST	0.0737057	0.0579854	-0.346086	0	1	1	no

ENSG00000187754	ENSG00000187754	SSX7	X:52673139-52683950	q1	q2	NOTEST	0	0	0	0	1	1	no

ENSG00000187758	ENSG00000187758	ADH1A	4:100010007-100222513	q1	q2	NOTEST	0.41013	0.12578	-1.70517	0	1	1	no

ENSG00000187762	ENSG00000187762	HSPE1P11	6:34857041-35059179	q1	q2	NOTEST	0	0.204083	inf	0	1	1	no

ENSG00000187763	ENSG00000187763	OR2B7P	6:28014211-28015145	q1	q2	NOTEST	0	0.0315977	inf	0	1	1	no

ENSG00000187764	ENSG00000187764	SEMA4D	9:91975701-92113045	q1	q2	OK	10.2871	10.5274	0.0333057	0.043317	0.93185	0.99995	no

ENSG00000187766	ENSG00000187766	KRTAP10-8	21:45917774-46131495	q1	q2	NOTEST	0	0	0	0	1	1	no

ENSG00000187772	ENSG00000187772	LIN28B	6:105404922-105531207	q1	q2	OK	8.84091	10.9917	0.314147	0.664529	0.33695	0.99995	no

ENSG00000187773	ENSG00000187773	FAM69C	18:72102962-72125179	q1	q2	OK	0.560717	0.65008	0.213344	0.194499	0.79175	0.99995	no

ENSG00000187775	ENSG00000187775	DNAH17	17:76374720-76573476	q1	q2	OK	1.49001	0.887336	-0.747769	-0.33327	0.43115	0.99995	no

ENSG00000187778	ENSG00000187778	MCRS1	12:49932939-49961936	q1	q2	OK	40.4768	37.9619	-0.092545	-0.2018	0.76735	0.99995	no

ENSG00000187783	ENSG00000187783	TMEM72	10:45306471-45491339	q1	q2	NOTEST	0.0173533	0.008183	-1.08451	0	1	1	no

ENSG00000187786	ENSG00000187786	SCXB	8:145317002-145331153	q1	q2	NOTEST	0.116908	0.0603341	-0.954332	0	1	1	no

ENSG00000187790	ENSG00000187790	FANCM	14:45605142-45670093	q1	q2	OK	6.91444	7.87548	0.187754	0.238289	0.6332	0.99995	no

ENSG00000187791	ENSG00000187791	FAM205CP	9:34889060-34895775	q1	q2	NOTEST	0	0	0	0	1	1	no

ENSG00000187792	ENSG00000187792	ZNF70	22:24083768-24093279	q1	q2	OK	3.28643	3.50708	0.0937449	0.160375	0.8212	0.99995	no

ENSG00000187796	ENSG00000187796	CARD9	9:139221931-139268133	q1	q2	OK	0.787145	0.482116	-0.707249	-0.324427	0.5705	0.99995	no

ENSG00000187800	ENSG00000187800	PEAR1	1:156863489-156886226	q1	q2	OK	1.90009	1.74731	-0.120933	-0.133883	0.80795	0.99995	no

ENSG00000187801	ENSG00000187801	ZFP69B	1:40915773-40929390	q1	q2	OK	5.00391	5.18818	0.0521716	0.0817015	0.9125	0.99995	no

ENSG00000187806	ENSG00000187806	TMEM202	15:72690613-72879692	q1	q2	NOTEST	0	0	0	0	1	1	no

ENSG00000187808	ENSG00000187808	SOWAHD	X:118892575-118894164	q1	q2	NOTEST	0.422226	0.188332	-1.16474	0	1	1	no

ENSG00000187811	ENSG00000187811	AP000867.1	11:71292900-71319086	q1	q2	NOTEST	0	0	0	0	1	1	no

ENSG00000187812	ENSG00000187812	RP11-24M17.5	15:76067893-76079239	q1	q2	NOTEST	0	0	0	0	1	1	no

ENSG00000187815	ENSG00000187815	ZFP69	1:40942886-40974333	q1	q2	OK	3.34491	3.33765	-0.0031336	-0.00449359	0.9949	0.99995	no

ENSG00000187821	ENSG00000187821	HELT	4:185939994-185941958	q1	q2	NOTEST	0.0369677	0	#NAME?	0	1	1	no

ENSG00000187823	ENSG00000187823	ZCCHC16	X:111697726-111700473	q1	q2	NOTEST	0	0.018055	inf	0	1	1	no

ENSG00000187824	ENSG00000187824	TMEM220	17:10583653-10718481	q1	q2	OK	0.92432	1.14061	0.303337	0.131815	0.73575	0.99995	no

ENSG00000187833	ENSG00000187833	C2orf78	2:74011315-74044274	q1	q2	NOTEST	0.00834066	0	#NAME?	0	1	1	no

ENSG00000187837	ENSG00000187837	HIST1H1C	6:26055967-26056699	q1	q2	OK	9.20472	7.04513	-0.385748	-0.507157	0.4654	0.99995	no

ENSG00000187838	ENSG00000187838	TMEM256-PLSCR3	17:7283852-7307456	q1	q2	OK	15.5419	13.0833	-0.248442	-0.327445	0.61115	0.99995	no

ENSG00000187840	ENSG00000187840	EIF4EBP1	8:37887858-37917883	q1	q2	OK	51.0196	46.8737	-0.122272	-0.253994	0.7139	0.99995	no

ENSG00000187847	ENSG00000187847	OR7E25P	19:9314983-9315893	q1	q2	NOTEST	0	0	0	0	1	1	no

ENSG00000187848	ENSG00000187848	P2RX2	12:133195365-133198972	q1	q2	NOTEST	0.0442622	0.0541849	0.291816	0	1	1	no

ENSG00000187855	ENSG00000187855	ASCL4	12:108168161-108170421	q1	q2	NOTEST	0	0	0	0	1	1	no

ENSG00000187857	ENSG00000187857	OR6C75	12:55758894-55759833	q1	q2	NOTEST	0	0	0	0	1	1	no

ENSG00000187860	ENSG00000187860	CCDC157	22:30727976-30825045	q1	q2	OK	4.07613	3.70672	-0.137057	-0.0582113	0.91725	0.99995	no

ENSG00000187862	ENSG00000187862	TTC24	1:156549518-156556562	q1	q2	NOTEST	0.0405818	0	#NAME?	0	1	1	no

ENSG00000187866	ENSG00000187866	FAM122A	9:71320574-71624092	q1	q2	OK	4.98768	4.21015	-0.244496	-0.250879	0.7083	0.99995	no

ENSG00000187867	ENSG00000187867	PALM3	19:14164176-14169971	q1	q2	OK	6.06994	5.65487	-0.102189	-0.132599	0.83995	0.99995	no

ENSG00000187870	ENSG00000187870	RNFT1P3	17:20646645-20657814	q1	q2	NOTEST	0	0	0	0	1	1	no

ENSG00000187871	ENSG00000187871	GFRAL	6:55192266-55267291	q1	q2	NOTEST	0	0	0	0	1	1	no

ENSG00000187889	ENSG00000187889	C1orf168	1:57184476-57285369	q1	q2	NOTEST	0.0423062	0.0279871	-0.596106	0	1	1	no

ENSG00000187893	ENSG00000187893	CXXC1P1	X:47566589-47612855	q1	q2	NOTEST	0	0.0271459	inf	0	1	1	no

ENSG00000187900	ENSG00000187900	OR5H7P	3:97952494-98176742	q1	q2	NOTEST	0	0	0	0	1	1	no

ENSG00000187902	ENSG00000187902	SHISA7	19:55940106-55954230	q1	q2	OK	0.867347	0.856003	-0.0189927	-0.0254825	0.9696	0.99995	no

ENSG00000187904	ENSG00000187904	AC097382.5	4:6910968-7059679	q1	q2	NOTEST	0	0	0	0	1	1	no

ENSG00000187905	ENSG00000187905	AC002472.13	22:21400248-21418457	q1	q2	NOTEST	0.0556966	0.0075977	-2.87396	0	1	1	no

ENSG00000187908	ENSG00000187908	DMBT1	10:124320180-124403252	q1	q2	NOTEST	0.0134416	0	#NAME?	0	1	1	no

ENSG00000187912	ENSG00000187912	CLEC17A	19:14693895-14721969	q1	q2	NOTEST	0.019788	0.0512808	1.37379	0	1	1	no

ENSG00000187918	ENSG00000187918	OR51I2	11:5274419-5667019	q1	q2	NOTEST	0	0	0	0	1	1	no

ENSG00000187922	ENSG00000187922	LCN10	9:139632618-139642980	q1	q2	NOTEST	0	0.0301855	inf	0	1	1	no

ENSG00000187942	ENSG00000187942	LDLRAD2	1:22138757-22263790	q1	q2	NOTEST	0.198703	0.0932505	-1.09143	0	1	1	no

ENSG00000187944	ENSG00000187944	C2orf66	2:197669725-197675000	q1	q2	OK	1.12151	0.713302	-0.652853	-0.658852	0.36375	0.99995	no

ENSG00000187950	ENSG00000187950	OVCH1	12:29542226-29650619	q1	q2	NOTEST	0.127361	0.0937473	-0.44207	0	1	1	no

ENSG00000187951	ENSG00000187951	ARHGAP11B	15:30909160-31065196	q1	q2	OK	13.8342	15.1035	0.126642	0.145979	0.75045	0.99995	no

ENSG00000187952	ENSG00000187952	HS6ST1P1	1:21754795-21756030	q1	q2	OK	0.897226	1.0358	0.207197	0.205465	0.7685	0.99995	no

ENSG00000187953	ENSG00000187953	PMS2CL	7:6749758-6793493	q1	q2	OK	9.41605	8.52354	-0.143669	-0.173594	0.79055	0.99995	no

ENSG00000187954	ENSG00000187954	CYHR1	8:145674964-145691060	q1	q2	OK	29.8643	24.4441	-0.288935	-0.466823	0.46965	0.99995	no

ENSG00000187955	ENSG00000187955	COL14A1	8:121072018-121384275	q1	q2	OK	2.73685	3.4285	0.325061	0.477201	0.3921	0.99995	no

ENSG00000187957	ENSG00000187957	DNER	2:230222344-230579274	q1	q2	OK	35.485	33.4002	-0.08735	-0.212093	0.76205	0.99995	no

ENSG00000187959	ENSG00000187959	CPSF4L	17:71161115-71258491	q1	q2	NOTEST	0.0465943	0.0737951	0.663372	0	1	1	no

ENSG00000187961	ENSG00000187961	KLHL17	1:895966-901095	q1	q2	OK	18.9846	15.6687	-0.276941	-0.378171	0.58765	0.99995	no

ENSG00000187969	ENSG00000187969	ZCCHC13	X:73524024-73524868	q1	q2	NOTEST	0	0	0	0	1	1	no

ENSG00000187979	ENSG00000187979	AC008079.9	22:18660804-18688682	q1	q2	NOTEST	0	0	0	0	1	1	no

ENSG00000187980	ENSG00000187980	PLA2G2C	1:20487745-20503917	q1	q2	NOTEST	0	0	0	0	1	1	no

ENSG00000187984	ENSG00000187984	ANKRD19P	9:95571669-95661890	q1	q2	NOTEST	0.392069	0.404446	0.0448425	0	1	1	no

ENSG00000187987	ENSG00000187987	ZSCAN23	6:28399706-28411279	q1	q2	OK	3.49779	2.97809	-0.232057	-0.230175	0.71115	0.99995	no

ENSG00000187988	ENSG00000187988	RP11-405L18.2	9:37477145-37478314	q1	q2	NOTEST	0	0.263013	inf	0	1	1	no

ENSG00000187990	ENSG00000187990	HIST1H2BG	6:26216427-26216872	q1	q2	OK	1.18763	0.880211	-0.43216	-0.322915	0.6386	0.99995	no

ENSG00000187994	ENSG00000187994	RINL	19:39358469-39368919	q1	q2	NOTEST	0.103719	0.116074	0.162375	0	1	1	no

ENSG00000187997	ENSG00000187997	C17orf99	17:76142433-76162258	q1	q2	NOTEST	0	0	0	0	1	1	no

ENSG00000187999	ENSG00000187999	HNRNPA1P61	2:33861568-33862492	q1	q2	NOTEST	0.0327797	0	#NAME?	0	1	1	no

ENSG00000188000	ENSG00000188000	OR7D2	19:9296278-9299493	q1	q2	NOTEST	0.0319076	0.0872238	1.45082	0	1	1	no

ENSG00000188001	ENSG00000188001	TPRG1	3:188659503-189043093	q1	q2	NOTEST	0.322853	0.407758	0.336837	0	1	1	no

ENSG00000188002	ENSG00000188002	RP11-43F13.1	5:1594740-1634120	q1	q2	OK	0.915269	2.93262	1.67992	0.698535	0.14665	0.99995	no

ENSG00000188004	ENSG00000188004	C1orf204	1:159796539-159869953	q1	q2	NOTEST	0.0976594	0.148738	0.606947	0	1	1	no

ENSG00000188010	ENSG00000188010	MORN2	2:39103102-39202590	q1	q2	OK	23.3444	20.8221	-0.164962	-0.268932	0.695	0.99995	no

ENSG00000188011	ENSG00000188011	CXXC11	2:242811751-242815975	q1	q2	NOTEST	0.294742	0.175738	-0.746027	0	1	1	no

ENSG00000188013	ENSG00000188013	MEIS3P2	17:20487070-20494493	q1	q2	OK	1.62283	1.27633	-0.346511	-0.382673	0.6002	0.99995	no

ENSG00000188015	ENSG00000188015	S100A3	1:153516088-153522612	q1	q2	NOTEST	0	0	0	0	1	1	no

ENSG00000188021	ENSG00000188021	UBQLN2	X:56590025-56593443	q1	q2	OK	27.3234	28.044	0.0375541	0.0907983	0.8938	0.99995	no

ENSG00000188026	ENSG00000188026	RILPL1	12:123942187-124018265	q1	q2	OK	9.36078	7.47909	-0.323767	-0.46511	0.47975	0.99995	no

ENSG00000188029	ENSG00000188029	CTSL3P	9:90387367-90401799	q1	q2	NOTEST	0	0	0	0	1	1	no

ENSG00000188032	ENSG00000188032	C19orf67	19:14192430-14196687	q1	q2	NOTEST	0.0193404	0.0913935	2.24047	0	1	1	no

ENSG00000188033	ENSG00000188033	ZNF490	19:12490002-12750912	q1	q2	OK	1.40663	1.44248	0.0363085	0.0130464	0.9794	0.99995	no

ENSG00000188037	ENSG00000188037	CLCN1	7:143013218-143049176	q1	q2	NOTEST	0.0168332	0.0154654	-0.122264	0	1	1	no

ENSG00000188038	ENSG00000188038	NRN1L	16:67906925-67922758	q1	q2	NOTEST	0.345966	0.318341	-0.120059	0	1	1	no

ENSG00000188039	ENSG00000188039	NWD1	19:16830786-16928774	q1	q2	NOTEST	0.0266442	0.00836454	-1.67146	0	1	1	no

ENSG00000188042	ENSG00000188042	ARL4C	2:235401684-235405697	q1	q2	OK	13.4845	11.6186	-0.214855	-0.477772	0.4996	0.99995	no

ENSG00000188050	ENSG00000188050	RNF133	7:121958480-122526813	q1	q2	NOTEST	0.0190652	0	#NAME?	0	1	1	no

ENSG00000188051	ENSG00000188051	TMEM221	19:17546317-17571763	q1	q2	OK	1.31088	1.2414	-0.0785635	-0.0821668	0.8958	0.99995	no

ENSG00000188056	ENSG00000188056	TREML4	6:41196061-41206620	q1	q2	NOTEST	0	0	0	0	1	1	no

ENSG00000188060	ENSG00000188060	RAB42	1:28915834-28969597	q1	q2	OK	0.957194	0.92807	-0.0445787	-0.0188427	0.9713	0.99995	no

ENSG00000188064	ENSG00000188064	WNT7B	22:46316241-46373009	q1	q2	OK	2.33034	2.1767	-0.0983993	-0.144296	0.82975	0.99995	no

ENSG00000188069	ENSG00000188069	OR51F1	11:4726156-5013659	q1	q2	NOTEST	0	0	0	0	1	1	no

ENSG00000188070	ENSG00000188070	C11orf95	11:63527363-63536113	q1	q2	OK	26.4169	25.2341	-0.0660856	-0.0904163	0.8179	0.99995	no

ENSG00000188073	ENSG00000188073	PMS2P10	7:74880904-74988262	q1	q2	NOTEST	0	0.0473238	inf	0	1	1	no

ENSG00000188076	ENSG00000188076	SCGB1C1	11:127114-207428	q1	q2	NOTEST	0	0	0	0	1	1	no

ENSG00000188078	ENSG00000188078	RP5-1119A7.11	22:36932702-36933651	q1	q2	NOTEST	0	0	0	0	1	1	no

ENSG00000188086	ENSG00000188086	PRSS45	3:46753604-46854064	q1	q2	NOTEST	0	0	0	0	1	1	no

ENSG00000188089	ENSG00000188089	PLA2G4E	15:42264960-42343388	q1	q2	NOTEST	0	0	0	0	1	1	no

ENSG00000188092	ENSG00000188092	GPR89B	1:147400505-147465753	q1	q2	NOTEST	0.104097	0.128064	0.29894	0	1	1	no

ENSG00000188095	ENSG00000188095	MESP2	15:90303821-90321982	q1	q2	NOTEST	0.126583	0.0708145	-0.837968	0	1	1	no

ENSG00000188100	ENSG00000188100	FAM25A	10:88695296-88784489	q1	q2	NOTEST	0	0	0	0	1	1	no

ENSG00000188101	ENSG00000188101	ALOX15P2	9:95675437-95683842	q1	q2	NOTEST	0	0	0	0	1	1	no

ENSG00000188107	ENSG00000188107	EYS	6:64345724-66417118	q1	q2	NOTEST	0.353432	0.241684	-0.548309	0	1	1	no

ENSG00000188112	ENSG00000188112	C6orf132	6:42068855-42110357	q1	q2	NOTEST	0	0.00821747	inf	0	1	1	no

ENSG00000188120	ENSG00000188120	DAZ1	Y:25275501-25345241	q1	q2	NOTEST	0	0	0	0	1	1	no

ENSG00000188124	ENSG00000188124	OR2AG2	11:6789217-6790286	q1	q2	NOTEST	0	0.0266385	inf	0	1	1	no

ENSG00000188130	ENSG00000188130	MAPK12	22:50683611-50700254	q1	q2	OK	22.9726	22.8574	-0.00725306	-0.012181	0.9846	0.99995	no

ENSG00000188133	ENSG00000188133	TMEM215	9:32783496-32787397	q1	q2	NOTEST	0.32721	0.256746	-0.349874	0	1	1	no

ENSG00000188152	ENSG00000188152	NUTM2G	9:99660347-99775862	q1	q2	NOTEST	0.233204	0.195907	-0.251427	0	1	1	no

ENSG00000188153	ENSG00000188153	COL4A5	X:107683073-107940775	q1	q2	OK	22.1415	20.0424	-0.143691	-0.169646	0.7227	0.99995	no

ENSG00000188155	ENSG00000188155	KRTAP10-6	21:45917774-46131495	q1	q2	NOTEST	0	0	0	0	1	1	no

ENSG00000188157	ENSG00000188157	AGRN	1:955502-991496	q1	q2	OK	77.1852	69.3177	-0.1551	-0.192143	0.64165	0.99995	no

ENSG00000188158	ENSG00000188158	NHS	X:17393542-17754114	q1	q2	OK	8.57344	8.89875	0.0537298	0.124497	0.85955	0.99995	no

ENSG00000188162	ENSG00000188162	OTOG	11:17568919-17668697	q1	q2	NOTEST	0.00243293	0.00479037	0.97744	0	1	1	no

ENSG00000188163	ENSG00000188163	FAM166A	9:140135664-140142222	q1	q2	NOTEST	0.387813	0.227819	-0.767473	0	1	1	no

ENSG00000188167	ENSG00000188167	TMPPE	3:33038099-33138722	q1	q2	NOTEST	0.361099	0.350418	-0.0433174	0	1	1	no

ENSG00000188171	ENSG00000188171	ZNF626	19:20718630-20844402	q1	q2	NOTEST	0.0823704	0	#NAME?	0	1	1	no

ENSG00000188175	ENSG00000188175	HEPACAM2	7:92817898-92855837	q1	q2	NOTEST	0.111848	0	#NAME?	0	1	1	no

ENSG00000188176	ENSG00000188176	SMTNL2	17:4487293-4511614	q1	q2	NOTEST	0.154866	0.112862	-0.456453	0	1	1	no

ENSG00000188177	ENSG00000188177	ZC3H6	2:113033170-113097640	q1	q2	OK	3.51771	3.53895	0.00868389	0.00877346	0.9861	0.99995	no

ENSG00000188185	ENSG00000188185	LINC00265	7:39773230-39832691	q1	q2	OK	3.89868	3.52376	-0.145874	-0.14978	0.77265	0.99995	no

ENSG00000188186	ENSG00000188186	LAMTOR4	7:99746529-99756338	q1	q2	OK	81.8859	77.3532	-0.0821538	-0.14419	0.83795	0.99995	no

ENSG00000188191	ENSG00000188191	PRKAR1B	7:588833-829190	q1	q2	OK	5.52135	4.78092	-0.207733	-0.165887	0.80575	0.99995	no

ENSG00000188199	ENSG00000188199	NUTM2B	10:81420646-81526085	q1	q2	NOTEST	0.321989	0.301023	-0.0971382	0	1	1	no

ENSG00000188206	ENSG00000188206	HNRNPU-AS1	1:244998623-245027844	q1	q2	OK	20.2957	22.5054	0.149098	0.0661689	0.89735	0.99995	no

ENSG00000188211	ENSG00000188211	NCR3LG1	11:17373272-17398888	q1	q2	OK	2.33048	2.97256	0.351076	0.618704	0.36975	0.99995	no

ENSG00000188215	ENSG00000188215	DCUN1D3	16:20621564-20936328	q1	q2	OK	3.42016	3.56752	0.0608586	0.0353492	0.9574	0.99995	no

ENSG00000188219	ENSG00000188219	POTEE	2:131862419-132111282	q1	q2	NOTEST	0.032642	0.0179845	-0.859972	0	1	1	no

ENSG00000188223	ENSG00000188223	AC002398.9	19:36230057-36245420	q1	q2	OK	0.723523	0.345565	-1.06608	-0.225278	0.71145	0.99995	no

ENSG00000188227	ENSG00000188227	ZNF793	19:37988062-38210691	q1	q2	OK	2.90791	2.81378	-0.0474725	-0.0238115	0.9486	0.99995	no

ENSG00000188229	ENSG00000188229	TUBB4B	9:140135664-140142222	q1	q2	OK	189.506	181.412	-0.0629743	-0.154175	0.82435	0.99995	no

ENSG00000188234	ENSG00000188234	AGAP4	10:46321041-46349323	q1	q2	OK	0.326237	0.817137	1.32466	0.685021	0.2683	0.99995	no

ENSG00000188242	ENSG00000188242	CTD-2228K2.5	5:443272-473213	q1	q2	OK	10.7915	10.2415	-0.07546	-0.0588513	0.91815	0.99995	no

ENSG00000188243	ENSG00000188243	COMMD6	13:76099349-76123575	q1	q2	OK	70.6653	79.0156	0.161137	0.289424	0.67695	0.99995	no

ENSG00000188257	ENSG00000188257	PLA2G2A	1:20301924-20306932	q1	q2	NOTEST	0.146086	0	#NAME?	0	1	1	no

ENSG00000188263	ENSG00000188263	IL17REL	22:50432941-50451088	q1	q2	NOTEST	0.0071892	0	#NAME?	0	1	1	no

ENSG00000188266	ENSG00000188266	HYKK	15:78799905-78829714	q1	q2	OK	0.391129	0.500918	0.35693	0.207629	0.7048	0.99995	no

ENSG00000188269	ENSG00000188269	OR7A5	19:14903120-14946188	q1	q2	NOTEST	0.0691554	0	#NAME?	0	1	1	no

ENSG00000188277	ENSG00000188277	C15orf62	15:41060066-41106767	q1	q2	OK	1.62469	1.40437	-0.210248	-0.123797	0.86455	0.99995	no

ENSG00000188279	ENSG00000188279	FAM25C	10:49199648-49207818	q1	q2	NOTEST	0	0	0	0	1	1	no

ENSG00000188280	ENSG00000188280	FAM230A	22:20692437-20745048	q1	q2	NOTEST	0	0.00265041	inf	0	1	1	no

ENSG00000188282	ENSG00000188282	RUFY4	2:218899682-218955304	q1	q2	NOTEST	0.0272864	0.00937228	-1.54171	0	1	1	no

ENSG00000188283	ENSG00000188283	ZNF383	19:37498758-37734828	q1	q2	OK	3.58893	3.37896	-0.0869738	-0.0650704	0.94255	0.99995	no

ENSG00000188290	ENSG00000188290	HES4	1:934341-935552	q1	q2	OK	22.8699	18.1465	-0.333757	-0.58007	0.4067	0.99995	no

ENSG00000188293	ENSG00000188293	IGFL1	19:46732220-46734500	q1	q2	NOTEST	0	0	0	0	1	1	no

ENSG00000188295	ENSG00000188295	ZNF669	1:247263263-247267674	q1	q2	OK	9.59915	10.0729	0.0694965	0.119967	0.8614	0.99995	no

ENSG00000188305	ENSG00000188305	C19orf35	19:2274630-2282181	q1	q2	NOTEST	0.0512798	0.0695177	0.43899	0	1	1	no

ENSG00000188306	ENSG00000188306	LRRIQ4	3:169539709-169555563	q1	q2	NOTEST	0.0299682	0.029314	-0.0318464	0	1	1	no

ENSG00000188312	ENSG00000188312	CENPP	9:95059639-95432547	q1	q2	OK	2.49162	2.57792	0.0491241	0.0468523	0.94125	0.99995	no

ENSG00000188313	ENSG00000188313	PLSCR1	3:146232966-146262651	q1	q2	OK	9.85326	11.2675	0.193491	0.291678	0.64365	0.99995	no

ENSG00000188314	ENSG00000188314	OR7D1P	19:9345819-9346812	q1	q2	NOTEST	0	0	0	0	1	1	no

ENSG00000188315	ENSG00000188315	C3orf62	3:49306034-49378145	q1	q2	OK	9.12587	7.90453	-0.207284	-0.170983	0.72435	0.99995	no

ENSG00000188316	ENSG00000188316	ENO4	10:118592511-118886097	q1	q2	OK	0.815478	0.600064	-0.442528	-0.113194	0.75475	0.99995	no

ENSG00000188321	ENSG00000188321	ZNF559	19:9434447-9493293	q1	q2	OK	7.20839	7.27739	0.0137448	0.0178308	0.9775	0.99995	no

ENSG00000188322	ENSG00000188322	SBK1	16:28303839-28335170	q1	q2	OK	34.7901	30.8598	-0.172949	-0.426535	0.53995	0.99995	no

ENSG00000188324	ENSG00000188324	OR6C6	12:55677533-55706860	q1	q2	NOTEST	0	0	0	0	1	1	no

ENSG00000188334	ENSG00000188334	BSPH1	19:48471302-48495427	q1	q2	NOTEST	0	0	0	0	1	1	no

ENSG00000188338	ENSG00000188338	SLC38A3	3:50242678-50258411	q1	q2	OK	2.60779	2.28606	-0.189965	-0.162433	0.71715	0.99995	no

ENSG00000188340	ENSG00000188340	OR6N2	1:158746471-158747425	q1	q2	NOTEST	0	0	0	0	1	1	no

ENSG00000188342	ENSG00000188342	GTF2F2	13:45694649-45858237	q1	q2	OK	34.3344	35.1459	0.0337041	0.0749674	0.91775	0.99995	no

ENSG00000188343	ENSG00000188343	FAM92A1	8:94225530-94753245	q1	q2	OK	34.43	38.8464	0.174118	0.198076	0.759	0.99995	no

ENSG00000188352	ENSG00000188352	FOCAD	9:20658307-21031635	q1	q2	OK	15.8143	15.21	-0.0562129	-0.113113	0.8483	0.99995	no

ENSG00000188365	ENSG00000188365	AC092171.2	7:5313999-5465045	q1	q2	OK	0.307781	0.486168	0.65955	0.0516976	0.7229	0.99995	no

ENSG00000188368	ENSG00000188368	PRR19	19:42801184-42814973	q1	q2	OK	2.59126	1.89792	-0.44923	-0.229974	0.79755	0.99995	no

ENSG00000188372	ENSG00000188372	ZP3	7:76018650-76071388	q1	q2	OK	1.17351	1.10928	-0.0812037	-0.0457126	0.93665	0.99995	no

ENSG00000188373	ENSG00000188373	C10orf99	10:85933493-85945050	q1	q2	NOTEST	0	0	0	0	1	1	no

ENSG00000188375	ENSG00000188375	H3F3C	12:31944122-31945175	q1	q2	OK	1.62777	2.16323	0.410293	0.436906	0.534	0.99995	no

ENSG00000188379	ENSG00000188379	IFNA2	9:21384253-21385396	q1	q2	NOTEST	0	0	0	0	1	1	no

ENSG00000188383	ENSG00000188383	AC018892.9	2:97746834-97748986	q1	q2	NOTEST	0.087815	0.082843	-0.0840867	0	1	1	no

ENSG00000188384	ENSG00000188384	CSPG4P8	15:82751613-82798350	q1	q2	OK	0.590866	0.580729	-0.024967	-0.0195763	0.9766	0.99995	no

ENSG00000188385	ENSG00000188385	JAKMIP3	10:133918174-133999466	q1	q2	OK	0.535481	0.296827	-0.851211	-0.93738	0.188	0.99995	no

ENSG00000188386	ENSG00000188386	PPP3R2	9:104331634-104500862	q1	q2	NOTEST	0.0231425	0.0072096	-1.68255	0	1	1	no

ENSG00000188388	ENSG00000188388	GOLGA6L3	15:85783702-85790410	q1	q2	NOTEST	0.0659387	0.164505	1.31894	0	1	1	no

ENSG00000188389	ENSG00000188389	PDCD1	2:242792032-242801060	q1	q2	NOTEST	0	0	0	0	1	1	no

ENSG00000188393	ENSG00000188393	CLEC2A	12:10051271-10084980	q1	q2	NOTEST	0	0	0	0	1	1	no

ENSG00000188394	ENSG00000188394	GPR21	9:125703111-125867145	q1	q2	NOTEST	0.0516497	0.0492212	-0.0694816	0	1	1	no

ENSG00000188396	ENSG00000188396	TCTEX1D4	1:45265896-45272957	q1	q2	NOTEST	0.435669	0.303477	-0.521647	0	1	1	no

ENSG00000188399	ENSG00000188399	ANKRD36P1	Y:28555961-28566682	q1	q2	NOTEST	0	0	0	0	1	1	no

ENSG00000188403	ENSG00000188403	IGHV1OR15-9	15:20169918-20170354	q1	q2	NOTEST	0	0	0	0	1	1	no

ENSG00000188404	ENSG00000188404	SELL	1:169631244-169863408	q1	q2	NOTEST	0.0688422	0.11498	0.740014	0	1	1	no

ENSG00000188408	ENSG00000188408	MAGEB5	X:26234285-26236387	q1	q2	NOTEST	0	0	0	0	1	1	no

ENSG00000188419	ENSG00000188419	CHM	X:85116184-85302566	q1	q2	OK	8.24948	9.92243	0.26639	0.366518	0.47685	0.99995	no

ENSG00000188424	ENSG00000188424	XXbac-B33L19.3	22:20326327-20350457	q1	q2	NOTEST	0	0	0	0	1	1	no

ENSG00000188425	ENSG00000188425	NANOS2	19:46416474-46418036	q1	q2	NOTEST	0	0.0180948	inf	0	1	1	no

ENSG00000188428	ENSG00000188428	BLOC1S5	6:7727029-8102811	q1	q2	OK	9.67573	11.0935	0.197268	0.129735	0.8368	0.99995	no

ENSG00000188438	ENSG00000188438	RP11-1396O13.14	4:9400929-9405291	q1	q2	NOTEST	0	0	0	0	1	1	no

ENSG00000188439	ENSG00000188439	OR4P1P	11:55450714-55453636	q1	q2	NOTEST	0	0	0	0	1	1	no

ENSG00000188451	ENSG00000188451	SRP72P2	6:158658367-158663829	q1	q2	NOTEST	0.129914	0.0981257	-0.404859	0	1	1	no

ENSG00000188452	ENSG00000188452	CERKL	2:182401402-182545603	q1	q2	OK	1.72444	2.05757	0.254809	0.170501	0.77745	0.99995	no

ENSG00000188459	ENSG00000188459	WASF4P	X:47657324-47664188	q1	q2	NOTEST	0	0	0	0	1	1	no

ENSG00000188460	ENSG00000188460	ACTBP11	1:224051427-224052553	q1	q2	NOTEST	0	0.024983	inf	0	1	1	no

ENSG00000188467	ENSG00000188467	SLC24A5	15:48413168-48470714	q1	q2	OK	3.83093	4.45439	0.217532	0.0627403	0.9207	0.99995	no

ENSG00000188474	ENSG00000188474	ZNF788	19:12203077-12267546	q1	q2	OK	1.9297	1.14518	-0.752799	-0.412876	0.5568	0.99995	no

ENSG00000188477	ENSG00000188477	AC003003.5	16:19297060-19333467	q1	q2	NOTEST	0.0108801	0.0339321	1.64096	0	1	1	no

ENSG00000188483	ENSG00000188483	IER5L	9:131937834-131972827	q1	q2	OK	10.0603	10.3192	0.0366577	0.0716087	0.9147	0.99995	no

ENSG00000188486	ENSG00000188486	H2AFX	11:118964563-118966177	q1	q2	OK	145.542	127.946	-0.185904	-0.455005	0.51335	0.99995	no

ENSG00000188487	ENSG00000188487	INSC	11:15133969-15268754	q1	q2	NOTEST	0.0166647	0.00967439	-0.784552	0	1	1	no

ENSG00000188488	ENSG00000188488	SERPINA5	14:95027427-95090983	q1	q2	NOTEST	0.0637227	0.0435791	-0.548173	0	1	1	no

ENSG00000188493	ENSG00000188493	C19orf54	19:41197433-41271294	q1	q2	OK	11.2222	9.73777	-0.204697	-0.140054	0.7717	0.99995	no

ENSG00000188501	ENSG00000188501	LCTL	15:66790354-66858317	q1	q2	OK	1.62949	1.30223	-0.323432	-0.0326125	0.7821	0.99995	no

ENSG00000188505	ENSG00000188505	NCCRP1	19:39687600-39692524	q1	q2	NOTEST	0.0548431	0.0268209	-1.03195	0	1	1	no

ENSG00000188508	ENSG00000188508	KRTDAP	19:35978225-35986460	q1	q2	NOTEST	0	0	0	0	1	1	no

ENSG00000188511	ENSG00000188511	C22orf34	22:49808175-50051190	q1	q2	NOTEST	0	0	0	0	1	1	no

ENSG00000188512	ENSG00000188512	RP1-273G13.2	8:33462373-33896526	q1	q2	NOTEST	0	0.199353	inf	0	1	1	no

ENSG00000188517	ENSG00000188517	COL25A1	4:109731876-110223813	q1	q2	OK	4.45234	4.58943	0.0437511	0.0406518	0.92105	0.99995	no

ENSG00000188522	ENSG00000188522	FAM83G	17:18853657-18950950	q1	q2	NOTEST	0.198611	0.0500733	-1.98784	0	1	1	no

ENSG00000188523	ENSG00000188523	C9orf171	9:135285429-135448704	q1	q2	OK	0.491214	0.207147	-1.2457	-0.889146	0.17785	0.99995	no

ENSG00000188525	ENSG00000188525	AC010969.1	2:10143285-10146158	q1	q2	NOTEST	0.0113111	0.0104002	-0.121122	0	1	1	no

ENSG00000188529	ENSG00000188529	SRSF10	1:24291293-24307417	q1	q2	OK	7.27246	8.94631	0.298849	0.378958	0.57855	0.99995	no

ENSG00000188536	ENSG00000188536	HBA2	16:222845-223709	q1	q2	NOTEST	0	0	0	0	1	1	no

ENSG00000188542	ENSG00000188542	DUSP28	2:241418838-241520789	q1	q2	OK	2.67601	2.30652	-0.214367	-0.119044	0.78615	0.99995	no

ENSG00000188549	ENSG00000188549	C15orf52	15:40623652-40633168	q1	q2	NOTEST	0.218316	0.159348	-0.454239	0	1	1	no

ENSG00000188554	ENSG00000188554	NBR1	17:41322497-41363708	q1	q2	OK	27.5653	23.9263	-0.204258	-0.489545	0.48515	0.99995	no

ENSG00000188558	ENSG00000188558	OR2G6	1:248684915-248685964	q1	q2	NOTEST	0	0	0	0	1	1	no

ENSG00000188559	ENSG00000188559	RALGAPA2	20:20370195-20693131	q1	q2	OK	11.0185	11.4824	0.0594959	0.12114	0.8421	0.99995	no

ENSG00000188566	ENSG00000188566	NDOR1	9:140100146-140111461	q1	q2	OK	7.45362	6.84145	-0.123639	-0.221649	0.7535	0.99995	no

ENSG00000188573	ENSG00000188573	FBLL1	5:167956120-167957639	q1	q2	OK	5.12742	4.80254	-0.0944358	-0.137045	0.8459	0.99995	no

ENSG00000188580	ENSG00000188580	NKAIN2	6:124125285-125146803	q1	q2	OK	0.767238	0.744871	-0.0426824	-0.0341584	0.9449	0.99995	no

ENSG00000188581	ENSG00000188581	KRTAP1-1	17:39196810-39197713	q1	q2	NOTEST	0	0	0	0	1	1	no

ENSG00000188582	ENSG00000188582	PAQR9	3:142668005-142779567	q1	q2	OK	0.756802	0.768981	0.023033	0.00418787	0.9916	0.99995	no

ENSG00000188585	ENSG00000188585	LINC00083	1:178465030-178468769	q1	q2	NOTEST	0	0	0	0	1	1	no

ENSG00000188596	ENSG00000188596	C12orf55	12:96883348-97269333	q1	q2	OK	0.608335	0.454668	-0.420053	-0.308367	0.60955	0.99995	no

ENSG00000188599	ENSG00000188599	NPIPP1	16:15068447-15248421	q1	q2	OK	14.3763	13.8145	-0.0575073	-0.0356876	0.96635	0.99995	no

ENSG00000188603	ENSG00000188603	CLN3	16:28467692-28510291	q1	q2	OK	14.5721	11.147	-0.386563	-0.645992	0.30395	0.99995	no

ENSG00000188610	ENSG00000188610	FAM72B	1:120835809-120855681	q1	q2	NOTEST	0.208833	0.121069	-0.786523	0	1	1	no

ENSG00000188611	ENSG00000188611	ASAH2	10:51942537-52008370	q1	q2	NOTEST	0.275962	0.234624	-0.234122	0	1	1	no

ENSG00000188612	ENSG00000188612	SUMO2	17:73131342-73179078	q1	q2	OK	201.291	222.731	0.146018	0.247489	0.70635	0.99995	no

ENSG00000188613	ENSG00000188613	NANOS1	10:120789227-120793854	q1	q2	OK	3.09877	2.89463	-0.0983183	-0.116362	0.8677	0.99995	no

ENSG00000188620	ENSG00000188620	HMX3	10:124895477-124897257	q1	q2	NOTEST	0.0243899	0	#NAME?	0	1	1	no

ENSG00000188624	ENSG00000188624	IGFL3	19:46604379-46706340	q1	q2	NOTEST	0	0	0	0	1	1	no

ENSG00000188626	ENSG00000188626	GOLGA8M	15:28947099-29002537	q1	q2	NOTEST	0.131047	0.0987222	-0.40864	0	1	1	no

ENSG00000188629	ENSG00000188629	ZNF177	19:9434447-9493293	q1	q2	OK	5.56032	6.47602	0.21994	0.248346	0.72865	0.99995	no

ENSG00000188636	ENSG00000188636	LDOC1L	22:44888451-44894178	q1	q2	OK	25.002	24.4037	-0.0349444	-0.0862104	0.8993	0.99995	no

ENSG00000188641	ENSG00000188641	DPYD	1:97543298-98386605	q1	q2	OK	3.21858	3.25616	0.0167489	0.0231042	0.97115	0.99995	no

ENSG00000188643	ENSG00000188643	S100A16	1:153579361-153585621	q1	q2	OK	6.24214	7.10958	0.187725	0.248655	0.7204	0.99995	no

ENSG00000188646	ENSG00000188646	RP11-361A23.2	12:75057553-75057921	q1	q2	NOTEST	0	0	0	0	1	1	no

ENSG00000188647	ENSG00000188647	PTAR1	9:72324437-72374875	q1	q2	OK	14.0674	15.272	0.118531	0.17194	0.7007	0.99995	no

ENSG00000188649	ENSG00000188649	CC2D2B	10:97471535-97849995	q1	q2	NOTEST	0.0471238	0.0579086	0.297323	0	1	1	no

ENSG00000188655	ENSG00000188655	RNASE9	14:21024251-21029090	q1	q2	NOTEST	0	0	0	0	1	1	no

ENSG00000188656	ENSG00000188656	TSPY7P	Y:9195405-9235047	q1	q2	NOTEST	0	0	0	0	1	1	no

ENSG00000188659	ENSG00000188659	FAM154B	15:82555150-82577271	q1	q2	OK	1.94827	1.6825	-0.21159	-0.165751	0.8021	0.99995	no

ENSG00000188660	ENSG00000188660	LINC00319	21:44866480-44873773	q1	q2	NOTEST	0.166875	0.171057	0.0357088	0	1	1	no

ENSG00000188662	ENSG00000188662	HILS1	17:48240883-48258539	q1	q2	NOTEST	0.0232887	0	#NAME?	0	1	1	no

ENSG00000188668	ENSG00000188668	OR7E90P	2:159731153-159732182	q1	q2	NOTEST	0	0.0279377	inf	0	1	1	no

ENSG00000188672	ENSG00000188672	RHCE	1:25568727-25756683	q1	q2	NOTEST	0.125948	0.116395	-0.113802	0	1	1	no

ENSG00000188674	ENSG00000188674	C2orf80	2:209030066-209054797	q1	q2	OK	1.57265	1.28847	-0.28754	-0.217921	0.6945	0.99995	no

ENSG00000188676	ENSG00000188676	IDO2	8:39759793-39873910	q1	q2	NOTEST	0	0	0	0	1	1	no

ENSG00000188677	ENSG00000188677	PARVB	22:44319618-44568829	q1	q2	NOTEST	0.395785	0.364924	-0.117119	0	1	1	no

ENSG00000188681	ENSG00000188681	TEKT4P2	21:9907189-9968585	q1	q2	NOTEST	0.0678491	0.0329646	-1.04141	0	1	1	no

ENSG00000188686	ENSG00000188686	SCXA	8:145486054-145515082	q1	q2	NOTEST	0.118812	0.0307901	-1.94814	0	1	1	no

ENSG00000188687	ENSG00000188687	SLC4A5	2:74425688-74621009	q1	q2	OK	1.37375	1.10547	-0.313465	-0.0357727	0.7834	0.99995	no

ENSG00000188690	ENSG00000188690	UROS	10:127477145-127511817	q1	q2	OK	27.4509	24.9341	-0.138735	-0.272552	0.6913	0.99995	no

ENSG00000188691	ENSG00000188691	RP11-451K18.7	11:5988406-5989724	q1	q2	NOTEST	0	0.0332336	inf	0	1	1	no

ENSG00000188693	ENSG00000188693	CTB-161K23.1	7:91741464-91810039	q1	q2	NOTEST	0.233447	0.136155	-0.777847	0	1	1	no

ENSG00000188694	ENSG00000188694	KRTAP24-1	21:31653628-31655276	q1	q2	NOTEST	0	0	0	0	1	1	no

ENSG00000188706	ENSG00000188706	ZDHHC9	X:128937263-128977885	q1	q2	OK	13.9647	14.8129	0.0850608	0.161771	0.79725	0.99995	no

ENSG00000188707	ENSG00000188707	ZBED6CL	7:149941004-150035239	q1	q2	NOTEST	0	0.0184769	inf	0	1	1	no

ENSG00000188710	ENSG00000188710	QRFP	9:133768814-133769225	q1	q2	NOTEST	0.230235	0.33763	0.552338	0	1	1	no

ENSG00000188712	ENSG00000188712	OR13D3P	9:107484615-107485552	q1	q2	NOTEST	0	0	0	0	1	1	no

ENSG00000188716	ENSG00000188716	DUPD1	10:76797593-76818272	q1	q2	NOTEST	0	0	0	0	1	1	no

ENSG00000188722	ENSG00000188722	AL391005.1	10:129534322-129534969	q1	q2	NOTEST	0	0	0	0	1	1	no

ENSG00000188725	ENSG00000188725	SMIM15	5:60453535-60560756	q1	q2	OK	27.2677	28.0709	0.0418794	0.0767639	0.9036	0.99995	no

ENSG00000188729	ENSG00000188729	OSTN	3:190917029-190983404	q1	q2	NOTEST	0	0.0162444	inf	0	1	1	no

ENSG00000188730	ENSG00000188730	VWC2	7:49813256-50199426	q1	q2	NOTEST	0.107624	0.0907592	-0.245888	0	1	1	no

ENSG00000188732	ENSG00000188732	FAM221A	7:23719748-23742868	q1	q2	OK	5.51466	6.5131	0.240073	0.301902	0.6666	0.99995	no

ENSG00000188735	ENSG00000188735	TMEM120B	12:122150657-122241812	q1	q2	OK	3.26749	2.91528	-0.164545	-0.153476	0.7836	0.99995	no

ENSG00000188738	ENSG00000188738	FSIP2	2:186584600-186698017	q1	q2	NOTEST	0.259785	0.210003	-0.306911	0	1	1	no

ENSG00000188739	ENSG00000188739	RBM34	1:235294497-235507847	q1	q2	OK	50.9264	46.3448	-0.136006	-0.104778	0.86075	0.99995	no

ENSG00000188747	ENSG00000188747	NOXA1	9:140317801-140336268	q1	q2	NOTEST	0.0556706	0	#NAME?	0	1	1	no

ENSG00000188755	ENSG00000188755	TBC1D3P2	17:60342065-60374387	q1	q2	NOTEST	0	0	0	0	1	1	no

ENSG00000188760	ENSG00000188760	TMEM198	2:220403668-220415317	q1	q2	OK	9.9984	9.33635	-0.0988382	-0.103941	0.87645	0.99995	no

ENSG00000188761	ENSG00000188761	BCL2L15	1:114304453-114456708	q1	q2	OK	0.379272	0.822567	1.1169	0.159685	0.34605	0.99995	no

ENSG00000188763	ENSG00000188763	FZD9	7:72848108-72850450	q1	q2	NOTEST	0.011681	0	#NAME?	0	1	1	no

ENSG00000188765	ENSG00000188765	TMSB4XP2	2:3642425-3692048	q1	q2	OK	50.0631	85.9854	0.780344	1.03148	0.26205	0.99995	no

ENSG00000188766	ENSG00000188766	SPRED3	19:38879060-38886881	q1	q2	OK	4.37575	4.41594	0.0131895	0.0132496	0.98615	0.99995	no

ENSG00000188770	ENSG00000188770	OPTC	1:203463270-203477992	q1	q2	NOTEST	0.0204752	0	#NAME?	0	1	1	no

ENSG00000188771	ENSG00000188771	PLET1	11:112097087-112140678	q1	q2	NOTEST	0.0176961	0.039885	1.17242	0	1	1	no

ENSG00000188778	ENSG00000188778	ADRB3	8:37820515-37824483	q1	q2	NOTEST	0	0.00838037	inf	0	1	1	no

ENSG00000188779	ENSG00000188779	SKOR1	15:68112041-68166204	q1	q2	NOTEST	0.339827	0.199671	-0.767179	0	1	1	no

ENSG00000188782	ENSG00000188782	CATSPER4	1:26517051-26529459	q1	q2	NOTEST	0.0290962	0.0141257	-1.04251	0	1	1	no

ENSG00000188783	ENSG00000188783	PRELP	1:203444955-203460480	q1	q2	NOTEST	0.28266	0.351306	0.313663	0	1	1	no

ENSG00000188784	ENSG00000188784	PLA2G2E	1:20246501-20250110	q1	q2	NOTEST	0	0	0	0	1	1	no

ENSG00000188785	ENSG00000188785	ZNF548	19:57874844-57988938	q1	q2	OK	10.5816	11.4683	0.116096	0.108737	0.80515	0.99995	no

ENSG00000188786	ENSG00000188786	MTF1	1:38275238-38325292	q1	q2	OK	3.88274	3.73968	-0.0541614	-0.0573179	0.8859	0.99995	no

ENSG00000188800	ENSG00000188800	TMCO2	1:40711618-40717363	q1	q2	NOTEST	0.0675934	0.0668814	-0.0152772	0	1	1	no

ENSG00000188801	ENSG00000188801	ZNF322P1	9:99918851-100140806	q1	q2	OK	0.460585	0.510382	0.148109	0.060198	0.88735	0.99995	no

ENSG00000188803	ENSG00000188803	SHISA6	17:11144579-11467380	q1	q2	OK	4.16092	4.58881	0.141216	0.178496	0.7881	0.99995	no

ENSG00000188807	ENSG00000188807	TMEM201	1:9648931-9674935	q1	q2	OK	12.3701	11.6204	-0.0901952	-0.17704	0.7744	0.99995	no

ENSG00000188811	ENSG00000188811	NHLRC3	13:39612442-39624246	q1	q2	OK	13.7247	13.4967	-0.0241604	-0.0401936	0.9447	0.99995	no

ENSG00000188816	ENSG00000188816	HMX2	10:124907637-124910188	q1	q2	NOTEST	0	0	0	0	1	1	no

ENSG00000188817	ENSG00000188817	SNTN	3:63638343-63664696	q1	q2	NOTEST	0	0	0	0	1	1	no

ENSG00000188818	ENSG00000188818	ZDHHC11	5:710470-919472	q1	q2	OK	8.56972	6.65279	-0.365289	-0.144652	0.654	0.99995	no

ENSG00000188820	ENSG00000188820	FAM26F	6:116781901-116784934	q1	q2	OK	0.485796	0.497168	0.0333829	0.0201228	0.97655	0.99995	no

ENSG00000188822	ENSG00000188822	CNR2	1:24197015-24285549	q1	q2	NOTEST	0.00938749	0.0139636	0.572862	0	1	1	no

ENSG00000188825	ENSG00000188825	LINC00910	17:41447212-41466567	q1	q2	OK	1.09711	0.176845	-2.63315	-0.99704	0.0467	0.99995	no

ENSG00000188827	ENSG00000188827	SLX4	16:3631181-3661599	q1	q2	OK	5.40599	5.46649	0.0160577	0.0315049	0.9628	0.99995	no

ENSG00000188828	ENSG00000188828	GLRA4	X:102962151-102983583	q1	q2	NOTEST	0	0.0288765	inf	0	1	1	no

ENSG00000188831	ENSG00000188831	DPPA3P2	14:36840369-36841588	q1	q2	NOTEST	0	0	0	0	1	1	no

ENSG00000188833	ENSG00000188833	ENTPD8	9:140317801-140336268	q1	q2	NOTEST	0.19655	0.0599563	-1.71292	0	1	1	no

ENSG00000188846	ENSG00000188846	RPL14	3:40498782-40503861	q1	q2	OK	481.004	464.083	-0.051669	-0.124498	0.85525	0.99995	no

ENSG00000188848	ENSG00000188848	BEND4	4:42112954-42154895	q1	q2	NOTEST	0.240884	0.274557	0.188764	0	1	1	no

ENSG00000188850	ENSG00000188850	RP11-159F24.2	5:43336265-43348818	q1	q2	NOTEST	0	0	0	0	1	1	no

ENSG00000188856	ENSG00000188856	RPSAP47	8:81471104-81471992	q1	q2	NOTEST	0.208917	0.408845	0.968626	0	1	1	no

ENSG00000188859	ENSG00000188859	FAM78B	1:166026673-166136206	q1	q2	NOTEST	0.269103	0.185144	-0.539512	0	1	1	no

ENSG00000188868	ENSG00000188868	ZNF563	19:12428290-12444534	q1	q2	OK	0.862462	0.742343	-0.216375	-0.194682	0.74635	0.99995	no

ENSG00000188869	ENSG00000188869	TMC3	15:81616717-81666554	q1	q2	NOTEST	0.00534132	0.0321762	2.59072	0	1	1	no

ENSG00000188873	ENSG00000188873	RPL10AP2	8:48001194-48110484	q1	q2	OK	0.47655	1.29171	1.43858	0.72498	0.4224	0.99995	no

ENSG00000188877	ENSG00000188877	POTEA	8:43147625-43218661	q1	q2	NOTEST	0	0	0	0	1	1	no

ENSG00000188878	ENSG00000188878	FBF1	17:73893140-73937221	q1	q2	OK	6.12563	5.1945	-0.237874	-0.172733	0.70255	0.99995	no

ENSG00000188883	ENSG00000188883	KLRG2	7:139137435-139168458	q1	q2	NOTEST	0.0125301	0.036227	1.53167	0	1	1	no

ENSG00000188886	ENSG00000188886	ASTL	2:96789588-96804175	q1	q2	NOTEST	0	0	0	0	1	1	no

ENSG00000188888	ENSG00000188888	GPR179	17:36481412-36499730	q1	q2	NOTEST	0	0	0	0	1	1	no

ENSG00000188895	ENSG00000188895	MSL1	17:38278550-38293042	q1	q2	OK	85.7067	92.0139	0.102444	0.202956	0.7344	0.99995	no

ENSG00000188897	ENSG00000188897	CTD-3088G3.8	16:11453701-11617444	q1	q2	OK	34.0846	29.6518	-0.200999	-0.274012	0.6236	0.99995	no

ENSG00000188906	ENSG00000188906	LRRK2	12:40579810-40763087	q1	q2	NOTEST	0.231987	0.161812	-0.519725	0	1	1	no

ENSG00000188909	ENSG00000188909	BSX	11:122848277-122852428	q1	q2	NOTEST	0	0	0	0	1	1	no

ENSG00000188910	ENSG00000188910	GJB3	1:35178337-35325417	q1	q2	NOTEST	0.246688	0.287107	0.218898	0	1	1	no

ENSG00000188916	ENSG00000188916	FAM196A	10:128593977-129250781	q1	q2	OK	1.06549	1.50445	0.497727	0.201148	0.8099	0.99995	no

ENSG00000188917	ENSG00000188917	TRMT2B	X:100264334-100351353	q1	q2	OK	4.31868	3.97408	-0.119971	-0.116252	0.8429	0.99995	no

ENSG00000188921	ENSG00000188921	PTPLAD2	9:20658307-21031635	q1	q2	OK	0.606901	0.369492	-0.71592	-0.17668	0.569	0.99995	no

ENSG00000188931	ENSG00000188931	C1orf192	1:161334520-161337664	q1	q2	OK	1.63011	2.0315	0.317576	0.320766	0.65485	0.99995	no

ENSG00000188933	ENSG00000188933	USP32P1	17:16592850-16719854	q1	q2	NOTEST	0.227333	0.114824	-0.985382	0	1	1	no

ENSG00000188937	ENSG00000188937	NYX	X:41306686-41334963	q1	q2	NOTEST	0.0464948	0.0462609	-0.00727386	0	1	1	no

ENSG00000188938	ENSG00000188938	FAM120AOS	9:96208775-96328397	q1	q2	OK	25.0236	25.7955	0.0438278	0.0511604	0.93815	0.99995	no

ENSG00000188958	ENSG00000188958	UTS2B	3:190984956-191116459	q1	q2	NOTEST	0.0500909	0.106439	1.0874	0	1	1	no

ENSG00000188959	ENSG00000188959	C9orf152	9:112952327-112970469	q1	q2	NOTEST	0.116202	0.512995	2.14231	0	1	1	no

ENSG00000188971	ENSG00000188971	RP11-427H3.3	2:69685128-70053596	q1	q2	OK	7.86359	7.81095	-0.00968907	-0.0192883	0.97725	0.99995	no

ENSG00000188976	ENSG00000188976	NOC2L	1:860259-894689	q1	q2	OK	29.1369	28.8045	-0.0165537	-0.0348469	0.9616	0.99995	no

ENSG00000188981	ENSG00000188981	MSANTD1	4:3246095-3273465	q1	q2	NOTEST	0.275814	0.271287	-0.0238759	0	1	1	no

ENSG00000188984	ENSG00000188984	AADACL3	1:12776117-12788726	q1	q2	NOTEST	0	0	0	0	1	1	no

ENSG00000188985	ENSG00000188985	DHFRP1	18:23713815-23773319	q1	q2	OK	46.389	47.7054	0.0403693	0.0714193	0.91885	0.99995	no

ENSG00000188986	ENSG00000188986	NELFB	9:140149624-140167998	q1	q2	OK	31.694	28.7145	-0.142434	-0.340745	0.61805	0.99995	no

ENSG00000188987	ENSG00000188987	HIST1H4D	6:26188937-26189304	q1	q2	NOTEST	0	0.425005	inf	0	1	1	no

ENSG00000188991	ENSG00000188991	SLC15A5	12:16341418-16430619	q1	q2	NOTEST	0	0.00844213	inf	0	1	1	no

ENSG00000188992	ENSG00000188992	LIPI	21:15399741-15583166	q1	q2	NOTEST	0.219409	0.283461	0.369526	0	1	1	no

ENSG00000188993	ENSG00000188993	LRRC66	4:52859867-52883786	q1	q2	NOTEST	0.378816	0.417223	0.139321	0	1	1	no

ENSG00000188994	ENSG00000188994	ZNF292	6:87860876-87973914	q1	q2	OK	19.5099	20.4351	0.066842	0.094388	0.8477	0.99995	no

ENSG00000188996	ENSG00000188996	HUS1B	6:485132-693117	q1	q2	NOTEST	0.234965	0.115838	-1.02034	0	1	1	no

ENSG00000188997	ENSG00000188997	KCTD21	11:77850816-77925757	q1	q2	OK	4.91098	4.70609	-0.0614802	-0.0741229	0.8946	0.99995	no

ENSG00000189001	ENSG00000189001	SBSN	19:36014268-36019253	q1	q2	NOTEST	0.058548	0.105756	0.853044	0	1	1	no

ENSG00000189002	ENSG00000189002	PROSP	3:90251465-90306565	q1	q2	NOTEST	0	0	0	0	1	1	no

ENSG00000189007	ENSG00000189007	ADAT2	6:143743968-143771810	q1	q2	OK	3.30712	4.03308	0.286308	0.256212	0.64745	0.99995	no

ENSG00000189011	ENSG00000189011	AC138783.12	7:74696065-74714647	q1	q2	NOTEST	0.0734284	0.148403	1.01511	0	1	1	no

ENSG00000189013	ENSG00000189013	KIR2DL4	19:55235968-55378662	q1	q2	NOTEST	0	0	0	0	1	1	no

ENSG00000189014	ENSG00000189014	FAM35DP	10:47379726-47420712	q1	q2	NOTEST	0.334957	0.426702	0.349253	0	1	1	no

ENSG00000189023	ENSG00000189023	MAGEB16	X:35816458-35821852	q1	q2	NOTEST	0	0	0	0	1	1	no

ENSG00000189030	ENSG00000189030	VHLL	1:156268414-156269428	q1	q2	NOTEST	0.116619	0.145825	0.322433	0	1	1	no

ENSG00000189037	ENSG00000189037	DUSP21	X:44703248-44704130	q1	q2	NOTEST	0	0	0	0	1	1	no

ENSG00000189042	ENSG00000189042	ZNF567	19:37178513-37263727	q1	q2	OK	6.70499	6.48549	-0.0480186	-0.0604765	0.91405	0.99995	no

ENSG00000189043	ENSG00000189043	NDUFA4	7:10971577-10979883	q1	q2	OK	79.771	81.1948	0.0255227	0.0461061	0.9286	0.99995	no

ENSG00000189045	ENSG00000189045	ANKDD1B	5:74907283-74967671	q1	q2	NOTEST	0.0293867	0.117128	1.99484	0	1	1	no

ENSG00000189046	ENSG00000189046	ALKBH2	12:109525995-109531436	q1	q2	OK	19.2571	18.931	-0.0246455	-0.039177	0.9588	0.99995	no

ENSG00000189050	ENSG00000189050	RNFT1	17:57970446-58096413	q1	q2	OK	11.3173	11.377	0.00758611	0.00619749	0.99055	0.99995	no

ENSG00000189051	ENSG00000189051	RNF222	17:8294021-8301144	q1	q2	NOTEST	0.06672	0.0737286	0.144104	0	1	1	no

ENSG00000189052	ENSG00000189052	CGB5	19:49526125-49552400	q1	q2	NOTEST	0	0	0	0	1	1	no

ENSG00000189056	ENSG00000189056	RELN	7:102937868-103629963	q1	q2	NOTEST	0.459095	0.449675	-0.0299124	0	1	1	no

ENSG00000189057	ENSG00000189057	FAM111B	11:58874657-58894883	q1	q2	OK	15.2658	17.8854	0.228475	0.504027	0.4495	0.99995	no

ENSG00000189058	ENSG00000189058	APOD	3:195295572-195311076	q1	q2	OK	0.681421	0.382898	-0.831588	-0.516341	0.3272	0.99995	no

ENSG00000189060	ENSG00000189060	H1F0	22:38201113-38203442	q1	q2	OK	3.88892	4.58031	0.236074	0.366782	0.5897	0.99995	no

ENSG00000189064	ENSG00000189064	GAGE2A	X:49354131-49361430	q1	q2	NOTEST	0	0	0	0	1	1	no

ENSG00000189067	ENSG00000189067	LITAF	16:11641852-11730237	q1	q2	OK	88.9802	86.2082	-0.0456584	-0.104225	0.87145	0.99995	no

ENSG00000189068	ENSG00000189068	VSTM1	19:54544078-54567207	q1	q2	NOTEST	0	0	0	0	1	1	no

ENSG00000189077	ENSG00000189077	TMEM120A	7:75528517-75623977	q1	q2	OK	0.920156	6.06519	2.7206	1.2005	0.0036	0.99995	no

ENSG00000189079	ENSG00000189079	ARID2	12:46123447-46301823	q1	q2	OK	17.6341	17.4495	-0.0151816	-0.0270482	0.95655	0.99995	no

ENSG00000189089	ENSG00000189089	RIMKLBP1	21:37402367-37498938	q1	q2	OK	0.284979	0.533186	0.903786	0.183111	0.62665	0.99995	no
[truncated: 22,301,402 more chars]
